# The catalytic asymmetric polyene cyclization of homofarnesol to ambrox

**DOI:** 10.1038/s41586-024-07757-7

**Published:** 2024-07-31

**Authors:** Na Luo, Mathias Turberg, Markus Leutzsch, Benjamin Mitschke, Sebastian Brunen, Vijay N. Wakchaure, Nils Nöthling, Mathias Schelwies, Ralf Pelzer, Benjamin List

**Affiliations:** 1 Max-Planck-Institut für Kohlenforschung, Mülheim an der Ruhr, Germany; 2 Synthesis and Homogeneous Catalysis, BASF SE, Ludwigshafen, Germany; 3 New Business Development Aroma Ingredients, BASF SE, Ludwigshafen, Germany

**Keywords:** Organocatalysis, Homogeneous catalysis

## Abstract

Polyene cyclizations are among the most complex and challenging transformations in biology. In a single reaction step, multiple carbon–carbon bonds, ring systems and stereogenic centres are constituted from simple, acyclic precursors^1–3^. Simultaneously achieving this kind of precise control over product distribution and stereochemistry poses a formidable task for chemists. In particular, the polyene cyclization of (3*E*,7*E*)-homofarnesol to the valuable naturally occurring ambergris odorant (−)-ambrox is recognized as a longstanding challenge in chemical synthesis^1,4–7^. Here we report a diastereoselective and enantioselective synthesis of (−)-ambrox and the sesquiterpene lactone natural product (+)-sclareolide by a catalytic asymmetric polyene cyclization by using a highly Brønsted-acidic and confined imidodiphosphorimidate catalyst in the presence of fluorinated alcohols. Several experiments, including deuterium-labelling studies, suggest that the reaction predominantly proceeds through a concerted pathway in line with the Stork–Eschenmoser hypothesis^8–10^. Mechanistic studies show the importance of the enzyme-like microenvironment of the imidodiphosphorimidate catalyst for attaining exceptionally high selectivities, previously thought to be achievable only in enzyme-catalysed polyene cyclizations.

## Abstract (web summary)

The catalytic asymmetric polyene cyclization of homofarnesol to ambrox is achieved using a highly Brønsted-acidic and confined imidodiphosphorimidate catalyst.

## Main

**Fig. 1:**
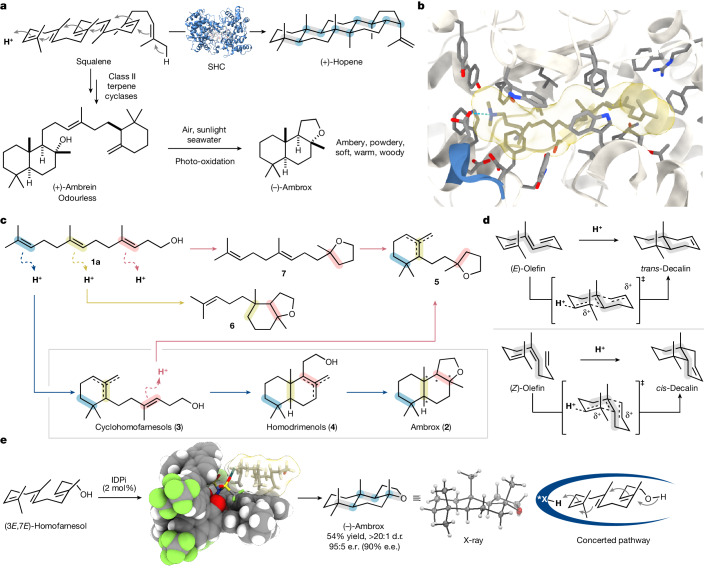
Origin of the ambergris odorant (−)-ambrox and its synthesis by polyene cyclizations. **a**, Polyene cyclization of squalene to (+)-hopene and (+)-ambrein catalysed by class II terpene cyclases and formation of the odorant (−)-ambrox from (+)-ambrein, the main constituent of ambergris. **b**, Active site of SHC in complex with its inhibitor 2-azasqualene (PDB code: 1UMP) (ref. ^13^). The conserved DXDD motif required for substrate protonation is highlighted. **c**, Mechanistic considerations for the polyene cyclization of (3*E*,7*E*)-homofarnesol to (−)-ambrox. **d**, Stork–Eschenmoser hypothesis for the stereospecific antiparallel addition of carbenium ion to an alkene in 1,5-polyolefin cyclizations. **e**, Catalytic asymmetric concerted polyene cyclization of (3*E*,7*E*)-homofarnesol to (−)-ambrox realized in this work. Depictions of SHC and the substrate/catalyst model have been rendered using UCSF Chimera X (refs. ^58,59^).

Polyene cyclizations are one of the most complex and challenging reactions in nature and assemble complex molecular architectures from structurally simple precursors^1–3^. The conversion of the acyclic triterpenoid squalene to pentacyclic (+)-hopene, for example, is catalysed by a single enzyme, squalene–hopene cyclase^11–13^ (SHC; Fig. 1a,b).

The cyclase promotes substrate preorganization and conformational selection according to the Stork–Eschenmoser hypothesis^8–10,14^ (Fig. 1c,d), stabilizes transient charges and specifically selects the initial protonation site^11,15^. Out of 512 theoretically possible stereoisomers, (+)-hopene is produced as a single all-equatorial stereoisomer. Squalene is also considered to be the biosynthetic precursor of the triterpene (+)-ambrein (Fig. 1a), the main constituent of ambergris, a grey waxy substance formed in the gastrointestinal tract of the sperm whale (*Physeter macrocephalus*)^16^. The use of ambergris as fragrance, condiment and medicine dates back to several centuries^5,7^. Until now, it is highly valued for its distinctive scent. The most important odorous component in ambergris is the rare naturally occurring terpenoid (−)-ambrox (**2a**), formed on photo-oxidation of (+)-ambrein^17,18^. Producing (−)-ambrox with high diastereo- and enantioselectivity is of particular importance, as each diastereomer and enantiomer differs in its odour threshold and exhibits a different and sometimes even disagreeable odour sensation^19,20^. Solely all-*trans* configured ambrox and other diastereomers that fulfil the triaxial rule established by Ohloff deliver the desired odour impression with warm amber tonalities^18,21,22^.

Until now, the most efficient syntheses of (−)-ambrox by polyene cyclization harness genetically engineered SHCs^23–31^. Inspired by SHC and other class II terpene cyclases^3^, chemists have long sought for small-molecule catalysts that can effect polyene cyclizations with similar efficiencies^1,6,14,32–35^. Pioneering work mediated by Lewis-acid-assisted Brønsted acids^4,36^ illustrates the difficulty of this transformation. In a stepwise process, (3*E*,7*E*)-homofarnesyl triethylsilyl ether is cyclized to cyclohomofarnesols and homodrimenols in the presence of two equivalents of SnCl_4_-coordinated (*R*)-2-(*o*-fluorobenzyloxy)-2′-hydroxy-1,1′-binaphthyl. A subsequent diastereoselective cyclization using excess trifluoroacetic acid (TFA, 10 equiv.) and SnCl_4_ (2 equiv.) affords (−)-ambrox (54% yield, 74:26 diastereomeric ratio (d.r.) and 87.5:12.5 enantiomeric ratio (e.r.)). The complexity becomes increasingly evident on consideration of potential intermediate steps en route to the desired fully cyclized product (Fig. 1c). First, regioselective protonation of the distal double bond of the polyene substrate is crucial to avoid the formation of unproductive side products **6** and **7**. Second, in the case of a stepwise process, undesirable diastereomers resulting from protonation of cyclohomofarnesols **3** are formed, as shown in previous comprehensive studies^37^. Third, exerting enantiocontrol over the entire cyclization sequence to produce the desired antipode of ambrox as a single enantiomer is extremely challenging. To the best of our knowledge, simultaneously achieving high chemo-, diastereo- and enantioselectivity in a catalytic asymmetric polyene cyclization towards (−)-ambrox has not been realized with small-molecule catalysts so far. Ideally, a suitable Brønsted-acid catalyst should not only exhibit sufficient acidity to protonate olefins but also provide a confined and enzyme-like pocket to effect the desired transformation with high stereoselectivity. Encouraged by our previous studies on catalytic asymmetric functionalizations of unactivated olefins using IDPi catalysts^38–42^, we proposed that this particular class of strong and confined chiral Brønsted acids^43–45^ would be ideally suited to enable the asymmetric polyene cyclization of homofarnesol. Here we describe the development and realization of a catalytic asymmetric synthesis of (−)-ambrox that proceeds with unprecedented stereoselectivities for small-molecule catalysts (Fig. 1e).

## Reaction development

**Fig. 2:**
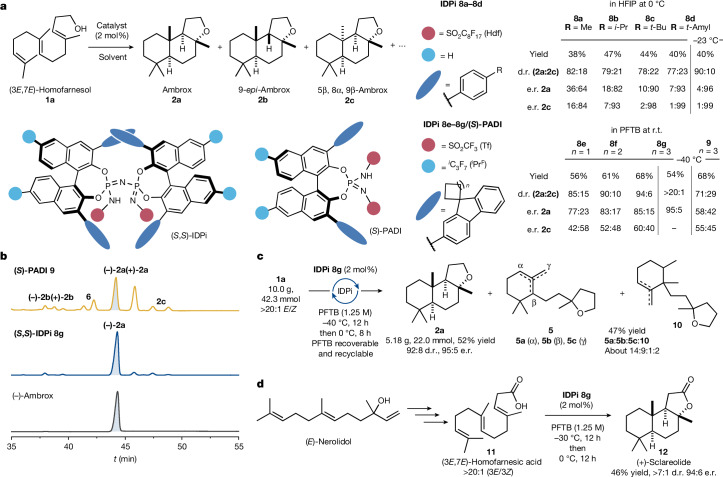
Reaction optimization and scale-up. **a**, Catalyst screening and optimization of the polyene cyclization of (3*E*,7*E*)-homofarnesol to (−)-ambrox. See Supplementary Information for details. **b**, Stacked gas chromatography traces of the crude reaction mixture obtained with PADI **9** (top) and IDPi **8g** (middle) as catalysts. Enantiopure (−)-ambrox is shown as a reference (bottom). **c**, Scale-up of the catalytic asymmetric polyene cyclization in the presence of IDPi catalyst **8g** in PFTB affording (−)-ambrox. **d**, Technical synthesis of (3*E*,7*E*)-homofarnesic acid **11** from (*E*)-nerolidol and catalytic asymmetric polyene cyclization of **11** to (+)-sclareolide. r.t., room temperature; d.r., diastereomeric ratio; e.r., enantiomeric ratio; e.e., enantiomeric excess.

At the onset of our study, extensive screening of solvents showed a substantial increase in reactivity in fluorinated alcohols, which was recently exploited in numerous transformations involving carbocationic intermediates^46–48^. Extensive studies on the role of perfluorinated solvents in diastereoselective polyene cyclizations suggest that perfluorinated alcohols assemble a catalytically active H-bonding network that controls substrate conformation and stabilizes cationic intermediates^46^ in line with previous studies^49–52^. Based on this initial result, we aimed to improve the selectivity using different chiral Brønsted acids in 1,1,1,3,3,3-hexafluoropropan-2-ol (HFIP). We observed that IDPi catalysts converted homofarnesol to ambrox and its minor diastereomer 5β,8α,9β-ambrox **2c** as opposed to other classes of previously developed chiral Brønsted acids (see Supplementary Information for full optimization studies). Subsequently, a screening of structurally diverse IDPi catalysts was conducted (Fig. 2a).

Combining the C_8_F_17_-perfluoroalkyl chain in the imidodiphosphorimidate (IDPi) core (catalyst **8d**) with a 4-isoamyl-C_6_H_4_ group in the 3,3′-position of the 1′-binaphthyl-2,2′-diol backbone delivered the desired product in 40% yield with good enantioselectivity (7:93 e.r.) alongside remaining cyclohomofarnesols **3**. The addition of 1*H*,1*H*-perfluoro-1-octanol enabled the reaction to run at lower reaction temperatures that provided ambrox with improved d.r. (90:10) and e.r. (4:96). Changing the solvent to perfluoro-*tert*-butanol (PFTB)^46^ gave similarly high reactivity and selectivity compared with HFIP. After extensive optimization, spirocyclohexyl-2-fluorenyl-substituted IDPi **8g** emerged as an optimal catalyst. (−)-Ambrox was obtained in 54% yield and with excellent diastereo- and enantioselectivity (d.r. >20:1; e.r. = 95:5). The IDPi catalyst thus effects the formation of two C–C bonds and one C–O bond in a single reaction step while simultaneously exerting stereocontrol over four stereogenic centres, including one quaternary stereogenic centre. Accordingly, two catalyst motifs under two different conditions (IDPi **8d**, HFIP; and IDPi **8g**, PFTB) emerged as optimal systems to provide ambrox **2a** in high yield, diastereo- and enantioselectivity. A pronounced effect of the 3,3′-substitutent on the diastereo- and enantioselectivity suggests that narrowing the active site of the IDPi catalyst presumably enables preferential binding of (3*E*,7*E*)-homofarnesol in its all-*trans* conformation in line with the Stork–Eschenmoser hypothesis^8–10^. The importance of a confined active site is further showcased by comparison of the IDPi catalyst **8g** with highly acidic but structurally less confined *N*,*N*′-bistriflylphosphoramidimidate (PADI) catalyst **9** (ref. ^53^), which gave a complex product mixture (Fig. 2b).

With the optimized conditions at hand, the scalability of the polyene cyclization was evaluated on a decagram scale. After quantitative recovery of the catalyst and purification, 5.2 g of (−)-ambrox (52% yield) with 92:8 d.r. and 95:5 e.r. and partially cyclized products **5** and **10** in 47% yield (Fig. 2c) were obtained. This corresponds to a theoretical volumetric productivity of 296 g l^−1^ in 20 h reaction time. Current optimized biocatalytic production processes of (−)-ambrox achieve full conversion of 300–450 g l^−1^ (3*E*,7*E*)-homofarnesol in 72 h (refs. ^25–29^). Several features of the biocatalytic process render it inherently sustainable, such as the use of bio-sourced (*E*)-β-farnesene produced from sugar fermentation, the use of water as a benign solvent and the convenient isolation of (−)-ambrox, which crystallizes directly from the reaction mixture. The theoretical volumetric productivity of the chemical route developed in this work is comparable to the enzymatic process and is achieved in a shorter reaction time. However, the need for a purification step to separate the catalyst and the side products from (−)-ambrox provides opportunities for further improvement. Owing to the environmental concerns about (per)fluorinated solvents, the solvent was recovered by simple distillation and another gram-scale experiment using recycled PFTB, and the quantitatively recovered IDPi catalyst **8g** furnished (−)-ambrox without deterioration of yield and diastereo- and enantioselectivity. The recycling experiments demonstrate that both the catalyst and the solvent can be readily recovered and reused, thereby increasing the sustainability of the approach. Finally, the method was also applied in an asymmetric synthesis of the sesquiterpene lactone natural product (+)-sclareolide, which is a key intermediate in several industrial syntheses of ambrox^5,7,54^. Subjecting (3*E*,7*E*)-homofarnesic acid, which is readily available on a technical scale from (*E*)-nerolidol^54^, to the reaction conditions in PFTB furnished the desired lactone natural product **12** in 46% yield, >7:1 d.r. and 94:6 e.r. (Fig. 2d).

## Mechanistic studies

**Fig. 3:**
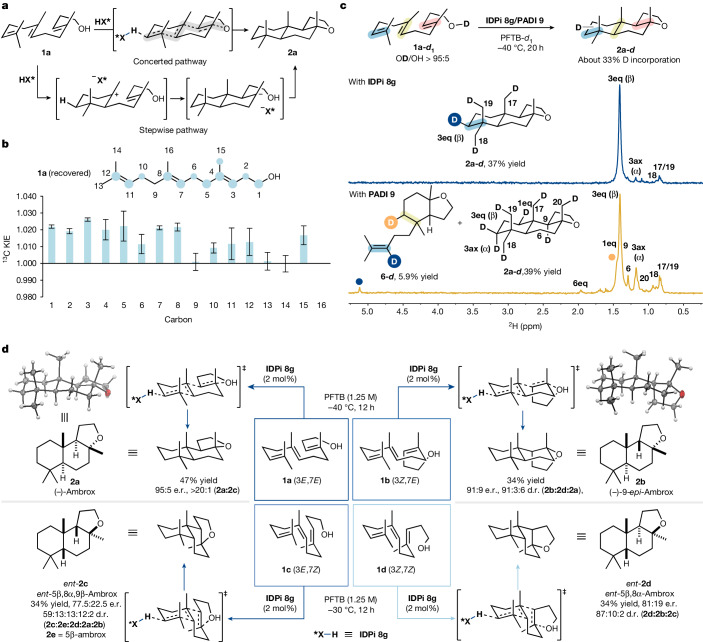
Mechanistic studies. **a**, Mechanistic scenarios for a concerted and a stepwise pathway. **b**, ^13^C KIEs observed at natural abundance in recovered **1a**. The experiment was performed in duplicate and the standard deviation is shown. **c**, Deuterium-labelling study in PFTB-*d*_1_ at −40 °C using (*S*,*S*)-IDPi catalyst **8g** or (*S*)-PADI catalyst **9** with the respective ^2^H{^1^H} NMR spectrum of the tricyclic ether fraction. **d**, Polyene cyclization of all possible homofarnesol diastereomers to the corresponding tricyclic ethers and rationalization of the obtained products by the respective transition states according to the Stork–Eschenmoser hypothesis.

To gain insights into the mechanism of the developed polyene cyclization and to investigate the origin of the high chemo-, diastereo- and enantioselectivity, several experiments were conducted. Different mechanistic scenarios for the formation of tricycle **2a** from polyene **1a** are conceivable. Construction of the tricyclic ring system from the linear polyene could proceed either by a concerted cyclization cascade or an entirely stepwise process by the formation of monocyclic and bicyclic intermediates (Fig. 3a).

Inspired by the kinetic isotope effect (KIE) studies at natural abundance to probe the concerted nature of the Diels–Alder cycloaddition^55,56^, we wondered whether a similar experiment could also provide insights into the nature of the polyene cyclization. KIEs would be expected at all double bond positions in case of a concerted polyene cyclization, as each bond formation would be involved in the rate-determining step. Small but statistically significant ^13^C KIEs were observed at each double bond of the recovered (3*E*,7*E*)-homofarnesol in two independent experiments (96% and 91% conversion at −40 °C) suggesting that protonation and C–C bond formation occur simultaneously (Fig. 3b). Next, deuterium-labelling studies were carried out to further clarify the mechanism of the IDPi-catalysed polyene cyclization and to investigate the selectivity of the initial protonation event. A single deuteration site^57^ would be expected in case of a concerted polyene cyclization, whereas multiple labelled positions would be characteristic of a stepwise process. Essentially, a single deuteration site (position 3eq) was observed by comparison of the ^2^H{^1^H} and ^1^H NMR spectra of the recovered product **2a** from the IDPi-catalysed polyene cyclization at −40 °C (Fig. 3c). This suggests that most of the (−)-ambrox formed at low temperature (−40 °C) in the presence of IDPi **8g** is generated by a concerted polyene cyclization. The proportion of (−)-ambrox formed by a stepwise pathway from γ-cyclohomofarnesol **3c** (as confirmed by deuterium incorporation at C-17) at −40 °C was estimated to be less than 5% according to ^2^H{^1^H} NMR spectroscopy (see Supplementary Information for details). The high selectivity for the equatorial position (3eq) is indicative of a stereospecific antiparallel addition onto the internal olefin, as proposed in refs. ^8–10^. Another important observation is the absence of any side products resulting from protonation or deuteronation of either internal or proximal double bond of (3*E*,7*E*)-homofarnesol. In comparison, product **2a** isolated from the PADI-catalysed polyene cyclization shows deuterium incorporation at multiple sites, some of which are diagnostic of a stepwise process. Furthermore, bicyclic side product **6** resulting from unproductive protonation of the internal double bond co-eluted with the tricyclic ether fraction and was identified by ^1^H and ^2^H NMR spectroscopies. This side product was not observed in the IDPi-catalysed polyene cyclization thus corroborating the high selectivity of the IDPi catalyst for productive protonation of the distal double bond.

The concerted nature of the IDPi-catalysed polyene cyclization was further studied by subjecting all possible diastereomers of homofarnesol **1** to the reaction conditions (Fig. 3d). For each diastereomer, the formation of the respective main isolated product can be rationalized with the Stork–Eschenmoser hypothesis^37^, in line with a predominantly concerted polyene cyclization. An increase in reaction temperature to −30 °C was necessary to convert (3*E*,7*Z*) and (3*Z*,7*Z*)-homofarnesol (**1c** and **1d**). We propose that access of sterically congested substrates such as cyclohomofarnesols **3** and (7*Z*)-isomers of homofarnesol (**1c** and **1d**) to the confined active site is precluded at lower temperatures.

**Fig. 4:**
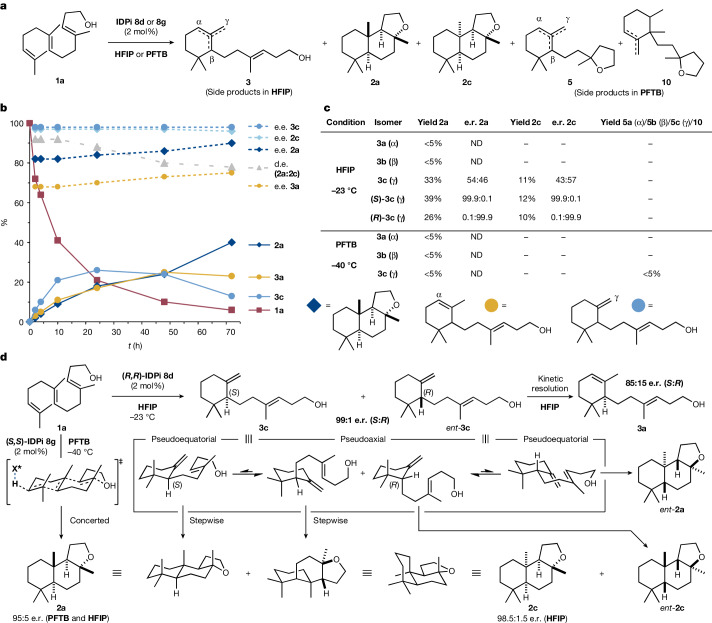
Origin of diastereo- and enantioselectivity, reactivity of monocyclic intermediates and mechanistic proposal. **a**, Identified intermediates and products in the reaction of **1a** to **2a** in HFIP or PFTB. **b**, Yield, enantiomeric excess (e.e.) and diastereomeric excess (d.e.; **2a**:**2c**) over time in HFIP/1*H*,1*H*-perfluorooctan-1-ol at −23 °C according to gas chromatography and HPLC analysis. **c**, Reactivity of cyclohomofarnesols **3** in HFIP and PFTB. **d**, Mechanistic proposal for the formation of **2a** and its diastereomer **2c** in HFIP/1*H*,1*H*-perfluorooctan-1-ol and PFTB.

To understand the origins of the different diastereo- and enantioselectivity observed in HFIP and PFTB (Figs. 2a and 4a), the reaction progress, as well as the enantiomeric and diastereomeric ratios, were monitored by gas chromatography and high-performance liquid chromatography (HPLC) over time (Fig. 4b). Whereas the enantioselectivity and diastereoselectivity remained essentially constant (95:5 e.r. and >20:1 d.r. for **2a**) in PFTB, an increase of the enantiomeric ratio of product **2a** was observed in HFIP on the progression of the reaction (91:9 → 95:5 e.r.). This was accompanied by a decrease in diastereoselectivity (**2a**:**2c** > 20:1 → 89:11). Cyclohomofarnesol intermediates **3a** and **3c** were also detected. The ratio of monocycles **3a**:**3c** changed from approximately 1:2 to 2:1 over the course of the reaction indicating that the kinetic exocyclic double bond product **3c** isomerizes to the thermodynamic endocyclic double bond isomer **3a** (ref. ^42^). The enantiomeric ratio of α-isomer **3a** was substantially lower (16:84 e.r.) in comparison with that of γ-isomer **3c** (1:99), suggesting a kinetic resolution. 5β,8α,9β-Ambrox (**2c**) was obtained in a similarly high enantiomeric ratio (1.5:98.5) throughout the reaction, indicating its formation by protonation of the axial conformer of **3c**. By contrast, the increase in the enantiomeric ratio of ambrox over time can be rationalized by protonation of the equatorial conformer of monocycle **3c** and formation of practically enantiopure ambrox, improving the overall enantiomeric ratio. Thus, protonation of both equatorial and axial conformers of essentially enantiopure γ-cyclohomofarnesol **3c** is probably responsible for the observed increase in enantioselectivity and concomitant drop in diastereoselectivity. In comparison with the observations in HFIP, the particularly high diastereoselectivity towards **2a** observed in PFTB at −40 °C seems to be the consequence of a highly concerted polyene cyclization and suppression of a stepwise pathway. To probe this hypothesis, the reactivity of the monocyclic intermediates was investigated by subjecting the individual isomers (**3a**, **3b** and **3c**) to the reaction conditions (Fig. 4c). Solely γ-cyclohomofarnesol **3c** showed appreciable conversions towards ambrox isomers **2a** and **2c** in HFIP, whereas α- and β-isomers **3a** and **3b** provided less than 5% of tricyclic ethers. When monocycles **3a**, **3b** and **3c** were used as substrates in PFTB at −40 °C, less than 5% conversion was observed. This result is in line with the deuterium-labelling experiment (Fig. 3c), showcasing that protonation of either monocyclic intermediate in a stepwise pathway does not occur to an appreciable extent at −40 °C. At −30 °C, partially cyclized **5** and isomerized **10** were obtained as the main products (see Supplementary Information for details), indicating that the confined active site of IDPi preferentially selects the more accessible double bond in the proximity of the alcohol over the congested cyclohexene. It is important to note that side products **5** and **10** predominantly originate from the protonation of monocyclic intermediates rather than from the undesired protonation of the proximal double bond of (3*E*,7*E*)-homofarnesol. The low amounts of **5** and **10** (<5%) observed at −40 °C underline this notion.

On the basis of the mechanistic studies conducted here, we suggest that the key to attaining the observed high diastereo- and enantioselectivity with catalyst **8g** is the preference of a concerted reaction pathway over a stepwise process (Fig. 4d). The scalable catalytic asymmetric polyene cyclization of (3*E*,7*E*)-homofarnesol to the important ambergris odorant (−)-ambrox reported in this study addresses a longstanding challenge in chemical synthesis and provides the desired product in unprecedented stereoselectivities. We believe that the method will find widespread application in related polyene cyclizations and may expedite the asymmetric synthesis of natural products, odorants and pharmaceuticals from readily available achiral starting materials.

## Online content

Any methods, additional references, Nature Portfolio reporting summaries, source data, extended data, supplementary information, acknowledgements, peer review information; details of author contributions and competing interests; and statements of data and code availability are available at 10.1038/s41586-024-07757-7.

### Supplementary information

Supplementary Information The file contains Supplementary Methods, detailed experimental procedures and Supplementary Mechanistic Discussions. Computational methods with *XYZ* coordinates, HPLC and gas chromatography traces, and tabulated X-ray crystallographic data are also included as well as Supplementary Figs. 1–95 and Supplementary Tables 1–37.

Supplementary Data This file contains the NMR spectra data.

## Data Availability

The experimental procedures and analytical data supporting the findings of the study are available in the manuscript and the Supplementary Information. Crystallographic data for compounds (−)-**2a**, (−)-**2b**, (±)-**12a**, (−)-**12b**, (±)-**12b**, (±)-**12d** and (−)-**12f** are provided in the Supplementary Information and are available free of charge from the Cambridge Crystallographic Data Centre (CCDC) under the deposition numbers CCDC-2338446: (−)-**2a**, CCDC-2338448: (−)-**2b**, CCDC-2338449: (±)-**12a**, CCDC-2338450: (−)-**12b**, CCDC-2338445: (±)-**12b**, CCDC-2338444: (±)-**12d** and CCDC-2338447: (−)-**12f**.
