# Supplementary material for: The catalytic asymmetric polyene cyclization of homofarnesol to ambrox
Source: Nature. 2024 Jul 31;632(8026):795–801. doi: 10.1038/s41586-024-07757-7 (PMC11338820; doi:10.1038/s41586-024-07757-7)
Supplement: Supplementary file 1 — The file contains Supplementary Methods, detailed experimental procedures and Supplementary Mechanistic Discussions. Computational methods with XYZ coordinates, HPLC and gas chromatography traces, and tabulated X-ray crystallographic data are also included as well as Supplementary Figs. 1–95 and Supplementary Tables 1–37. [file 41586_2024_7757_MOESM1_ESM.pdf]

nature portfolio

https://doi.org/10.1038/s41586-024-07757-7

Supplementary information

The catalytic asymmetric polyene cyclization of homofarnesol to ambrox

In the format provided by the authors and unedited

Supplementary Information

The catalytic asymmetric polyene cyclization of homofarnesol to ambrox

Na Luo[1,4], Mathias Turberg[1,4], Markus Leutzsch[1], Benjamin Mitschke[1], Sebastian Brunen[1], Vijay N. Wakchaure[1], Nils Nöthling[1], Mathias Schelwies[2], Ralf Pelzer[3], Benjamin List[1]*

[1]Max-Planck-Institut für Kohlenforschung, Kaiser-Wilhelm-Platz 1, D-45470 Mülheim an der Ruhr, Germany. [2]BASF SE, Synthesis and Homogeneous Catalysis, Carl-Bosch Straße 38, D-67056 Ludwigshafen, Germany. [3]BASF SE, New Business Development Aroma Ingredients, Carl-Bosch Straße 38, D-67056 Ludwigshafen, Germany. [4]These authors contributed equally: Na Luo, Mathias Turberg. e-mail: list@kofo.mpg.de

The PDF file includes:

Materials and Methods
Supplementary Text
Figs. S1 to S95
Tables S1 to S37
References S1–S63

Table of Contents

1 Materials and Methods 4
1.1 General Information 4
1.2 Synthesis of Substrates 9
1.2.1 Synthesis of Homofarnesol Diastereomers 9
1.2.2 Synthesis of Cyclohomofarnesols 14
1.3 Synthesis of Product Standards 26
1.4 Catalyst Synthesis 42
1.4.1 Synthesis of Imidodiphosphorimidate (IDPi) Catalysts 42
1.4.2 Synthesis of Phosphoramidimidate (PADI) Catalyst 9 50
1.5 Catalytic Asymmetric Polyene Cyclization towards Ambrox 51
1.5.1 Reaction Development 51
1.5.2 Scale-up Experiments 57
1.5.3 Catalyst and Solvent Recycling Experiment 80
1.5.4 Supplementary Discussion on the Ecological Properties of HFIP and PFTB 83
1.6 Polyene Cyclization of Homofarnesol Diastereomers 84
1.6.1 General Procedure 84
1.6.2 Synthesis of (−)-9-*epi*-Ambrox 91
1.7 Polyene Cyclization of Technical (3*E/Z*,7*E*)-Homofarnesol (1a/1b) 93
1.8 Catalytic Asymmetric Polyene Cyclization towards Sclareolide 96
1.8.1 Synthesis of (3*E*,7*E*)-Homofarnesic Acid 96
1.8.2 Polyene Cyclization of (3*E*,7*E*)-Homofarnesic Acid to Sclareolide 97
2 Mechanistic Studies 100
2.1 Preliminary Natural Abundance Kinetic Isotope Effect (KIE) Studies 100
2.1.1 Natural Abundance KIE Measurements of (3*E*,7*E*)-Homofarnesol (1a) 101
2.1.2 KIE Calculation 103
2.1.3 Discussion of the KIE Results 108
2.2 Deuterium Labeling Studies 109
2.2.1 Synthesis of (3*E*,7*E*)-Homofarnesol-d_1 109
2.2.2 General Procedure for the Deuterium Labeling Experiments 112
2.2.3 IDPi-Catalyzed Polyene Cyclization in PFTB-d_1 at −40 °C 113
2.2.4 PADI-Catalyzed Polyene Cyclization in PFTB-d_1 at −40 °C 123
2.2.5 Discussion of the Results 127
2.3 Kinetic Studies 128
2.3.1 Qualitative NMR Study: Time-Dependent Conversion in HFIP 128
2.3.2 GC/HPLC Study: Time-Dependent Conversion in HFIP 132

2.4 Reactivity of Cyclohomofarnesols ... 134
2.4.1 General Procedure ... 134
2.4.2 Reactivity of Cyclohomofarnesols in HFIP ... 134
2.4.3 Reactivity of Cyclohomofarnesols in PFTB ... 138
2.4.4 Summary and Supplementary Mechanistic Discussion ... 140
2.5 Investigation of the Solvent Effect on Substrate Conformation ... 143
2.6 Assignment of the Absolute Configuration ... 145
3 Computational Methods ... 149
3.1.1 Computational Details ... 149
3.1.2 XYZ Coordinates ... 149
3.1.3 Supplementary Discussion ... 154
4 Copies of GC Traces ... 156
5 Copies of HPLC Traces ... 192
6 X-Ray Crystallographic Data ... 208
7 References ... 250

1 Materials and Methods

1.1 General Information

All air and/or moisture sensitive reactions were conducted under inert gas atmosphere (argon) using standard Schlenk techniques, unless otherwise stated. Glassware (flasks and reaction vials) was stored in an oven at 80 °C overnight and/or flame-dried or dried using a heat-gun (650 °C) under high vacuum ($1 \cdot 10^{-3}$ mbar) prior to use (the reaction apparatus was backfilled with argon and the procedure was repeated at least two times). Teflon reaction vessels were dried in an oven at 80 °C and heated with a heat-gun (120 °C) under high vacuum. The addition of liquids, solvents and solutions of compounds was performed with argon-flushed (3 ×) LuerLock® or Hamilton® syringes, or oven-dried (80 °C) stainless steel or polyethylene cannulas through a rubber septum or under counterflow of argon. Solid compounds were added under counterflow of argon. Unless otherwise stated, all reactions were magnetically stirred using PTFE- or glass-coated magnetic stir bars and monitored by TLC, LC/MS, GC/MS or NMR spectroscopy. Room temperature (r.t.) refers to an average temperature of typically 23 °C. Reactions at lower temperatures (T < r.t.) were cooled to the specified temperature using appropriate cooling baths or cryostats, respectively. The reaction vessel was immersed in cooling baths inside a Dewar vessel, filled with ice/water (T = 0 °C), ice/acetone (T = −10 °C), dry-ice/acetone (the amount of dry-ice was adjusted to reach the desired specified temperature −10 °C ≥ T ≥ −78 °C), dry-ice/propan-2-ol (−77 °C), or dry-ice/ethanol (−72 °C). Alternatively, the reaction vessel was placed in an aluminum block inside a cryostat set to the desired temperature. Reactions requiring elevated temperatures were heated to the specified temperature using silicon oil baths (r.t. ≤ T ≤ 180 °C), aluminum heating blocks or sand baths (T ≥ 180 °C). Reaction temperatures correspond to the temperature of the external water/oil/sand bath or cooling bath temperature unless stated otherwise. The temperature of large scale reactions was monitored with an internal thermometer. Solvents were removed by rotary evaporation at 40 °C under reduced pressure (10 mbar) using a Büchi rotary evaporator (Rotavapor® R-300) connected to a *vacuubrand* pump followed by drying under high vacuum ($1 \cdot 10^{-3}$ mbar) at ambient temperature. Yields refer to isolated yields of chromatographically and spectroscopically (NMR, >95%) pure materials unless otherwise stated.

Solvents

Solvents were dried by distillation from appropriate drying agents[1] in the technical department of the Max-Planck-Institut für Kohlenforschung and received in Schlenk flasks under an atmosphere of argon. Dry solvents were stored over dry 4 Å molecular sieves except for (deuterated) methanol, ethanol and propan-2-ol which were stored over 3 Å molecular sieves for at least 72 h prior to use.[2] Absolute anhydrous acetone, acetonitrile, benzene, cyclohexane, DMF, DME, DMSO, NMP, trifluorotoluene, pyridine, di(n-butyl)ether, 1,4-dioxane, MTBE and DCE were purchased from Sigma-Aldrich and used as received. Deaeration of solvents was performed by sparging with argon for at least 10 min whilst stirring or by using the freeze-pump-thaw method (3 cycles).

Chemicals

Chemicals were purchased from commercial vendors (ABCR, Acros Organics, Alfa Aesar, Apollo Scientific, Fluorochem, Manchester Organics, Sigma-Aldrich, TCI) and used without further purification unless otherwise noted. Triethylamine, diisopropylamine and diisopropylethylamine were distilled from CaH_2 under argon atmosphere prior to use.

Inert gas

Dry argon (>99.5% purity) was purchased from Air Liquide.

Thin-Layer Chromatography (TLC) and Preparative TLC (pTLC)

Monitoring reactions, analysis of column fractions and determination of retardation factors (R_f values) was performed by thin-layer chromatography on glass or plastic plates coated with silica gel 60 or aluminum oxide (0.20 mm) containing a F_{254} fluorescence indicator from Macherey-Nagel. Qualitative analysis and visualization was accomplished by irradiation with UV light at λ = 254 nm and/or by immersion in different staining solutions (specified for each compound in the respective experimental procedure) followed by heating with a heat-gun at 300 °C until dryness. The following stains were typically used and prepared as described below:

- **Cerium ammonium molybdate (CAM) stain**
  $Ce(SO_4)_2$ (cerium sulfate: 5.0 g) and $(NH_4)_6Mo_7O_{24} \cdot 4\ H_2O$ (ammonium molybdate 25.0 g) were dissolved in H_2O (450 mL) and concentrated H_2SO_4 (50 mL)
- **Phosphomolybdic acid (PMA)**
  Phosphomolybdic acid (10 g) was dissolved in ethanol (100 mL)
- **$KMnO_4$ stain**
  $KMnO_4$ (1.5 g) and K_2CO_3 (10 g) were dissolved in H_2O (200 mL) prior to the addition of 10% aq. NaOH (1.25 mL)
- **Bromocresol green stain (for carboxylic acids)**
  $KMnO_4$ (1.5 g) and K_2CO_3 (10 g) were dissolved in H_2O (200 mL) prior to the addition of 10% aq. NaOH (1.25 mL)

Both CAM and PMA stain were found to be particularly suitable for visualization of the tricyclic lactones or ethers prepared in this work.

Preparative thin-layer chromatography was accomplished on silica gel 60 glass plates SIL G-25 UV_{254} and SIL G-100 UV_{254} from Machery-Nagel coated with 0.25 mm (for amounts ≤ 20 mg) and 1.0 mm silica gel (for amounts > 20 mg), respectively.

(Flash) Column Chromatography

(Flash) Column chromatography was performed using silica gel (60 Å, 230−400 mesh, particle size: 43−63 µm) from Merck or aluminum oxide (neutral, activated, Brockmann activity grade I; activity grade II with 3 wt% H_2O; activity grade III with 6 wt% H_2O) from Sigma-Aldrich using technical grade solvents. The solvent mixtures and volume ratios (*v/v*) used as mobile phase for chromatography are specified in the corresponding experiment. Flash column chromatography was performed in glass columns by applying slightly elevated air or argon (0.3 bar) pressure.[3] Automated column chromatography was conducted on a Biotage Isolera Spektra Four system, using SNAP Ultra HP-Sphere 25 µm cartridges or SNAP Ultra C18 HP-Sphere 25 µm reversed phase cartridges.

Preparative High Performance Liquid Chromatography

Preparative high performance liquid chromatography (Prep-HPLC) was performed on a Shimadzu LC-20AP (SIL-20A HT autosampler, CTO-20AC column over, SPD-20A diode array detector, FRC-10A fraction collector), Shimadzu LC-20AR (SIL-20A HT autosampler, CTO-20AC column over, SPD-20A diode array detector, FRC-10A fraction collector), Shimadzu LC-8A (CTO-10AC column over, SPD-10VP diode array detector, FRC-10A fraction collector, SCL-10AVP controller) or Agilent Technologies 1260 Preparative Binary Pump (G7157A autosampler, 1260 diode array detector WR, 1290 Prep FC, 1260 Prep Valve FC). HPLC-grade solvents from Sigma-Aldrich were used as mobile phase. The respective stationary phase and employed solvent mixtures are specified for each experiment.

Nuclear Magnetic Resonance Spectroscopy (NMR)

^{1}H, ^{2}H, ^{13}C, ^{19}F, ^{31}P nuclear magnetic resonance (NMR) spectra were acquired on Bruker Avance™ NEO 600 MHz (equipped with a BBO CryoProbe), Avance™ III 600 MHz (equipped with a TCI CryoProbe), Avance™ III 500 MHz, or Avance™ III HD Nanobay 300 spectrometers in a suitable deuterated solvent. The respective measuring frequencies and utilized solvents are specified in the individual experiment. ^{1}H and ^{2}H chemical shifts are reported in ppm (δ) relative to tetramethylsilane (TMS) with the residual solvent resonance serving as the internal reference (δ 7.26 ppm for $CDCl_3$; δ 5.32 ppm for CD_2Cl_2, δ 2.05 ppm for acetone-d_6, δ 7.16 ppm for benzene-d_6, δ 2.09 ppm for toluene-d_8).[4,5] Data are provided as follows: chemical shift in ppm, resonance multiplicity (s = singlet, d = doublet, t = triplet, q = quartet, p = pentet, s = sextet, h = heptet, m = multiplet, br = broad), coupling constant J in Hz and integration/number of protons. ^{13}C chemical shifts are reported in ppm from tetramethylsilane (TMS) with the solvent resonance as the internal standard ($CDCl_3$ δ 77.16 ppm; CD_2Cl_2 δ 53.84 ppm; δ 206.26, 29.84 ppm for acetone-d_6, δ 128.60 ppm for benzene-d_6, δ 20.40 ppm for toluene-d_8). ^{13}C, ^{19}F, ^{31}P NMR spectra were referenced according to the respective Ξ-values (following the IUPAC recommendations from 2008) relative to the internal references set in ^{1}H NMR spectra (e.g. ^{19}F: $CFCl_3$, ^{29}Si: $SiMe_4$, ^{31}P: H_3PO_4, each 0.00 ppm).[6] Heteronuclear NMR spectra (^{2}H, ^{13}C, ^{19}F, ^{31}P) were generally acquired with broadband proton decoupling unless noted otherwise.

All spectra were recorded at 298 K unless otherwise noted and processed with MestReNova 15.0.0. Kinetic data were analyzed using the reaction monitoring plugin from Mestrelab. Multiplicity and coupling constants are reported as observed.

Infrared (IR) spectroscopy

IR spectra were acquired on a Perkin Elmer Spectrum Two FT-IR spectrometer on an UATR Two crystal plate in the neat state. Absorption bands are reported in wavenumbers (cm^{-1}) and intensities are provided as follows: br = broad, w = weak, m = medium, s = strong.

Mass Spectrometry (MS)

Electron impact (EI, 70 eV) mass spectrometry was performed on a Thermo Fisher Scientific ISQ 7000 Single Quadrupole GC-MS system (LRMS), Thermo Fisher Scientific Q Exactive GC Orbitrap GC-MS/MS system (LRMS and HRMS) or Finnigan MAT 95 (LRMS and HRMS). Chemical ionization (CI) mass spectrometry was performed on a Thermo Fisher Scientific Q Exactive GC Orbitrap GC-MS/MS system (LRMS and HRMS). Electrospray ionization (ESI) was performed on a Thermo Finnigan LTQ-FT Ultra (LRMS and HRMS) or Thermo Fisher Scientific Q Exactive Plus Hybrid Quadrupol-Orbitrap (LRMS and HRMS). For each respective experiment, the ionization method and mode of detection is and all masses are reported in atomic mass units divided by elementary charge (*m/z*) with an intensity normalized to the most intense peak.

Gas Chromatography (GC)

Gas Chromatography was performed on HP 6890 and 5890 Series instruments equipped with a split-mode capillary injection system and a flame ionization detector (FID) using hydrogen (H_2) as carrier gas. Enantiomeric ratios were determined by comparing the sample with an appropriate corresponding racemic mixture. The (chiral) stationary phases, mobile phases and detailed conditions are provided in the individual experiment. For quantitative GC-analysis of the reaction mixtures, the response factors of starting materials, identified intermediates, products and the internal standard were determined and the quantification was validated using the calibration curve method for each respective component.

High Performance Liquid Chromatography (HPLC)

High performance liquid chromatography was performed on a Shimadzu LC-20AD (SIL-20AC autosampler, DGU-20A5 degasser, CTO-20AC column oven, SPD-M20A diode array detector, CMB-20A controller) or a Shimadzu LC-20AB (SIL-20AC HT autosampler, DGU-20A5 degasser, CTO-20AC column oven, SPD-M20A diode array detector) using Daicel columns with a chiral stationary phase. All solvents used were HPLC-grade solvents purchased from Sigma-Aldrich. The chiral stationary phase and the respective mobile phase are indicated for each experiment.

Specific Rotations

Specific rotations $[\alpha]_{\lambda}^{T}$ were measured using a Rudolph RA AUTOPOL® IV Automatic Polarimeter at the indicated temperature T (value provided in °C) with a sodium lamp (sodium D line, $\lambda = 589$ nm) unless stated otherwise. Measurements were performed in acid resistant cells (cell length typically $l = 50$ mm unless otherwise noted). The concentration c of the sample is provided in g/(100 mL) and the respective solvent is specified in the individual experiment. The specific rotation is calculated according to equation (1).

$$[\alpha]_{\lambda}^{T} = \frac{100 \cdot \alpha}{l \cdot c} \tag{1}$$

Melting Points

Melting points (m.p.) were measured on a Büchi 540 melting point apparatus in open glass capillaries and are uncorrected.

X-Ray Crystallography

Single crystals suitable for X-ray diffraction were grown as specified in the respective experiment. X-ray crystal structure analyses were performed on a Bruker AXS Enraf-Nonius KappaCCD diffractometer with a FR591 rotating Mo-anode X-ray source Incoatec Helios focusing multilayer optics, a Bruker-AXS Kappa Mach3 with APEX-II detector and FR591 rotating anode X-ray source with Incoatec Helios mirrors, and a Bruker-AXS Kappa Mach3 with APEX-II detector and IμS microfocus Mo-anode X-ray source and Incoatec Helios mirrors. Data were faceindexed, absorption corrected, and scaled using the program SADABS (Bruker AXS, 2014). The structures were solved by SHELXT or SHELXS and refined using SHELXL, all programs from G. M. Sheldrick (Göttingen, 2014),[7–9] or OLEX2[10] and the NoSpherA2[11] routine. The X-ray crystal structure analyses were performed in the X-ray department of the Max-Planck-Institut für Kohlenforschung. Crystal structures were visualized and rendered using the program Mercury using version 4.2.0 developed by The Cambridge Crystallographic Data Centre (CCDC).[12]

Nomenclature

Nomenclature of compounds follows the suggestions proposed by the molecule editing program ChemDraw Professional 20.1.1 developed and distributed by PerkinElmer®. Certain tricyclic ethers and lactones are named according to the prevailing nomenclature used in the literature (i.e. the use of absolute stereodescriptors α and β, originally introduced for the nomenclature of steroids).[13]

1.2 Synthesis of Substrates

The individual homofarnesol diastereomers were synthesized according to reported literature procedures.[14,15]

1.2.1 Synthesis of Homofarnesol Diastereomers

Synthesis of (3*E*,7*E*)-homofarnesol (1a)

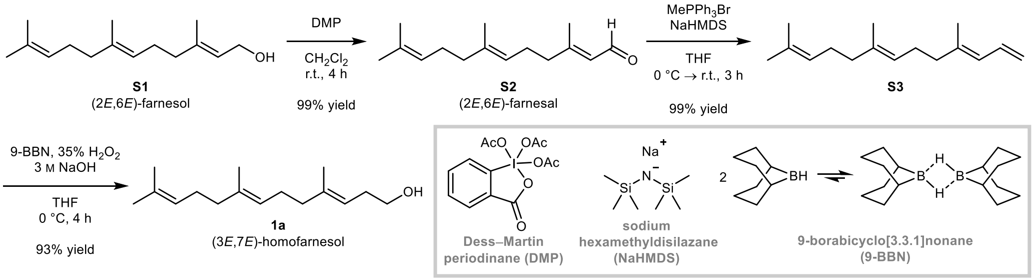

Following a procedure reported by Yamamoto et. al.:[14]

To a stirred mixture of commercially available (2*E,*6*E*)-farnesol (**S1**, 2.00 g, 9.0 mmol, 1 equiv.) in dry DCM (100 mL) under argon was added Dess–Martin periodinane (5.72 g, 13.6 mmol, 1.5 equiv.), the resulting mixture was stirred for 4 h. Saturated aqueous sodium bicarbonate solution (80 mL) was added dropwise, the mixture was stirred 10 min, then extracted with dichloromethane (3 × 50 mL). The organic layer was dried with $MgSO_4$, and the solvent was removed under reduced pressure. The crude product was purified by chromatography (isocratic elution with hexane/ethyl acetate 19:1 *v/v*) to give the product **S2** (1.98 g, 8.91 mmol, 99%) as a clear colorless oil. To a suspension of methyltriphenylphosphonium bromide (1.96 g, 5.50 mmol, 1.1 equiv.) in THF (20 mL) was added sodium bis(trimethylsilyl)amide (NaHMDS, 1.0 M solution in THF, 5.26 mL, 5.26 mmol, 1.05 equiv.) at −78 °C, then warmed to 0 °C. After reaching 0 °C, the mixture was stirred for 30 minutes, then cooled again to −78°C. To the reaction mixture was added crude (2*E,*6*E*)-farnesal (**S2**, 1.10 g, 5.00 mmol, 1.0 equiv.) in THF (5 mL). The resulting solution was warmed to room temperature, and stirred for 3 h before being quenched by the addition of MeOH (1 mL). The resulting mixture was concentrated, and diluted with Et_2O and hexanes (1:1 *v/v*). The majority of triphenylphosphine oxide was removed by filtration through silica gel. Evaporation of the filtrate under reduced pressure afforded 1.10 g of a yellow oil. Purification by chromatography on silica gel (isocratic elution with hexanes) furnished **S3** as a colorless oil (1.08 g, 4.94 mmol, 99%). To a solution of $BH_3 \cdot$ THF complex (1.0 M solution in THF, 5.05 mL, 5.05 mmol, 1.1 equiv.) was added 2-methyl-2-butene (2.0 M solution in THF, 5.50 ml, 11.0 mmol, 2.4 equiv.) at −30 °C. The solution was warmed to 0 °C and stirred for 2 h. To the resulting mixture was added a solution of **S3** (1.00 g, 4.58 mmol, 1 equiv.) in THF (5 mL) at 0 °C. The reaction mixture was stirred for 2 h at 0 °C and warmed to room temperature, where it was allowed to remain for an additional 12 h. Upon cooling the reaction mixture to −10 °C, 3 M aqueous NaOH (3 mL) and 30% H_2O_2 (2 mL) were added. The solution was stirred at room temperature for 3 h. The mixture was then poured into water and extracted with ethyl acetate. The organic layer was washed with water and brine, and dried over

anhydrous $MgSO_4$. Evaporation of ethyl acetate under reduced pressure yielded a colorless oil which was purified by chromatography on silica gel (hexanes/CH_2Cl_2 1:5 *v/v*), to give the product **1a** as a colorless oil (1.00 g, 93%).

NMR data for (3*E*,7*E*)-homofarnesol (**1a**):
^{1}H NMR (501 MHz, $CDCl_3$): δ (ppm) = 5.14–5.08 (m, 3H), 3.63–3.60 (t, *J* = 6.5 Hz, 2H), 2.31–2.27 (q, *J* = 6.5 Hz, 2H), 2.11–1.96 (m, 8H), 1.68 (s, 3H), 1.65 (s, 3H), 1.60 (s, 6H).
^{13}C NMR (126 MHz, $CDCl_3$): δ (ppm) = 139.1, 135.4, 131.5, 124.5, 124.1, 120.0, 62.6, 39.95, 39.85, 31.7, 26.9, 26.6, 25.8, 17.8, 16.4, 16.2.

The spectroscopic data of **1a** are in accordance with reported literature data.[14,15]

Alternative synthesis of (3*E*,7*E*)-homofarnesol (1a)

Isomerically pure (3*E*,7*E*)-homofarnesol (**1a**) can also be synthesized from (*E*)-nerolidol (**S4**) via the sequence outlined below. Reduction of (3*E*,7*E*)-isopropyl homofarnesate (**S5a**) obtained according to literature[16] affords **1a** in excellent yield and diastereomeric ratio.

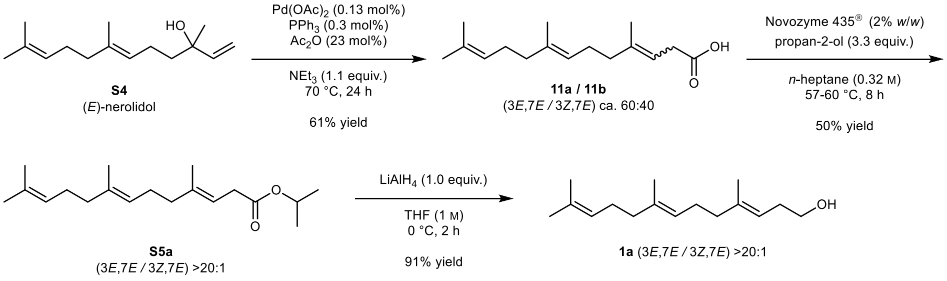

A flame-dried 250 mL round-bottom flask under argon was charged with isopropyl (3*E*,7*E*)-4,8,12-trimethyltrideca-3,7,11-trienoate (**S5a**, 7.31 g, 25.0 mmol, 1.0 equiv.) and a PTFE-coated magnetic stir bar. Dry THF (25 mL) was added and the resulting colorless solution was cooled to 0 °C. After 15 min, $LiAlH_4$ (1 M in THF, 25.0 mL, 25.0 mmol, 1.0 equiv.) was added dropwise and the resulting colorless reaction mixture was stirred for 2 h at 0 °C. After the elapsed time, a Fieser work-up[17] was performed: the reaction mixture was diluted with MTBE (25 mL), water (1 mL) was carefully added, followed by 15% aqueous NaOH (1 mL) and additional water (3 mL). The resulting cloudy solution was stirred at room temperature for 30 min. Anhydrous $MgSO_4$ was transferred to the mixture and the resulting colorless suspension was stirred for another 15 min. The suspension was filtered over a short pad of silica (d × l = 8 × 3 cm) and both flask and pad were rinsed with ethyl acetate (3 × 50 mL). The combined filtrate was concentrated under reduced pressure to afford the crude product as a colorless oil. Purification by flash column chromatography on silica gel using hexanes/MTBE as eluent (gradient elution 4:1 → 2:1 *v/v*) furnished (3*E*,7*E*)-homofarnesol (**1a**) as a colorless oil (>20:1 *E/Z*, 5.39 g, 22.8 mmol, 91% yield).

Physical state: colorless oil.

TLC (SiO_2, hexanes/MTBE 4:1, *v/v*): R_f = 0.31 (CAM stain).

^{1}H NMR (501 MHz, $CDCl_3$): δ (ppm) = 5.13 (tq, *J* = 7.2, 1.4 Hz, 1H), 5.11–5.06 (m, 2H), 3.61 (t, *J* = 6.5 Hz, 2H), 2.29 (q, *J* = 6.8 Hz, 2H), 2.14–2.07 (m, 2H), 2.05 (app q, *J* = 6.7 Hz, 4H), 1.98 (app dd, *J* = 9.1, 6.1 Hz, 2H), 1.68 (q, *J* = 1.4 Hz, 3H), 1.65 (s, 3H), 1.60 (s, 6H), 1.38 (s, 1H, OH).

^{13}C{^{1}H} NMR (126 MHz, $CDCl_3$): δ (ppm) = 139.1, 135.4, 131.5, 124.5, 124.1, 120.0, 62.6, 39.9, 39.8, 31.6, 26.9, 26.6, 25.8, 17.8, 16.4, 16.2.

IR (ATR, neat): $\tilde{\nu}_{max}$ (cm^{-1}) = 3330 (w, br; **OH**), 2965 (m), 2916, 1668 (w), 1441 (m), 1377 (m), 1046 (s), 835 (m).

HRMS (GC-EI$^+$): *m/z* calcd. for $C_{16}H_{28}O$ $[M]^+$: 236.213465, found: 236.213290.

Synthesis of (3*Z*,7*E*)-homofarnesol (1b)

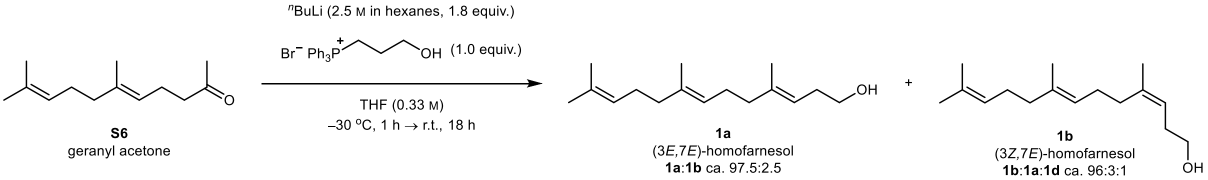

According to a previously reported procedure[15]: *n*-Butyllithium (2.5 M in hexanes, 7.2 mL, 18.0 mmol, 1.8 equiv.) was transferred dropwise to a stirred slurry of (3-hydroxypropyl-triphenylphosphonium bromide (4.00 g, 9.97 mmol, 1.0 equiv.) in THF (25 mL) at −30 °C under argon. The mixture warmed to room temperature and stirred for 3 h. The resulting dark red reaction mixture was cooled to −30 °C and a solution of geranyl acetone (**S6**, 1.94 g, 10.0 mmol, 1.0 equiv.) in THF (5 mL) was added dropwise. The resultant orange-beige mixture was stirred for 1 h at −30 °C and for additional 18 h at room temperature. The mixture was subsequently poured into 10% aqueous NH_4Cl (40 mL), brine was added, and the organic phase was separated. The aqueous phase was extracted with Et_2O (4 × 20 mL), and the combined organic layers were concentrated under reduced pressure. Hexanes were added to the residual oil, and the resulting solution was dried over Na_2SO_4, and filtered. The filtrate was concentrated and subjected to fractional distillation in vacuo to afford unreacted starting ketone (ca. 50% recovery) and a diastereoisomeric mixture of the product alcohol (ca. 1:1 3*E/Z*, 0.47 g, 1.99 mmol, 20% yield). The mixture was further purified by chromatography on silica gel eluting with hexanes/ethyl acetate (isocratic elution 19:1 *v/v*), to give the product **1b** as a colorless oil.

NMR data for (3*Z*,7*E*)-homofarnesol (**1b**; ratio **1b**:**1a**:**1d** ca. 96:3:1)

^{1}H NMR (501 MHz, $CDCl_3$): δ (ppm) = 5.14-5.06 (m, 3H), 3.61-3.58 (t, *J* = 6.6 Hz, 2 H), 2.29-2.25 (q, *J* = 6.6 Hz, 2 H), 2.07-1.95 (m, 8H), 1.72 (s, 3H), 1.67 (s, 3H), 1.59 (s, 6H).

^{13}C NMR (126 MHz, $CDCl_3$): δ 139.0, 135.5, 131.4, 124.4, 124.0, 120.8, 62.7, 39.8, 32.1, 31.5, 26.8, 26.6, 25.8, 23.6, 17.8, 16.1.

The NMR data are in agreement with the data reported in the literature.[15]

Synthesis of (3*E*,7*Z*)-homofarnesol (1c) and (3*Z*,7*Z*)-homofarnesol (1d)

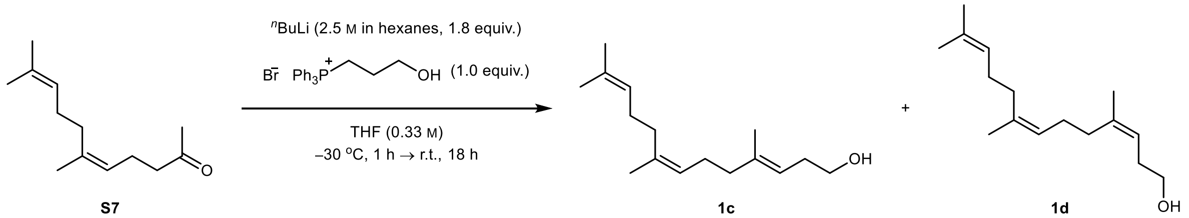

S7
neryl acetone

1c
(3*E*,7*Z*)-homofarnesol
1c:**1d**:**1a** ca. 91:8:1

1d
(3*Z*,7*Z*)-homofarnesol
1d:**1c**:**1b** ca. 94:5:1

According to a previously reported procedure[15]: *n*-Butyllithium (2.5 M in hexanes, 7.2 mL, 18.0 mmol, 1.8 equiv.) was transferred dropwise to a stirred slurry of (3-hydroxypropyl-triphenylphosphonium bromide (4.00 g, 9.97 mmol, 1.0 equiv.) in THF (25 mL) at −30 °C under argon. The mixture warmed to room temperature and stirred for 3 h. The resulting dark red reaction mixture was cooled to −30 °C and a solution of neryl acetone (**S7**, 1.94 g, 10.0 mmol, 1.0 equiv.) in THF (5 mL) was added dropwise. The resultant orange-beige mixture was stirred for 1 h at −30 °C and for additional 18 h at room temperature. The mixture was subsequently poured into 10% aqueous NH_4Cl (40 mL), brine was added, and the organic phase was separated. The aqueous phase was extracted with Et_2O (4 × 20 mL), and the combined organic layers were concentrated under reduced pressure. Hexanes were added to the residual oil, and the resulting solution was dried over Na_2SO_4, and filtered. The filtrate was concentrated and subjected to fractional distillation in vacuo to afford unreacted ketone (ca. 50% recovery) and a diastereoisomeric mixture of the product alcohols (ca. 1:1 3*E/Z*, 0.49 g, 2.07 mmol, 21% yield). Further purification of the mixture by column chromatography on silica gel eluting with hexanes/ethyl acetate (19:1 *v/v*, isocratic elution) furnished **1c** and **1d** as colorless oils.

NMR data for (3*E,*7*Z*)-homofarnesol (**1c**, ratio **1c**:**1d**:**1a** ca. 91:8:1)
^{1}H NMR (501 MHz, $CDCl_3$): δ (ppm) = 5.14–5.08 (m, 3H), 3.63-3.60 (t, *J* = 6.5 Hz, 2 H), 2.31–2.27 (q, *J* = 6.5 Hz, 2 H), 2.11–2.01 (m, 8H), 1.73 (s, 6H), 1.64 (s, 3H), 1.61 (s, 3H).
^{13}C NMR (126 MHz, $CDCl_3$): δ (ppm) = 139.0, 135.6, 131.7, 124.9, 124.4, 120.0, 62.5, 40.2, 32.1, 31.6, 26.7, 26.5, 25.8, 23.5, 17.7, 16.3.

NMR data for (3*Z,*7*Z*)-homofarnesol (**1d**; ratio **1d**:**1c**:**1a** ca. 94:5:1):
^{1}H NMR (501 MHz, $CDCl_3$): δ (ppm) = 5.15–5.10 (m, 3H), 3.62-3.60 (t, *J* = 6.5 Hz, 2 H), 2.30–2.26 (q, *J* = 6.5 Hz, 2 H), 2.08–2.04 (m, 8H), 1.73 (s, 3H), 1.69 (s, 6H), 1.61 (s, 3H).
^{13}C NMR (126 MHz, $CDCl_3$): δ (ppm) = 138.9, 135.7, 131.7, 124.9, 124.4, 120.9, 62.7, 32.4, 32.1, 31.5, 26.8, 26.5, 25.8, 23.7, 23.5, 17.8.

The NMR data are in accordance with data reported in the literature.[15]

1.2.2 Synthesis of Cyclohomofarnesols

Synthesis of (±)-(*E*)-α-cyclohomofarnesol

4-(2,6,6-trimethylcyclohex-2-en-1-yl)butan-2-one

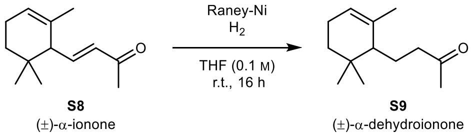

To a 250 mL round bottom flask was given (±)-α-ionone (**S8**, 5.40 mL, 5.00 g, 26.0 mmol, 1.0 equiv.) and THF (55 mL). Raney nickel (50% suspension in water, 1.3 g) was added, the reaction vessel was flushed with hydrogen, and stirred for 16 h at room temperature under an atmosphere of hydrogen whereupon complete conversion of (±)-α-ionone was observed. The mixture was filtered over a plug of Celite® 545 and the solids were discarded as aqueous solution. The filtrate was concentrated under reduced pressure and the crude mixture was purified via flash column chromatography on silica gel (isocratic elution with hexanes/MTBE 25:1 *v/v*) to yield (±)-α-dehydroionone (**S9**, 4.11 g, 21.2 mmol, 81%) as colorless oil.

^{1}H NMR (501 MHz, $CDCl_3$): δ (ppm) = 5.36–5.31 (m, 1H), 2.53–2.36 (m, 2H), 2.12 (s, 3H), 1.99–1.93 (m, 2H), 1.76 (ddt, *J* = 14.6, 9.9, 5.6 Hz, 1H), 1.66 (q, *J* = 1.9 Hz, 3H), 1.64–1.54 (m, 1H), 1.46 (t, *J* = 5.0 Hz, 1H), 1.43 (s, 1H), 1.15–1.11 (m, 1H), 0.91 (s, 3H), 0.86 (s, 3H).
^{13}C NMR (126 MHz, CD_2Cl_2): δ (ppm) = 209.3, 135.7, 121.2, 48.6, 43.9, 32.7, 31.7, 30.1, 27.8, 27.7, 24.5, 23.6, 23.1.

The analytical data is in agreement with the reported literature.[15,18]

(±)-(*E*)- and (±)-(*Z*)-α-cyclohomofarnesol (*E*- and *Z*-3a)

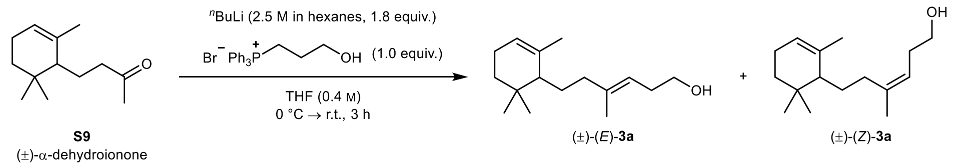

Following a modified literature procedure,[15] a flame-dried 25 mL Schlenk tube was charged with (±)-α-dehydroionone (**S9**, 4.13 g, 10.3 mmol, 1.0 equiv.). Anhydrous THF (14 mL) was added and the mixture was cooled to –30 °C. A solution of *n*-BuLi (2.5 M in hexanes, 7.4 mL, 18.5 mmol, 1.8 equiv.) was added dropwise over the course of 1 h, the mixture was then stirred for 1 h at –30 °C, then slowly warmed to room temperature and subsequently stirred at this temperature for 48 h. The mixture was then poured onto an aqueous solution of HCl (10%, 50 mL). Et_2O (50 mL) was added and the layers were separated. The aqueous layer was extracted with Et_2O (3 × 50 mL) and the combined organic layers were washed with brine and dried over Na_2SO_4. The solids were filtered off and the filtrate was concentrated in vacuo. The crude mixture was then purified via flash column chromatography on silica gel (gradient elution with hexanes/MTBE 10:1 → 7.5:1 *v/v*) to yield the desired homoallylic alcohols as colorless oils (isomerically enriched, *Z*-isomer: *E/Z*-ratio = 15:85, *E*-isomer: *E*/*Z*-ratio = 61:39). To isolate the pure *E*-isomer, the mixture was purified via preparative liquid chromatography (MC-Pack PVA-Sil, 250 mm, eluent: hexanes/propan-2-ol 99:1 *v/v*, 22.5 mL/min, 10.6 MPa, 308 K) to yield the desired isomer as colorless oil.

Spectroscopic data for the (±)-(*E*)-**3a**:

^{1}H NMR (501 MHz, $CDCl_3$): δ (ppm) = 5.30 (t, *J* = 3.5 Hz, 1H), 5.10 (t, *J* = 7.3 Hz, 1H), 3.62 (q, *J* = 6.1 Hz, 2 H), 2.28 (dtd, *J* = 7.6, 6.4, 1.2 Hz, 2 H), 2.13–2.01 (m, 2 H), 2.00–1.92 (m, 2 H), 1.73 (q, *J* = 1.3 Hz, 3 H), 1.70 (q, *J* = 1.9 Hz, 3 H), 1.52–1.28 (m, 6 H), 0.95 (s, 3 H), 0.87 (s, 3H).

The spectroscopic data is in agreement with the reported literature.[15]

Synthesis of (*E*)-β-cyclohomofarnesol (3b)

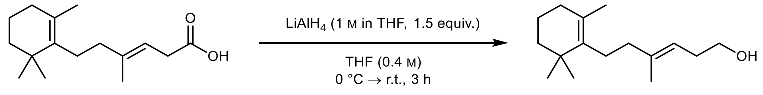

S10 **(*E*)-3b**

To a flame-dried Schlenk tube under argon was added $LiAlH_4$ (1.0 M solution in THF, 6.0 mL, 6.00 mmol, 1.5 equiv.) and the solution was diluted with THF (6 mL). The Schlenk tube was cooled to 0 °C and a solution of (*E*)-4-methyl-6-(2,6,6-trimethylcyclohex-1-en-1-yl)hex-3-enoic acid (**S10**, 1.00 g, 3.99 mmol, 1.0 equiv.) in THF (4 mL) was added dropwise. The mixture was allowed to warm to room temperature and subsequently stirred at this temperature for 3 h. Upon complete conversion of the starting material (detected via TLC analysis), the reaction was quenched via addition of a saturated solution of NH_4Cl (20 mL). The mixture was stirred at room temperature for 15 min, the formed solids were then filtered off and the layers of the filtrate were separated. The aqueous layer was extracted with MTBE (3 × 50 mL), the combined organic layers were washed with brine, dried over Na_2SO_4, and then concentrated under reduced pressure. The crude mixture was purified via flash column chromatography on silica gel (isocratic elution with hexanes/ethyl acetate 20:1 *v/v*) to afford (*E*)-β-cyclohomofarnesol (**3b**, 874 mg, 3.70 mmol, 93%) as colorless oil.

^{1}H NMR (501 MHz, CD_2Cl_2): δ (ppm) = 5.17 (ddt, *J* = 8.5, 7.3, 1.3 Hz, 1H), 3.58 (q, *J* = 6.3 Hz, 2H), 2.26 (dtd, *J* = 7.4, 6.5, 0.9 Hz, 2H), 2.11–2.02 (m, 5H), 1.94–1.89 (m, 2H), 1.69 (s, 3H), 1.61 (s, 3H), 1.60–1.54 (m, 3H), 1.45–1.36 (m, 3H), 1.00 (s, 6H).

^{13}C NMR (126 MHz, CD_2Cl_2): δ (ppm) = 134.0, 137.1, 127.2, 119.3, 62.7, 40.5, 40.0, 35.1, 32.9, 31.7, 28.8, 28.0, 20.0, 19.7, 16.4.

The acquired NMR data is in accordance with the data reported in the literature.[19]

Synthesis of (±)-(*E*)-γ-cyclohomofarnesol

Known approaches for the synthesis of (±)-(*E*)-γ-cyclohomofarnesol (**3c**) rely on the availability of γ-ionone which is subjected to a selective reduction of the conjugated double bond followed by a Wittig reaction of the formed γ-dehydroionone.[15] Unfortunately, γ-ionone was not commercially available at the time, therefore a new synthesis of isomerically pure (±)-(*E*)-**3c** from commercially available starting material was devised. The retrosynthetic analysis and route followed in this work are outlined below. In contrast to the Wittig approach, which affords 1:1 (*E*/*Z*)-mixtures, a tedious separation of the isomers can be circumvented due to the excellent diastereoselectivity of the sequence.

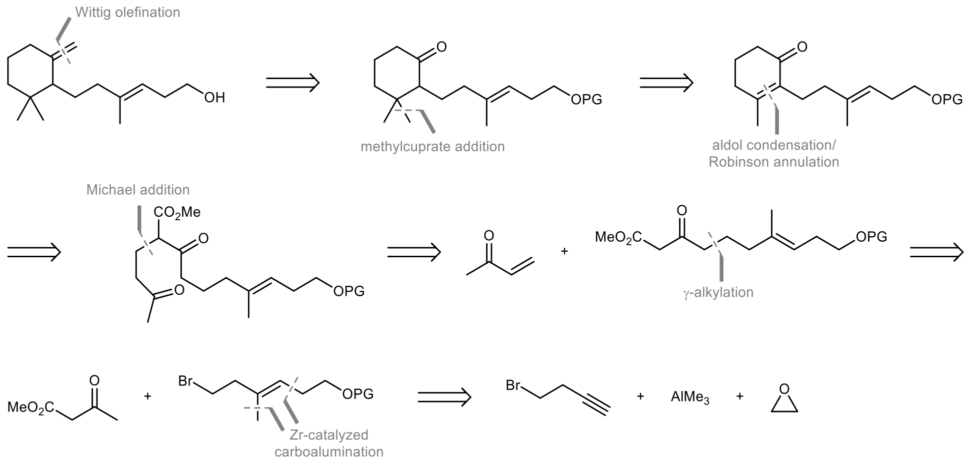

Scheme 1: Retrosynthetic analysis for the synthesis of (±)-(*E*)-γ-cyclohomofarnesol devised in this work. PG = protecting group.

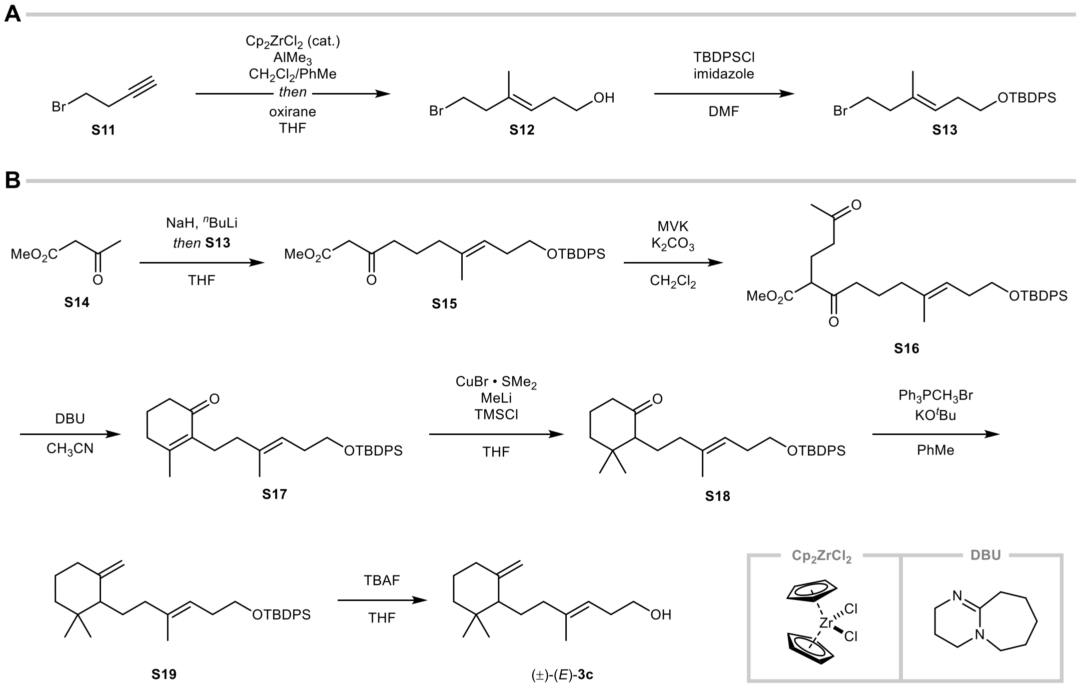

Scheme 2: Synthesis of (±)-(*E*)-γ-cyclohomofarnesol. A) Preparation of the homoallylbromide fragment. Reagents and conditions: a) Cp_2ZrCl_2 (25 mol%), $AlMe_3$ (2.5 equiv.), CH_2Cl_2/PhMe (1.2:1 *v/v*), 0 °C → r.t., 12 h, *then* oxirane (2.5–3.3 M in THF, 2.3 equiv.), 0 °C → r.t., 12 h; b) TBDPSCl, imidazole (1.1 equiv. each), DMF, r.t., 30 min. B) Completion of the synthesis. Reagents and conditions: a) NaH, ^{n}BuLi (1.1 equiv. each), THF, *then* **S13**, b) methyl vinyl ketone (MVK), K_2CO_3 (1.0 equiv. each), CH_2Cl_2, r.t., 16 h, c) DBU (1.2 equiv.), CH_3CN, reflux, d) CuBr · SMe_2 (1.5 equiv.), MeLi (2.1 equiv.), TMSCl (3.1 equiv.), THF, e) Ph_3PCH_3Br (4.0 equiv.), KO^{t}Bu, PhMe, reflux, f) TBAF (2.0 equiv.), THF, r.t., 1 h.

(*E*)-6-bromo-4-methylhex-3-en-1-ol (S12)

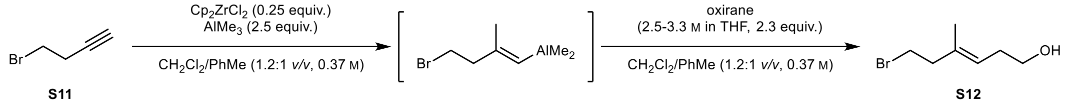

A flame-dried 250 mL two-neck round-bottom flask fitted with an argon outlet and a rubber septum under argon was charged with Cp_2ZrCl_2 (1.46 g, 4.99 mmol, 0.25 equiv.) and dry CH_2Cl_2 (20.0 mL). The resulting colorless solution was cooled to 0 °C and $AlMe_3$ (2.0 M solution in toluene, 25.0 mL, 18.8 g, 50 mmol, 2.5 equiv.) was added dropwise. The resulting yellow solution was stirred for 30 min at 0 °C, and a solution of 4-bromobut-1-yne (**S11**, 1.94 mL, 2.75 g, 20.1 mmol) in CH_2Cl_2 (10.0 mL, 2.0 M) was added dropwise over 10 min. After the addition was complete, the ice bath was removed and the reaction mixture was stirred at room temperature for 12 h. After the elapsed time, an aliquot of the yellow-orange reaction mixture (approx. 0.1 mL) was removed, treated with D_2O and analyzed by ^1H NMR spectroscopy. Analysis of crude reaction mixture indicated >95% deuterium incorporation in the *trans*-position thus confirming essentially quantitative conversion of the alkyne starting material to the desired alane. The yellow-orange solution was cooled to 0 °C and commercially available oxirane (2.5-3.3 M solution in THF, 23 mmol, 7.0 mL) was rapidly added in one portion (upon addition the yellow-orange color gradually changed to yellow). After stirring for 3 h at 0 °C, the ice bath was removed and the reaction mixture as stirred at room temperature overnight. As incomplete conversion was observed after 12 h at room temperature, an additional portion of oxirane (2.5-3.3 M solution in THF, 23 mmol, 7.0 mL) was transferred to the reaction mixture and the reaction mixture was stirred for further 12 h at room temperature. After the reaction was deemed complete according to ^1H NMR analysis, the pale yellow reaction mixture was carefully poured into an ice-cold aqueous saturated potassium sodium tartrate solution (200 mL). After vigorous stirring for 1 h, the mixture was filtered over a pad of Celite® 545 into a separatory funnel. The aqueous phase was extracted with CH_2Cl_2 (3 × 50 mL), the combined organic extracts were washed with brine, dried over Na_2SO_4, filtered, and concentrated under reduced pressure. Purification by flash column chromatography on silica gel eluting with hexanes/MTBE (gradient elution: 2:1 → 3:2 → 1:1 *v/v*) afforded **S12** (1.74 g, 8.99 mmol, 45% yield) as a colorless oil.

Physical state: colorless oil.
TLC (SiO_2, hexanes/MTBE 2:1, *v/v*): R_f = 0.18 (CAM stain).
^{1}H NMR (501 MHz, $CDCl_3$): δ (ppm) = 5.25 (tq, J = 7.4, 1.4 Hz, 1H), 3.65 (t, J = 6.4 Hz, 2H), 3.47 (t, J = 7.2 Hz, 2H), 2.57 (t, J = 7.1 Hz, 2H), 2.31 (q, J = 6.6 Hz, 2H), 1.67 (s, 3H), 1.48 (br s, 1H).
^{13}C{^{1}H} NMR (126 MHz, $CDCl_3$): δ (ppm) = 135.4, 123.8, 62.3, 42.8, 31.9, 31.6, 15.8.

(*E*)-((6-Bromo-4-methylhex-3-en-1-yl)oxy)(*tert*-butyl)diphenylsilane (S13)

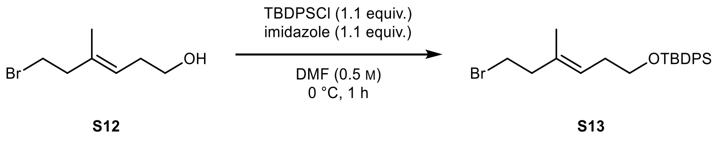

To a solution of **S12** (515 mg, 2.67 mmol, 1.0 equiv.) and imidazole (211 mg, 3.10 mmol, 1.1 equiv.) in dry *N,N*-dimethylformamide (DMF, 5.0 mL, 0.5 M) under an atmosphere of argon at 0 °C was added *tert*-butylchlorodiphenylsilane (TBDPSCl, 0.74 mL, 782 mg, 2.85 mmol, 1.1 equiv.). The resulting colorless solution was stirred at 0 °C for 1 h. After the elapsed time, the reaction mixture was diluted with water (10 mL) and Et_2O (10 mL). The organic phase was separated and the aqueous phase was extracted with Et_2O (3 × 10 mL). The combined organic layers were washed with water (10 mL) and brine (2 × 10 mL), dried over Na_2SO_4, filtered, and concentrated under reduced pressure. Purification by flash column chromatography on silica gel eluting with hexanes/MTBE (gradient elution: hexanes → hexanes/MTBE 39:1 → 19:1 → 9:1 *v/v*) afforded **S13** as a colorless oil (975 mg, 2.26 mmol, 85% yield).

Physical state: colorless oil.

TLC (SiO_2, hexanes/MTBE 19:1, *v/v*): R_f = 0.16 (UV: λ = 254 nm, $KMnO_4$ stain).

^{1}H NMR (501 MHz, CD_2Cl_2): δ (ppm) = 7.72–7.63 (m, 4H), 7.46–7.35 (m, 6H), 5.26 (tq, *J* = 7.3, 1.4 Hz, 1H), 3.67 (t, *J* = 6.8 Hz, 2H), 3.42 (t, *J* = 7.6 Hz, 2H), 2.53 (t, *J* = 7.5 Hz, 2H), 2.30 (q, *J* = 7.2 Hz, 2H), 1.60 (s, 3H), 1.04 (s, 9H).

^{13}C{^{1}H} NMR (126 MHz, CD_2Cl_2): δ (ppm) = 136.0, 134.5, 134.4, 130.0, 128.0, 124.3, 63.9, 43.4, 32.1, 31.9, 27.0, 19.5, 15.9.

^{1}H/^{29}Si HMBC NMR (501/99 MHz, CD_2Cl_2): δ (ppm) = 7.68/−4.9, 3.67/−4.9, 1.05/−4.9.

HRMS (ESI$^+$): *m/z* calcd. for $C_{23}H_{31}OSiBrNa^+$ $[M+Na]^+$: 453.121988, found: 453.121860.

Methyl (*E*)-10-((*tert*-butyldiphenylsilyl)oxy)-7-methyl-3-oxodec-7-enoate (S15)

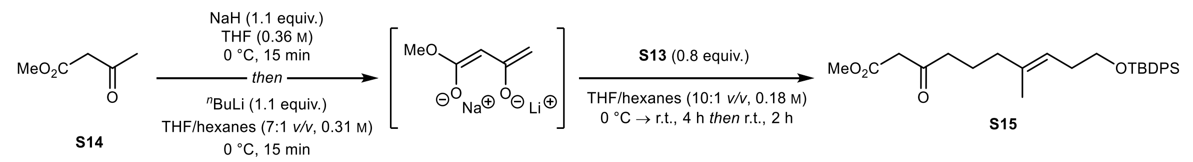

A flame-dried 50 mL two-neck round-bottom flask fitted with an argon outlet and a rubber septum was charged with sodium hydride (60% *w/w* suspension in mineral oil, 111 mg, 2.78 mmol, 1.1 equiv.). Dry THF (7 mL) was added and the resulting grey suspension was cooled to 0 °C. Neat methyl acetoacetate (**S14**, 0.27 mL, 291 mg, 2.50 mmol, 1.0 equiv.) was transferred dropwise via syringe to the stirred suspension, and the mixture was stirred for 15 min at 0 °C. Then, *n*-butyllithium (2.5 M solution in hexanes, 1.00 mL, 2.50 mmol, 1.0 equiv.) was added dropwise and the resulting bright orange solution was stirred for 15 min at 0 °C. After the elapsed time, a solution of **S13** in dry THF (1.0 mL, flask rinsed with additional 2 ×1.0 mL THF) was transferred to the stirred bright orange solution of the dianion. The resulting yellow-orange reaction mixture was allowed to warm to room temperature over 4 h and stirred for an additional 2 h at room temperature. Then, the reaction mixture was diluted with Et_2O (10 mL) and poured into ice-cold 0.5 M aqueous hydrochloric acid (50 mL). The organic layer was separated and the aqueous layer was extracted with Et_2O (3 × 20 mL). The combined organic extracts were washed with water (20 mL) and brine (20 mL), dried over Na_2SO_4, filtered, and concentrated under reduced pressure to afford the crude product as a yellow oil. Purification by flash column chromatography on silica gel using hexanes/ethyl acetate as eluent (gradient elution 9:1 → 4:1 *v/v*) afforded a less polar (R_f = 0.70, SiO_2; hexanes/ethyl acetate 4:1 *v/v*) fraction of leftover starting material (264 mg, 0.61 mmol, 31% yield) alongside the more polar desired γ-alkylated product **S15** (595 mg, 1.28 mmol, 64% yield, 92% based on recovered starting material) as an inconsequential mixture of keto-enol tautomers.

Physical state: colorless oil.

TLC (SiO_2, hexanes/ethyl acetate 4:1, *v/v*): R_f = 0.42 (UV: λ = 254 nm; $KMnO_4$ stain).

^{1}H NMR (501 MHz, $CDCl_3$): δ (ppm, major tautomer) = 7.70–7.63 (m, 4H), 7.45–7.35 (m, 6H), 5.12 (ddt, *J* = 10.0, 7.2, 1.4 Hz, 1H), 3.71 (s, 3H), 3.64 (t, *J* = 7.0 Hz, 2H), 3.40 (s, 2H), 2.47 (t, *J* = 7.3 Hz, 2H), 2.26 (q, *J* = 7.1 Hz, 2H), 1.97 (t, *J* = 7.4 Hz, 2H), 1.69 (p, *J* = 7.3 Hz, 2H), 1.53 (s, 3H), 1.05 (s, 9H).

^{13}C{^{1}H} NMR (126 MHz, $CDCl_3$): δ (ppm) = 202.8, 167.8, 136.2, 135.7, 134.2, 129.7, 127.7, 121.6, 63.9, 52.4, 49.2, 42.5, 38.9, 31.7, 27.0, 21.6, 19.3, 15.9.

^{1}H/^{29}Si HMBC NMR (501/99 MHz, $CDCl_3$): δ (ppm) = 7.70/−4.7, 3.64/−4.7, 1.04/−4.7.

HRMS (ESI$^+$): *m/z* calcd. for $C_{28}H_{38}O_4SiNa^+$ [M+Na]$^+$: 489.243158, found: 489.243100.

(*E*)-2-(6-((*tert*-Butyldiphenylsilyl)oxy)-3-methylhex-3-en-1-yl)-3-methylcyclohex-2-en-1-one (S17)

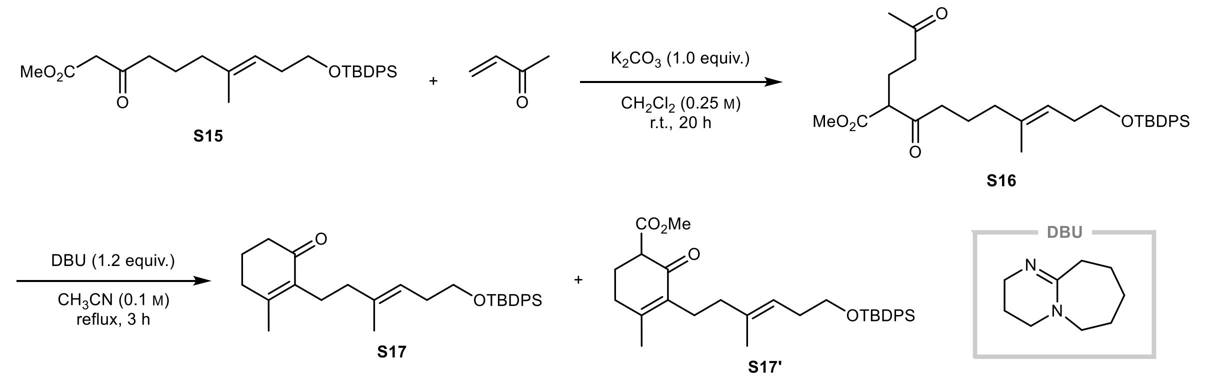

Construction of the required cyclohexanone was accomplished via a Michael addition/aldol condensation/decarboxylation sequence according to a slightly modified reported literature procedure for related substrates. An oven-dried 25 mL round-bottom flask was charged with anhydrous potassium carbonate (160 mg, 1.16 mmol, 1.0 equiv.) and **S15** (523 mg, 1.12 mmol, 1.0 equiv.). The flask was fitted with a rubber septum, evacuated and flushed with argon. A balloon filled with argon was attached and dry CH_2Cl_2 (3.5 mL) was added. In a separate vial under argon, 3-buten-2-one (0.11 mL, 95.0 mg, 1.22 mmol, 1.1 equiv.) was dissolved in dry CH_2Cl_2 (0.5 mL). The solution was added dropwise to the vigorously stirred suspension (vial was rinsed with 2 × 0.5 mL CH_2Cl_2) and the resulting colorless reaction mixture was stirred at room temperature for 18 h. After the elapsed time, water (10 mL) was added and the biphasic mixture was transferred to a separatory funnel. The organic phase was separated and washed with brine. The aqueous phase was extracted with Et_2O (3 × 10 mL). The combined organic extracts were washed with brine, dried over Na_2SO_4, filtered, and concentrated under reduced pressure to afford the crude product as a colorless oil, which was used in the subsequent step without further purification. Crude **S16** (601 mg, 1.12 mmol, 1.0 equiv.) was transferred to an oven-dried round bottom flask. The reaction vessel was fitted with a reflux condenser equipped with a rubber septum attached to a vacuum/argon line, and the apparatus was evacuated and flushed with argon. Dry acetonitrile (11 mL, 0.1 M) was added, followed by 1,8-diazabicyclo[5.4.0]undec-7-ene (DBU, 0.20 mL, 204 mg, 1.34 mmol, 1.2 equiv.), and the reaction mixture was immersed in a preheated sand bath at 90 °C. After refluxing the solution for 3 h, heating bath was removed. After cooling to room temperature, the reaction mixture was diluted with water (20 mL) and 1 M aqueous hydrochloric acid (20 mL). The aqueous phase was extracted with Et_2O (3 × 20 mL), the combined organic layers were dried over Na_2SO_4, filtered, and concentrated under reduced pressure. Purification by flash column chromatography on silica gel using hexanes/ethyl acetate as eluent (isocratic elution with 5:1 *v/v*) afforded the desired cyclohexenone **S17** (433 mg, 0.94 mmol, 84% yield over two steps) alongside more polar β-ketoester **S17'** (57.7 mg, 0.11 mmol, 9.9% yield).

Characterization data for cyclohexenone S17:

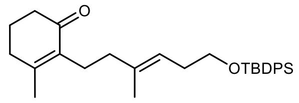

S17

Physical state: colorless oil.

TLC (SiO_2, hexanes/ethyl acetate 4:1, *v/v*): $R_f = 0.35$ (UV: $\lambda = 254$ nm, $KMnO_4$ stain).

^{1}H NMR (501 MHz, $CDCl_3$): δ (ppm) = 7.74–7.63 (m, 4H), 7.45–7.33 (m, 6H), 5.09 (tq, *J* = 7.2, 1.3 Hz, 1H), 3.61 (t, *J* = 7.2 Hz, 2H), 2.38–2.31 (m, 4H), 2.30–2.22 (m, 4H), 1.96–1.84 (m, 4H), 1.88 (s, 3H), 1.59 (s, 3H), 1.05 (s, 9H).

^{13}C{^{1}H} NMR (126 MHz, $CDCl_3$): δ (ppm) = 198.7, 155.3, 137.3, 135.7, 135.6, 134.2, 129.6, 127.7, 120.6, 63.9, 39.0, 38.0, 33.0, 31.7, 27.0, 24.2, 22.4, 21.3, 19.3, 16.3.

^{1}H/^{29}Si HMBC NMR (501/99 MHz, $CDCl_3$): δ (ppm) = 7.67/−4.7, 3.61/−4.7, 1.05/−4.7.

HRMS (ESI^+): *m/z* calcd. for $C_{30}H_{40}O_2SiNa^+$ $[M+Na]^+$: 483.26898, found: 483.26907.

(*E*)-2-(6-((*tert*-Butyldiphenylsilyl)oxy)-3-methylhex-3-en-1-yl)-3,3-dimethylcyclohexan-1-one (S18)

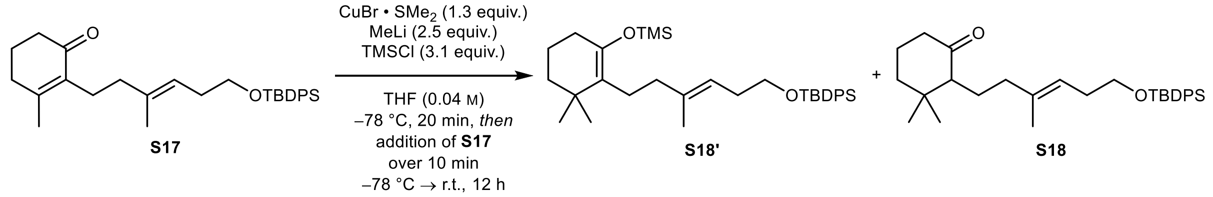

A flame-dried 50 mL two-neck round-bottom flask under argon was charged with copper(I) bromide dimethyl sulfide complex (CuBr · Me_2S, 214 mg, 1.04 mmol, 1.25 equiv.) and dry THF (10 mL). The resulting suspension was cooled to 0 °C and methyllithium (1.6 M in Et_2O, 1.30 mL, 2.08 mmol, 2.49 equiv.) was added dropwise to the slurry (upon addition a gradual change from a colorless slurry over a bright orange-yellow suspension to a colorless solution was observed indicating formation of the dimethylcuprate species). The resulting clear colorless solution was stirred at 0 °C for 15 min and subsequently cooled to −78 °C. Chlorotrimethylsilane (0.33 mL, 282 mg, 2.60 mmol, 3.1 equiv.) was added dropwise and the mixture was stirred for additional 5 min at −78 °C. A solution of cyclohexenone **S17** in THF (5 mL, flask rinsed with 2 × 2.5 mL) was added dropwise over 10 min (gradual color change from colorless over yellow to orange upon addition) and the resulting orange solution was allowed to warm to room temperature overnight. After 14 h, the dark brown solution was treated with aqueous saturated NH_4Cl (10 mL) and the resulting suspension was filtered over a pad of Celite® 545 into a separatory funnel eluting with CH_2Cl_2 (3 × 20 mL). Aqueous hydrochloric acid (0.5 M, 50 mL) was added, the biphasic mixture was vigorously shaken. The aqueous layer was extracted with CH_2Cl_2 (3 × 20 mL), the combined organic layers were dried over Na_2SO_4, filtered and concentrated under reduced pressure to afford the crude product as a pale yellow oil. Purification by flash column chromatography on silica gel using hexanes/MTBE as eluent (9:1 *v/v*, isocratic elution) furnished **S18** as a colorless oil (318 mg, 668 µmol, 80% yield). Flushing the column with MTBE provided residual starting material **S17** (54.1 mg, 117 µmol, 14% yield).

Physical state: colorless oil.

TLC (SiO_2, hexanes/MTBE 9:1, *v/v*): $R_f = 0.30$ (UV: λ = 254 nm, $KMnO_4$ stain)

^{1}H NMR (501 MHz, $CDCl_3$): δ (ppm) = 7.73–7.63 (m, 4H), 7.45–7.32 (m, 6H), 5.09 (tq, *J* = 7.1, 1.3 Hz, 1H), 3.63 (t, *J* = 7.0 Hz, 2H), 2.33–2.23 (m, 3H), 2.24–2.17 (m, 1H), 2.09–2.02 (m, 1H), 2.00–1.92 (m, 1H), 1.91–1.83 (m, 1H), 1.82–1.72 (m, 3H), 1.60–1.56 (m, 2H), 1.54 (s, 3H), 1.41–1.31 (m, 1H), 1.05 (s, 9H), 1.00 (s, 3H), 0.76 (s, 3H).

^{13}C{^{1}H} NMR (126 MHz, $CDCl_3$): δ (ppm) = 213.5, 137.2, 135.7, 134.2, 129.7, 127.7, 120.8, 63.9, 60.3, 41.4, 39.8, 39.3, 38.8, 31.7, 29.6, 27.0, 23.4, 22.4, 22.3, 19.4, 16.2.

^{1}H/^{29}Si HMBC NMR (501/99 MHz, $CDCl_3$): δ (ppm) = 7.68/−4.7, 3.63/−4.7, 1.05/−4.7.

HRMS (ESI^+): *m/z* calcd. for $C_{31}H_{44}O_2SiNa^+$ $[M+Na]^+$: 499.30028, found: 499.30053..

(*E*)-*tert*-Butyl((6-(2,2-dimethyl-6-methylenecyclohexyl)-4-methylhex-3-en-1-yl)oxy)diphenyl-silane (S19)

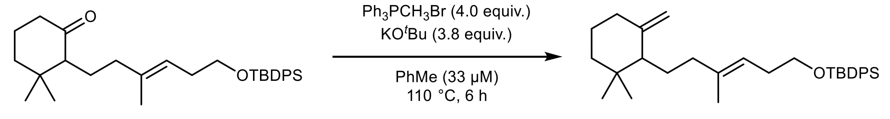

A flame-dried 25 mL two-neck round-bottom flask fitted with a reflux condenser, an argon outlet and a rubber septum was charged with potassium *tert*-butoxide (KO^{t}Bu, 64.0 mg, 0.57 mmol, 1.9 equiv.) and dry toluene (3 mL). Methyltri(phenyl)phosphonium bromide (214 mg, 0.60 mmol, 2.0 equiv.) was added and the resulting bright yellow solution was heated to reflux for 1 h. After the elapsed time, a solution of ketone **S18** (143 mg, 0.30 mmol, 1.0 equiv.) in dry toluene (1 mL, flask rinsed with 2 × 1 mL toluene) was transferred via syringe under reflux to the stirred solution of the ylide. After 4 h at 110 °C, TLC and ^{1}H NMR analysis of the crude reaction mixture indicated incomplete conversion (approximately 73% to the desired olefin) and another portion of previously prepared ylide (prepared from 214 mg, 0.60 mmol, 2.0 equiv. Ph_3PCH_3Br and 64.0 mg, 0.57 mmol, 1.9 equiv. KO^{t}Bu) in dry toluene (3 mL) was added. The reaction mixture was stirred for another 2 h at reflux. After cooling to room temperature, water (10 mL) and brine (10 mL) were added. The aqueous phase was extracted with Et_2O (3 × 20 mL), the combined organic extracts were dried over Na_2SO_4, filtered and concentrated under reduced pressure. Purification by flash column chromatography on silica gel using hexanes/MTBE (gradient elution: hexanes → hexanes/MTBE 39:1 *v/v*) afforded the title compound **S19** as a colorless oil (130 mg, 274 µmol, 91% yield).

Physical state: colorless oil.

TLC (SiO_2, hexanes/MTBE 19:1, *v/v*): R_f = 0.58 (UV: λ = 254 nm, $KMnO_4$ stain).

^{1}H NMR (501 MHz, benzene-d_6): δ (ppm) = 7.86–7.76 (m, 4H), 7.29–7.19 (m, 6H), 5.31 (tq, *J* = 7.2, 1.3 Hz, 1H), 4.86 (dt, *J* = 2.5, 1.1 Hz, 1H), 4.68 (d, *J* = 2.5 Hz, 1H), 3.73 (t, *J* = 6.9 Hz, 2H), 2.37 (q, *J* = 7.0 Hz, 2H), 2.10 (ddd, *J* = 14.9, 10.6, 4.8 Hz, 1H), 2.06–2.00 (m, 1H), 1.96 (dt, *J* = 13.1, 5.0 Hz, 1H), 1.86 (ddd, *J* = 14.1, 10.4, 6.0 Hz, 1H), 1.71 (dd, *J* = 11.3, 3.3 Hz, 1H), 1.62 (dddd, *J* = 14.0, 10.8, 6.1, 3.3 Hz, 1H), 1.57 (s, 3H), 1.53–1.44 (m, 3H), 1.38 (ddd, *J* = 13.2, 9.8, 5.0 Hz, 1H), 1.20 (s, 9H), 1.12 (dt, *J* = 13.3, 4.9 Hz, 1H), 0.94 (s, 3H), 0.84 (s, 3H).

^{13}C{^{1}H} NMR (126 MHz, benzene-d_6): δ (ppm) = 149.5, 137.7, 136.1, 134.5, 129.9, 128.4, 120.8, 109.4, 64.2, 54.0, 38.8, 36.5, 35.0, 32.8, 32.1, 28.6, 27.1, 26.5, 25.2, 24.1, 19.5, 16.4.

^{1}H/^{29}Si HMBC NMR (501/99 MHz, benzene-d_6): δ (ppm) = 7.80/−4.6, 3.73/−4.6, 1.20/−4.6.

HRMS (ESI$^+$): *m/z* calcd. for $C_{32}H_{46}OSiNa^+$ [M+Na]$^+$: 497.32101, found: 497.32138.

(*E*)-6-(2,2-Dimethyl-6-methylenecyclohexyl)-4-methylhex-3-en-1-ol (3c)

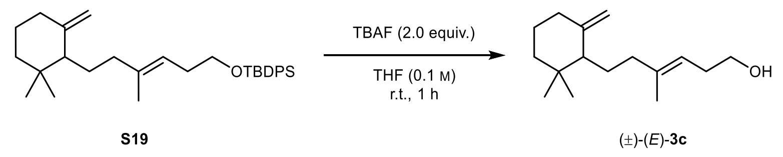

Tetrabutylammonium fluoride (TBAF, 1 M solution in THF, 0.50 mL, 0.50 mmol, 2.0 equiv.) was transferred dropwise to a stirred solution of **S19** (118 mg, .0.25 mmol, 1.0 equiv.) in dry THF (2 mL) under an atmosphere of argon. The resulting yellow solution was stirred at room temperature for 1 h after which time TLC analysis indicated essentially quantitative deprotection and formation of the more polar desired alcohol. Silica gel (ca. 0.5 g) was added and the solvent was removed under reduced pressure. Subsequent purification by flash column chromatography on silica gel eluting with hexanes/MTBE (4:1 → 2:1 *v/v*, gradient elution) furnished (±)-(*E*)-γ-cyclohomofarnesol (**3c**, 52.8 mg, 0.22 mmol, 93% yield) as a colorless oil.

Physical state: colorless oil.

TLC (SiO_2, *n*-pentane/MTBE 2:1, *v/v*): $R_f = 0.33$ ($KMnO_4$ stain)

^{1}H NMR (501 MHz, $CDCl_3$): δ (ppm) = 5.12 (tq, *J* = 7.3, 1.3 Hz, 1H), 4.79–4.72 (m, 1H), 4.53 (d, *J* = 2.4 Hz, 1H), 3.62 (q, *J* = 6.1 Hz, 2H), 2.29 (q, *J* = 6.8 Hz, 2H), 2.11–2.03 (m, 1H), 1.98 (ddt, *J* = 15.0, 10.2, 4.8 Hz, 2H), 1.78 (ddd, *J* = 14.1, 10.2, 6.1 Hz, 1H), 1.68 (dd, *J* = 11.4, 3.4 Hz, 1H), 1.64 (s, 3H), 1.60–1.39 (m, 6H), 1.35 (t, *J* = 5.6 Hz, 1H), 1.21 (ddd, *J* = 13.1, 6.2, 4.2 Hz, 1H), 0.91 (s, 3H), 0.83 (s, 3H).

^{13}C{^{1}H} NMR (126 MHz, $CDCl_3$): δ (ppm) = 149.5, 139.7, 119.6, 109.0, 62.7, 53.9, 38.5, 36.5, 35.0, 32.6, 31.7, 28.6, 26.4, 24.9, 23.9, 16.5.

HRMS (GC-EI$^+$): *m/z* calcd. for $C_{16}H_{28}O^+$ $[M]^+$: 236.213465, found: 236.213610.

GC (achiral): DB-Waxetr 0.25/0.25df G/701, 30.0 m, temperature: 220/ 50 5/min 260 12/min 280, 3 min iso/ 350, gas: 0.6 bar H_2, sample size: 1.0 µL, split ratio: 80:1, t_R = 32.72 min (95.46%).

2D-HPLC: ^{1}D (1st dimension, achiral stationary phase): 100 mm VDSpher PUR 100 SIL, 4.6 mm i.D., *n*-heptane/propan-2-ol 95.5:0.5 *v/v*, 1.0 mL/min, 20.9 MPa, 288 K, UV: λ = 205 nm): t_R = 11.25–11.29 (sampling range). **^{2}D** (2nd dimension, chiral stationary phase): (2 × 150 mm Chiralcel OD-3, 4.6 mm i.D. column 2+1, *n*-heptane/propan-2-ol 95.5:0.5 *v/v*, 1.0 mL/min, 14.5 MPa, 288 K, UV: λ = 205 nm): t_{R1} = 24.14 min (50.6%), t_{R2} = 25.49 min (49.4%).

A small amount (ca. 20 mg) was separated by preparative HPLC to afford (*S*)-(*E*)-γ-cyclohomofarnesol and (*R*)-(*E*)-γ-cyclohomofarnesol which were used for mechanistic studies (see attached HPLC traces).

1.3 Synthesis of Product Standards

Synthesis of (±)-sclareolide (12a), (±)-9-*epi*-sclareolide (12b), and (±)-5β,8α,9β-sclareolide (12c)

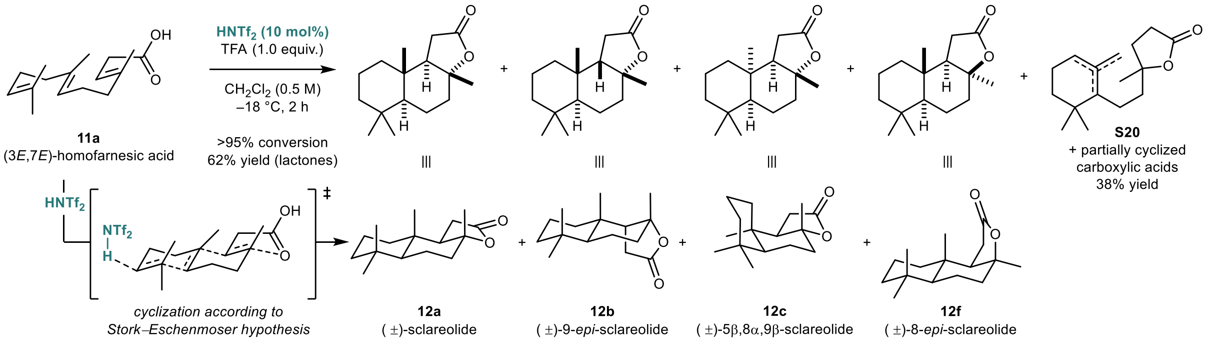

To a 10 mL oven-dried pear-shaped two-neck flask equipped with a PTFE-coated magnetic stir bar under an atmosphere of argon (3 × vacuum/argon cycle) was added a stock solution of $HNTf_2$ in CD_2Cl_2 (0.50 M, 300 µL, 0.15 mmol, 0.10 equiv.), trifluoroacetic acid (115 µL, 171 mg, 1.50 mmol, 1.00 equiv.) and CH_2Cl_2 (2.70 mL, 0.50 M with respect to homofarnesic acid). The colorless solution was cooled to −18 °C (ice/NaCl bath) and stirred (700 rpm) for 10 min. After the elapsed time, neat homofarnesic acid (HMFS, 0.40 mL, 376 mg, 1.50 mmol) was transferred via syringe pump (13.3 µL/min) over 30 min to the stirred solution of the catalyst. After the addition was complete, the resulting pale brown-red solution was stirred for an additional hour at −18 °C. After 90 min reaction time, the reaction was treated with triethylamine (0.50 mL, 364 mg, 3.60 mmol, 2.40 equiv.) and mesitylene (210 µL, 181 mg, 1.51 mmol, 1.01 equiv.) was added as internal standard. Full consumption of the starting material was observed by TLC and ^1H NMR analysis of an aliquot (approx. 0.1 mL) of the pale yellow crude reaction mixture indicated approximately 52% yield of the desired product. The crude reaction mixture was transferred to a flask containing Celite® (approx. 2 g), concentrated under reduced pressure, and the resulting free-flowing off-white powder was subjected to purification by flash column chromatography on silica gel (Merck, 60 Å, 40-63 µm, 230-400 mesh, 20 g, d × l = 2.5 × 15 cm) using hexanes/ethyl acetate as eluent (gradient elution: 100:0, 50 mL → 19:1, 100 mL → 9:1, 200 mL → 4:1, 400 mL → 1:1, 100 mL hexanes/ethyl acetate, *v/v*). Fractions containing the product were pooled, concentrated under reduced pressure and filtered over sand/cotton wool to remove traces of silica gel. The least polar fractions typically consist of a mixture of completely cyclized tricyclic lactones (267 mg, 1.07 mmol, 71% yield; 62% yield of identified lactone diastereomers, **12a**:**12b**:**12c**:**12f** = 17:4:2.7:1 d.r., other lactone diastereomers <10%) whereas more polar fractions contained a more complex mixture of partially cyclized compounds (**S20**). After chromatography of the mixture, the combined yield was 375 mg (1.50 mmol, 99.6%) thus indicating excellent mass balance. The relative stereochemistry of the major product (±)-sclareolide was confirmed by single crystal X-ray diffraction.

Structures of the three main lactone components in the mixture were assigned by comparison of the NMR data with authentic samples and/or available literature data (see Fig. S1–S3)[20,21] or by comparing the GC retention times with commercially available or previously synthesized authentic standards.

The sample was used as racemic standard of sclareolide (**12a**), 9-*epi*-sclareolide (**12b**), and 5β,8α,9β-sclareolide (**12c**) for gas chromatography on a chiral stationary phase.

(±)-sclareolide (12a, major diastereomer):

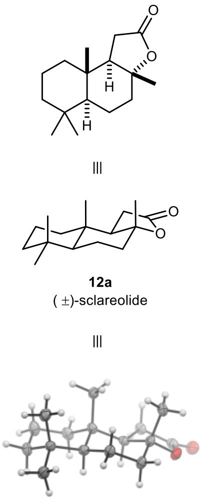

12a
(±)-sclareolide
[X-Ray]

Physical state: colorless solid.

TLC (SiO_2, hexanes/EtOAc 9:1, *v/v*): R_f = 0.19 (CAM stain).

^{1}H NMR (600 MHz, $CDCl_3$): δ (ppm) = 2.40 (dd, *J* = 16.2, 14.8 Hz, 1H), 2.23 (dd, *J* = 16.2, 6.5 Hz, 1H), 2.08 (dt, *J* = 11.9, 3.4 Hz, 1H), 1.97 (dd, *J* = 14.8, 6.5 Hz, 1H), 1.88 (dq, *J* = 14.3, 3.9 Hz, 1H), 1.73–1.60 (m, 2H), 1.49–1.32 (m, 4H), 1.33 (s, 3H), 1.20 (td, *J* = 13.6, 4.2 Hz, 1H), 1.06 (dd, *J* = 12.7, 3.0 Hz, 2H), 0.91 (s, 3H), 0.89 (s, 3H), 0.84 (s, 3H).

^{13}C{^{1}H} NMR (126 MHz, $CDCl_3$): δ (ppm) = 177.0, 86.5, 59.2, 56.8, 42.3, 39.6, 38.8, 36.2, 33.3, 33.2, 28.8, 21.7, 21.0, 20.7, 18.2, 15.2.

HRMS (GC-EI$^+$): *m/z* calcd. for $C_{16}H_{26}O_2$ $[M]^+$: 250.192730, found: 250.192860.

X-Ray: CCDC-2338449.

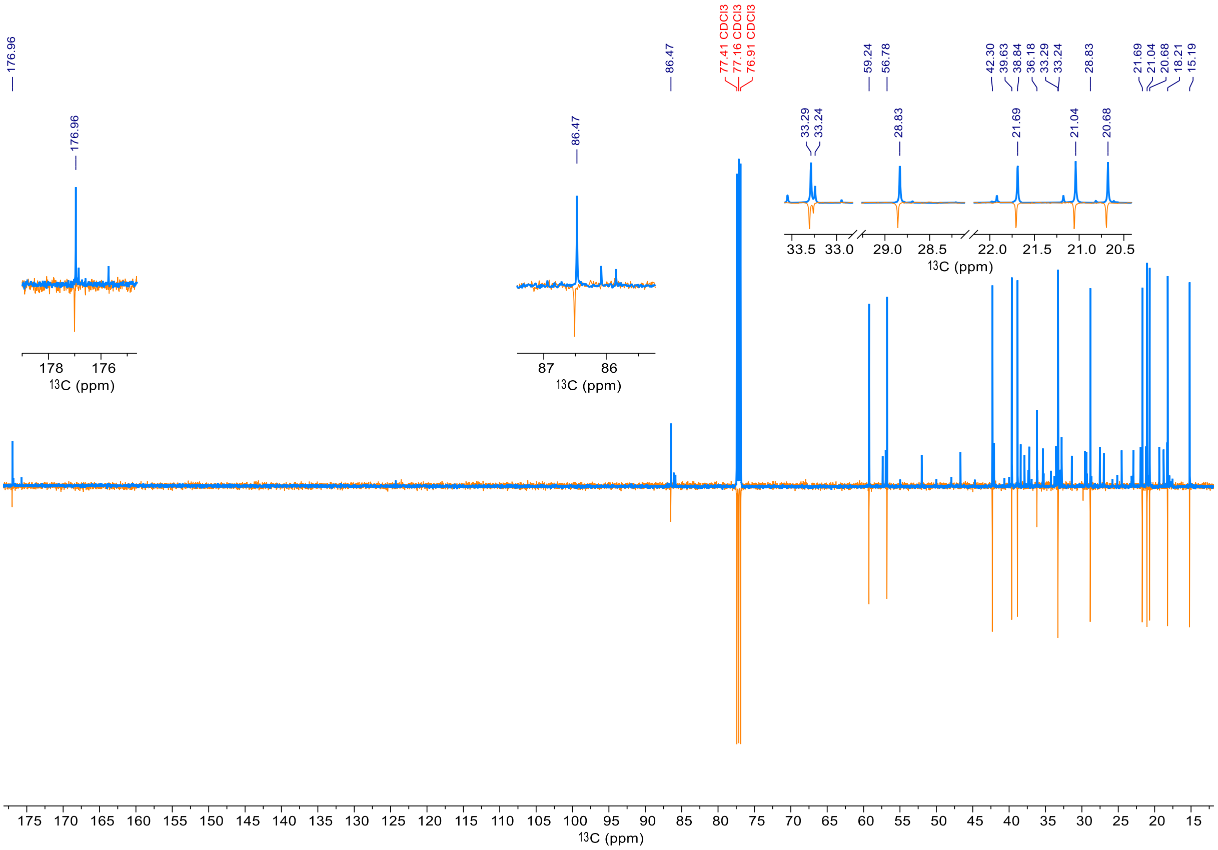

Fig. S1 | Comparison of the ^{13}C NMR (126 MHz, $CDCl_3$) spectrum of synthetic (±)-sclareolide (**12a**, blue, top) with the spectrum of a commercially available (purchased from TCI) authentic sample of (+)-sclareolide (orange, bottom).

GC Data for the lactone fraction containing racemic **12a**, **12b** and **12c**:

GC (achiral) (FFAP 0.25/0.25df G/396 15.0 m, temperature: 220/60 5/min, 250 iso 5 min iso/350, 0.50 bar H_2, sample size: 0.2 µL): 9-*epi*-sclareolide: t_R(**12b**) = 28.10 min (13.82), sclareolide: t_R(**12a**) = 28.29 min (68.74%), 5*β*,8*α*,9*β*-sclareolide: t_R(**12c**) = 28.78 min (10.45%).

GC (chiral) (Hydrodex-β-TBDAc-CD 0.25/?df G/681; 25.0 m, temperature: 220/80 0.8/min 200 10/min 220 5min iso/350, 0.60 bar H_2, sample size: 1.0 µL, split ratio: 40:1): 9-*epi*-sclareolide (**12b**): t_{R1} = 135.10 min (7.48%), t_{R2} = 137.80 min (9.24), sclareolide (**12a**): t_{R1} = 137.22 min (35.66%), t_{R2} = 143.20 min (35.38%), 5β,8α,9β-sclareolide (**12c**): t_{R1} = 139.95 min (5.77%), t_{R2} = 146.04 min (5.77%).

^{13}C NMR and HRMS data for (±)-9-*epi*-sclareolide (12b, minor diastereomer):

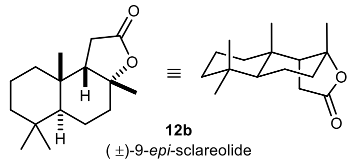

12b
(±)-9-*epi*-sclareolide

^{13}C{^{1}H} NMR (126 MHz, $CDCl_3$): δ (ppm) = 175.7, 86.1, 57.0, 46.7, 42.1, 38.4, 37.2, 36.1, 33.5, 32.9, 32.8, 27.5, 22.9, 21.9, 19.4, 18.3

HRMS (GC-EI$^+$): *m/z* calcd. for $C_{16}H_{26}O_2$ $[M]^+$: 250.192730, found: 250.192770.

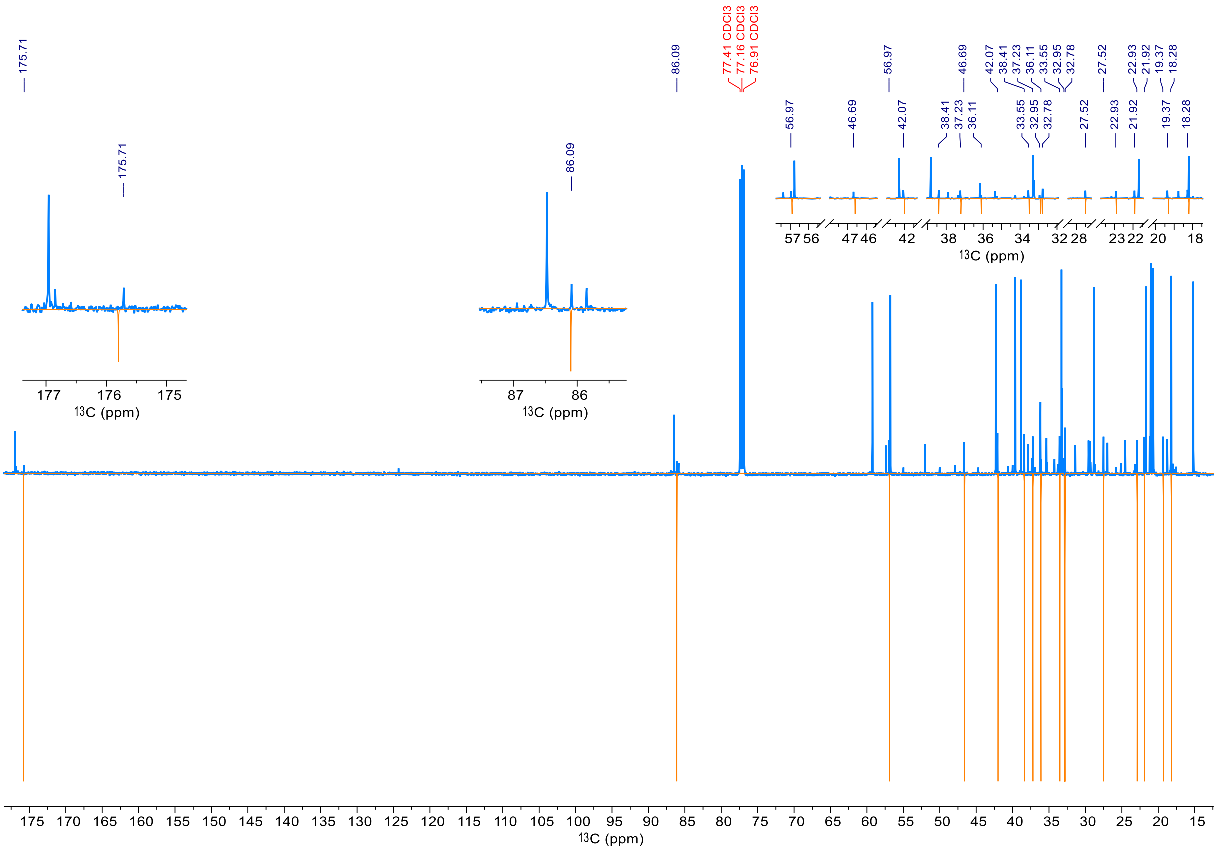

Fig. S2 | Comparison of the ^{13}C NMR (126 MHz, $CDCl_3$) spectrum of synthetic (±)-9-*epi*-sclareolide (**12b**, blue, top) with a spectrum of (−)-**12b** generated from available literature data (orange, bottom).[21]

^{13}C NMR and HRMS data for (±)-5β,8α,9β-sclareolide (12c, minor diastereomer):

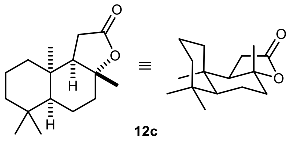

12c
(±)-5β,8α,9β-sclareolide

^{13}C{^{1}H} NMR (126 MHz, $CDCl_3$): δ (ppm) = 176.7, 85.7, 57.2, 51.9, 37.7, 37.2, 35.2, 34.1, 32.6, 31.2, 29.4, 29.2, 26.8, 24.4, 21.0, 18.6.

HRMS (GC-EI$^+$): *m/z* calcd for $C_{16}H_{26}O_2$ [M]$^+$: 250.192730, found: 250.192730.

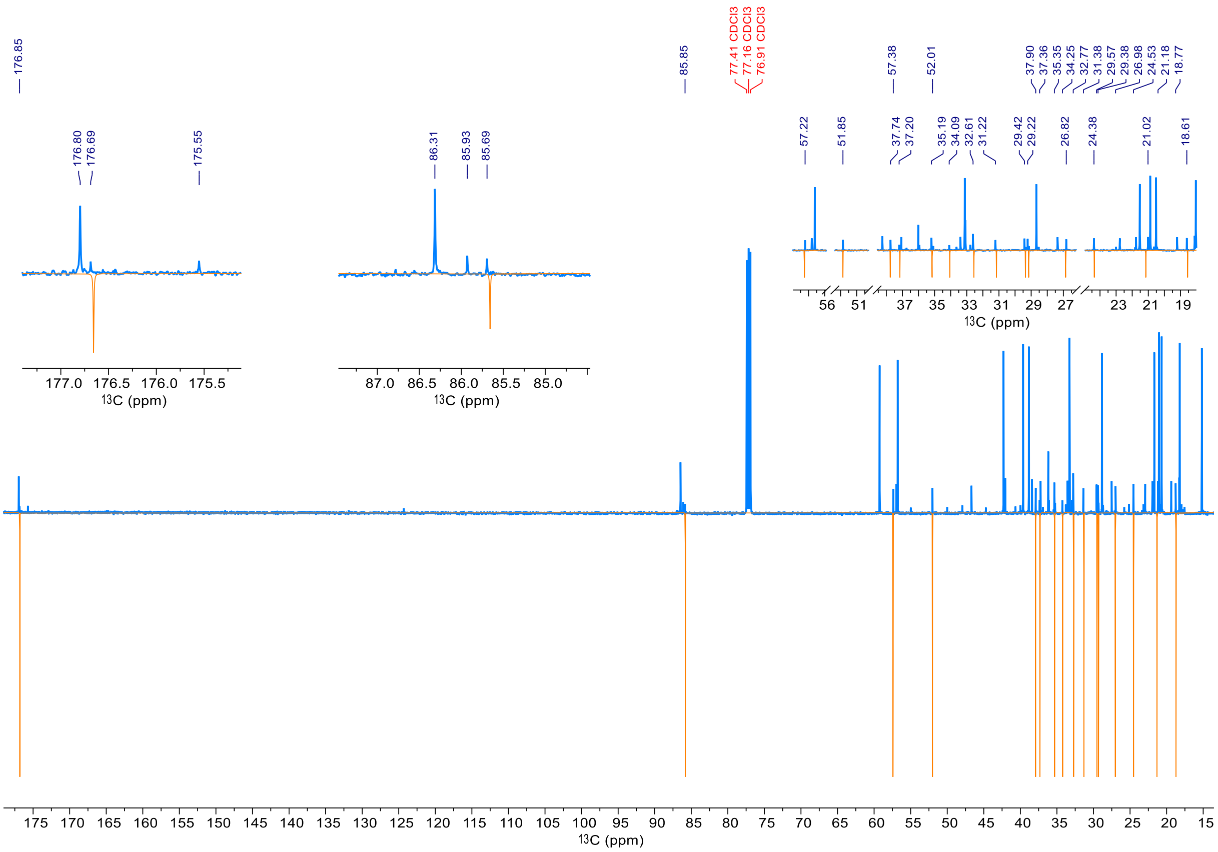

Fig. S3 | Comparison of the ^{13}C NMR (126 MHz, $CDCl_3$) spectrum of synthetic (±)-5β,8α,9β-sclareolide (**12c**, minor diastereomer, blue, top) with a spectrum of **12c** generated from available literature data (orange, bottom).[20]

Synthesis of (±)-9-*epi*-sclareolide (12b) and (±)-5β,8α-sclareolide (12d)

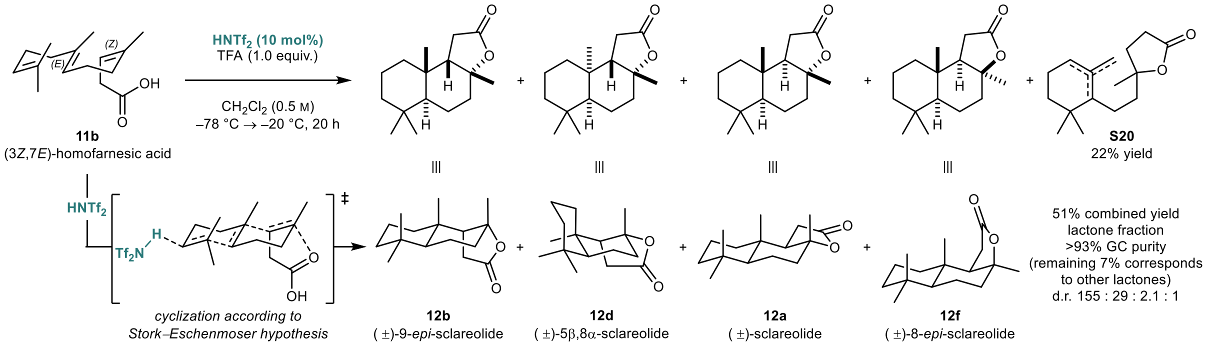

A flame-dried 10 mL Schlenk tube under argon equipped with a PTFE-coated magnetic stir bar was charged with a solution of (3*Z*,7*E*)-homofarnesic acid (1.0 mL, 2 M in CH_2Cl_2, 501 mg, 2.00 mmol; flask rinsed with 1.0 mL and 1.6 mL CH_2Cl_2). The solution was cooled to −78 °C (dry ice/acetone cooling bath) and trifluoroacetic acid (TFA, 154 µL, 229 mg, 2.01 mmol, 1.0 equiv.) was transferred to the reaction vessel followed by dropwise addition of $HNTf_2$ (0.5 M stock solution in CD_2Cl_2, 0.40 mL, 28.1 mg, 0.20 mmol, 0.10 equiv., 10 mol%). The resulting pale yellow solution was placed inside a cryostat at −20 °C and stirred at this temperature for 20 h. After the elapsed time, the reaction mixture was neutralized with triethylamine (0.50 mL, 363 mg, 3.59 mmol, 1.8 equiv.; color change from orange-red to pale yellow upon addition), and the reaction mixture was concentrated onto Celite®. Purification by flash column chromatography on silica gel eluting with hexanes/ethyl acetate (gradient elution: 19:1 → 9:1 → 7:3 → 4:1, *v/v*) afforded a lactone fraction (255 mg, 1.01 mmol, 51%, **12b**:**12d**:**12a**:**12f**: ca. 155:29:2.1:1 d.r.) as a colorless wax and a fraction containing mainly partially cyclized compounds (**S20**, 112 mg, 0.45 mmol, 22% yield, ca. 19:81 α:β) as pale yellow oil. Recrystallization of the lactone fraction from hexanes in a fridge at 4 °C afforded colorless crystals that consisted of a mixture of (±)-9-*epi*-sclareolide and 5β,8α-sclareolide (ca. 84:16 d.r.). The structures were confirmed by comparison of ^{13}C NMR spectra available in the literature (see Figs. S4 and S5) and additionally secured by single crystal X-ray diffraction studies (see Fig. S6).

Analytical data for (±)-9-*epi*-sclareolide (major diastereomer):

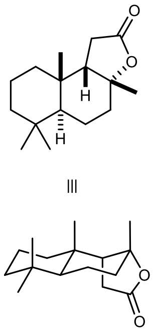

12b
(±)-9-*epi*-sclareolide

Physical state: colorless wax.

TLC (SiO_2, hexanes/EtOAc 9:1, *v/v*): R_f = 0.19 (CAM stain).

^{1}H NMR (501 MHz, $CDCl_3$): δ (ppm) = 2.61 (dd, *J* = 17.3, 13.6 Hz, 1H), 2.40 (dd, *J* = 17.3, 8.3 Hz, 1H), 2.06–1.97 (m, 2H), 1.70–1.58 (m, 2H), 1.55 (s, 3H), 1.57–1.48 (m, 1H), 1.47–1.38 (m, 2H), 1.34–1.20 (m, 2H), 1.20–1.11 (m, 2H), 1.11 (s, 3H), 1.14–1.05 (m, 1H), 0.91 (s, 3H), 0.83 (s, 3H).

$^{13}C\{^1H\}$ NMR (126 MHz, $CDCl_3$): δ (ppm) = 175.7, 86.1, 57.0, 46.7, 42.1, 38.4, 37.2, 36.1, 33.6, 32.9, 32.8, 27.5, 22.9, 21.9, 19.4, 18.3.

GC (achiral) (FFAP 0.25/0.25df G/396; 15.0 m, temperature: 220/60 5/min, 250 5 min iso/350, 0.50 bar H_2, sample size: 0.2 µL): 8-*epi*-sclareolide: t_R (**12f**) = 27.50 min (0.50%), 9-*epi*-sclareolide: t_R (**12b**) = 28.77 min (77.35%), sclareolide: t_R (**12a**) = 28.91 min (1.05%), 5β,8α-sclareolide: t_R (**12d**) = 29.16 min (14.49%).

GC (chiral) (Hydrodex-β-TBDAc-CD 0.25/?df G/681; 25.0 m, temperature: 220/80 0.4/min 200 10/min 220 5min iso/350, 0.60 bar H_2, sample size: 0.2 µL): 9-*epi*-sclareolide: t_{R1} = 235.27 min (49.78%), t_{R2} = 242.05 min (50.22%).

HRMS (GC-EI$^+$): *m/z* calcd. for $C_{16}H_{26}O_2^+$ $[M]^+$: 250.192730, found: 250.192640.

X-Ray Crystallography: CCDC-2338445.

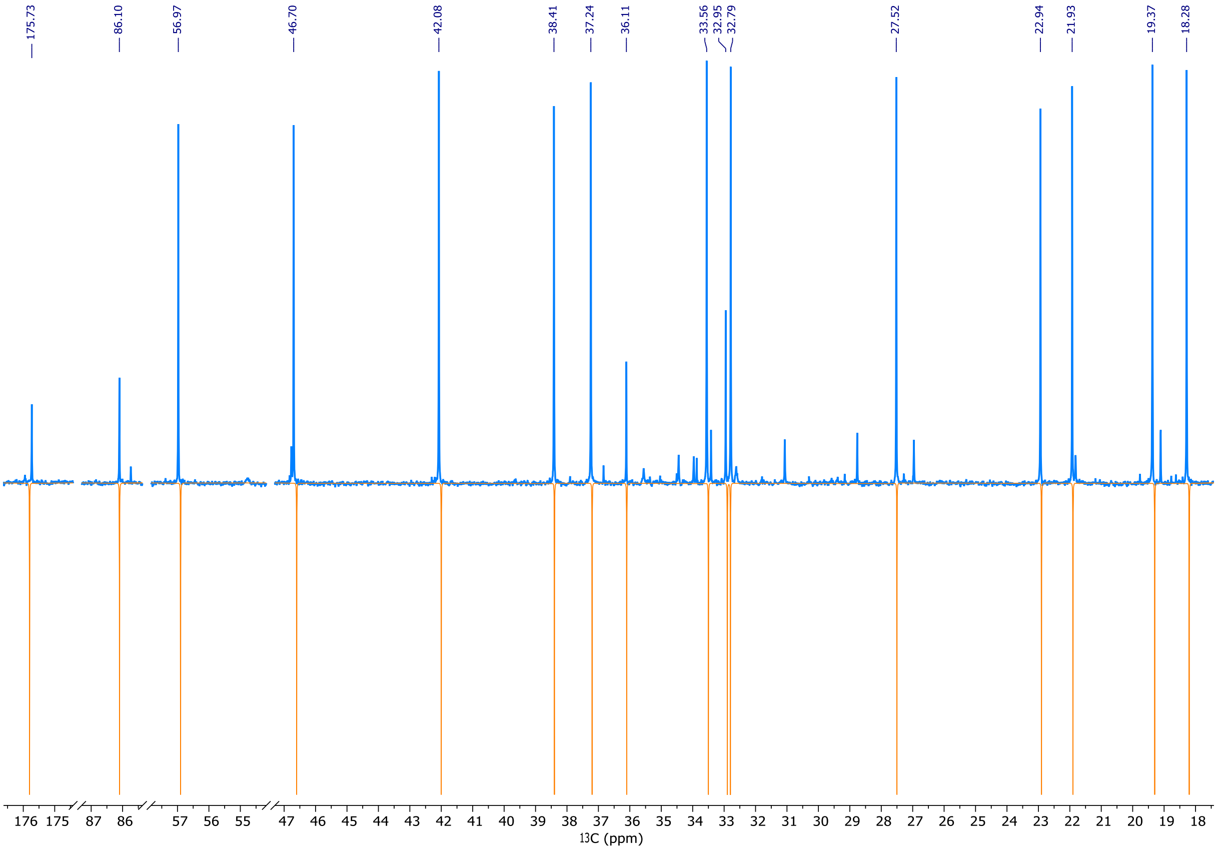

Fig. S4 | Comparison of the ^{13}C NMR spectrum (126 MHz, $CDCl_3$; referenced on centermost peak of $CDCl_3$ at 77.16 ppm) of the isolated lactone fraction with a ^{13}C NMR spectrum of 9-*epi*-sclareolide generated from available literature data.[20]

Partial analytical data for (±)-5β,8α-sclareolide (minor diastereomer):

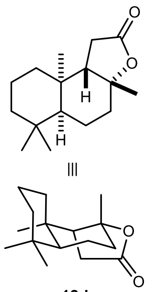

12d
(±)-5β,8α-sclareolide

Physical state: colorless wax.

TLC (SiO_2, hexanes/EtOAc 9:1, *v/v*): R_f = 0.19 (CAM stain).

^{1}H NMR (501 MHz, $CDCl_3$): δ (ppm) = 2.61 (dd, J = 17.5, 11.9 Hz, 1H), 2.47 (dd, J = 17.5, 8.1 Hz, 1H), 1.51 (s, 3H), 1.12 (s, 3H), 1.08 (s, 3H), 0.93 (s, 3H). Due to signal overlap only diagnostic peaks of the minor diastereomer are reported.

$^{13}C\{^1H\}$ NMR (126 MHz, $CDCl_3$): δ (ppm) = 175.9, 85.7, 54.8, 46.8, 36.8, 35.6, 34.4, 34.0, 33.9, 33.4, 32.6, 31.1, 28.8, 27.0, 21.8, 19.1.

GC (achiral) (FFAP 0.25/0.25df G/396; 15.0 m, temperature: 220/60 5/min, 250 5 min iso/350, 0.50 bar H_2, sample size: 0.2 µL): 9-*epi*-sclareolide: t_R (**12b**) = 28.77 min (77.35%), sclareolide: t_R (**12a**) = 28.91 min (1.05%), 5β,8α-sclareolide: t_R (**12d**) = 29.16 min (14.49%).
GC (chiral) (Hydrodex-β-TBDAc-CD 0.25/?df G/681; 25.0 m, temperature: 220/80 0.4/min 200 10/min 220 5min iso/350, 0.60 bar H_2, sample size: 0.2 µL): 9-*epi*-sclareolide (**12b**): t_{R1} = 235.27 min (49.78%), t_{R2} = 242.05 min (50.22%), 5β,8α-sclareolide (**12d**): t_{R1} = 240.76 min (8.04%), t_{R2} = 242.90 min (7.85%).
HRMS (GC-EI$^+$): *m/z* calcd. for $C_{16}H_{26}O_2^+$ $[M]^+$: 250.192730, found: 250.192680.
X-Ray Crystallography: CCDC-2338444.

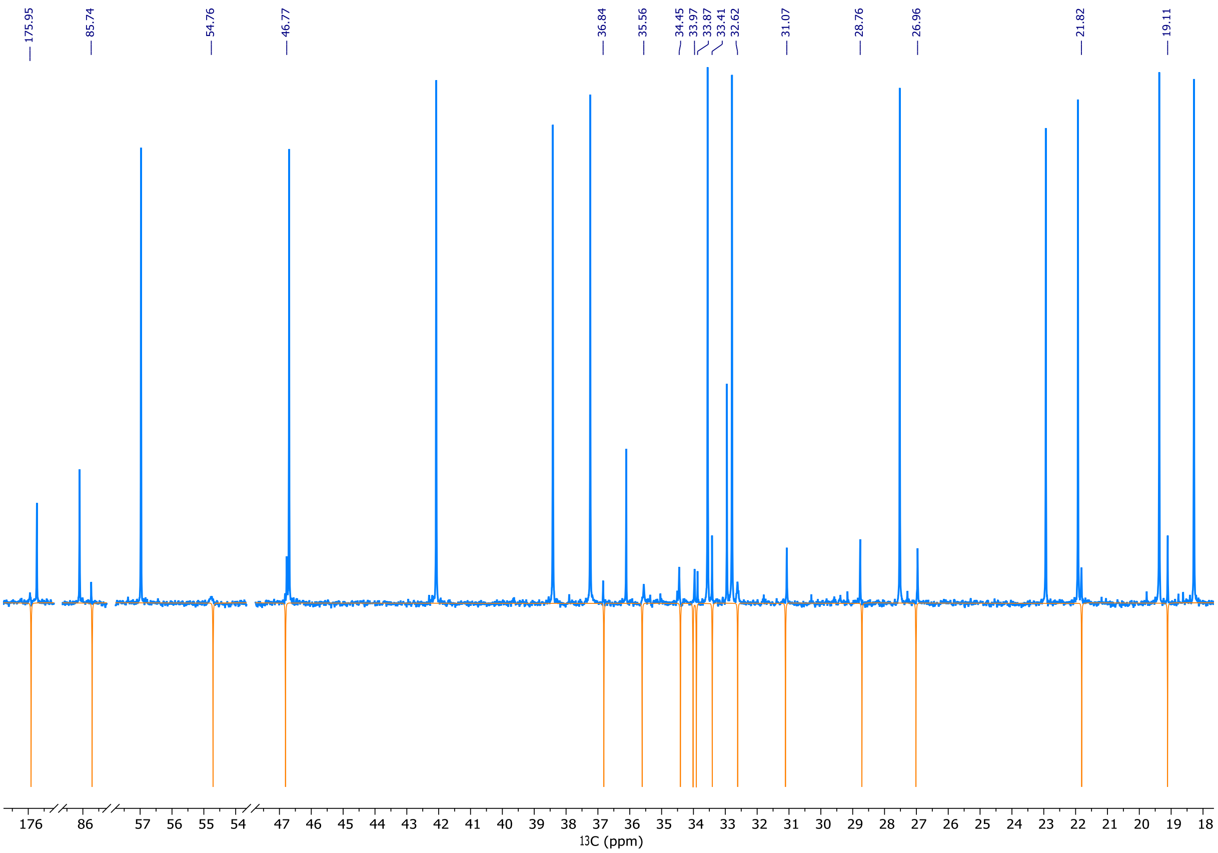

Fig. S5 | Comparison of the ^{13}C NMR spectrum (126 MHz, $CDCl_3$; referenced on the centermost peak of $CDCl_3$ at 77.16 ppm) of the isolated lactone fraction (blue, top) with a ^{13}C NMR spectrum of 5β,8α-sclareolide (**12d**, orange, bottom) generated from available literature data.[20]

Comparison of the X-ray crystal structures of (±)-9-*epi*- (**12b**) and 5β,8α-Sclareolide (**12d**):

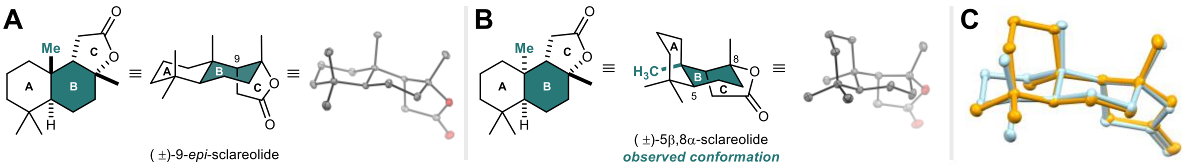

Fig. S6 | A, ORTEP (Oak Ridge Thermal Ellipsoid Plot) representation of the solid state structure of the major diastereomer (±)-9-*epi*-sclareolide (**12b**). **B**, ORTEP representation of the solid state structure of the minor diastereomer (±)-5β,8α-sclareolide (**12d**). **C**, Overlay of both X-ray structures (light blue: **12b**, orange: **12d**) created with the structure overlay tool implemented in Mercury. Thermal ellipsoids are set at a 50% probability level and hydrogen atoms are omitted for clarity in all structures.

Analytical data for the partially cyclized side product mixture of (*E*)-α/β-cyclohomofarnesic acid isomers (±)-(*E*)-S20:

TLC (SiO_2, hexanes/EtOAc 9:1, *v/v*): R_f = 0.15 (CAM stain).

^{1}H NMR (501 MHz, $CDCl_3$): δ (ppm) = δ 2.69–2.51 (m, 2H), 2.17–2.10 (m, 1H), 2.12–2.04 (m, 2H), 2.04–1.96 (m, 1H), 1.90 (t, J = 6.3 Hz, 2H), 1.81–1.67 (m, 2H), 1.58 (s, 3H), 1.59–1.50 (m, 4H), 1.42 (s, 3H), 0.98 (s, 6H).

^{13}C{^{1}H} NMR (126 MHz, $CDCl_3$): δ (ppm) = 176.9, 136.0, 127.9, 87.1, 41.1, 39.9, 35.2, 33.1, 32.9, 29.3, 28.8, 28.7, 25.5, 22.7, 19.9, 19.6.

GC (achiral) (FFAP 0.25/0.25df G/396, 15.0 m, temperature: 220 °C (injector) 60 °C 5 °C/min, 250 °C iso 5 °C/min/iso/350 (detector), 0.50 bar H_2, sample size: 0.2 µL): t_R (α-cyclohomofarnesic acid) = 24.47 min (14.70%), t_R (β-cyclohomofarnesic acid) = 24.95 min (62.46%).

GC (chiral) (Hydrodex-β-TBDAc-CD 0.25/0.25df G/681; 25.0 m, temperature: 220/80 0.8/min 200 10/min 220 5min iso/350, 0.60 bar H_2, sample size: 0.2 µL): t_R (β-cyclohomofarnesic acid + *ent*-α-cyclohomofarnesic acid) = 116.45 min (45.62%), t_R (β-cyclohomofarnesic acid) = 117.61 min (40.91%).

HRMS (GC-EI$^+$): *m/z* calcd. for $C_{16}H_{26}O_2^+$ $[M]^+$: 250.192730, found: 250.192420.

The NMR data are in good agreement with reported literature data.[22]

Synthesis of (±)-9-*epi*-ambrox (2b) and (±)-5*β*,8*α*-ambrox (2d)

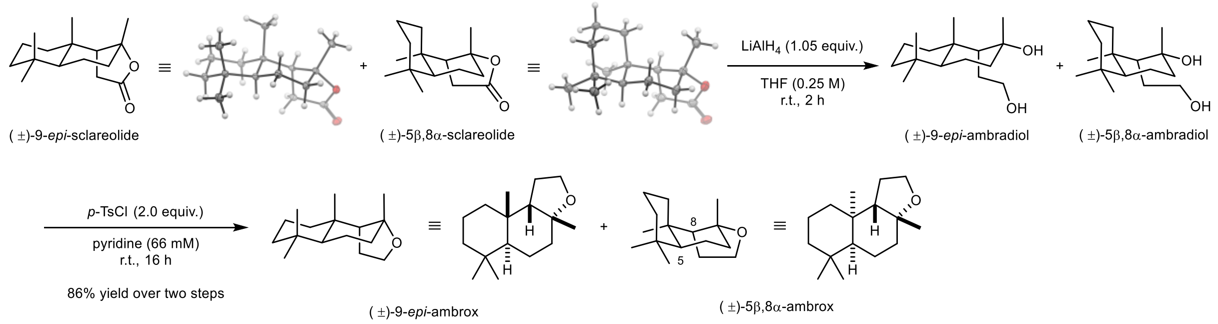

A mixture of racemic 9-*epi*-ambrox and 5β,8α-ambrox was synthesized from the corresponding lactones via the respective ambradiols.

Step 1: lactone reduction

A diastereomeric mixture of (±)-9-*epi*-sclareolide and (±)-5β,8α-sclareolide (ca. 84:16 d.r., 223 mg, 0.89 mmol, 1.0 equiv.) was transferred to an oven-dried 10 mL pear-shaped flask equipped with a PTFE-coated magnetic stir bar. The flask was closed with a rubber septum, evacuated, and placed under an atmosphere of argon (3 ×). The starting material was dissolved in dry THF (2.6 mL). $LiAlH_4$ (1 M solution in THF, 0.93 mL, 0.93 mmol, 1.0 equiv.) was added dropwise at room temperature and the resulting colorless solution was stirred at this temperature for 2 h. After the elapsed time, propan-2-ol (0.5 mL) was carefully added and the resulting cloudy solution was stirred for another 15 min. The colorless suspension was poured into water (10 mL) and the aqueous phase was extracted with MTBE (3 × 10 mL). The combined organic layers were dried over $MgSO_4$, filtered and concentrated under reduced pressure to afford the crude product as a colorless solid which was used in the subsequent step without further purification.

Step 2: cyclization

The thus obtained diastereomeric mixture of 1,4-diols (ca. 84:16 d.r., 226 mg, 0.89 mmol) was transferred to a two-neck 50 mL round-bottom flask equipped with a PTFE-coated magnetic stir bar and an argon outlet. The reaction vessel was closed with a rubber septum, evacuated, and flushed with argon (3 ×). The starting material was dissolved in dry pyridine (13.5 mL, 66 mM) and *p*-toluenesulfonyl chloride (339 mg, 1.78 mmol, 2.0 equiv.) was transferred to the stirred colorless solution (slightly exothermic reaction upon addition). After the addition was complete, the reaction vessel was sealed with a rubber septum and a balloon filled with argon was attached. The resulting pale orange solution was stirred at room temperature for 16 h. After the elapsed time, aqueous saturated $NaHCO_3$ solution (20 mL) was added carefully (*Caution: gas evolution*), followed by MTBE (10 mL). The aqueous phase was extracted with MTBE (3 × 10 mL), the combined organic layers were washed with aqueous saturated $NaHCO_3$ (20 mL), water (20 mL), and brine (20 mL), dried over $MgSO_4$, filtered, and concentrated under reduced pressure. Purification by flash column chromatography on silica gel using hexanes/MTBE (isocratic elution: 19:1 *v/v*) as eluent afforded a diastereomeric mixture of (±)-9-*epi*-

ambrox and (±)-5β,8α-ambrox as a colorless oil (87:13 d.r., 181 mg, 0.77 mmol, 86% yield over two steps).

Analytical data for (±)-9-*epi*-ambrox (major diastereomer):

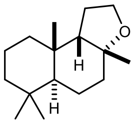

2b
(±)-9-*epi*-ambrox

TLC (SiO_2, hexanes/MTBE 19:1, *v/v*): R_f = 0.20 (CAM stain).

^{1}H NMR (501 MHz, $CDCl_3$): δ (ppm) = 3.85 (ddd, *J* = 9.6, 8.3, 3.1 Hz, 1H), 3.77 (q, *J* = 8.5 Hz, 1H), 2.04 (tt, *J* = 12.0, 9.1 Hz, 1H), 1.91 (dtd, *J* = 12.2, 8.0, 3.1 Hz, 1H), 1.73–1.60 (m, 1H), 1.59–1.52 (m, 4H), 1.43–1.38 (m, 2H), 1.37 (s, 3H), 1.31–1.25 (m, 3H), 1.19 (dd, *J* = 12.2, 1.4 Hz, 1H), 1.14 (td, *J* = 14.3, 5.1 Hz, 1H), 1.10 (s, 3H), 0.89 (s, 3H), 0.82 (s, 3H).

^{13}C{^{1}H} NMR (126 MHz, $CDCl_3$): δ (ppm) = 81.0, 64.2, 59.2, 46.9, 42.5, 38.8, 36.2, 35.9, 33.7, 33.1, 29.0, 27.9, 23.0, 21.9, 20.6, 18.7.

HRMS (GC-EI$^+$): *m/z* calcd. for $C_{16}H_{28}O^+$ [M]$^+$: 236.213465, found: 236.213420.

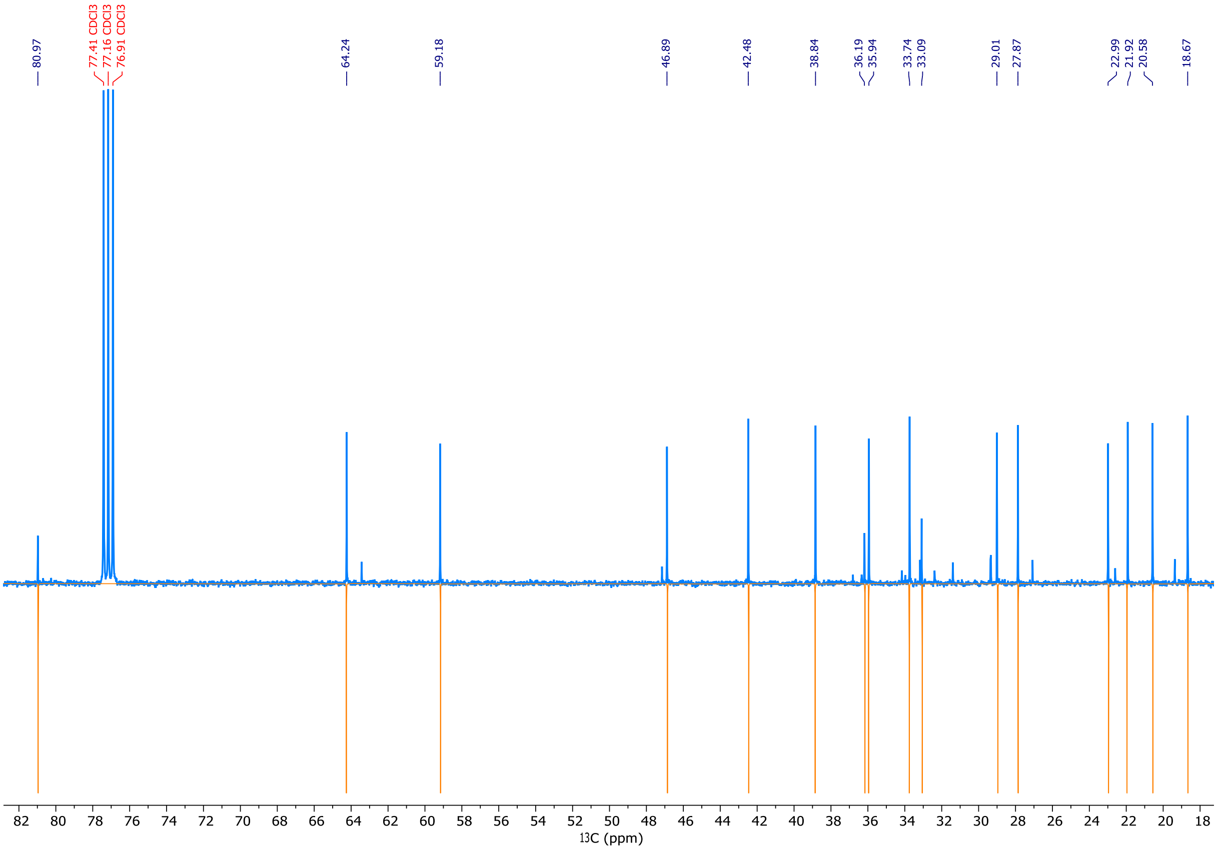

Fig. S7 | Comparison of the ^{13}C NMR spectrum (126 MHz, $CDCl_3$; referenced on centermost peak of $CDCl_3$ at 77.16 ppm) of the isolated lactone fraction with a ^{13}C NMR spectrum of 9-*epi*-ambrox generated from available literature data.[20]

Partial analytical data for (±)-5β,8α-ambrox (minor diastereomer):

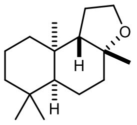

2d

(±)-5β,8α-ambrox

TLC (SiO_2, hexanes/MTBE 19:1, *v/v*): R_f = 0.20 (CAM stain).

¹H NMR (501 MHz, $CDCl_3$): δ (ppm) = 3.75 (q, *J* = 8.6 Hz, 1H), 1.80 (td, *J* = 12.6, 4.1 Hz, 1H), 1.31 (s, 3H), 1.10 (s, 3H), 1.08 (s, 3H), 1.03–0.96 (m, 1H), 0.90–0.89 (m, 3H). Due to signal overlap only diagnostic peaks of the minor diastereomer are reported.

$^{13}C\{^1H\}$ NMR (126 MHz, $CDCl_3$): δ (ppm) = 80.3, 63.4, 56.2, 47.2, 36.8, 36.3, 34.2, 34.0, 33.2, 32.4, 31.4, 29.4, 29.3, 27.1, 22.6, 19.4.

HRMS (GC-EI$^+$): *m/z* calcd. for $C_{16}H_{28}O^+$ [M]$^+$: 236.213465, found: 236.213390.

GC Data for (±)-9-*epi*-ambrox (**2b**, major) and (±)-5β,8α-ambrox (**2d**, minor):

GC (achiral) (DB-1 0.25/0.25df G/701 30.0 m; 220/60 5/min 200 12/min 300, 3 min iso/350, 0.50 bar H_2, sample size: 0.2 µL): 9-*epi*-ambrox: t_R(**2b**) = 24.98 min (85.95%), 5β,8α-ambrox: t_R(**2d**) = 25.17 min (13.04%), ambrox: t_R(**2a**) = 25.55 min (1.01%).

GC (chiral) (G-TA 0.25/?df G/448 30.0 m; 220/60 85 iso/ 350, 0.50 bar H_2, sample size: 1.0 µL): (−)-9-*epi*-ambrox: t_R(**2b**) = 409.74 min (43.72%), 5β,8α-ambrox: t_R(**2d**) = 426.93 min (6.64%), (+)-9-*epi*-ambrox: t_R(*ent*-**2b**) = 444.27 min (43.14%), *ent*-5β,8α-ambrox, t_R(*ent*-**2d**) = 474.75 min (6.49%).

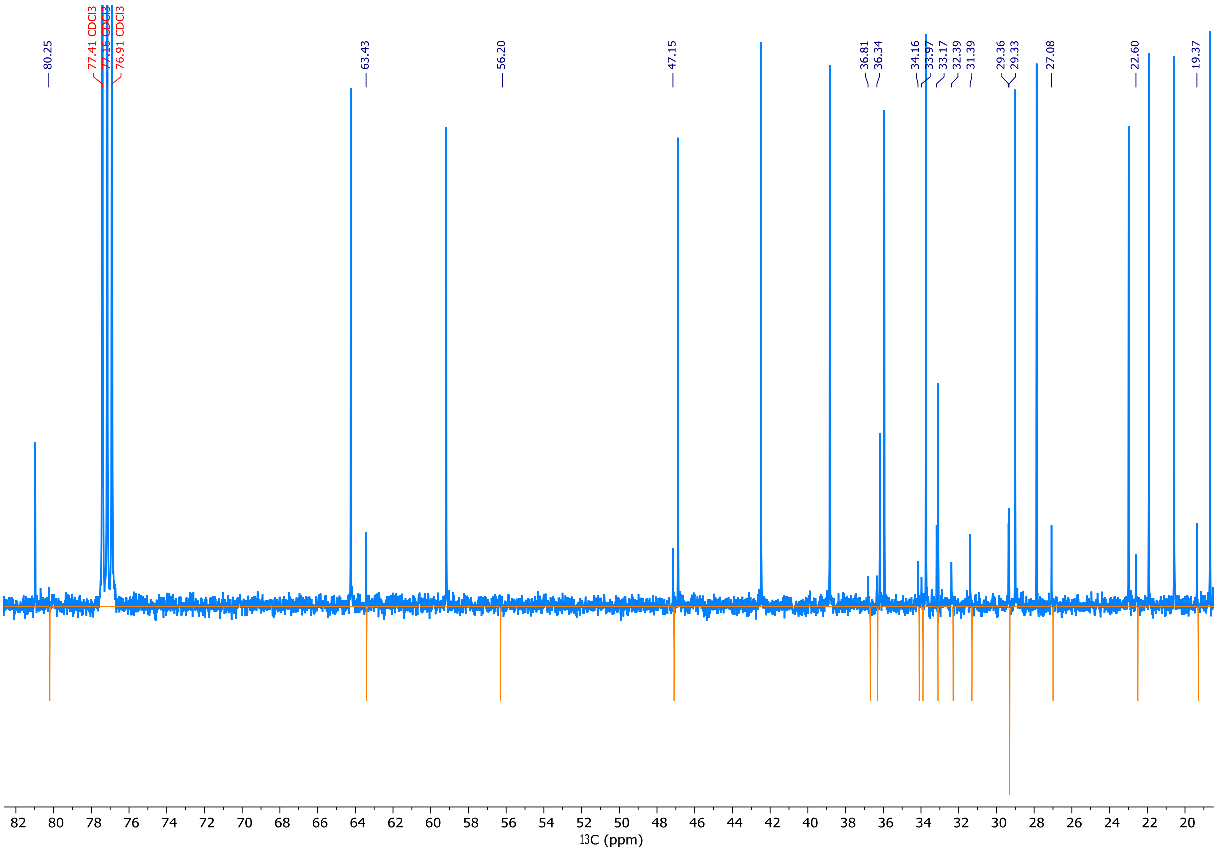

Fig. S8 | Comparison of the ^{13}C NMR spectrum (126 MHz, $CDCl_3$; referenced on centermost peak of $CDCl_3$ at 77.16 ppm) of the isolated lactone fraction with a ^{13}C NMR spectrum of 5β,8α-ambrox generated from available literature data.[20]

Synthesis of (−)-9-*epi*-sclareolide (12b) and (−)-9-*epi*-ambrox (2b)

The synthesis of enantiopure (−)-9-*epi*-ambrox as reference material for GC analysis and for assignment of the absolute configuration was performed according to the procedure outlined below.

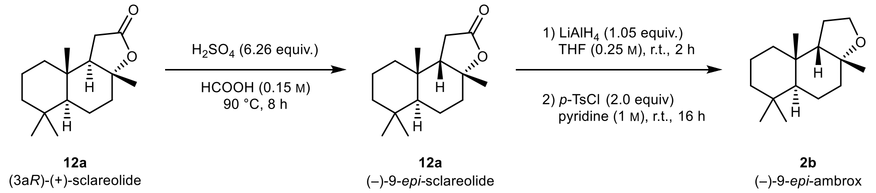

Briefly, commercially available (+)-sclareolide (**12a**) was isomerized to the thermodynamically most stable isomer (−)-9-*epi*-sclareolide (**12b**) in the presence of excess sulfuric acid in formic acid following a procedure previously described by Lucius[23] and Ohloff.[24] A subsequent reduction of the thus obtained lactone with lithium aluminum hydride ($LiAlH_4$) afforded pure (−)-(9βH)-13,14,15,16-tetranor-8α,12-diol (9-*epi*-ambradiol). Cyclization of the diol to (−)-9-*epi*-ambrox (**2b**) was accomplished with *p*-toluenesulfonyl chloride (*p*-TsCl) in pyridine.

Synthesis of (−)-9-*epi*-Sclareolide (12b)

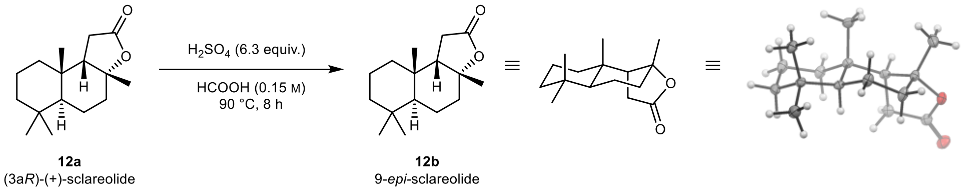

Commercially available (3a*R*)-(+)-sclareolide (**12a**, purchased from TCI, 5.82 g, 23.3 mmol, 1.0 equiv.) was transferred to a 250 mL round-bottom flask and dissolved in formic acid (155 mL, 0.15 M). The flask was fitted with a reflux condenser and immersed in a preheated oil bath at 90 °C. Concentrated H_2SO_4 (96-97%, 7.8 mL, 146 mmol, 6.3 equiv.) was added dropwise. The resulting yellow solution was stirred at 90 °C for 8 h after the addition was complete. After the elapsed time, the reaction mixture was poured into ice water (400 mL), diluted with MTBE (200 mL) and carefully treated with aq. sat. Na_2CO_3 (300 mL, *Caution: gas evolution upon addition*). The organic phase was separated and the aqueous phase was extracted with MTBE (2 × 200 mL). The combined organic layers were washed with aq. sat. Na_2CO_3 (3 × 250 mL), water (200 mL) and brine (200 mL), dried over $MgSO_4$, filtered, and concentrated under reduced pressure to afford the crude product as a crystalline colorless solid. Recrystallization from Et_2O (slow evaporation of a saturated solution at room temperature) afforded the product as colorless plate/needle-shaped crystals. The crystals were collected by suction filtration, washed with cold Et_2O (3 × 10 mL) and air-dried to afford (−)-9-*epi*-sclareolide (**12b**) as a colorless crystalline solid (single diastereo- and enantiomer, 3.85 g, 15.4 mmol, 66% yield). The obtained crystals were suitable for X-ray diffraction studies which additionally confirmed the expected absolute configuration.

Analytical data for (−)-9-*epi*-sclareolide (12b, 12-nor-9-*epi*-ambreinolide):

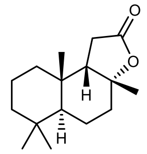

III

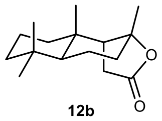

12b
(–)-9-*epi*-sclareolide

III

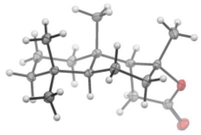

[X-Ray]

Physical state: colorless crystalline solid.

^{1}H NMR (501 MHz, $CDCl_3$): δ (ppm) = 2.62 (dd, J = 17.3, 13.6 Hz, 1H), 2.41 (dd, J = 17.3, 8.3 Hz, 1H), 2.06–1.95 (m, 2H), 1.69–1.52 (m, 3H), 1.56 (s, 3H), 1.47–1.39 (m, 2H), 1.33–1.22 (m, 2H), 1.20–1.07 (m, 3H), 1.11 (s, 3H), 0.92 (s, 3H), 0.83 (s, 3H).

^{13}C{^{1}H} NMR (126 MHz, $CDCl_3$): δ (ppm) = 175.7, 86.1, 57.0, 46.7, 42.1, 38.4, 37.3, 36.1, 33.6, 33.0, 32.8, 27.5, 22.9, 21.9, 19.4, 18.3.

IR (ATR, neat): $\tilde{\upsilon}_{max}$ (cm^{-1}) = 2961 (w), 2924 (m), 2863 (w), 1753 (s), 1450 (w), 1385 (w), 1295 (w), 1269 (w), 1260 (w), 1231 (m), 1196 (w), 1167 (m), 1125 (w), 1090 (m), 1016 (w), 943 (s), 825 (w), 666 (w), 610 (w), 569 (w), 544 (w).

HRMS (ESI^+): *m/z* calcd. for $C_{16}H_{26}O_2$ $[M]^+$: 250.192730, found: 250.192790.

$[\alpha]_D^{25}$ = −51.2 (c = 1.0, $CHCl_3$); Lit.: $[\alpha]_D^{20}$ = −50.8 (c = 1.0, $CHCl_3$).[25]

GC (achiral) (FFAP 0.25/0.25df G/396; 15.0 m, temperature: 220/60 5/min, 250 5 min iso/350, 0.50 bar H_2, sample size: 0.2 μL): 9-*epi*-sclareolide: t_R(**12b**) = 28.80 min (>99%).

GC (chiral) (Hydrodex-β-TBDAc-CD 0.25/?df G/681; 25.0 m, temperature: 220/80 0.4/min 200 10/min 220 5min iso/350, 0.60 bar H_2, sample size: 0.2 μL): (−)-9-*epi*-sclareolide: t_R(**12b**) = 241.85 min (100%).

X-Ray Crystallography: CCDC-2338450.

Synthesis of (−)-9-*epi*-Ambrox (2b)

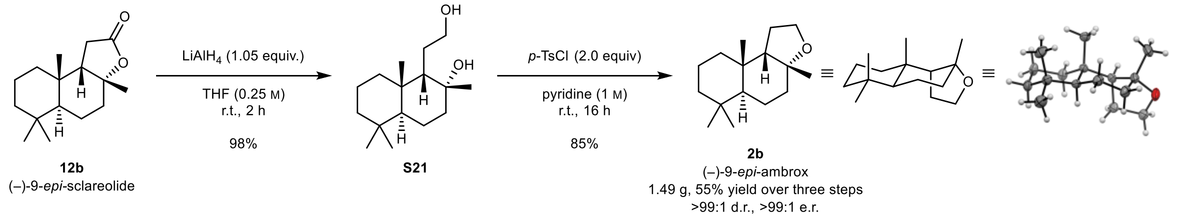

A flame-dried 100 mL Schlenk flask under argon was charged with (−)-9-*epi*-sclareolide (**12b**, 2.00 g, 8.00 mmol, 1.0 equiv.) and dry THF (23.6 mL). $LiAlH_4$ (1 M in THF, 8.40 mL, 8.40 mmol, 1.05 equiv.) was transferred dropwise to the stirred colorless solution. The resulting colorless reaction mixture was stirred at room temperature for 2 h. After the elapsed time, propan-2-ol (4 mL) was added dropwise (*Caution: gas evolution upon addition*), and the mixture was stirred for 15 min at room temperature. Then, the colorless suspension was poured into water (100 mL) and the aqueous phase was extracted with MTBE (3 × 100 mL). The combined organic layers were dried over Na_2SO_4, filtered and concentrated under reduced pressure to afford the crude product as a colorless solid (1.99 g, 7.81 mmol, 98% yield) which was used in the subsequent step without any further purification. Diol **S21** (1.99 g, 7.81 mmol, 1.0 equiv.) was transferred to a flame-dried 100 mL two-neck round-bottom flask fitted with an argon outlet and a rubber septum. The reaction vessel was subjected to vacuum/argon cycles (3 ×). The solid was dissolved in dry pyridine (8.0 mL, 1 M) and *p*-toluenesulfonyl chloride (3.05 g, 16.0 mmol, 2.0 equiv.) was added under a counterflow of argon (slight exotherm observed during the addition). The resulting pale brown solution was stirred at room temperature overnight. After 16 h, aq. sat. $NaHCO_3$ (50 mL) was added carefully, the reaction mixture was diluted with MTBE (50 mL), and the aqueous phase was extracted with MTBE (3 × 50 mL). The combined organic layers were washed with aq. sat. $NaHCO_3$ (50 mL), water (50 mL), HCl (10 wt% in water, 50 mL), water (50 mL) and brine (50 mL), dried over $MgSO_4$, filtered and concentrated under reduced pressure. Purification by flash column chromatography on silica gel using hexanes/MTBE (19:1 *v/v*, isocratic elution) as eluent afforded the title compound **2b** as a colorless oil (1.49 g, 7.44 mmol, 85% yield) that solidified upon storage on dry-ice. Crystals suitable for X-ray diffraction were grown by repeated warming and slow cooling of the melt. Capillary crystallization provided crystals of sufficient quality to confirm the absolute configuration. *Note:* (−)-9-*epi*-ambrox has been previously isolated as a colorless oil. The crystalline form obtained herein enabled the first characterization of this olfactory important diastereomer by single-crystal X-ray diffraction.

Characterization data for (−)-(9β*H*)-13,14,15,16-tetranor-8α,12-diol (S21, 9-*epi*-ambradiol):

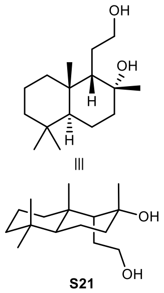

S21
(−)-9-*epi*-ambradiol

Physical state: colorless solid.

^{1}H NMR (501 MHz, $CDCl_3$): δ (ppm) = 3.75 (ddd, *J* = 9.5, 5.6, 3.6 Hz, 1H), 3.39 (td, *J* = 10.2, 4.4 Hz, 1H), 2.87 (br s, 1H, OH), 2.23 (br s, 1H, OH), 1.97 (dddd, *J* = 14.7, 10.7, 5.7, 3.8 Hz, 1H), 1.74 (dq, *J* = 14.9, 3.9 Hz, 1H), 1.66 (tt, *J* = 12.8, 3.2 Hz, 1H), 1.56 (tt, *J* = 11.0, 3.6 Hz, 4H), 1.48 (s, 3H), 1.44–1.34 (m, 2H), 1.33–1.23 (m, 2H), 1.16–1.06 (m, 2H), 1.09 (s, 3H), 0.99 (dd, *J* = 12.4, 2.6 Hz, 1H), 0.85 (s, 3H), 0.78 (s, 3H).

^{13}C{^{1}H} NMR (126 MHz, $CDCl_3$): δ (ppm) = 73.1, 64.6, 58.0, 46.6, 42.4, 38.8, 38.1, 36.2, 33.2, 33.0, 32.2, 29.6, 24.7, 21.4, 20.9, 18.6.

IR (ATR, neat): $\tilde{\upsilon}_{max}$ (cm^{-1}) = 3338 (br w), 2956 (w), 2940 (w), 2917 (w), 2872 (w), 1463 (w), 1382 (w), 1108 (w), 1108 (w), 1089 (w), 1035 (m), 1021 (s), 1007 (m), 972 (w), 936 (w), 903 (w), 894 (w), 783 (w), 645 (m), 629 (m), 587 (w), 564 (w), 546 (w).

HRMS (ESI^+): *m/z* calcd. for $C_{16}H_{30}O_2Na$ $[M+Na]^+$: 277.213799, found: 277.214010.

$[\alpha]_D^{25}$ = −13.5 (*c* = 0.8, $CHCl_3$); Lit.: $[\alpha]_D^{20}$ = −13.6 (*c* = 1, $CHCl_3$).[26]

Characterization data for (−)-9-*epi*-ambrox (2b):

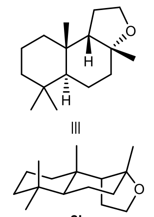

2b
(−)-9-*epi*-ambrox

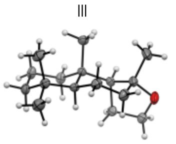

Physical state: colorless crystalline solid.

M.p.: 31.5–32.5 °C (MTBE).

TLC (SiO_2, hexanes/MTBE 19:1, *v/v*): R_f = 0.20 (CAM stain).

^{1}H NMR (600 MHz, $CDCl_3$): δ (ppm) = 3.84 (ddd, *J* = 9.6, 8.4, 3.1 Hz, 1H), 3.76 (td, *J* = 8.9, 8.4, 8.0 Hz, 1H), 2.03 (tt, *J* = 12.1, 9.6, 8.9 Hz, 1H), 1.90 (dtd, *J* = 12.2, 8.0, 3.1 Hz, 1H), 1.69–1.60 (m, 1H), 1.59–1.48 (m, 4H), 1.42–1.37 (m, 2H), 1.36 (d, *J* = 0.7 Hz, 3H), 1.29–1.25 (m, 2H), 1.26–1.20 (m, 1H), 1.18 (dd, *J* = 12.3, 1.7 Hz, 1H), 1.18–1.10 (m, 1H), 1.09 (s, 3H), 0.88 (s, 3H), 0.81 (s, 3H).

^{13}C{^{1}H} NMR (151 MHz, $CDCl_3$): δ (ppm) = 81.0, 64.2, 59.1, 46.9, 42.5, 38.8, 36.2, 35.9, 33.7, 33.1, 29.0, 27.8, 23.0, 21.9, 20.6, 18.6.

IR (ATR, neat): $\tilde{\upsilon}_{max}$ (cm^{-1}) = 2932 (s), 2867 (s), 1459 (m), 1383 (m), 1209 (w), 1127 (m), 1098 (m), 1060 (s), 1048 (s), 970 (w), 943 (w), 861 (w), 843 (w), 637 (w), 537 (w).

HRMS (GC-EI^+): *m/z* calcd. for $C_{16}H_{28}O$ $[M]^+$: 236.213465, found: 236.213330.

$[\alpha]_D^{25}$ = −8.0 (*c* = 1.0, $CHCl_3$); Lit.: −8.0 (*c* = 0.98, $CHCl_3$).[27]

GC (achiral) (DB-1 0.25/0.25df G/701, 30.0 m, temperature: 220/60 5/min 200 12/min 300, 3 min iso / 350, 0.5 bar H_2, sample size: 0.2 µL): (−)-9-*epi*-ambrox: t_R (**2b**) = 24.98 min (99.95%).

GC (chiral) (G-TA 0.25/?df; G/448, 30.0 m, temperature: 220/85 500 min iso 6/min 180, 3 min iso / 350, 0.5 bar H_2, sample size 1.0 µL): (−)-9-*epi*-ambrox: t_R (**2b**) = 414.06 min (>99%).

X-Ray Crystallography: CCDC-2338448.

NMR assignment for (−)-9-*epi*-Ambrox (2b):

Table S1 | Tabulated NMR assignments (^{1}H NMR: 600 MHz, ^{13}C NMR: 151 MHz, $CDCl_3$) for (−)-9-*epi*-ambrox (**2b**) with an ORTEP representation of the X-ray crystal structure of **2b** highlighting key NOE interactions for assignment of the stereochemistry by NMR spectroscopy. Thermal ellipsoids (anisotropic displacement parameters) are drawn at 50% probability level.

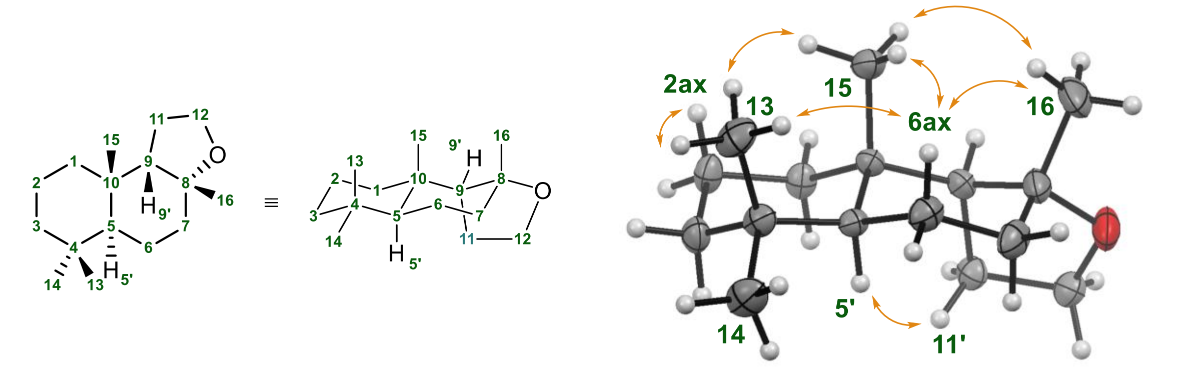

Atom	δ (ppm)	*J*	COSY	HSQC	HMBC	NOESY
1 C	38.808			1	2eq, 3ax, 3eq, 5', 15	
H2	1.266		2ax, 2eq, 15	1	2, 3, 5, 9, 10, 15	11', 11", 15
2 C	18.649			2ax, 2eq	1, 3ax, 3eq	
Hax	1.640		1, 3ax, 3eq	2	3, 4, 10	13, 15
Heq	1.393		1, 3ax, 3eq	2	1, 3, 4, 10	
3 C	42.452			3ax, 3eq	1, 2ax, 2eq, 13, 14	
Hax	1.140		2ax, 2eq, 3ax, 13	3	1, 2, 4, 5, 13, 14	
Heq	1.398		2ax, 2eq, 3eq	3	1, 2, 4, 5	13
4 C	33.062				2ax, 2eq, 3ax, 3eq, 5', 6ax, 6eq, 13, 14	
5 C	46.855			5'	1, 3ax, 3eq, 6ax, 6eq, 7', 7", 9, 13, 14, 15	
5' H	1.181	12.30(6ax), 1.80(6eq)	6ax, 6eq	5	1, 4, 6, 7, 9, 10, 13, 14, 15	11', 14
6 C	20.552			6ax, 6eq	5', 7', 7"	
Hax	1.240	12.30(5')	5', 6eq, 7"	6	4, 5, 7, 10	13
Heq	1.543	1.80(5')	5', 6ax, 7"	6	4, 5, 7, 8, 10	14
7 C	35.913			7', 7"	5', 6ax, 6eq, 9, 16	
H'	1.527		16	7	5, 6, 8, 9, 16	
H"	1.544		6ax, 6eq	7	5, 6, 8, 9, 16	12"
8 C	80.962				6eq, 7', 7", 9, 11", 12', 12", 16	

Atom	δ (ppm)	*J*	COSY	HSQC	HMBC	NOESY
9 C	59.146			9	1, 5', 7', 7", 11', 11", 12', 12", 15, 16	
H	1.562	8.00(11"), 12.10(11')	11', 11"	9	5, 7, 8, 10, 11, 15	12', 15, 16
10 C	36.160				1, 2ax, 2eq, 5', 6ax, 6eq, 9, 11', 15	
11 C	28.980			11', 11"	9, 12', 12"	
H'	2.035	12.10(9), 12.20(11"), 8.90(12'), 9.60(12")	9, 11", 12', 12"	11	9, 10, 12	1, 5'
H"	1.900	8.00(9), 8.00(12'), 3.00(12"), 12.20(11')	9, 11', 12', 12"	11	8, 9	1, 12'
12 C	64.216			12', 12"	11'	
H'	3.761	8.00(11"), 8.40(12"), 8.90(11')	11', 11"	12	8, 9, 11	9, 11"
H"	3.841	3.00(11"), 8.40(12'), 9.60(11')	11', 11"	12	8, 9, 11	7"
13 C	21.900			13	3ax, 5', 14	
H3	0.808		3ax, 14	13	3, 4, 5, 14	2ax, 3eq, 6ax, 14, 15, 16
14 C	33.719			14	3ax, 5', 13	
H3	0.883		13	14	3, 4, 5, 13	5', 6eq, 13
15 C	22.967			15	1, 5', 9	
H3	1.091		1	15	1, 5, 9, 10	1, 2ax, 9, 13, 16
16 C	27.842			16	7', 7"	
H3	1.364		7'	16	7, 8, 9	9, 13, 15

1.4 Catalyst Synthesis

1.4.1 Synthesis of Imidodiphosphorimidate (IDPi) Catalysts

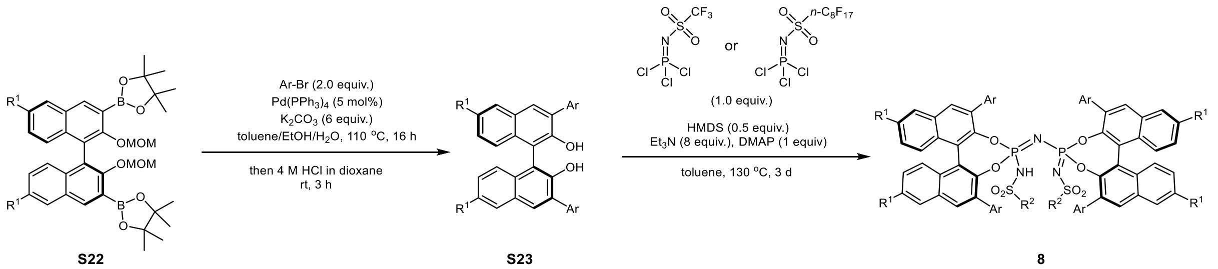

To a two-neck round-bottom flask with a condenser was added **S22** (0.50 mmol, 1.0 equiv.), the respective aryl bromide (Ar–Br, 1.00 mmol, 2.0 equiv), tetrakis(triphenylphosphine)palladium (25.0 µmol, 0.05 equiv) and K_2CO_3 (3.00 mmol, 6.0 equiv) under an atmosphere of argon. Degassed toluene (3 mL), ethanol (2 mL) and water (1 mL) were sequentially added. The mixture was then heated to 110 °C and stirred overnight. After cooling to room temperature, the organic layer was separated and the aqueous phase was extracted with ethyl acetate (3 × 10 mL). The organic phase was combined, filtered through a thin layer of silica gel using a Büchner funnel, and the silica gel layer was eluted with additional ethyl acetate. The solvent was removed under reduced pressure and the crude MOM-protected diol was obtained. Subsequently, the crude product was dissolved in a small amount of DCM (1 mL). A solution of HCl (4 M in 1,4-dioxane, 3 mL) was added at room temperature and the mixture was stirred for 3 h. The solvent was evaporated under reduced pressure and the crude product was purified by column chromatography to afford the corresponding diol **S23**.

In a Schlenk tube under argon, a suspension of 3,3'-substituted (*S,S*)-BINOL (**S23**, 0.21 mmol, 2.1 equiv.) in toluene (0.8 mL) was treated with ((trifluormethyl)sulfonyl)phosphorimidoyl trichloride[28] or ((perfluorooctyl)sulfonyl)phosphorimidoyl trichloride[29] (0.21 mmol, 2.1 equiv.) and triethyl amine (1.60 mmol, 16 equiv.). The reaction mixture was stirred for 45 min at room temperature, then 4-dimethylaminopyridine (DMAP, 0.10 mmol, 1.0 equiv.) and neat 1,1,1,3,3,3-hexamethyldisilazane (HMDS, 0.10 mmol, 1.0 equiv.) were added. The reaction mixture was stirred for additional 15 min at room temperature, the Schlenk tube was subsequently sealed and heated to 130 °C for 3 d. After cooling to room temperature, aqueous HCl (10%) was added and the mixture was extracted with DCM. The combined organic layers were washed with brine, dried over $MgSO_4$, and concentrated under reduced pressure. The crude material was purified by column chromatography on silica gel to afford the desired product as a salt. The corresponding IDPi Brønsted acids were obtained after acidification in DCM with aq. HCl (6 M) and evaporation of the solvent followed by drying under high vacuum as typically off-white solids. Acidification procedure: The salt was dissolved in DCM (5 mL), then aq. HCl (6 M, 8 mL) was added, and the mixture was stirred vigorously for 10 minutes. The organic layer was separated and the aqueous phase was extracted several times with DCM until no product could be detected by TLC in the last DCM extract. The organic layers were combined and the solvent was removed under reduced pressure. The obtained IDPi solid was dried under high vacuum for 16 h at room temperature.

(*S*)-3,3'-Di-*p*-tolyl-[1,1'-binaphthalene]-2,2'-diol (S23a)

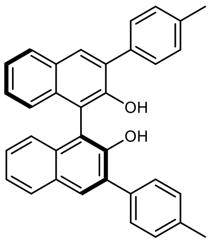

S23a

87% yield; **^{1}H NMR** (501 MHz, $CDCl_3$): δ (ppm) = 8.00 (s, 2H), 7.91 (dd, *J* = 8.1, 1.3 Hz, 2H), 7.68–7.57 (m, 4H), 7.38 (ddd, *J* = 8.1, 6.7, 1.3 Hz, 2H), 7.34–7.27 (m, 6H), 7.23 (dd, *J* = 8.5, 1.1 Hz, 2H), 5.35 (s, 2H), 2.43 (s, 6H).

^{13}C NMR (126 MHz, $CDCl_3$): δ (ppm) = 150.3, 137.8, 134.7, 133.0, 131.3, 130.8, 129.6, 129.4, 128.5, 127.3, 124.5, 124.4, 112.6, 21.4.

HRMS (ESI^-): *m/z* calcd. for $C_{34}H_{26}O_2$ $[M-H]^-$: 465.1860, found 465.1864.

(*S*)-3,3'-Bis(4-isopropylphenyl)-[1,1'-binaphthalene]-2,2'-diol (S23b)

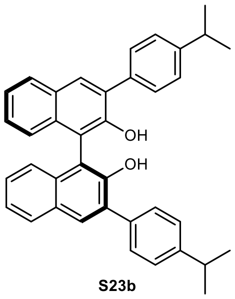

S23b

89% yield; **^{1}H NMR** (501 MHz, $CDCl_3$): 8.02 (s, 2H), 7.91 (dd, *J* = 8.3, 1.4 Hz, 2H), 7.71 – 7.62 (m, 4H), 7.41 – 7.34 (m, 6H), 7.30 (ddd, *J* = 8.3, 6.8, 1.4 Hz, 2H), 7.22 (dd, *J* = 8.3, 1.1 Hz, 2H), 2.99 (hept, *J* = 6.9 Hz, 2H), 1.32 (d, *J* = 6.9 Hz, 12H).

^{13}C NMR (126 MHz, $CDCl_3$): δ (ppm) = 150.3, 148.7, 135.0, 133.0, 131.3, 130.8, 129.7, 129.6, 128.5, 127.3, 126.8, 124.5, 124.4, 112.6, 34.1, 24.1.

HRMS (ESI^-): *m/z* calcd. for $C_{38}H_{34}O_2$ $[M-H]^-$: 521.2486, found 521.2487.

(*S*)-3,3'-bis(4-(*tert*-butyl)phenyl)-[1,1'-binaphthalene]-2,2'-diol (S23c)

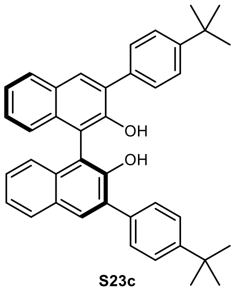

S23c

90% yield; **^{1}H NMR** (501 MHz, $CDCl_3$): δ (ppm) = 8.03 (s, 2H), 7.92 (dd, *J* = 7.8, 1.1 Hz, 2H), 7.74–7.64 (m, 4H), 7.57–7.49 (m, 4H), 7.39 (ddd, *J* = 8.1, 6.8, 1.3 Hz, 2H), 7.31 (ddd, *J* = 8.2, 6.8, 1.3 Hz, 2H), 7.23 (dd, *J* = 8.4, 1.1 Hz, 2H), 5.39 (s, 2H), 1.39 (s, 18H).

^{13}C NMR (126 MHz, $CDCl_3$): δ (ppm) = 150.9, 150.4, 134.6, 133.0, 131.3, 130.7, 129.6, 129.4, 128.5, 127.3, 125.7, 124.5, 124.4, 112.6, 34.8, 31.5.

HRMS (ESI^-): *m/z* calcd. for $C_{40}H_{38}O_2$ $[M-H]^-$: 549.2799, found 549.2805.

(*S*)-3,3'-bis(4-(*tert*-pentyl)phenyl)-[1,1'-binaphthalene]-2,2'-diol (S23d)

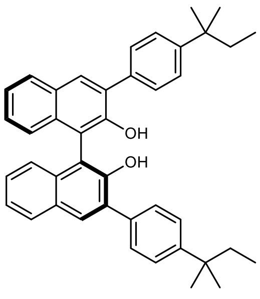

S23d

90% yield; **^{1}H NMR** (501 MHz, $CDCl_3$): δ (ppm) = 8.04 (s, 2H), 7.92 (dd, *J* = 8.3, 1.1 Hz, 2H), 7.73–7.63 (m, 4H), 7.50–7.43 (m, 4H), 7.38 (ddd, *J* = 8.1, 6.7, 1.3 Hz, 2H), 7.31 (ddd, *J* = 8.2, 6.8, 1.3 Hz, 2H), 7.23 (dd, *J* = 8.5, 1.2 Hz, 2H), 5.39 (s, 2H), 1.71 (q, *J* = 7.4 Hz, 4H), 1.35 (s, 12H), 0.76 (t, *J* = 7.4 Hz, 6H).

^{13}C NMR (126 MHz, $CDCl_3$): δ (ppm) = 150.4, 149.4, 134.5, 133.0, 131.3, 130.7, 129.6, 129.3, 128.5, 127.3, 126.3, 124.5, 124.4, 112.6, 38.0, 37.0, 28.6, 9.36.

HRMS (ESI^-): *m/z* calcd. for $C_{42}H_{42}O_2$ $[M-H]^-$: 577.3112, found 577.3116.

(*S*)-6,6'-Bis(perfluoropropan-2-yl)-3,3'-di(spiro[cyclobutane-1,9'-fluoren]-2'-yl)-[1,1'-binaphthalene]-2,2'-diol (S23e)

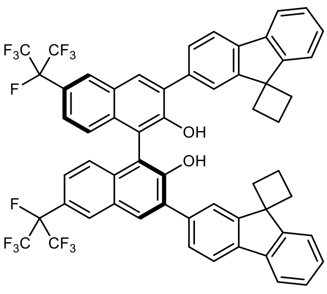

S23e

82% yield; ^{1}**H NMR** (501 MHz, $CDCl_3$): δ (ppm) = 8.27 (d, *J* = 2.0 Hz, 2H), 8.23 (s, 2H), 8.10 (d, *J* = 1.6 Hz, 2H), 7.82 (dd, *J* = 7.6, 1.6 Hz, 4H), 7.74 (dd, *J* = 7.2, 1.6 Hz, 2H), 7.69 (dd, *J* = 7.8, 1.6 Hz, 2H), 7.53 (dd, *J* = 9.0, 1.9 Hz, 2H), 7.45–7.35 (m, 6H), 5.68 (s, 2H), 2.78–2.64 (m, 8H), 2.49–2.41 (m, 4H).

^{13}**C NMR** (126 MHz, $CDCl_3$): δ (ppm) = 153.2, 152.8, 151.9, 139.9, 138.8, 135.6, 134.0, 132.8, 132.2, 128.8, 128.6, 128.2, 127.3, 127.2, 127.1, 125.5, 124.1, 123.3, 122.8, 120.00, 119.97, 112.9, 52.2, 33.4, 17.2 (other signals not detected or observed).

^{19}**F NMR** (471 MHz, $CDCl_3$): δ (ppm) = −75.39 (dq, *J* = 14.9, 7.8 Hz), −181.89 (p, *J* = 8.7 Hz).

HRMS (ESI^-): *m/z* calcd. for $C_{58}H_{36}O_2F_{14}$ $[M-H]^-$: 1029.2419, found 1029.2426.

(*S*)-6,6'-bis(perfluoropropan-2-yl)-3,3'-di(spiro[cyclopentane-1,9'-fluoren]-2'-yl)-[1,1'-binaphthalene]-2,2'-diol (S23f)

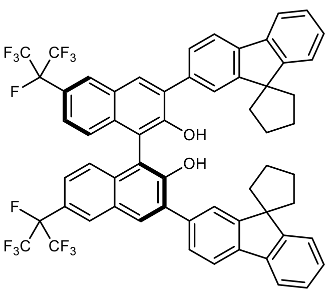

S23f

Physical state: colorless solid.

^{1}**H NMR** (600 MHz, $CDCl_3$): δ (ppm) = 8.26 (d, *J* = 2.0 Hz, 2H), 8.19 (d, *J* = 0.7 Hz, 2H), 7.85 (dd, *J* = 7.9, 0.6 Hz, 2H), 7.79–7.74 (m, 4H), 7.68 (dd, *J* = 7.8, 1.6 Hz, 2H), 7.52 (dd, *J* = 8.9, 2.2 Hz, 2H), 7.48 (ddd, *J* = 6.7, 1.8, 0.7 Hz, 2H), 7.40 (dq, *J* = 9.0, 0.8 Hz, 2H), 7.36 (pd, *J* = 7.4, 1.4 Hz, 4H), 5.65 (s, 2H), 2.22–2.11 (m, 16H).

^{13}**C{^{1}H} NMR** (126 MHz, $CDCl_3$): δ (ppm) = 155.2, 154.8, 151.8, 140.1, 138.9, 135.4, 134.0, 134.0, 132.8, 132.1, 128.7 (d, $^{4}J_{C-F}$ = 2.2 Hz), 128.3, 128.1, 127.1 (d, $^{3}J_{C-F}$ = 11.6 Hz), 127.0, 125.5 (d, $^{4}J_{C-F}$ = 2.4 Hz), 124.2, 123.3 (d, $^{3}J_{C-F}$ = 9.7 Hz), 123.1, 122.7 (d, $^{2}J_{C-F}$ = 20.4 Hz), 120.9 (qdd, $^{1}J_{C-F}$ = 287.3, $^{2}J_{C-F}$ = 27.9, $^{3}J_{C-F}$ = 9.5 Hz), 120.1, 120.0, 112.8, 91.8 (dhept, $^{1}J_{C-F}$ = 201.9, $^{2}J_{C-F}$ = 32.6 Hz), 58.0, 40.0, 39.9, 27.1, 27.1.

^{19}**F NMR** (565 MHz, $CDCl_3$): δ (ppm) = −75.38 (dq, *J* = 21.2, 7.5 Hz, 12F), −181.86 (hept, *J* = 7.3 Hz, 2F).

HRMS (ESI^-): *m/z* calcd. for $C_{60}H_{39}F_{14}O_2$ $[M–H]^-$: 1057.273204, found: 1057.274040.

(*S*)-6,6'-bis(perfluoropropan-2-yl)-3,3'-di(spiro[cyclohexane-1,9'-fluoren]-2'-yl)-[1,1'-binaphthalene]-2,2'-diol (S23g)

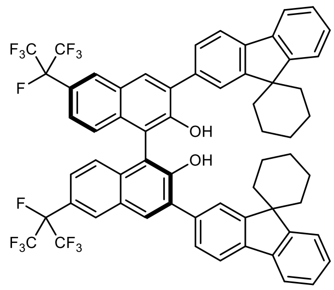

S23g

85% yield; ^{1}**H NMR** (501 MHz, $CDCl_3$): δ (ppm) = 8.27 (d, *J* = 2.0 Hz, 2H), 8.20 (s, 2H), 7.99 (d, *J* = 1.6 Hz, 2H), 7.88 (d, *J* = 7.8 Hz, 2H), 7.81 (dd, *J* = 7.4, 1.3 Hz, 2H), 7.71 (dd, *J* = 7.8, 1.6 Hz, 4H), 7.52 (dd, *J* = 9.3, 1.9 Hz, 2H), 7.43–7.38 (m, 4H), 7.35 (td, *J* = 7.4, 1.3 Hz, 2H), 5.66 (s, 2H), 2.02–1.91 (m, 8H), 1.90–1.76 (m, 12H).

^{13}**C NMR** (126 MHz, $CDCl_3$): δ (ppm) = 154.1, 153.6, 151.9, 140.2, 139.1, 134.8, 134.0, 132.9, 132.2, 128.7, 127.4, 127.2, 127.1, 125.7, 125.5, 124.8, 123.3, 122.8, 122.6, 120.27, 120.25, 112.8, 50.7, 35.8, 25.7, 22.9 (other signals not detected or observed).

^{19}**F NMR** (471 MHz, $CDCl_3$): δ (ppm) = −75.40 (dq, *J* = 16.4, 8.1 Hz), −181.90 (p, *J* = 7.7 Hz).

HRMS (ESI^-): *m/z* calcd. for $C_{62}H_{44}O_2F_{14}$ $[M–H]^-$: 1085.3045, found 1085.3047.

(*S*)-Imidodiphosphorimidate (8a)

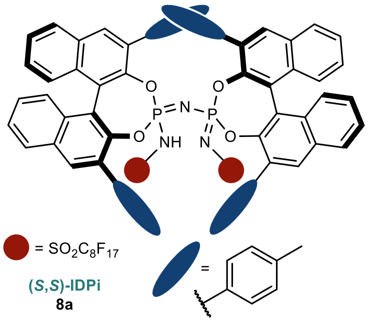

56% yield; **^{1}H NMR** (501 MHz, $CDCl_3$): δ (ppm) = 8.09 (s, 2H), 8.02 (d, *J* = 8.2 Hz, 4H), 7.79 (ddd, *J* = 8.1, 6.7, 1.1 Hz, 2H), 7.70 (d, *J* = 8.5 Hz, 2H), 7.60 (ddd, *J* = 8.3, 6.7, 1.3 Hz, 2H), 7.55 (ddd, *J* = 8.1, 6.4, 1.5 Hz, 2H), 7.41–7.30 (m, 4H), 7.15 (d, *J* = 8.2 Hz, 4H), 7.10 (d, *J* = 8.0 Hz, 4H), 7.07 (s, 2H), 6.85 (d, *J* = 7.9 Hz, 4H), 6.47 (d, *J* = 7.9 Hz, 4H), 2.28 (s, 6H), 2.19 (s, 6H).

^{13}C NMR (126 MHz, $CDCl_3$): δ (ppm) = 143.8, 143.2, 138.0, 137.9, 133.8, 133.5, 132.62, 132.56, 132.3, 132.1, 131.9, 131.7, 131.6, 130.7, 129.6, 129.0, 128.9, 128.4, 127.3, 127.2, 127.1, 126.8, 126.5, 123.5, 121.9, 21.4, 20.8 (other signals not detected or observed).

^{19}F NMR (471 MHz, $CDCl_3$): δ (ppm) = −80.78 (t, *J* = 9.7 Hz, 6F), −110.68 – −112.71 (m, 4F), −119.65 – −120.36 (m, 4F), −121.27 – −121.56 (m, 4F), −121.55 – −121.79 (m, 4F), −121.78 – −122.05 (m, 4F), −122.51 – −122.92 (m, 4F), −126.09 (td, *J* = 15.0, 6.2 Hz, 4F).

^{31}P NMR (203 MHz, $CDCl_3$): δ (ppm) = −17.46.

HRMS (ESI^-): *m/z* calcd. for $C_{84}H_{49}O_8N_3F_{34}P_2S_2$ $[M–H]^-$: 1998.1821, found 1998.1855.

(*S*)-Imidodiphosphorimidate (8b)

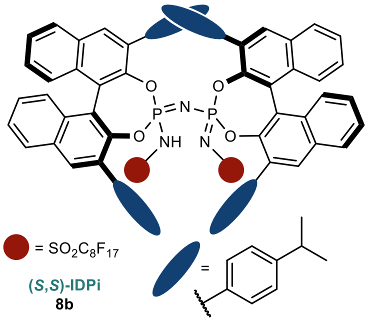

62% yield; **^{1}H NMR** (501 MHz, $CDCl_3$) δ (ppm) = 8.13 (s, 2H), 8.03 (d, *J* = 8.3 Hz, 2H), 7.99 (d, *J* = 8.2 Hz, 2H), 7.80 (ddd, *J* = 8.0, 6.6, 1.2 Hz, 2H), 7.70 (d, *J* = 8.5 Hz, 2H), 7.62 (ddd, *J* = 8.3, 6.7, 1.3 Hz, 2H), 7.55 (ddd, *J* = 8.1, 5.4, 2.5 Hz, 2H), 7.39–7.29 (m, 4H), 7.22 (d, *J* = 8.4 Hz, 4H), 7.17 (d, *J* = 8.1 Hz, 4H), 7.06 (s, 2H), 6.90 (d, *J* = 7.9 Hz, 4H), 6.63 (d, *J* = 8.4 Hz, 4H), 2.85 (h, *J* = 7.0 Hz, 2H), 2.77 (h, *J* = 6.9 Hz, 2H), 1.22 (d, *J* = 6.9 Hz, 6H), 1.21 (d, *J* = 7.0 Hz, 6H), 1.13 (d, *J* = 6.9 Hz, 6H), 1.00 (d, *J* = 6.9 Hz, 6H).**^{13}C NMR** (126 MHz, $CDCl_3$) δ (ppm) = 149.0, 148.4, 144.1, 143.2, 133.61, 133.55, 133.0, 132.9, 132.3, 132.0, 131.9, 131.8, 131.7, 131.0, 129.9, 129.5, 129.1, 128.9, 127.3, 127.22, 127.17, 126.7, 126.5, 126.4, 125.6, 123.4, 121.9, 33.9, 33.4, 23.8, 23.6, 23.5 (other signals not detected or observed).

^{19}F NMR (471 MHz, $CDCl_3$): δ (ppm) = −80.80 (t, *J* = 10.0 Hz, 6F), −110.71 – −112.06 (m, 4F), −119.74 – −120.06 (m, 4F), −121.21 – −121.58 (m, 4F), −121.60 – −121.81 (m, 4F), -121.80 – −122.07 (m, 4F), −122.51 – −123.02 (m, 4F), −126.11 (td, *J* = 14.8, 6.5 Hz, 4F).

^{31}P NMR (203 MHz, $CDCl_3$): δ (ppm) = −16.85.

HRMS (ESI^-): *m/z* calcd. for $C_{92}H_{65}O_8N_3F_{34}P_2S_2$ $[M–H]^-$: 2110.3073, found 2110.3108.

(*S*)-Imidodiphosphorimidate (8c)

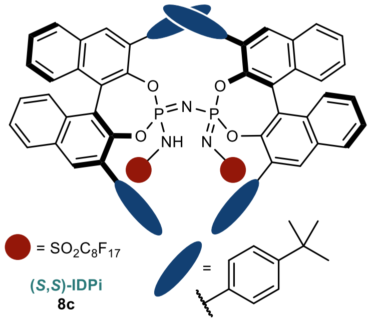

65% yield; ^{1}**H NMR** (501 MHz, CDCl$_3$) δ (ppm) = 8.13 (s, 2H), 8.01 (d, *J* = 8.2 Hz, 2H), 7.95 (d, *J* = 8.3 Hz, 2H), 7.78 (t, *J* = 7.2 Hz, 2H), 7.67 (d, *J* = 8.6 Hz, 2H), 7.61 (ddd, *J* = 8.3, 6.6, 1.3 Hz, 2H), 7.53 (ddd, *J* = 8.2, 5.9, 1.8 Hz, 2H), 7.35–7.27 (m, 8H), 7.26–7.20 (m, 4H), 7.10–6.95 (m, 6H), 6.67 (d, *J* = 8.4 Hz, 4H), 1.26 (s, 18H), 1.10 (s, 18H).

^{13}**C NMR** (126 MHz, CDCl$_3$): δ (ppm) = 151.3, 150.9, 144.2, 143.2, 133.5, 133.3, 132.7, 132.5, 132.3, 132.0, 131.82, 131.76, 131.67, 131.2, 129.7, 129.2, 128.9, 127.4, 127.2, 126.6, 126.5, 126.3, 125.5, 124.6, 123.4, 121.9, 34.2, 31.2, 31.0 (other signals not detected or observed).

^{19}**F NMR** (471 MHz, CDCl$_3$): δ (ppm) = −80.79 (t, *J* = 10.0 Hz, 6F), −110.56 – −112.11 (m, 4F), −119.69 – −120.15 (m, 4F), −121.15 – −121.53 (m, 4F), −121.66 – −121.85 (m, 4F), −121.85 – −122.05 (m, 4F), −122.51 – −122.91 (m, 4F), −126.11 (td, *J* = 15.4, 7.0 Hz, 4F).

^{31}**P NMR** (203 MHz, CDCl$_3$): δ (ppm) = −16.62.

HRMS (ESI$^-$): *m/z* calcd. for $C_{96}H_{73}O_8N_3F_{34}P_2S_2$ [M-H]$^-$: 2166.3699, found 2166.3699.

(*S*)-Imidodiphosphorimidate (8d)

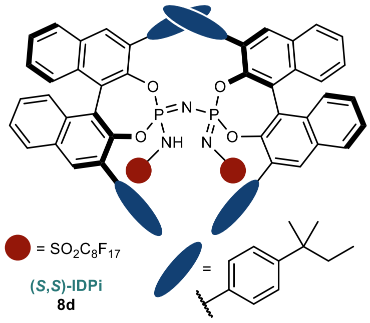

70% yield; ^{1}**H NMR** (501 MHz, CDCl$_3$): δ (ppm) = 8.13 (s, 2H), 8.00 (d, *J* = 8.2 Hz, 2H), 7.94 (d, *J* = 8.3 Hz, 2H), 7.77 (t, *J* = 7.5 Hz, 2H), 7.64 (d, *J* = 8.5 Hz, 2H), 7.59 (dd, *J* = 8.7, 6.5 Hz, 2H), 7.52 (t, *J* = 7.4 Hz, 2H), 7.31–7.26 (m, 4H), 7.26–7.22 (m, 8H), 7.01 (s, 2H), 6.94 (d, *J* = 8.3 Hz, 4H), 6.67 (d, *J* = 8.2 Hz, 4H), 1.66–1.50 (m, 4H), 1.46–1.35 (m, 4H), 1.22 (s, 6H), 1.21 (s, 6H), 1.11 (s, 6H), 0.99 (s, 6H), 0.63 (t, *J* = 7.4 Hz, 6H), 0.52 (t, *J* = 7.4 Hz, 6H).

^{13}**C NMR** (126 MHz, CDCl$_3$): δ (ppm) = 149.70, 149.65, 144.3, 143.2, 133.4, 132.6, 132.5, 132.4, 132.0, 131.8, 131.2, 129.7, 129.2, 129.0, 128.9, 127.4, 127.2, 127.1, 126.6, 126.5, 126.3, 126.2, 125.2, 123.3, 121.8, 37.8, 37.6, 37.1, 36.7, 29.9, 28.2, 28.0, 27.9, 27.4 (other signals not detected or observed).

^{19}**F NMR** (471 MHz, CDCl$_3$): δ (ppm) = −80.78 (t, *J* = 10.2 Hz, 6F), −110.37 – −111.93 (m, 4F), −119.71 – −120.06 (m, 4F), −121.22 – −121.48 (m, 4F), −121.63 – −121.85 (m, 4F), −121.82 – −122.11 (m, 4F), −122.54 – −122.87 (m, 4F), −126.10 (dt, *J* = 17.8, 9.1 Hz, 4F).

^{31}**P NMR** (203 MHz, CDCl$_3$): δ (ppm) = −16.31.

HRMS (ESI$^-$): *m/z* calcd. for $C_{100}H_{81}O_8N_3F_{34}P_2S_2$ [M−H]$^-$: 2222.4325, found 2222.4331.

(*S*)-Imidodiphosphorimidate (8e)

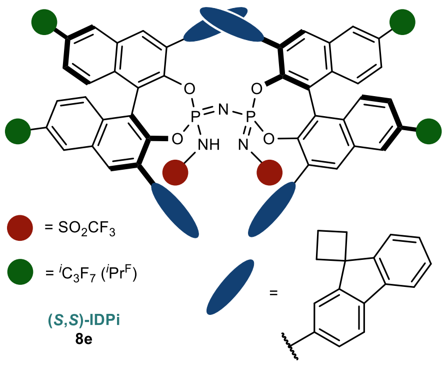

60% yield; **^{1}H NMR** (501 MHz, $CDCl_3$): δ (ppm) = 8.40 (t, *J* = 2.5 Hz, 4H), 8.24 (s, 2H), 7.99 (d, *J* = 9.1 Hz, 2H), 7.94 (d, *J* = 9.2 Hz, 2H), 7.83 (d, *J* = 7.6 Hz, 2H), 7.81 (d, *J* = 1.6 Hz, 2H), 7.69–7.59 (m, 8H), 7.51 (d, *J* = 7.6 Hz, 2H), 7.36–7.29 (m, 4H), 7.28–7.25 (m, 4H), 7.24–7.16 (m, 6H), 6.47 (d, *J* = 7.9 Hz, 2H), 6.40 (d, *J* = 8.0 Hz, 2H), 5.76 (d, *J* = 7.9 Hz, 2H), 2.86–2.75 (m, 2H), 2.72–2.65 (m, 2H), 2.64–2.50 (m, 6H), 2.44–2.19 (m, 14H).

^{13}C NMR (126 MHz, $CDCl_3$): δ (ppm) = 140.3, 139.3, 138.6, 138.2, 136.7, 136.5, 134.1, 133.0, 132.7, 132.3, 132.0, 131.5, 131.1, 128.8, 128.6, 128.1, 122.8, 127.5, 126.9, 124.3, 123.7, 123.4, 122.7, 122.6, 121.3, 120.0, 119.3, 118.7, 118.3, 52.1, 33.9, 33.1, 32.7, 32.5, 17.0 (other signals not detected or observed).

^{19}F NMR (471 MHz, $CDCl_3$): δ (ppm) = −74.99 – −75.12 (m, 12F), −75.16 – −75.25 (m, 12F), −78.34 (s, 6F), −181.86 (hept, *J* = 7.0 Hz, 2F), −182.06 (br s, 2F).

^{31}P NMR (203 MHz, $CDCl_3$): δ (ppm) = −15.98.

HRMS (ESI$^-$): *m/z* calcd. for $C_{118}H_{69}O_8N_3F_{34}P_2S_2$ [M−H]$^-$: 2426.3386, found 2426.3396.

(*S*)-Imidodiphosphorimidate (8f)

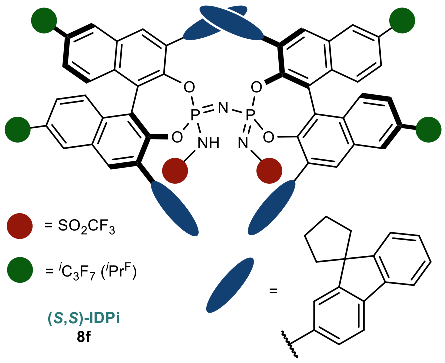

65% yield; **^{1}H NMR** (501 MHz, $CDCl_3$): δ (ppm) = 8.37 (t, *J* = 2.7 Hz, 4H), 8.19 (s, 2H), 7.94 (d, *J* = 9.1 Hz, 2H), 7.91 (d, *J* = 9.3 Hz, 2H), 7.64 (d, *J* = 9.2 Hz, 2H), 7.57 (d, *J* = 9.0 Hz, 2H), 7.54 (d, *J* = 7.5 Hz, 2H), 7.51 (d, *J* = 1.6 Hz, 2H), 7.46 (d, *J* = 7.6 Hz, 2H), 7.34–7.30 (m, 4H), 7.29–7.25 (m, 4H), 7.23 (d, *J* = 1.6 Hz, 2H), 7.22–7.16 (m, 8H), 6.58 (d, *J* = 8.0 Hz, 2H), 6.47 (d, *J* = 7.9 Hz, 2H), 5.84 (dd, *J* = 7.8, 1.6 Hz, 2H), 2.20–1.98 (m, 24H), 1.87–1.74 (m, 8H).

^{13}C NMR (126 MHz, $CDCl_3$): δ 155.0, 154.9, 154.2, 140.6, 139.7, 138.8, 138.3, 136.7, 136.4, 133.9, 133.1, 132.6, 132.2, 132.0, 131.5, 131.0, 128.5, 128.4, 127.9, 127.2, 136.6, 124.0, 123.8, 123.3, 123.0, 121.2, 120.1, 119.9, 119.3, 118.8, 118.6, 58.03, 57.97, 40.2, 40.0, 39.0, 27.2, 26.9, 26.8, 26.6 (other signals not detected or observed).

^{19}F NMR (471 MHz, $CDCl_3$): δ (ppm) = −74.99 – −75.14 (m, 12F), −75.17 – −75.28 (m, 12F), −78.33 (s, 6F), −181.88 (hept, *J* = 7.4 Hz, 2F), −182.06 (br s, 2F).

^{31}P NMR (203 MHz, $CDCl_3$): δ (ppm) = −16.38.

HRMS (ESI$^-$): *m/z* calcd. for $C_{122}H_{77}O_8N_3F_{34}P_2S_2$ [M−H]$^-$: 2482.4012, found 2482.4020.

(*S*)-Imidodiphosphorimidate (8g)

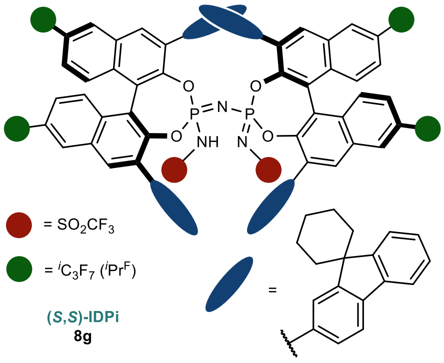

(*S*,*S*)-IDPi
8g

62% yield; ^{1}**H NMR** (501 MHz, $CDCl_3$): δ (ppm) = 8.37 (d, *J* = 5.6 Hz, 4H), 8.20 (s, 2H), 7.98 (d, *J* = 9.2 Hz, 2H), 7.90 (d, *J* = 9.1 Hz, 2H), 7.78 (d, *J* = 7.4 Hz, 2H), 7.72 (s, 2H), 7.63 (d, *J* = 9.1 Hz, 2H), 7.59 (app dd, *J* = 7.5, 4.1 Hz, 4H), 7.55 (d, *J* = 9.0 Hz, 2H), 7.38 (app t, *J* = 9.5 Hz, 4H), 7.31–7.19 (m, 10H), 7.17 (s, 2H), 6.63 (d, *J* = 8.1 Hz, 2H), 6.49 (d, *J* = 8.0 Hz, 2H), 6.09 (d, *J* = 7.9 Hz, 2H), 2.01–1.66 (m, 32H), 1.50–1.36 (m, 8H).

^{13}**C NMR** (126 MHz, $CDCl_3$): δ (ppm) = 155.0, 154.9, 154.2, 140.6, 139.7, 138.8, 138.3, 136.9, 135.7, 134.6, 133.9, 133.1, 132.6, 132.2, 132.0, 131.5, 131.0, 128.5, 128.4, 127.9, 127.7, 127.2, 126.6, 126.2, 125.4, 124.0, 123.8, 123.3, 123.0, 121.2, 120.1, 119.9, 119.3, 118.6, 58.03, 57.97, 40.2, 40.0, 39.0, 27.2, 26.9, 26.8, 26.6 (other signals not detected or observed).

^{19}**F NMR** (471 MHz, $CDCl_3$): δ (ppm) = −75.05 – −75.16 (m, 18F), −75.25 – −75.32 (m, 6F), −78.28 (s, 6F), −181.93 (hept, *J* = 7.4 Hz, 2F), −182.14 (br s, 2F).

^{31}**P NMR** (203 MHz, $CDCl_3$): δ (ppm) = −16.06.

HRMS (ESI$^-$): *m/z* calcd. for $C_{126}H_{85}O_8N_3F_{34}P_2S_2$ [M−H]$^-$: 2538.4638, found 2538.4640.

1.4.2 Synthesis of Phosphoramidimidate (PADI) Catalyst 9

Synthesis of PADI catalyst **9** was performed according to a reported literature procedure.[30] In a flame-dried Schlenk tube, (*S*)-BINOL **S23g** (108.7 mg, 0.10 mmol), *N*-triflylphosphorimidoyl trichloride (29.9 mg, 0.105 mmol), toluene (1.0 mL) and DIPEA (0.14 mL, 0.80 mmol) were added under argon atmosphere. The reaction mixture was stirred at room temperature for 30 min. Then, trifluoromethanesulfonamide (16.4 mg, 0.11 mmol) was added under a counterflow of argon. After the reaction mixture was stirred at room temperature for 5 h, aqueous HCl (10 mL, 1.0 M) was added and the mixture was extracted with DCM. The combined organic layers were washed with water and brine, dried over $MgSO_4$, filtered, and concentrated under reduced pressure. Purification by column chromatography on silica gel (isocratic elution with hexane/EtOAc 1:1 *v/v*) afforded the desired product as a salt. Acidification procedure: The product as a salt was dissolved in DCM (5 mL), aqueous HCl (6 M, 8 mL) was added, and the biphasic mixture was stirred vigorously for 10 minutes. The organic layer was separated and the aqueous phase was extracted several times with DCM until no product was detectable by TLC in the last DCM extract. The organic layers were combined and the solvent was removed under reduced pressure. The resulting solid was dried under high vacuum for 16 h at room temperature, affording PADI **9** acid as slightly yellow solid (78 mg, 55% yield).

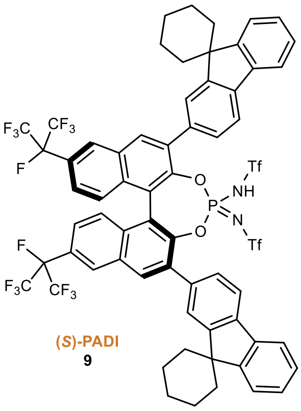

(*S*)-PADI
9

^{1}H NMR (501 MHz, $CDCl_3$): δ (ppm) = 8.35 (s, 2H), 8.28 (s, 2H), 7.93 (s, 2H), 7.79 (d, *J* = 7.9 Hz, 2H), 7.75 (d, *J* = 7.4 Hz, 2H), 7.72 (d, *J* = 9.3 Hz, 4H), 7.57 (d, *J* = 9.3 Hz, 2H), 7.54 (d, *J* = 9.1 Hz, 2H), 7.35 (app t, *J* = 7.3 Hz, 2H), 7.30 (app t, *J* = 7.4 Hz, 2H), 1.95–1.66 (m, 20H).

^{13}C NMR (126 MHz, $CDCl_3$): δ (ppm) = 153.84, 153.80, 145.7, 145.6, 139.7, 139.3, 136.24, 136.21, 134.3, 133.0, 132.5, 131.4, 129.4, 128.1, 127.5, 127.3, 127.1, 125.9, 125.3, 125.1, 125.0, 123.0, 122.5, 120.2, 119.6, 50.8, 35.8, 35.6, 26.6, 23.0, 22.8 (other signals not detected or observed).

^{19}F NMR (471 MHz, $CDCl_3$): δ (ppm) = −75.09 (app p, *J* = 8.2 Hz, 6F), −75.32 (app p, *J* = 8.5 Hz, 6F), −79.22 (s, 6F), −181.88 (hept, *J* = 8.2 Hz, 2F).

^{31}P NMR (203 MHz, $CDCl_3$): δ (ppm) = −4.12.

HRMS (ESI$^-$): *m/z* calcd. for $C_{64}H_{43}O_6N_3F_{20}PS_2$ $[M-H]^-$: 1409.1908, found 1409.1909.

1.5 Catalytic Asymmetric Polyene Cyclization towards Ambrox

1.5.1 Reaction Development

General procedure for the optimization of the catalytic asymmetric polyene cyclizations: The respective Brønsted acid catalyst and a PTFE-coated magnetic stir bar were transferred to a 1.5 mL headspace screw-cap glass vial. The respective solvent was added, and the resultant solution was stirred at the indicated temperature for 10 min. Neat (3*E*,7*E*)-homofarnesol **1a** was slowly added via a Hamilton® syringe and the reaction mixture was stirred at the indicated temperature. The reaction was monitored by thin-layer chromatography (TLC, hexanes/ethyl acetate 5:1 *v/v* as eluent, visualization with phosphomolybdic acid stain, PMA).

Table S2 | Screening of solvents in the catalytic asymmetric polyene cyclization of (3*E*,7*E*)-homofarnesol (**1a**) to ambrox (**2a**).

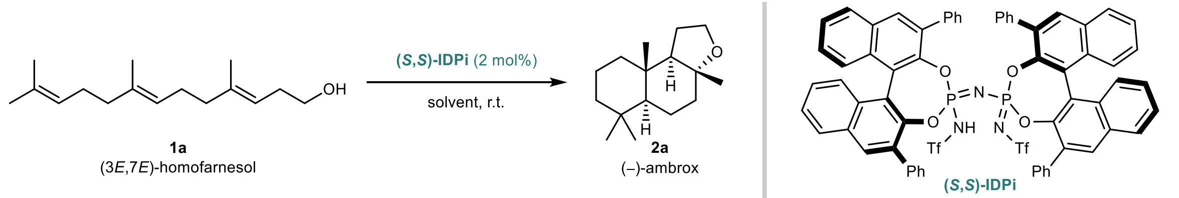

Entry	Solvent	Time	Yield	e.r. of ambrox
1	CH_2Cl_2	7 d	13%	55:45
2	Cyclohexane	7 d	trace	n.d.
3	MTBE	7 d	trace	n.d.
4	THF	7 d	trace	48:52
5	1,4-Dioxane	7 d	trace	55:45
6	Ethyl acetate	7 d	11%	51:49
7	CH_3CN	7 d	trace	52:48
8	Toluene	7 d	trace	45:55
9	HFIP	24 h	42%	51:49
10	TFE	7 d	trace	n.d.
11	PFTB	2 d	trace	54:48

Reactions were performed with (3*E*,7*E*)-homofarnesol **1a** (0.05 mmol) and IDPi catalyst (2 mol%) in solvent (0.5 mL, 0.1 M), yield was determined by ^1H NMR using dibromomethane as internal standard, the enantiomeric ratio (e.r.) was determined by GC on a chiral stationary phase. MTBE: methyl *tert*-butyl ether; THF: tetrahydrofuran; HFIP: 1,1,1,3,3,3-hexafluoro-propan-2-ol; TFE: 2,2,2-trifluoroethanol; PFTB: perfluoro-*tert*-butanol.

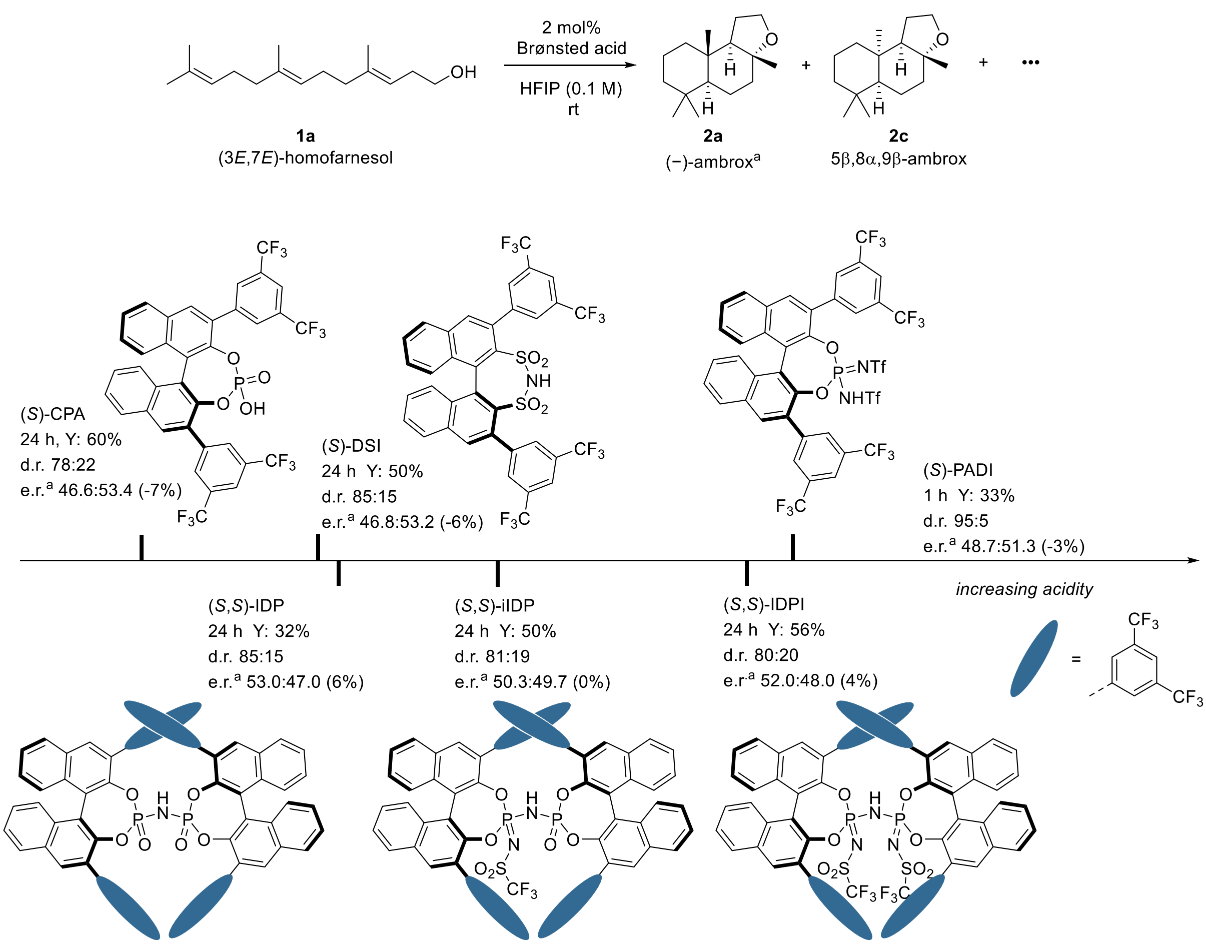

Fig. S9 | Initial screening with commonly used Brønsted acid catalysts. Reactions were performed with (3*E*,7*E*)-homofarnesol (0.05 mmol) and the respective Brønsted acid catalyst (2 mol%) in HFIP (0.5 mL, 0.1 M); yield and diastereomeric ratio (d.r.) was determined by ^{1}H NMR spectroscopy using dibromomethane as internal standard, enantiomeric ratio (e.r.) of ambrox was determined by GC on a chiral stationary phase. HFIP: 1,1,1,3,3,3-hexafluoropropan-2-ol.

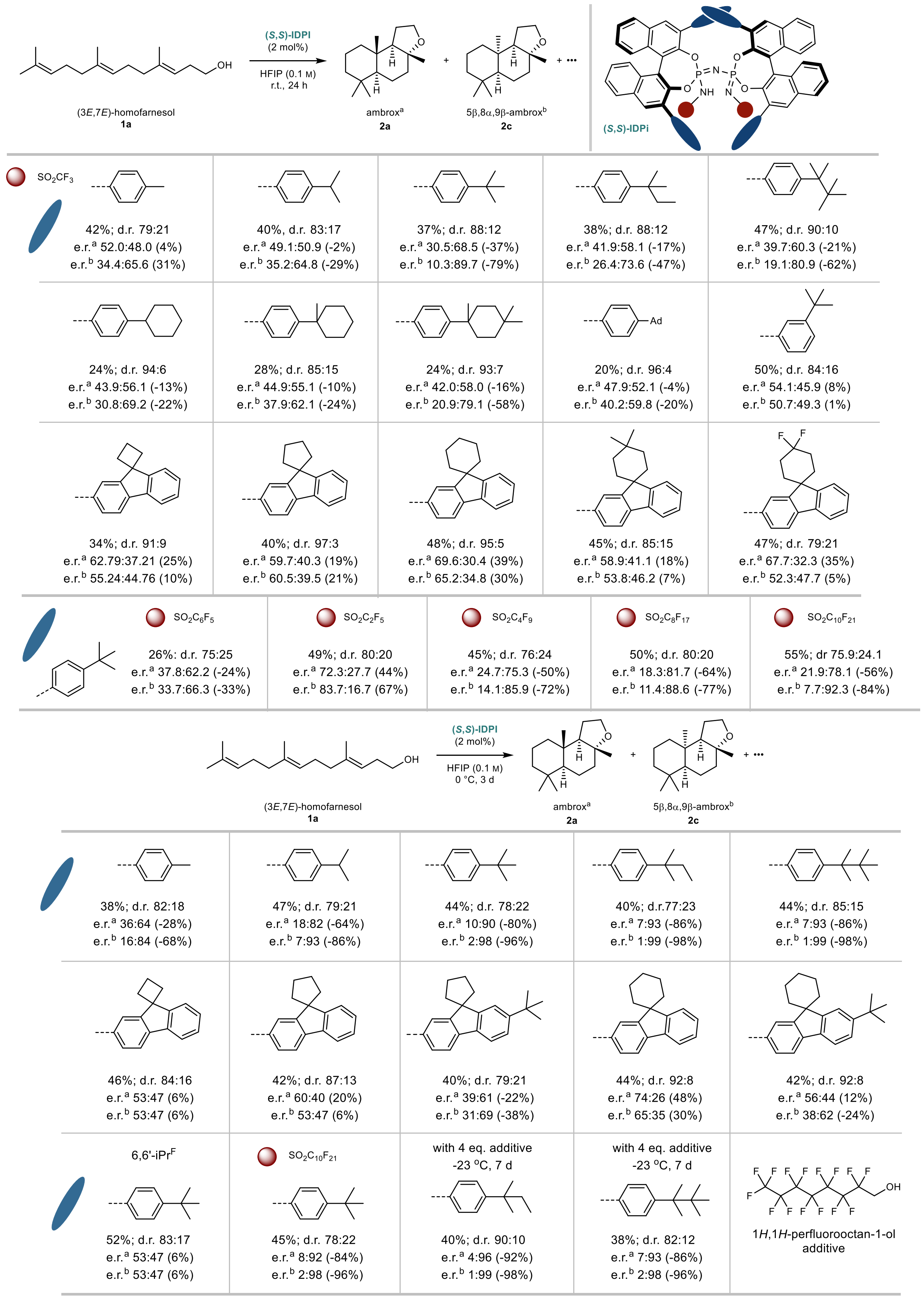

Fig. S10 | Screening of different IDPi catalysts using HFIP as solvent. Reactions were performed with (3*E*,7*E*)-homofarnesol (0.05 mmol) and IDPi catalysts (2 mol%) in HFIP (0.5 mL, 0.1 M), yield and diastereomeric ratio (d.r.) were determined by ^{1}H NMR spectroscopy using dibromomethane as internal standard, enantiomeric ratios (e.r.) were determined by GC on a chiral stationary phase. HFIP: 1,1,1,3,3,3-hexafluoro-2-propanol.

Characterization of the minor diastereomer

To identify and assign the major diastereomer formed in the polyene cyclization of (3*E*,7*E*)-homofarnesol (**1a**) catalyzed by IDPi catalysts in HFIP, a reaction providing a d.r. of approximately 75:25 in favor of ambrox (**2a**) was performed on a larger scale. The tricyclic ethers were isolated and the respective isomers were identified by comparison of the acquired ^{13}C NMR spectrum with data reported in the literature (vide infra, Fig. S11).[20]

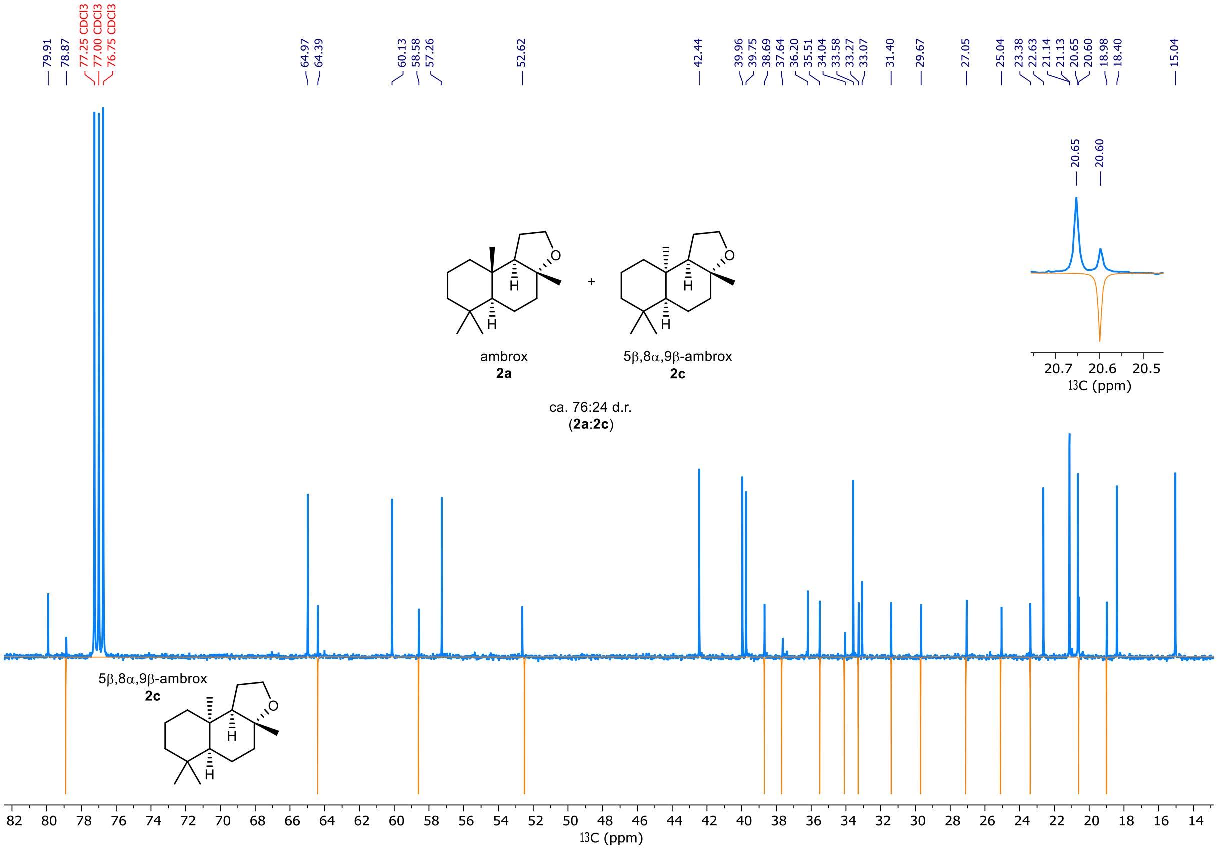

Fig. S11 | Comparison of the ^{13}C NMR (126 MHz, $CDCl_3$) spectrum of the tricyclic ether fraction (blue, top) obtained in HFIP with a spectrum of 5β,8α,9β-ambrox generated from reported literature data (orange, bottom).[20] The ratio of ambrox (**2a**) to 5β,8α,9β-ambrox (**2c**) was approximately 76:24 judged by the corresponding ^{1}H NMR spectrum.

The identity of the minor diastereomer was thus unequivocally established as 5β,8α,9β-ambrox (**2c**).

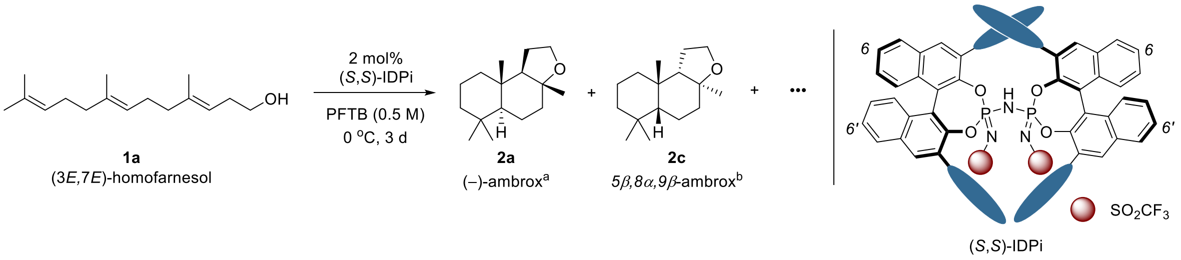

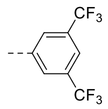 54%; d.r. 88:12<br>e.r.[a] 64:36 (28%)<br>e.r.[b] 48:52 (-4%)	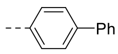 30%; d.r. 79:21<br>e.r.[a] 66:32 (32%)<br>e.r.[b] 35:65 (-30%)	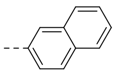 36%; d.r. 94:6<br>e.r.[a] 64:36 (28%)<br>e.r.[b] 43:57 (-14%)	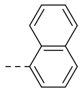 38%; d.r. 96:4<br>e.r.[a] 66:34 (32%)<br>e.r.[b] 47:53 (-6%)	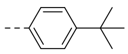 trace; d.r. >20:1<br>e.r.[a] 34:66 (-32%)
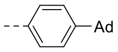 30%; d.r. 89:11<br>e.r.[a] 47:53 (-6%)<br>e.r.[b] 43:57 (-14%)	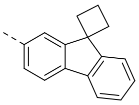 30%; d.r. 91:9<br>e.r.[a] 73:27 (46%)<br>e.r.[b] 46:54 (-8%)	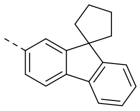 trace; d.r. 94:6<br>e.r.[a] 78:22 (56%)<br>e.r.[b] 53:47 (6%)	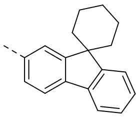 trace; d.r. >20:1<br>e.r.[a] 82:18 (64%)	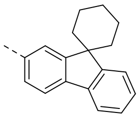 rt, 1 d<br>trace; d.r. >20:1<br>e.r.[a] 73:27 (46%)
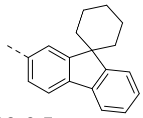 $SO_2C_6F_5$<br>rt, 1 d<br>14%; d.r. >20:1<br>e.r.[a] 66:34 (32%)	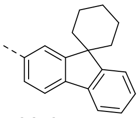 $SO_2C_4F_9$<br>rt, 1 d<br>34%; d.r. >20:1<br>e.r.[a] 70:30 (40%)	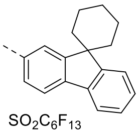 $SO_2C_6F_{13}$<br>rt, 1 d<br>31%; d.r. >20:1<br>e.r.[a] 74:26 (48%)	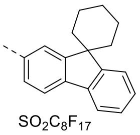 $SO_2C_8F_{17}$<br>rt, 1 d<br>39%; d.r. 85:15<br>e.r.[a] 66:34 (32%)<br>e.r.[b] 67:33 (34%)	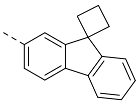 SO_2CF_3 6,6'-*i*-Pr^{F7}<br>rt, 0.5 d<br>56%; d.r. 85:15<br>e.r.[a] 77:23 (54%)<br>e.r.[b] 42:58 (-16%)
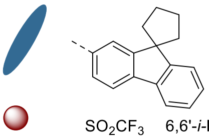 SO_2CF_3 6,6'-*i*-Pr^{F7}<br>rt, 0.5 d<br>61%; d.r. 90:10<br>e.r.[a] 83:17 (66%)<br>e.r.[b] 52:48 (4%)	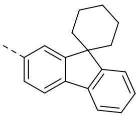 SO_2CF_3 6,6'-*i*-Pr^{F7}<br>rt, 0.5 d<br>68%; d.r. 94:6<br>e.r.[a] 86:14 (72%)<br>e.r.[b] 60:40 (20%)	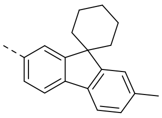 SO_2CF_3 6,6'-*i*-Pr^{F7}<br>rt, 0.5 d<br>65%; d.r. 94:6<br>e.r.[a] 86:14 (72%)<br>e.r.[b] 70:30 (40%)	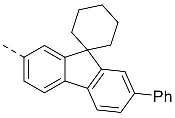 SO_2CF_3 6,6'-*i*-Pr^{F7}<br>rt, 0.5 d<br>44%; d.r. 82:18<br>e.r.[a] 74:26 (58%)<br>e.r.[b] 52:48 (4%)	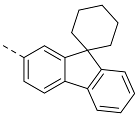 $SO_2C_4F_9$ 6,6'-*i*-Pr^{F7}<br>rt, 0.5 d<br>58%; d.r. 94:6<br>e.r.[a] 85:15 (70%)<br>e.r.[b] 71:29 (42%)

Fig. S12 | Screening of the different IDPi catalysts using PFTB as solvent. Reactions were performed with (3*E*,7*E*)-homofarnesol (0.025 mmol) and IDPi catalysts (2 mol%) in PFTB (0.02 mL, 1.25 M), yield and diastereomeric ratio (d.r.) were determined by ^{1}H NMR spectroscopy using dibromomethane as internal standard, enantiomeric ratios (e.r.) were determined by GC on a chiral stationary phase. PFTB: perfluoro-*tert*-butanol.

Table S3 | Screening of the temperature and catalyst loading in PFTB.

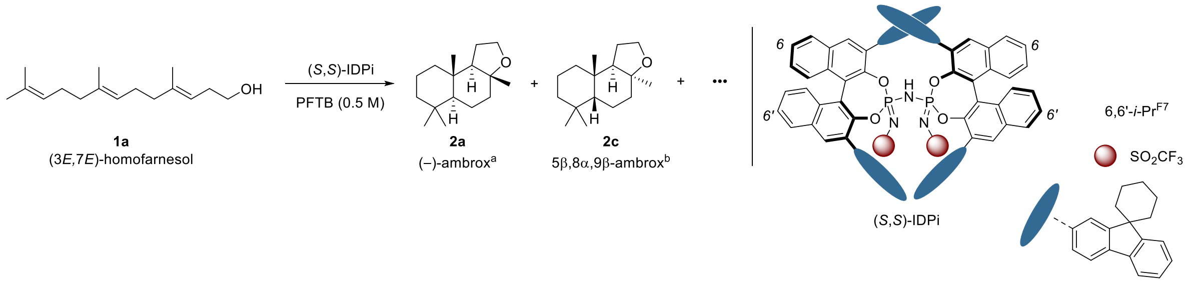

Entry	(*S*,*S*)-IDPi	*T*	*t*	yield of ambrox **2a**	d.r. **2a:2c**	e.r. of ambrox **2a**	e.r. of 5β,8α,9β-ambrox **2c**
1	2 mol%	Rt	12 h	68%	94:6	86:14 (72%)	60:40 (20%)
2	2 mol%	0 °C	12 h	65%	91:9	90:10 (80%)	68:32 (36%)
3	2 mol%	−16 °C	12 h	51%	93:7	93:7 (86%)	76:24 (52%)
4	2 mol%	−20 °C	12 h	50%	>20:1	93:7 (86%)	78:22 (56%)
5	2 mol%	−30 °C	12 h	47%	>20:1	95:5 (90%)	78:22 (56%)
6	2 mol%	−40 °C	12 h	43%	>20:1	95:5 (90%)	80:20 (60%)
7	2 mol%	−50 °C	12 h	38%	>20:1	96:4 (92%)	–
8	1 mol%	−30 °C	12 h	47%	>20:1	94:6 (88%)	83:17 (66%)
9	5 mol%	−30 °C	12 h	49%	>20:1	95:5 (90%)	81:19 (62%)
10	2 mol%	−40 °C to 0 °C	12 h *then* 8 h	54%	>20:1	95:5 (90%)	79:21 (58%)

[a] Reactions were performed with (*3E,7E*)-homofarnesol (0.025 mmol) and IDPi catalysts in PFTB (0.02 mL, 1.25 M), yield and diastereomeric ratio (d.r.) were determined by ^{1}H NMR spectroscopy using dibromomethane as internal standard, enantiomeric ratios (e.r.) were determined by GC on a chiral stationary phase. PFTB: perfluoro-*tert*-butanol.

1.5.2 Scale-up Experiments

Scale-up Experiment at −40 °C (5 mmol scale)

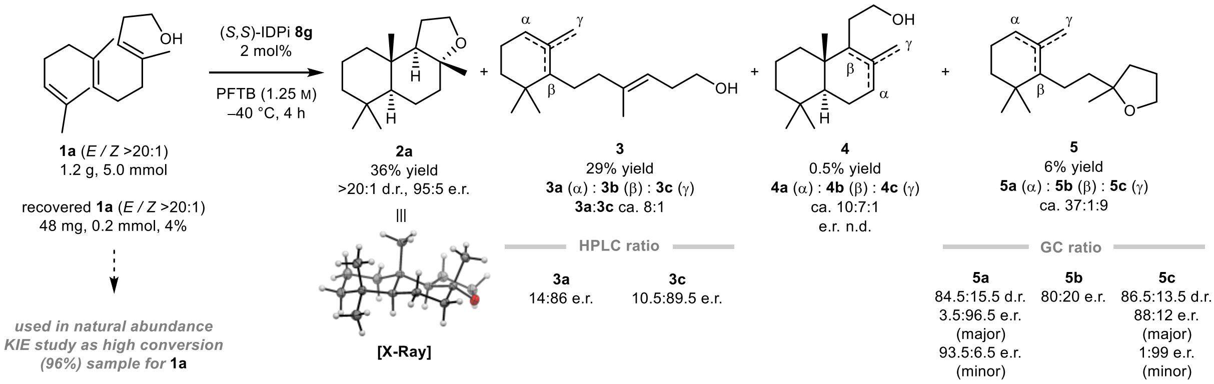

A 100 mL Schlenk flask under argon equipped with a PTFE-coated magnetic stir bar was charged with (3*E*,7*E*)-homofarnesol **1a** (1.18 g, 5.00 mmol, 1.0 equiv.) and IDPi catalyst **8g** (254 mg, 0.10 mmol, 2 mol%). The mixture was cooled to −40 °C and stirred for 10 min. Perfluoro-*tert*-butanol (PFTB, 4.0 mL, 1.25 M) was added and the reaction was stirred for 4 h at −40 °C. After the elapsed time, the reaction mixture was neutralized with triethylamine, and the solvent was evaporated under reduced pressure. The residue was subjected to column chromatography on silica gel (gradient elution with hexanes/ethyl acetate 50:1 → 1:4 *v/v*) to give a nonpolar fraction containing partially cyclized products **5** (70 mg, 296 µmol, 6% yield, ratio **5a**:**5b**:**5c** ca. 40:1:9) alongside isomerized product **10**, a tricyclic ether fraction containing (−)-ambrox **2a** (426 mg, 1.80 mmol, 36% yield, >20:1 d.r., 95:5 e.r.), and a polar fraction containing a mixture of residual starting material **1a**, cyclohomofarnesols **3** and traces of homodrimenols **4** (400 mg, 1.69 mmol, 34% yield). The mixture of residual **1a** and **3** was further separated by preparative HPLC (250 mm Multokrom 100-3-Si, 20 mm i.d., 3 µm, *i*-hexane/propan-2-ol 99.5:0.5 *v/v*, 14.8 mL/min, 12.7 MPa, 298 K, 205 nm, UV), to furnish pure **1a** (48.0 mg, 203 µmol, 4% yield, >20:1 *E*/*Z*), **3** (352 mg, 1.49 mmol, 30% yield, ratio **3a**:**3c** ca. 8:1; traces of **3b** were also detected), and traces of homodrimenols **4** (5.40 mg, 22.8 µmol, 0.5% yield, ratio **4a**:**4b**:**4c** ca. 10:7:1). Recovered and purified **1a** was used as high conversion sample (96%) in the natural abundance KIE study (see section 2.1). For catalyst recovery, the column was flushed with hexanes/ethyl acetate (1:4 *v/v*) to afford the IDPi catalyst **8g** as a salt. IDPi **8g** was obtained after acidification in DCM with aq. HCl (6 M) and evaporation of the solvent followed by drying under high vacuum as a colorless solid (253 mg, 0.10 mmol, IDPi recovery >99%). The purity of the recovered catalyst was verified by ^{1}H and ^{31}P NMR spectroscopy.

Characterization data for (−)-ambrox (2a) synthesized at −40 °C:

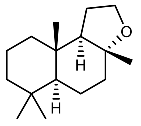

2a
36% yield
>20:1 d.r., 95:5 e.r.

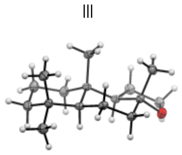

Physical state: colorless solid.

TLC (hexanes/MTBE 9:1 *v/v*): R_f = 0.28 (PMA stain).

M.p.: 67–71 °C (>20:1 d.r. and 95:5 e.r.; >95% GC purity).

^{1}H NMR (501 MHz, $CDCl_3$): δ (ppm) = 3.95–3.87 (m, 1H), 3.82 (q, *J* = 8.1 Hz, 1H), 1.94 (dt, *J* = 11.5, 3.2 Hz, 1H), 1.78–1.61 (m, 4H), 1.51–1.45 (m, 1H), 1.45–1.36 (m, 4H), 1.29 (tdd, *J* = 14.1, 12.6, 3.2 Hz, 1H), 1.18 (td, *J* = 13.5, 4.2 Hz, 1H), 1.08 (s, 3H), 1.03 (td, *J* = 12.7, 3.5 Hz, 1H), 0.96 (dd, *J* = 12.3, 2.7 Hz, 1H), 0.87 (s, 3H), 0.83 (s, 3H), 0.83 (s, 3H).

^{13}C{^{1}H} NMR (126 MHz, $CDCl_3$): δ (ppm) = 80.1, 65.1, 64.2, 60.3, 57.4, 42.6, 40.1, 39.9, 36.4, 33.7, 33.2, 22.8, 21.3, 20.8, 18.6, 15.2.

HRMS (GC-EI$^+$): *m/z* calcd. for $C_{16}H_{28}O$ $[M]^+$: 236.213465, found: 236.213290.

$[\alpha]_D^{25}$ = −21.6 ($CHCl_3$, *c* = 1.0; >20:1 d.r., 95:5 e.r.). Lit.: $[\alpha]_D^{20}$ = −24.7 ($CHCl_3$, *c* = 1.0).[24]

GC (achiral) (Optima-35 0.25/0.25df G/706, 29.0 m; temperature: 220/50 5/min 200 12/min 350, 5 min iso/ 350, 0.60 bar H_2, sample size: 0.2 µL, split ratio: 120:1): 9-*epi*-ambrox: t_R(**2b**) = 26.71 min (2.29%), 5β-ambrox: t_R(**2e**) = 26.96 min (0.62%), 5β,8α-ambrox: t_R(**2d**) = 27.03 min (0.02%), ambrox: t_R(**2a**) = 27.54 min (95.42%), 5β,8α,9β-ambrox: t_R(**2c**) = 27.97 min (1.65%).

Diastereomeric ratio: **2a** : **2b** : **2c** : **2d** : **2e** = 95.57 : 2.26 : 1.58 : 0.10 : 0.49; >20:1 d.r. (**2a**).

GC (chiral) (BGB 176/BGB-15 0.25/0.25df G/618, 30.0 m; temperature: 220/140, 60 min iso 8/min 240/ 350, 0.60 bar H_2, sample size: 1.0 µL): (−)-9-*epi*-ambrox: t_R(**2b**) = 41.88 min (2.06%), 5β-ambrox: t_R(**2e**) = 42.38 min (0.10%), (+)-9-*epi*-ambrox, t_R(**2b**) = 42.92 min (0.20%), 5β,8α-ambrox: t_R(**2d**) = 43.64 min (0.49%), (−)-ambrox: t_R(**2a**) = 49.38 min (90.61%), (+)-ambrox: t_R(**2a**) = 51.17 min (4.96%), 5β,8α,9β-ambrox: t_R(**2c**) = 52.95 min (1.25%), *ent*-5β,8α,9β-ambrox: t_R(**2c**) = 54.70 min (0.33%).

Enantiomeric ratios of **2a**, **2b** and **2c**: (−)-ambrox (**2a**): e.r. = 95:5; (−)-9-*epi*-ambrox (**2b**): e.r. = 91:9; 5β,8α,9β-ambrox: (**2c**): e.r. = 79:21.

X-Ray Crystallography: CCDC-2338446.

Crystals suitable for single crystal X-ray diffraction studies were grown from a concentrated solution in cyclopentane in a fridge overnight.

NMR assignment for (−)-ambrox (**2a**):

The spectroscopic data of synthetic (−)-ambrox (**2a**) obtained in the IDPi-catalyzed asymmetric polyene cyclization of (3*E*,7*E*)-homofarnesol (**1a**) are in good agreement with the data and assignments reported in the literature.[24,31] Assignments of the individual positions according to 2D-NMR are provided in the table below.

Table S4 | Tabulated NMR assignments (^1H NMR: 600 MHz, ^{13}C NMR: 151 MHz, $CDCl_3$) and 2D NMR correlations for (−)-ambrox (**2a**).

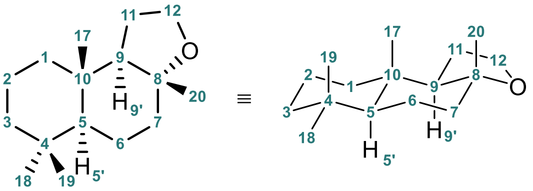

Atom	J	δ (ppm)	HSQC	COSY	HMBC	NOESY
1 C		40.118	1ax, 1eq		2ax, 3ax, 17	
Hax (α)	13.0(2ax), 3.7(2eq), 13.0(1eq)	1.031	1	1eq, 2ax, 2eq, 17	2, 3, 9, 10, 17	3ax
Heq (β)	13.0(1ax)	1.468	1	1ax, 2ax, 2eq	2, 3, 5, 10	2ax, 17
2 C		18.564	2ax, 2eq		1ax, 1eq, 3ax	
Hax(β)	13.0(1ax)	1.663	2	1ax, 1eq, 3ax	1, 3, 4, 10	1eq, 17, 19
Heq(α)	3.7(1ax)	1.418	2	1ax, 1eq, 3ax		
3 C		42.600	3ax, 3eq		1ax, 1eq, 2ax, 5', 18, 19	
Hax (α)		1.178	3	2ax, 2eq, 3eq, 19	1, 2, 4, 18, 19	1ax, 5', 18
Heq (β)		1.404	3	3ax		18, 19
4 C		33.231			2ax, 3ax, 5', 6eq, 18, 19	
5 C		57.420	5'		1eq, 6ax, 6eq, 7eq, 17, 18, 19	
5' H (α)	12.4(6ax), 2.7(6eq)	0.957	5	6ax, 6eq	3, 4, 6, 7, 9, 10, 17, 19	3ax, 7ax, 18
6 C		20.814	6ax, 6eq		5', 7eq	
Hax (β)	12.4(5')	1.294	6	5', 6eq, 7eq	5, 7, 10	6eq, 7eq, 17, 19, 20
Heq (α)	2.7(5')	1.746	6	5', 6ax, 7eq	4, 5, 7, 8, 10	6ax, 7eq, 18
7 C		39.913	7ax, 7eq		5', 6ax, 6eq, 20	
Hax (α)	11.6(7eq)	1.401	7	7eq		5'
Heq (β)	11.6(7ax), 3.2(n.a.), 3.2(n.a.)	1.938	7	6ax, 6eq, 7ax	5, 6, 8, 9, 20	6ax, 6eq, 20

Atom	J	δ (ppm)	HSQC	COSY	HMBC	NOESY
8 C		80.063			6eq, 7eq, 11, 12', 12'', 20	
9 C		60.285	9'		1ax, 5', 7eq, 11, 12', 12'', 17, 20	
9' H		1.389	9	11, 12'', 17		
10 C		36.353			1ax, 1eq, 2ax, 5', 6ax, 6eq, 17	
11 C		22.796	11		12', 12''	
H2		1.722	11	9', 12', 12''	8, 9, 12	17, 20
12 C		65.138	12', 12''		11	
H'		3.820	12	11, 12''	8, 9, 11	
H''		3.911	12	9', 11, 12'	8, 9, 11	20
17 C		15.200	17		1ax, 5'	
H3		0.835	17	1ax, 9'	1, 5, 9, 10	1eq, 2ax, 6ax, 11, 20
18 C		33.743	18		3ax, 19	
H3		0.873	18	19	3, 4, 5, 19	3ax, 3eq, 5', 6eq, 19
19 C		21.292	19		3ax, 5', 18	
H3		0.828	19	3ax, 18	3, 4, 5, 18	2ax, 3eq, 6ax, 18
20 C		21.303	20		7eq	
H3		1.082	20		7, 8, 9	6ax, 7eq, 11, 12'', 17

Purification of the fraction containing polar components by preparative HPLC

Additional purification of the most polar fraction obtained after flash column chromatography was accomplished by preparative HPLC (see Fig. S13). The respective fractions containing the separated components were pooled, concentrated under reduced pressure, and further purified by flash column chromatography on silica gel (gradient elution with hexanes/MTBE 4:1 → 1:1 *v/v*) to remove minor impurities (high boiling alkanes).

HPLC (achiral): 250 mm Multokrom 100-Si, 20 mm i.D., 3µm; *i*-hexan/propan-2-ol 99.5:0.5 *v/v*, 14.8 mL/min, UV: λ = 205 nm, 298 K

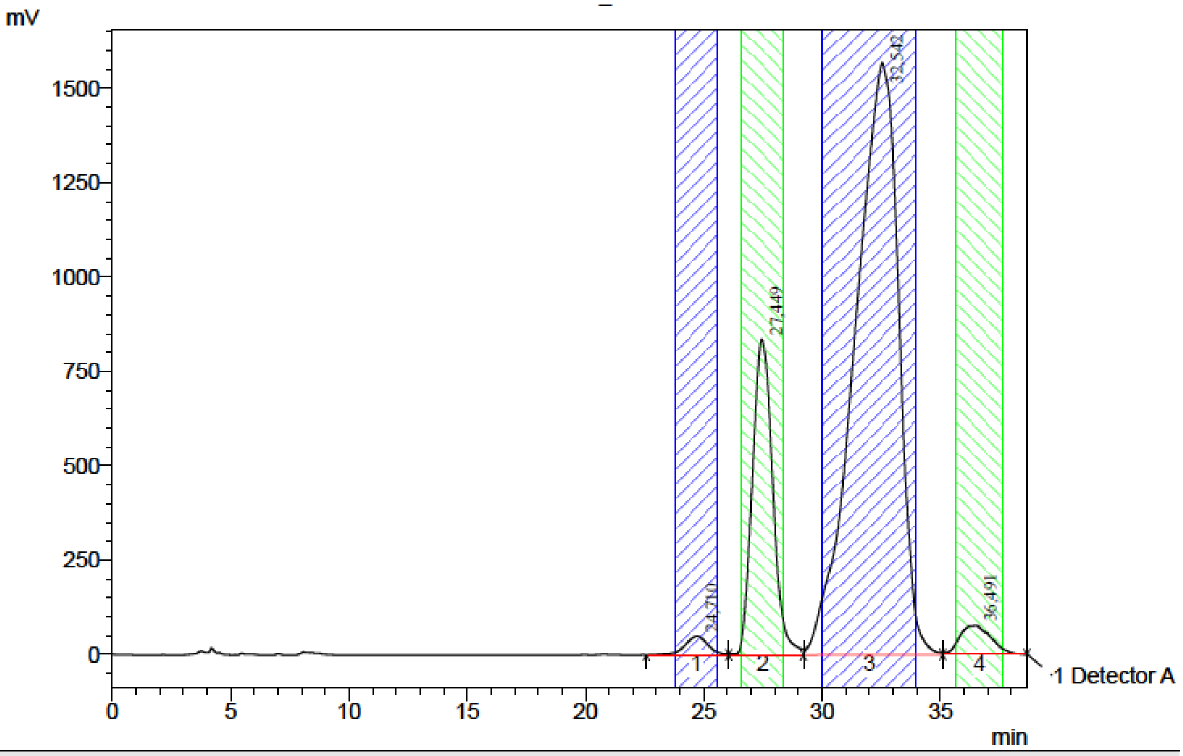

peak #	t_R / min	area / %	peak name	Structure
1	24.71	1.26	not identified	n.d.
2	27.45	19.14	(3*E*,7*E*)-homofarnesol (**1a**)	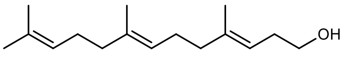 **1a** (*E* / *Z* >20:1)
3	32.54	76.87	(*E*)-α- and (*E*)-γ-cyclohomofarnesol (**3a/3c**)	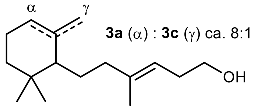 **3a** (α) : **3c** (γ) ca. 8:1
4	36.49	2.73	homodrimenols **4**	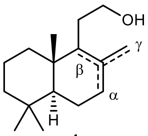 **4**

Fig. S13 | HPLC chromatogram of the polar fraction containing a mixture of (3*E*,7*E*)-homofarnesol (**1a**), (*E*)-α-cyclohomofarnesol (**3a**) and (*E*)-γ-cyclohomofarnesol (**3c**) and traces of homodrimenols (**4**).

Analysis of recovered starting material (1a)

The remaining starting material (3*E*,7*E*)-homofarnesol (**1a**) was recovered in the second fraction after preparative HPLC in 4% isolated yield. Comparison of the ^{1}H NMR spectrum of **1a** recovered from the reaction mixture with spectra of individually synthesized homofarnesol double bond isomers confirmed that no double bond isomerization at either double bond position occurred in course of the IDPi-catalyzed polyene cyclization (see Fig. S14). This observation is in line with a selective protonation at the distal position, where an *E*/*Z*-isomerization is not possible.

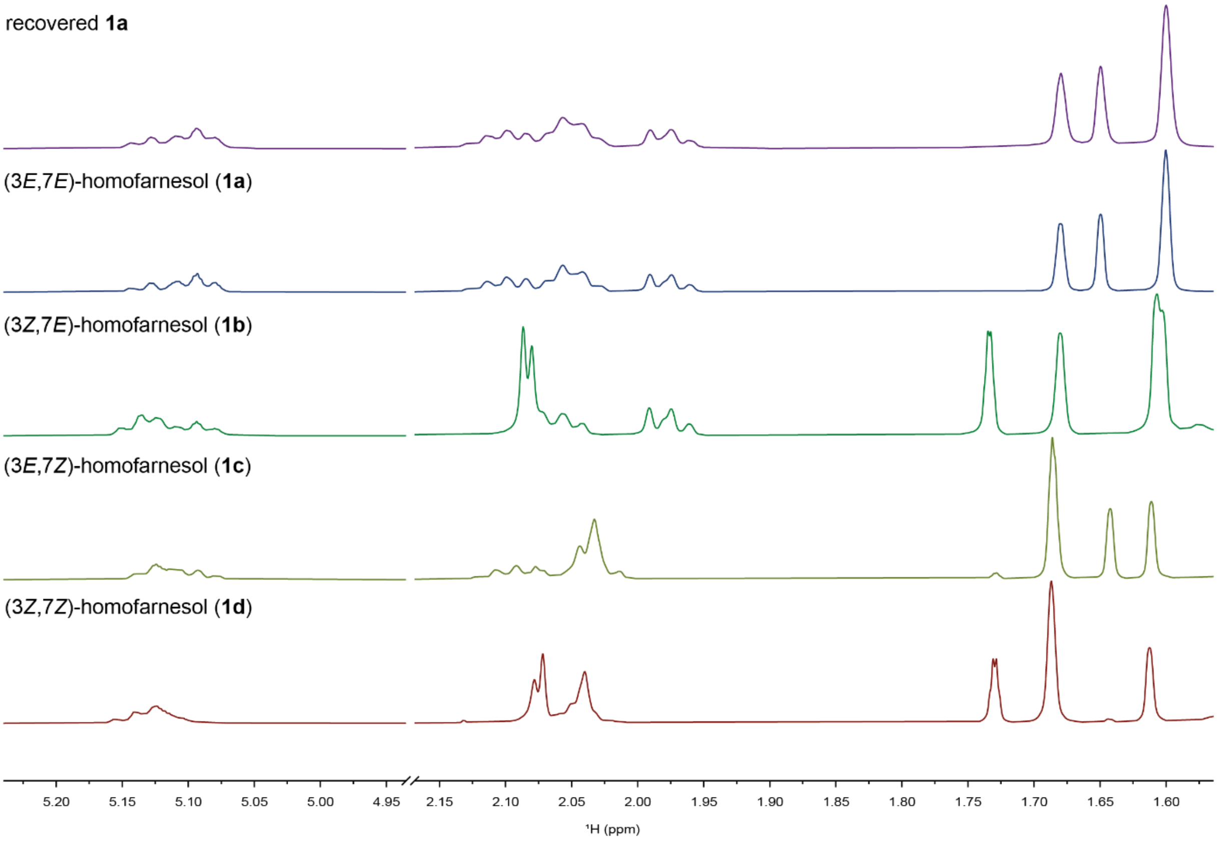

Fig. S14 | Excerpt of stacked ^{1}H NMR spectra of recovered **1a** and individually synthesized double bond isomers of homofarnesol showing regions of interest with recognizable differences between the isomers.

The recovered starting material also served as high conversion sample in the subsequent natural abundance KIE study (see section 2.1).

Characterization data for cyclohomofarnesols (3)

Fraction 3 obtained after preparative HPLC mainly consisted of (*S*)-(*E*)-α-cyclohomofarnesol (**3a**, major regioisomer) and (*S*)-(*E*)-γ-cyclohomofarnesol (**3c**, minor regioisomer). Traces of the β-isomer (**3b**) were also observed in the ^{1}H NMR spectrum.

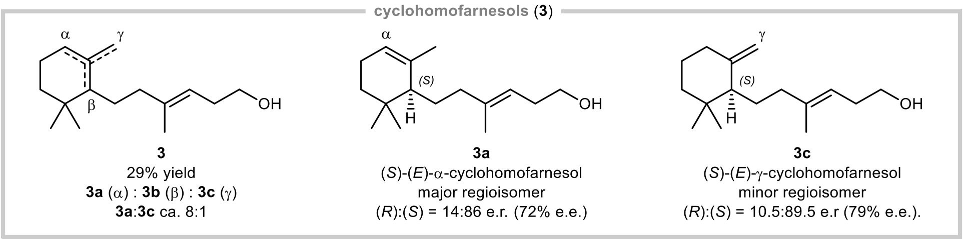

The identity of the individual cyclohomofarnesol isomers was confirmed by comparison of the NMR spectroscopic data, as well as GC and HPLC retention times with the data of previously synthesized pure standards.

Characterization data for α-cyclohomofarnesol (**3a**):

^{1}H NMR (600 MHz, $CDCl_3$): δ (ppm) = 5.31–5.25 (m, 1H), 5.13 (tq, *J* = 7.4, 1.4 Hz, 1H), 3.62 (t, *J* = 6.5 Hz, 2H), 2.32–2.25 (m, 2H), 2.08–2.02 (m, 2H), 1.97–1.93 (m, 2H), 1.67 (q, *J* = 1.7 Hz, 3H), 1.66–1.64 (m, 3H), 1.55–1.49 (m, 1H), 1.46–1.34 (m, 3H), 1.12 (ddd, *J* = 13.3, 6.0, 3.4 Hz, 1H), 0.92 (s, 3H), 0.86 (s, 3H).

^{13}C NMR (151 MHz, $CDCl_3$): δ (ppm) = 139.8, 136.9, 120.1, 119.7, 62.7, 49.2, 40.8, 32.7, 31.7, 31.7, 30.0, 27.7, 27.6, 23.6, 23.2, 16.5.

GC (chiral) (BGB-176/BGB-15, 0.25/0.25df G/618, 30.0 m; temperature: 220/110 470 min iso 8/min 240/ 350, 0.60 bar H_2, sample size: 1.0 µL, split ratio: 10:1): (*S*)-(*E*)-α-cyclohomofarnesol: t_R(**3a**) = 391.95 min (87.27%), (*R*)-(*E*)-α-cyclohomofarnesol: t_R(*ent*-**3a**) = 420.62 min (12.73%); (*S*):(*R*) = 87.27:12.73 e.r. (74.5% e.e.).

Characterization data for γ-cyclohomofarnesol (**3c**):

^{1}H NMR (600 MHz, $CDCl_3$): δ (ppm) = 5.12 (th, *J* = 7.3, 1.3 Hz, 1H), 4.76 (ddd, *J* = 2.5, 1.4, 0.8 Hz, 1H), 4.53 (dt, *J* = 2.5, 0.8 Hz, 1H), 3.62 (t, *J* = 6.5 Hz, 2H), 2.31–2.26 (m, 2H), 2.10–2.03 (m, 1H), 2.02–1.94 (m, 2H), 1.78 (ddd, *J* = 14.0, 10.4, 5.8 Hz, 1H), 1.68 (dd, *J* = 11.4, 3.3 Hz, 1H), 1.64–1.63 (m, 3H), 1.60–1.39 (m, 5H), 1.36 (s, 1H), 1.21 (dt, *J* = 13.3, 4.4 Hz, 1H), 0.91 (s, 3H), 0.83 (s, 3H).

^{13}C NMR (151 MHz, $CDCl_3$): δ (ppm) = 149.5, 139.7, 119.6, 109.0, 62.7, 53.9, 38.5, 36.5, 35.0, 32.6, 31.7, 28.6, 26.4, 24.9, 23.9, 16.5.

Chromatographic data for a mixture of **3a** (major), **3c** (minor), **3b** (traces) and residual **1a**:

GC (achiral) (DB-Waxetr 0.25/0.25df G/770, 30.0 m; temperature: 220/50 5/min 260 12/min 280, 3 min iso/ 350, 0.60 bar H_2, sample size: 1.0 µL, split ratio: 120:1): (*E*)-γ-cyclohomofarnesol: t_R(**3c**) = 32.66 min (14.20%), (3*E*,7*E*)-homofarnesol: t_R(**1a**) = 32.99 min (12.10%), (*E*)-α-cyclohomofarnesol: t_R(**3a**) = 33.25 min (53.55%), (*E*)-β-cyclohomofarnesol: t_R(**3b**) = 33.63 min (0.81%).

HPLC (achiral) (100 mm VDSpher PUR 100 SIL, 4.6 mm i.D., *n*-heptane/propan-2-ol 95.5:0.5 *v/v*, 1.0 mL/min, 20.8 MPa, 288 K, UV: λ = 205 nm): (*E*)-γ-cyclohomofarnesol: t_R(**3c**) = 11.64 min, (*E*)-α-cyclohomofarnesol: t_R(**3a**) = 12.01 min.

Analytical 2D-HPLC method for 3a: ^{1}D (1st dimension, achiral stationary phase, 100 mm VDSpher PUR 100 SIL, 4.6 mm i.D., *n*-heptane/propan-2-ol 95.5:0.5 *v/v*, 1.0 mL/min, 20.8 MPa, 288 K, UV: λ = 205 nm): t_R = 11.99–12.03 (sampling range). **^{2}D** (2nd dimension, chiral stationary phase, 150 mm Chiralpak IG-3, 4.6 mm i.D., *n*-heptane/propan-2-ol = 99.5:0.5 *v/v*, 1.0 mL/min, 9.7 MPa, 288 K, UV: λ = 220 nm): (*R*)-(*E*)-α-cyclohomofarnesol t_R(*ent*-**3a**) = 10.91 min (13.75%), (*S*)-(*E*)-α-cyclohomofarnesol: t_R(**3a**) = 13.48 min (86.25%); (*S*):(*R*) = 86.25:13.75 e.r. (72.5% e.e.).

Analytical 2D-HPLC method for 3c: ^{1}D (1st dimension, achiral stationary phase, 100 mm VDSpher PUR 100 SIL, 4.6 mm i.D., *n*-heptane/propan-2-ol 95.5:0.5 *v/v*, 1.0 mL/min, 20.8 MPa, 288 K, UV: λ = 205 nm): t_R = 11.58–11.62 (sampling range). **^{2}D** (2nd dimension, chiral stationary phase, 2 × 150 mm Chiralcel OD-3, 4.6 mm i.D., *n*-heptane/propan-2-ol 95.5:0.5 *v/v*, 1.0 mL/min, 14.3 MPa, 288 K, UV: λ = 220 nm): (*R*)-(*E*)-γ-cyclohomofarnesol t_R(*ent*-**3c**) = 29.36 min (10.37%), (*S*)-(*E*)-γ-cyclohomofarnesol: t_R(**3c**) = 31.23 min (89.64%); (*S*):(*R*) = 89.5:10.5 e.r. (79% e.e.).

NMR assignments for α-cyclohomofarnesol (**3a**):

The observed NOEs suggest that **3a** adopts a pseudoaxial conformation (most likely the major contributing conformer at 298 K). The equatorial positioning of H3α was assigned based on the observed ^{3}J values ($^{3}J_{3eq}$ = 5.7, 3.4 Hz). In contrast to this, H3β has larger values ($^{3}J_{3ax}$ = 9.7, 7.2 Hz) based on the 1D TOCSY and HSQC data and is thus located at the axial position. Note that H3β is in the equatorial position of the A ring in (−)-ambrox (**2a**). The same observation holds true for the axial position of the methyl group at position 18 and the equatorial position of the methyl group at position 19.

Table S5 | Tabulated NMR assignments and 2D NMR correlations for γ-cyclohomofarnesol (**3c**).

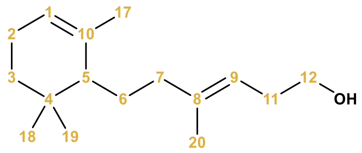

Atom	*J*	δ (ppm)	HSQC	COSY	HMBC	NOESY
1 C		120.117	1		3ax, 3eq, 17	
H		5.282	1	2, 17		2, 17
2 C		23.172	2		3eq	
H2		1.948	2	1, 3ax, 3eq		1, 3ax, 3eq, 18
3 C		31.747	3ax, 3eq		18, 19	
Hax (β)	13.3(3eq),9.7(2ax),7.2(2eq)	1.416	3	2, 3eq	1, 4, 5	2, 3eq, 19
Heq (α)	13.3(3ax),5.7(n.a.),3.4(n.a.)	1.118	3	2, 3ax	1, 2, 4, 5	2, 3ax, 18, 19
4 C		32.733			3ax, 3eq, 6b, 18, 19	
5 C		49.169	5'		3ax, 3eq, 6b, 7, 17, 18, 19	
5' H (α)		1.405	5	17	7, 10	17, 18, 19
6 C		30.014	6a, 6b		7	
Ha		1.382	6	7		17
Hb		1.522	6	7	4, 5, 7, 8, 10	19
7 C		40.809	7		5', 6b, 9', 20	
H2		2.046	7	6a, 6b, 20	5, 6, 8, 9, 20	9', 17, 19, 20
8 C		139.847			6b, 7, 11, 20	

Atom	*J*	δ (ppm)	HSQC	COSY	HMBC	NOESY
9 C		119.684	9'		7, 11, 12, 20	
9' H	7.4(11), 1.4(n.a.)	5.131	9	11, 20	7, 11, 12, 20	7, 11, 12
10 C		136.853			5', 6b, 17	
11 C		31.667	11		9', 12	
H2	6.5(12), 7.4(9')	2.282	11	9', 12	8, 9, 12	9', 20
12 C		62.673	12		9', 11	
H2	6.5(11)	3.623	12	11	9, 11	9'
17 C		23.641	17			
H3	1.7(n.a.)	1.671	17	1, 5'	1, 5, 10	1, 5', 6a, 7
18 C		27.687	18		19	
H3 (α)		0.862	18	19	3, 4, 5, 19	2, 3eq, 5'
19 C		27.604	19		18	
H3 (β)		0.921	19	18	3, 4, 5, 18	3ax, 3eq, 5', 6b, 7
20 C		16.481	20		7, 9'	
H3		1.648	20	7, 9'	7, 8, 9	7, 11

NMR assignments for γ-cyclohomofarnesol (**3c**):

The observed NOEs (see Fig. S15 for an excerpt of the ^{1}H,^{1}H-NOESY spectrum) suggest that **3c** adopts a pseudoaxial conformation (i.e. the pseudoaxial conformer is most likely the major contributing conformer at 298 K). The conformational equilibrium of γ-cyclohomofarnesol **3c** has been previously discussed in the literature by Snowden and coworkers (see *J. Org. Chem.* **1992**, *57*, 955–960; discussion on page 959, reference 29 and 30).[15] The equatorial positioning of H3α was determined based on the observed ^3J values and by analysis of the ^{1}H,^{1}H-COSY spectrum that revealed key long range W-couplings to H1eq and H5', respectively. In contrast to this, H3β has larger values judged by the 2D HSQC data and is thus located at the axial position. Note that H3β is in the equatorial position of the A ring in (−)-ambrox (**2a**).

Table S6 | Tabulated NMR assignments and 2D NMR correlations for γ-cyclohomofarnesol (**3c**).

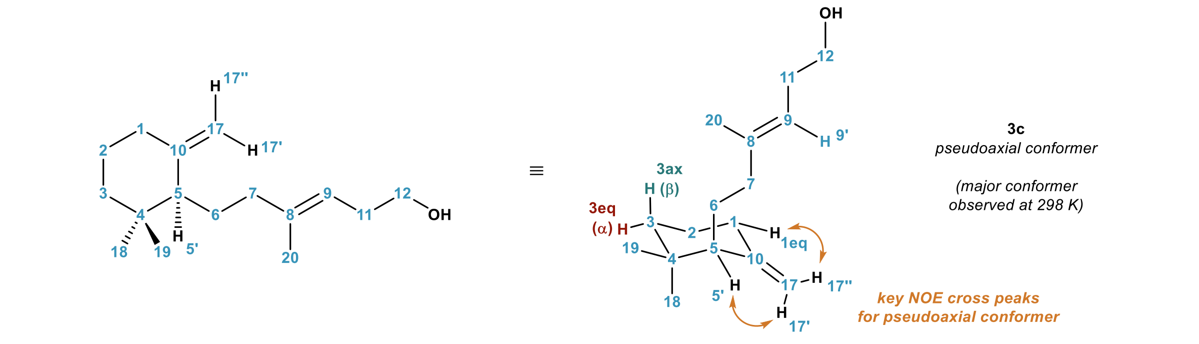

Atom	J	δ (ppm)	HSQC	COSY	HMBC	NOESY
1 C		32.657	1ax, 1eq		2, 3ax, 3eq, 5', 17', 17''	
Hax		2.060	1	1eq, 2, 17', 17''	10, 17	2, 17''
Heq		1.995	1	1ax	10, 17	17''
2 C		23.853	2		3eq	
H2		1.528	2	1ax, 3ax, 3eq	1, 3, 4, 10	1ax, 3ax, 3eq, 18
3 C		36.432	3ax, 3eq		2, 5', 18, 19	
Hax (β)	13.2 (3eq), 8.80 (2ax), 5.80 (2eq)	1.460	3	2, 3eq	1, 4, 5, 18, 19	2, 3eq, 19
Heq (α)	13.2 (3ax), 4.74 (n.a.), 4.74 (n.a.)	1.208	3	2, 3ax	1, 2, 4, 5, 18, 19	2, 3ax, 18, 19
4 C		35.038			2, 3ax, 3eq, 5', 6b, 18, 19	
5 C		53.863	5'		3ax, 3eq, 6b, 7', 7'', 17', 17'', 18, 19	
5' H		1.681	5	6a, 6b, 17'	1, 3, 4, 6, 7, 10, 17, 18	17', 18, 19
6 C		24.902	6a, 6b		5', 7', 7''	
Ha		1.432	6	5', 7', 7''	8, 10	9', 17'
Hb		1.556	6	5', 7', 7''	4, 5, 7, 8, 10	9', 19
7 C		38.539	7', 7''		5', 6b, 9', 20	
H'		1.977	7	6a, 6b, 7'', 9', 11, 20	5, 6, 8, 9, 20	9', 17', 20
H''		1.779	7	6a, 6b, 7', 9', 11	5, 6, 8, 9, 20	9', 17', 20
8 C		139.724			6a, 6b, 7', 7'', 11, 20	

Atom	J	δ (ppm)	HSQC	COSY	HMBC	NOESY
9 C		119.565	9'		7', 7'', 11, 12, 20	
9' H	7.3(11), 1.3(n.a.)	5.115	9	7', 7'', 11, 20	7, 11, 12, 20	6a, 6b, 7', 7'', 11, 12
10 C		149.459			1ax, 1eq, 2, 5', 6a, 6b, 17', 17''	
11 C		31.678	11		9', 12	
H2	6.5(12), 7.3(9')	2.287	11	7', 7'', 9', 12, 20	8, 9, 12	9', 12, 20
12 C		62.692	12		9', 11	
H2	6.5(11)	3.623	12	11	9, 11	9', 11
OH		1.358				
17 C		109.040	17', 17''		1ax, 1eq, 5'	
H'	2.5(17''), 0.8(n.a.)	4.534	17	1ax, 5'	1, 5, 10	5', 6a, 7', 7''
H''	2.5(17'), 1.4(n.a.), 0.8(n.a.)	4.756	17	1ax	1, 5, 10	1ax, 1eq
18 C		28.568	18		3ax, 3eq, 5', 19	
H3		0.910	18	19	3, 4, 5, 19	2, 3eq, 5', 19
19 C		26.409	19		3ax, 3eq, 18	
H3		0.833	19	18	3, 4, 5, 18	3ax, 3eq, 5', 6b, 18
20 C		16.506	20		7', 7'', 9'	
H3		1.638	20	7', 9', 11	7, 8, 9	7', 7'', 11

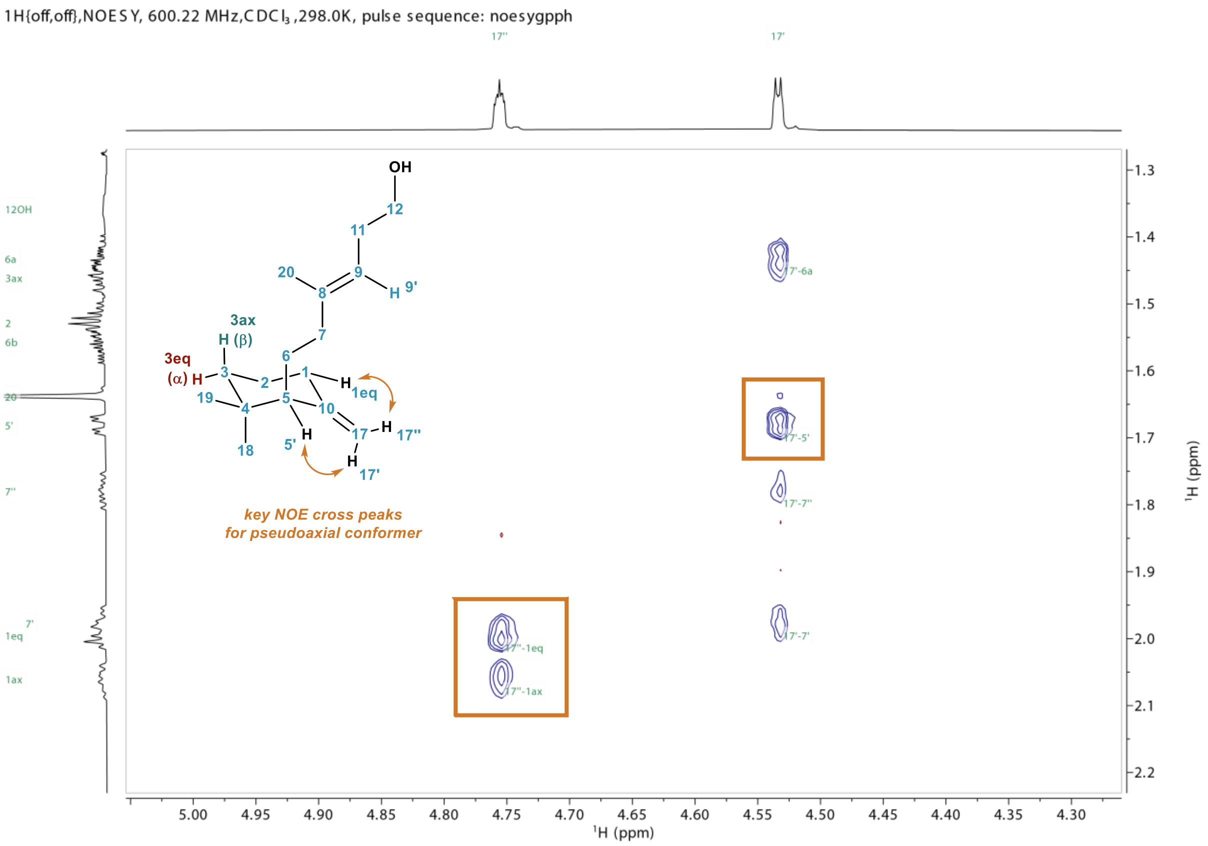

Fig. S15 | Excerpt of the ^{1}H,^{1}H-NOESY (600 MHz, $CDCl_3$) spectrum of **3c** highlighting key NOE interactions indicative for the pseudoaxial conformer.

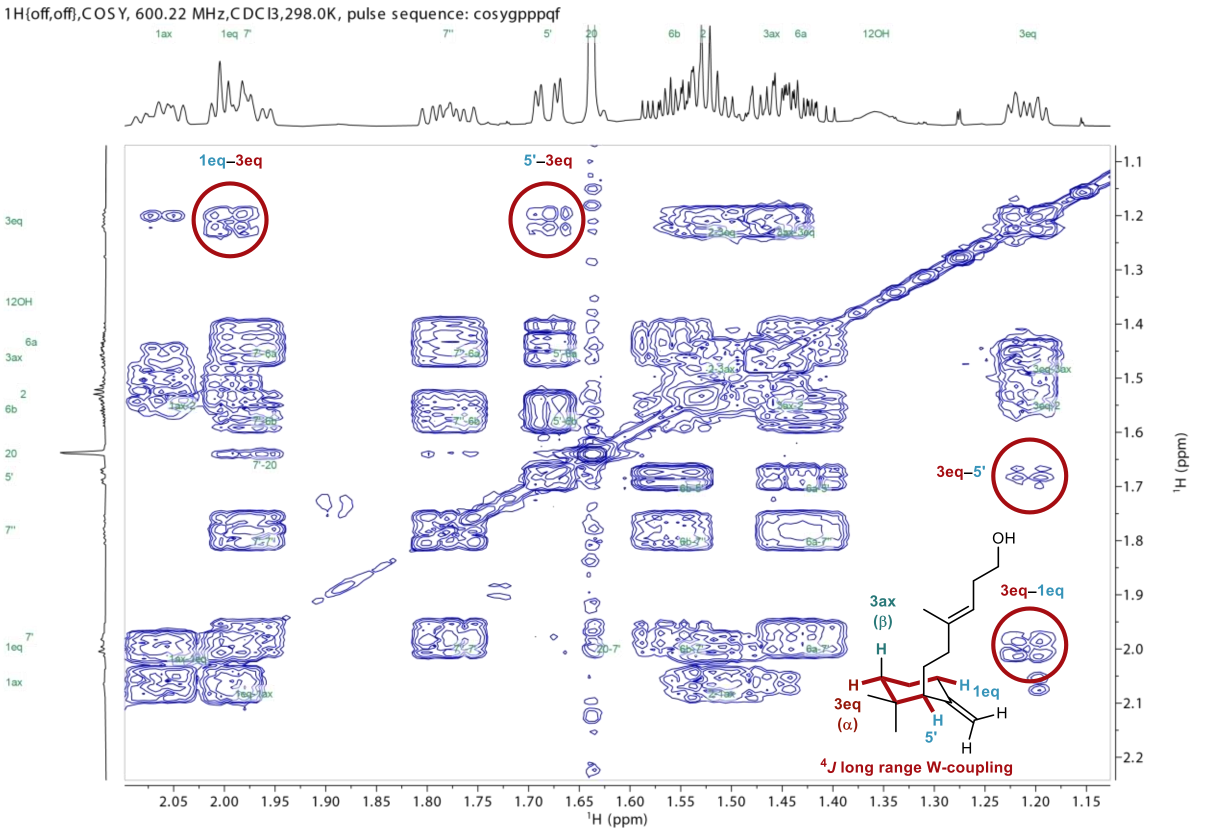

Fig. S16 | Excerpt of the ^{1}H,^{1}H-COSY (600 MHz, $CDCl_3$) spectrum of **3c** highlighting key ^{4}J long range W-couplings indicative for the pseudoaxial conformer.

Characterization data for homodrimenols (4)

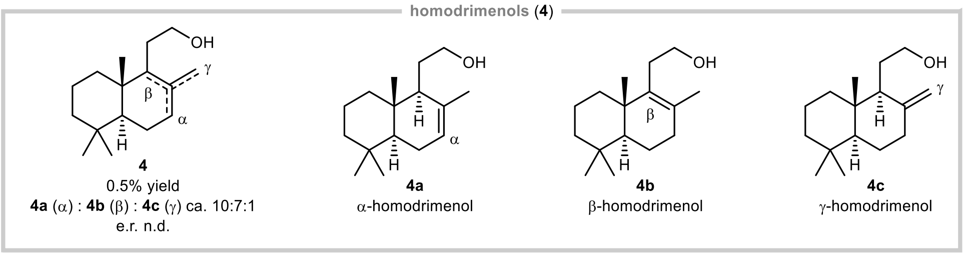

A trace amount of homodrimenols (5.40 mg, 22.8 µmol, 0.5% yield, 90% purity, mixture of regio-isomers; **4a**:**4b**:**4c** approximately 10:7:1) was separated from the polar fraction by preparative HPLC. The identity of the individual components was confirmed by comparison of measured ^{1}H and in particular ^{13}C NMR spectra with reported literature data.

Diagnostic ^{1}H and ^{13}C NMR signals for α-homodrimenol (**4a**) are provided below:

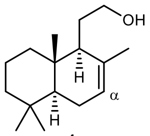

4a
α-homodrimenol

^{1}H NMR (501 MHz, $CDCl_3$): δ (ppm) = 5.42 (td, *J* = 3.7, 2.0 Hz, 1H), 3.79 (td, *J* = 9.8, 5.0 Hz, 1H), 3.64–3.51 (m, 2H), 2.05–1.92 (m, 1H), 1.90–1.80 (m, 1H), 1.72 (ddt, *J* = 14.0, 6.5, 1.7 Hz, 1H), 1.67 (dt, *J* = 2.4, 1.2 Hz, 3H), 1.67–1.61 (m, 1H), 1.61–1.40 (m, 4H), 1.43–1.37 (m, 1H), 1.19 (dd, *J* = 12.1, 4.8 Hz, 1H), 1.14 (dd, *J* = 13.5, 3.4 Hz, 1H), 0.96 (td, *J* = 13.2, 3.8 Hz, 1H), 0.88 (s, 3H), 0.85 (s, 3H), 0.77 (s, 3H).

^{13}C{^{1}H} NMR (126 MHz, $CDCl_3$): δ (ppm) = 134.7, 122.8, 64.6, 50.9, 50.3, 42.4, 39.4, 36.6, 33.3, 33.1, 30.6, 24.0, 22.2, 22.0, 18.9, 13.7. See Fig. S17 for comparison with literature data.

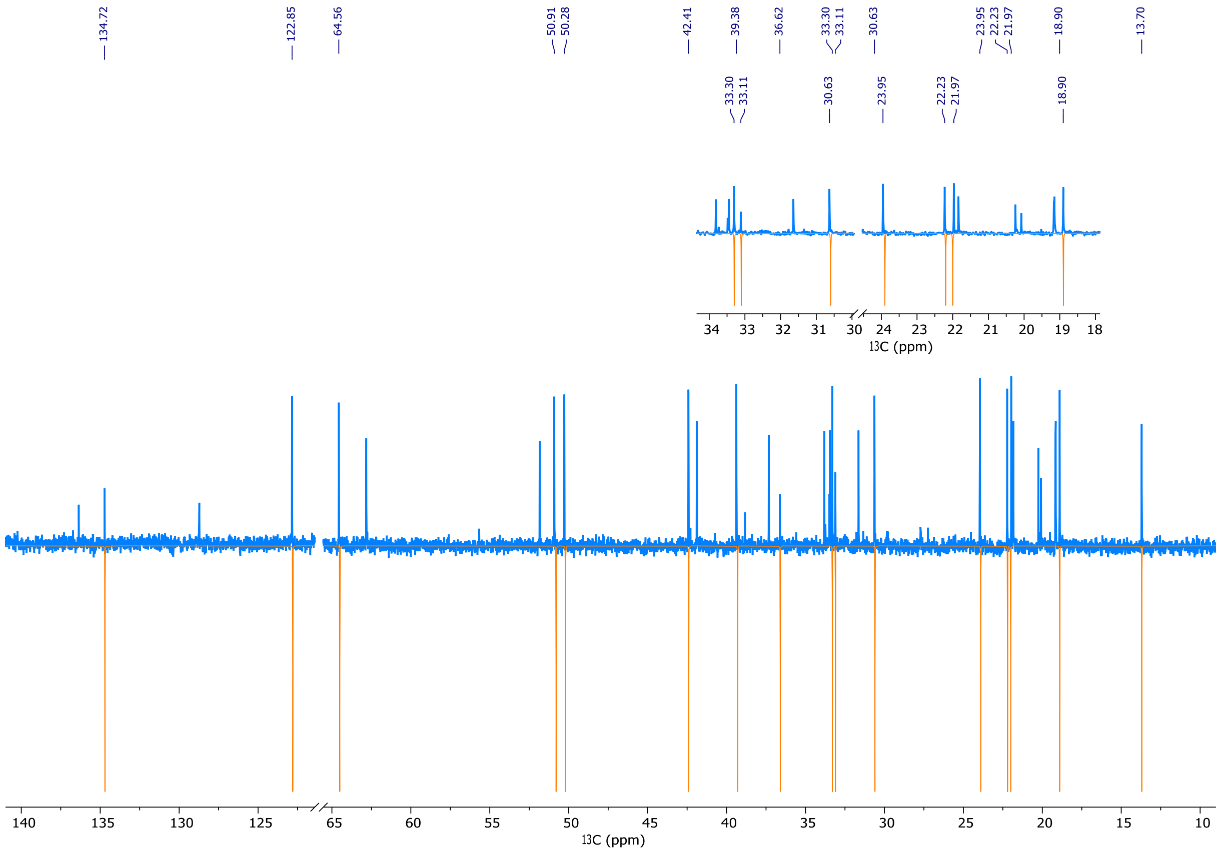

Fig. S17 | Comparison of the measured ^{13}C NMR (126 MHz, $CDCl_3$) spectrum (blue) with a ^{13}C NMR spectrum of α-homodrimenol (**4a**) generated from reported literature data (orange).[32]

Diagnostic ^{1}H and ^{13}C NMR signals for β-homodrimenol (**4b**) are provided below:

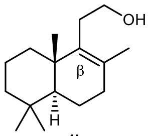

4b
β-homodrimenol

^{1}H NMR (501 MHz, $CDCl_3$): δ (ppm) = 3.65–3.58 (m, 1H), 3.60–3.52 (m, 1H), 2.39 (ddd, *J* = 17.5, 9.0, 3.9 Hz, 1H), 2.24 (ddd, *J* = 14.0, 9.9, 5.9 Hz, 1H), 2.07–1.98 (m, 1H), 1.99–1.93 (m, 1H), 1.61 (s, 3H), 1.12–1.07 (m, 1H), 0.95 (s, 3H), 0.88 (s, 3H), 0.83 (s, 3H).

^{13}C{^{1}H} NMR (126 MHz, $CDCl_3$): δ (ppm) = 136.36, 128.72, 62.82, 51.84, 41.88, 38.83, 37.32, 33.81, 33.48, 33.45, 31.64, 21.84, 20.24, 20.08, 19.17, 19.15. See Fig. S18 for comparison with literature data.

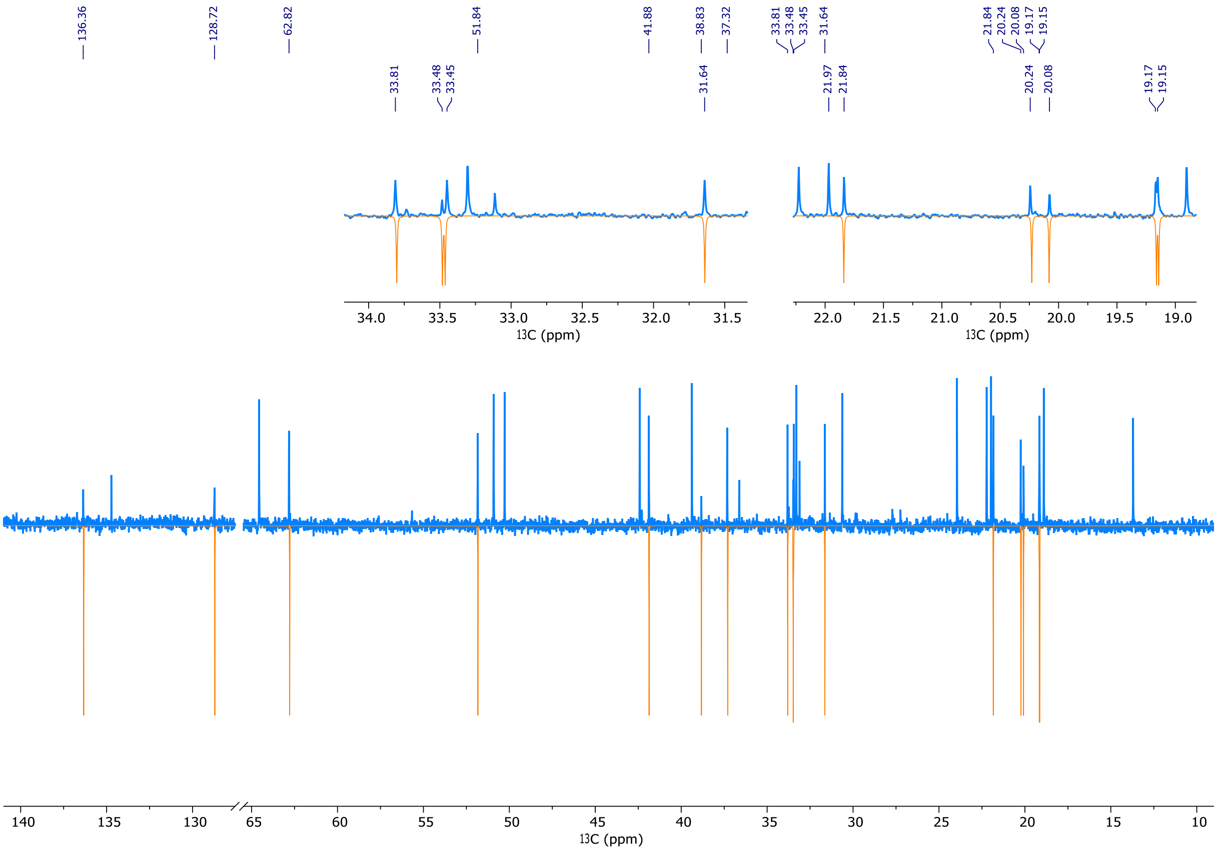

Fig. S18 | Comparison of the measured ^{13}C NMR (126 MHz, $CDCl_3$) spectrum (blue) with a ^{13}C NMR spectrum of β-homodrimenol (**4b**) generated from literature data (orange).[33]

Diagnostic ^{1}H and ^{13}C NMR signals for γ-homodrimenol (**4c**) are provided below:

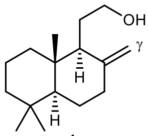

4c
γ-homodrimenol

^{1}H NMR (501 MHz, $CDCl_3$): δ (ppm) = 4.83 (q, *J* = 1.7 Hz, 1H), 4.54 (q, *J* = 1.5 Hz, 1H), 3.72 (td, *J* = 7.7, 3.3 Hz, 1H), 3.53–3.49 (m, 1H), 0.87 (s, 3H), 0.81 (s, 3H), 0.69 (s, 3H); only characteristic peaks are reported due to the low content in the mixture and significant signal overlap.

^{13}C{^{1}H} NMR (126 MHz, $CDCl_3$): δ (ppm) = 148.92, 106.53, 62.76, 55.66, 53.00, 42.32, 39.60, 39.19, 38.38, 33.73, 27.24, 24.52, 21.84, 19.52, 14.64. See Fig. S19 for comparison with literature data.

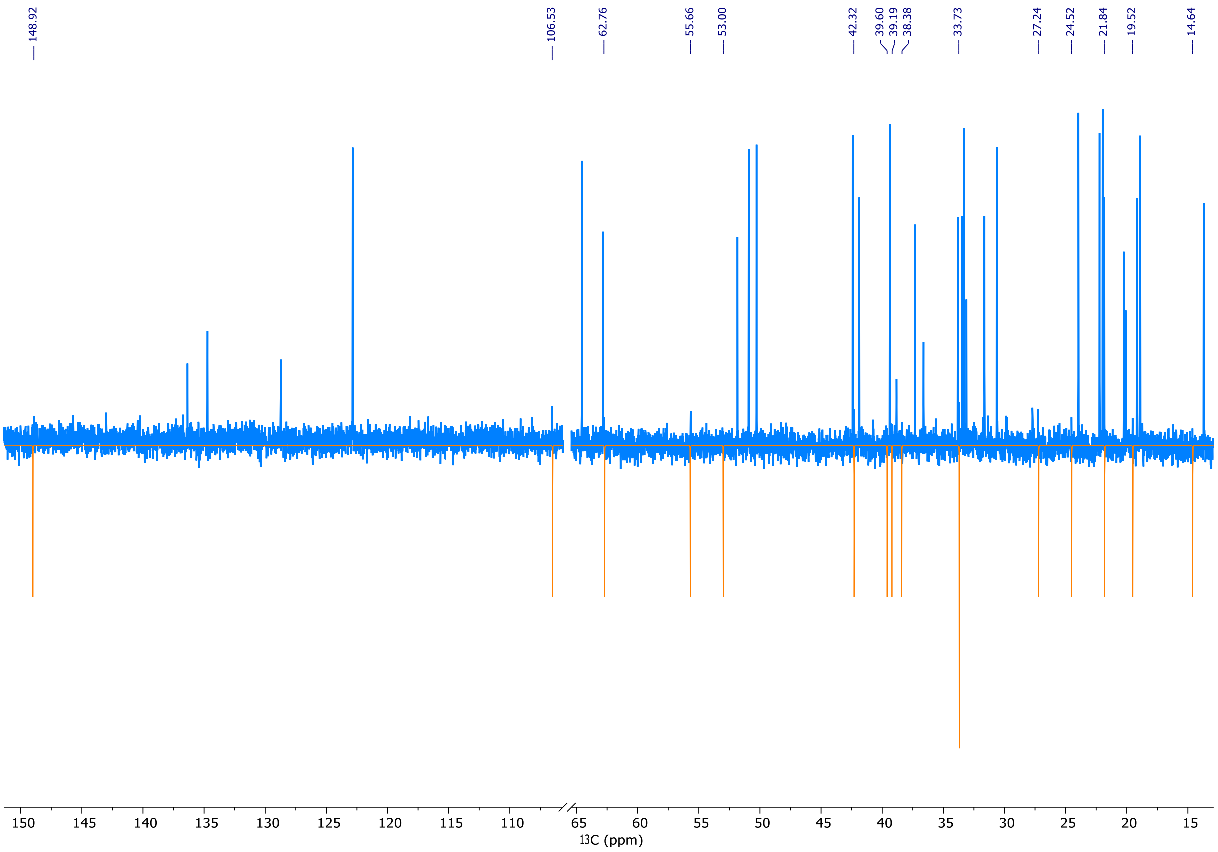

Fig. S19 | Comparison of the measured ^{13}C NMR (126 MHz, $CDCl_3$) spectrum (blue) with a ^{13}C NMR spectrum of γ-homodrimenol (**4c**) generated from literature data (orange).[34,35]

GC and HPLC data for a mixture of homodrimenols (**4a**:**4b**:**4c** approximately 10:7:1):

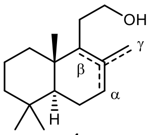

4
0.5% yield
4a (α) : **4b** (β) : **4c** (γ) ca. 10:7:1
e.r. n.d.

GC (achiral) (DB-Waxetr 0.25/0.25df G/770; 30.0 m, temperature: 220/50 5/min 260 12/min 280, 3 min iso/ 350, 0.60 bar H_2, sample size: 1.0 µL, split ratio 40:1): β-homodrimenol: t_R(**4b**) = 36.11 min (34.67%), γ-homodrimenol: t_R(**4c**) = 36.62 min (4.91%), α-homodrimenol: t_R(**4a**) = 37.96 min (50.61%).

HPLC (achiral) (250 mm Multokrom 100-Si, 20 mm i.D., 3µm; *i*-hexan/propan-2-ol 99.5:0.5 *v/v*, 14.8 mL/min, UV: λ = 205 nm, 298 K): t_R(**4a**/**4b**/**4c**) = 36.49 min (2.73%).

To ensure full conversion of the starting material **1a**, cyclohomofarnesols **3**, and homodrimenols **4**, the scale-up experiment was repeated and the reaction mixture was warmed to 0 °C after 12 h reaction time at −40 °C and stirred for an additional 8 h at 0 °C(see below).

Scale-up Experiment at −40 °C → 0 °C (5 mmol scale)

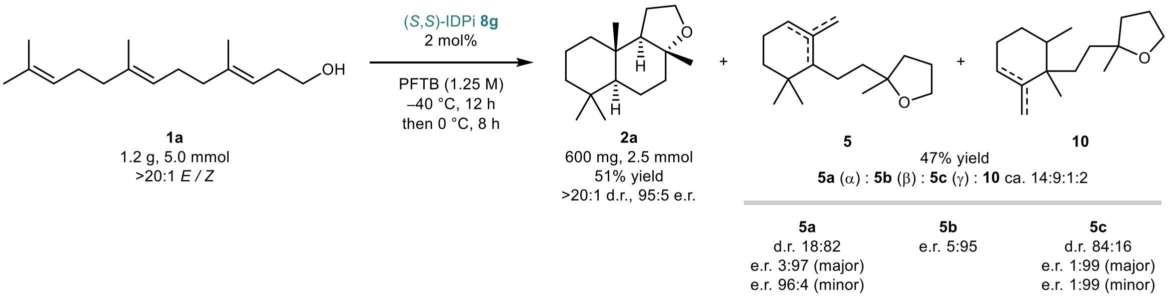

A 100 mL Schlenk flask under argon equipped with a PTFE-coated magnetic stir bar was charged with (3*E,*7*E*)-homofarnesol **1a** (1.18 g, 5.00 mmol, 1.0 equiv.) and (*S*,*S*)-IDPi catalyst **8g** (254 mg, 0.10 mmol, 2 mol%). The mixture was cooled to −40 °C and stirred for 10 min. Perfluoro-*tert*-butanol (PFTB, 4.0 mL, 5.7 equiv., 1.25 M) was added and the reaction mixture was stirred for 12 h until full consumption of the starting material was observed as indicated by TLC. Afterwards, the reaction temperature was increased to 0 °C and the reaction mixture was then stirred for another 8 h at this temperature. The resulting mixture was neutralized with triethylamine, the solvent was evaporated under reduced pressure, and the residue was subjected to column chromatography on silica gel (gradient elution with hexanes/ethyl acetate 50:1 → 1:4 *v/v*) to give ambrox **2a** in 51% yield (603 mg, 2.55 mmol, 91:9 d.r., 95:5 e.r.), alongside a mixture of partially cyclized products **5** and rearranged product **10** in 47% yield (**5a**:**5b**:**5c**:**10** ca. 14:9:1:2). To recover the IDPi catalyst, the column was eluted with hexanes/ethyl acetate (1:4) providing (*S*,*S*)-IDPi catalyst **8g** as a salt. Acidification in DCM with aq. HCl (6 M) followed by evaporation of the solvent under reduced pressure and drying under high vacuum furnished protonated (*S*,*S*)-IDPi catalyst **8g** as a colorless solid 253 mg (IDPi recovery >99%). The purity of the recovered catalyst was verified by ^{1}H and ^{31}P NMR spectroscopy.

GC Data for the crude reaction mixture:

GC (achiral) (DB-5MS 0.25/0.25df, 30.0 m, temperature: 220/ 159 iso 50 min, 20/min, 340 iso 5 min /350, 0.60 bar H_2, sample size: 0.2 µL): t_{R1} = 19.7 min (**5c**), t_{R2} = 20.7 min (**5a**), t_{R3} = 23.1 (**5b**), t_{R4} = 36.9 min (ambrox, **2a**), t_{R5} = 39.0 min (5β,8α,9β-ambrox, **2b**).

GC data for a sample of **2a** obtained at −40 °C → 0 °C:

GC (achiral) (Optima-35 0.25/0.25df G/706, 29.0 m; temperature: 220/50 5/min 200 12/min 350, 5 min iso/ 350, 0.60 bar H_2, sample size: 0.2 µL, split ratio: 120:1): 9-*epi*-ambrox: t_R(**2b**) = 26.71 min (2.91%), 5β-ambrox: t_R(**2e**) = 26.97 min (1.31%), 5β,8α-ambrox: t_R(**2d**) = 27.04 min (0.17%), ambrox: t_R(**2a**) = 27.52 min (90.73%), 5β,8α,9β-ambrox: t_R(**2c**) = 27.97 min (4.86%).

GC (chiral) (BGB 176/BGB-15 0.25/0.25df G/618, 30.0 m; temperature: 220/140, 60 min iso 8/min 240/ 350, 0.60 bar H_2, sample size: 0.2 µL): (−)-9-*epi*-ambrox: t_R(**2b**) = 41.92 min (2.61%), 5β-ambrox: t_R(**2e**) = 42.41 min (0.28%), (−)-9-*epi*-ambrox: t_R(**2b**) = 42.96 min (0.31%), 5β,8α-ambrox: t_R(**2d**) = 43.70 min (1.14%), (−)-ambrox: t_R(**2a**) = 49.08 min (86.68%), (+)-ambrox: t_R(**2a**) = 51.14 min (4.71%), 5β,8α,9β-ambrox: t_R(**2c**) = 52.94 min (3.24%), *ent*-5β,8α,9β-ambrox: t_R(**2c**) = 52.94 min (1.03%).

Characterization data for partially cyclized compounds 5 and isomerized products 10:

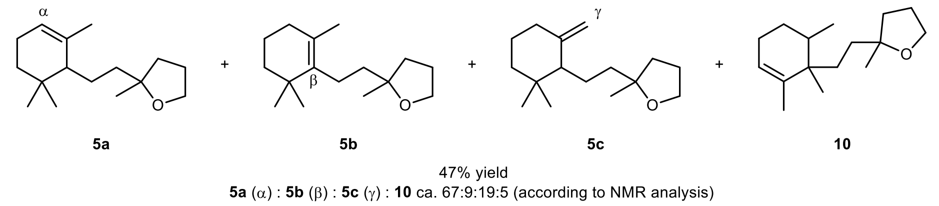

The major components in the least polar fraction are a mixture of partially cyclized compounds **5** and isomerized product **10**. A possible mechanistic proposal for the formation of partially cyclized compounds **5** from cyclohomofarnesols **3** or (3*E*,7*E*)-homofarnesol is provided below (Scheme 3).

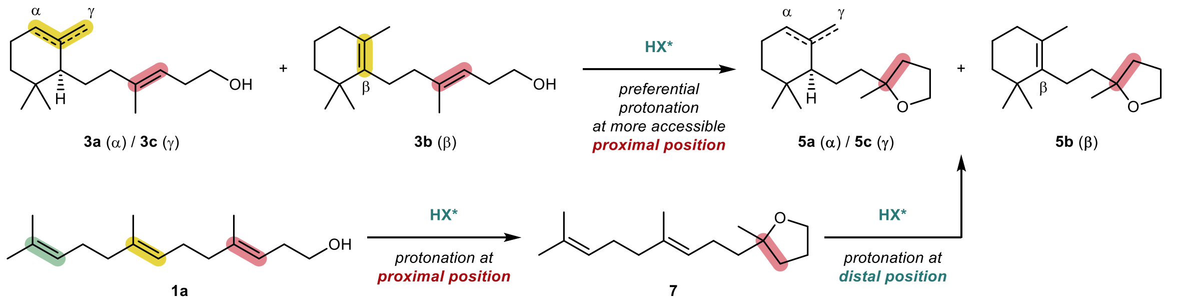

Scheme 3 | Two possible pathways for the formation of the observed partially cyclized products **5**.

It is worth mentioning that only minor amounts of **5** are formed at low temperatures and that tetrahydrofuran **7**, resulting from protonation of the proximal position of **1a**, could not be detected in the reaction mixture. Thus, the formation of **5** likely proceeds via cyclohomofarnesols **3** which have been isolated and characterized as intermediates in the reaction mixture.

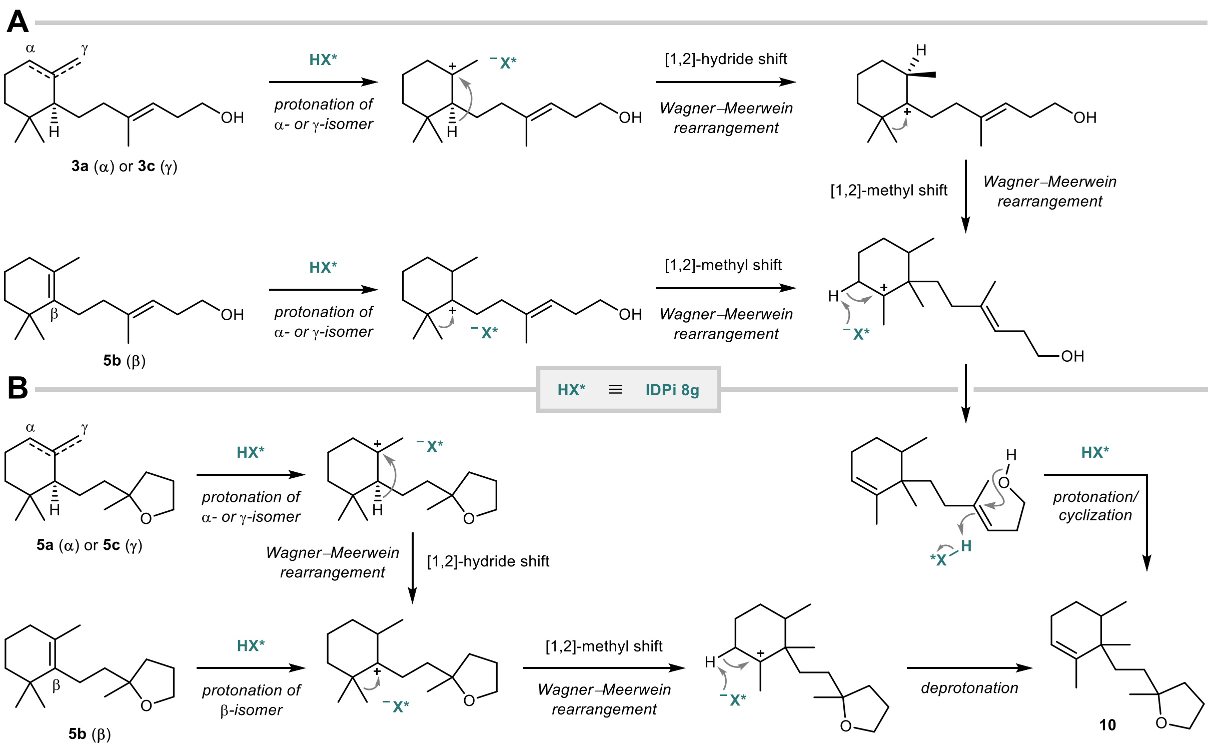

Scheme 4 | Possible mechanistic scenarios for the formation of isomerized side product **10**.

Notably, the amount of isomerized product **10** increases to appreciable amounts in reactions that were warmed from −40 °C to 0 °C. This suggests that **10** probably originates from a subsequent isomerization reaction at higher reaction temperatures. Two possible mechanistic pathways for the formation of **10** from cyclohomofarnesols **3** or partially cyclized products **5** are provided in Scheme 4. The two scenarios solely differ in the order of reaction steps. In the first proposal, cyclohomofarnesols undergo either one (β-isomer, **3b**) or two (α-isomer, **3a** and γ-isomer **3c**) Wagner–Meerwein rearrangements resulting in the formation of isomerized cyclohomofarnesols. A subsequent cyclization at the double bond in proximity to the hydroxy group closes the tetrahydrofuran and furnishes the isolated isomerized product **10** (Scheme 4A). In contrast to this, the abovementioned reaction steps are reversed in the second mechanistic scenario (Scheme 4B).

Characterization data for partially cyclized side products **5** and isomerized product **10** are provided on the following pages (see Fig. S20 for an overview).

Chiral GC data for 5a-5c:

Lipodex-G 0.25/0.00df G/602, 25 m, temperature: 220/60 iso 1000 min, 0.50 bar H_2, sample size: 1 µL, t_{R1} = 529.0 min (**5c**), t_{R2} = 550.8 min (**5c**), t_{R3} = 567.3 min (**5c**), t_{R4} = 601.3 min (**5a**), t_{R5} = 634.1 min (**5a**), t_{R6} = 693.3 min (**5a**), t_{R7} = 708.1 min (**5a**), t_{R8} = 739.3 min (**5b**), t_{R9} = 758.8 min (**5b**).

Cyclodextrin-H 0.25/0.125df G/632, 24 m, temperature: 220/60 iso 1000 min, 0.50 bar H_2, sample size: 1 µL, t_{R1} = 387.8 min (**5c**), t_{R2} = 397.5 min (**5c**), t_{R3} = 407.4 min (**5c**), t_{R4} = 422.0 min (**5c**).

Due to multiple overlapping signals and small shift differences in the ^{1}H and ^{13}C NMR spectrum, the stereochemistry of the individual diastereomers could not be distinguished.

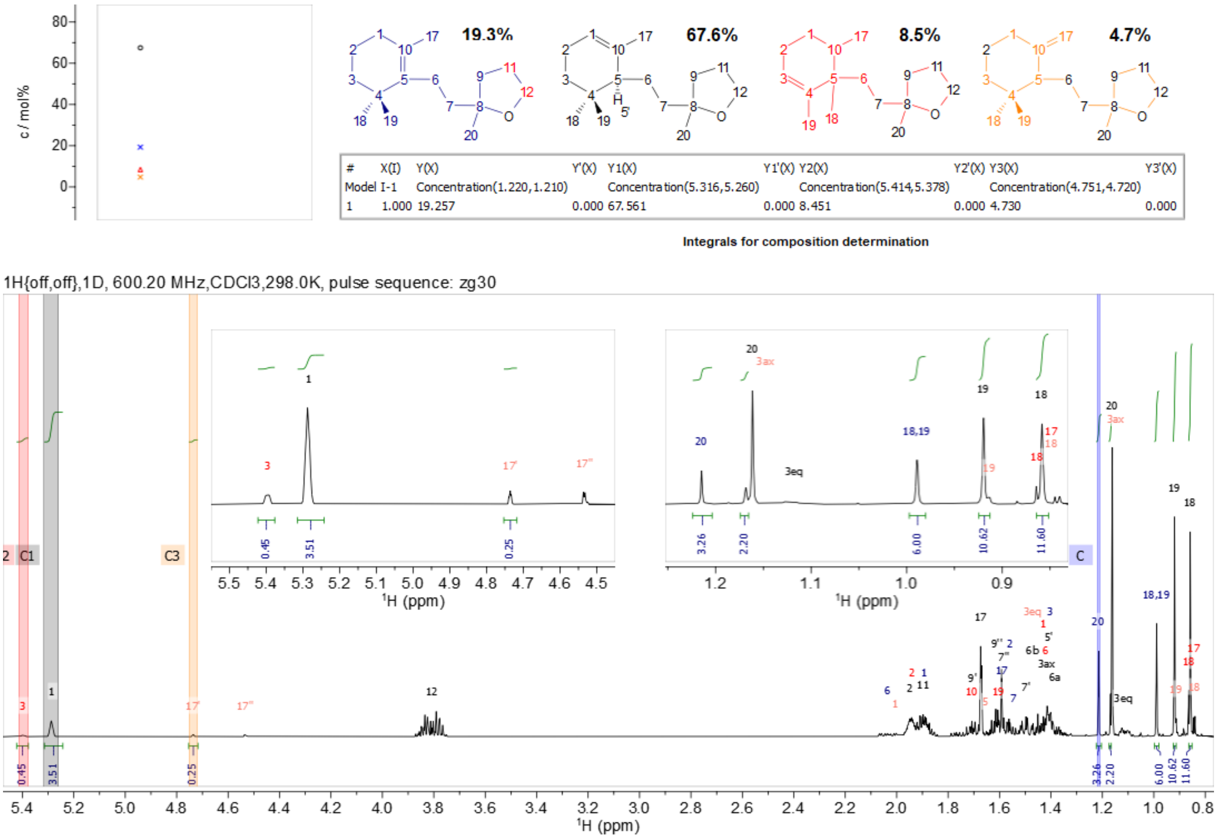

Fig. S20: Excerpt of the ^{1}H NMR (600 MHz, $CDCl_3$) of the fraction containing partially cyclized products **5** and isomerized product **10**. Integration regions used to determine the relative composition are highlighted.

The d.r. of the major component is approximately 82:18 judging from the ^{13}C NMR signals and is in line with the gas chromatographic analysis of the sample. Full assignment of the minor components was not possible. However, characteristic signals in the 1D ^{1}H and ^{13}C as well as 2D NMR datasets were identified which permitted the assignment of the individual components in the sample. An overview with all chemical shifts assignments is provided below.

Table S7 | NMR assignments for partially cyclized product 5a (α-isomer, major component).

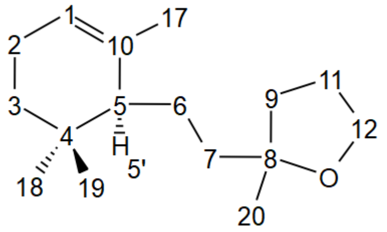

Atom	*J*	δ (ppm)	HSQC	COSY	HMBC	NOESY
**1 C**		120.219	1		3ax, 3eq, 17	
**H**		5.282	1	2, 17	2, 3, 5, 17	2, 17
**2 C**		23.162	2		1, 3eq	
**H2**		1.948	2	1, 3ax, 3eq, 17		1, 3ax, 3eq, 18
**3 C**		31.881	3ax, 3eq		1, 18, 19	
**Hax**	13.3(3eq)	1.416	3	2, 3eq	1, 4, 5	2, 3eq, 19
**Heq**	13.3(3ax), 5.7(n.a.), 3.4(n.a.)	1.118	3	2, 3ax	1, 2, 4, 5	2, 3ax, 18, 19
**4 C**		32.753			3ax, 3eq, 6b, 18, 19	
**5 C**		49.603	5'		1, 3ax, 3eq, 6b, 7', 7", 17, 18, 19	
**5' H**		1.405	5	17	7, 10	17, 18, 19
**6 C**		25.936	6a, 6b		7', 7"	
**Ha**		1.382	6	7', 7"		17
**Hb**		1.470	6	7', 7"	4, 5, 7, 8, 10	19
**7 C**		41.530	7', 7"		5', 6b, 9', 20	
**H'**		1.496	7	6a, 6b	5, 6, 8, 9, 20	17, 19, 20
**H"**		1.581	7	6a, 6b	5, 6, 8, 9, 20	17, 19, 20
**8 C**		83.029			6b, 7', 7", 11, 12, 20	
**9 C**		36.535	9', 9"		7', 7", 11, 12, 20	
**H'**		1.702	9	11	7, 11, 12, 20	
**H"**		1.609	9	11	11, 12, 20	
**10 C**		136.622			5', 6b, 17	
**11 C**		26.200	11		9', 9", 12	
**H2**		1.896	11	9', 9", 12	8, 9, 12	20
**12 C**		67.096	12		9', 9", 11	
**H2**		3.806	12	11	8, 9, 11	
**17 C**		23.586	17		1	
**H3**	1.7(n.a.)	1.671	17	1, 2, 5'	1, 5, 10	1, 5', 6a, 7', 7"
**18 C**		27.858	18		19	
**H3**		0.859	18	19	3, 4, 5, 19	2, 3eq, 5'
**19 C**		27.643	19		18	
**H3**		0.919	19	18	3, 4, 5, 18	3ax, 3eq, 5', 6b, 7', 7"
**20 C**		25.637	20		7', 7", 9', 9"	
**H3**		1.161	20		7, 8, 9	7', 7", 11

Table S8 | Partial NMR assignments for partially cyclized product **5b** (β-isomer).

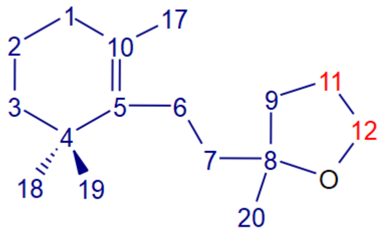

Atom	δ (ppm)	HSQC	HMBC	NOESY
**1 C**	32.925	1	17	
**H2**	1.889	1	2, 3, 5, 10, 17	
**2 C**	19.698	2	1	
**H2**	1.557	2		
**3 C**	40.045	3	1, 18, 19	
**H2**	1.402	3		18, 19
**4 C**	35.232		18, 19	
**5 C**	137.120		1, 6, 17, 18, 19	
**6 C**	23.634	6	7	
**H2**	2.034	6	5, 7, 10	18, 19
**7 C**	41.134	7	6, 20	
**H2**	1.544	7	6	18, 19
**8 C**	82.883		20	
**9 C**	36.832		20	
**H2**				
**10 C**	126.910		1, 6, 17	
**11 C**				
**H2**				
**12 C**				
**H2**				
**17 C**	19.853	17	1	
**H3**	1.590	17	1, 5, 10	
**18 C**	28.795	18	19	
**H3**	0.989	18	3, 4, 5, 19	3, 6, 7
**19 C**	28.784	19	18	
**H3**	0.989	19	3, 4, 5, 18	3, 6, 7
**20 C**	25.493	20		
**H3**	1.214	20	7, 8, 9	

Table S9 | Partial NMR assignments for partially cyclized product 5c (γ-isomer).

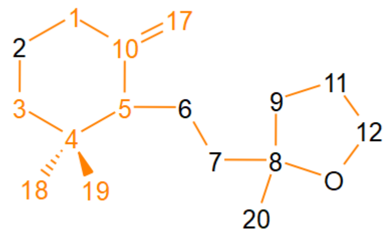

Atom	δ (ppm)	HSQC	HMBC
**1 C**	32.459	1	17'
**H2**	2.004	1	
**2 C**			
**H2**			
**3 C**	35.143	3ax, 3eq	18, 19
**Hax**	1.152	3	
**Heq**	1.471	3	
**4 C**	36.303		18, 19
**5 C**	54.684	5	17', 18, 19
**H**	1.653	5	
**6 C**			
**H2**			
**7 C**			
**H2**			
**8 C**			
**9 C**			
**H2**			

Atom	δ (ppm)	HSQC	HMBC
10 C	149.510		
11 C			
H2			
12 C			
H2			
17 C	109.093	17', 17''	
H'	4.733	17	1, 5
H''	4.531	17	
18 C	26.472	18	19
H3	0.840	18	3, 4, 5, 19
19 C	28.578	19	18
H3	0.913	19	3, 4, 5, 18
20 C			
H3			

Table S10 | Partial NMR assignments for isomerized product 10.

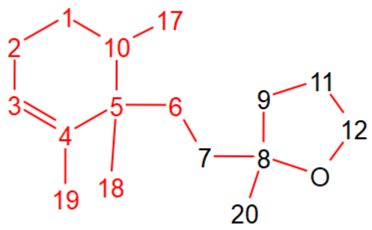

Atom	δ (ppm)	HSQC	COSY	HMBC	NOESY
**1 C**	27.198	1		3, 17	
**H2**	1.428	1		5	
**2 C**	25.690	2			
**H2**	1.938	2	3		3
**3 C**	124.218	3			
**H**	5.396	3	2, 19	1, 5, 19	2, 19
**4 C**	139.834			6, 19	
**5 C**	40.274			1, 3, 6, 17, 19	
**6 C**	31.213	6		18	
**H2**	1.417	6		4, 5	
**7 C**					
**H2**					
**8 C**					
**9 C**					
**H2**					
**10 C**	33.368	10		17, 18	
**H**	1.706	10	17		
**11 C**					
**H2**					
**12 C**					
**H2**					
**17 C**	15.931	17			
**H3**	0.844	17	10	1, 5, 10	
**18 C**	21.351	18			
**H3**	0.864	18		6, 10	
**19 C**	19.253	19		3	
**H3**	1.603	19	3	4, 5	3
**20 C**					
**H3**					

Scale-up Experiment at −40 °C → 0 °C (42 mmol scale)

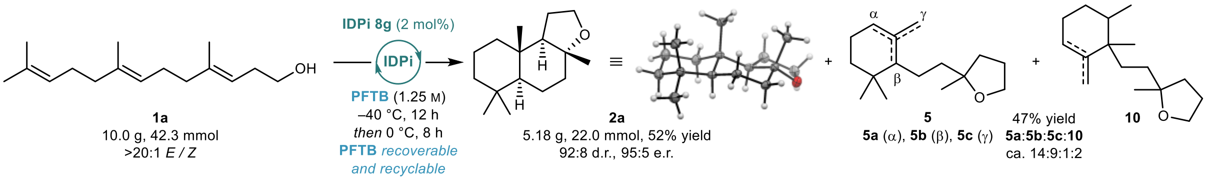

A 100 mL Schlenk flask under argon equipped with a PTFE-coated magnetic stir bar was charged with (3*E*,7*E*)-homofarnesol **1a** (10.0 g, 42.3 mmol, 1.0 equiv.) and IDPi catalyst **8g** (2.15 g, 0.85 mmol, 2 mol%). The mixture was cooled to −40 °C and stirred for 10 min. Perfluoro-*tert*-butanol (PFTB, 33.8 mL, 1.25 M, 5.7 equiv.) was added and the reaction was stirred for 12 h. Then, the reaction mixture was stirred at 0 °C for another 8 h to ensure full consumption of monocyclic intermediates. To recover the solvent, the resulting mixture was distilled under reduced pressure. To this end, a distillation bridge with a Schlenk tube immersed in dry-ice or liquid nitrogen and connected to a membrane vacuum pump was attached to the reaction vessel. The temperature of the reaction mixture was kept at 0 °C, PFTB was collected at $p \approx 1$ mbar and obtained as a clear colorless liquid. The residue was subjected to column chromatography on silica gel (gradient elution with hexanes/ethyl acetate 50:1 → 1:4 *v/v*) to give ambrox **2a** in 52% yield (5.18 g, 22.0 mmol, 92:8 d.r., 95:5 e.r.), and a mixture of partially cyclized product **5a**-**5c** alongside isomerized product **10** in 47% yield (4.70 g, 19.9 mmol, **5a**:**5b**:**5c**:**10** ca. 14:9:1:2). To recover the IDPi catalyst, the column was eluted with hexanes/ethyl acetate (1:4 *v/v*) to afford the IDPi catalyst **8g** as a salt. The protonated IDPi **8g** was obtained after acidification in DCM with aq. HCl (6 M) and evaporation of the solvent, followed by drying under high vacuum as a colorless solid (2.14 g, 0.85 mmol, IDPi recovery >99%). The purity of the recovered catalyst was verified by ^1H and ^{31}P NMR spectroscopy.

The obtained isolated yield corresponds to a theoretical volumetric productivity of 296 g · L^{-1} in 20 h reaction time. Current optimised biocatalytic production processes of (−)-ambrox achieve full conversion of 300–450 g · L^{-1} (3*E*,7*E*)-homofarnesol in 72 h (WO 2023/175123). Several features of the biocatalytic process render it inherently sustainable, such as the use of biosourced (*E*)-β-farnesene produced from sugar fermentation, the use of water as a benign solvent, and the convenient isolation of (−)-ambrox, which crystallises directly from the reaction mixture. The theoretical volumetric productivity of the chemical route developed in this work is comparable to the enzymatic process and is achieved in a shorter reaction time. However, the need for a purification step to separate the catalyst and the side products from (−)-ambrox leaves room for further improvement.

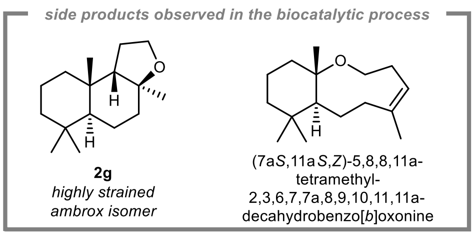

Interestingly, a highly strained ambrox derivative and a bicyclic decahydrobenzo[*b*]oxonine (see box on the right) were observed as side products in the biocatalytic process but could not be observed in the IDPi-catalyzed asymmetric polyene cyclization. One explanation for the absence of both

side products in this work could be the lower reaction temperatures (−40 °C and −30 °C → 0 °C) at which the reaction is carried out compared to the biocatalytic process. Another reason for the absence of the highly strained compound could be a preferential selection of the all-*trans* conformer leading to (−)-ambrox. The absence of the bicyclic compound can be tentatively explained by invoking a second catalyst molecule that interacts with the hydroxy group, thus likely making a cyclization to the mentioned bicycle geometrically impossible. Instead, the other possible bicyclic product (compound **5**) is formed, probably via a sterically less congested transition state.

A comparison to selected previously reported routes towards (−)-ambrox is provided in the table below.

Table S11 | Comparison of total syntheses of previous and current synthetic approaches toward (−)-ambrox.

Entry	Starting material / approach	Authors / Ref.	No. of steps	Key step(s)	Total yield	d.r.	e.r.
1	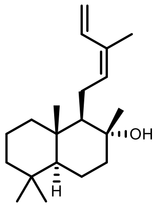 **(*Z*)-abienol**<br>from Canadian balsam (chiral pool)	Barrero et. al., *Tetrahedron* **1993**, *49*,10405–10412.	3	oxidative degradation (ozonolysis) reduction, cyclization	85	>99:1	>99:1
2	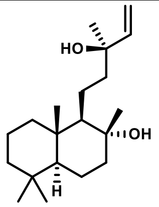 **(+)-sclareol**<br>from clary sage (chiral pool)	Barrero et. al., *Tetrahedron* **1993**, *49*,10405–10412	4	oxidative degradation reduction, cyclization	52–72%	>99:1	>99:1
3	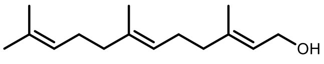 **(2*E*,6*E*)-farnesol**	Yamamoto et. al., *J. Am. Chem. Soc.* **2002**, *124*, 3647–3655.	5	asymmetric polyene cyclization mediated by a chiral Lewis-assisted Brønsted acid	38% (54%[a])	3:1	87.5:12.5
4	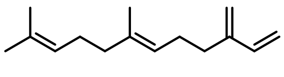 **(*E*)-β-farnesene**<br>obtained from sugar fermentation	WO 2023/175123 Eichhorn, Hauer, Schneider and Ellwood, Tse, WO2022/136232, Int. Pat. to Givaudan	4	synthesis of farnesene from sugar fermentation, ethoxycarbonylation, biocatalytic polyene cyclization catalysed by engineered squalene–hopene–cyclase variants	63% (91%[a])	>99:1	>99:1
5	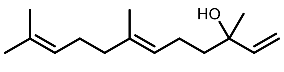 **(*E*)-nerolidol**	List, Schelwies, et. al. *this work* Max-Planck-Institut für Kohlenforschung / BASF	4	catalytic asymmetric polyene cyclization, Pd-catalyzed low pressure carbonylation, enzyme-catalysed isomer resolution	24% (54%[a])	92:8, up to >20:1	95:5

[a]Yield of the polyene cyclization key step.

1.5.3 Catalyst and Solvent Recycling Experiment

Considering the environmental concerns with respect to (per)fluorinated compounds, the possibility to recycle both solvent and catalyst was investigated. To this end, a scale-up was performed as described above. Upon completion of the reaction, the solvent was recovered by vacuum distillation taking advantage of the particular physicochemical properties of PFTB (b.p. = 45 °C at 1013 mbar). Recovered catalyst and solvent were subsequently subjected to another cycle under identical reaction conditions.

First cycle

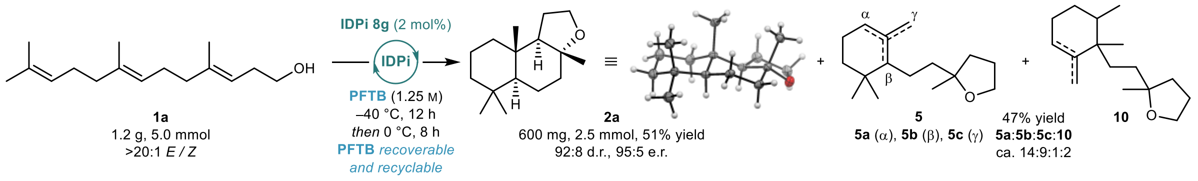

A 100 mL Schlenk flask under argon equipped with a PTFE-coated magnetic stir bar was charged with (3*E*,7*E*)-homofarnesol **1a** (1.18 g, 5.00 mmol, 1.0 equiv.) and IDPi catalyst **8g** (254 mg, 0.10 mmol, 2 mol%). The mixture was cooled to −40 °C and stirred for 10 min. Perfluoro-*tert*-butanol (PFTB, 4.0 mL, 1.25 M, 5.7 equiv.) was added and the reaction mixture was stirred for 12 h until full consumption of the starting material was observed as indicated by TLC. Then, the reaction mixture was stirred at 0 °C for another 8 h to ensure full consumption of monocyclic intermediates. To recover the solvent, the reaction mixture was distilled under reduced pressure. To this end, a distillation bridge with a Schlenk tube immersed in dry-ice or liquid nitrogen and connection to a membrane vacuum pump was attached to the reaction vessel. The temperature of the reaction mixture was kept at 0 °C and PFTB was collected at $p \approx 1$ mbar and obtained as a clear colorless liquid. The residue was subjected to column chromatography on silica gel (gradient elution with hexanes/ethyl acetate 50:1 → 1:4 *v/v*) to give ambrox **2a** in 51% yield (603 mg, 2.55 mmol, 92:8 d.r., 95:5 e.r.), and a mixture of partially cyclized product **5a-5c** alongside isomerized product **10** in 47% yield (**5a**:**5b**:**5c**:**10** ca. 14:9:1:2). To recover the IDPi catalyst, the column was eluted with hexanes/ethyl acetate (1:4 *v/v*) to afford the IDPi catalyst **8g** as a salt. Protonated IDPi **8g** was obtained after acidification in DCM with aq. HCl (6 M) and evaporation of the solvent, followed by drying under high vacuum as a colorless solid (253 mg, 0.10 mmol, IDPi recovery >99%). The purity of the recovered catalyst was verified by ^{1}H and ^{31}P NMR spectroscopy.

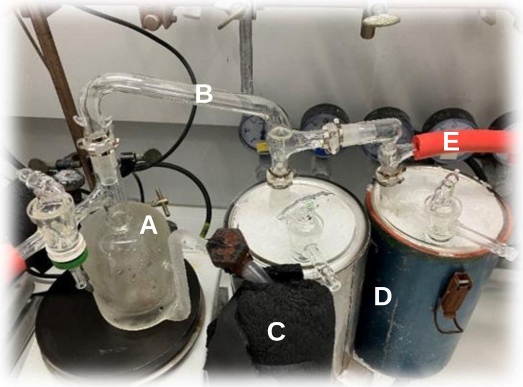

Fig. S21 | Experimental setup for the recovery of PFTB from the crude reaction mixture. (**A**) Reaction vessel with cooling. (**B**) Distillation bridge. (**C**) Connection of the glass cooling mantle to a continuous flow cryostat filled with a cooling liquid (ethanol) to adjust the temperature between −40 °C and room temperature. (**D**) Receiving distillation Schlenk tubes immersed in dry-ice. (**E**) Connection to membrane vacuum pump. For distillation, the cooling was switched off and PFTB was distilled at 0 → 25 °C and a pressure of p = 1 mbar.

Second cycle

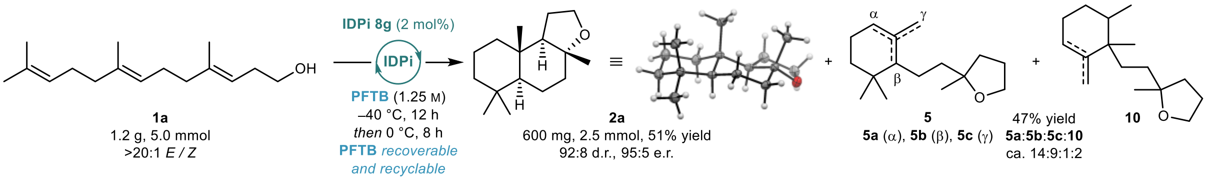

A 100 mL Schlenk flask under argon equipped with a PTFE-coated magnetic stir bar was charged with (3*E,*7*E*)-homofarnesol **1a** (709 mg, 3.0 mmol, 1.0 equiv.) and previously recovered IDPi catalyst **8g** (152 mg, 0.06 mmol, 2 mol%). The mixture was cooled to −40 ºC and stirred for 10 min. Recovered PFTB (2.4 mL, 1.25 M, 5.7 equiv.) was added and the reaction mixture was stirred for 12 h until full consumption of the starting material was observed as indicated by TLC. Then, the solution was stirred at 0 °C for another 8 h. Recovery of PFTB was performed as described above and the residue was subjected to column chromatography on silica gel (gradient elution with hexanes/ethyl acetate 50:1 → 1:4 *v/v*) to give ambrox **2a** in 51% yield (361 mg, 1.53 mmol, 92:8 d.r., 95:5 e.r.), and a mixture of partially cyclized products **5a-5c**, and **10** in 47% yield.

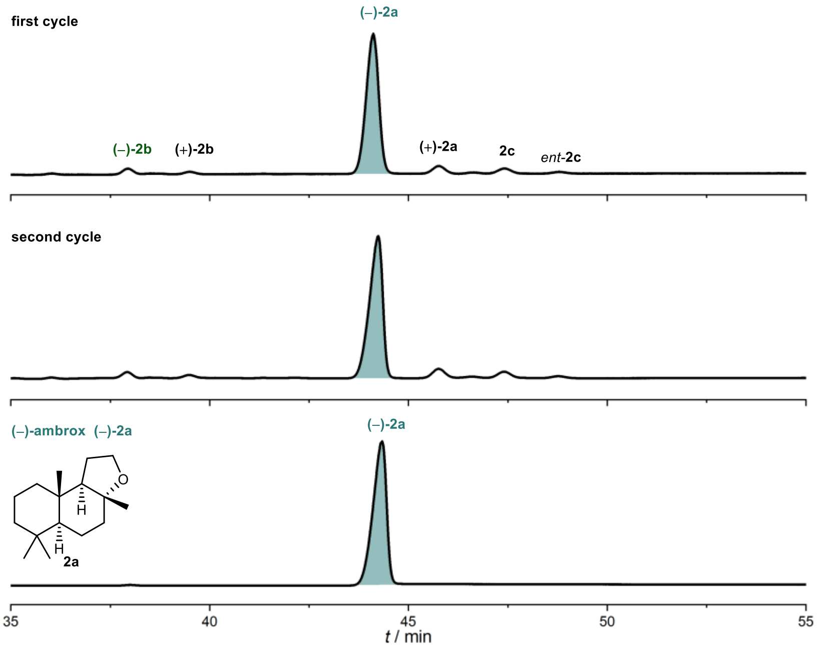

Fig. S22 | GC traces of the first and second cycle from the solvent and catalyst recycling experiment compared to authentic (−)-ambrox (**2a**).

GC retention times for the crude reaction mixture:

GC (achiral) (DB-5MS 0.25/0.25df, 30.0 m, temperature: 220/ 159 iso 50 min, 20/min, 340 iso 5 min, 0.60 bar H_2, sample size: 0.2 µL): t_{R1} = 19.7 min (**5c**), t_{R2} = 20.7 min (**5a**), t_{R3} = 23.1 (**5b**), t_{R4} = 36.9 min (ambrox **2a**), t_{R5} = 39.0 min (5*β*,8*α*,9*β*-ambrox **2b**).

GC data for ambrox (**2a**):

GC (chiral) (BGB 176 0.25/0.25df, 30.0 m, temperature: 220/ 150 iso 60 min, 20/min, 220 iso 5 min, 0.60 bar H_2, sample size: 0.2 µL): t_{R1} = 44.6 min (ambrox **2a**, major), t_{R2} = 46.2 min (*ent*-ambrox *ent*-**2a**, minor).

Chiral GC data for partially cyclized compounds **5a** and **5b**:

GC (chiral) (Lipodex-G 0.25/0.00df G/602, 25 m, temperature: 220/ 60 iso 1000 min, 0.50 bar H_2, sample size: 1.0 µL): t_{R1} = 529.0 min (**5c**), t_{R2} = 550.8 min (**5c**), t_{R3} = 567.3 min (**5c**), t_{R4} = 601.3 min (**5a**), t_{R5} = 634.1 min (**5a**), t_{R6} = 693.3 min (**5a**), t_{R7} = 708.1 min (**5a**), t_{R8} = 739.3 min (**5b**), t_{R9} = 758.8 min (**5b**).

Chiral GC data for partially cyclized compound **5c**:

GC (chiral) (Cyclodextrin-H 0.25/0.125df G/632, 24 m, temperature: 220/ 60 iso 1000 min, 0.50 bar H_2, sample size: 1.0 µL): t_{R1} = 387.8 min (**5c**), t_{R2} = 397.5 min (**5c**), t_{R3} = 407.4 min (**5c**), t_{R4} = 422.0 min (**5c**).

1.5.4 Supplementary Discussion on the Ecological Properties of HFIP and PFTB

Data on the persistence, (bio)degradability, bioaccumulative potential, and the endocrine disrupting properties of 1,1,1,3,3,3-hexafluoropropan-2-ol (HFIP, CAS: 920-66-1) are available in the safety data sheet.[36] As expectable, HFIP was found to be not readily biodegradable after exposure to air for 28 days. The bioaccumulative potential was evaluated in Eurasian carps (*Cyprinus carpio*) with an aqueous solution of HFIP (1 mg/L) at 25 °C for 42 days. As opposed to other well-investigated perfluorinated alkyl carboxylic and sulfonic acids, the bioaccumulative potential was assessed to be low, based on a measured bioconcentration factor (BCF) between 1.1 and 1.4 (OECD test guideline 305).[36,37] According to guidelines and criteria installed by the United States Environmental Protection Agency (EPA) under the Toxic Substances Control Act (TSCA) or by the European Union under the REACH (Registration, Evaluation, Authorisation and Restriction of Chemicals) regulation, a substance is not considered to be bioaccumulative if its BCF is below 1000 (TSCA)[38,39] or 2000 (REACH).[40]

Similarly, the bioaccumulative potential of nonafluoro-*tert*-butyl alcohol (perfluoro-*tert* butyl alcohol, PFTB, CAS: 2378-02-1) was assessed to be low based on its octanol/water coefficient of log K_{OW} = 1.977.[41] Chemicals with a log K_{OW} >5 are considered to be bioaccumulative.[40]

With respect to the endocrine disrupting potential the following assessment is provided in the safety data sheet:[36] "The substance/mixture does not contain components considered to have endocrine disrupting properties according to REACH Article 57(f) or Commission Delegated regulation (EU) 2017/2100 or Commission Regulation (EU) 2018/605 at levels of 0.1% or higher." This stands in contrast to the well-documented endocrine disrupting potential of perfluoroalkyl carboxylic and sulfonic acids.[42] Currently available (eco)toxicological data suggests that HFIP and PFTB are not considered to be endocrine disruptors and bioaccumulative, as opposed to perfluoralkyl carboxylic and sulfonic acids. Nevertheless, the classification of HIFP and PFTB as very mobile (ionizable polar compounds) and very persistent compounds poses a significant hazard for the contamination of aquatic ecosystems and drinking water. It should be mentioned that both compounds are highly volatile (HFIP: b.p. = 59 °C, vapor pressure: ρ = 20.5 kPa at 298 K, 25 °C; PFTB: b.p. = 45 °C, vapor pressure: ρ = 35.8 kPa at 273 K, 20 °C) and are most likely removed from contaminated water through aeration during water treatment. However, based on the aforementioned available data, it should be clear that appropriate precautions and safety measures must be installed to avoid a release of either HFIP and PFTB into the environment. An effective solvent recycling strategy within a closed process should ideally minimize the release of perfluorinated alcohols into the environment and mitigate potential environmental hazards.

1.6 Polyene Cyclization of Homofarnesol Diastereomers

To probe the reactivity and selectivity of the catalyst towards other double bond isomers of **1a**, and to gain initial experimental insights into the reaction mechanism, all previously synthesized homofarnesol isomers were individually subjected to the reaction conditions in PFTB using (*S*,*S*)-IDPi **8g** as catalyst.

1.6.1 General Procedure

A 2 mL headspace screw-cap glass vial equipped with a PTFE-coated magnetic stir bar was charged with the respective homofarnesol isomer (28.4 mg, 0.12 mmol, 1.0 equiv.) and IDPi catalyst **8g** (2 mol%). The mixture was cooled to desired temperature and stirred for 10 min. Perfluoro-*tert*-butanol (PFTB, 100 µL, 1.25 M, 5.7 equiv.) was added and the reaction was stirred for 12 h. The resulting mixture was neutralized with triethylamine, the solvent was evaporated under reduced pressure and the residue was subjected to column chromatography on silica gel (hexanes/ethyl acetate 10:1 *v/v*). Typically, three components (cyclohomofarnesol **3**, partially cyclized products **5**, and tricyclic ethers **2**) were obtained. The enantiomeric ratio (e.r.) of **2** and **5** were determined by chiral GC (methods are provided below). The e.r. of **3a** and **3c** was determined by chiral HPLC.

GC methods for tricyclic ethers 2:

GC (achiral) (DB-5MS 0.25/0.25df, 30.0 m, temperature: 220/ 159 iso 50 min, 20/min 340 iso 5 min, 0.60 bar H_2, sample size: 0.2 µL): t_{R1} = 36.9 min (ambrox), t_{R2} = 39.0 min (5*β*,8*α*,9*β*-ambrox).

GC (chiral) (BGB 176 0.25/0.25df, 30.0 m, temperature: 220/ 150 iso 60 min, 20/min, 220 iso 5 min, 0.60 bar H_2, sample size: 0.2 µL): t_{R1} = 38.4 min (**2b**), t_{R2} = 39.1 min (*ent*-**2b**), t_{R3} = 39.9 min (**2d**), t_{R4} = 41.2 min (*ent*-**2d**), t_{R5} = 44.3 min (**2a**), t_{R6} = 46.0 min (*ent*-**2a**), t_{R1} = 47.8 min (**2d**), t_{R2} = 49.2 min (*ent*-**2d**).

GC methods for 5:

GC (achiral) (Stabilwax 0.25/0.25df, G/353 60.0 m, temperature: 220/60 1/min 150 10/min 260, 1.10 bar H_2, sample size: 0.2 µL); t_{R1} = 71.4 min (**5c**), t_{R2} = 71.6 min (**5c**), t_{R3} = 73.6 min (**5a**), t_{R4} = 74.1 min (**5a**), t_{R5} = 77.2 min (**5b**).

GC (chiral) (Lipodex-G 0.25/0.00df G/602, 25.0 m, temperature: 220/60 iso 1000 min, 0.50 bar H_2, sample size: 1 µL): t_{R1} = 529.0 min (**5c**), t_{R2} = 550.8 min (**5c**), t_{R3} = 567.3 (**5c**), t_{R4} = 601.3 min (**5a**), t_{R5} = 634.1 min (**5a**), t_{R6} = 693.3 min (**5a**), t_{R7} = 708.1 min (**5a**), t_{R8} = 739.3 min (**5b**), t_{R9} = 758.8 min (**5b**).

GC (chiral) (Cyclodextrin-H 0.25/0.125df G/632, 24.0 m, temperature: 220/ 60 iso 1000 min, 0.50 bar H_2, sample size: 1 µL): t_{R1} = 387.8 min (**5c**), t_{R2} = 397.5 min (**5c**), t_{R3} = 407.4 (**5c**), t_{R4} = 422.0 min (**5c**).

HPLC methods for 3:

Preparative HPLC separation conditions for **3a** and **3c**:

HPLC (achiral) (250 mm Multokrom 100-3 Si column, *n*-heptane/propan-2-ol = 99.5:0.5 *v/v*, flow rate = 1.0 mL/min, 16.5 MPa, 288 K, UV: λ = 205 nm); t_{R1} = 26.4 min (**3c**), t_{R2} = 27.1 min (**3a**).

After separation of the isomers on an achiral stationary phase, the enantiomeric excess of **3a** and **3c** can be determined by HPLC on a chiral stationary phase.

Analytical HPLC separation for **3a**:

HPLC (chiral) (150 mm Chiralcel IG-3 column. Conditions: *n*-heptane/propan-2-ol = 99.5:0.5, flow rate = 1.0 mL/min, 9.8 MPa, 288 K, UV: λ = 205 nm): t_{R1} = 9.31 min (**3a**), t_{R2} = 11.28 min (**3a**).

Analytical HPLC separation for **3c**:

HPLC (chiral) (2×150 mm Chiralcel OD-3 column. Conditions: *n*-heptane/2-propanol = 99.5:0.5, flow rate = 1.0 mL/min, 14.3 MPa, 288 K, UV: λ = 205 nm): t_{R1} = 24.79 min (**3c**), t_{R2} = 26.36 min (**3c**).

Alternatively, analytical separation of the isomeric mixture of **3a** and **3c** to determine the enantiomeric ratios can be performed by two-dimensional HPLC:

Analytical 2D-HPLC method for 3a: ^{1}**D** (1st dimension, achiral stationary phase): 100 mm VDSpher PUR 100 SIL, 4.6 mm i.D., *n*-heptane/propan-2-ol 95.5:0.5 *v/v*, 1.0 mL/min, 20.9 MPa, 288 K, UV: λ = 205 nm): t_R = 11.25–11.29 (sampling range). ^{2}**D** (2nd dimension, chiral stationary phase): 150 mm Chiralcel IG-3, *n*-heptane/propan-2-ol = 99.5:0.5 *v/v*, flow rate = 1.0 mL/min, 9.8 MPa, 288 K, UV-vis detection at λ = 205 nm, t_{R1} = 9.31 min (**3a**), t_{R2} = 11.28 min (**3a**).

2D-HPLC method for 3c: ^{1}**D** (1st dimension, achiral stationary phase): 100 mm VDSpher PUR 100 SIL, 4.6 mm i.D., *n*-heptane/propan-2-ol 95.5:0.5 *v/v*, 1.0 mL/min, 20.9 MPa, 288 K, UV: λ = 205 nm): t_R = 11.25–11.29 (sampling range). ^{2}**D** (2nd dimension, chiral stationary phase): (2 × 150 mm Chiralcel OD-3, 4.6 mm i.D. column 2+1, *n*-heptane/propan-2-ol 95.5:0.5 *v/v*, 1.0 mL/min, 14.5 MPa, 288 K, UV: λ = 205 nm): t_{R1} = 24.79 min (**3c**), t_{R2} = 26.36 min (**3c**).

Polyene cyclization of (3*E*,7*E*)-homofarnesol (1a)

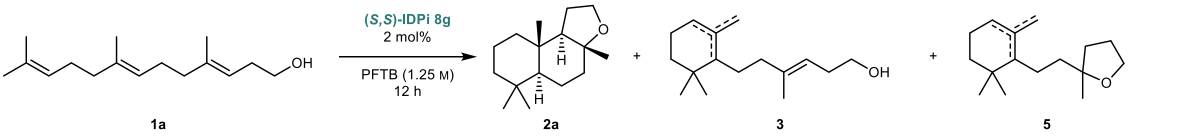

	2a	3	5
–40 °C<br>(9% **1a** left)	47% yield of **2a**<br>>20:1 d.r.<br>e.r. of **2a** 95:5	44% yield<br>**3a** (α):**3c** (γ)<br>ca. 84:16<br>**HPLC ratio**<br>**3a** e.r. 14:86<br>**3c** e.r.11:89	–
–30 °C	50% yield of **2a**<br>>20:1 d.r.<br>e.r. of **2a** 94:6	19% yield<br>**3a** (α):3b (β):**3c** (γ)<br>ca. 14:2:1<br>**HPLC ratio**<br>**3a** e.r. 27:73<br>**3c** e.r. 36:64	31% yield<br>**5a** (α):**5c** (γ)<br>ca. 29:2<br>**GC ratio**<br>**5a** 25:75 d.r., 3:97 e.r. (major), 94:6 e.r. (minor)<br>**5b** 8:92 e.r.<br>**5c** 67:33 d.r., 6:94 e.r. (major), 13:87 e.r. (minor)

Fig. S23 | Catalytic asymmetric polyene cyclization of (3*E*,7*E*)-homofarnesol (**1a**) in PFTB.

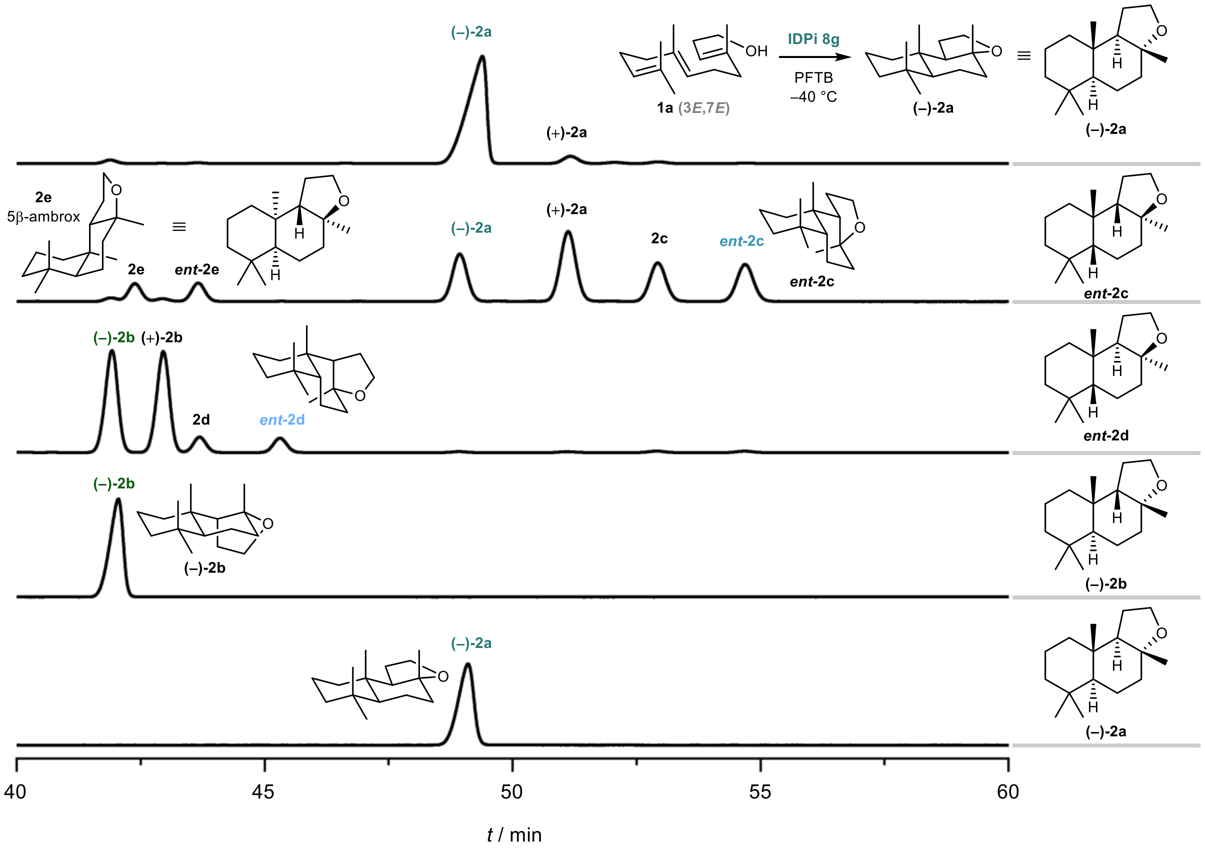

Fig. S24 | Excerpts of stacked GC traces showing the retention time range of the tricyclic ether products. The crude reaction mixture of the catalytic asymmetric polyene cyclization of (3*E*,7*E*)-homofarnesol (**1a**) in PFTB (first GC trace) is compared with authentic standards of the respective ambrox diastereomers (GC traces 2–5).

Polyene cyclization of (3*Z*,7*E*)-homofarnesol (1b)

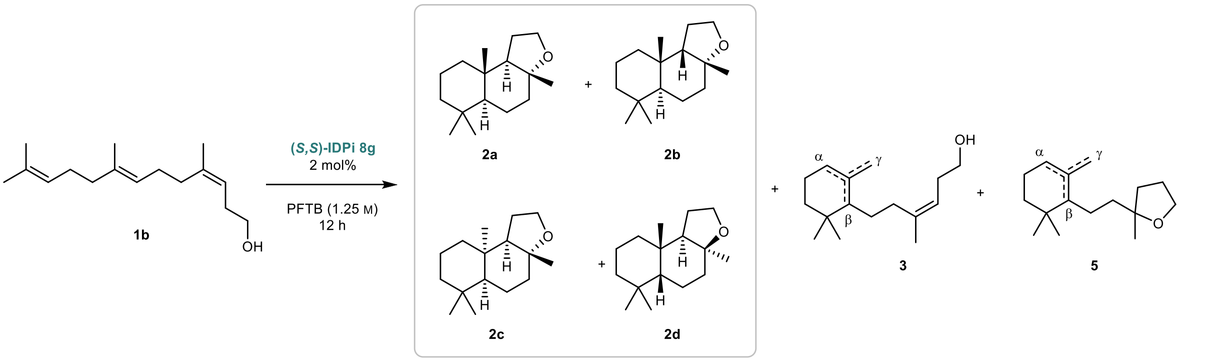

	2	3	5
–40 °C	34% yield of **2b**<br>>20:1 d.r.<br>e.r. of **2b** 93:7	46% yield<br>**3a** (α):**3b** (β):**3c** (γ)<br>ca. 30:9:7<br>**HPLC ratio**<br>n.d.	19% yield<br>**5a** (α):**5b** (β):**5c** (γ)<br>ca. 10:4:5<br>**GC ratio**<br>**5a**: 29:71 d.r., 18:82 e.r. (major), 82:18 e.r. (minor)<br>**5b**: 15:85 e.r.<br>**5c**: 55:45 d.r., 3:97 e.r. (major), 61:39 e.r. (minor)
–30 °C	48% yield of **2b**<br>**2a**:**2b**:**2d** 6:91:3 d.r.<br>e.r. of **2a** 87:13<br>e.r. of **2b** 91:9<br>e.r. of **2d** 73:27	–	51% yield<br>**5a** (α):**5b** (β):**5c** (γ)<br>ca. 34:15:2<br>**GC ratio**<br>**5a**: 29:71 d.r., 17:83 e.r. (major), 83:17 e.r. (minor)<br>**5b**: 14:86 e.r.<br>**5c**: 63:37 d.r., 5:95 e.r. (major), 37:63 e.r. (minor)

Fig. S25: Catalytic asymmetric polyene cyclization of (3*Z*,7*E*)-homofarnesol (**1b**) in PFTB.

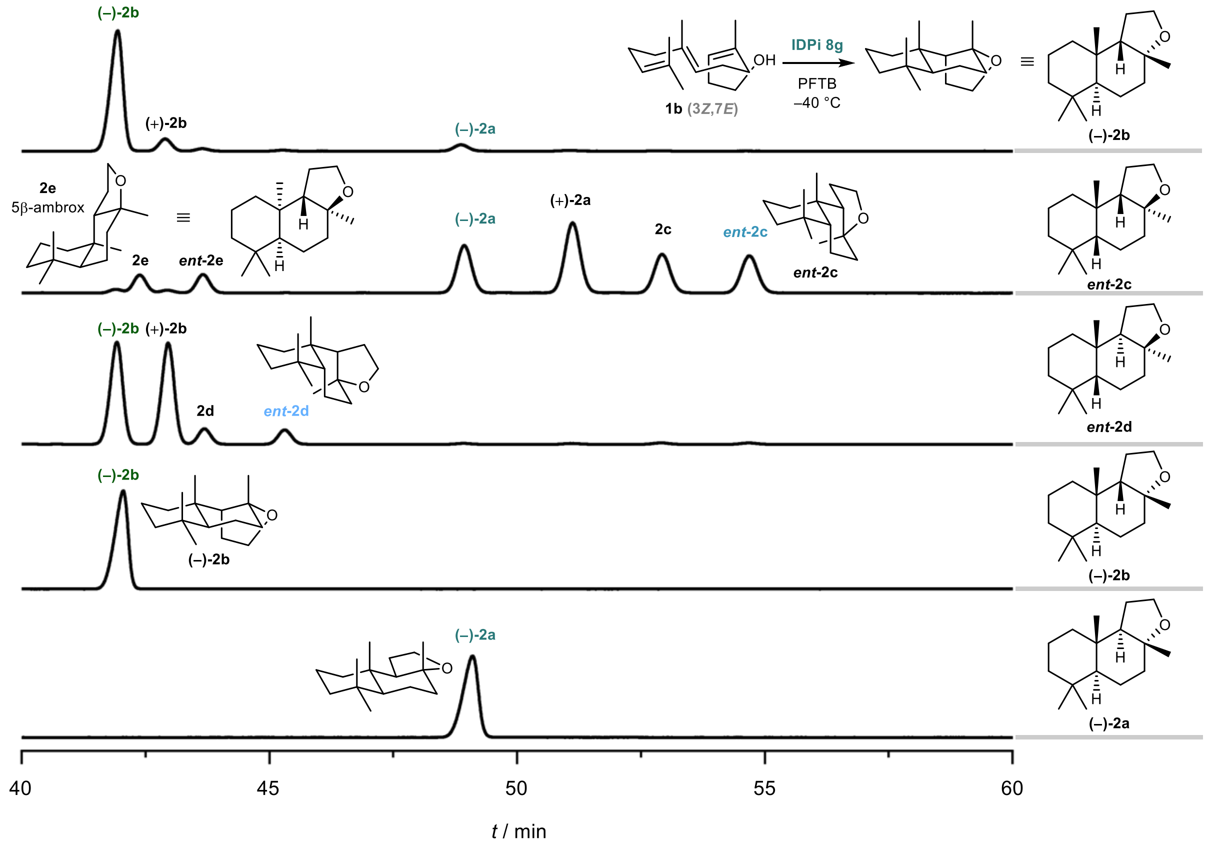

Fig. S26 | Excerpts of stacked GC traces showing the retention time range of the tricyclic ether products. The crude reaction mixture of the catalytic asymmetric polyene cyclization of (3*Z*,7*E*-homofarnesol) (**1b**) in PFTB (first GC trace) is compared with authentic standards of ambrox diastereomers (GC traces 2–5).

Polyene cyclization of (3*E*,7*Z*)-homofarnesol (1c)

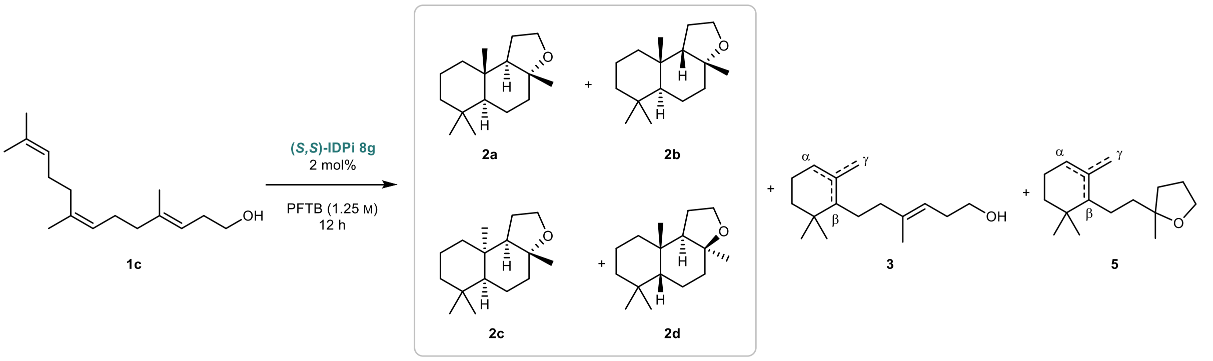

	2a–2d	3	5
–40 °C	–	–	–
–30 °C (13% **1c** left)	29% yield of **2c**<br>**2a**:**2b**:**2c**:**2d** 13:2:66:19<br>e.r. of **2a** 70:30<br>e.r. of **2b** 77:23<br>e.r. of **2c** 22:78<br>e.r. of **2d** 35:65	21% yield<br>**3a** (α):**3c** (γ)<br>ca. 81:19<br>**HPLC ratio**<br>**3a** 61:39 e.r.<br>**3c** 68:32 e.r.	51% yield<br>**5a** (α):**5b** (β):**5c** (γ)<br>ca. 34:15:2<br>**GC ratio**<br>**5a** 58:42 d.r., 6:94 e.r. (minor), 95:5 e.r. (major)<br>**5b** 1:99 e.r.<br>**5c** 35:65 d.r., 11:89 e.r. (minor), 6:94 e.r. (major)

Fig. S27: Catalytic asymmetric polyene cyclization of (3*E*,7*Z*)-homofarnesol **1c** in PFTB.

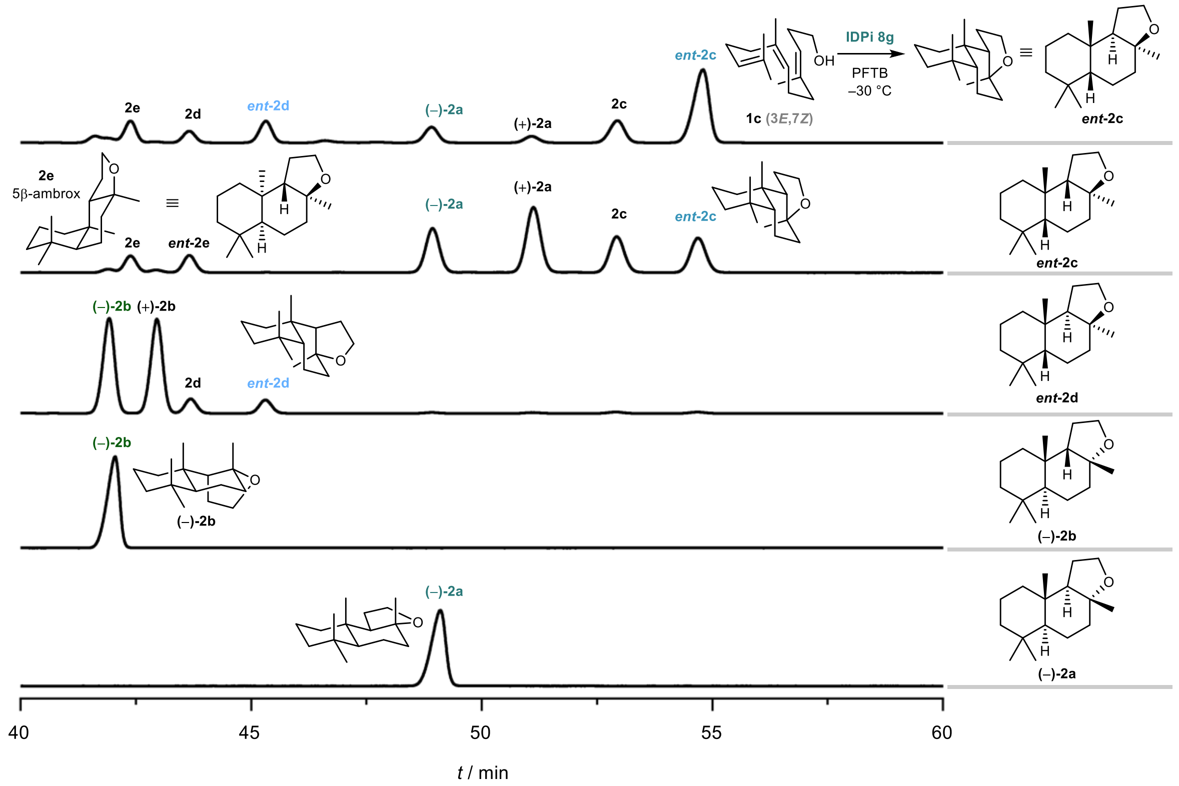

Fig. S28 | Excerpts of stacked GC traces showing the retention time range of the tricyclic ether products. The crude reaction mixture of the catalytic asymmetric polyene cyclization of (3*E*,7*Z*-homofarnesol) (**1c**) in PFTB (first GC trace) is compared with authentic standards of the respective ambrox diastereomers (GC traces 2–5).

Polyene cyclization of (3*Z*,7*Z*)-homofarnesol (1d)

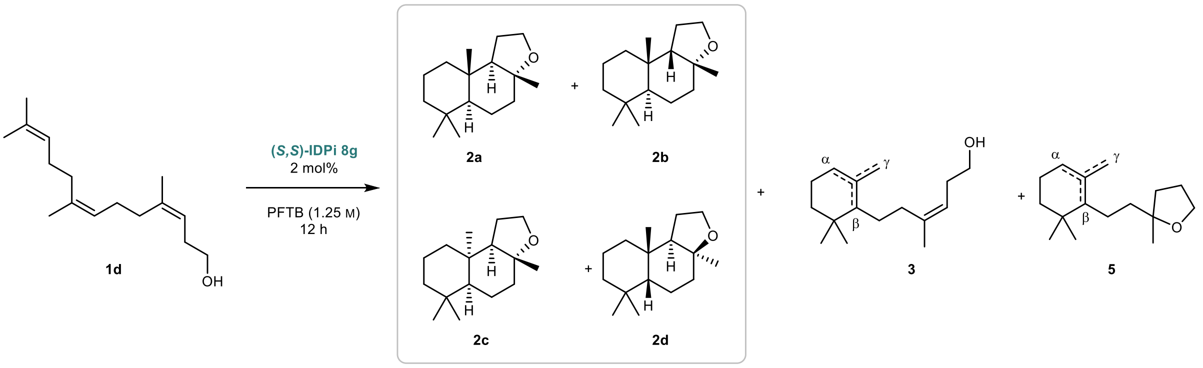

–40 °C	–	–	–
–30 °C<br>(6% **1d** left)	29% yield of **2d**<br>**2b**:**2c**:**2d** 12:2:86<br>e.r. of **2b** 80:20<br>e.r. of **2c** 32:68<br>e.r. of **2d** 19:81	25% yield<br>**3a** (α)<br>**HPLC ratio**<br>n.d.	37% yield<br>**5a** (α):**5b** (β):**5c** (γ)<br>ca. 25:6:6<br>**GC ratio**<br>**5a** 60:40 d.r., 22:78 e.r. (minor), 82:18 e.r. (major)<br>**5b** 27:73 e.r.<br>**5c** 31:69 d.r., 25:75 e.r. (minor), 14:86 e.r. (major)

Fig. S29 | Catalytic asymmetric polyene cyclization of (3*Z*,7*Z*)-homofarnesol (**1d**) in PFTB.

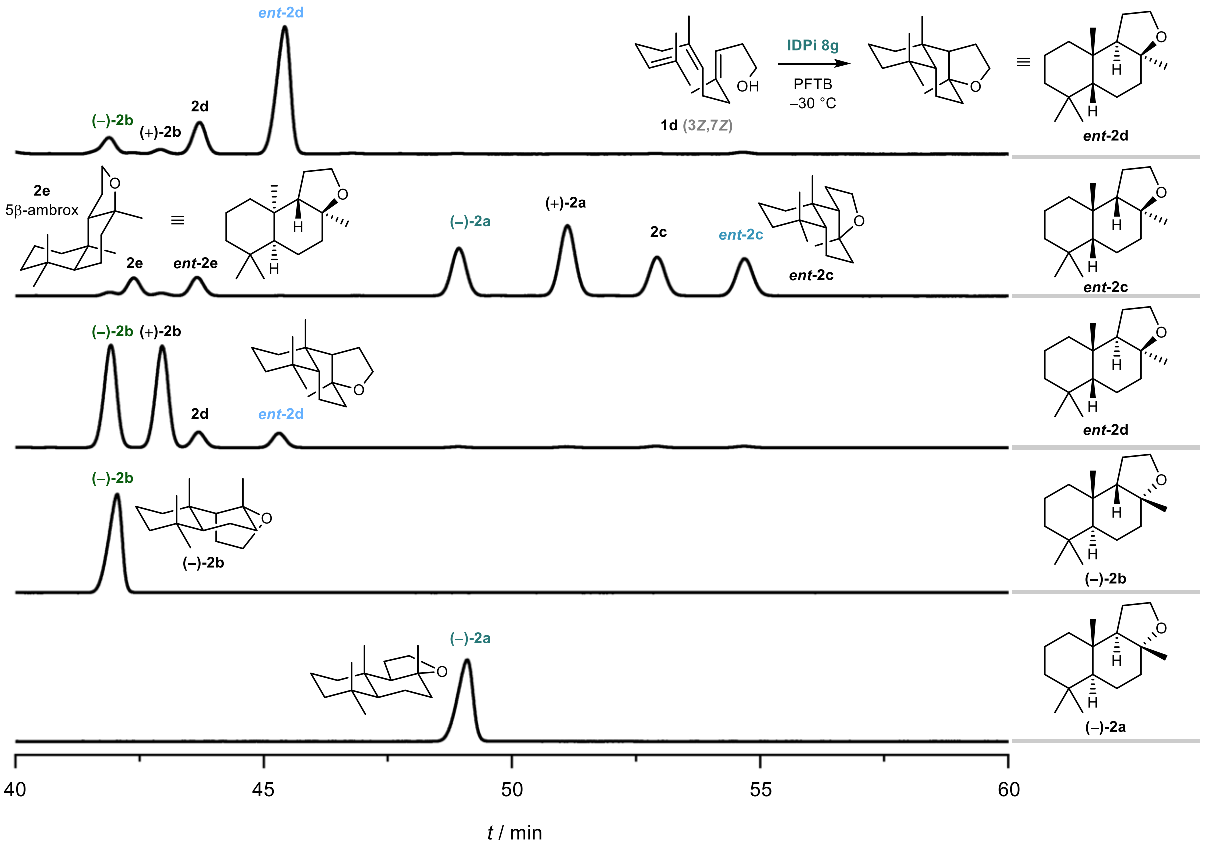

Fig. S30 | Excerpts of stacked GC traces showing the retention time range of the tricyclic ether products. The crude reaction mixture of the catalytic asymmetric polyene cyclization of (3*Z*,7*Z*)-homofarnesol (**1d**) in PFTB (first GC trace) is compared with authentic standards of ambrox diastereomers (GC traces 2–5).

Summary of the results obtained for the individual isomers

The respective major product obtained for each possible diastereomer of homofarnesol (**1**) in the presence of IDPi catalyst **8g** under the reaction conditions in PFTB at low temperatures (−40 °C ≤ T ≤ −30 °C) can be rationalized with the corresponding Stork–Eschenmoser transition state (see Fig. S31). This observation is in line with a predominantly concerted polyene cyclization.

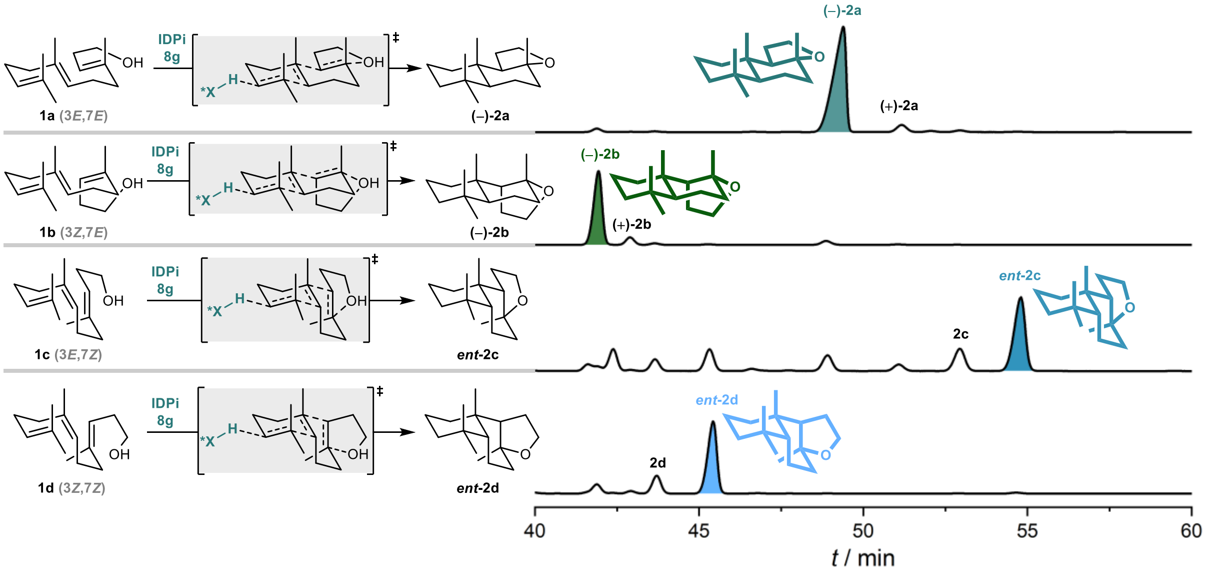

Fig. S31 | Summary of the IDPi-catalyzed polyene cyclizations of homofarnesol diastereomers in PFTB. The major product diastereomer is highlighted in each case.

It is worth mentioning that the slightly diminished selectivity observed for the (3*E*,7*Z*)-isomer (**1c**) is also a consequence of the lower purity of the starting material (due to difficulties in the separation of the isomers). The observed d.r. (82:18) of 5β,8α,9β-ambrox (**2c**) to 5β,8α-ambrox (**2d**) approximately reflects the isomeric ratio (91:9) of the corresponding starting materials (3*E*,7*Z*)-homofarnesol (**1c**) and (3*Z*,7*Z*)-homofarnesol (**1d**).

1.6.2 Synthesis of (−)-9-*epi*-Ambrox

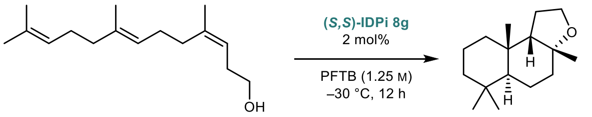

1b (–)-2b

A 2 mL headspace screw-cap glass vial equipped with a PTFE-coated magnetic stir bar was charged with (3*Z*,7*E*)-homofarnesol (28.4 mg, 0.12 mmol, 1.0 equiv.) and IDPi catalyst **8g** (2 mol%). The mixture was cooled to desired temperature and stirred for 10 min. Perfluoro-*tert*-butanol (PFTB, 100 µL, 1.25 M, 5.7 equiv.) was added and the reaction was stirred for 12 h. The resulting mixture was neutralized with triethylamine, the solvent was evaporated under reduced pressure and the residue was purified by flash column chromatography on silica gel (hexanes/ethyl acetate 10:1 *v/v*) to afford (−)-9-*epi*-ambrox (13.6 mg, 57.6 µmol, 48%, 91:9 d.r., 91:1 e.r.) as a colorless oil. The identity of (−)-9-*epi*-ambrox was confirmed by comparison of the GC retention time and the ^{13}C NMR spectrum (Fig. S32) with a reference standard, previously synthesized from commercially available (+)-sclareolide (see section 1.3).

^1H NMR (501 MHz, $CDCl_3$): δ (ppm) = 3.85 (ddd, *J* = 9.5, 8.3, 3.1 Hz, 1H), 3.77 (q, *J* = 8.4 Hz, 1H), 2.04 (tt, *J* = 12.2, 9.1 Hz, 1H), 1.97–1.86 (m, 1H), 1.74–1.60 (m, 1H), 1.59–1.52 (m, 4H), 1.43–1.38 (m, 2H), 1.37 (s, 3H), 1.31–1.22 (m, 3H), 1.19 (dd, *J* = 12.2, 1.5 Hz, 1H), 1.17–1.11 (m, 1H), 1.10 (s, 3H), 0.89 (s, 3H), 0.82 (s, 3H).

$^{13}C\{^1H\}$ NMR (126 MHz, $CDCl_3$): δ (ppm) = 81.0, 64.2, 59.2, 46.9, 42.5, 38.8, 36.2, 35.9, 33.7, 33.1, 29.0, 27.9, 23.0, 21.9, 20.6, 18.7.

HRMS (GC-EI^+): *m/z* calcd. for $C_{16}H_{28}O$ $[M]^+$: 236.213465, found: 236.213550.

GC (achiral) (Optima-35 0.25/0.25df G/706, 29.0 m; temperature: 220/50 5/min 200 12/min 350, 5 min iso/ 350, 0.60 bar H_2, sample size: 0.2 µL, split ratio: 80:1): 9-*epi*-ambrox: t_R(**2b**) = 26.76 min (91.32%), 5β-ambrox: t_R(**2e**) = 26.98 min (0.06%), 5β,8α-ambrox: t_R(**2d**) = 27.04 min (2.51%), ambrox: t_R(**2a**) = 27.48 min (5.80%), 5β,8α,9β-ambrox: t_R(**2c**) = 27.96 min (0.31%).

Diastereomeric ratio: **2b** : **2a** : **2d** : **2c** : **2e** = 91.32 : 5.81 : 2.51 : 0.31 : 0.06; >10:1 d.r. (**2b**).

GC (chiral) (BGB 176/BGB-15 0.25/0.25df G/618, 30.0 m; temperature: 220/140, 60 min iso 8/min 240/ 350, 0.60 bar H_2, sample size: 0.2 µL): (−)-9-*epi*-ambrox: t_R(**2b**) = 41.95 min (82.72%), (+)-9-*epi*-ambrox, t_R(**2b**) = 42.90 min (8.40%), 5β,8α-ambrox: t_R(**2d**) = 43.66 min (1.83%), *ent*-5β,8α-ambrox: t_R(**2d**) = 45.29 min (0.68%), (−)-ambrox: t_R(**2a**) = 48.86 min (5.22%), (+)-ambrox: t_R(**2a**) = 51.05 min (0.77%), 5β,8α,9β-ambrox: t_R(**2c**) = 52.85 min (0.24%), *ent*-5β,8α,9β-ambrox: t_R(**2c**) = 54.64 min (0.14%).

Enantiomeric ratios of **2a**, **2b** and **2c**: (−)-ambrox (**2a**): e.r. = 95:5; (−)-9-*epi*-ambrox (**2b**): e.r. = 91:9; 5β,8α,9β-ambrox: (**2c**): e.r. = 79:21.

$[\alpha]_D^{25}$ = −4.0 (*c* = 0.35, $CHCl_3$; 91:9 d.r. and 91:9 e.r.); Lit.: $[\alpha]_D^{25}$ = −6.0 (*c* = 0.98, $CHCl_3$).[27]

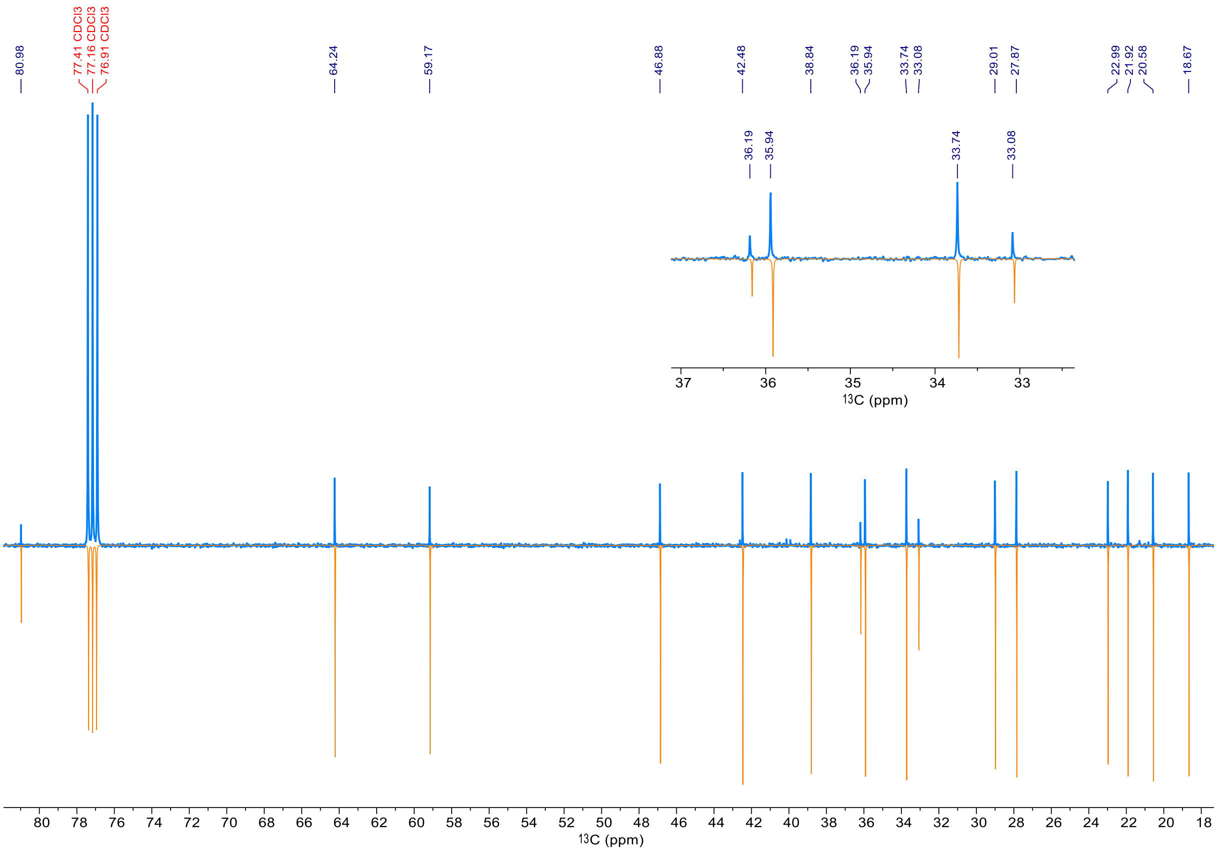

Fig. S32 | Comparison of the ^{13}C NMR (501 MHz, $CDCl_3$) spectra of synthetic (−)-9-*epi*-ambrox (**2b**, top, blue) obtained via the IDPi-catalyzed asymmetric polyene cyclization with an authentic sample of **2b** (bottom, orange) prepared via reduction and cyclization of enantiopure (−)-9-*epi*-sclareolide.

1.7 Polyene Cyclization of Technical (3*E/Z*,7*E*)-Homofarnesol (1a/1b)

To further probe the applicability of the IDPi-catalyzed asymmetric polyene cyclization, a mixture of technical homofarnesol isomers was subjected to the reaction conditions. The required starting material is readily available from (*E*)-nerolidol via a palladium-catalyzed low pressure carbonylation. A subsequent reduction of the initially obtained homofarnesic acid isomers (3*E/Z* ca. 60:40) provides the starting material (3*E/Z*,7*E*)-homofarnesol (*E/Z* ca. 60:40).

Synthesis of (3*E/Z*,7*E*)-homofarnesol (1a/1b)

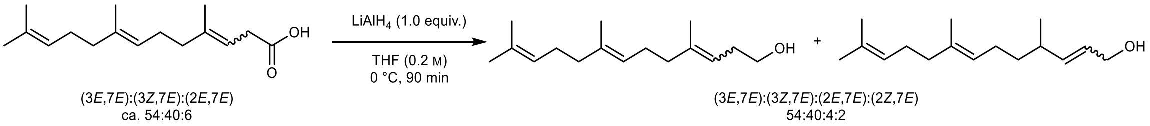

An isomeric mixture of homofarnesic acid (obtained via low-pressure carbonylation of nerolidol;[16] 3*E*,7*E*:3*Z*,7*E*:2*E*,7*E* ca. 54:40:6; 1.34 mL, 1.25 g, 5.00 mmol, 1.0 equiv.) was transferred to a flame-dried 100 mL Schlenk flask equipped with a rubber septum under argon. The starting material was dissolved in anhydrous THF (20 mL) and the resulting colorless solution was cooled to 0 °C using an ice/water bath. $LiAlH_4$ (1 M solution in THF, 5.00 mL, 5.00 mmol, 1.0 equiv.) was added dropwise over 5 min. The reaction mixture was stirred for 90 min at 0 °C. After the elapsed time, ^{1}H NMR spectrosocpy and TLC (hexanes/ethyl acetate 4:1 *v/v*) indicated complete consumption of the starting material and formation of a less polar product. A Fieser workup was performed:[17] the reaction mixture was diluted with MTBE (20 mL), 0.2 mL water was added carefully at 0 °C, followed by 0.2 mL 15% aqueous NaOH and 0.6 mL water, and the resulting cloudy solution was stirred at room temperature for 30 min. After the elapsed time, anhydrous $MgSO_4$ was added, and the suspension was stirred for additional 15 min. The suspension was filtered over a pad of Na_2SO_4/Celite® 545, and concentrated under reduced pressure to afford the crude product as an oil. Purification by flash column chromatography on silica gel using hexanes/MTBE as eluent (isocratic elution, 4:1 *v/v*) afforded an isomeric mixture of homofarnesols (major isomers: **1a/1b**) as a colorless oil (3*E*,7*E*:3*Z*,7*E*:2*E*,7*E*:2*Z*,7*E* ca. 54:40:4:2; 1.00 g, 4.23 mmol, 85% yield).

TLC (SiO_2, hexanes/MTBE 4:1 *v/v*): R_f (3*Z*,7*E*) = 0.31, R_f (3*E*,7*E*) = 0.25 (CAM stain).
NMR data for (3*E*,7*E*:3*Z*,7*E*)-homofarnesol with 3*E*:3*Z* ca. 57:43.
^{1}H NMR (501 MHz, $CDCl_3$): δ (ppm) = 5.23–5.02 (m, 3H), 3.61 (q, *J* = 6.1 Hz, 2H), 2.29 (q, *J* = 6.8 Hz, 2H), 2.14–2.01 (m, 6H), 1.98 (dd, *J* = 9.2, 6.1 Hz, 2H), 1.73 (q, *J* = 1.3 Hz, 1H; 3*Z*,7*E*-isomer), 1.68 (s, 3H), 1.65 (d, *J* = 1.3 Hz, 2H; 3*E*,7*E*-isomer), 1.62–1.58 (m, 6H), 1.40–1.33 (m, 1H, OH).
^{13}C NMR (126 MHz, $CDCl_3$): δ (ppm) = 139.1, 139.1, 135.6, 135.4, 131.5, 131.5, 127.4, 124.5, 124.5, 124.1, 124.0, 120.8, 120.0, 64.1, 62.8, 62.6, 39.9, 39.9, 39.9, 37.2, 37.0, 36.0, 32.1, 31.7, 31.6, 26.9, 26.8, 26.7, 26.6, 25.8, 25.8, 23.7, 17.8, 16.4, 16.2, 16.1.

Diagnostic peaks for (2*E*,7*E*)-homofarnesol with 2*E*:2*Z* ca. 66:34:

^{1}H NMR (501 MHz, $CDCl_3$): δ (ppm) = 5.66–5.52 (m, 2H), 4.10 (t, *J* = 4.9 Hz, 2H), 0.99 (d, *J* = 6.8 Hz, 3H), 0.89 (d, *J* = 6.6 Hz, 2H).

GC Data for (3*E*,7*E*:3*Z*,7*E*)-homofarnesol with 3*E*:3*Z* ca. 57:43:

GC (achiral) (Optima-35 0.25/0.25df G/706, 29.0 m, temperature: 220/ 60 5/min to 300/350, 0.60 bar H_2, sample size: 0.2 μ, split ratio: 60:1): $t_{R(2E,7E)}$ = 25.18 min (3.47%), $t_{R(2Z,7E)}$ = 25.55 min (1.79%), $t_{R(3Z,7E)}$ = 25.71 min (39.74%), $t_{R(3E,7E)}$ = 26.00 min (53.70%).

Polyene cyclization of technical (3*E*/*Z*,7*E*)-homofarnesol (1a/1b)

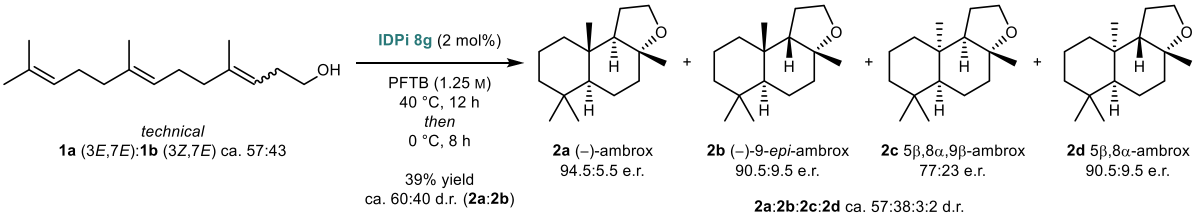

A 100 mL Schlenk flask under argon equipped with a PTFE-coated magnetic stir bar was charged with a technical mixture of (3*E*/*Z*,7*E*)-homofarnesol (**1a**:**1b** = 57:43, 386 mg, 1.63 mmol, 1.0 equiv.) and IDPi catalyst (83.0 mg, 33 µmol, 2 mol%). The mixture was cooled to −40 °C and stirred for 10 min. PFTB (1.3 mL, 5.7 equiv. 1.25 M) was added and the reaction was stirred for 12 h until full consumption of the starting material was observed as indicated by TLC. Then, the temperature was raised to 0 °C and the reaction was stirred for another 8 h. After the elapsed time, triethylamine was added, the solvent was evaporated under reduced pressure, and the residue was subjected to column chromatography on silica gel (gradient elution with hexanes/ethyl acetate 50:1 → 4:1 *v/v*) to give a mixture of diastereomers predominantly containing (−)-ambrox **2a** and (−)-9-*epi* ambrox **2b** (150 mg, 0.64 mmol, 39% yield, **2a**:**2b**:**2c**:**2d** ca. 57:38:3:2 d.r.; 94.5:5.5 e.r. for **2a**, 90.5:5.5 e.r. for **2b**, 77:23 e.r. for **2c**, and 90.5:9.5 e.r. for **2d**). Notably, minor amounts of 2*E*,7*E*/2*Z*,7*E*-homofarnesol (ca. 6%) present in the starting material were found to be unreactive under the reaction conditions.

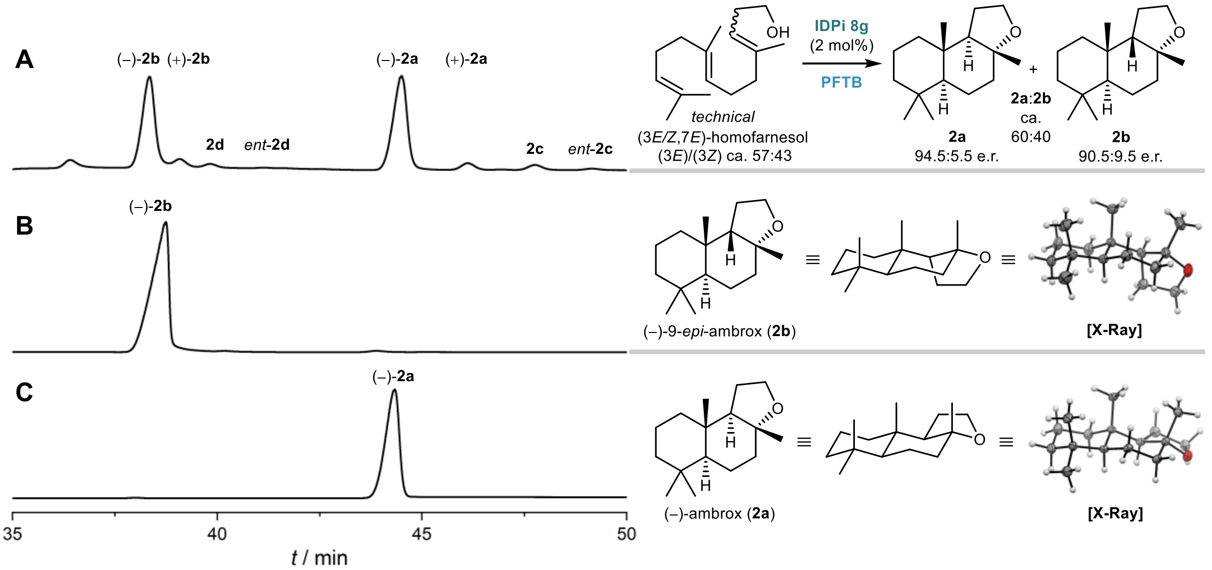

Fig. S33 | A, Excerpt of the GC trace from the reaction mixture obtained with technical (3*E*/*Z*,7*E*)-homofarnesol showing the tricyclic ether retention time region. **B**, GC traces of pure (−)-9-*epi*-ambrox (**2b**) as reference. **C**, GC traces of pure (−)-ambrox (**2a**) as reference.

The result also provides tentative evidence for a concerted reaction pathway, as the diastereomeric ratio of the starting material (**1a**:**1b** ca. 57:43) is approximately reflected in the diastereomeric ratio of the tricyclic ether products **2a**, (−)-ambrox and **2b**, (−)-9-*epi*-ambrox which were obtained with a d.r. of approximately 60:40 (**2a**:**2b**) according to ^{1}H NMR spectroscopic analysis.

1.8 Catalytic Asymmetric Polyene Cyclization towards Sclareolide

1.8.1 Synthesis of (3*E*,7*E*)-Homofarnesic Acid

The synthesis of (3*E*,7*E*)-homofarnesic acid (**11**) was accomplished by saponification of its respective isopropyl ester which was synthesized according to literature.[16]

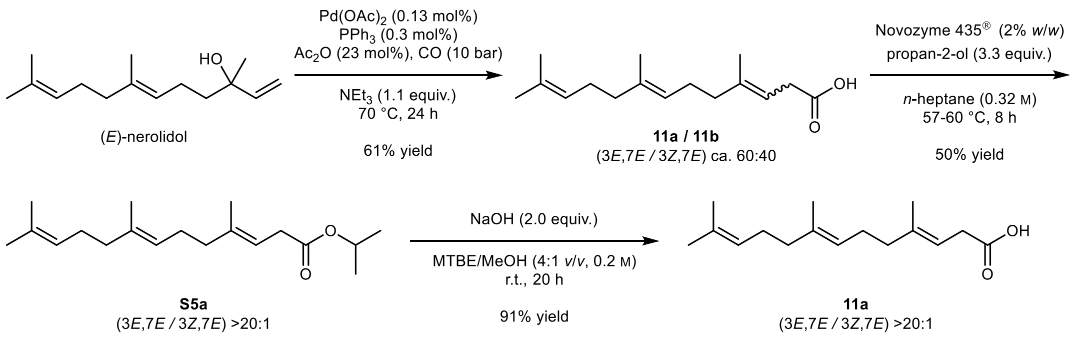

A 500 mL two-neck flask equipped with a PTFE-coated magnetic stir bar was charged with isopropyl (3*E*,7*E*)-4,8,12-trimethyltrideca-3,7,11-trienoate (**S5a**, 11.7 g, 40.0 mmol, 1.0 equiv.) and the reaction vessel was evacuated and flushed with argon (3 ×). The starting material was dissolved in MTBE (160 mL) and a freshly prepared solution of NaOH (3.20 g, 80.0 mmol, 2.0 equiv.) in methanol (40 mL, 2 M) was added. The resulting yellow solution was stirred at room temperature for 20 h. Analysis of the reaction mixture by thin-layer chromatography (hexanes/ethyl acetate 4:1 *v/v*, CAM stain) after 20 h reaction time indicated full conversion of the starting material. The yellow solution was concentrated under reduced pressure (150 mbar, 40 °C) to afford a yellow solid residue. Water (160 mL) was transferred to the mixture and the resulting pale yellow soapy solution was cooled to 0 °C (ice bath). Then, aqueous HCl (10 wt% in H_2O, 30 mL) was added carefully until the aqueous solution was adjusted to a pH of 1-2 (checked with universal indicator paper). Upon addition of the aqueous HCl, the formation of a colorless precipitate was observed. MTBE (100 mL) was transferred to the flask and the organic phase was separated. The aqueous phase was extracted with MTBE (4× 100 mL), the combined organic layers were dried over anhydrous sodium sulfate, and concentrated under reduced pressure to afford the crude product as a yellow oil. Purification by flash column chromatography on silica gel (gradient elution with hexanes/ethyl acetate; 1:0 → 19:1 → 9:1 → 4:1 *v/v*) afforded (3*E*,7*E*)-homofarnesic acid **11** (9.07 g, 36.2 mmol, 91% yield, >20:1 *E/Z*) as a colorless oil.

Physical state: colorless oil.

TLC (SiO_2, hexanes/EtOAc 4:1, *v/v*): $R_f = 0.31$ (bromocresol green or CAM stain).

^{1}H NMR (501 MHz, $CDCl_3$): δ (ppm) = 5.32 (tq, J = 7.1, 1.4 Hz, 1H), 5.09 (tdq, J = 5.5, 4.2, 1.3 Hz, 2H), 3.09 (d, J = 7.1 Hz, 2H), 2.14–2.03 (m, 6H), 1.97 (dd, J = 9.1, 6.1 Hz, 2H), 1.68 (q, J = 1.3 Hz, 3H), 1.65 (q, J = 1.3 Hz, 3H), 1.60 (s, 6H).

^{13}C{^{1}H} NMR (126 MHz, $CDCl_3$): δ (ppm) = 177.7, 140.0, 135.5, 131.5, 124.5, 123.9, 115.1, 39.8, 39.7, 33.5, 26.9, 26.5, 25.8, 17.8, 16.5, 16.2.

HRMS (GC-EI$^+$): *m/z* calcd. for $C_{16}H_{26}O_2$ $[M]^+$: 250.192730, found: 250.192700.

1.8.2 Polyene Cyclization of (3*E*,7*E*)-Homofarnesic Acid to Sclareolide

Cyclization at −30 °C:

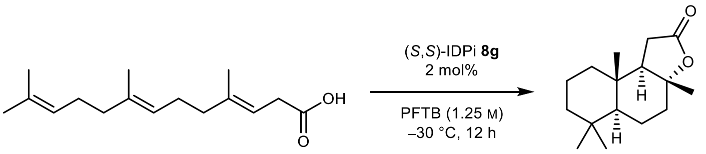

11a
>20:1 *E* / *Z*

12a
30% yield
>20:1 d.r., 95:5 e.r.

A screw-cap vial was charged with a magnetic stir bar, IDPi catalyst **8g** (2 mol%), and perfluoro-*tert*-butanol (PFTB, 20 µL, 5.7 equiv.) was added. The reaction solution was cooled to −30°C and stirred for 10 min. Neat (3*E,*7*E*)-homofarnesic acid (**11a**, 25.0 µmol, 1.0 equiv.) was added to the reaction vial, and the reaction was stirred at −30 °C for 12 hours. The reaction mixture was treated with triethylamine and slowly warmed to room temperature, followed by evaporation of the solvent under reduced pressure. The yield of sclareolide and diastereomeric ratio (d.r.) were determined by ^{1}H NMR using CH_2Br_2 as internal standard. The enantiomeric ratio (e.r.) of **12a** was determined by gas chromatography on a chiral stationary phase.

GC (achiral) (DB-5MS 0.25/0.25df, 30.0 m, temperature: 220/ 50 iso 5 min, 15/min, 340 iso 5 min, 0.60 bar H_2, sample size: 0.2 µL): sclareolide: t_R(**12a**) = 19.8 min, 5β,8α,9β-sclareolide: t_{R2}(**12c**) = 19.9 min.

GC (chiral) (Ivadex-1 0.25/0.25df, 30.0 m, temperature: 220/180 0.5/min 200, 10/min, 220 iso 5 min, 0.60 bar H_2, sample size: 0.2 µL) (+)-sclareolide: t_R = 36.8 min (**12a**, major, 95%), (−)-sclareolide: t_R = 37.4 min (*ent*-**12a**, minor, 5%); e.r. (**12a**) = 95:5.

Cyclization at −30 °C → 0 °C:

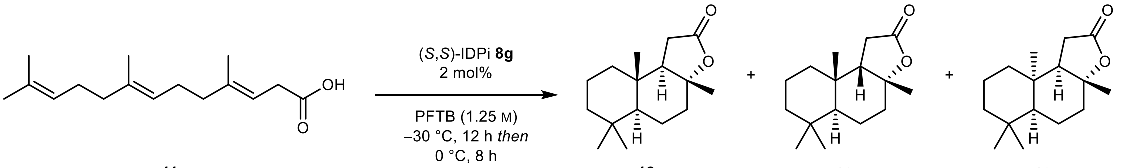

11a
>20:1 *E* / *Z*

12a
(+)-sclareolide
46% yield
>7:1 d.r., 94:6 e.r.

12b
(−)-9-*epi*-sclareolide
91.5:8.5 e.r.

12c
5β,8α,9β-sclareolide
76:24 e.r.

A screw-cap vial was charged with a magnetic stir bar and IDPi catalyst **8g** (2 mol%). Perfluoro-*tert*-butanol (PFTB, 20 μL, 5.7 equiv., 1.25 M) was added and the reaction solution was cooled to the reaction temperature of −30 °C and stirred for 10 min. Neat (3*E*,7*E*)-homofarnesic acid (**11a**, 6.26 mg, 25.0 μmol, 1.0 equiv.) was added to the reaction vial, and the reaction was stirred at −30 °C for 12 hours and at 0 °C for another 8 hours. The reaction mixture was treated with triethylamine and slowly warmed to room temperature, followed by evaporation of the solvent under reduced pressure. Purification by flash column chromatography on silica gel (gradient elution with hexanes/ethyl acetate 19:1 → 4:1 *v/v*) afforded the title compound as a colorless solid (2.88 mg, 11.5 μmol, 46% yield, >7:1 d.r., 94:6 e.r.). The diastereomeric ratio (d.r.) and enantiomeric ratio (e.r.) of lactone natural product **12a** were determined by gas chromatography on an achiral and chiral stationary phase, respectively.

Physical state: colorless solid.

^{1}H NMR (501 MHz, $CDCl_3$): δ (ppm) = 2.44–2.36 (m, 1H), 2.23 (dd, *J* = 16.2, 6.5 Hz, 1H), 2.07 (dt, *J* = 11.9, 3.3 Hz, 1H), 1.97 (dd, *J* = 14.8, 6.5 Hz, 1H), 1.88 (dq, *J* = 14.0, 3.2 Hz, 1H), 1.74–1.61 (m, 2H), 1.49–1.36 (m, 4H), 1.33 (s, 3H), 1.20 (td, *J* = 13.5, 4.3 Hz, 1H), 1.04 (ddd, *J* = 15.3, 13.1, 7.0 Hz, 2H), 0.91 (s, 3H), 0.88 (s, 3H), 0.84 (s, 3H).

$^{13}C\{^1H\}$ NMR (126 MHz, $CDCl_3$): δ (ppm) = 177.0, 86.5, 59.3, 56.8, 42.3, 39.7, 38.9, 36.2, 33.32, 33.27, 28.9, 21.7, 21.1, 20.7, 18.2, 15.2.

HRMS (GC-EI$^+$): *m/z* calcd. for $C_{16}H_{26}O_2$ $[M]^+$: 250.192730, found: 250.192630.

GC (achiral) (FFAP 0.25/0.25df G/396, 15.0 m; temperature: 220/60 5/min 250 5 min iso/350, 0.50 bar H_2, sample size: 1.0 μL): 9-*epi*-sclareolide: t_R(**12b**) = 28.12 min (0.95%), sclareolide t_R(**12a**) = 28.33 min (83.13%), 5β,8α,9β-sclareolide: t_R(**12c**) = 28.79 min (10.24%).

Diastereomeric ratio: **12a** : **12b** : **12c** = 88:11:1; >7:1 d.r. (**12a**).

GC (chiral) (Hydrodex-β-TBDAc-CD 0.25/0.25df G/681, 25.0 m; temperature: 220/80, 0.8/min 200 10/min 220 5 min iso/350, 0.60 bar H_2, sample size: 1.0 μL, split ratio: 20:1): (+)-9-*epi*-sclareolide: t_R(**12b**) = 134.96 min (0.26%), (+)-sclareolide, t_R(**12a**) = 137.21 min (81.14%), (−)-9-*epi*-sclareolide: t_R(**12b**) = 137.83 min (2.80%), 5β,8α,9β-sclareolide: t_R(**12c**) = 139.94 (8.00%), (−)-sclareolide: t_R(**12a**) = 143.20 min (5.30%), *ent*-5β,8α,9β-sclareolide: t_R(**12c**) = 146.04 min (2.50%).

Enantiomeric ratios of **12a**, **12b** and **12c**: (+)-sclareolide (**12a**): e.r. = 94:6; (−)-9-*epi*-sclareolide (**12b**): e.r. = 91.5:8.5; 5β,8α,9β-sclareolide: (**12c**): e.r. = 76:24.

$[\alpha]_D^{25}$ = +26.7 (*c* = 0.30, $CHCl_3$; 89:11 d.r. and 94:6 e.r.); Lit.: $[\alpha]_D^{25}$ = +43 (*c* = 0.49, $CHCl_3$).[16]

The identity of (+)-sclareolide (**12a**) in the synthetic sample was confirmed by comparison of the GC retention time and the ^{13}C NMR spectrum with an authentic sample of commercially available (+)-sclareolide (see Fig. S34).

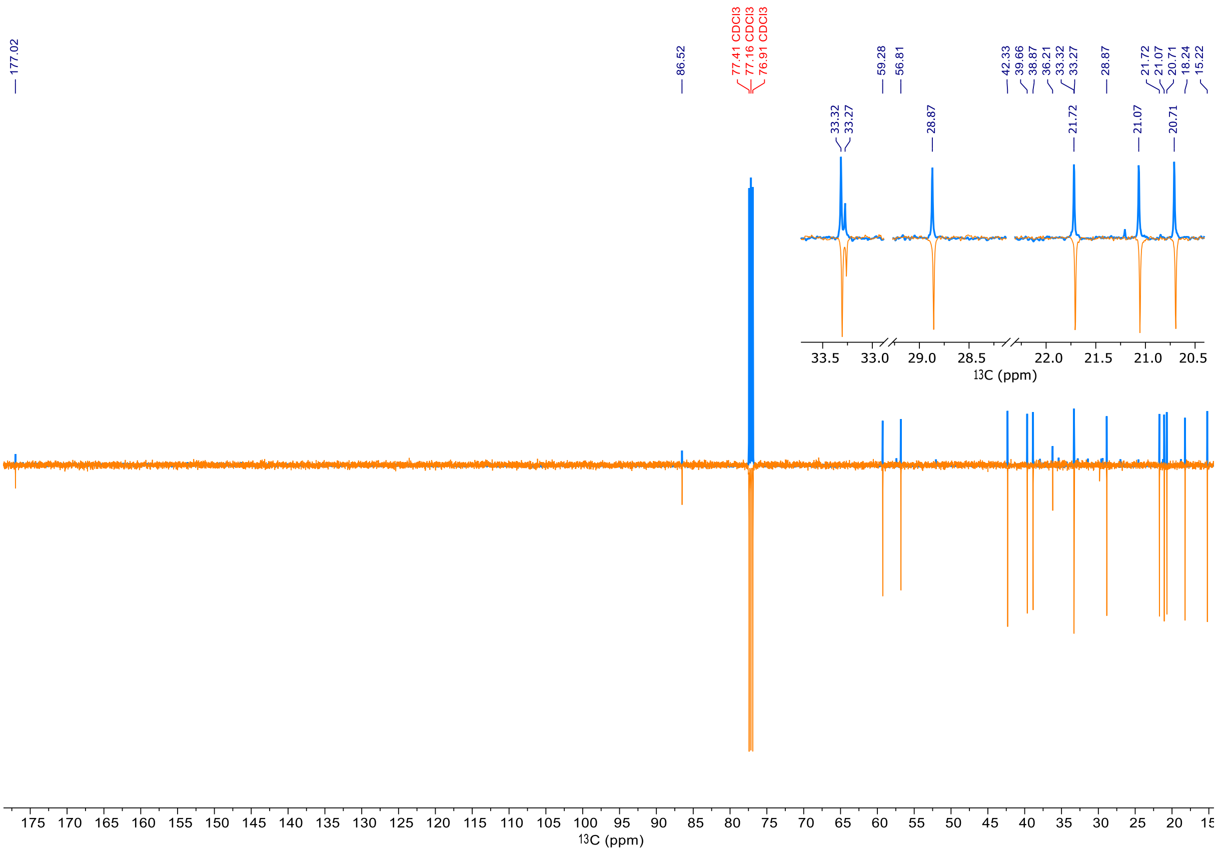

Fig. S34 | Comparison of the ^{13}C NMR (126 MHz, $CDCl_3$) spectrum of synthetic (+)-sclareolide (**12a**, top, blue) with the ^{13}C NMR (126 MHz, $CDCl_3$) spectrum of a commercially available authentic sample of (+)-sclareolide (purchased from TCI, bottom, orange).

2 Mechanistic Studies

2.1 Preliminary Natural Abundance Kinetic Isotope Effect (KIE) Studies

To evaluate whether the IDPi-catalyzed asymmetric polyene cyclization of (3*E*,7*E*)-homofarnesol (**1a**) proceeds through a concerted or stepwise process at low temperatures, preliminary ^{13}C KIE studies at natural abundance were performed.

Synthesis of the reference sample of (3*E*,7*E*)-homofarnesol

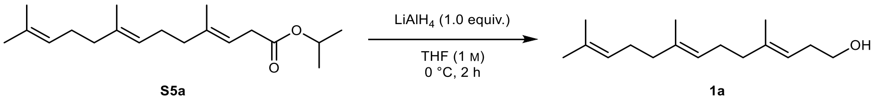

A flame-dried 250 mL round-bottom flask under argon was charged with isopropyl (3*E*,7*E*)-4,8,12-trimethyltrideca-3,7,11-trienoate (**S5a**, 7.31 g, 25.0 mmol, 1.0 equiv.) and a PTFE-coated magnetic stir bar. Dry THF (25 mL) was added and the resulting colorless solution was cooled to 0 °C. After 15 min, $LiAlH_4$ (1 M in THF, 25.0 mL, 25.0 mmol, 1.0 equiv.) was added dropwise and the resulting colorless reaction mixture was stirred for 2 h at 0 °C. After the elapsed time, a Fieser work-up was performed: the reaction mixture was diluted with MTBE (25 mL), water (1 mL) was carefully added, followed by 15% aqueous NaOH (1 mL) and additional water (3 mL). The resulting cloudy solution was stirred at room temperature for 30 min. Anhydrous $MgSO_4$ was transferred to the mixture and the resulting colorless suspension was stirred for another 15 min. The suspension was filtered over a short pad of silica (d × l = 8 × 3 cm) and both flask and pad were rinsed with ethyl acetate (3 × 50 mL). The combined filtrate was concentrated under reduced pressure to afford the crude product as a colorless oil. Purification by flash column chromatography on silica gel using hexanes/MTBE as eluent (gradient elution 4:1 → 2:1 *v/v*) furnished (3*E*,7*E*)-homofarnesol (**1a**) as a colorless oil (>20:1 *E/Z*, 5.39 g, 22.8 mmol, 91% yield).

Physical state: colorless oil.

TLC (SiO_2, hexanes/MTBE 4:1, *v/v*): R_f = 0.31 (CAM stain).

^{1}H NMR (600 MHz, toluene-d_8): δ (ppm) = 5.22–5.16 (m, 2H), 5.10 (tq, *J* = 7.3, 1.3 Hz, 1H), 3.39 (t, *J* = 6.6 Hz, 2H), 2.17–2.09 (m, 6H), 2.07–1.99 (m, 4H), 1.68 (q, *J* = 1.4 Hz, 3H), 1.58 (s, 3H), 1.57 (s, 3H), 1.54 (s, 3H), 0.91 (br s, 1H, OH).

^{13}C{^{1}H} NMR (151 MHz, toluene-d_8): δ (ppm) = 137.8, 135.2, 131.1, 125.0, 124.6, 121.0, 62.3, 40.183, 40.176, 32.1, 27.2, 26.9, 25.8, 17.7, 16.11, 16.07.

IR (ATR, neat): $\tilde{\nu}_{max}$ (cm^{-1}) = 3330 (w, br; **OH**), 2965 (m), 2916, 1668 (w), 1441 (m), 1377 (m), 1046 (s), 835 (m).

HRMS (GC-EI$^+$): *m/z* calcd. for $C_{16}H_{28}O$ $[M]^+$: 236.213465, found: 236.213290.

2.1.1 Natural Abundance KIE Measurements of (3*E*,7*E*)-Homofarnesol (1a)

For all experiments, an identical batch of **1a**, prepared as described above, was used as reference material (unreacted starting material) and as substrate in the polyene cyclization (converted starting material).

Preparation of both reference samples used for the analysis of the (3*E*,7*E*)-homofarnesol **1a**: Starting material **1a** (50.0 mg, 212 µmol) was dissolved in 200 µL $CDCl_3$ and transferred to a 3 mm Norell® NMR tube using a Hamilton® syringe. The NMR tube was placed in a dry-ice/propan-2-ol cooling bath and subsequently flame-sealed under air. A second sample was prepared in the same manner as described above.

Preparation of the high conversion sample (>96%) of recovered (3*E*,7*E*)-homofarnesol **1a** (see section 1.5.2; scale-up at −40 °C): Recovered starting material **1a** (29.4 mg, 124 µmol) obtained after preparative HPLC from the crude reaction mixture of the IDPi-catalyzed polyene cyclization (see above) was dissolved in 200 µL $CDCl_3$ and transferred to a 3 mm Norell® NMR tube using a Hamilton® syringe. The NMR tube was placed in a dry-ice/propan-2-ol cooling bath and subsequently flame-sealed under air. Quantitative single-pulse ^{13}C NMR with inverse-gated (IG) ^{1}H-decoupling ($^{13}C\{^{1}H\}_{IG}$) measurements were performed to determine the relative ^{13}C isotope ratios (R / R_0).

NMR data acquisition

Quantitative ^{13}C NMR spectra were acquired on a 600 MHz Bruker Avance Neo NMR spectrometer at 298 K equipped with a cryogenically-cooled BBO probe using an inverse-gated decoupling ^{13}C NMR sequence after a pi/6 pulse (^{13}C pulse length 3.33 µs; Bruker pulse sequence: zgig30) with a pulse offset at 75 ppm and an acquisition time of 2.2437828 s. The raw FID contained 178078 complex data points with a dwell time of 12.6 µs (spectral with: 262.9132 ppm) and for a spectrum 64 (for reference samples) and 128 FIDs (for reaction sample) were averaged. A relaxation delay time (d1; time between scans) of 110 s was used (≈7.6 times of T_1(C-12); longest carbon T_1 of the molecule).

NMR data processing

After acquisition the FIDs were zero filled to 512k data points and Fourier transformed with an EM window function (lb = 0.3 Hz) in Bruker Topspin 4.0.6. In order to remove the baseline curvature from the cryoprobe data, the resulting spectra were phased and baseline corrected with the Bruker Deep-learning based phase and baseline correction command *apbk*. (Ref: S. Bruderer, F. Paruzzo, C. Bolliger; Deep learning-based phase and baseline correction of 1D ^{1}H NMR Spectra; Bruker Application notes, link:
https://www.bruker.com/content/dam/bruker/int/en/resources/bbio/magnetic-resonance/application-notes/T186209_Bruker%20Whitepaper%20Deep%20Learning%20in%20NMR.pdf).

The resulting spectra were visually checked and the processed spectra were then imported into Mestrelab MNOVA. The imported spectra of each sample were stacked and the signals were integrated ±0.05 ppm around the peak maxima.

Spectral assignments for (3*E*,7*E*)-homofarnesol (**1a**) are listed in the table below.

Table S12 | NMR assignment data of (3*E*,7*E*)-homofarnesol (**1a**) in $CDCl_3$ at 298K.

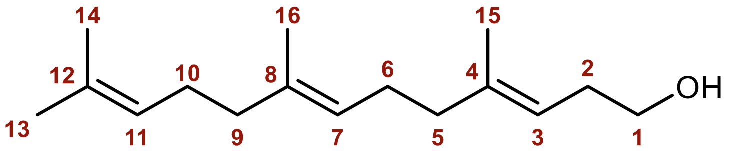

Atom	*J*	δ/ppm	T_1/s	HSQC	COSY	HMBC	NOESY
1 C		62.374	1.76	1		2, 3	
H2	6.6(2)	3.569		1	2	2, 3	
2 C		31.556	1.96	2		1, 3	
H2	6.6(1)	2.254		2	1, 3, 5, 15	1, 3, 4	15
3 C		119.993	2.73	3		1, 2, 5, 15	
H		5.107		3	2, 5, 15	1, 2, 5, 15	5
4 C		138.570	7.60			2, 5, 6, 15	
5 C		39.841	1.63	5		3, 6, 7, 15	
H2		2.012		5	2, 3	3, 4, 6, 7, 8, 15	3, 15
6 C		26.543	1.58	6		5, 7	
H2		2.076		6	7, 9, 16	4, 5, 7, 8	15, 16
7 C		124.062	2.73	7		5, 6, 9, 16	
H		5.073		7	6, 9, 16	5, 6, 9, 16	9
8 C		135.228	8.80			5, 6, 9, 10, 16	
9 C		39.752	1.97	9		7, 10, 11, 16	
H2		1.952		9	6, 7	7, 8, 10, 11, 16	7, 16

Atom	*J*	δ/ppm	T_1/s	HSQC	COSY	HMBC	NOESY
10 C		26.781	2.08	10		9, 11	
H2		2.036		10	11, 13, 14	8, 9, 11, 12	14
11 C		124.407	4.66	11		9, 10, 13, 14	
H		5.067		11	10, 13, 14	9, 10, 13, 14	13
12 C		131.265	14.53			10, 13, 14	
13 C		25.711	3.77	13		11, 14	
H3		1.653		13	10, 11	11, 12, 14	11
14 C		17.686	9.87	14		11, 13	
H3		1.575		14	10, 11	11, 12, 13	10
15 C		16.205	7.96	15		3, 5	
H3		1.619		15	2, 3	3, 4, 5	2, 5, 6
16 C		16.029	7.79	16		7, 9	
H3		1.575		16	6, 7	7, 8, 9	6, 9
17 O							
H							

For quantitative ^{13}C NMR measurements a relaxation delay of d1 = 110 s was used (≈ 7.5 T_1(C-12)).

^{13}C relaxation times were determined by using an inversion-recovery NMR pulse sequence.

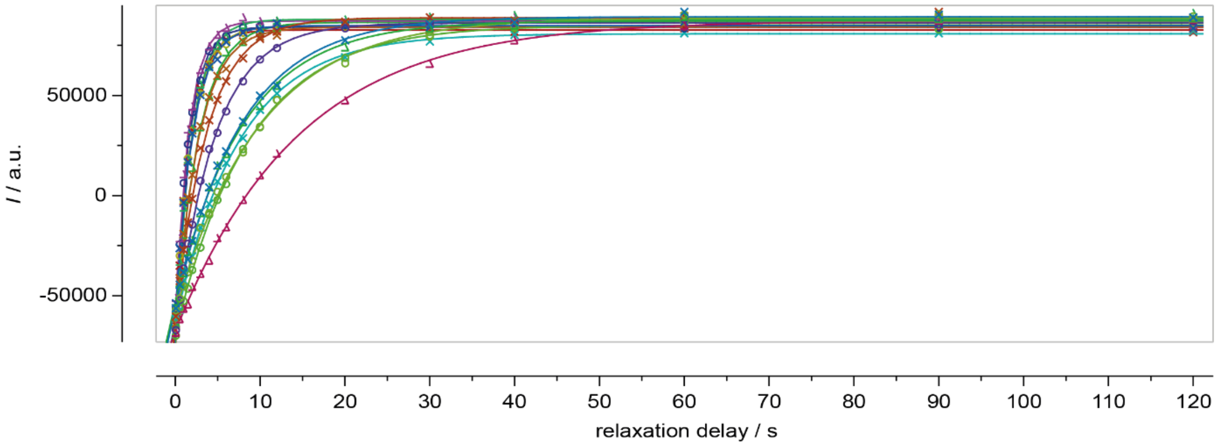

Fig. S35 | Recovery profile obtained from the inversion-recovery experiment for the individual carbon nuclei of **1a** after plotting peak integral with respect to the relaxation delay.

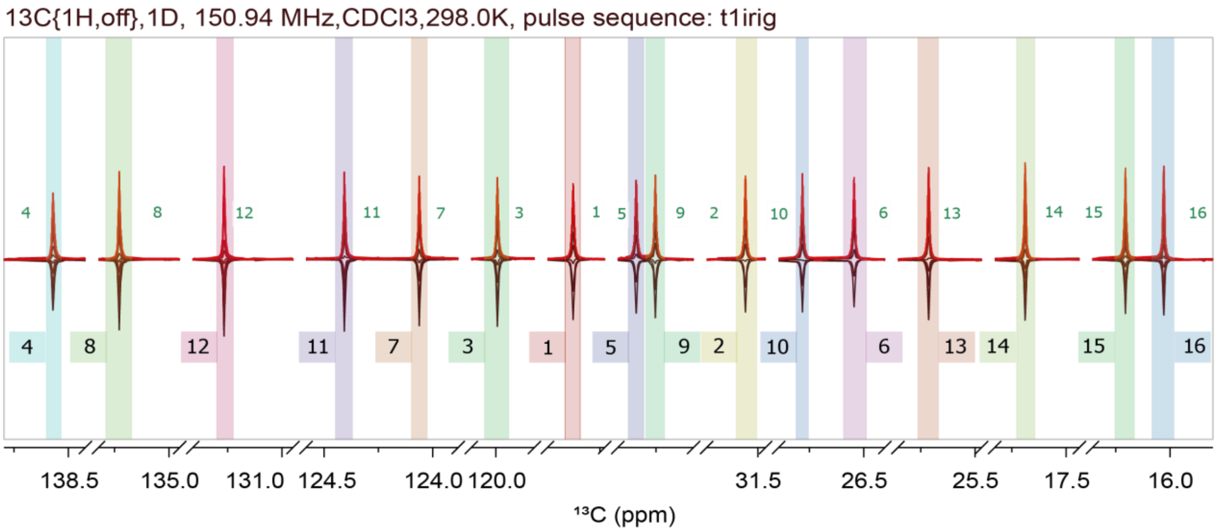

Fig. S36 | Integration regions of interest with the respective assignments.

2.1.2 KIE Calculation

Kinetic isotope effects of the re-isolated starting materials at high conversions F were calculated with the following equation (2):[43]

$$\mathrm{KIE} \;=\; \frac{\ln(1-F)}{\ln(1-F)\,(R/R_0)}\,\Delta F \tag{2}$$

Where R / R_0 is the relative signal ratio of a carbon signal compared to the reference signal from the unreacted starting material. Uncertainties of the KIEs (ΔKIE) were estimated using the following equations:

$$\Delta\mathrm{KIE}_F \;=\; \left|\frac{-\ln(R/R_0)}{(1-F)\,\ln^2[(1-F)(R/R_0)]}\,\Delta F\right| \tag{3}$$

$$\Delta\mathrm{KIE}_R \;=\; \frac{-\ln(1-F)}{(R/R_0)\,\ln^2[(1-F)(R/R_0)]}\,\Delta(R/R_0) \tag{4}$$

The absolute and relative (internally referenced to C16) NMR integrals of both reference samples (sample 1: #1-7, black; sample 2: #8-18, maroon) and the 96% conversion sample (#21-25; navy) as well as KIE results are listed in Table S13 shown below. Excerpts of stacked quantitative ^{13}C NMR spectra of reference samples and the 96% conversion sample with integration regions of interest are provided in Fig. S37. Relative signal ratios R / R_0 and KIEs are shown in Fig. S38.

The experiment was repeated and the ^{13}C KIEs at natural abundance for a sample obtained at 91% conversion of (3*E*,7*E*)-homofarnesol were determined in the same manner as described for the first experiment (see Table S14 and Fig. S39).

Table S13 | NMR integrals and KIE results of the first natural abundance ^{13}C KIE experiment for a sample obtained at 96% conversion of the starting material (3*E*,7*E*)-homofarnesol.

	Integrals (10^{-12} a.u.)																
	#	C1	C2	C3	C4	C5	C6	C7	C8	C9	C10	C11	C12	C13	C14	C15	C16
Ref	1	0.6912	0.7135	0.6492	0.6400	0.7076	0.7353	0.6619	0.6475	0.7472	0.7480	0.6734	0.6777	0.7479	0.7538	0.7321	0.7642
	2	0.6919	0.7208	0.6568	0.6414	0.7046	0.7363	0.6582	0.6433	0.7528	0.7535	0.6710	0.6752	0.7524	0.7560	0.7270	0.7624
	3	0.6895	0.7139	0.6509	0.6422	0.7175	0.7344	0.6637	0.6521	0.7361	0.7404	0.6800	0.6764	0.7505	0.7621	0.7266	0.7612
	4	0.7408	0.7546	0.6900	0.6962	0.7500	0.7768	0.7050	0.6893	0.7838	0.7818	0.7337	0.7255	0.7903	0.7912	0.7613	0.7988
	5	0.7337	0.7649	0.6974	0.6893	0.7489	0.7690	0.7084	0.6964	0.7940	0.7846	0.7354	0.7263	0.7948	0.7992	0.7727	0.8013
	6	0.7375	0.7500	0.6965	0.6917	0.7563	0.7760	0.7239	0.6988	0.7928	0.7897	0.7281	0.7375	0.7909	0.8001	0.7666	0.8012
	7	0.7351	0.7522	0.6951	0.6939	0.7500	0.7645	0.7130	0.6946	0.7988	0.7892	0.7378	0.7212	0.8037	0.7985	0.7598	0.8163
	8	0.7288	0.7610	0.6929	0.6843	0.7435	0.7673	0.7112	0.6955	0.7818	0.7869	0.7234	0.7251	0.7945	0.8056	0.7718	0.8020
	11	0.7856	0.8036	0.7429	0.7285	0.7932	0.8202	0.7600	0.7371	0.8335	0.8303	0.7808	0.7740	0.8362	0.8400	0.8144	0.8471
	12	0.7868	0.8020	0.7376	0.7364	0.7971	0.8302	0.7406	0.7418	0.8372	0.8440	0.7773	0.7695	0.8440	0.8499	0.8207	0.8546
	13	0.7792	0.8087	0.7331	0.7282	0.8010	0.8302	0.7597	0.7514	0.8463	0.8310	0.7751	0.7657	0.8423	0.8552	0.8168	0.8563
	14	0.7876	0.8094	0.7445	0.7474	0.7894	0.8199	0.7524	0.7419	0.8321	0.8421	0.7702	0.7735	0.8491	0.8531	0.8175	0.8524
	15	0.7821	0.8007	0.7487	0.7387	0.7935	0.8244	0.7582	0.7367	0.8389	0.8292	0.7778	0.7697	0.8275	0.8445	0.8130	0.8526
	16	0.7764	0.8065	0.7296	0.7345	0.7925	0.8296	0.7568	0.7410	0.8305	0.8323	0.7721	0.7831	0.8449	0.8419	0.8169	0.8498
	17	0.7771	0.8098	0.7447	0.7338	0.8021	0.8233	0.7537	0.7413	0.8530	0.8441	0.7730	0.7704	0.8408	0.8492	0.8171	0.8624
	18	0.7815	0.8132	0.7486	0.7371	0.7902	0.8271	0.7561	0.7422	0.8295	0.8292	0.7754	0.7725	0.8310	0.8462	0.8185	0.8589
96%	21	1.0441	1.0708	1.0026	0.9882	1.0547	1.0427	1.0075	0.9942	1.0546	1.0741	1.0196	1.0133	1.0760	1.0710	1.0576	1.0750
	22	1.0563	1.0664	1.0056	1.0067	1.0480	1.0594	1.0008	1.0014	1.0705	1.0713	1.0288	1.0027	1.0482	1.0740	1.0699	1.0706
	23	1.0478	1.0605	1.0061	0.9881	1.0480	1.0585	1.0129	0.9954	1.0663	1.0782	1.0212	1.0365	1.0832	1.0699	1.0605	1.0742
	24	1.0486	1.0657	1.0005	0.9782	1.0389	1.0537	1.0077	0.9929	1.0625	1.0630	1.0218	1.0244	1.0716	1.0750	1.0633	1.0569
	25	1.0494	1.0727	0.9909	0.9864	1.0483	1.0639	0.9961	0.9870	1.0687	1.0668	1.0308	1.0332	1.0744	1.0759	1.0677	1.0722

	relative Integrals (reference signal C16)																
	#	C1	C2	C3	C4	C5	C6	C7	C8	C9	C10	C11	C12	C13	C14	C15	C16
Ref	1	0.9044	0.9336	0.8495	0.8375	0.9259	0.9621	0.8661	0.8473	0.9777	0.9788	0.8811	0.8867	0.9786	0.9864	0.9580	1.0000
	2	0.9076	0.9454	0.8616	0.8414	0.9243	0.9658	0.8633	0.8438	0.9875	0.9884	0.8802	0.8857	0.9870	0.9917	0.9536	1.0000
	3	0.9058	0.9379	0.8551	0.8436	0.9426	0.9648	0.8720	0.8567	0.9671	0.9727	0.8933	0.8885	0.9860	1.0012	0.9546	1.0000
	4	0.9274	0.9446	0.8638	0.8715	0.9388	0.9724	0.8825	0.8629	0.9812	0.9786	0.9184	0.9082	0.9892	0.9905	0.9531	1.0000
	5	0.9157	0.9546	0.8704	0.8603	0.9347	0.9597	0.8840	0.8691	0.9909	0.9791	0.9177	0.9064	0.9919	0.9974	0.9644	1.0000
	6	0.9205	0.9361	0.8693	0.8634	0.9439	0.9686	0.9035	0.8722	0.9895	0.9856	0.9088	0.9205	0.9872	0.9986	0.9568	1.0000
	7	0.9005	0.9215	0.8515	0.8501	0.9188	0.9366	0.8734	0.8510	0.9786	0.9668	0.9038	0.8835	0.9846	0.9782	0.9308	1.0000
	8	0.9087	0.9489	0.8640	0.8532	0.9270	0.9567	0.8868	0.8672	0.9748	0.9811	0.9020	0.9041	0.9906	1.0045	0.9623	1.0000
	11	0.9274	0.9487	0.8770	0.8601	0.9364	0.9683	0.8972	0.8702	0.9840	0.9802	0.9218	0.9137	0.9872	0.9917	0.9614	1.0000
	12	0.9207	0.9385	0.8631	0.8617	0.9327	0.9715	0.8667	0.8680	0.9797	0.9876	0.9096	0.9005	0.9877	0.9945	0.9604	1.0000
	13	0.9100	0.9444	0.8562	0.8504	0.9355	0.9696	0.8872	0.8775	0.9884	0.9704	0.9053	0.8943	0.9837	0.9988	0.9539	1.0000
	14	0.9239	0.9495	0.8733	0.8768	0.9261	0.9619	0.8827	0.8703	0.9762	0.9879	0.9035	0.9074	0.9961	1.0008	0.9590	1.0000
	15	0.9173	0.9392	0.8781	0.8664	0.9307	0.9669	0.8893	0.8641	0.9840	0.9725	0.9122	0.9028	0.9706	0.9905	0.9536	1.0000
	16	0.9137	0.9491	0.8586	0.8643	0.9327	0.9763	0.8906	0.8720	0.9773	0.9795	0.9086	0.9216	0.9943	0.9908	0.9614	1.0000
	17	0.9011	0.9391	0.8635	0.8508	0.9302	0.9547	0.8740	0.8596	0.9891	0.9788	0.8963	0.8933	0.9750	0.9847	0.9475	1.0000
	18	0.9099	0.9469	0.8717	0.8582	0.9201	0.9631	0.8803	0.8641	0.9658	0.9654	0.9029	0.8995	0.9675	0.9853	0.9530	1.0000
96%	21	0.9713	0.9961	0.9326	0.9193	0.9811	0.9700	0.9372	0.9248	0.9810	0.9992	0.9484	0.9426	1.0010	0.9963	0.9838	1.0000
	22	0.9866	0.9960	0.9393	0.9403	0.9788	0.9895	0.9348	0.9353	0.9999	1.0006	0.9610	0.9366	0.9791	1.0032	0.9994	1.0000
	23	0.9754	0.9872	0.9366	0.9198	0.9756	0.9854	0.9429	0.9266	0.9926	1.0037	0.9507	0.9648	1.0084	0.9960	0.9873	1.0000
	24	0.9922	1.0084	0.9467	0.9256	0.9830	0.9970	0.9534	0.9395	1.0054	1.0058	0.9669	0.9693	1.0139	1.0171	1.0061	1.0000
	25	0.9787	1.0005	0.9242	0.9200	0.9778	0.9923	0.9290	0.9206	0.9967	0.9950	0.9614	0.9637	1.0020	1.0034	0.9958	1.0000

		averaged relative Integrals															
Ref		0.9134	0.9424	0.8642	0.8569	0.9313	0.9637	0.8812	0.8635	0.9807	0.9783	0.9041	0.9010	0.9848	0.9928	0.9552	1.0000
		0.0088	0.0081	0.0087	0.0109	0.0074	0.0092	0.0114	0.0096	0.0076	0.0072	0.0119	0.0119	0.0081	0.0071	0.0079	0.0000
	Δ	± 0.96%	± 0.86%	± 1.00%	± 1.27%	± 0.79%	± 0.96%	± 1.29%	± 1.11%	± 0.77%	± 0.73%	± 1.32%	± 1.32%	± 0.82%	± 0.72%	± 0.83%	± 0.00%
96%		0.9809	0.9976	0.9359	0.9250	0.9793	0.9868	0.9395	0.9294	0.9951	1.0009	0.9577	0.9554	1.0009	1.0032	0.9945	1.0000
		± 0.0085	± 0.0077	± 0.0083	± 0.0089	± 0.0029	± 0.0103	± 0.0093	± 0.0078	± 0.0092	± 0.0042	± 0.0078	± 0.0147	± 0.0133	± 0.0086	± 0.0090	± 0.0000
	Δ	± 0.87%	± 0.77%	± 0.89%	± 0.96%	± 0.30%	± 1.05%	± 0.99%	± 0.84%	± 0.92%	± 0.42%	± 0.82%	± 1.54%	± 1.33%	± 0.85%	± 0.91%	± 0.00%

R/R_0		1.0739	1.0587	1.0830	1.0795	1.0515	1.0240	1.0661	1.0763	1.0147	1.0230	1.0593	1.0603	1.0163	1.0104	1.0411	1.0000
	Δ	± 0.0139	± 0.0122	± 0.0145	± 0.0172	± 0.0089	± 0.0145	± 0.0173	± 0.0150	± 0.0122	± 0.0086	± 0.0164	± 0.0215	± 0.0159	± 0.0113	± 0.0128	± 0.0000

KIE		1.023	1.018	1.025	1.024	1.016	1.007	1.020	1.023	1.005	1.007	1.018	1.019	1.005	1.003	1.013	1.000
	Δ*R*	± 0.004	± 0.004	± 0.004	± 0.005	± 0.003	± 0.004	± 0.005	± 0.005	± 0.004	± 0.003	± 0.005	± 0.007	± 0.005	± 0.003	± 0.004	± 0.000
	Δ*F*	±0.002	±0.001	±0.002	±0.002	±0.001	±0.001	±0.002	±0.002	±0.000	±0.001	±0.001	±0.001	±0.000	±0.000	±0.001	±0.000
	ΣΔ	±0.006	±0.005	±0.006	±0.007	±0.004	±0.005	±0.007	±0.006	±0.004	±0.003	±0.006	±0.008	±0.005	±0.004	±0.005	±0.000

Table S14 | ^{13}C NMR integrals and KIE results of the second natural abundance ^{13}C KIE experiment for a sample obtained at 91% conversion of the starting material (3*E*,7*E*)-homofarnesol.

	#	C1	C2	C3	C4	C5	C6	C7	C8	C9	C10	C11	C12	C13	C14	C15	C16
		Integrals (10^{-12} a.u.)															
Ref	1	0.7159	0.7285	0.6695	0.6666	0.7180	0.7511	0.6802	0.6807	0.7765	0.7629	0.6968	0.7015	0.7850	0.7971	0.7520	0.7812
	2	0.7275	0.7461	0.6668	0.6708	0.7275	0.7556	0.6869	0.6709	0.7828	0.7697	0.7148	0.7095	0.7911	0.7966	0.7509	0.7923
	3	0.7129	0.7436	0.6673	0.6639	0.7273	0.7575	0.6746	0.6720	0.7814	0.7636	0.7138	0.7052	0.7820	0.7901	0.7485	0.7915
	4	0.7143	0.7318	0.6674	0.6665	0.7123	0.7573	0.6807	0.6823	0.7731	0.7481	0.7153	0.7018	0.7820	0.7935	0.7441	0.7976
	5	0.7084	0.7295	0.6682	0.6815	0.7168	0.7447	0.6776	0.6624	0.7711	0.7650	0.7040	0.7132	0.7788	0.7932	0.7486	0.7857
	6	0.7158	0.7431	0.6632	0.6760	0.7319	0.7478	0.6767	0.6683	0.7786	0.7479	0.7086	0.7103	0.7776	0.7852	0.7485	0.7916
	7	0.7215	0.7392	0.6715	0.6745	0.7214	0.7555	0.6750	0.6738	0.7780	0.7673	0.7113	0.7086	0.7855	0.7895	0.7530	0.7945
	8	0.7153	0.7408	0.6703	0.6653	0.7100	0.7586	0.6709	0.6721	0.7783	0.7625	0.7088	0.7053	0.7867	0.7993	0.7449	0.7899
	11	0.7297	0.7489	0.6724	0.6856	0.7123	0.7641	0.6756	0.6772	0.7854	0.7670	0.7121	0.7091	0.7848	0.7913	0.7557	0.8044
	12	0.7226	0.7426	0.6810	0.6856	0.7236	0.7590	0.6849	0.6760	0.7826	0.7617	0.7153	0.7125	0.7873	0.7970	0.7482	0.7923
	13	0.7217	0.7394	0.6756	0.6754	0.7326	0.7579	0.6807	0.6738	0.7969	0.7740	0.7022	0.7106	0.7881	0.7933	0.7533	0.7916
	14	0.7224	0.7491	0.6740	0.6763	0.7272	0.7507	0.6861	0.6746	0.7843	0.7634	0.7121	0.7150	0.7950	0.7980	0.7551	0.7964
	15	0.7216	0.7408	0.6782	0.6746	0.7256	0.7501	0.6838	0.6701	0.7792	0.7747	0.7162	0.7171	0.7901	0.7937	0.7487	0.7955
	16	0.7204	0.7445	0.6662	0.6676	0.7209	0.7473	0.6807	0.6781	0.7884	0.7603	0.7191	0.7200	0.7909	0.7860	0.7491	0.8018
	17	0.7215	0.7339	0.6724	0.6766	0.7211	0.7599	0.6902	0.6762	0.7815	0.7635	0.7119	0.7073	0.7859	0.7860	0.7536	0.7878
	18	0.7157	0.7377	0.6805	0.6686	0.7260	0.7543	0.6799	0.6618	0.7813	0.7587	0.7146	0.7042	0.7783	0.7876	0.7451	0.7920
91%	21	0.9885	1.0100	0.9461	0.9308	1.0046	1.0093	0.9536	0.9502	1.0154	1.0225	0.9595	0.9609	1.0225	1.0195	1.0141	1.0261
	22	0.9442	0.9577	0.8885	0.8719	0.9713	0.9782	0.8884	0.8655	0.9628	0.9799	0.8880	0.8814	0.9743	0.9581	0.9870	0.9848
	23	0.9402	0.9712	0.8750	0.8658	0.9551	0.9828	0.8896	0.8794	0.9669	0.9773	0.8903	0.9019	0.9795	0.9840	0.9661	0.9980
	24	0.9288	0.9578	0.8900	0.8690	0.9561	0.9692	0.8995	0.8792	0.9626	0.9694	0.9056	0.8992	0.9647	0.9682	0.9788	0.9706
	25	0.9326	0.9492	0.8657	0.8489	0.9512	0.9549	0.8688	0.8546	0.9554	0.9586	0.8807	0.8831	0.9476	0.9658	0.9668	0.9832
	26	0.9754	0.9872	0.9366	0.9198	0.9756	0.9854	0.9429	0.9266	0.9926	1.0037	0.9507	0.9648	1.0084	0.9960	0.9873	1.0000
	27	0.9922	1.0084	0.9467	0.9256	0.9830	0.9970	0.9534	0.9395	1.0054	1.0058	0.9669	0.9693	1.0139	1.0171	1.0061	1.0000
	28	0.9787	1.0005	0.9242	0.9200	0.9778	0.9923	0.9290	0.9206	0.9967	0.9950	0.9614	0.9637	1.0020	1.0034	0.9958	1.0000

	#	C1	C2	C3	C4	C5	C6	C7	C8	C9	C10	C11	C12	C13	C14	C15	C16
		relative Integrals (reference signal C16)															
Ref	1	0.9164	0.9325	0.8571	0.8533	0.9191	0.9615	0.8707	0.8714	0.9940	0.9766	0.8920	0.8979	1.0049	1.0203	0.9626	1.0000
	2	0.9182	0.9417	0.8416	0.8467	0.9182	0.9536	0.8670	0.8467	0.9880	0.9714	0.9021	0.8955	0.9985	1.0054	0.9477	1.0000
	3	0.9007	0.9394	0.8430	0.8388	0.9189	0.9570	0.8523	0.8490	0.9872	0.9647	0.9018	0.8909	0.9880	0.9982	0.9457	1.0000
	4	0.8955	0.9174	0.8368	0.8356	0.8930	0.9495	0.8534	0.8554	0.9693	0.9379	0.8968	0.8799	0.9804	0.9949	0.9329	1.0000
	5	0.9016	0.9286	0.8505	0.8674	0.9123	0.9478	0.8624	0.8430	0.9814	0.9737	0.8961	0.9078	0.9913	1.0095	0.9528	1.0000
	6	0.9042	0.9386	0.8378	0.8539	0.9245	0.9446	0.8548	0.8442	0.9835	0.9447	0.8951	0.8973	0.9823	0.9919	0.9455	1.0000
	7	0.9081	0.9303	0.8451	0.8489	0.9080	0.9509	0.8496	0.8481	0.9791	0.9657	0.8953	0.8918	0.9886	0.9937	0.9478	1.0000
	8	0.9056	0.9378	0.8486	0.8423	0.8988	0.9603	0.8493	0.8508	0.9853	0.9654	0.8974	0.8929	0.9959	1.0119	0.9431	1.0000
	11	0.9072	0.9309	0.8359	0.8524	0.8855	0.9498	0.8399	0.8418	0.9764	0.9535	0.8852	0.8815	0.9757	0.9837	0.9395	1.0000
	12	0.9121	0.9373	0.8596	0.8654	0.9133	0.9581	0.8645	0.8533	0.9878	0.9614	0.9029	0.8993	0.9937	1.0060	0.9444	1.0000
	13	0.9117	0.9341	0.8534	0.8532	0.9254	0.9575	0.8599	0.8512	1.0066	0.9777	0.8870	0.8976	0.9956	1.0021	0.9515	1.0000
	14	0.9071	0.9406	0.8463	0.8492	0.9131	0.9426	0.8615	0.8470	0.9848	0.9586	0.8942	0.8978	0.9982	1.0020	0.9481	1.0000
	15	0.9071	0.9312	0.8525	0.8480	0.9122	0.9429	0.8596	0.8423	0.9795	0.9739	0.9003	0.9015	0.9932	0.9977	0.9411	1.0000
	16	0.8984	0.9284	0.8308	0.8326	0.8990	0.9320	0.8489	0.8457	0.9832	0.9482	0.8968	0.8980	0.9863	0.9802	0.9342	1.0000
	17	0.9159	0.9316	0.8536	0.8589	0.9154	0.9646	0.8761	0.8584	0.9921	0.9691	0.9037	0.8979	0.9977	0.9977	0.9566	1.0000
	18	0.9036	0.9314	0.8592	0.8442	0.9166	0.9523	0.8585	0.8355	0.9865	0.9580	0.9022	0.8891	0.9827	0.9944	0.9407	1.0000
91%	21	0.9633	0.9843	0.9220	0.9071	0.9790	0.9836	0.9293	0.9261	0.9896	0.9965	0.9351	0.9364	0.9965	0.9936	0.9883	1.0000
	22	0.9587	0.9725	0.9022	0.8854	0.9863	0.9933	0.9021	0.8788	0.9776	0.9951	0.9017	0.8951	0.9894	0.9729	1.0022	1.0000
	23	0.9421	0.9731	0.8768	0.8676	0.9570	0.9848	0.8913	0.8811	0.9688	0.9793	0.8921	0.9037	0.9815	0.9860	0.9680	1.0000
	24	0.9569	0.9868	0.9170	0.8953	0.9850	0.9985	0.9268	0.9058	0.9918	0.9988	0.9330	0.9264	0.9939	0.9975	1.0085	1.0000
	25	0.9485	0.9654	0.8805	0.8634	0.9674	0.9711	0.8836	0.8692	0.9717	0.9749	0.8957	0.8982	0.9637	0.9823	0.9832	1.0000
	26	0.9506	0.9602	0.8933	0.8734	0.9531	0.9686	0.8863	0.8742	0.9641	0.9764	0.8837	0.8942	0.9765	0.9859	0.9762	1.0000
	27	0.9569	0.9865	0.9153	0.8799	0.9879	0.9963	0.9092	0.8925	0.9845	0.9975	0.9154	0.9186	0.9891	1.0046	1.0095	1.0000
	28	0.9508	1.0012	0.9091	0.8828	0.9745	1.0018	0.9016	0.8920	0.9830	0.9925	0.9024	0.9079	0.9872	0.9999	1.0106	1.0000

		averaged relative Integrals															
Ref		0.9071	0.9332	0.8470	0.8494	0.9108	0.9516	0.8580	0.8490	0.9853	0.9625	0.8968	0.8948	0.9908	0.9993	0.9459	1.0000
	Δ	0.0065	0.0061	0.0088	0.0097	0.0113	0.0084	0.0092	0.0082	0.0083	0.0118	0.0054	0.0070	0.0079	0.0102	0.0077	0.0000
		± 0.72%	± 0.65%	± 1.04%	± 1.14%	± 1.24%	± 0.89%	± 1.07%	± 0.97%	± 0.84%	± 1.22%	± 0.61%	± 0.79%	± 0.80%	± 1.02%	± 0.82%	± 0.00%
91%		0.9535	0.9788	0.9020	0.8819	0.9738	0.9873	0.9038	0.8900	0.9789	0.9889	0.9074	0.9100	0.9847	0.9903	0.9933	1.0000
	Δ	± 0.0067	± 0.0134	± 0.0170	± 0.0144	± 0.0134	± 0.0124	± 0.0173	± 0.0187	± 0.0100	± 0.0102	± 0.0188	± 0.0156	± 0.0106	± 0.0104	± 0.0166	± 0.0000
		± 0.71%	± 1.37%	± 1.89%	± 1.63%	± 1.38%	± 1.26%	± 1.91%	± 2.10%	± 1.02%	± 1.03%	± 2.07%	± 1.71%	± 1.08%	± 1.05%	± 1.67%	± 0.00%
R/R_0		1.0512	1.0488	1.0650	1.0382	1.0691	1.0375	1.0533	1.0483	0.9935	1.0273	1.0118	1.0171	0.9939	0.9910	1.0501	1.0000
	Δ	± 0.0106	± 0.0159	± 0.0229	± 0.0207	± 0.0198	± 0.0160	± 0.0231	± 0.0243	± 0.0131	± 0.0164	± 0.0219	± 0.0192	± 0.0133	± 0.0145	± 0.0195	± 0.0000
KIE		1.021	1.020	1.027	1.016	1.029	1.016	1.022	1.020	0.997	1.011	1.005	1.007	0.997	0.996	1.021	1.000
	Δ*R*	± 0.004	± 0.007	± 0.009	± 0.009	± 0.008	± 0.007	± 0.009	± 0.010	± 0.005	± 0.007	± 0.009	± 0.008	± 0.006	± 0.006	± 0.008	± 0.000
	Δ*F*	±0.001	±0.001	±0.001	±0.001	±0.001	±0.001	±0.001	±0.001	±0.000	±0.001	±0.000	±0.000	±0.000	±0.000	±0.001	±0.000
	ΣΔ	±0.005	±0.007	±0.011	±0.009	±0.009	±0.007	±0.011	±0.011	±0.006	±0.007	±0.009	±0.008	±0.006	±0.006	±0.009	±0.000

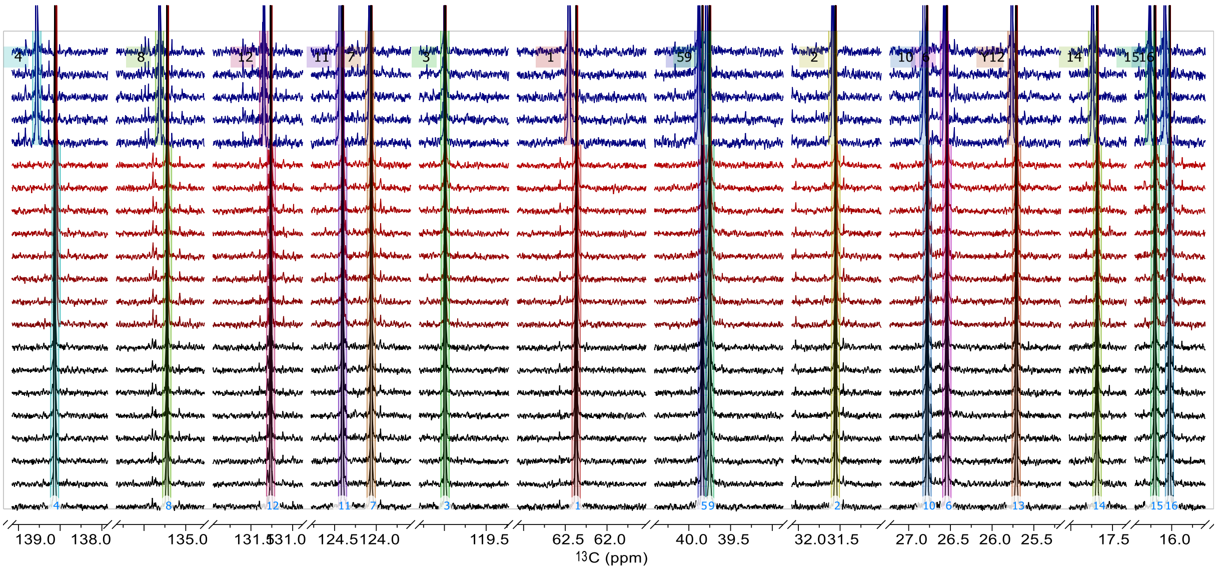

Fig. S37 | Stacked quantitative ^{13}C NMR spectra of sample the two reference samples (black and maroon) and reisolated residual starting material at 96% conversion (navy) showing integration regions of signals of interest.

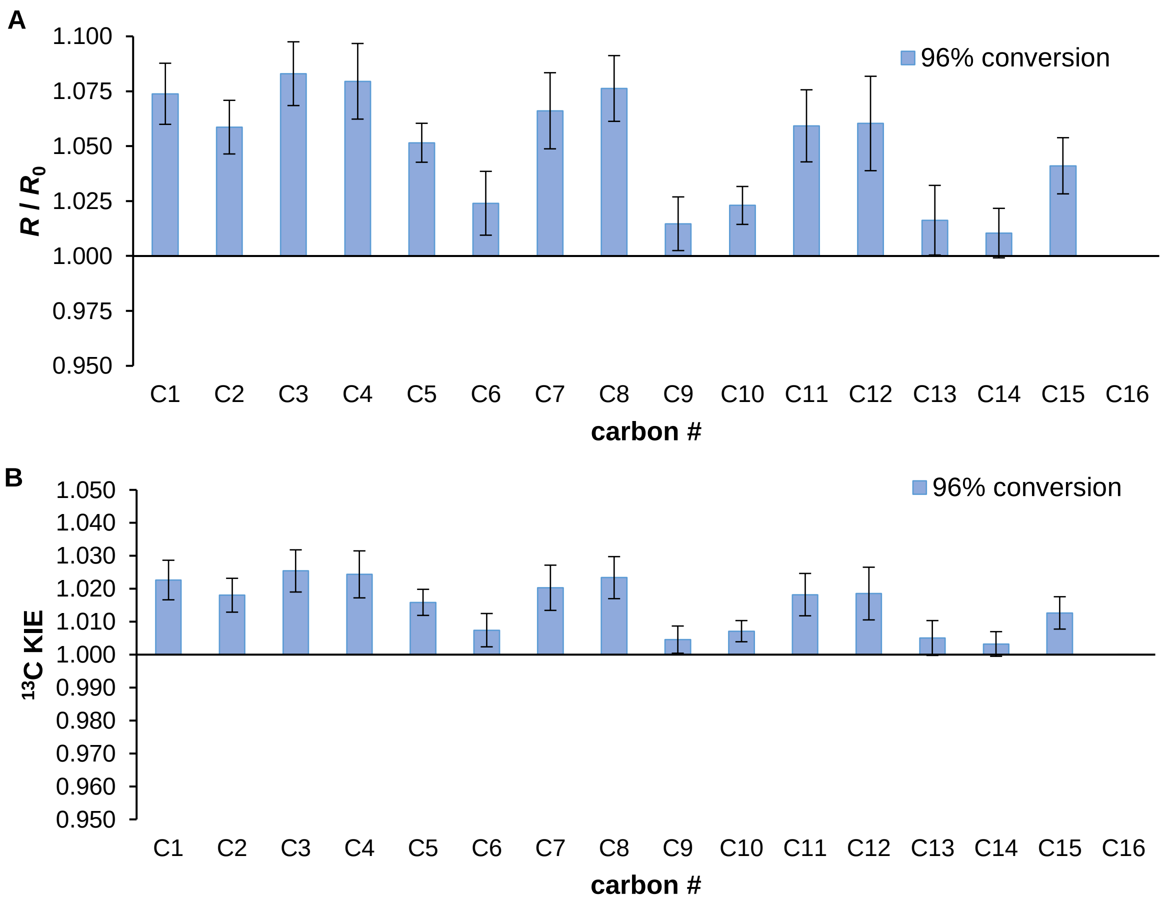

Fig. S38 | Relative ^{13}C isotope ratios (R / R_0, top, **A**) and KIEs (bottom, **B**) observed at the respective positions of **1a** for a sample with 96% conversion of **1a**. The error bars reported for R / R_0 are calculated from the standard deviations of the relative integrals (C16 as reference) of the reference sample (n = 2 × 9 measurements) and the 96% conversion sample (n = 5 measurements). The error bars reported for the KIEs correspond to the uncertainties calculated according to equations (3) and (4).

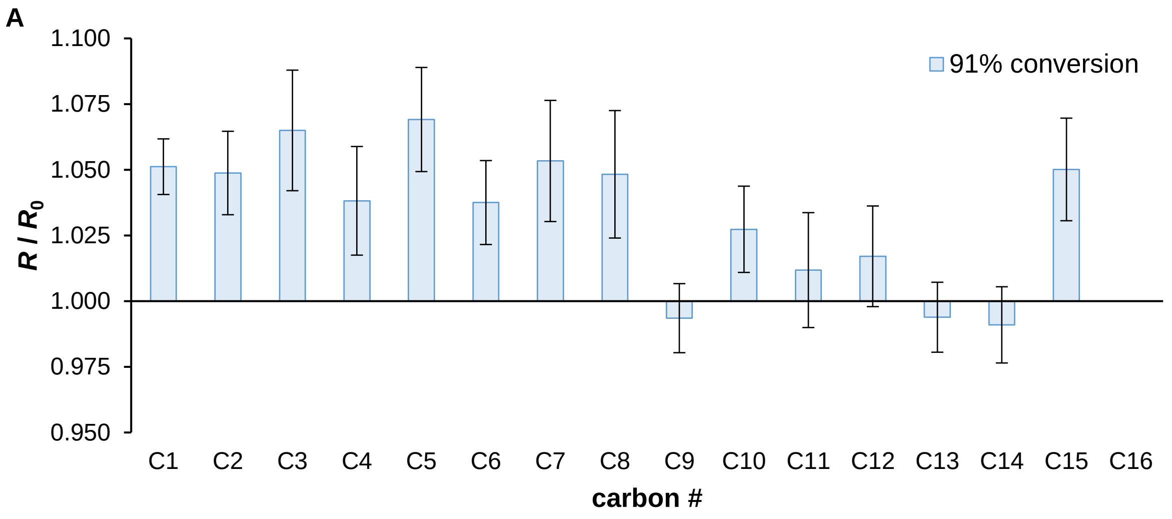

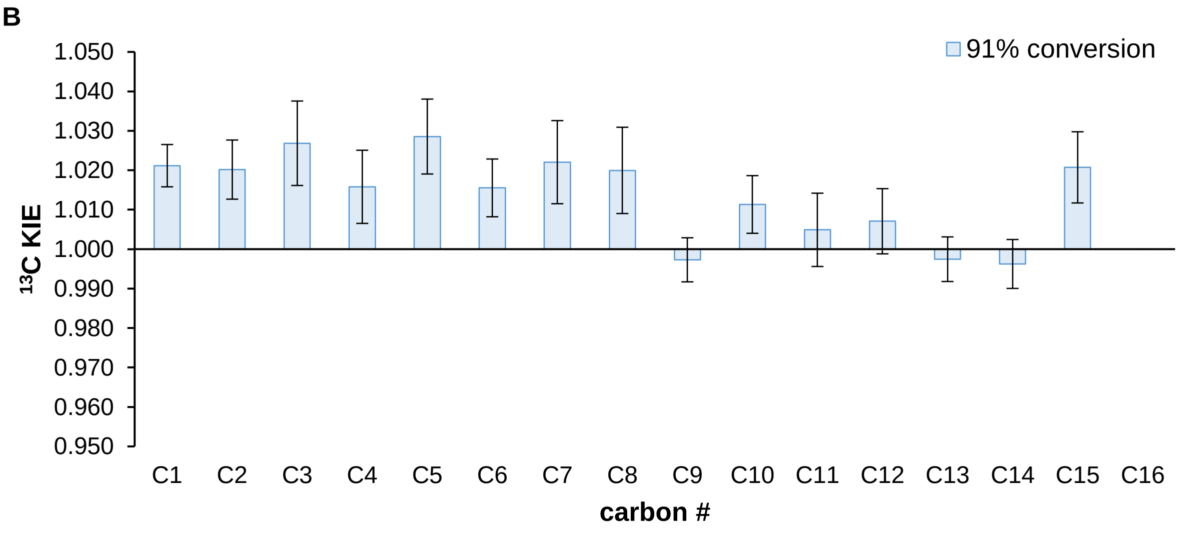

Fig. S 39 | Relative ^{13}C isotope ratios (R / R_0, top, **A**) and KIEs (bottom, **B**) observed at the respective positions of **1a** for a sample with 96% conversion of **1a**. The error bars reported for R / R_0 are calculated from the standard deviations of the relative integrals (C16 as reference) of the reference sample (n = 2 × 9 measurements) and the 96% conversion sample (n = 8 measurements). The error bars reported for the KIEs correspond to the uncertainties calculated according to equations (3) and (4).

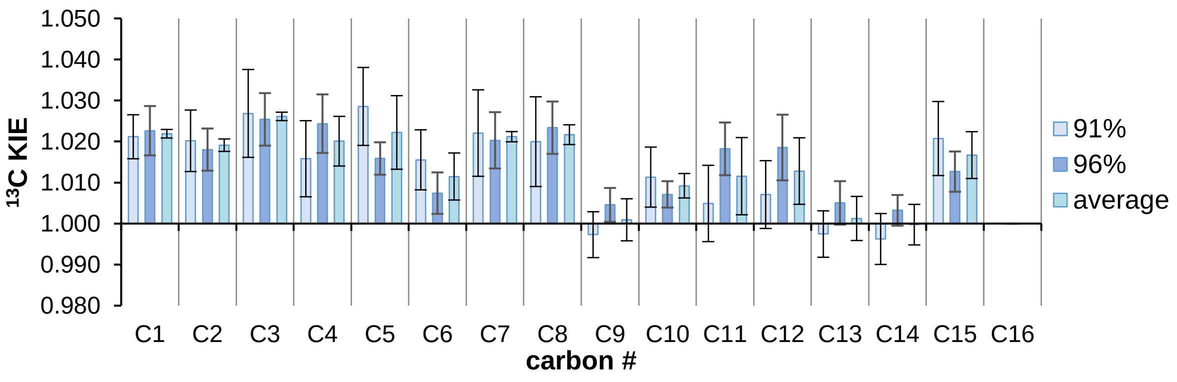

Fig. S 40 | Comparison of the observed KIEs of the first sample (96% conversion of starting material **1a**, blue, middle) and the second sample (91% conversion of starting material **1a**, light blue, left). The average of both measurements is also shown (blue green, right) and the standard deviation (s.d.) is shown.

2.1.3 Discussion of the KIE Results

The KIE data show a clear trend with respect to the observation of small but statistically significant KIEs at all double bond positions. In case of a concerted polyene cyclization, each double bond would be involved in the rate-determining step. As a consequence, a KIE would be expected at each position. It is important to note that the desired product is only obtained in 36% yield, the remaining material being cyclohomofarnesols **3a** and **3c** and traces of homodrimenols **4**. Thus, the formation of each isolated product is reflected in the KIE of the reisolated starting material. The normal KIEs observed at C1, C2, C5, and C15 might be interpreted as a specific interaction of the hydroxy group to another catalyst molecule (or the solvent) and conformational preselection of the substrate. However, further studies, in particular computational analyses, are required to validate the experimental results.

Although the ^{13}C KIE studies at natural abundance provided provisional evidence that protonation and C–C bond formation occur simultaneously, further mechanistic studies were deemed necessary to get a clearer picture of the mechanism. Therefore, deuterium-labeling experiments were carried out which would additionally allow to investigate the selectivity of the initial protonation (or deuteronation) event. Whereas the observation of a single deuteration site would be expected in case of a concerted polyene cyclization, multiple labeled positions would be characteristic for a stepwise process. To scrutinize the developed IDPi-catalyzed polyene cyclization, (3*E*,7*E*)-homofarnesol-d_1 was synthesized (by exchanging the proton at the hydroxy group for deuterium) and subjected to the reaction conditions in PFTB-d_1.

2.2 Deuterium Labeling Studies

To probe the regioselectivity in the initial protonation event and the influence of the catalyst structure on the diastereoselectivity, deuterated (3*E*,7*E*)-homofarnesol-d_1 (**1a-*d*₁**) was subjected to the reaction conditions with IDPi **8g** and PADI **9** in PFTB at −40 °C. The required starting material **1a-*d*₁** (O**D**) was prepared as described below. PFTB-d_1 was prepared according to a procedure reported by Gulder and coworkers.[44]

2.2.1 Synthesis of (3*E*,7*E*)-Homofarnesol-d_1

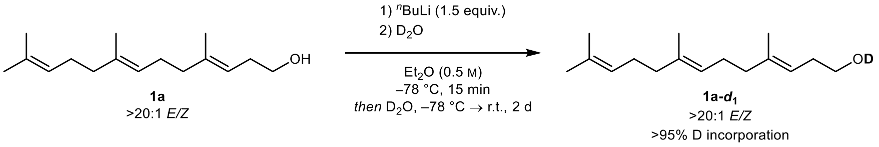

A flame-dried 50 mL Schlenk tube under argon was charged with (3*E*,7*E*)-homofarnesol (1.12 g, 4.75 mmol, 1.0 equiv.) and dry Et_2O (10 mL). The colorless solution was cooled to −78 °C and *n*-butyllithium (2.5 M in hexanes, 2.85 mL, 7.13 mmol, 1.5 equiv.) was added dropwise. After stirring the reaction mixture for 15 min at −78 °C, deuterium oxide (99.96 % D, 11.1 g, 10.0 mL, 553 mmol) was carefully added and the mixture was allowed to warm to room temperature. The resulting biphasic mixture was vigorously stirred (1000 rpm) at room temperature for 2 d and subsequently transferred to an oven-dried separatory funnel under argon. The aqueous phase was separated and the organic phase was washed with deuterium oxide (2 × 10 mL). The phases were separated and the organic phase was concentrated under reduced pressure to afford the crude product as a colorless oil. Purification by vacuum distillation (b.p. 82–84 °C at 3.3–3.4 · 10^{-2} mbar) furnished homofarnesol-d_1 as a colorless oil (>95% D incorporation according to ^{1}H NMR spectroscopy in toluene-d_8, 0.95 g, 4.00 mmol, 84% yield) which was stored in a Schlenk tube under argon to prevent exchange of the deuterium with air moisture.

Physical state: colorless oil.

B.p.: 82–84 °C at 3.3–3.4 · 10^{-2} mbar.

TLC (SiO_2, hexanes/MTBE 19:1, *v/v*): R_f = 0.20 (CAM stain).

^{1}H NMR (600 MHz, toluene-d_8): 5.22–5.16 (m, 2H), 5.10 (tq, *J* = 7.3, 1.4 Hz, 1H), 3.38 (tq, *J* = 6.5, 1.3 Hz, 2H), 2.17–2.09 (m, 6H), 2.07–2.00 (m, 4H), 1.67 (q, *J* = 1.3 Hz, 3H), 1.58 (q, *J* = 1.0 Hz, 3H), 1.57 (s, 3H), 1.54 (q, *J* = 1.0 Hz, 3H).

^{13}C{^{1}H} NMR (151 MHz, toluene-d_8): δ (ppm) = 137.80, 135.19, 131.05, 124.96, 124.60, 121.00, 62.21 (**CH_2OD**), 40.19, 40.18, 32.05 (**CH_2**CH_2**OD**), 27.22, 26.91, 25.81, 17.69, 16.11, 16.07.

^{2}H{^{1}H} NMR (91 MHz, toluene-d_8): δ (ppm) = 0.89 (br s, **OD**)

IR (ATR, neat): $\tilde{\upsilon}_{max}$ (cm^{-1}) = 2965 (m), 2916 (m), 2474 (w, br; **OD**), 1668 (w), 1441 (m), 1377 (m), 1046 (s), 835 (m).

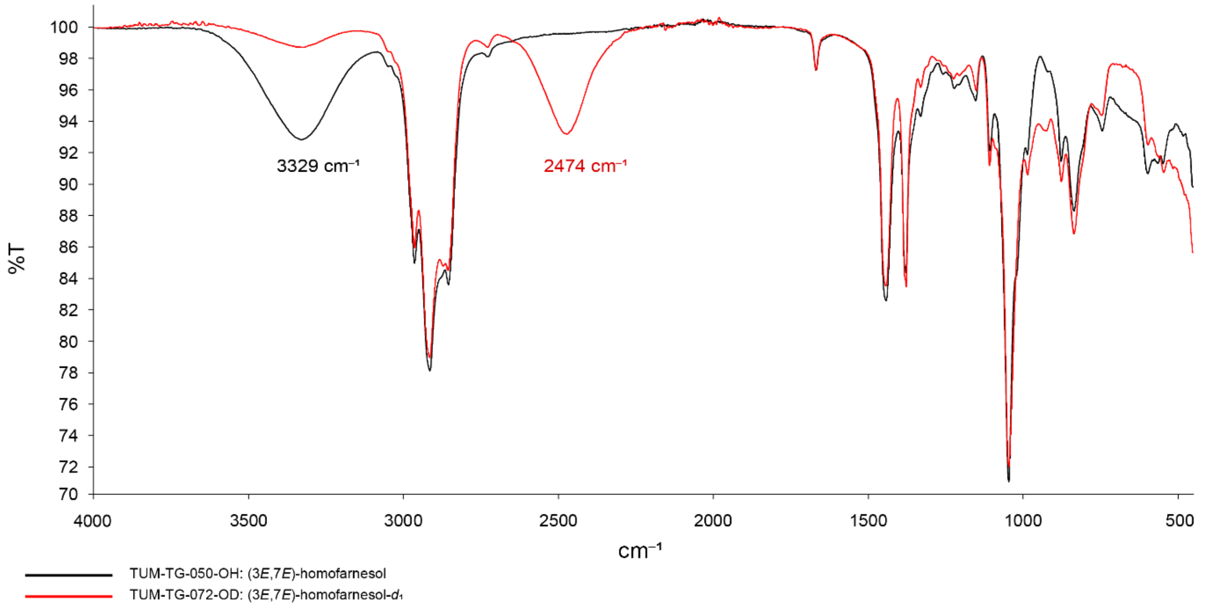

Fig. S41 | Superimposed infrared spectra (IR) of (3*E*,7*E*)-homofarnesol (**1a**, black) and (3*E*,7*E*)-homofarnesol-d$_1$ (**1a**-d$_1$, red).

Determination of the deuterium incorporation was accomplished by comparison of the ^{1}H NMR spectra of **1a** and **1a**-d_1 by comparison of the integral at 0.88–0.92 ppm (OH group) versus the olefinic CH of the terminal prenyl moiety.

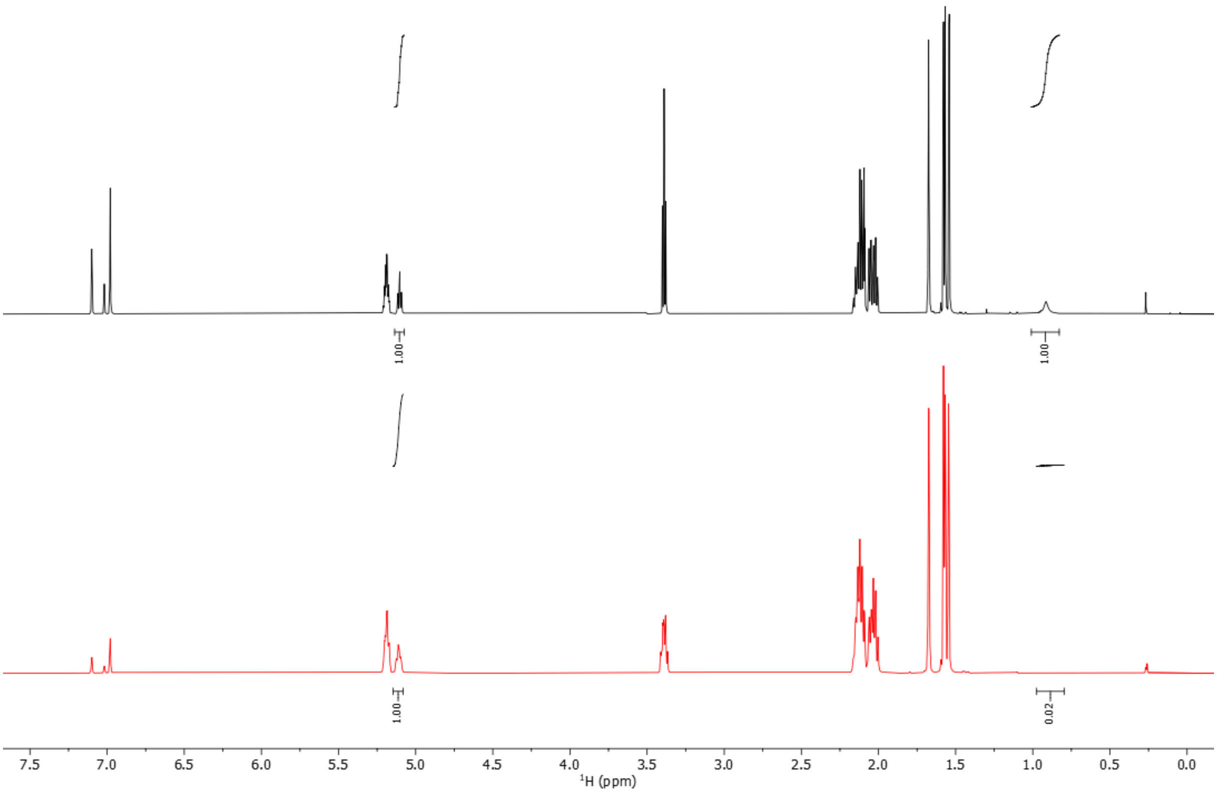

Fig. S42 | Stacked ^{1}H NMR (501 MHz, toluene-d_8) spectra of (3*E*,7*E*)-homofarnesol (**1a**, black, top) and (3*E*,7*E*)-homofarnesol-d_1 (**1a**-d_1, red, bottom) in anhydrous toluene-d_8 with the respective integrals used to estimate the deuterium incorporation.

It is also possible to extract the deuterium incorporation from the ^{13}C NMR spectrum by comparing the ratio of the respective isotopomers $[D_0]$:$[D_1]$. Due to the isotope effect on the chemical shift, an upfield shift of the signal at 62.35 ppm (CH_2-group adjacent to the hydroxy group in **1a**; $[D_0]$, OH) to 62.21 ppm (**1a**-d_1, $[D_1]$, O**D**) in anhydrous toluene-d_8 is observed. Integration of the respective signals directly affords the isotopomer ratio. A comparable, but less pronounced, upfield shift is observed for the neighboring CH_2-group (**C**H_2CH_2OH/**C**H_2CH_2OD) which shifts from 32.10 ppm in **1a** ($[D_0]$, OH) to 32.05 ppm in the deuterated isotopomer **1a**-d_1 ($[D_1]$, O**D**). The procedure for determination of the deuterium incorporation by ^{13}C NMR spectroscopy is illustrated in Fig. S43 for a sample with an isotopomer ratio of approximately 70:30 $[D_1]$:$[D_0]$ (**1a**-d_1:**1a**).

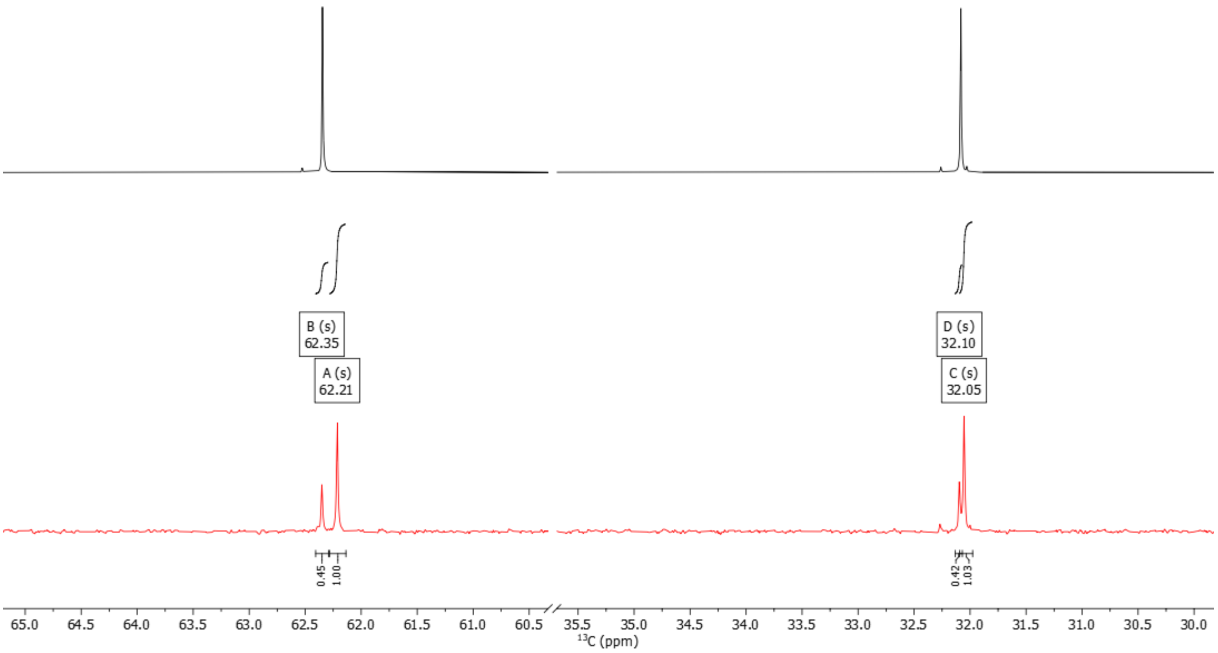

Fig. S43 | Stacked ^{13}C NMR (126 MHz, $CDCl_3$) spectra of (3*E*,7*E*)-homofarnesol (**1a**, black, top) and (3*E*,7*E*)-homofarnesol-d_1 (**1a**-d_1:1a approx. 70:30, red, bottom) in anhydrous toluene-d_8 with the respective integrals used to estimate the deuterium incorporation.

2.2.2 General Procedure for the Deuterium Labeling Experiments

The respective catalyst (2.50 µmol, 0.02 equiv., 2 mol%) and a PTFE-coated magnetic stir bar were transferred to an oven-dried 1.5 mL headspace screw-cap glass vial. The vial was closed with a screw-cap containing a silicone/rubber septum, evacuated, and flushed with argon. Perfluoro-*tert*-butanol-d_1 (PFTB-d_1, 100 µL, 169 mg, 0.71 mmol, 5.7 equiv., 1.25 M) was added and the resulting suspension (with IDPi **8g**) or solution (with PADI **9**) was stirred for 15 min at room temperature. The vial was placed inside an aluminum block on dry-ice and neat (3*E*,7*E*)-homofarnesol-d_1 (**1a-*d*₁**, 34.4 µL, 29.7 mg, 125 µmol, 1.0 equiv.) was transferred to the frozen mixture using an oven-dried Hamilton® syringe flushed with argon. After the addition was complete, the screw-cap was quickly replaced with a new cap under a counterflow of argon. The vial was placed inside a cryostat at −40 °C and stirred for the indicated time. The reaction time was slightly increased compared to the standard reaction conditions to account for the previously described kinetic isotope effect in deuterated PFTB. After the elapsed reaction time (as specified in the individual experiments), triethylamine (20.0 µL, 14.5 mg, 0.14 mmol, 1.1 equiv.) was added, an aliquot was removed, and subsequently analyzed by ^{1}H NMR spectroscopy. The neutralized reaction mixture was concentrated onto Celite® and purified by flash column chromatography on silica gel using hexanes/MTBE (gradient elution: 19:1 → 9:1 → 2:1 *v/v*) as eluent. Depending on the conditions, three fractions were typically obtained: (1) the least polar fraction consisting of partially cyclized **5** and isomerized **10**, (2) a second fraction containing tricyclic ethers and impurities with similar polarity, and (3) a polar fraction containing residual starting material **1a** alongside cyclohomofarnesols **3** and traces of homodrimenols **4**. The purification and characterization of the respective fractions is described in detail in the individual experiments. The isolated fractions were subsequently characterized by ^{1}H, ^{13}C, $^{2}H\{^{1}H\}$, and 2D-NMR spectroscopy.

2.2.3 IDPi-Catalyzed Polyene Cyclization in PFTB-d_1 at −40 °C

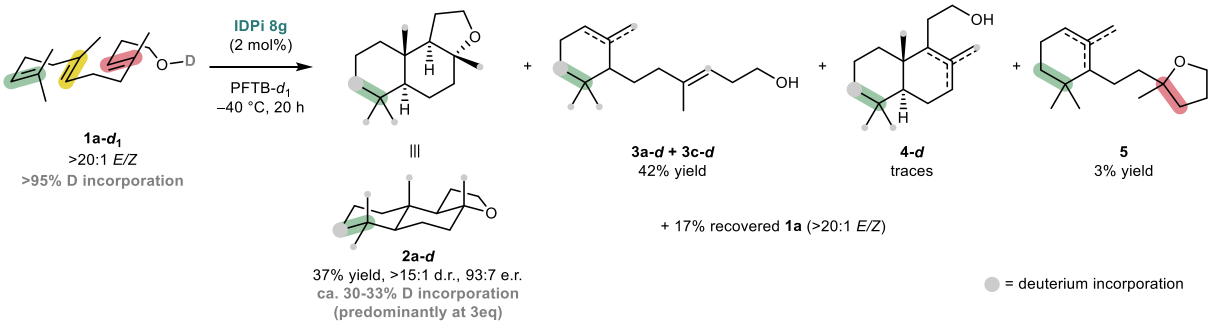

The experiment was performed according to the general procedure using IDPi catalyst **8g** (6.35 mg, 2.50 µmol, 0.02 equiv., 2 mol%) in PFTB-d_1 at −40 °C for 20 h. Purification by flash column chromatography on silica gel (gradient elution with hexanes/MTBE 19:1 → 9:1 → 2:1 *v/v*) afforded three fractions (results are summarized in the table provided below).

Table S15 | Isolated fractions of the IDPi-catalyzed polyene cyclization of **1a**-d_1 in PFTB-d_1 at −40 °C after purification by flash column chromatography.

fraction	R_f (SiO_2, solvent)	*m* / mg	*n* / µmol	yield / %	major identified components
1	0.27 (hexanes/MTBE 19:1 *v/v*, PMA stain)	0.90	3.79	3.0	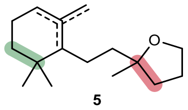 5 3% yield
2	0.13 (hexanes/MTBE 19:1 *v/v*, PMA stain) 0.28 (hexanes/MTBE 9:1 *v/v*, PMA stain)	10.9	45.9	37	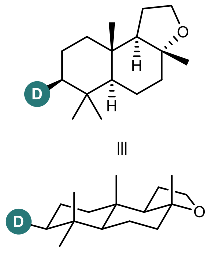 2a-d (–)-ambrox-d
3	0.03 (hexanes/MTBE 19:1 *v/v*, PMA stain) 0.29 (hexanes/MTBE 2:1 *v/v*, PMA stain)	17.5	73.7	59	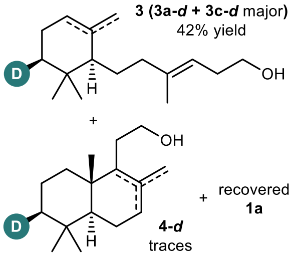 3 (3a-d + 3c-d major) 42% yield + 4-d traces + recovered 1a
Mass balance		29.3	123	99	

Analysis of the Deuterium Incorporation in the Individual Fractions

Analysis of fraction 1:

The least polar fraction (1) contained partially cyclized compound **5** (mixture of isomers). Due to the low isolated yield (<5%), no analysis of the deuterium incorporation was carried out.

Analysis of fraction 2:

Analysis of the second fraction by gas chromatography indicated the presence of (−)-ambrox (**2a**, 94%) as major component alongside minor amounts of (−)-9-*epi*-ambrox (**2b**) and 5β,8α,9β-ambrox (**2c**). The deuterium incorporation in the fraction was analyzed by nuclear magnetic resonance (NMR) spectroscopy, mass spectrometry (MS), and gas chromatography (GC). Both MS and NMR indicated a deuterium incorporation of approximately 33% in the tricyclic ether fraction. For MS analysis, the isotopomer ratio of the individual components was determined by comparison with previously prepared pure product standards (isotopic labeling at natural abundance) as reference material. The determination of the degree of deuterium incorporation by MS was carried out for (−)-ambrox (**2a**) and (−)-9-*epi*-ambrox (**2b**), as all other possible diastereomers are only present in negligible quantities. In addition, reference samples are commercially available or readily accessible by synthesis.

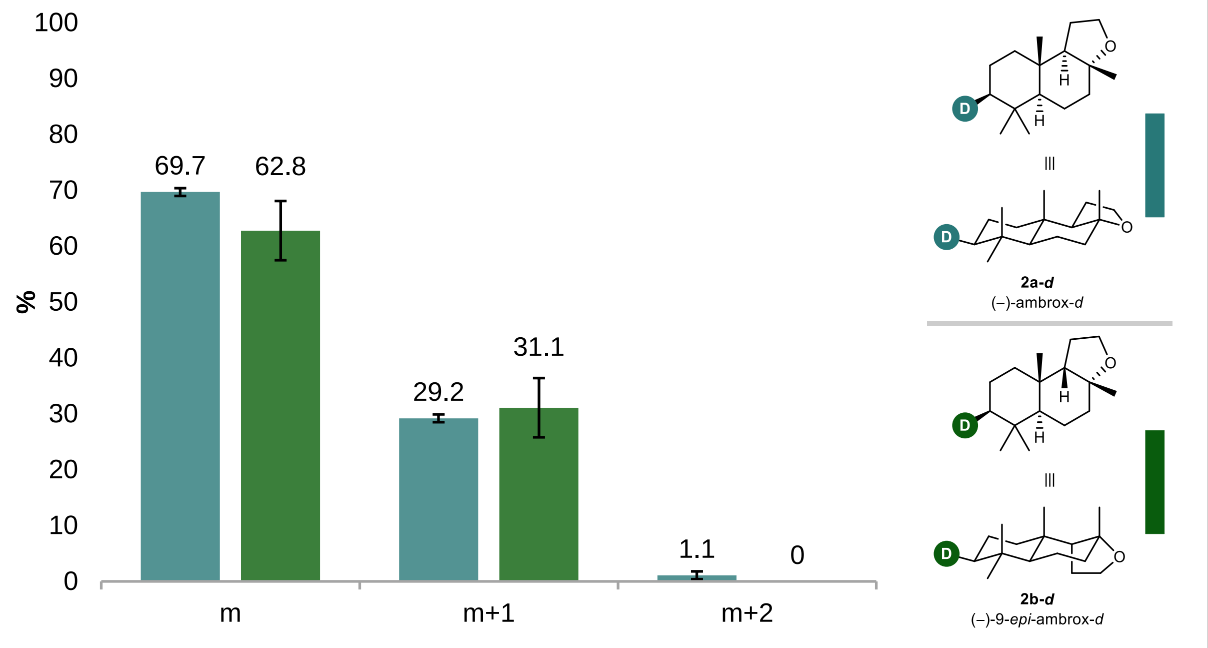

Fig. S44 | Degree of deuteration in (−)-ambrox (**2a**, myrtle green, left) and (−)-9-epi-ambrox (**2b**, dark green, right) as determined by mass spectrometry with the standard deviation shown.

Analysis of the data obtained by MS indicates an approximate isotopomer ratio of $[D_0]:[D_1]:[D_2] \approx$ 63:27:1. The low degree of deuterium incorporation (approximately 33%) could be ascribed to facile exchange of the deuterated hydroxy groups with adventitious water. Another explanation could be a (putatively concerted) deprotonation of initially formed carbocationic intermediates thus generating cyclohomofarnesols **3a** and **3c** alongside protonated IDPi catalyst **8g**. Protonation of starting material with protonated IDPi catalyst **8g** would then provide **2a** without a deuteration site. A significant KIE on the initial protonation event is expected and has been previously observed in a related diastereoselective polyene cyclization in PFTB. Thus, the amount of **2a** formed via protonation with **8g**-d_0 is expected to by higher compared to **2a** formed via deuteration of **8g**-d_1. The high amount of the $[D_1]$-isotopomer (29.2 ± 0.7%) compared to the content of $[D_2]$-isotopomer (1.1 ± 0.7%) is in line with a concerted process wherein a singular deuteronation (or protonation) event induces cyclization to the tricyclic ether.

In case of a stepwise pathway, a higher content of [D_2]- or [D_3]-isotopomers would be expected. Identity and composition of the isotopomers present in the sample were also determined by ^{13}C NMR spectroscopy. The degree of deuteration was established by integration of selected suitable signals (shifted upfield due to isotope effect on the chemical shift) adjacent to the primary site of deuteration (i.e. C3) of the individual isotopomers.

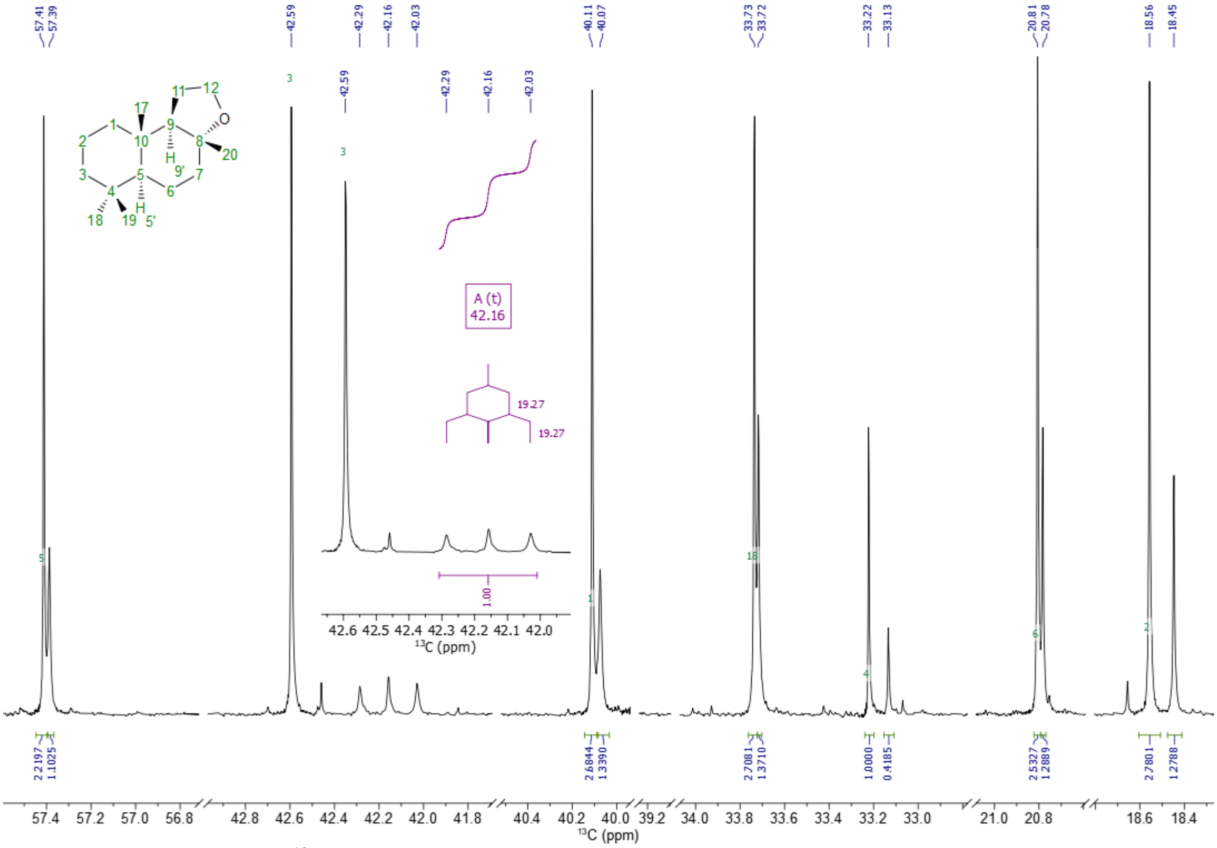

Fig. S45 | Excerpts of the ^{13}C NMR (151 MHz, $CDCl_3$) spectrum of the tricyclic ether fraction with integration regions of interest used for estimating the degree of deuteration in (−)-ambrox (**2a**). The insert shows the characteristic triplet of the CHD group at the major deuteration site (C3).

An isotopomer ratio of approximately 68:32 [D_0]:[D_1] was determined according to the integration of both detected isotopomers. The corresponding degree of deuteration for (−)-ambrox **2a** (32.5 ± 1.7%) is in good agreement with the value determined by MS analysis (29.2 ± 0.7% for the [D_1]-isotopomer).

Table S16 | Determination of the degree of deuteration (−)-ambrox **2a** by ^{13}C NMR spectroscopy. The error bars represent the standard deviation of the investigated positions (n = 6).

^{13}C NMR	Integral		content / %	
Atom	CH_2	CHD	CH_2	CHD
C1	2.6844	1.3390	66.72	33.28
C2	2.7801	1.2788	68.49	31.51
C4	1.0000	0.4185	70.50	29.50
C5	2.2197	1.1025	66.81	33.19
C6	2.5327	1.2889	66.27	33.73
C18	2.7081	1.3710	66.39	33.61
average / %			67.53	32.47
standard deviation / %			1.7	1.7

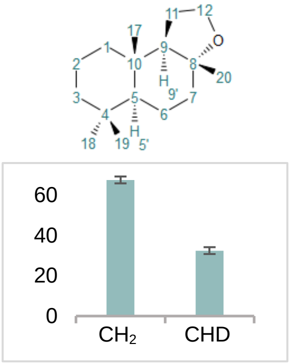

The error was calculated using the sample standard deviation with Bessel's correction according to equations (5) and (6), where s is the sample standard deviation, s^2 the unbiased sample variance, n the sample size, x_i the observed values of the sample items, and $\bar{x}$ denotes the mean value of the observed values.

$$s = \sqrt{s^2} \tag{5}$$

$$s^2 = \frac{1}{(n-1)} \sum_{i=1}^{n} (x_i - \bar{x})^2 \tag{6}$$

Comparison of the ^{1}H and $^{2}H\{^{1}H\}$ NMR spectra as well as the ^{1}H-^{13}C HSQC spectrum permits identification of the exact position of the incorporated deuterium. Examination of the ^{1}H-^{13}C HSQC spectrum revealed that in course of the IDPi-catalyzed polyene cyclization the deuterium labeling occurred essentially at a single site, namely the equatorial position at C3 in (−)-ambrox **2a**.

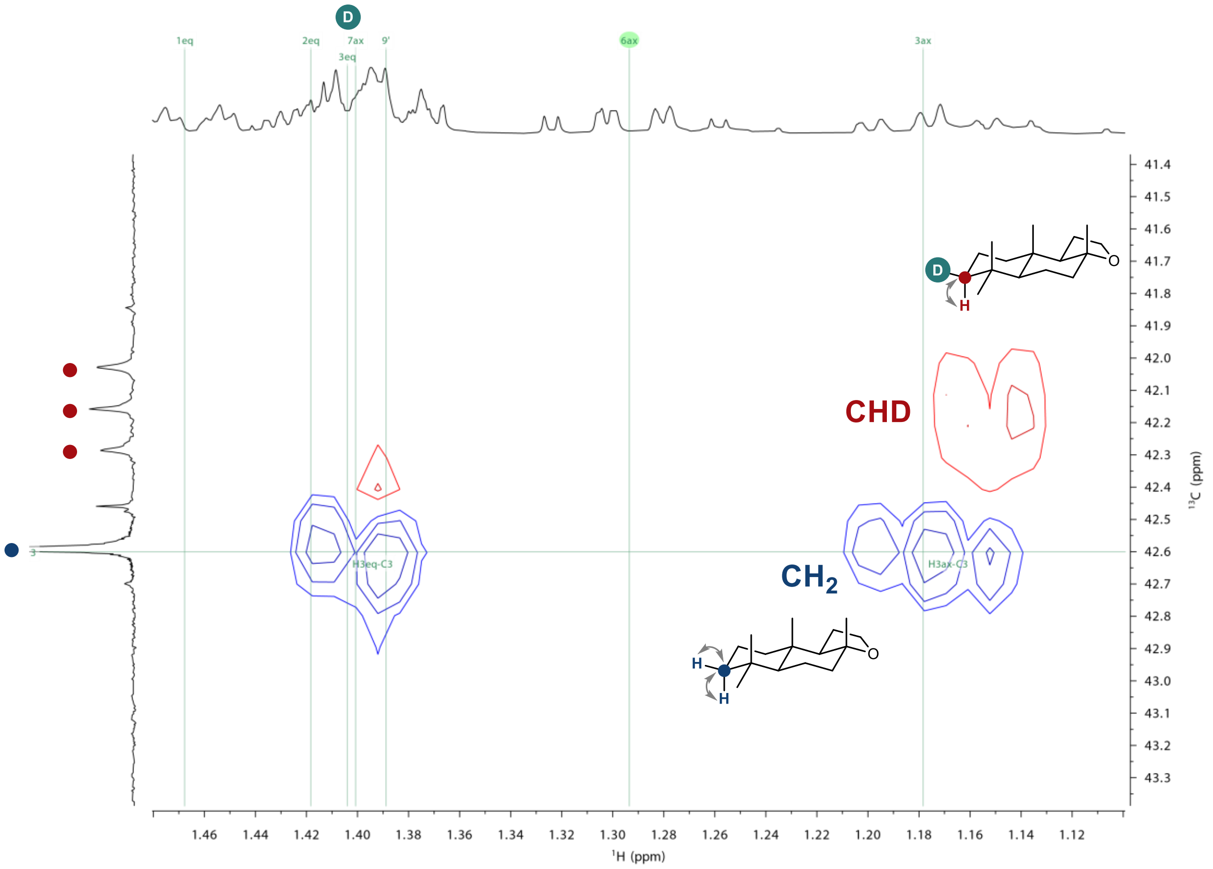

Fig. S46 | Excerpt of the ^{1}H-^{13}C-HSQC (600 MHz, 151 MHz, $CDCl_3$) spectrum of the tricyclic ether fraction showing the major site of deuterium incorporation (C3eq).

The $^{2}H\{^{1}H\}$ NMR spectrum confirms the presence of a major deuteration site at C3eq, alongside minor deuterium incorporation at C3ax (CHD at C3eq:C3ax >40:1), and in the region of the methyl groups C17, C18, C19 and C20. Integration of the respective signals indicates <5% deuterium incorporation at the methyl group positions compared to the major deuteration site at C3. This confirms the hypothesis based on comparison of the isotopomer ratios that (−)-ambrox **2a** is predominantly generated via a concerted reaction pathway at low temperatures. Deuterium incorporation at C18 and C19 (i.e. the

geminal methyl groups at C4) can be ascribed to a (putatively concerted) protonation and deprotonation at C3 providing a 1,1-disubstituted olefin.

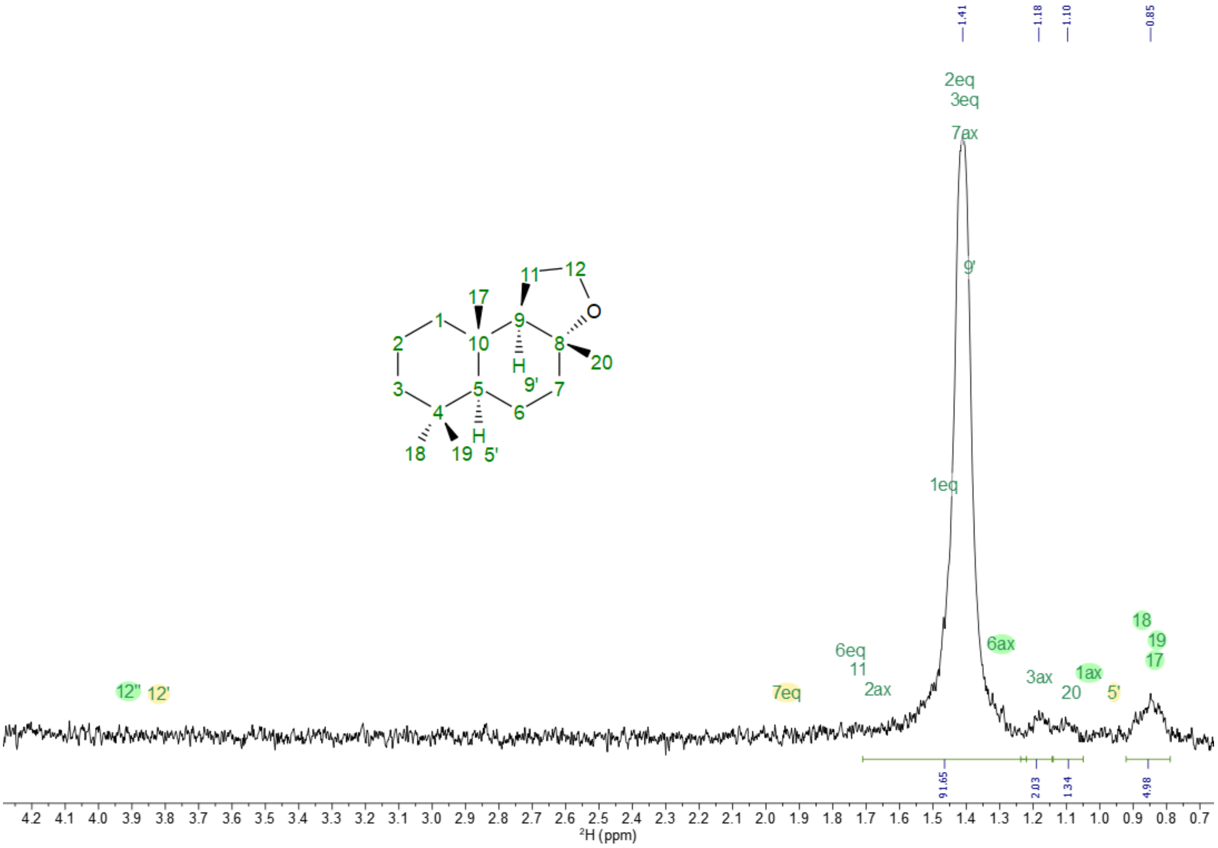

Fig. S47 | Excerpt of the $^2H\{^1H\}$ NMR (92 MHz, $CDCl_3$) spectrum of the tricyclic ether fraction containing **2a**.

Protonation (or deuteronation) of the thus generated double bond isomer would manifest itself in deuterium incorporation at C18 or C19. In contrast to this, deuterium incorporation at C17 and C20 would be characteristic for a pathway via γ-cyclohomofarnesol (**3c**) and γ-homodrimenol (**4c**) respectively. It is worthy of note, that **4c** could indeed be isolated in trace quantities from an experiment on a larger scale, suggesting that a stepwise pathway is viable but most likely less important compared to the proposed concerted cyclization pathway.

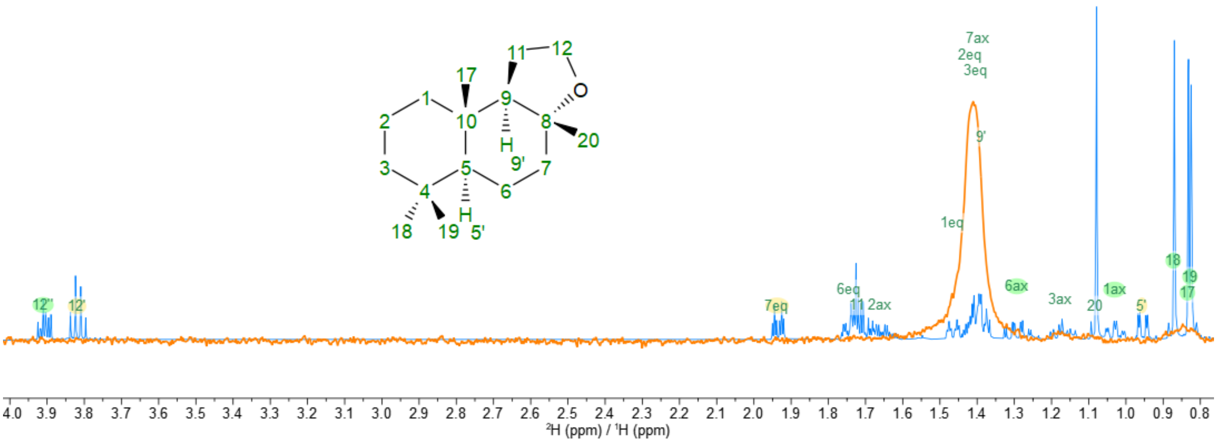

Fig. S48 | Superimposed ^1H (600 MHz, $CDCl_3$, blue) and $^2H\{^1H\}$ NMR (92 MHz, $CDCl_3$, orange) spectra of the tricyclic ether fraction containing **2a**.

Characterization data for a mixture of isotopomers **2a**/**2a**-*d*1 (ca. 67:33):

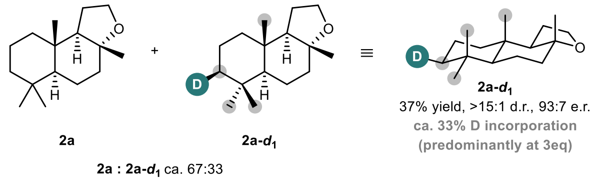

^{1}H NMR (600 MHz, $CDCl_3$): δ (ppm) = 3.93–3.88 (m, 1H), 3.82 (dt, *J* = 8.6, 8.0 Hz, 1H), 1.94 (dt, *J* = 11.6, 3.2 Hz, 1H), 1.77–1.70 (m, 3H), 1.70–1.60 (m, 1H), 1.46 (dt, *J* = 12.7, 3.4 Hz, 1H), 1.45–1.34 (m, 4H), 1.34–1.24 (m, 1H), 1.18 (td, *J* = 14.2, 5.5 Hz, 1H), 1.08 (d, *J* = 1.0 Hz, 3H), 1.03 (td, *J* = 13.1, 3.8 Hz, 1H), 0.95 (dd, *J* = 12.4, 2.7 Hz, 1H), 0.87 (s, 3H), 0.83 (d, *J* = 0.9 Hz, 3H), 0.83 (d, *J* = 0.8 Hz, 3H).
^{13}C{^{1}H} NMR (151 MHz, $CDCl_3$): δ (ppm) = 80.05 (C8, C_q), 65.13 (C12, CH_2), 60.28 (C9, CH), 57.41 (C5, CH), 57.39 (C5, CH; **2a**-*d*1), 42.59 (C3, CH_2), 42.16 (C3, CHD, t, *J* = 19.3 Hz; **2a**-*d*1), 40.11 (C1, CH_2), 40.07 (C1, CH_2; **2a**-*d*1), 39.90 (C7, CH_2), 36.34 (C10, C_q), 33.74 (C18, CH_3), 33.72 (C18, CH_3; **2a**-*d*1), 33.22 (C4, C_q), 33.13 (C4, C_q; **2a**-*d*1), 22.79 (C11, CH_2), 21.29 (C20, CH_3), 21.28 (C19, CH_3), 21.25 (C19, CH_3; **2a**-*d*1), 20.81 (C6, CH_2), 20.78 (C6, CH_2; **2a**-*d*1), 18.56 (C2, CH_2), 18.45 (C2, CH_2; **2a**-*d*1), 15.19 (C17, CH_3).
^{2}H{^{1}H} NMR (92 MHz, $CDCl_3$): δ 1.41 (C3eq, ca. 92%), 1.18 (C3ax, ca. 2%), 1.10 (C20, ca. 1%), 0.87 (C18), 0.84 (C17), 0.82 (19); (<5%); the given percentages refer to the individual contribution to the overall deuterium content.
GC data for **2a** obtained in the deuterium labeling experiment at −40 °C for 20 h in PFTB:
GC (achiral) (Optima-35 0.25/0.25df G/706, 29.0 m; temperature: 220/50 5/min 200 12/min 350, 5 min iso/ 350, 0.60 bar H_2, sample size: 0.2 µL, split ratio: 120:1): 9-*epi*-ambrox: t_R(**2b**) = 26.8/ min (4.00%), 5β-ambrox: t_R(**2e**) = 27.13 min (0.62%), ambrox: t_R(**2a**) = 27.67 min (93.81%), 5β,8α,9β-ambrox: t_R(**2c**) = 28.13 min (1.57%).
Diastereomeric ratio: **2a**:**2b**:**2c**:**2e** = 151 : 6.5 : 2.5 : 1 d.r.; >15:1 d.r. (**2a**).
GC (chiral) (BGB 176/BGB-15 0.25/0.25df G/618, 30.0 m; temperature: 220/140, 60 min iso 8/min 240/ 350, 0.60 bar H_2, sample size: 1.0 µL): (−)-9-*epi*-ambrox: t_R(**2b**) = 41.96 min (3.66%), 5β-ambrox: t_R(**2e**) = 42.38 min (0.09%), (+)-9-*epi*-ambrox, t_R(*ent*-**2b**) = 43.01 min (0.35%), *ent*-5β-ambrox: t_R(*ent*-**2e**) = 43.71 min (0.48%), (−)-ambrox: t_R(**2a**) = 49.17 min (87.11%), (+)-ambrox: t_R(*ent*-**2a**) = 51.20 min (6.91%), 5β,8α,9β-ambrox: t_R(**2c**) = 52.98 min (1.01%), *ent*-5β,8α,9β-ambrox: t_R(*ent*-**2c**) = 54.78 min (0.40%).
Enantiomeric ratios of **2a**, **2b** and **2c**: (−)-ambrox (**2a**): e.r. = 92.5:7.5; (−)-9-*epi*-ambrox (**2b**): e.r. = 91:9; 5β,8α,9β-ambrox: (**2c**): e.r. = 71.5:28.5; 5β-ambrox (**2e**).

Analysis of fraction 3:

The major components in the most polar fraction (3) are cyclohomofarnesols **3** (α-isomer **3a** and γ-isomer **3c**), residual starting material **1a**, as well as traces of homodrimenols **4**. Following the procedure described above, the fraction was analyzed by NMR spectroscopy, MS, and GC. Determination of the degree of deuteration was solely possible for (*E*)-α-cyclohomofarnesol (**3a**) as the fragmentation pattern of the γ-isomer (**3c**) precluded the acquisition of statistically significant data.

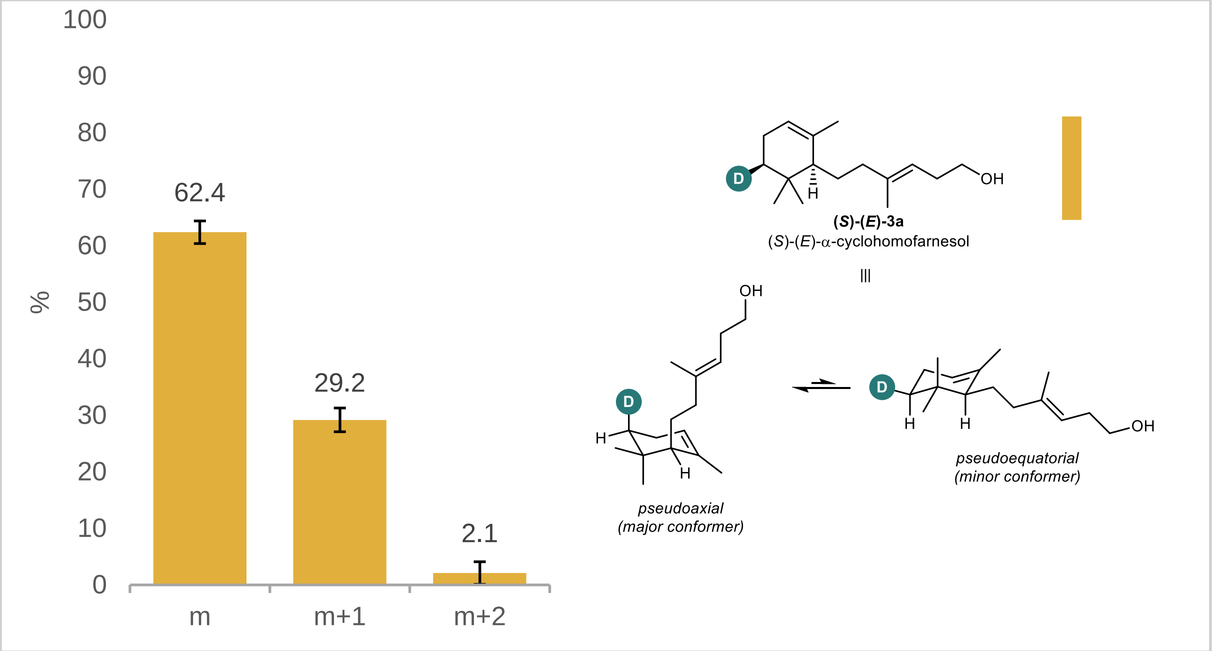

Fig. S 49 | Degree of deuteration in (*S*)-(*E*)-α-cyclohomofarnesol (**3a**) as determined by MS analysis with the standard deviation shown. The major site of deuteration is highlighted in green.

According to MS analysis, the major isotopomers in the reaction mixture are [D_0]-**3a** (62.4 ± 2%) and [D_1]-**3a** (29.2 ± 2%) which is comparable to the isotopomer ratio observed in the tricyclic ether fraction. Following the procedure outlined above for **2a**, the isotopomer ratio was also determined by ^{13}C NMR spectroscopy. Approximately 35% deuterium was located at the axial position (3ax) in (*E*)-α-cyclohomofarnesol (**3a**) based on the [D_0]:[D_1] isotopomer ratios of the adjacent carbon positions.

Table S17 | Degree of deuteration in (*E*)-α-cyclohomofarnesol (**3a**) as determined by ^{13}C NMR spectroscopy. The error bars represent the standard deviation of the investigated positions (n = 2).

^{13}C NMR	Integral		content / %	
**Atom**	**CH_2**	**CHD**	**CH_2**	**CHD**
C2	1	0.5201	65.79	34.21
C4	0.429	0.2376	64.36	35.64
	**average / %**		**65.08**	**34.93**
	**standard deviation / %**		**1.0**	**1.0**

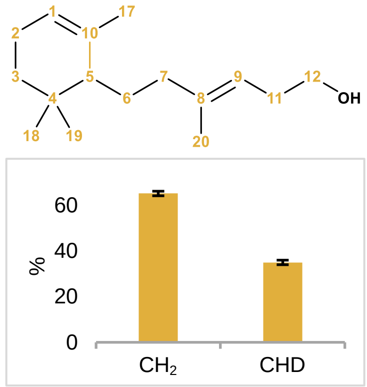

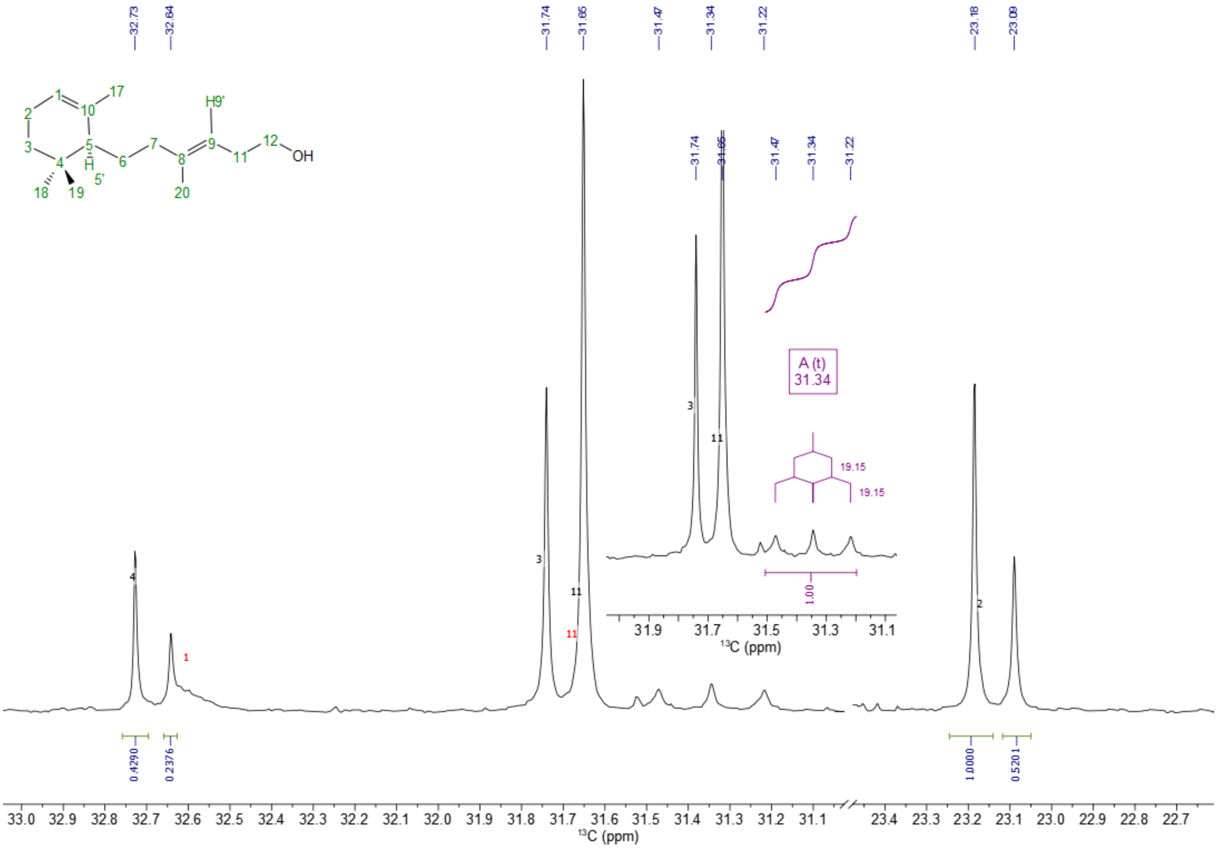

Fig. S50 | Excerpt of the ^{13}C NMR spectrum of a mixture of **3a**:**3c** (ca. 83:17) purified by preparative HPLC. The major deuteration site and integration regions used for estimating the isotopomer ratio of **3a** are shown.

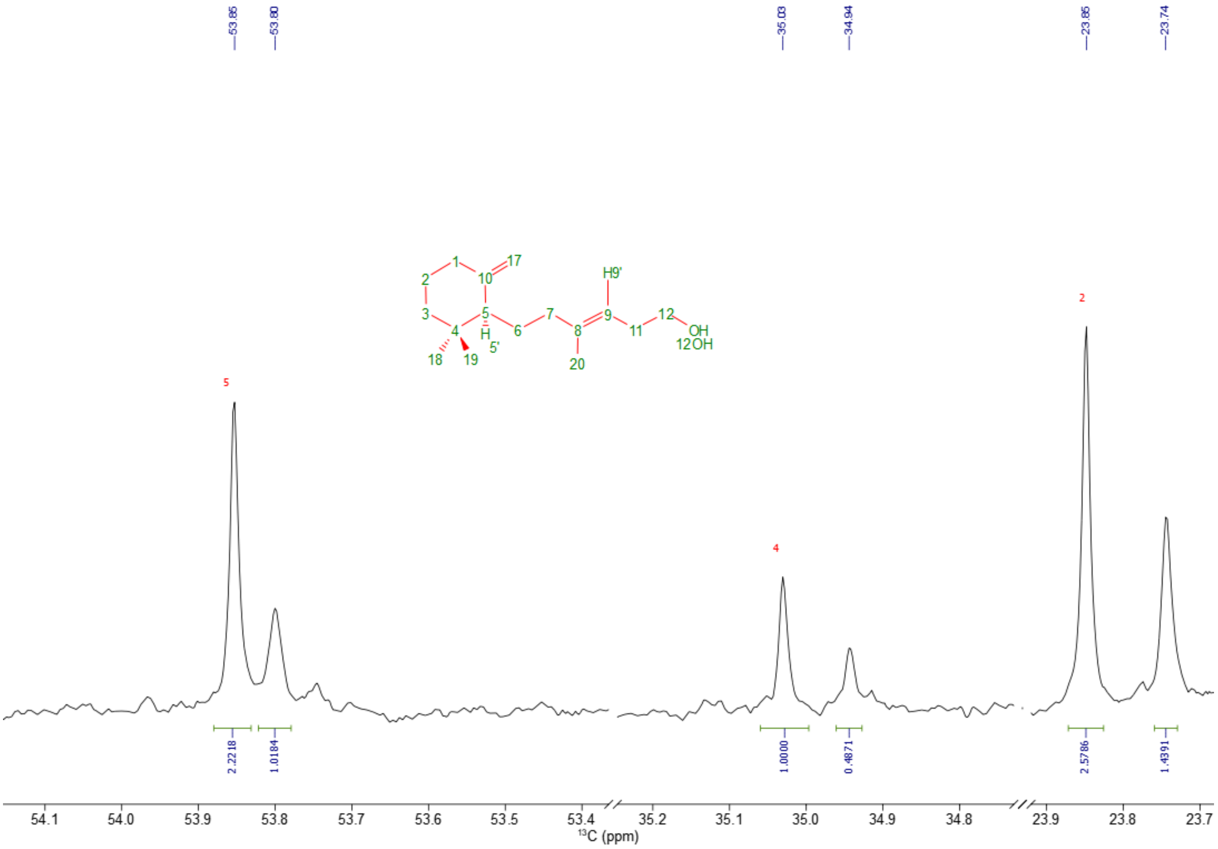

Fig. S51 | Excerpt of the ^{13}C NMR spectrum of a mixture of **3a**:**3c** (ca. 83:17) purified by preparative HPLC. Integration regions used for estimating the isotopomer ratio of **3c** are shown.

The deuterium incorporation for the γ-isomer (**3c**) was estimated analogously by ^{13}C NMR spectroscopy. A deuterium incorporation of roughly 33% at position 3ax was determined.

Table S18 | Determination of the degree of deuteration of **3c** by ^{13}C NMR spectroscopy. The error bars represent the standard deviation of the investigated positions (n = 3).

^{13}C NMR	integral		content / %	
**Atom**	**CH_2**	**CHD**	**CH_2**	**CHD**
C2	2.5786	1.4391	64.18	35.82
C4	1.0000	0.4871	67.24	32.76
C5	2.2218	1.0184	68.57	31.43
	**average / %**		**66.66**	**33.34**
	**standard deviation / %**		**2.3**	**2.3**

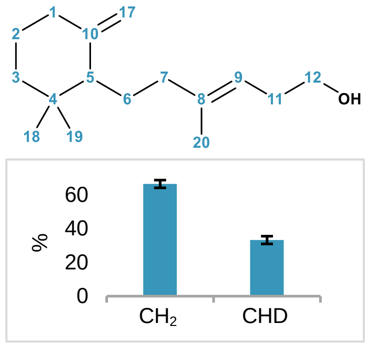

It is important to note that the axial position at C3 (3ax, β-orientation according to the steroid nomenclature, above the ring plane) of the thermodynamically more stable pseudoaxial conformers of **3a** and **3c** (major contributing conformer observed at 298 K by NMR spectroscopy) switches to the equatorial position upon ring flip to the pseudoequatorial conformer (3eq, β-orientation above the ring plan is maintained). The same position (3eq, β-orientation) corresponds to the major deuteration site in (−)-ambrox **2a** (see Fig. S52).

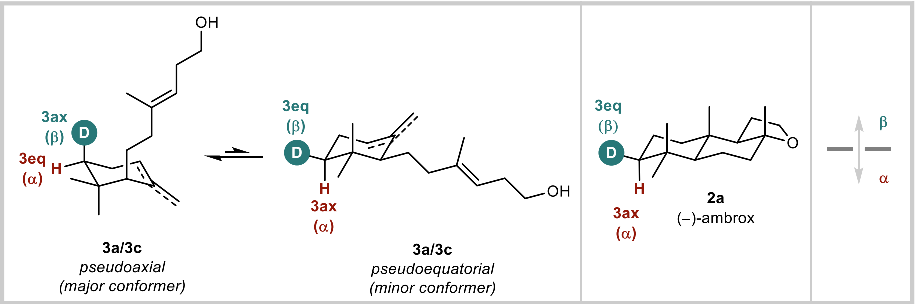

Fig. S52 | Major site of deuteration identified in cyclohomofarnesols (**3a** and **3c**) and (−)-ambrox (**2a**). Labeling scheme according to the steroid nomenclature is provided at the indicated positions.

Superposition of the ^{1}H and ^{2}H{^{1}H} NMR spectra of the polar fraction prior to purification by preparative HPLC shows several minor sites of deuterium incorporation in addition to the major deuteration site at 3ax. Deuterium incorporation at positions 17', 17'' (for **3c**) and 17 (for **3a**) in the ^{2}H{^{1}H} NMR spectrum of the polar fraction additionally reveals that the γ-isomer undergoes an isomerization to the thermodynamically more stable α-isomer via protonation/deuteronation of the exocyclic double bond. By contrast, only trace amounts of deuterium were detected at positions 1 and 1' which confirms that the α-isomer is not a productive intermediate towards (−)-ambrox (**2a**) at low temperatures. Similarly, positions 9 and 9' also contain only a negligible amount of deuterium which further supports the hypothesis that the IDPi catalyst is extraordinarily selective at protonating the desired distal double bond. Traces of homodrimenols (**4**) are also detectable, most importantly α-homodrimenol (**4a**), where deuterium incorporation at the CH-group of the α-olefin is observed. A minor

amount of deuterium is also detectable at the terminal position of the starting material (3*E*,7*E*)-homofarnesol (**1a**) suggesting that a (concerted) protonation and deprotonation of the transient carbocationic intermediate to give a 1,1-disubstituted olefin could occur under the reaction conditions. Separation of residual **1a** from cyclohomofarnesols **3** was accomplished by preparative HPLC. Analysis of recovered **1a** by ^{1}H NMR spectroscopy indicated no isomerization at either double bond position. The absence of any other significant deuterium signals in the ^{2}H{^{1}H} NMR spectrum confirms this observation. This suggests that the initial protonation or deuteronation event of **1a** either effects the formation of monocyclic intermediates **3** or trace amounts of a 1,1-disubstituted olefin by subsequent (putatively concerted) deprotonation of the tertiary carbocation intermediate or a concerted downstream cyclization to (−)-ambrox **2a**.

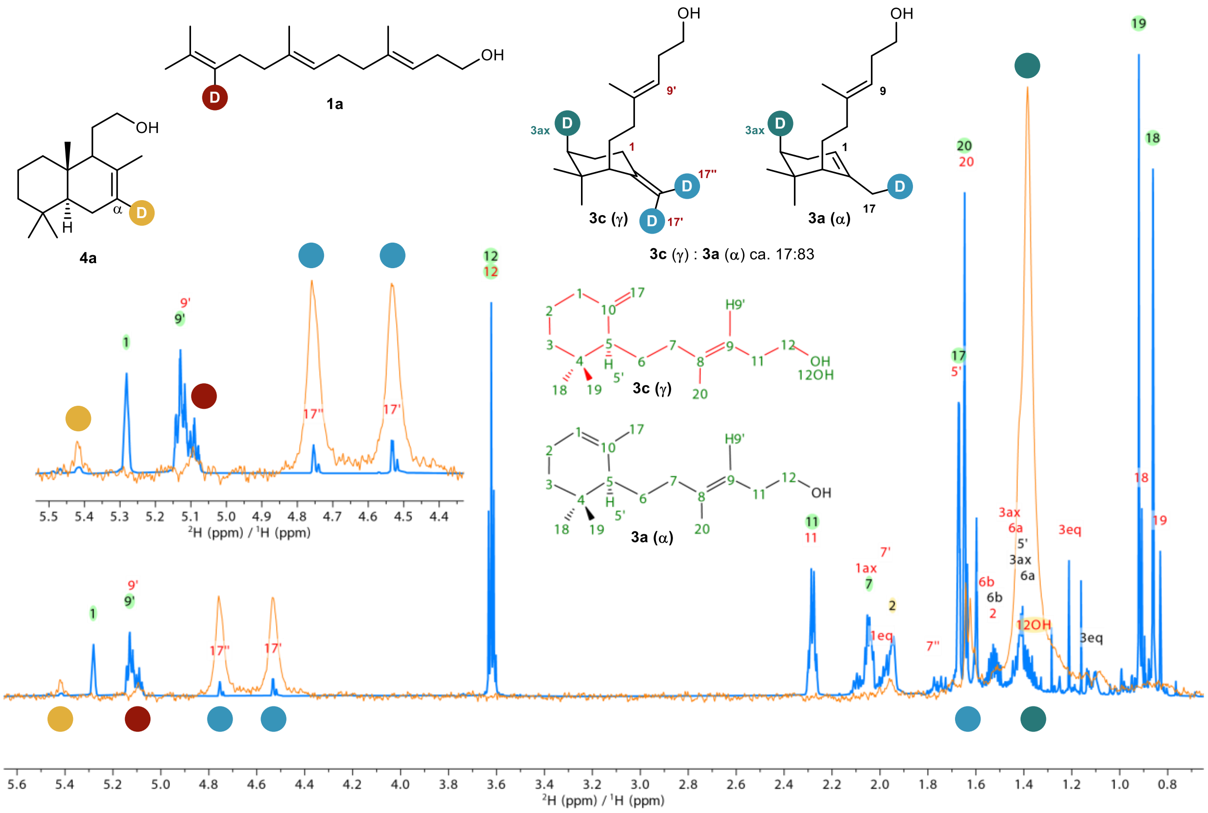

Fig. S53 | Excerpt of superimposed ^{1}H (600 MHz, $CDCl_3$, blue) and ^{2}H{^{1}H} NMR (92 MHz, $CDCl_3$, orange) spectra prior to purification by preparative HPLC. Characteristic deuterated positions are highlighted and color-coded according to the structures inserted in the spectrum.

2.2.4 PADI-Catalyzed Polyene Cyclization in PFTB-d_1 at −40 °C

The experiment was performed according to the general procedure using PADI catalyst **9** (3.53 mg, 2.50 µmol, 0.02 equiv., 2 mol%) in PFTB at −40 °C for 20 h. Purification by flash column chromatography on silica gel (gradient elution with hexanes/MTBE 19:1 → 9:1 → 2:1 *v/v*) afforded three fractions. Due to difficulties encountered in the separation of PADI catalyst **9** from residual starting material and monocyclic intermediates, solely the tricyclic ether fraction was analyzed by ^{1}H, ^{2}H{^{1}H} and ^{13}C NMR spectroscopy.

Table S19 | Isolated fractions of the PADI-catalyzed polyene cyclization of **1a**-d_1 in PFTB-d_1 at −40 °C after purification by flash column chromatography.

fraction	R_f (SiO$_2$, solvent)	*m* / mg	*n* / µmol	yield / %	major identified components
1	0.27 (hexanes/MTBE 19:1 *v/v*, PMA stain)	0.80	3.37	2.7	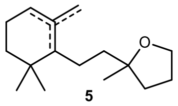 **5** 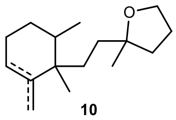 **10**
2	0.13 (hexanes/MTBE 19:1 *v/v*, PMA stain)<br>0.28 (hexanes/MTBE 9:1 *v/v*, PMA stain)	13.3	56.0	45	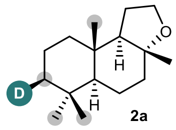 **2a** 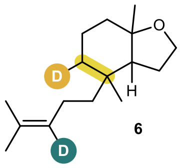 **6**
3	0.03 (hexanes/MTBE 19:1 *v/v*, PMA stain)<br>0.29 (hexanes/MTBE 2:1 *v/v*, PMA stain)	6.70	28.2	23	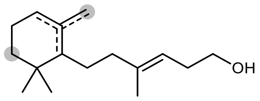 **3** 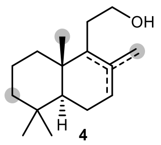 **4** 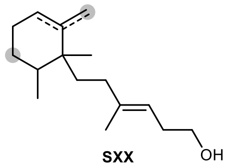 **SXX**
Mass balance		20.8	87.6	71	

Analysis of the tricyclic ether fraction (2) obtained with PADI-catalyst **9** at −40 °C in PFTB by MS indicated a higher amount of [D$_1$] and [D$_2$] isotopomers of **2a** compared to the product fraction obtained with IDPi-catalyst **8g**. The higher degree of deuterium incorporation, in particular the increase of the

[D_2] isotopomer, can be ascribed to a stepwise process which involves more than one deuteronation (or protonation) step to provide the desired product. Any monocyclic intermediates such as cyclohomofarnesols **3** thus appear to be susceptible to protonation (or deuteronation) by the PADI catalyst, even at low reaction temperatures. The higher amount of [D_1]-isotopomer (36.9% ± 0.7%) observed with PADi **9** compared to IDPi **8g** (29.2% ± 0.7%) could also be explained by the lower basicity of **9** compared to **8g** which would result in less deprotonation and consequently less exchange to the protonated catalyst. Assuming a KIE in the initiation step, the protonated catalyst would most likely convert **1a** with a higher rate compared to the deuterated catalyst resulting in a lower amount of [D_1]-isotopomer.

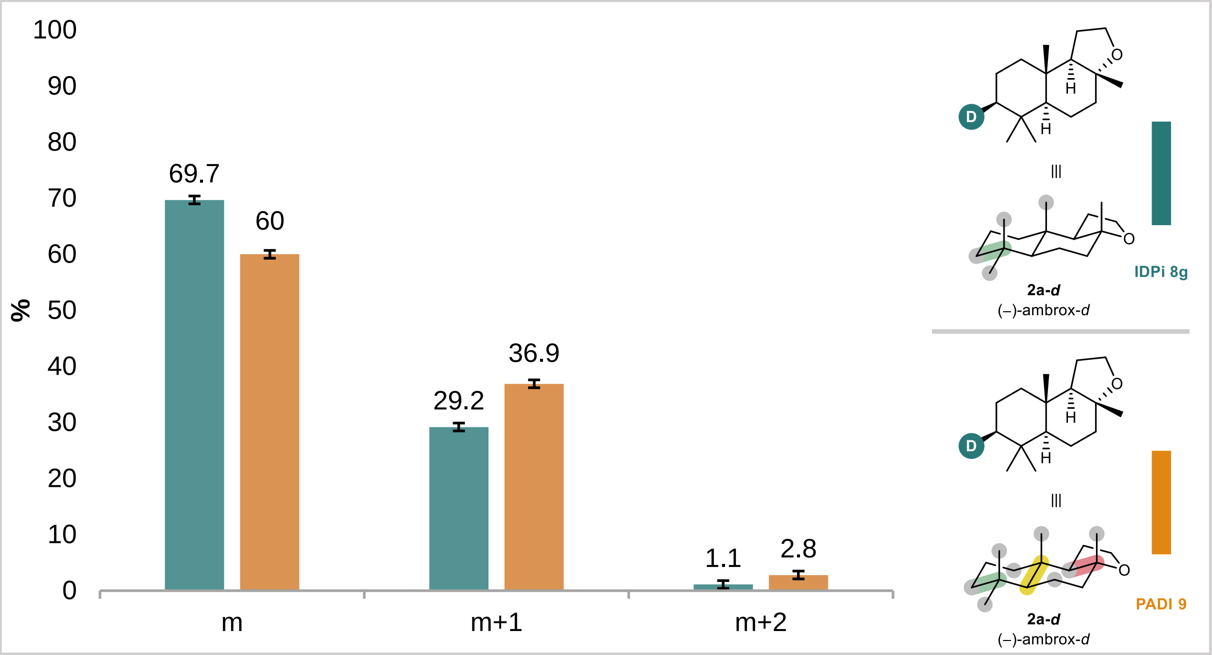

Fig. S54 | Degree of deuteration in (−)-ambrox (**2a**) obtained at −40 °C in PFTB in the presence of IDPi catalyst **8g** (myrtle green, left) or PADI catalyst **9** (orange, right) as determined by mass spectrometry with the standard deviation shown.

A comparable increase of the [D_1]-isotopomer (39.4 ± 1.1%) in the PADI-catalyzed polyene cyclization of **1a** compared to the IDPi-catalyzed reaction (32.5 ± 1.7%) was observed by ^{13}C NMR spectroscopy.

Table S20 |Determination of the degree of deuteration by ^{13}C NMR spectroscopy for (−)-ambrox **2a** obtained in the PADI-catalyzed polyene cyclization of **1a** at −40 °C in PFTB. The error bars represent the standard deviation of the three investigated positions (n = 5).

^{13}C NMR	Integral		content / %	
**atom**	**CH_2**	**CHD**	**CH_2**	**CHD**
**C1**	0.8507	0.5701	59.9	40.1
**C2**	1	0.6154	61.9	38.1
**C4**	0.3174	0.22	59.1	40.9
**C5**	0.8315	0.52	61.5	38.5
**C6**	0.9145	0.5966	60.5	39.5
**average / %**			**60.6**	**39.4**
**error / %**			**1.1**	**1.1**

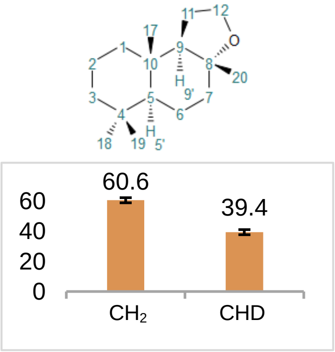

Detailed analysis of the ether fraction obtained with PADI catalyst **9** led to the identification of a partially cyclized side product (**6**) that arises from undesired protonation (or deuteronation) of the internal double bond in **1a**. The absence of **6** in the IDPi-catalyzed reaction polyene cyclization showcases the regioselectivity of the IDPi imparted by its confined active site for protonating most accessible distal double bond. Partial characterization data for side product **6** are provided below. The proposed structure is plausible based on 2D NMR analysis. Due to multiple signal overlaps (including partially deuterated species) some of the assignments may be incorrect.

Table S21 | Tentative assignment of side product **6** based on ^{1}H, ^{13}C, and 2D NMR spectroscopic data.

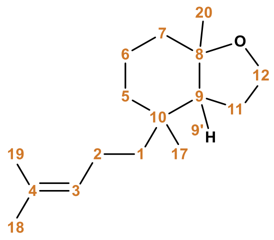

Atom	δ (ppm)	Predicted Shift	HSQC	HMBC
1 C	45.142	41.17	1', 1"	2, 3
H'	1.287		1	17
H"	1.180		1	17
2 C	22.156	23.23	2	3
H2	1.929		2	1, 3, 4
3 C	125.113	124.95	3	2, 18, 19
H	5.074		3	1, 2, 18, 19
4 C	131.264	131.39		2, 18, 19
5 C	37.538	39.62	5', 5"	
H'	1.441		5	
H"	1.159		5	
6 C	21.122	18.98	6', 6"	
H'	1.493		6	
H"	1.696		6	
7 C	38.725	39.22	7', 7"	20
H'	1.305		7	
H"	1.853		7	
8 C	80.041	83.66		20

Atom	δ (ppm)	Predicted Shift	HSQC	HMBC
9 C	55.502	53.19	9'	20
9' H	1.372		9	
10 C	35.807	36.49		
11 C	23.726	25.95	11"	
H'	1.696			
H"	1.810		11	
12 C	64.155	67.34	12', 12"	
H'	3.854		12	
H"	3.759		12	
17 C	17.817	20.90		1', 1"
H3	0.829			
18 C	25.840	24.30	18	3, 19
H3	1.673		18	3, 4, 19
19 C	17.718	19.18	19	3, 18, 19
H3	1.592		19	3, 4, 18, 19
20 C	20.526	21.15	20	
H3	1.082		20	7, 8, 9

Partial characterization data for side product **6** (mixture of isotopomers):

^{1}H NMR (600 MHz, $CDCl_3$): δ (ppm) = 5.07 (thept, J = 7.2, 1.5 Hz, C3-H, 1H), 3.85 (C12-H', 1H), 3.76 (C12-H", 1H), 1.93 (C2-H_2, 2H), 1.85 (C7-H", 1H), 1.81 (C11-H", 1H), 1.70 (C-6H", 1H), 1.70 (C11-H', 1H), 1.67 (C18-H_3, 3H), 1.59 (C19-H_3, 3H), 1.49 (C6-H', 1H), 1.44 (C5-H', 1H), 1.37 (C9-H', 1H), 1.31 (C7-H', 1H), 1.29 (C1-H', 1H), 1.16 (C5-H", 1H), 1.18 (C1-H", 1H), 1.08 (C20-H_3, 3H), 0.83 (C17-H_3, 3H).

^{13}C{^{1}H} NMR (151 MHz, $CDCl_3$): δ (ppm) = 131.3 (C4), 125.1 (C3), 80.0 (C8), 64.2 (C12), 55.5 (C9), 45.1 (C1), 38.7 (C7), 37.5 (C5, CH_2), **37.1 (t, $^{1}J_{C-D}$ = 19.5 Hz, CHD)**, 35.8 (C10), 25.8 (C18), 23.7 (C11), 22.2 (C2), 21.1 (C6), 20.5 (C20), 17.8 (C18), 17.7 (C19).

^{2}H{^{1}H} NMR (92 MHz, $CDCl_3$): δ (ppm) = 5.12 (C3-D), 1.45 (C5-D'), 1.16 (C5-D").

GC data for a mixture of tricyclic ethers **2** and partially cyclized compound **6**:

GC (achiral) (Optima-35 0.25/0.25df G/706, 29.0 m; temperature: 220/50 5/min 200 12/min 350, 5 min iso/ 350, 0.60 bar H_2, sample size: 0.2 µL, split ratio: 120:1): partially cyclized product **6**: t_R(minor) = 26.37 min (4.27%), t_R(major) = 26.80 min (13.21%), 9-*epi*-ambrox: t_R(**2b**) = 26.88 min (7.09%), 5β-ambrox: t_R(**2e**) = 27.14 min (4.02%), ambrox: t_R(**2a**) = 27.68 min (64.82%), 5β,8α,9β-ambrox: t_R(**2c**) = 28.14 min (6.61%).

Chemoselectivity (**2**:**6**): 82.5:17.5; isomeric ratio: **2a**:**2b**:**2c**:**2e** = 16 : 1.8 : 1.6 : 1 d.r.; ca. 79:21 d.r. (**2a**).

GC (chiral) (BGB 176/BGB-15 0.25/0.25df G/618, 30.0 m; temperature: 220/140, 60 min iso 8/min 240/ 350, 0.60 bar H_2, sample size: 1.0 µL): (−)-9-*epi*-ambrox: t_R(**2b**) = 41.92 min (5.22%), 5β-ambrox: t_R(**2e**) = 42.37 min (1.43%), (+)-9-*epi*-ambrox, t_R(*ent*-**2b**) = 42.96 min (3.80%), partially cyclized side product t_R(**6**, minor) = 46.69 min (3.68%), t_R(**6**, major) = 47.48 min (9.79%), (−)-ambrox: t_R(**2a**) = 49.04 min (39.94%), (+)-ambrox: t_R(*ent*-**2a**) = 51.19 min (27.51%), 5β,8α,9β-ambrox: t_R(**2c**) = 52.94 min (3.49%), *ent*-5β,8α,9β-ambrox: t_R(*ent*-**2c**) = 54.73 min (2.80%).

Enantiomeric ratios of **2a**, **2b**, **2c**, **2e**, and **6**: (−)-ambrox (**2a**): e.r. = 59:41; (−)-9-*epi*-ambrox (**2b**): e.r. = 57.5:42.5; 5β,8α,9β-ambrox: (**2c**): e.r. = 55.5:44.5; partially cyclized side product **6**: e.r. = 27.5:72.5.

Compared to the $^2H\{^1H\}$ NMR (92 MHz, $CDCl_3$) spectrum of the tricyclic ether fraction obtained with IDPi-catalyst **8g**, the product fraction obtained from the experiment with PADI catalyst **9** shows deuterium incorporation at multiple positions, some of which are characteristic for a stepwise pathway involving protonation of cyclohomofarnesols (e.g. D incorporation at position 17, 20, 7, 9'). The low chemoselectivity in the initial protonation event of PADI catalyst **9** results in a lower mass balance (71%) compared to the reaction performed with IDPi catalyst **8g** (99%). Furthermore, the ability of PADI catalyst **9** to protonate monocyclic intermediates at low temperatures leads to the formation of multiple undesired diastereomers which is reflected by the significantly diminished diastereoselectivity compared to the reaction performed with IDPi **8g** (ca. 94:6 d.r. in favor of **2a** in case of the IDPi-catalyzed reaction and ca. 79:21 d.r. in favor of **2a** when PADI **9** was used).

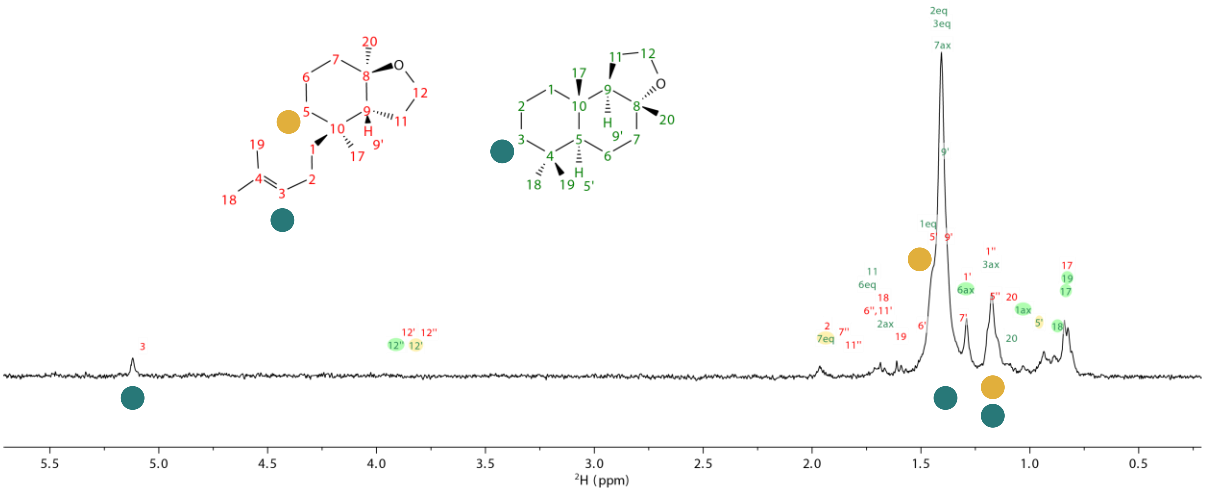

Fig. S55 | $^2H\{^1H\}$ NMR (92 MHz, $CDCl_3$) spectrum of the ether fraction obtained with PADI catalyst **9** at −40 °C in PFTB.

2.2.5 Discussion of the Results

The deuterium labeling experiments clearly highlight the importance of a confined active site to obtain high selectivities towards the desired product in the catalytic asymmetric polyene cyclization of (3*E*,7*E*)-homofarnesol (**1a**) to (−)-ambrox (**2a**). Based on the MS and NMR analyses for both samples obtained at −40 °C in PFTB, it can be concluded that IDPi-catalyst **8g** predominantly converts **1a** into **2a** through a concerted polyene cyclization, wherein a single protonation (or deuteronation) event triggers the cyclization cascade and formation of the desired product. The protonation (or deuteronation) of monocyclic intermediates (i.e. cyclohomofarnesols **3**), formed upon (putatively concerted) deprotonation of a transient carbocationic species by the IDPi counteranion, appears to be less significant at −40 °C. The confined active site thus enables the preferential protonation of the most accessible distal double bond over the more sterically encumbered double bonds of the cyclohexene scaffold present in the cyclohomofarnesols. In contrast to this, the less confined PADI catalyst **9** catalyzes the formation of **2a** via cyclohomofarnesols to a significant extent, which results in a decreased diastereoselectivity. Furthermore, the isolation of a side product **6** formed upon protonation (or deuteronation) of the internal double bond showcases the lower regioselectivity of PADI **9** in the initial substrate protonation (or deuteronation) compared to IDPi **8g** which did not generate detectable quantities of **6**.

2.3 Kinetic Studies

2.3.1 Qualitative NMR Study: Time-Dependent Conversion in HFIP

To gain qualitative mechanistic insights into the IDPi-catalyzed polyene cyclization of **1a** in HFIP/1*H*,1*H*-perfluorooctan-1-ol, the reaction was monitored over time by NMR spectroscopy.

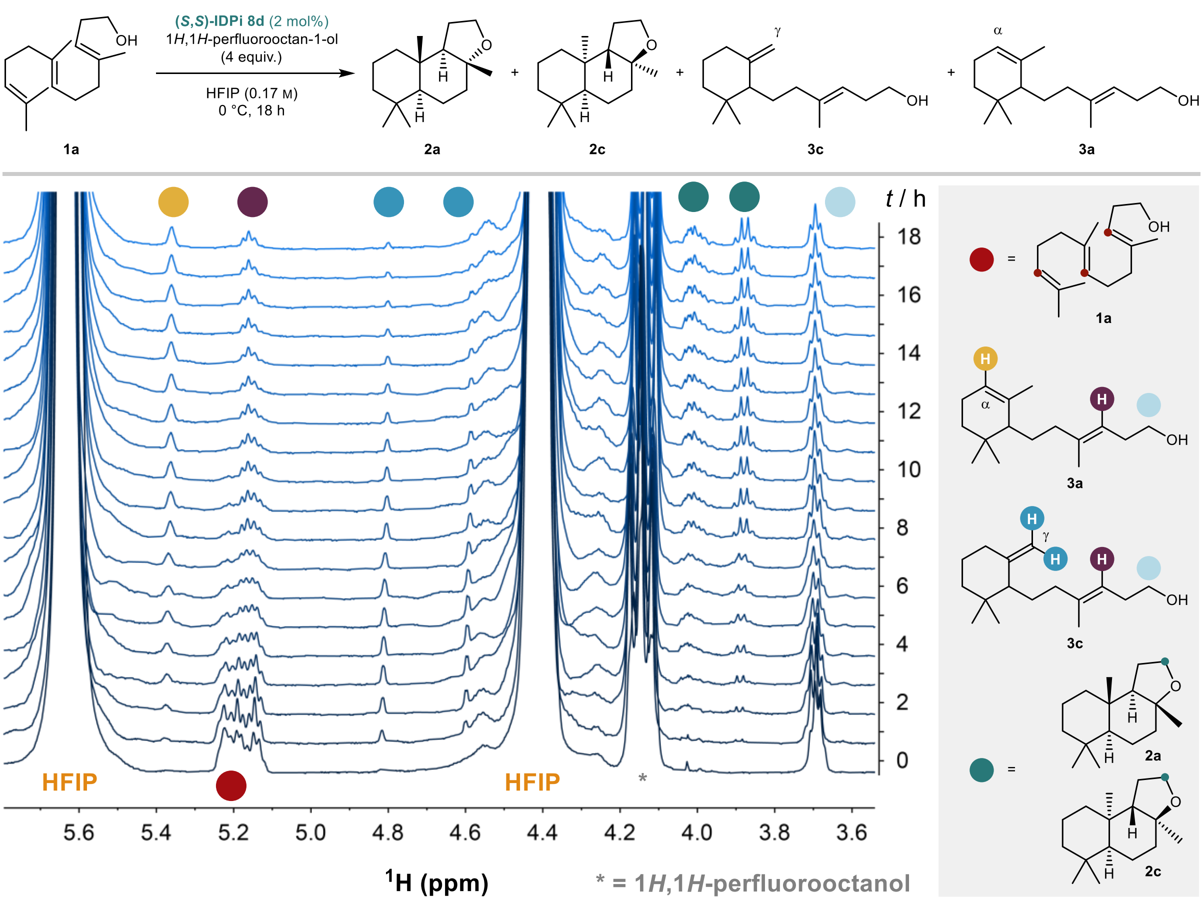

Fig. S56 | Excerpt of stacked ^{1}H NMR (500 MHz, lock on acetone-d_6) spectra of the IDPi-catalyzed polyene cyclization of **1a** in HFIP/1*H*,1*H*-perfluorooctan-1-ol at 0 °C.

(*S*,*S*)-IDPi catalyst **8d** (4.87 mg, 2.00 µmol, 2 mol%) was transferred to a 5 mm Norell® NMR tube. The NMR tube was sealed with a rubber septum, evacuated and flushed with argon (3 ×) and a solution of 1*H*,1*H*-perfluorooctan-1-ol (160 mg, 400 µmol, 4 equiv.) in 1,1,1,3,3,3-hexafluoropropan-2-ol (HFIP, 400 µL) was transferred via a Hamilton® syringe to the NMR tube, followed by additional HFIP (200 µL). The NMR tube was immersed in a cooling bath at −72 °C (dry-ice/ethanol) and neat (3*E*,7*E*)-homofarnesol (**1a**, 27.0 µL, 23.3 mg, 98.6 µmol, 1.0 equiv.) was added via Hamilton® syringe. The NMR tube was removed from the cooling bath, gently warmed until the frozen reaction mixture liquified, and vortexed to ensure complete dissolution of catalyst and substrate. A coaxial insert filled with acetone-d_6 (200 µL) for locking was introduced, and the sample was placed inside a 500 MHz NMR spectrometer cooled to 0 °C. ^{1}H spectra were acquired periodically to analyze the reaction progress. The concentrations of the respective components were estimated by integration of regions with characteristic signals and (if possible) with minimal overlap.

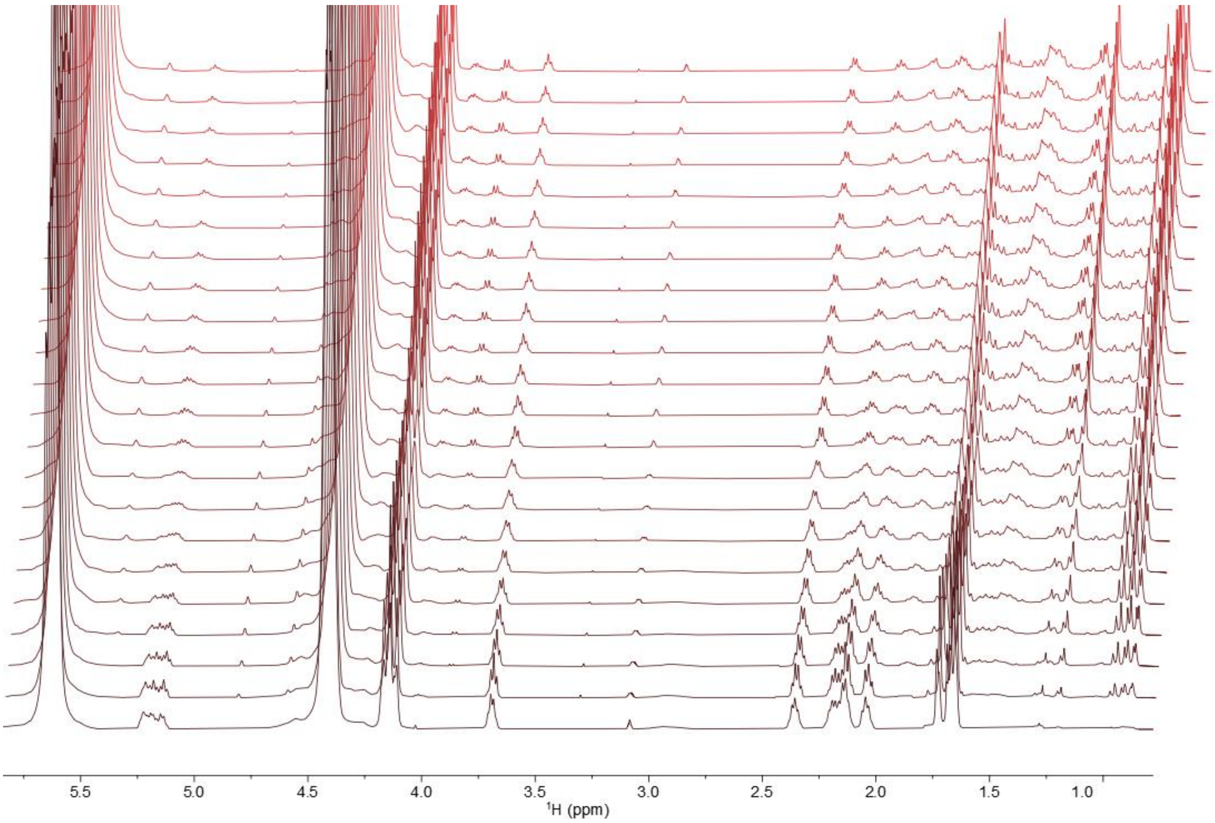

Fig. S57 | Excerpt of stacked ^{1}H NMR spectra (full region of interest) acquired during the polyene cyclization of **1a** in HFIP/1*H*,1*H*-perfluorooctan-1-ol at 0 °C catalyzed by (*S*,*S*)-IDPi catalyst **8d**.

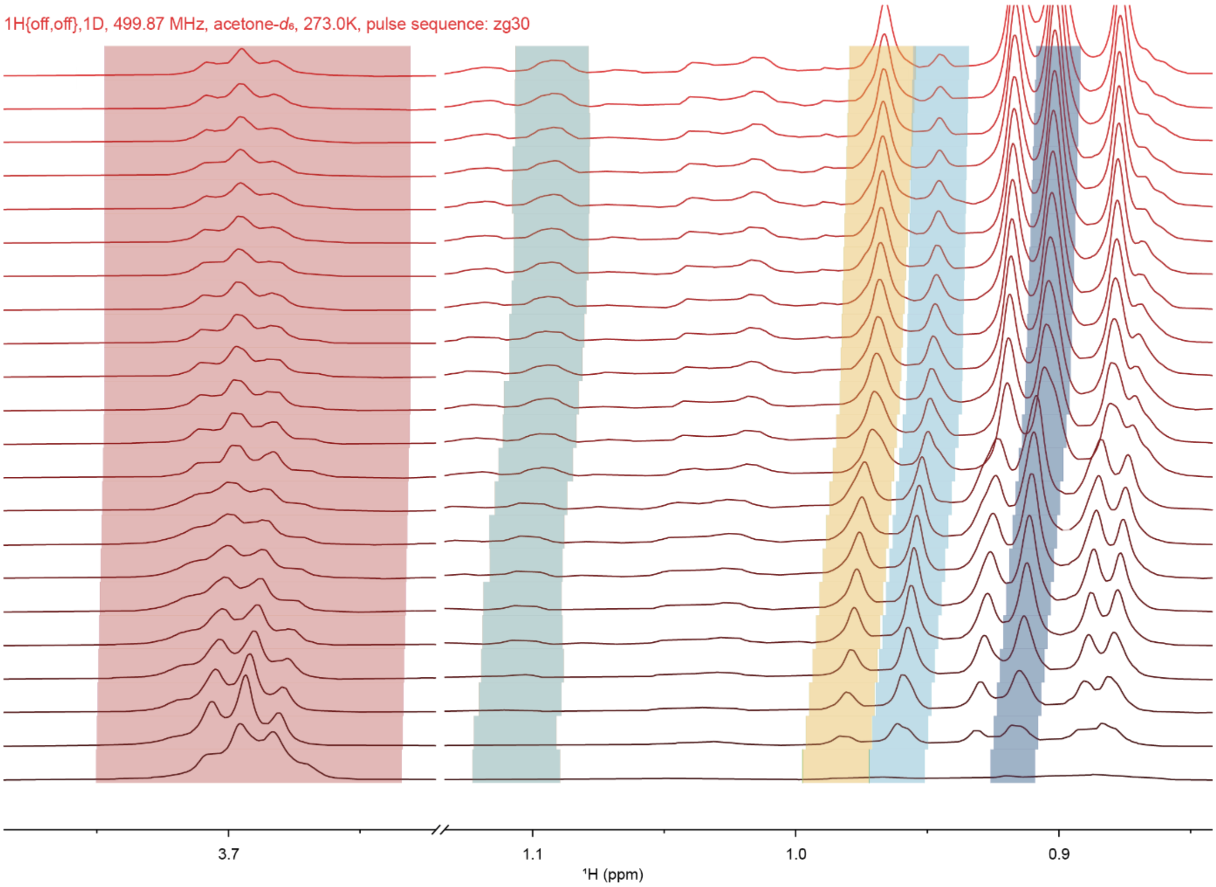

Fig. S58 | Excerpt of stacked ^{1}H NMR spectra acquired during the polyene cyclization of **1a** in HFIP/1*H*,1*H*-perfluorooctan-1-ol at 0 °C catalyzed by (*S*,*S*)-IDPi catalyst **8d**. Integration regions of interest used to estimate the concentrations of the respective components are highlighted (color coding see Fig. S56).

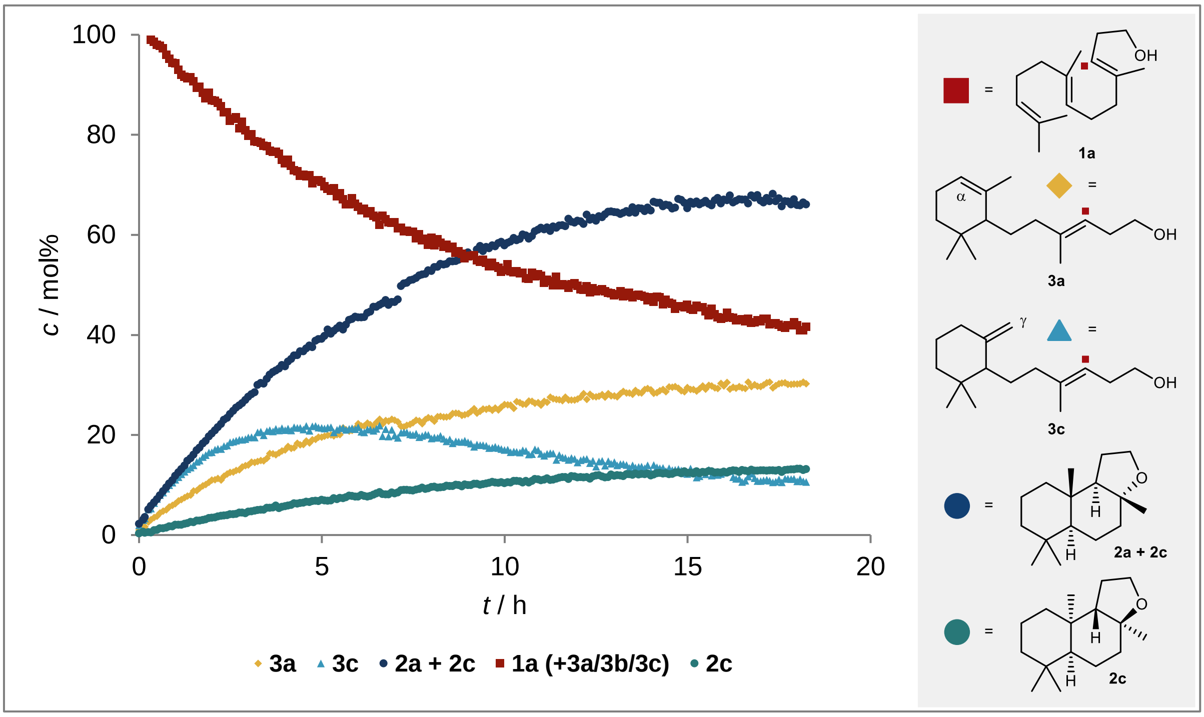

Fig. S59 | Concentration-time curve of the polyene cyclization of **1a** in HFIP/1*H*,1*H*-perfluorooctan-1-ol at 0 °C catalyzed by (*S*,*S*)-IDPi catalyst **8d**.

Due to the complex nature of the reaction resulting in multiple signal overlaps and the challenges associated with the data acquisition (protonated solvents; coaxial insert for locking), the provided data are intended to give a qualitative overview of the intermediates and components in the polyene cyclization of **1a**. Nevertheless, the NMR data clearly show the presence of monocyclic cyclohomofarnesol intermediates (**3**) and allow a statement about their reactivity. The exocyclic double bond isomer γ-cyclohomofarnesol (**3c**) for instance is initially formed in higher quantities compared to the thermodynamically more stable endocyclic double bond isomer α-cyclohomofarnesol (**3a**). Upon progression of the reaction, **3c** either converts to **3a** by protonation and subsequent deprotonation at the α-position or engages in an ensuing cyclization to provide ambrox (**2a**) and its diastereomer 5β,8α,9β-ambrox (**2c**). By contrast, **3a** accumulates in the reaction mixture and does not appear to participate to a significant extent in the polyene cyclization towards **2a** and **2c**.

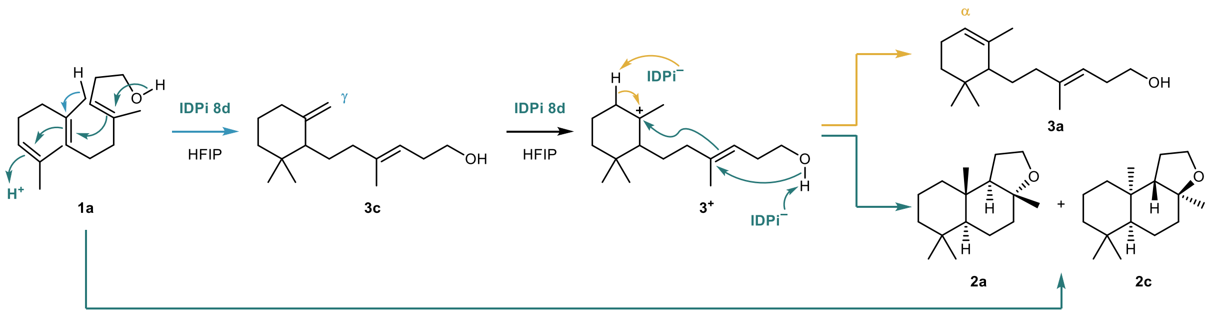

Scheme 5 | Putative mechanistic scenario for the IDPi-catalyzed polyene cyclization in HFIP based on NMR data.

The data suggest that γ-cyclohomofarnesol **3c** is a productive intermediate in the IDPi-catalyzed polyene cyclization of **1a** towards ambrox **2a** in HFIP/1*H*,1*H*-perfluorooctan-1-ol. The endocyclic double bond

isomer **3a** is significantly less prone to protonation by the confined IDPi catalyst, thus explaining its accumulation in the reaction mixture.

In addition to monitoring the reaction progress of the polyene cyclization, the stability of (*S*,*S*)-IDPi catalyst **8d** was evaluated by ^{31}P NMR spectroscopy. To this end, a ^{31}P NMR spectrum of the crude reaction mixture was acquired after completion of the polyene cyclization and compared with a ^{31}P NMR spectrum of pure IDPi catalyst **8d** (see Fig. S60). The ^{31}P NMR spectra clearly show no signs of decomposition or deactivation of the IDPi catalyst due to alkylation by interception of putative carbocationic intermediates.

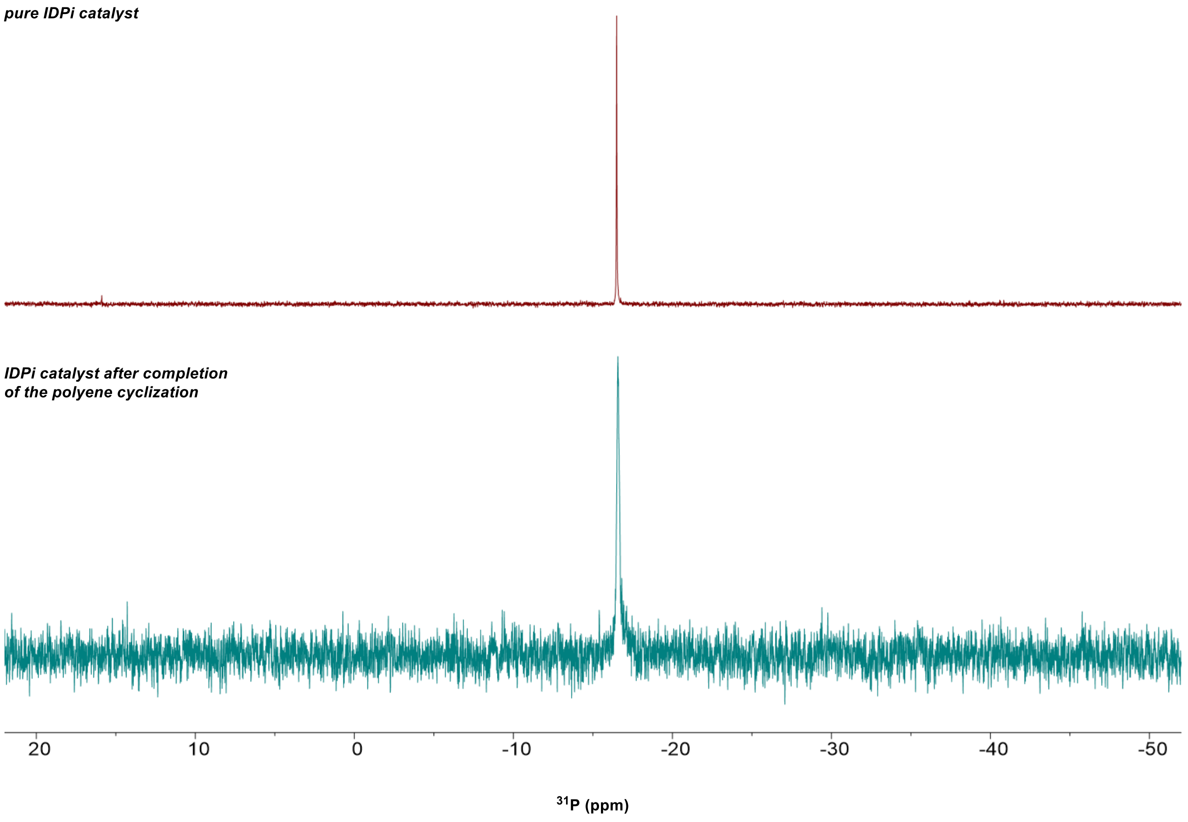

Fig. S60 | Stacked ^{31}P NMR (202 MHz, HFIP/1*H*,1*H*-perfluorooctan-1-ol, lock on acetone-d_6) spectra of (*S*,*S*)-IDPi catalyst **8d** before (top, maroon) and after (bottom, teal) the polyene cyclization of **1a** indicating no alkylated catalyst species or significant decomposition.

2.3.2 GC/HPLC Study: Time-Dependent Conversion in HFIP

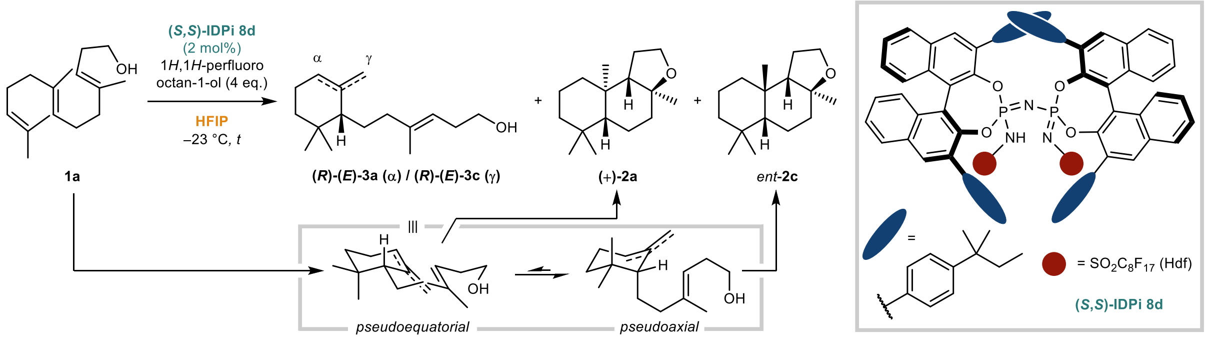

In order to gain additional information about the development of diastereomeric and enantiomeric ratio of products and intermediates over time, reactions in HFIP/1*H*,1*H*-perfluorooctan-1-ol at −23 °C were run in parallel and stopped at different points of time. The components isolated from the individual experiments were subsequently analyzed by GC and HPLC.

Table S22 | Yield, enantiomeric ratio (e.r.), enantiomeric excess (e.e.), and diastereomeric ratio (d.r.; **2a**:**2c**) over time in HFIP/1*H*,1*H*-perfluorooctanol at −23 °C according to GC and HPLC analysis.

t / h	**1a** / %	(+)-**2a**		*ent*-**2c**	**2a**:**2c**	(***R***)-**3a**		(***R***)-**3c**	
		yield / %	e.e. / %	e.r. / %	d.r.	yield / %	e.e. / %	yield / %	e.e. / %
2	72	2	82	97	>20:1	3	68	6	99
4	64	4	82	97	>20:1	5	68	10	99
10	41	9	82	97	97:3	11	68	21	99
24	21	18	84	97	94:6	17	70	26	99
48	10	24	86	97	90:10	25	73	24	99
72	6	40	90	97	89:11	23	75	13	99

Time-dependent conversion of homofarnesol **1a** (0.125 mmol, 1.0 equiv) in the presence of 1*H*,1*H*-perfluoro-octan-1-ol (4.0 equiv), and (*S*,*S*)-IDPi **8g** (2 mol %) in HFIP (250 µL, 0.5 M) at −23°C. Reactions were treated with triethylamine at the indicated time, the products were separated by flash column chromatography, and the diastereomeric and enantiomeric ratios of the individual components were subsequently analyzed by ^{1}H NMR spectroscopy, GC, and HPLC.

Monitoring yield, diastereo- and enantioselectivity over time revealed that (+)-ambrox is initially obtained with an e.r. of 91:1 when (*S*,*S*)-IDPi **8d** is used as catalyst. In line with the previously conducted NMR study, (+)-**2a** is formed alongside **3c** and **3a**. Upon progression of the reaction, the exocyclic double bond isomer **3c** converts to the more thermodynamically stable endocyclic double bond isomer **3a** and to (+)-**2a** and *ent*-**2c**. The different enantiomeric ratio of **3c** (99:1 e.r.) and **3a** (84:16 e.r.) suggest a kinetic resolution in the double bond isomerization step. The comparable high enantiomeric ratio (98.5:1.5) of the side product diastereomer *ent*-5β,8α,9β-ambrox (*ent*-**2c**) indicates its formation via cyclization of the pseudoaxial conformer of **3c**. Similarly, the increase in the enantiomeric ratio of (+)-**2a** (from 91:9 e.r. → 95:5 e.r.) can be rationalized by its formation in high stereoselectivity from the pseudoequatorial conformer of **3c**. The concomitant decrease in diastereoselectivity can be ascribed to the abovementioned formation of *ent*-**2c** via the axial conformer of (*R*)-(*E*)-**3c** or (*R*)-(*E*)-**3a**.

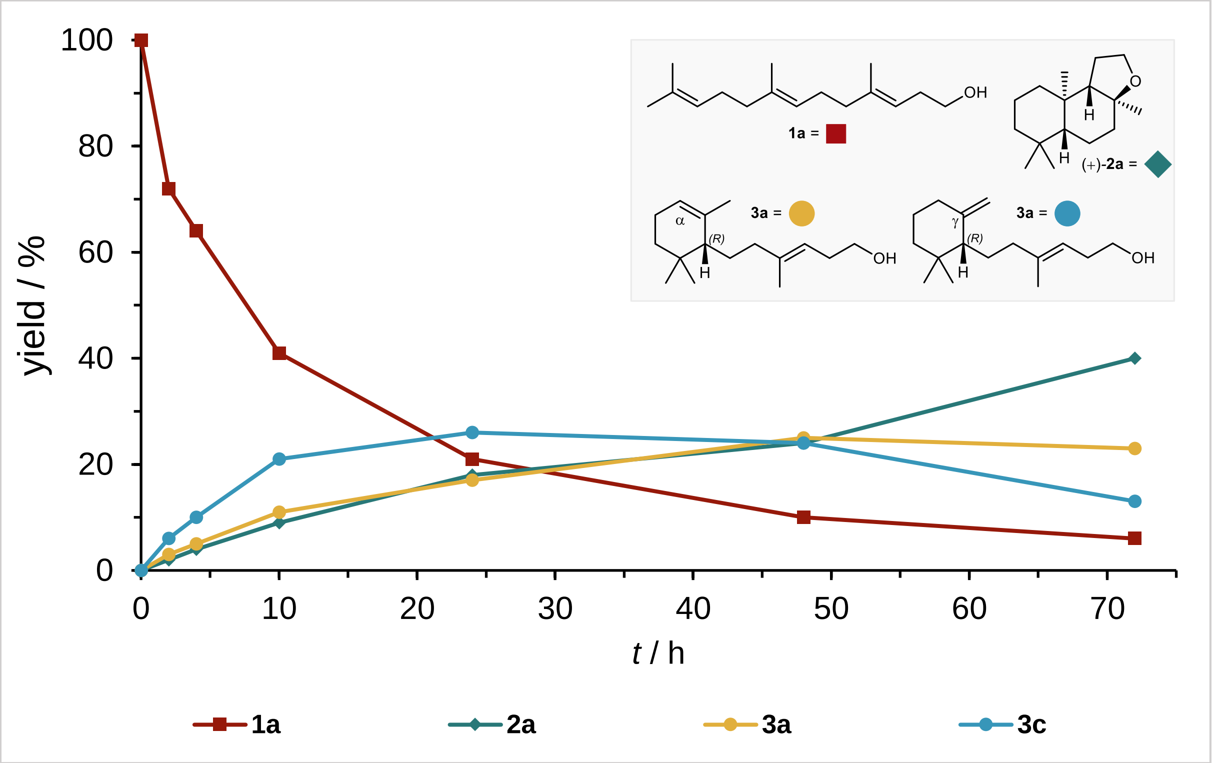

Fig. S61 | Time-dependent conversion of **1a** in the polyene cyclization catalyzed by (*S*,*S*)-IDPi **8d** in HFIP/1*H*,1*H*-perfluoro-octan-1-ol at −23 °C.

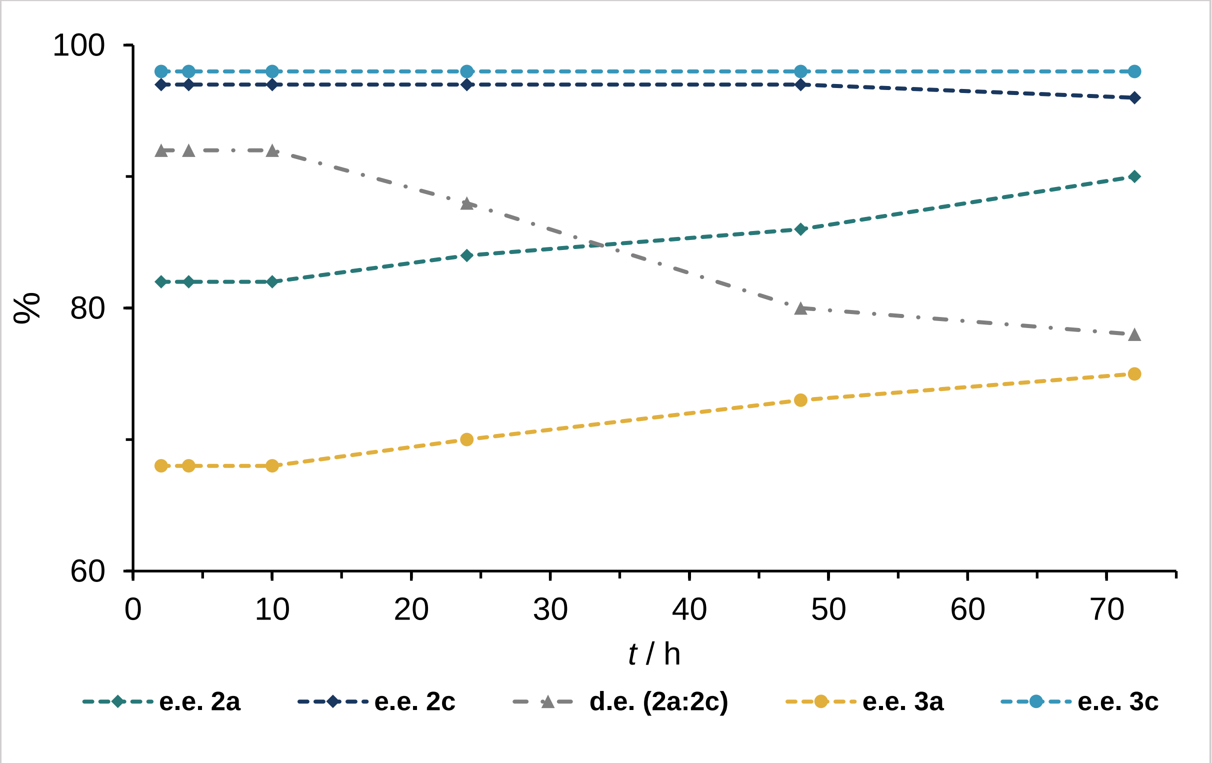

Fig. S62 | Development of the enantiomeric excess (e.e.) and diastereomeric excess (d.e.) of cyclohomofarnesols **3a** and **3c** as well as (+)-ambrox (**2a**) and *ent*-5β,8α,9β-ambrox (**2c**) over time in the polyene cyclization catalyzed by (*S*,*S*)-IDPi **8d** in HFIP/1*H*,1*H*-perfluoro-octan-1-ol at −23 °C.

2.4 Reactivity of Cyclohomofarnesols

To uncover the role and reactivity of cyclohomofarnesols (**3**) as intermediates in the IDPi-catalyzed asymmetric polyene cyclization, the respective cyclohomofarnesol double bond isomers (α = **3a**, β = **3b**, γ = **3c**) were subjected to the reaction conditions in HFIP and PFTB.

2.4.1 General Procedure

A 2 mL GC-vial equipped with a PTFE-coated magnetic stir bar was charged with cyclohomofarnesol **3** (25.0 µmol, 1.0 equiv.) and IDPi catalyst **8d** or **8g** (2 mol%). The mixture was cooled to desired temperature and stirred for 10 min. The respective solvent HFIP/1*H*,1*H*-perfluorooctan-1-ol (50 µL) or PFTB (20 µL, 1.25 M) was added and the reaction was stirred at the indicated temperature. The resulting mixture was neutralized with Et_3N, the solvent was evaporated under reduced pressure, and the residue was subjected to a short column to separate the catalyst. Deviations from the general procedure are specified in the individual experiments.

2.4.2 Reactivity of Cyclohomofarnesols in HFIP

Reactivity of (±)-(*E*)-α-cyclohomofarnesol (3a) in HFIP

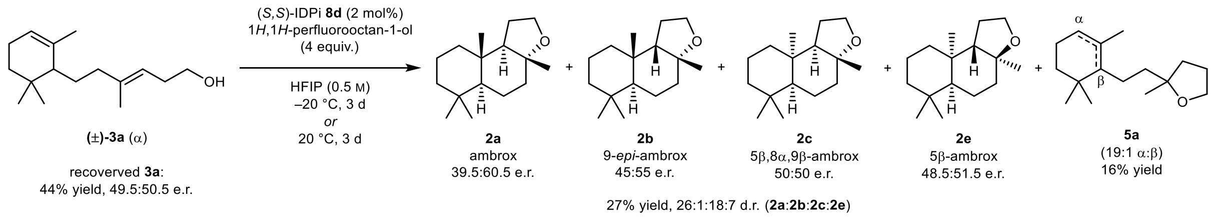

Two separate oven-dried 1.5 mL headspace screw-cap glass vials were charged with IDPi catalyst **8d** (2.42 mg, 1.00 µmol, 0.02 equiv., 2 mol%) and a PTFE-coated magnetic stir bar. The vials were evacuated, flushed with argon (3 ×) and cooled to −77 °C (propan-2-ol/dry-ice cooling bath). A solution of 1*H*,1*H*-perfluorooctan-1-ol (2 M, 80.0 mg, 0.20 mmol, 4.3 equiv.) and (±)-(*E*)-α-cyclohomofarnesol (**3a**, 11.1 mg, 47.0 µmol, 1.0 equiv.) in 1,1,1,3,3,3-hexafluoropropan-2-ol (HFIP, 100 µL, 160 mg, 0.95 mmol, 20 equiv.) was added dropwise to the catalyst, and the vials were placed inside a cryostat at −20 °C and 20 °C, respectively. After three days reaction time, the reactions were neutralized with triethylamine, 1,3,5-trimethylbenzene (mesitylene, 7.00 µL, 6.05 mg, 1.07 equiv.) was added as internal standard and the reactions were analyzed by ^{1}H NMR spectroscopy. Due to the low conversion of (±)-**3a** at −20 °C, only the reaction performed at 20 °C was analyzed. Purification by flash column chromatography on silica gel using hexanes/MTBE (gradient elution: 19:1 → 9:1 → 2:1 *v/v*) as eluent afforded partially cyclized side product **5a** (1.73 mg, 7.32 µmol, 16% yield), a tricyclic ether fraction mainly containing **2a, 2b**, **2c**, and **2e** (3.05 mg, 12.9 µmol, 27% yield, d.r. and e.r. shown in scheme above), alongside recovered starting material **3a** (4.92 mg, 20.8 µmol, 44% yield, 49.5:50.5 e.r.).

GC data for recovered **3a**:

GC (achiral) (DB-Waxetr 0.25/0.25df G/770, 30.0 m, temperature: 220/50 5/min 260 12/min 280, 3 min iso/ 350, 0.60 bar H_2, sample size: 1.0 µL, split ratio: 120:1, γ-isomer: t_R(**3c**) = 32.75 min (0.18%), α-isomer: t_R(**3a**) = 33.35 min (88.88%), β-isomer: t_R(**3b**) = 33.72 min (4.9%).

GC (chiral) (BGB-176/BGB-15 0.25/0.25df G/618, 30.0 m, temperature: 220/ 110 iso 350, 0.50 bar H_2, sample size: 1.0 µL, split ratio: 20:1): t_R(**3a**) = 396 min, t_R(*ent*-**3a**) = 419 min, 49.5:50.5 e.r. (1% e.e.).

GC data for the tricyclic ether fraction (**2**):

GC (achiral) (Optima-35 0.25/0.25df G/706, 29.0 m, temperature: 220/50 5/min 200 12/min 320, 5 min iso/ 350, 0.60 bar H_2, sample size: 1.0 µL): 9-*epi*-ambrox: t_R(**2b**) = 26.93 min (1.89%), 5β-ambrox: t_R(**2e**) = 27.19 min (48.94%), ambrox: t_R(**2a**) = 27.71 min (48.94%) 5β,8α,9β-ambrox: t_R(**2c**) = 28.20 min (34.11%).

GC (chiral) (BGB-176/BGB-15 0.25/0.25df G/618, 30.0 m, temperature: 220/ 140 iso/ 350, 0.60 bar H_2, sample size: 1.0 µL): (−)-9-*epi*-ambrox: t_R(**2b**) = 44.06 min (0.81%), (+)-9-*epi*-ambrox: t_R(*ent*-**2b**) = 45.16 min (1.00%), 45:55 e.r. (10% e.e.); 5β-ambrox: t_R(**2e**) = 44.57 min (6.57 %), *ent*-5β-ambrox: t_R(*ent*-**2e**) = 45.91 min (6.95%), 48.5:51.5 e.r. (3% e.e.); (−)-ambrox: t_R(**2a**) = 51.51 min (19.71%), (+)-ambrox: t_R(*ent*-**2a**) = 53.84 min (30.21%), 39.5:60.5 e.r. (21% e.e.); 5β,8α,9β-ambrox: t_R(**2c**) = 55.71 min (17.42%), *ent*-5β,8α,9β-ambrox: t_R(*ent*-**2c**) = 57.56 min (17.33%), 50:50 e.r. (0% e.e.).

Due to the presence of essentially racemic 5β-ambrox (**5e**) the sample was used as reference material for gas chromatographic analysis.

Reactivity of (*E*)-β-cyclohomofarnesol (3b) in HFIP

Due to the low conversion (<5%) of (*E*)-β-cyclohomofarnesol in HFIP in the presence of IDPi catalyst **8d**, the reaction temperature was increased to 20 °C.

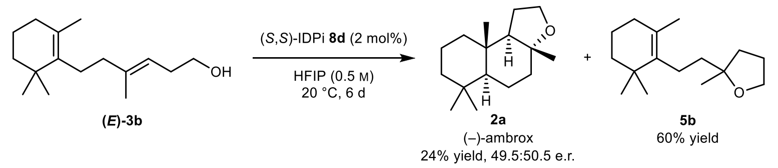

IDPi catalyst **8d** (18 mg, 8.50 µmol, 2 mol%) was transferred to a 4 mL headspace screw-cap vial followed by HFIP (0.85 mL). The mixture was stirred at 20 °C and (*E*)-β-cyclohomofarnesol (**3b**, 0.11 mL, 0.42 mmol, 1.0 equiv.) was added dropwise and the mixture was stirred for 6 d at 20 °C. The reaction was then quenched via addition of triethylamine (100 µL) and the solvent was removed in a stream of argon. The crude mixture was purified via flash column chromatography on silica gel (gradient elution with hexanes/MTBE 50:1 → 30:1 *v/v*) to yield **5b** (60.0 mg, 0.25 mmol, 60%) as colorless oil and **2a** (24.0 mg, 0.1 mmol, 24%, **2a**:**2c** ca. 82.5:17.5 d.r., e.r. **2a** = 49.5:50.5, e.r. **2c** = 48.5:51.5) as colorless oil.

^{1}H NMR (501 MHz, $CDCl_3$): δ (ppm) = 3.88–3.77 (m, 2H), 2.07 (dt, *J* = 12.7, 6.5 Hz, 1H), 2.01 (td, *J* = 12.6, 5.1 Hz, 1H), 1.95–1.87 (m, 4H), 1.77 (dt, *J* = 12.1, 7.5 Hz, 1H), 1.67–1.61 (m, 2H), 1.59 (s, 3H), 1.57–1.49 (m, 3H), 1.42–1.38 (m, 2H), 1.21 (s, 3H), 0.99 (s, 6H).

^{13}C NMR (126 MHz, $CDCl_3$): δ (ppm) = 137.1, 126.9, 82.9, 67.2, 41.1, 40.1, 36.8, 35.2, 32.9, 28.8, 26.3, 25.5, 23.6, 19.8, 19.7.

The spectroscopic data are in agreement with the reported literature.[15]

Reactivity of (±)-(*E*)-γ-cyclohomofarnesol (3c) in HFIP

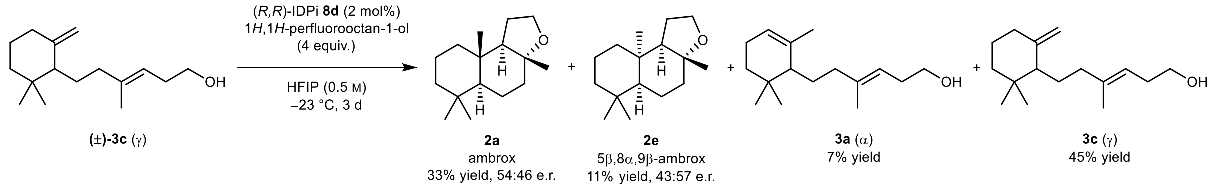

The reaction was performed according to the general procedure using (±)-(*E*)-γ-cyclohomofarnesol (**3c**) as starting material with (*R,R*)-IDPi **8d** as catalyst in HFIP/1*H*,1*H*-perfluorooctan-1-ol as solvent. The major compound in the reaction mixture was unreacted starting material **3c** (45% yield) alongside its thermodynamically more stable endocyclic double bond isomer **3a** (7% yield) generated upon isomerization of **3c** via deprotonation of the transient carbocation. The remaining material was a mixture of ambrox (**2a**, 33% yield, major diastereomer) and 5β,8α,9β-ambrox (**2c**, 11% yield, minor diastereomer) was obtained. A small preference for (−)-ambrox was observed (54:46 e.r.) whereas the opposite enantiomer of **2c** was slightly preferred, suggesting a possible kinetic resolution via isomerization between **3a** and **3c**, diastereoselective protonation of the enantiomers, or a diastereodivergent process wherein (*S*)-(*E*)-**3c** reacts to give (−)-**2a** and (*R*)-(*E*)-**3c** preferentially provides *ent*-**2c** (vide infra). To gain insights on the influence of the existing stereogenic center in **3c** on the outcome of the reaction, (±)-(*E*)-**3c** was separated into its enantiomers which were subsequently investigated in separate experiments.

Reactivity of enantiopure (*S*)- and (*R*)-(*E*)-γ-cyclohomofarnesol (3c) in HFIP

The reaction was performed according to the general procedure using (*S*)-(*E*)-γ-cyclohomofarnesol (**3c**) as starting material and (*R,R*)-IDPi **8d** as catalyst in HFIP/1*H*,1*H*-perfluorooctan-1-ol as solvent.

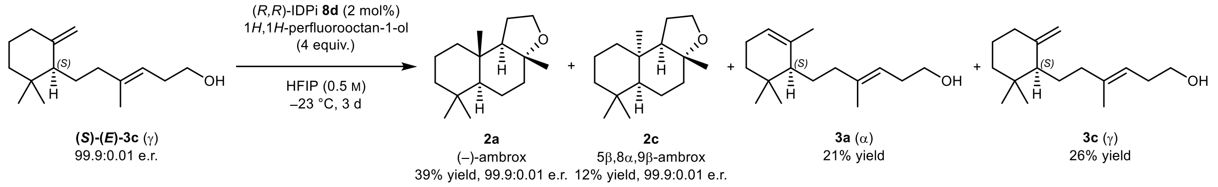

(−)-Ambrox (**2a**) was formed as major product in 39% yield and 99.9:0.01 e.r. alongside its diastereomer 5β,8α,9β-ambrox (**2c**, 12% yield 99.9:0.01 e.r.). The remaining components were residual starting material (*S*)-(*E*)-γ-cyclohomofarnesol (**3c**, 26% yield) and its thermodynamically more stable endocyclic double isomer (*S*)-(*E*)-α-cyclohomofarnesol (**3a**, 21% yield).

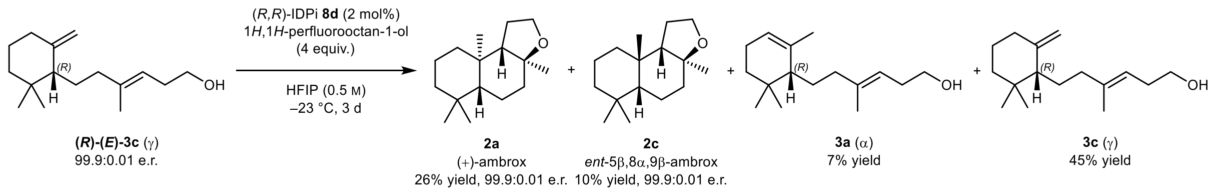

The reaction was performed according to the general procedure using (*R*)-(*E*)-γ-cyclohomofarnesol (**3c**) as starting material and (*R,R*)-IDPi **8d** as catalyst in HFIP/1*H*,1*H*-perfluorooctan-1-ol as solvent. In this

case, the respective antipodes of **2a** and **2c** were formed: (+)-ambrox was obtained in 26% yield and 99.9:0.01 e.r. alongside *ent*-5β,8α,9β-ambrox (10% yield, 99.9:0.01 e.r.). The majority of the remaining components was residual (*R*)-(*E*)-γ-cyclohomofarnesol (45% yield). In contrast to the reaction with (*S*)-(*E*)-γ-cyclohomofarnesol, only a minor amount of (*R*)-(*E*)-**3c** isomerized to the endocyclic double bond isomer (*R*)-(*E*)-**3a**. This suggests that the rate of isomerization of (*R*)-(*E*)-**3c** (mismatched pair) towards the thermodynamically more stable isomer (*R*)-(*E*)-**3a** is slower compared to (*S*)-(*E*)-**3c** (matched pair). In addition, lower yields of (+)-**2a** and *ent*-**2c** and diastereoselectivity towards **2a** (**2a**:**2c** = 72:28 d.r.; ca. 2.6:1 d.r.) was observed when (*R*)-(*E*)-**3c** was used as substrate with (*R*,*R*)-IDPi **8g** compared to the reaction with (*S*)-(*E*)-**3c** which provided (−)-**2a** and **2c** in a combined yield of 51% and a slightly increased diastereoselectivity towards (−)-**2a** (**2a**:**2c** = 76.5:23.5 d.r.; 3.3:1 d.r.). This observation is in line with a matched/mismatched mechanistic scenario common for substrates with an existing stereogenic center.[45]

2.4.3 Reactivity of Cyclohomofarnesols in PFTB

Reactivity of (±)-(*E*)-α-cyclohomofarnesol (3a) in PFTB

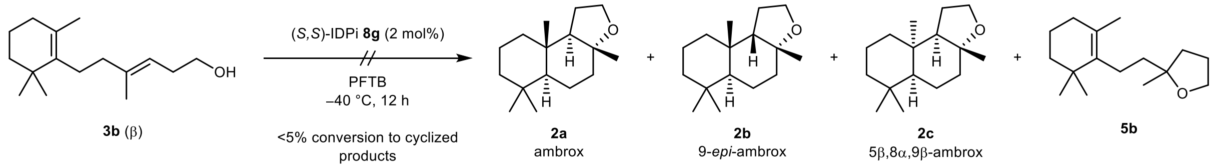

The reaction was performed according to the general procedure using (±)-(*E*)-α-cyclohomofarnesol (**3a**) as substrate. Less than 5% conversion towards tricyclic ethers (**2**) or partially cyclized products (**5**) was observed according to ^{1}H NMR analysis of the crude reaction mixture using CH_2Br_2 as internal standard.

Reactivity of (*E*)-β-cyclohomofarnesol (3b) in PFTB

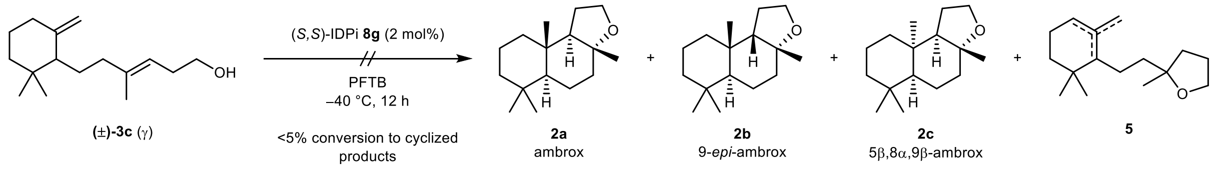

The reaction was performed according to the general procedure using (±)-(*E*)-β-cyclohomofarnesol (**3b**) as substrate. Less than 5% conversion towards tricyclic ethers (**2**) or partially cyclized products (**5**) was observed according to ^{1}H NMR analysis of the crude reaction mixture using CH_2Br_2 as internal standard.

Reactivity of (±)-(*E*)-γ-cyclohomofarnesol (3c) in PFTB

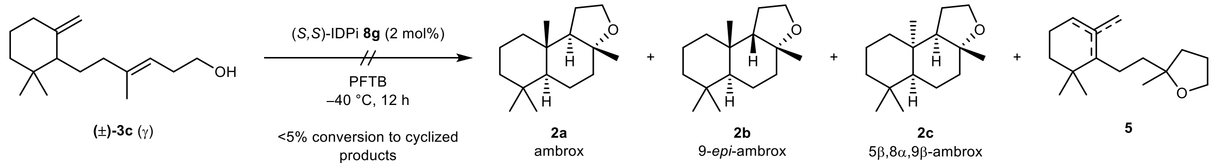

The reaction was performed according to the general procedure using (±)-(*E*)-γ-cyclohomofarnesol (**3c**) as substrate. Less than 5% conversion towards tricyclic ethers (**2**) or partially cyclized products (**5**) was

observed according to ^{1}H NMR analysis of the crude reaction mixture using CH_2Br_2 as internal standard. The reactivity was additionally evaluated at −30 °C to gather information on the diastereoselectivity and enantioselectivity of the reaction with **3c** as substrate.

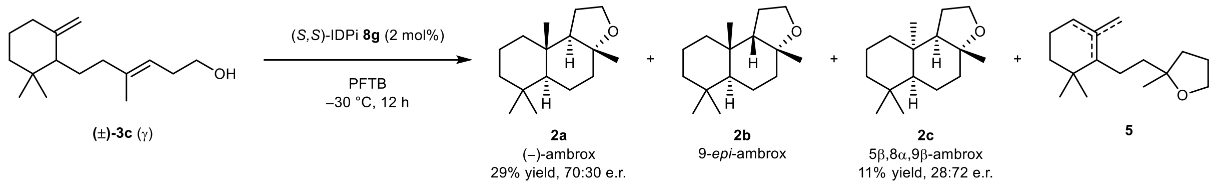

At −30 °C, conversion of (±)-(*E*)-**3c** to (−)-ambrox (**2a**, 29% yield, 70:30 e.r.) and its diastereomer 5β,8α,9β-ambrox **2c** (11% yield, 28:72 e.r.) was observed. Remarkably, whereas (−)-ambrox (**2a**) was obtained as major isomer, the opposite enantiomer of **2c** was predominantly formed. This suggests that a diastereodivergent resolution is occurring wherein (*S*)-(*E*)-**3c** preferably affords (−)-ambrox (**2a**) whereas (*R*)-(*E*)-**3c** predominantly provides *ent*-**2c**.

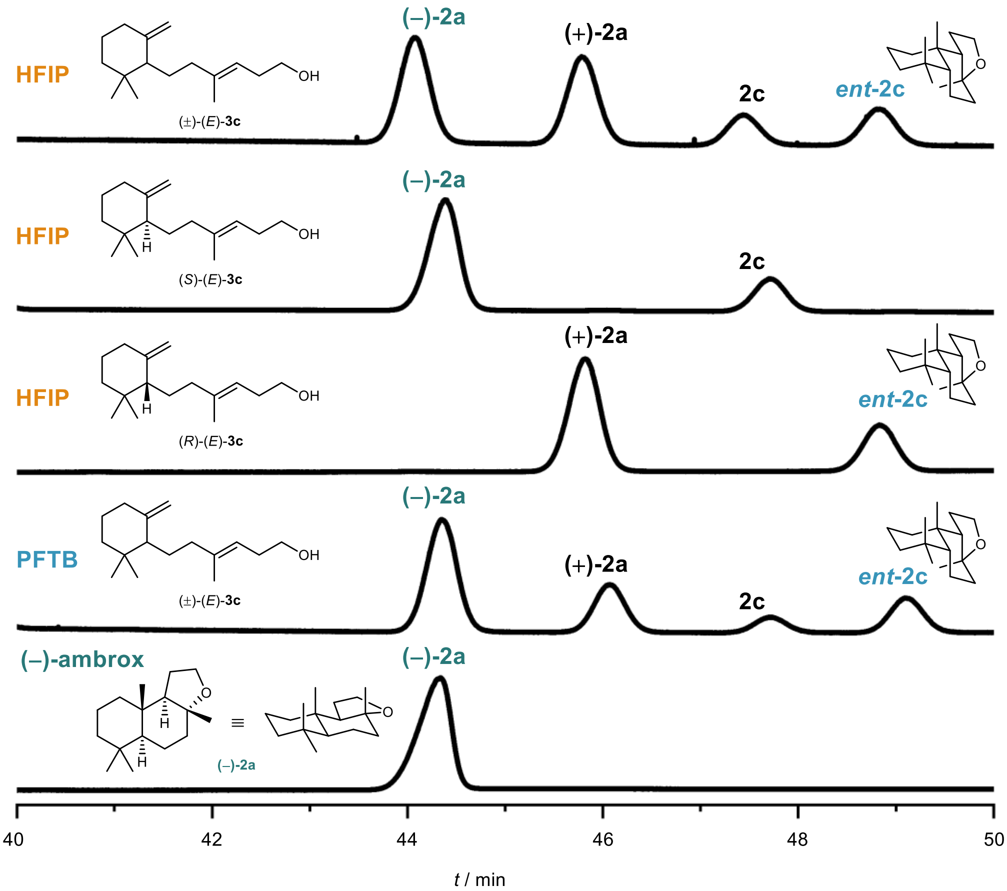

Fig. S63 | Excerpt of stacked GC traces of the polyene cyclizations of γ-cyclohomofarnesol (**3c**) in HFIP and PFTB showing the retention time region of tricyclic ethers.

2.4.4 Summary and Supplementary Mechanistic Discussion

Summary of the reactivity of cyclohomofarnesols (3) in HFIP

When HFIP is used as solvent in the polyene cyclization of **1a** to **2a**, (*E*)-γ-cyclohomofarnesol (**3c**) was identified as the most reactive double bond isomer towards ambrox (**2a**) as neither the α- (**3a**) nor the β-isomer (**3b**) showed appreciable conversions towards tricyclic ethers.

Table S23 | Summary of the reactivity of cyclohomofarnesols **3** in the catalytic asymmetric polyene cyclization using (*R*,*R*)-IDPi **8d** in HFIP.

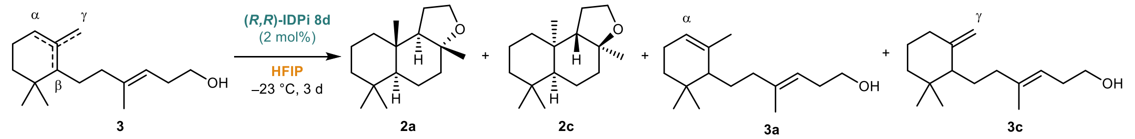

isomer	2a yield	2a e.r.	2c yield	2c e.r.	3a yield	3c yield
**3a** (α)	–	–	–	–	–	–
**3b** (β)	–	–	–	–	–	–
**3c** (γ)	33%	54:46	11%	43:57	16%	39%
(*S*)-**3c** (γ)	39%	99.9:0.1	12%	99.9:0.1	21%	26%
(*R*)-**3c** (γ)	26%	0.1:99.9	10%	0.1:99.9	7%	45%

When enantiopure isomers of **3c** were used as substrate, it was found that the existing stereogenic center at the cyclohexene ring defines the stereochemistry of the three stereogenic centers generated upon conversion to **2a**. Thus, (*S*)-(*E*)-**3c** provided (−)-ambrox (**2a**), whereas (*R*)-(*E*)-**3c** provided the respective antipode (+)-ambrox (*ent*-**2a**).

Summary of the reactivity of cyclohomofarnesols (3) in HFIP

In PFTB, neither cyclohomofarnesol isomer showed appreciable conversions towards tricyclic ethers at the reaction temperature (−40 °C). Upon increasing the reaction temperature to −30 °C (±)-(*E*)-γ-cyclohomofarnesol (**3c**) was found to be reactive, providing (−)-ambrox (**2a**, 70:30 e.r.) and notably the antipode of 5β,8α,9β-ambrox (*ent*-**2c**, 28:72 e.r.), indicating a kinetic resolution wherein the (*S*)-enantiomer reacts towards **2a** whereas the (*R*)-enantiomer preferentially provides *ent*-**2c**.

Table S24 | Summary of the reactivity of cyclohomofarnesols **3** in the catalytic asymmetric polyene cyclization using (*S*,*S*)-IDPi **8g** in PFTB.

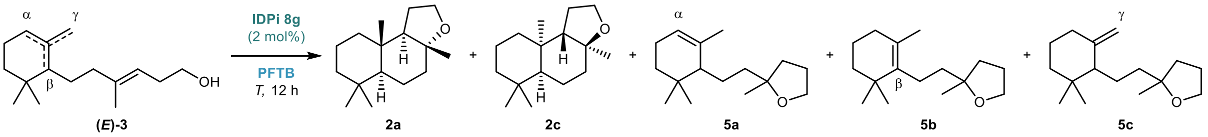

	isomer	2a yield	2a e.r.	2c yield	2c e.r.	5a yield	5a e.r.	5b yield	5b e.r.	5c yield	5c e.r.
–40 °C	(±)-**3a** (α)	–	–	–	–	–	–	–	–	–	–
	**3b** (β)	–	–	–	–	–	–	–	–	–	–
	(±)-**3c** (γ)	<5%	n.d.	–	–	–	–	–	–	–	–
–30 °C	(±)-**3c** (γ)	29%	70:30	11%	28:72	48%<br>d.r. 46:54	2:98 (major)<br>99:1 (minor)	trace	93:7	10%<br>d.r. 19:81	10:90 (major)<br>1:99 (minor)

Supplementary mechanistic discussion

Based on the results obtained with the individual enantiomers of **3c**, a detailed mechanistic picture for the IDPi-catalyzed polyene cyclization can be developed.

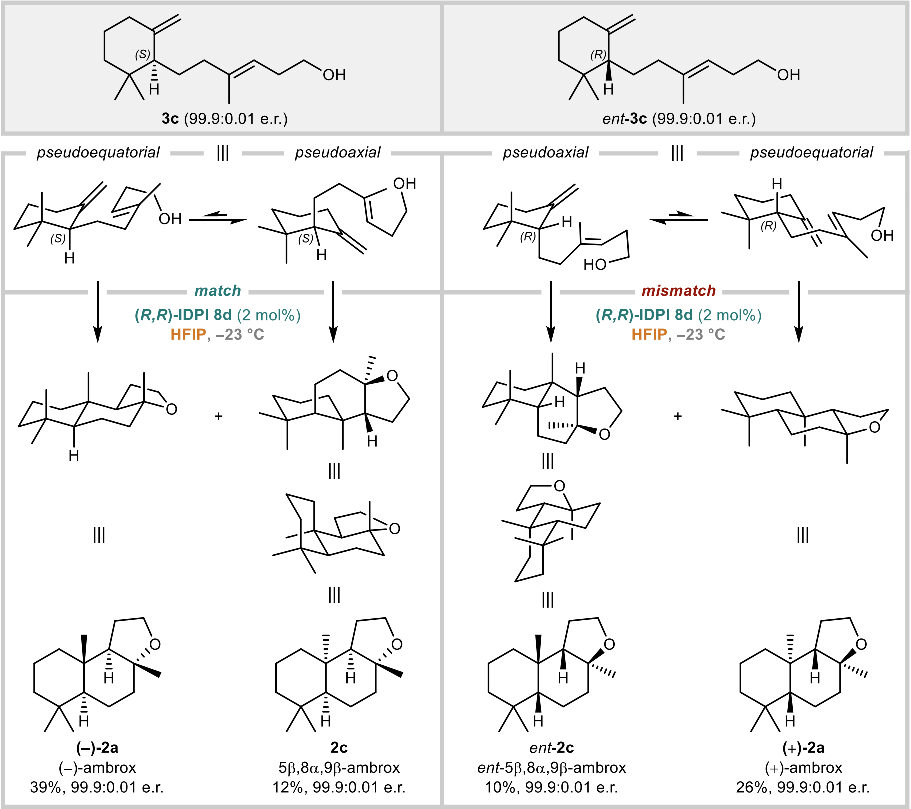

Fig. S64 | Mechanistic rationale for the reactivity of the individual enantiomers of **3c** and possible match/mismatch scenario with (*R*,*R*)-IDPi catalyst **8d** in HFIP.

As discussed above in the experiment with the respective enantiomers of **3c**, a match/mismatch scenario is conceivable, wherein the (*S*)-enantiomer provides a slightly improved diastereoselectivity and yield towards (−)-**2a** (match) compared to the opposite enantiomer (mismatch). It is worth mentioning that the diastereoselectivity towards **2a** is relatively high (**2a**:**2c** >3:1) considering that (*E*)-γ-cyclohomofarnesol (**3c**) predominantly adopts a thermodynamically preferred pseudoaxial conformation (due to the allylic strain in the pseudoequatorial conformer). The observed preference towards **2a** stands in contrast to results obtained by Snowden et. al. in polyene cyclizations of **3c** mediated by fluorosulfonic acid (FSO_3H) in 2-nitropropane at −90 °C. When **3c** was subjected to the aforementioned reaction conditions, a racemic mixture of **2a** (ambrox, 14%), **2b** (9-*epi*-ambrox, 11%), **2c** (5β,8α,9β-ambrox, 34%), and **2e** (5β-ambrox, 16%) was obtained.[15] In this case, **2c** and **2e** resulting from protonation of the major pseudoaxial conformer of **3c** are formed as the major product, resulting in a product distribution between tricyclic ethers with a *cis*-fused A/B ring and a *trans*-A/B ring junction of 2:1 (**2c**+**2e** = 50% *cis*-ring, **2a**+**2b** = 25% *trans*-ring). This suggests that the product distribution reflects a conformer equilibrium of approximately 2:1 (pseudoaxial/pseudoequatorial) prior to cyclization under the reaction conditions. The result obtained with the IDPi catalyst clearly points towards a different mechanistic scenario in which conformational inversion of the cyclohomofarnesol

substrate (or a putative cyclohexyl carbocation intermediate) occurs more rapidly than closure of the second (B) ring. This is in line with observations from Yamamoto et. al. in diastereoselective stepwise cyclizations of triethyl silyl-protected monocyclic intermediates mediated by Lewis acid-assisted chiral Brønsted acids.[14]

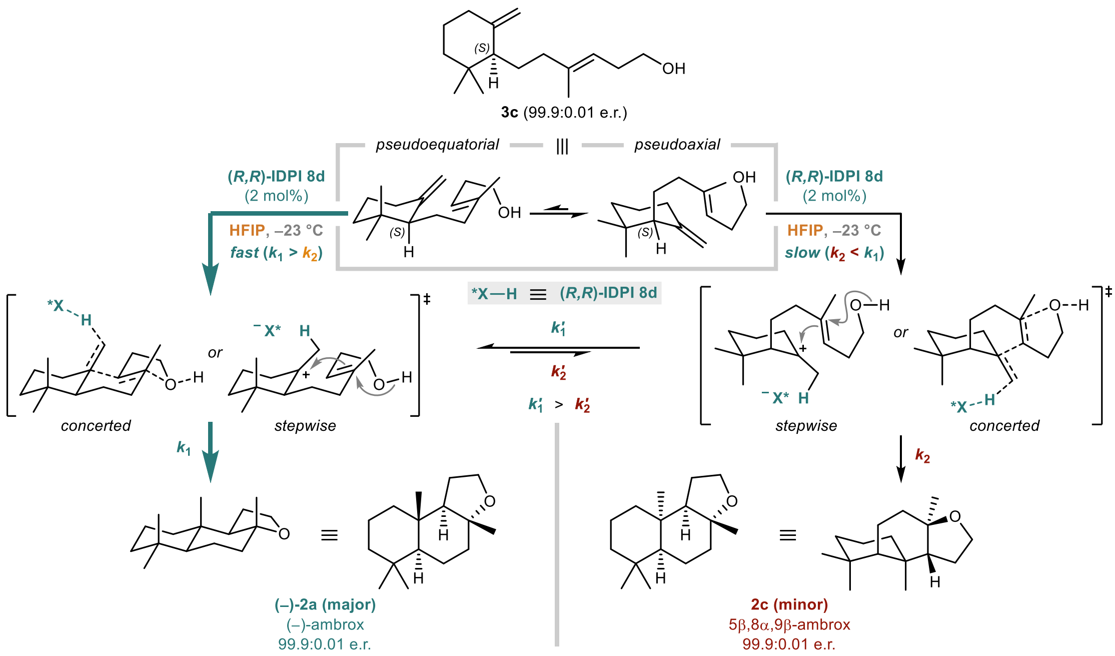

Fig. S65 | Proposed Curtin–Hammett scenario for the IDPi-catalyzed polyene cyclization of **3c** in HFIP.

Thus, a Curtin–Hammett scenario is conceivable wherein the product ratio is dependent on the energy difference between the respective rate-limiting transition states (TS). Based on the observed product distribution, it can be assumed that the TS towards **2a** via protonation of the pseudoequatorial conformer of **3c** is lower in energy compared to the TS towards **2c** via protonation of the pseudoaxial conformer of **3c**. For the sake of completeness, it should be mentioned at this point that the formation of a *cis*-fused A/B ring can also occur via protonation of the pseudoequatorial conformer of **3c** with a pseudoaxial orientation of the side chain.

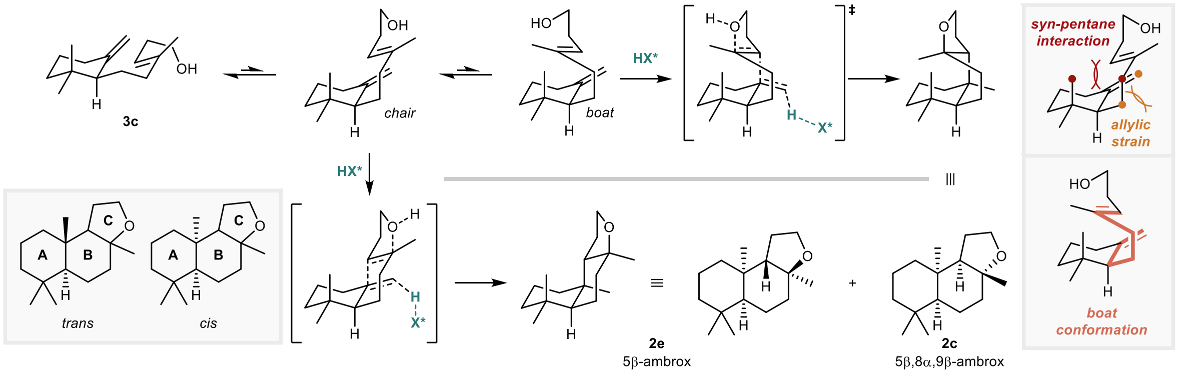

Fig. S66 | Mechanistic proposal for the formation of the *cis*-fused diastereomers 5β,8α,9β-ambrox (**2c**) and 5β-ambrox (**5e**) via protonation of the pseudoaxial conformer of **3c**.

In light of considerable enthalpic penalties associated with the adoption of such conformations (allylic strain and *syn*-pentane interactions) and the formation of **2c** via a highly congested boat transition state, these pathways seem less likely to occur under the reaction conditions.

2.5 Investigation of the Solvent Effect on Substrate Conformation

To evaluate the influence of solvents on the conformation of (3*E*,7*E*)-homofarnesol, 1D-^{1}H-NOESY experiments were performed. To this end, (3*E*,7*E*)-homofarnesol (16.4 µL, 14.1 mg, 0.06 mmol) was dissolved in the respective solvent (0.6 mL) and the resulting solutions (0.1 M) were analyzed by NMR spectroscopy, in particular by performing a 1D-^{1}H-NOESY experiment. The CH_2-group adjacent to the hydroxy group of **1a** (position 1) was integrated and the selected region was selectively excited using the tool **[Setup Selective 1D Experiment]** implemented in Bruker TopSpin 4.0.6 and the standard pulse sequence *selnogpzs.2* (mixing time d8 = 0.3 s and number of scans = 32).

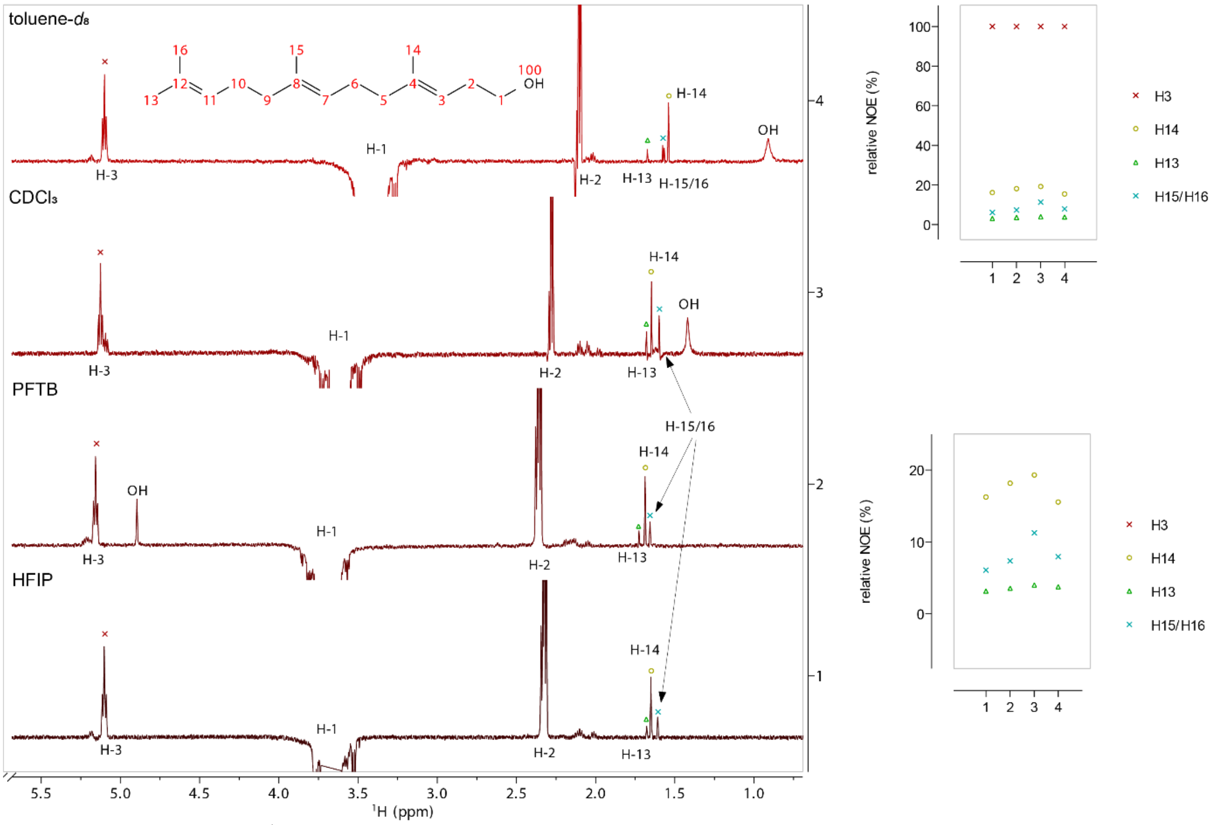

Fig. S67 | Stacked 1D-^{1}H-NOESY of (3*E*,7*E*)-homofarnesol **1a** in different solvents: 1,1,1,3,3,3-hexafluoro-propan-2-ol, HFIP (1), perfluoro-*tert*-butanol, PFTB (2), chloroform-*d*, $CDCl_3$ (3), toluene-d_8 (4). Positive signals are characteristic for interactions of the protons with the excited CH_2-group (position 1) through space. The observed NOEs of the diagnostic methyl groups of **1a** relative to position 3 (H-3) in the respective solvents are depicted on the right.

The 1D-^{1}H-NOESY experiments reveal through-space interactions between the irradiated CH_2-group adjacent to the hydroxyl group (position 1, H-1) and the diagnostic methyl groups (proximal: H14, internal: H15, terminal: H13/H16) in all investigated solvents. This connotes that the starting material **1a** can adopt a coiled conformation in each solvent. This observation stands in contrast to the study performed by the Gulder group which revealed that solely perfluorinated solvents such as PFTB trigger a coiled conformation of homogeranyl benzene.[44] The hypothesis is corroborated by the lack of significant interactions between the internal and distal methyl groups and the irradiated arene in homogeranyl benzene dissolved in solvents such as chloroform-*d*, cyclohexane-d_{12} or methanol-d_4. It is likely that the different choice of substrate accounts for the contrasting observations between the two

studies. Homogeranyl benzene, a hydrocarbon substrate, is significantly less polar compared to the homoallylic alcohol (3*E*,7*E*)-homofarnesol **1a**. The structure of **1a** is reminiscent of typical surfactants with a characteristic hydrophobic “tail” and a hydrophilic “head”. In less polar solvents such as $CDCl_3$ and toluene-d_8, micellar arrangements and coiled conformations that minimize interactions between the polar hydroxyl group and the hydrophobic solvent are likely favored over linear structures that expose the polar region to the solvent. The observed NOE interactions, indicating a coiled conformation in both nonpolar solvents, can thus be interpreted as a consequence of the abovementioned surfactant-like behavior of **1a**.

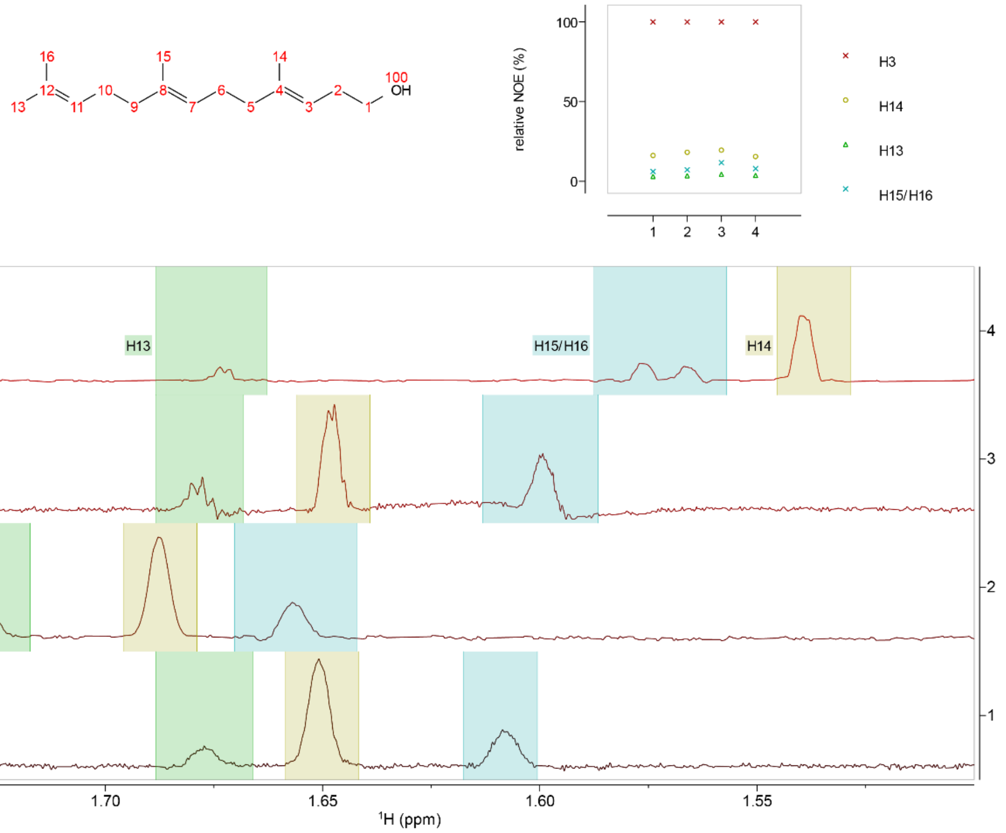

Fig. S68 | Enlarged excerpt (spectral region of the methyl groups from 1.76–1.50 ppm) of the stacked 1D ^{1}H-NOESY spectra of (3*E*,7*E*)-homofarnesol **1a** in different solvents: 1,1,1,3,3,3-hexafluoro-propan-2-ol, HFIP (1), perfluoro-tert-butanol, PFTB (2), chloroform-d_1, $CDCl_3$ (3), toluene-d_8 (4).

In context of the investigated IDPi-catalyzed polyene cyclization of **1a** to **2a** in HFIP or PFTB, the high diastereoselectivity cannot be exclusively attributed to the property of fluorinated alcohols to enforce a folded substrate conformation. The 1D-^{1}H-NOESY experiments clearly show no significant differences between fluorinated alcohols and more common solvents such as toluene and chloroform. Other effects such as the stabilization of transient cationic charges and the ability to rapidly shuttle protons via a Grotthuß mechanism are thus more likely contributing to the observed enhancement in reactivity in comparison with other solvents.

2.6 Assignment of the Absolute Configuration

Determination of the absolute configuration of ambrox (2a)

The absolute configuration of ambrox (**2a**) was unequivocally determined by comparison of the GC retention times with an authentic reference standard of (−)-ambrox, comparison of the optical rotation with available literature data, and by single-crystal X-Ray diffraction.

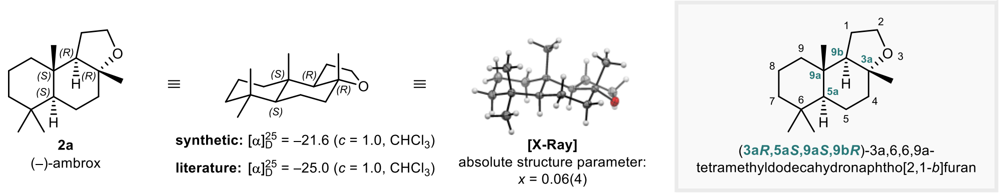

Fig. S69 | Assignment of the absolute configuration of synthetic ambrox (**2a**).

The absolute configuration of synthetic ambrox (**2a**) was thus assigned as 3a*R*,5a*S*,9a*S*,9b*R* (IUPAC numbering).

Determination of the absolute configuration of 9-*epi*-ambrox (2b)

The absolute configuration of 9-*epi*-ambrox (**2b**) was unequivocally determined by comparison of the GC retention times with a previously synthesized reference standard of enantiopure (−)-9-*epi*-ambrox (the absolute configuration of which was additionally confirmed by single-crystal X-Ray diffraction), and comparison of the optical rotation with available literature data.

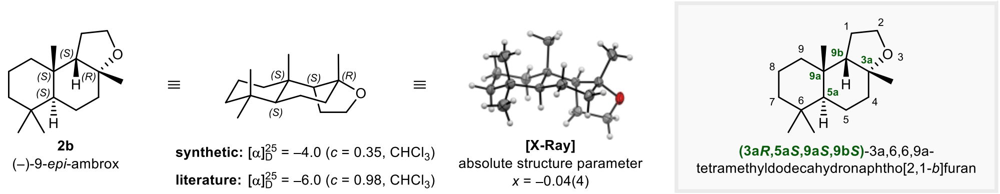

Fig. S70 | Assignment of the absolute configuration of synthetic 9-*epi*-ambrox (**2b**).

The absolute configuration of synthetic 9-*epi*-ambrox (**2b**) was thus assigned as 3a*R*,5a*S*,9a*S*,9b*S* (IUPAC numbering).

Determination of the absolute configuration of 5β,8α,9β-ambrox (2c)

The absolute configuration of 5β,8α,9β-ambrox (**2c**) was indirectly assigned by tracing back its formation via enantiopure intermediates with known absolute configuration. The formation of 5β,8α,9β-ambrox (**2c**) can be rationalized by diastereoselective cyclization of the pseudoaxial conformer of the monocyclic intermediate (*E*)-γ-cyclohomofarnesol (**3c**). When enantiopure (*S*)-(*E*)-**3c** (99.9:0.01 e.r.) was used as substrate in the presence of (*R*,*R*)-IDPi catalyst **8d**, the formation of (−)-ambrox (**2a**, 99.9:0.01 e.r.) was observed alongside 5β,8α,9β-ambrox (**2c**, 99.9:0.01 e.r.). The stereochemistry of the enantiopure starting material (*S*)-(*E*)-**3c** can thus be unequivocally confirmed by comparison with the major product of the reaction, (−)-ambrox (**2a**), whose absolute configuration was confirmed by comparison with authentic enantiopure reference material. The absolute configuration of **2c** can thus be

deduced from the absolute configuration of **3c** since the existing stereogenic center in **3c** defines the stereochemistry of the newly generated three stereogenic centers.

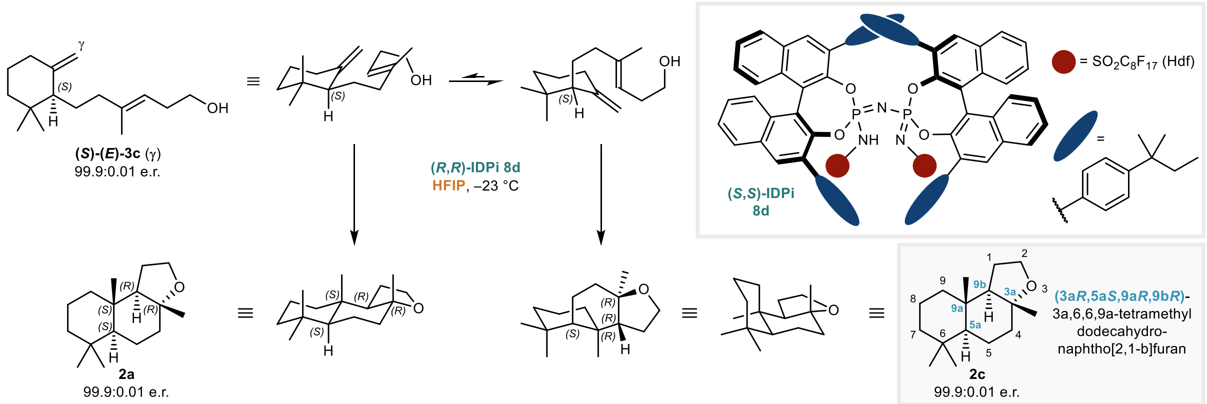

Fig. S71 | Assignment of the absolute configuration of 5β,8α,9β-ambrox (**2c**) by tracing back its origin from diastereoselective protonation of enantiopure γ-cyclohomofarnesol (*S*)-(*E*)-**3c** with known absolute configuration.

Determination of the absolute configuration of (*E*)-α- (3a) and (*E*)-γ-cyclohomofarnesol (3c)

The absolute configurations of (*E*)-α-cyclohomofarnesol (**3a**) and (*E*)-γ-cyclohomofarnesol (**3c**) were indirectly assigned by comparison with the known absolute configuration of the major products (−)-ambrox (**2a**) from (*S*)-(*E*)-**3a/3c** and (+)-ambrox (*ent*-**2a**) from (*R*)-(*E*)-**3a/3c**, respectively.

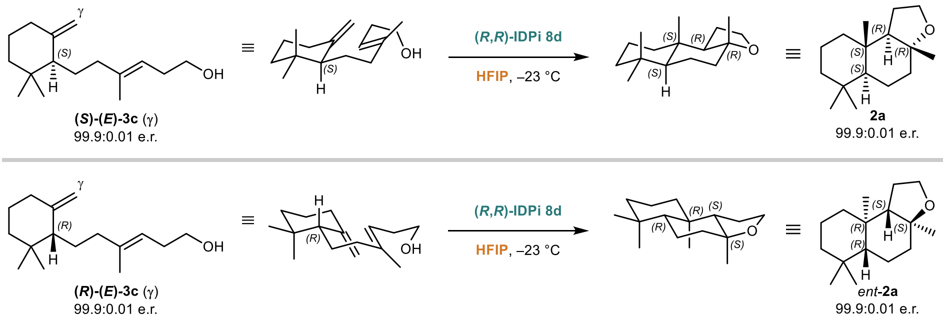

Fig. S72 | Assignment of the absolute configuration of cyclohomofarnesol **3c** based on its diastereoselective conversion into **2a** with known absolute configuration.

Subjecting enantiopure isomers of **3c** to the asymmetric polyene cyclization with (*R*,*R*)-IDPi catalyst provided (−)-ambrox (**2a**) and (+)-ambrox (*ent*-**2a**), respectively. Based on these results, the stereochemistry at the cyclohexene ring of the starting material was assigned as (*S*) in the first case and (*R*) in the second case. The absolute configuration of **3a** was assigned by analogy.

Partial assignment of the stereochemistry of side products 5 and 10

Unfortunately, it was not possible to determine the relative and absolute configurations of partially cyclized product **5** and isomerized product **10**. Based on the known absolute configuration of the tricyclic ether products (most importantly ambrox **2a** and 9-*epi*-ambrox **2b**), the absolute configuration of the respective cyclohomofarnesols **3** can be deduced (as described above). Assuming that the e.r. observed for the cyclohomofarnesol isomers **3a** (α-isomer) and **3c** (γ-isomer) also translates into the partially cyclized products **5**, the absolute configuration at the cyclohexene ring can be tentatively assigned accordingly.

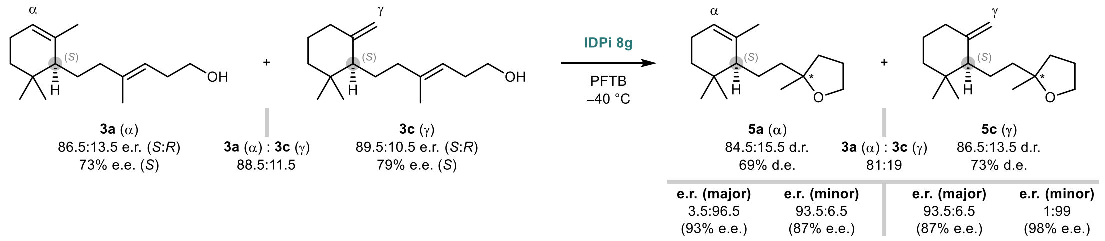

Fig. S73 | Partial assignment of the absolute configuration of partially cyclized products **5a** and **5c** based on their formation from cyclohomofarnesols **3a** and **3c** with known absolute configurations.

Indeed, the d.r. (84.5:15.5 d.r. for **5a** and 86.5:13.5 for **5c**) of the partially cyclized products **5a** and **5c** is in good agreement with the e.r. observed for the cyclohomofarnesol intermediates **3a** and **3c** at −40 °C (86.5:13.5 d.r. for **3a** and 89.5:10.5 for **3c**). For the cyclohomofarnesols **3a** and **3c** the major enantiomer was previously assigned as (*S*) and the minor enantiomer as (*R*). The absolute configuration at the cyclohexene ring of **5a** and **5c** was thus assigned as (*S*) for the major diastereomer and (*R*) for the minor diastereomer. The stereochemistry at the cyclohexene ring of isomerized side product **10** can possibly be deduced in a similar fashion assuming that the formation of **10** proceeds via two consecutive Wagner–Meerwein rearrangements, namely a [1,2]-hydride shift followed by a [1,2]-methyl shift.

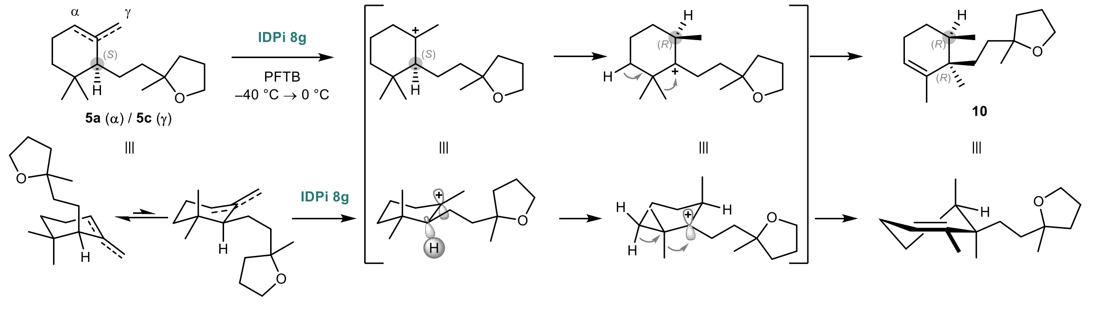

Fig. S74 | Tentative assignment of the stereochemistry at the cyclohexene ring of isomerized side product **10**.

The Wagner–Meerwein rearrangement is a thermally allowed [$_{\omega}0_s+_{\sigma}2_s$] pericyclic process that proceeds suprafacially with stereochemical retention according to the Woodward–Hoffmann rules. Therefore, in order for the hydride shift to occur, the rearrangement from **5** to **10** must take place from the pseudo-equatorial isomer of **5a** or **5c**. Based on the mechanistic proposal outlined in Fig. S74 the stereochemistry at the cyclohexene ring was thus tentatively assigned as (1*R*,6*R*).

Determination of the absolute configuration of sclareolide (12a)

The absolute configuration of sclareolide (**12a**) was unequivocally confirmed by comparison of the GC retention time with authentic (+)-sclareolide and the measured optical rotation with reported literature data.[16] The absolute configuration of synthetic sclareolide (**12a**) was thus assigned as 3a*R*,5a*S*,9a*S*,9b*R* (IUPAC numbering).

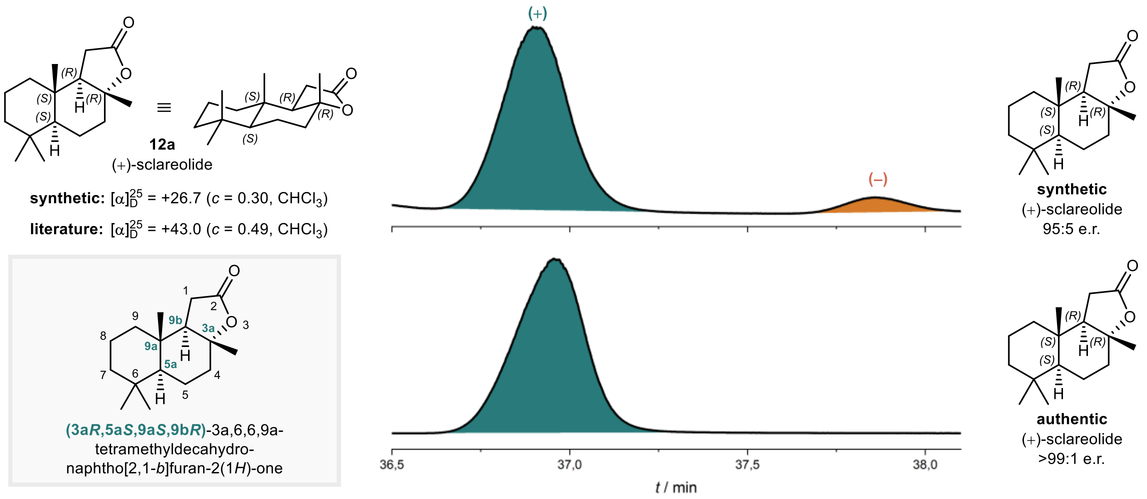

Fig. S75 | Determination of the absolute configuration of sclareolide **12a**.

3 Computational Methods

3.1.1 Computational Details

Preliminary structures were generated at the GFN2-xTB[46] level using the xtb program version 6.6.0, followed by exploration of the conformational landscape at the same level of theory using Grimme's Conformer–Rotamer Ensemble Sampling Tool (CREST)[47], version 2.12. The lowest resulting conformer was subjected to a constrained optimization at the GFN2-xTB[46] level of theory, fixing the distance between all bond-forming atoms to 2.1 Å.

All DFT calculations were conducted using ORCA version 5.0.3.[48] The GFN2-xTB conformer was subjected to a DFT-level geometry optimization with the same constraints using the r^2SCAN-3c composite method.[49] The Resolution of Identity (RI) approximation[50] in the Split-RI-J variant[51] using a corresponding auxiliary basis set[52] was used.

Structures were rendered using UCSF ChimeraX version 1.5.[53,54]

3.1.2 XYZ Coordinates

Plausible transition state model

```
305

  C   8.71790898148335    -1.11450571261089    -1.89795545466876
  C   8.32780818335706    -1.41532364901009    -0.56615096548395
  C   7.01839061701012    -1.24395521566395    -0.17044791206423
  C   7.75900303834851    -0.73191830802107    -2.81643380380095
  C   6.40338386630326    -0.56885519404938    -2.43174976037928
  C   6.02894227536052    -0.74352451841686    -1.05962428502186
  H   6.73990307100197    -1.49300016488626     0.85430386852386
  H   9.05878636765386    -1.77917571030929     0.15729768671171
  H   8.03264097252099    -0.54861147885436    -3.85760010665228
  C   4.68817992923129    -0.42161864346604    -0.64516132504029
  C   5.41068426203090    -0.25947304855631    -3.39559936901235
  C   4.08280698885627    -0.12225377014021    -3.04243983994616
  C   3.75187985196973    -0.17318850874959    -1.65393893734028
  H   5.69373551219399    -0.18272625058883    -4.44989797197952
  C   4.32556181554788    -0.35402928629495     0.79642620973959
  C   5.15978287821400     0.30978184093912     1.76945681350680
  C   3.17982694721940    -0.98793121152539     1.27767243555500
  C   4.86159740637436     0.16892115463618     3.16773870918085
  C   3.68523664661307    -0.50972324826608     3.57625723623270
  C   2.82622777380330    -1.06688108901037     2.65089140703346
  C   6.30309078917857     1.08059653607214     1.42433351216949
  C   7.17799533956297     1.54722250738783     2.38333584726460
  C   6.93155171143193     1.31239946091319     3.76061203850815
  C   5.76418683400289     0.67697463360088     4.13878003892363
  H   5.53273702126443     0.52439727260803     5.19485334125971
  H   6.50973440315018     1.30336447398746     0.37741941277778
  H   8.05583223586894     2.10693065189921     2.05952802516173
  H   3.46101726576727    -0.60030242058828     4.64335163997795
  O   2.45262028913321     0.14867680777577    -1.32487159912499
  O   2.35369817020683    -1.67241779149751     0.41322082201360
  C   10.16730705492414    -1.15979422270046    -2.32821283237008
  C   10.93371449651189     0.06837238226742    -1.76538504180443
  C   10.86889149457926    -2.50125285476810    -1.97966985663028
  F   10.26898894405871    -1.04991812095785    -3.69865919474208
  F   12.22658082212611     0.04288045379888    -2.10880328454525
  F   10.85255498209444     0.12287890799456    -0.43048170920688
  F   10.39268080852640     1.18773884656354    -2.26730035275359
  F   12.02574218668009    -2.61989564870683    -2.63510756014381
```

F	11.12562531335031	-2.58827539793546	-0.66254469926812
F	10.08559968988463	-3.52671998644522	-2.32636075149058
C	7.91580191423852	1.74109291498365	4.82895509068734
C	7.93682213162284	3.28005543544148	5.02111456847085
C	9.33521907902938	1.16132261159353	4.57314652504593
F	9.93802238279902	1.79179350906623	3.55160130347326
F	9.24468435212314	-0.13866782943777	4.27586469628767
F	10.10714314997624	1.28704696901418	5.65495971467543
F	8.23450390872753	3.91717624190094	3.87681219998334
F	8.82677556140482	3.65179659203956	5.94623028783595
F	6.72480504386657	3.68308710713452	5.42443557310711
F	7.53988071280755	1.24125693575865	6.05796841686314
C	3.01141278776716	0.00699629252825	-4.07069540383864
C	2.70023827970454	1.23121164312448	-4.69302733751438
C	2.29735437630942	-1.15433978244692	-4.40878333412075
C	1.22714594532798	-1.09717729769215	-5.29741370965834
C	0.88167710472930	0.13209296197936	-5.85882097502121
C	1.62945123023757	1.30168632008174	-5.58733961564145
H	3.29372737812631	2.11362649109656	-4.44562693964162
H	2.59786406295929	-2.10479067332876	-3.96566788704512
H	0.65052596386081	-1.99993129563257	-5.51430123905290
C	-0.24407013877443	0.47575251104023	-6.71982332013088
C	1.02256041467992	2.49934630070179	-6.32761679692747
C	-0.17566004466643	1.85158976003812	-7.01038810428193
C	-1.29605317270870	-0.30940763381097	-7.20472306805535
C	-1.15260299752620	2.44873873568315	-7.80500170731086
C	-2.27542085642999	0.29580780030102	-7.99689806617456
C	-2.20340218728169	1.66335904798020	-8.29877537502347
H	-1.11431484923459	3.51817697324253	-8.03841292761065
H	-1.35707511672201	-1.37200570462553	-6.95261759371002
H	-3.10958699842001	-0.29918955824447	-8.38176474816752
H	-2.97900069368948	2.12337243410639	-8.91878310392269
C	1.60084243786508	-1.80517541100842	3.06815636144065
C	1.69126422117599	-3.17327625503473	3.38089976263439
C	0.36538065251502	-1.14115586567899	3.17692612503143
C	-0.77013343871050	-1.81446336839135	3.63431793030377
C	0.56287307948816	-3.84552782103518	3.84075614056419
C	-0.66065689009472	-3.16344812539525	3.99014254545096
H	0.29951285827776	-0.08742369043027	2.89634878072122
H	2.65235034449805	-3.68490243037488	3.26475316548983
H	-1.72275884973804	-1.28294702992672	3.71628271279392
C	-1.63051901740251	-4.08704579340775	4.57871981235425
C	0.43386275664147	-5.30666933825034	4.25160934659619
C	-1.00668232940132	-5.34618361046165	4.76842929776249
C	-2.94860567984856	-3.86184245879306	4.98854662323849
C	-1.72614334490246	-6.37440851577978	5.38273444423468
C	-3.04281350256437	-6.14354320002538	5.80732085815654
C	-3.64961399582857	-4.89881014061378	5.61192211276571
H	-3.41717835783073	-2.88505083777011	4.83206836267832
H	-4.67567390355900	-4.73951504545779	5.95637396203311
H	-1.28627638894597	-7.35944551997312	5.54740165594235
H	-3.60156982173954	-6.94957416845082	6.29163308242293
C	1.49399315945892	-5.67155958226050	5.32279090755648
C	0.64172452739745	-6.20618302110120	3.00110968159057
C	1.58959169231714	-7.16766500081853	5.63226196996649
H	1.29556173390916	-5.08923136784458	6.23897691797104
H	2.47478962583251	-5.33298873534997	4.93995261326372
C	1.77720847963180	-7.99910288522966	4.36017536745468
H	0.68782623532520	-7.50371590372625	6.17319296760793
H	2.43322073157472	-7.33935719937740	6.32369120419626
C	0.69186659565147	-7.69915556814377	3.32260469053965
H	1.79515223955141	-9.07592415857109	4.60351631285683
H	2.76437048201455	-7.76209512713177	3.91779520634255
H	-0.29200117136654	-8.04091336787720	3.69389713116538
H	0.88544115721025	-8.26598774565412	2.39456440894518
H	1.59890055903928	-5.91100576377148	2.53446808830658
H	-0.14247580986748	-5.98036956866593	2.25706235812779
C	0.52191559204995	3.61895219466322	-5.37356851137349
C	1.97766153379991	3.10405349371739	-7.39118489203608
C	1.63227040225423	4.50465390215471	-4.80834574807529

H	-0.06978741179728	3.15492641775359	-4.56757917205905
H	-0.17020719136151	4.25846747024626	-5.95349606814364
C	3.09058157104817	3.97023005119483	-6.79978330714667
H	1.36673509718552	3.73206750792014	-8.06740297770559
H	2.39183223438674	2.28736011592073	-8.00764412609602
C	2.51825839091855	5.07982922284301	-5.91454813352603
H	2.25275358174555	3.93493930436637	-4.09707864123846
H	1.18076954629906	5.31896595653007	-4.21418087450254
H	3.33211877685447	5.68670816098337	-5.48024165747576
H	1.91656109134959	5.76505417301533	-6.54421225371497
H	3.68995277121043	4.40401660767800	-7.61998249821171
H	3.78733224090872	3.34271142255532	-6.21450492315301
C	-3.55133537706419	6.41896998127577	3.47361409654580
C	-4.49510540326529	5.37611321707732	3.27091717673857
C	-4.19824470425677	4.32270034934529	2.43010021331265
C	-2.31954474981750	6.35564713700395	2.84959598590547
C	-1.98589401456759	5.27239647618915	1.99519324118718
C	-2.95565989598621	4.24662588100199	1.74727103274388
H	-4.93447371462020	3.52820714936573	2.29172625174123
H	-5.46036694884526	5.39695392572170	3.77789980989537
H	-1.57702838470231	7.13887382803128	3.01422348856461
C	-2.62089021522663	3.19626319173900	0.83212198097790
C	-0.70304855524619	5.18477010878417	1.40334445685961
C	-0.32047280631766	4.11978906054611	0.59661309225649
C	-1.33323907665223	3.14916367095982	0.30908968018606
H	0.01059414123682	5.98873199422915	1.60159361217456
C	-3.62620106019962	2.17265862356327	0.44605061025264
C	-4.86621911544808	2.55420025586508	-0.16315091222596
C	-3.37208950981115	0.81795527165312	0.64480558145180
C	-5.84107106069863	1.54249112078441	-0.43289134743928
C	-5.58032156117307	0.20541212940719	-0.05220852281936
C	-4.36887528069793	-0.19666046497762	0.49713247338822
C	-5.14014066117541	3.88007024841565	-0.58926104262893
C	-6.32329843492833	4.19991060429997	-1.22248137173296
C	-7.30421187949831	3.19905075952734	-1.45857758223303
C	-7.05497950779114	1.89440639286656	-1.07735113981185
H	-7.79119276204970	1.11234132278244	-1.27286786192728
H	-4.38945702837580	4.65722936202215	-0.43354562595912
H	-6.49130987602620	5.22668445985533	-1.54942174543586
H	-6.35422014147506	-0.54246253912958	-0.24369663254784
O	-1.03172763053018	2.16017895155237	-0.58267522274087
O	-2.07575050074668	0.43645593074172	0.96916411477939
C	-3.86013572586686	7.60587205287052	4.36337962203035
C	-4.98507198157264	8.48639665582493	3.75765477497828
C	-4.15154511499214	7.18035847280876	5.82743289175059
F	-2.76606819495156	8.44053079167399	4.44966476526839
F	-5.22453947407791	9.56385958921137	4.51255357921516
F	-6.12994070481774	7.79946310876019	3.63389377120724
F	-4.60575279557534	8.90090237556037	2.54471199206787
F	-5.32057448288573	6.52554357855944	5.92113151857314
F	-3.17770283491560	6.37221270260712	6.25792870851952
F	-4.20144354635448	8.24044956899423	6.63810452876412
C	-8.60809061661445	3.51795653167211	-2.16048107996270
C	-8.37487114093542	3.81225368621688	-3.66630605526404
C	-9.40355483517616	4.64323741957213	-1.44559218650692
F	-8.81214192580243	5.83722639940341	-1.60991238550389
F	-9.47152464853315	4.38017331141335	-0.13593113046175
F	-10.64958736988877	4.72643699076511	-1.91779716625931
F	-7.54823949545232	4.85201228508338	-3.83551635434126
F	-9.52414941974151	4.08140384366175	-4.29658885914029
F	-7.82700478525582	2.73495178609305	-4.23691923097006
F	-9.44670823444114	2.42237928190228	-2.13774710750066
C	1.07726909179589	3.98268391661203	0.12404323456867
C	2.11609520987513	4.47755357359758	0.95032804390956
C	1.42216762605343	3.36193750436310	-1.09744899638187
C	2.75730053701104	3.19514903667637	-1.46022743479636
C	3.76812844074813	3.67273100396059	-0.62689568546421
C	3.44554877145237	4.33796574797792	0.57413755341002
H	1.86308001004789	4.93677228089744	1.90936227875200

H	0.65421125288627	2.99161370537034	-1.77510874205781
H	2.99449967817100	2.67056054753732	-2.38685946507818
C	5.22051495894795	3.60805990761333	-0.75014867578131
C	4.70636470261013	4.79140720895180	1.30063910149459
C	5.79280086281583	4.26038753553082	0.36553529831147
C	6.02034091556953	2.99736643452223	-1.72322166460676
C	7.18073399132336	4.30974435371912	0.49581751404224
C	7.41069209047107	3.04051520511258	-1.57595243278636
C	7.98544034976759	3.69752106297830	-0.47832486820996
H	7.65194123872915	4.78823678595906	1.35909483107161
H	5.56713656699414	2.47356120801089	-2.57026948547756
H	8.05497795546482	2.55157014126417	-2.31221850351727
H	9.07476492729017	3.71827724209122	-0.37442414431398
C	-4.21119749595360	-1.59735721868257	0.96733421507647
C	-5.35923346278553	-2.22816797518635	1.50944211389695
C	-3.01098654546367	-2.34087950881653	0.90170943431899
C	-2.95710855877929	-3.66728359436276	1.33579662607191
C	-5.31205385580946	-3.55182816457211	1.92284674539408
C	-4.10956403833667	-4.27764582146847	1.83386004210491
H	-2.10903275921849	-1.90813188963523	0.47451321567987
H	-6.28152412987794	-1.65094025248373	1.62332421372161
H	-2.01304550135824	-4.21338584971899	1.27584699728326
C	-4.34430593560628	-5.62314127908113	2.34541682395196
C	-6.43042580467234	-4.40352168734999	2.51111042324845
C	-5.69616186233933	-5.72619873045750	2.76237142488595
C	-3.46478951101661	-6.70504680187711	2.44391135384711
C	-6.14764755665777	-6.93801619976565	3.29190217877853
C	-5.26419245655922	-8.02570198902941	3.38973691292848
C	-3.93485950392778	-7.91269376957941	2.96629238264494
H	-2.42069223647352	-6.59876230208756	2.13598157997957
H	-3.25615014695601	-8.76585000002879	3.06279563738916
H	-7.17554302820033	-7.06529561750743	3.63597318409090
H	-5.62264860483558	-8.97076715656030	3.80911318294118
C	-7.57729060688843	-4.54511367217154	1.47274178991235
C	-6.99297418145633	-3.74346418632757	3.79685240752738
C	-8.83061395510631	-5.23023315177528	2.01825239372917
H	-7.19034853541709	-5.07378843505164	0.58406525330128
H	-7.84708154923212	-3.52581218254235	1.13746727180536
C	-9.33986361677172	-4.53074865079985	3.28152523312362
H	-8.61681016290149	-6.29221132419780	2.23553348447866
H	-9.61558341112319	-5.22979680116482	1.24099330457003
C	-8.24664300389867	-4.42441255557016	4.34775721267805
H	-10.22319862558005	-5.05632338029472	3.68370403019866
H	-9.67825482137035	-3.51117678479858	3.01265900273228
H	-7.99501600582542	-5.42783564991277	4.73489787917268
H	-8.61839331601554	-3.85199587595113	5.21537844685610
H	-7.24427614186825	-2.69609942861228	3.54489874417922
H	-6.19628588676447	-3.69990401428918	4.55707111094295
C	4.73563933777140	6.34812875325112	1.41566416310357
C	4.83625484797825	4.15951919210971	2.72023576061805
C	5.62654077667040	6.82214585521164	2.56326949677689
H	3.70467413434420	6.70592799373328	1.59051415023738
H	5.05273228063473	6.77561020159681	0.44979501059061
C	4.26446587964056	5.05784736261841	3.81488515733941
H	5.90469774385922	3.99337114982341	2.92814774482235
H	4.36416178933842	3.16275983916268	2.71836092045443
C	5.07223536848845	6.36487647959314	3.92963314333834
H	5.72615943638054	7.92044588039279	2.53678565474788
H	6.64644546258338	6.42640027584998	2.41784949746171
H	4.43593604549907	7.15534247742950	4.36522806743113
H	5.90937556148916	6.22021193717028	4.63424240861899
H	4.26591636082803	4.52253287684709	4.77871169841765
H	3.20566245132609	5.28253108145901	3.59762871603341
P	1.39117189188767	-0.87624761066575	-0.63532577349405
P	-1.06943841069093	0.54792185575839	-0.33170616654801
N	0.35283787924020	0.04446143003225	0.13516520547001
N	0.75524375891304	-1.97863207852492	-1.59830159497025
S	1.38798369256736	-3.40056209802646	-1.92953271815454
O	2.85228904486786	-3.44889268666747	-2.01894173394714
O	0.59198100208675	-4.05681457397332	-2.97475326044818

C	1.01662696211632	-4.40896196159360	-0.32696866920174
F	0.18208703241348	-3.75002209389161	0.48841740027307
F	0.43378207672101	-5.58478165089354	-0.64049297474091
F	2.14177552895295	-4.66706910151389	0.33109196232096
N	-1.84458874705839	-0.13911751708446	-1.55735108620198
S	-1.44214274306040	0.20486042777838	-3.07039294600099
O	-1.61362874528382	-0.95422468044545	-3.95014627078398
O	-0.22693301224942	1.02932451787820	-3.17437849248858
C	-2.85639411680280	1.34094096853244	-3.57186836681206
F	-2.71215272708049	1.70432345733594	-4.84624607989181
F	-4.02698441353824	0.70782006153013	-3.42871777769396
F	-2.87163972119529	2.44128388102016	-2.80857223378375
C	-1.37611780626275	-6.26607127188703	-3.67740203240869
C	-1.89639917649876	-4.95126053342299	-4.25367485674134
C	-2.47167622487883	-7.33423704147359	-3.44955328695894
H	-0.82702231011559	-6.07113164613793	-2.74470561217847
H	-0.62549192405391	-6.68356877411894	-4.36986184764377
C	-2.92982275888616	-4.19420498716953	-3.44696184318399
H	-2.28050094817897	-5.11448441188167	-5.27742995274498
H	-1.01672731523787	-4.27438430070456	-4.30693973716682
C	-4.30939580763938	-5.72065334814250	-3.02656501888621
C	-3.49883590221198	-6.79828460858397	-2.48313358368652
H	-2.00549859236250	-8.25313001351721	-3.05394307205170
H	-2.94836561964110	-7.58293492801041	-4.41454070029838
C	-2.48461825837035	-3.61702576162156	-2.13957384616639
H	-1.85161956924652	-2.73701956032440	-2.35814291640016
H	-1.88091255333596	-4.30379706968096	-1.53244290621667
H	-3.33912058561491	-3.25869566437455	-1.54653361002364
C	-3.77434455323105	-3.24920612174007	-4.26147235590908
H	-3.10765535046378	-2.45812359769913	-4.64909679504379
H	-4.52975213194862	-2.73600716984233	-3.64600898367733
H	-4.26256560348644	-3.74669054785499	-5.11494163454419
C	-5.49831683136229	-5.26766613359859	-2.24638125016584
H	-4.55183794297007	-5.89409033605568	-4.08642051690223
C	-5.00652298744907	-8.25983330178871	-2.51061164108998
C	-5.97716972279759	-7.65777812811918	-1.67809589125279
C	-6.59958047484161	-6.41294409904841	-2.21385788137912
H	-5.97178481938774	-4.38610608336141	-2.70687796764635
H	-5.23710279077546	-4.98731987529704	-1.21082859956658
H	-7.44511362296043	-6.07154324223516	-1.59496619944070
H	-6.95661929187871	-6.55969080890162	-3.24621834312750
C	-4.73416103753935	-9.69694090897736	-2.18974305387209
H	-5.19630098616751	-8.10280581847093	-3.58115834719572
C	-6.12864024638282	-10.35027065180598	-2.35797871727064
H	-4.35868541224215	-9.84059263604222	-1.16379626174120
H	-4.01499801102080	-10.16913155527677	-2.87702161216314
O	-7.10099558184782	-9.42375626404975	-1.84643085931906
H	-6.20039059200789	-11.30253864932435	-1.80428518145805
H	-6.31426696312870	-10.54858137826585	-3.42869925219185
C	-3.03058643498357	-6.76150561881548	-1.04825191662500
H	-3.66486295241311	-6.15248487619773	-0.39250419443677
H	-2.96248446005538	-7.77632259958181	-0.62544771462316
H	-2.01573876206431	-6.33469796268481	-1.00197723095410
C	-6.00417131649065	-7.79634871516654	-0.18904633394230
H	-5.47781702927199	-8.67883231436858	0.19446145398364
H	-5.54733737375836	-6.90639981621313	0.27774443118112
H	-7.04418413464062	-7.83087578030784	0.16957700696594
H	-7.89732408647345	-9.45622666838081	-2.39333179448171

3.1.3 Supplementary Discussion

The results obtained in the deuterium labeling study revealed position 3eq of (−)-ambrox (**2a**) as major site of protonation. Based on this observation a plausible transition state model was created (see below).

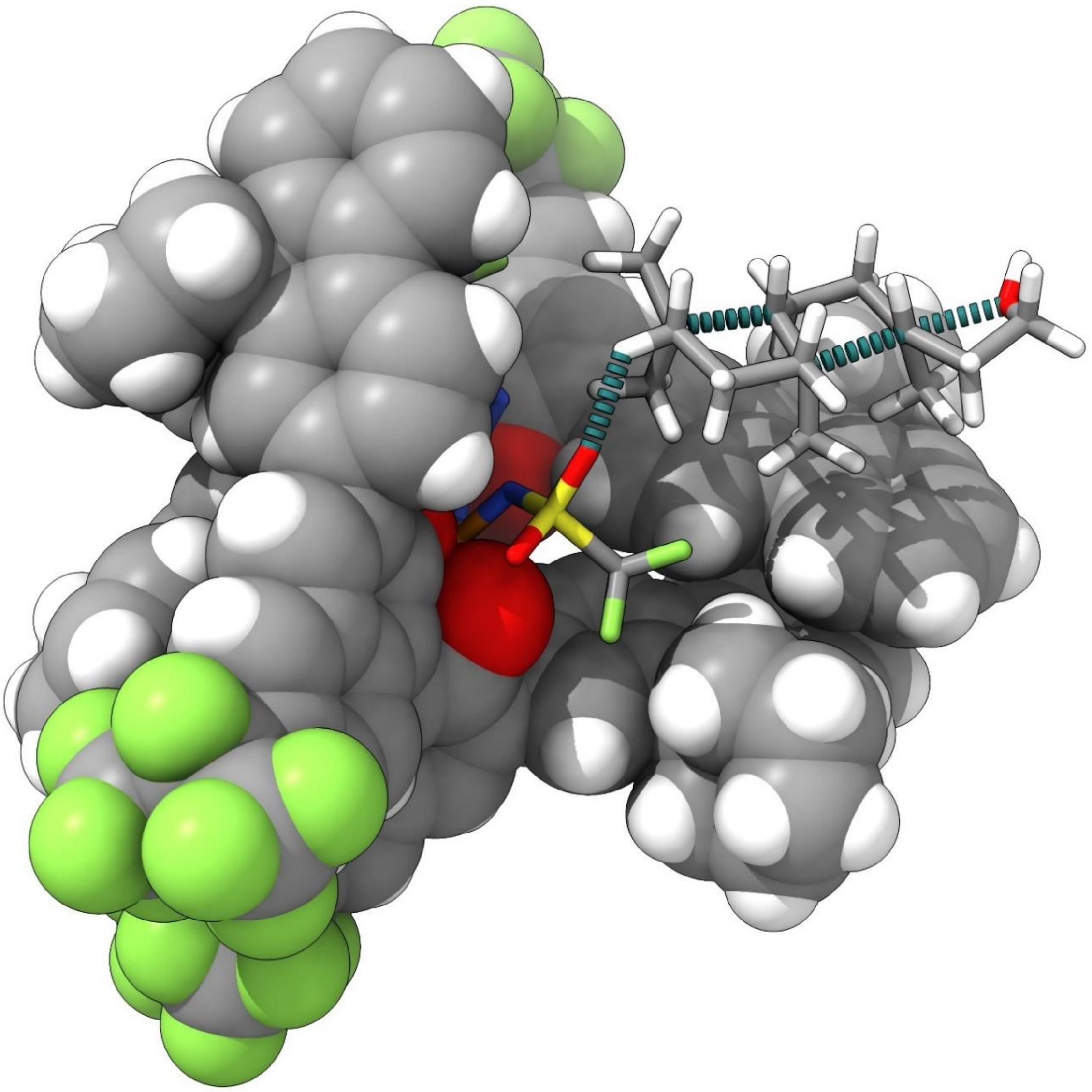

Fig. S76 | DFT-calculated structure of the ion pair of protonated substrate (3*E*,7*E*)-homofarnesol (**1a**, protonation at the terminal double bond, corresponding to position 3eq in the product **2a**) and IDPi **8g**. Atomic distance between the three bonds (2 × C–C, 1 × C–O) to be formed to provide (−)-ambrox (**2a**) are fixed to 2.1 Å. Bonds that are about to be formed are indicated in dark green.

The result of the preliminary DFT-study is supported by several observations of the deuterium labeling experiment. The hydrogen atoms of the methyl groups at the terminal double bond position point into the catalyst pocket and are polarized as a result of the adjacent carbocation. Non-classical C–H···O interactions between the sulfonyl oxygens and the hydrogen atoms pointing inward are observed. This suggests that a concerted protonation (or deuteronation) / deprotonation at the terminal double could occur that would lead to the formation of a 1,1-disubstituted double bond species. A minor amount of deuterium at the terminal double bond was indeed observed in the $^{2}H\{^{1}H\}$ NMR spectrum. Deuteronation of the formed 1,1-disubstituted double bond species followed by a polyene cyclization

would then furnish tricycle **2a** with deuterium incorporation at either methyl group of the *gem*-dimethyl moiety. This assumption is corroborated by minor amounts of deuterium at positions 18 and 19.

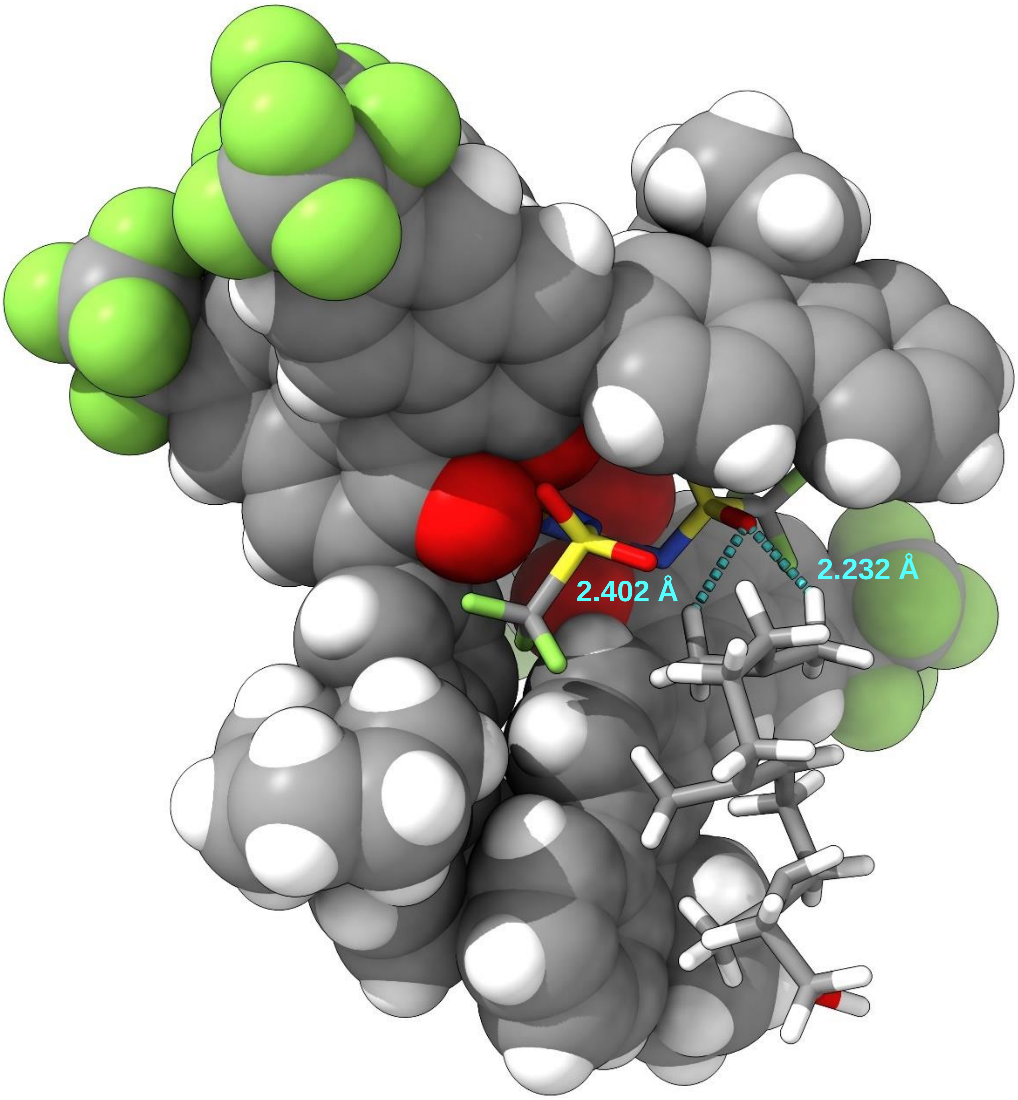

Fig. S77 | Depiction of the plausible ion pair structure with nonclassical C–H···O interactions highlighted in cyan.

Putative carbocationic intermediates or transient positive charge can be efficiently stabilized by cation–π interactions considering the close proximity of the respective positively charged positions to the aromatic fluorenyl-substituent of IDPi catalyst **8g**. This would support a concerted process wherein stabilization of transient charges is crucial to ensure full conversion to the fully cyclized product.

Another interesting observation is the accessibility of the hydroxy group of homofarnesol **1a**. It can be speculated that a second IDPi molecule could act as a base to pick up the proton that has to be released upon completion of the cyclization sequence. The rapid distribution, exchange, and transfer of protons is most likely facilitated by a mechanism which is similar to the one proposed by Grotthuß for protons in aqueous solutions.[55,56]

4 Copies of GC Traces

GC trace of (3*E*,7*E*)-homofarnesol (**1a**).

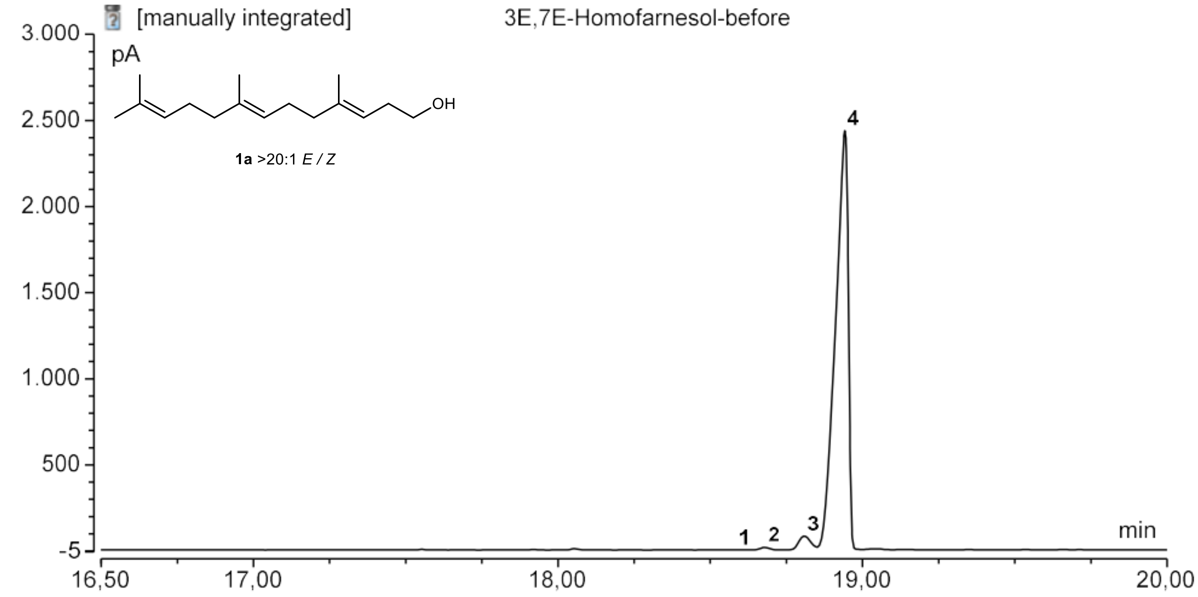

peak #	t_R / min	relative area / %	peak name
1	18.61	0.00	(3*Z*,7*Z*)-homofarnesol (**1d**)
2	18.90	0.30	(3*E*,7*Z*)-homofarnesol (**1c**)
3	18.81	2.40	(3*Z*,7*E*)-homofarnesol (**1b**)
4	18.89	97.30	(3*E*,7*E*)-homofarnesol (**1a**)

GC trace of (3*Z*,7*E*)-homofarnesol (**1b**).

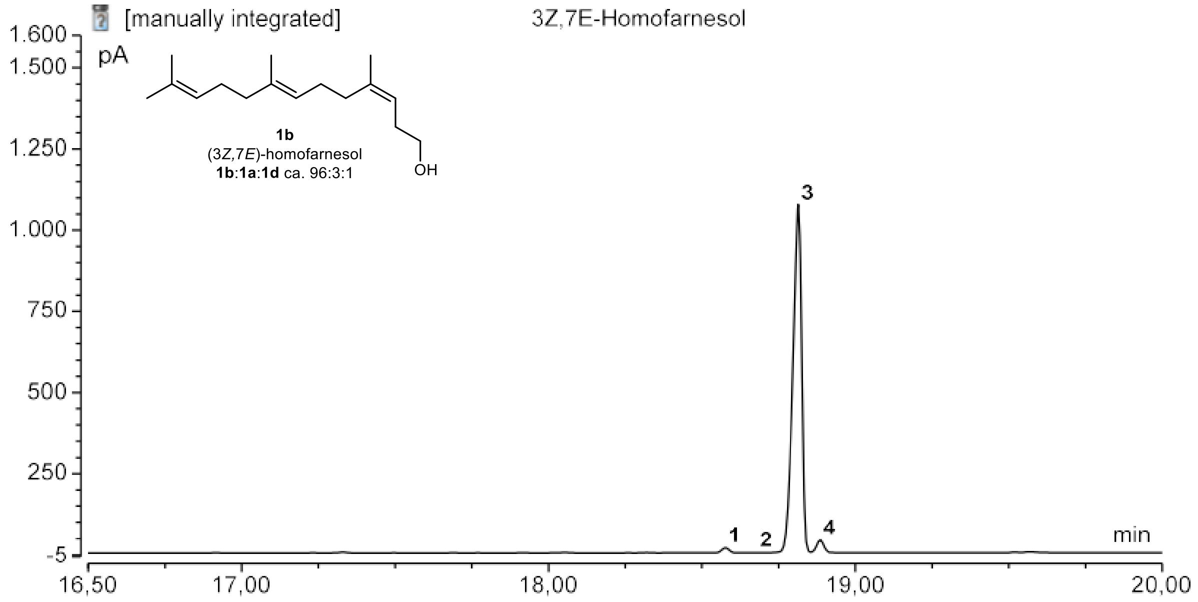

peak #	t_R / min	relative area / %	peak name
1	18.61	1.06	(3*Z*,7*Z*)-homofarnesol (**1d**)
2	18.90	0.00	(3*E*,7*Z*)-homofarnesol (**1c**)
3	18.81	96.29	(3*Z*,7*E*)-homofarnesol (**1b**)
4	18.89	2.65	(3*E*,7*E*)-homofarnesol (**1a**)

GC trace of (3*E*,7*Z*)-homofarnesol (**1c**).

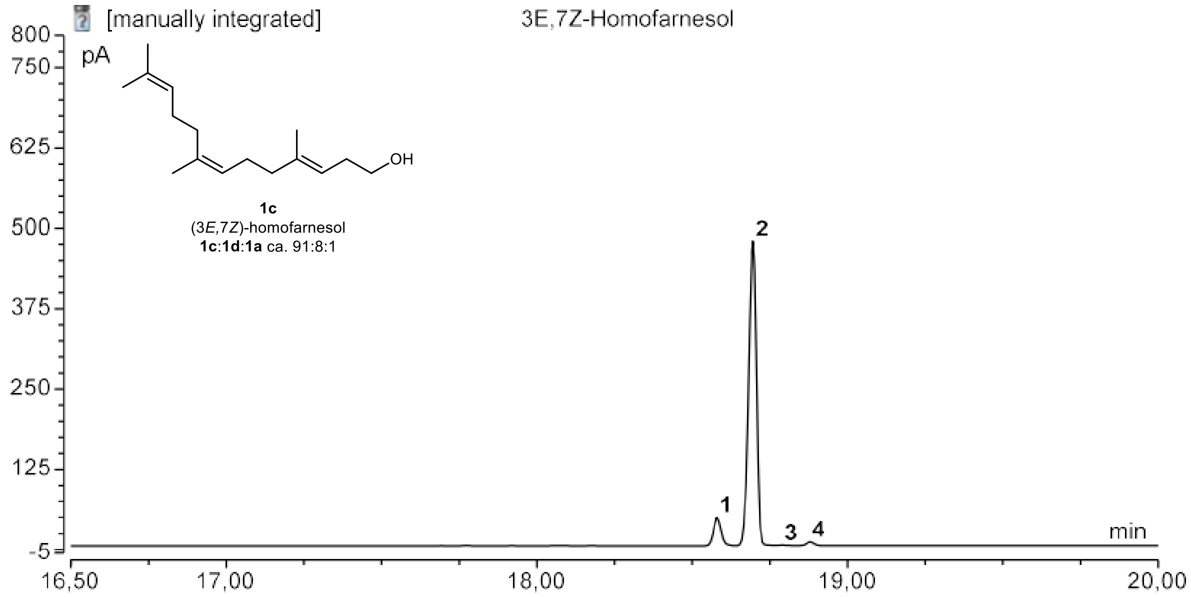

peak #	t_R / min	relative area / %	peak name
1	18.61	8.40	(3*Z*,7*Z*)-homofarnesol (**1d**)
2	18.90	90.48	(3*E*,7*Z*)-homofarnesol (**1c**)
3	18.81	0.07	(3*Z*,7*E*)-homofarnesol (**1b**)
4	18.89	1.06	(3*E*,7*E*)-homofarnesol (**1a**)

GC trace of (3*Z*,7*Z*)-homofarnesol (**1d**).

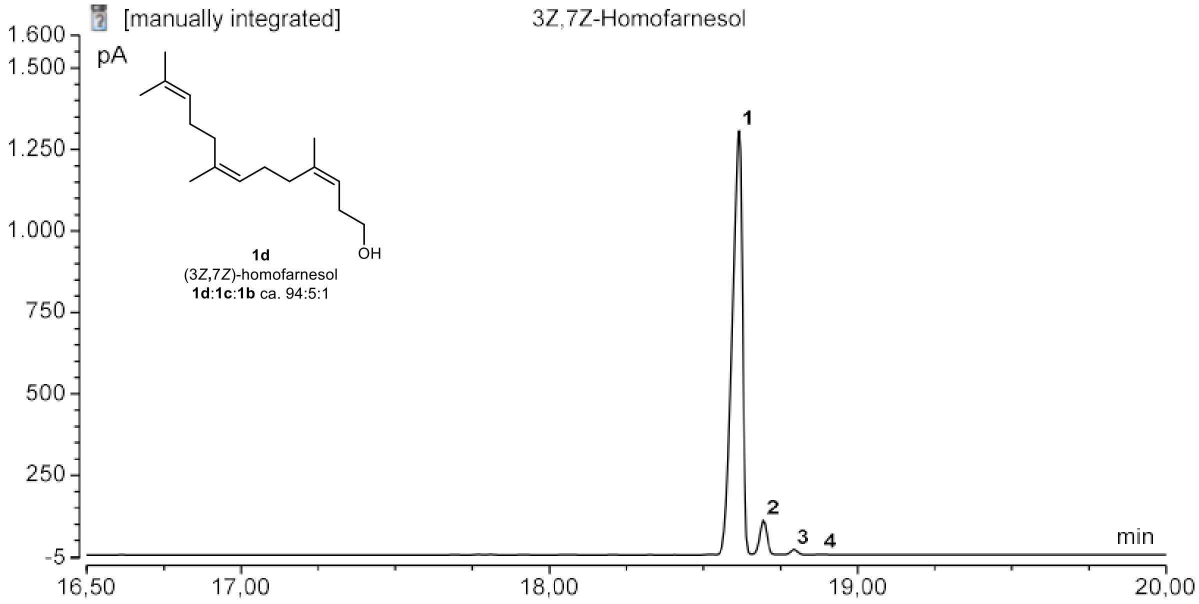

peak #	t_R / min	relative area / %	peak name
1	18.61	94.19	(3*Z*,7*Z*)-homofarnesol (**1d**)
2	18.90	5.05	(3*E*,7*Z*)-homofarnesol (**1c**)
3	18.81	0.73	(3*Z*,7*E*)-homofarnesol (**1b**)
4	18.89	0.03	(3*E*,7*E*)-homofarnesol (**1a**)

GC trace of technical (3*E*/*Z*,7*E*)-homofarnesol (*E*)-**1a** / (*Z*)-**1b** ca. 57:43.

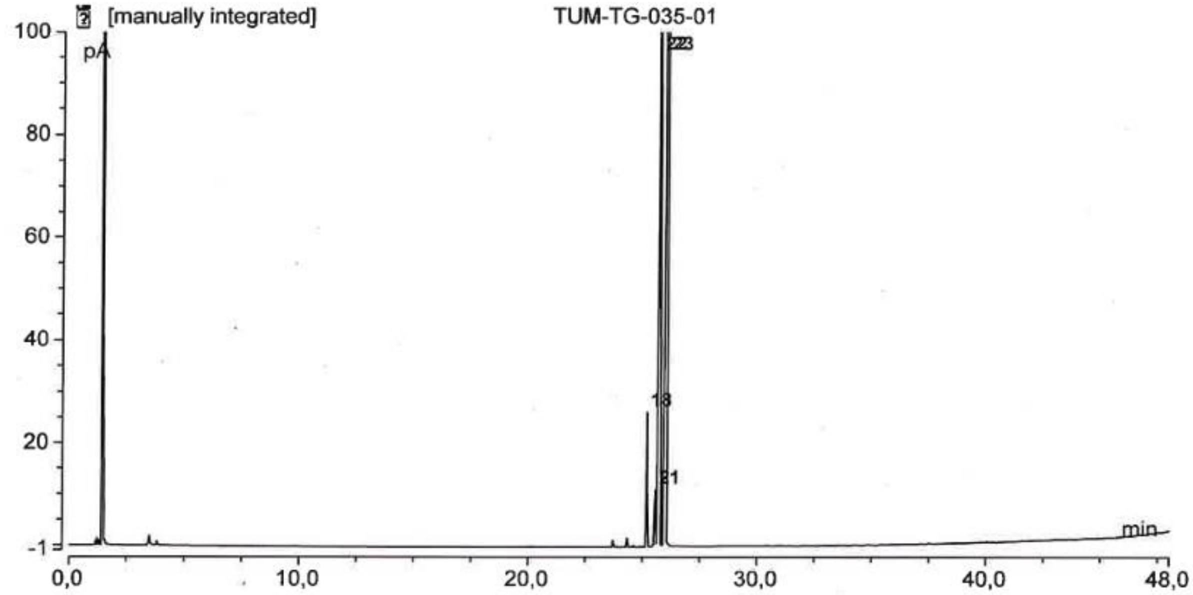

**GC (achiral)** (Optima-35 0.25/0.25df G/706, 29.0 m, temperature: 220/ 60 5/min to 300/350, 0.60 bar H_2, sample size: 0.2 µ, split ratio: 60:1).			
**peak #**	t_R **/ min**	**relative area / %**	**peak name**
18	25.18	3.51	(2*E*,7*E*)-homofarnesol (**1b**)
21	25.55	1.82	(2*Z*,7*E*)-homofarnesol (**1a**)
22	25.71	40.26	(3*Z*,7*E*)-homofarnesol (**1b**)
23	26.00	54.41	(3*E*,7*E*)-homofarnesol (**1a**)

GC trace of (3*E*,7*E*)-homofarnesol as reference (**1a**).

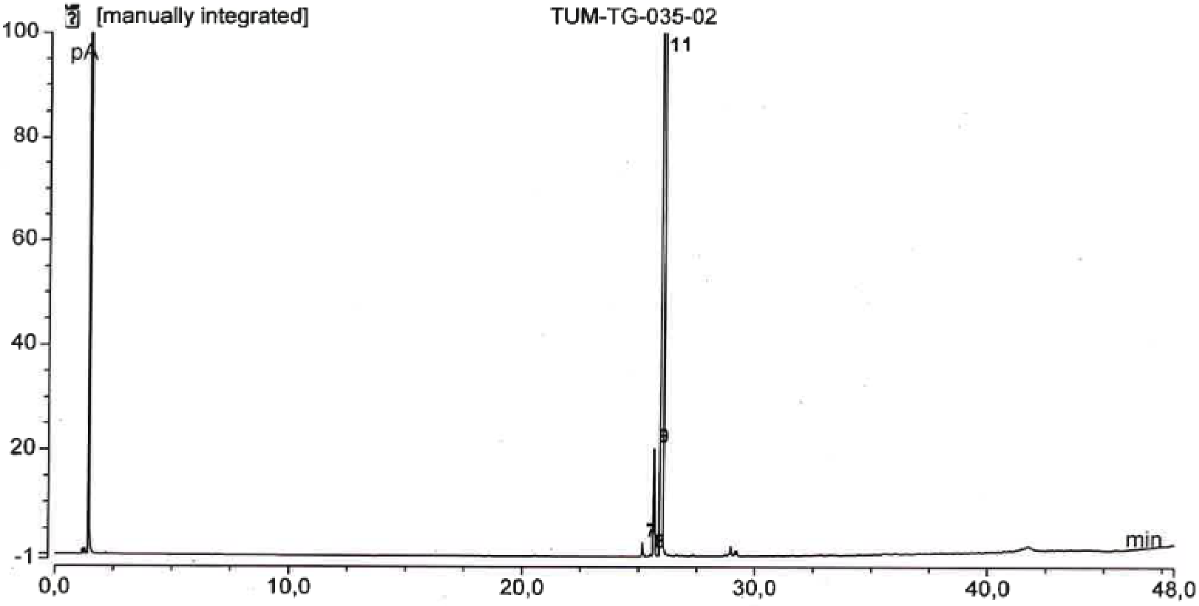

**peak #**	t_R **/ min**	**relative area / %**	**peak name**
9	25.67	2.92	(3*Z*,7*E*)-homofarnesol (**1b**)
11	26.03	94.97	(3*E*,7*E*)-homofarnesol (**1a**)

GC trace (chiral stationary phase) of a synthetic sample containing ambrox (**2a**), 9-*epi*-ambrox (**2b**), 5β,8α,9β-ambrox (**2c**) and 5β-ambrox (**5e**) prepared via polyene cyclization of (±)-(*E*)--α-cyclohomofarnesol (**3a**) at 20 °C using (*S*,*S*)-IDPi catalyst **8d** in HFIP.

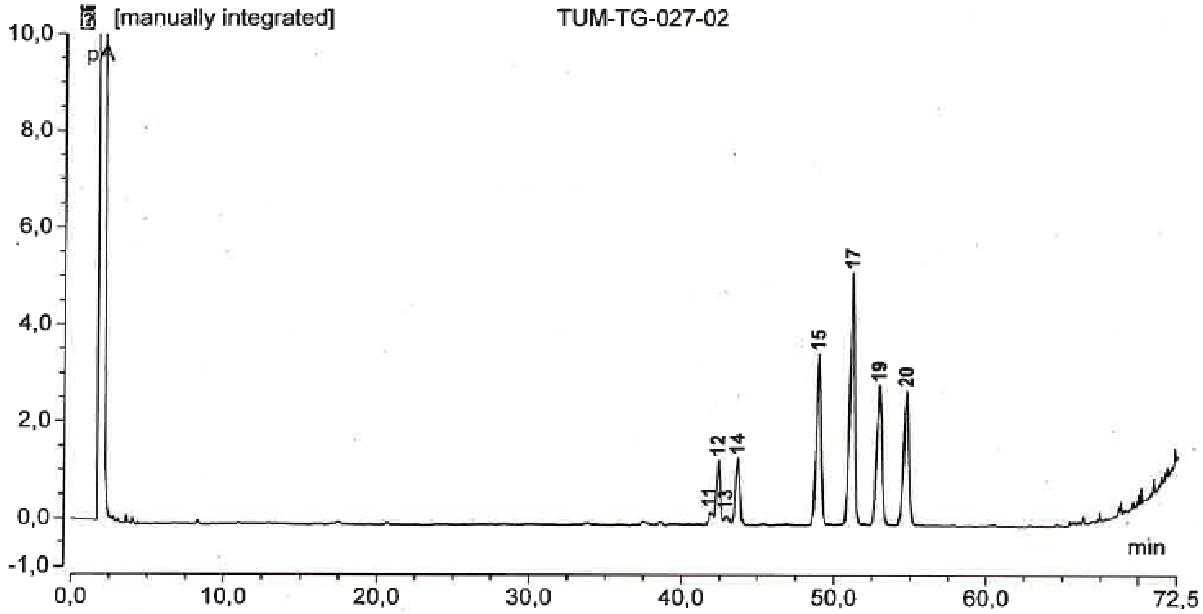

**GC (chiral)** (BGB 176/BGB-15 0.25/0.25df G/618, 30.0 m; temperature: 220/140, 60 min iso 8/min 240/ 350, 0.60 bar H_2, sample size: 1.0 µL).				
**peak #**	t_R **/ min**	**area / %**	**peak name**	**structure**
11	41.90	1.28	(−)-9-*epi*-ambrox (**2b**)	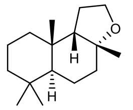
12	42.38	6.58	5β-ambrox (**2e**)	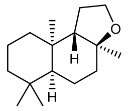
13	42.95	1.06	(+)-9-*epi*-ambrox (*ent*-**2b**)	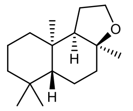
14	43.66	6.99	*ent*-5β-ambrox (*ent*-**2e**)<br>+5β,8α-ambrox (**2d**)	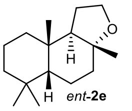 *ent*-**2e** + 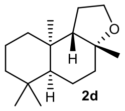 **2d**
15	48.93	19.63	(−)-ambrox (**2a**)	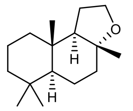
17	51.12	30.18	(+)-ambrox (*ent*-**2a**)	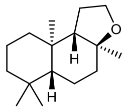
19	52.93	17.17	5β,8α,9β-ambrox (**2c**)	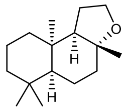
20	54.70	17.12	5β,8α,9β-ambrox (*ent*-**2c**)	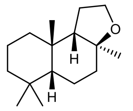

Synthetic sample of (±)-9-*epi*-ambrox (**2b**) and (±)-5β,8α-ambrox.

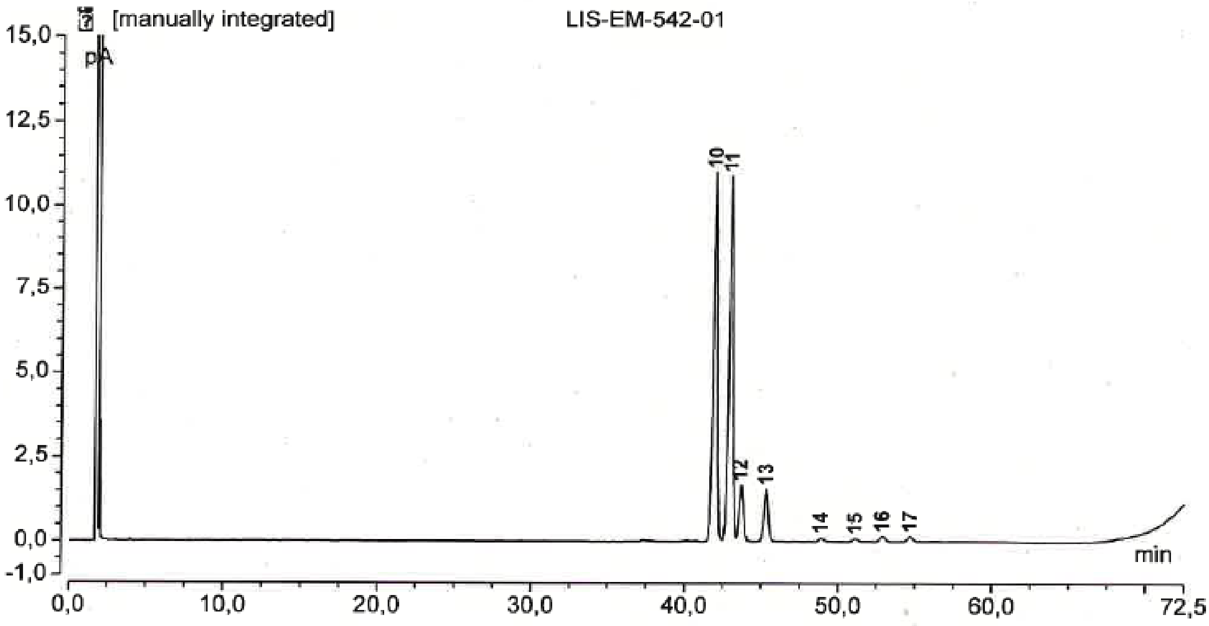

**GC (chiral)** (BGB 176/BGB-15 0.25/0.25df G/618, 30.0 m; temperature: 220/140, 60 min iso 8/min 240/ 350, 0.60 bar H_2, sample size: 1.0 µL).				
**peak #**	**t_R / min**	**area / %**	**peak name**	**structure**
10	41.92	42.28	(−)-9-*epi*-ambrox (**2b**)	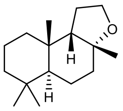
11	42.95	42.10	(+)-9-*epi*-ambrox (*ent*-**2b**)	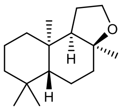
12	43.70	6.51	5β,8α-ambrox (**2d**)	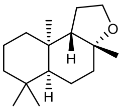
13	45.31	6.37	*ent*-5β,8α-ambrox (*ent*-**2d**)	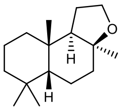
14	48.89	0.51	(−)-ambrox (**2a**)	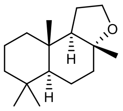
15	51.13	0.54	(+)-ambrox (*ent*-**2a**)	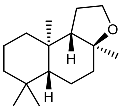
16	52.89	0.88	5β,8α,9β-ambrox (**2c**)	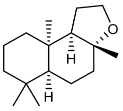
17	54.69	0.81	5β,8α,9β-ambrox (*ent*-**2c**)	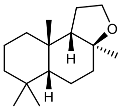

Chiral GC trace of a commercially available reference standard (authentic sample) of (−)-ambrox (**2a**).

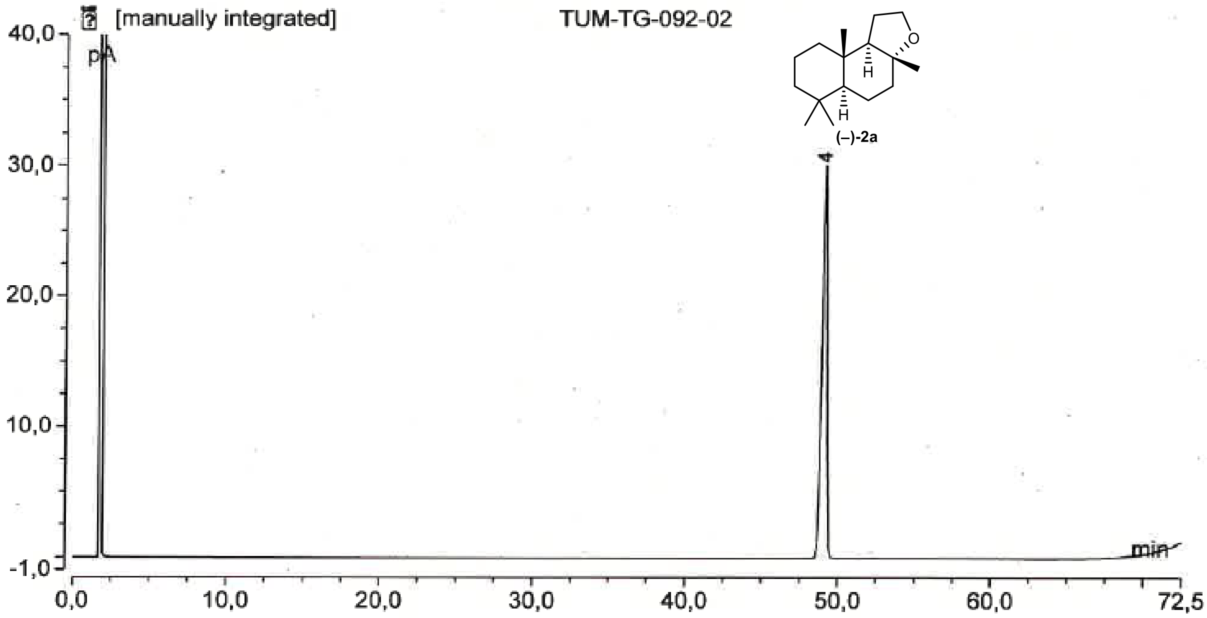

**GC (chiral)** (BGB 176/BGB-15 0.25/0.25df G/618, 30.0 m; temperature: 220/140, 60 min iso 8/min 240/ 350, 0.60 bar H_2, sample size: 1.0 µL).			
**peak #**	t_R **/ min**	**relative area / %**	**peak name**
4	49.11	100	(−)-ambrox (**2a**)

Chiral GC trace of synthetic (−)-9-*epi*-ambrox (**2b**) as reference material.

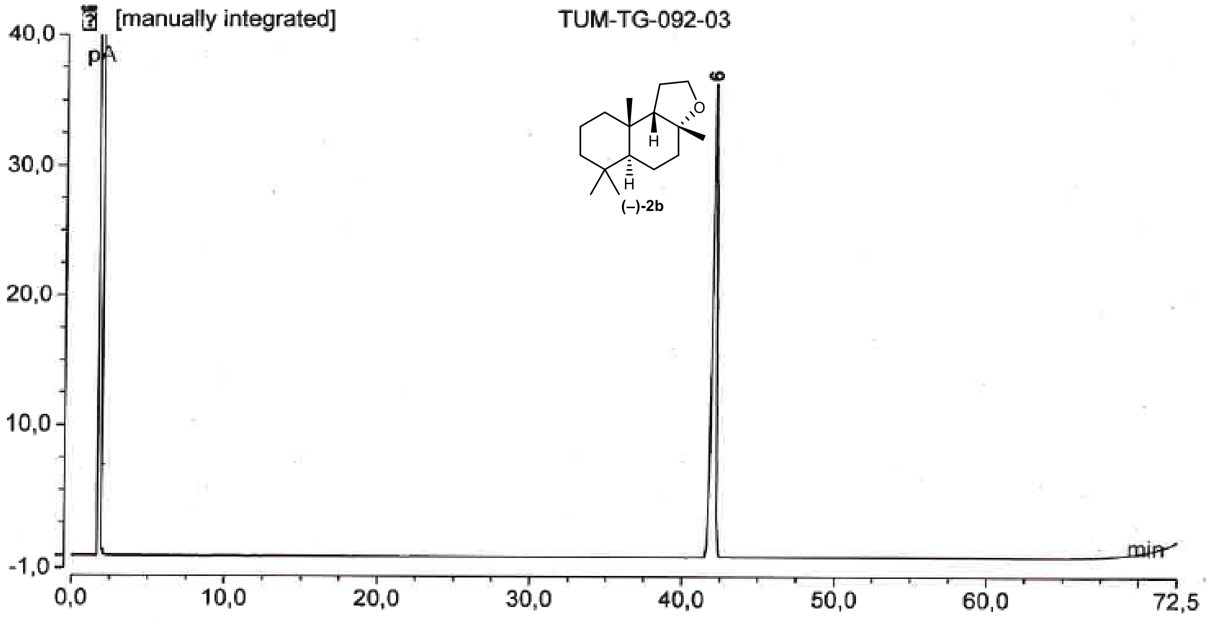

**GC (chiral)** (BGB 176/BGB-15 0.25/0.25df G/618, 30.0 m; temperature: 220/140, 60 min iso 8/min 240/ 350, 0.60 bar H_2, sample size: 1.0 µL).			
**peak #**	t_R **/ min**	**area / %**	**peak name**
6	42.06	100	(−)-9-*epi*-ambrox (**2b**)

Achiral GC trace of a sample of **2a** prepared at −40 °C in PFTB with IDPi catalyst **8g**.

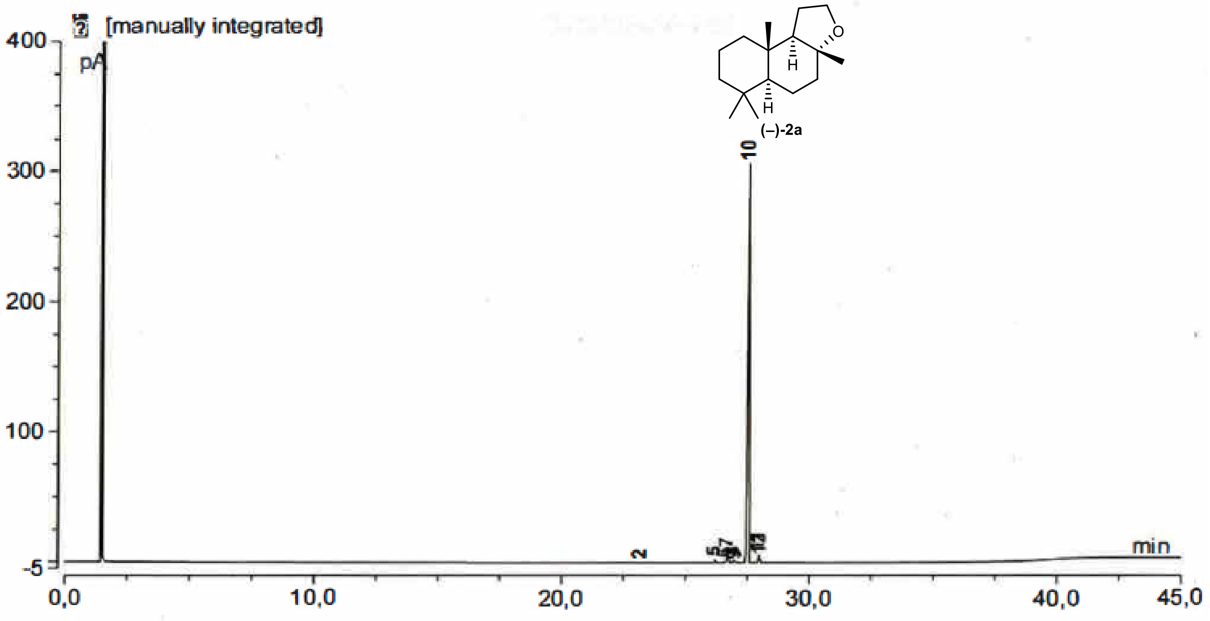

GC (achiral) (Optima-35 0.25/0.25df G/706, 29.0 m; temperature: 220/50 5/min 200 12/min 350, 5 min iso/ 350, 0.60 bar H_2, sample size: 0.2 µL, split ratio: 120:1).				
**peak #**	***t*R / min**	**area / %**	**peak name**	**Structure**
7	26.71	2.29	9-*epi*-ambrox (**2b**)	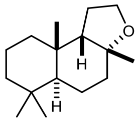 **2b**
8	26.96	0.62	5β-ambrox (**2e**)	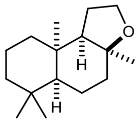 **2e**
9	27.03	0.02	5β,8α-ambrox (**2d**)	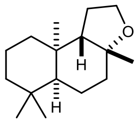 **2d**
10	27.54	95.42	ambrox (**2a**)	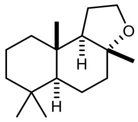 **2a**
11	27.97	1.65	5β,8α,9β-ambrox (**2c**)	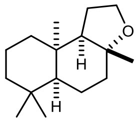 **2c**

Chiral GC trace of a sample of **2a** prepared at −40 °C in PFTB with IDPi catalyst **8g**.

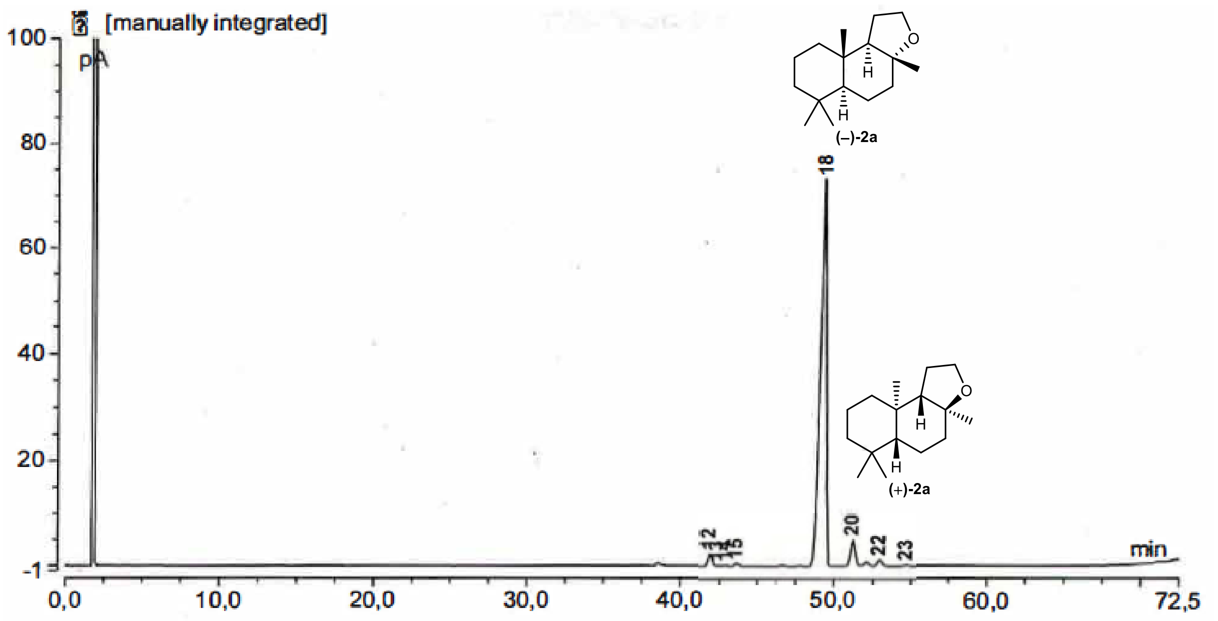

**GC (chiral)** (BGB 176/BGB-15 0.25/0.25df G/618, 30.0 m; temperature: 220/140, 60 min iso 8/min 240/ 350, 0.60 bar H_2, sample size: 1.0 µL).				
**peak #**	t_R **/ min**	**area / %**	**peak name**	**structure**
12	41.88	2.06	(−)-9-*epi*-ambrox (**2b**)	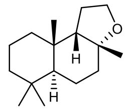
13	42.38	0.10	5β-ambrox (**2e**)	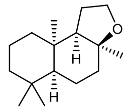
14	42.94	0.20	(+)-9-*epi*-ambrox (*ent*-**2b**)	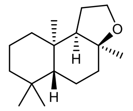
15	43.64	0.49	*ent*-5β-ambrox (**2e**)<br>+5β,8α-ambrox (**2d**)	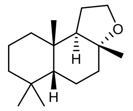
18	49.38	90.61	(−)-ambrox (**2a**)<br>95:5 e.r.	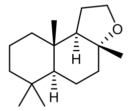
20	51.17	4.96	(+)-ambrox (*ent*-**2a**)	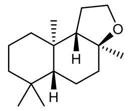
22	52.95	1.25	5β,8α,9β-ambrox (**2c**)	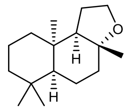
23	54.70	0.33	5β,8α,9β-ambrox (*ent*-**2c**)	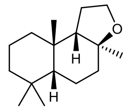

Achiral GC trace of a sample of **2a** prepared at −40 °C → 0 °C in PFTB with IDPi catalyst **8g**.

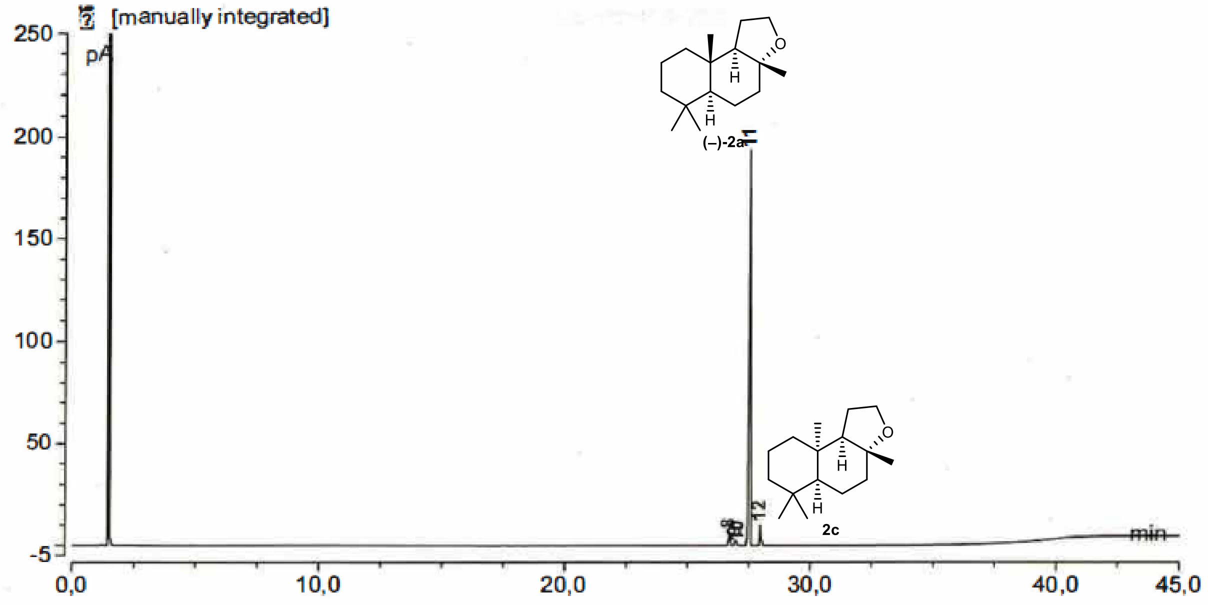

**GC (achiral)** (Optima-35 0.25/0.25df G/706, 29.0 m; temperature: 220/50 5/min 200 12/min 350, 5 min iso/ 350, 0.60 bar H_2, sample size: 0.2 µL, split ratio: 120:1).				
**peak #**	**t_R / min**	**area / %**	**peak name**	**structure**
8	26.71	2.91	9-*epi*-ambrox (**2b**)	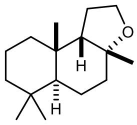 **2b**
9	26.97	1.31	5β-ambrox (**2e**)	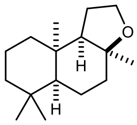 **2e**
10	27.04	0.19	5β,8α-ambrox (**2d**)	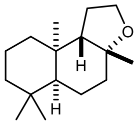 **2d**
11	27.52	90.73	ambrox (**2a**)<br>91:9 d.r.	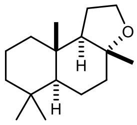 **2a**
12	27.97	4.86	5β,8α,9β-ambrox (**2c**)	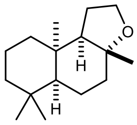 **2c**

Chiral GC trace of a sample containing **2a** prepared at −40 °C → 0 °C in PFTB with IDPi catalyst **8g**.

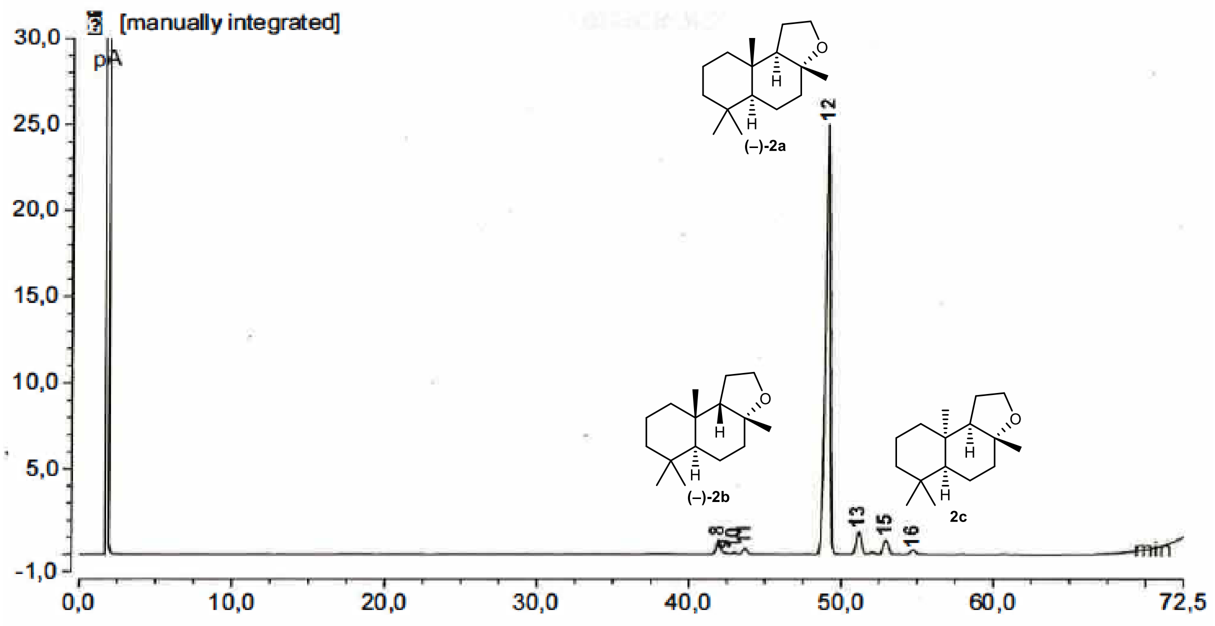

GC (chiral) (BGB 176/BGB-15 0.25/0.25df G/618, 30.0 m; temperature: 220/140, 60 min iso 8/min 240/ 350, 0.60 bar H_2, sample size: 0.2 µL).				
**peak #**	**t_R / min**	**area / %**	**peak name**	**structure**
8	41.92	2.61	(−)-9-*epi*-ambrox (**2b**)	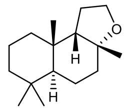
9	42.41	0.28	5β-ambrox (**2e**)	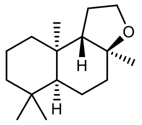
10	42.96	0.31	(+)-9-*epi*-ambrox (*ent*-**2b**)	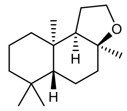
11	43.70	1.14	*ent*-5β-ambrox (**2e**)<br>+5β,8α-ambrox (**2d**)	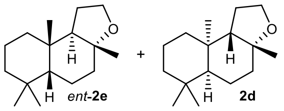
12	49.08	86.86	(−)-ambrox (**2a**)<br>91:9 d.r., 95:5 e.r.	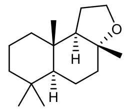
13	51.14	4.71	(+)-ambrox (*ent*-**2a**)	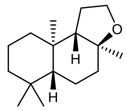
15	52.94	3.24	5β,8α,9β-ambrox (**2c**)	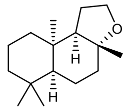
16	54.71	1.03	5β,8α,9β-ambrox (*ent*-**2c**)	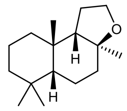

Achiral GC trace of **2a**-d_1 (ca. 33% D) prepared at −40 °C in PFTB-d_1 with IDPi catalyst **8g**.

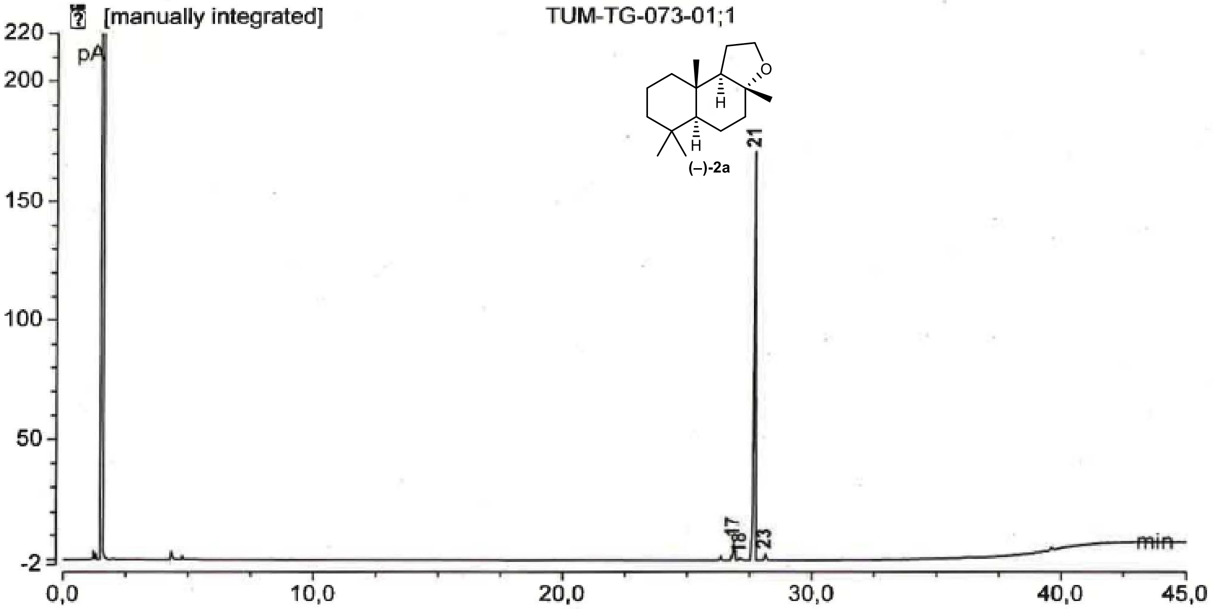

**GC (achiral)** (Optima-35 0.25/0.25df G/706, 29.0 m; temperature: 220/50 5/min 200 12/min 350, 5 min iso/ 350, 0.60 bar H_2, sample size: 1.0 µL).				
**peak #**	t_R **/ min**	**area / %**	**peak name**	**Structure**
17	26.87	4.00	9-*epi*-ambrox (**2b**)	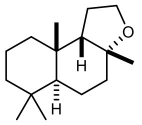 **2b**
18	27.13	0.62	5β-ambrox (**2e**)	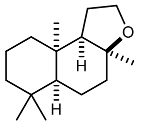 **2e**
21	27.67	93.81	ambrox (**2a**)<br>94:6 d.r.	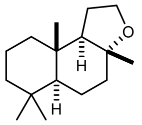 **2a**
23	28.13	1.57	5β,8α,9β-ambrox (**2c**)	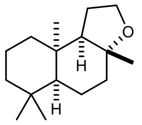 **2c**

Chiral GC trace of **2a**-d_1 (ca. 33% D) prepared at −40 °C in PFTB-d_1 with IDPi catalyst **8g**.

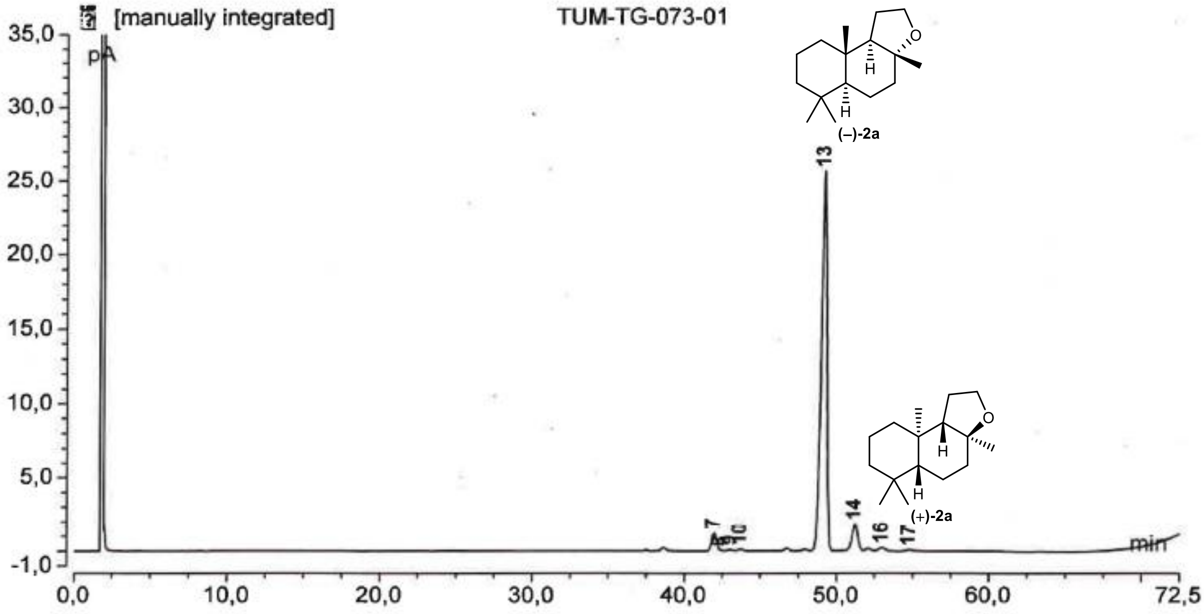

GC (chiral) (BGB 176/BGB-15 0.25/0.25df G/618, 30.0 m; temperature: 220/140, 60 min iso 8/min 240/ 350, 0.60 bar H_2, sample size: 1.0 µL).

peak #	t_R / min	area / %	peak name	Structure
7	41.96	3.66	(−)-9-*epi*-ambrox (**2b**)	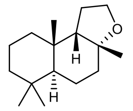
8	42.38	0.09	5β-ambrox (**2e**)	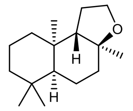
9	43.01	0.35	(+)-9-*epi*-ambrox (*ent*-**2b**)	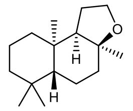
10	43.71	0.48	*ent*-5β-ambrox (*ent*-**2e**)	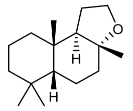
12	49.17	87.11	(−)-ambrox (**2a**)<br>92.5:7.5 e.r.	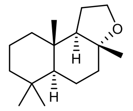
13	51.20	6.91	(+)-ambrox (*ent*-**2a**)	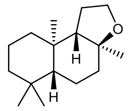
16	52.98	1.01	5β,8α,9β-ambrox (**2c**)	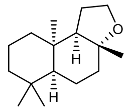
17	54.78	0.40	5β,8α,9β-ambrox (*ent*-**2c**)	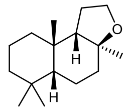

Achiral GC data for **2a**-d_1 (ca. 33% D) prepared at −40 °C in PFTB-d_1 with PADI catalyst **9**.

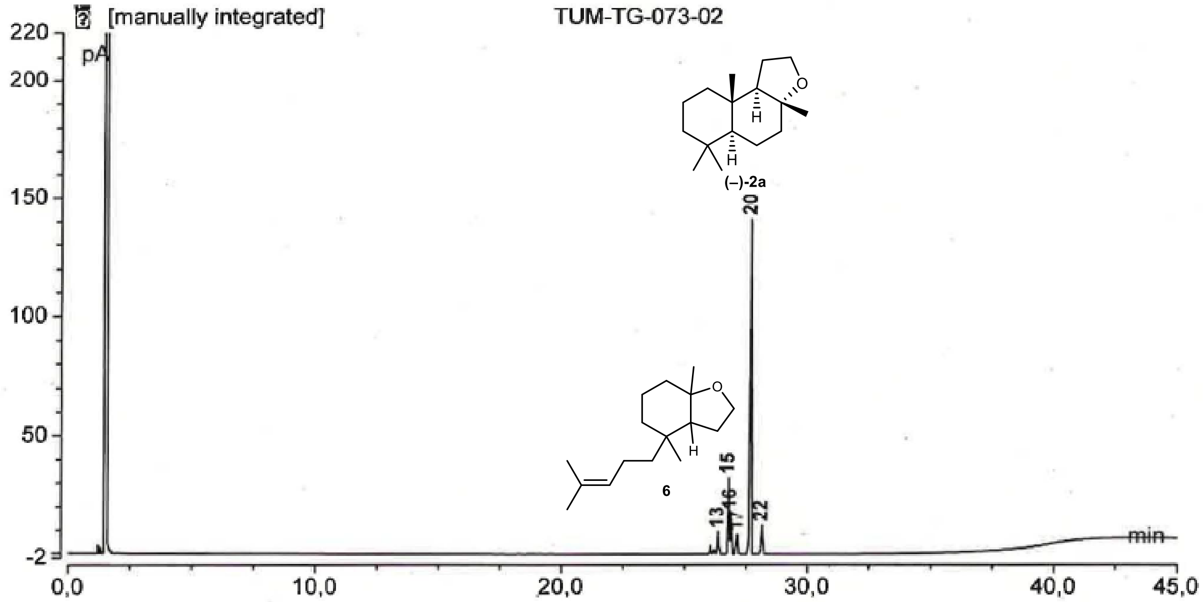

**GC (achiral)** (Optima-35 0.25/0.25df G/706, 29.0 m; temperature: 220/50 5/min 200 12/min 350, 5 min iso/ 350, 0.60 bar H_2, sample size: 1.0 µL).				
**peak #**	t_R **/ min**	**area / %**	**peak name**	**structure**
13	26.37	4.37	partially cyclized side product **6**	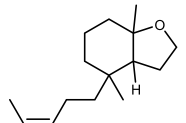 **6**
15	26.80	13.21		
16	26.88	7.06	9-*epi*-ambrox (**2b**)	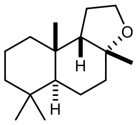 **2b**
17	27.14	4.02	5β-ambrox (**2e**)	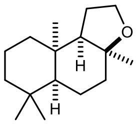 **2e**
20	27.68	64.82	ambrox (**2a**) 79:21 d.r.	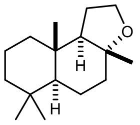 **2a**
22	28.14	6.61	5β,8α,9β-ambrox (**2c**)	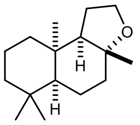 **2c**

Chiral GC data for **2a**-d_1 (ca. 33% D) prepared at −40 °C in PFTB-d_1 with PADI catalyst **9**.

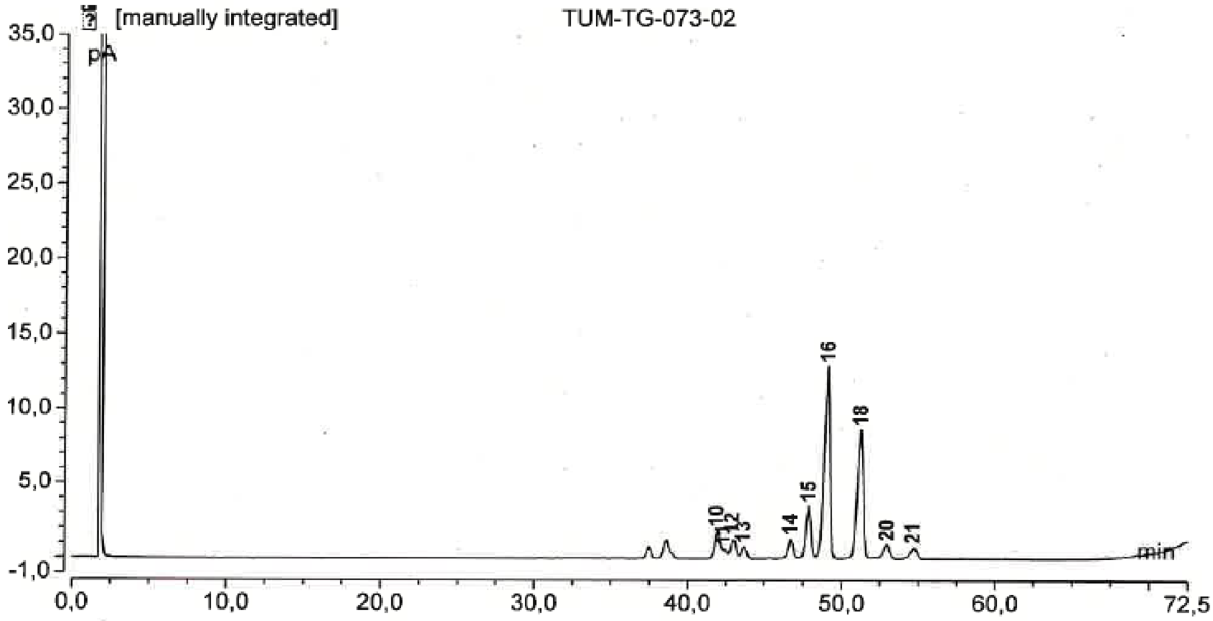

**GC (chiral)** (BGB 176/BGB-15 0.25/0.25df G/618, 30.0 m; temperature: 220/140, 60 min iso 8/min 240/ 350, 0.60 bar H_2, sample size: 1.0 µL).				
**peak #**	***t*$_R$ / min**	**area / %**	**peak name**	**structure**
10	41.92	5.22	(−)-9-*epi*-ambrox (**2b**)	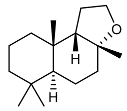
11	42.37	1.43	5β-ambrox (**2e**)	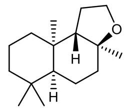
12	42.96	3.80	(+)-9-*epi*-ambrox (*ent*-**2b**)	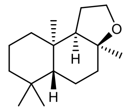
13	43.71	0.48	*ent*-5β-ambrox (*ent*-**2e**)	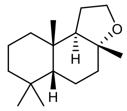
14	46.69	3.68	partially cyclized compound **6**	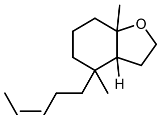 6
15	47.84	9.79		
16	49.04	39.94	(−)-ambrox (**2a**)	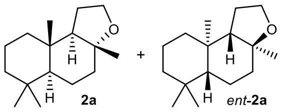
18	51.19	27.51	(+)-ambrox (*ent*-**2a**)	
20	52.94	3.49	5β,8α,9β-ambrox (**2c**)	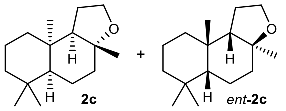
21	54.73	2.80	5β,8α,9β-ambrox (*ent*-**2c**)	

Achiral GC trace of **3**-d_1 (ca. 33% D) prepared at −40 °C in PFTB with IDPi catalyst **8g**.

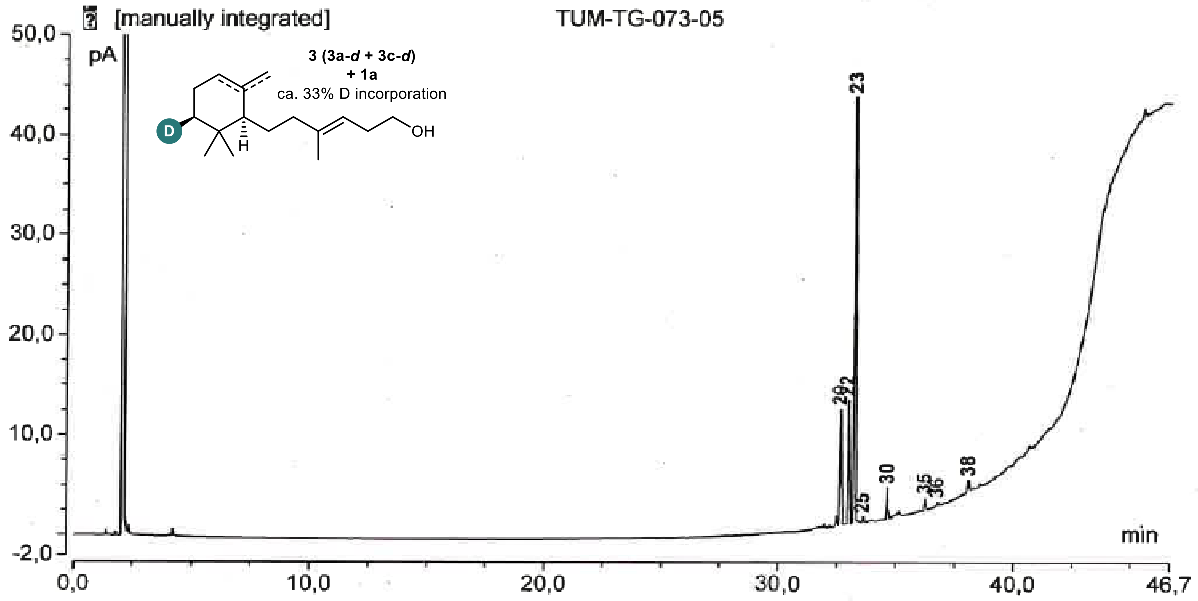

**GC (achiral)** (DB-Waxetr 0.25/0.25df G/770, 30.0 m; temperature: 220/50 5/min 260 12/min 280, 3 min iso/ 350, 0.60 bar H_2, sample size: 1.0 µL, split ratio: 120:1).				
**peak #**	**t_R / min**	**area / %**	**peak name**	**structure**
20	32.66	14.20	(*E*)-γ-cyclohomofarnesol **3c**	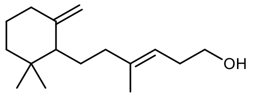 (*E*)-**3c**
22	32.99	12.10	(3*E*,7*E*)-homofarnesol **1a**	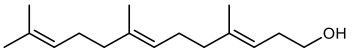 (3*E*,7*E*)-homofarnesol (**1a**)
23	33.25	53.55	(*E*)-α-cyclohomofarnesol **3a**	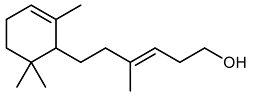 (*S*)-(*E*)-**3a**
25	33.63	0.81	(*E*)-β-cyclohomofarnesol **3b**	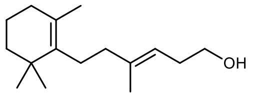 (*E*)-**3b**

Chiral GC trace of (±)-(*E*)-α-cyclohomofarnesol (**3a**).

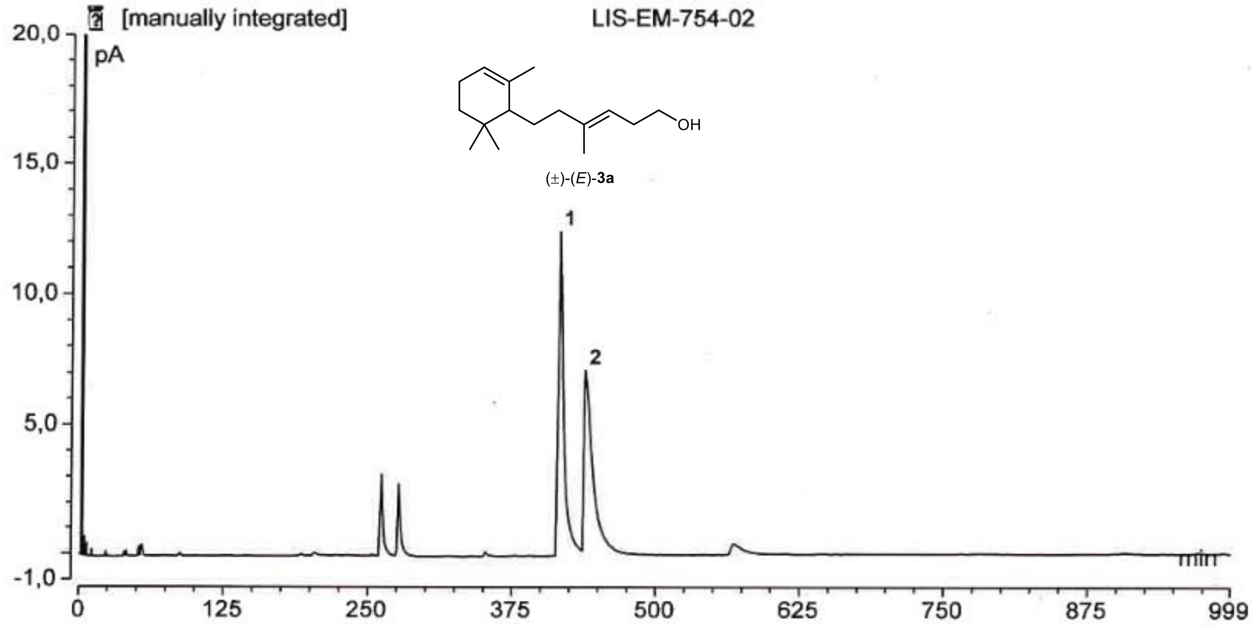

**GC (chiral)** (BGB-176/BGB-15, 0.25/0.25df G/618, 30.0 m; temperature: 220/110 470 min iso 8/min 240/ 350, 0.60 bar H_2, sample size: 1.0 µL, split ratio: 20:1).			
**peak #**	***t*$_R$ / min**	**area / %**	**peak name**
1	415.57	49.26	(*S*)-(*E*)-α-cyclohomofarnesol (**3a**)
2	437.98	50.74	(*R*)-(*E*)-α-cyclohomofarnesol (*ent*-**3a**)

Chiral GC trace of **2a**-d_1 (ca. 33% D) prepared at −40 °C in PFTB with IDPi catalyst **8g**.

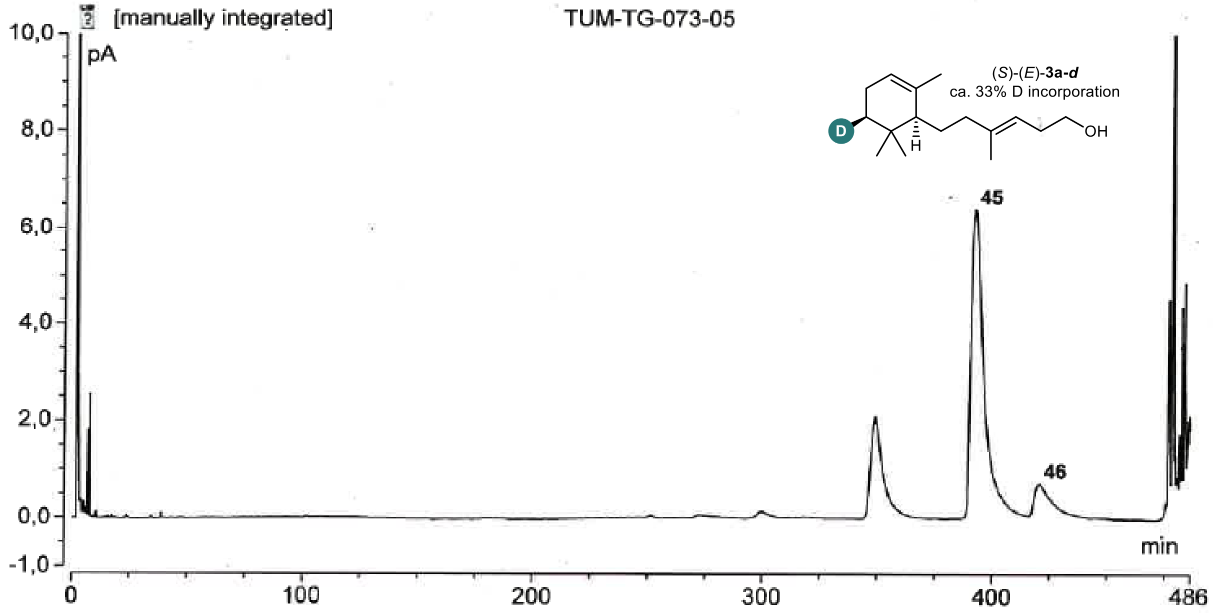

**GC (chiral)** (BGB-176/BGB-15, 0.25/0.25df G/618, 30.0 m; temperature: 220/110 470 min iso 8/min 240/ 350, 0.60 bar H_2, sample size: 1.0 µL, split ratio: 10:1).			
**peak #**	**t_R / min**	**area / %**	**peak name**
45	391.95	87.27	(*S*)-(*E*)-α-cyclohomofarnesol (**3a**)
46	420.62	12.73	(*R*)-(*E*)-α-cyclohomofarnesol (*ent*-**3a**)

Achiral GC trace of a sample containing homodrimenols (**4**).

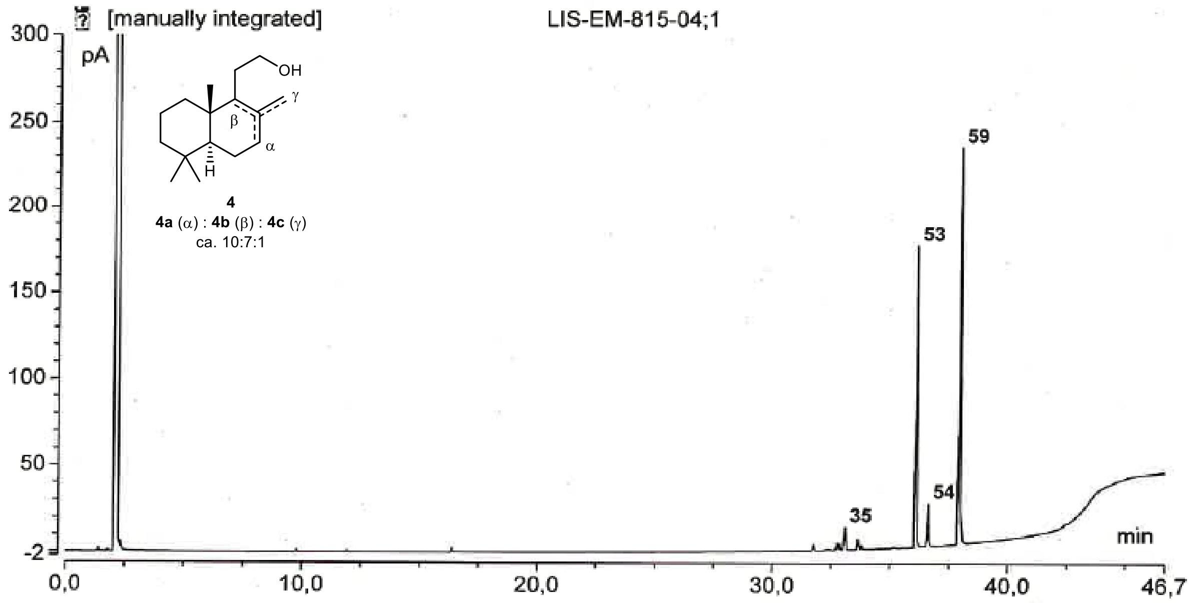

**GC (achiral)** (DB-Waxetr 0.25/0.25df G/770; 30.0 m, temperature: 220/50 5/min 260 12/min 280, 3 min iso/ 350, 0.60 bar H_2, sample size: 1.0 µL, split ratio 40:1).				
**peak #**	***t*R / min**	**area / %**	**peak name**	**structure**
53	36.11	34.67	β-homodrimenol **4b**	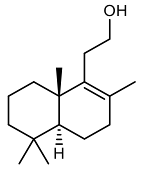 **4b**
54	36.62	4.91	γ-homodrimenol **4c**	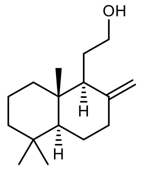 **4c**
59	37.96	50.61	α-homodrimenol **4a**	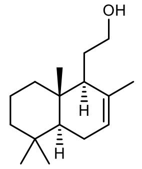 **4a**

GC chromatogram (achiral stationary phase) for a mixture of partially cyclized side products **5a**, **5b**, and **5c** obtained with a 1:1 mixture of (*S*,*S*)- and (*R*,*R*)-IDPi **8g** from (±)-(*E*)-γ-cyclohomofarnesol (**3c**). The sample serves as reference for the separation of regioisomers and diastereomers.

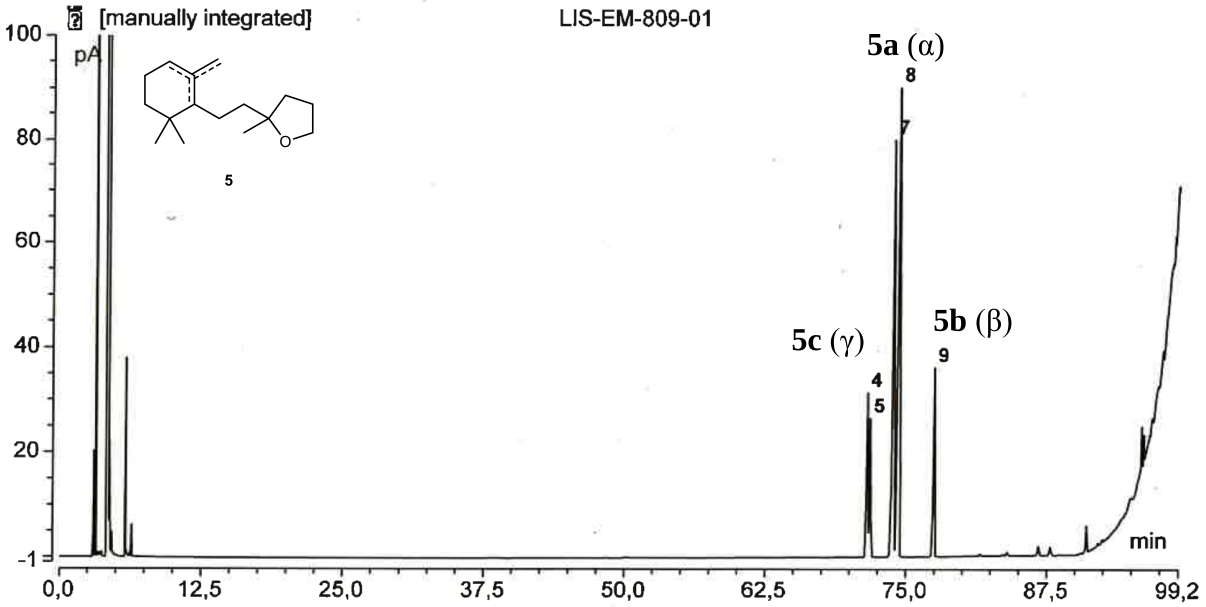

**GC (achiral)** (Stabilwax 0.25/0.25df, G/353 60.0 m, temperature: 220/60 1/min 150 12/min 260/350, 1.10 bar H_2, sample size: 1.0 µL, split ratio: 20:1).				
**peak #**	***t*$_R$ / min**	**area / %**	**peak name**	**structure**
4	71.58	10.47	**(*S*)-5c**	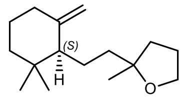 (*S*)-**5c**
5	71.82	8.07	**(*R*)-5c**	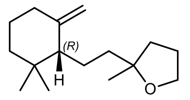 (*R*)-**5c**
7	73.89	33.35	**(*R*)-5a**	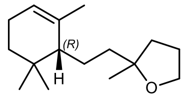 (*R*)-**5a**
8	74.35	36.00	**(*S*)-5a**	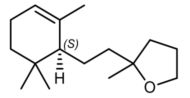 (*S*)-**5a**
9	77.49	12.10	**5b**	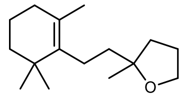 **5b**

GC chromatogram (achiral stationary phase) for a mixture predominantly containing partially cyclized side products **5a** and **5b** obtained with a 1:1 mixture of (*S*,*S*)- and (*R*,*R*)-IDPi **8g** from (±)-(*E*)-α-cyclohomofarnesol (**3a**). The sample serves as reference for the separation and assignment of regioisomers and diastereomers.

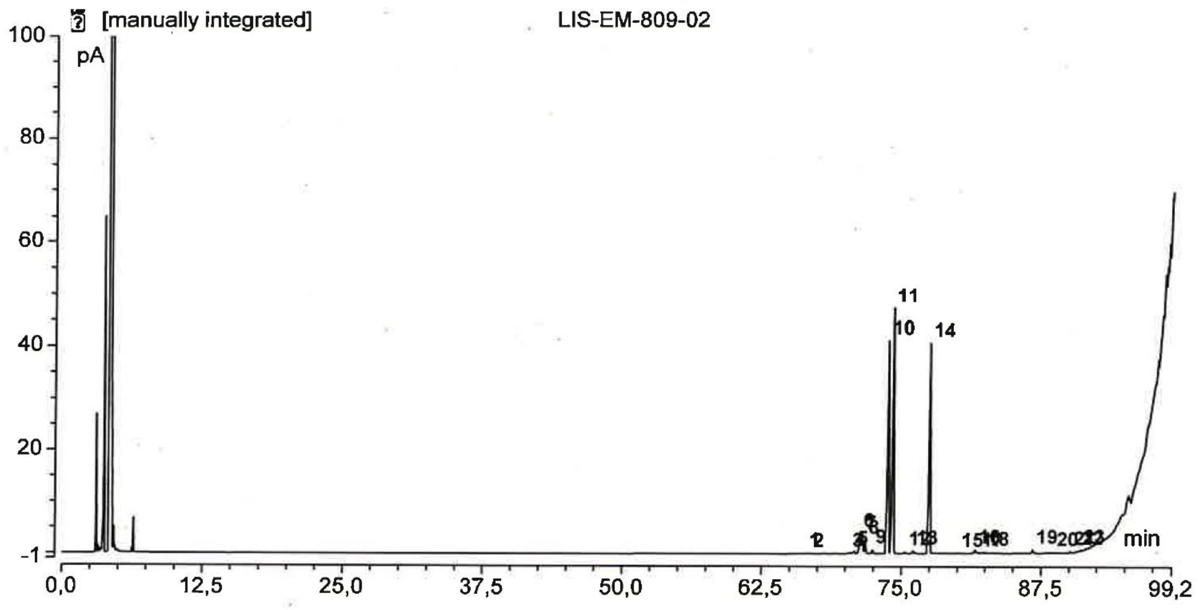

**GC (achiral)** (Stabilwax 0.25/0.25df, G/353 60.0 m, temperature: 220/60 1/min 150 12/min 260/350, 1.10 bar H_2, sample size: 1.0 µL, split ratio: 20:1).				
**peak #**	**t_R / min**	**area / %**	**peak name**	**structure**
7	71.50	2.15	**(*S*)-5c**	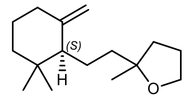 (*S*)-**5c**
8	71.76	1.73	**(*R*)-5c**	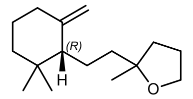 (*R*)-**5c**
10	73.79	28.94	**(*R*)-5a**	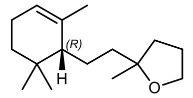 (*R*)-**5a**
11	74.23	32.21	**(*S*)-5a**	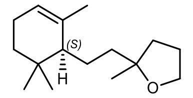 (*S*)-**5a**
14	77.48	27.75	**5b**	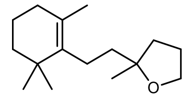 **5b**

GC chromatogram (achiral stationary phase) for a mixture predominantly containing partially cyclized side product **5b** obtained with a 1:1 mixture of (*S*,*S*)- and (*R*,*R*)-IDPi **8g** from (*E*)-β-cyclohomofarnesol (**3b**). The sample serves as reference for the separation and assignment of diastereomers and regioisomers.

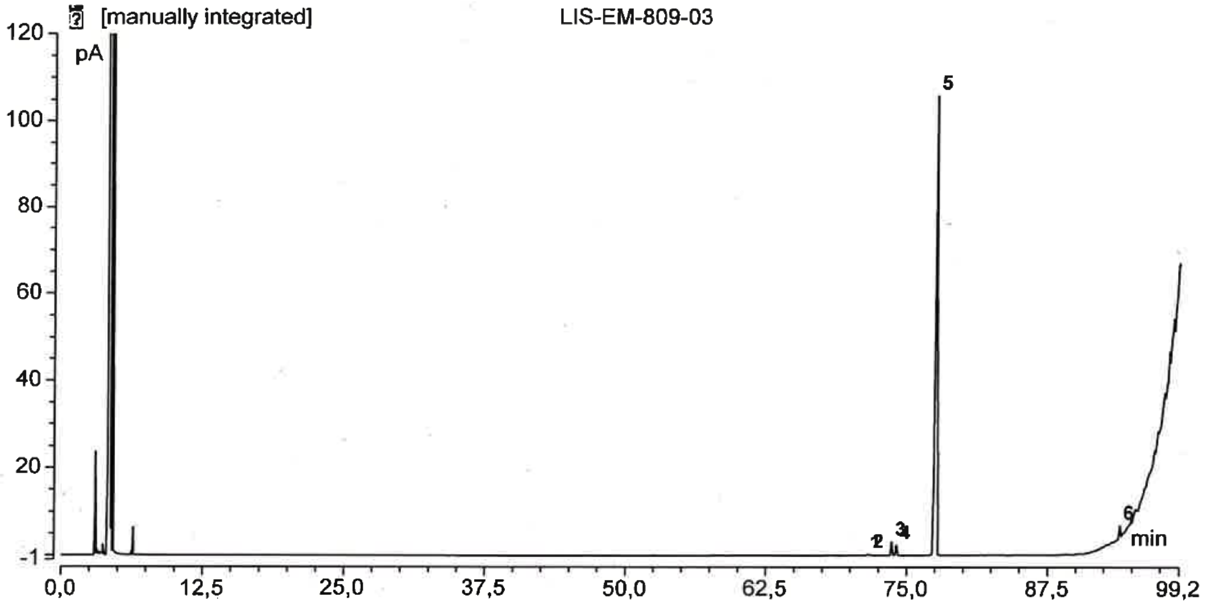

**GC (achiral)** (Stabilwax 0.25/0.25df, G/353 60.0 m, temperature: 220/60 1/min 150 12/min 260/350, 1.10 bar H_2, sample size: 1.0 µL, split ratio: 20:1).				
**peak #**	***t*$_R$ / min**	**area / %**	**peak name**	**structure**
1	71.45	0.18	**(*S*)-5c**	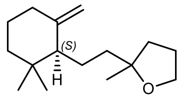 (*S*)-**5c**
2	71.70	0.20	**(*R*)-5c**	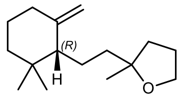 (*R*)-**5c**
3	73.67	1.92	**(*R*)-5a**	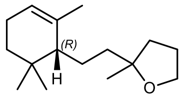 (*R*)-**5a**
4	74.08	1.48	**(*S*)-5a**	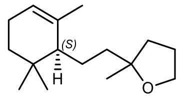 (*S*)-**5a**
5	77.59	95.42	**5b**	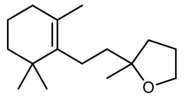 **5b**

GC chromatogram (chiral stationary phase) for a mixture of partially cyclized side products **5a**, **5b**, and **5c** obtained with a 1:1 mixture of (*S*,*S*)- and (*R*,*R*)-IDPi **8g** from (±)-(*E*)-γ-cyclohomofarnesol (**3c**). The sample serves as reference for the separation of regioisomers, diastereomers, and enantiomers. The enantiomeric ratio of one diastereomer of **5a** and of **5b** can be determined using the conditions provided in the table below. The absolute configuration at the cyclohexene was tentatively assigned based on reactions with enantioenriched **3a** and **3c** (with known absolute configurations). The absolute configuration at the tetrahydrofuran could not be determined.

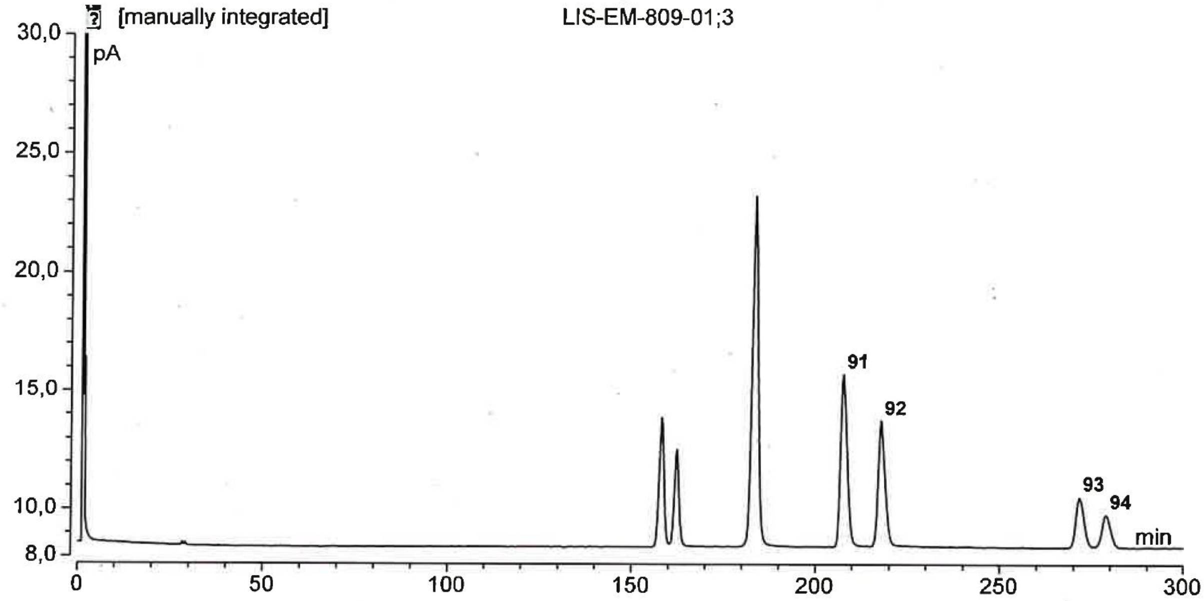

GC (chiral)				
**GC (chiral)** (Ivadex-1/PS086 G/662 24.5 m, temperature: 220/90 iso/350, 0.50 bar H_2, sample size: 1.0 µL, split ratio: 10:1).				
**peak #**	**t_R / min**	**area / %**	**peak name**	**structure**
91	207.29	42.57	**(*R*)-5a**	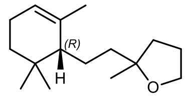 (*R*)-**5a**
92	217.51	32.34	*ent*-**(*R*)-5a**	
93	271.45	14.86	**5b**	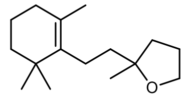 **5b**
94	278.78	10.22	*ent*-**5b**	

GC chromatogram (chiral stationary phase) for a mixture of partially cyclized side products **5a**, **5b**, and **5c** obtained with a 1:1 mixture of (*S*,*S*)- and (*R*,*R*)-IDPi **8g** from (±)-(*E*)-γ-cyclohomofarnesol (**3c**). The sample serves as reference for the separation of regioisomers, diastereomers, and enantiomers. The enantiomeric ratio of both diastereomers of **5c** and one diastereomer of **5a** can be determined using the conditions provided in the table below. The absolute configuration at the cyclohexene was tentatively assigned based on reactions with enantioenriched **3a** and **3c** (with known absolute configurations). The absolute configuration at the tetrahydrofuran could not be determined.

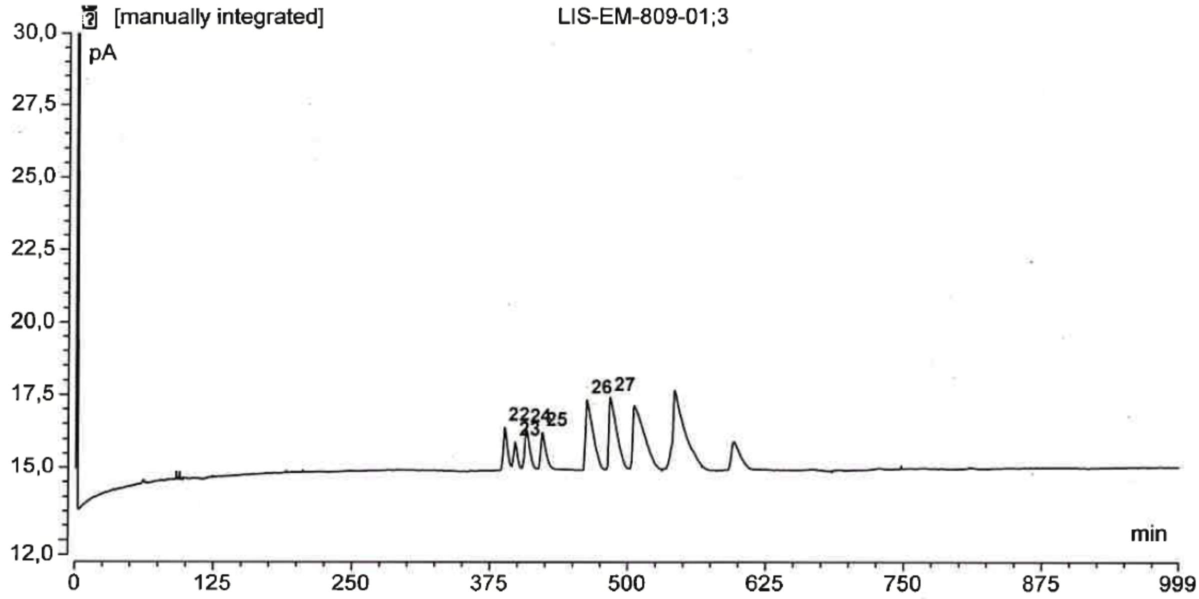

GC (chiral) (Cyclodextrin-H 0.25/0.125df, G/632 24.0 m, temperature: 220/60 iso/350, 0.50 bar H_2, sample size: 1.0 µL, split ratio: 10:1).

peak #	t_R / min	area / %	peak name	structure
22	387.82	10.25	**(*R*)-5c**	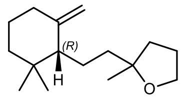 (*R*)-**5c**
23	397.48	6.48	*ent*-**(*R*)-5c**	
24	407.40	12.27	**(*S*)-5c**	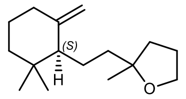 (*S*)-**5c**
25	422.00	11.17	*ent*-**(*S*)-5c**	
26	462.53	29.17	**(*R*)-5a**	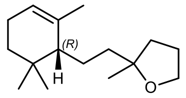 (*R*)-**5a**
27	483.44	30.66	**(*S*)-5a**	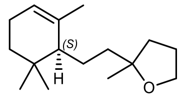 (*S*)-**5a**

GC chromatogram (achiral stationary phase) for partially cyclized products **5** obtained in the scale-up experiment at −40 °C.

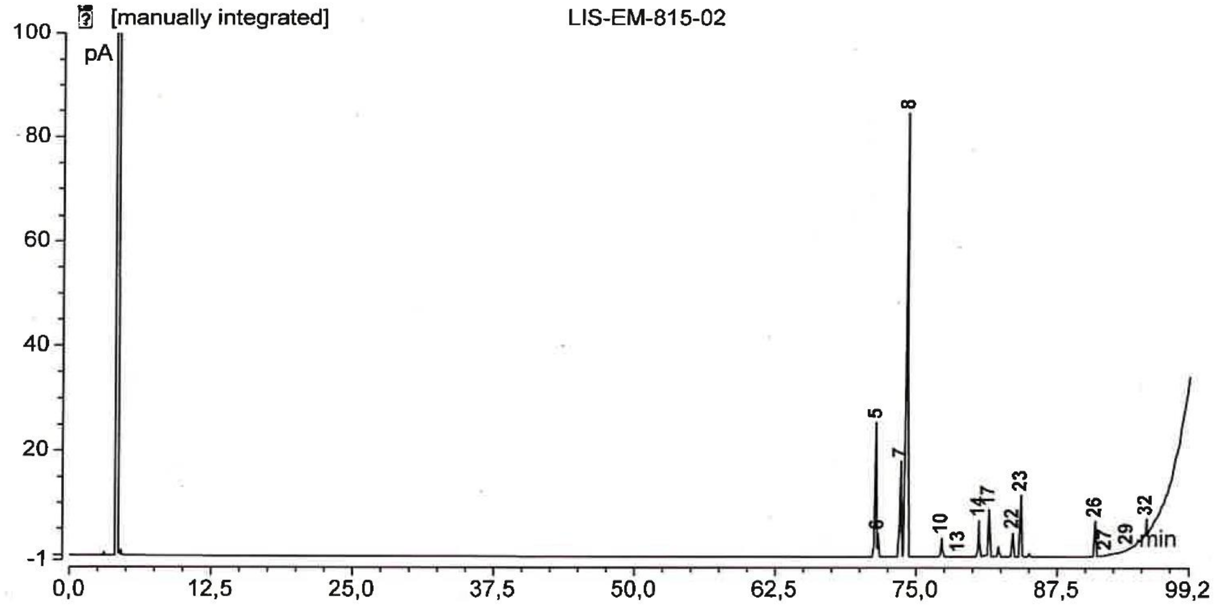

**GC (achiral)** (Stabilwax 0.25/0.25df, G/353 60.0 m, temperature: 220/60 1/min 150 12/min 260/350, 1.10 bar H_2, sample size: 0.2 µL, split ratio: 60:1).				
**peak #**	***t*$_R$ / min**	**area / %**	**peak name**	**structure**
5	71.39	12.61	**(*S*)-5c**	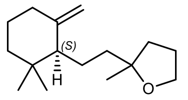 (*S*)-**5c**
6	71.60	1.95	**(*R*)-5c**	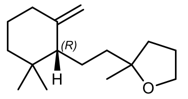 (*R*)-**5c**
7	73.59	9.55	**(*R*)-5a**	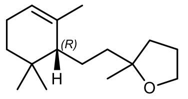 (*R*)-**5a**
8	74.14	51.76	**(*S*)-5a**	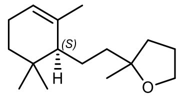 (*S*)-**5a**
10	77.24	1.67	**5b**	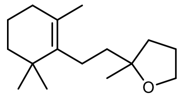 **5b**
17	81.44	4.55	**10**	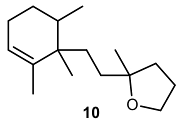 **10**

GC conditions (chiral stationary phase) for determining the e.r. of partially cyclized product **5a** (one diastereomer) and **5b**.

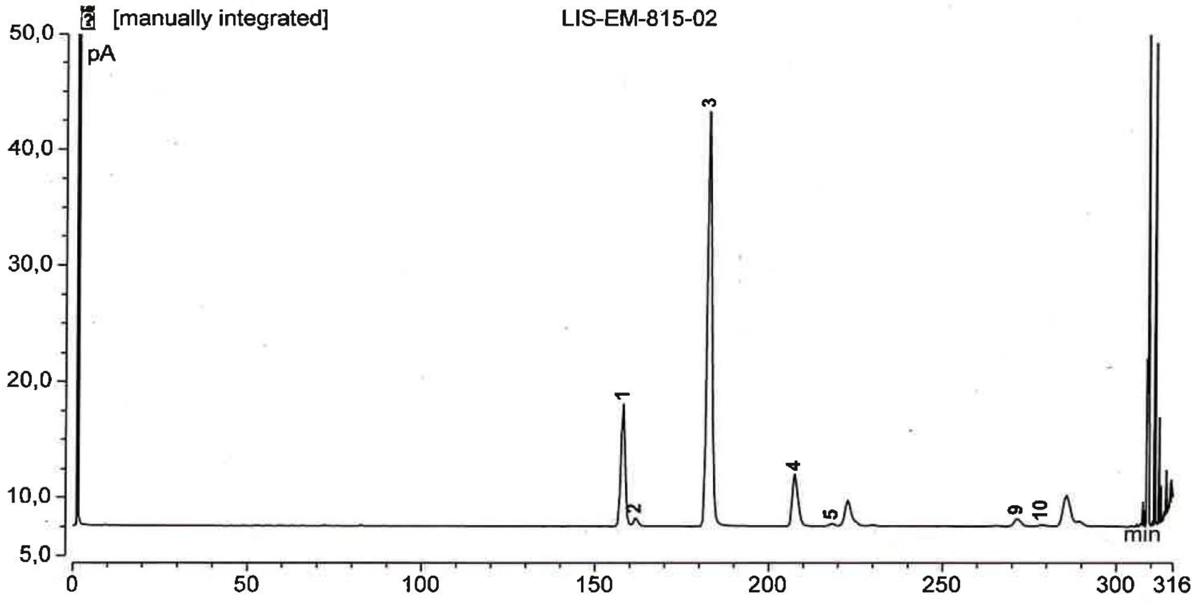

GC (chiral) (Ivadex-1/PS086 G/662 24.5 m, temperature: 220/90 300 min iso 8/min 220/350, 0.50 bar H_2, sample size: 1.0 µL, split ratio: 40:1).

peak #	t_R / min	area / %	peak name	structure
3	207.34	10.10	**(*R*)-5a**	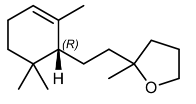 (*R*)-**5a**
4	218.16	0.50	*ent*-**(*R*)-5a**	
9	271.39	1.85	**5b**	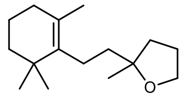 **5b**
10	278.43	0.44	*ent*-**5b**	

GC conditions (chiral stationary phase) for determining the e.r. of both diastereomers of partially cyclized product **5a**.

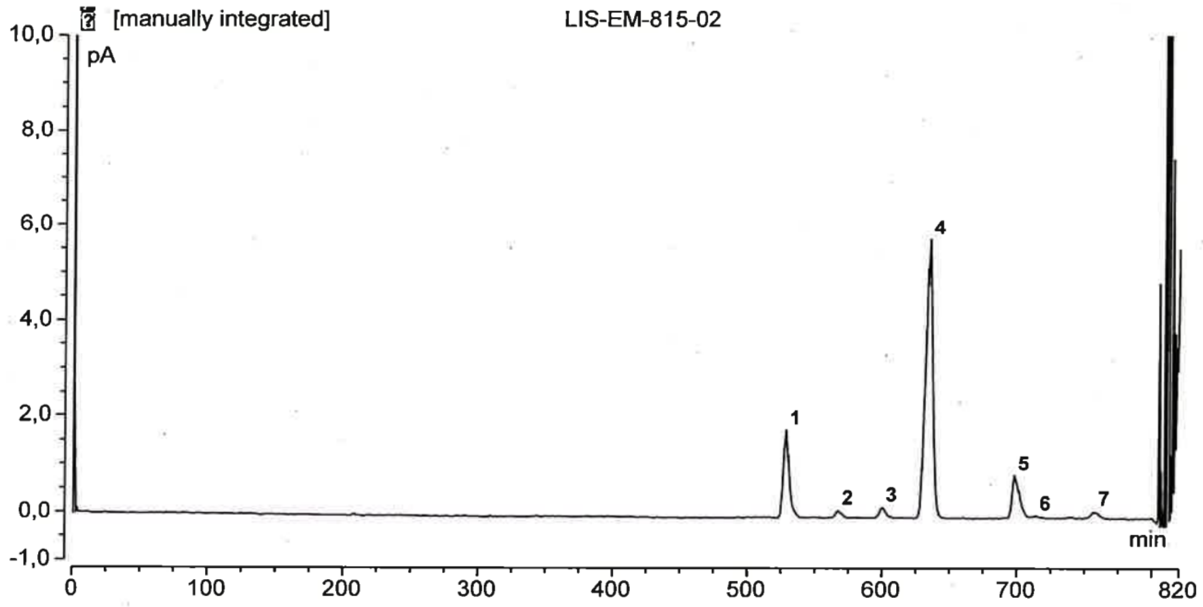

**GC (chiral)** (Lipodex-G 0.25/?df G/602, 25.0 m, temperature: 220/60 800 min iso 8/min 220/350, 0.50 bar H_2, sample size: 1.0 µL, split ratio: 60:1).				
**peak #**	***t*R / min**	**area / %**	**peak name**	**structure**
1	528.57	17.19	**5c**	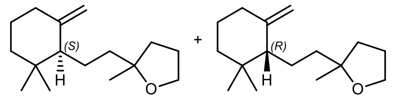 (*S*)-**5c** (*R*)-**5c**
2	567.53	1.54	**5c**	
3	600.03	2.38	**5a**	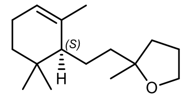 (*S*)-**5a**
4	634.73	66.19	**5a**	
5	697.97	10.11	**5a**	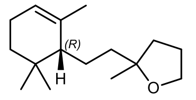 (*R*)-**5a**
6	714.02	0.68	**5a**	
7	757.70	1.91	**5b**	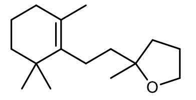 **5b**

GC (chiral stationary phase) conditions for determining the e.r. of both diastereomers of partially cyclized product **5c**.

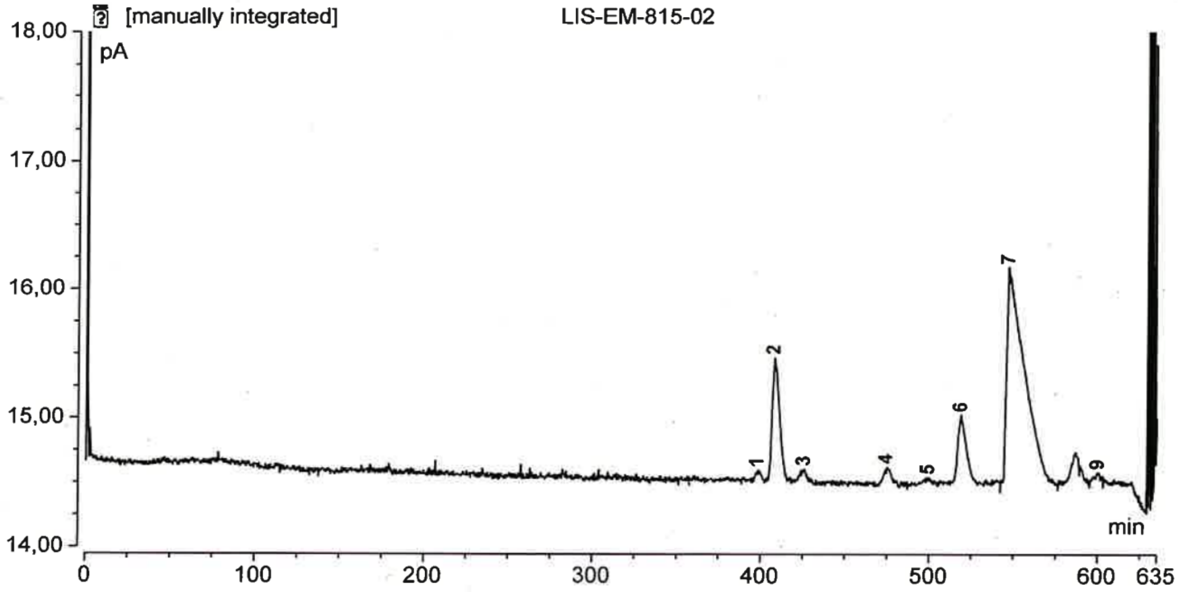

GC (chiral) (Cyclodextrin-H 0.25/0.125df, G/632 24.0 m, temperature: 220/60 iso/350, 0.50 bar H_2, sample size: 1.0 µL, split ratio: 80:1).

peak #	t_R / min	area / %	peak name	structure
–	387.82	n.d.	**(*R*)-5c**	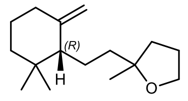
1	398.24	1.14	*ent*-**(*R*)-5c**	(*R*)-**5c**
2	407.98	16.26	**(*S*)-5c**	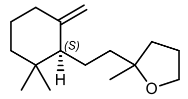
3	425.85	2.15	*ent*-**(*S*)-5c**	(*S*)-**5c**
5	499.47	0.74	**(*R*)-5a**	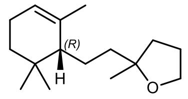
6	518.80	9.90	*ent*-**(*R*)-5a**	(*R*)-**5a**

GC chromatogram (achiral stationary phase) for partially cyclized products **5** and isomerized product **10** obtained in the scale-up experiment at −40 °C → 0 °C.

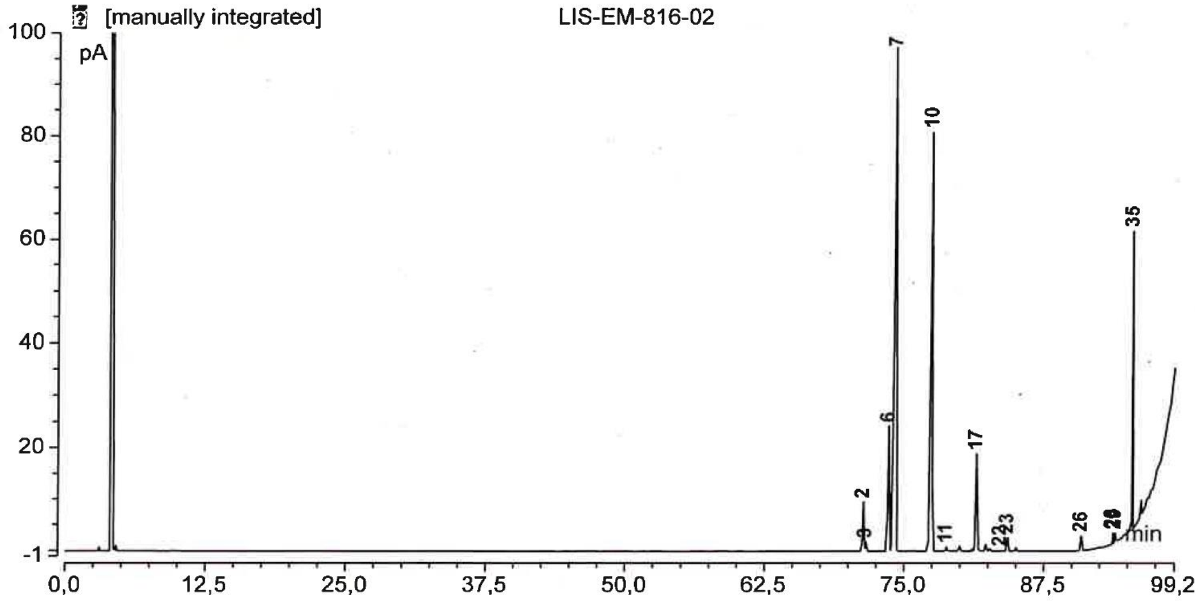

GC (achiral) (Stabilwax 0.25/0.25df, G/353 60.0 m, temperature: 220/60 1/min 150 12/min 260/350, 1.10 bar H_2, sample size: 0.2 µL, split ratio: 60:1).				
**peak #**	***t*R / min**	**area / %**	**peak name**	**structure**
2	71.36	2.90	**(*S*)-5c**	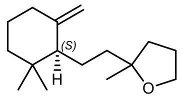 (*S*)-**5c**
3	71.59	0.54	**(*R*)-5c**	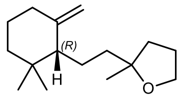 (*R*)-**5c**
6	73.61	8.35	**(*R*)-5a**	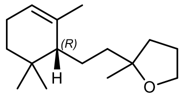 (*R*)-**5a**
7	74.17	39.33	**(*S*)-5a**	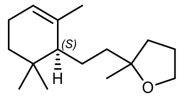 (*S*)-**5a**
10	77.42	31.14	**5b**	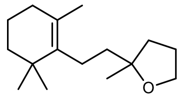 **5b**
17	81.46	6.26	**10**	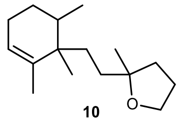 **10**

GC conditions (chiral stationary phase) for determining the e.r. of partially cyclized product **5a** (one diastereomer) and **5b**.

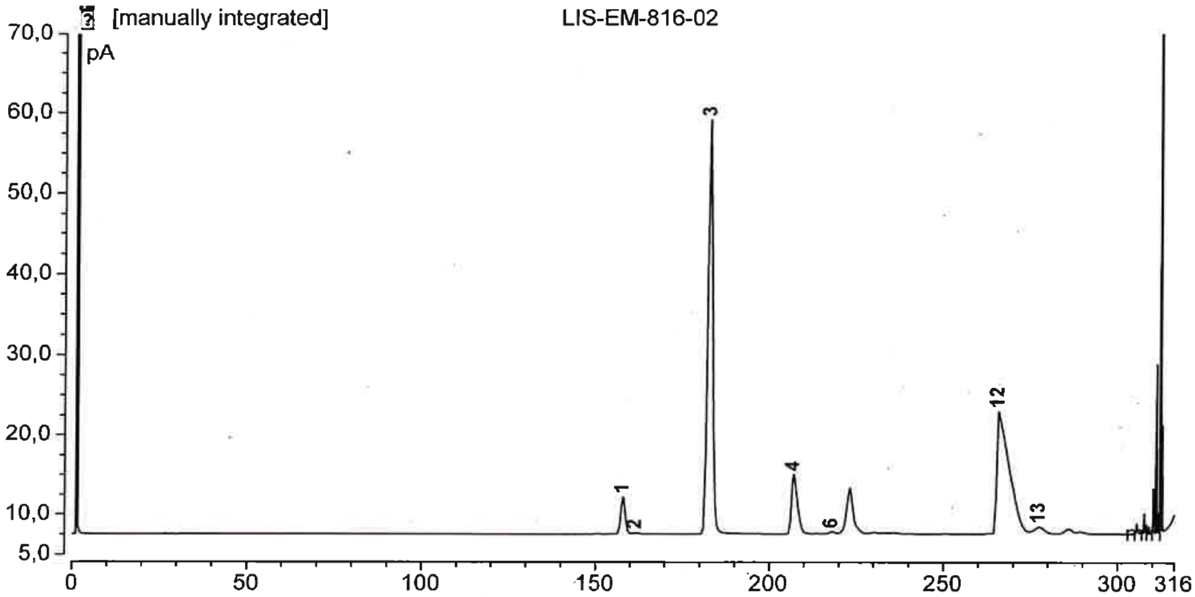

GC (chiral) (Ivadex-1/PS086 G/662 24.5 m, temperature: 220/90 300 min iso 8/min 220/350, 0.50 bar H_2, sample size: 1.0 µL, split ratio: 40:1).

peak #	t_R / min	area / %	peak name	structure
4	206.85	8.61	**(*R*)-5a**	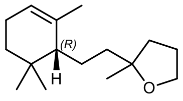 (*R*)-5a
6	217.91	0.36	*ent*-**(*R*)-5a**	
12	265.64	36.45	**5b+10**	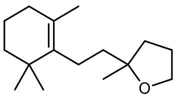 5b + 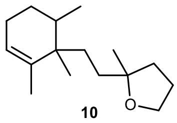 10
13	277.42	1.95		

GC conditions (chiral stationary phase) for determining the e.r. of both diastereomers of partially cyclized product **5a**.

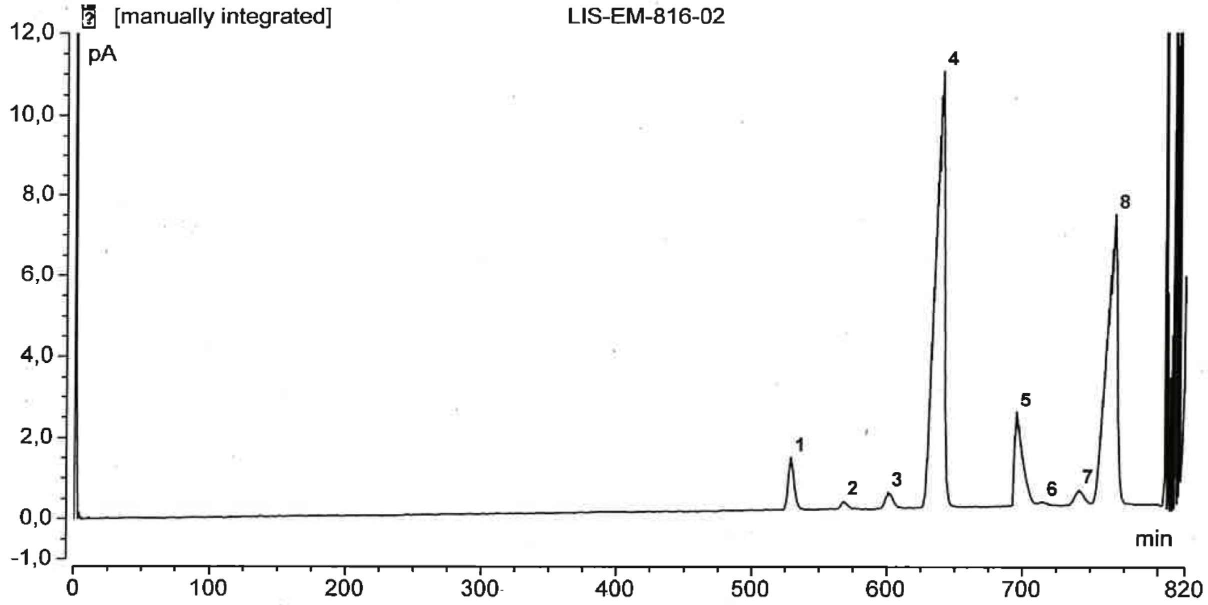

GC (chiral) (Lipodex-G 0.25/?df G/602, 25.0 m, temperature: 220/60 800 min iso 8/min 220/350, 0.50 bar H_2, sample size: 1.0 µL, split ratio: 40:1).

peak #	t_R / min	area / %	peak name	structure
1	528.80	3.61	**5c**	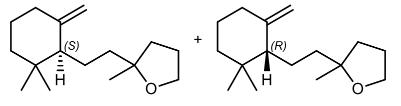 (*S*)-**5c** (*R*)-**5c**
2	567.71	0.55	**5c**	
3	600.56	1.41	**5a**	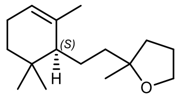 (*S*)-**5a**
4	634.73	47.35	**5a**	
5	695.04	8.76	**5a**	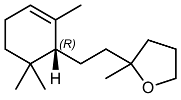 (*R*)-**5a**
6	714.85	0.36	**5a**	
7	741.14	1.71	**5b + 10**	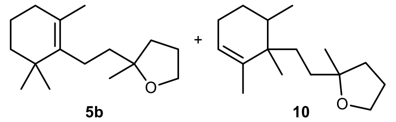 **5b** **10**
8	757.70	36.26		

GC (chiral stationary phase) conditions for determining the e.r. of both diastereomers of partially cyclized product **5c**.

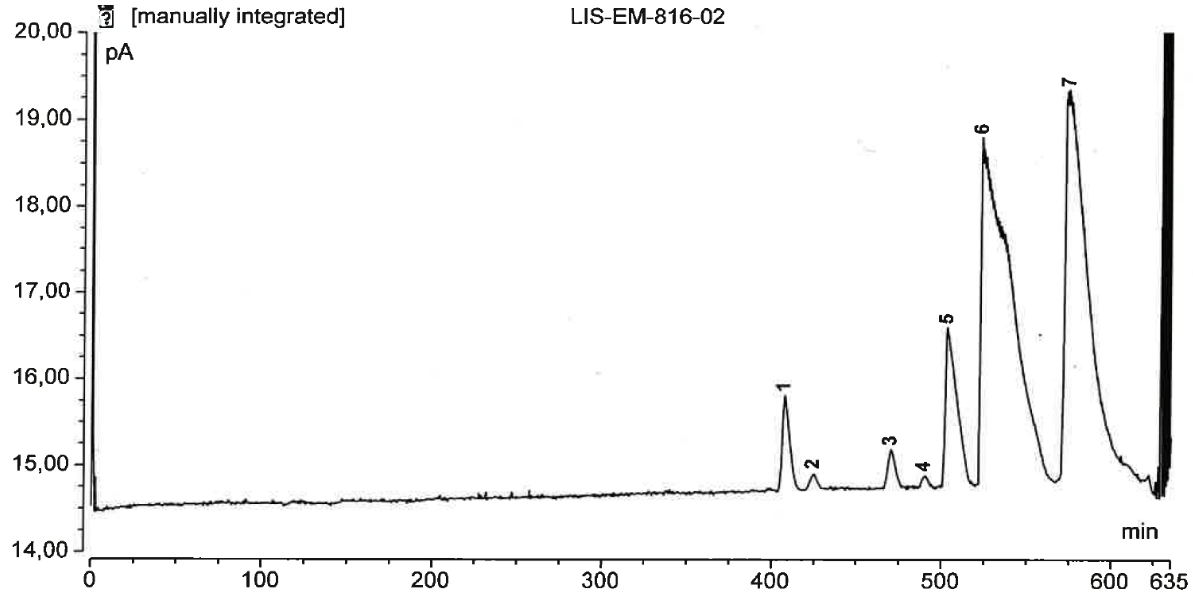

GC (chiral) (Cyclodextrin-H 0.25/0.125df, G/632 24.0 m, temperature: 220/60 iso/350, 0.50 bar H_2, sample size: 1.0 µL, split ratio: 50:1).

peak #	t_R / min	area / %	peak name	structure
1	407.49	3.20	**(*S*)-5c**	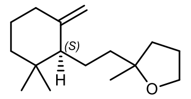 (*S*)-**5c**
2	424.62	0.60	*ent*-**(*S*)-5c**	
4	489.90	0.38	**(*R*)-5a**	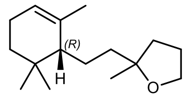 (*R*)-**5a**
5	502.88	8.18	*ent*-**(*R*)-5a**	

GC (achiral stationary phase) conditions for determining the d.r. of sclareolide (**12a**), 9-*epi*-sclareolide (**12b**), 5β,8α,9β-sclareolide (**12c**), and 8-*epi*-sclareolide (**12f**).

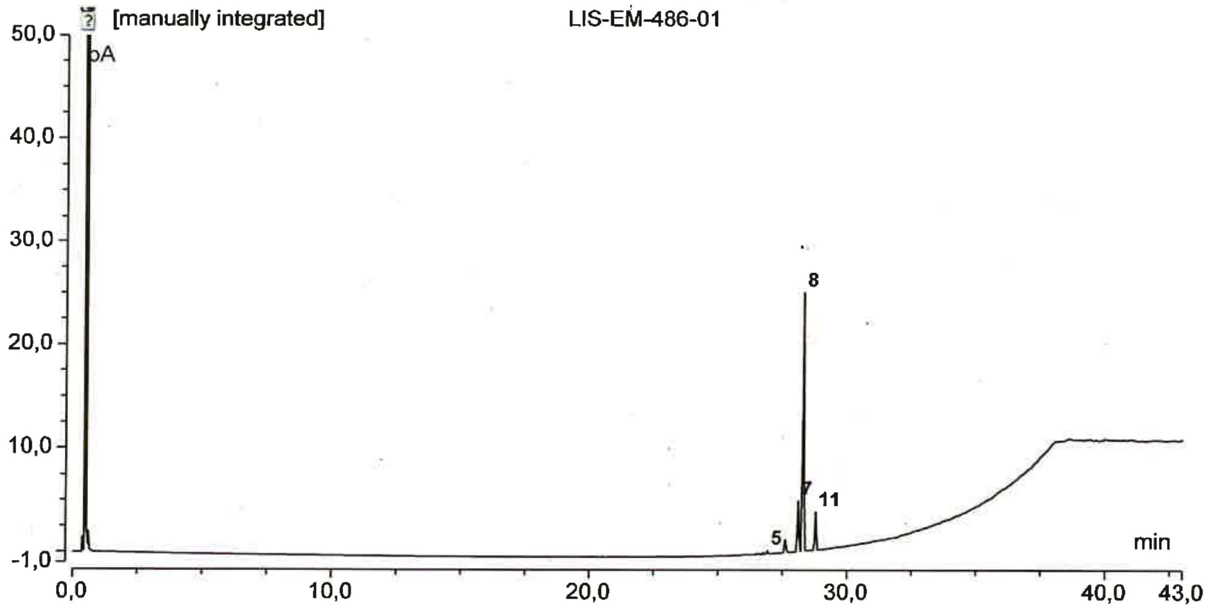

**GC (achiral)** (FFAP 0.25/0.25df, G/396 15.0 m, temperature: 220/60 5/min 250 5 min iso/350, 0.50 bar H_2, sample size: 0.2 µL, split ratio: 60:1).				
**peak #**	**t_R / min**	**area / %**	**peak name**	**structure**
5	26.92	0.88	8-*epi*-sclareolide (**12f**)	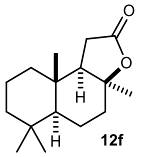 12f
7	28.10	13.82	9-*epi*-sclareolide (**12b**)	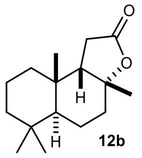 12b
8	28.29	68.74	sclareolide (**12a**)	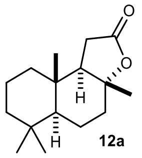 12a
11	28.78	10.45	5β,8α,9β-sclareolide (**12c**)	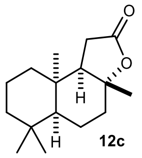 12c

GC chromatogram (achiral stationary phase) of a racemic sample containing 9-*epi*-sclareolide (**12b**) and 5β,8α-sclareolide (**12d**).

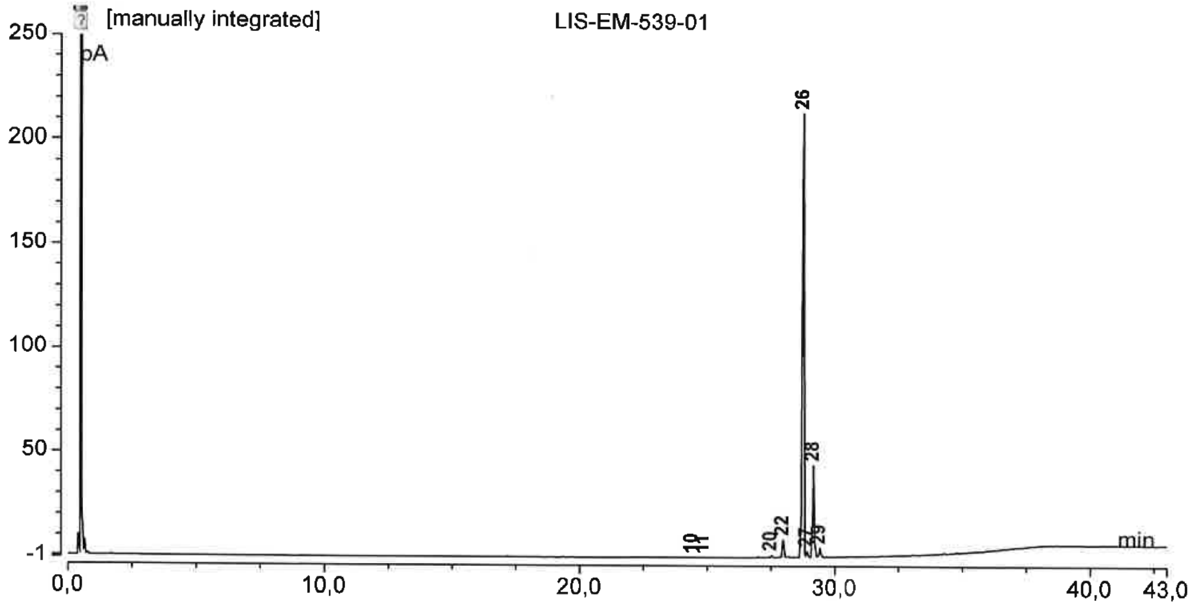

**GC (achiral)** (FFAP 0.25/0.25df, G/396 15.0 m, temperature: 220/60 5/min 250 5 min iso/350, 0.50 bar H_2, sample size: 0.2 µL, split ratio: 60:1).				
**peak #**	**t_R / min**	**area / %**	**peak name**	**structure**
20	27.50	0.50	8-*epi*-sclareolide (**12f**)	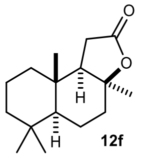 12f
26	28.77	77.35	9-*epi*-sclareolide (**12b**)	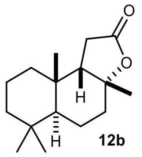 12b
27	28.91	1.05	sclareolide (**12a**)	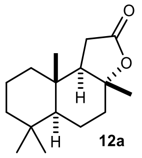 12a
28	29.16	14.49	5β,8α-sclareolide (**12d**)	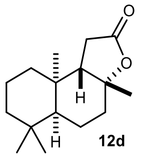 12d
29	29.41	1.55	5β,8α,9β-sclareolide (**12c**)	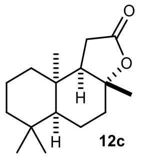 12c

GC chromatogram (chiral stationary phase) of a racemic sample containing 9-*epi*-sclareolide (**12b**) and 5β,8α-sclareolide (**12d**).

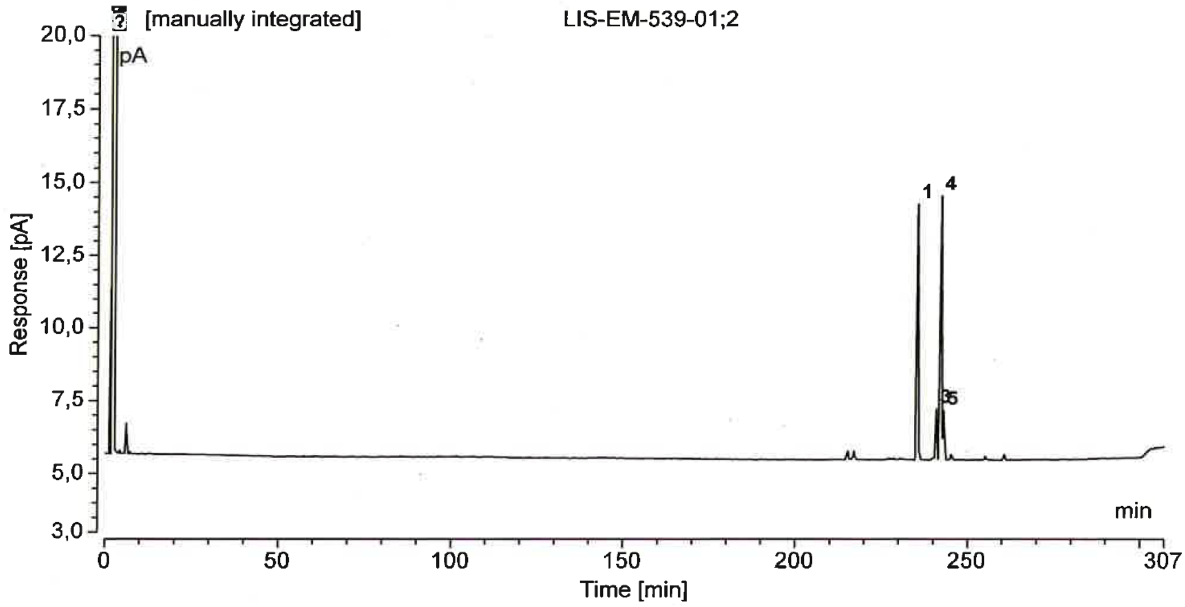

**GC (chiral)** (Hydrodex-β-TBDAc-CD 0.25/?df, G/681 25.0 m, temperature: 220/80 0.4/min 200 10/min 220 5 min iso/350, 0.60 bar H_2, sample size: 0.2 µL).				
**peak #**	***t*R / min**	**area / %**	**peak name**	**structure**
1	235.27	41.86	(+)-9-*epi*-sclareolide (*ent*- **12b**)	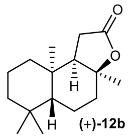
2	240.76	8.04	5β,8α-sclareolide (**12d**)	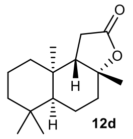
3	242.05	42.24	(−)-9-*epi*-sclareolide (**12b**)	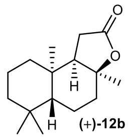
4	242.90	7.85	5β,8α-sclareolide (*ent*-**12d**)	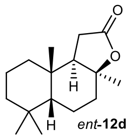

GC (chiral stationary phase) conditions for determining the e.r of sclareolide (**12a**), 9-*epi*-sclareolide (**12b**), 5β,8α,9β-sclareolide (**12c**), and 8-*epi*-sclareolide (**12f**).

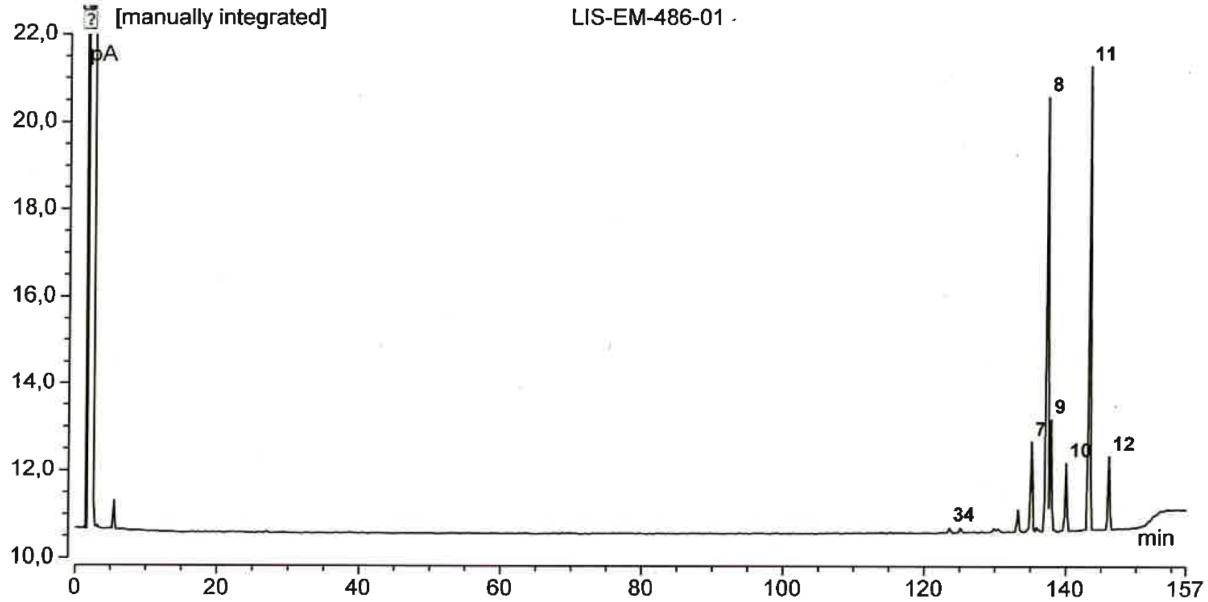

**GC (chiral)** (Hydrodex-β-TBDAc-CD 0.25/?df, G/681 25.0 m, temperature: 220/80 0.8/min 200 10/min 220 5 min iso/350, 0.60 bar H_2, sample size: 1.0 µL, split ratio: 40:1).				
**peak #**	***t*R / min**	**area / %**	**peak name**	**structure**
3	123.54	0.48	8-*epi*-sclareolide (**12f**)	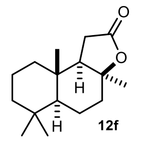
4	125.07	0.41		
7	135.10	7.48	(+)-9-*epi*-sclareolide (*ent*-**12b**)	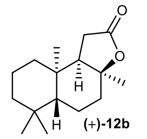
8	137.22	35.66	(+)-sclareolide (**12a**)	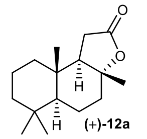
9	137.80	9.24	(−)-9-*epi*-sclareolide (**12b**)	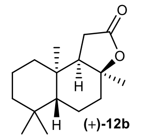
11	143.20	35.38	(−)-sclareolide (*ent*-**12a**)	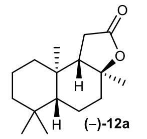
10	139.95	5.77	5β,8α,9β-sclareolide (**12c**)	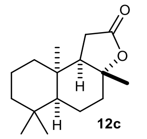
12	146.04	5.58		

GC chromatogram (achiral stationary phase) of synthetic **12a** preapred at −40 °C → 0 °C.

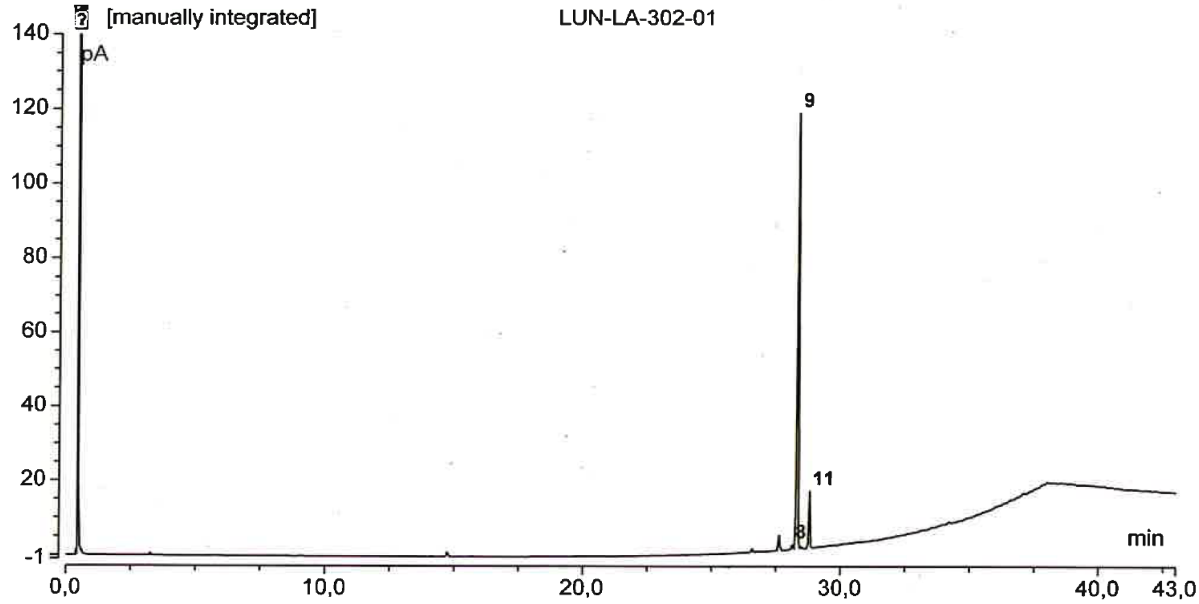

**GC (achiral)** (FFAP 0.25/0.25df, G/396 15.0 m, temperature: 220/60 5/min 250 5 min iso/350, 0.50 bar H_2, sample size: 0.2 µL).				
**peak #**	***t*$_R$ / min**	**area / %**	**peak name**	**structure**
8	28.12	0.95	9-*epi*-sclareolide (**12b**)	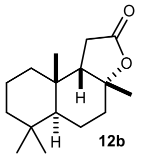 12b
9	28.33	83.13	sclareolide (**12a**)	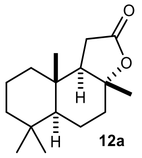 12a
11	28.79	10.24	5β,8α,9β-sclareolide (**12c**)	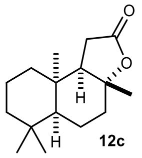 12c

GC chromatogram (chiral stationary phase) of synthetic **12a** preapred at −40 °C → 0 °C.

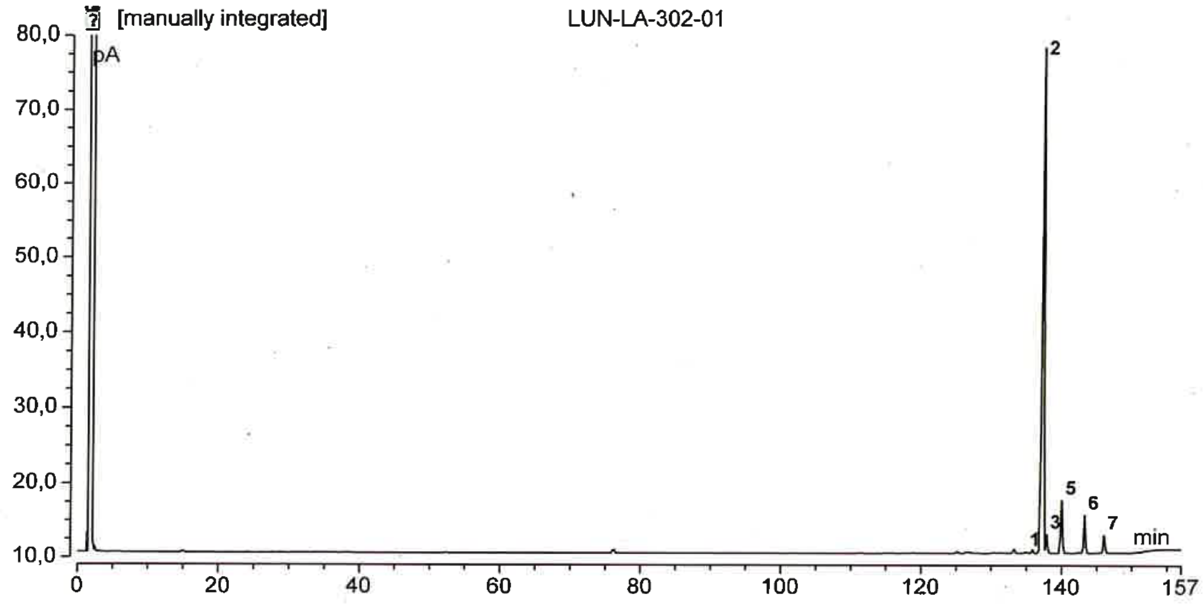

**GC (chiral)** (Hydrodex-β-TBDAc-CD 0.25/?df, G/681 25.0 m, temperature: 220/80 0.8/min 200 10/min 220 5 min iso/350, 0.60 bar H_2, sample size: 1.0 µL, split ratio: 20:1).				
**peak #**	t_R **/ min**	**area / %**	**peak name**	**structure**
7	134.96	0.26	(+)-9-*epi*-sclareolide (*ent-* **12b**)	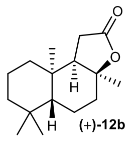
2	137.21	81.14	(+)-sclareolide (**12a**)	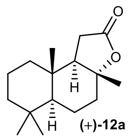
3	137.83	2.80	(−)-9-*epi*-sclareolide (**12b**)	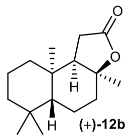
6	143.20	5.30	(−)-sclareolide (*ent*-**12a**)	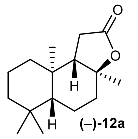
5	139.94	5.30	5β,8α,9β-sclareolide (**12c**)	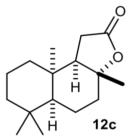
7	146.04	2.50		

5 Copies of HPLC Traces

Stacked HPLC chromatograms (achiral stationary phase) of synthetic (±)-(*E*)-γ-cyclohomofarnesol (**3c**, blue), (±)-(*E*)-α-cyclohomofarnesol (**3a**, red), and a mixture of **3a** and **3c** (green).

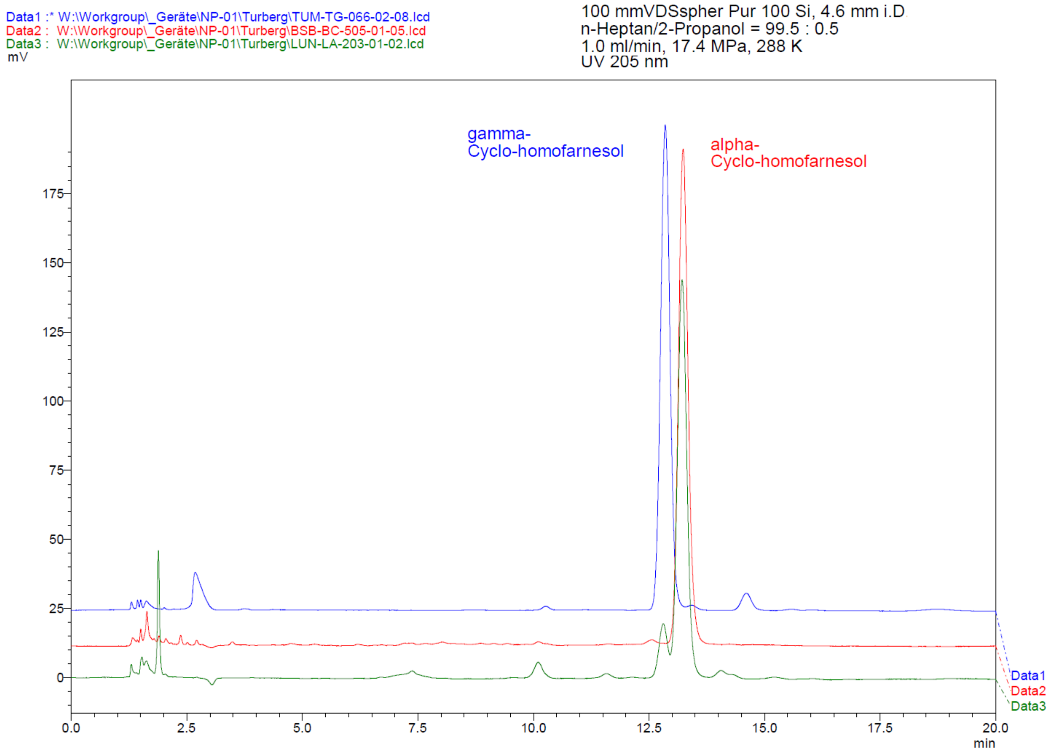

Stacked HPLC chromatograms (chiral stationary phase) of synthetic (±)-(*E*)-γ-cyclohomofarnesol (**3c**, blue) and (±)-(*E*)-α-cyclohomofarnesol (**3a**, red), indicating overlap of peaks thus necessitating a separation of both isomers on an achiral stationary phase prior to measuring the enantiomeric ratio.

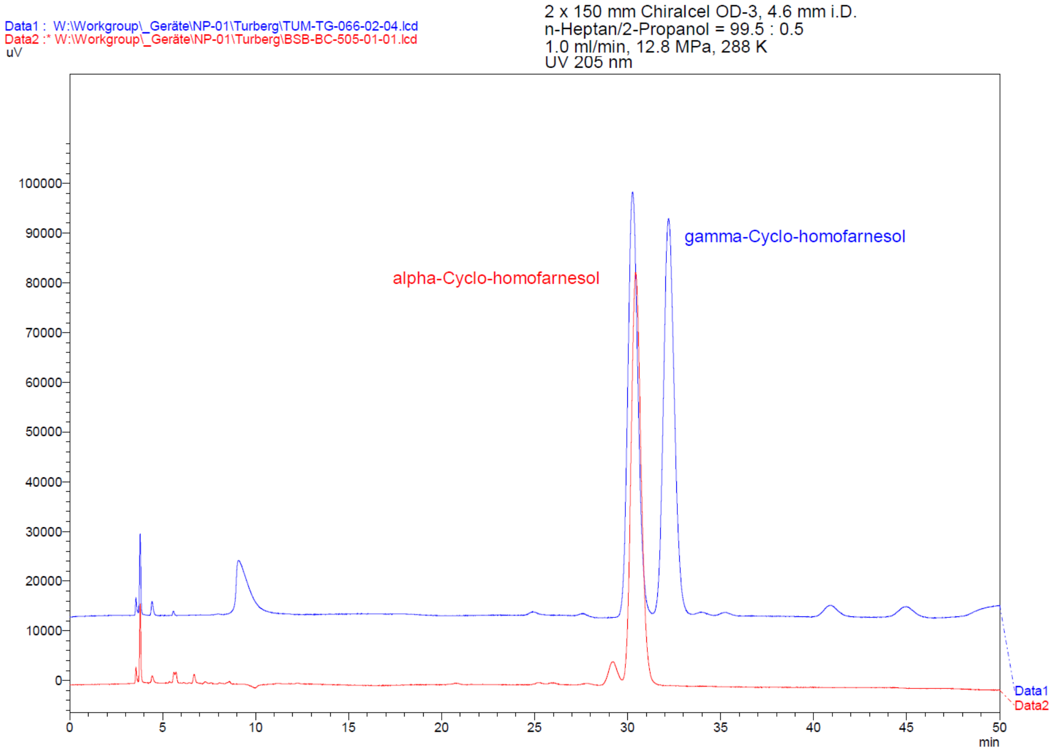

2D-HPLC separation of (±)-(*E*)-γ-cyclohomofarnesol (**3c**).

¹D chromatogram(s)

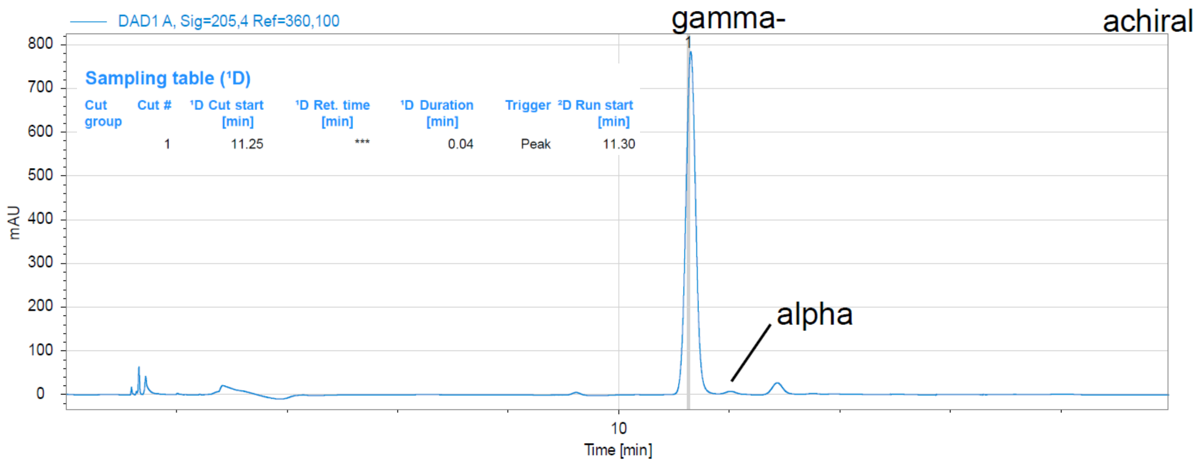

Cut# : 1

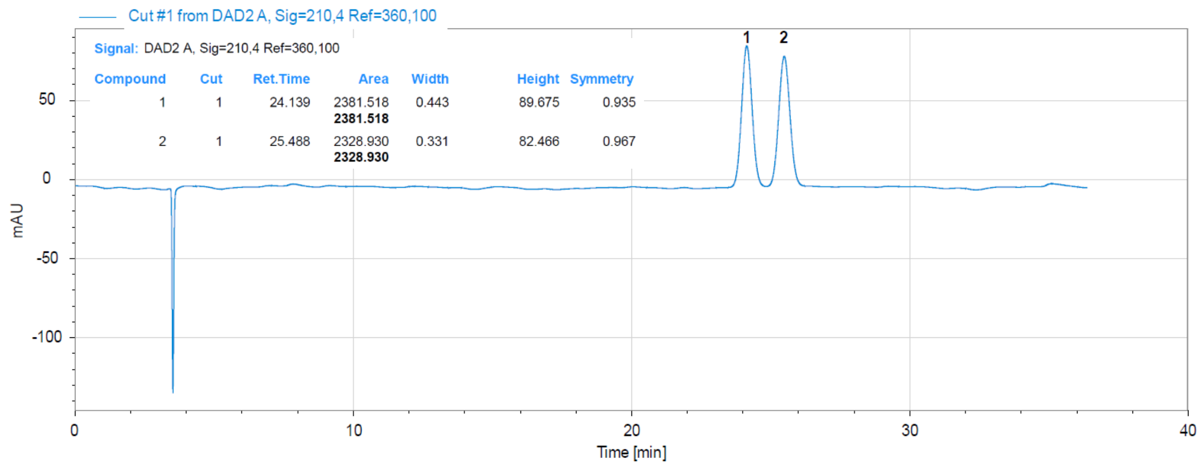

Component table

Signal: DAD2 A, Sig=210,4 Ref=360,100

chiral

Component	¹D Sampling range [min]	Ret.Time ²D [min]	Area	Area%
1	11.25 - 11.29	24.139	2381.518	50.558
2	11.25 - 11.29	25.488	2328.930	49.442

¹D (1st dimension, achiral stationary phase): 100 mm VDSpher PUR 100 SIL, 4.6 mm i.D., *n*-heptane/propan-2-ol 95.5:0.5 *v/v*, 1.0 mL/min, 20.9 MPa, 288 K, UV: λ = 205 nm): t_R = 11.25–11.29 (sampling range).
²D (2nd dimension, chiral stationary phase, 2 × 150 mm Chiralcel OD-3, 4.6 mm i.D., *n*-heptane/propan-2-ol 95.5:0.5 *v/v*, 1.0 mL/min, 14.3 MPa, 288 K, UV: λ = 220 nm).

peak #	t_R (²D) / min	area / %	peak name
1	24.139	50.558	(*R*)-(*E*)-γ-cyclohomofarnesol (*ent*-**3a**)
2	25.488	49.442	(*S*)-(*E*)-γ-cyclohomofarnesol (**3a**)

HPLC chromatogram (chiral stationary phase) of (±)-(*E*)-γ-cyclohomofarnesol (**3c**).

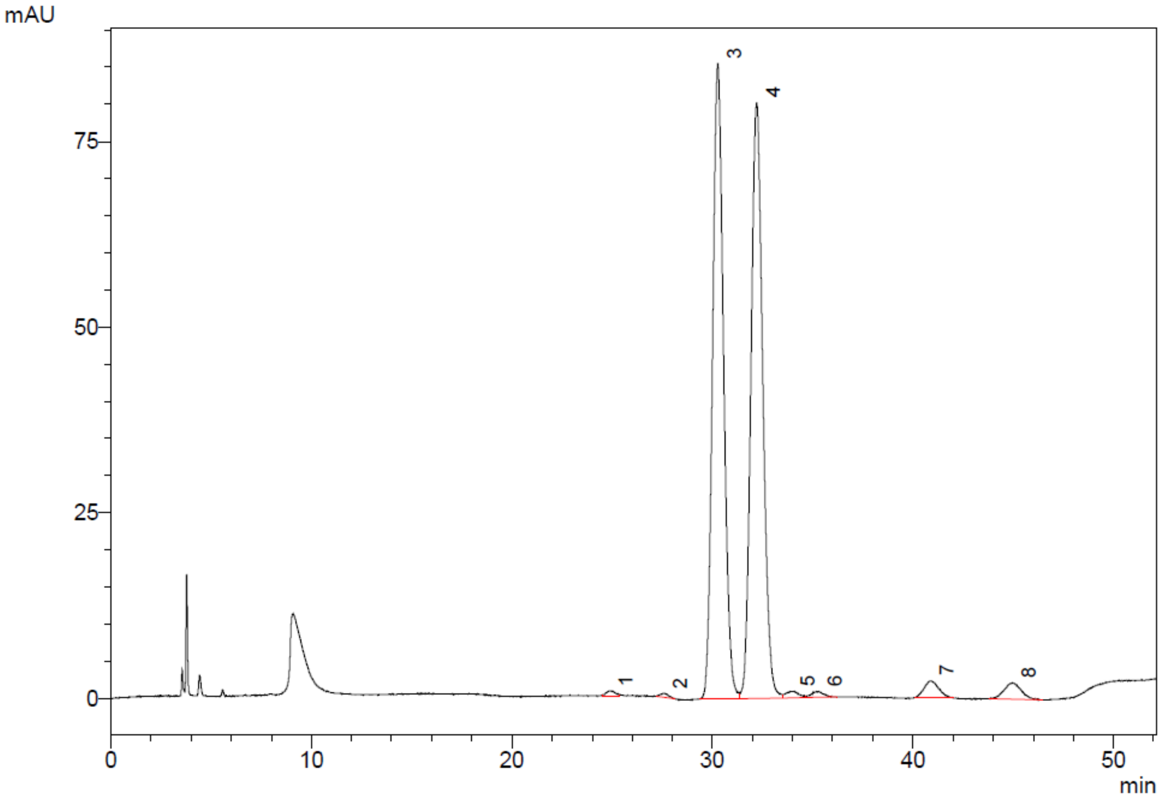

**HPLC** (chiral stationary phase) (2 × 150 mm Chiralcel OD-3, 4.6 mm i.D., *n*-heptane/propan-2-ol 95.5:0.5 *v/v*, 1.0 mL/min, 14.3 MPa, 288 K, UV: λ = 220 nm).			
**peak #**	t_R **/ min**	**area / %**	**peak name**
3	30.27	47.24	(*R*)-(*E*)-γ-cyclohomofarnesol (*ent*-**3a**)
4	32.20	47.57	(*S*)-(*E*)-γ-cyclohomofarnesol (**3a**)

Preparative separation of the enantiomers of (*E*)-γ-cyclohomofarnesol (**3c**)

**Data file:**	W:\Workgroup_Geräte\NP-06 Scout\Kundendaten\Luo\LIS-EM-825-01-011.D
**Sample name:**	LIS-EM-825-01-
**Description:**	2 µL LIS-EM-825-01 (GS in 1ml n-Heptan)<br>davon 20 µL in 180 µL n-Heptan<br>150 mm Chiralcel OD-3, 4.6 mm i.D.<br>n-Heptan / 2-Propanol = 99.5 : 0.5<br>1.0 mL / min, 10.5 MPa, 288 K<br>UV, 205 nm

Instrument: NP-06 geclustert

Column Chiral Art Amylose-SA length 150.00 i.D. 4.60

Injection date: 1/19/2024 7:59:18 AM
Acq. method: Luo.M
Analysis method: Luo.M
Last changed: 1/22/2024 9:09:30 AM
(modified after loading)

Location: D1F-A1
Injection volume: 2.000
Acq. operator: SYSTEM

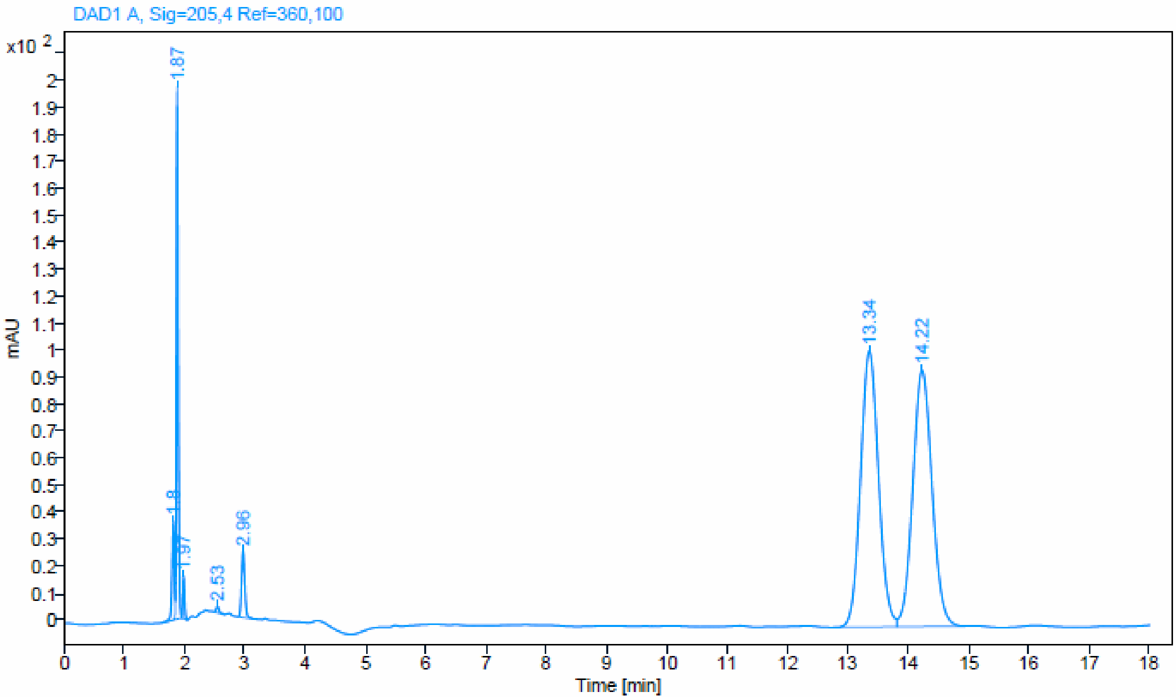

Signal: DAD1 A, Sig=205,4 Ref=360,100

RT [min]	Area%	Name
1.80	1.96	
1.87	8.09	
1.97	0.61	
2.53	0.16	
2.96	1.86	
13.34	43.70	
14.22	43.62	

Gerät : P-1 präp

Operator : Ke
Data Acquired: 1/18/2024 1:45:48 PM
Sample Name : LIS-EM-825-01
Vial # : -1
Injection Volume : 1000 uL
Data File Name : LIS-EM-825-01-OD-H-05.lcd
Method File Name : Luo.lcm

1000 µL LIS-EM-825-01, 2. Lauf 3.1 mg
15.8 mg in 1 ml i-Hexan, davon 200 µL in 800 µL i-Hexan
250 mm Chiralcel OD-H 5 µm, 20.0 mm i.D.
ODH05J-AX001
i-Hexan/2-Propanol = 99.5 : 0.5 (v/v)
15.0 mL/min, 5.8 MPa, 288 K
205 nm, analytische Zelle

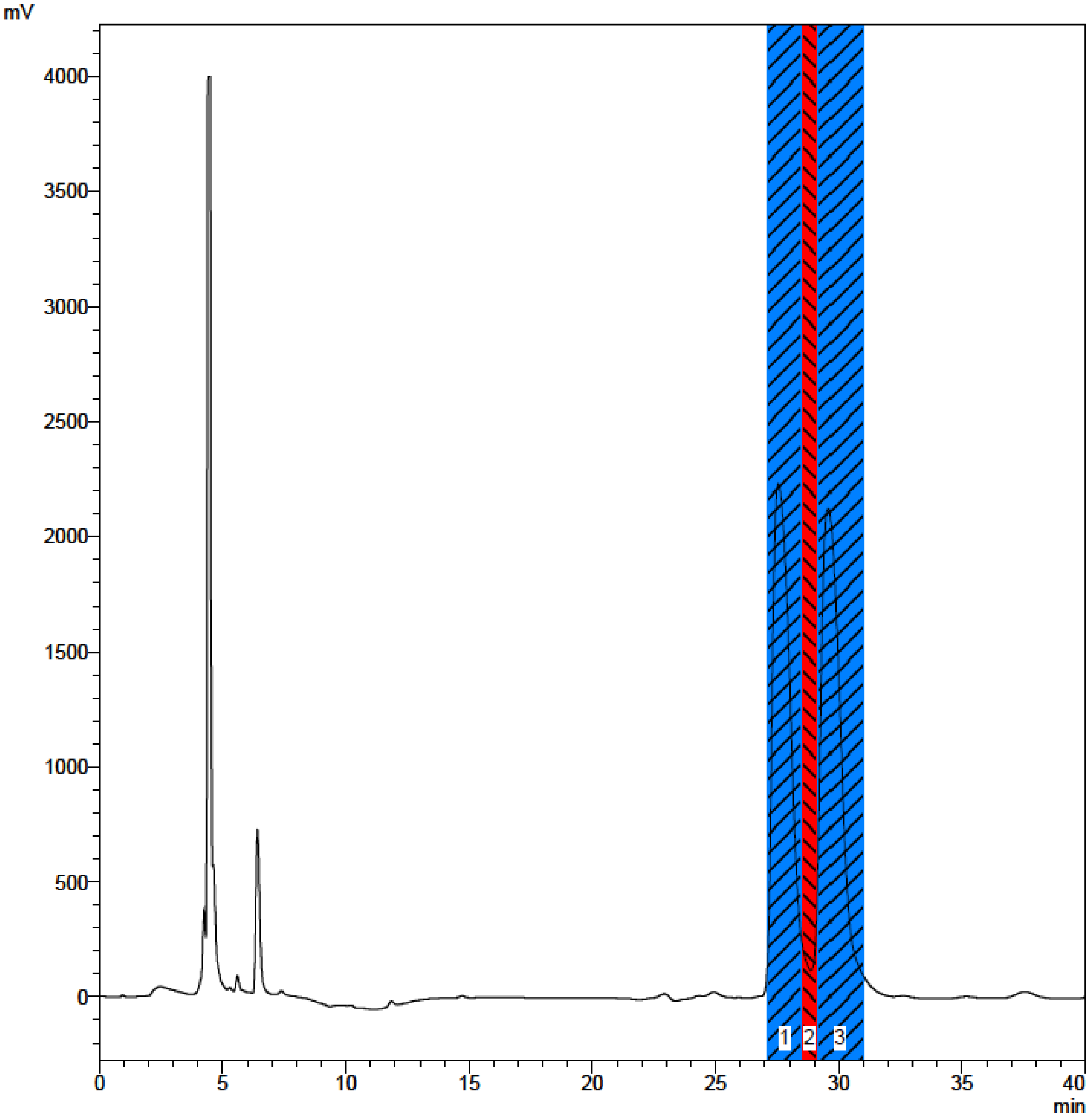

1 Detector A / 205nm

**Data file:**	W:\Workgroup_Geräte\NP-06 Scout\Kundendaten\Luo\LIS-EM-825-01-G10-003.D
**Sample name:**	LIS-EM-825-01-G10-
**Description:**	3 µL LIS-EM-825-01-10 gesamt während der Aufarbeitung<br>20 µL in 180 µL n-Heptan<br>150 mm Chiralcel OD-3, 4.6 mm i.D.<br>n-Heptan / 2-Propanol = 99.5 : 0.5<br>1.0 mL / min, 10.5 MPa, 288 K<br>UV, 205 nm

Instrument: NP-06 geclustert

Column Chiral Art Amylose-SA length 150.00 i.D. 4.60

Injection date: 1/19/2024 2:46:06 PM
Acq. method: Luo.M
Analysis method: Luo.M
Last changed: 1/22/2024 9:09:30 AM
(modified after loading)

Location: D1F-A3
Injection volume: 3.000
Acq. operator: SYSTEM

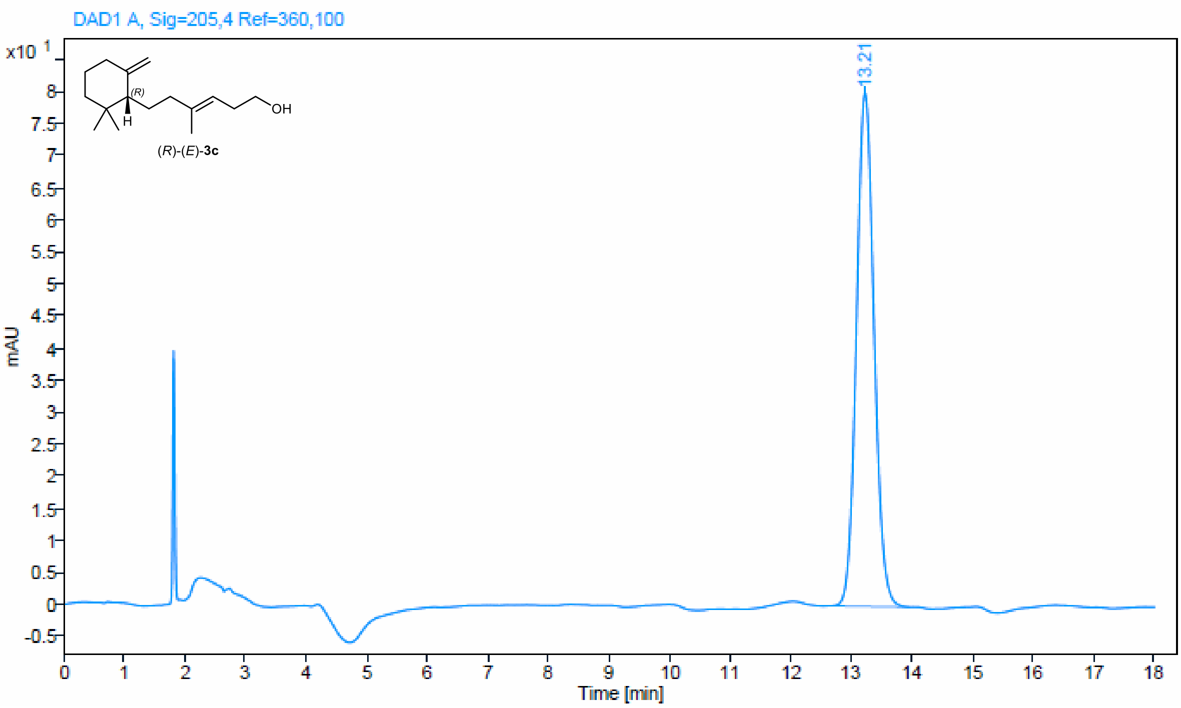

Signal: DAD1 A, Sig=205,4 Ref=360,100

RT [min]	Area%	Name
13.21	100.00	

Data file: W:\Workgroup_Geräte\NP-06 Scout\Kundendaten\Luo\LIS-EM-825-01-G30-002.D

Sample name: LIS-EM-825-01-G30-

Description: 3 µL LIS-EM-825-01-30 gesamt
20 µL in 180 µL n-Heptan
150 mm Chiralcel OD-3, 4.6 mm i.D.
n-Heptan / 2-Propanol = 99.5 : 0.5
1.0 mL / min, 10.5 MPa, 288 K
UV, 205 nm

Instrument: NP-06 geclustert

Column Chiral Art Amylose-SA length 150.00 i.D. 4.60

Injection date: 1/19/2024 10:14:56 AM

Acq. method: Luo.M

Analysis method: Luo.M

Last changed: 1/19/2024 2:11:53 PM (modified after loading)

Location: D1F-E2

Injection volume: 3.000

Acq. operator: SYSTEM

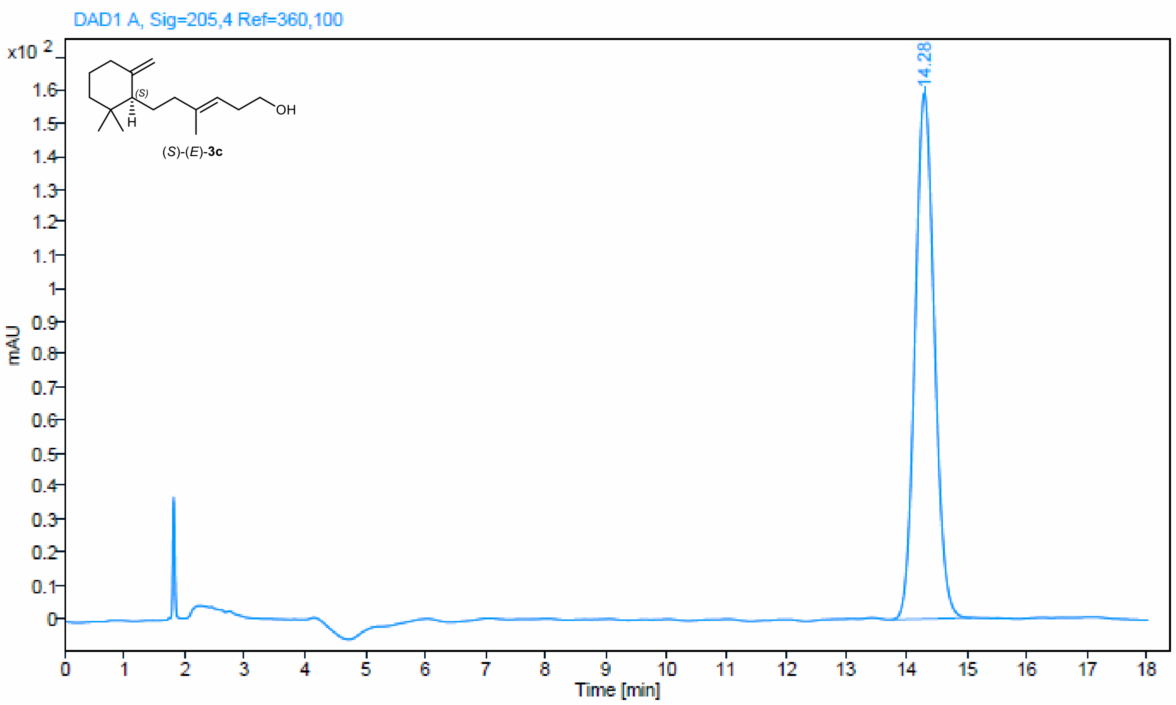

Signal: DAD1 A, Sig=205,4 Ref=360,100

RT [min]	Area%	Name
14.28	100.00	

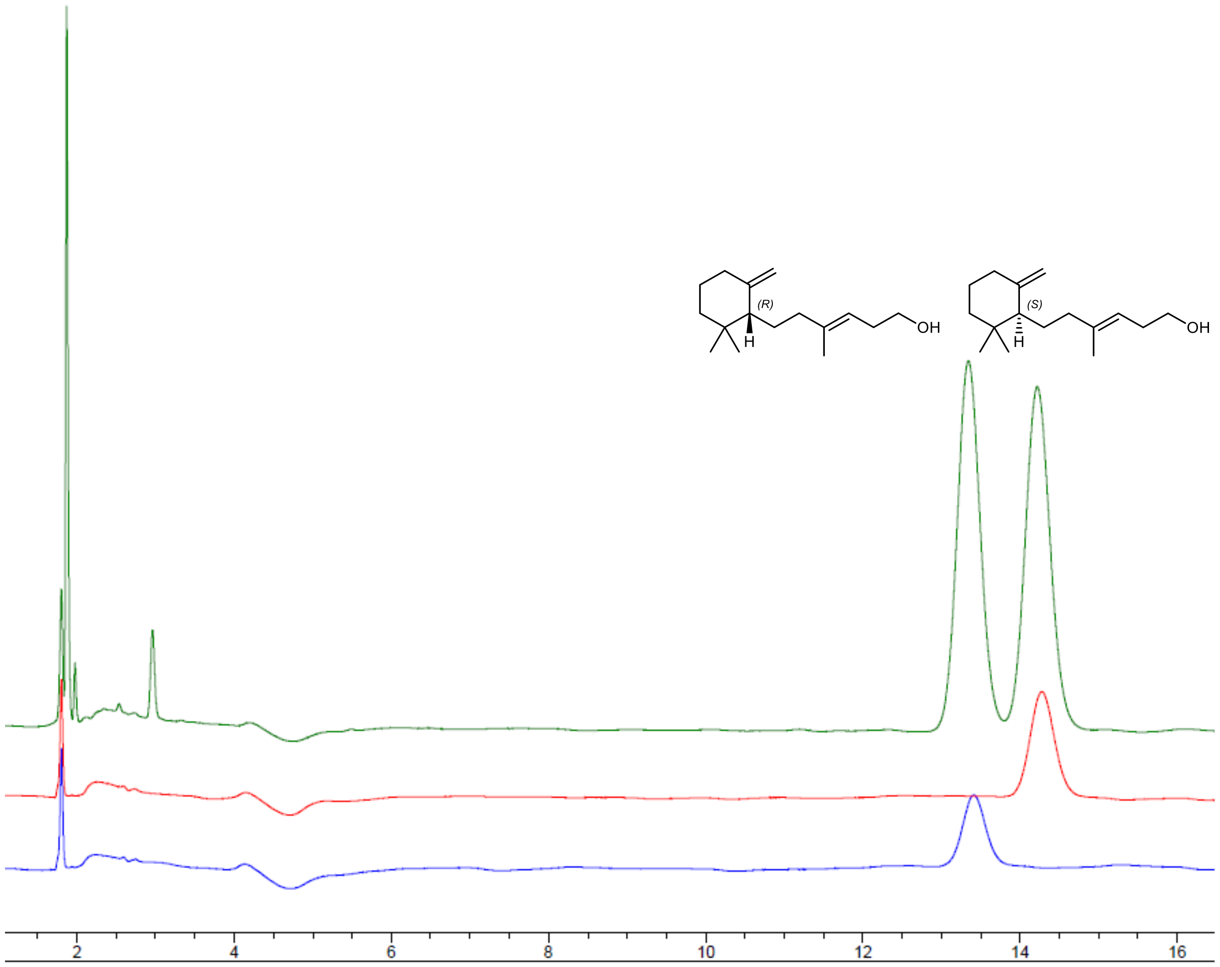

(R)
H
OH
(S)
H
OH
2
4
6
8
10
12
14
16

Chromatograms of the polar components isolated from the scale-up reaction at −40 °C in PFTB

HPLC chromatogram of the polar fraction containing a mixture of (3*E*,7*E*)-homofarnesol (**1a**), (*E*)-α-cyclohomofarnesol (**3a**) and (*E*)-γ-cyclohomofarnesol (**3c**) and traces of homodrimenols (**4**).

HPLC (achiral): 250 mm Multokrom 100-Si, 20 mm i.D., 3µm; *i*-hexan/propan-2-ol 99.5:0.5 *v/v*, 14.8 mL/min, UV: λ = 205 nm, 298 K.

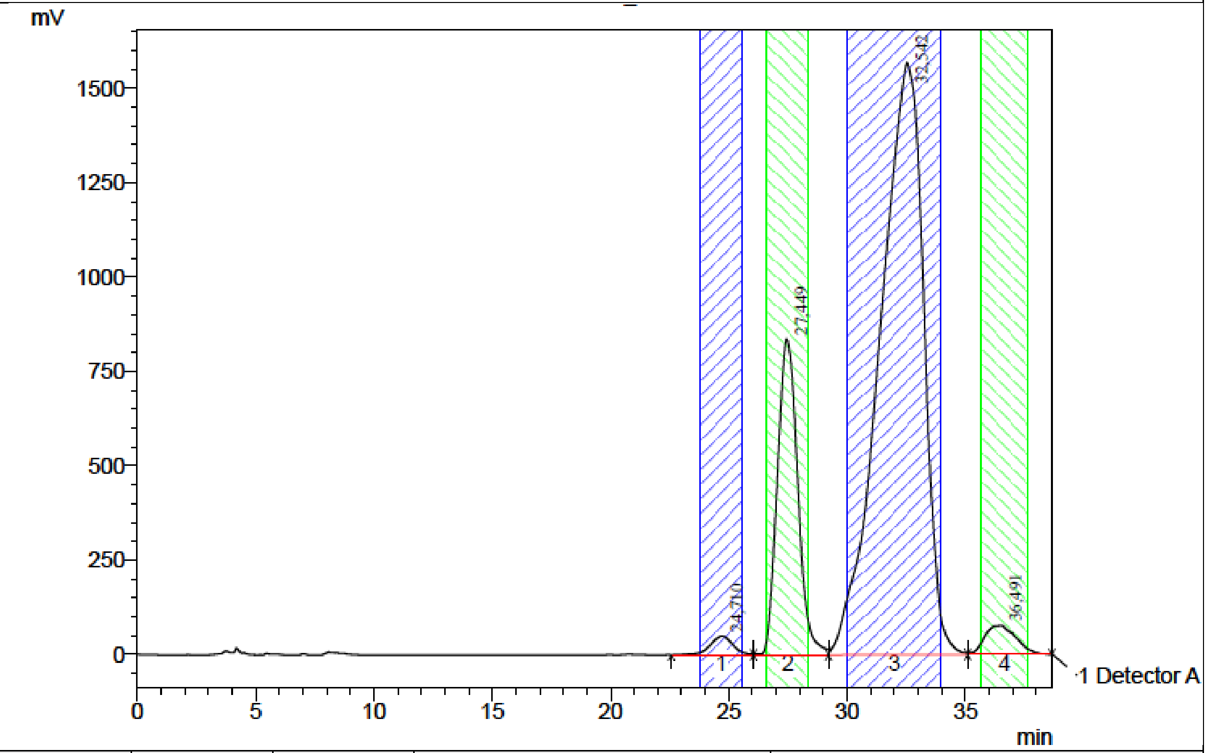

peak #	t_R / min	area / %	peak name	structure
1	24.71	1.26	not identified	n.d.
2	27.45	19.14	(3*E*,7*E*)-homofarnesol (**1a**)	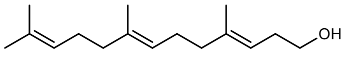 **1a** (*E* / *Z* >20:1)
3	32.54	76.87	(*E*)-α- and (*E*)-γ-cyclohomofarnesol (**3a/3c**)	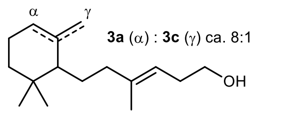
4	36.49	2.73	homodrimenols **4**	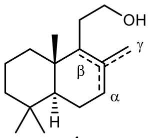 **4**

Chromatogram of recovered (3*E*,7*E*)-homofarnesol (**1a**).

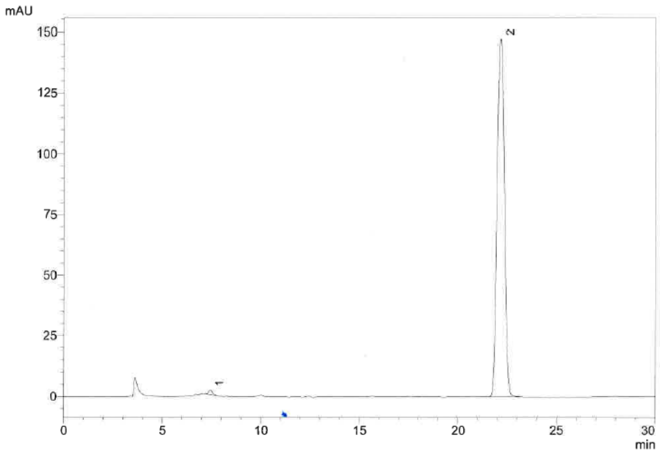

HPLC (achiral) (250 mm Multokrom 100-3-SI, 4.6 mm i.D., *i*-hexane/propan-2-ol = 99.5:0.5 *v/v*, 1 mL/min, 7.0 MPa, 308 K, UV: λ = 205 nm).

peak #	t_R / min	area / %	peak name	structure
2	22.16	99.32	(3*E*,7*E*)-homofarnesol **1a**	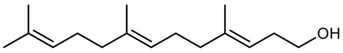

Chromatogram of the cyclohomofarnesol (**3**) fraction recovered from the scale-up reaction at −40 °C.

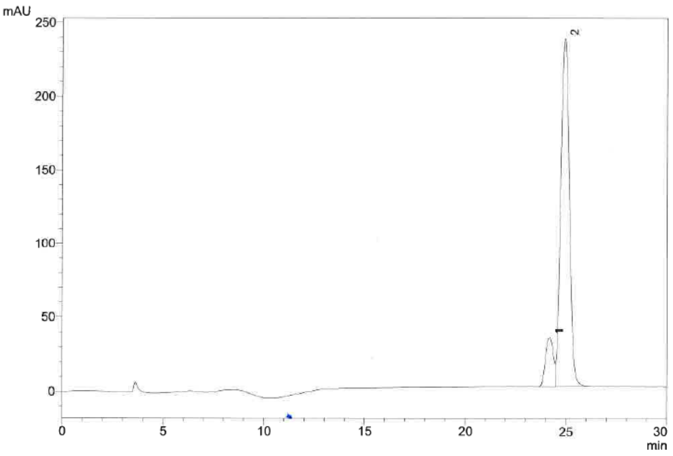

HPLC (achiral) (250 mm Multokrom 100-3-SI, 4.6 mm i.D., *i*-hexane/propan-2-ol = 99.5:0.5 *v/v*, 1 mL/min, 7.0 MPa, 308 K, UV: λ = 205 nm).

peak #	t_R / min	area / %	peak name	structure
1	25.18	11.22	(*E*)-γ-cyclohomofarnesol **3c**	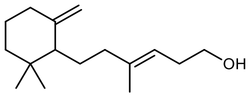 (*E*)-**3c**
2	24.93	88.78	(*E*)-α-cyclohomofarnesol **3a**	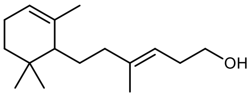 (*E*)-**3a**

Chromatogram of **3a** and **3c** obtained after preparative HPLC.

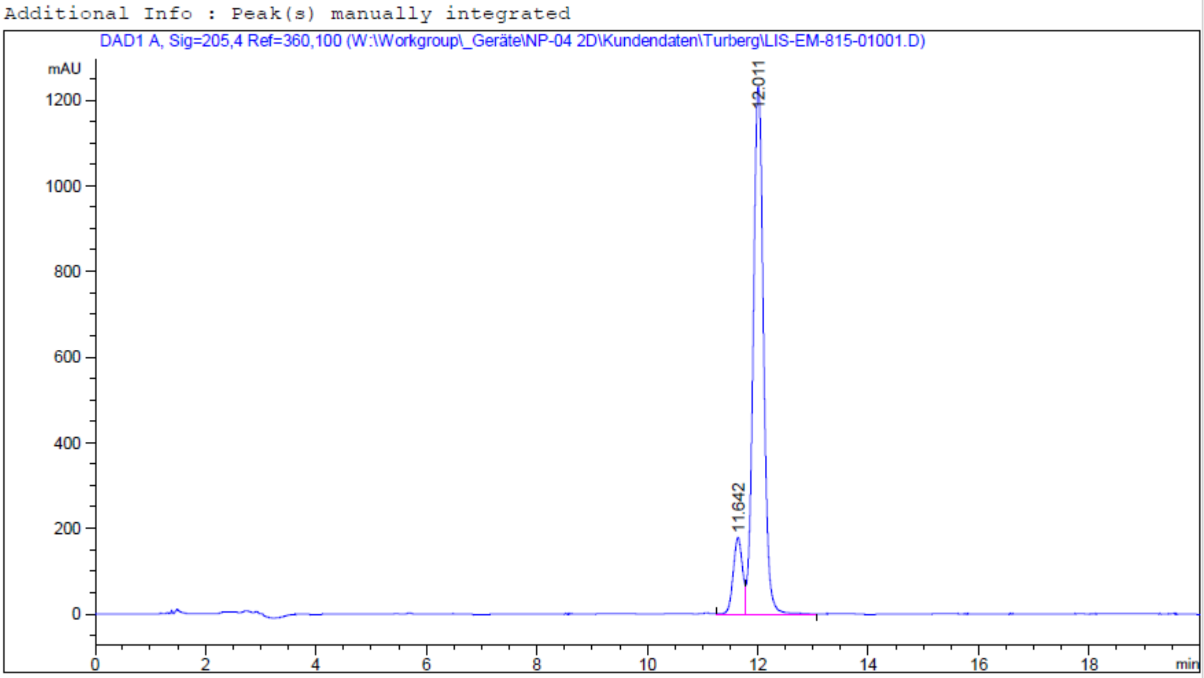

**HPLC (achiral)** (100 mm VDSpher PUR 100 SIL, 4.6 mm i.D., *n*-heptane/propan-2-ol = 99.5:0.5 *v*/*v*, 1 mL/min, 20.8 MPa, 288 K, UV: λ = 205 nm)				
**peak #**	**t_R / min**	**area / %**	**peak name**	**structure**
1	11.642	11.5513	(*E*)-γ-cyclohomofarnesol **3c**	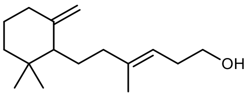
2	12.011	88.4487	(*E*)-α-cyclohomofarnesol **3a**	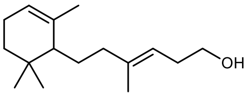

2D-HPLC separation of (*E*)-α-cyclohomofarnesol (**3a**) enantiomers.

¹D chromatogram(s)

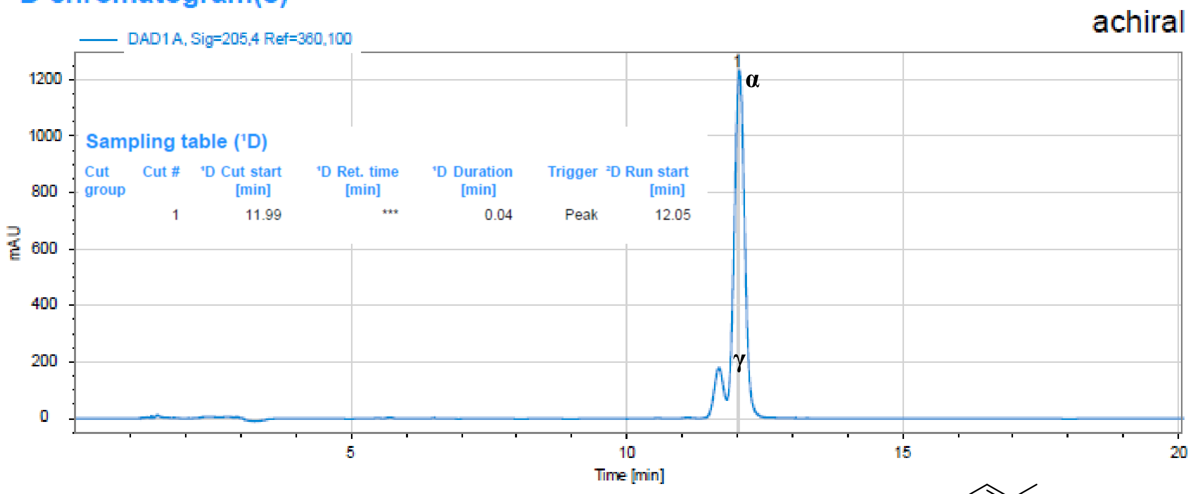

Cut# : 1

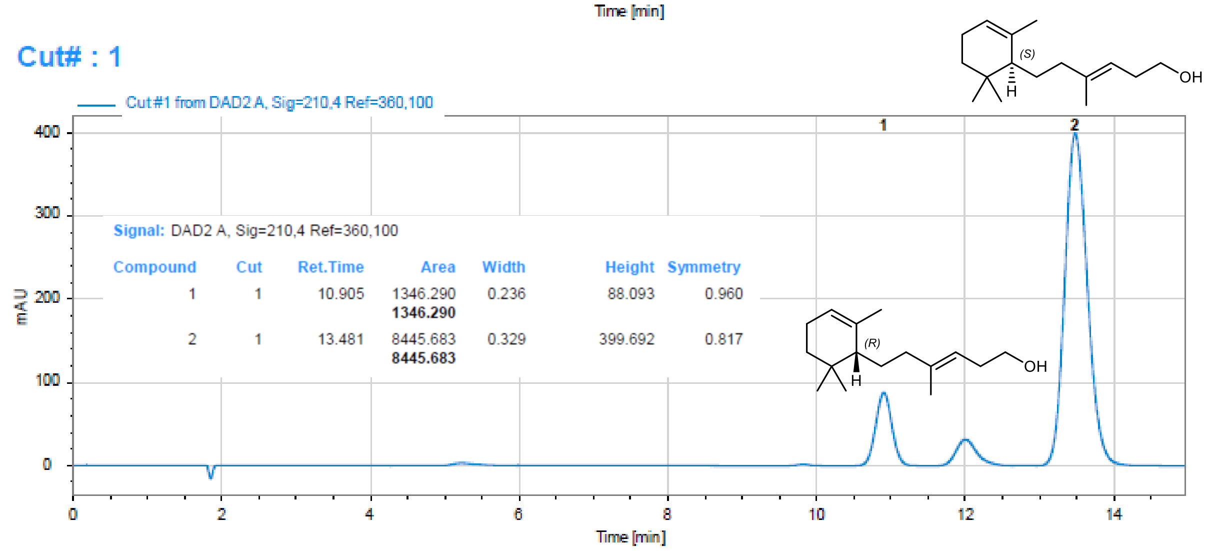

Component table

chiral

Signal: DAD2 A, Sig=210,4 Ref=360,100

Component	¹D Sampling range [min]	Ret.Time ²D [min]	Area	Area%	
1	11.99 - 12.03	10.905	1346.290	13.749	1st enantiomer
2	11.99 - 12.03	13.481	8445.683	86.251	2nd enantiomer

= 72.5 % ee

¹D (1st dimension, achiral stationary phase, 100 mm VDSpher PUR 100 SIL, 4.6 mm i.D., *n*-heptane/propan-2-ol 95.5:0.5 *v/v*, 1.0 mL/min, 20.8 MPa, 288 K, UV: λ = 205 nm): t_R = 11.99–12.03 (sampling range).
²D (2nd dimension, chiral stationary phase, 150 mm Chiralpak IG-3, 4.6 mm i.D., *n*-heptane/propan-2-ol = 99.5:0.5 *v/v*, 1.0 mL/min, 9.7 MPa, 288 K, UV: λ = 220 nm).

peak #	t_R / min	area / %	peak name
1	10.905	13.749	(*R*)-(*E*)-α-cyclohomofarnesol (*ent*-**3a**)
2	13.481	86.251	(*S*)-(*E*)-α-cyclohomofarnesol (**3a**)

2D-HPLC separation of (*E*)-γ-cyclohomofarnesol (**3c**) enantiomers.

¹D chromatogram(s)

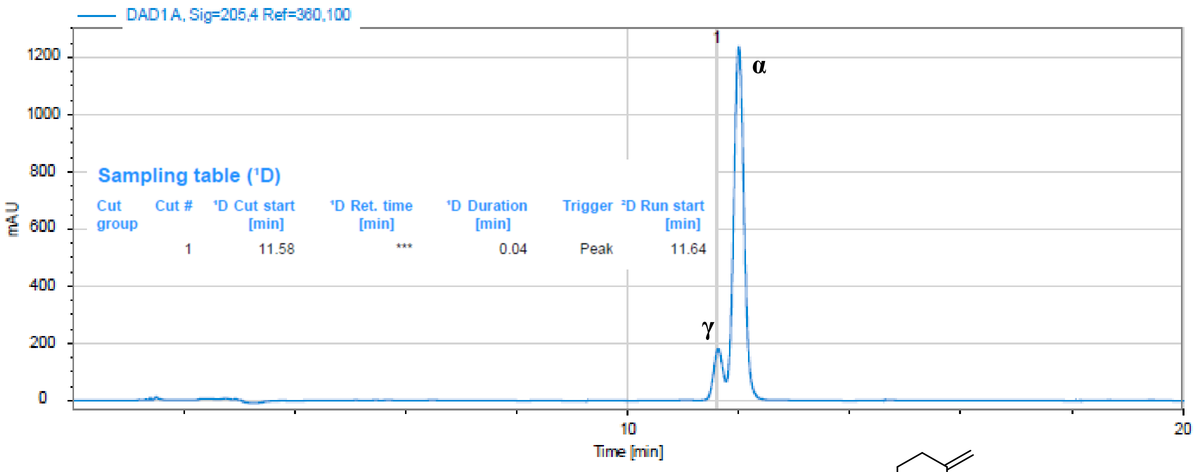

Cut# : 1

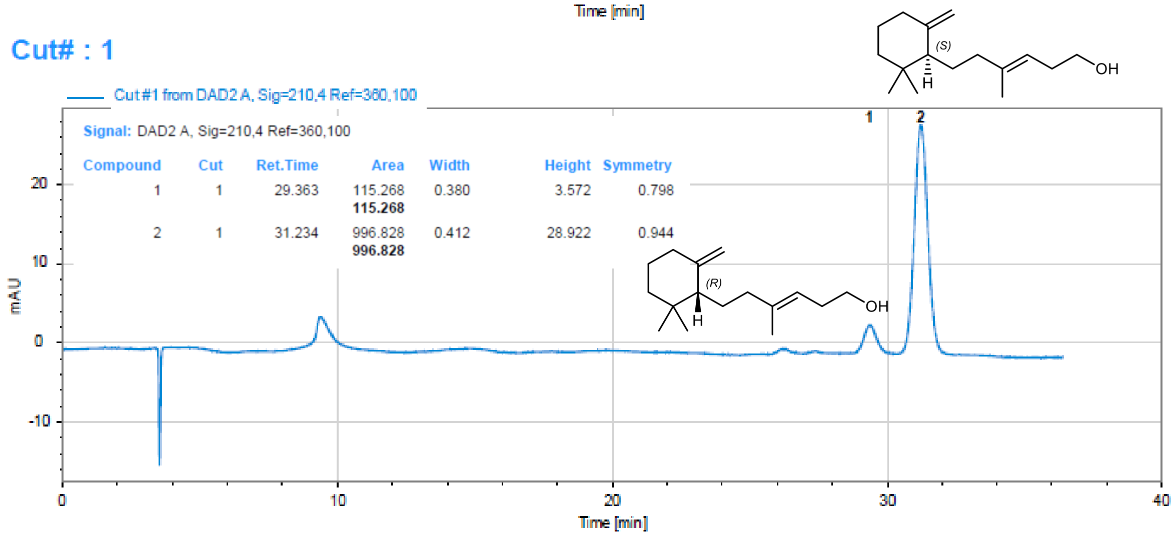

Component table

Signal: DAD2 A, Sig=210,4 Ref=360,100

Component	¹D Sampling range [min]	Ret.Time ²D [min]	Area	Area%	
1	11.58 - 11.62	29.363	115.268	10.365	1st enantiomer
2	11.58 - 11.62	31.234	996.828	89.635	2nd enantiomer

= 79.3 % ee

**¹D** (1st dimension, achiral stationary phase): 100 mm VDSpher PUR 100 SIL, 4.6 mm i.D., *n*-heptane/propan-2-ol 95.5:0.5 *v*/*v*, 1.0 mL/min, 20.9 MPa, 288 K, UV: λ = 205 nm): t_R = 11.58–11.62 (sampling range).<br>**²D** (2nd dimension, chiral stationary phase, 2 × 150 mm Chiralcel OD-3, 4.6 mm i.D., *n*-heptane/propan-2-ol 95.5:0.5 *v*/*v*, 1.0 mL/min, 14.3 MPa, 288 K, UV: λ = 220 nm).			
**peak #**	**t_R / min**	**area / %**	**peak name**
1	29.363	10.365	(*R*)-(*E*)-γ-cyclohomofarnesol (*ent*-**3a**)
2	31.234	89.635	(*S*)-(*E*)-γ-cyclohomofarnesol (**3a**)

HPLC chromatograms of the polar components isolated in the deuterium labeling experiment at −40 °C in PFTB-*d*₁

HPLC chromatogram (achiral stationary phase) of the product fraction containing polar components.

Additional Info : Peak(s) manually integrated

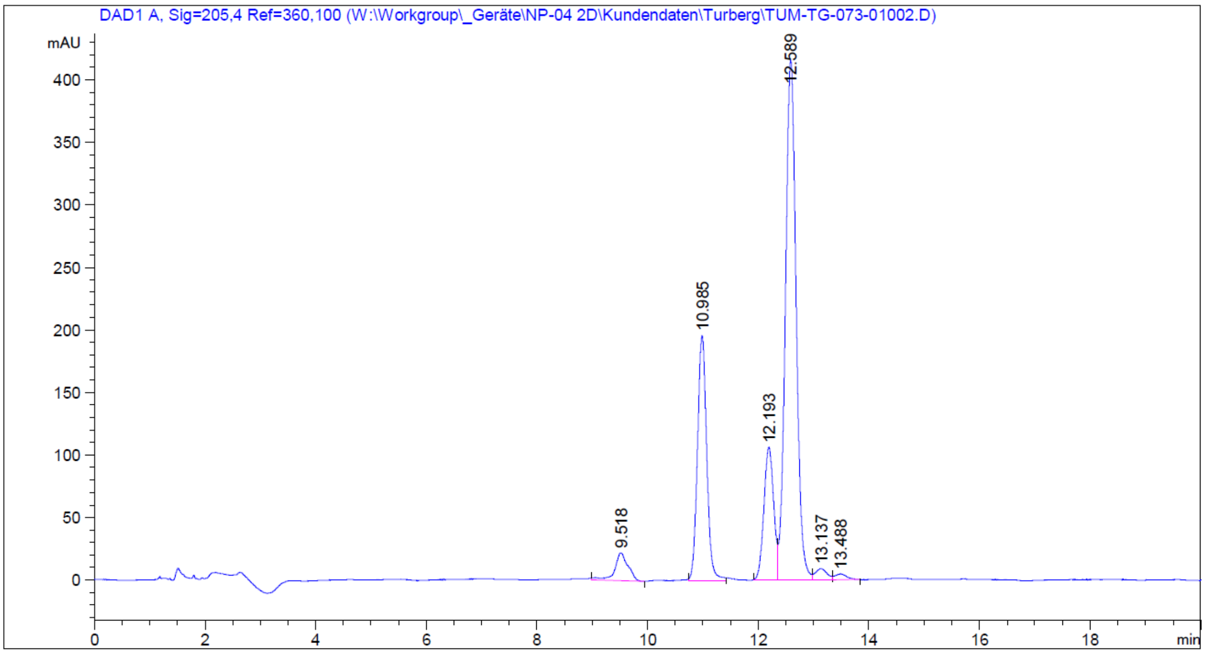

**HPLC (achiral)** (100 mm VDSpher Pur 100 Sil, 4.6 mm i.D., *n*-heptane/propan-2-ol = 99.5:0.5 *v/v*, 1 mL/min, 20.9 MPa, 288 K, UV: λ = 205 nm)				
**peak #**	**t_R / min**	**area / %**	**peak name**	**structure**
2	10.985	23.2699	(3*E*,7*E*)-homofarnesol (**1a**)	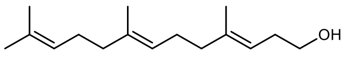
3	12.193	13.4094	(*E*)-γ-cyclohomofarnesol **3c**	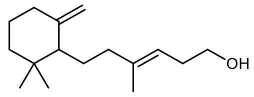
4	12.589	57.5148	(*E*)-α-cyclohomofarnesol **3a**	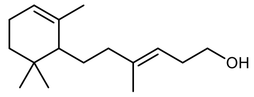

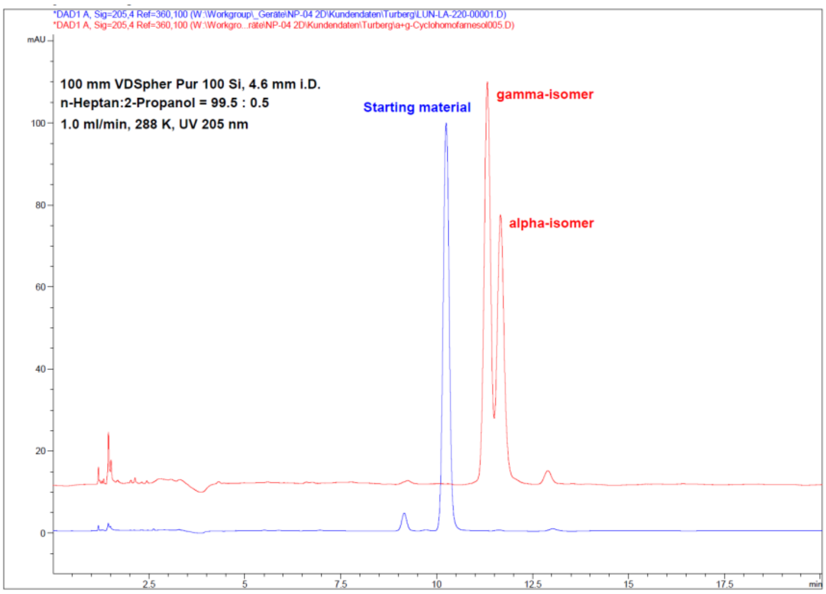

Overlayed HPLC chromatograms of starting material (3*E*,7*E*)-homofarnesol (**1a**, blue) with a deliberately prepared mixture (**3a** + **3c**, red) of (±)-(*E*)-γ-cyclohomofarnesol (**3c**) and (±)-(*E*)-α-cyclohomofarnesol (**3a**).

2D-HPLC separation of (E)-α-cyclohomofarnesol (**3a**) enantiomers.

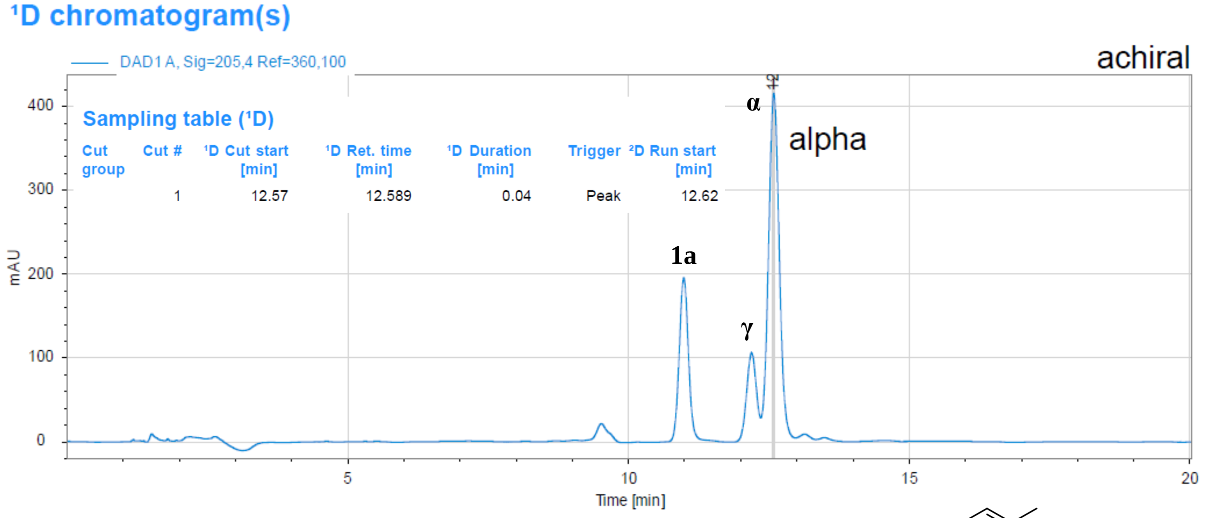

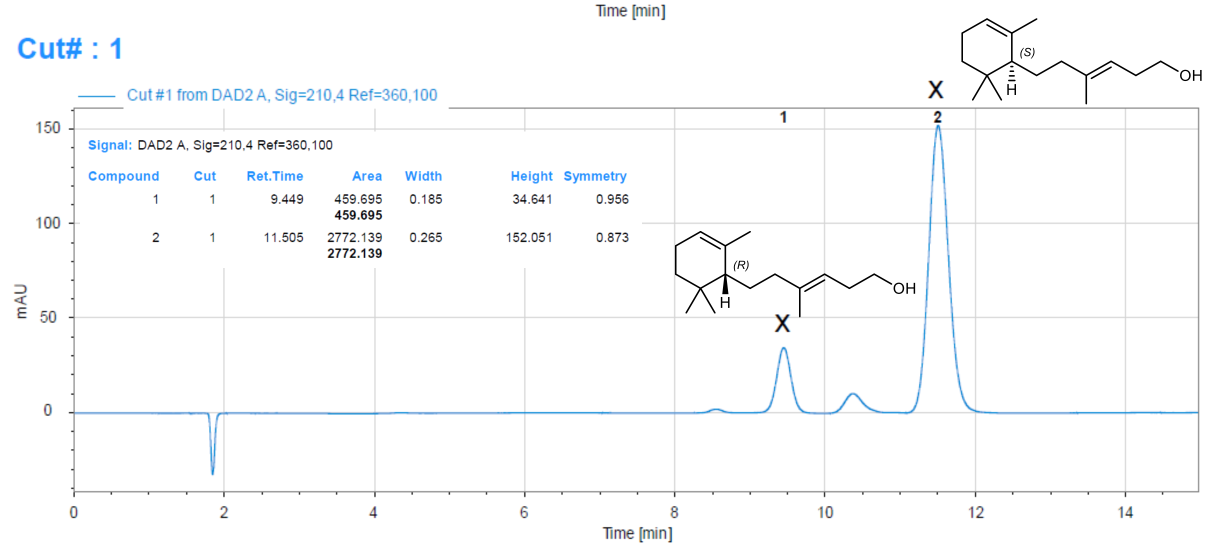

Component table

Signal: DAD2 A, Sig=210,4 Ref=360,100

chiral

Component	¹D Sampling range [min]	Ret.Time ²D [min]	Area	Area%
1	12.57 - 12.61	9.449	459.695	14.224
2	12.57 - 12.61	11.505	2772.139	85.776

= 71.6 % ee

¹D (1st dimension, achiral stationary phase, 100 mm VDSpher Pur 100 Sil, 4.6 mm i.D., n-heptane/propan-2-ol 95.5:0.5 v/v, 1.0 mL/min, 20.9 MPa, 288 K, UV: λ = 205 nm): t_R = 12.57–12.61 (sampling range).
²D (2nd dimension, chiral stationary phase, 150 mm Chiralpak IG-3, 4.6 mm i.D., n-heptane/propan-2-ol = 99.5:0.5 v/v, 1.0 mL/min, 9.8 MPa, 288 K, UV: λ = 220 nm).

peak #	t_R / min	area / %	peak name
1	9.449	14.224	(R)-(E)-α-cyclohomofarnesol (*ent*-**3a**)
2	11.505	85.776	(S)-(E)-α-cyclohomofarnesol (**3a**)

2D-HPLC separation of (*E*)-γ-cyclohomofarnesol (**3c**) enantiomers.

¹D chromatogram(s)

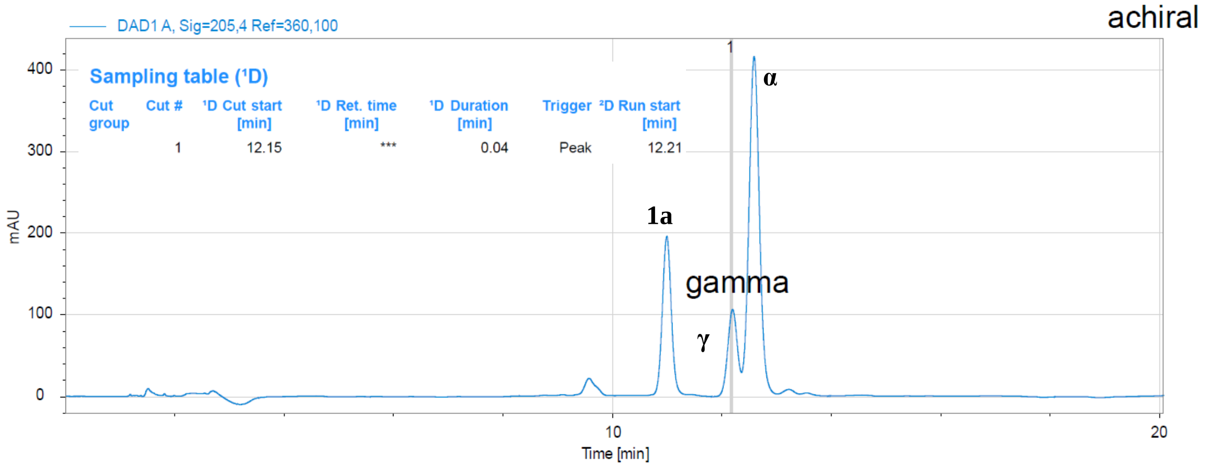

Cut# : 1

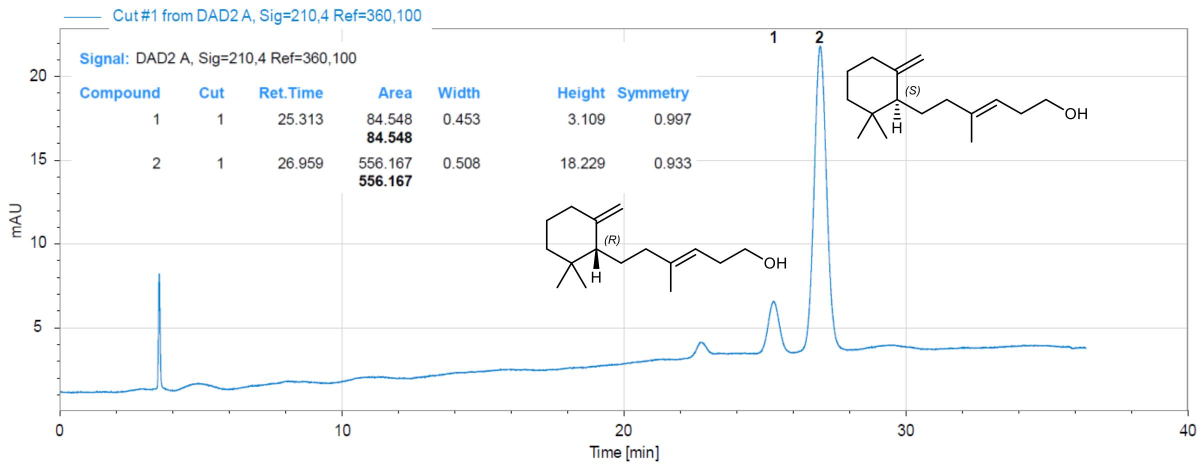

Component table

Signal: DAD2 A, Sig=210,4 Ref=360,100

chiral

Component	¹D Sampling range [min]	Ret.Time ²D [min]	Area	Area%
1	12.15 - 12.19	25.313	84.548	13.196
2	12.15 - 12.19	26.959	556.167	86.804

= 73.7 % ee

¹D (1st dimension, achiral stationary phase): 100 mm VDSpher PUR 100 SIL, 4.6 mm i.D., *n*-heptane/propan-2-ol 95.5:0.5 *v*/*v*, 1.0 mL/min, 20.9 MPa, 288 K, UV: λ = 205 nm): t_R = 12.15–12.19 (sampling range).
²D (2nd dimension, chiral stationary phase, 2 × 150 mm Chiralcel OD-3, 4.6 mm i.D., *n*-heptane/propan-2-ol 95.5:0.5 *v*/*v*, 1.0 mL/min, 9.8 MPa, 288 K, UV: λ = 220 nm).

peak #	t_R / min	area / %	peak name
1	25.313	13.196	(*R*)-(*E*)-γ-cyclohomofarnesol (*ent*-**3a**)
2	26.959	86.804	(*S*)-(*E*)-γ-cyclohomofarnesol (**3a**)

6 X-Ray Crystallographic Data

A summary of the crystal structures reported in this work are provided below in Fig. S78. Single crystals of (−)-8-*epi*-sclareolide (**12f**) were obtained by acid-catalyzed isomerization of commercially available (+)-sclareolide followed by crystallization from diethyl ether.

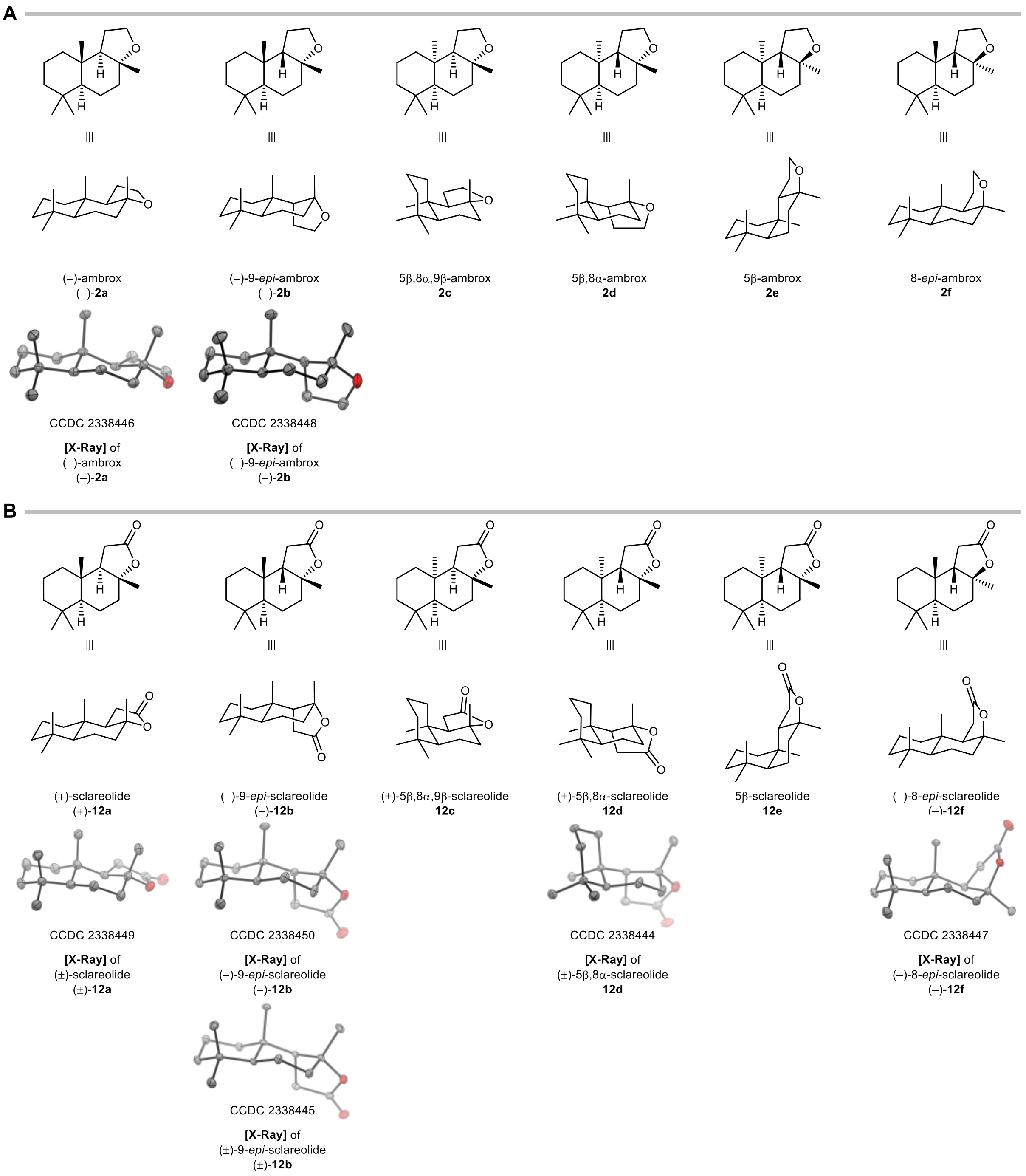

Fig. S78 | A, Overview of ambrox diastereomers with respective crystal structures reported in this work. ORTEP ellipsoids are represented at the 50% probability level. Hydrogen atoms are omitted for clarity. **B**, Overview of sclareolide diastereomers with the corresponding crystal structures reported in this work. ORTEP ellipsoids are represented at the 50% probability level. Hydrogen atoms are omitted for clarity.

Crystallographic data for (−)-ambrox (2a)

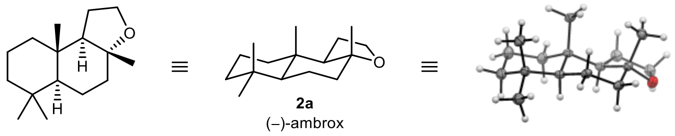

2a
(−)-ambrox

To confirm the structure and absolute configuration of (−)-ambrox, single crystal X-ray diffraction studies (SC-XRD) were conducted. To this end, a microcrystalline sample of (−)-ambrox obtained in the IDPi-catalyzed polyene cyclization of (3*E*,7*E*)-homofarnesol at −40 °C in PFTB on a 5.0 mmol scale was recrystallized. Crystals of (−)-ambrox suitable for diffraction were obtained by recrystallization from cyclopentane in a fridge overnight. The measurement was carried out using Cu-K_α radiation (λ = 1.54178 Å) to obtain as much of the anomalous dispersion of the light atoms as possible. This resulted in a statistically significant absolute structure parameter of x = 0.06(4) thus unequivocally confirming the absolute configuration of (−)-ambrox obtained in the IDPi-catalyzed polyene cyclization of (3*E*,7*E*)-homofarnesol. (−)-Ambrox crystallizes in the (non-centrosymmetric) Sohncke space group $P2_1$ (No. 4) with two independent molecules per asymmetric unit. An overlay with the corresponding deviations is depicted in Fig. S79 and confirms the identical relative and absolute configuration of both molecules.

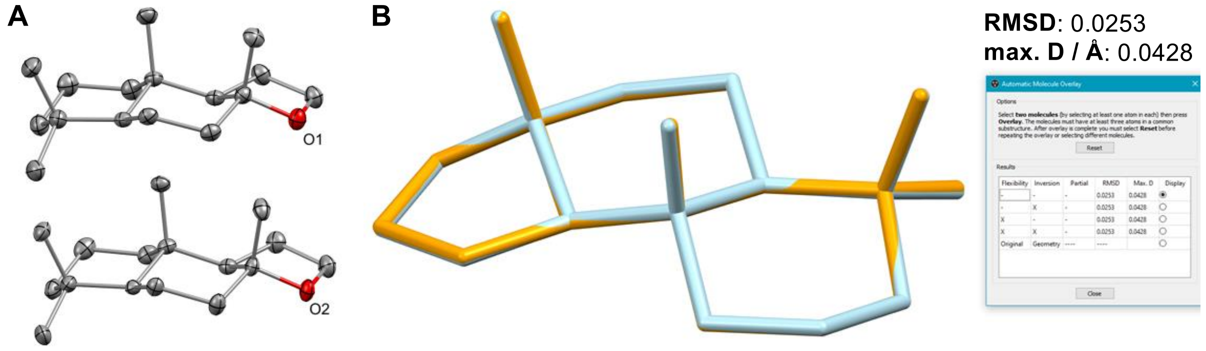

Fig. S79 | A, ORTEP depiction of the two symmetry independent molecules of (−)-ambrox (**2a**) in the asymmetric unit. Thermal ellipsoids (anisotropic displacement parameters) are drawn at 50% probability level. Hydrogen atoms are omitted for clarity. **B**, Overlay of both symmetry independent molecules created with the molecule overlay tool implemented in Mercury; RMSD: root-mean square deviation of both structures, max. D: maximum distance between two equivalent atoms in the molecule overlay.

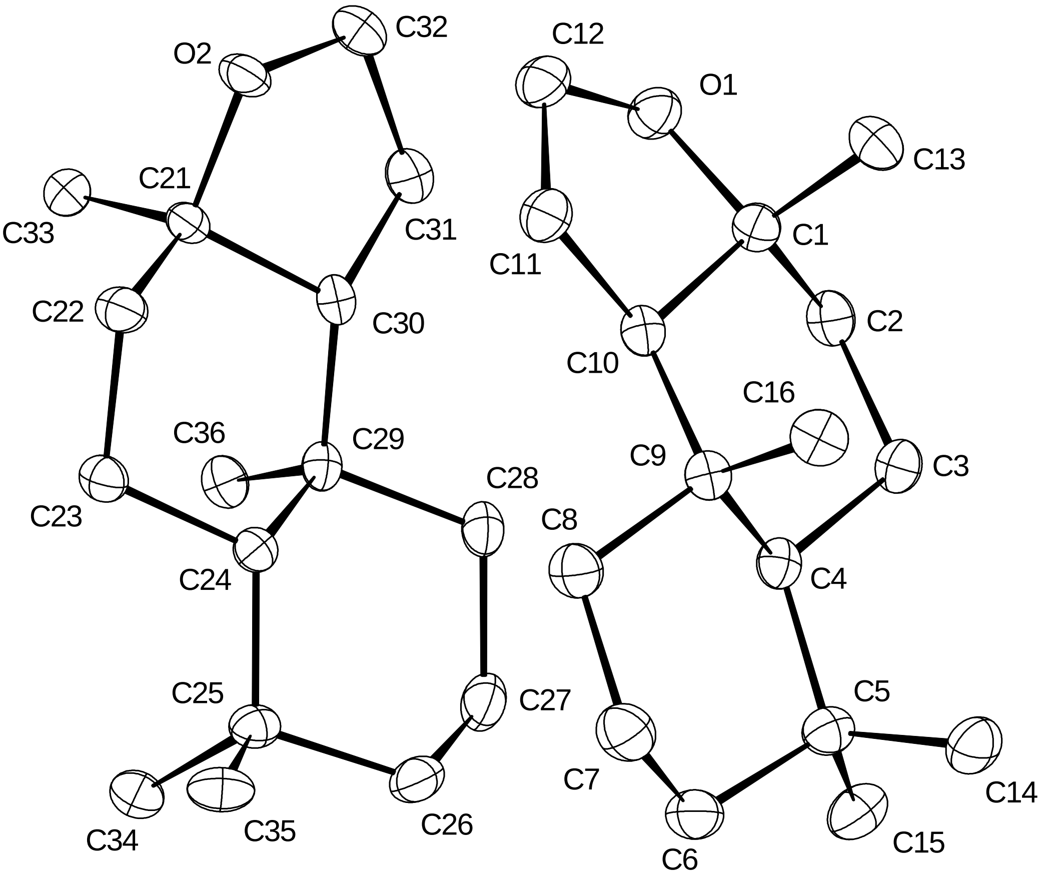

Fig. S80 | The molecular structure of (−)-ambrox (**2a**). Hydrogen atoms have been removed for clarity.

X-ray Crystal Structure Analysis of (−)-ambrox (**2a**):

C_{16} H_{28} O, M_r = 236.38 g · mol^{-1}, colorless plate, crystal size 0.221 × 0.13 × 0.06 mm^3, monoclinic, space group $P2_1$ [4], a = 7.5405(3) Å, b = 10.7267(3) Å, c = 17.2126(6) Å, β = 95.958(2)°, V = 1384.72(8) Å^3, T = 100(2) K, Z = 4, D_{calc} = 1.134 g · cm^3, λ = 1.54178 Å, μ(Cu-K$_\alpha$) = 0.509 mm^{-1}, Numerical correction ($T_{\min}$ = 0.91980, $T_{\max}$ = 0.97706), Bruker-AXS Kappa Mach3 with APEX-II detector and FR591 rotating anode X-ray source with Incoatec Helios mirrors, 2.581 < θ < 74.375°, 47333 measured reflections, 4969 independent reflections, 4770 reflections with $I > 2\sigma(I)$, R_{int} = 0.0325. The structure was solved by *SHELXT* and refined by full-matrix least-squares (*SHELXL*) against F^2 to R_1 = 0.0326 [$I > 2\sigma(I)$], wR_2 = 0.0824 [all data], 331 parameters, 1 restraints and absolute structure parameter x = 0.06(4).

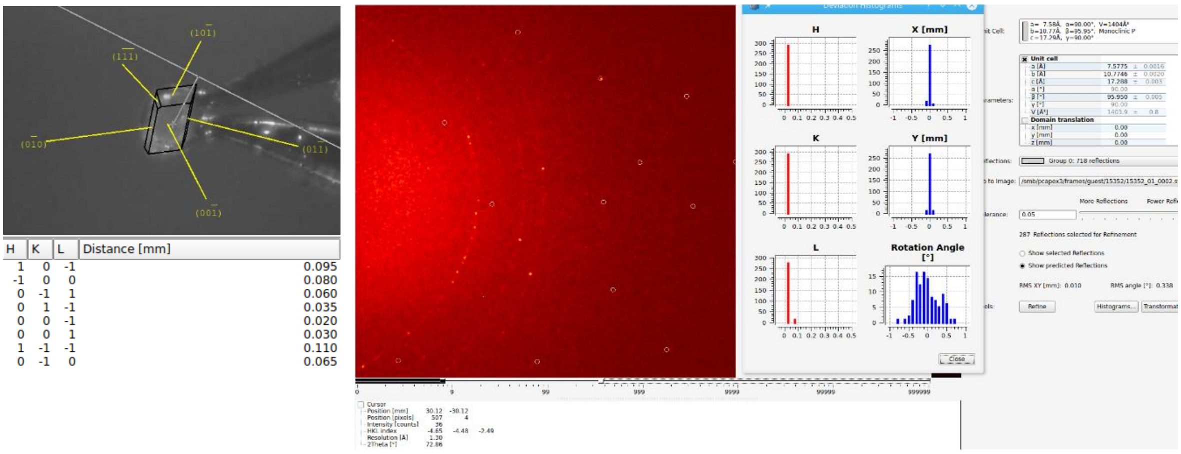

Fig. S81 | Crystal faces and unit cell determination/refinement of (−)-ambrox (**2a**).

INTENSITY STATISTICS FOR DATASET

Resolution	#Data	#Theory	%Complete	Redundancy	Mean I	Mean I/s	Rmerge	Rsigma
Inf - 3.39	76	76	100.0	9.46	133.26	87.39	0.0304	0.0112
3.39 - 2.27	179	179	100.0	9.18	48.60	79.49	0.0231	0.0110
2.27 - 1.79	253	253	100.0	10.55	35.97	79.02	0.0255	0.0110
1.79 - 1.57	248	248	100.0	10.15	16.36	69.84	0.0261	0.0123
1.57 - 1.43	248	248	100.0	9.10	13.49	56.73	0.0289	0.0143
1.43 - 1.32	247	252	98.0	8.03	11.99	52.33	0.0316	0.0154
1.32 - 1.24	263	273	96.3	8.69	13.93	55.71	0.0338	0.0166
1.24 - 1.18	239	247	96.8	14.09	12.55	76.93	0.0319	0.0120
1.18 - 1.12	292	308	94.8	12.97	13.79	70.96	0.0309	0.0127
1.12 - 1.07	272	284	95.8	13.08	9.58	65.53	0.0337	0.0144
1.07 - 1.04	215	228	94.3	11.39	6.89	57.69	0.0354	0.0169
1.04 - 1.00	287	306	93.8	11.22	6.19	55.29	0.0433	0.0173
1.00 - 0.97	237	272	87.1	9.92	5.75	50.35	0.0463	0.0256
0.97 - 0.94	278	317	87.7	9.35	5.16	49.12	0.0478	0.0247
0.94 - 0.92	210	237	88.6	9.63	3.76	43.99	0.0595	0.0273
0.92 - 0.90	248	271	91.5	9.20	3.08	38.45	0.0664	0.0360
0.90 - 0.88	235	265	88.7	8.48	3.01	38.36	0.0640	0.0336
0.88 - 0.86	265	316	83.9	5.47	3.14	28.84	0.0828	0.0484
0.86 - 0.84	240	307	78.2	2.03	2.18	10.96	0.1507	0.1354
0.84 - 0.82	296	369	80.2	1.80	2.51	11.51	0.1365	0.1153
0.82 - 0.79	180	667	27.0	0.47	2.69	9.81	0.0888	0.1279
0.79 - 0.78	5	113	4.4	0.07	5.29	12.26	0.0581	0.0793
0.88 - 0.78	986	1772	55.6	1.88	2.65	15.73	0.1000	0.1000
Inf - 0.78	5013	6036	83.1	7.86	12.47	50.65	0.0324	0.0176

Complete .cif-data are available under the CCDC number CCDC-2338446.

Table S25 | Crystal data and structure refinement for (−)-ambrox (2a) obtained as product in an IDPi-catalyzed polyene cyclization of (3*E*,7*E*)-homofarnesol at −40 °C in PFTB.

Identification code	15352 / CCDC-2338446	
Empirical formula	$C_{16}H_{28}O$	
Colour	colorless	
Formula weight	236.38 g · mol^{-1}	
Temperature	100(2) K	
Wavelength	1.54178 Å	
Crystal system	monoclinic	
Space group	$P2_1$, (No. 4)	
Unit cell dimensions	a = 7.5405(3) Å	α = 90°.
	b = 10.7267(3) Å	β = 95.958(2)°.
	c = 17.2126(6) Å	γ = 90°.
Volume	1384.72(8) Å^3	
Z	4	
Density (calculated)	1.134 Mg · m^{-3}	
Absorption coefficient	0.509 mm^{-1}	
F(000)	528 e	
Crystal size	0.221 × 0.13 × 0.06 mm^3	
θ range for data collection	2.581 to 74.375°.	
Index ranges	$-9 \le h \le 9$, $-13 \le k \le 12$, $-20 \le l \le 21$	
Reflections collected	47333	
Independent reflections	4969 [R_{int} = 0.0325]	
Reflections with $I > 2\sigma(I)$	4770	
Completeness to θ = 67.679°	97.2 %	
Absorption correction	Gaussian	
Max. and min. transmission	0.97706 and 0.91980	
Refinement method	Full-matrix least-squares on F^2	
Data / restraints / parameters	4969 / 1 / 331	
Goodness-of-fit on F^2	1.138	
Final R indices [$I > 2\sigma(I)$]	R_1 = 0.0326	wR_2 = 0.0791
R indices (all data)	R_1 = 0.0366	wR_2 = 0.0824
Absolute structure parameter	0.06(4)	
Extinction coefficient	n/a	

Table S26 | Bond lengths [Å] and angles [°] for (−)-ambrox (**2a**) obtained as product in an IDPi-catalyzed polyene cyclization of (3*E*,7*E*)-homofarnesol at −40 °C in PFTB.

O(1)-C(1)	1.443(3)	O(1)-C(12)	1.445(3)
C(1)-C(2)	1.521(3)	C(1)-C(10)	1.533(3)
C(1)-C(13)	1.525(3)	C(2)-C(3)	1.538(3)
C(3)-C(4)	1.536(3)	C(4)-H(4)	1.01(3)
C(4)-C(5)	1.554(3)	C(4)-C(9)	1.563(3)
C(5)-C(6)	1.539(3)	C(5)-C(14)	1.528(3)
C(5)-C(15)	1.535(3)	C(6)-C(7)	1.525(4)
C(7)-C(8)	1.529(3)	C(8)-C(9)	1.536(3)
C(9)-C(10)	1.538(3)	C(9)-C(16)	1.537(3)
C(10)-H(10)	0.98(3)	C(10)-C(11)	1.521(3)
C(11)-C(12)	1.536(3)	O(2)-C(21)	1.448(3)
O(2)-C(32)	1.444(3)	C(21)-C(22)	1.512(3)
C(21)-C(30)	1.532(3)	C(21)-C(33)	1.530(3)
C(22)-C(23)	1.539(3)	C(23)-C(24)	1.537(3)
C(24)-H(24)	0.99(3)	C(24)-C(25)	1.550(3)
C(24)-C(29)	1.555(3)	C(25)-C(26)	1.539(3)
C(25)-C(34)	1.530(3)	C(25)-C(35)	1.532(3)
C(26)-C(27)	1.520(3)	C(27)-C(28)	1.523(3)
C(28)-C(29)	1.540(3)	C(29)-C(30)	1.537(3)
C(29)-C(36)	1.539(3)	C(30)-H(30)	0.95(3)
C(30)-C(31)	1.519(3)	C(31)-C(32)	1.542(3)
C(1)-O(1)-C(12)	106.62(16)	O(1)-C(1)-C(2)	110.85(18)
O(1)-C(1)-C(10)	100.79(16)	O(1)-C(1)-C(13)	106.91(19)
C(2)-C(1)-C(10)	108.72(19)	C(2)-C(1)-C(13)	110.91(18)
C(13)-C(1)-C(10)	118.13(19)	C(1)-C(2)-C(3)	109.60(18)
C(4)-C(3)-C(2)	112.52(17)	C(3)-C(4)-H(4)	104.6(14)
C(3)-C(4)-C(5)	114.75(18)	C(3)-C(4)-C(9)	111.28(18)
C(5)-C(4)-H(4)	102.8(14)	C(5)-C(4)-C(9)	116.29(18)
C(9)-C(4)-H(4)	105.5(14)	C(6)-C(5)-C(4)	108.36(18)
C(14)-C(5)-C(4)	113.9(2)	C(14)-C(5)-C(6)	110.8(2)
C(14)-C(5)-C(15)	107.35(19)	C(15)-C(5)-C(4)	109.26(19)
C(15)-C(5)-C(6)	107.0(2)	C(7)-C(6)-C(5)	115.1(2)
C(6)-C(7)-C(8)	110.9(2)	C(7)-C(8)-C(9)	112.41(19)
C(8)-C(9)-C(4)	108.35(18)	C(8)-C(9)-C(10)	109.11(18)

C(8)-C(9)-C(16)	108.62(18)	C(10)-C(9)-C(4)	103.12(17)
C(16)-C(9)-C(4)	115.47(17)	C(16)-C(9)-C(10)	111.92(19)
C(1)-C(10)-C(9)	116.86(17)	C(1)-C(10)-H(10)	102.7(13)
C(9)-C(10)-H(10)	104.3(13)	C(11)-C(10)-C(1)	101.90(18)
C(11)-C(10)-C(9)	123.08(19)	C(11)-C(10)-H(10)	105.9(14)
C(10)-C(11)-C(12)	101.26(18)	O(1)-C(12)-C(11)	107.74(18)
C(32)-O(2)-C(21)	106.73(17)	O(2)-C(21)-C(22)	111.08(18)
O(2)-C(21)-C(30)	100.71(16)	O(2)-C(21)-C(33)	106.69(17)
C(22)-C(21)-C(30)	109.38(18)	C(22)-C(21)-C(33)	111.33(18)
C(33)-C(21)-C(30)	117.09(19)	C(21)-C(22)-C(23)	109.53(18)
C(24)-C(23)-C(22)	112.15(18)	C(23)-C(24)-H(24)	105.5(14)
C(23)-C(24)-C(25)	114.53(18)	C(23)-C(24)-C(29)	111.59(17)
C(25)-C(24)-H(24)	102.8(14)	C(25)-C(24)-C(29)	116.01(18)
C(29)-C(24)-H(24)	104.8(14)	C(26)-C(25)-C(24)	107.93(18)
C(34)-C(25)-C(24)	114.73(18)	C(34)-C(25)-C(26)	110.74(19)
C(34)-C(25)-C(35)	107.48(19)	C(35)-C(25)-C(24)	108.82(18)
C(35)-C(25)-C(26)	106.84(19)	C(27)-C(26)-C(25)	114.51(19)
C(26)-C(27)-C(28)	111.4(2)	C(27)-C(28)-C(29)	112.23(18)
C(28)-C(29)-C(24)	108.58(17)	C(30)-C(29)-C(24)	104.06(17)
C(30)-C(29)-C(28)	108.76(17)	C(30)-C(29)-C(36)	112.08(17)
C(36)-C(29)-C(24)	114.45(18)	C(36)-C(29)-C(28)	108.70(18)
C(21)-C(30)-C(29)	115.94(17)	C(21)-C(30)-H(30)	102.5(14)
C(29)-C(30)-H(30)	104.7(14)	C(31)-C(30)-C(21)	102.03(18)
C(31)-C(30)-C(29)	123.32(19)	C(31)-C(30)-H(30)	106.2(14)
C(30)-C(31)-C(32)	101.31(18)	O(2)-C(32)-C(31)	107.53(18)

Crystallographic data for (−)-9-*epi*-ambrox (2b)

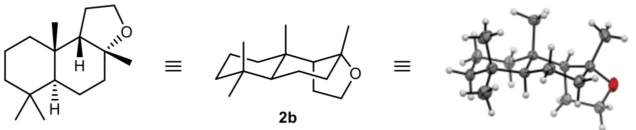

2b
(–)-9-*epi*-ambrox

Crystals of diastereo- and enantiopure (−)-9-*epi*-ambrox (**2b**, >50:1 d.r., >99.5:0.5 e.r.) suitable for diffraction were obtained by repeated heating and slow cooling of the low melting waxy solid. While crystals obtained this way were of sufficient quality for confirmation of the expected relative configuration, the crystals diffracted poorly resulting in a low completeness (92.1%) and an A-level alert in the checkCIF routine. Crystals of sufficient quality for determination of the absolute configuration could be obtained using in situ capillary crystallization as an alternative method[57–59] which has been successfully applied at our institute in several cases[60,61] including for the determination of the absolute configuration of enantioenriched or enantiopure compounds.[62,63] To this end, the neat waxy sample was filled into a borosilicate glass capillary with 0.5 mm outer diameter and gently chilled in a stream of cold air. The obtained polycrystalline material was subsequently locally warmed with a heat gun to slightly above its melting temperature. Repeating the cooling/warming cycle afforded single crystals suitable for X-ray diffraction. The capillary was rapidly transferred to the diffractometer for collection of the data set. The absolute structure parameter of $x = 0.21(11)$ obtained from the initial measurement with Mo-K_α radiation (λ = 0.71073 Å) suggested the correct absolute configuration, but was not statistically significant. The measurement was therefore repeated with Cu-K_α radiation (λ = 1.54178 Å) which eventually afforded a statistically significant Flack parameter of $x = -0.04(4)$, thus unequivocally confirming the absolute configuration of (−)-9-*epi*-ambrox (**2b**).

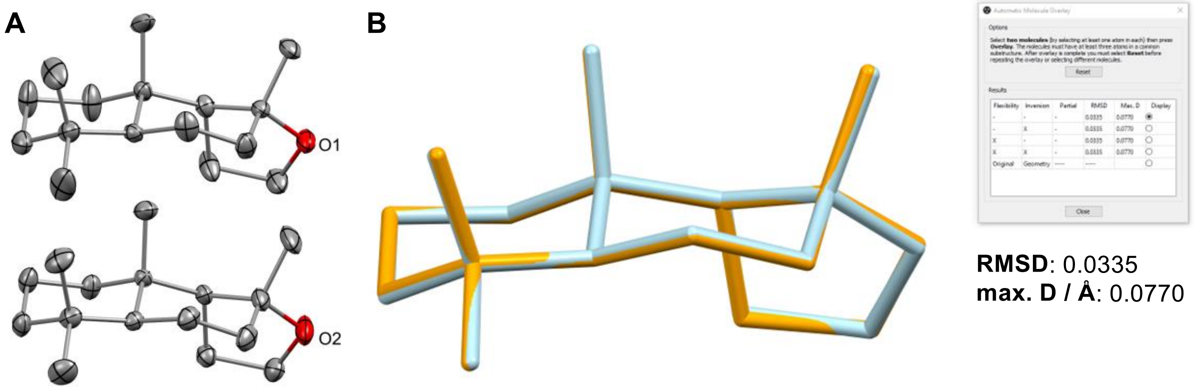

Fig. S82 | A, Solid state structure of (−)-9-*epi*-ambrox (**2b**) with two symmetry independent molecules per asymmetric unit. Thermal ellipsoids (anisotropic displacement parameters) are drawn at 50% probability level. Hydrogen atoms are omitted for clarity. **B**, Overlay of both symmetry independent molecules created with the molecule overlay tool implemented in Mercury; RMSD: root-mean square deviation of both structures, max. D: maximum distance between two equivalent atoms in the molecule overlay.

(−)-9-*epi*-Ambrox crystallizes in the (non-centrosymmetric) Sohncke space group $C222_1$ (No. 20) with two independent molecules per asymmetric unit. An overlay of both structures with the corresponding deviations is shown in Fig. S82.

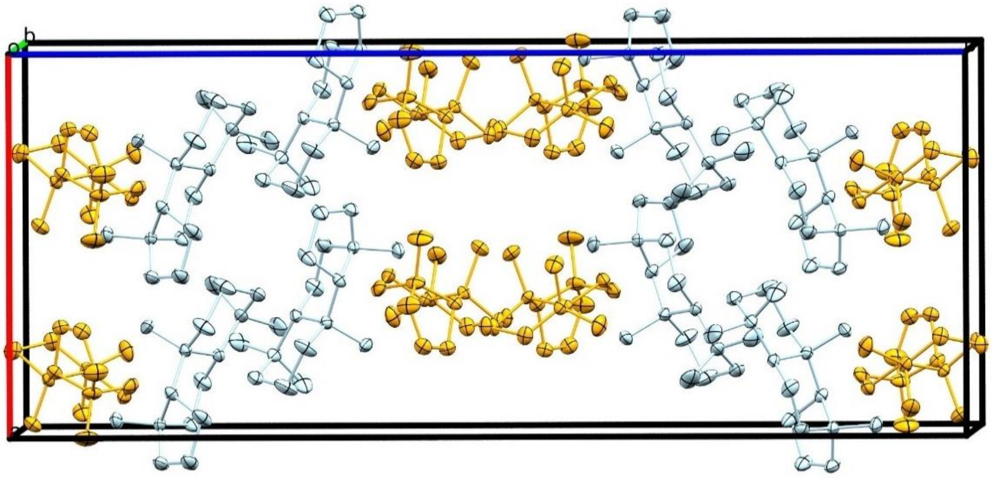

Fig. S83 | View of the solid state structure of (−)-9-*epi*-ambrox (**2b**) almost along the crystallographic b axis. Both symmetry independent molecules are shown in blue and orange, respectively.

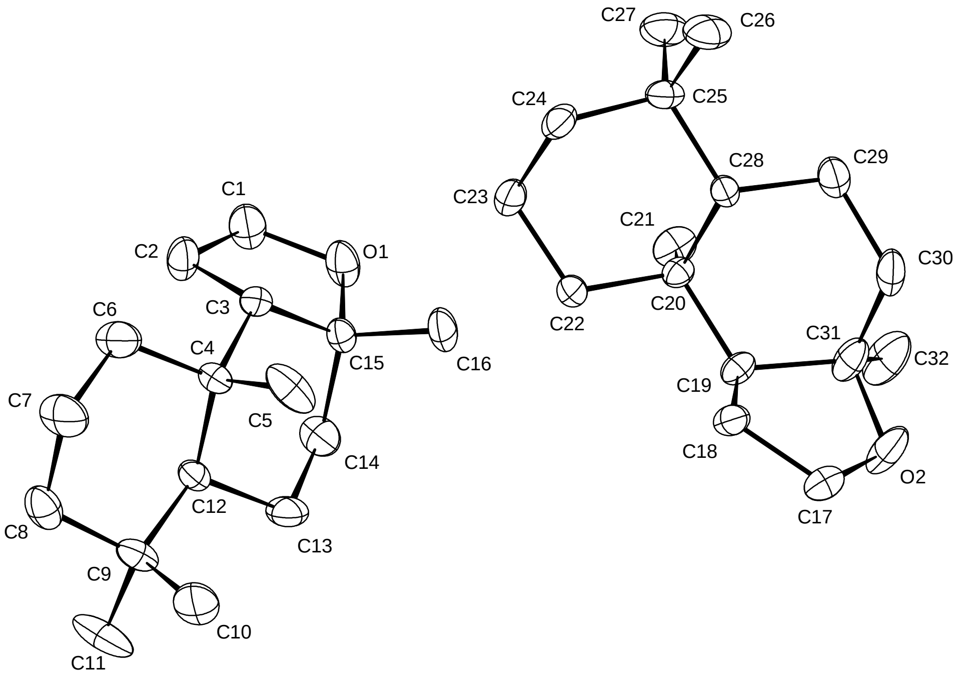

Fig. S84 | The molecular structure of (−)-9-*epi*-ambrox (**2b**). Hydrogen atoms have been removed for clarity.

X-ray Crystal Structure Analysis of (−)-9-epi-ambrox (**2b**):

$C_{16}H_{28}O$, M_r = 236.400 g · mol^{-1}, colorless capillary, crystal size 0.749 × 0.619 × 0.200 mm^3, orthorhombic, space group $C222_1$ [20], a = 12.529(3) Å, b = 14.555(3) Å, c = 31.280(7) Å, V = 5704(2) Å^3, T = 100(2) K, Z = 16, D_{calc} = 1.101 g · cm^3, λ = 1.54178 Å, μ(Cu-K$_\alpha$) = 0.494 mm^{-1}, Numerical correction ($T_{\min}$ = 0.76318, $T_{\max}$ = 0.90797), Bruker-AXS Kappa Mach3 with APEX-II detector and FR591 rotating anode X-ray source with Incoatec Helios mirrors, 2.83 < θ < 70.06°, 77434 measured reflections, 5252 independent reflections, 5004 reflections with $I > 2\sigma(I)$, R_{int} = 0.0602. The structure was solved by *SHELXT* and refined by full-matrix least-squares (*SHELXL*). The final structure refinement was performed by olex2.refine 1.5 (L-M) together with NoSpherA2 (atomic form factors) against F^2 to R_1 = 0.0347 [$I > 2\sigma(I)$], wR_2 = 0.0779 [all data] with 475 parameters, 0 restraints and an absolute structure parameter x = −0.04(4).

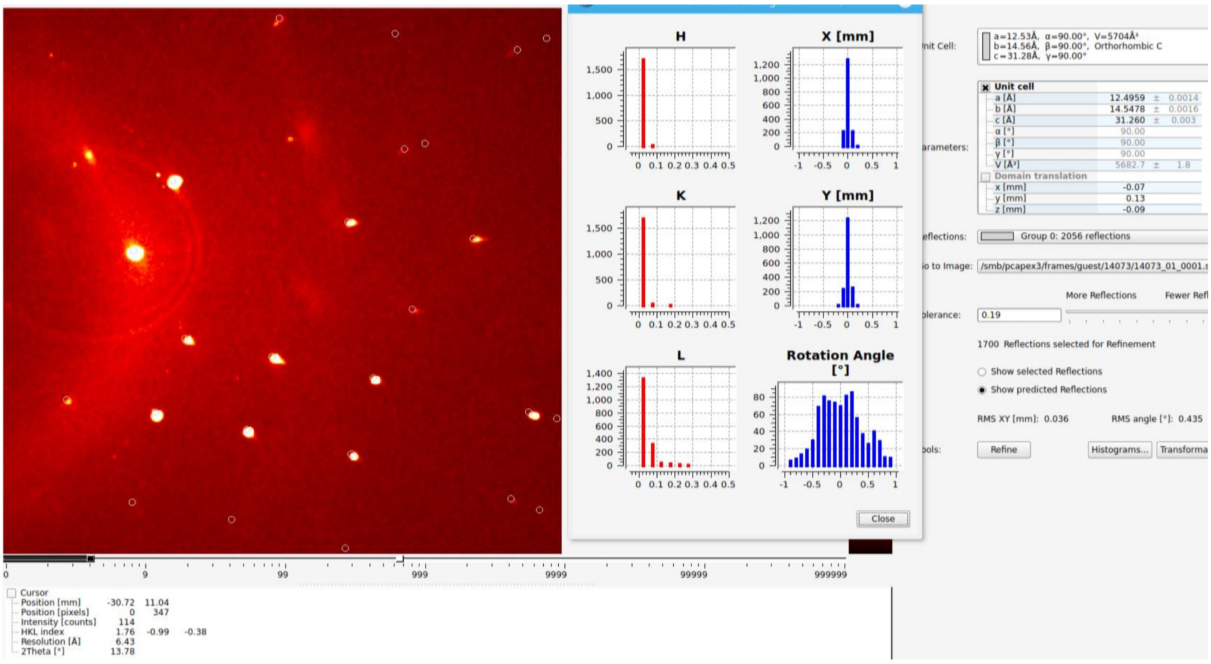

Fig. S85 | Unit cell determination/refinement of (−)-9-*epi*-ambrox (**2b**).

INTENSITY STATISTICS FOR DATASET

Resolution	#Data	#Theory	%Complete	Redundancy	Mean I	Mean I/s	Rmerge	Rsigma
Inf - 3.61	47	51	92.2	16.20	145.46	84.94	0.0488	0.0128
3.61 - 2.32	107	111	96.4	22.94	118.32	103.27	0.0351	0.0096
2.32 - 1.81	151	158	95.6	24.09	92.69	105.50	0.0391	0.0105
1.81 - 1.56	159	163	97.5	25.31	42.36	100.87	0.0452	0.0158
1.56 - 1.41	155	160	96.9	23.58	31.11	98.64	0.0494	0.0153
1.41 - 1.31	150	151	99.3	21.34	26.94	87.67	0.0576	0.0195
1.31 - 1.23	152	153	99.3	28.63	31.59	98.61	0.0686	0.0274
1.23 - 1.17	140	144	97.2	39.28	27.13	111.99	0.0729	0.0165
1.17 - 1.11	173	176	98.3	40.33	22.47	114.87	0.0698	0.0090
1.11 - 1.07	131	132	99.2	37.89	17.73	104.13	0.0811	0.0104
1.07 - 1.03	169	171	98.8	35.75	15.09	102.32	0.0715	0.0204
1.03 - 1.00	138	140	98.6	33.86	11.88	92.12	0.0852	0.0103
1.00 - 0.96	200	201	99.5	30.22	9.10	75.75	0.0945	0.0203
0.96 - 0.94	132	132	100.0	30.39	7.63	76.51	0.0814	0.0131
0.94 - 0.92	129	130	99.2	29.82	5.92	69.02	0.0961	0.0126
0.92 - 0.90	143	143	100.0	27.36	5.54	65.18	0.0993	0.0154
0.90 - 0.87	222	226	98.2	22.86	5.16	60.11	0.1075	0.0163
0.87 - 0.86	102	102	100.0	8.00	3.74	20.80	0.1352	0.0420
0.86 - 0.84	171	172	99.4	6.40	4.20	20.48	0.1230	0.0417
0.84 - 0.83	111	111	100.0	6.10	3.95	19.70	0.1312	0.0423
0.83 - 0.81	158	174	90.8	3.93	4.05	17.86	0.1283	0.0533
0.91 - 0.81	850	871	97.6	12.31	4.46	34.51	0.1117	0.0331
Inf - 0.81	3040	3101	98.0	25.03	24.93	77.99	0.0573	0.0149

Complete .cif-data are available under the CCDC number CCDC-2338448.

The crystal was grown from the melt in a long glass capillary. The maximum crystal size was estimated based on the diffraction geometry. For data acquisition, a fixed chi angle of 54.7° was chosen to ensure that the exposed crystalline volume was as small as possible consistent with a high redundancy.
The final structure refinement was carried out with using aspherical scattering factors with NoSpherA2.[11] DFT-calculated with ORCA using a B3LYP functional and def2-TZVPP basis set, whereby the H atom positions were refined using isotropic atomic displacement parameters.
NoSpherA2 implementation of HAR makes use of tailor-made aspherical atomic form factors calculated on-the-fly from a Hirshfeld-partitioned electron density (ED) - not from spherical-atom form factors. The ED is calculated from a gaussian basis set single determinant SCF wave function - either Hartree-Fock or DFT using selected functional - for a fragment of the crystal. This fragment can be embedded in an electrostatic crystal field by employing cluster charges or modelled using implicit solvation models, depending on the software used. The following options were used:

SOFTWARE:	ORCA 5.0
PARTITIONING:	NoSpherA2
INT ACCURACY:	High
METHOD:	B3LYP
BASIS SET:	def2-TZVPP
CHARGE:	0
MULTIPLICITY:	1
DATE:	2024-02-27_21-06-15

Table S27 | Crystal data and structure refinement for (−)-9-*epi*-ambrox (**2b**)

Identification code	14073 / CCDC-2338448	
Empirical formula	$C_{16}H_{28}O$	
Colour	colorless	
Formula weight	236.400 g · mol^{-1}	
Temperature	100(2) K	
Wavelength	1.54178 Å	
Crystal system	orthorhombic	
Space group	$C222_1$, (No. 20)	
Unit cell dimensions	a = 12.529(3) Å	α = 90°.
	b = 14.555(3) Å	β = 90°.
	c = 31.280(7) Å	γ = 90°.
Volume	5704(2) Å^3	
Z	16	
Density (calculated)	1.101 Mg · m^{-3}	
Absorption coefficient	0.494 mm^{-1}	
F(000)	2117.750 e	
Crystal size	0.749 × 0.619 × 0.200 mm^3	
θ range for data collection	2.83 to 70.06°	
Index ranges	$-15 \le h \le 15$, $-17 \le k \le 17$, $-38 \le l \le 38$	
Reflections collected	77434	
Independent reflections	5252 [R_{int} = 0.0602]	
Reflections with $I > 2\sigma(I)$	5004	
Completeness to θ = 67.6786°	98.01 %	
Absorption correction	Gaussian	
Max. and min. transmission	0.90797 and 0.76318	
Refinement method	Full-matrix least-squares on F^2	
Data / restraints / parameters	5252 / 0 / 475	
Goodness-of-fit on F^2	1.0656	
Final R indices [$I > 2\sigma(I)$]	R_1 = 0.0347	wR_2 = 0.0767
R indices (all data)	R_1 = 0.0424	wR_2 = 0.0779
Absolute structure parameter	−0.04(4)	
Largest diff. peak and hole	0.2207 and −0.2322 e · Å^{-3}	

Table S28 | Bond lengths [Å] and angles [°] for (−)-9-*epi*-ambrox (**2b**).

O(1)-C(1)	1.408(2)	O(1)-C(15)	1.444(2)
C(1)-C(2)	1.522(3)	C(1)-H(1a)	1.07(3)
C(1)-H(1b)	1.06(3)	C(2)-C(3)	1.536(3)
C(2)-H(2a)	1.06(3)	C(2)-H(2b)	1.11(3)
C(3)-C(4)	1.557(2)	C(3)-H(3)	1.10(3)
C(3)-C(15)	1.545(2)	C(4)-C(5)	1.540(3)
C(4)-C(6)	1.541(2)	C(4)-C(12)	1.553(2)
C(5)-H(5a)	1.07(3)	C(5)-H(5b)	1.10(3)
C(5)-H(5c)	1.09(3)	C(6)-C(7)	1.528(3)
C(6)-H(6a)	1.12(3)	C(6)-H(6b)	1.09(3)
C(7)-C(8)	1.526(3)	C(7)-H(7a)	1.08(3)
C(7)-H(7b)	1.10(3)	C(8)-C(9)	1.540(3)
C(8)-H(8a)	1.09(3)	C(8)-H(8b)	1.12(3)
C(9)-C(10)	1.527(3)	C(9)-C(11)	1.532(3)
C(9)-C(12)	1.557(2)	C(10)-H(10a)	1.08(3)
C(10)-H(10b)	1.07(3)	C(10)-H(10c)	1.03(3)
C(11)-H(11a)	1.08(3)	C(11)-H(11b)	1.07(3)
C(11)-H(11c)	1.07(3)	H(12)-C(12)	1.14(3)
H(13a)-C(13)	1.09(3)	H(13b)-C(13)	1.11(3)
H(14a)-C(14)	1.06(3)	H(14b)-C(14)	1.11(3)
H(16a)-C(16)	1.12(3)	H(16b)-C(16)	1.11(3)
H(16c)-C(16)	1.08(3)	H(17a)-C(17)	1.11(3)
H(17b)-C(17)	1.10(3)	H(18a)-C(18)	1.08(3)
H(18b)-C(18)	1.06(3)	H(19)-C(19)	1.11(3)
H(21a)-C(21)	1.06(3)	H(21b)-C(21)	1.06(3)
H(21c)-C(21)	1.10(3)	H(22a)-C(22)	1.08(3)
H(22b)-C(22)	1.10(3)	H(23a)-C(23)	1.07(3)
H(23b)-C(23)	1.13(3)	H(24a)-C(24)	1.12(3)
H(24b)-C(24)	1.06(3)	H(26a)-C(26)	1.09(3)
H(26b)-C(26)	1.07(3)	H(26c)-C(26)	1.12(3)
H(27a)-C(27)	1.10(3)	H(27b)-C(27)	1.08(3)
H(27c)-C(27)	1.10(3)	H(28)-C(28)	1.11(3)
H(29a)-C(29)	1.07(3)	H(29b)-C(29)	1.07(3)
H(30a)-C(30)	1.11(3)	H(30b)-C(30)	1.09(3)
H(32a)-C(32)	1.07(3)	H(32b)-C(32)	1.12(3)
H(32c)-C(32)	1.05(3)	C(12)-C(13)	1.534(2)
C(13)-C(14)	1.528(3)	C(14)-C(15)	1.529(2)
C(15)-C(16)	1.518(2)	O(2)-C(17)	1.423(2)

O(2)-C(31)	1.443(2)	C(17)-C(18)	1.527(2)
C(18)-C(19)	1.543(2)	C(19)-C(20)	1.556(2)
C(19)-C(31)	1.547(2)	C(20)-C(21)	1.546(2)
C(20)-C(22)	1.542(2)	C(20)-C(28)	1.554(2)
C(22)-C(23)	1.529(2)	C(23)-C(24)	1.520(3)
C(24)-C(25)	1.542(3)	C(25)-C(26)	1.538(2)
C(25)-C(27)	1.538(3)	C(25)-C(28)	1.557(2)
C(28)-C(29)	1.535(2)	C(29)-C(30)	1.530(3)
C(30)-C(31)	1.538(3)	C(31)-C(32)	1.527(3)
C(15)-O(1)-C(1)	109.87(13)	C(2)-C(1)-O(1)	108.50(15)
H(1a)-C(1)-O(1)	105.8(14)	H(1a)-C(1)-C(2)	112.6(14)
H(1b)-C(1)-O(1)	115.3(14)	H(1b)-C(1)-C(2)	111.7(14)
H(1b)-C(1)-H(1a)	102.8(19)	C(3)-C(2)-C(1)	103.78(15)
H(2a)-C(2)-C(1)	113.6(14)	H(2a)-C(2)-C(3)	110.0(14)
H(2b)-C(2)-C(1)	113.5(13)	H(2b)-C(2)-C(3)	117.6(14)
H(2b)-C(2)-H(2a)	98.8(19)	C(4)-C(3)-C(2)	116.89(14)
H(3)-C(3)-C(2)	104.3(14)	H(3)-C(3)-C(4)	107.6(14)
C(15)-C(3)-C(2)	102.44(15)	C(15)-C(3)-C(4)	116.47(14)
C(15)-C(3)-H(3)	108.3(14)	C(5)-C(4)-C(3)	107.72(14)
C(6)-C(4)-C(3)	109.07(14)	C(6)-C(4)-C(5)	108.08(17)
C(12)-C(4)-C(3)	108.99(13)	C(12)-C(4)-C(5)	113.96(15)
C(12)-C(4)-C(6)	108.91(14)	H(5a)-C(5)-C(4)	113.5(15)
H(5b)-C(5)-C(4)	114.4(16)	H(5b)-C(5)-H(5a)	103(2)
H(5c)-C(5)-C(4)	110.4(16)	H(5c)-C(5)-H(5a)	108(2)
H(5c)-C(5)-H(5b)	107(2)	C(7)-C(6)-C(4)	113.48(17)
H(6a)-C(6)-C(4)	111.1(13)	H(6a)-C(6)-C(7)	110.6(14)
H(6b)-C(6)-C(4)	109.3(14)	H(6b)-C(6)-C(7)	108.1(14)
H(6b)-C(6)-H(6a)	103.8(19)	C(8)-C(7)-C(6)	110.44(18)
H(7a)-C(7)-C(6)	112.0(14)	H(7a)-C(7)-C(8)	109.6(14)
H(7b)-C(7)-C(6)	116.1(14)	H(7b)-C(7)-C(8)	106.2(14)
H(7b)-C(7)-H(7a)	102.0(19)	C(9)-C(8)-C(7)	113.31(17)
H(8a)-C(8)-C(7)	107.7(13)	H(8a)-C(8)-C(9)	108.9(13)
H(8b)-C(8)-C(7)	111.9(13)	H(8b)-C(8)-C(9)	109.3(13)
H(8b)-C(8)-H(8a)	105.4(17)	C(10)-C(9)-C(8)	110.21(17)
C(11)-C(9)-C(8)	106.31(17)	C(11)-C(9)-C(10)	107.79(19)
C(12)-C(9)-C(8)	108.14(14)	C(12)-C(9)-C(10)	114.67(15)
C(12)-C(9)-C(11)	109.40(15)	H(10a)-C(10)-C(9)	112.9(16)
H(10b)-C(10)-C(9)	115.8(16)	H(10b)-C(10)-H(10a)	101(2)

H(10c)-C(10)-C(9)	111.7(16)	H(10c)-C(10)-H(10a)	104(2)
H(10c)-C(10)-H(10b)	110(2)	H(11a)-C(11)-C(9)	114.6(16)
H(11b)-C(11)-C(9)	109.6(17)	H(11b)-C(11)-H(11a)	108(2)
H(11c)-C(11)-C(9)	110.0(15)	H(11c)-C(11)-H(11a)	110(2)
H(11c)-C(11)-H(11b)	103(2)	C(9)-C(12)-C(4)	115.87(14)
H(12)-C(12)-C(4)	102.9(13)	H(12)-C(12)-C(9)	103.0(13)
C(13)-C(12)-C(4)	110.42(13)	C(13)-C(12)-C(9)	115.24(15)
C(13)-C(12)-H(12)	108.1(13)	H(13b)-C(13)-H(13a)	104.0(19)
C(12)-C(13)-H(13a)	110.4(14)	C(12)-C(13)-H(13b)	113.1(14)
C(14)-C(13)-H(13a)	110.1(14)	C(14)-C(13)-H(13b)	109.1(13)
C(14)-C(13)-C(12)	110.02(16)	H(14b)-C(14)-H(14a)	105.9(18)
C(13)-C(14)-H(14a)	111.2(14)	C(13)-C(14)-H(14b)	108.1(13)
C(15)-C(14)-H(14a)	108.9(14)	C(15)-C(14)-H(14b)	109.1(13)
C(15)-C(14)-C(13)	113.37(15)	C(3)-C(15)-O(1)	103.80(13)
C(14)-C(15)-O(1)	108.97(14)	C(14)-C(15)-C(3)	110.79(14)
C(16)-C(15)-O(1)	104.93(14)	C(16)-C(15)-C(3)	115.41(15)
C(16)-C(15)-C(14)	112.25(16)	H(16b)-C(16)-H(16a)	111(2)
H(16c)-C(16)-H(16a)	105(2)	H(16c)-C(16)-H(16b)	110(2)
C(15)-C(16)-H(16a)	109.7(15)	C(15)-C(16)-H(16b)	108.8(15)
C(15)-C(16)-H(16c)	112.5(15)	C(31)-O(2)-C(17)	110.09(13)
H(17b)-C(17)-H(17a)	107.1(18)	O(2)-C(17)-H(17a)	107.6(13)
O(2)-C(17)-H(17b)	110.2(13)	C(18)-C(17)-H(17a)	114.7(13)
C(18)-C(17)-H(17b)	109.8(14)	C(18)-C(17)-O(2)	107.39(14)
H(18b)-C(18)-H(18a)	102.7(19)	C(17)-C(18)-H(18a)	111.0(13)
C(17)-C(18)-H(18b)	112.7(14)	C(19)-C(18)-H(18a)	113.9(13)
C(19)-C(18)-H(18b)	113.3(14)	C(19)-C(18)-C(17)	103.63(14)
C(18)-C(19)-H(19)	109.4(14)	C(20)-C(19)-H(19)	105.8(14)
C(20)-C(19)-C(18)	116.95(14)	C(31)-C(19)-H(19)	106.7(14)
C(31)-C(19)-C(18)	101.33(13)	C(31)-C(19)-C(20)	116.17(14)
C(21)-C(20)-C(19)	108.15(14)	C(22)-C(20)-C(19)	108.60(14)
C(22)-C(20)-C(21)	108.03(15)	C(28)-C(20)-C(19)	108.83(13)
C(28)-C(20)-C(21)	113.92(14)	C(28)-C(20)-C(22)	109.18(13)
H(21b)-C(21)-H(21a)	105(2)	H(21c)-C(21)-H(21a)	109(2)
H(21c)-C(21)-H(21b)	106(2)	C(20)-C(21)-H(21a)	113.8(15)
C(20)-C(21)-H(21b)	108.7(16)	C(20)-C(21)-H(21c)	113.7(15)
H(22b)-C(22)-H(22a)	105.7(19)	C(20)-C(22)-H(22a)	109.8(14)
C(20)-C(22)-H(22b)	110.9(13)	C(23)-C(22)-H(22a)	110.3(14)
C(23)-C(22)-H(22b)	107.0(14)	C(23)-C(22)-C(20)	112.90(15)
H(23b)-C(23)-H(23a)	106.6(19)	C(22)-C(23)-H(23a)	110.3(15)

C(22)-C(23)-H(23b)	107.5(14)	C(24)-C(23)-H(23a)	112.3(14)
C(24)-C(23)-H(23b)	109.5(13)	C(24)-C(23)-C(22)	110.43(15)
H(24b)-C(24)-H(24a)	105(2)	C(23)-C(24)-H(24a)	109.9(13)
C(23)-C(24)-H(24b)	111.9(14)	C(25)-C(24)-H(24a)	106.9(13)
C(25)-C(24)-H(24b)	108.0(14)	C(25)-C(24)-C(23)	114.52(15)
C(26)-C(25)-C(24)	106.45(16)	C(27)-C(25)-C(24)	110.48(16)
C(27)-C(25)-C(26)	107.06(16)	C(28)-C(25)-C(24)	108.16(14)
C(28)-C(25)-C(26)	109.52(15)	C(28)-C(25)-C(27)	114.85(16)
H(26b)-C(26)-H(26a)	104(2)	H(26c)-C(26)-H(26a)	105(2)
H(26c)-C(26)-H(26b)	114(2)	C(25)-C(26)-H(26a)	112.6(15)
C(25)-C(26)-H(26b)	111.7(16)	C(25)-C(26)-H(26c)	109.4(15)
H(27b)-C(27)-H(27a)	106(2)	H(27c)-C(27)-H(27a)	116(2)
H(27c)-C(27)-H(27b)	103(2)	C(25)-C(27)-H(27a)	108.0(15)
C(25)-C(27)-H(27b)	111.7(16)	C(25)-C(27)-H(27c)	111.7(15)
C(20)-C(28)-H(28)	104.7(13)	C(25)-C(28)-H(28)	103.1(12)
C(25)-C(28)-C(20)	115.70(13)	C(29)-C(28)-H(28)	105.5(13)
C(29)-C(28)-C(20)	111.14(14)	C(29)-C(28)-C(25)	115.10(14)
H(29b)-C(29)-H(29a)	102.8(19)	C(28)-C(29)-H(29a)	112.9(14)
C(28)-C(29)-H(29b)	110.6(13)	C(30)-C(29)-H(29a)	108.8(14)
C(30)-C(29)-H(29b)	111.1(14)	C(30)-C(29)-C(28)	110.47(15)
H(30b)-C(30)-H(30a)	105.2(19)	C(29)-C(30)-H(30a)	108.2(14)
C(29)-C(30)-H(30b)	112.1(14)	C(31)-C(30)-H(30a)	110.9(14)
C(31)-C(30)-H(30b)	106.9(13)	C(31)-C(30)-C(29)	113.14(16)
C(19)-C(31)-O(2)	103.40(14)	C(30)-C(31)-O(2)	109.73(16)
C(30)-C(31)-C(19)	110.67(14)	C(32)-C(31)-O(2)	105.18(15)
C(32)-C(31)-C(19)	115.67(18)	C(32)-C(31)-C(30)	111.60(18)
H(32b)-C(32)-H(32a)	111(2)	H(32c)-C(32)-H(32a)	104(2)
H(32c)-C(32)-H(32b)	108(2)	C(31)-C(32)-H(32a)	111.5(17)
C(31)-C(32)-H(32b)	111.4(15)	C(31)-C(32)-H(32c)	110.2(16)

Crystallographic data for (−)-9-*epi*-sclareolide (12b)

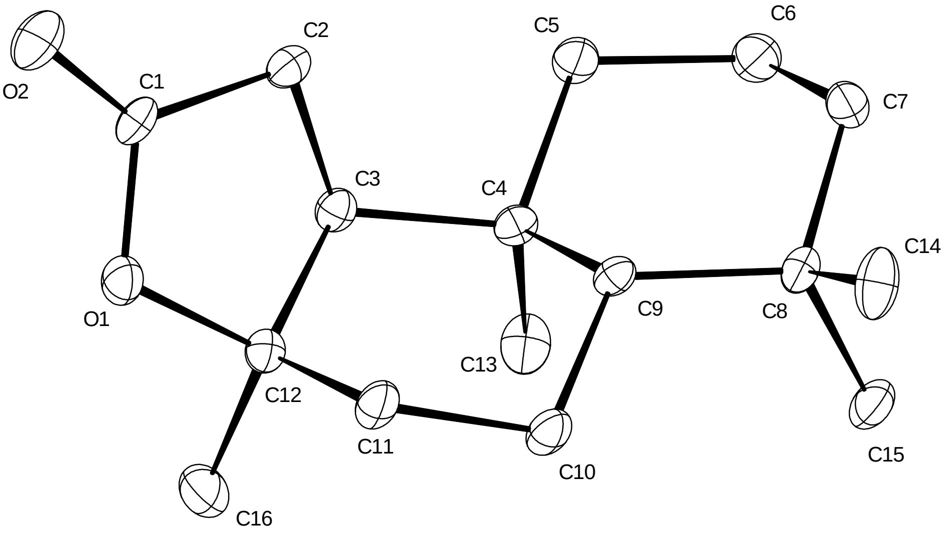

Fig. S86 | The molecular structure of (−)-9-*epi*-sclareolide (**12b**). Hydrogen atoms have been removed for clarity.

X-ray Crystal Structure Analysis of (−)-9-epi-sclareolide (**12b**):

$C_{16}H_{26}O_2$, M_r = 250.384 g · mol^{-1}, colorless block, crystal size 0.156 × 0.151 × 0.143 mm^3, monoclinic, space group $P2_1$ [4], a = 7.1888(3) Å, b = 10.8360(5) Å, c = 9.5651(4) Å, β = 110.064(2), V = 699.88(5) Å^3, T = 100(2) K, Z = 2, D_{calc} = 1.188 g · cm^3, λ = 1.54178 Å, μ(Cu-K$_\alpha$) = 0.590 mm^{-1}, Numerical correction ($T_{\min}$ = 0.93139, $T_{\max}$ = 0.97004), Bruker-AXS Kappa Mach3 with APEX-II detector and FR591 rotating anode X-ray source with Incoatec Helios mirrors, 4.92 < θ< 71.94°, 25589 measured reflections, 2601 independent reflections, 2376 reflections with $I > 2\sigma(I)$, R_{int} = 0.0446. The structure was solved by *SHELXT* and refined by full-matrix least-squares (*SHELXL*). The final structure refinement was performed by olex2.refine 1.5 (L-M) together with NoSpherA2 (atomic form factors) against F^2 to R_1 = 0.0292 [$I > 2\sigma(I)$], wR_2 = 0.0641 [all data] with 243 parameters, 1 restraints and an absolute structure parameter x = 0.02(7).

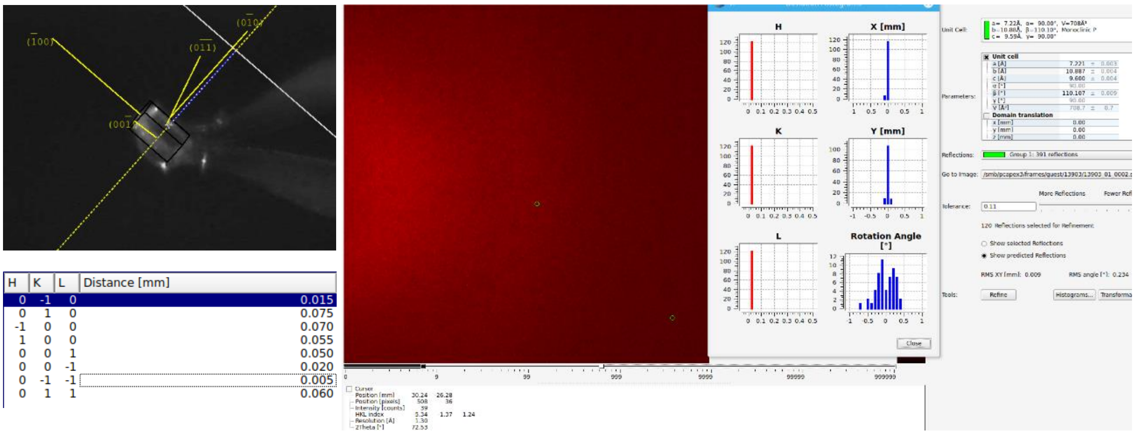

Fig. S87 | Crystal faces and unit cell determination/refinement of (−)-9-*epi*-sclareolide (**12b**).

INTENSITY STATISTICS FOR DATASET

Resolution	#Data	#Theory	%Complete	Redundancy	Mean I	Mean I/s	Rmerge	Rsigma
Inf - 3.37	40	40	100.0	9.05	240.75	84.70	0.0289	0.0103
3.37 - 2.25	91	91	100.0	9.05	93.44	76.91	0.0244	0.0105
2.25 - 1.78	131	131	100.0	10.17	72.15	73.00	0.0301	0.0114
1.78 - 1.56	137	137	100.0	10.01	39.00	62.13	0.0314	0.0132
1.56 - 1.41	124	124	100.0	9.07	24.87	48.26	0.0336	0.0163
1.41 - 1.31	141	141	100.0	7.71	24.23	41.04	0.0362	0.0191
1.31 - 1.23	129	129	100.0	9.69	26.04	42.46	0.0430	0.0204
1.23 - 1.17	125	125	100.0	15.36	27.99	50.41	0.0438	0.0158
1.17 - 1.12	140	140	100.0	14.31	20.79	43.36	0.0475	0.0200
1.12 - 1.08	114	114	100.0	13.89	23.04	45.12	0.0437	0.0191
1.08 - 1.04	140	140	100.0	13.30	15.71	35.73	0.0549	0.0241
1.04 - 1.00	160	161	99.4	13.27	12.07	31.62	0.0812	0.0282
1.00 - 0.97	130	145	89.7	10.41	12.31	25.46	0.0795	0.0345
0.97 - 0.95	94	101	93.1	11.38	9.84	23.50	0.1075	0.0365
0.95 - 0.92	167	180	92.8	10.12	8.21	21.29	0.1107	0.0415
0.92 - 0.90	103	111	92.8	10.17	8.00	21.21	0.1149	0.0401
0.90 - 0.88	143	153	93.5	9.97	6.00	19.16	0.1272	0.0490
0.88 - 0.86	139	155	89.7	5.73	4.73	10.40	0.2077	0.1325
0.86 - 0.84	160	178	89.9	1.97	5.03	4.12	0.2060	0.2485
0.84 - 0.83	75	87	86.2	2.02	4.85	4.49	0.1815	0.2055
0.83 - 0.81	131	171	76.6	1.54	3.76	3.76	0.1894	0.2746
0.91 - 0.81	702	802	87.5	4.71	5.06	9.61	0.1544	0.1564
Inf - 0.81	2614	2754	94.9	9.32	24.43	34.51	0.0445	0.0241

Complete .cif-data are available under the CCDC number CCDC-2338450.

The final structure refinement was carried out with using aspherical scattering factors with NoSpherA2.[11] DFT-calculated with ORCA using a B3LYP functional and def2-TZVPP basis set, whereby the H atom positions were refined using isotropic atomic displacement parameters.

NoSpherA2 implementation of HAR makes use of tailor-made aspherical atomic form factors calculated on-the-fly from a Hirshfeld-partitioned electron density (ED) - not from spherical-atom form factors. The ED is calculated from a gaussian basis set single determinant SCF wave function - either Hartree-Fock or DFT using selected functional - for a fragment of the crystal. This fragment can be embedded in an electrostatic crystal field by employing cluster charges or modelled using implicit solvation models, depending on the software used. The following options were used:

SOFTWARE:	ORCA 5.0
PARTITIONING:	NoSpherA2
INT ACCURACY:	High
METHOD:	B3LYP
BASIS SET:	def2-TZVPP
CHARGE:	0
MULTIPLICITY:	1
DATE:	2024-02-27_21-02-49

Table S29 | Crystal data and structure refinement for (−)-9-*epi*-sclareolide (**12b**).

Identification code	13903 / CCDC-2338450	
Empirical formula	$C_{16}H_{26}O_2$	
Colour	colorless	
Formula weight	250.384 g · mol^{-1}	
Temperature	100(2) K	
Wavelength	1.54178 Å	
Crystal system	monoclinic	
Space group	$P2_1$, (No. 4)	
Unit cell dimensions	a = 7.1888(3) Å	α = 90°.
	b = 10.8360(5) Å	β = 110.064(2)°.
	c = 9.5651(4) Å	γ = 90°.
Volume	699.88(5) Å^3	
Z	2	
Density (calculated)	1.118 Mg · m^{-3}	
Absorption coefficient	0.590 mm^{-1}	
F(000)	276.824 e	
Crystal size	0.156 × 0.151 × 0.143 mm^3	
θ range for data collection	4.92 to 71.94°.	
Index ranges	$-8 \le h \le 8$, $-13 \le k \le 13$, $-11 \le l \le 11$	
Reflections collected	25589	
Independent reflections	2601 [R_{int} = 0.0446]	
Reflections with $I > 2\sigma(I)$	2376	
Completeness to θ = 67.6786°	99.93 %	
Absorption correction	Gaussian	
Max. and min. transmission	0.97004 and 0.93139	
Refinement method	Full-matrix least-squares on F^2	
Data / restraints / parameters	2601 / 1 / 243	
Goodness-of-fit on F^2	1.0422	
Final R indices [$I > 2\sigma(I)$]	R_1 = 0.0292	wR_2 = 0.0570
R indices (all data)	R_1 = 0.0366	wR_2 = 0.0641
Absolute structure parameter	0.02(7)	
Largest diff. peak and hole	0.1670 and −0.1952 e · Å^{-3}	

Table 30 | Bond lengths [Å] and angles [°] for (−)-9-*epi*-sclareolide (**12b**).

O(1)-C(1)	1.341(2)	O(1)-C(12)	1.4712(19)
O(2)-C(1)	1.210(2)	C(1)-C(2)	1.503(2)
C(2)-H(2a)	1.129(19)	C(2)-H(2b)	1.069(18)
C(2)-C(3)	1.528(2)	C(3)-H(3)	1.10(2)
C(3)-C(4)	1.559(2)	C(3)-C(12)	1.549(2)
C(4)-C(5)	1.540(2)	C(4)-C(9)	1.553(2)
C(4)-C(13)	1.543(2)	C(5)-H(5a)	1.079(18)
C(5)-H(5b)	1.130(19)	C(5)-C(6)	1.528(2)
C(6)-H(6a)	1.09(2)	C(6)-H(6b)	1.091(18)
C(6)-C(7)	1.527(3)	C(7)-H(7a)	1.124(19)
C(7)-H(7b)	1.123(18)	C(7)-C(8)	1.541(2)
C(8)-C(9)	1.557(2)	C(8)-C(14)	1.538(2)
C(8)-C(15)	1.537(2)	C(9)-H(9)	1.121(19)
C(9)-C(10)	1.534(2)	C(10)-H(10a)	1.093(19)
C(10)-H(10b)	1.096(18)	C(10)-C(11)	1.530(2)
C(11)-H(11a)	1.093(18)	C(11)-H(11b)	1.13(2)
C(11)-C(12)	1.526(2)	C(12)-C(16)	1.513(2)
C(13)-H(13a)	1.11(2)	C(13)-H(13b)	1.11(2)
C(13)-H(13c)	1.08(2)	C(14)-H(14a)	1.10(2)
C(14)-H(14b)	1.13(2)	C(14)-H(14c)	1.11(2)
C(15)-H(15a)	1.07(2)	C(15)-H(15b)	1.142(19)
C(15)-H(15c)	1.069(18)	C(16)-H(16a)	1.10(2)
C(16)-H(16b)	1.07(2)	C(16)-H(16c)	1.08(2)
C(12)-O(1)-C(1)	110.79(12)	O(2)-C(1)-O(1)	121.54(15)
C(2)-C(1)-O(1)	110.06(13)	C(2)-C(1)-O(2)	128.40(15)
H(2a)-C(2)-C(1)	110.4(10)	H(2b)-C(2)-C(1)	110.4(9)
H(2b)-C(2)-H(2a)	103.2(13)	C(3)-C(2)-C(1)	103.70(13)
C(3)-C(2)-H(2a)	114.0(9)	C(3)-C(2)-H(2b)	115.3(10)
H(3)-C(3)-C(2)	106.8(11)	C(4)-C(3)-C(2)	117.29(13)
C(4)-C(3)-H(3)	107.2(11)	C(12)-C(3)-C(2)	101.43(13)
C(12)-C(3)-H(3)	107.4(11)	C(12)-C(3)-C(4)	116.05(13)
C(5)-C(4)-C(3)	108.56(13)	C(9)-C(4)-C(3)	109.11(13)
C(9)-C(4)-C(5)	108.96(13)	C(13)-C(4)-C(3)	107.44(13)
C(13)-C(4)-C(5)	108.52(14)	C(13)-C(4)-C(9)	114.12(13)
H(5a)-C(5)-C(4)	109.1(9)	H(5b)-C(5)-C(4)	109.2(9)
H(5b)-C(5)-H(5a)	106.6(13)	C(6)-C(5)-C(4)	112.41(14)
C(6)-C(5)-H(5a)	110.8(9)	C(6)-C(5)-H(5b)	108.6(9)

H(6a)-C(6)-C(5)	110.9(10)	H(6b)-C(6)-C(5)	108.9(10)
H(6b)-C(6)-H(6a)	105.0(13)	C(7)-C(6)-C(5)	110.70(14)
C(7)-C(6)-H(6a)	110.2(10)	C(7)-C(6)-H(6b)	111.0(10)
H(7a)-C(7)-C(6)	107.7(10)	H(7b)-C(7)-C(6)	109.4(9)
H(7b)-C(7)-H(7a)	105.6(13)	C(8)-C(7)-C(6)	113.97(14)
C(8)-C(7)-H(7a)	108.7(10)	C(8)-C(7)-H(7b)	111.1(9)
C(9)-C(8)-C(7)	108.44(13)	C(14)-C(8)-C(7)	107.07(14)
C(14)-C(8)-C(9)	109.07(13)	C(15)-C(8)-C(7)	110.44(14)
C(15)-C(8)-C(9)	114.31(14)	C(15)-C(8)-C(14)	107.26(14)
C(8)-C(9)-C(4)	116.42(13)	H(9)-C(9)-C(4)	106.0(10)
H(9)-C(9)-C(8)	101.7(10)	C(10)-C(9)-C(4)	110.97(13)
C(10)-C(9)-C(8)	114.88(13)	C(10)-C(9)-H(9)	105.3(10)
H(10a)-C(10)-C(9)	112.1(9)	H(10b)-C(10)-C(9)	111.8(9)
H(10b)-C(10)-H(10a)	101.4(13)	C(11)-C(10)-C(9)	109.51(13)
C(11)-C(10)-H(10a)	111.9(9)	C(11)-C(10)-H(10b)	109.9(10)
H(11a)-C(11)-C(10)	110.8(10)	H(11b)-C(11)-C(10)	107.4(9)
H(11b)-C(11)-H(11a)	107.7(13)	C(12)-C(11)-C(10)	112.50(14)
C(12)-C(11)-H(11a)	109.0(9)	C(12)-C(11)-H(11b)	109.3(9)
C(3)-C(12)-O(1)	103.27(12)	C(11)-C(12)-O(1)	107.35(13)
C(11)-C(12)-C(3)	112.14(13)	C(16)-C(12)-O(1)	105.52(13)
C(16)-C(12)-C(3)	115.44(15)	C(16)-C(12)-C(11)	112.14(15)
H(13a)-C(13)-C(4)	115.3(12)	H(13b)-C(13)-C(4)	112.7(10)
H(13b)-C(13)-H(13a)	105.7(15)	H(13c)-C(13)-C(4)	109.3(11)
H(13c)-C(13)-H(13a)	106.3(15)	H(13c)-C(13)-H(13b)	107.2(15)
H(14a)-C(14)-C(8)	109.7(11)	H(14b)-C(14)-C(8)	113.2(11)
H(14b)-C(14)-H(14a)	106.6(15)	H(14c)-C(14)-C(8)	112.5(11)
H(14c)-C(14)-H(14a)	105.3(15)	H(14c)-C(14)-H(14b)	109.1(16)
H(15a)-C(15)-C(8)	114.2(10)	H(15b)-C(15)-C(8)	114.8(10)
H(15b)-C(15)-H(15a)	103.7(14)	H(15c)-C(15)-C(8)	109.8(10)
H(15c)-C(15)-H(15a)	106.4(14)	H(15c)-C(15)-H(15b)	107.3(13)
H(16a)-C(16)-C(12)	109.9(11)	H(16b)-C(16)-C(12)	111.5(11)
H(16b)-C(16)-H(16a)	108.5(16)	H(16c)-C(16)-C(12)	112.0(12)
H(16c)-C(16)-H(16a)	108.5(16)	H(16c)-C(16)-H(16b)	106.4(17)

Crystallographic data for (−)-8-*epi*-sclareolide (12f)

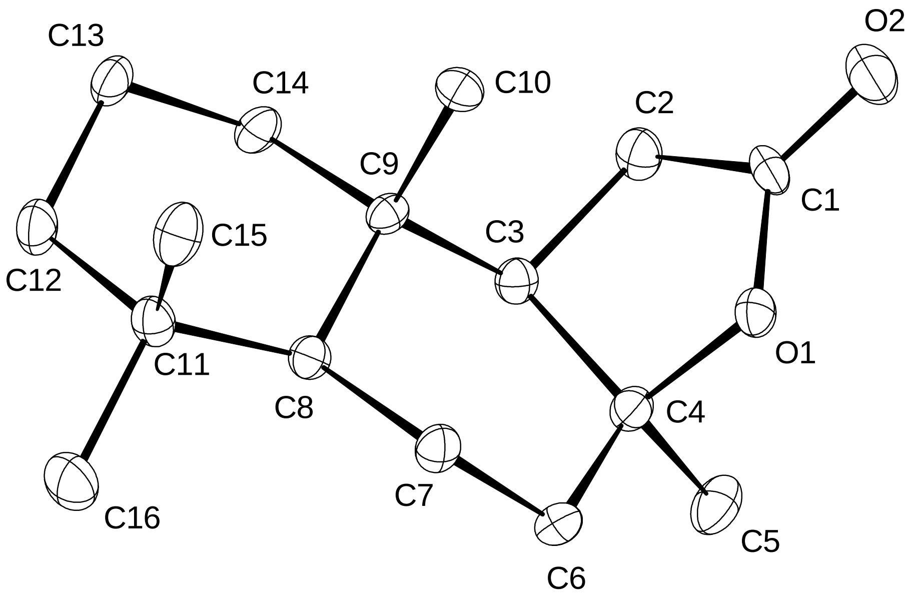

Fig. S88 | The molecular structure of (−)-8-*epi*-sclareolide (**12f**). H atoms have been removed for clarity.

X-ray Crystal Structure Analysis of (−)-8-epi-sclareolide (**12f**):

$C_{16}H_{26}O_2$, M_r = 250.384 g · mol^{-1}, colorless needle, crystal size 0.556 × 0.362 × 0.326 mm^3, orthorhombic, space group $P2_12_12_1$ [19], a = 6.1260(2) Å, b = 11.3566(4) Å, c = 19.8294(7) Å, V = 1379.54(8) Å^3, T = 100(2) K, Z = 4, D_{calc} = 1.206 g · cm^3, λ = 1.54178 Å, μ(Cu-K$_\alpha$) = 0.598 mm^{-1}, Numerical correction ($T_{\min}$ = 0.80067, $T_{\max}$ = 0.88373), Bruker-AXS Kappa Mach3 with APEX-II detector and FR591 rotating anode X-ray source with Incoatec Helios mirrors, 4.46 < θ < 72.52°, 47957 measured reflections, 2697 independent reflections, 2672 reflections with $I > 2\sigma(I)$, R_{int} = 0.0318. The structure was solved by *SHELXT* and refined by full-matrix least-squares (*SHELXL*). The final structure refinement was performed by olex2.refine 1.5 (L-M) together with NoSpherA2 (atomic form factors) against F^2 to R_1 = 0.0349 [$I > 2\sigma(I)$], wR_2 = 0.0833 [all data] with 241 parameters, 0 restraints and an absolute structure parameter x = 0.01(2).

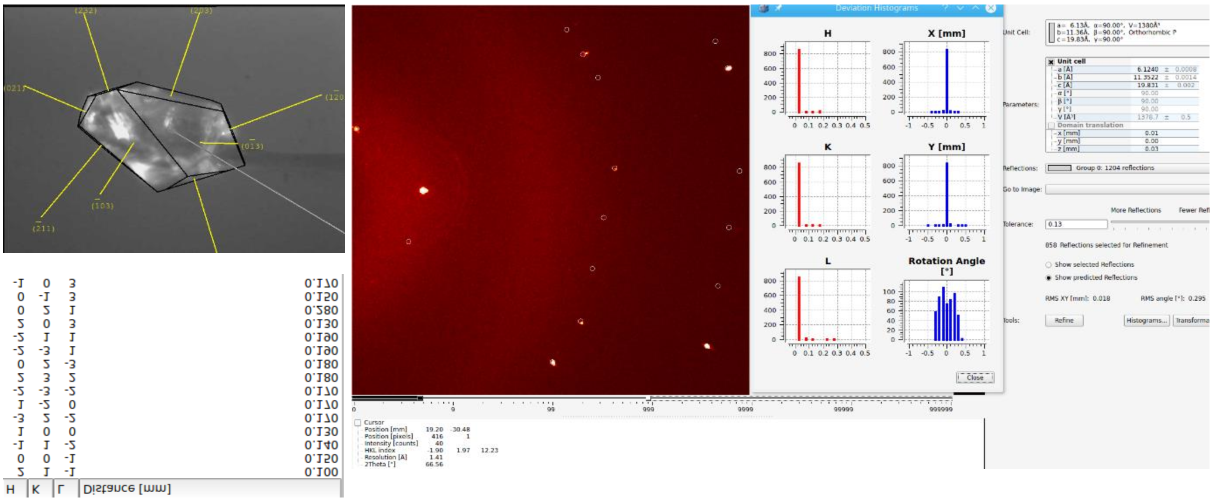

Fig. S89 | Unit cell determination/refinement of (−)-8-*epi*-sclareolide (**12f**).

INTENSITY STATISTICS FOR DATASET

Resolution	#Data	#Theory	%Complete	Redundancy	Mean I	Mean I/s	Rmerge	Rsigma
Inf - 3.18	41	49	83.7	12.76	194.43	91.89	0.0375	0.0135
3.18 - 2.20	95	96	99.0	17.15	147.40	102.29	0.0309	0.0103
2.20 - 1.76	138	138	100.0	20.79	99.73	114.32	0.0385	0.0080
1.76 - 1.52	136	136	100.0	18.85	59.43	103.15	0.0310	0.0084
1.52 - 1.38	140	140	100.0	17.89	27.63	89.13	0.0337	0.0094
1.38 - 1.28	134	134	100.0	13.66	51.88	86.59	0.0388	0.0105
1.28 - 1.21	133	133	100.0	19.88	36.81	100.95	0.0324	0.0092
1.21 - 1.15	143	143	100.0	30.86	40.04	137.39	0.0262	0.0066
1.15 - 1.10	139	139	100.0	28.01	31.95	129.36	0.0240	0.0072
1.10 - 1.06	127	127	100.0	28.15	27.99	130.87	0.0238	0.0072
1.06 - 1.02	140	140	100.0	24.53	24.93	114.68	0.0277	0.0075
1.02 - 0.99	126	126	100.0	22.88	17.31	103.05	0.0286	0.0085
0.99 - 0.96	153	153	100.0	21.29	18.83	102.98	0.0294	0.0089
0.96 - 0.93	163	163	100.0	20.42	14.34	98.74	0.0297	0.0096
0.93 - 0.91	121	121	100.0	17.85	13.37	95.55	0.0291	0.0101
0.91 - 0.89	133	133	100.0	18.01	10.25	86.91	0.0313	0.0103
0.89 - 0.87	131	131	100.0	16.63	8.06	81.71	0.0352	0.0107
0.87 - 0.85	178	180	98.9	5.59	8.31	30.12	0.0648	0.0405
0.85 - 0.84	77	77	100.0	3.10	8.33	15.50	0.0682	0.0533
0.84 - 0.82	187	192	97.4	2.69	6.97	14.94	0.0625	0.0588
0.82 - 0.81	85	118	72.0	1.28	6.49	11.07	0.0677	0.0677
0.91 - 0.81	791	831	95.2	7.80	8.08	41.15	0.0414	0.0365
Inf - 0.81	2720	2769	98.2	17.37	33.88	88.25	0.0318	0.0111

Complete .cif-data are available under the CCDC number CCDC-2338447.

The final structure refinement was carried out with using aspherical scattering factors with NoSpherA2.[11] DFT-calculated with ORCA using a B3LYP functional and def2-TZVPP basis set, whereby the H atom positions were refined using isotropic atomic displacement parameters.

NoSpherA2 implementation of HAR makes use of tailor-made aspherical atomic form factors calculated on-the-fly from a Hirshfeld-partitioned electron density (ED) - not from spherical-atom form factors. The ED is calculated from a gaussian basis set single determinant SCF wave function - either Hartree-Fock or DFT using selected functional - for a fragment of the crystal. This fragment can be embedded in an electrostatic crystal field by employing cluster charges or modelled using implicit solvation models, depending on the software used. The following options were used:

SOFTWARE:	ORCA 5.0
PARTITIONING:	NoSpherA2
INT ACCURACY:	High
METHOD:	B3LYP
BASIS SET:	def2-TZVPP
CHARGE:	0
MULTIPLICITY:	1
DATE:	2024-02-27_20-46-16

Table S30 | Crystal data and structure refinement for (−)-8-*epi*-sclareolide (**12f**).

Identification code	12416 / CCDC-2338447	
Empirical formula	$C_{16}H_{26}O_2$	
Colour	colorless	
Formula weight	250.384 g · mol^{-1}	
Temperature	100(2) K	
Wavelength	1.54178 Å	
Crystal system	orthorhombic	
Space group	$P2_12_12_1$, (No. 19)	
Unit cell dimensions	a = 6.1260(2) Å	α = 90°.
	b = 11.3566(4) Å	β = 90°.
	c = 19.8294(7) Å	γ = 90°.
Volume	1379.54(8) Å^3	
Z	4	
Density (calculated)	1.206 Mg · m^{-3}	
Absorption coefficient	0.598 mm^{-1}	
F(000)	553.647 e	
Crystal size	0.556 × 0.362 × 0.326 mm^3	
θ range for data collection	4.46 to 72.52°.	
Index ranges	$-7 \le h \le 7$, $-12 \le k \le 13$, $-24 \le l \le 24$	
Reflections collected	47957	
Independent reflections	2697 [R_{int} = 0.0318]	
Reflections with $I > 2\sigma(I)$	2672	
Completeness to θ = 67.6786°	99.52 %	
Absorption correction	Gaussian	
Max. and min. transmission	0.88373 and 0.80067	
Refinement method	Full-matrix least-squares on F^2	
Data / restraints / parameters	2697 / 0 / 241	
Goodness-of-fit on F^2	1.0264	
Final R indices [$I > 2\sigma(I)$]	R_1 = 0.0349	wR_2 = 0.0819
R indices (all data)	R_1 = 0.0356	wR_2 = 0.0833
Absolute structure parameter	0.01(2)	
Largest diff. peak and hole	0.1588 and −0.2003 e · Å^{-3}	

Table S31 | Bond lengths [Å] and angles [°] for (−)-8-*epi*-sclareolide (**12f**).

O(1)-C(1)	1.351(2)	O(1)-C(4)	1.471(2)
O(2)-C(1)	1.207(2)	C(1)-C(2)	1.517(3)
C(2)-H(2a)	1.11(2)	C(2)-H(2b)	1.12(2)
C(2)-C(3)	1.539(2)	C(3)-H(3)	1.12(2)
C(3)-C(4)	1.543(2)	C(3)-C(9)	1.567(2)
C(4)-C(5)	1.528(3)	C(4)-C(6)	1.522(2)
C(5)-H(5a)	1.10(2)	C(5)-H(5b)	1.14(2)
C(5)-H(5c)	1.07(2)	C(6)-H(6a)	1.12(2)
C(6)-H(6b)	1.10(2)	C(6)-C(7)	1.523(3)
C(7)-H(7a)	1.13(2)	C(7)-H(7b)	1.085(19)
C(7)-C(8)	1.535(2)	C(8)-H(8)	1.12(2)
C(8)-C(9)	1.558(2)	C(8)-C(11)	1.560(2)
C(9)-C(10)	1.541(2)	C(9)-C(14)	1.543(2)
C(10)-H(10a)	1.12(2)	C(10)-H(10b)	1.14(2)
C(10)-H(10c)	1.10(2)	C(11)-C(12)	1.537(2)
C(11)-C(15)	1.541(3)	C(11)-C(16)	1.536(3)
C(12)-H(12a)	1.16(2)	C(12)-H(12b)	1.12(2)
C(12)-C(13)	1.530(2)	C(13)-H(13a)	1.15(2)
C(13)-H(13b)	1.10(2)	C(13)-C(14)	1.528(2)
C(14)-H(14a)	1.13(2)	C(14)-H(14b)	1.10(2)
C(15)-H(15a)	1.11(2)	C(15)-H(15b)	1.11(2)
C(15)-H(15c)	1.09(2)	C(16)-H(16a)	1.09(3)
C(16)-H(16b)	1.11(2)	C(16)-H(16c)	1.13(2)
C(4)-O(1)-C(1)	109.32(13)	O(2)-C(1)-O(1)	122.06(17)
C(2)-C(1)-O(1)	109.71(14)	C(2)-C(1)-O(2)	128.22(17)
H(2a)-C(2)-C(1)	113.2(11)	H(2b)-C(2)-C(1)	109.1(11)
H(2b)-C(2)-H(2a)	104.5(15)	C(3)-C(2)-C(1)	102.53(14)
C(3)-C(2)-H(2a)	115.4(10)	C(3)-C(2)-H(2b)	112.1(10)
H(3)-C(3)-C(2)	110.9(10)	C(4)-C(3)-C(2)	99.72(13)
C(4)-C(3)-H(3)	110.3(10)	C(9)-C(3)-C(2)	113.02(15)
C(9)-C(3)-H(3)	106.3(10)	C(9)-C(3)-C(4)	116.60(14)
C(3)-C(4)-O(1)	103.06(13)	C(5)-C(4)-O(1)	106.62(14)
C(5)-C(4)-C(3)	112.96(15)	C(6)-C(4)-O(1)	109.01(14)
C(6)-C(4)-C(3)	115.48(14)	C(6)-C(4)-C(5)	109.12(16)
H(5a)-C(5)-C(4)	112.6(13)	H(5b)-C(5)-C(4)	109.7(12)
H(5b)-C(5)-H(5a)	106.5(17)	H(5c)-C(5)-C(4)	111.5(12)
H(5c)-C(5)-H(5a)	106.8(17)	H(5c)-C(5)-H(5b)	109.5(17)

H(6a)-C(6)-C(4)	109.3(10)	H(6b)-C(6)-C(4)	105.1(10)
H(6b)-C(6)-H(6a)	105.5(15)	C(7)-C(6)-C(4)	114.70(15)
C(7)-C(6)-H(6a)	111.6(11)	C(7)-C(6)-H(6b)	110.0(10)
H(7a)-C(7)-C(6)	111.1(10)	H(7b)-C(7)-C(6)	109.2(11)
H(7b)-C(7)-H(7a)	102.8(15)	C(8)-C(7)-C(6)	110.10(15)
C(8)-C(7)-H(7a)	111.3(10)	C(8)-C(7)-H(7b)	112.2(10)
H(8)-C(8)-C(7)	107.5(10)	C(9)-C(8)-C(7)	109.74(13)
C(9)-C(8)-H(8)	103.1(10)	C(11)-C(8)-C(7)	114.48(15)
C(11)-C(8)-H(8)	104.4(10)	C(11)-C(8)-C(9)	116.42(14)
C(8)-C(9)-C(3)	108.15(14)	C(10)-C(9)-C(3)	111.51(14)
C(10)-C(9)-C(8)	112.53(14)	C(14)-C(9)-C(3)	107.47(14)
C(14)-C(9)-C(8)	108.76(13)	C(14)-C(9)-C(10)	108.28(14)
H(10a)-C(10)-C(9)	112.1(11)	H(10b)-C(10)-C(9)	114.7(11)
H(10b)-C(10)-H(10a)	102.2(15)	H(10c)-C(10)-C(9)	115.1(11)
H(10c)-C(10)-H(10a)	106.8(15)	H(10c)-C(10)-H(10b)	104.8(15)
C(12)-C(11)-C(8)	109.25(15)	C(15)-C(11)-C(8)	115.22(16)
C(15)-C(11)-C(12)	110.33(17)	C(16)-C(11)-C(8)	108.25(15)
C(16)-C(11)-C(12)	106.72(15)	C(16)-C(11)-C(15)	106.70(17)
H(12a)-C(12)-C(11)	110.3(11)	H(12b)-C(12)-C(11)	108.9(11)
H(12b)-C(12)-H(12a)	107.0(15)	C(13)-C(12)-C(11)	114.15(14)
C(13)-C(12)-H(12a)	108.0(11)	C(13)-C(12)-H(12b)	108.2(11)
H(13a)-C(13)-C(12)	110.7(10)	H(13b)-C(13)-C(12)	109.4(10)
H(13b)-C(13)-H(13a)	106.9(14)	C(14)-C(13)-C(12)	110.54(15)
C(14)-C(13)-H(13a)	109.8(10)	C(14)-C(13)-H(13b)	109.4(10)
C(13)-C(14)-C(9)	112.58(14)	H(14a)-C(14)-C(9)	108.9(11)
H(14a)-C(14)-C(13)	109.3(10)	H(14b)-C(14)-C(9)	111.5(10)
H(14b)-C(14)-C(13)	110.1(10)	H(14b)-C(14)-H(14a)	104.1(14)
H(15a)-C(15)-C(11)	112.9(13)	H(15b)-C(15)-C(11)	115.3(12)
H(15b)-C(15)-H(15a)	105.5(16)	H(15c)-C(15)-C(11)	108.0(13)
H(15c)-C(15)-H(15a)	108.1(17)	H(15c)-C(15)-H(15b)	106.6(17)
H(16a)-C(16)-C(11)	113.4(13)	H(16b)-C(16)-C(11)	111.9(13)
H(16b)-C(16)-H(16a)	107.3(19)	H(16c)-C(16)-C(11)	109.7(12)
H(16c)-C(16)-H(16a)	108.8(18)	H(16c)-C(16)-H(16b)	105.4(17)

Crystallographic data for (±)-sclareolide (12a)

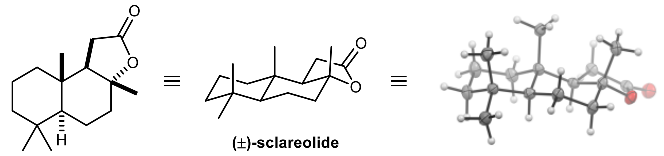

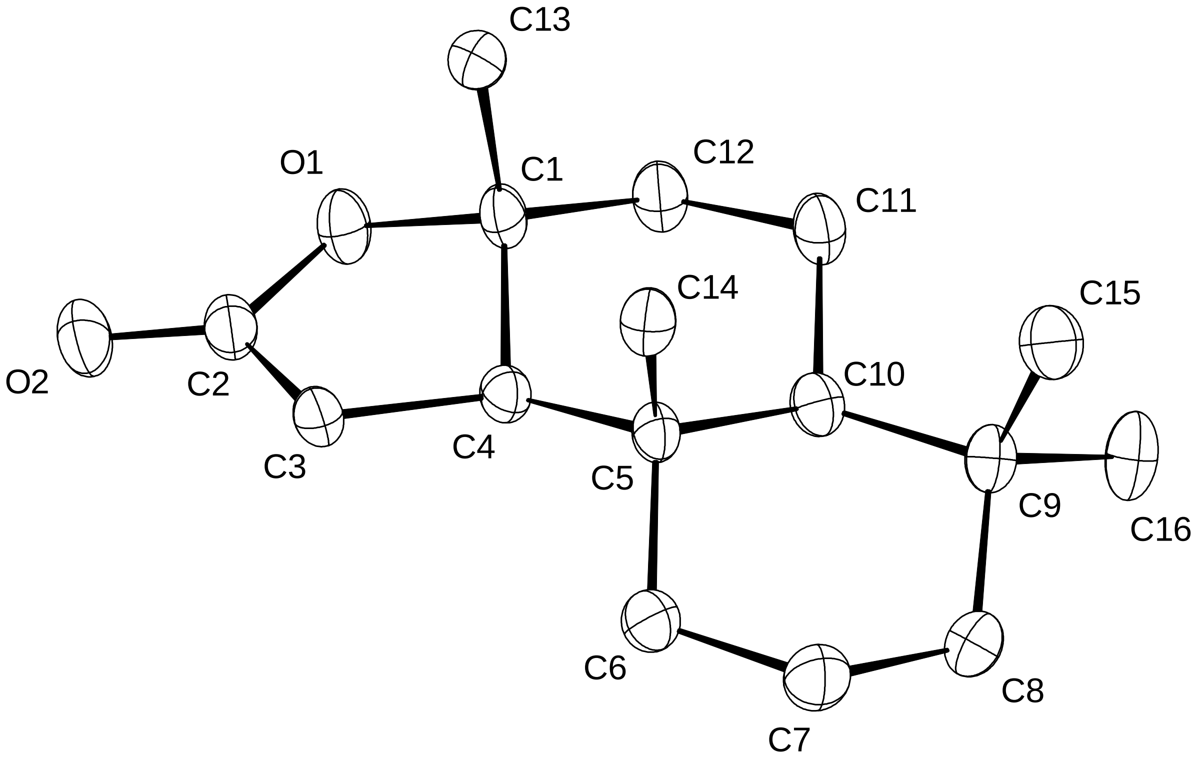

Fig. S90 | The molecular structure of (±)-sclareolide (**12a**). Hydrogen atoms have been removed for clarity.

X-ray Crystal Structure Analysis of (±)-sclareolide (**12a**):

$C_{16}H_{26}O_2$, $M_r = 250.37$ g · mol^{-1}, colorless plate, crystal size 0.22 × 0.10 × 0.03 mm^3, monoclinic, space group *C*2/*c* [15], a = 27.4844(10) Å, b = 6.2688(13) Å, c = 16.423(3) Å, β = 101.084(11)°, V = 2776.8(7) Å^3, T = 100(2) K, Z = 8, D_{calc} = 1.198 g · cm^3, λ = 0.71073 Å, μ(Mo-K$_\alpha$) = 0.076 mm^{-1}, Numerical correction ($T_{\min}$ = 0.98701, $T_{\max}$ 0.99773), Bruker AXS Enraf-Nonius KappaCCD diffractometer with a FR591 rotating Mo-anode X-ray source Incoatec Helios focusing multilayer optics, 3.336 < θ < 30.505°, 13949 measured reflections, 4211 independent reflections, 2370 reflections with $I > 2\sigma(I)$, R_{int} = 0.0862. The structure was solved by *SHELXS* and refined by full-matrix least-squares (*SHELXL*) against F^2 to R_1 = 0.0771 [$I > 2\sigma(I)$], wR_2 = 0.1936 [all data] with 175 parameters and 0 restraints.

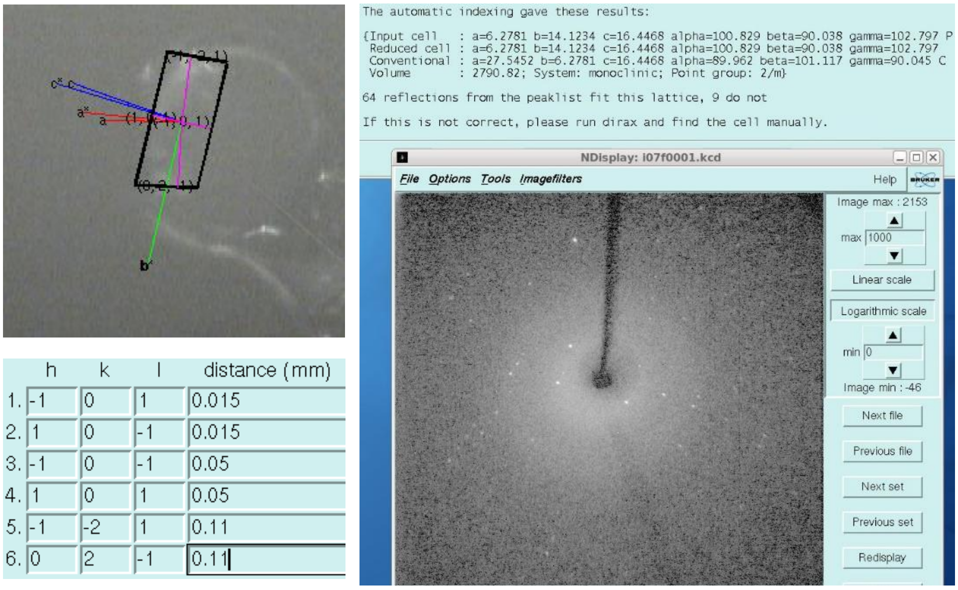

Fig. S91| Crystal faces and unit cell determination/refinement of (−)-9-*epi*-sclareolide (**12b**).

INTENSITY STATISTICS FOR DATASET

Resolution	#Data	#Theory	%Complete	Redundancy	Mean I	Mean I/s	Rmerge	Rsigma
Inf - 2.74	86	93	92.5	3.40	91.95	37.51	0.0228	0.0160
2.74 - 1.84	194	194	100.0	4.27	41.93	27.85	0.0286	0.0221
1.84 - 1.45	280	280	100.0	4.31	17.66	17.33	0.0426	0.0348
1.45 - 1.25	281	281	100.0	4.22	12.02	12.80	0.0556	0.0495
1.25 - 1.14	278	278	100.0	4.00	13.06	11.54	0.0623	0.0541
1.14 - 1.05	289	289	100.0	3.80	8.32	8.65	0.0840	0.0787
1.05 - 0.99	264	264	100.0	3.48	6.04	6.44	0.1176	0.1112
0.99 - 0.93	327	328	99.7	3.31	3.92	4.52	0.1608	0.1679
0.93 - 0.89	268	268	100.0	3.16	2.90	3.47	0.2328	0.2369
0.89 - 0.86	249	250	99.6	3.00	2.67	3.01	0.2442	0.2735
0.86 - 0.83	269	270	99.6	2.95	2.15	2.48	0.2949	0.3544
0.83 - 0.80	320	320	100.0	2.81	2.42	2.31	0.3083	0.3613
0.80 - 0.77	363	366	99.2	2.67	2.49	2.24	0.3120	0.3700
0.77 - 0.75	286	286	100.0	2.50	2.33	2.04	0.3497	0.4321
0.75 - 0.74	143	144	99.3	2.53	2.59	2.01	0.3297	0.4093
0.74 - 0.72	315	318	99.1	2.42	2.10	1.68	0.3996	0.5249
0.72 - 0.70	355	358	99.2	2.35	2.05	1.53	0.4216	0.5712
0.70 - 0.69	195	197	99.0	2.24	1.50	1.02	0.5626	0.8073
0.69 - 0.67	437	442	98.9	2.21	1.29	0.89	0.6272	1.0129
0.67 - 0.66	251	252	99.6	2.01	1.17	0.70	0.6765	1.2070
0.66 - 0.65	112	116	96.6	1.75	0.96	0.58	0.8177	1.5421
0.75 - 0.65	1808	1827	99.0	2.25	1.67	1.21	0.4929	0.7429
Inf - 0.65	5562	5594	99.4	3.01	7.31	5.87	0.0927	0.1263

Complete .cif-data are available under the CCDC number CCDC-2338449.

Table S32 | Crystal data and structure refinement for (±)-sclareolide (**12a**).

Identification code	12203 / CCDC-2338449	
Empirical formula	$C_{16}H_{26}O_2$	
Colour	colorless	
Formula weight	250.37 g · mol^{-1}	
Temperature	100(2) K	
Wavelength	0.71073 Å	
Crystal system	monoclinic	
Space group	*C*2/c, (No. 15)	
Unit cell dimensions	a = 27.4844(10) Å	α = 90°.
	b = 6.2688(13) Å	β = 101.084(11)°.
	c = 16.423(3) Å	γ = 90°.
Volume	2776.8(7) Å^3	
Z	8	
Density (calculated)	1.198 Mg · m^{-3}	
Absorption coefficient	0.076 mm^{-1}	
F(000)	1104 e	
Crystal size	0.22 × 0.10 × 0.03 mm^3	
θ range for data collection	3.336 to 30.505°.	
Index ranges	$-37 \leq h \leq 39$, $-8 \leq k \leq 8$, $-23 \leq l \leq 23$	
Reflections collected	13949	
Independent reflections	4211 [R_{int} = 0.0862]	
Reflections with $I > 2\sigma(I)$	2370	
Completeness to θ = 25.242°	99.6 %	
Absorption correction	Gaussian	
Max. and min. transmission	1.00 and 0.99	
Refinement method	Full-matrix least-squares on F^2	
Data / restraints / parameters	4211 / 0 / 175	
Goodness-of-fit on F^2	1.037	
Final *R* indices [$I > 2\sigma(I)$]	R_1 = 0.0771	wR_2 = 0.1608
R indices (all data)	R_1 = 0.1483	wR_2 = 0.1936
Extinction coefficient	n/a	

Table S33 | Bond lengths [Å] and angles [°] for (−)-8-*epi*-sclareolide (**12f**).

O(1)-C(1)	1.483(2)	O(1)-C(2)	1.360(3)
O(2)-C(2)	1.199(2)	C(1)-C(4)	1.533(3)
C(1)-C(12)	1.512(3)	C(1)-C(13)	1.526(3)
C(2)-C(3)	1.519(3)	C(3)-C(4)	1.521(3)
C(4)-C(5)	1.541(3)	C(5)-C(6)	1.531(3)
C(5)-C(10)	1.566(3)	C(5)-C(14)	1.538(3)
C(6)-C(7)	1.539(3)	C(7)-C(8)	1.533(3)
C(8)-C(9)	1.533(3)	C(9)-C(10)	1.559(3)
C(9)-C(15)	1.537(3)	C(9)-C(16)	1.534(3)
C(10)-C(11)	1.531(3)	C(11)-C(12)	1.548(3)
C(2)-O(1)-C(1)	108.33(17)	O(1)-C(1)-C(4)	100.61(16)
O(1)-C(1)-C(12)	111.99(18)	O(1)-C(1)-C(13)	104.23(16)
C(12)-C(1)-C(4)	108.94(18)	C(12)-C(1)-C(13)	111.45(19)
C(13)-C(1)-C(4)	119.0(2)	O(1)-C(2)-C(3)	110.32(17)
O(2)-C(2)-O(1)	121.0(2)	O(2)-C(2)-C(3)	128.7(2)
C(2)-C(3)-C(4)	100.73(18)	C(1)-C(4)-C(5)	116.17(17)
C(3)-C(4)-C(1)	101.67(17)	C(3)-C(4)-C(5)	123.80(19)
C(4)-C(5)-C(10)	103.61(17)	C(6)-C(5)-C(4)	109.30(18)
C(6)-C(5)-C(10)	107.97(17)	C(6)-C(5)-C(14)	108.85(19)
C(14)-C(5)-C(4)	111.42(17)	C(14)-C(5)-C(10)	115.46(18)
C(5)-C(6)-C(7)	112.03(19)	C(8)-C(7)-C(6)	111.5(2)
C(9)-C(8)-C(7)	114.30(19)	C(8)-C(9)-C(10)	108.15(18)
C(8)-C(9)-C(15)	110.70(19)	C(8)-C(9)-C(16)	107.03(19)
C(15)-C(9)-C(10)	114.39(18)	C(16)-C(9)-C(10)	109.48(18)
C(16)-C(9)-C(15)	106.84(19)	C(9)-C(10)-C(5)	115.19(18)
C(11)-C(10)-C(5)	112.77(18)	C(11)-C(10)-C(9)	114.58(18)
C(10)-C(11)-C(12)	112.90(18)	C(1)-C(12)-C(11)	107.85(18)

Crystallographic data for (±)-9-*epi*-sclareolide (12b)

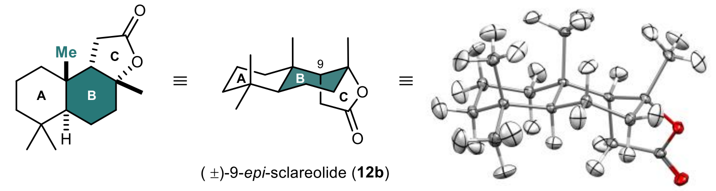

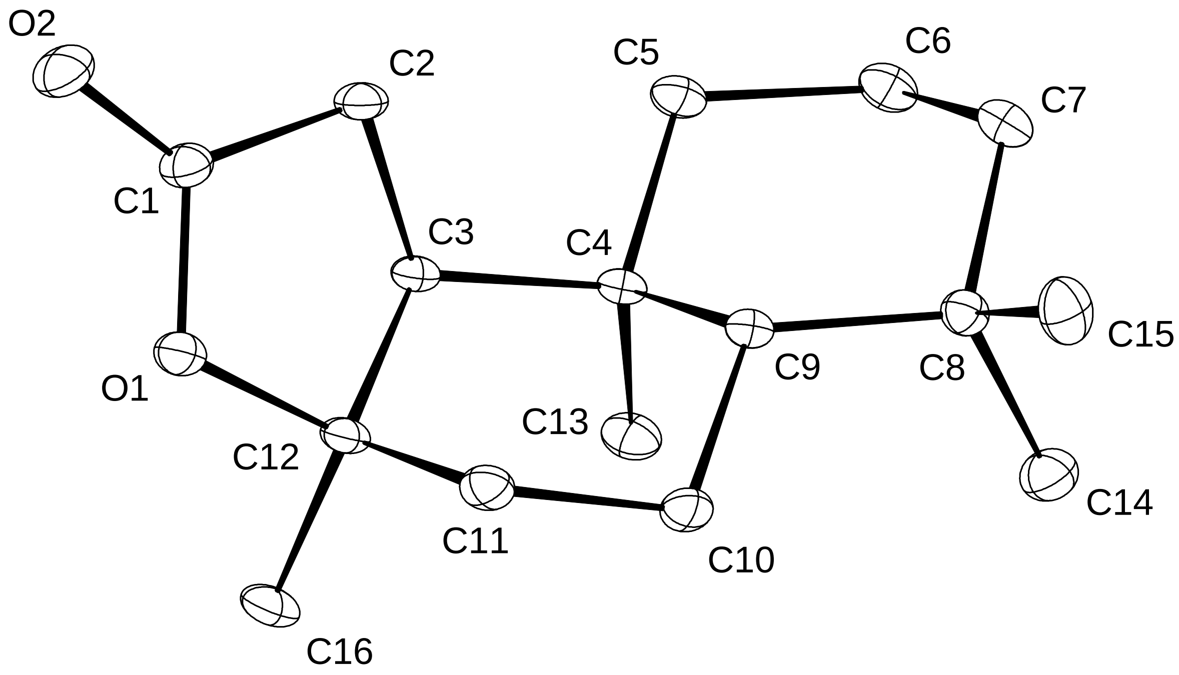

Fig. S92 | The molecular structure of (±)-9-*epi*-sclareolide (**12b**). Hydrogen atoms have been removed for clarity.

X-ray Crystal Structure Analysis of (±)-9-epi-sclareolide (12b):

$C_{16}H_{26}O_2$, M_r = 250.384 g · mol^{-1}, colorless prism, crystal size 0.082 × 0.052 × 0.034 mm^3, monoclinic, space group $P2_1/c$ [14], a = 11.9316(6) Å, b = 15.9764(7) Å, c = 7.3499(4) Å, , β = 93.508(2)°, V = 1398.44(12) Å^3, T = 100(2) K, Z = 4, D_{calc} = 1.189 g · cm^3, λ = 0.71073 Å, μ(Mo-K$_\alpha$) = 0.076 mm^{-1}, Numerical correction (T_{min} = 0.99407, T_{max} = 0.99754), Bruker-AXS Kappa Mach3 with APEX-II detector and IµS microfocus Mo-anode X-ray source and Incoatec Helios mirrors, 1.71 < θ < 31.50°, 46908 measured reflections, 4633 independent reflections, 3814 reflections with $I > 2\sigma(I)$, R_{int} = 0.0421. The structure was solved by *SHELXT* and refined by full-matrix least-squares (*SHELXL*). The final structure refinement was performed by olex2.refine 1.5 (L-M) together with NoSpherA2 (atomic form factors) against F^2 to R_1 = 0.0250 [$I > 2\sigma(I)$], wR_2 = 0.0546 [all data] with 397 parameters and 0 restraints.

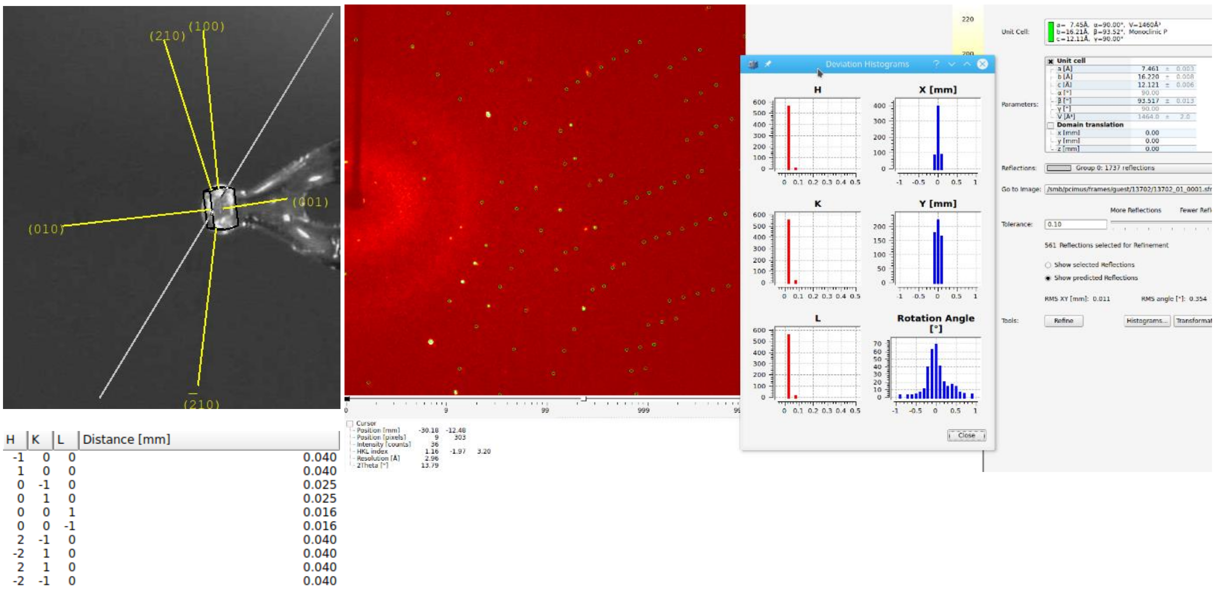

Fig. S93 | Crystal faces and unit cell determination/refinement of (±)-9-*epi*-sclareolide (**12b**).

INTENSITY STATISTICS FOR DATASET

Resolution	#Data	#Theory	%Complete	Redundancy	Mean I	Mean I/s	Rmerge	Rsigma
Inf - 2.89	72	72	100.0	16.06	57.01	83.12	0.0254	0.0097
2.89 - 1.89	171	171	100.0	17.63	22.72	73.20	0.0247	0.0102
1.89 - 1.48	244	244	100.0	17.91	11.19	61.93	0.0299	0.0108
1.48 - 1.29	244	244	100.0	17.84	6.93	50.17	0.0351	0.0123
1.29 - 1.17	238	238	100.0	17.22	7.17	48.00	0.0389	0.0131
1.17 - 1.08	258	258	100.0	15.73	6.19	43.93	0.0454	0.0148
1.08 - 1.02	233	233	100.0	12.07	4.39	32.84	0.0539	0.0202
1.02 - 0.97	226	226	100.0	10.50	3.44	27.94	0.0640	0.0254
0.97 - 0.92	279	279	100.0	9.17	2.43	20.30	0.0785	0.0342
0.92 - 0.89	212	212	100.0	8.08	1.94	15.29	0.0926	0.0439
0.89 - 0.86	234	234	100.0	7.84	1.97	15.19	0.0918	0.0460
0.86 - 0.83	266	266	100.0	7.53	1.89	14.64	0.0974	0.0489
0.83 - 0.80	287	287	100.0	7.20	1.72	12.49	0.1025	0.0557
0.80 - 0.78	260	260	100.0	6.99	2.17	13.25	0.0950	0.0498
0.78 - 0.76	239	239	100.0	6.84	1.97	12.70	0.0989	0.0554
0.76 - 0.75	142	142	100.0	6.67	1.93	12.61	0.1052	0.0580
0.75 - 0.73	296	297	99.7	6.52	1.99	11.32	0.1105	0.0599
0.73 - 0.71	350	351	99.7	6.18	1.72	9.95	0.1246	0.0721
0.71 - 0.70	162	162	100.0	6.06	1.79	10.20	0.1326	0.0723
0.70 - 0.69	208	208	100.0	6.02	1.45	8.58	0.1438	0.0864
0.69 - 0.68	169	182	92.9	4.49	1.17	6.71	0.1859	0.1287
0.78 - 0.68	1566	1581	99.1	6.16	1.74	10.36	0.1206	0.0708
Inf - 0.68	4790	4805	99.7	9.98	4.88	25.51	0.0415	0.0236

Complete .cif-data are available under the CCDC number CCDC-2338445.

The final structure refinement was carried out with using aspherical scattering factors with NoSpherA2. DFT-calculated with ORCA using a B3LYP functional and def2-TZVPP basis set, whereby the H atom positions were refined using anisotropic atomic displacement parameters.
NoSpherA2 implementation of HAR makes use of tailor-made aspherical atomic form factors calculated on-the-fly from a Hirshfeld-partitioned electron density (ED) - not from spherical-atom form factors. The ED is calculated from a gaussian basis set single determinant SCF wave function - either Hartree-Fock or DFT using selected functional - for a fragment of the crystal. This fragment can be embedded in an electrostatic crystal field by employing cluster charges or modelled using implicit solvation models, depending on the software used. The following options were used:

SOFTWARE:	ORCA 5.0
PARTITIONING:	NoSpherA2
INT ACCURACY:	High
METHOD:	B3LYP
BASIS SET:	def2-TZVPP
CHARGE:	0
MULTIPLICITY:	1
DATE:	2024-02-27_20-59-25

Table S34 | Crystal data and structure refinement for (±)-9-*epi*-sclareolide (**12b**).

Identification code	13702 / CCDC-2338445	
Empirical formula	$C_{16}H_{26}O_2$	
Colour	colorless	
Formula weight	250.384 g · mol^{-1}	
Temperature	100(2) K	
Wavelength	0.71073 Å	
Crystal system	monoclinic	
Space group	$P2_1$/c, (No. 14)	
Unit cell dimensions	a = 11.9316(6) Å	α = 90°.
	b = 15.9764(7) Å	β = 93.508(2)°.
	c = 7.3499(4) Å	γ = 90°.
Volume	1398.44(12) Å^3	
Z	4	
Density (calculated)	1.189 Mg · m^{-3}	
Absorption coefficient	0.076 mm^{-1}	
F(000)	552.312 e	
Crystal size	0.082 × 0.052 × 0.034 mm^3	
θ range for data collection	1.71 to 31.50°.	
Index ranges	$-17 \le h \le 17$, $-23 \le k \le 23$, $-10 \le l \le 10$	
Reflections collected	46908	
Independent reflections	4633 [R_{int} = 0.0421]	
Reflections with $I > 2\sigma(I)$	3814	
Completeness to θ = 67.679°	100.0 %	
Absorption correction	Gaussian	
Max. and min. transmission	0.99754 and 0.99407	
Refinement method	Full-matrix least-squares on F^2	
Data / restraints / parameters	4633 / 0 / 397	
Goodness-of-fit on F^2	1.1958	
Final R indices [$I > 2\sigma(I)$]	R_1 = 0.0250	wR_2 = 0.0507
R indices (all data)	R_1 = 0.0372	wR_2 = 0.0546
Largest diff. peak and hole	0.2467 and −0.1940 e · Å^{-3}	

Table S35 | Bond lengths [Å] and angles [°] for (±)-9-*epi*-sclareolide (**12b**).

O(1)-C(1)	1.3476(6)	O(1)-C(12)	1.4754(6)
O(2)-C(1)	1.2060(6)	C(1)-C(2)	1.5058(7)
C(2)-H(2a)	1.076(7)	C(2)-H(2b)	1.102(7)
C(2)-C(3)	1.5325(7)	C(3)-H(3)	1.089(6)
C(3)-C(4)	1.5511(7)	C(3)-C(12)	1.5490(7)
C(4)-C(5)	1.5423(7)	C(4)-C(9)	1.5538(7)
C(4)-C(13)	1.5453(7)	C(5)-H(5a)	1.095(6)
C(5)-H(5b)	1.105(6)	C(5)-C(6)	1.5325(7)
C(6)-H(6a)	1.100(7)	C(6)-H(6b)	1.089(6)
C(6)-C(7)	1.5290(8)	C(7)-H(7a)	1.090(7)
C(7)-H(7b)	1.081(7)	C(7)-C(8)	1.5414(7)
C(8)-C(9)	1.5575(7)	C(8)-C(14)	1.5388(7)
C(8)-C(15)	1.5368(7)	C(9)-H(9)	1.103(6)
C(9)-C(10)	1.5339(7)	C(10)-H(10a)	1.089(6)
C(10)-H(10b)	1.080(7)	C(10)-C(11)	1.5325(7)
C(11)-H(11a)	1.104(6)	C(11)-H(11b)	1.102(7)
C(11)-C(12)	1.5298(7)	C(12)-C(16)	1.5204(7)
C(13)-H(13a)	1.097(7)	C(13)-H(13b)	1.090(7)
C(13)-H(13c)	1.084(7)	C(14)-H(14a)	1.098(7)
C(14)-H(14b)	1.061(8)	C(14)-H(14c)	1.088(7)
C(15)-H(15a)	1.094(7)	C(15)-H(15b)	1.073(7)
C(15)-H(15c)	1.093(7)	C(16)-H(16a)	1.078(7)
C(16)-H(16b)	1.078(7)	C(16)-H(16c)	1.080(7)
C(12)-O(1)-C(1)	110.89(4)	O(2)-C(1)-O(1)	121.54(5)
C(2)-C(1)-O(1)	110.06(4)	C(2)-C(1)-O(2)	128.39(5)
H(2a)-C(2)-C(1)	109.7(4)	H(2b)-C(2)-C(1)	106.3(4)
H(2b)-C(2)-H(2a)	110.4(5)	C(3)-C(2)-C(1)	102.76(4)
C(3)-C(2)-H(2a)	113.6(4)	C(3)-C(2)-H(2b)	113.5(3)
H(3)-C(3)-C(2)	106.3(3)	C(4)-C(3)-C(2)	117.09(4)
C(4)-C(3)-H(3)	106.9(3)	C(12)-C(3)-C(2)	101.74(4)
C(12)-C(3)-H(3)	107.8(3)	C(12)-C(3)-C(4)	116.26(4)
C(5)-C(4)-C(3)	108.64(4)	C(9)-C(4)-C(3)	108.92(4)
C(9)-C(4)-C(5)	109.46(4)	C(13)-C(4)-C(3)	107.74(4)
C(13)-C(4)-C(5)	107.88(4)	C(13)-C(4)-C(9)	114.07(4)
H(5a)-C(5)-C(4)	109.3(4)	H(5b)-C(5)-C(4)	109.2(3)
H(5b)-C(5)-H(5a)	107.8(5)	C(6)-C(5)-C(4)	112.38(4)
C(6)-C(5)-H(5a)	111.1(4)	C(6)-C(5)-H(5b)	107.0(3)

H(6a)-C(6)-C(5)	111.4(3)	H(6b)-C(6)-C(5)	110.1(4)
H(6b)-C(6)-H(6a)	104.3(5)	C(7)-C(6)-C(5)	110.12(4)
C(7)-C(6)-H(6a)	110.6(4)	C(7)-C(6)-H(6b)	110.2(4)
H(7a)-C(7)-C(6)	112.2(4)	H(7b)-C(7)-C(6)	108.0(4)
H(7b)-C(7)-H(7a)	104.9(5)	C(8)-C(7)-C(6)	114.08(4)
C(8)-C(7)-H(7a)	108.9(4)	C(8)-C(7)-H(7b)	108.3(3)
C(9)-C(8)-C(7)	108.42(4)	C(14)-C(8)-C(7)	110.58(4)
C(14)-C(8)-C(9)	114.32(4)	C(15)-C(8)-C(7)	107.16(4)
C(15)-C(8)-C(9)	108.88(4)	C(15)-C(8)-C(14)	107.24(4)
C(8)-C(9)-C(4)	116.13(4)	H(9)-C(9)-C(4)	104.3(3)
H(9)-C(9)-C(8)	103.7(3)	C(10)-C(9)-C(4)	110.32(4)
C(10)-C(9)-C(8)	114.85(4)	C(10)-C(9)-H(9)	106.2(3)
H(10a)-C(10)-C(9)	110.1(3)	H(10b)-C(10)-C(9)	111.7(3)
H(10b)-C(10)-H(10a)	104.2(5)	C(11)-C(10)-C(9)	110.32(4)
C(11)-C(10)-H(10a)	112.3(4)	C(11)-C(10)-H(10b)	108.2(4)
H(11a)-C(11)-C(10)	111.6(4)	H(11b)-C(11)-C(10)	108.5(3)
H(11b)-C(11)-H(11a)	105.6(5)	C(12)-C(11)-C(10)	112.67(4)
C(12)-C(11)-H(11a)	109.8(4)	C(12)-C(11)-H(11b)	108.4(3)
C(3)-C(12)-O(1)	103.23(4)	C(11)-C(12)-O(1)	107.49(4)
C(11)-C(12)-C(3)	112.32(4)	C(16)-C(12)-O(1)	104.89(4)
C(16)-C(12)-C(3)	115.21(4)	C(16)-C(12)-C(11)	112.64(4)
H(13a)-C(13)-C(4)	113.4(4)	H(13b)-C(13)-C(4)	109.4(4)
H(13b)-C(13)-H(13a)	108.8(6)	H(13c)-C(13)-C(4)	112.5(3)
H(13c)-C(13)-H(13a)	105.6(5)	H(13c)-C(13)-H(13b)	106.9(6)
H(14a)-C(14)-C(8)	111.0(4)	H(14b)-C(14)-C(8)	112.5(4)
H(14b)-C(14)-H(14a)	108.6(6)	H(14c)-C(14)-C(8)	109.7(4)
H(14c)-C(14)-H(14a)	107.7(6)	H(14c)-C(14)-H(14b)	107.1(6)
H(15a)-C(15)-C(8)	111.5(4)	H(15b)-C(15)-C(8)	113.5(4)
H(15b)-C(15)-H(15a)	107.1(6)	H(15c)-C(15)-C(8)	109.9(4)
H(15c)-C(15)-H(15a)	108.3(6)	H(15c)-C(15)-H(15b)	106.3(6)
H(16a)-C(16)-C(12)	108.4(4)	H(16b)-C(16)-C(12)	110.5(4)
H(16b)-C(16)-H(16a)	109.2(6)	H(16c)-C(16)-C(12)	111.7(4)
H(16c)-C(16)-H(16a)	108.0(6)	H(16c)-C(16)-H(16b)	109.0(6)

Crystallographic data for (±)-5β,8α-sclareolide (12d)

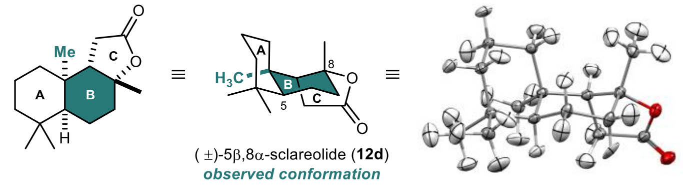

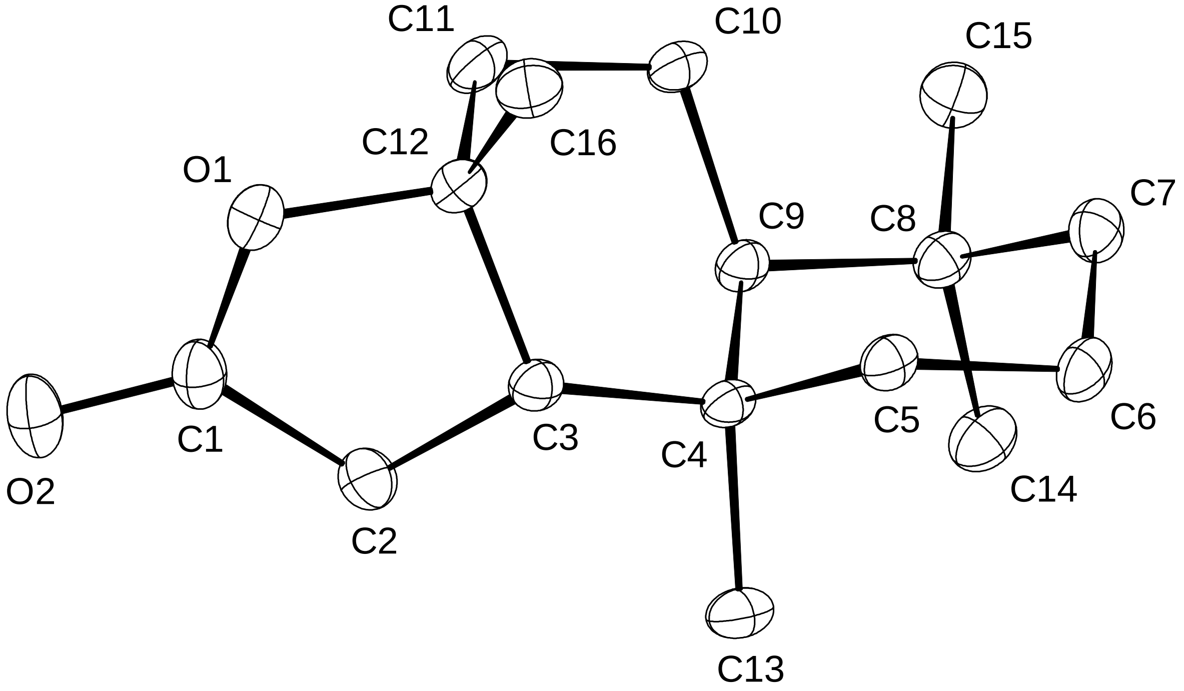

Fig. S94 | The molecular structure of (±)-5β,8α-sclareolide (**12d**). Hydrogen atoms have been removed for clarity.

X-ray Crystal Structure Analysis of (±)-5*β*,8*α*-sclareolide (**12d**):

$C_{16}H_{26}O_2$, M_r = 250.384 g · mol^{-1}, colorless plate, crystal size 0.154 × 0.108 × 0.041 mm^3, triclinic, space group *P*-1 [2], *a* = 7.1407(5) Å, *b* = 8.5755(6) Å, *c* = 12.1829(8) Å, α = 109.081(3)°, β = 97.153(4)°, γ = 90.890(4)°, *V* = 698.29(8) Å^3, *T* = 100(2) K, *Z* = 2, D_{calc} = 1.191 g · cm^3, λ = 0.71073 Å, μ(Mo-K$_\alpha$) = 0.076 mm^{-1}, Numerical correction ($T_{\min}$ = 0.99165, $T_{\max}$ = 0.99740), Bruker-AXS Kappa Mach3 with APEX-II detector and IµS microfocus Mo-anode X-ray source and Incoatec Helios mirrors, 1.79 < θ < 27.87°, 16184 measured reflections, 3317 independent reflections, 2722 reflections with *I* > 2σ(*I*), R_{int} = 0.0303. The structure was solved by *SHELXT* and refined by full-matrix least-squares (*SHELXL*). The final structure refinement was performed by olex2.refine 1.5 (L-M) together with NoSpherA2 (atomic form factors) against F^2 to R_1 = 0.0247 [$I > 2\sigma(I)$], wR_2 = 0.0523 [all data] with 397 parameters and 0 restraints.

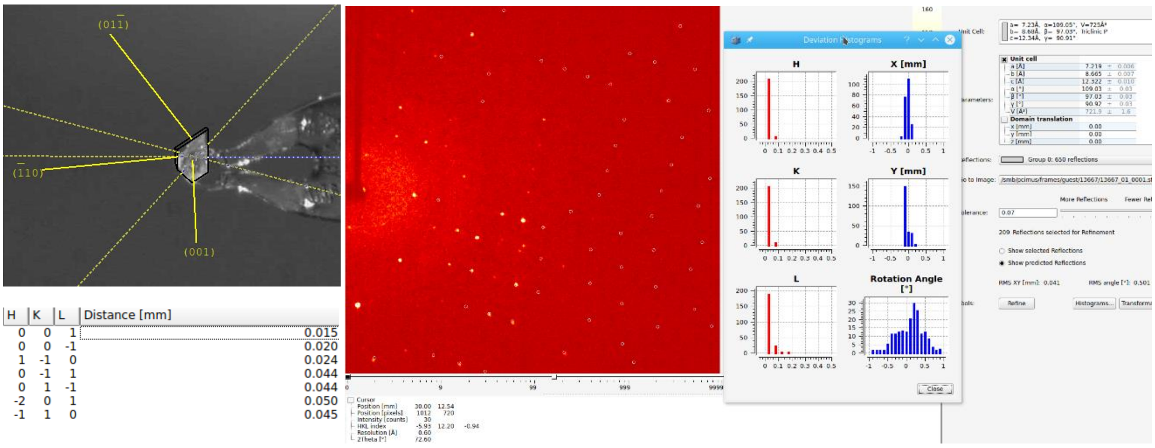

Fig. S95 | Crystal faces and unit cell determination/refinement of (±)-5β,8α-sclareolide (**12d**).

INTENSITY STATISTICS FOR DATASET

Resolution	#Data	#Theory	%Complete	Redundancy	Mean I	Mean I/s	Rmerge	Rsigma
Inf - 3.00	53	54	98.1	7.07	123.48	72.95	0.0279	0.0129
3.00 - 2.04	121	121	100.0	7.00	41.08	53.77	0.0213	0.0148
2.04 - 1.61	178	182	97.8	6.99	30.23	53.96	0.0198	0.0143
1.61 - 1.40	172	173	99.4	7.07	12.90	44.62	0.0234	0.0176
1.40 - 1.28	172	175	98.3	6.87	12.43	41.19	0.0255	0.0175
1.28 - 1.18	184	185	99.5	6.91	12.85	39.72	0.0259	0.0183
1.18 - 1.11	176	177	99.4	6.17	11.67	37.28	0.0273	0.0212
1.11 - 1.06	161	163	98.8	5.87	10.02	33.96	0.0331	0.0221
1.06 - 1.01	186	186	100.0	5.04	6.55	25.13	0.0410	0.0300
1.01 - 0.97	190	191	99.5	4.52	4.75	18.61	0.0525	0.0378
0.97 - 0.94	154	155	99.4	4.31	3.93	16.76	0.0593	0.0441
0.94 - 0.91	180	182	98.9	3.97	4.03	14.68	0.0541	0.0445
0.91 - 0.88	200	201	99.5	3.79	3.26	13.06	0.0717	0.0556
0.88 - 0.86	150	151	99.3	3.42	2.91	12.21	0.0740	0.0662
0.86 - 0.84	175	175	100.0	3.50	3.06	12.17	0.0751	0.0649
0.84 - 0.82	187	188	99.5	3.48	2.77	10.88	0.0832	0.0705
0.82 - 0.80	187	187	100.0	3.47	3.13	11.80	0.0792	0.0650
0.80 - 0.78	234	235	99.6	3.11	2.72	10.15	0.0862	0.0809
0.78 - 0.77	122	124	98.4	3.15	2.79	9.12	0.1048	0.0804
0.77 - 0.76	135	136	99.3	3.12	2.66	9.56	0.1034	0.0866
0.76 - 0.75	164	194	84.5	2.42	3.27	9.45	0.0791	0.0786
0.85 - 0.75	1121	1156	97.0	3.15	2.88	10.39	0.0861	0.0758
Inf - 0.75	3481	3535	98.5	4.71	10.15	24.32	0.0303	0.0252

Complete .cif-data are available under the CCDC number CCDC-2338444.

The final structure refinement was carried out with using aspherical scattering factors with NoSpherA2.[11] DFT-calculated with ORCA using a B3LYP functional and def2-TZVPP basis set, whereby the H atom positions were refined using anisotropic atomic displacement parameters.
NoSpherA2 implementation of HAR makes use of tailor-made aspherical atomic form factors calculated on-the-fly from a Hirshfeld-partitioned electron density (ED) - not from spherical-atom form factors. The ED is calculated from a gaussian basis set single determinant SCF wave function - either Hartree-Fock or DFT using selected functional - for a fragment of the crystal. This fragment can be embedded in an electrostatic crystal field by employing cluster charges or modelled using implicit solvation models, depending on the software used. The following options were used:

SOFTWARE:	ORCA 5.0
PARTITIONING:	NoSpherA2
INT ACCURACY:	High
METHOD:	B3LYP
BASIS SET:	def2-TZVPP
CHARGE:	0
MULTIPLICITY:	1
DATE:	2024-02-27_20-57-18

Table S36 | Crystal data and structure refinement for (±)-5β,8α-sclareolide (**12d**).

Identification code	13667 / CCDC-2338444	
Empirical formula	$C_{16}H_{26}O_2$	
Colour	colorless	
Formula weight	250.384 g · mol^{-1}	
Temperature	100(2) K	
Wavelength	0.71073 Å	
Crystal system	triclinic	
Space group	$P\bar{1}$, (No. 2)	
Unit cell dimensions	a = 7.1407(5) Å	α = 109.081(3)°.
	b = 8.5755(6) Å	β = 97.153(4)°.
	c = 12.1829(8) Å	γ = 90.890(4)°.
Volume	698.29(8) Å^3	
Z	2	
Density (calculated)	1.191 Mg · m^{-3}	
Absorption coefficient	0.076 mm^{-1}	
F(000)	276.156 e	
Crystal size	0.154 × 0.108 × 0.041 mm^3	
θ range for data collection	1.79 to 27.87°.	
Index ranges	$-9 \leq h \leq 9$, $-11 \leq k \leq 11$, $-16 \leq l \leq 16$	
Reflections collected	16184	
Independent reflections	3317 [R_{int} = 0.0303]	
Reflections with $I > 2\sigma(I)$	2722	
Completeness to θ = 25.2417°	99.25 %	
Absorption correction	Gaussian	
Max. and min. transmission	0.99740 and 0.99165	
Refinement method	Full-matrix least-squares on F^2	
Data / restraints / parameters	3317 / 0 / 397	
Goodness-of-fit on F^2	1.1788	
Final R indices [$I > 2\sigma(I)$]	R_1 = 0.0247	wR_2 = 0.0480
R indices (all data)	R_1 = 0.0364	wR_2 = 0.0523
Largest diff. peak and hole	0.1811 and −0.1374 e · Å^{-3}	

Table S37 | Bond lengths [Å] and angles [°] for (±)-5β,8α-sclareolide (**12d**).

O(1)-C(1)	1.3427(9)	O(1)-C(12)	1.4754(8)
O(2)-C(1)	1.2063(9)	C(1)-C(2)	1.5071(10)
C(2)-H(2a)	1.078(9)	C(2)-H(2b)	1.093(10)
C(2)-C(3)	1.5349(10)	C(3)-H(3)	1.078(9)
C(3)-C(4)	1.5601(9)	C(3)-C(12)	1.5417(9)
C(4)-C(5)	1.5414(10)	C(4)-C(9)	1.5640(10)
C(4)-C(13)	1.5446(9)	C(5)-H(5a)	1.079(8)
C(5)-H(5b)	1.076(8)	C(5)-C(6)	1.5333(10)
C(6)-H(6a)	1.094(9)	C(6)-H(6b)	1.078(8)
C(6)-C(7)	1.5298(11)	C(7)-H(7a)	1.081(8)
C(7)-H(7b)	1.097(8)	C(7)-C(8)	1.5397(10)
C(8)-C(9)	1.5711(9)	C(8)-C(14)	1.5449(10)
C(8)-C(15)	1.5409(10)	C(9)-H(9)	1.115(9)
C(9)-C(10)	1.5421(10)	C(10)-H(10a)	1.088(9)
C(10)-H(10b)	1.095(9)	C(10)-C(11)	1.5264(10)
C(11)-H(11a)	1.083(9)	C(11)-H(11b)	1.084(10)
C(11)-C(12)	1.5249(11)	C(12)-C(16)	1.5162(11)
C(13)-H(13a)	1.087(9)	C(13)-H(13b)	1.088(10)
C(13)-H(13c)	1.088(9)	C(14)-H(14a)	1.071(10)
C(14)-H(14b)	1.082(10)	C(14)-H(14c)	1.074(9)
C(15)-H(15a)	1.078(10)	C(15)-H(15b)	1.088(8)
C(15)-H(15c)	1.083(9)	C(16)-H(16a)	1.088(10)
C(16)-H(16b)	1.067(10)	C(16)-H(16c)	1.080(9)
C(12)-O(1)-C(1)	110.29(5)	O(2)-C(1)-O(1)	121.44(7)
C(2)-C(1)-O(1)	110.10(6)	C(2)-C(1)-O(2)	128.46(7)
H(2a)-C(2)-C(1)	108.4(5)	H(2b)-C(2)-C(1)	107.3(5)
H(2b)-C(2)-H(2a)	108.3(7)	C(3)-C(2)-C(1)	103.88(6)
C(3)-C(2)-H(2a)	114.6(5)	C(3)-C(2)-H(2b)	113.8(5)
H(3)-C(3)-C(2)	106.6(4)	C(4)-C(3)-C(2)	115.49(6)
C(4)-C(3)-H(3)	106.7(4)	C(12)-C(3)-C(2)	101.44(6)
C(12)-C(3)-H(3)	108.0(4)	C(12)-C(3)-C(4)	117.89(5)
C(5)-C(4)-C(3)	108.99(6)	C(9)-C(4)-C(3)	111.97(5)
C(9)-C(4)-C(5)	109.60(6)	C(13)-C(4)-C(3)	104.63(5)
C(13)-C(4)-C(5)	108.19(6)	C(13)-C(4)-C(9)	113.25(6)
H(5a)-C(5)-C(4)	110.4(5)	H(5b)-C(5)-C(4)	108.0(5)
H(5b)-C(5)-H(5a)	107.0(6)	C(6)-C(5)-C(4)	112.75(6)
C(6)-C(5)-H(5a)	107.8(4)	C(6)-C(5)-H(5b)	110.8(5)

H(6a)-C(6)-C(5)	107.8(4)	H(6b)-C(6)-C(5)	109.8(5)
H(6b)-C(6)-H(6a)	107.4(7)	C(7)-C(6)-C(5)	110.87(6)
C(7)-C(6)-H(6a)	109.5(5)	C(7)-C(6)-H(6b)	111.4(5)
H(7a)-C(7)-C(6)	109.1(5)	H(7b)-C(7)-C(6)	111.5(5)
H(7b)-C(7)-H(7a)	105.8(6)	C(8)-C(7)-C(6)	113.64(6)
C(8)-C(7)-H(7a)	108.3(5)	C(8)-C(7)-H(7b)	108.2(5)
C(9)-C(8)-C(7)	110.30(6)	C(14)-C(8)-C(7)	110.22(6)
C(14)-C(8)-C(9)	112.03(6)	C(15)-C(8)-C(7)	108.16(6)
C(15)-C(8)-C(9)	110.45(6)	C(15)-C(8)-C(14)	105.51(6)
C(8)-C(9)-C(4)	114.23(5)	H(9)-C(9)-C(4)	108.9(4)
H(9)-C(9)-C(8)	106.3(4)	C(10)-C(9)-C(4)	110.32(6)
C(10)-C(9)-C(8)	111.10(5)	C(10)-C(9)-H(9)	105.5(4)
H(10a)-C(10)-C(9)	110.6(4)	H(10b)-C(10)-C(9)	109.0(5)
H(10b)-C(10)-H(10a)	105.8(7)	C(11)-C(10)-C(9)	112.73(6)
C(11)-C(10)-H(10a)	110.4(4)	C(11)-C(10)-H(10b)	108.0(5)
H(11a)-C(11)-C(10)	109.4(5)	H(11b)-C(11)-C(10)	108.6(5)
H(11b)-C(11)-H(11a)	107.9(7)	C(12)-C(11)-C(10)	111.74(6)
C(12)-C(11)-H(11a)	109.7(5)	C(12)-C(11)-H(11b)	109.4(5)
C(3)-C(12)-O(1)	103.42(5)	C(11)-C(12)-O(1)	107.00(6)
C(11)-C(12)-C(3)	110.57(6)	C(16)-C(12)-O(1)	105.50(6)
C(16)-C(12)-C(3)	116.14(6)	C(16)-C(12)-C(11)	113.18(7)
H(13a)-C(13)-C(4)	108.3(5)	H(13b)-C(13)-C(4)	110.4(5)
H(13b)-C(13)-H(13a)	108.3(7)	H(13c)-C(13)-C(4)	113.9(4)
H(13c)-C(13)-H(13a)	107.9(7)	H(13c)-C(13)-H(13b)	108.0(7)
H(14a)-C(14)-C(8)	114.4(5)	H(14b)-C(14)-C(8)	110.1(5)
H(14b)-C(14)-H(14a)	107.4(8)	H(14c)-C(14)-C(8)	109.7(5)
H(14c)-C(14)-H(14a)	106.6(7)	H(14c)-C(14)-H(14b)	108.5(8)
H(15a)-C(15)-C(8)	111.3(5)	H(15b)-C(15)-C(8)	113.7(5)
H(15b)-C(15)-H(15a)	106.3(7)	H(15c)-C(15)-C(8)	109.0(5)
H(15c)-C(15)-H(15a)	109.3(7)	H(15c)-C(15)-H(15b)	107.1(7)
H(16a)-C(16)-C(12)	108.9(5)	H(16b)-C(16)-C(12)	111.7(5)
H(16b)-C(16)-H(16a)	109.0(8)	H(16c)-C(16)-C(12)	111.5(5)
H(16c)-C(16)-H(16a)	106.8(7)	H(16c)-C(16)-H(16b)	108.8(8)

7 References

1. Armarego, W. L. F. & Chai, C. L. L. *Purification of Laboratory Chemicals 8th edition.* (2017).
2. Burfield, D. R. & Smithers, R. H. Desiccant Efficiency in Solvent Drying. 3. Dipolar Aprotic Solvents. *J. Org. Chem.* **43,** 3966–3968 (1978).
3. Still, W. C., Kahn, M. & Mitra, A. Rapid Chromatographic Technique for Preparative Separations with Moderate Resolution. *J. Org. Chem.* **43,** 2923–2925 (1978).
4. Gottlieb, H. E., Kotlyar, V. & Nudelman, A. NMR Chemical Shifts of Common Laboratory Solvents as Trace Impurities. *J. Org. Chem.* **62,** 7512–7515 (1997).
5. Fulmer, G. R., Miller, A. J. M., Sherden, N. H., Gottlieb, H. E., Nudelman, A., Stoltz, B. M., Bercaw, J. E. & Goldberg, K. I. NMR Chemical Shifts of Trace Impurities: Common Laboratory Solvents, Organics, and Gases in Deuterated Solvents Relevant to the Organometallic Chemist. *Organometallics* **29,** 2176–2179 (2010).
6. Harris, R. K., Becker, E. D., Cabral De Menezes, S. M., Granger, P., Hoffman, R. E. & Zilm, K. W. Further conventions for NMR shielding and chemical shifts (IUPAC Recommendations 2008). *Pure Appl. Chem.* **80,** 59–84 (2008).
7. Sheldrick, G. M. A short history of SHELX. *Acta Crystallogr. Sect. A Found. Crystallogr.* **64,** 112–122 (2008).
8. Sheldrick, G. M. Crystal structure refinement with SHELXL. *Acta Crystallogr. Sect. C Struct. Chem.* **71,** 3–8 (2015).
9. Sheldrick, G. M. *SHELXT* – Integrated space-group and crystal-structure determination. *Acta Crystallogr. Sect. A Found. Adv.* **71,** 3–8 (2015).
10. Dolomanov, O. V., Bourhis, L. J., Gildea, R. J., Howard, J. A. K. & Puschmann, H. OLEX2: A complete structure solution, refinement and analysis program. *J. Appl. Crystallogr.* **42,** 339–341 (2009).
11. Kleemiss, F., Dolomanov, O. V., Bodensteiner, M., Peyerimhoff, N., Midgley, L., Bourhis, L. J., Genoni, A., Malaspina, L. A., Jayatilaka, D., Spencer, J. L., White, F., Grundkötter-Stock, B., Steinhauer, S., Lentz, D., Puschmann, H. & Grabowsky, S. Accurate crystal structures and chemical properties from NoSpherA2. *Chem. Sci.* **12,** 1675–1692 (2021).
12. Macrae, C. F., Bruno, I. J., Chisholm, J. A., Edgington, P. R., McCabe, P., Pidcock, E., Rodriguez-Monge, L., Taylor, R., Van De Streek, J. & Wood, P. A. Mercury CSD 2.0 - New features for the visualization and investigation of crystal structures. *J. Appl. Crystallogr.* **41,** 466–470 (2008).
13. The Nomenclature of Steroids. *Eur. J. Biochem.* **10,** 1–19 (2005).
14. Ishihara, K., Ishibashi, H. & Yamamoto, H. Enantio- and Diastereoselective Stepwise Cyclization of Polyprenoids Induced by Chiral and Achiral LBAs. A New Entry to (−)-Ambrox, (+)-Podocarpa-8,11,13-triene Diterpenoids, and (−)-Tetracyclic Polyprenoid of Sedimentary Origin. *J. Am. Chem. Soc.* **124,** 3647–3655 (2002).
15. Snowden, R. L., Eichenberger, J. C., Linder, S. M., Sonnay, P., Vial, C. & Schulte-Elte, K. H. Internal Nucleophilic Termination in Biomimetic Acid Mediated Polyene Cyclizations: Stereochemical and Mechanistic Implications. Synthesis of (±)-Ambrox And Its Diastereoisomers. *J. Org. Chem.* **57,** 955–960 (1992).
16. Schelwies, M., Paciello, R., Pelzer, R., Siegel, W. & Breuer, M. Palladium-Catalyzed Low Pressure Carbonylation of Allylic Alcohols by Catalytic Anhydride Activation. *Chem. – A Eur. J.* **27,** 9263–9266 (2021).
17. Fieser, L. F. & Fieser, M. *Reagents for organic synthesis*. *Reagents Org. Synth.* **1,** (Wiley, 1967).
18. Baker, B. A., Bošković, Ž. V. & Lipshutz, B. H. (BDP)CuH: A “Hot” Stryker’s Reagent for Use in Achiral Conjugate Reductions. *Org. Lett.* **10,** 289–292 (2008).
19. Barrero, A. F., Altarejos, J., Alvarez-Manzaneda, E. J., Ramos, J. M. & Salido, S. Synthesis of (±)-ambrox from (E)-nerolidol and β-ionone via allylic alcohol [2,3] sigmatropic rearrangement. *J. Org. Chem.* **61,** 2215–2218 (1996).
20. Escher, S., Giersch, W., Niclass, Y., Bernardinelli, G. & Ohloff, G. Configuration-Odor Relationships in 5β-Ambrox. *Helv. Chim. Acta* **73,** 1935–1947 (1990).
21. Li, F. & Renata, H. A Chiral-Pool-Based Strategy to Access *trans-syn*-Fused Drimane Meroterpenoids: Chemoenzymatic Total Syntheses of Polysin, *N*-Acetyl-polyveoline and the

Chrodrimanins. *J. Am. Chem. Soc.* **143,** 18280–18286 (2021).
22. Saito, A., Matsushita, H., Tsujino, Y. & Kaneko, H. Synthesis of (±)-Norambreinolide by Cyclization of Trans-β-Monocyclohomofarnesic acid. *Chem. Lett.* **10**, 757–760 (1981).
23. Lucius, G. Cyclisation homologer Sesquiterpensäuren, III. Über die säurekatalysierte Cyclisation der Homofarnesylsäure. *Chem. Ber.* **93,** 2663–2667 (1960).
24. Ohloff, G., Giersch, W., Pickenhagen, W., Furrer, A. & Frei, B. Significance of the Geminal Dimethyl Group in the Odor Principle of *Ambrox* ®. *Helv. Chim. Acta* **68,** 2022–2029 (1985).
25. Cortés, M., Armstrong, V., Reyes, M. E., Lopez, J. & Madariaga, E. Formal synthesis of Ambrox® and 9-epiambrox. *Synth. Commun.* **26,** 1995–2002 (1996).
26. Ohloff, G. & Giersch, W. Structure-Activity Relationships in Odor Perception of Drimane Derivatives. *Croat. Chem. Acta* **58**, 491–509 (1985).
27. Paquette, L. A. & Maleczka, R. E. Enantioselective total synthesis of (-)-9-epi-Ambrox, a potent ambergris-type olfactory agent. *J. Org. Chem.* **56,** 912–913 (1991).
28. Lee, S., Kaib, P. & List, B. N-Triflylphosphorimidoyl Trichloride: A Versatile Reagent for the Synthesis of Strong Chiral Brønsted Acids. *Synlett* **28,** 1478–1480 (2017).
29. Gatzenmeier, T., Turberg, M., Yepes, D., Xie, Y., Neese, F., Bistoni, G. & List, B. Scalable and Highly Diastereo- and Enantioselective Catalytic Diels-Alder Reaction of α,β-Unsaturated Methyl Esters. *J. Am. Chem. Soc.* **140,** 12671–12676 (2018).
30. Kaib, P. & List, B. Highly Acidic BINOL-Derived Phosphoramidimidates and their Application in the Brønsted Acid Catalyzed Synthesis of α-Tocopherol. *Synlett* **27,** 156–158 (2015).
31. Suwancharoen, S., Pornpakakul, S. & Muangsin, N. Synthesis of ent-ambrox® from (−)-nidorellol. *Tetrahedron Lett.* **53,** 5418–5421 (2012).
32. Menger, M. & Christmann, M. Formal synthesis of actinoranone using a one-pot semipinacol rearrangement/Wittig reaction. *Tetrahedron* **75,** 10–16 (2019).
33. Aricu, A. N., Kuchkova, K. I., Barba, A. N., Dragalin, I. P., Shova, S. G., Vornicu, N., Gorinchoi, E. K., Sekara, E. S., Lungu, L. V., Niculaua, M., Ungur, N. D. & Vlad, P. F. Synthesis from norambreinolide, structure, and antimicrobial activity of dihomodrimane sesquiterpenoids with azine, hydrazide, and dihydrazide fragments. *Chem. Nat. Compd.* **52,** 1029–1036 (2016).
34. Villamizar, J., Plata, F., Canudas, N., Tropper, E., Fuentes, J. & Orcajo, A. New Access to Sesquiterpene Hydroquinones: Synthesis of (+)- ent -Chromazonarol. *Synth. Commun.* **36,** 311–320 (2006).
35. Taguchi, H., Kawaguchi, M., Nagamitsu, T. & Ohtawa, M. Concise syntheses of (−)-habiterpenol and (+)-2,3- epi -habiterpenol via redox radical cyclization of alkenylsilane. *Org. Biomol. Chem.* **21,** 6129–6133 (2023).
36. Safety Data Sheet 1,1,1,3,3,3-hexafluoropropan-2-ol (HFIP). Available at: https://www.sigmaaldrich.com/DE/en/sds/aldrich/105228?userType=anonymous, retrieved: 2024-02-23.
37. *Test No. 305: Bioaccumulation in Fish: Aqueous and Dietary Exposure*. (OECD, 2012). Available at: doi:10.1787/9789264185296-en.
38. PBT Profiler. Available at: https://web.archive.org/web/20160501194153/http://www.pbtprofiler.net/criteria.asp, retrieved: 2024-02-23.
39. Usepa, Ocspp, Oppt & Rad. *Sustainable Futures / P2 Framework Manual 2012 EPA-748-B12-001 7. Estimating Persistence, Bioaccumulation, and Toxicity Using the PBT Profiler*. at <http://www.pbtprofiler.net/criteria.asp.>
40. *Sustainable Futures / P2 Framework Manual 2012 EPA-748-B12-001 7. Estimating Persistence, Bioaccumulation, and Toxicity Using the PBT Profiler*, retrieved: 2024-02-23.
41. Safety Data Sheet Perfluoro-*tert*-butanol (PFTB). Available at: https://store.apolloscientific.co.uk/product/perfluoro-*tert*-butanol, and https://www.sigmaaldrich.com/DE/en/sds/aldrich/331023?userType=anonymous, retrieved: 2024-03-06.
42. *OECD/UNEP Global PFC Group Synthesis paper on per- and polyfluorinated chemicals (PFCs)*. (2013).
43. Singleton, D. A. & Thomas, A. A. High-Precision Simultaneous Determination of Multiple

Small Kinetic Isotope Effects at Natural Abundance. *J. Am. Chem. Soc.* **117,** 9357–9358 (1995).
44. Arnold, A. M., Dullinger, P., Biswas, A., Jandl, C., Horinek, D. & Gulder, T. Enzyme-like polyene cyclizations catalyzed by dynamic, self-assembled, supramolecular fluoro alcohol-amine clusters. *Nat. Commun.* **14,** 813 (2023).
45. Masamune, S., Choy, W., Petersen, J. S. & Sita, L. R. Double Asymmetric Synthesis and a New Strategy for Stereochemical Control in Organic Synthesis. *Angew. Chemie Int. Ed.* **24,** 1–30 (1985).
46. Bannwarth, C., Ehlert, S. & Grimme, S. GFN2-xTB - An Accurate and Broadly Parametrized Self-Consistent Tight-Binding Quantum Chemical Method with Multipole Electrostatics and Density-Dependent Dispersion Contributions. *J. Chem. Theory Comput.* **15,** 1652–1671 (2019).
47. Pracht, P., Bohle, F. & Grimme, S. Automated exploration of the low-energy chemical space with fast quantum chemical methods. *Phys. Chem. Chem. Phys.* **22,** 7169–7192 (2020).
48. Neese, F. Software update: The ORCA program system—Version 5.0. *WIREs Comput. Mol. Sci.* **12**, (2022).
49. Grimme, S., Hansen, A., Ehlert, S. & Mewes, J.-M. r2SCAN-3c: A "Swiss army knife" composite electronic-structure method. *J. Chem. Phys.* **154,** (2021).
50. Eichkorn, K., Treutler, O., Öhm, H., Häser, M. & Ahlrichs, R. Auxiliary basis sets to approximate Coulomb potentials. *Chem. Phys. Lett.* **240,** 283–290 (1995).
51. Neese, F. An improvement of the resolution of the identity approximation for the formation of the Coulomb matrix. *J. Comput. Chem.* **24,** 1740–1747 (2003).
52. Weigend, F. Accurate Coulomb-fitting basis sets for H to Rn. *Phys. Chem. Chem. Phys.* **8,** 1057–1065 (2006).
53. Goddard, T. D., Huang, C. C., Meng, E. C., Pettersen, E. F., Couch, G. S., Morris, J. H. & Ferrin, T. E. UCSF ChimeraX: Meeting modern challenges in visualization and analysis. *Protein Sci.* **27,** 14–25 (2018).
54. Pettersen, E. F., Goddard, T. D., Huang, C. C., Meng, E. C., Couch, G. S., Croll, T. I., Morris, J. H. & Ferrin, T. E. UCSF ChimeraX: Structure visualization for researchers, educators, and developers. *Protein Sci.* **30,** 70–82 (2021).
55. de Grotthuss, C. J. T. Sur la décomposition de l'eau et des corps qu'elle tient en dissolution à l'aide de l'électricité galvanique. *Ann. Chim.* **LVIII,** 54–74 (1806).
56. Agmon, N. The Grotthuss mechanism. *Chem. Phys. Lett.* **244,** 456–462 (1995).
57. Kaufman, H. S. & Fankuchen, I. A Low Temperature Single Crystal X-Ray Diffraction Technique. *Rev. Sci. Instrum.* **20,** 733–734 (1949).
58. Brodalla, D., Mootz, D., Boese, R. & Osswald, W. Programmed crystal growth on a diffractometer with focused heat radiation. *J. Appl. Crystallogr.* **18,** 316–319 (1985).
59. Boese, R. Special issue on In Situ Crystallization. *Zeitschrift für Krist. - Cryst. Mater.* **229,** 595–601 (2014).
60. Bodach, A., Nöthling, N. & Felderhoff, M. Activation of Molecular Hydrogen by Inter- and Intramolecular Al−N Lewis Pairs. *Eur. J. Inorg. Chem.* **2021,** 1240–1243 (2021).
61. Seidel, R. W., Goddard, R., Nöthling, N. & Lehmann, C. W. In situ cryocrystallization and solid-state structures of furfural and some derivatives. *CrystEngComm* **21,** 3295–3303 (2019).
62. Buchsteiner, M., Martinez-Rodriguez, L., Jerabek, P., Pozo, I., Patzer, M., Nöthling, N., Lehmann, C. W. & Fürstner, A. Catalytic Asymmetric Fluorination of Copper Carbene Complexes: Preparative Advances and a Mechanistic Rationale. *Chem. – A Eur. J.* **26,** 2509–2515 (2020).
63. Patzer, M., Nöthling, N., Goddard, R. & Lehmann, C. W. Absolute Configuration of In Situ Crystallized (+)-γ-Decalactone. *Chemistry* **3,** 578–584 (2021).
