# Supplementary material for: The catalytic asymmetric polyene cyclization of homofarnesol to ambrox
Source: Nature. 2024 Jul 31;632(8026):795–801. doi: 10.1038/s41586-024-07757-7 (PMC11338820; doi:10.1038/s41586-024-07757-7)
Supplement: Supplementary file 2 — This file contains the NMR spectra data. [file 41586_2024_7757_MOESM2_ESM.pdf]

Copies of NMR Spectra

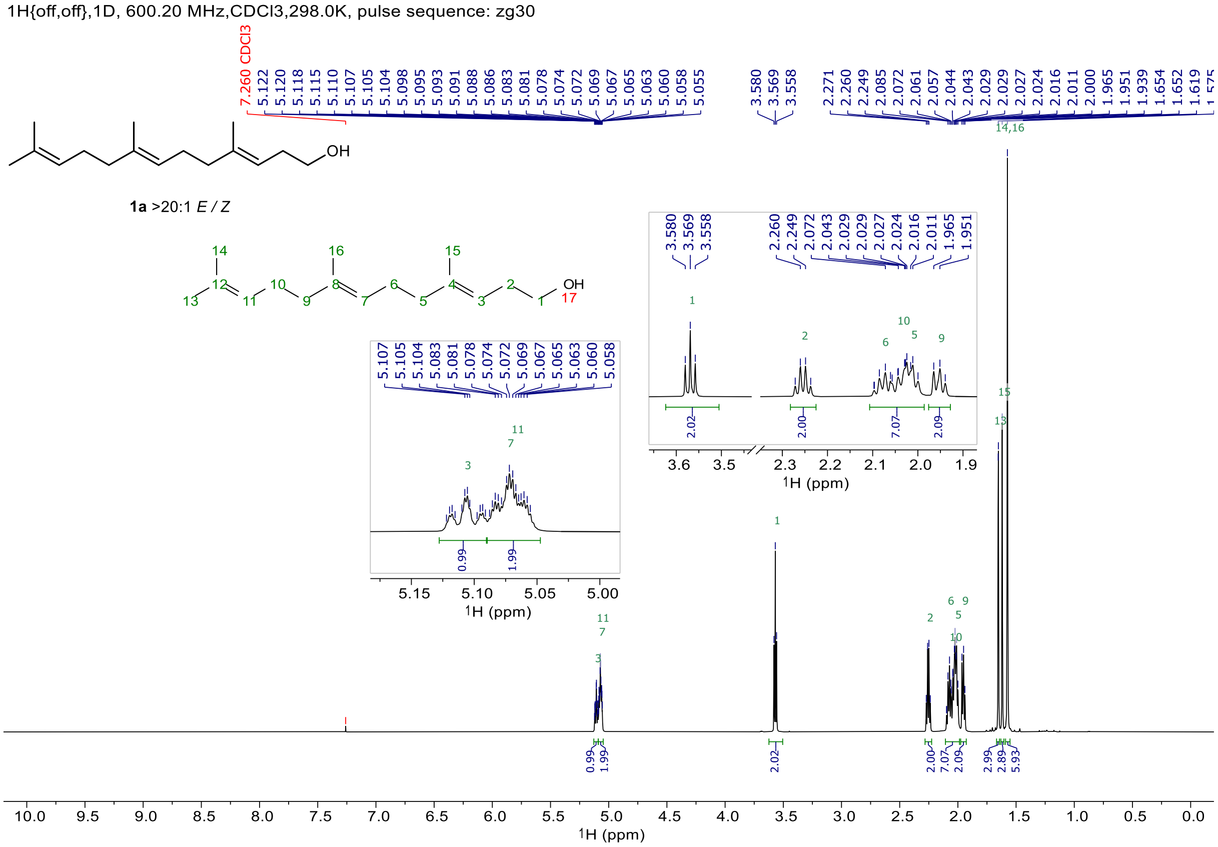

^{1}**H NMR** (600 MHz, $CDCl_3$) spectrum of (3*E*,7*E*)-homofarnesol **1a** with assignments

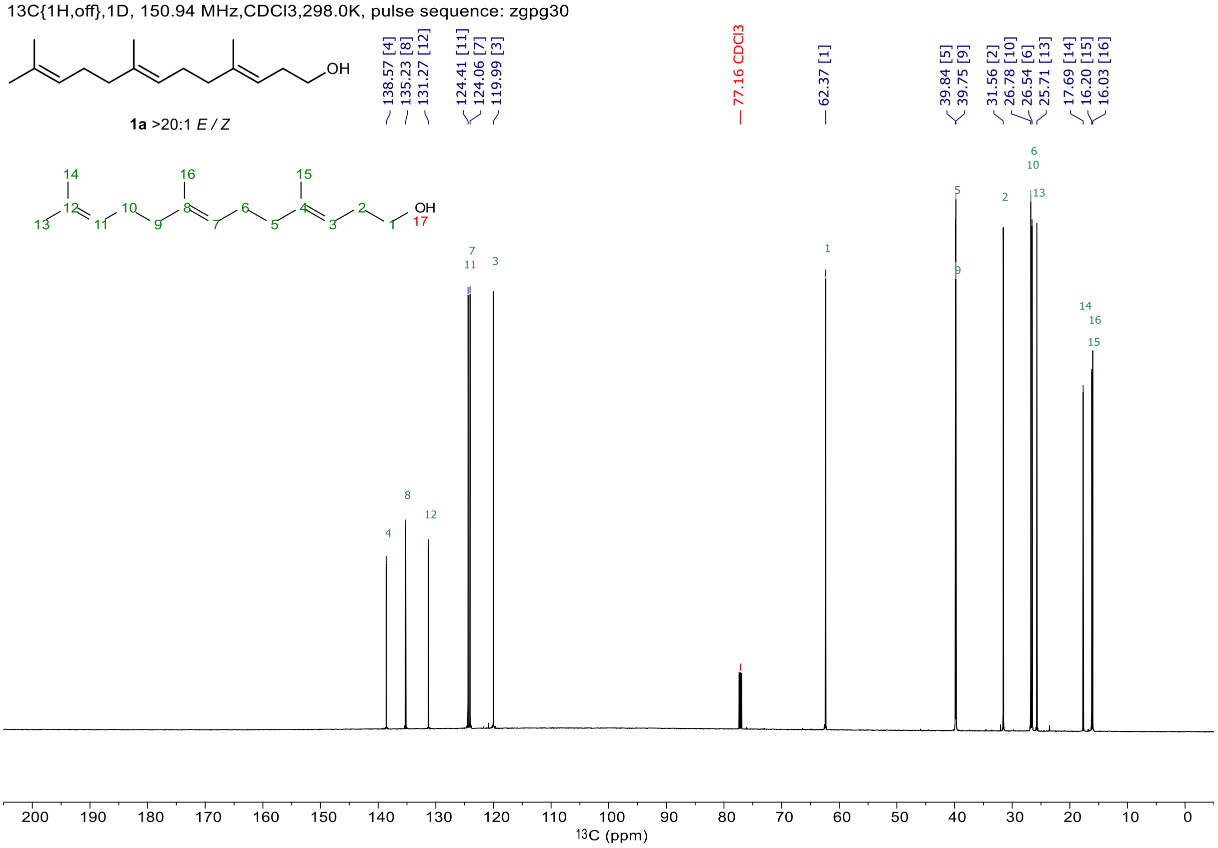

^{13}**C NMR** (151 MHz, $CDCl_3$) spectrum of (3*E*,7*E*)-homofarnesol **1a** with assignments

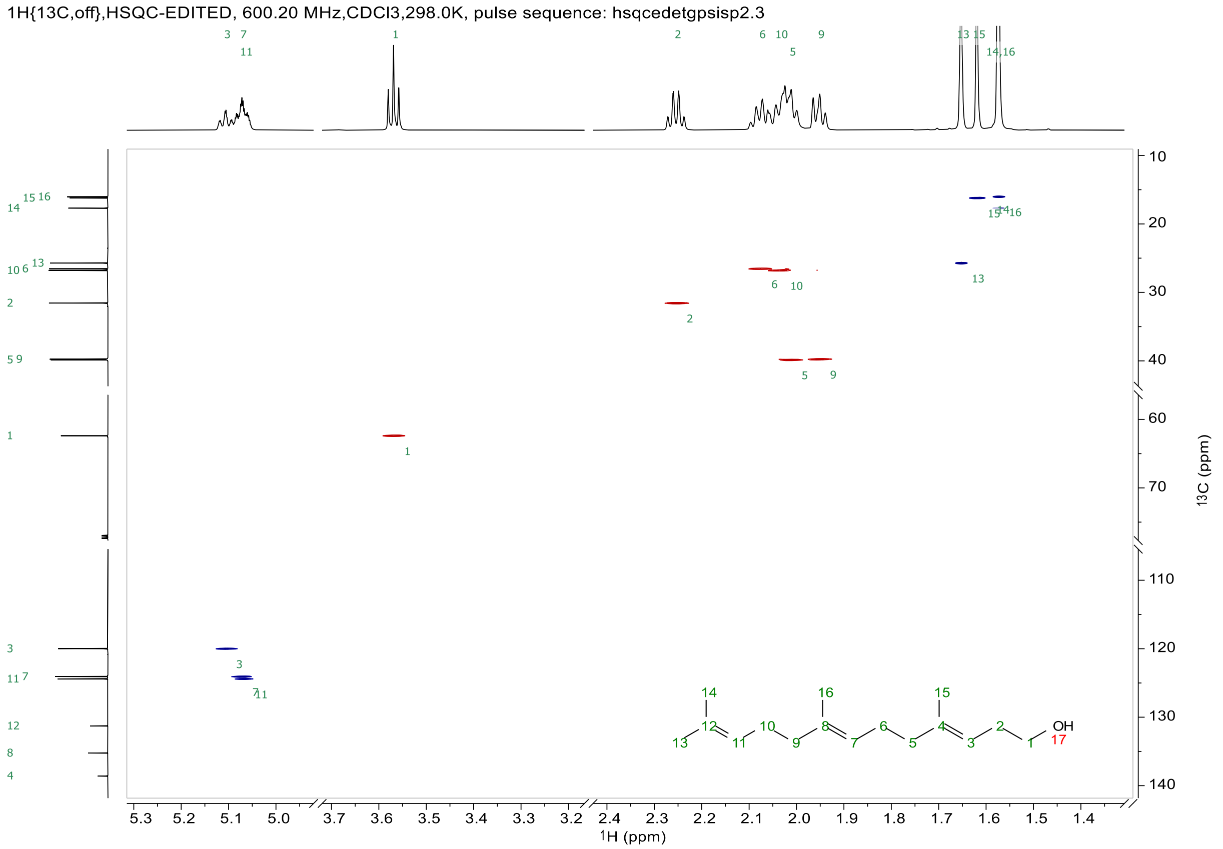

Multiplicity-edited **¹H,¹³C-HSQC** (600 MHz, 151 MHz, $CDCl_3$) spectrum of (3*E*,7*E*)-homofarnesol **1a**

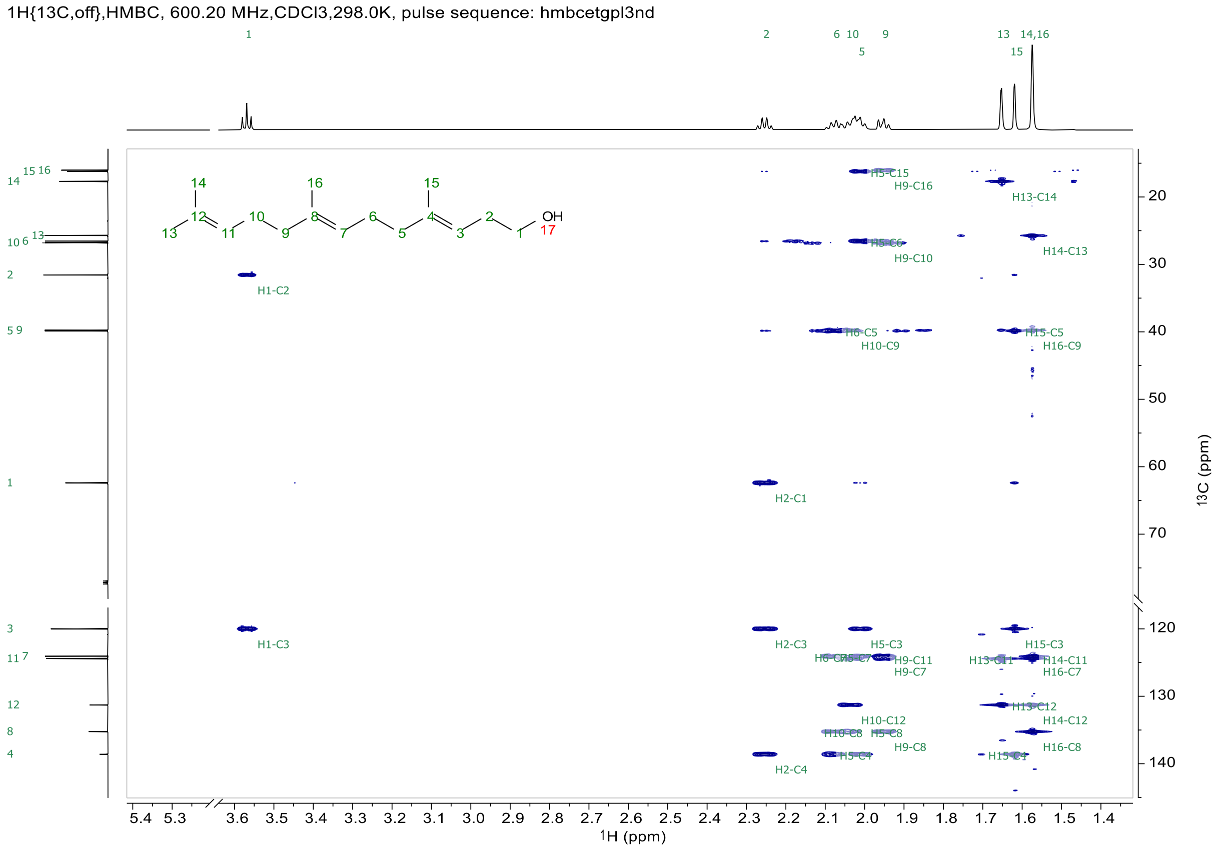

¹H,¹³C-HMBC (600 MHz, 151 MHz, $CDCl_3$) spectrum of (3*E*,7*E*)-homofarnesol **1a**

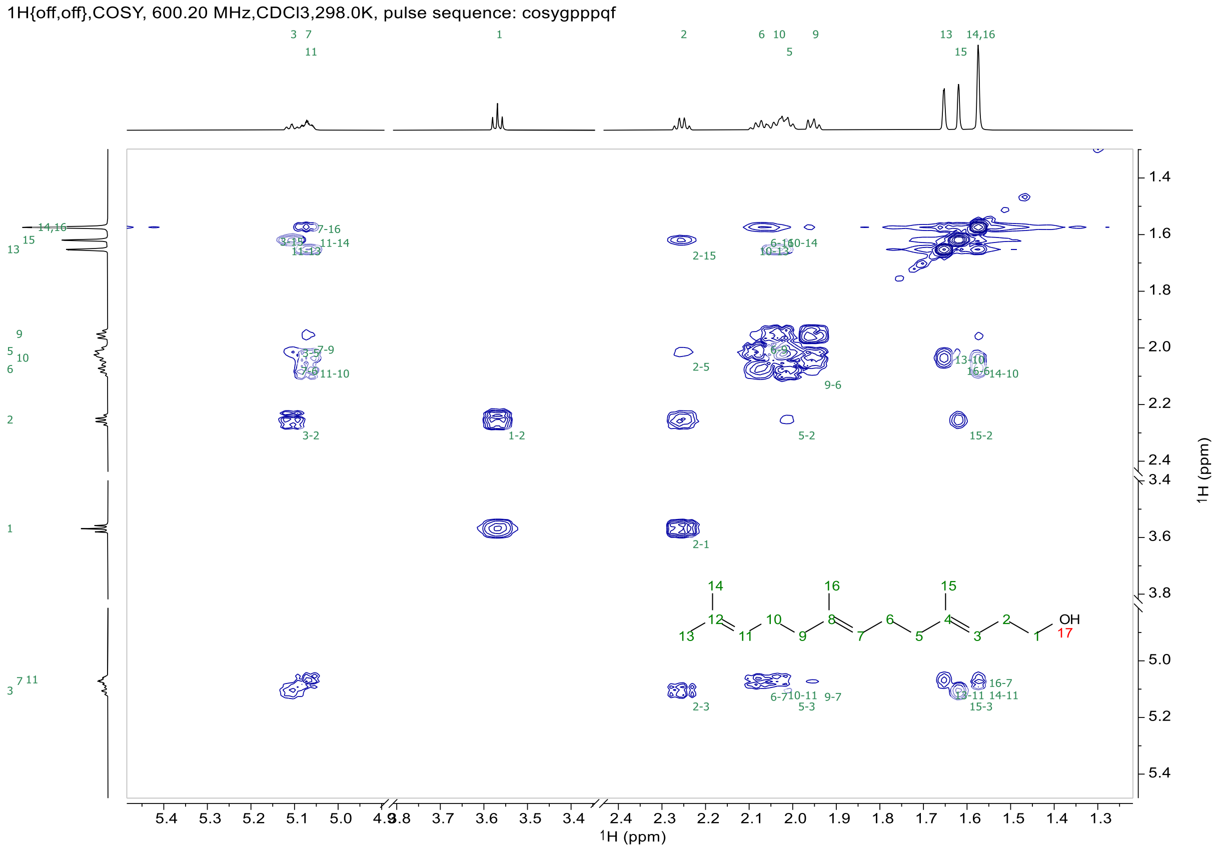

^{1}H,^{1}H-COSY (600 MHz, $CDCl_3$) spectrum of (3*E*,7*E*)-homofarnesol **1a**

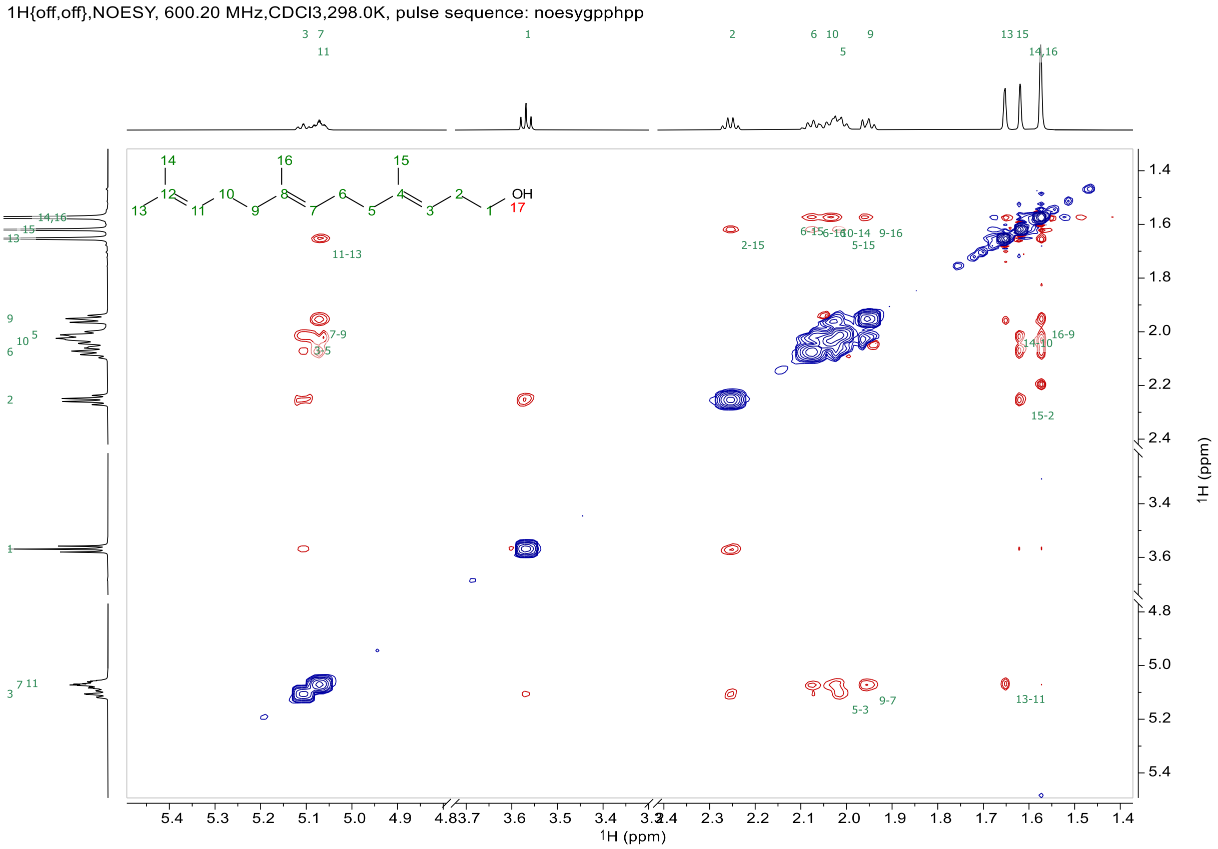

^{1}H,^{1}H-NOESY (600 MHz, $CDCl_3$) spectrum of (3*E*,7*E*)-homofarnesol **1a**

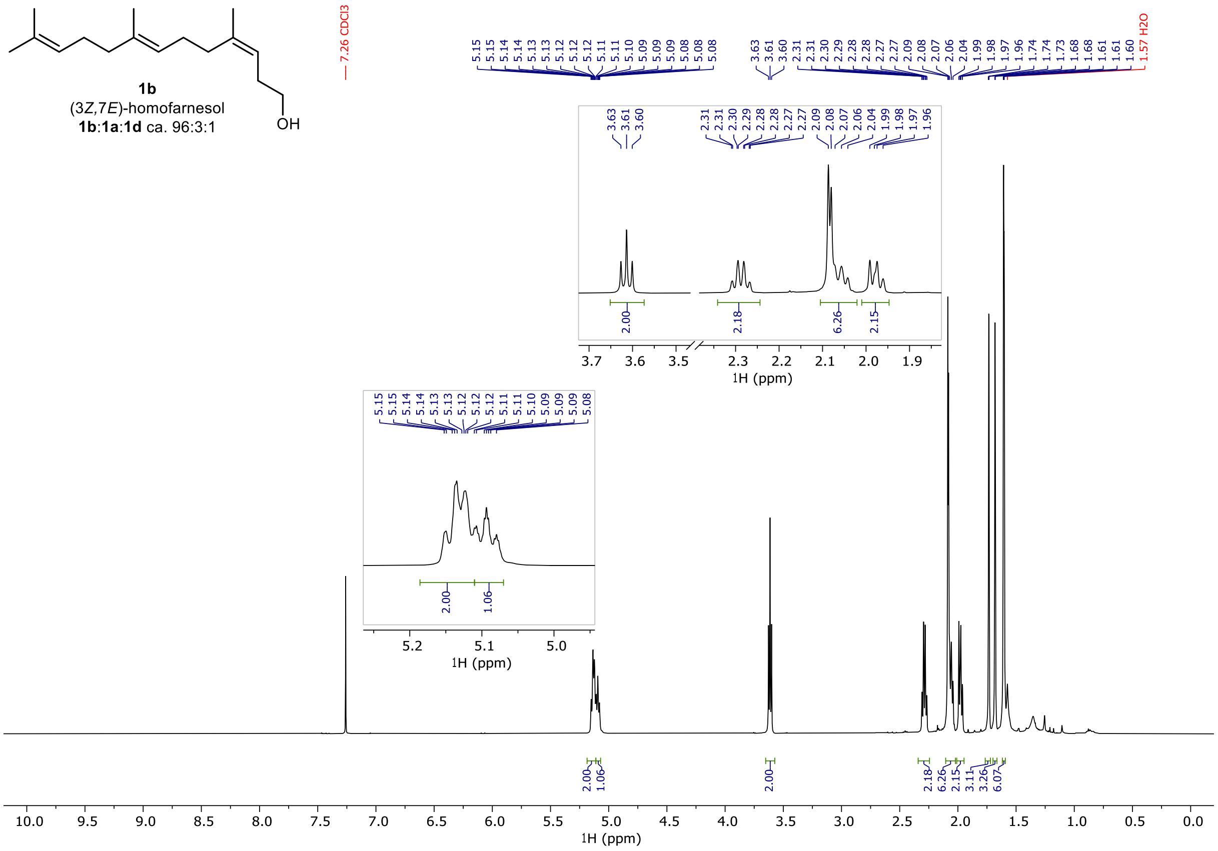

^{1}H NMR (501 MHz, $CDCl_3$) spectrum of (3*Z*,7*E*)-homofarnesol **1b**

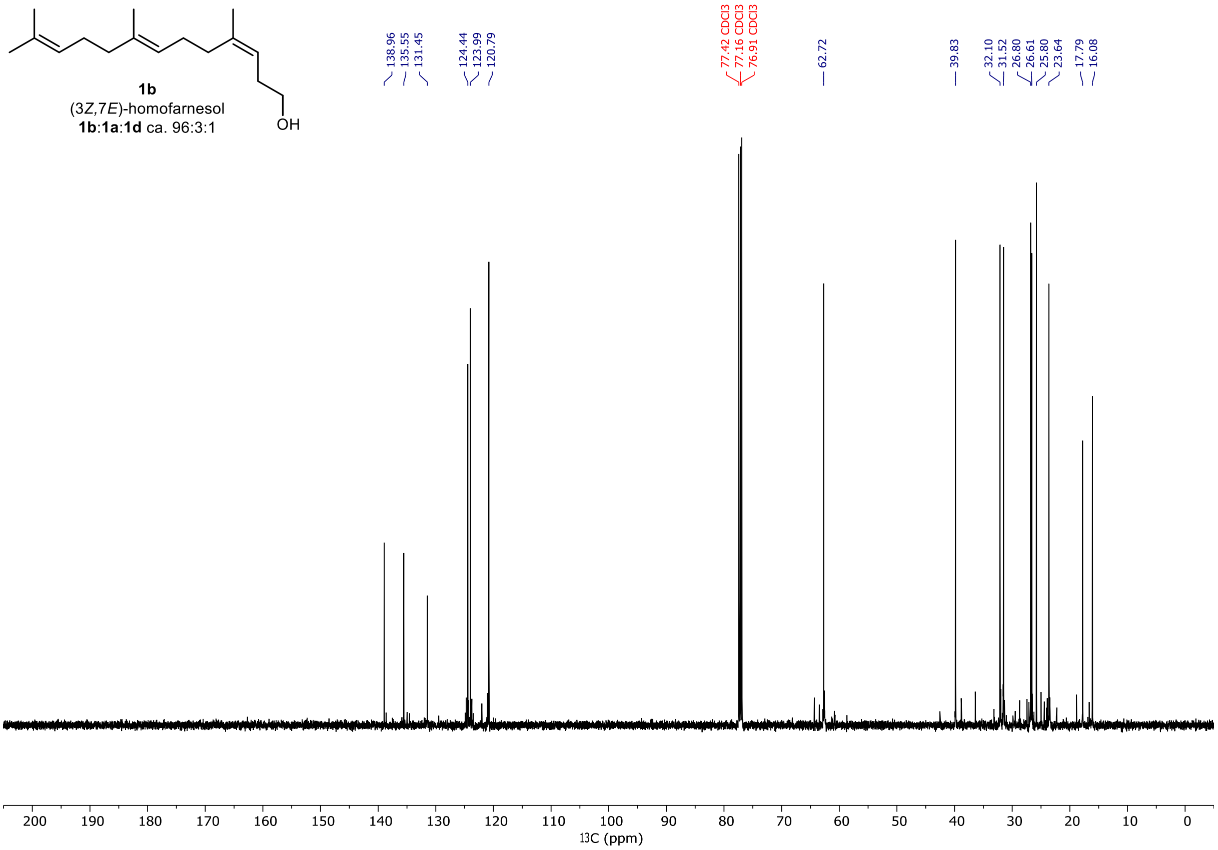

^{13}C NMR (126 MHz, $CDCl_3$) spectrum of (3*Z*,7*E*)-homofarnesol **1b**

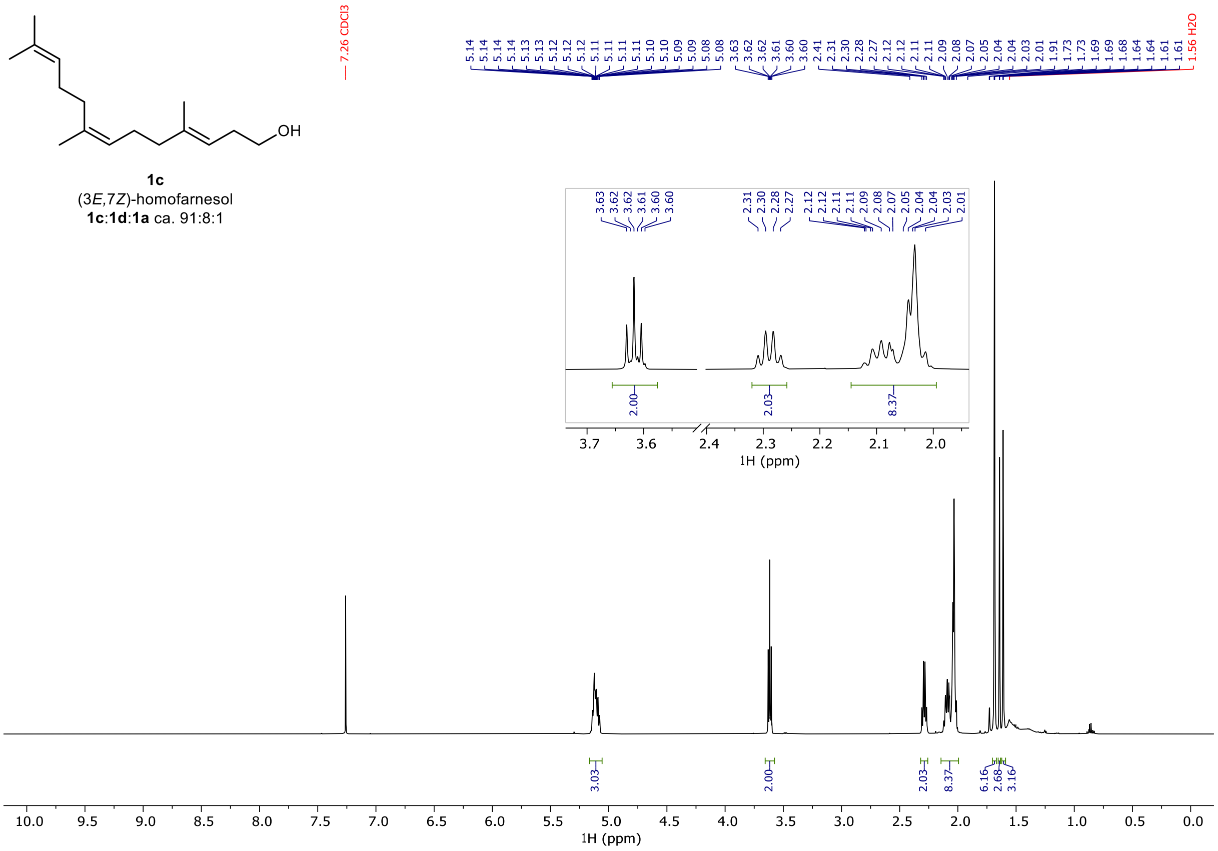

^{1}H NMR (501 MHz, $CDCl_3$) spectrum of (3*E*,7*Z*)-homofarnesol **1c**

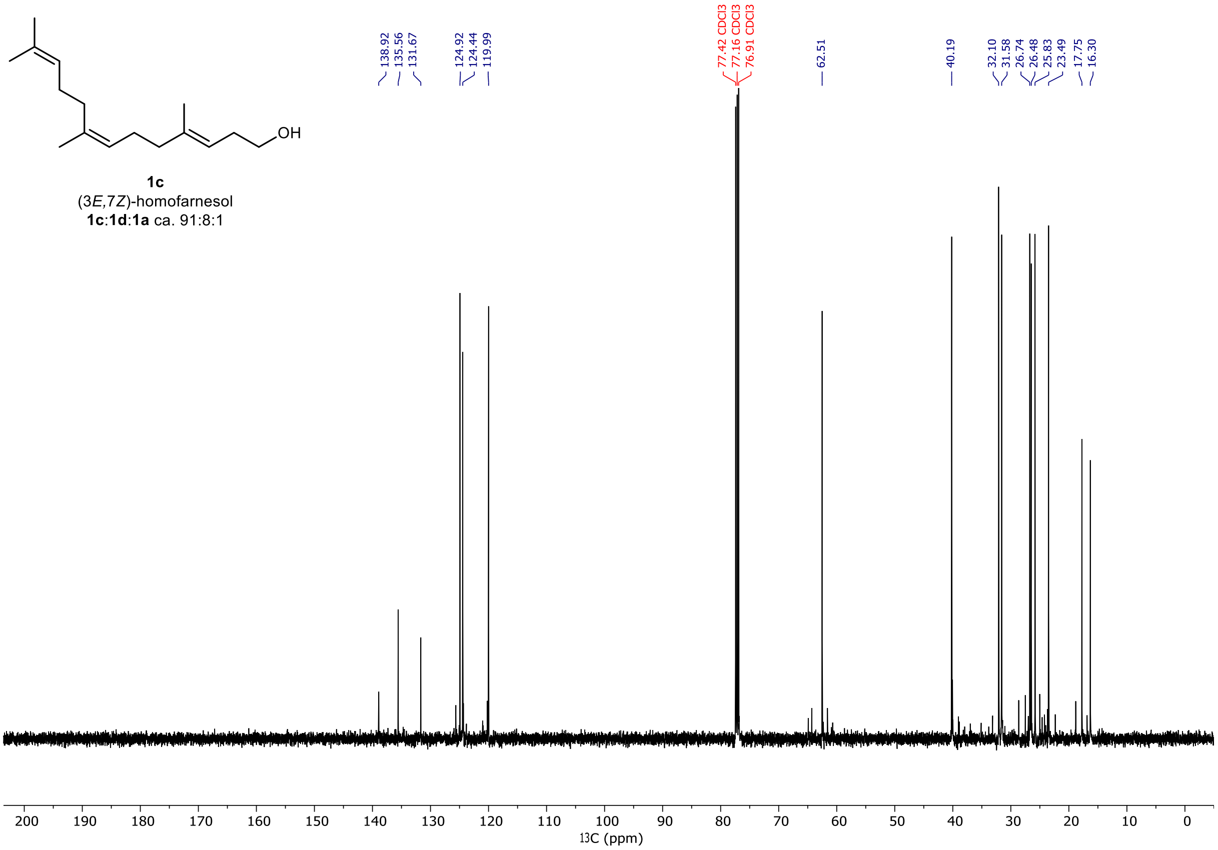

^{13}C NMR (126 MHz, $CDCl_3$) spectrum of (3*E*,7*Z*)-homofarnesol **1c**

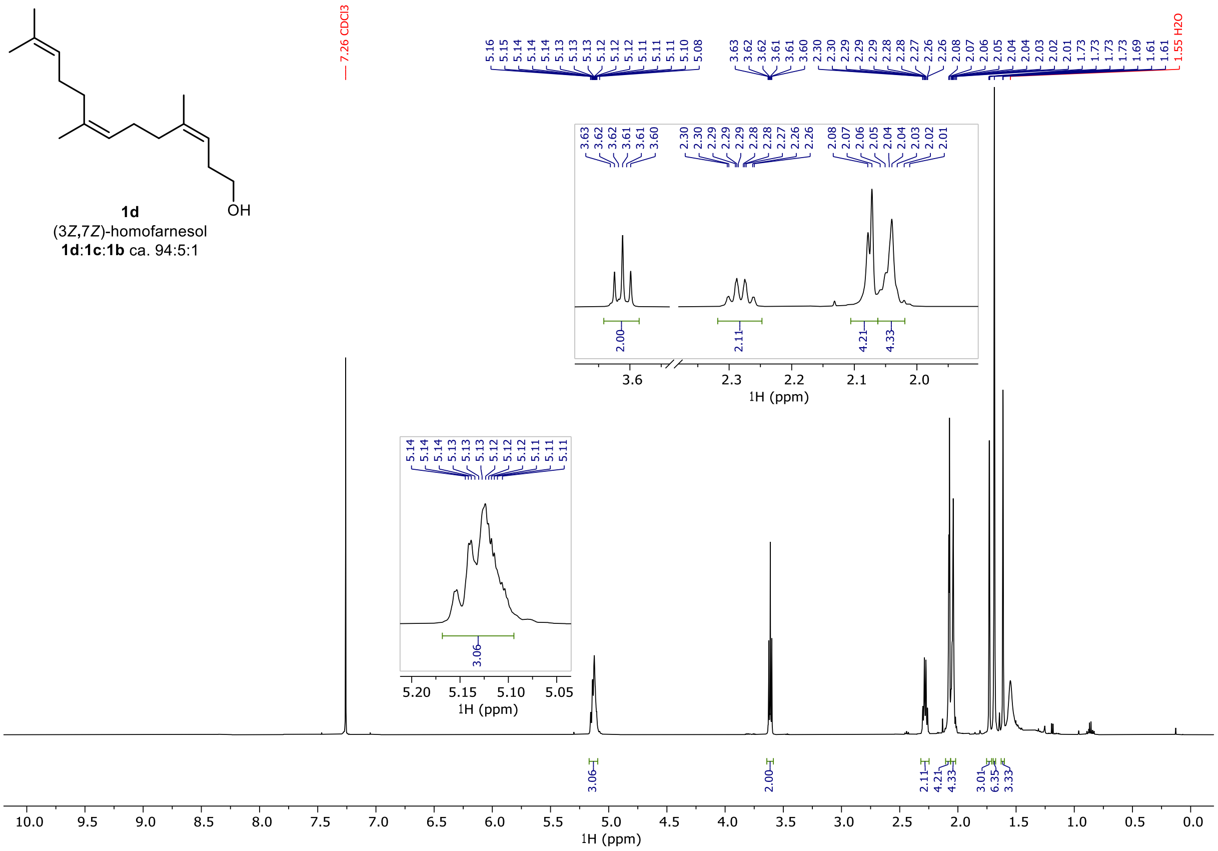

^{1}**H NMR** (501 MHz, CDCl$_3$) spectrum of (3*Z*,7*Z*)-homofarnesol **1d**

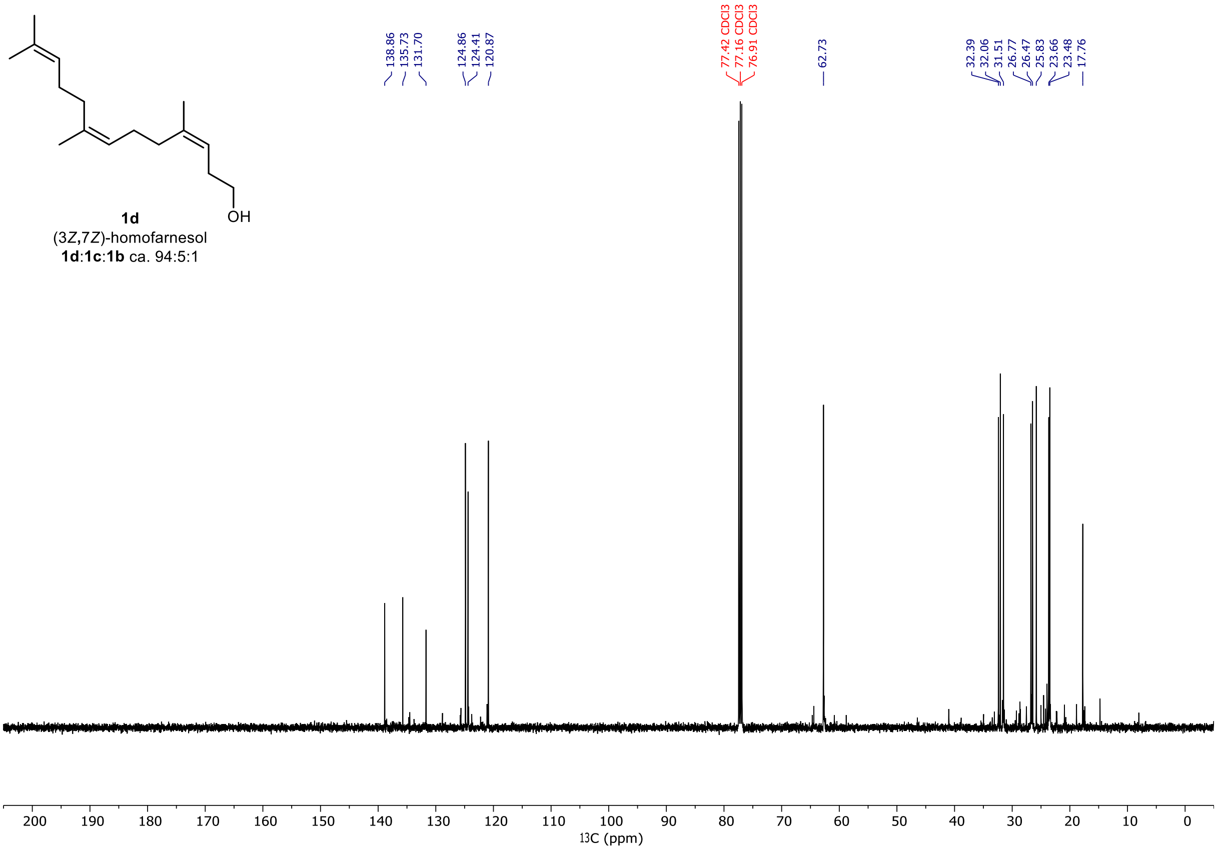

^{13}**C NMR** (126 MHz, CDCl$_3$) spectrum of (3*Z*,7*Z*)-homofarnesol **1d**

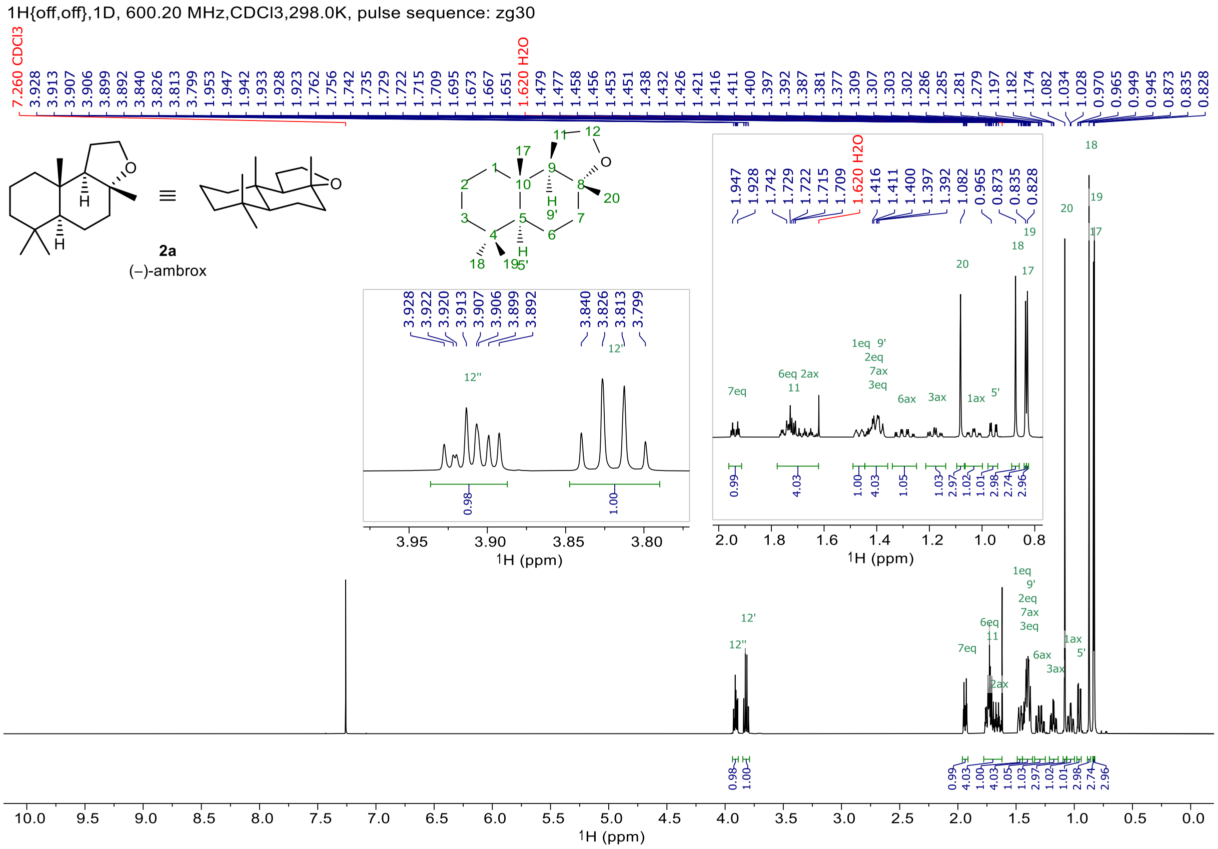

^{1}H NMR (600 MHz, $CDCl_3$) spectrum of authentic (−)-ambrox **2a** with assignments

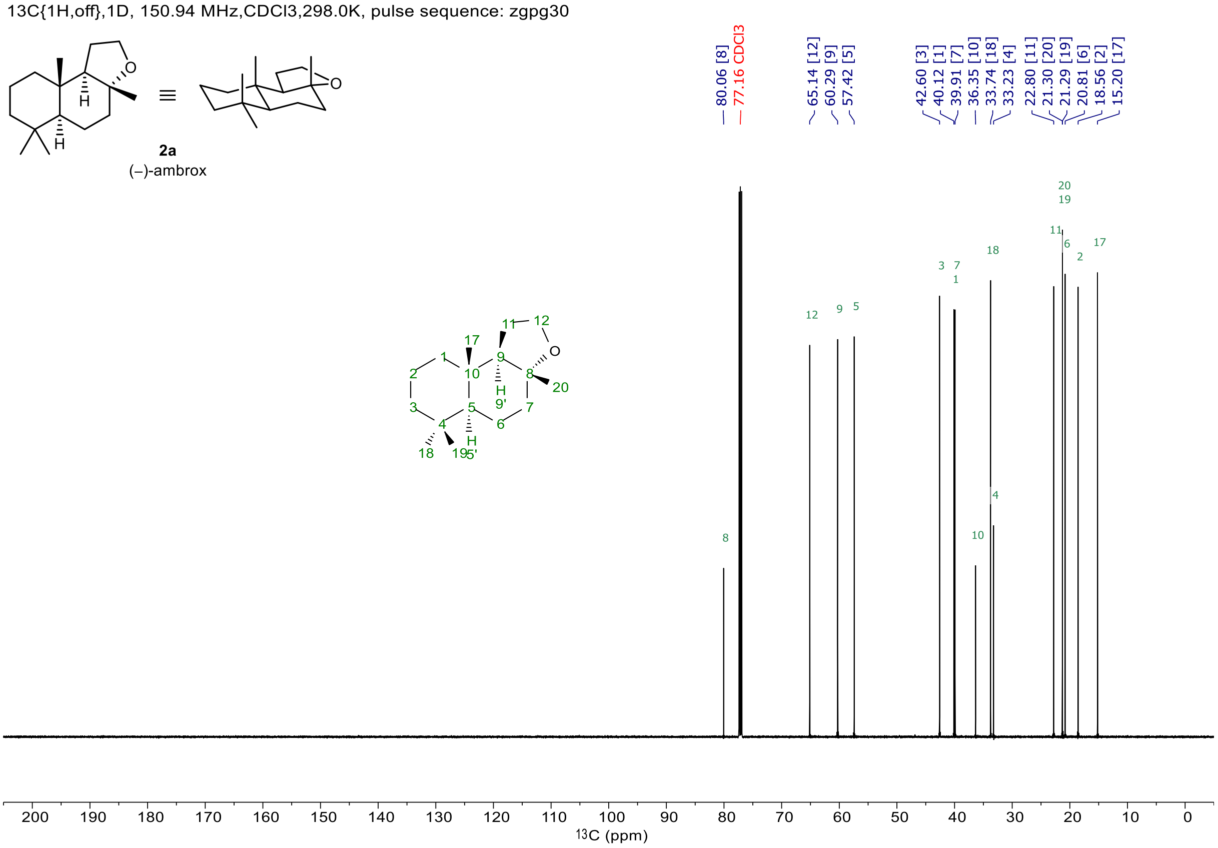

^{13}C NMR (151 MHz, $CDCl_3$) spectrum of authentic (−)-ambrox **2a** with assignments

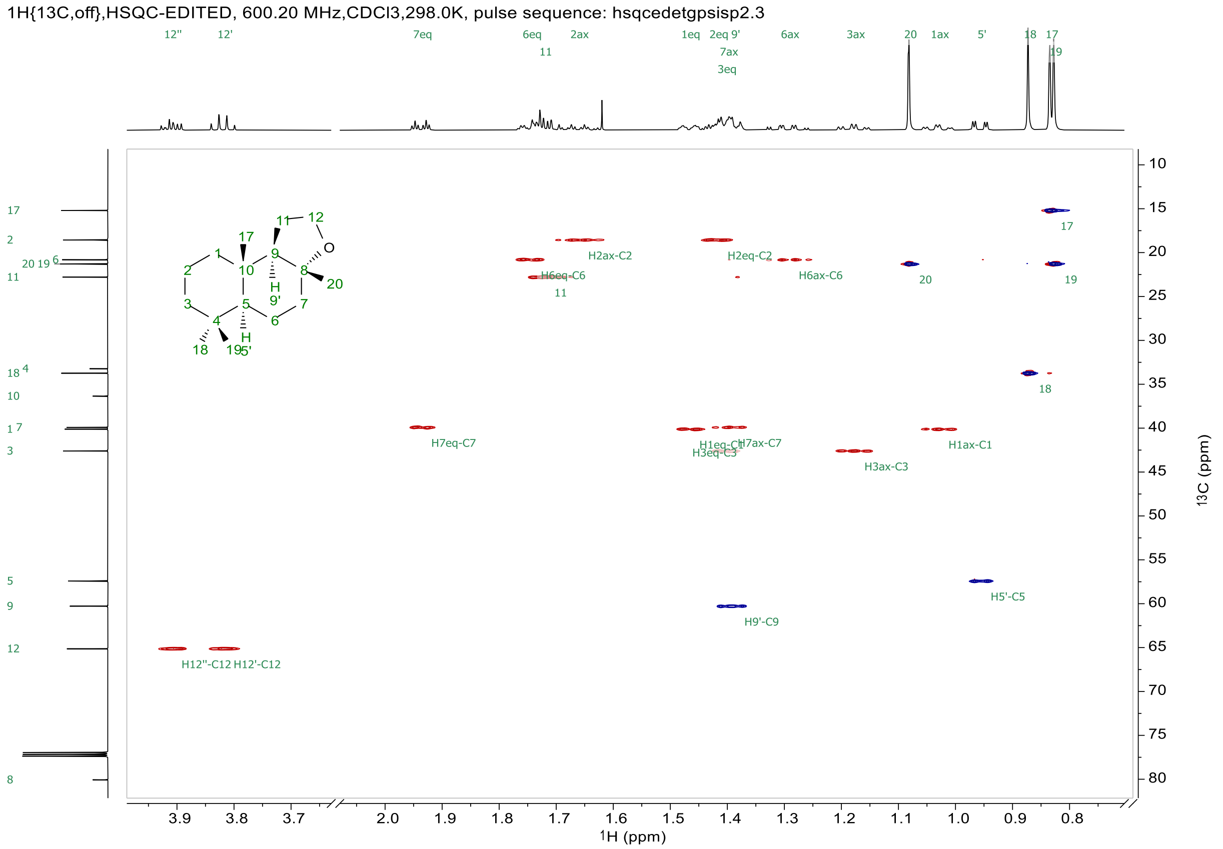

Multiplicity-edited **¹H,¹³C-HSQC** (600 MHz, 151 MHz, $CDCl_3$) spectrum of authentic (−)-ambrox **2a**

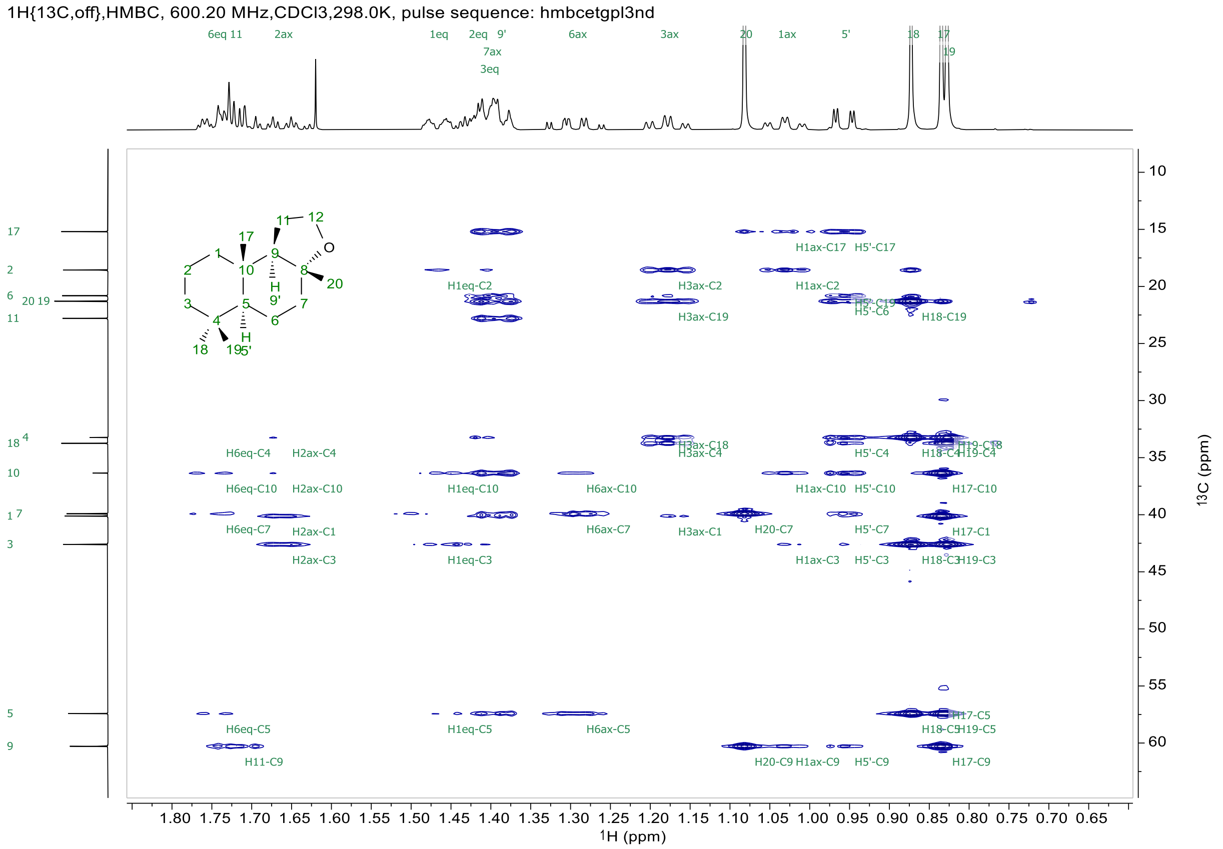

¹H,¹³C-HMBC (600 MHz, 151 MHz, $CDCl_3$) spectrum of authentic (−)-ambrox **2a**

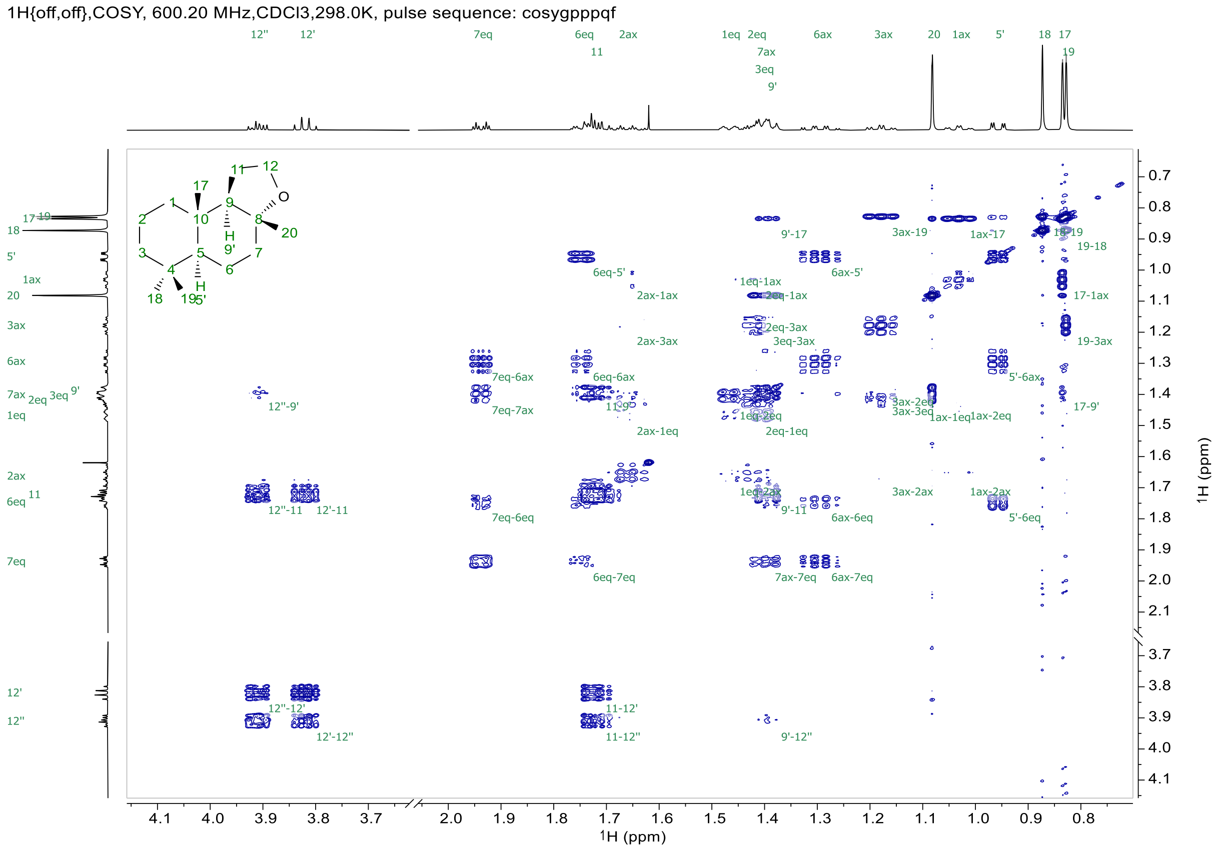

^{1}H,^{1}H-COSY (600 MHz, $CDCl_3$) spectrum of authentic (−)-ambrox **2a**

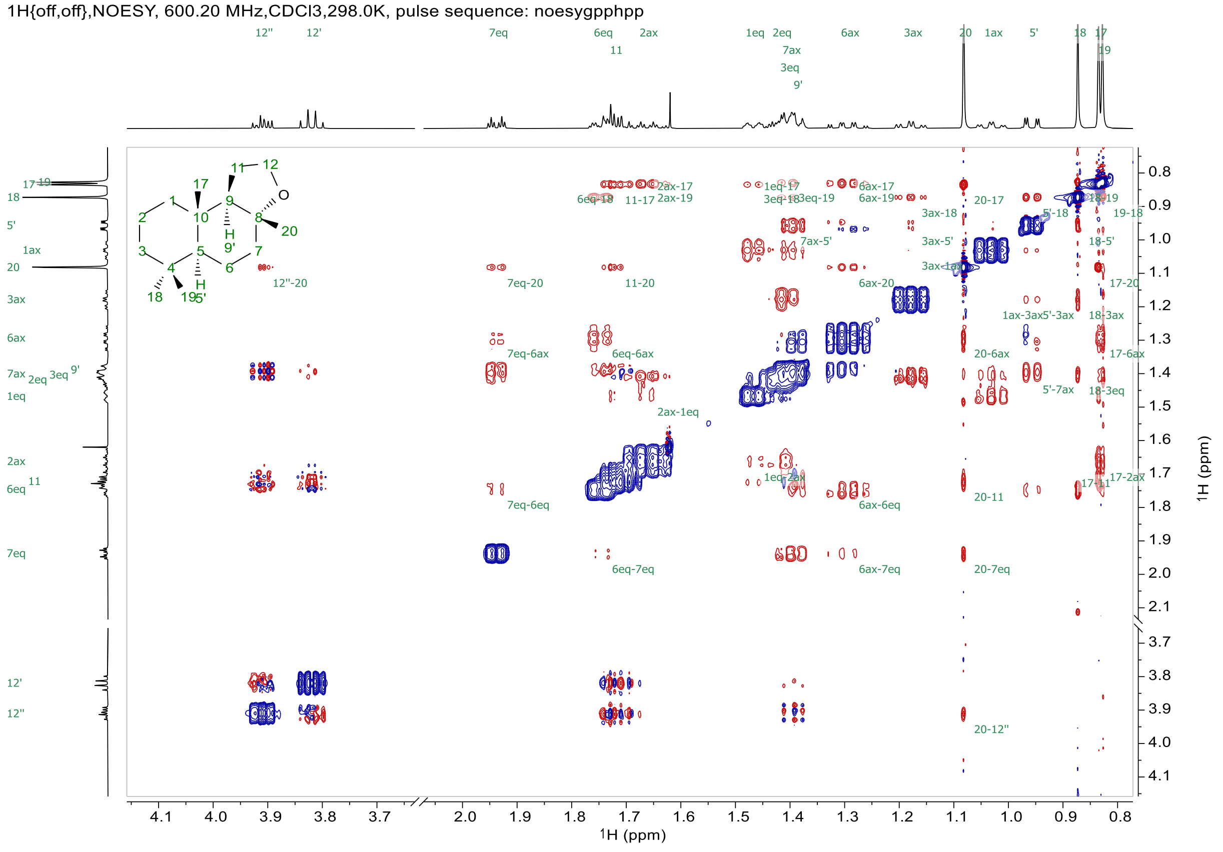

^{1}H,^{1}H-NOESY (600 MHz, $CDCl_3$) spectrum of authentic (−)-ambrox **2a**

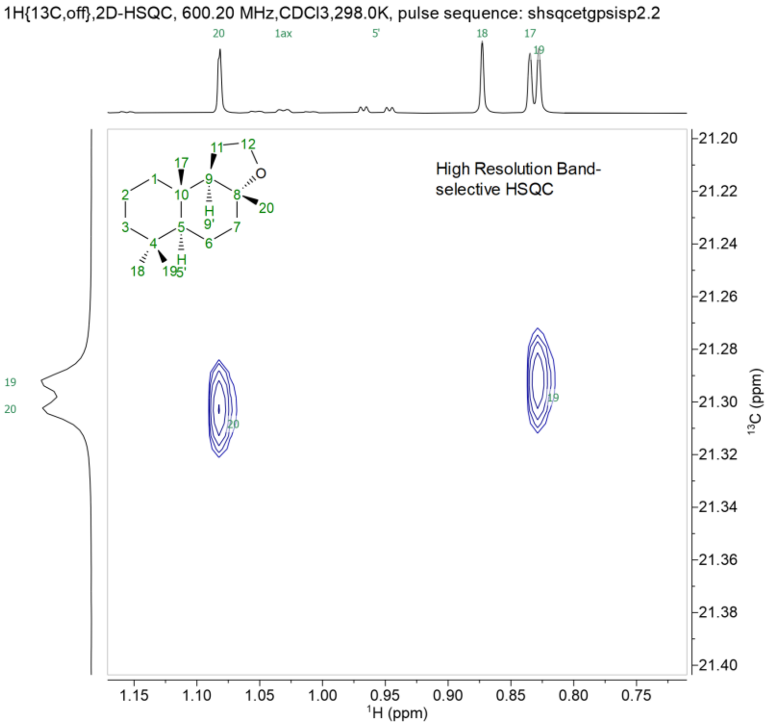

High resolution band-selective **^{1}H,^{13}C-HSQC** (600 MHz, 151 MHz, $CDCl_3$) spectrum of authentic (−)-ambrox **2a**

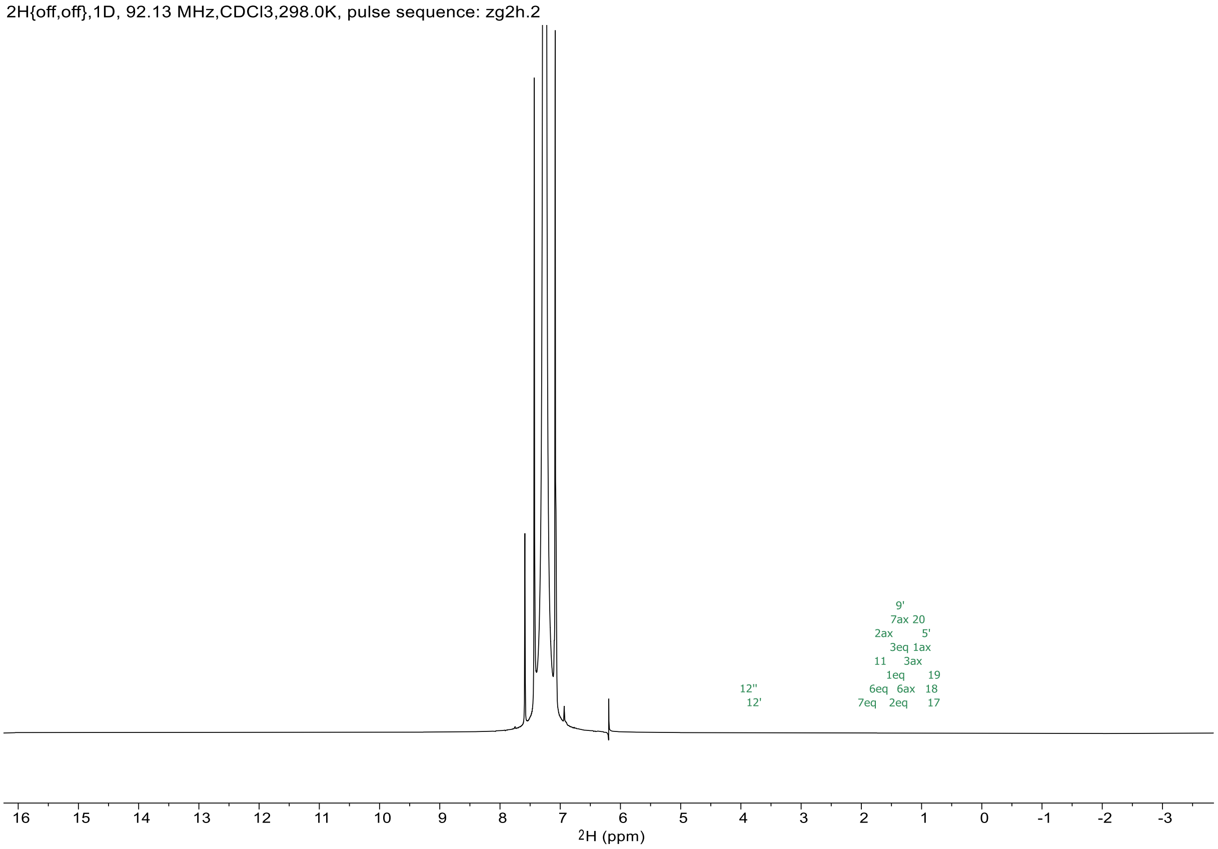

^{2}H NMR (92 MHz, $CDCl_3$) spectrum of authentic (−)-ambrox **2a**

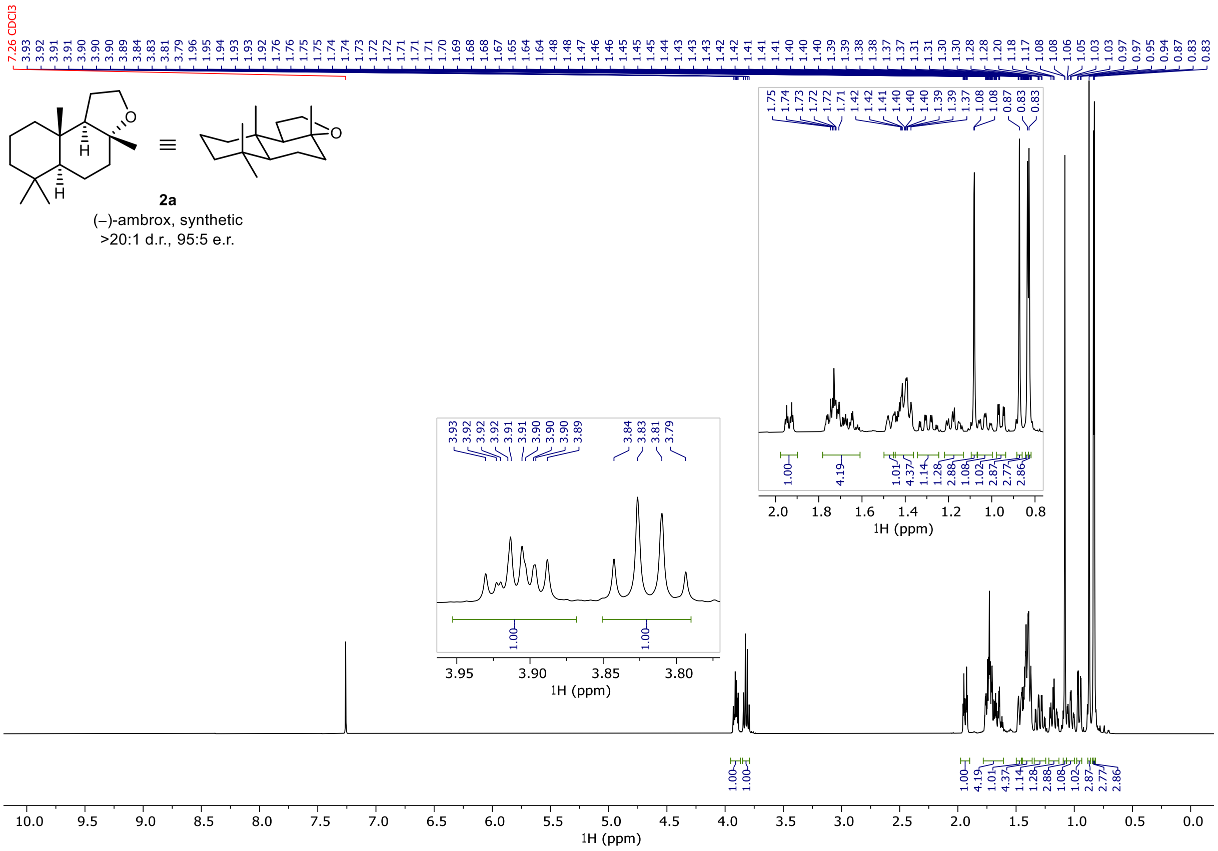

^{1}H NMR (501 MHz, $CDCl_3$) spectrum of synthetic (−)-ambrox **2a** obtained as major product in the IDPi-catalyzed polyene cyclization of (3*E*,7*E*)-homofarnesol to (−)-ambrox

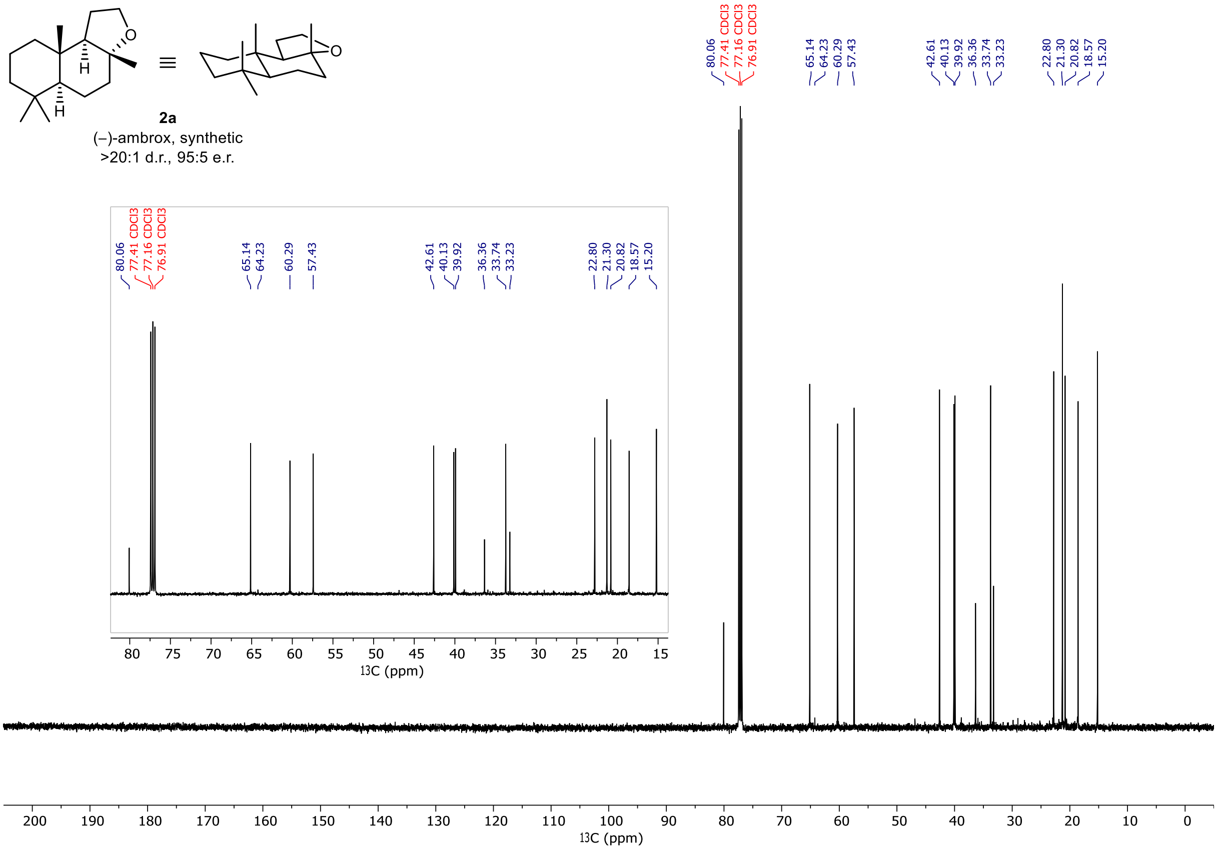

^{13}C NMR (126 MHz, $CDCl_3$) spectrum of synthetic (−)-ambrox **2a** obtained as major product in the IDPi-catalyzed polyene cyclization of (3*E*,7*E*)-homofarnesol to (−)-ambrox

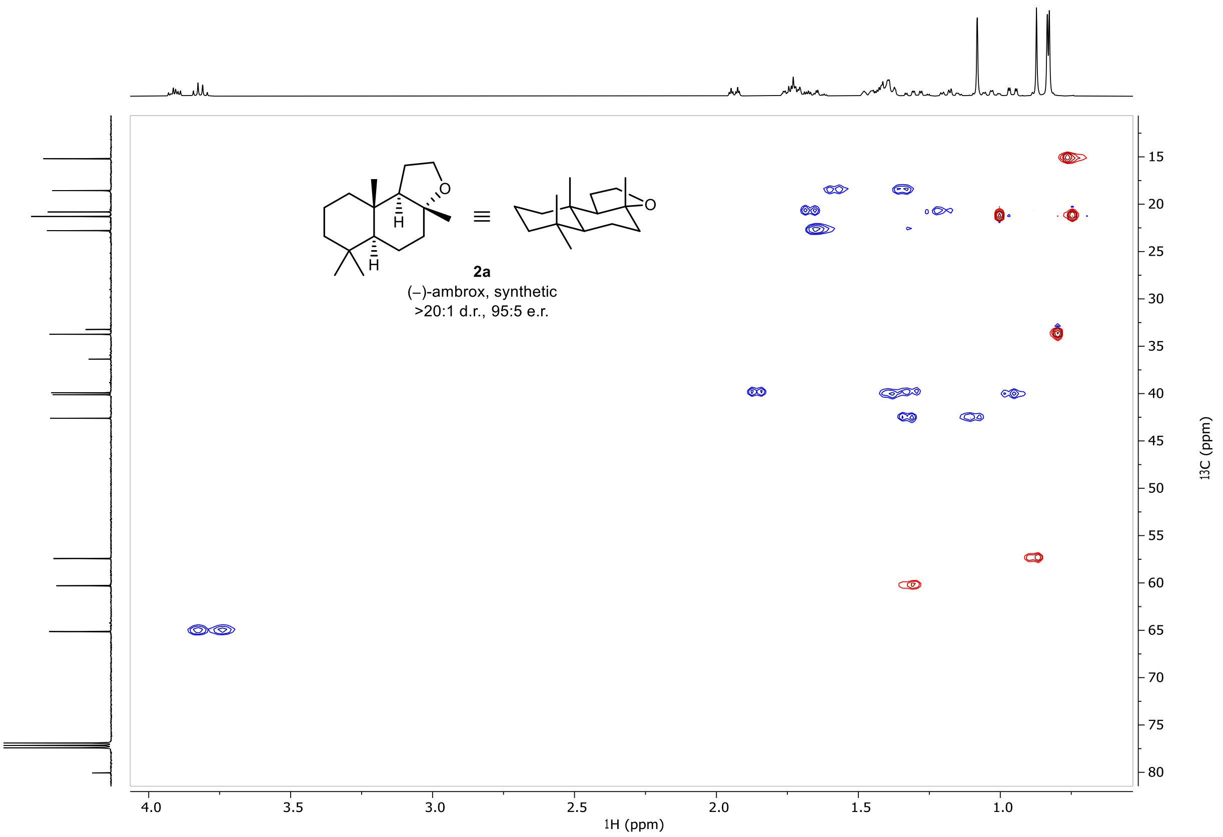

^{1}H,^{13}C-HSQC (501 MHz, 126 MHz, $CDCl_3$) spectrum of synthetic (−)-ambrox **2a** obtained as major product in the IDPi-catalyzed polyene cyclization of (3*E*,7*E*)-homofarnesol to (−)-ambrox

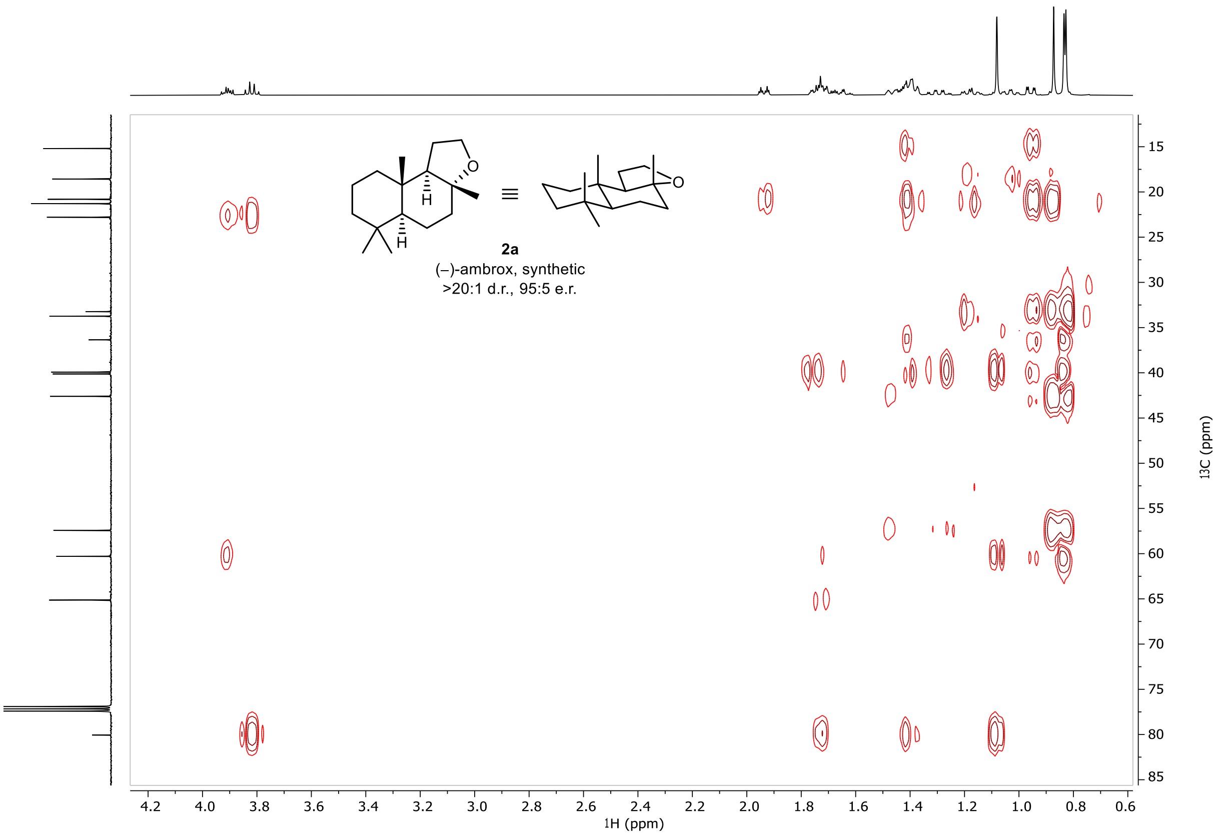

^{1}H,^{13}C-HMBC (501 MHz, 126 MHz, $CDCl_3$) spectrum of synthetic (−)-ambrox **2a** obtained as major product in the IDPi-catalyzed polyene cyclization of (3*E*,7*E*)-homofarnesol to (−)-ambrox

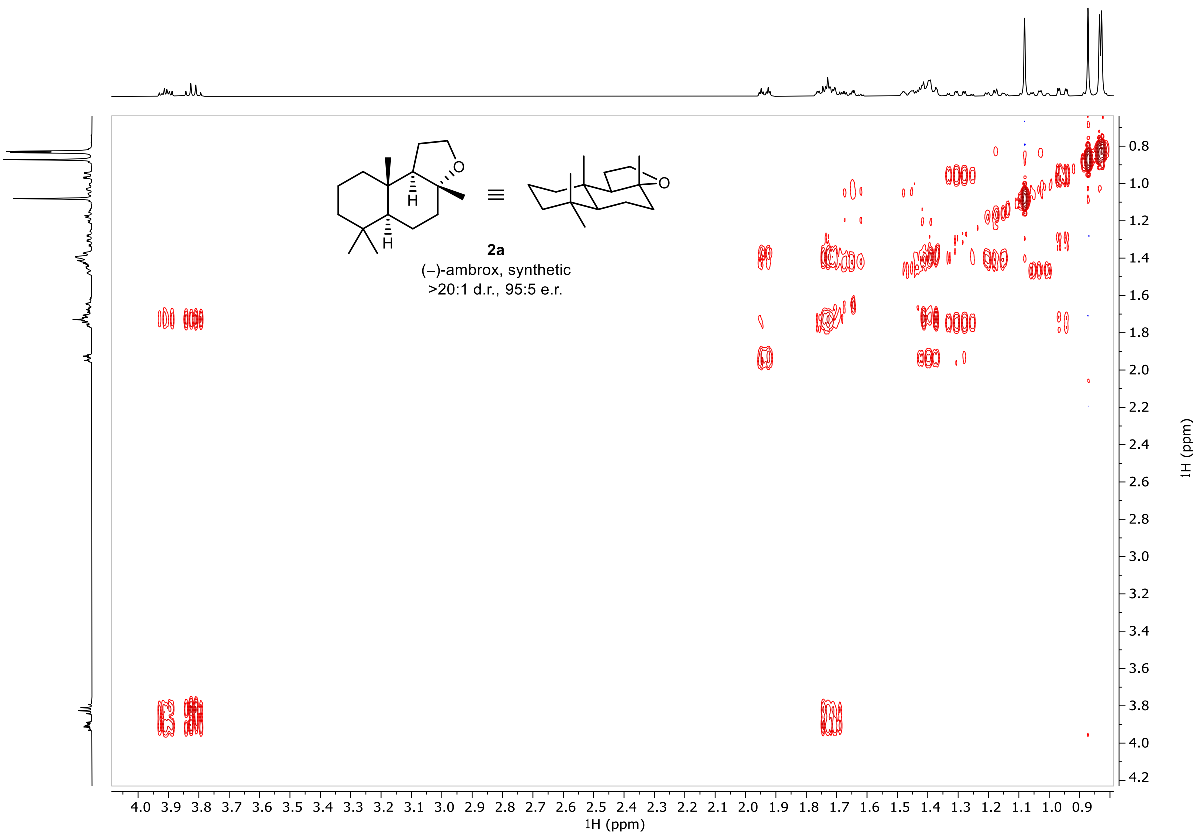

^{1}H,^{1}H-COSY (501 MHz, $CDCl_3$) spectrum of synthetic (−)-ambrox **2a** obtained as major product in the IDPi-catalyzed polyene cyclization of (3*E*,7*E*)-homofarnesol to (−)-ambrox

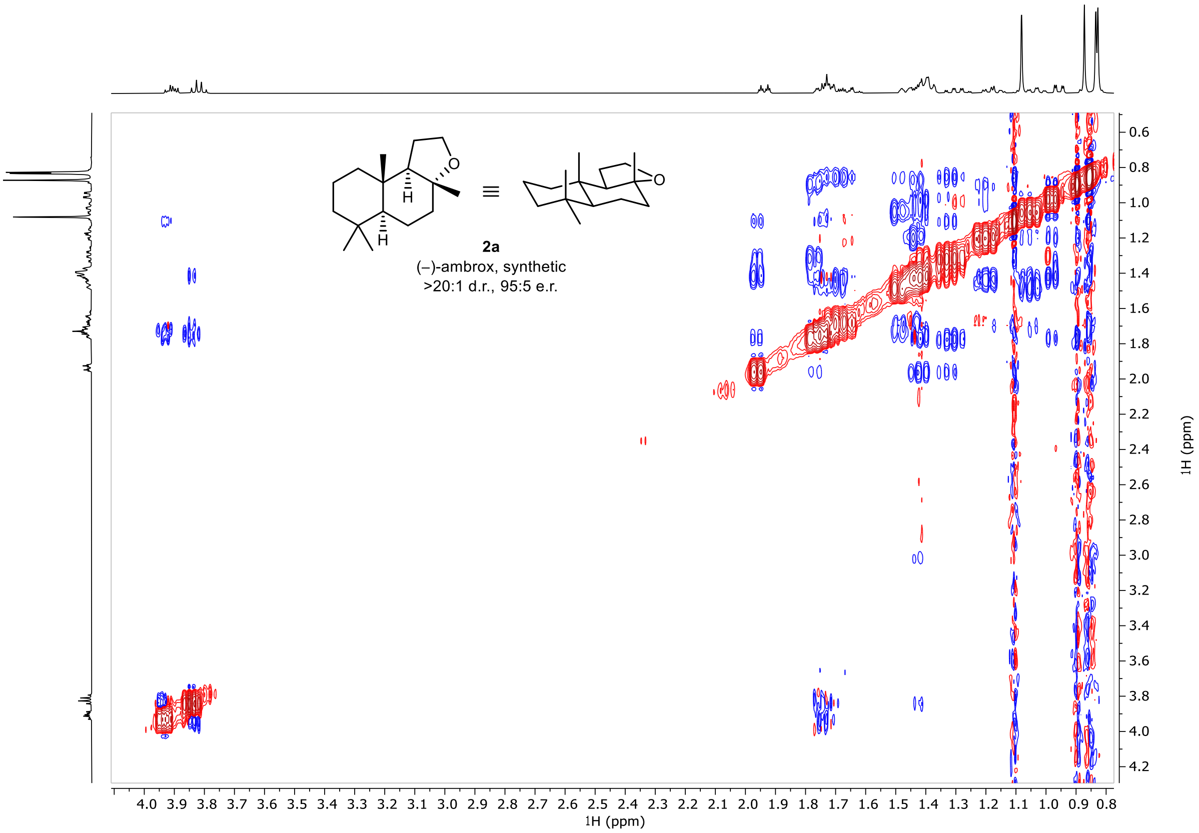

^{1}H,^{1}H-NOESY (501 MHz, $CDCl_3$) spectrum of synthetic (−)-ambrox **2a** obtained as major product in the IDPi-catalyzed polyene cyclization of (3*E*,7*E*)-homofarnesol to (−)-ambrox

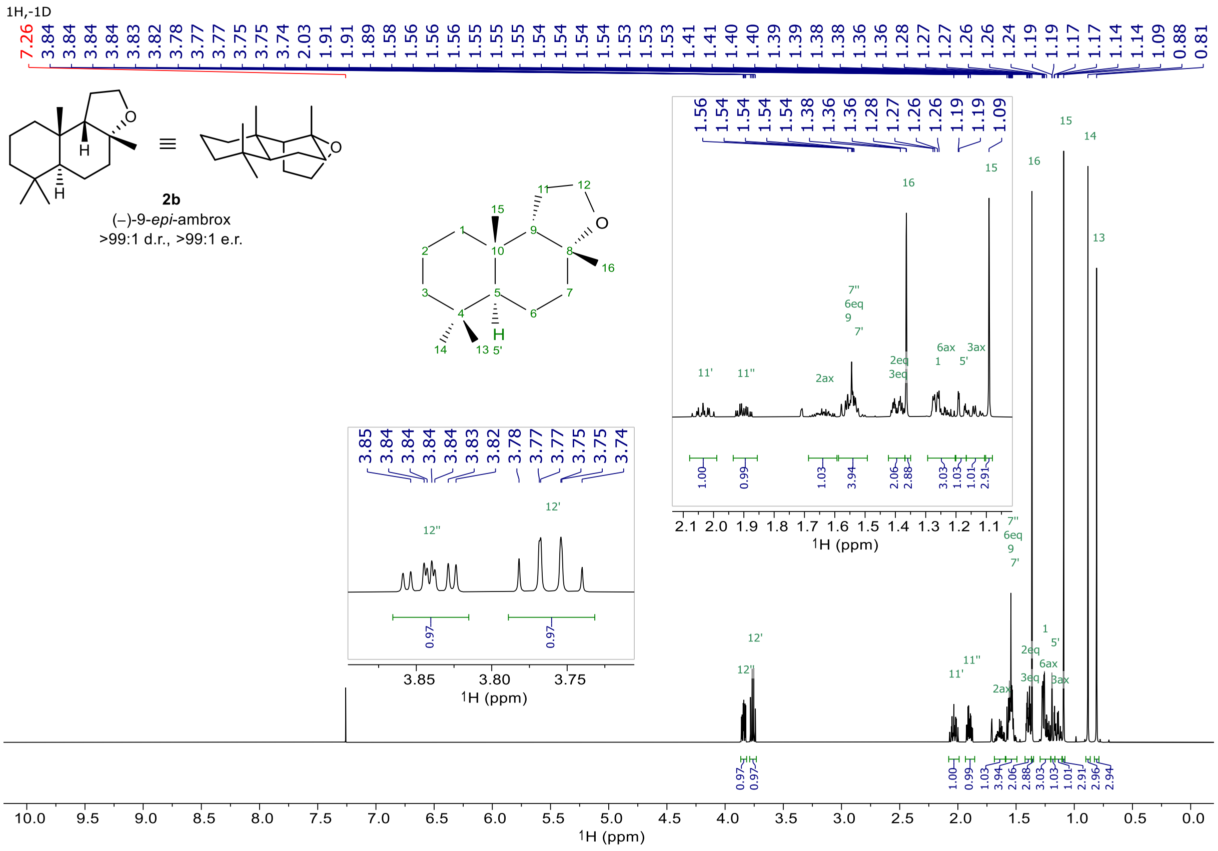

^{1}H NMR (600 MHz, $CDCl_3$) spectrum of synthetic (−)-9-*epi*-ambrox **2b** (reference sample) with assignments

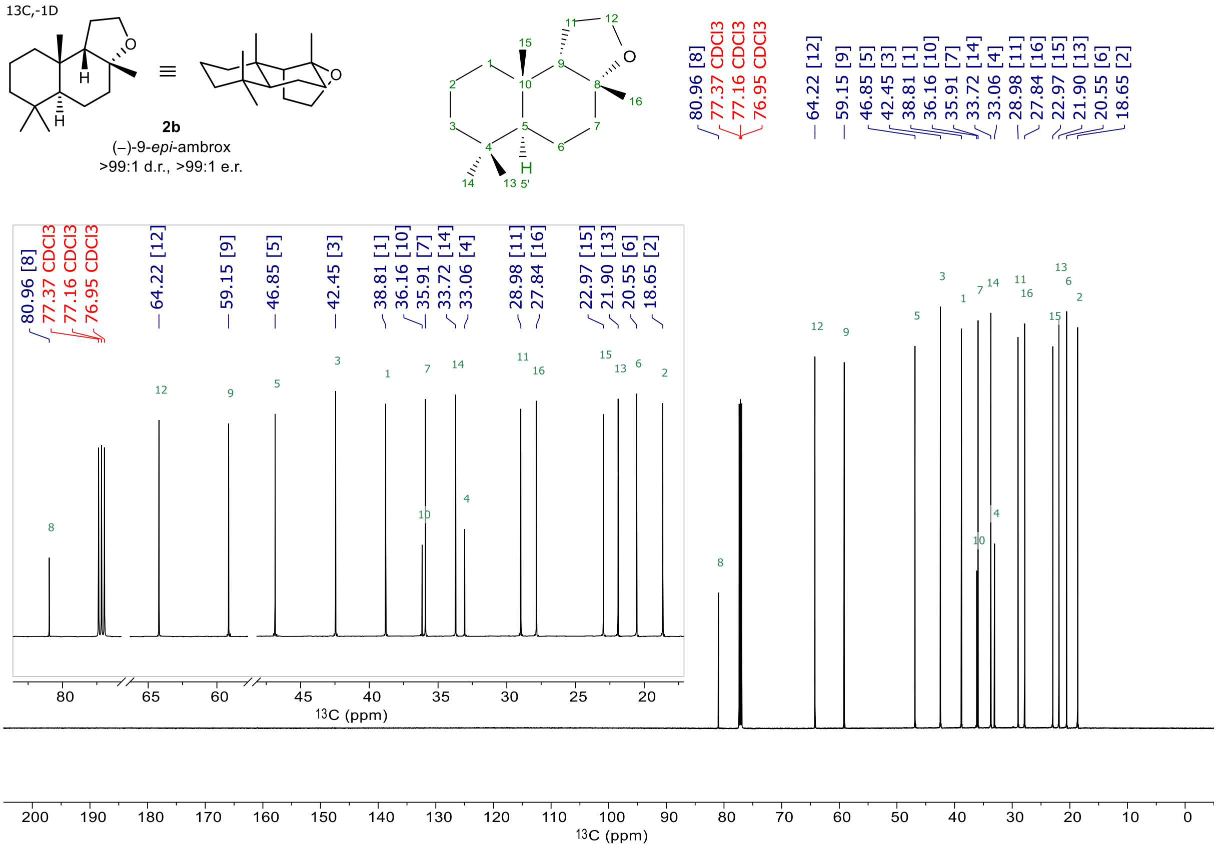

^{13}C NMR (151 MHz, $CDCl_3$) spectrum of synthetic (−)-9-*epi*-ambrox **2b** (reference sample) with assignments

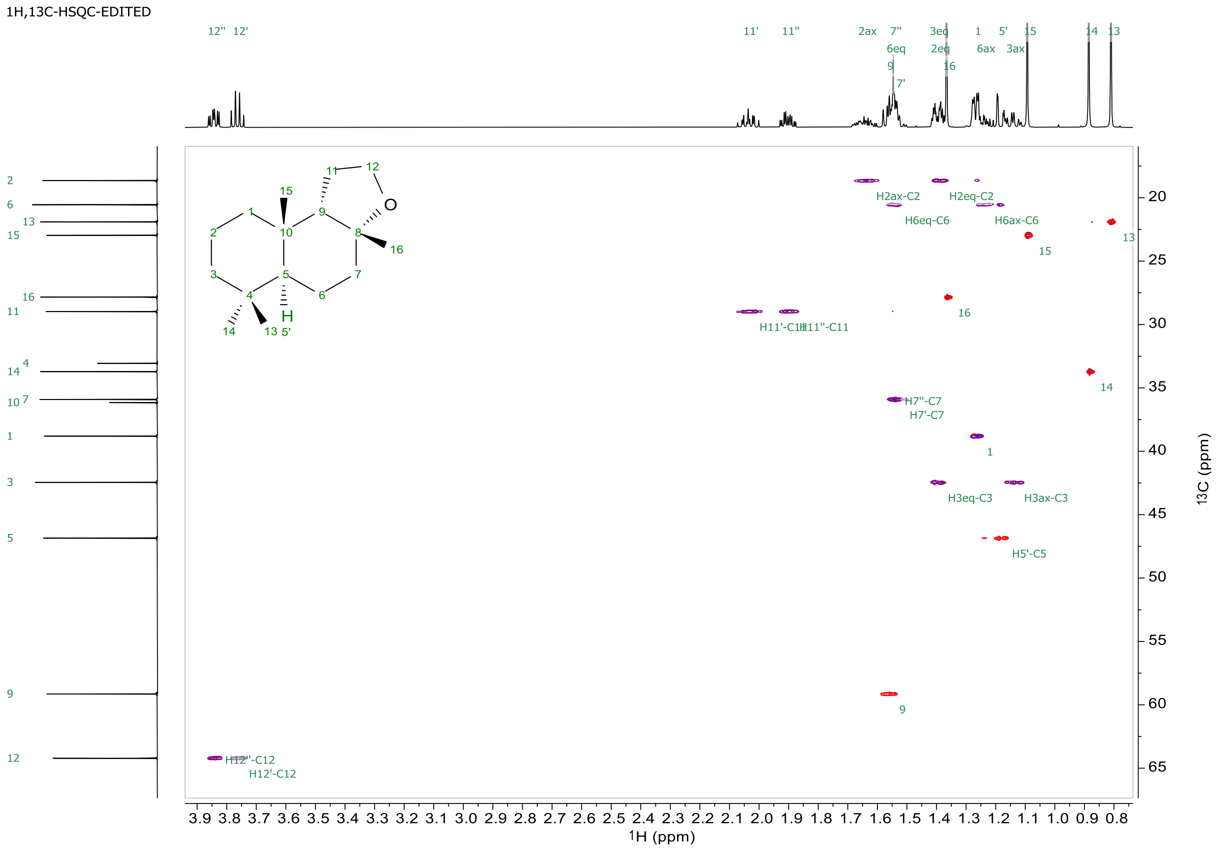

Multiplicity-edited **¹H,¹³C-HSQC** (600 MHz, 151 MHz) spectrum of synthetic (−)-9-*epi*-ambrox **2b** (reference sample) with assignments

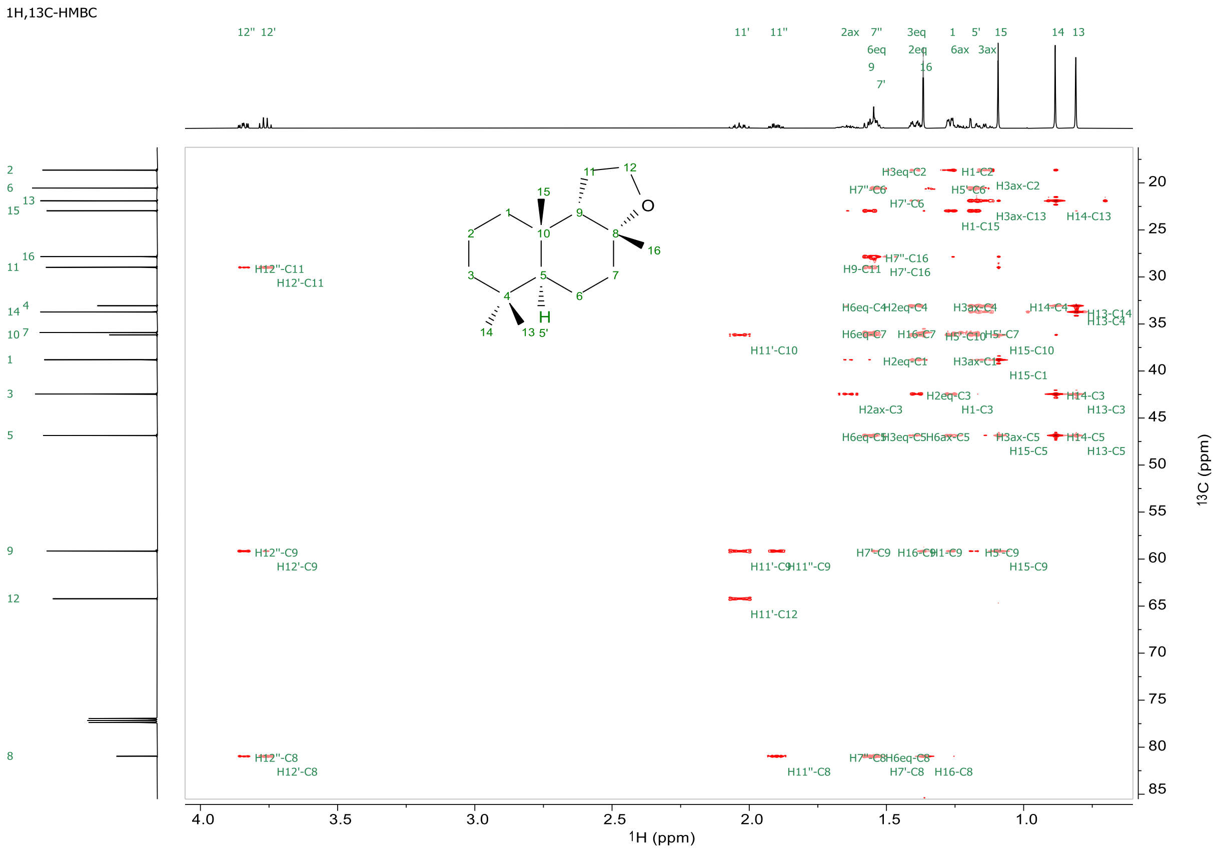

¹H,¹³C-HMBC (600 MHz, 151 MHz) spectrum of synthetic (−)-9-*epi*-ambrox **2b** (reference sample) with assignments

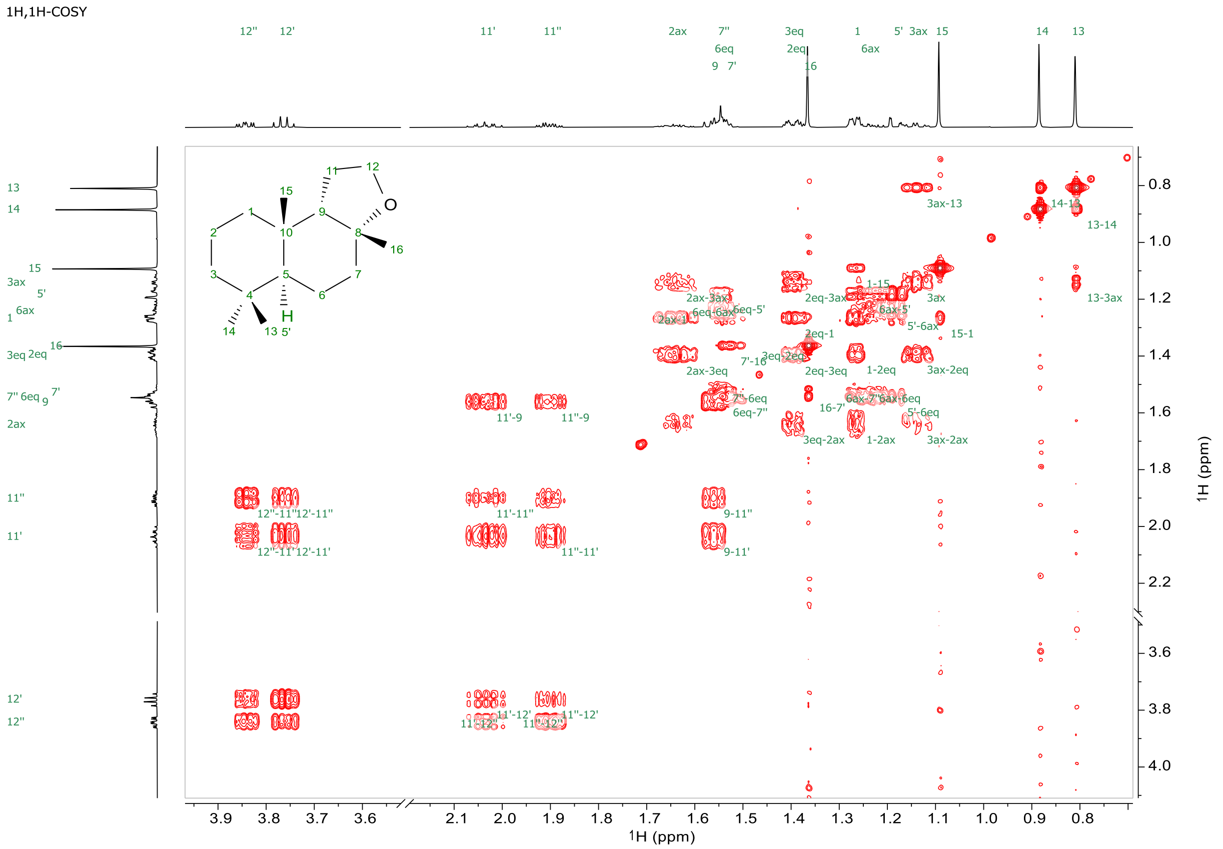

^{1}H,^{1}H-COSY (600 MHz, $CDCl_3$) spectrum of synthetic (−)-9-*epi*-ambrox **2b** (reference sample) with assignments

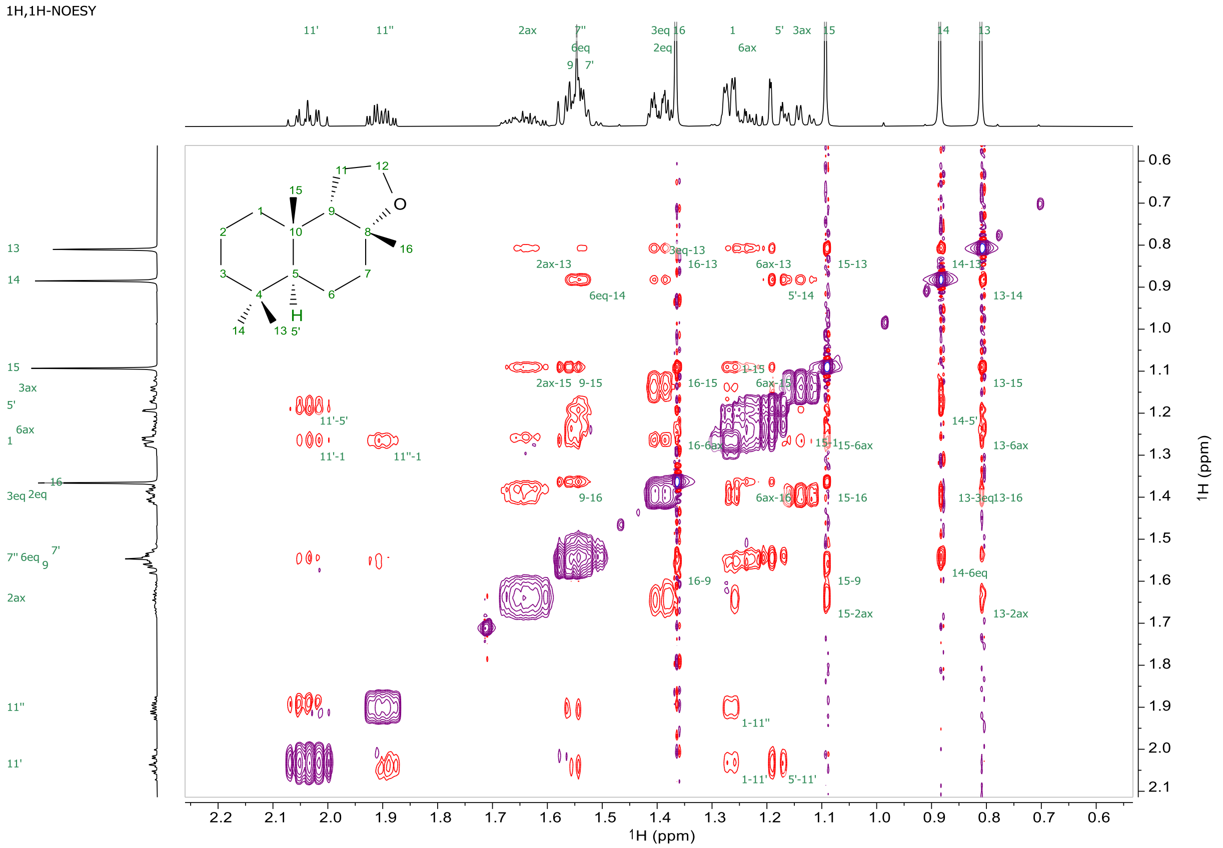

^{1}H,^{1}H-NOESY (600 MHz, $CDCl_3$) spectrum of synthetic (−)-9-*epi*-ambrox **2b** (reference sample) with assignments

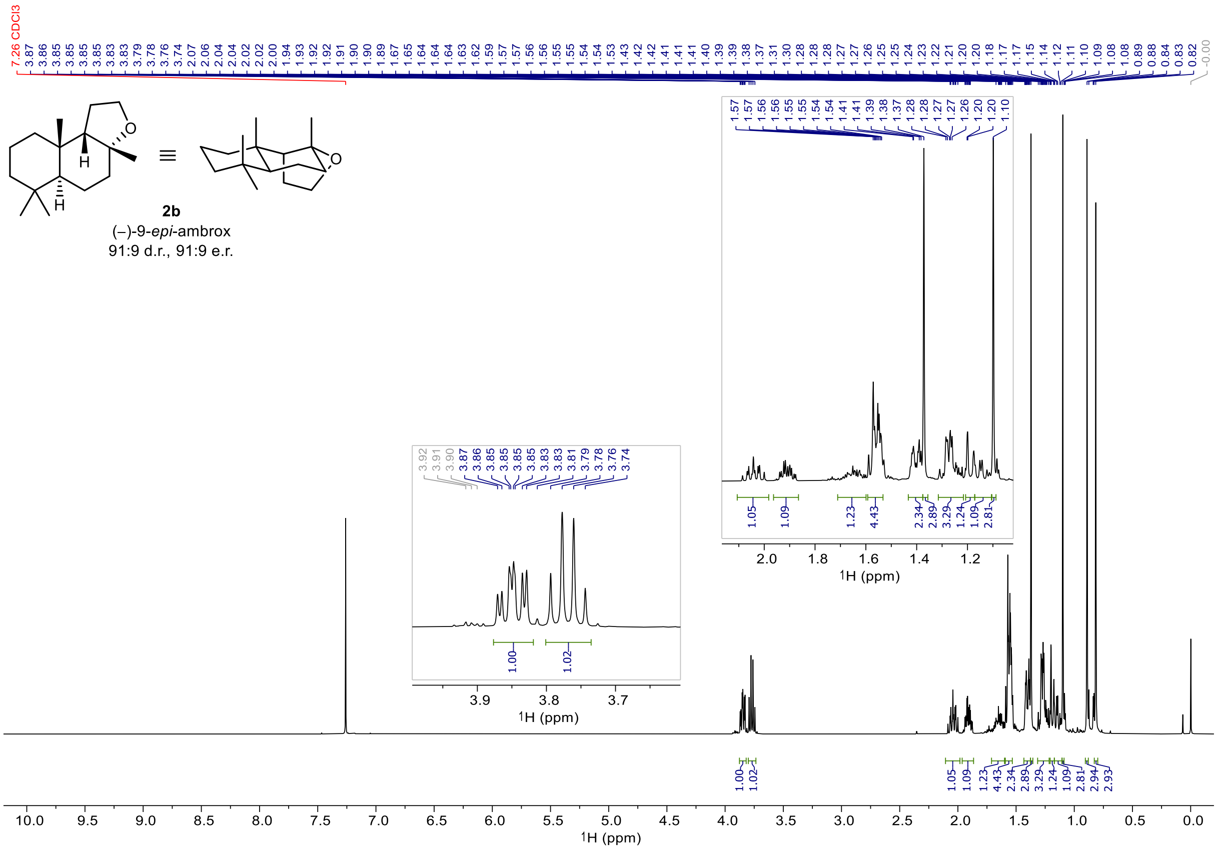

^{1}H NMR (501 MHz, $CDCl_3$) spectrum of synthetic (−)-9-*epi*-ambrox **2b** obtained as major product in the IDPi-catalyzed polyene cyclization of (3*Z*,7*E*)-homofarnesol to (−)-ambrox

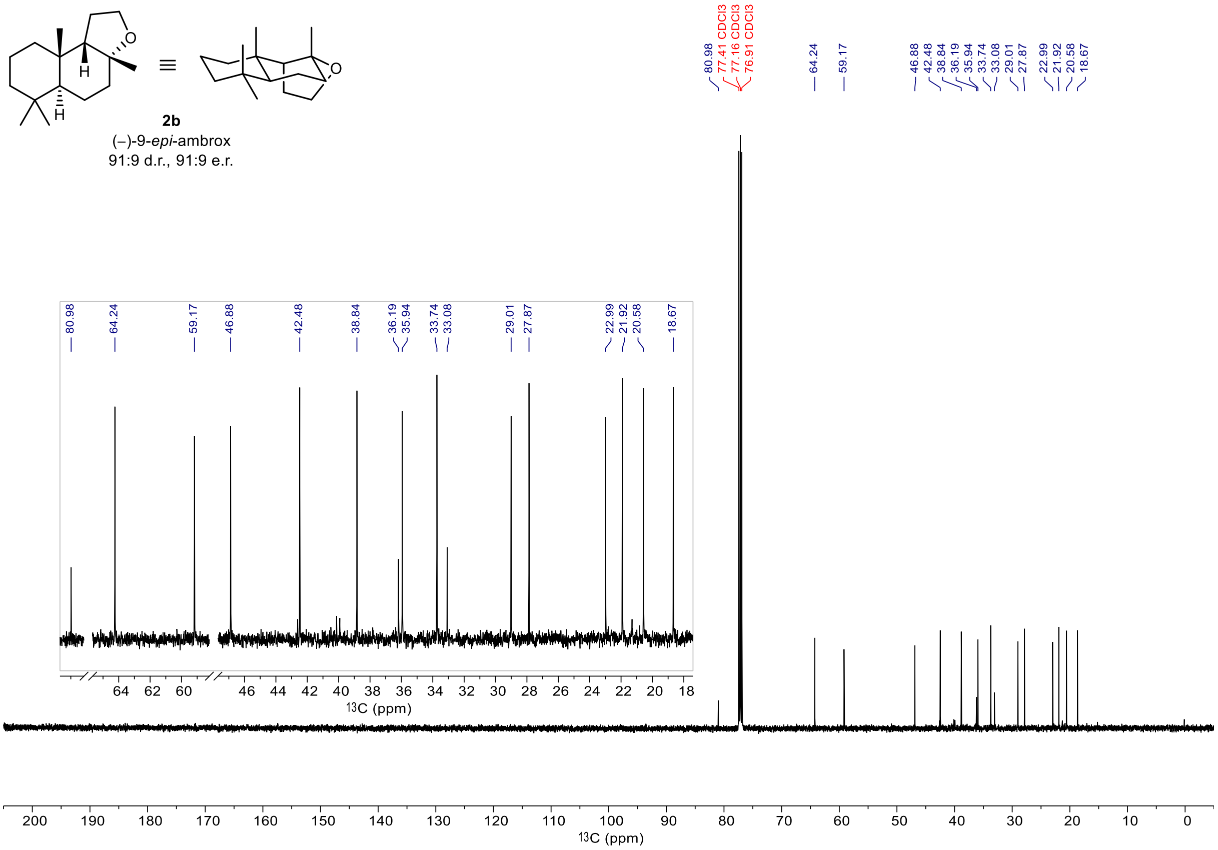

^{13}C NMR (151 MHz, $CDCl_3$) spectrum of synthetic (−)-9-*epi*-ambrox **2b** obtained as major product in the IDPi-catalyzed polyene cyclization of (3*Z*,7*E*)-homofarnesol to (−)-ambrox

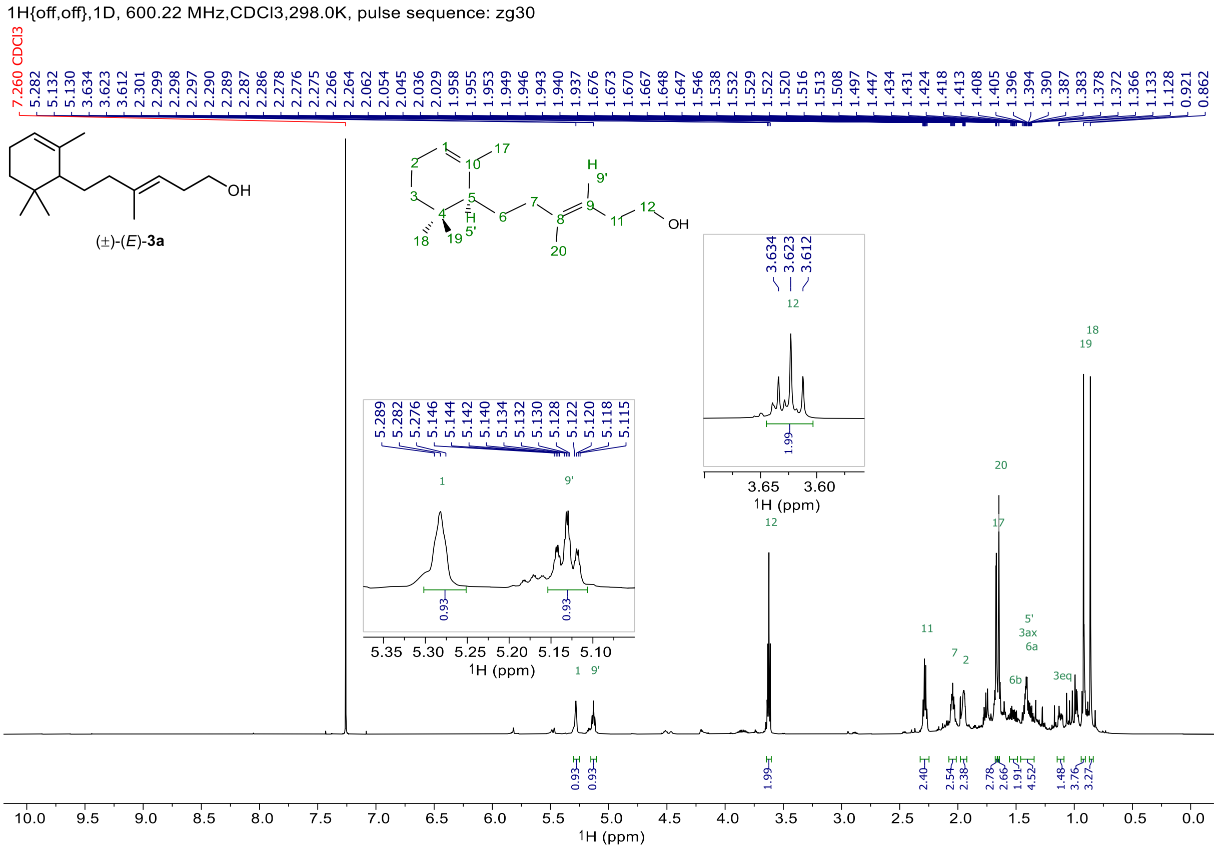

^{1}H NMR (600 MHz, $CDCl_3$) spectrum of (±)-(*E*)-α-cyclohomofarnesol **3a** with assignments

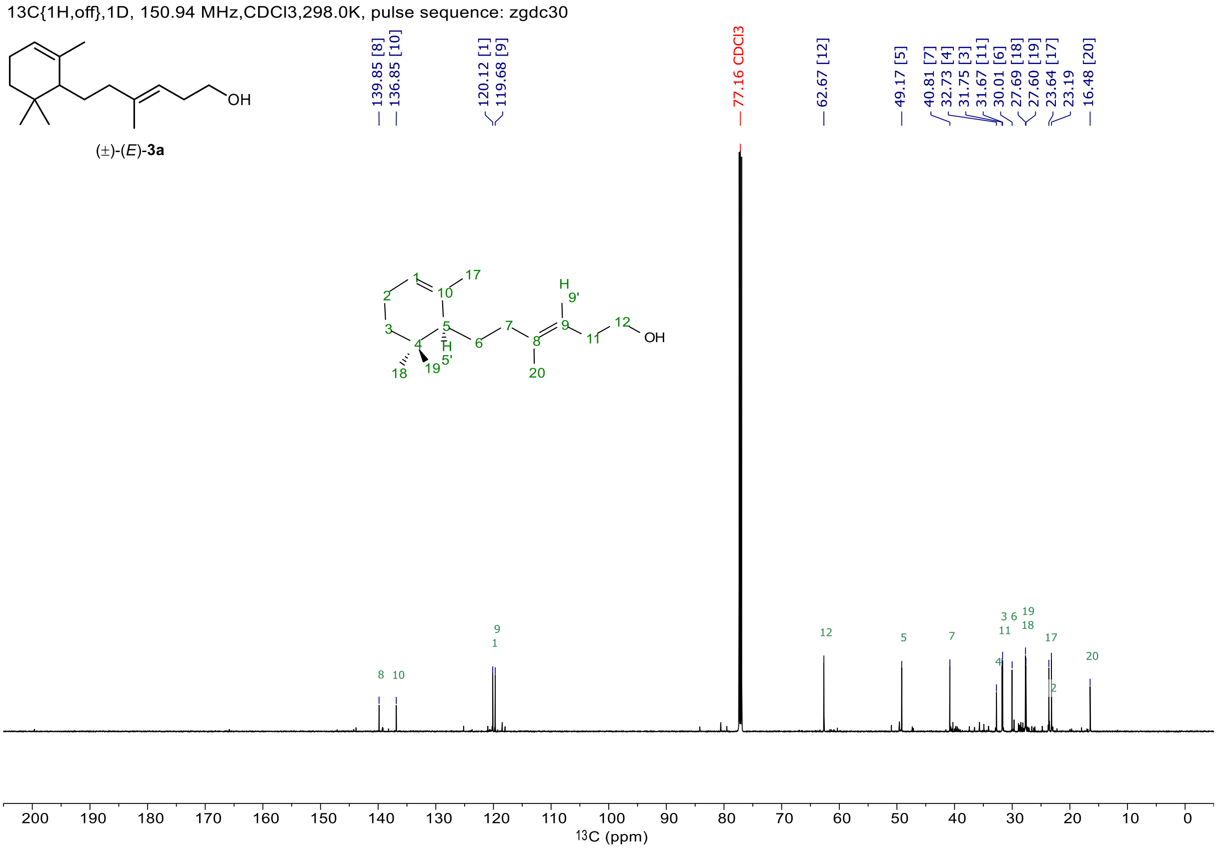

^{13}C NMR (151 MHz, $CDCl_3$) spectrum of (±)-(*E*)-α-cyclohomofarnesol **3a** with assignments

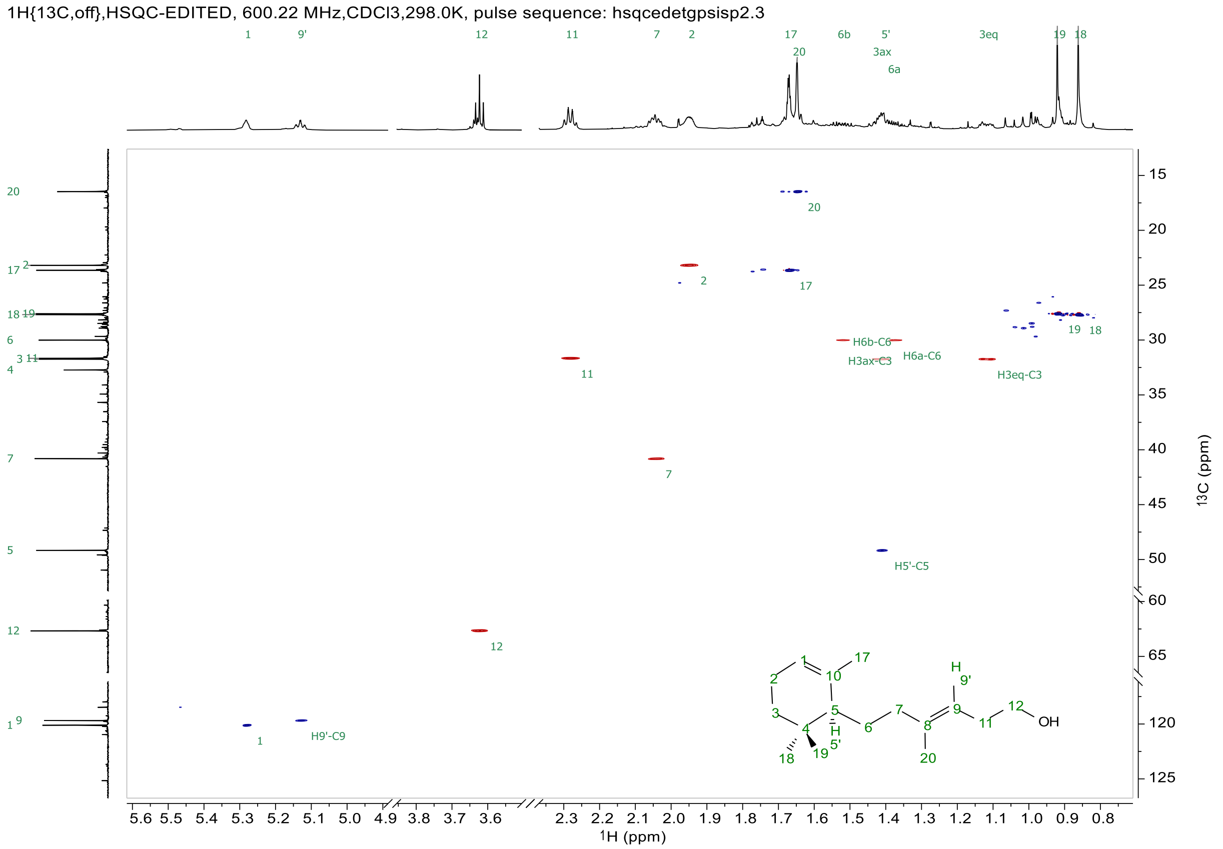

Multiplicity-edited **^{1}H,^{13}C-HSQC** (600 MHz, 151 MHz) spectrum of (±)-(*E*)-α-cyclohomofarnesol **3a** with assignments

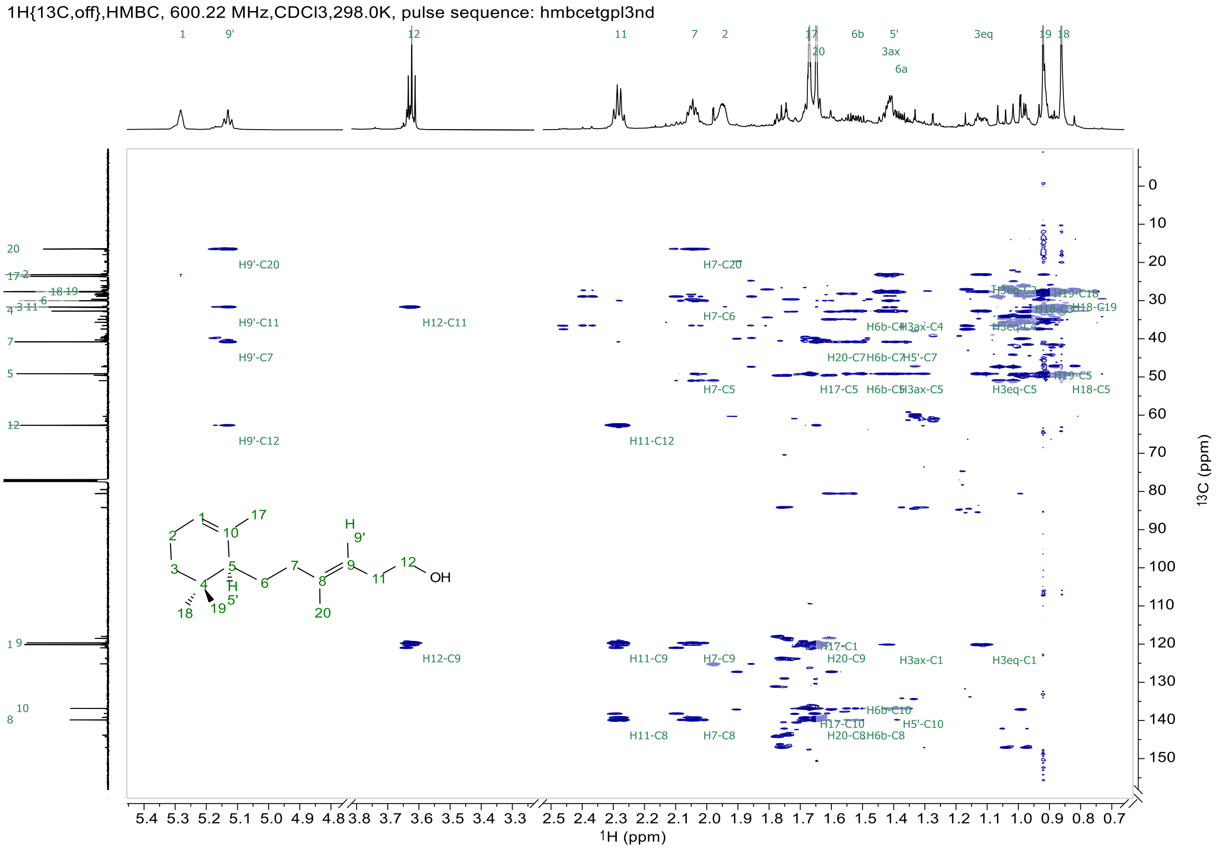

^{1}H,^{13}C-HMBC (600 MHz, 151 MHz) spectrum of (±)-(*E*)-α-cyclohomofarnesol **3a** with assignments

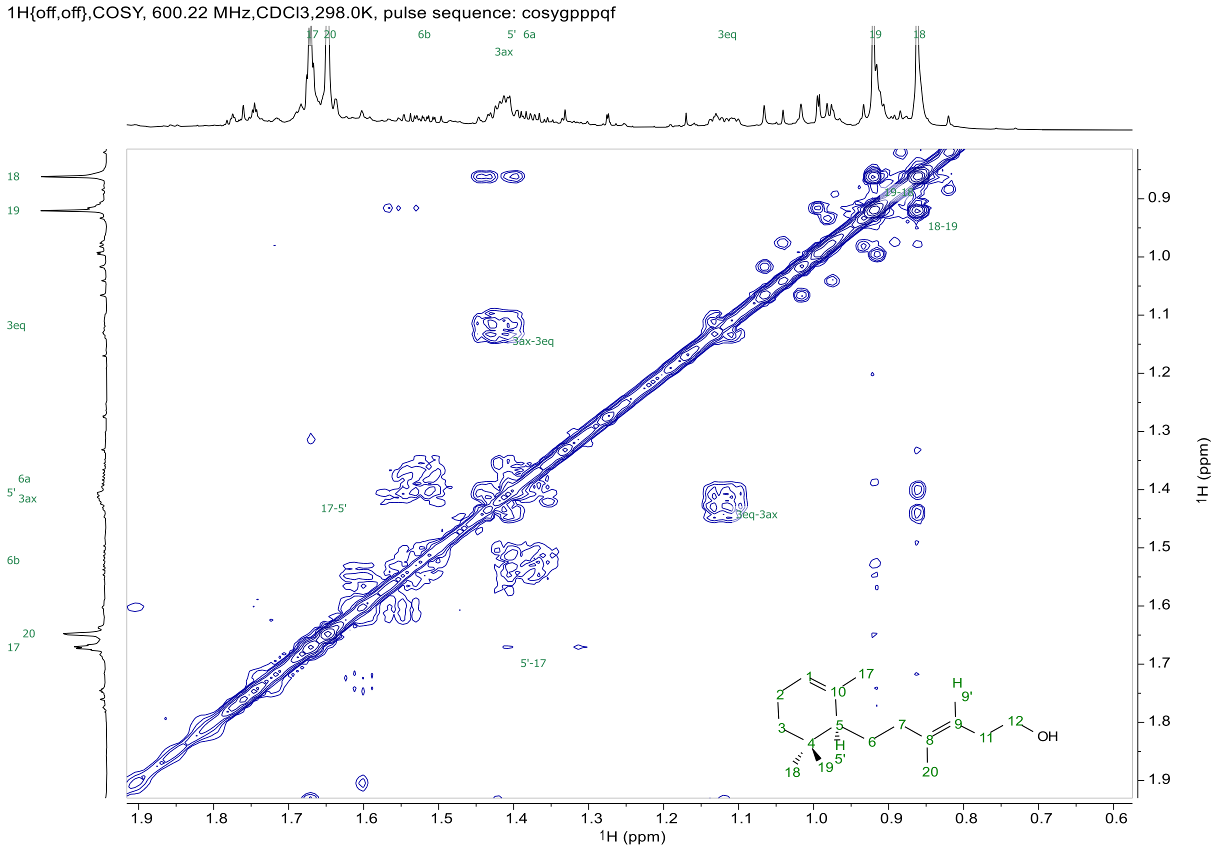

^{1}H,^{1}H-COSY (600 MHz, CDCl$_3$) spectrum of (±)-(*E*)-α-cyclohomofarnesol **3a** with assignments

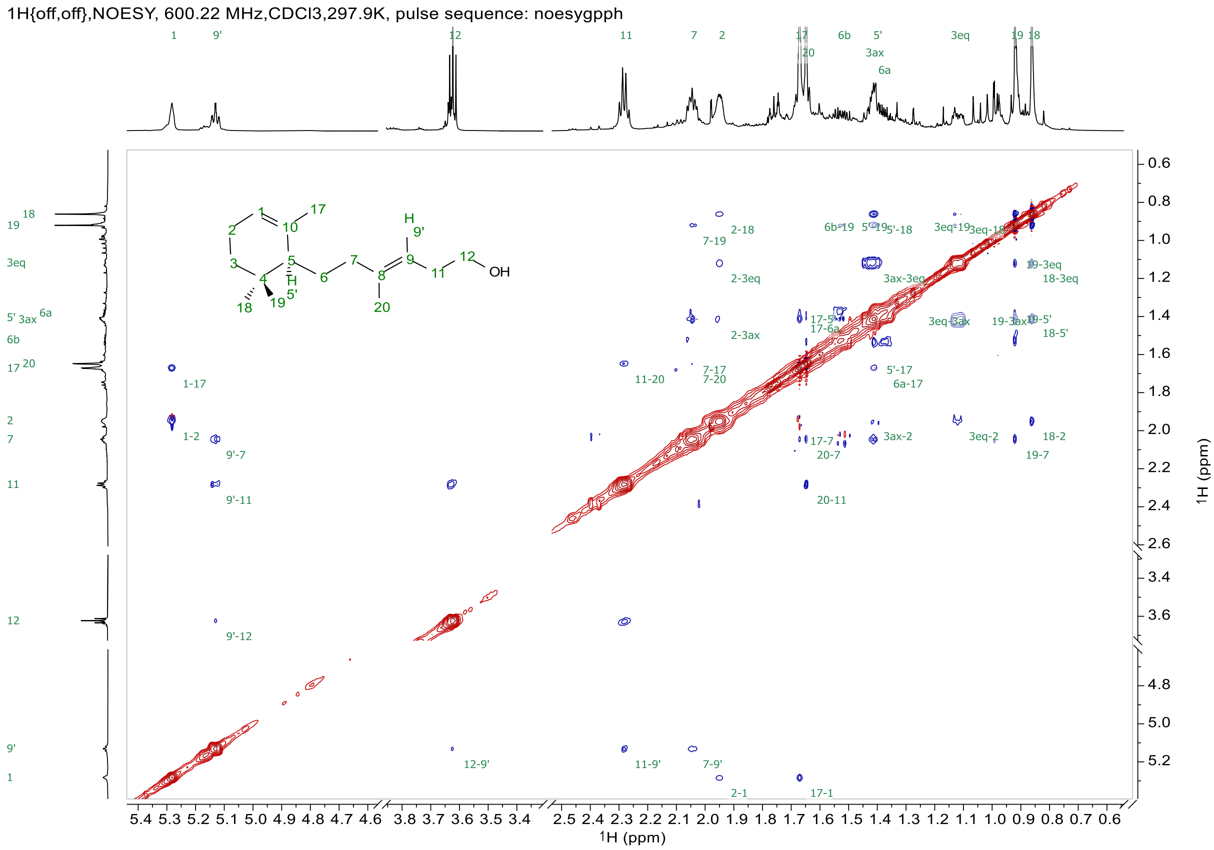

^{1}H,^{1}H-NOESY (600 MHz, CDCl$_3$) spectrum of (±)-(*E*)-α-cyclohomofarnesol **3a** with assignments

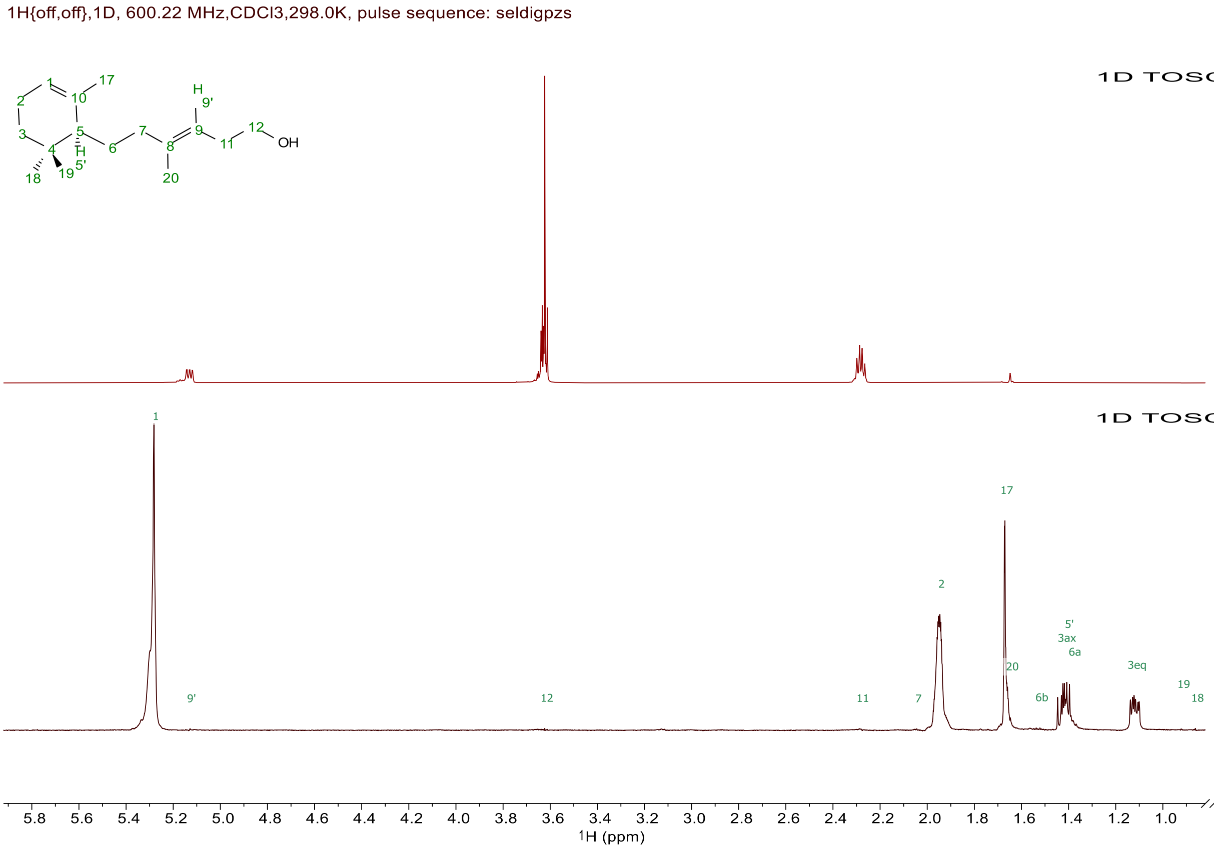

1D TOCSY (600 MHz, $CDCl_3$) spectra of (±)-(*E*)-α-cyclohomofarnesol **3a** with assignments after irradiation of H9 (top) or H1 (bottom)

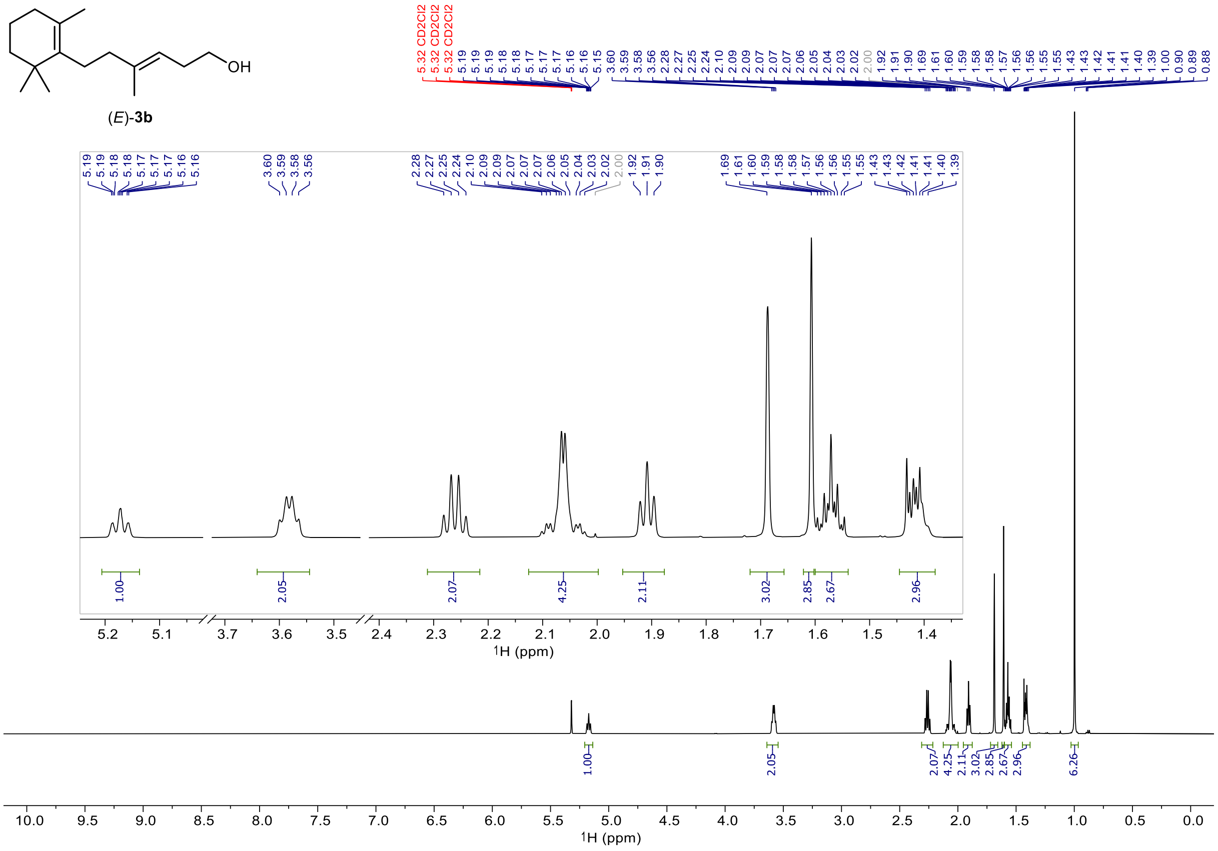

^{1}H NMR (501 MHz, CD_2Cl_2) spectrum of (*E*)-β-cyclohomofarnesol **3b**

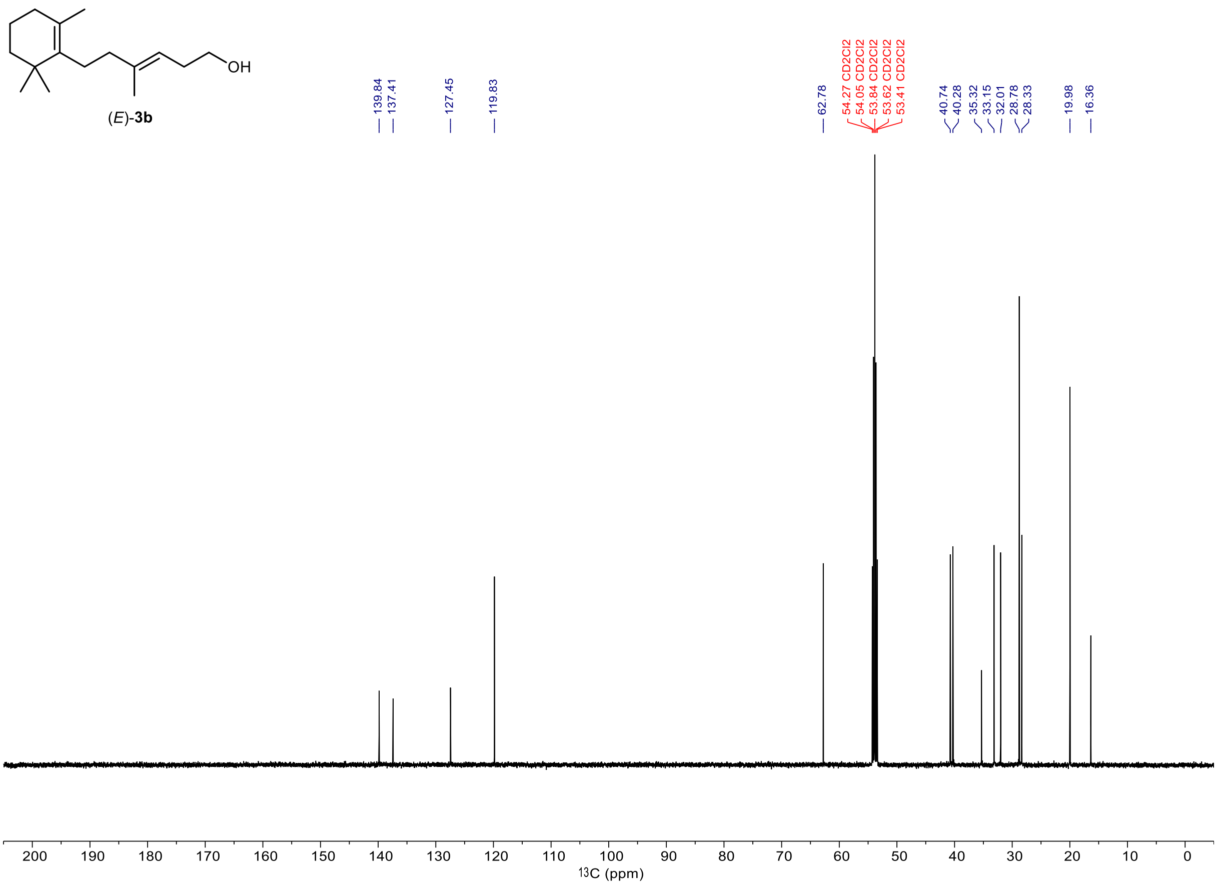

^{13}C NMR (126 MHz, CD_2Cl_2) spectrum of (*E*)-β-cyclohomofarnesol **3b**

Synthesis of (±)-(*E*)-γ-cyclohomofarnesol (3c)

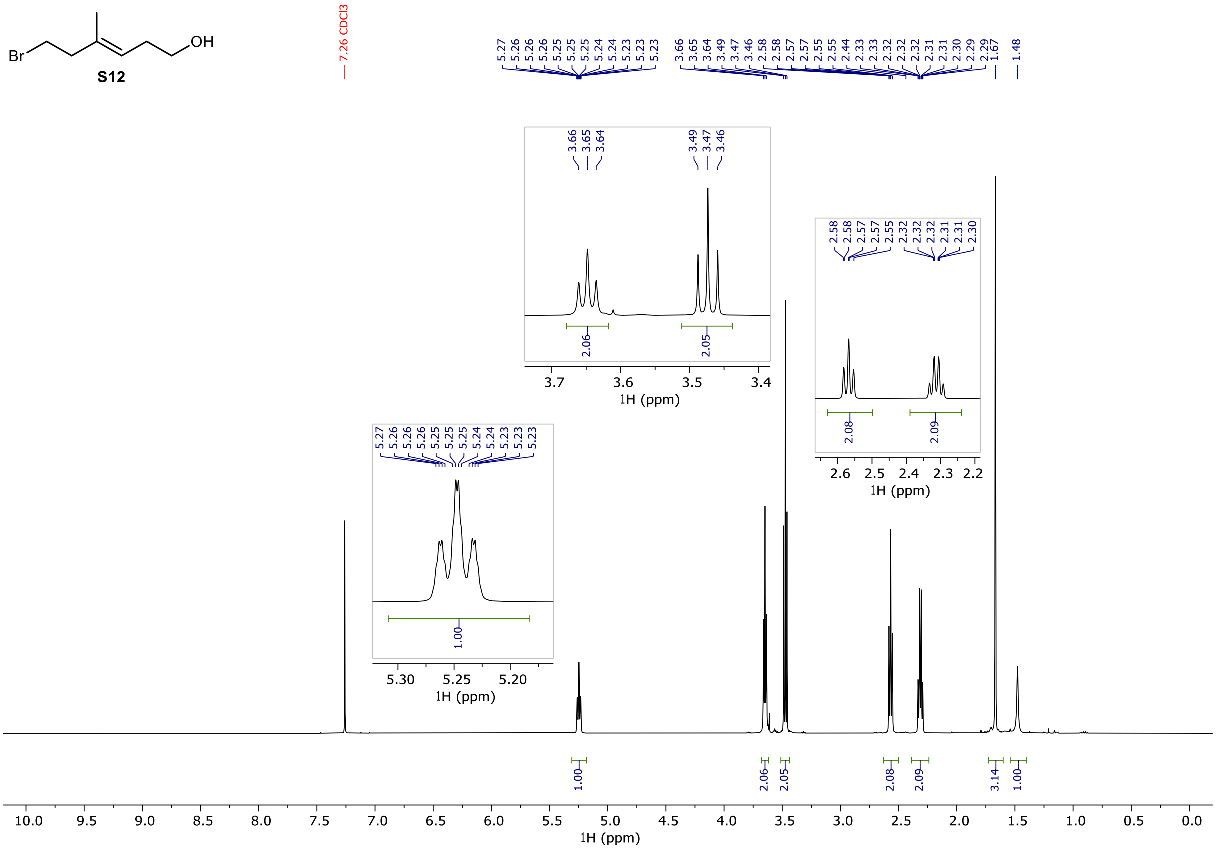

^{1}H NMR (501 MHz, $CDCl_3$) spectrum of compound **S12**

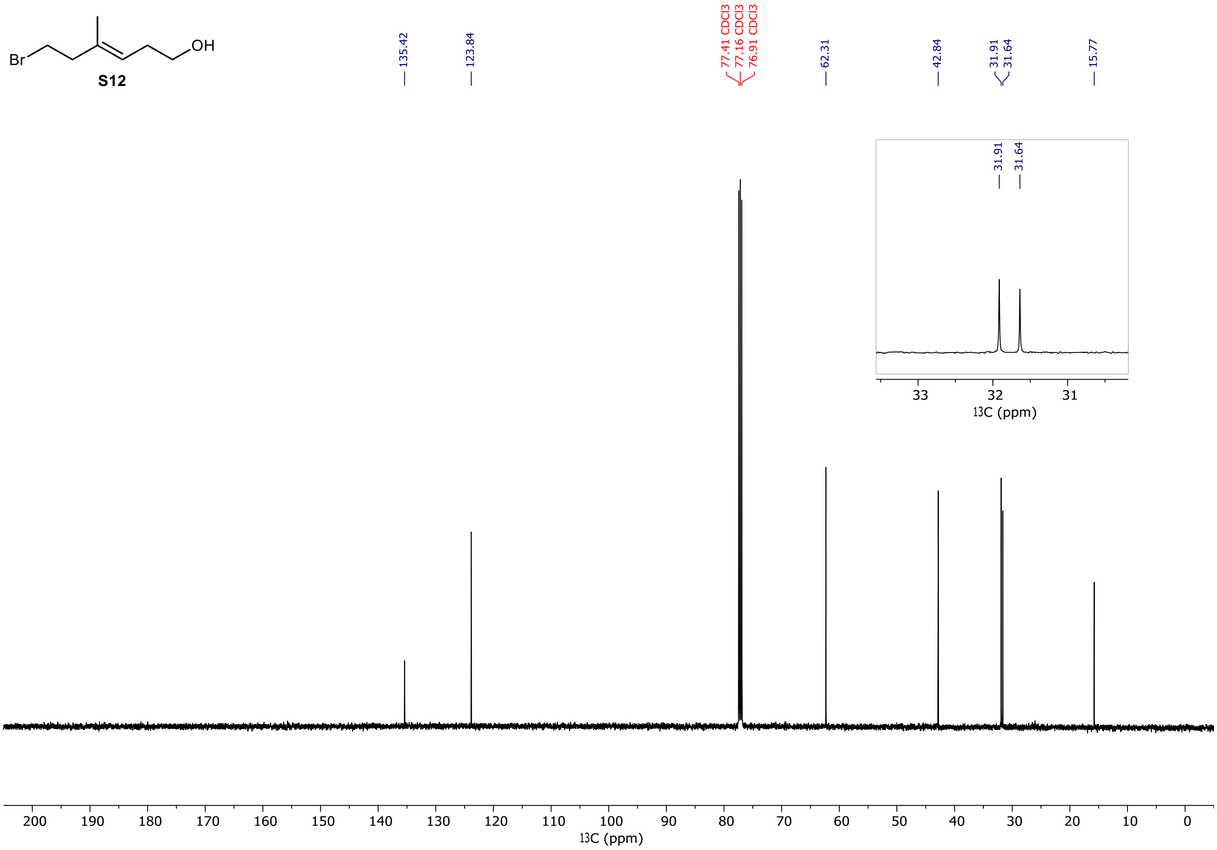

^{13}C NMR (126 MHz, $CDCl_3$) spectrum of compound **S12**

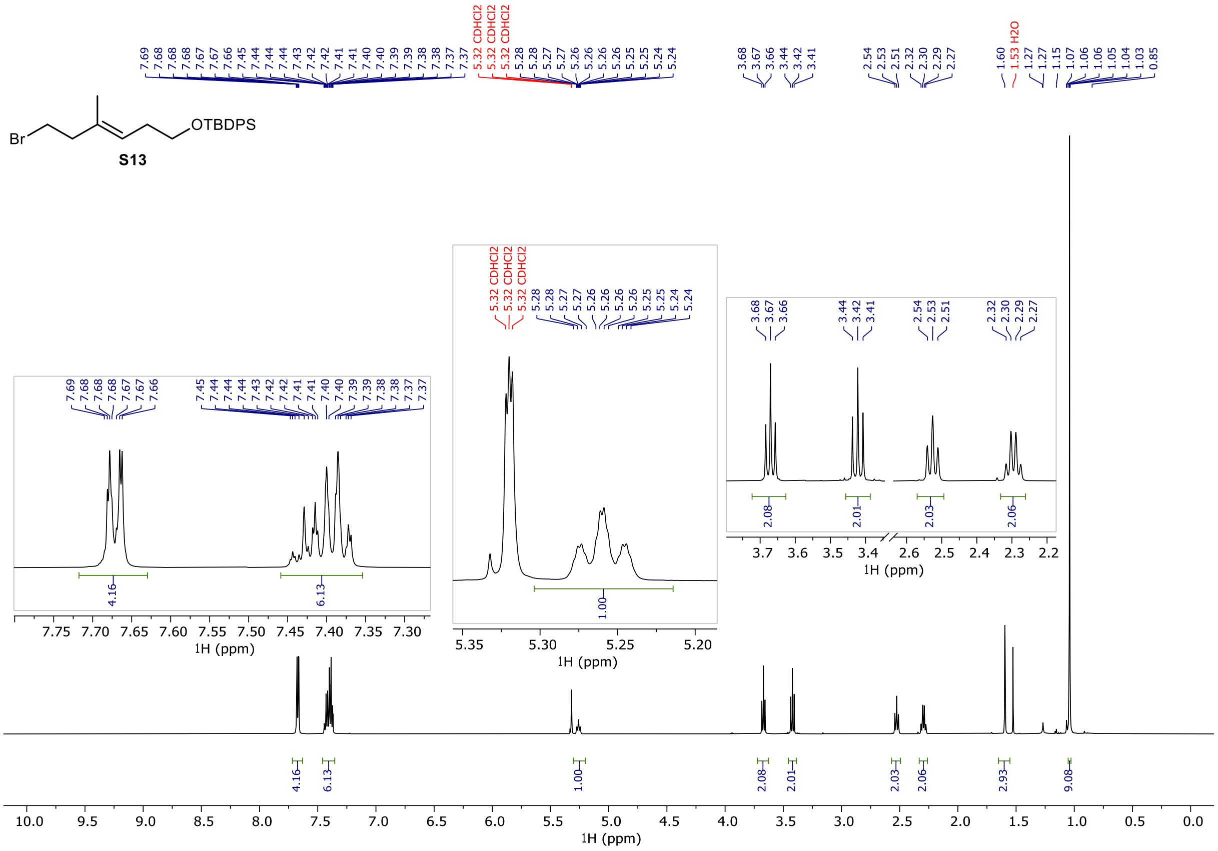

^{1}H NMR (501 MHz, CD_2Cl_2) spectrum of compound **S13**

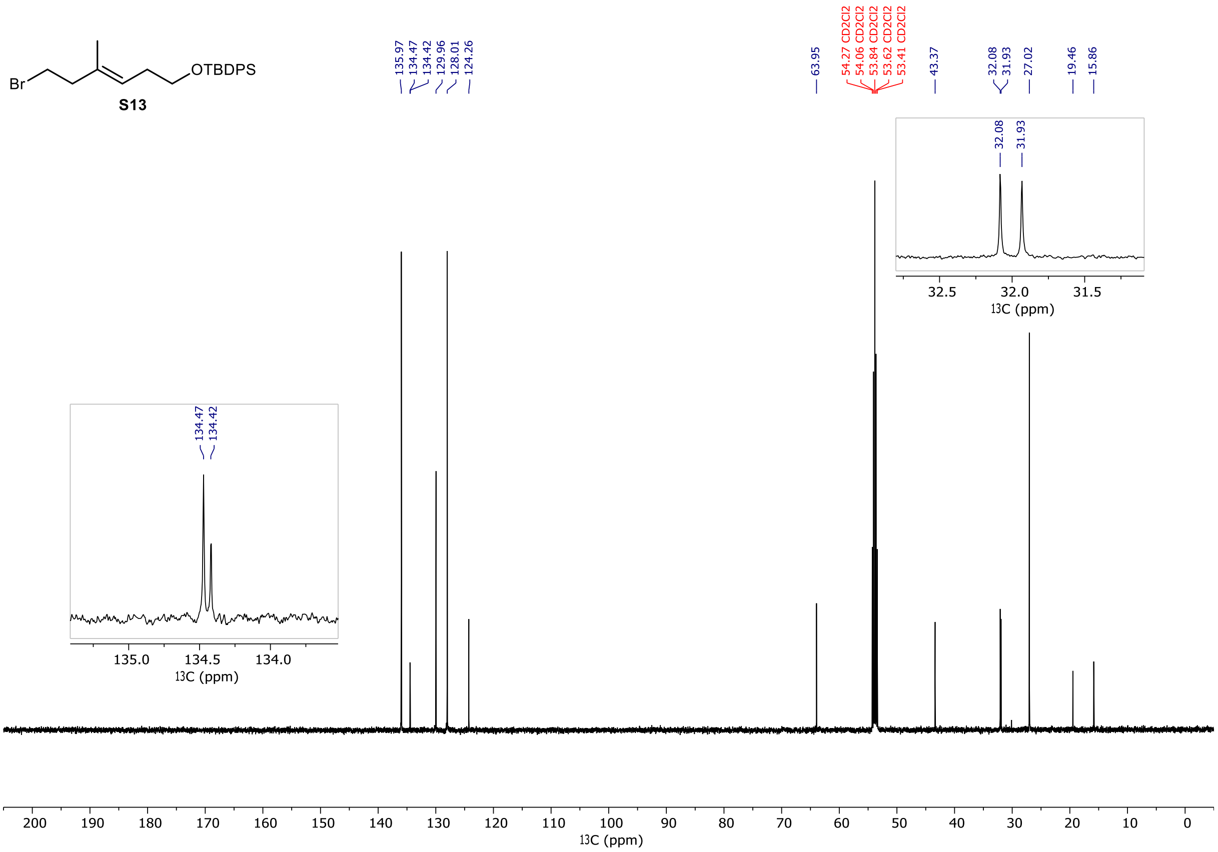

^{13}C NMR (126 MHz, CD_2Cl_2) spectrum of compound **S13**

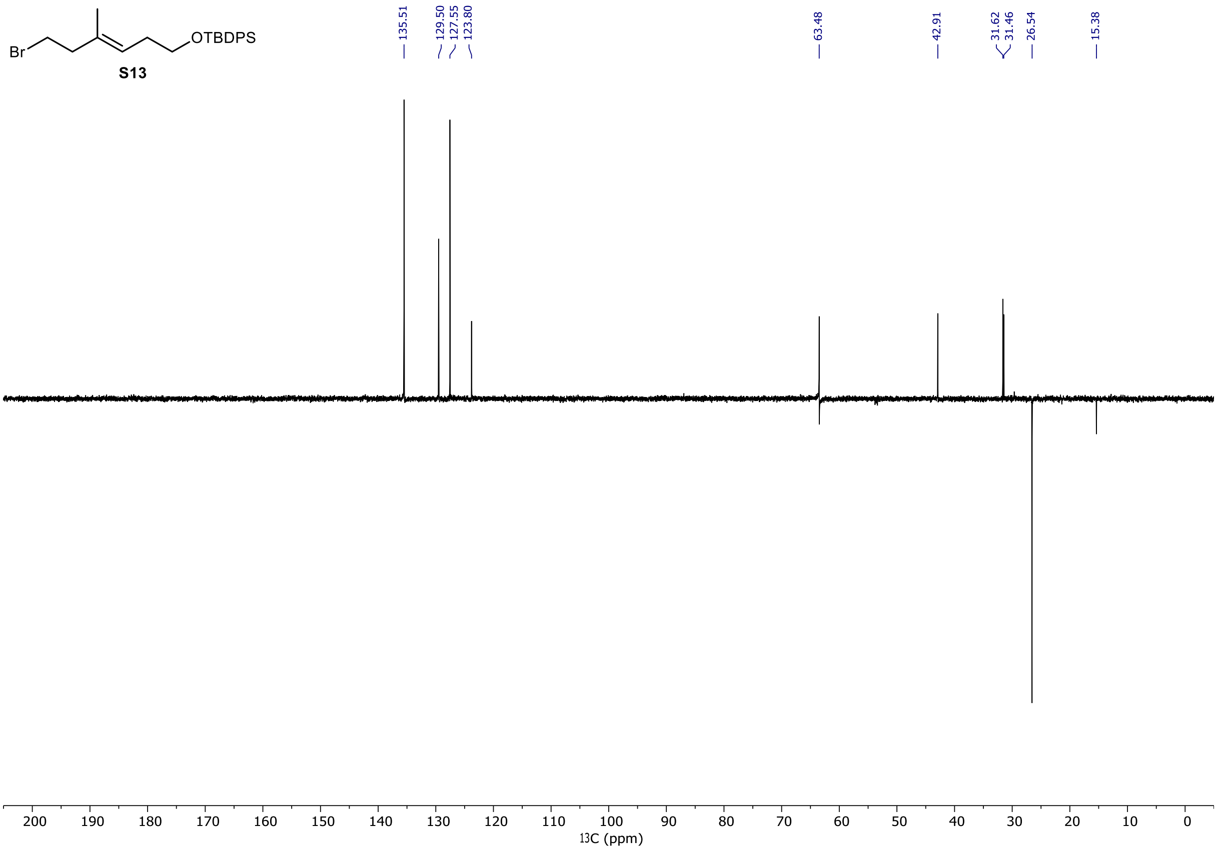

135-DEPT NMR (126 MHz, CD_2Cl_2) spectrum of compound **S13**

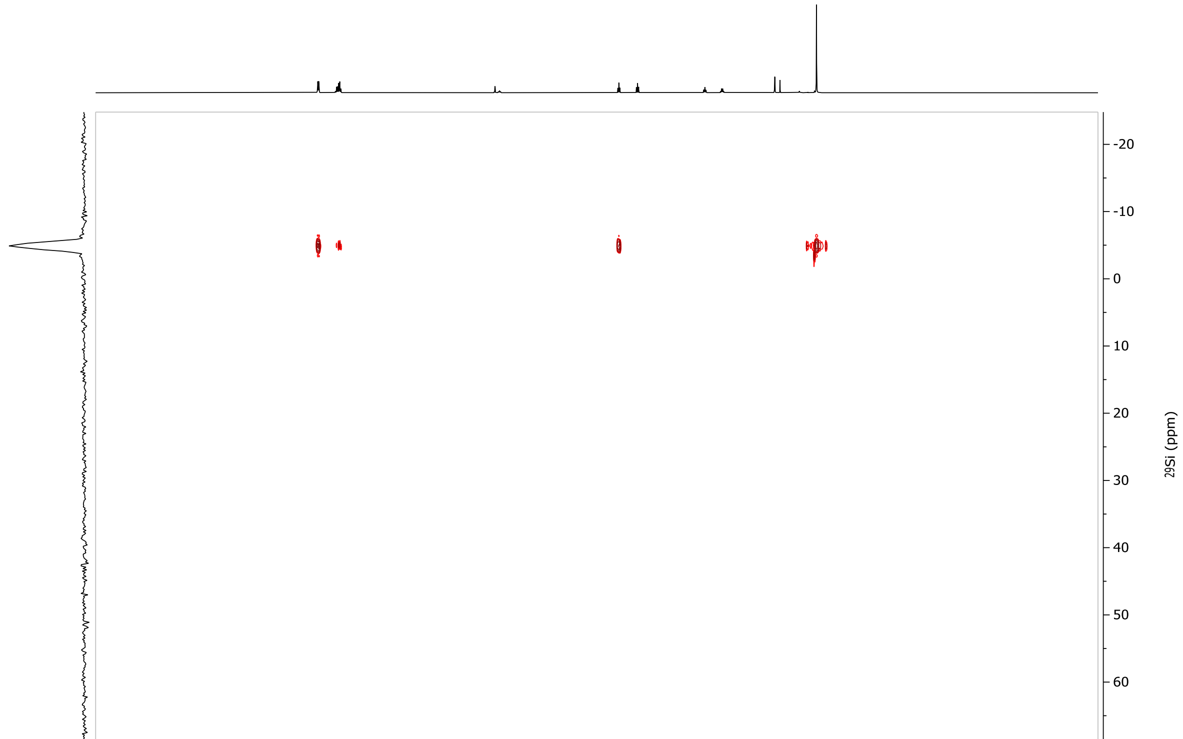

^{1}H,^{29}Si-HMBC (501 MHz, 99 MHz, CD_2Cl_2) spectrum of compound **S13**

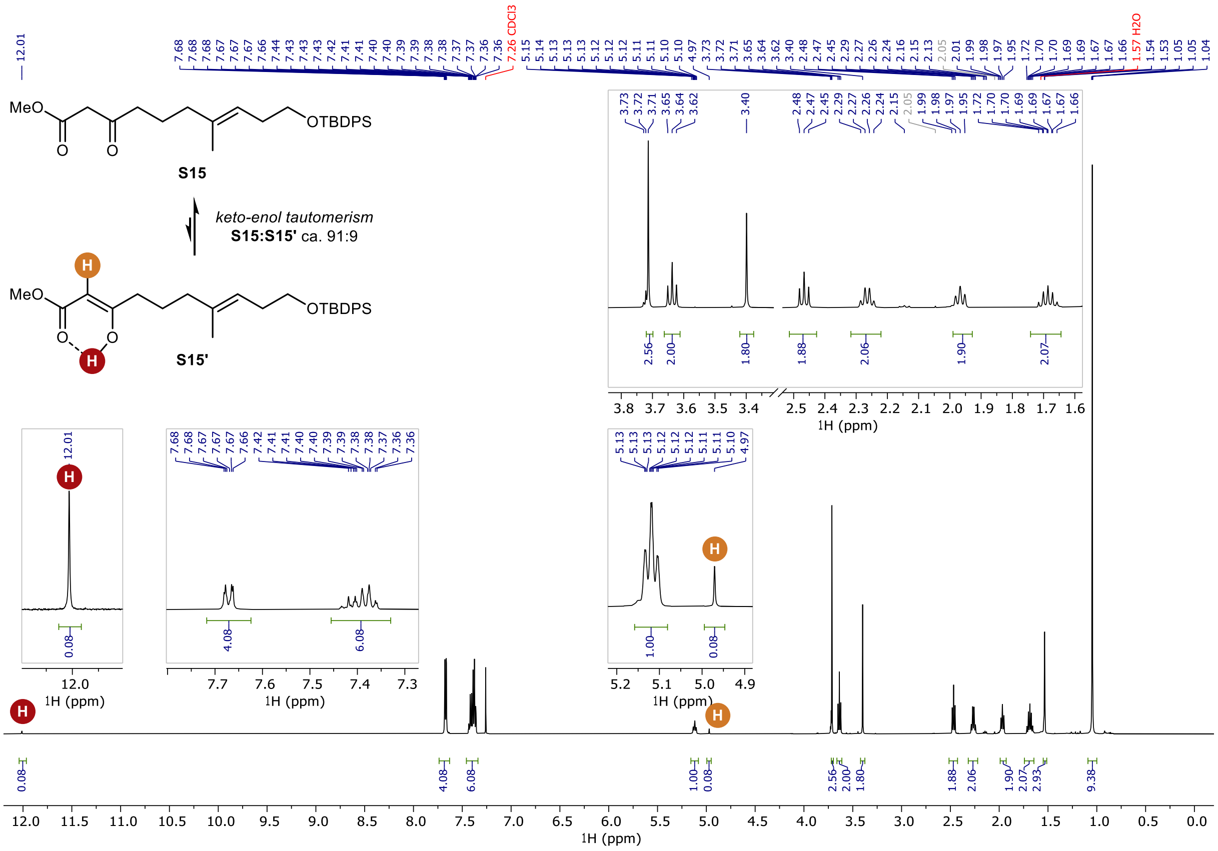

^{1}**H NMR** (501 MHz, $CDCl_3$) spectrum of compound **S15**

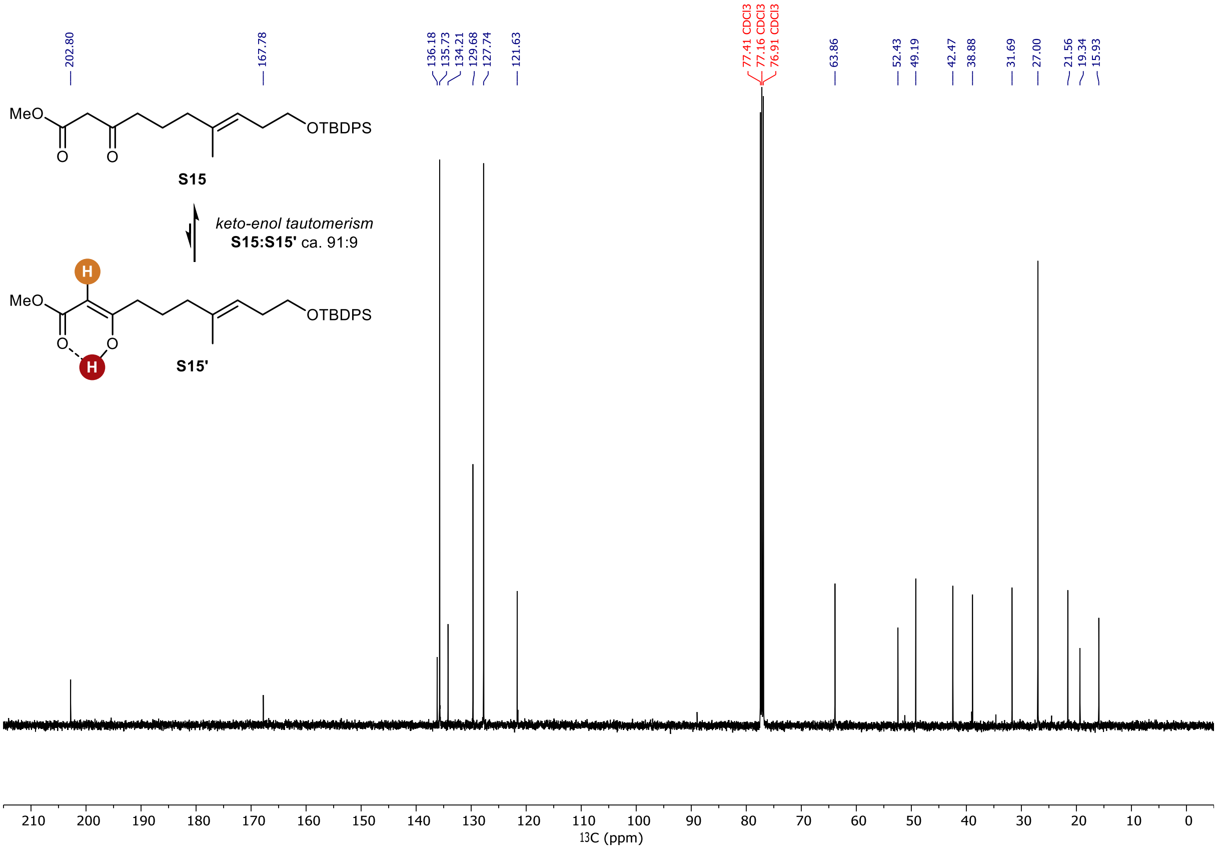

^{13}**C NMR** (126 MHz, $CDCl_3$) spectrum of compound **S15**

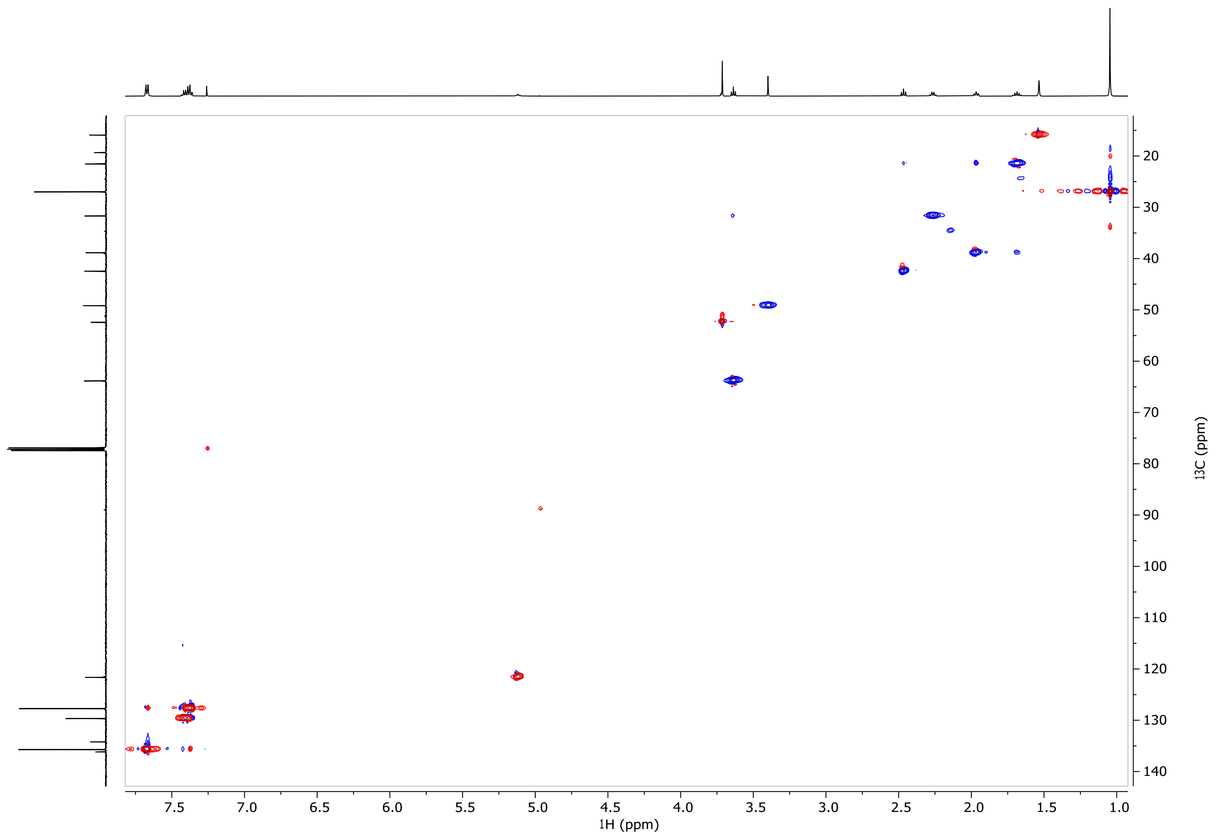

^{1}H,^{13}C-HSQC (501 MHz, 126 MHz, $CDCl_3$) spectrum of compound **S15**

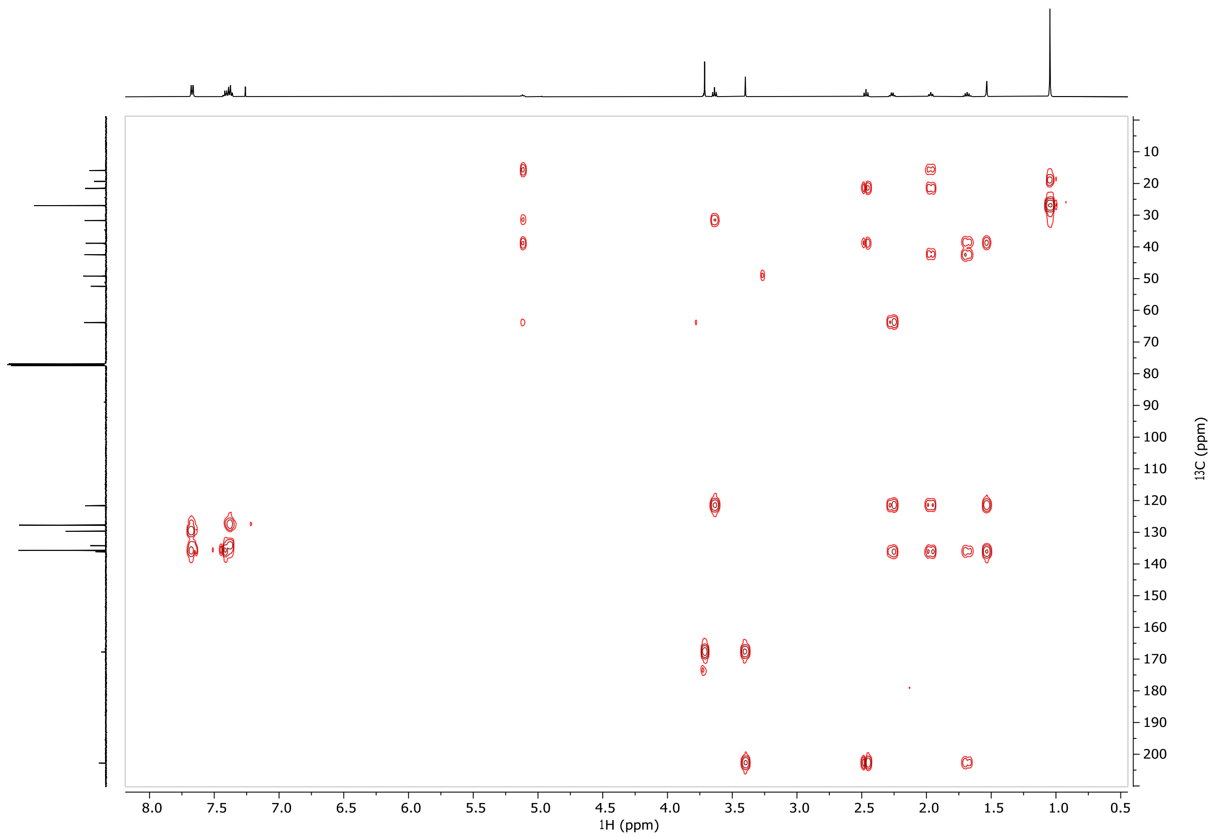

^{1}H,^{13}C-HMBC (501 MHz, 126 MHz, $CDCl_3$) spectrum of compound **S15**

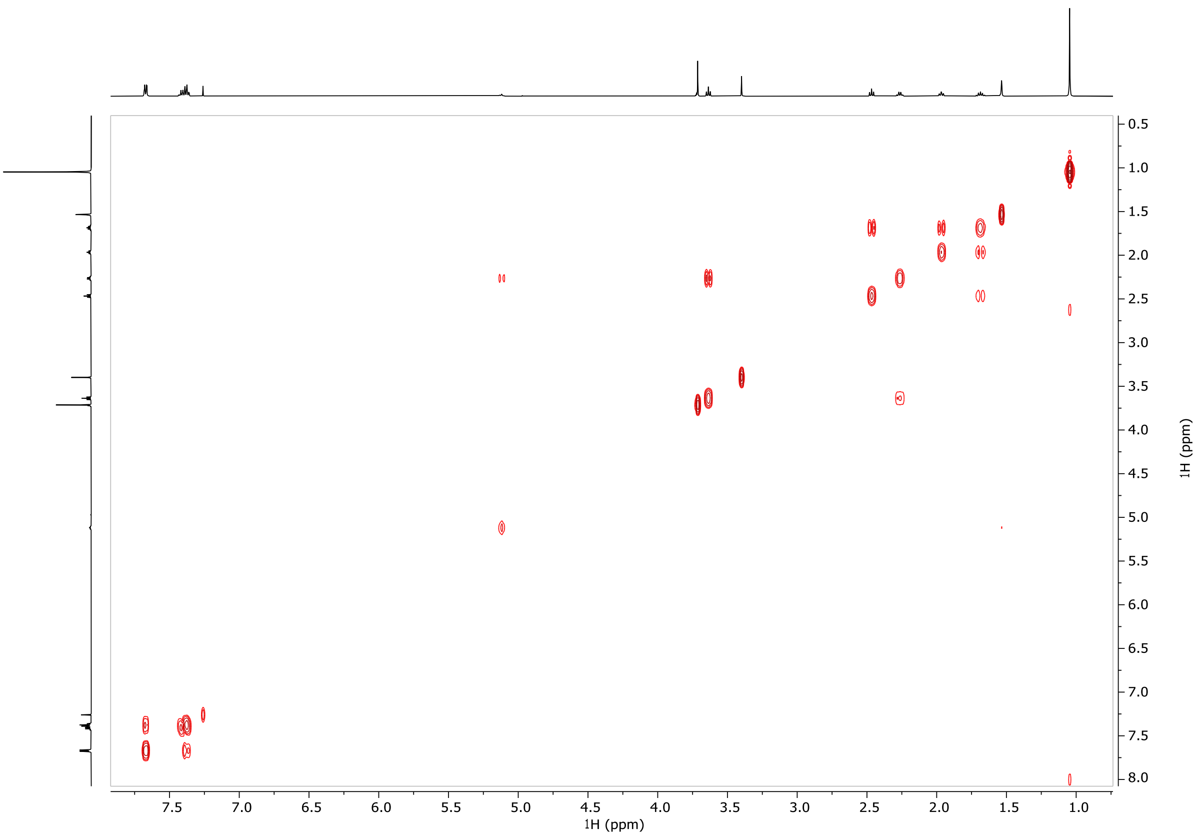

$^{1}H,^{1}H$-COSY (501 MHz, $CDCl_3$) spectrum of compound **S15**

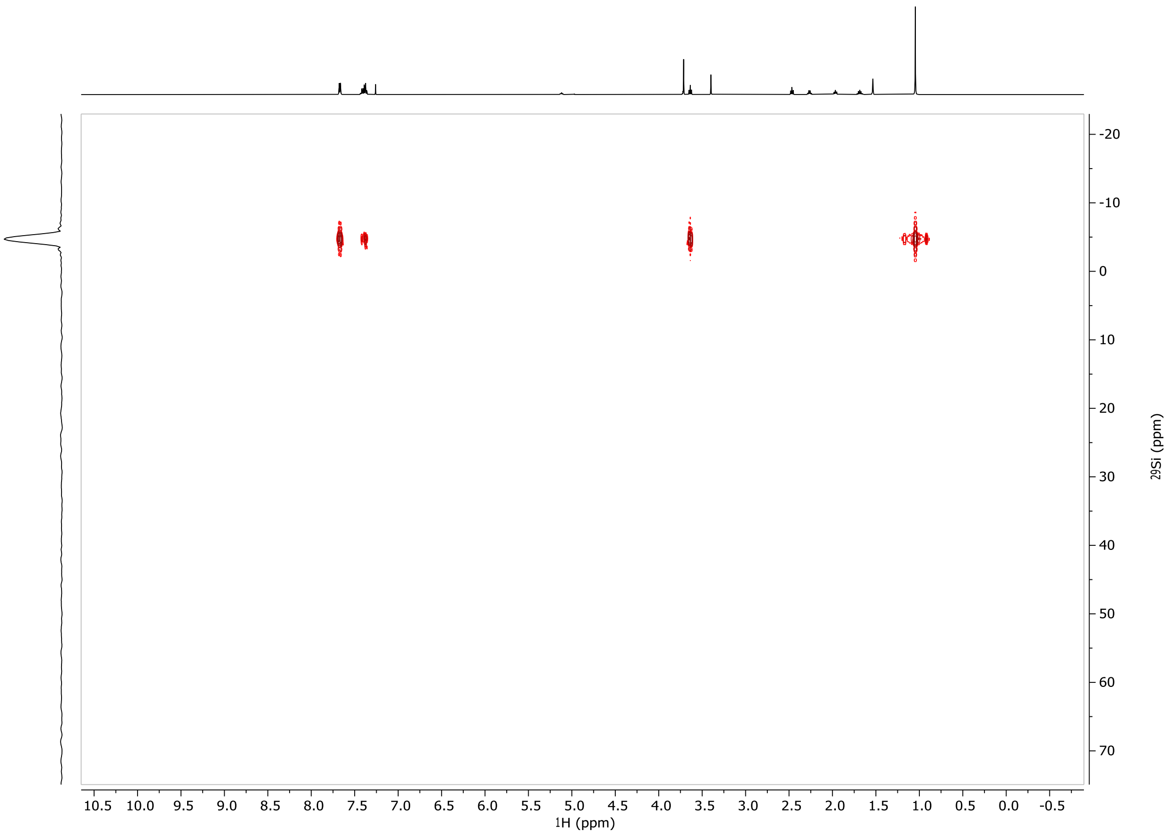

$^{1}H,^{29}Si$-HMBC (501 MHz, 99 MHz, $CDCl_3$) spectrum of compound **S15**

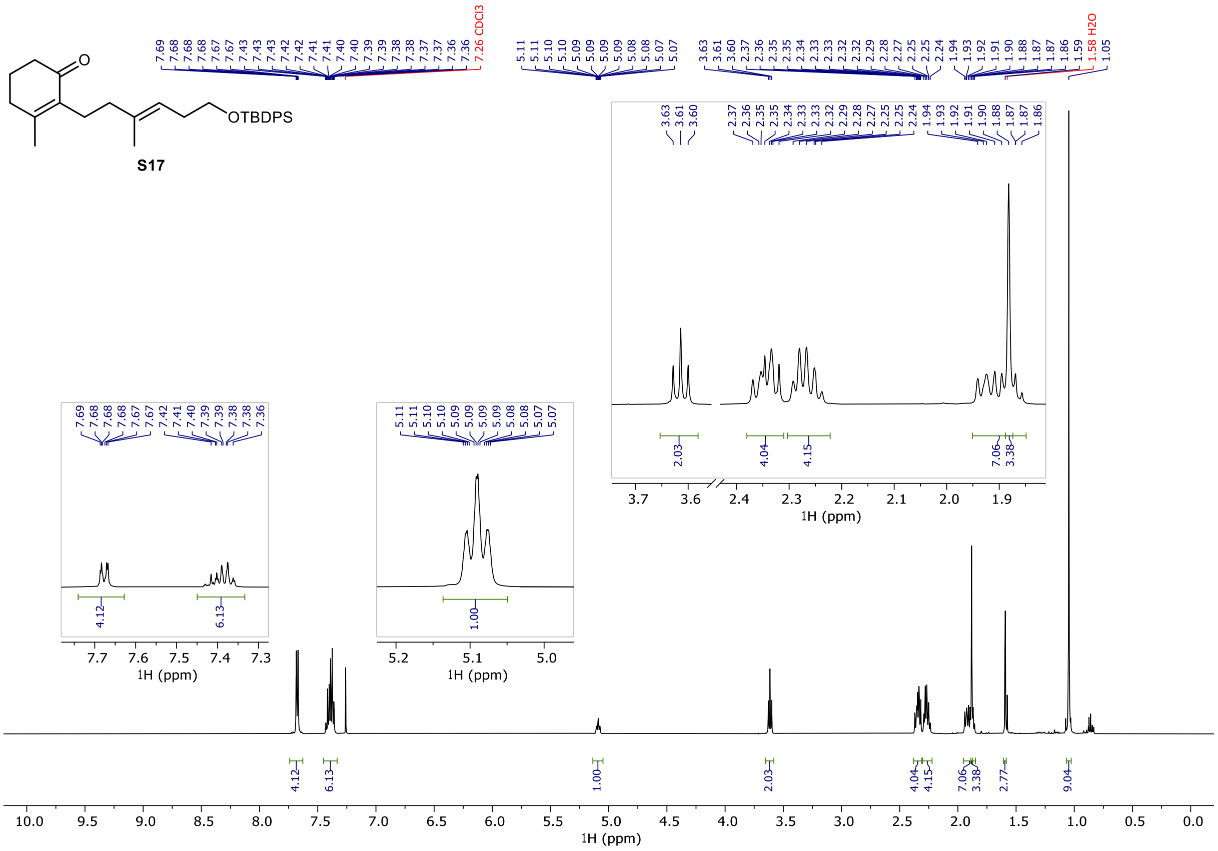

^{1}**H NMR** (501 MHz, $CDCl_3$) spectrum of compound **S17**

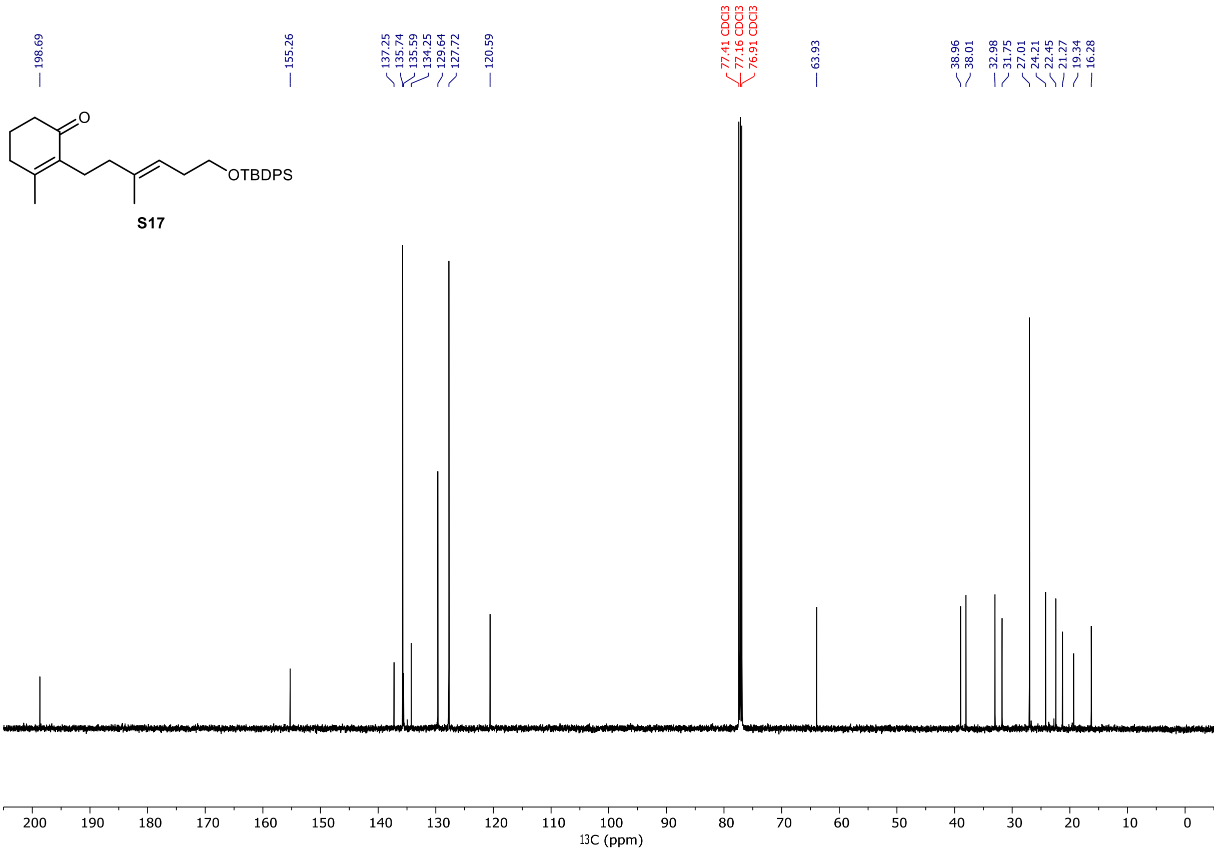

^{13}**C NMR** (126 MHz, $CDCl_3$) spectrum of compound **S17**

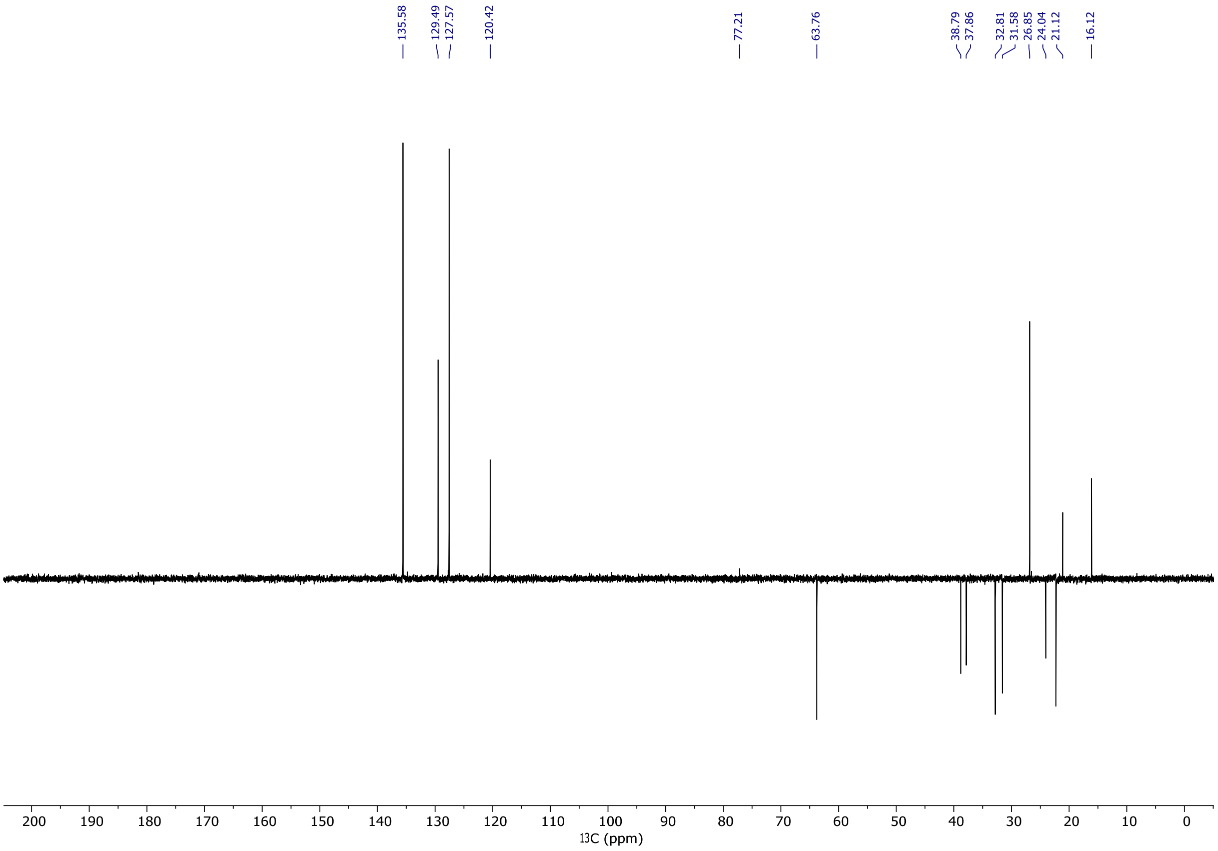

135-DEPT NMR (126 MHz, CD_2Cl_2) spectrum of compound **S17**

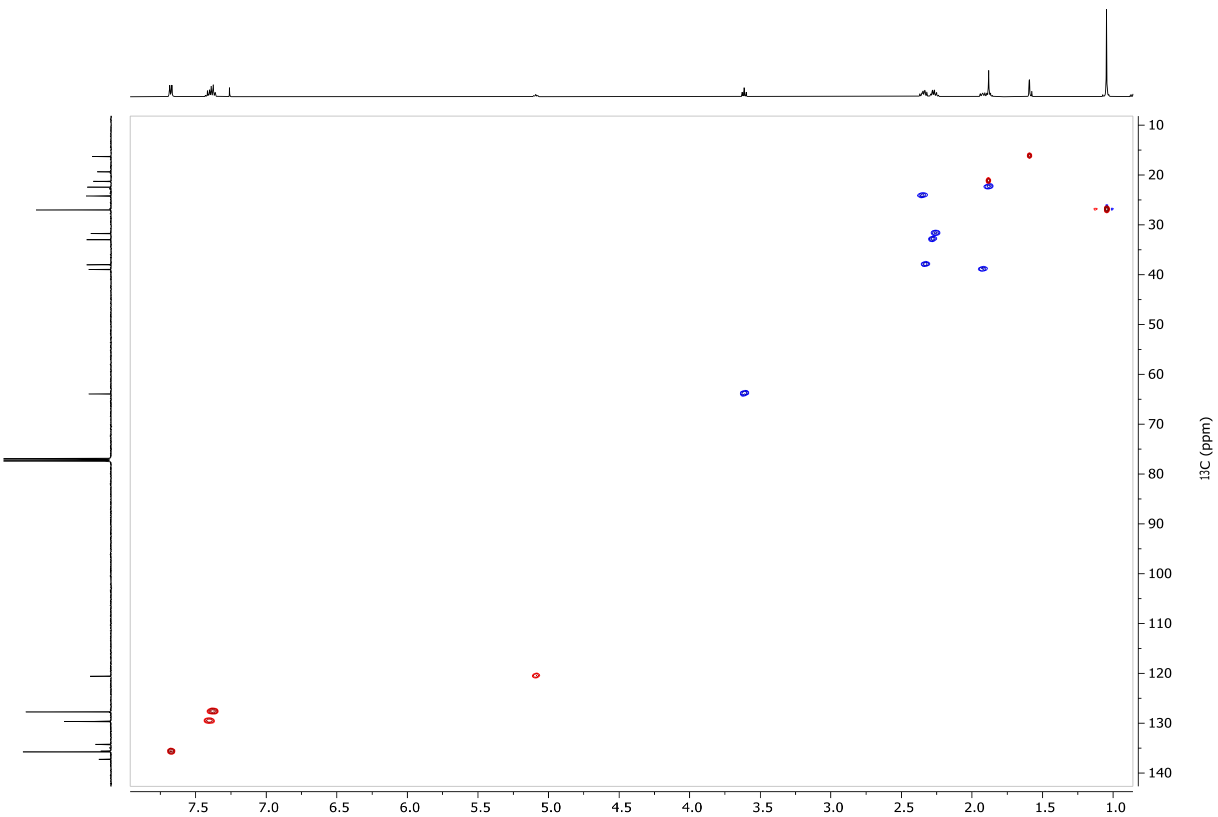

^{1}H,^{13}C-HSQC (501 MHz, 126 MHz, $CDCl_3$) spectrum of compound **S17**

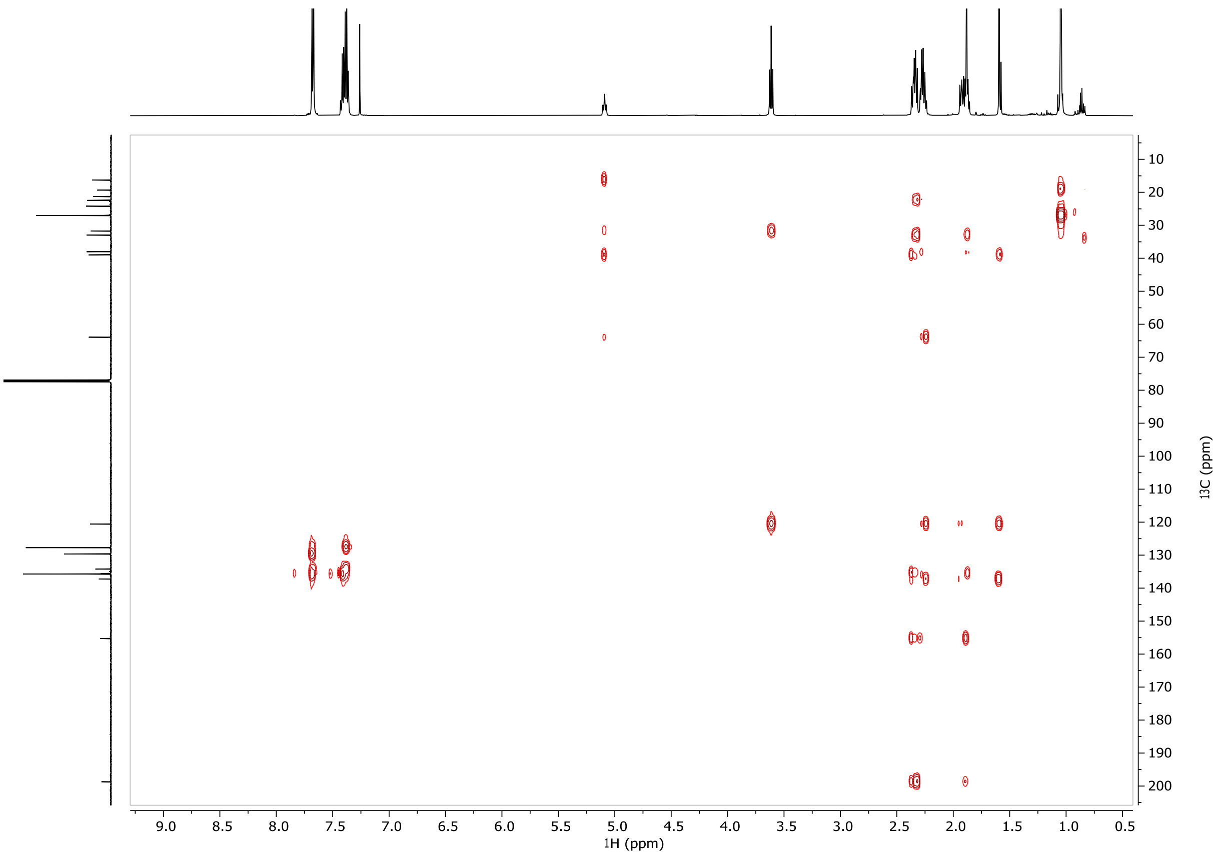

^{1}H,^{13}C-HMBC(501 MHz, 126 MHz $CDCl_3$) spectrum of compound **S17**

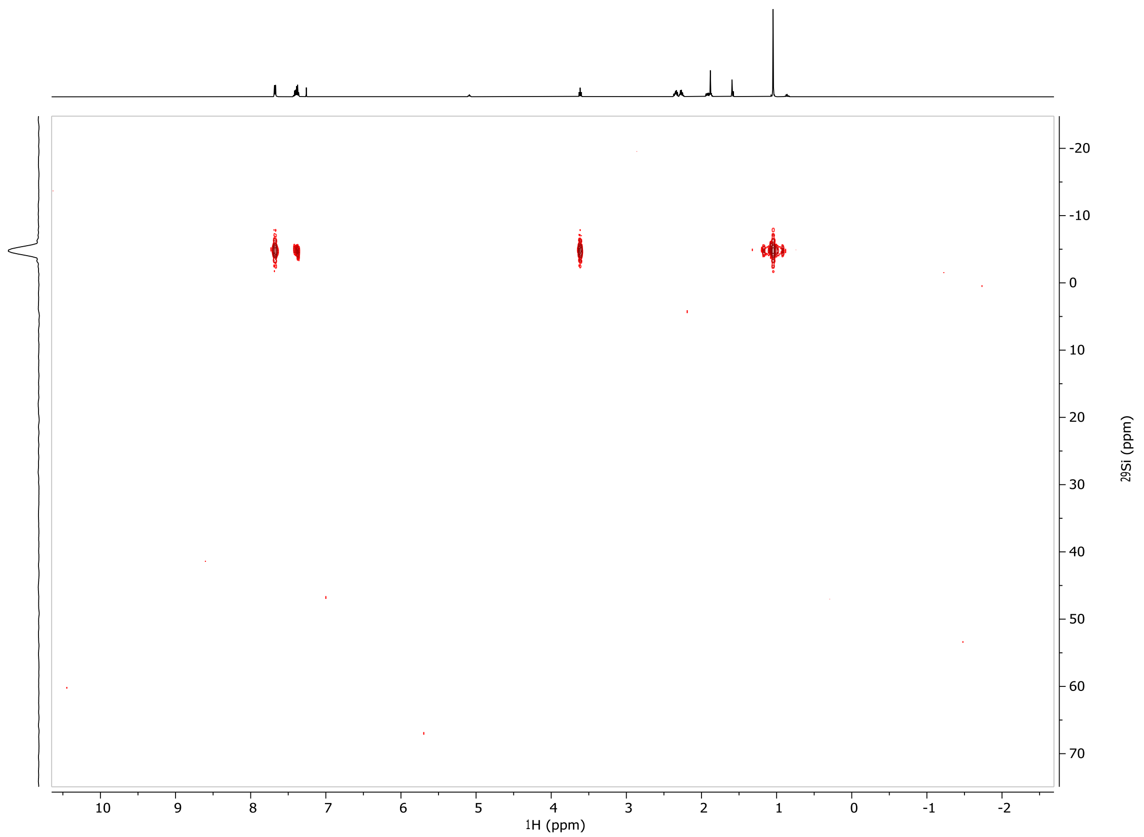

^{1}H,^{29}Si-HMBC (501 MHz, 99 MHz, $CDCl_3$) spectrum of compound **S17**

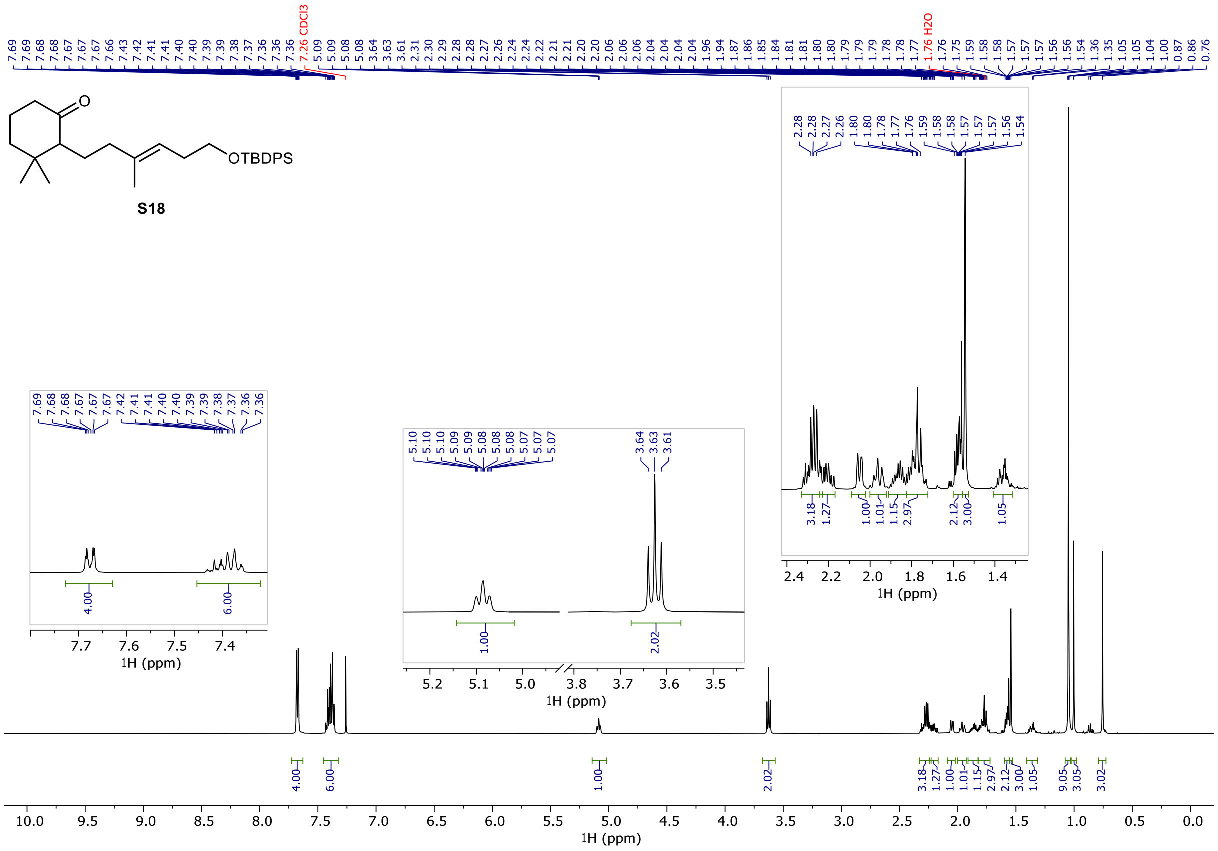

^{1}H NMR (501 MHz, $CDCl_3$) spectrum of compound **S18**

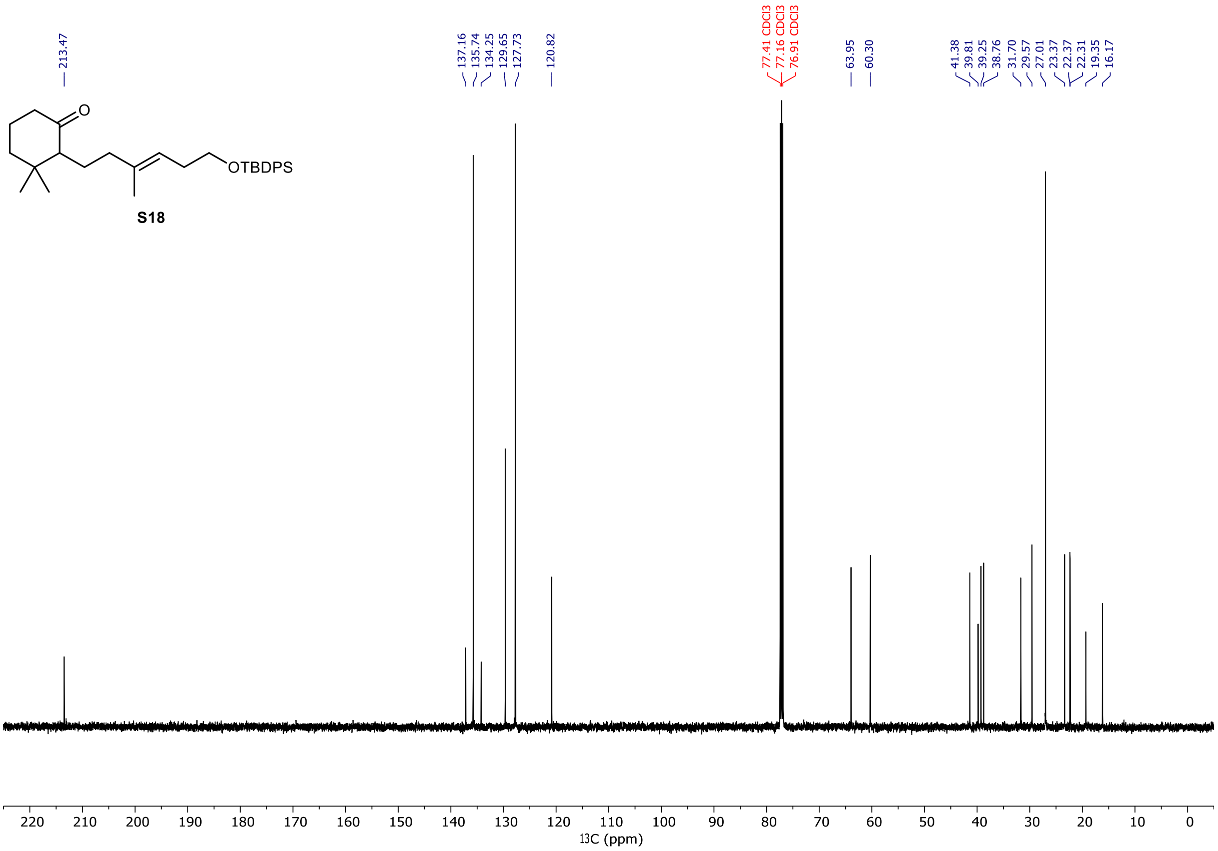

^{13}C NMR (126 MHz, $CDCl_3$) spectrum of compound **S18**

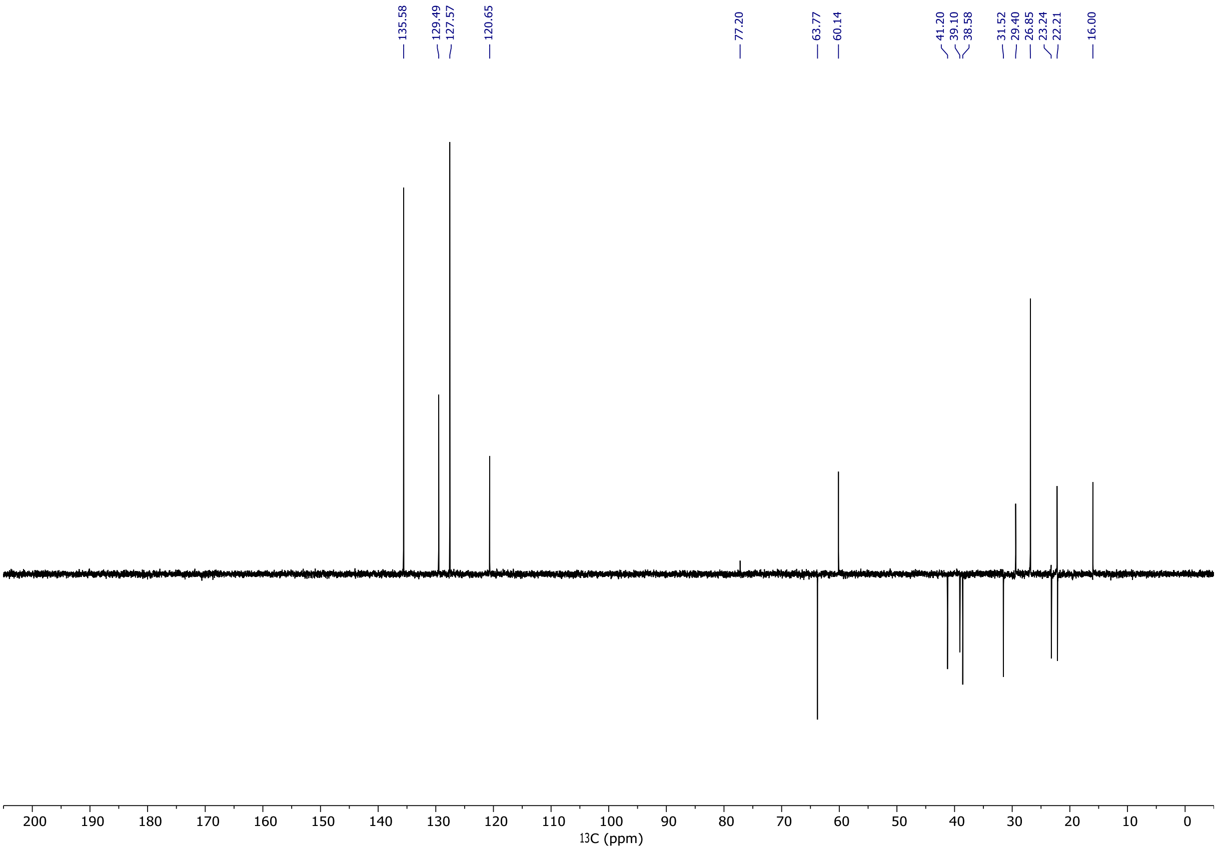

135-DEPT NMR (126 MHz, $CDCl_3$) spectrum of compound **S18**

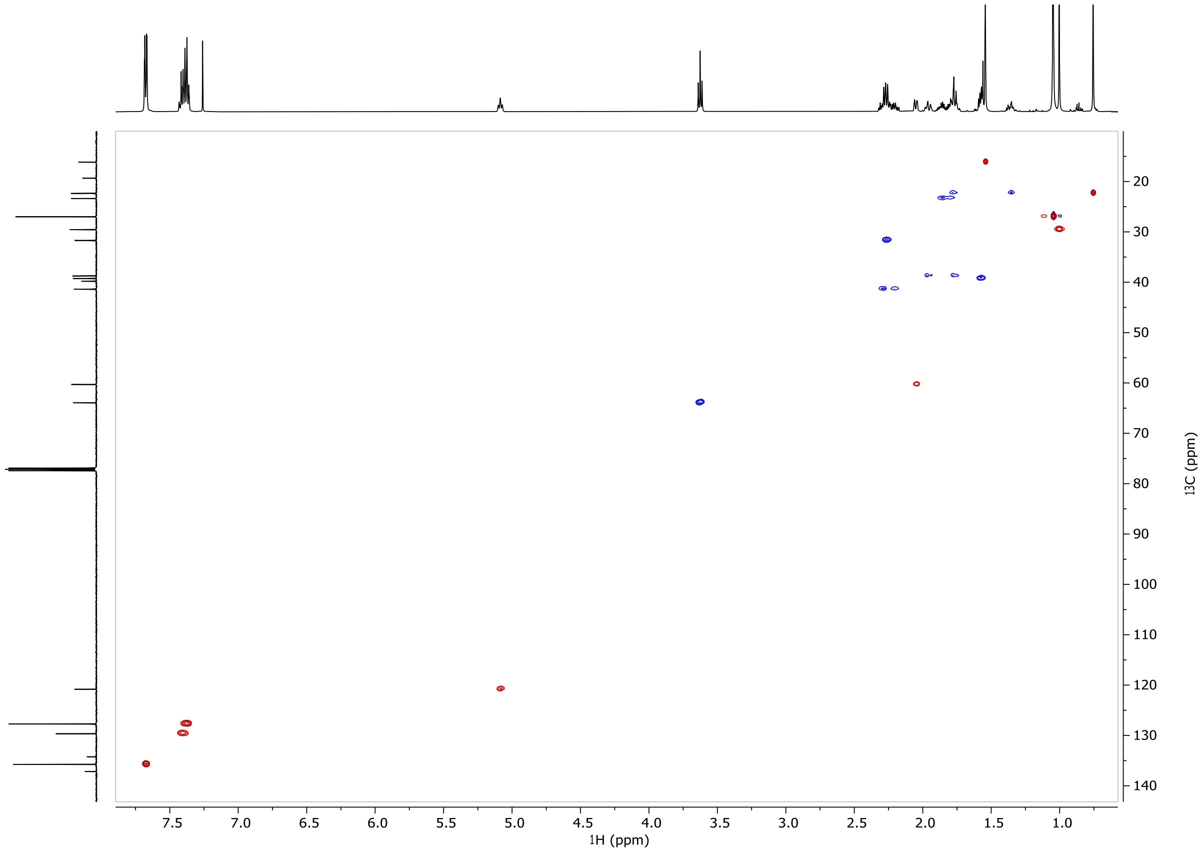

^{1}H,^{13}C-HSQC (501 MHz, 126 MHz, $CDCl_3$) spectrum of compound **S18**

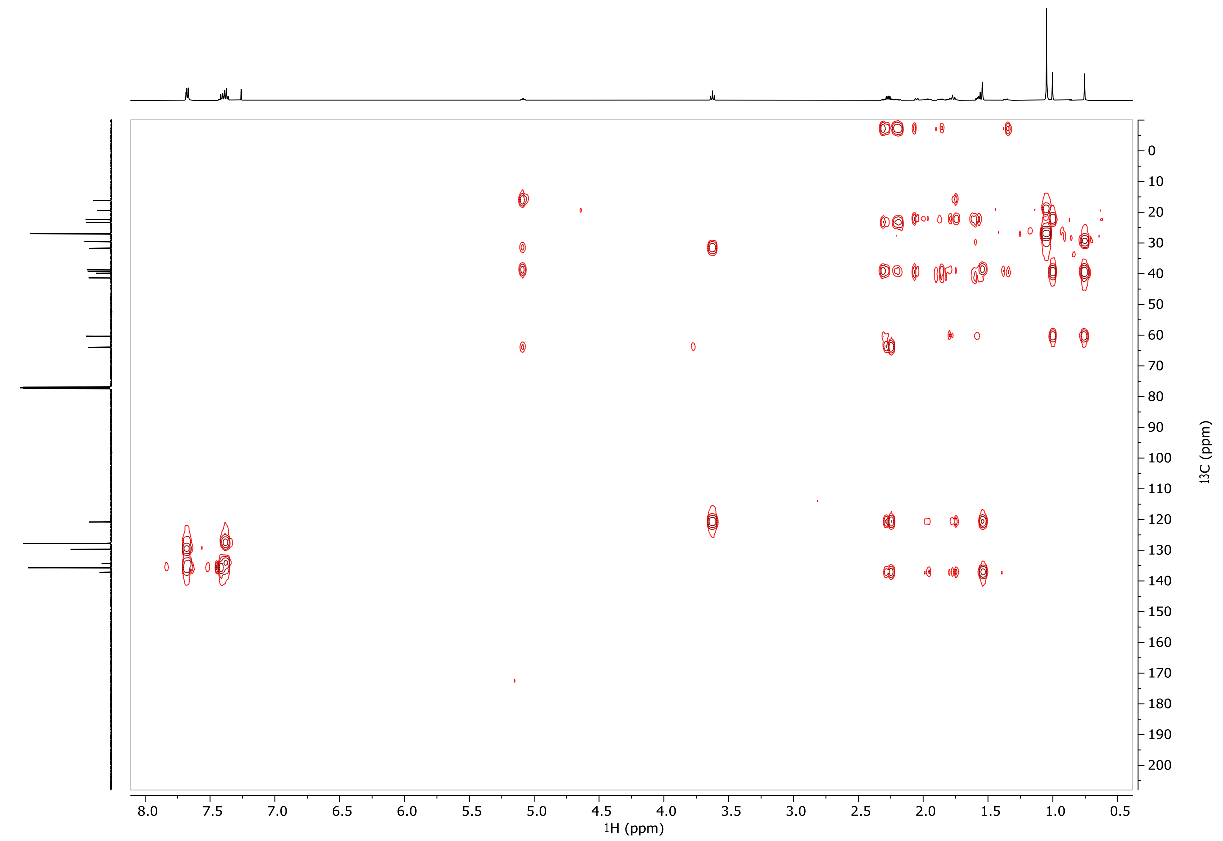

^{1}H,^{13}C-HMBC(501 MHz, 126 MHz, $CDCl_3$) spectrum of compound **S18**

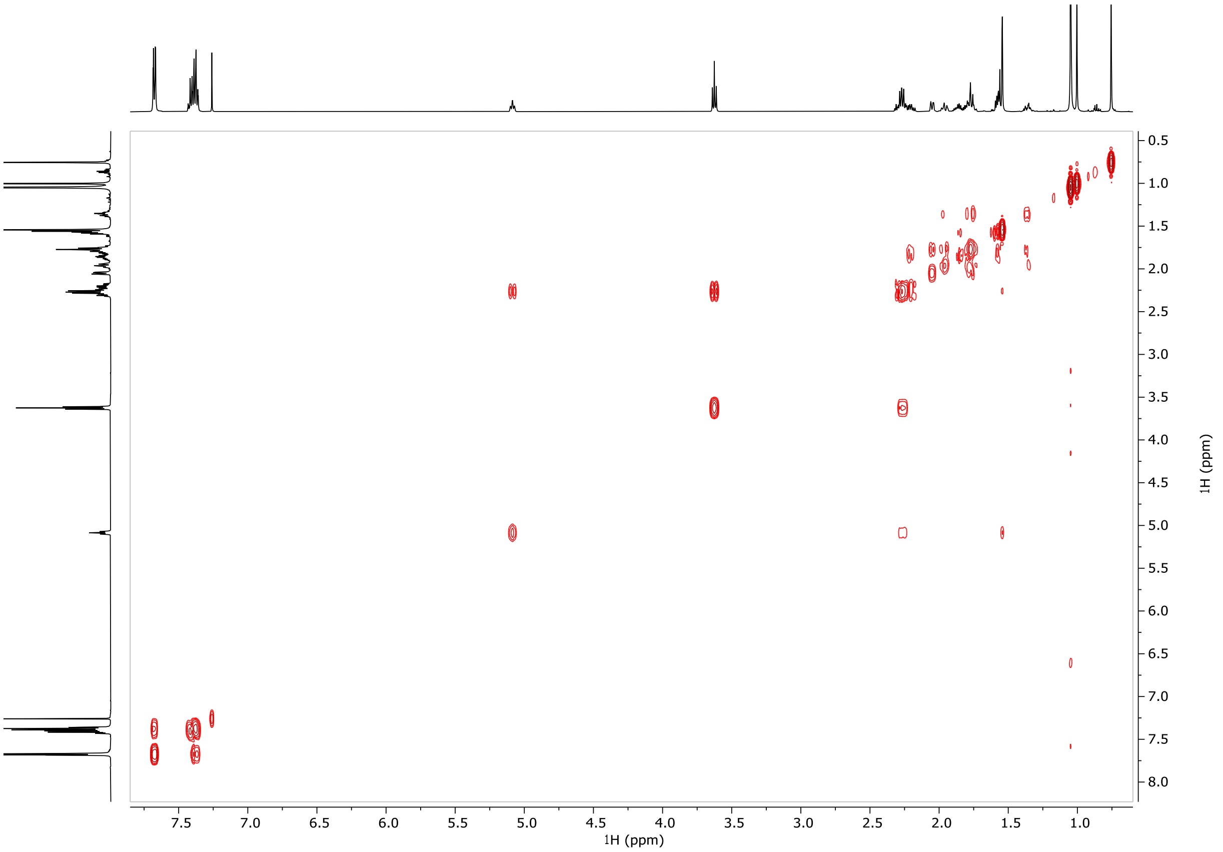

^{1}H,^{1}H-COSY (501 MHz, $CDCl_3$) spectrum of compound **S18**

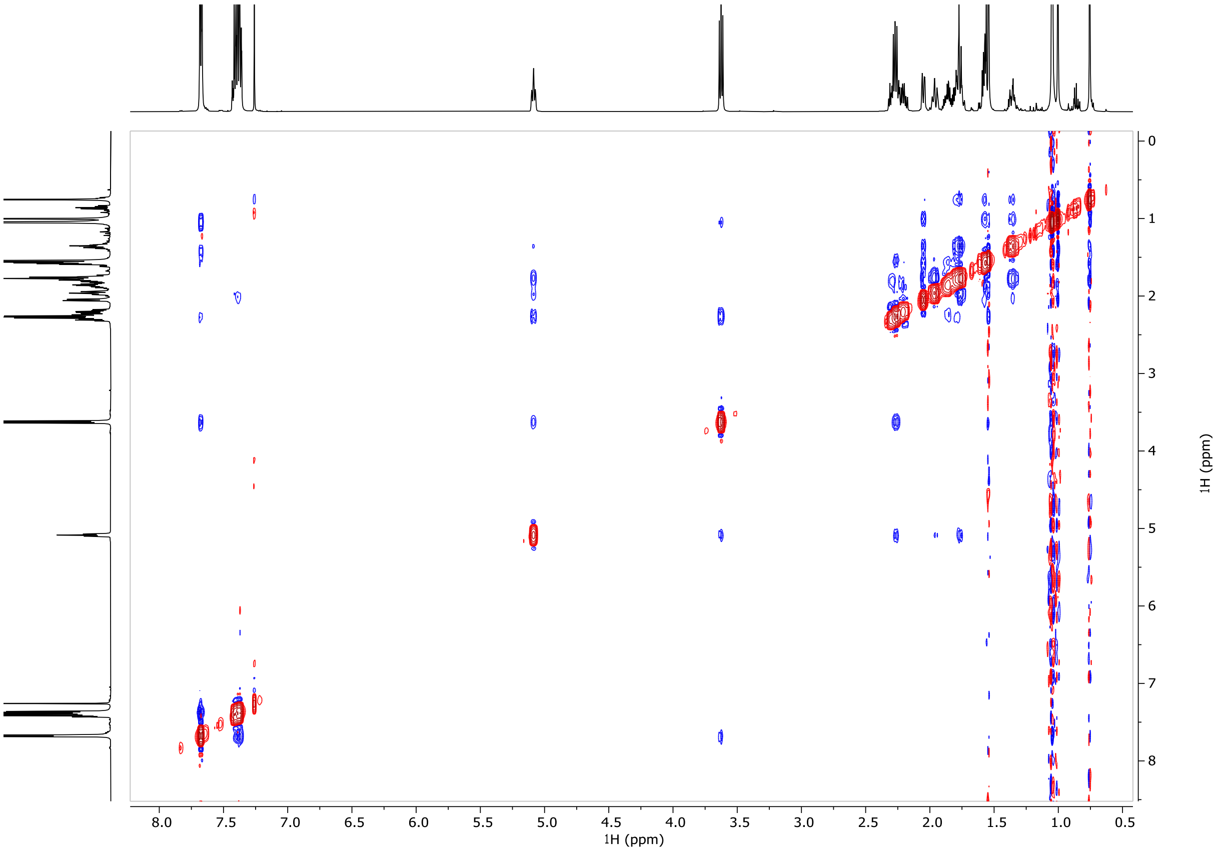

^{1}H,^{1}H-NOESY (501 MHz, $CDCl_3$) spectrum of compound **S18**

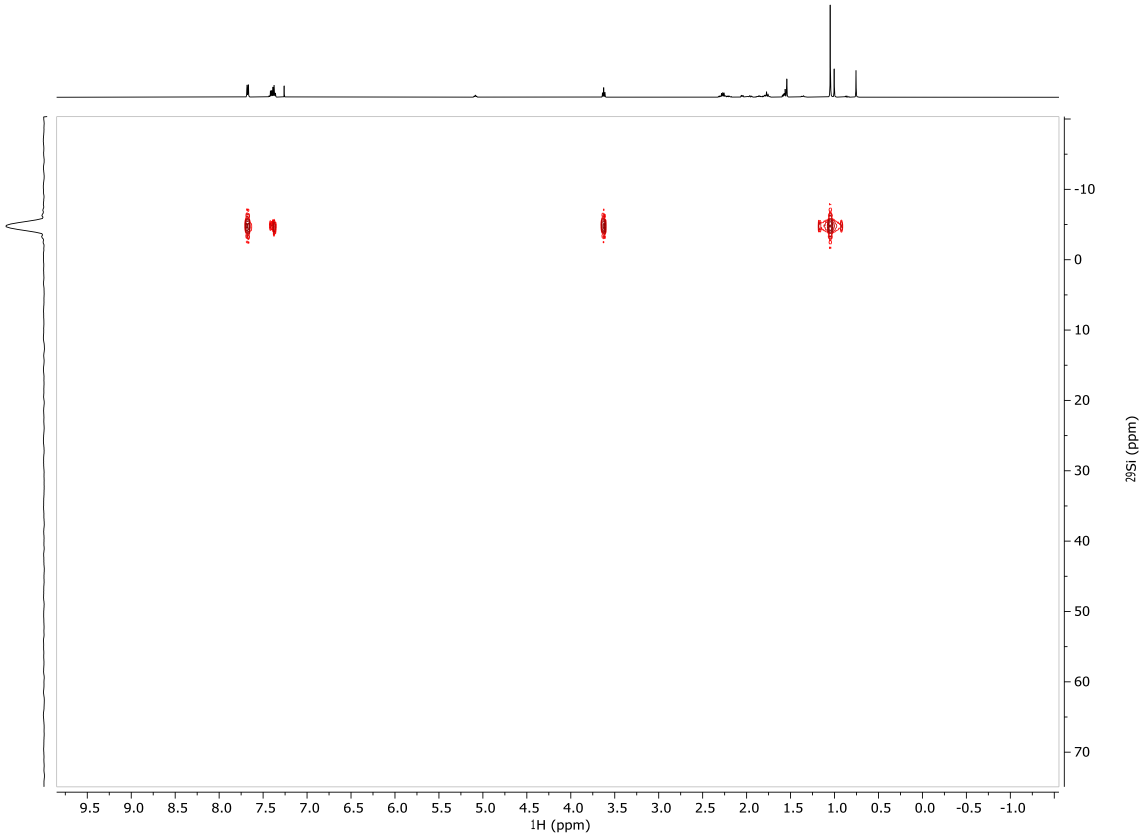

^{1}H,^{29}Si-HMBC (501 MHz, 99 MHz, $CDCl_3$) spectrum of compound **S18**

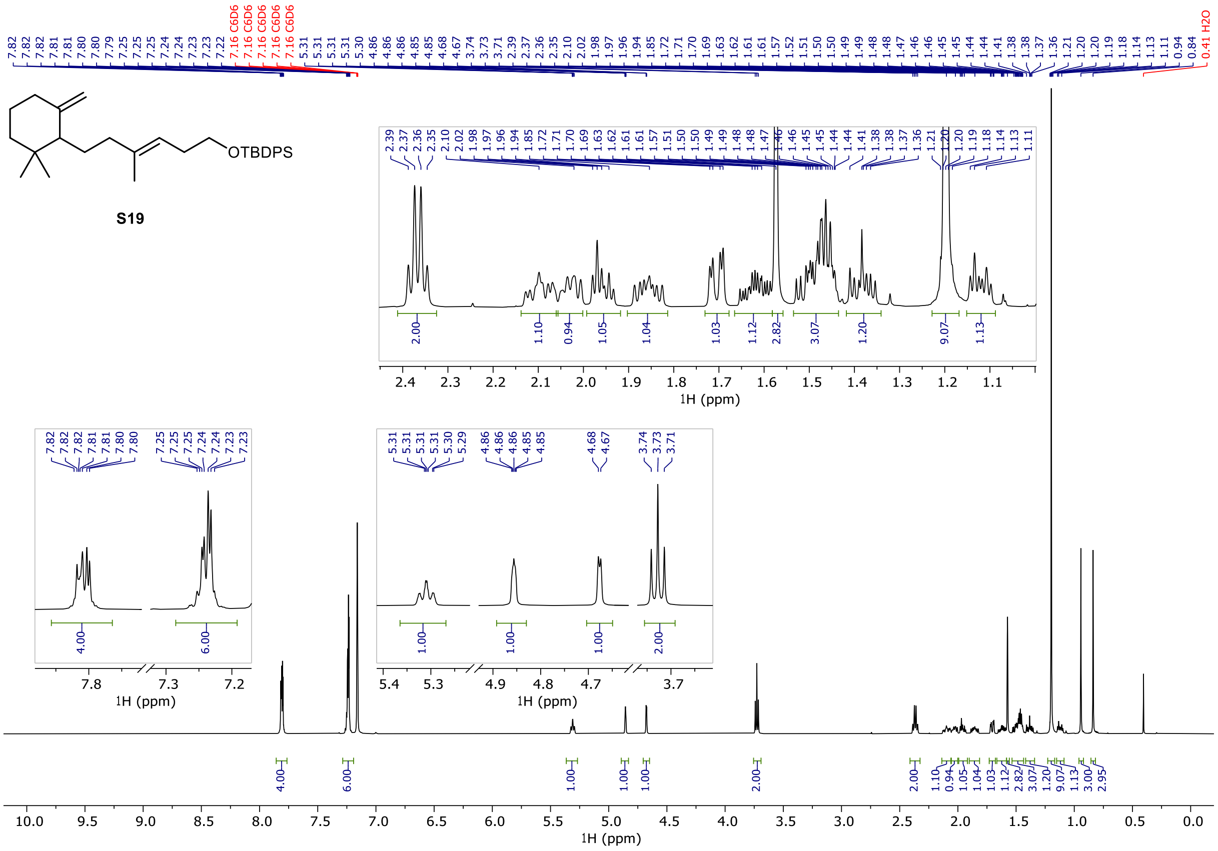

^{1}H NMR (501 MHz, benzene-d_6) spectrum of compound **S19**

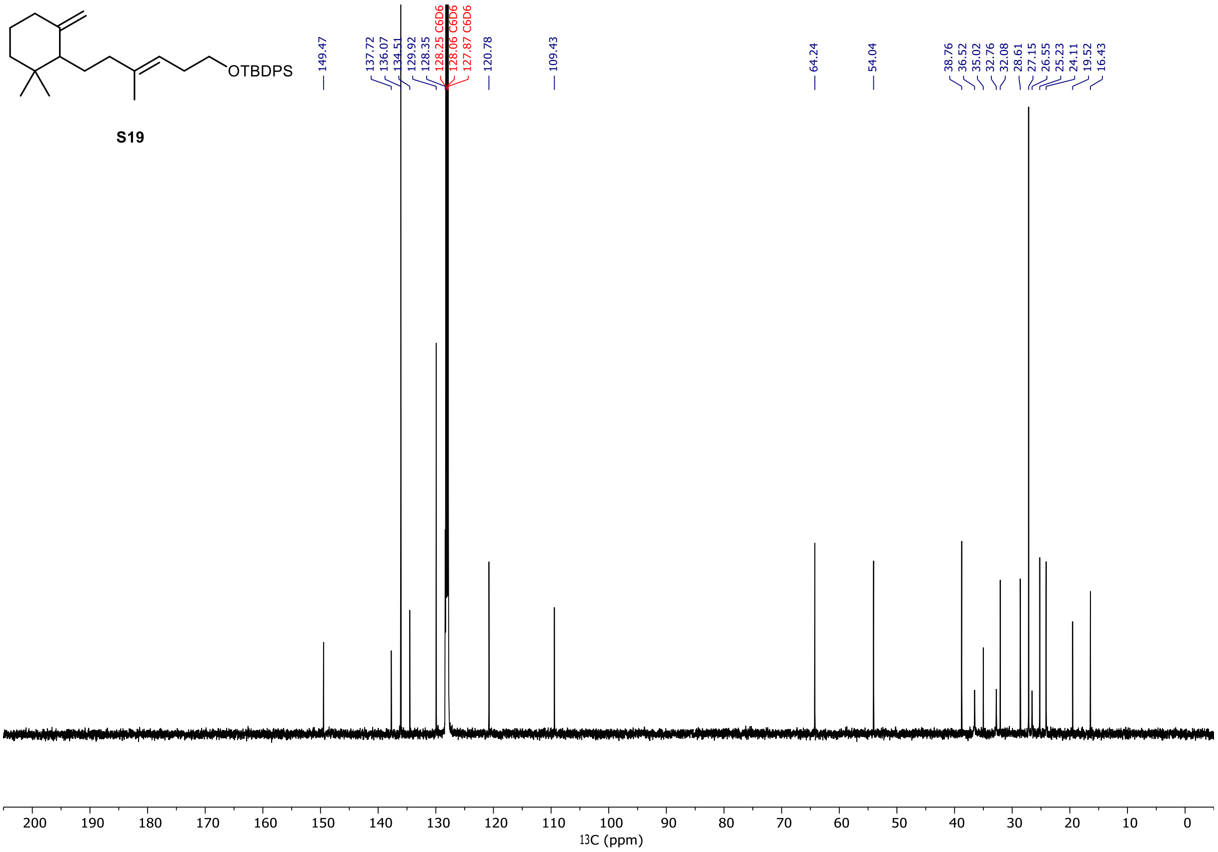

^{13}C NMR (126 MHz, benzene-d_6) spectrum of compound **S19**

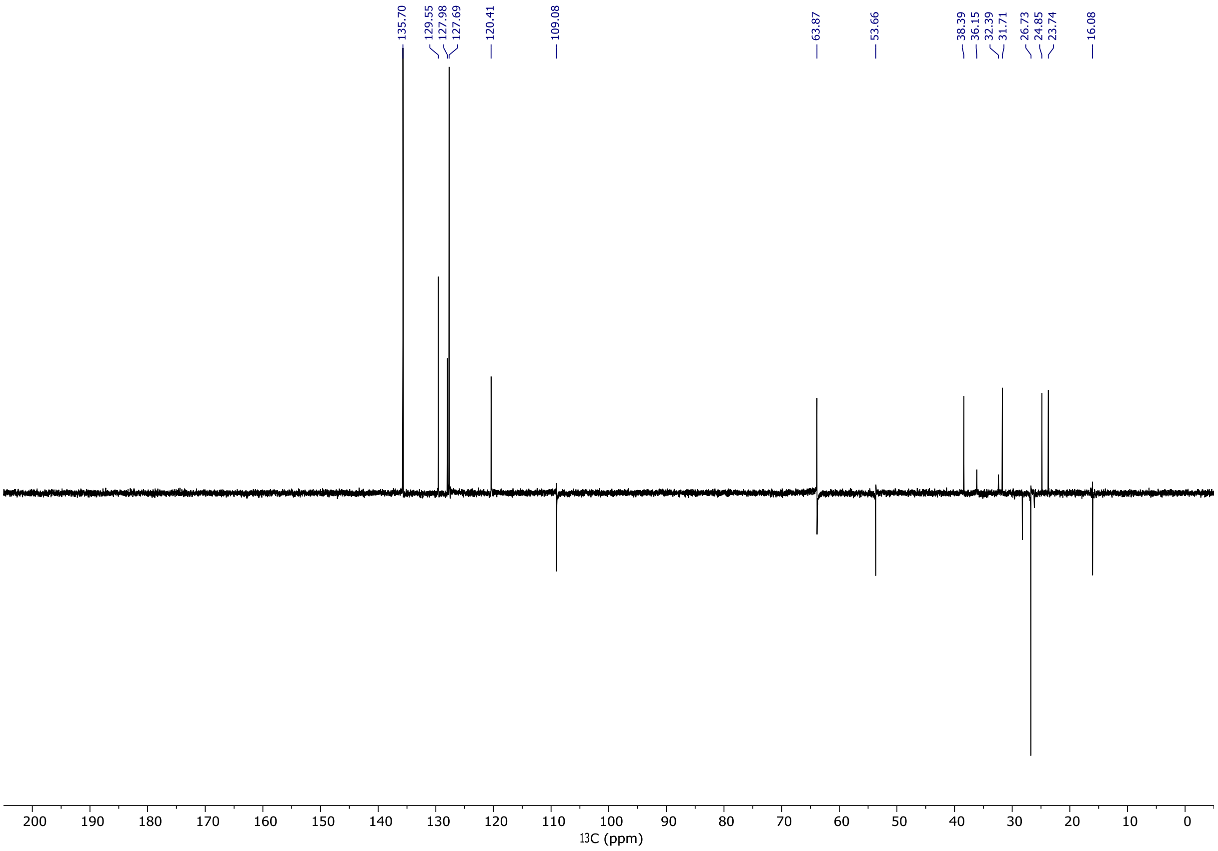

135-DEPT NMR (126 MHz, benzene-d_6) spectrum of compound **S19**

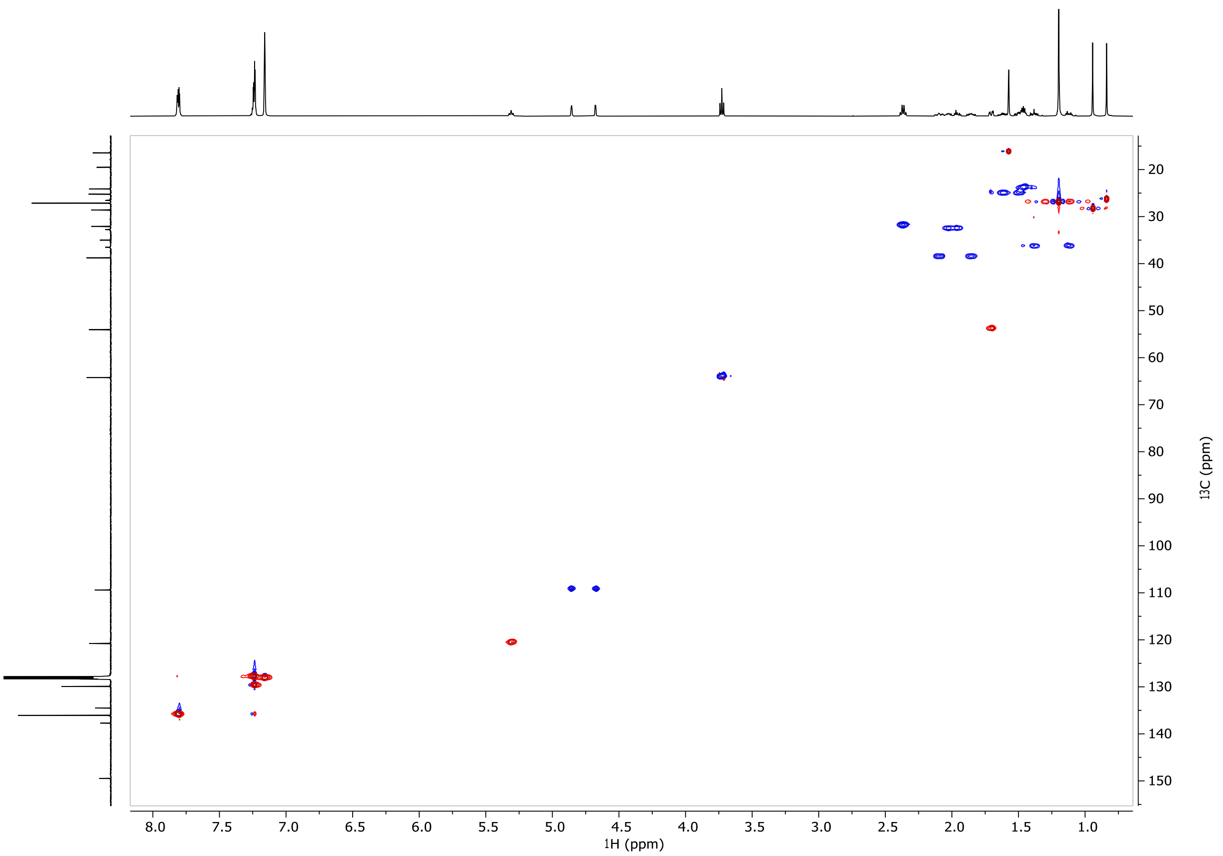

^{1}H,^{13}C-HSQC (501 MHz, 126 MHz, benzene-d_6) spectrum of compound **S19**

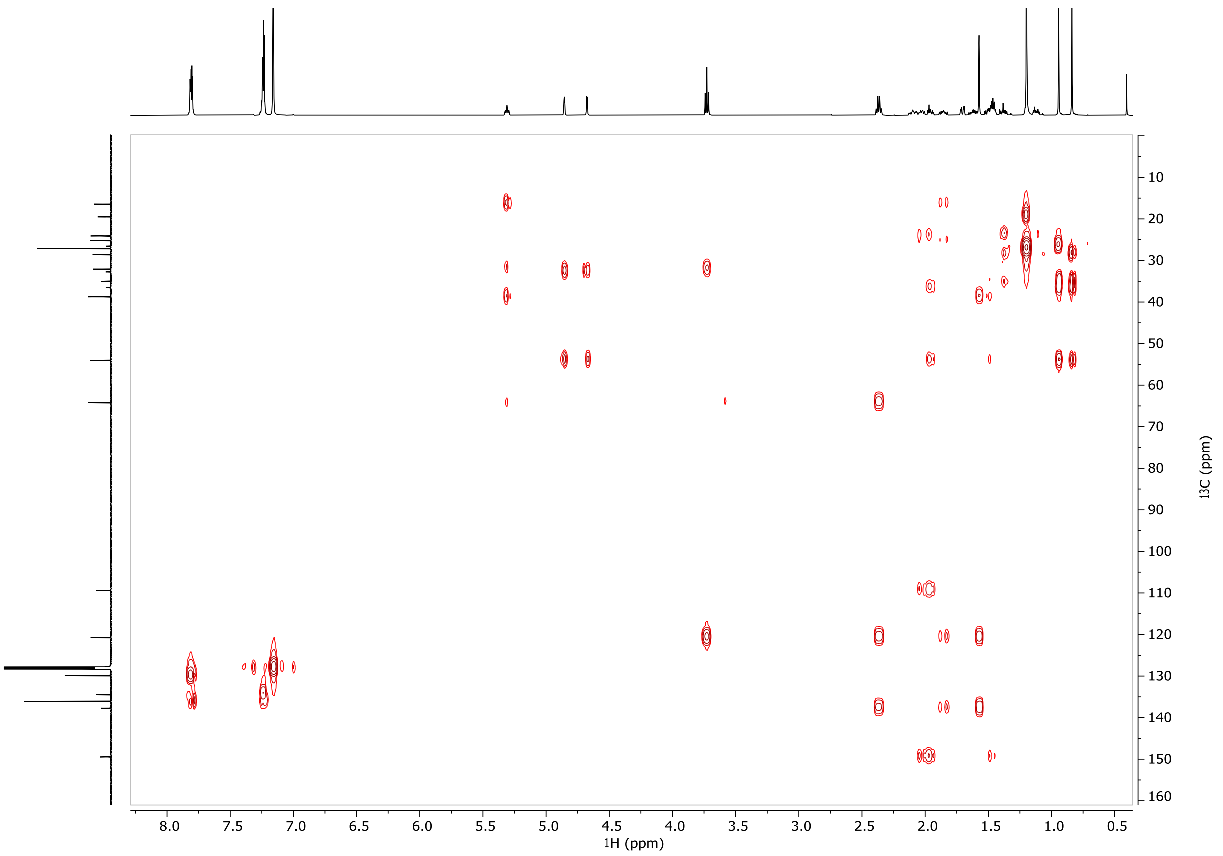

^{1}H,^{13}C-HMBC(501 MHz, 126 MH, benzene-d_6) spectrum of compound **S19**

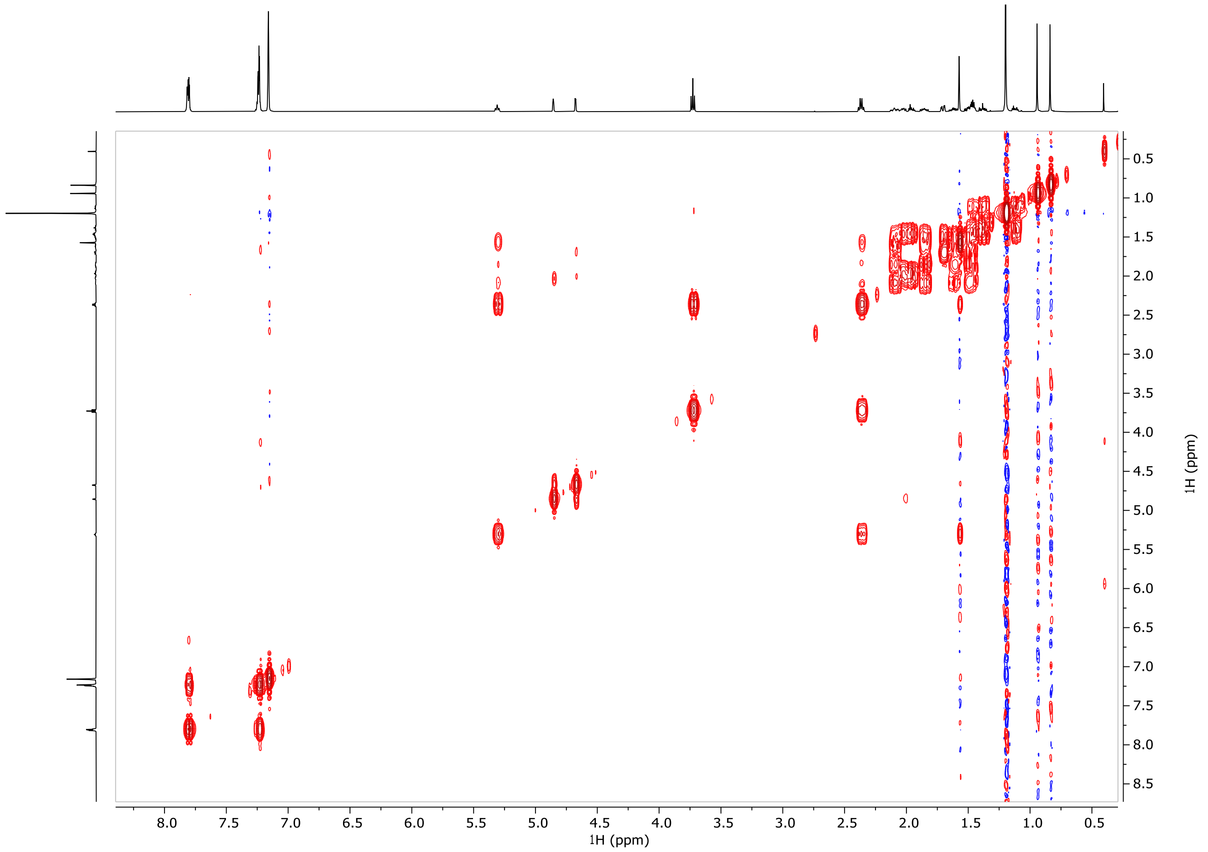

^{1}H,^{1}H-COSY (501 MHz, benzene-d_6) spectrum of compound **S19**

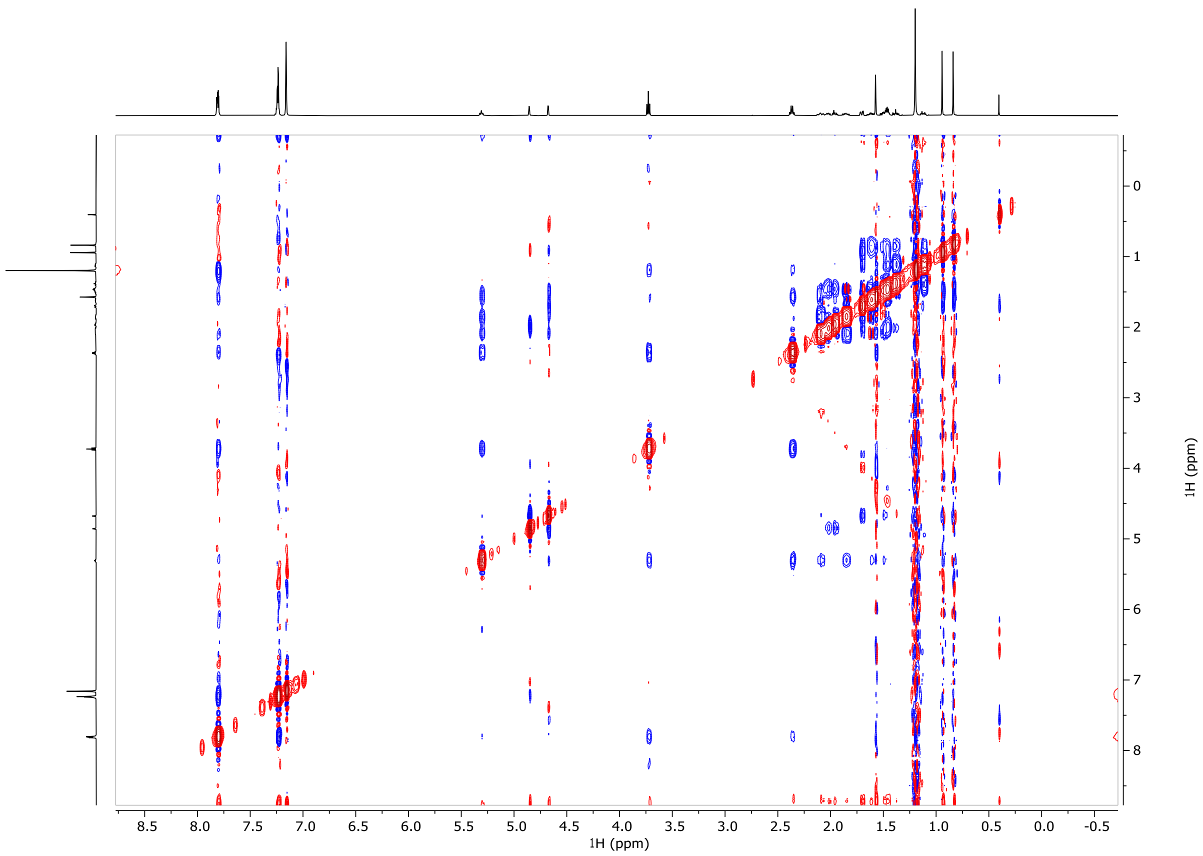

^{1}H,^{1}H-NOESY (501 MHz, benzene-d_6) spectrum of compound **S19**

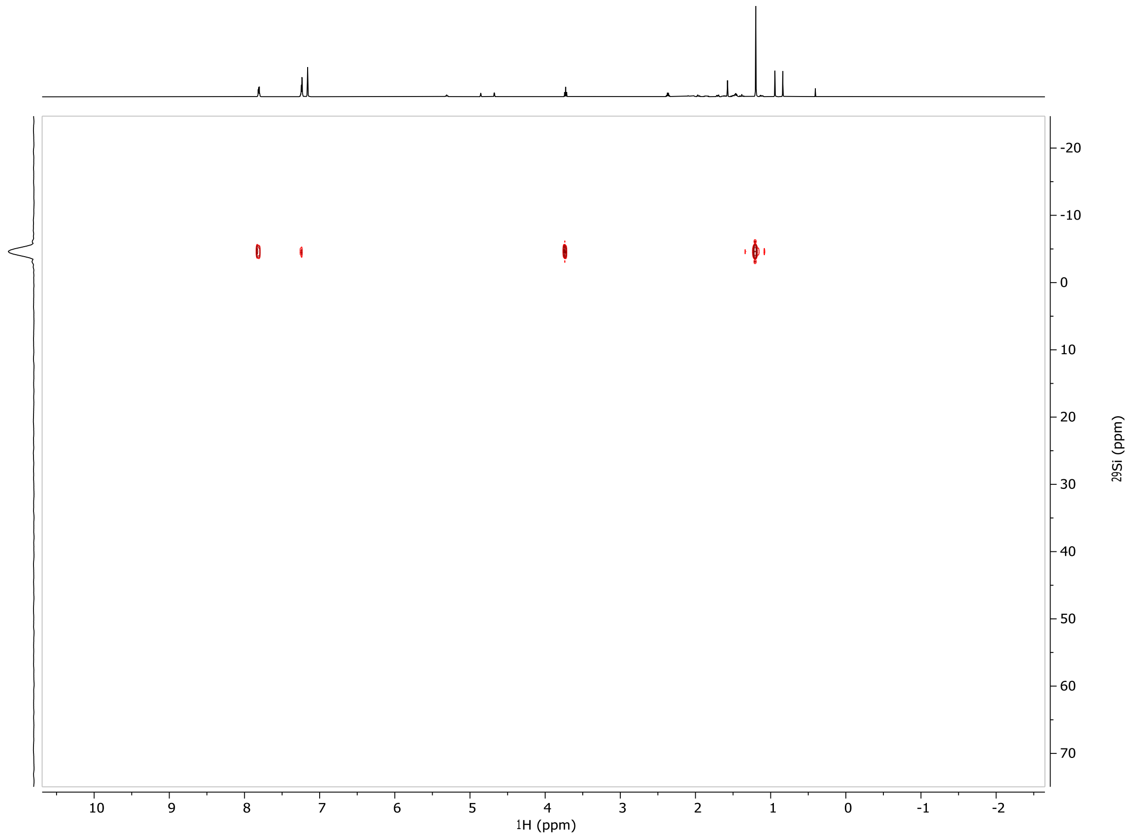

^{1}H,^{29}Si-HMBC (501 MHz, 99 MHz, benzene-d_6) spectrum of compound **S19**

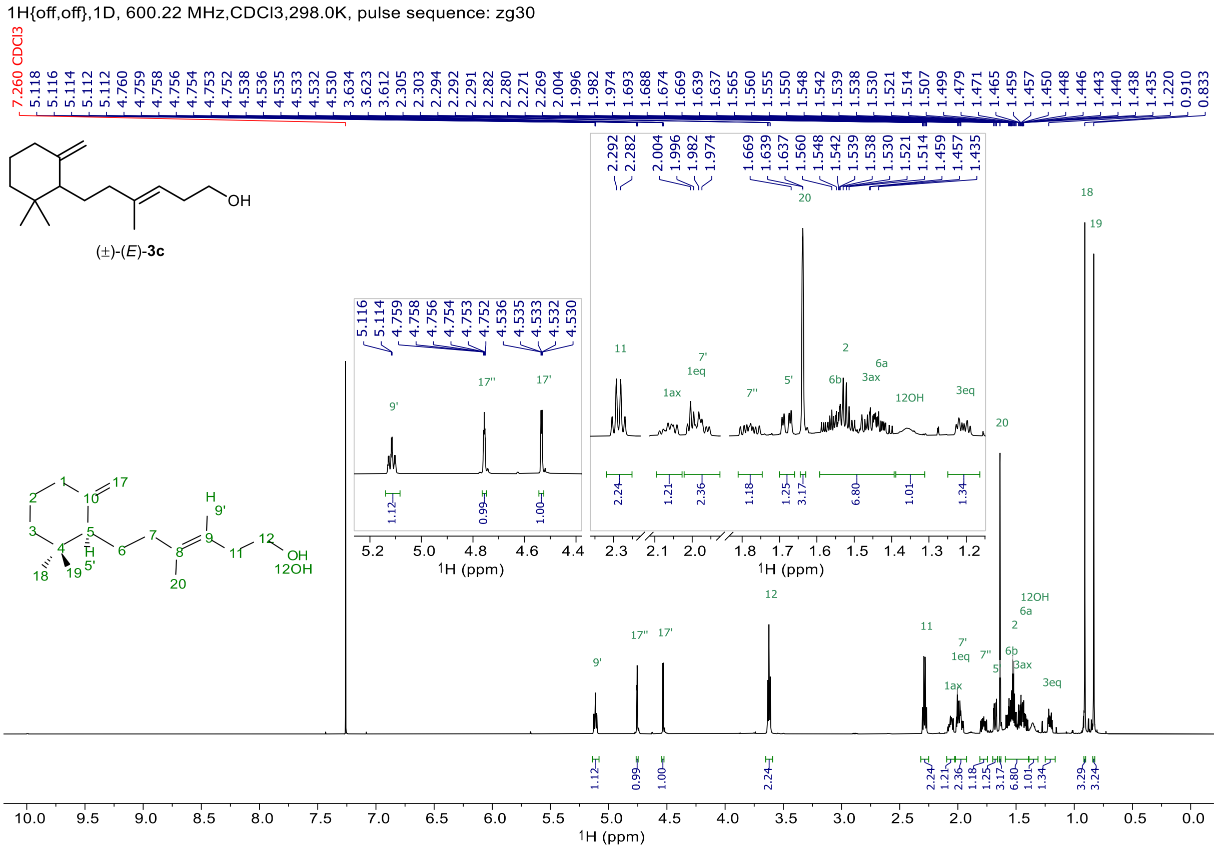

¹H NMR (600 MHz, $CDCl_3$) spectrum of (±)-(*E*)-γ-cyclohomofarnesol **3c** with assignments

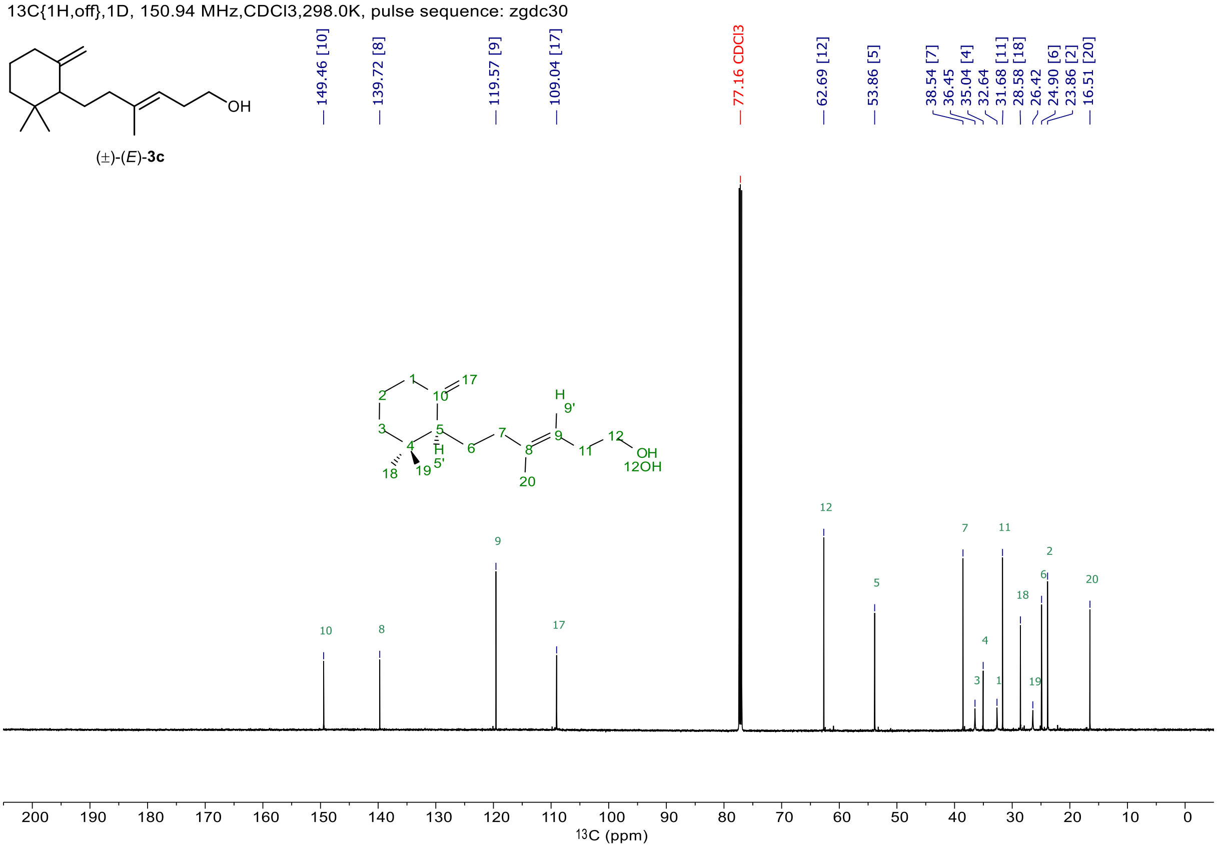

¹³C NMR (151 MHz, $CDCl_3$) spectrum of (±)-(*E*)-γ-cyclohomofarnesol **3c** with assignments

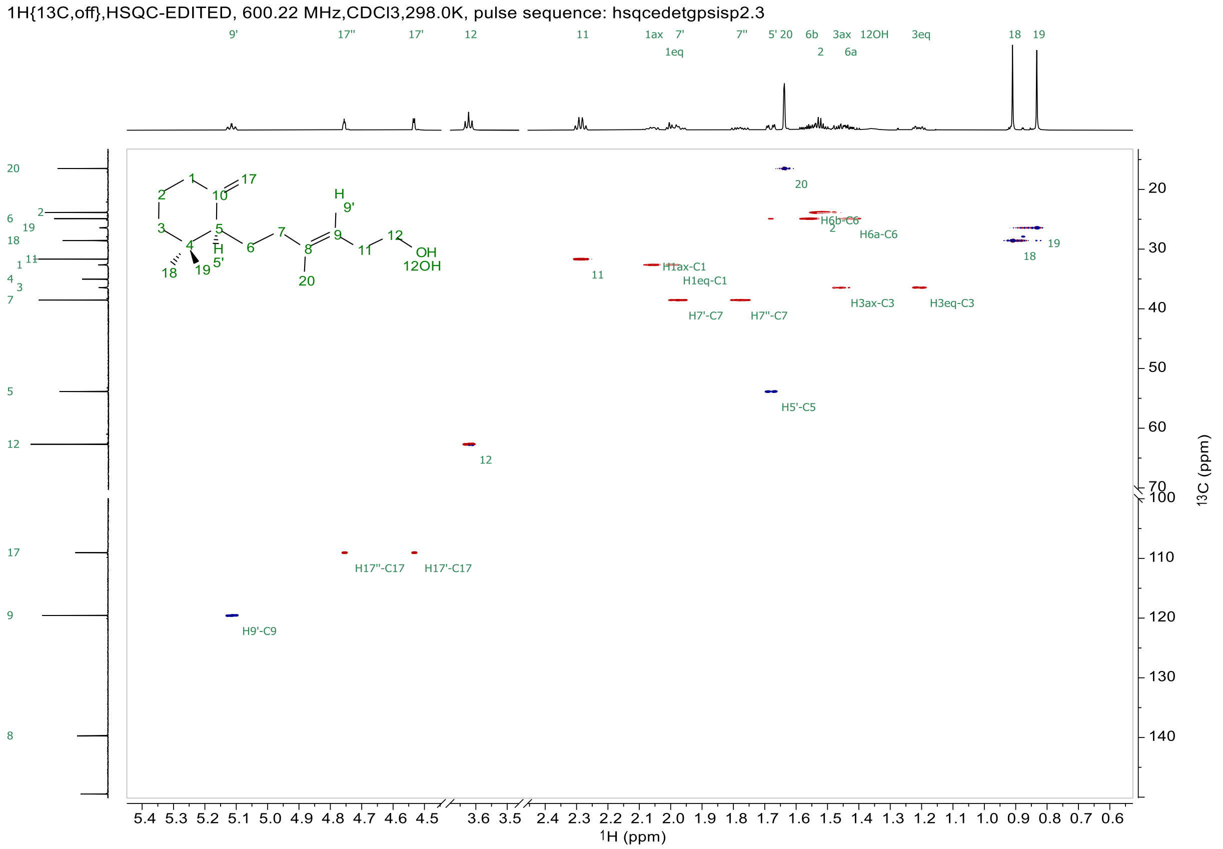

Multiplicity-edited **^{1}H,^{13}C-HSQC** (600 MHz, 151 MHz) spectrum of (±)-(*E*)-γ-cyclohomofarnesol **3c** with assignments

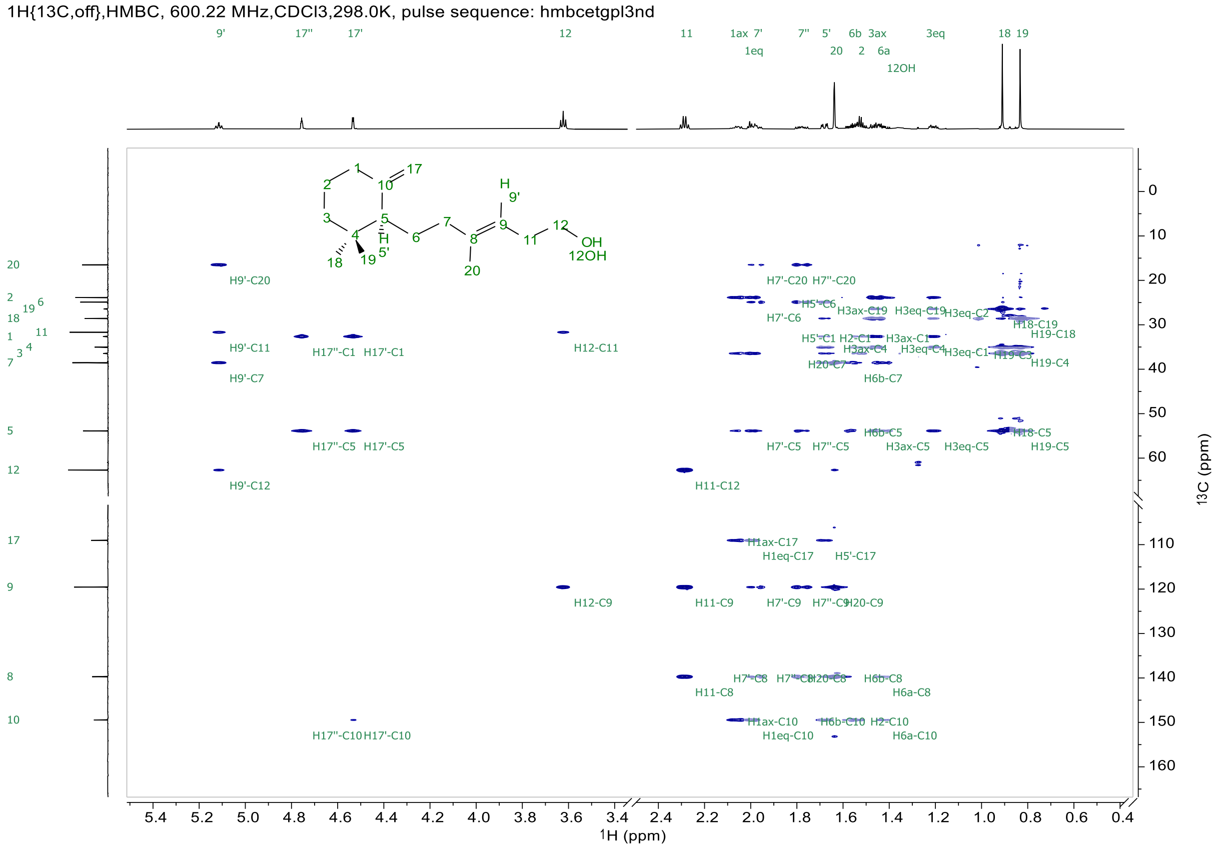

^{1}H,^{13}C-HMBC (600 MHz, 151 MHz) spectrum of (±)-(*E*)-γ-cyclohomofarnesol **3c** with assignments

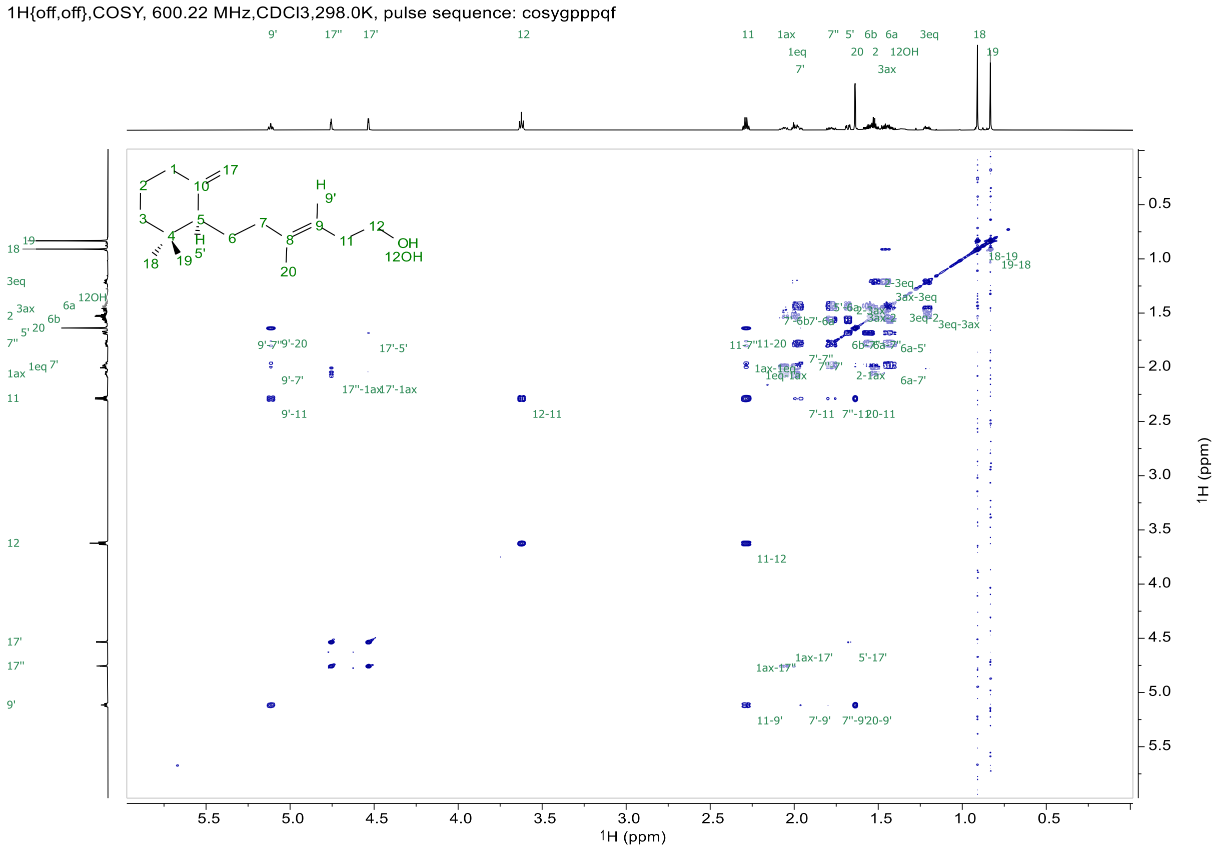

^{1}**H,**^{1}**H-COSY** (600 MHz, $CDCl_3$) spectrum of (±)-(*E*)-γ-cyclohomofarnesol (**3c**) with assignments

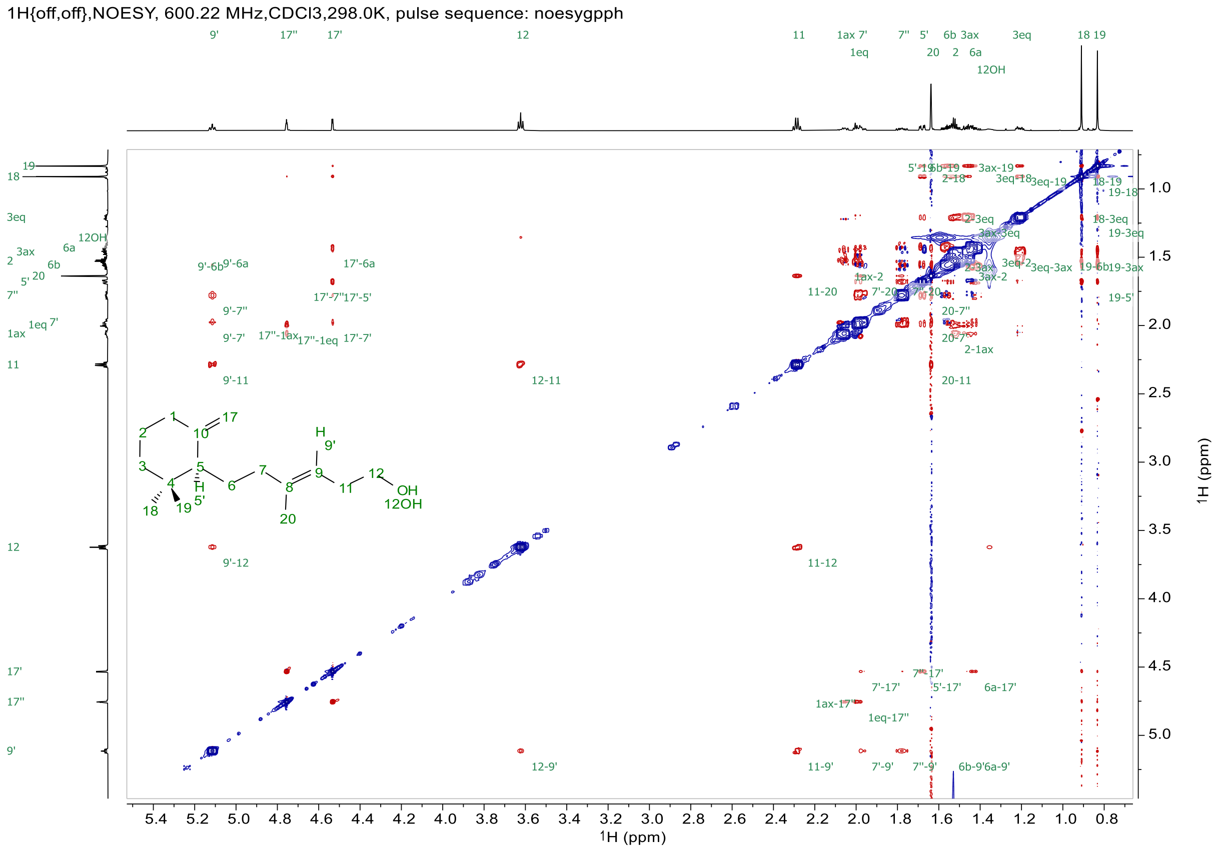

^{1}**H,**^{1}**H-NOESY** (600 MHz, $CDCl_3$) spectrum of (±)-(*E*)-γ-cyclohomofarnesol (**3c**) with assignments

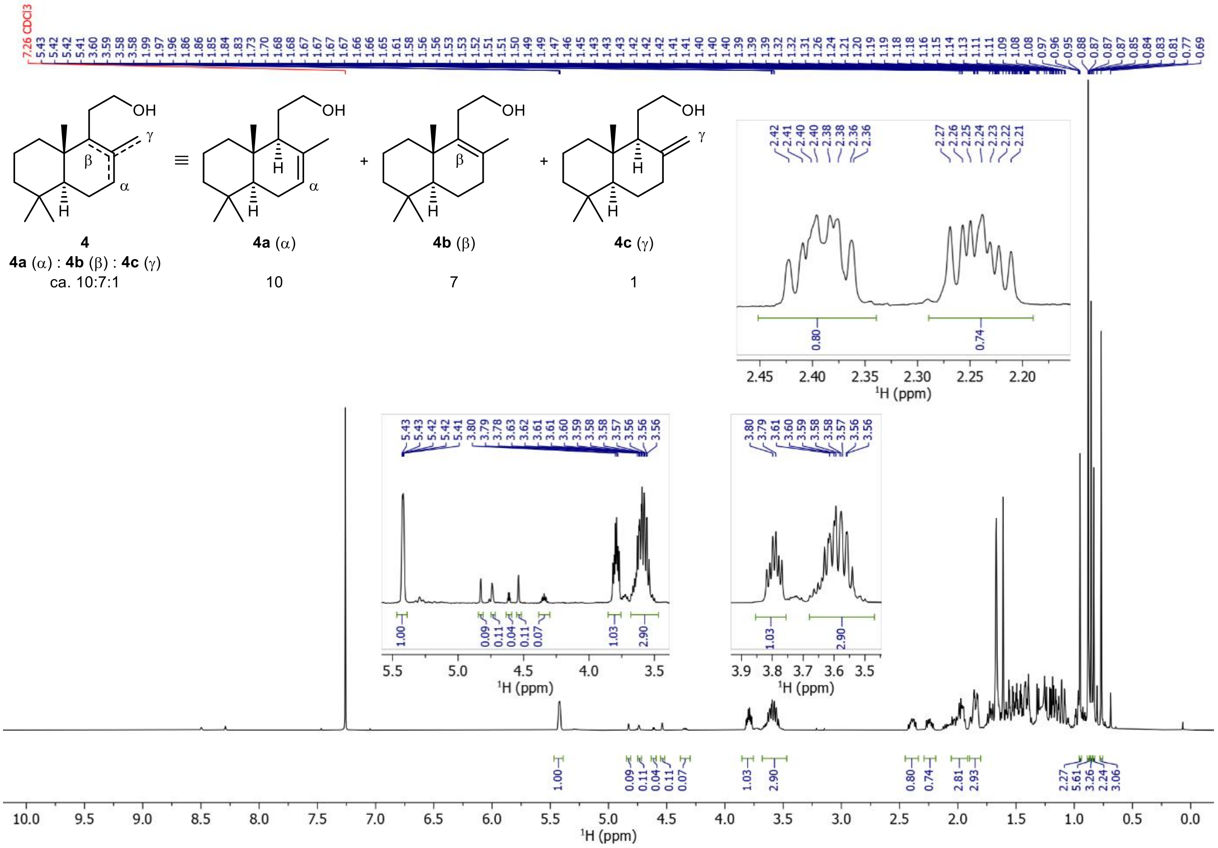

^{1}H NMR (501 MHz, $CDCl_3$) spectrum of a mixture of homodrimenols **4** obtained in trace quantities as side product in the IDPi-catalyzed polyene cyclization of (3*E*,7*E*)-homofarnesol to (−)-ambrox

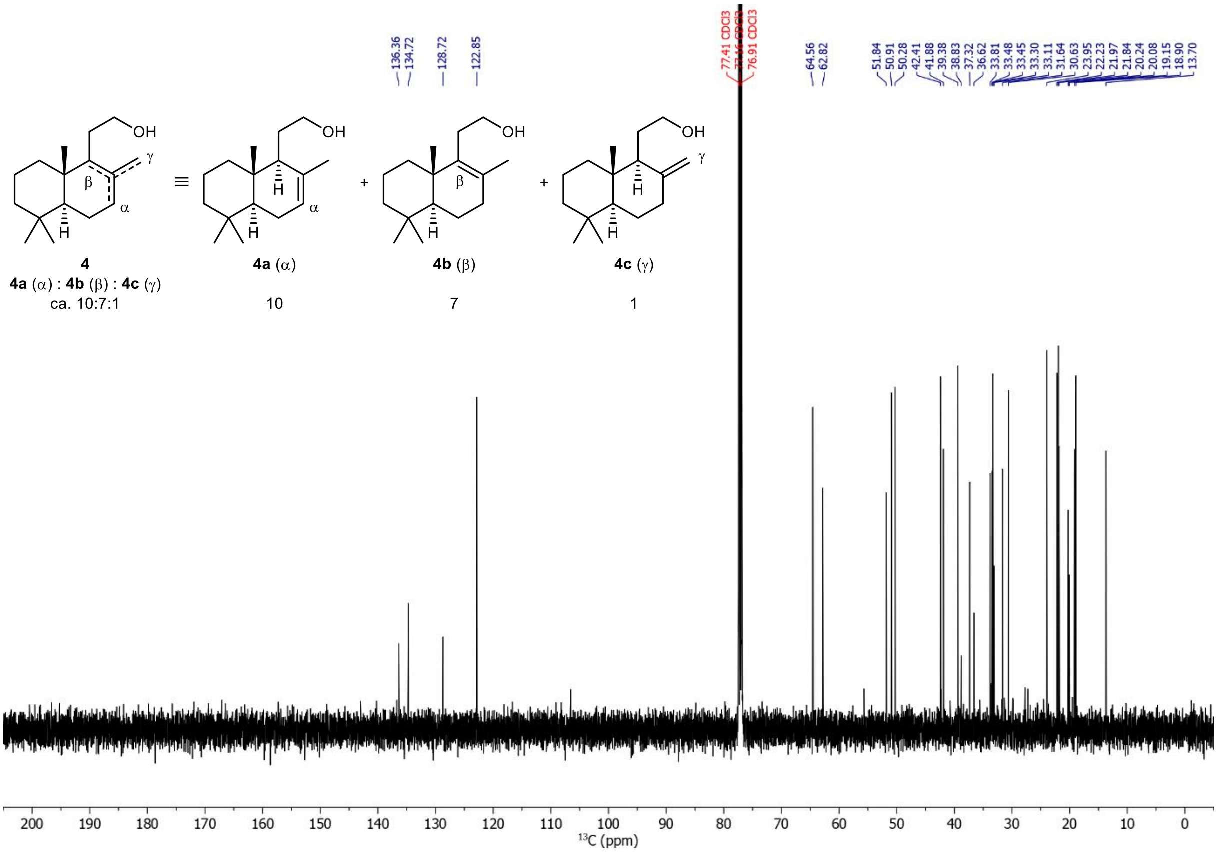

^{13}C NMR (151 MHz, $CDCl_3$) spectrum of a mixture of homodrimenols **4** obtained in trace quantities as side product in the IDPi-catalyzed polyene cyclization of (3*E*,7*E*)-homofarnesol to (−)-ambrox

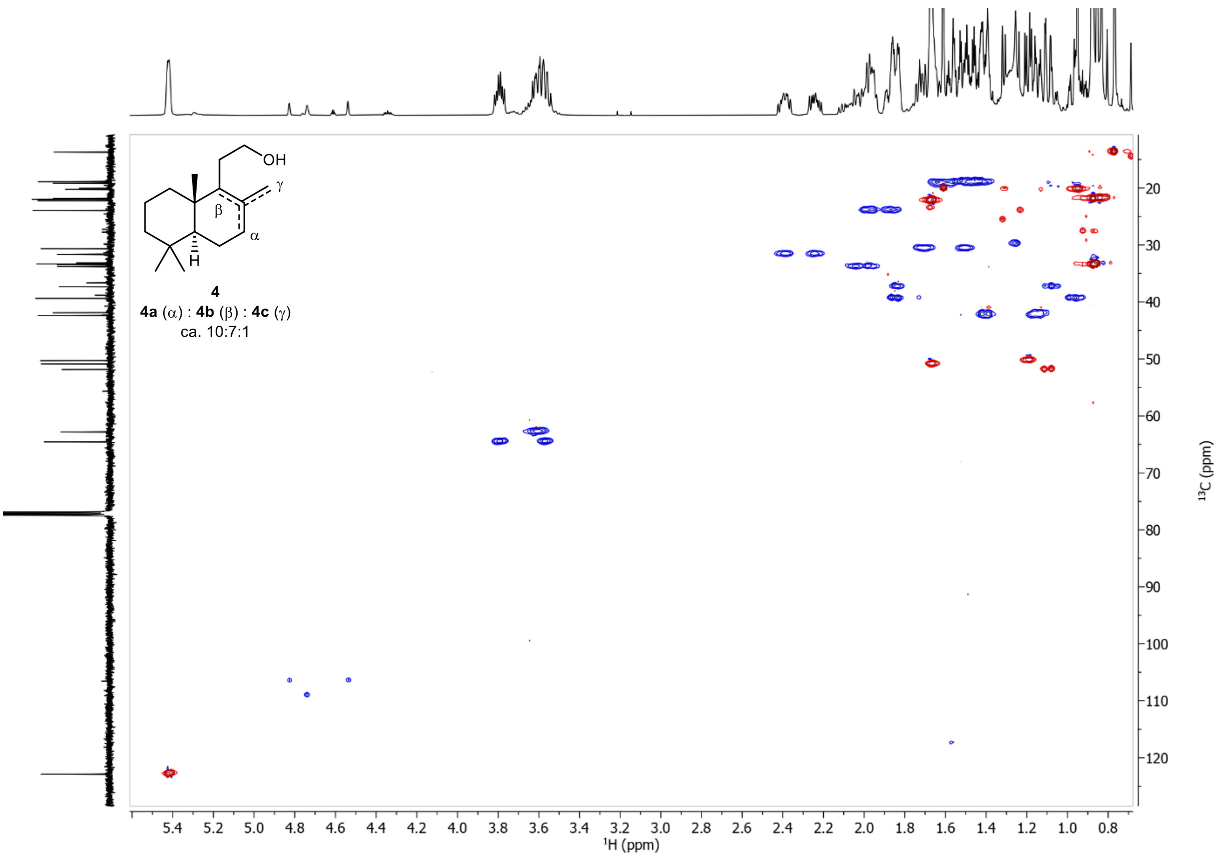

^{1}H,^{13}C-HSQC (501 MHz, 126 MHz, $CDCl_3$) spectrum of homodrimenols **4** obtained in trace quantities as side product in the IDPi-catalyzed polyene cyclization of (3*E*,7*E*)-homofarnesol to (−)-ambrox

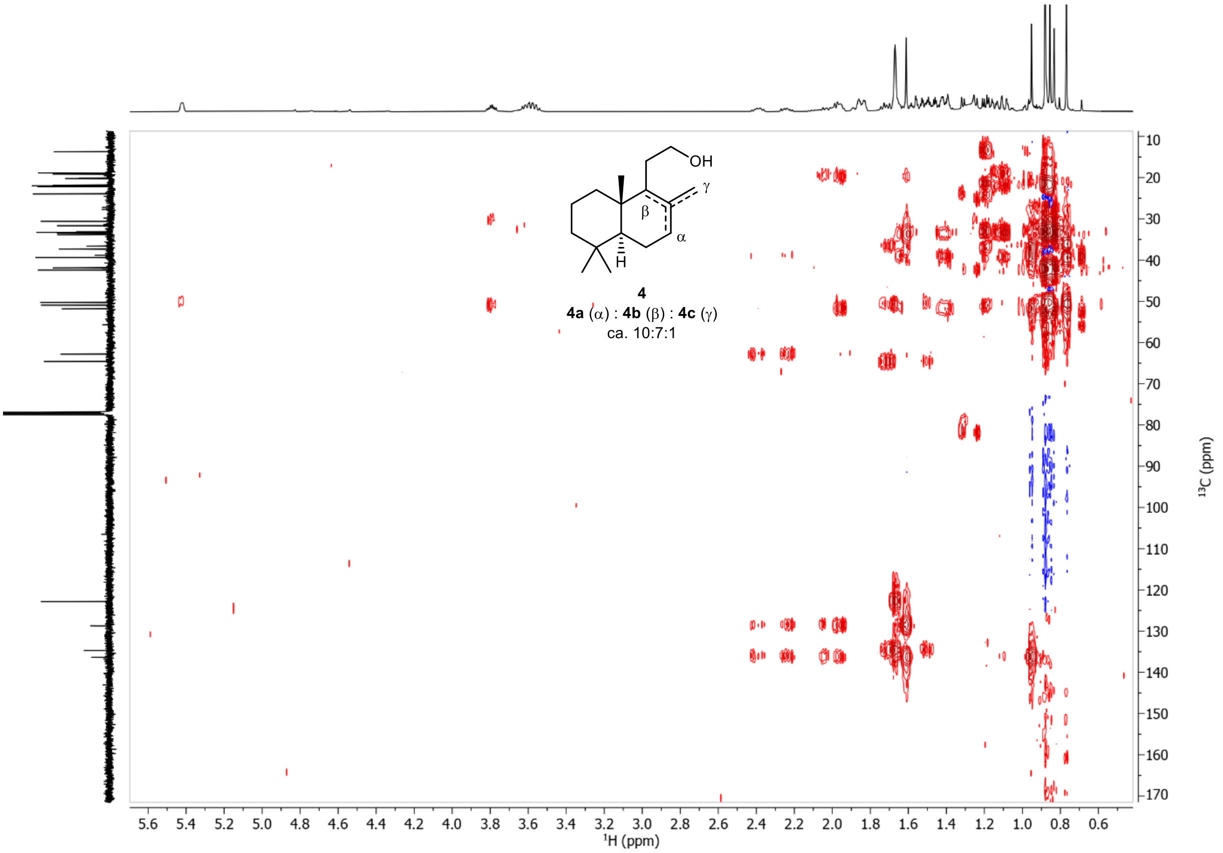

^{1}H,^{13}C-HMBC (501 MHz, 126 MHz, $CDCl_3$) spectrum of homodrimenols **4** obtained in trace amounts as side product in the IDPi-catalyzed polyene cyclization of (3*E*,7*E*)-homofarnesol to (−)-ambrox

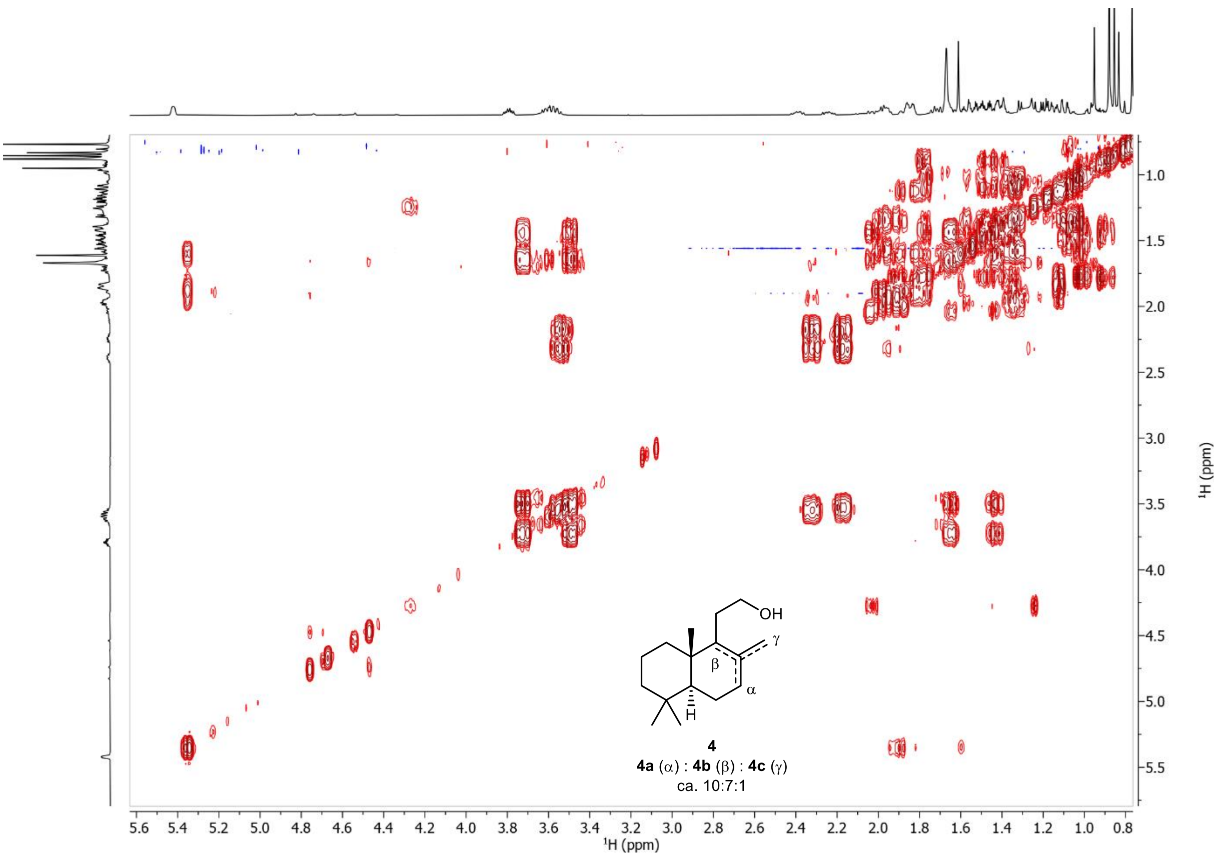

^{1}H,^{1}H-COSY (501 MHz, $CDCl_3$) spectrum of homodrimenols **4** obtained in trace quantities as side product in the IDPi-catalyzed polyene cyclization of (3*E*,7*E*)-homofarnesol to (−)-ambrox

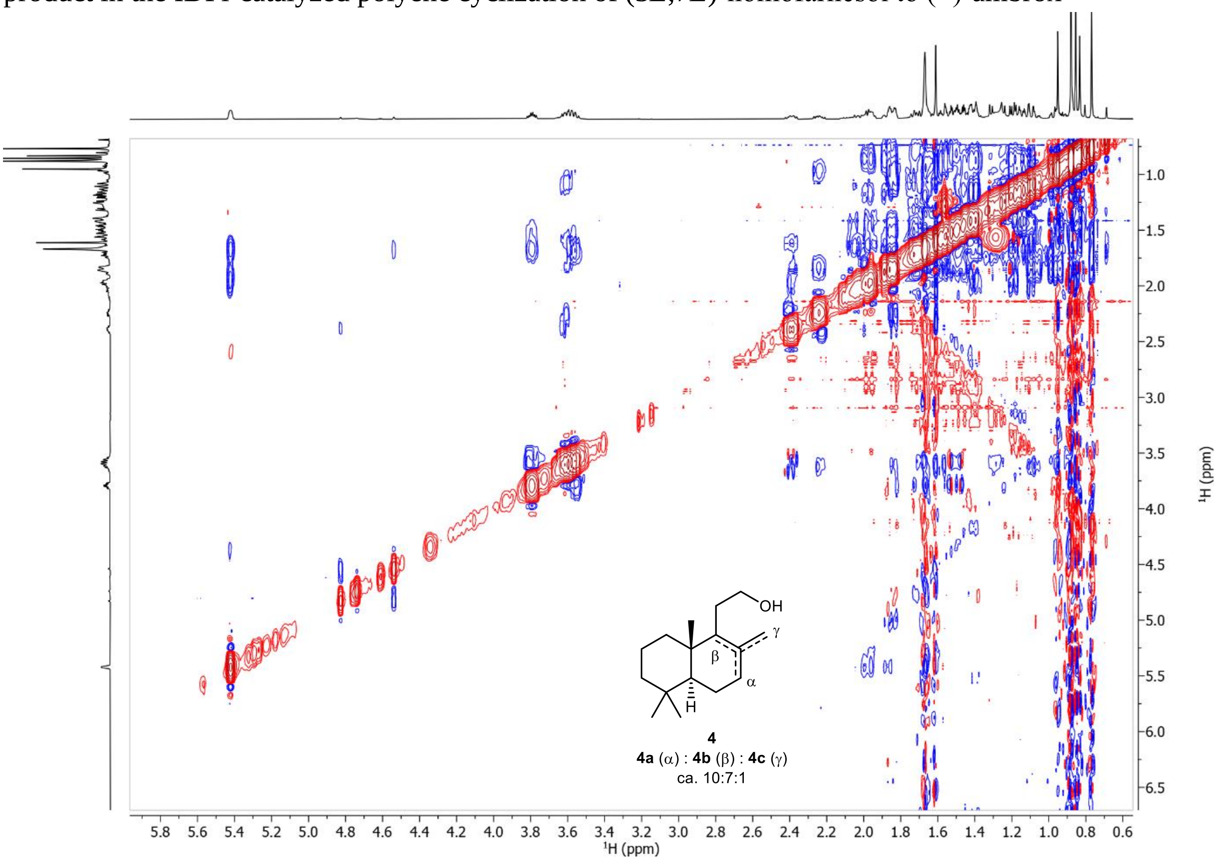

^{1}H,^{1}H-NOESY (501 MHz, $CDCl_3$) spectrum of homodrimenols **4** obtained in trace quantities as side product in the IDPi-catalyzed polyene cyclization of (3*E*,7*E*)-homofarnesol to (−)-ambrox

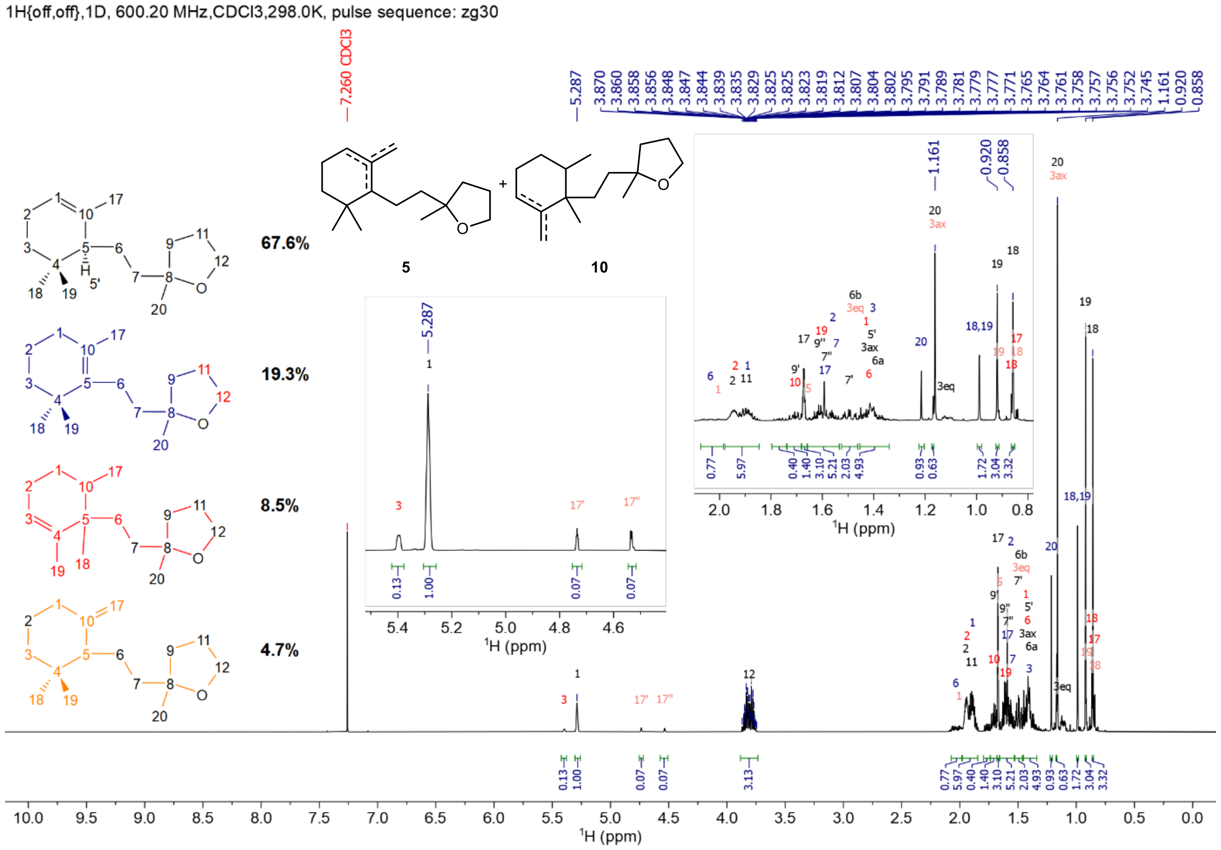

^{1}H NMR (600 MHz, $CDCl_3$) spectrum of partially cyclized products **5** and isomerized product **10** obtained in the IDPi-catalyzed polyene cyclization of (3*E*,7*E*)-homofarnesol to (−)-ambrox.

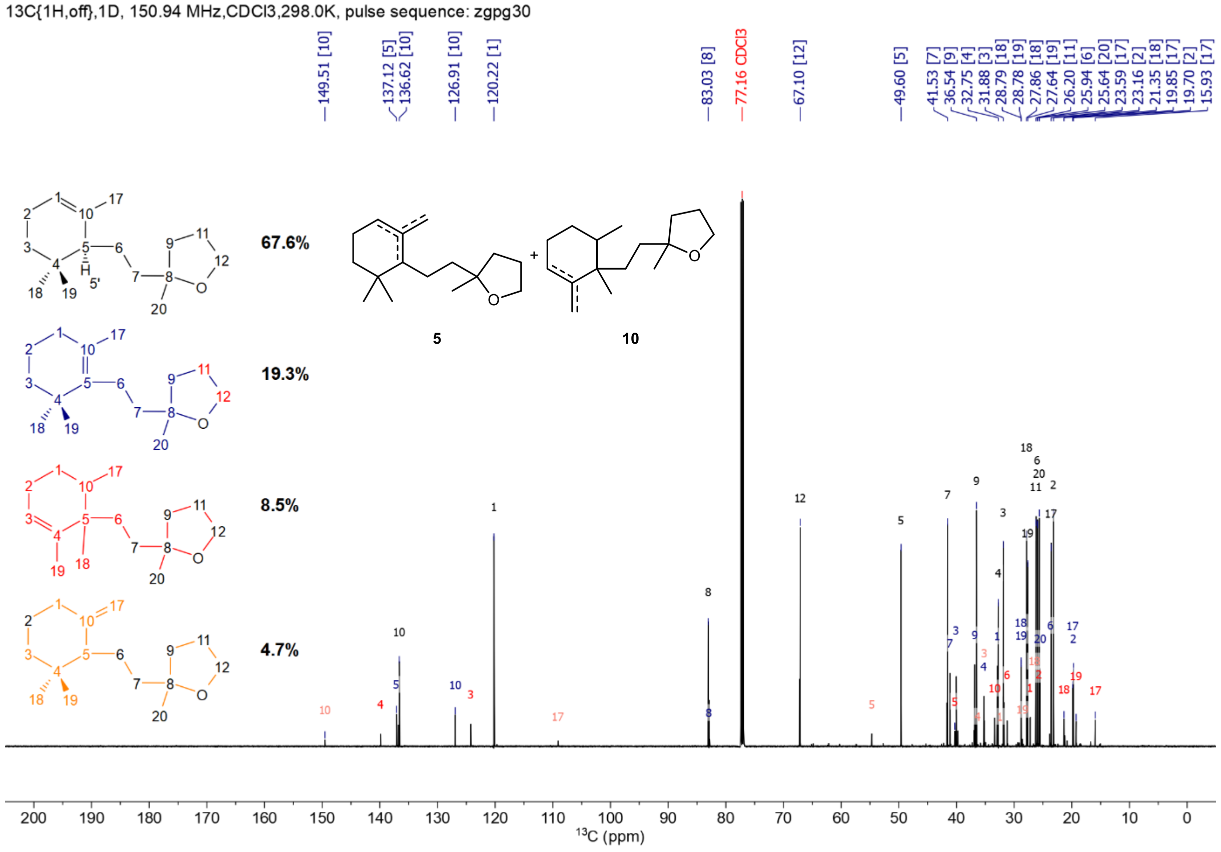

^{13}C NMR (151 MHz, $CDCl_3$) spectrum of partially cyclized products **5** and isomerized product **10** obtained in the IDPi-catalyzed polyene cyclization of (3*E*,7*E*)-homofarnesol to (−)-ambrox.

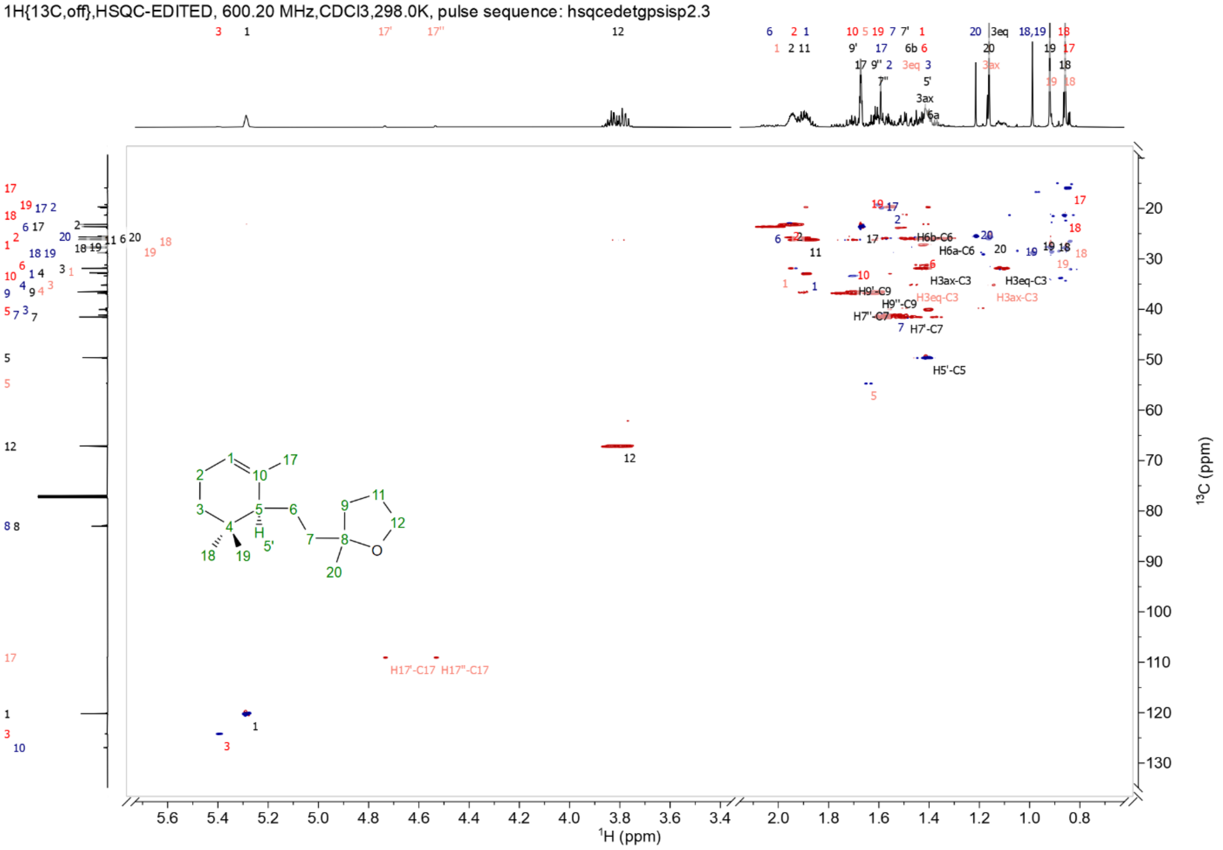

Multiplicity-edited **^{1}H,^{13}C-HSQC** (600 MHz, 151 MHz, $CDCl_3$) spectrum of partially cyclized products **5** and isomerized product **10**.

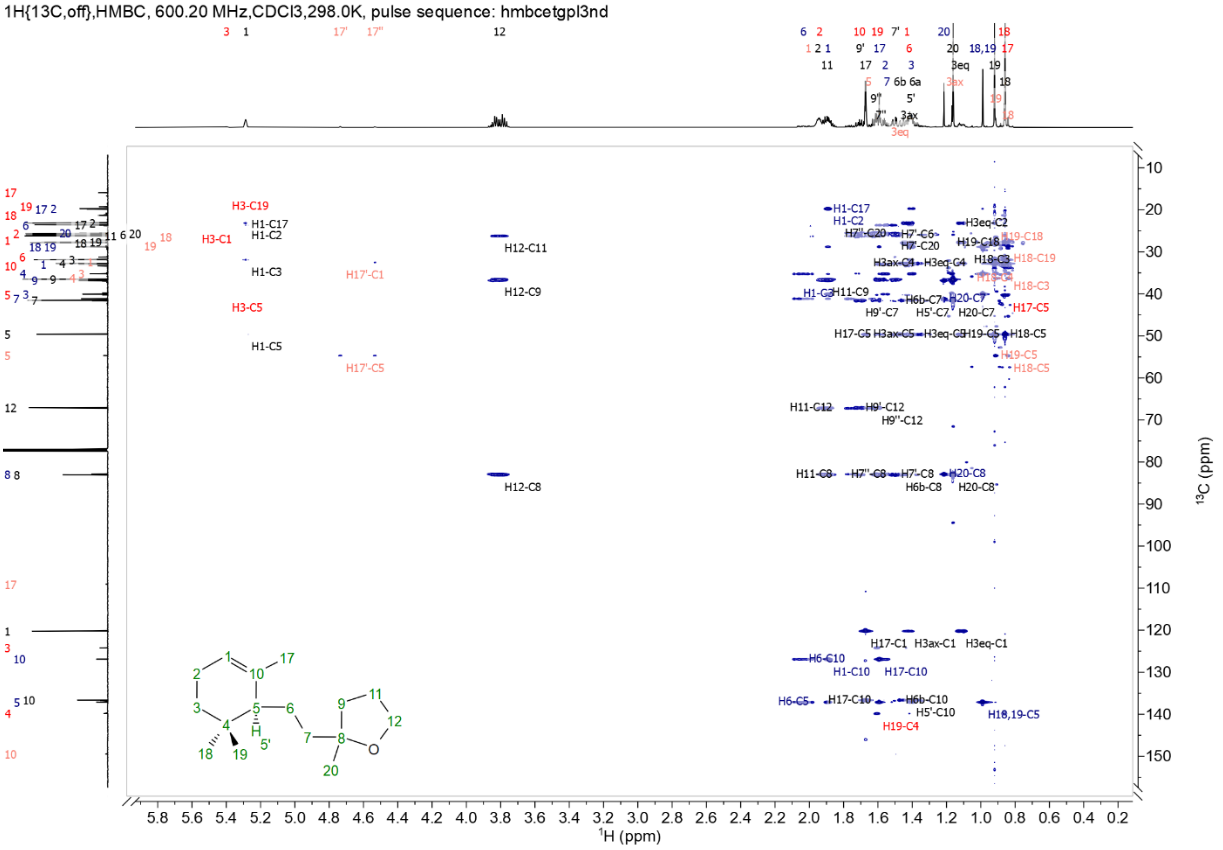

$^{1}H,^{13}C$-HMBC (600 MHz, 151 MHz, $CDCl_3$) spectrum of partially cyclized products **5** and isomerized product **10**.

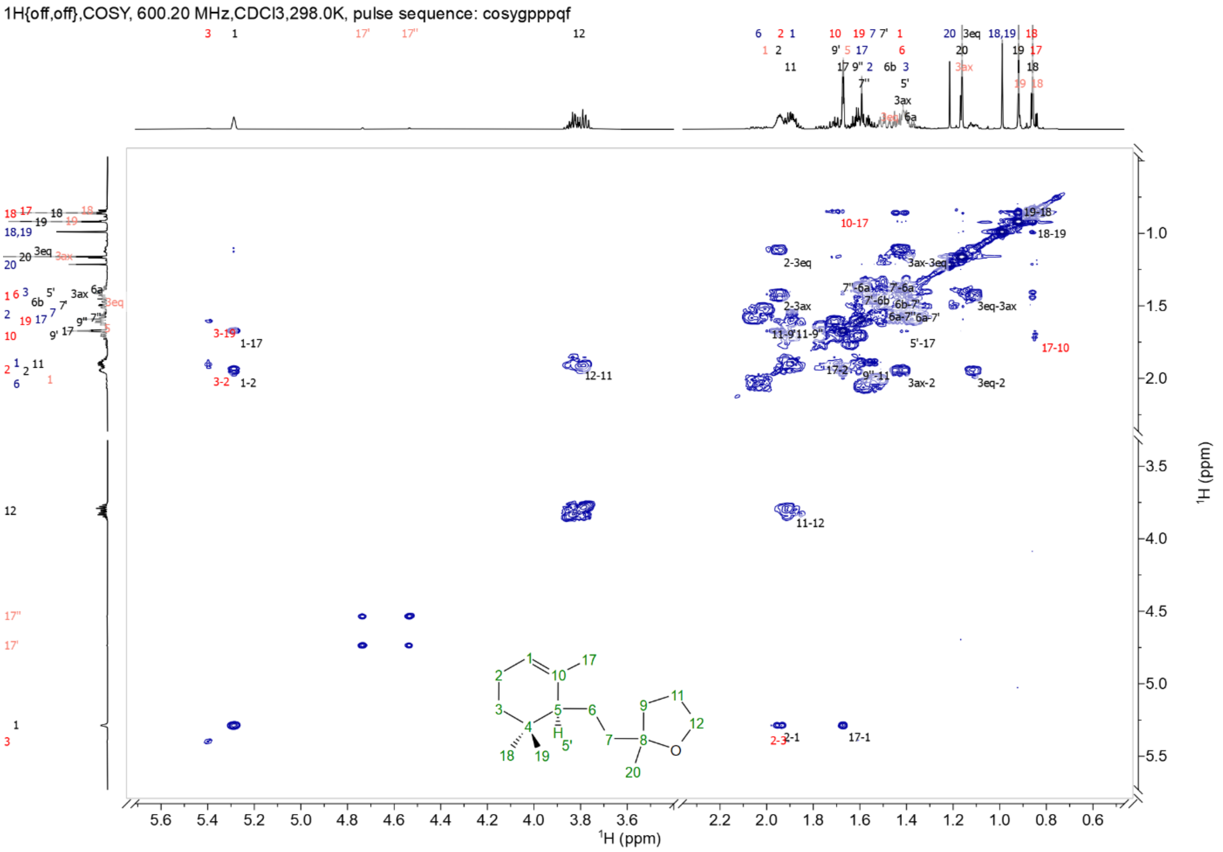

¹H,¹H-COSY (600 MHz, $CDCl_3$) spectrum of partially cyclized products **5** and isomerized product **10**.

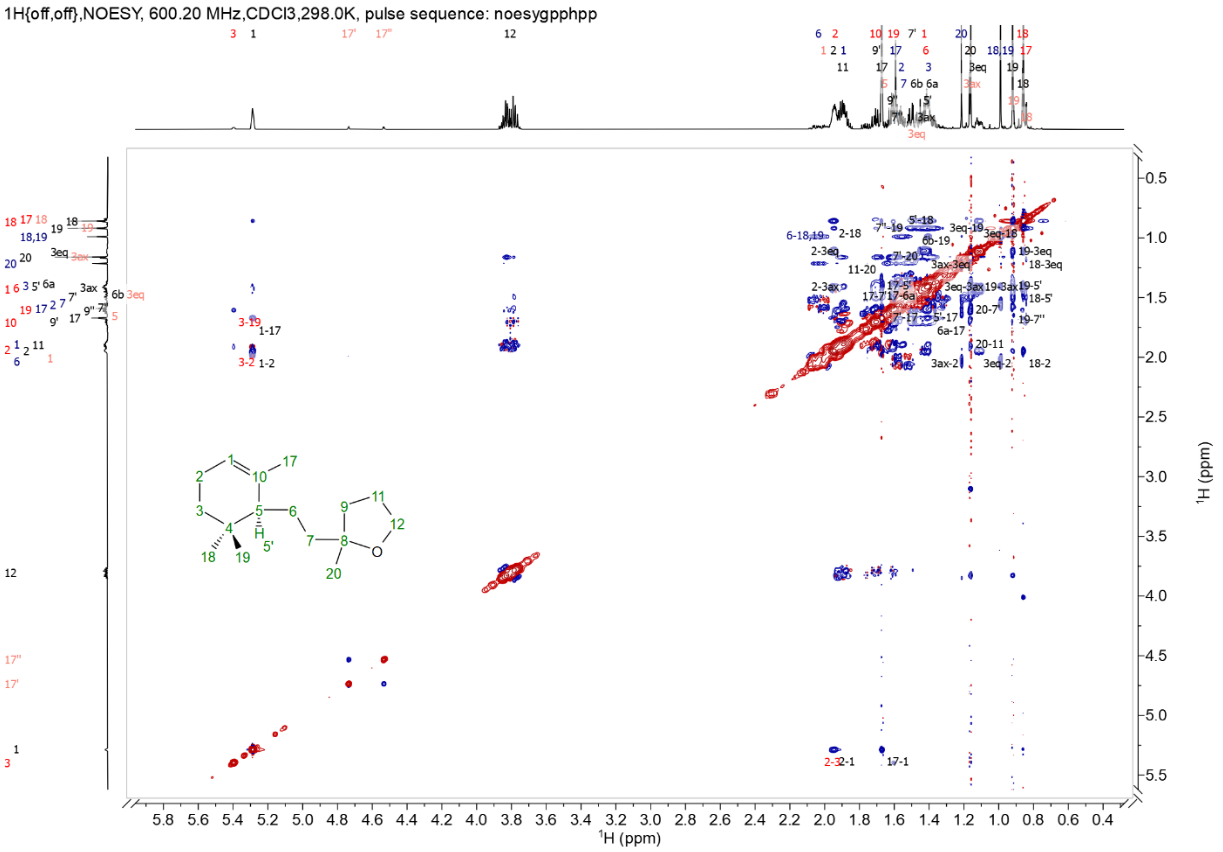

¹H,¹H-NOESY (600 MHz, $CDCl_3$) of partially cyclized products **5** and isomerized product **10**.

1H{off,off},1D, 600.20 MHz,CDCl3,298.0K, pulse sequence: seldigpzs

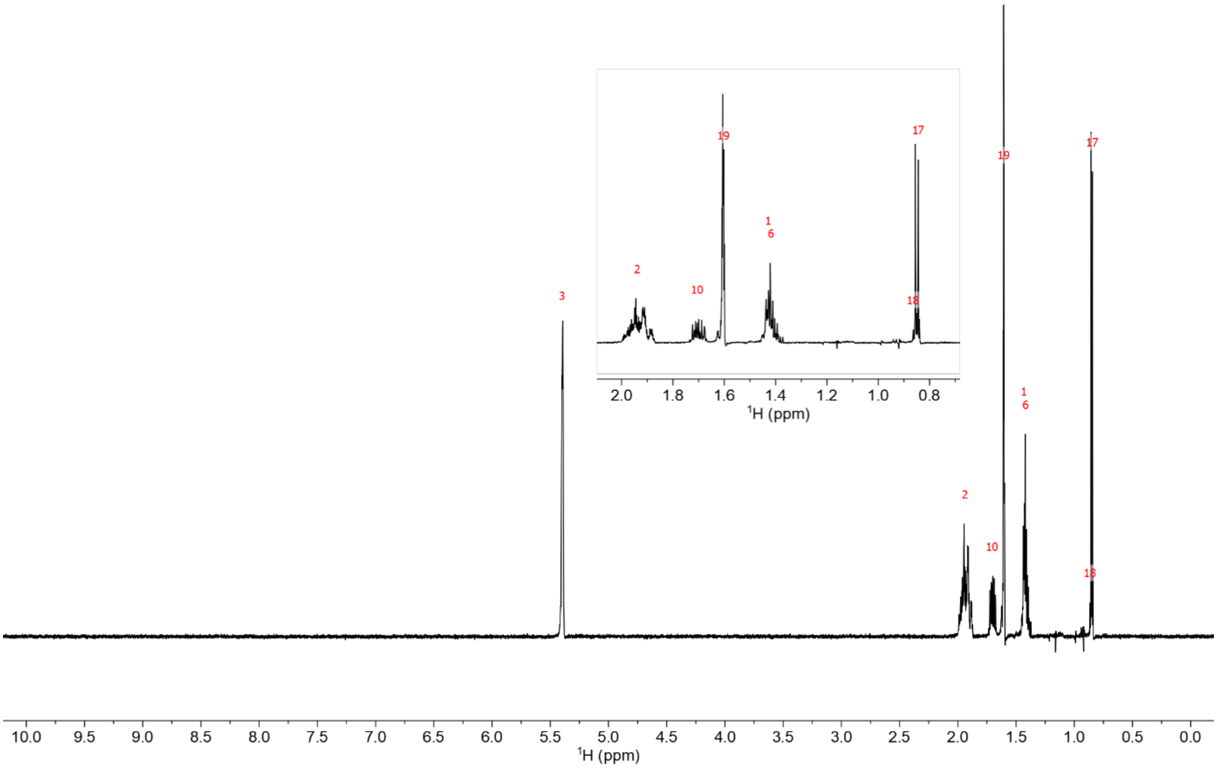

1D TOCSY (600 MHz, $CDCl_3$) spectrum of isomerized product **10** with assignments after irradiation of H3

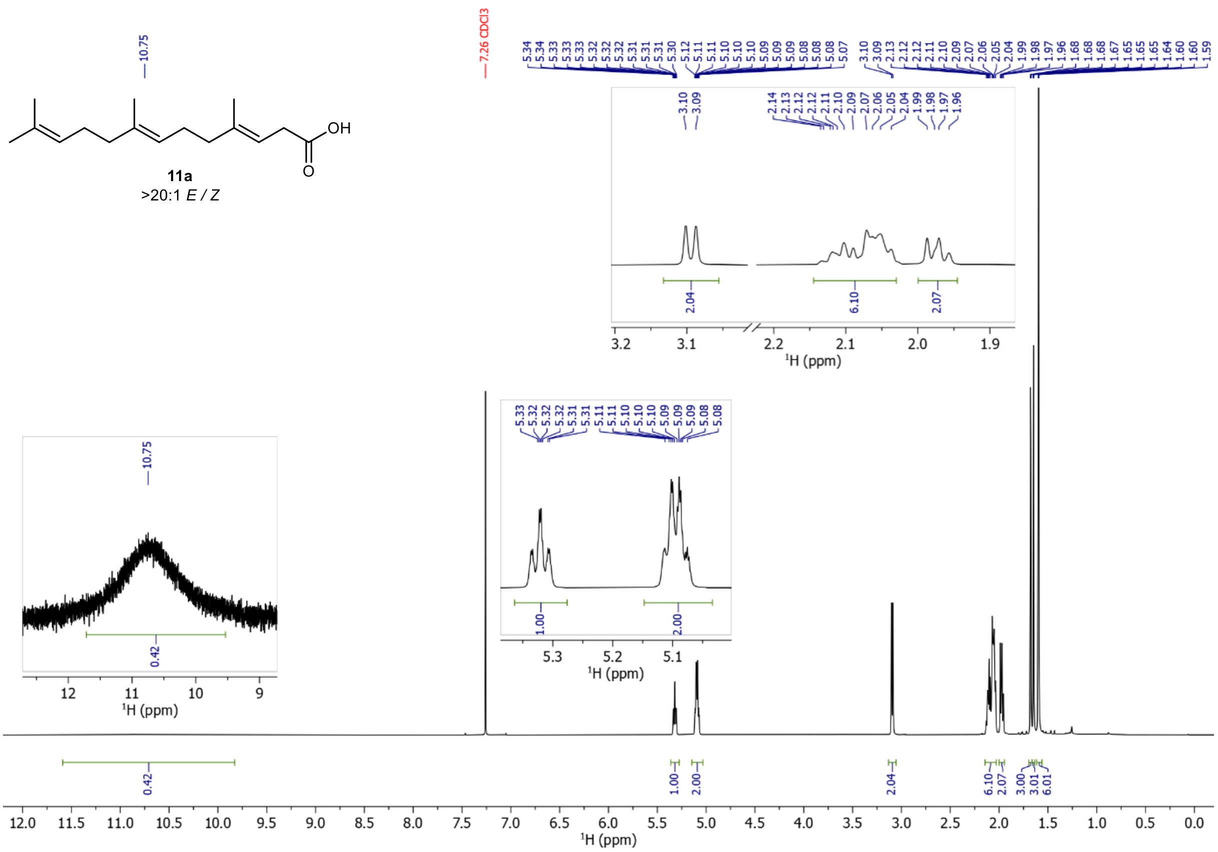

^{1}H NMR (501 MHz, $CDCl_3$) spectrum of (3*E*,7*E*)-homofarnesic acid **11a**

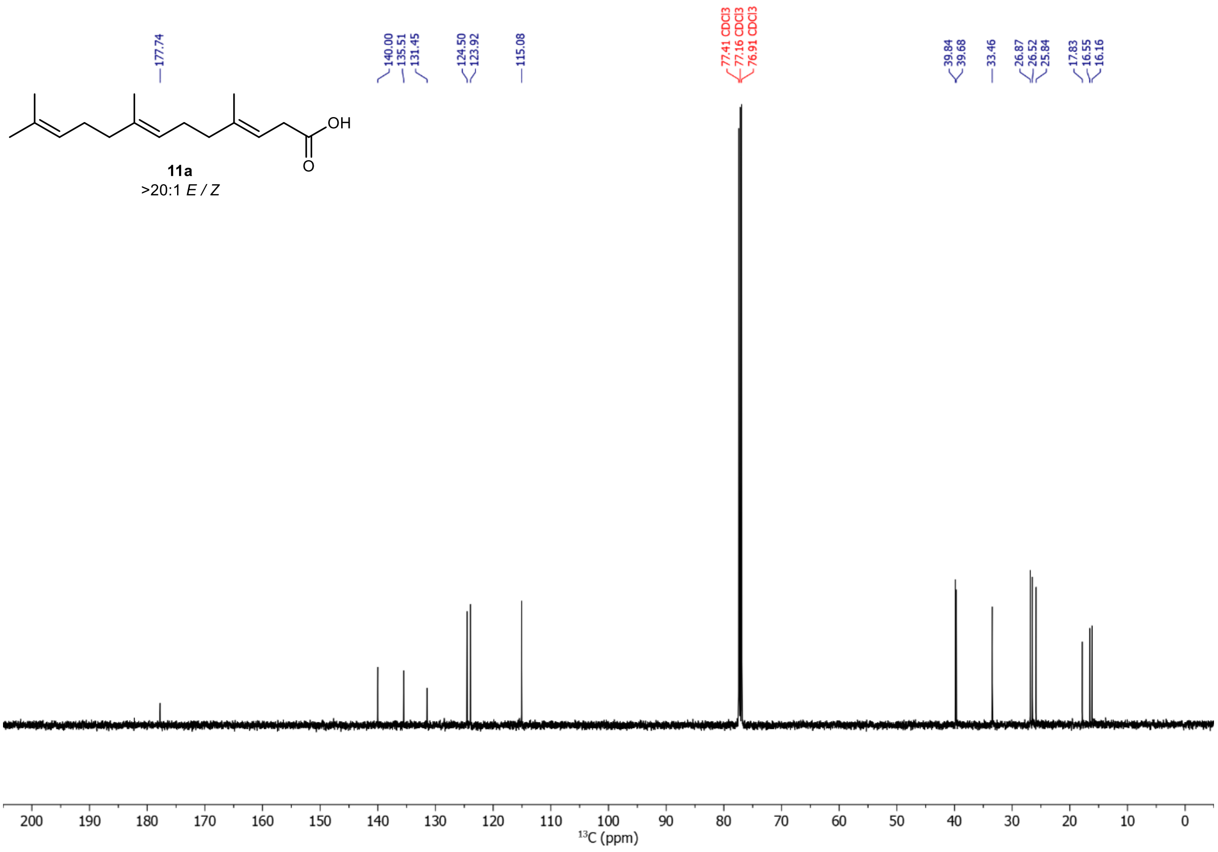

^{13}C NMR (126 MHz, $CDCl_3$) spectrum of (3*E*,7*E*)-homofarnesic acid **11a**

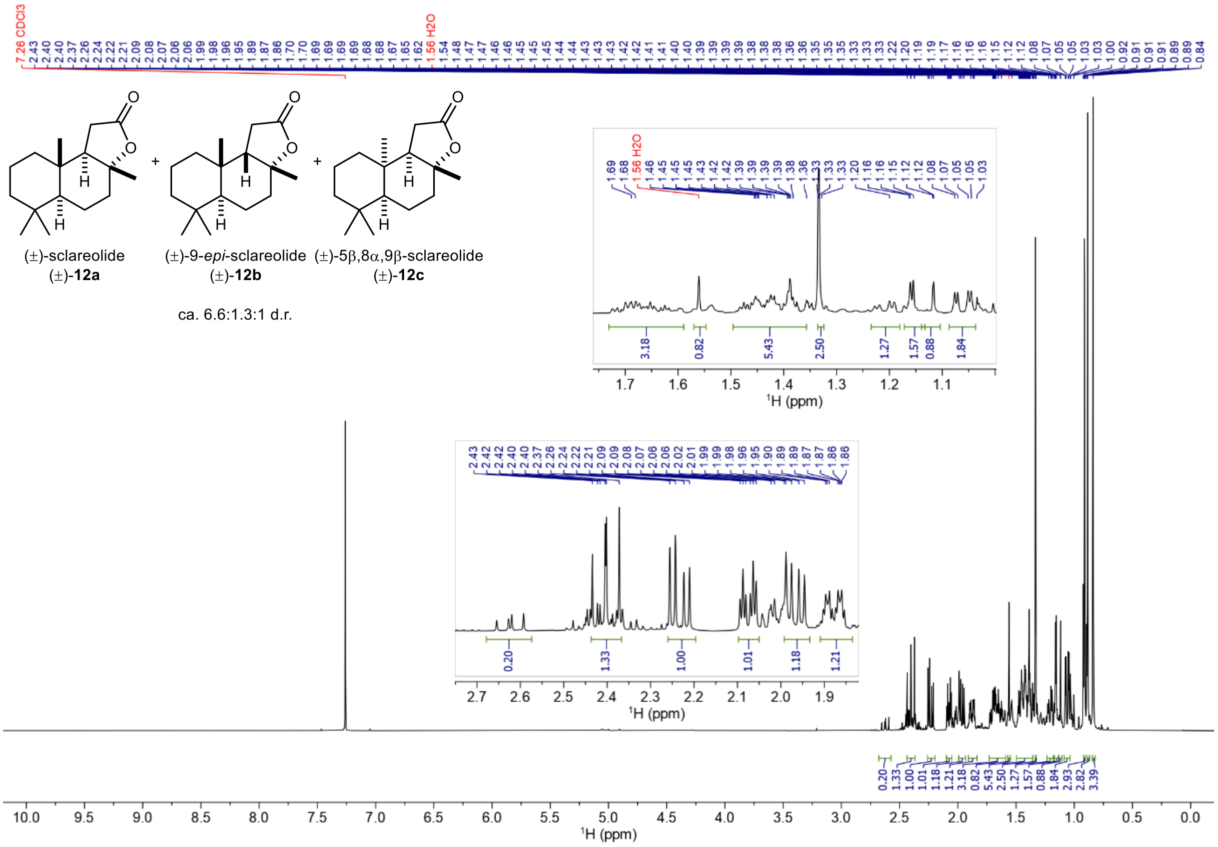

^{1}H NMR (501 MHz, $CDCl_3$) spectrum of a mixture predominantly containing (±)-sclareolide **12a**, (±)-9-*epi*-sclareolide **12b**, and (±)-5β,8α,9β-sclareolide **12c**

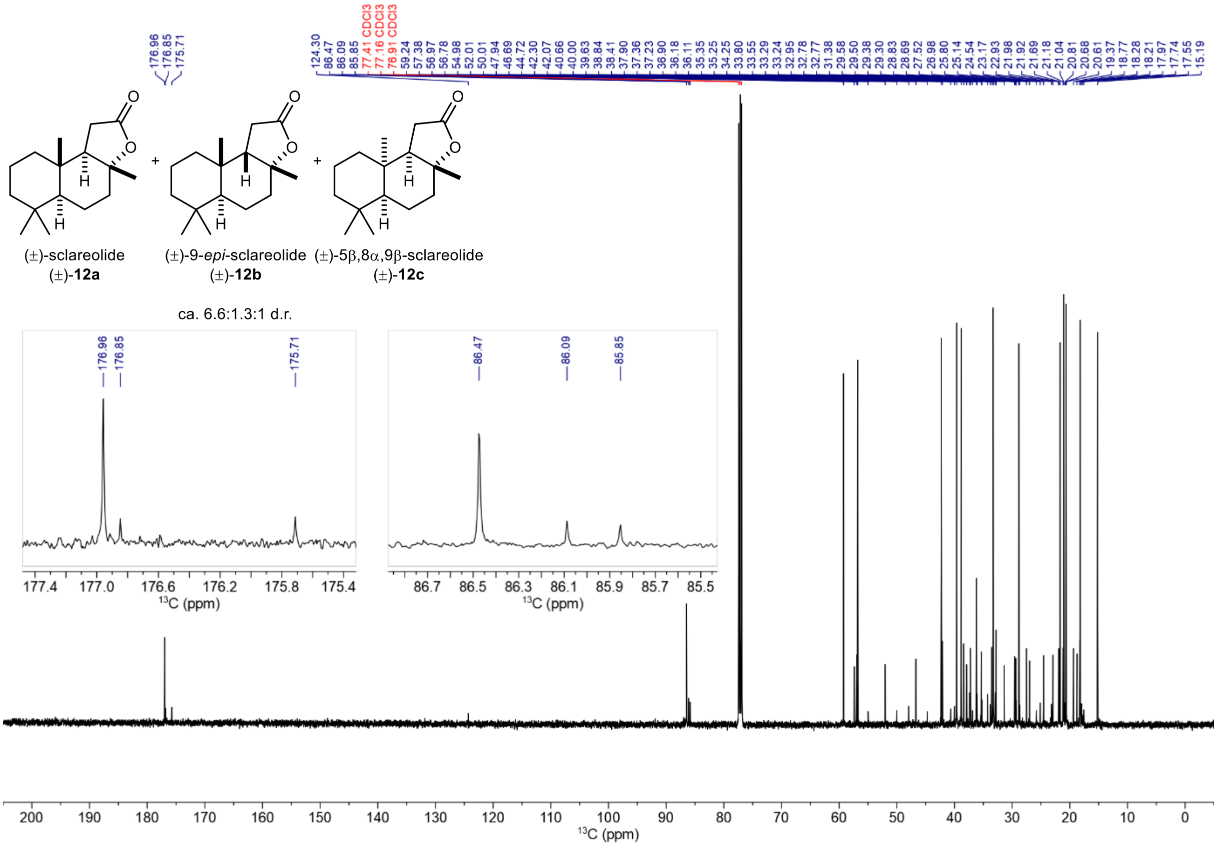

^{13}C NMR (126 MHz, $CDCl_3$) spectrum of a mixture predominantly containing (±)-sclareolide **12a**, (±)-9-*epi*-sclareolide **12b**, and (±)-5β,8α,9β-sclareolide **12c**

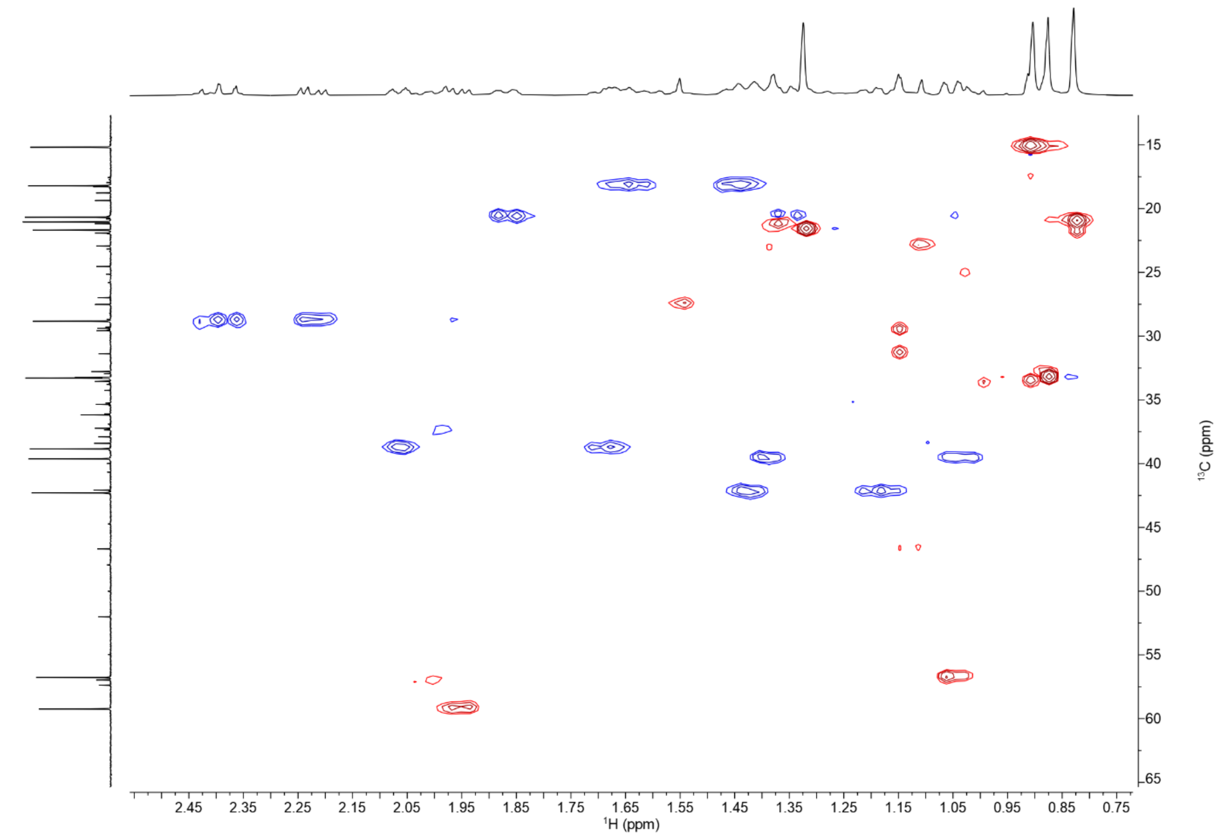

¹H,¹³C-HSQC (501 MHz, 126 MHz, $CDCl_3$) spectrum of a mixture predominantly containing (±)-sclareolide **12a**, (±)-9-*epi*-sclareolide **12b**, and (±)-5β,8α,9β-sclareolide **12c**

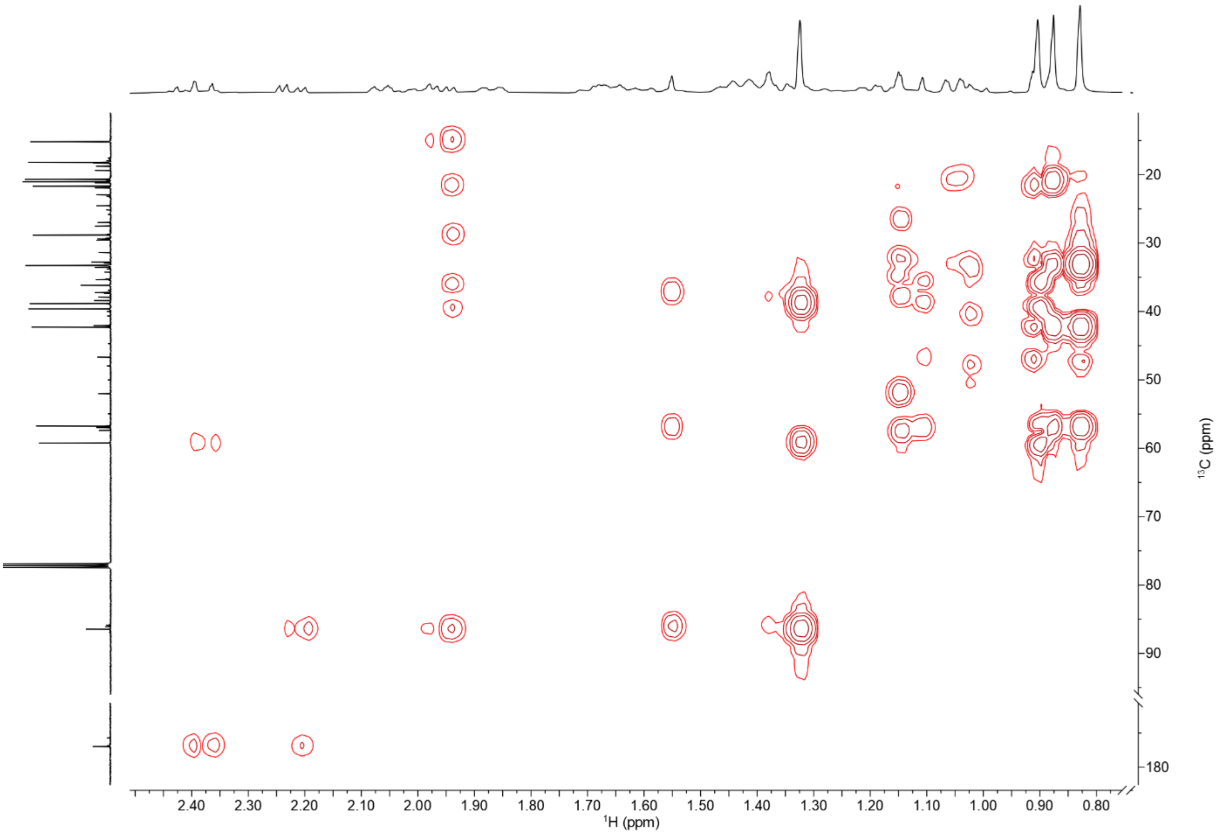

¹H,¹³C-HMBC (501 MHz, 126 MHz, $CDCl_3$) spectrum of a mixture predominantly containing (±)-sclareolide **12a**, (±)-9-*epi*-sclareolide **12b**, and (±)-5β,8α,9β-sclareolide **12c**

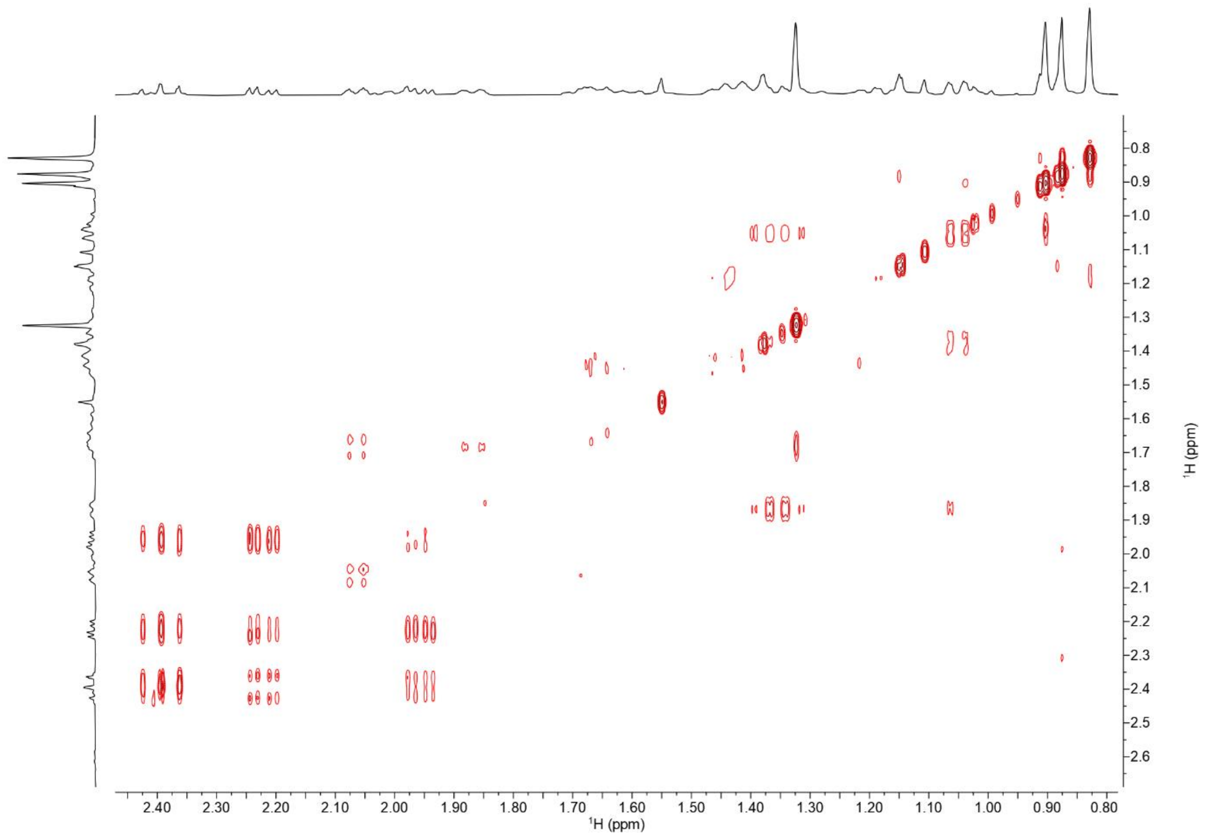

^{1}H,^{1}H-COSY (501 MHz, 126 MHz, $CDCl_3$) spectrum of a mixture predominantly containing (±)-sclareolide **12a**, (±)-9-*epi*-sclareolide **12b**, and (±)-5β,8α,9β-sclareolide **12c**

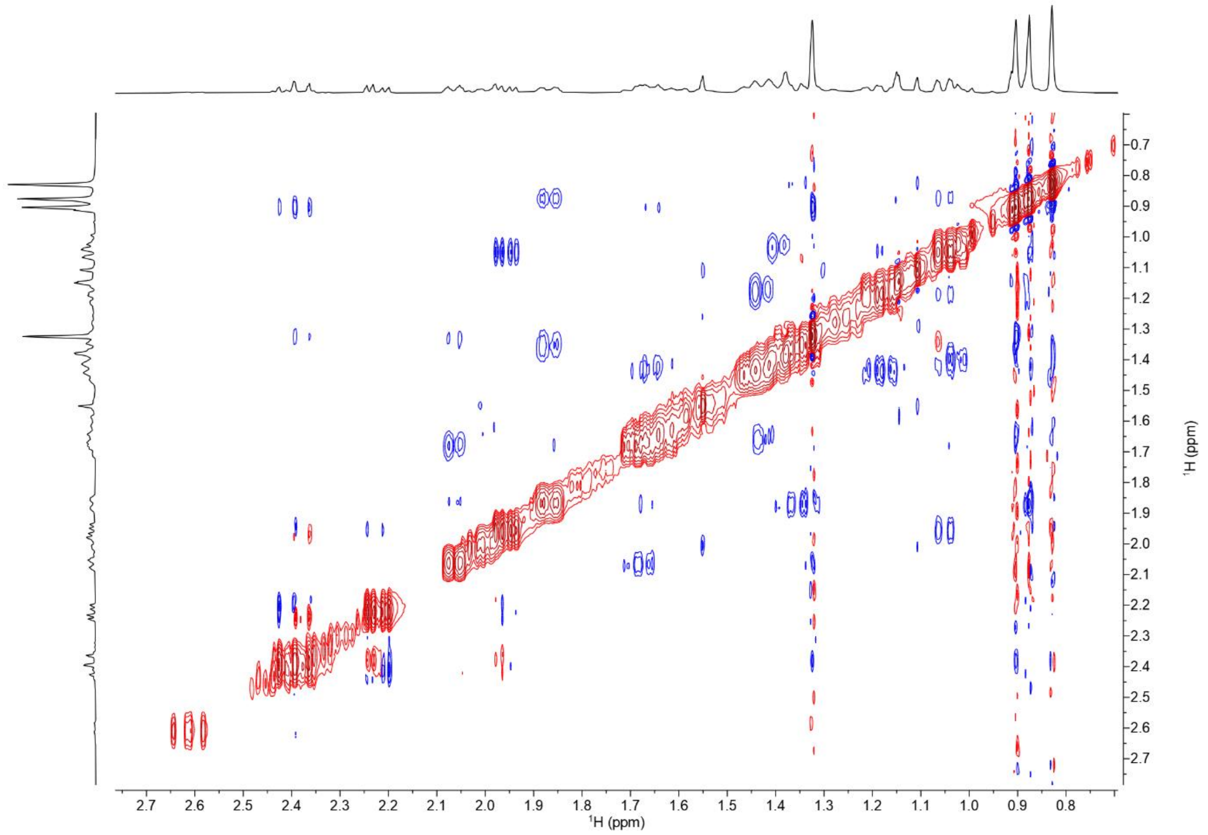

^{1}H,^{1}H-NOESY (501 MHz, 126 MHz, $CDCl_3$) spectrum of a mixture predominantly containing (±)-sclareolide **12a**, (±)-9-*epi*-sclareolide **12b**, and (±)-5β,8α,9β-sclareolide **12c**

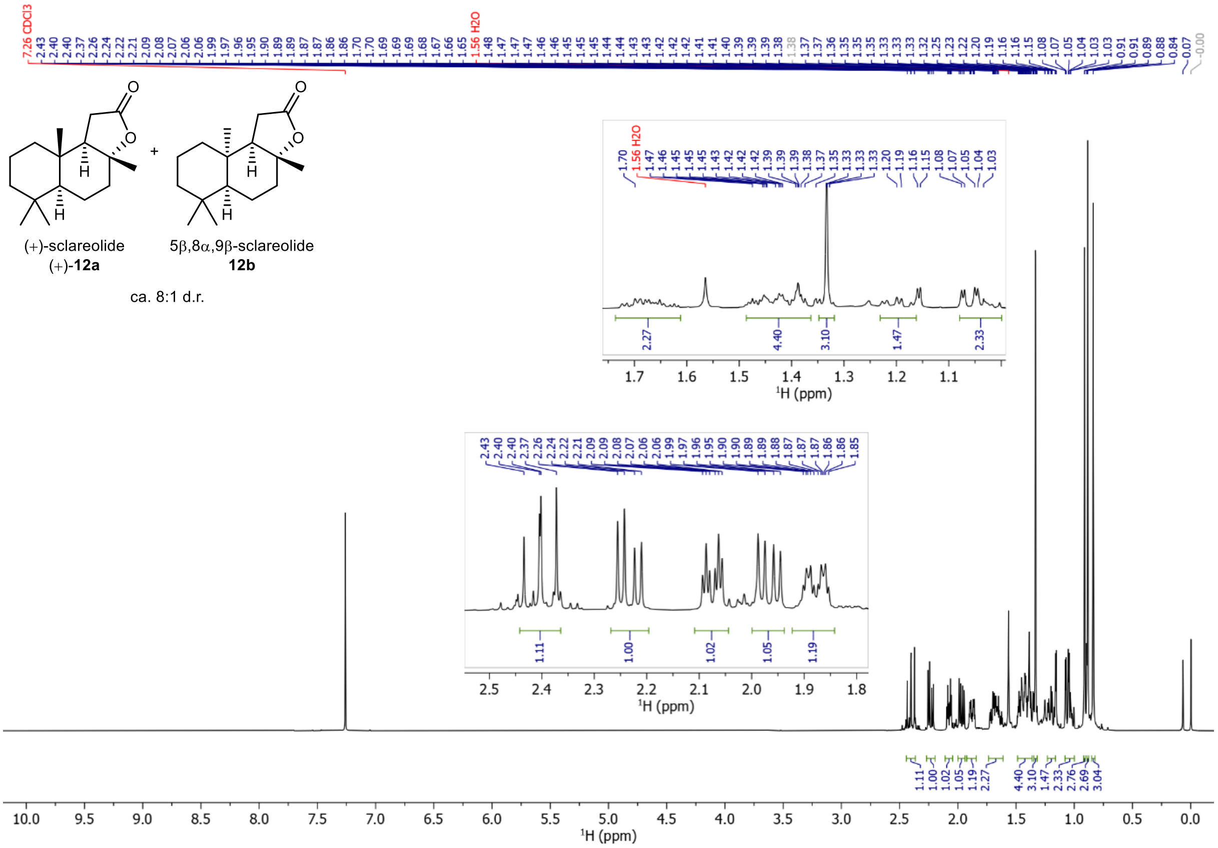

^{1}H NMR (600 MHz, $CDCl_3$) spectrum of a mixture of synthetic (+)-sclareolide **12b** and 5β,8α,9β-sclareolide **12c** obtained in the IDPi-catalyzed polyene cyclization of (3*E*,7*E*)-homofarnesic acid **11a**

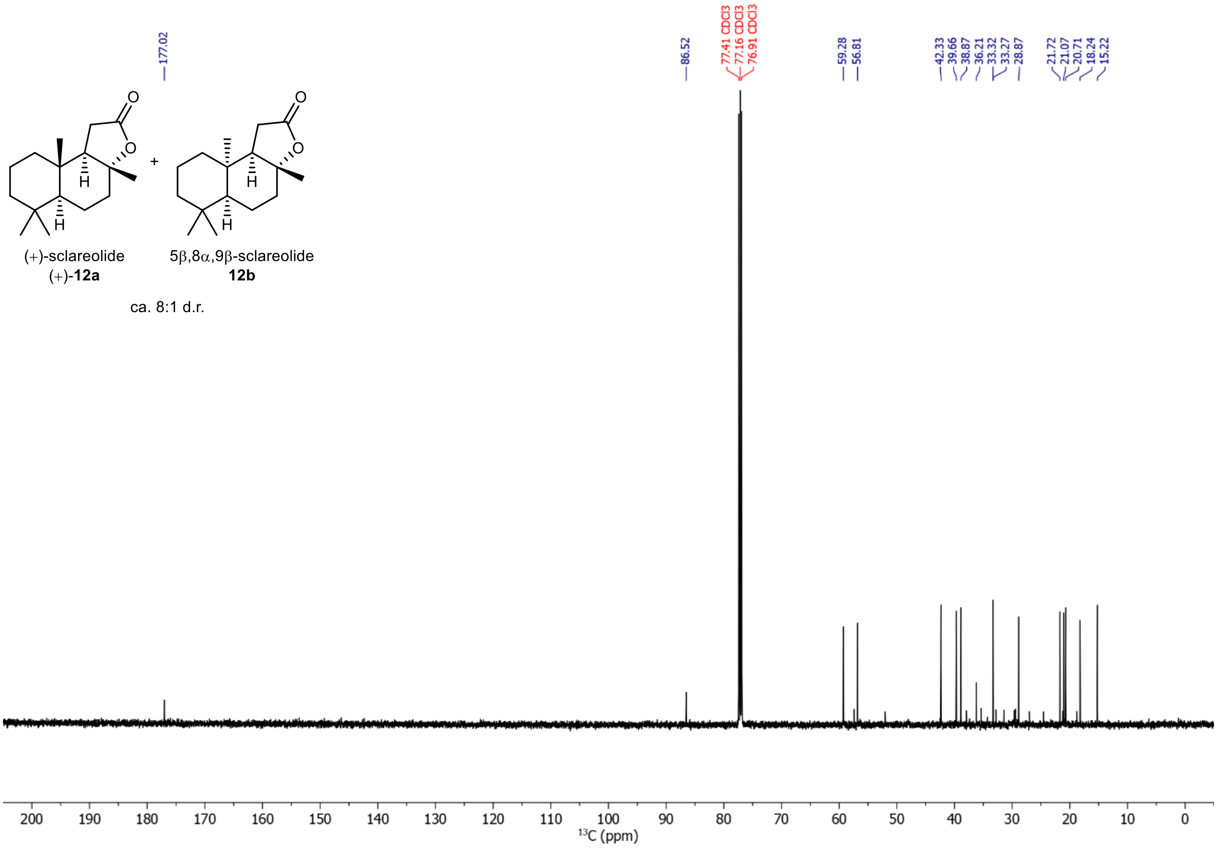

^{13}C NMR (151 MHz, $CDCl_3$) spectrum of a mixture of synthetic (+)-sclareolide **12b** and 5β,8α,9β-sclareolide **12c** obtained in the IDPi-catalyzed polyene cyclization of (3*E*,7*E*)-homofarnesic acid **11a**

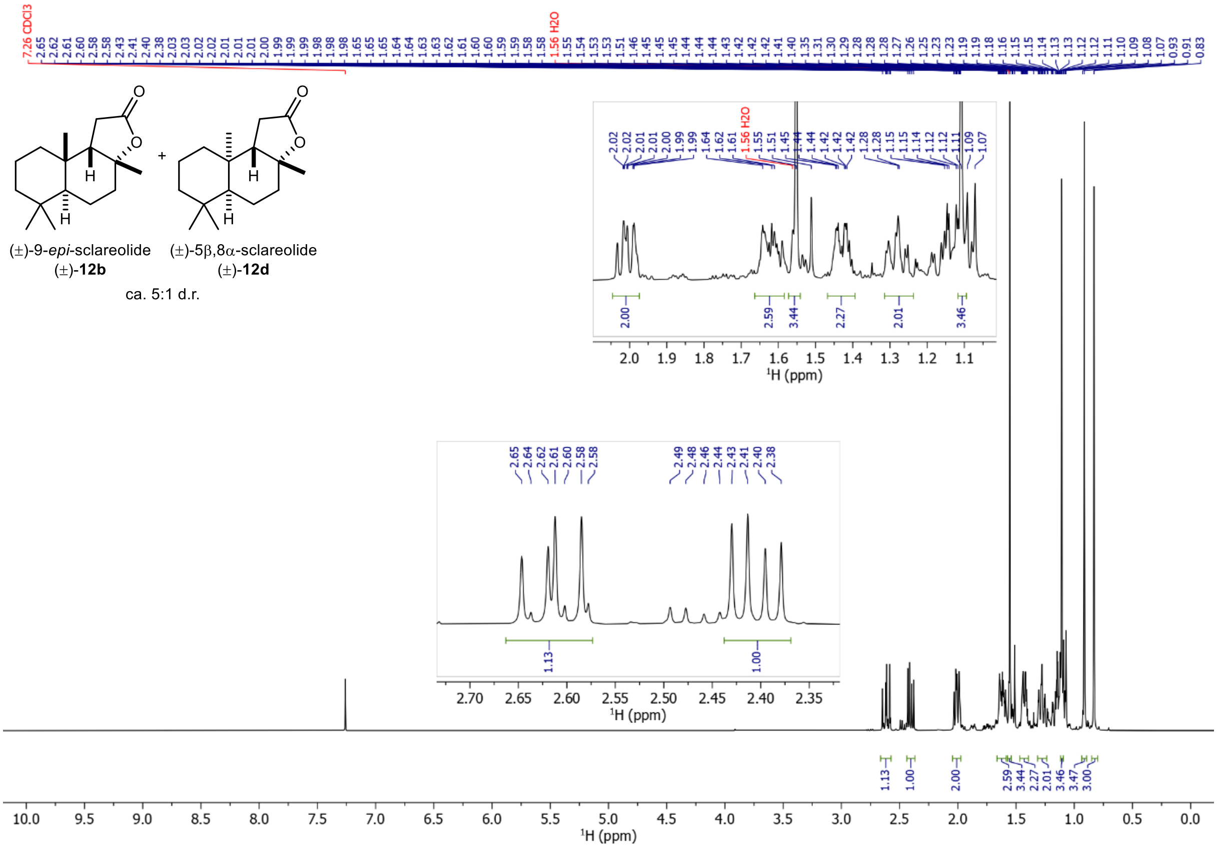

^{1}H NMR (501 MHz, $CDCl_3$) spectrum of a mixture predominantly containing (±)-9-*epi*-sclareolide **12b**, and (±)-5β,8α-sclareolide **12d**

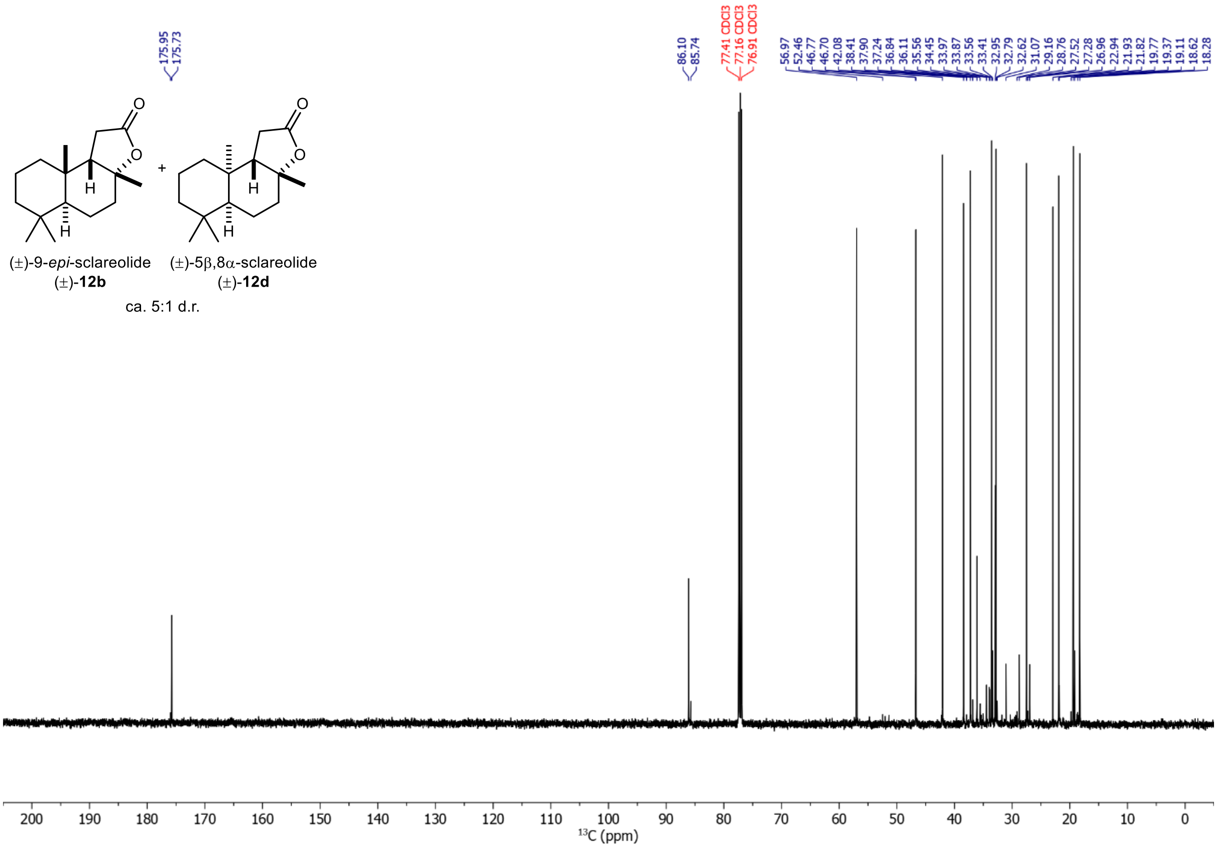

^{13}C NMR (126 MHz, $CDCl_3$) spectrum of a mixture predominantly containing (±)-9-*epi*-sclareolide **12b**, and (±)-5β,8α-sclareolide **12d**

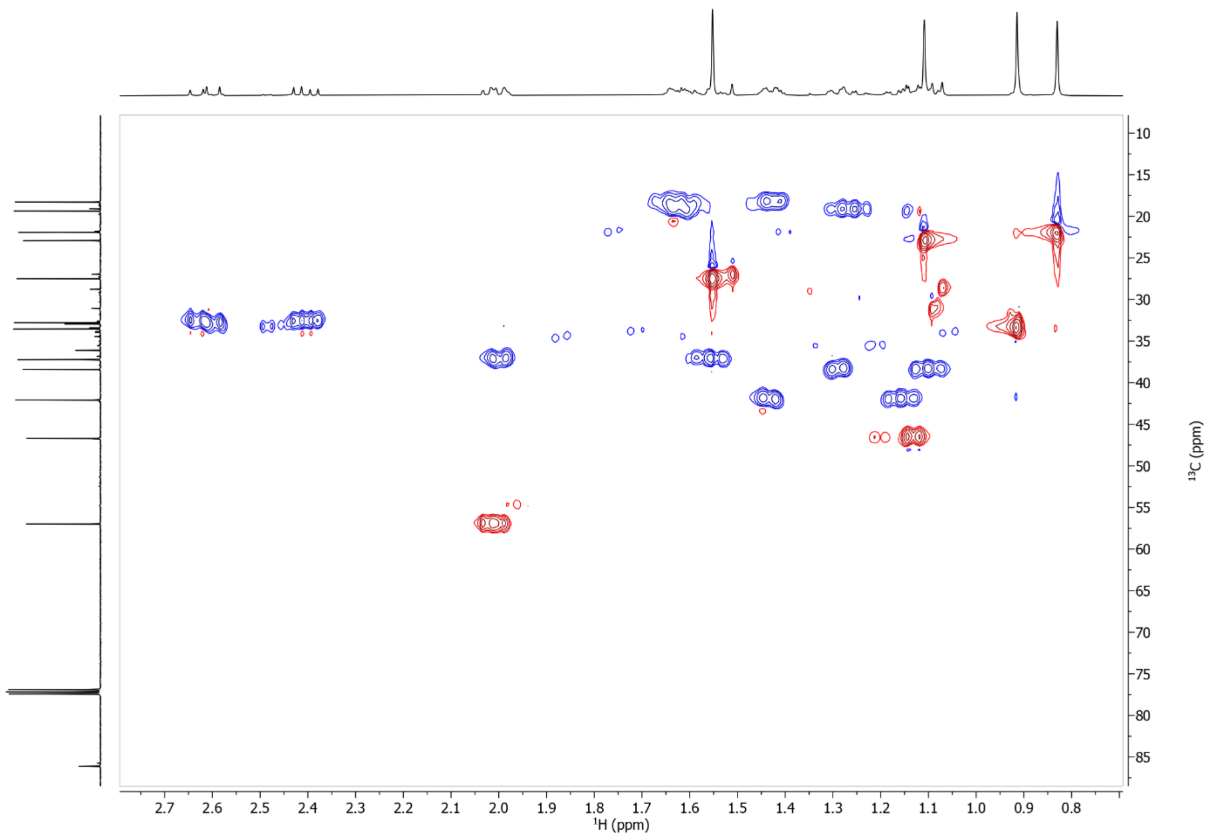

^{1}H,^{13}C-HSQC (501 MHz, 126 MHz, $CDCl_3$) spectrum of a mixture predominantly containing (±)-9-*epi*-sclareolide **12b**, and (±)-5β,8α-sclareolide **12d**

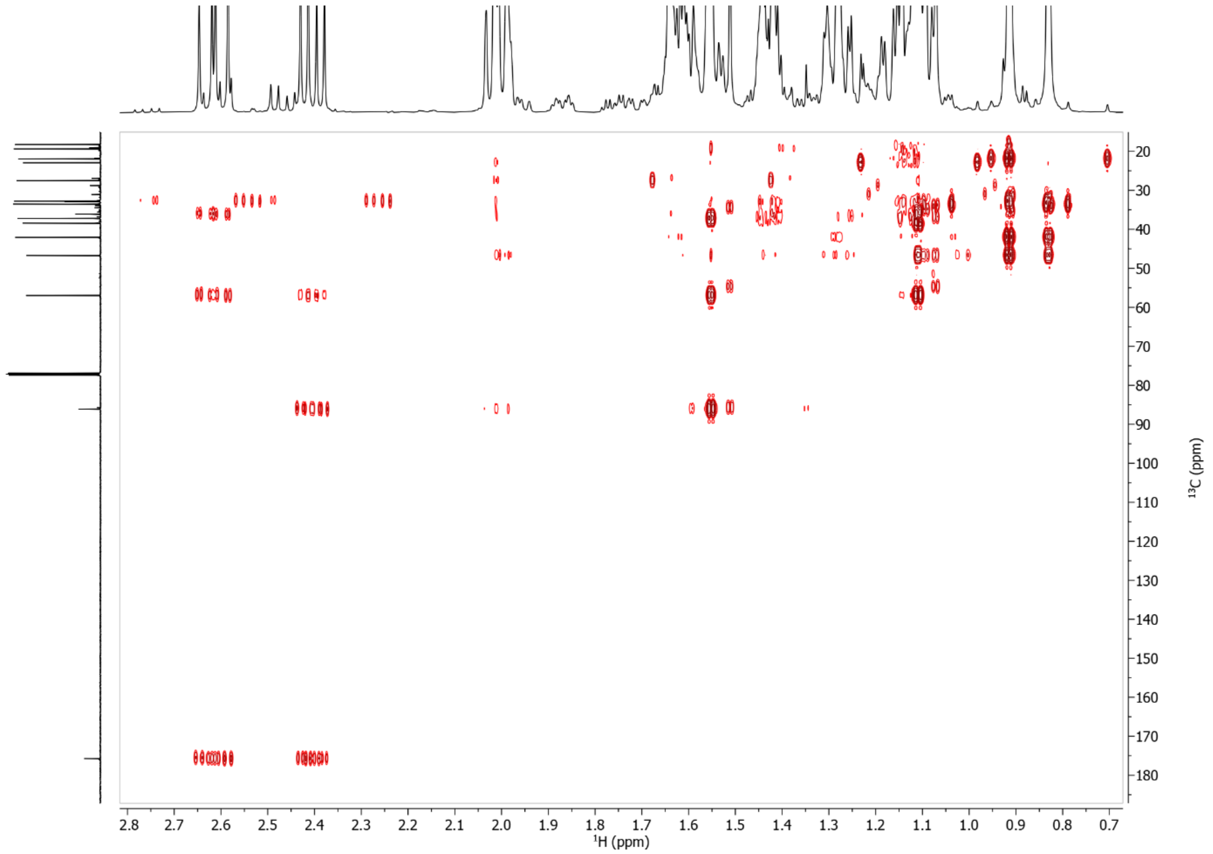

^{1}H,^{13}C-HMBC (501 MHz, 126 MHz, $CDCl_3$) spectrum of a mixture predominantly containing (±)-9-*epi*-sclareolide **12b**, and (±)-5β,8α-sclareolide **12d**

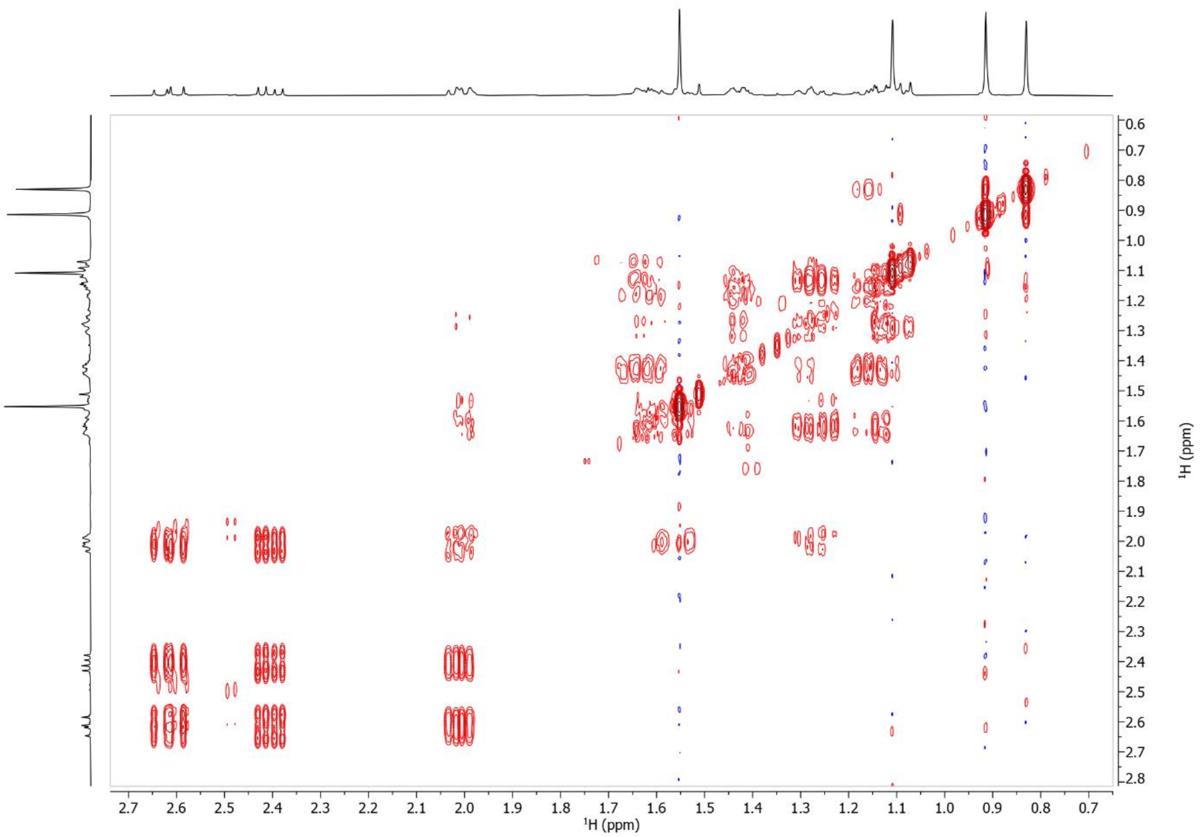

^{1}H,^{1}H-COSY (501 MHz, $CDCl_3$) spectrum of a mixture predominantly containing (±)-9-*epi*-sclareolide **12b**, and (±)-5β,8α-sclareolide **12d**

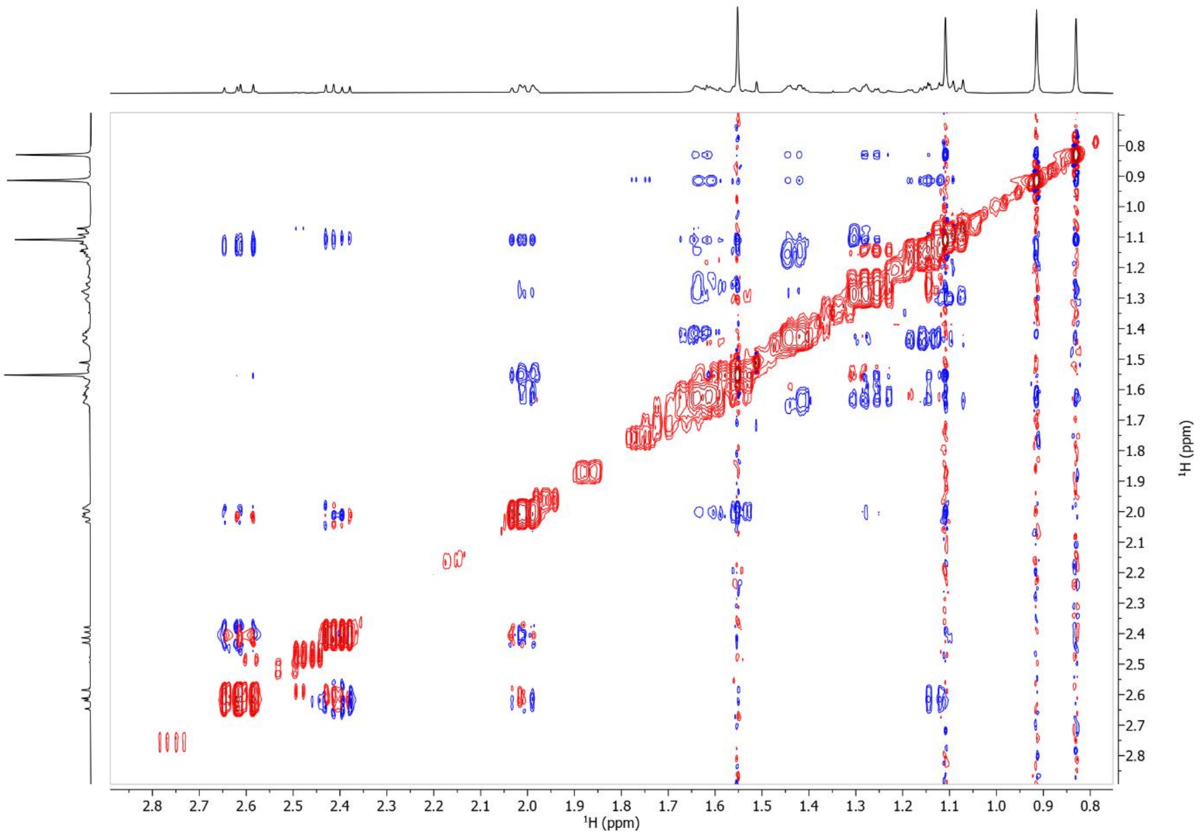

^{1}H,^{1}H-NOESY (501 MHz, $CDCl_3$) spectrum of a mixture predominantly containing (±)-9-*epi*-sclareolide **12b**, and (±)-5β,8α-sclareolide **12d**

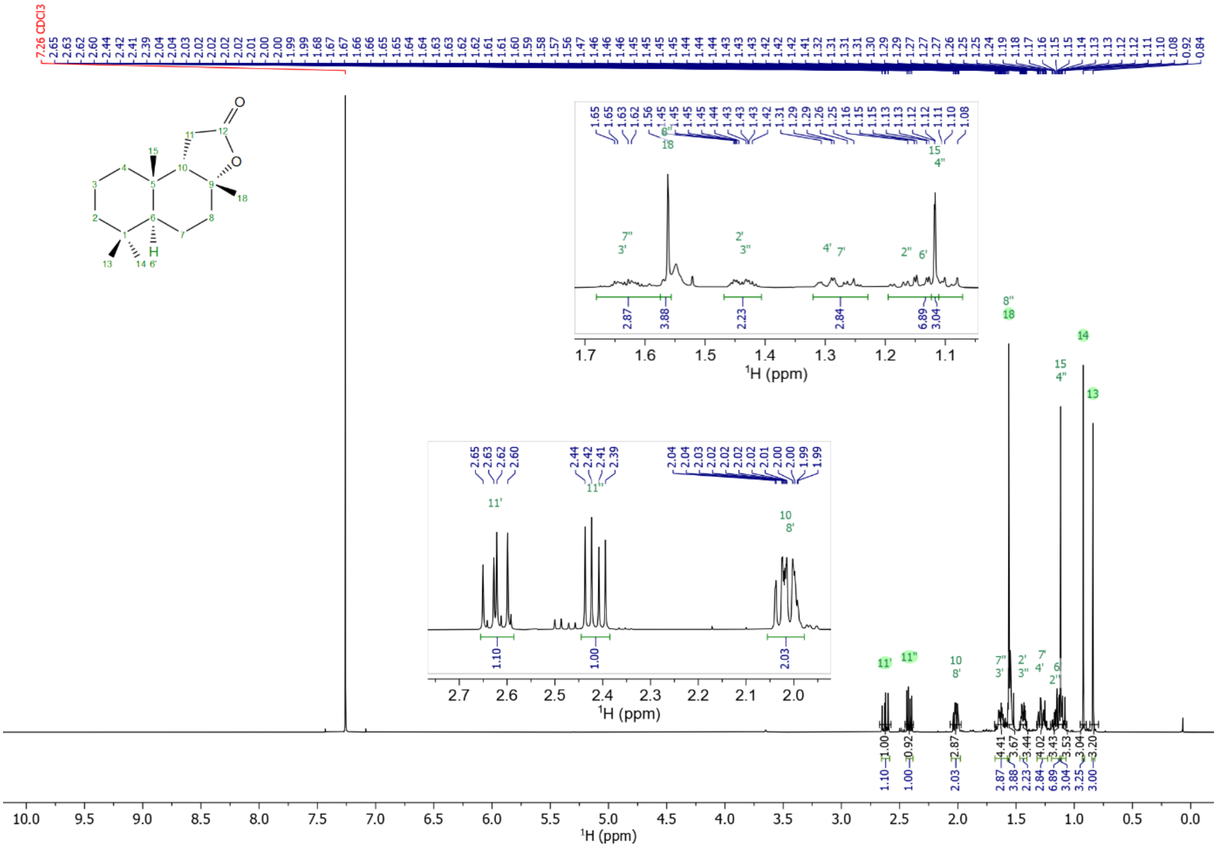

^{1}H NMR (600 MHz, $CDCl_3$) spectrum of a mixture predominantly containing (±)-9-*epi*-sclareolide **12b**, and (±)-5β,8α-sclareolide **12d** with assignments for **12b**

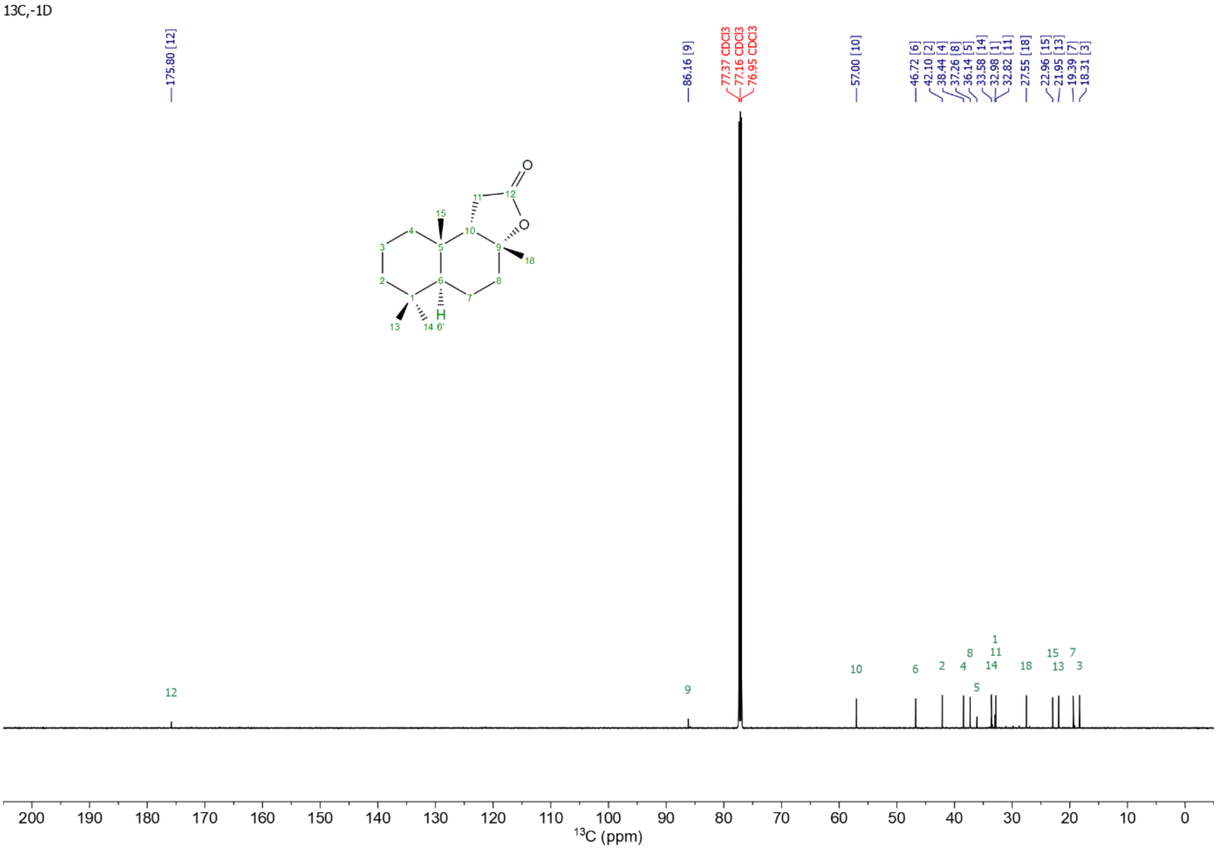

^{13}C NMR (151 MHz, $CDCl_3$) spectrum of a mixture predominantly containing (±)-9-*epi*-sclareolide **12b**, and (±)-5β,8α-sclareolide **12d** with assignments for **12b**

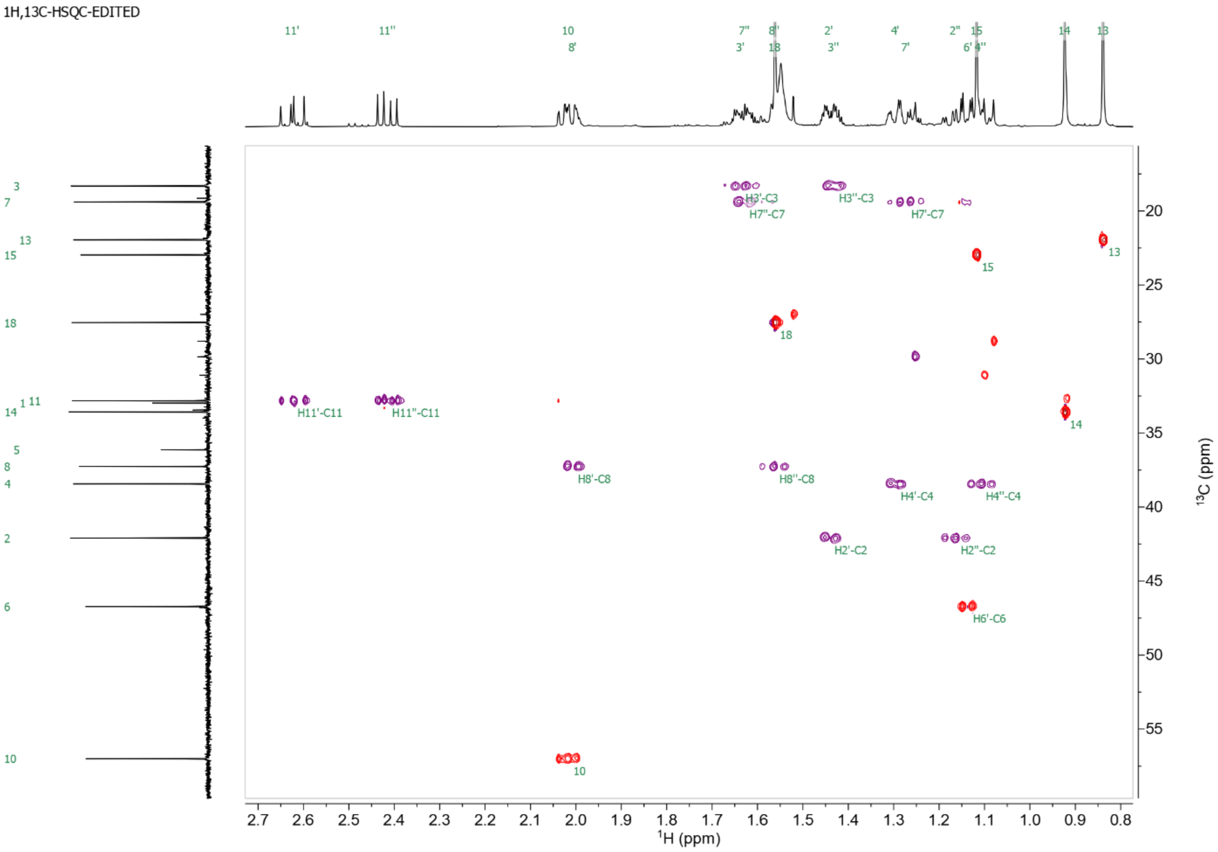

Multiplicity-edited **¹H,¹³C-HSQC** (600 MHz, 151 MHz, $CDCl_3$) spectrum of a mixture predominantly containing (±)-9-*epi*-sclareolide **12b**, and (±)-5β,8α-sclareolide **12d** with assignments for **12b**

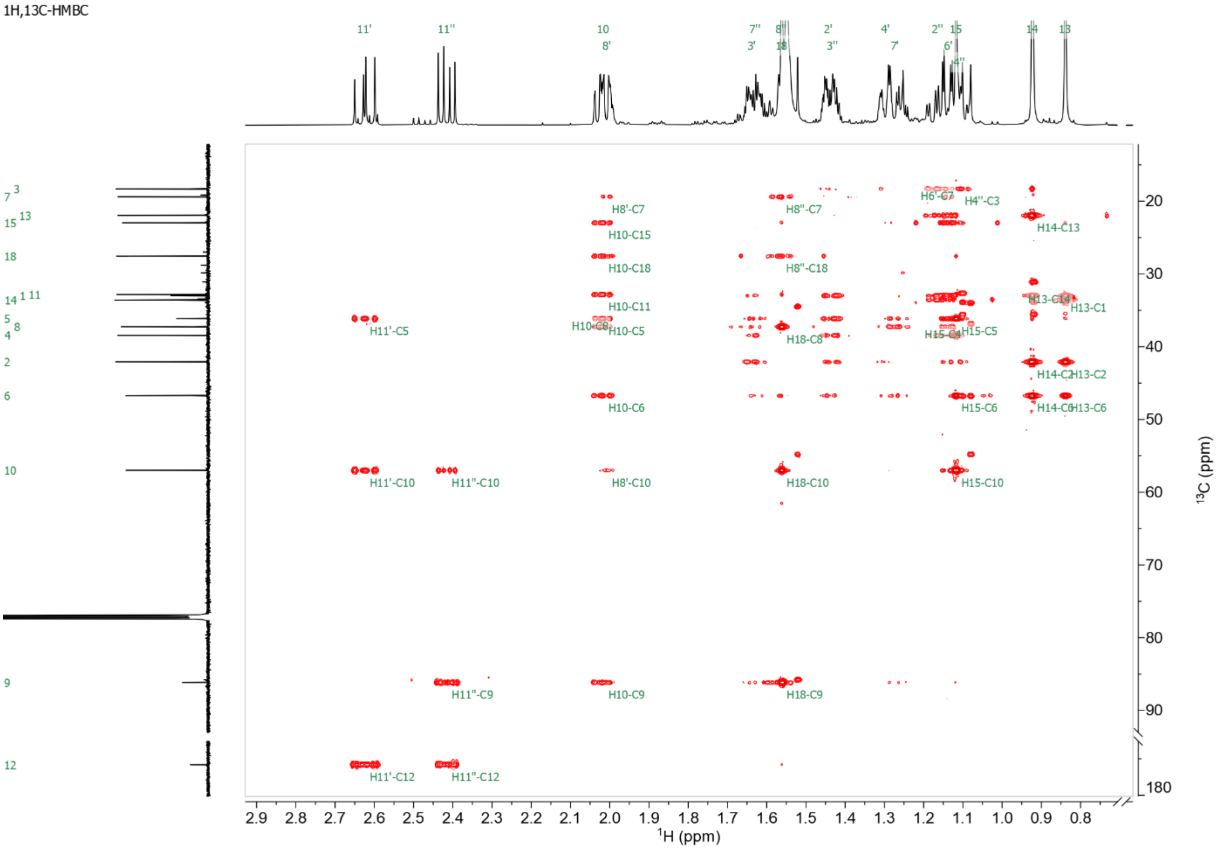

¹H,¹³C-HMBC (600 MHz, 151 MHz, $CDCl_3$) spectrum of a mixture predominantly containing (±)-9-*epi*-sclareolide **12b**, and (±)-5β,8α-sclareolide **12d** with assignments for **12b**

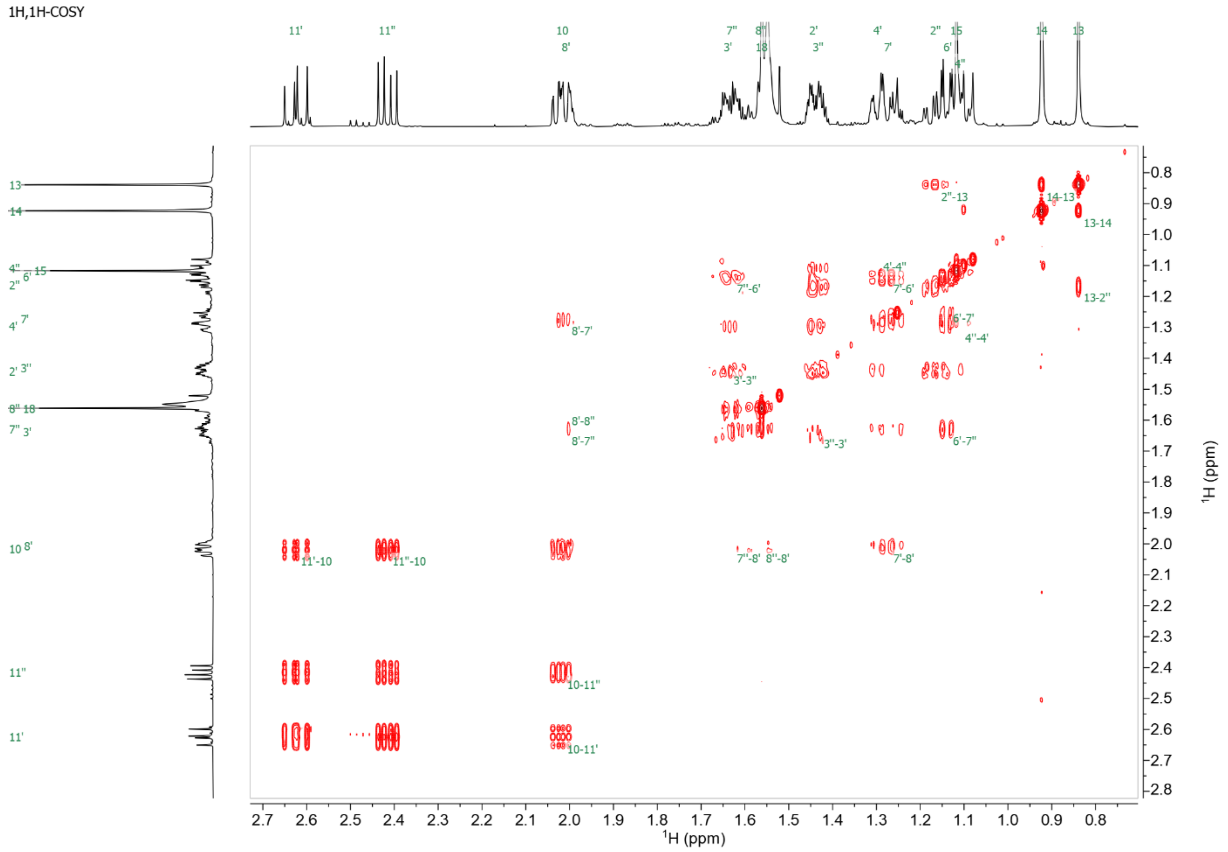

^{1}H,^{1}H-COSY (600 MHz, $CDCl_3$) spectrum of a mixture predominantly containing (±)-9-*epi*-sclareolide **12b**, and (±)-5β,8α-sclareolide **12d** with assignments for **12b**

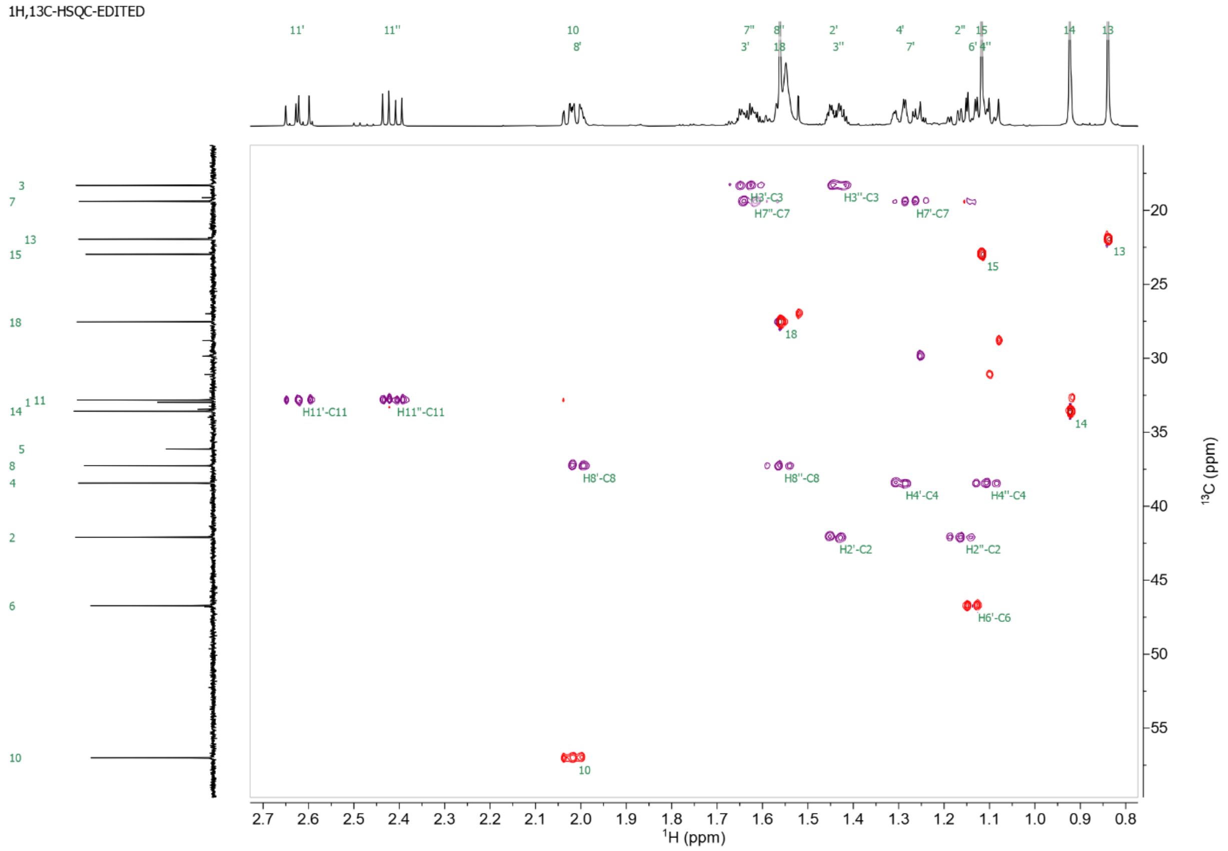

^{1}H,^{1}H-NOESY (600 MHz, $CDCl_3$) spectrum of a mixture predominantly containing (±)-9-*epi*-sclareolide **12b**, and (±)-5β,8α-sclareolide **12d** with assignments for **12b**

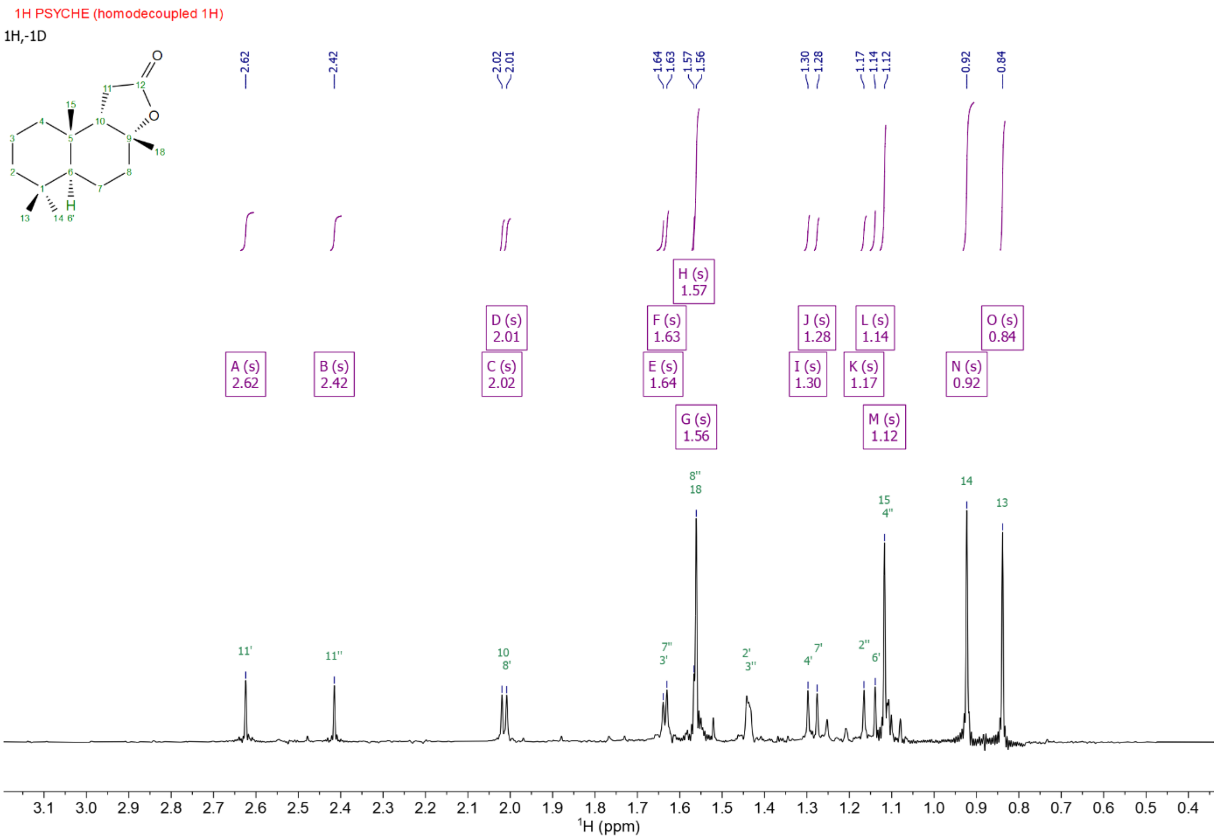

¹H-PSYCHE (Pure Shift Yielded by CHirp Excitation, homodecoupled ¹H NMR; 600 MHz, $CDCl_3$) spectrum of a mixture predominantly containing (±)-9-*epi*-sclareolide **12b**, and (±)-5β,8α-sclareolide **12d** with assignments for **12b**

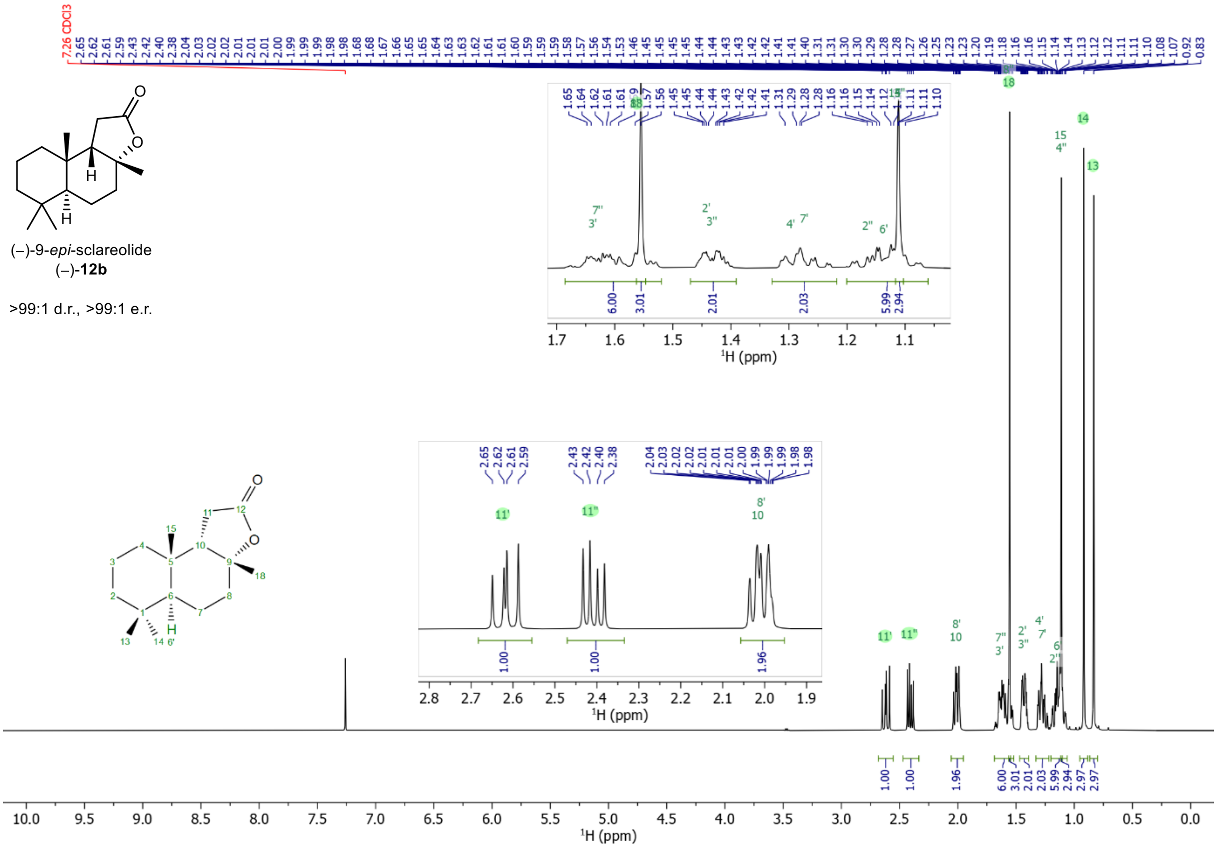

^{1}H NMR (501 MHz, $CDCl_3$) spectrum of synthetic (−)-9-*epi*-sclareolide **12b** with assignments prepared as reference material from (+)-sclareolide

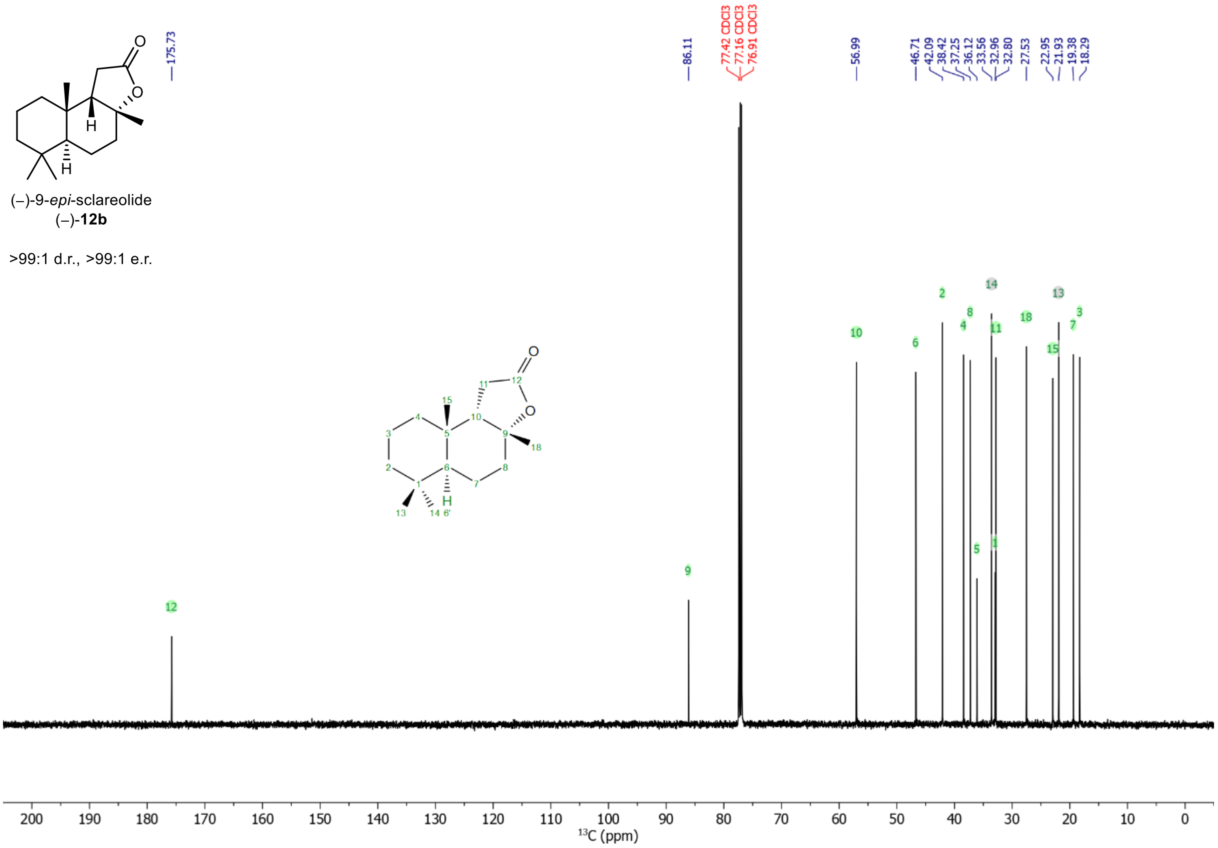

^{13}C NMR (126 MHz, $CDCl_3$) spectrum of synthetic (−)-9-*epi*-sclareolide **12b** with assignments prepared as reference material from (+)-sclareolide

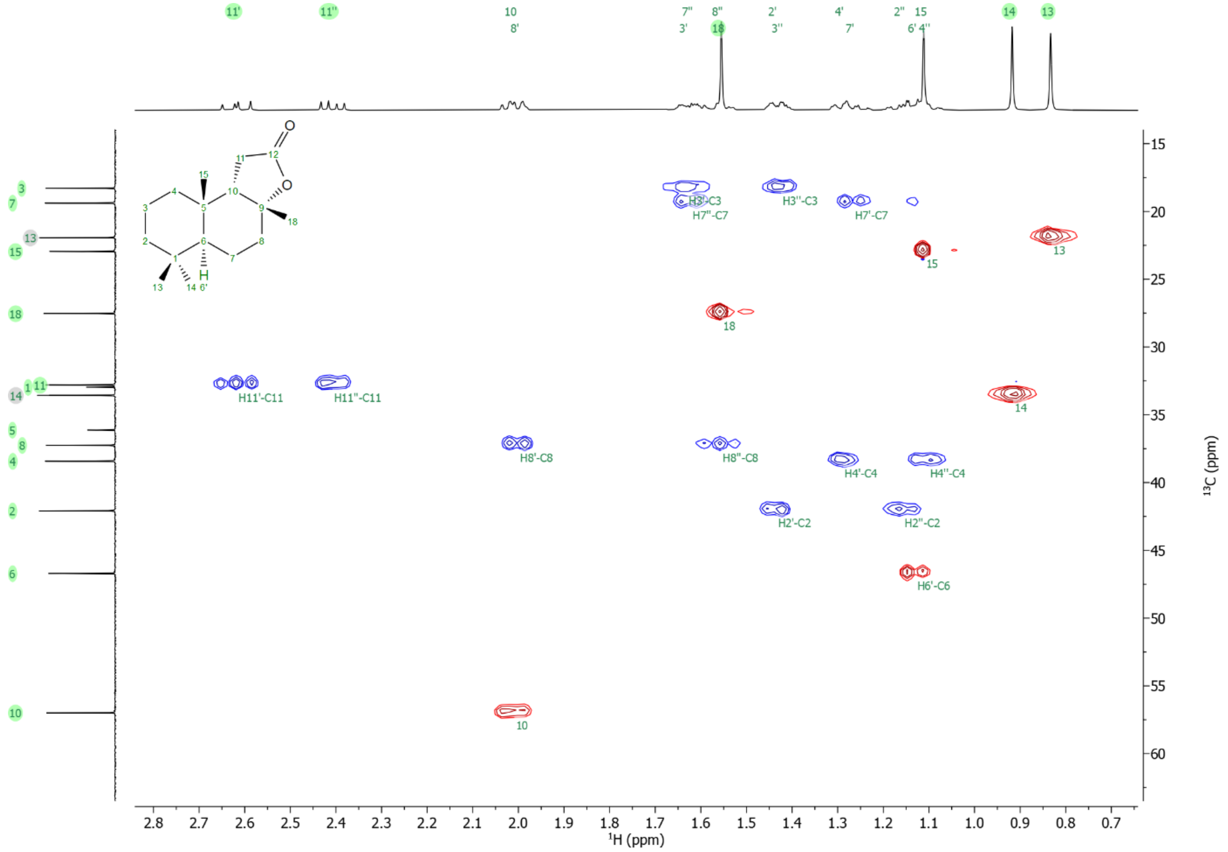

^{1}H,^{13}C-HSQC (501 MHz, 126 MHz, $CDCl_3$) spectrum of synthetic (−)-9-*epi*-sclareolide **12b** with assignments prepared as reference material from (+)-sclareolide

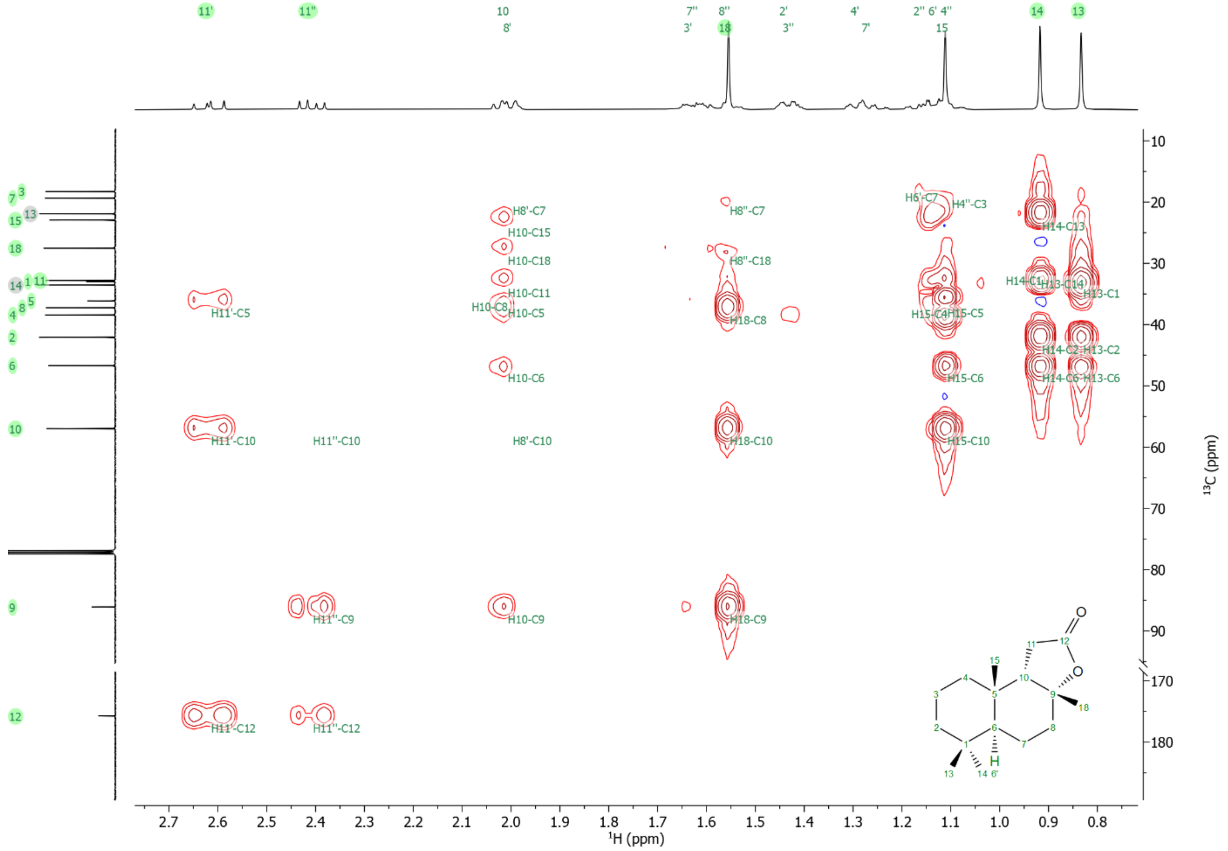

^{1}H,^{13}C-HMBC (501 MHz, 126 MHz, $CDCl_3$) spectrum of synthetic (−)-9-*epi*-sclareolide **12b** with assignments prepared as reference material from (+)-sclareolide

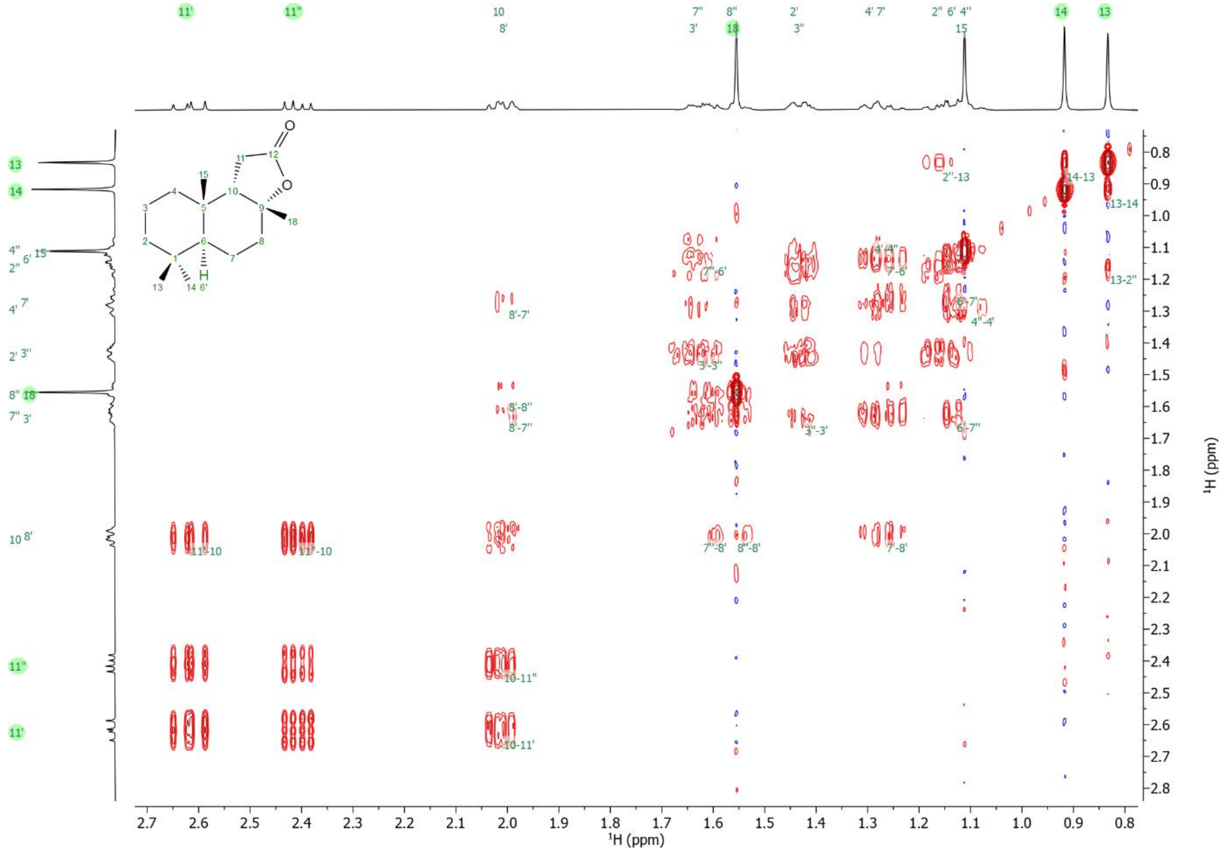

^{1}H,^{1}H-COSY (501 MHz, $CDCl_3$) spectrum of synthetic (−)-9-*epi*-sclareolide **12b** with assignments prepared as reference material from (+)-sclareolide

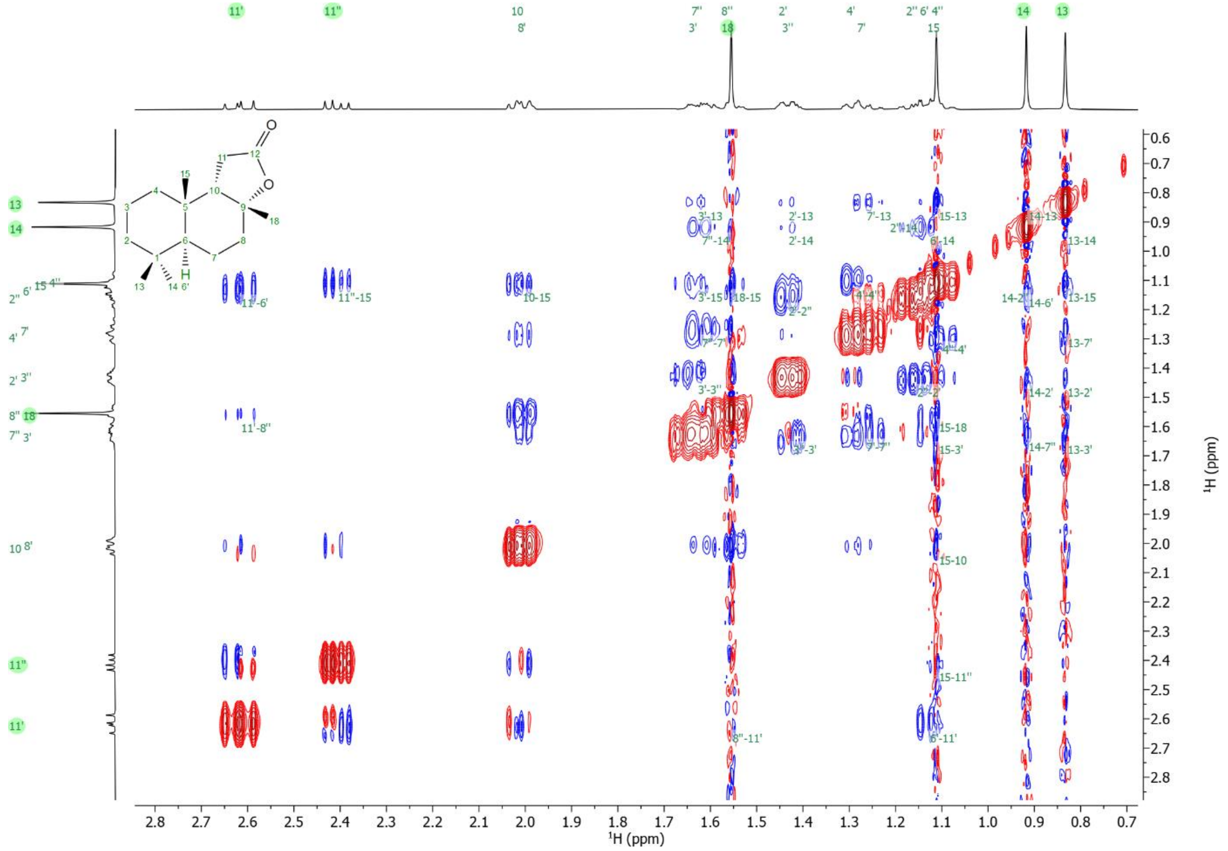

^{1}H,^{1}H-NOESY (501 MHz, $CDCl_3$) spectrum of synthetic (−)-9-*epi*-sclareolide **12b** with assignments prepared as reference material from (+)-sclareolide

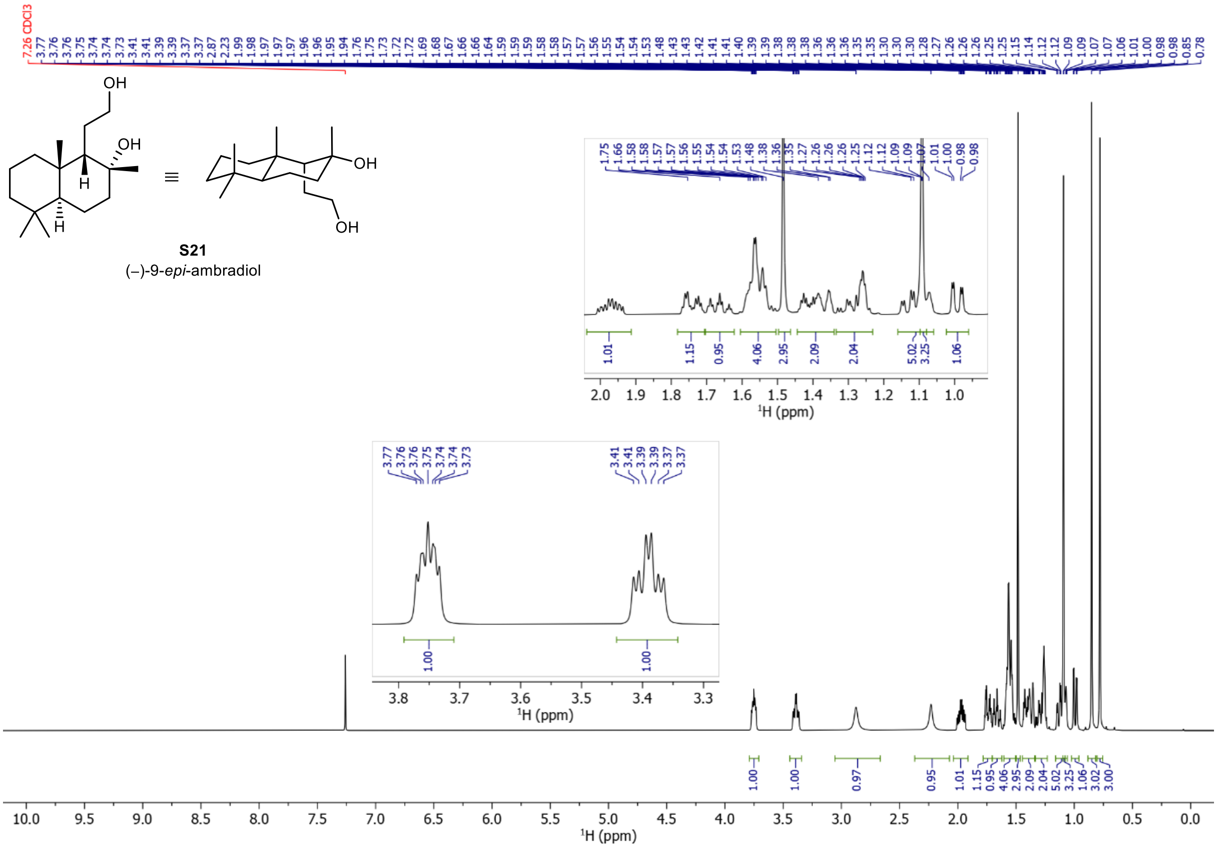

^{1}H NMR (501 MHz, $CDCl_3$) spectrum of (−)-9-*epi*-ambradiol **S21**

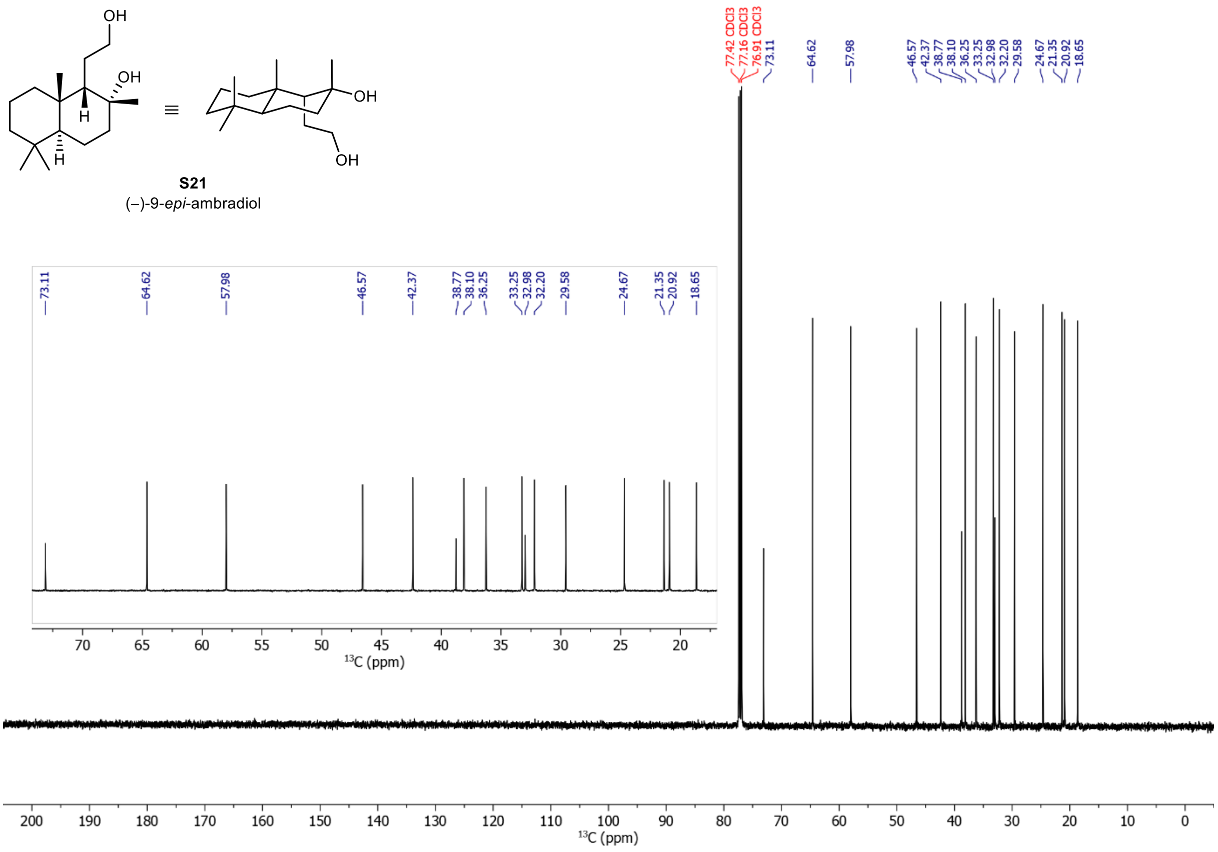

^{13}C NMR (126 MHz, $CDCl_3$) spectrum of (−)-9-*epi*-ambradiol **S21**

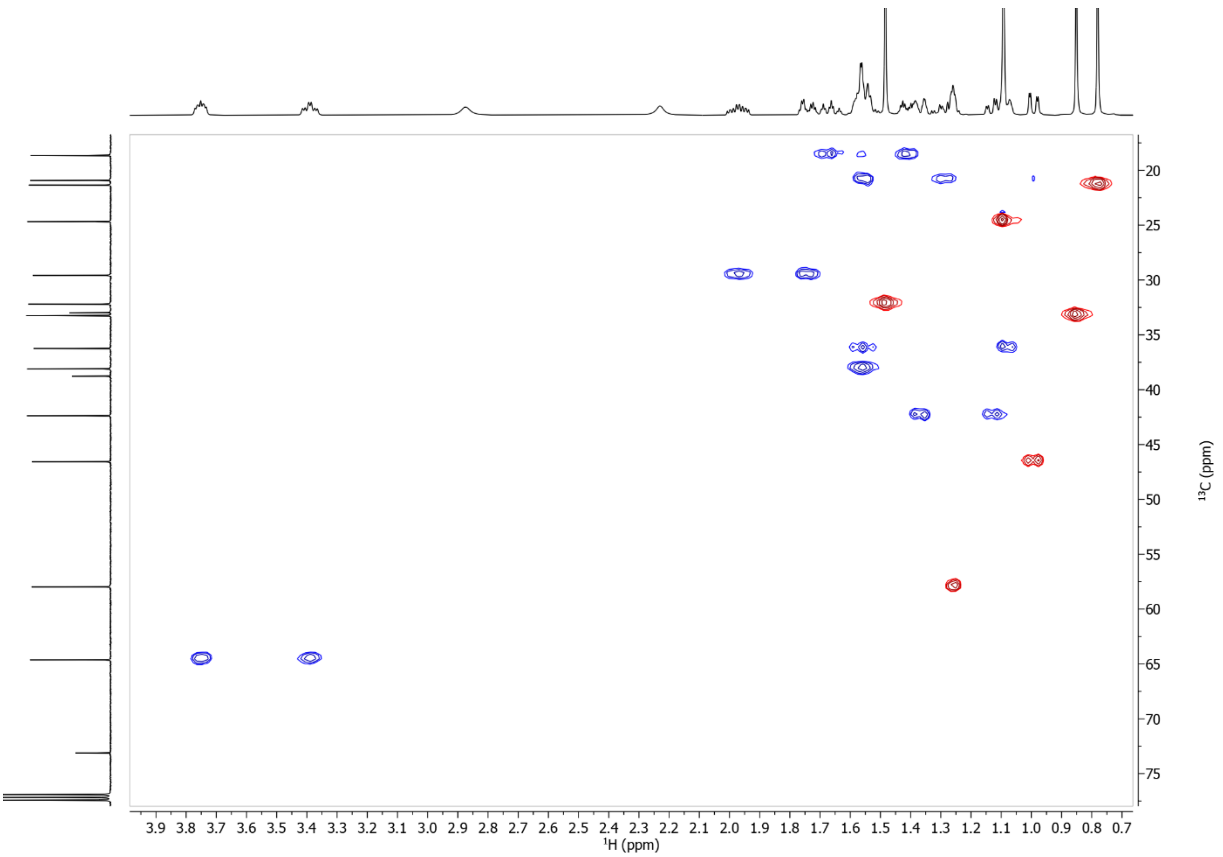

^{1}H,^{13}C-HSQC (501 MHz, 126 MHz, $CDCl_3$) spectrum of (−)-9-*epi*-ambradiol **S21**

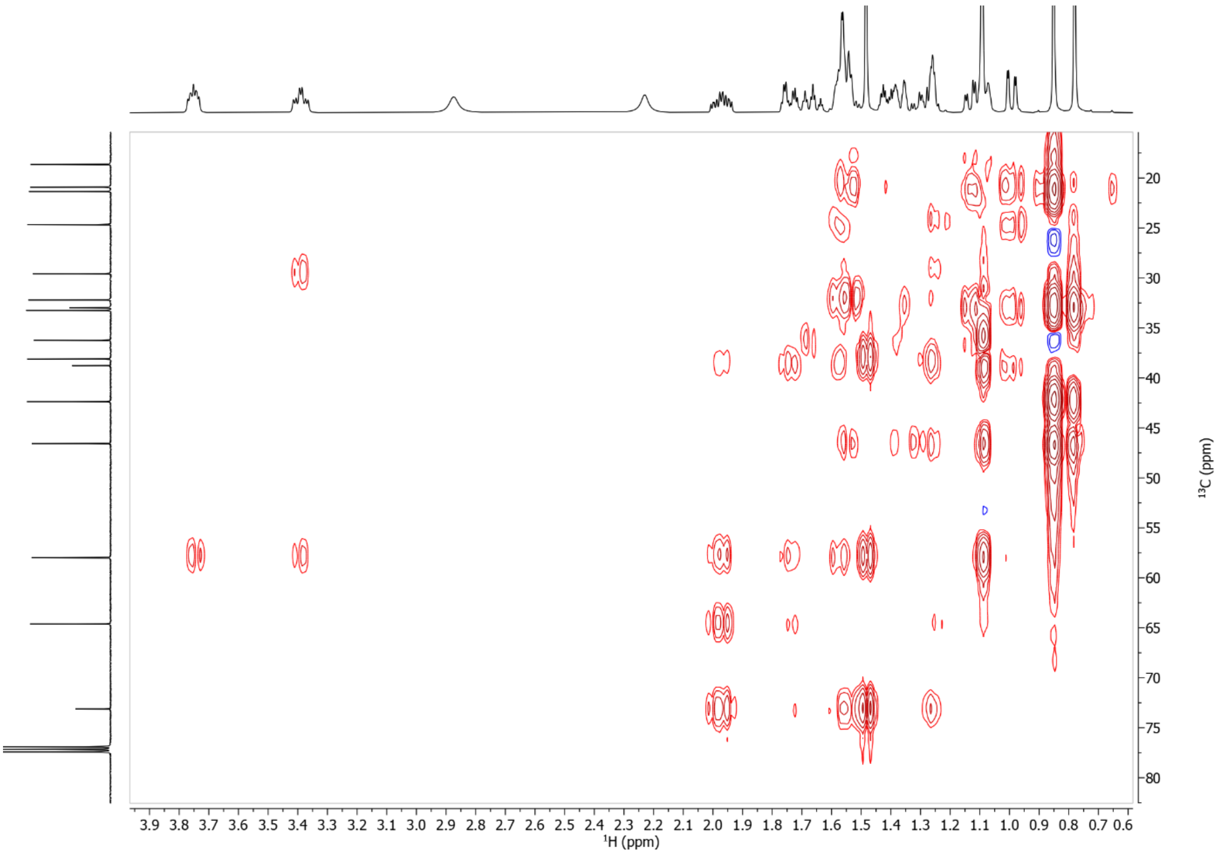

^{1}H,^{13}C-HMBC (501 MHz, 126 MHz, $CDCl_3$) spectrum of (−)-9-*epi*-ambradiol **S21**

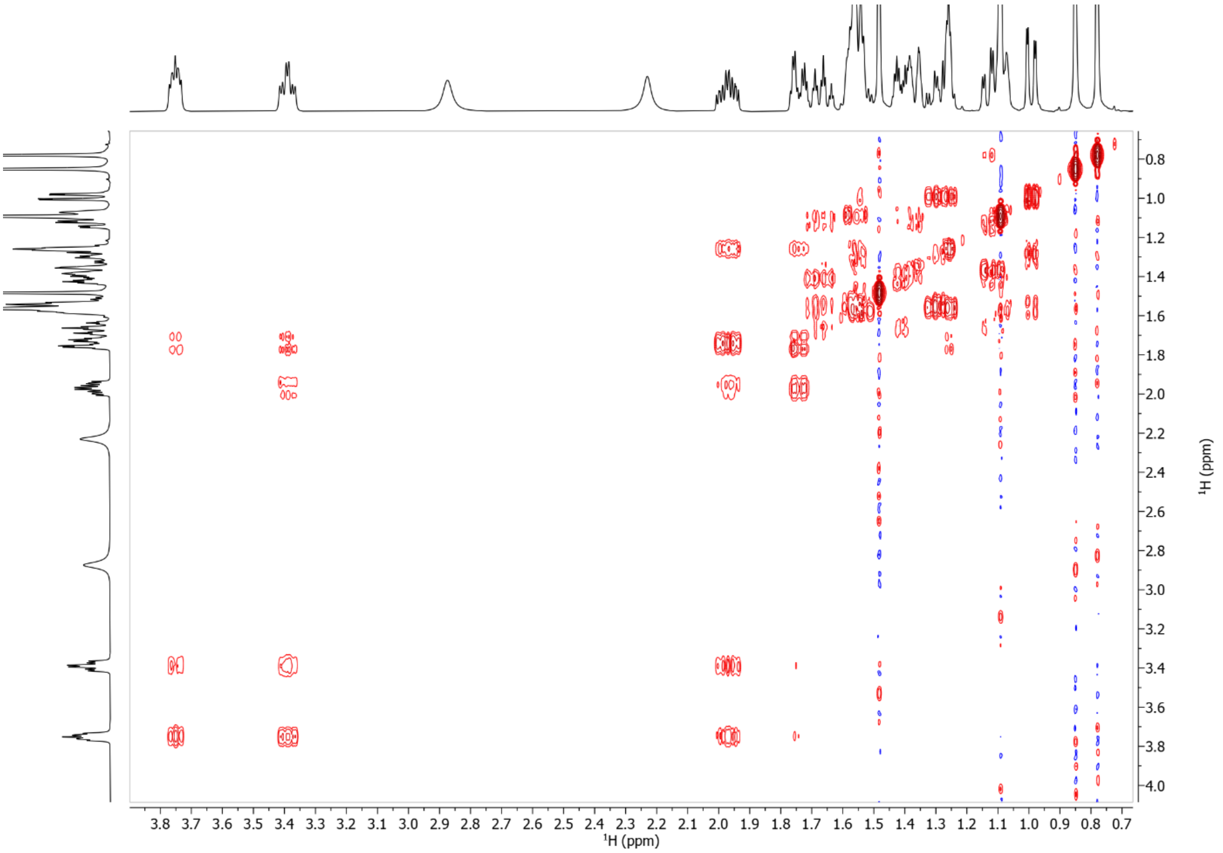

^{1}H,^{1}H-COSY (501 MHz, $CDCl_3$) spectrum of (−)-9-*epi*-ambradiol **S21**

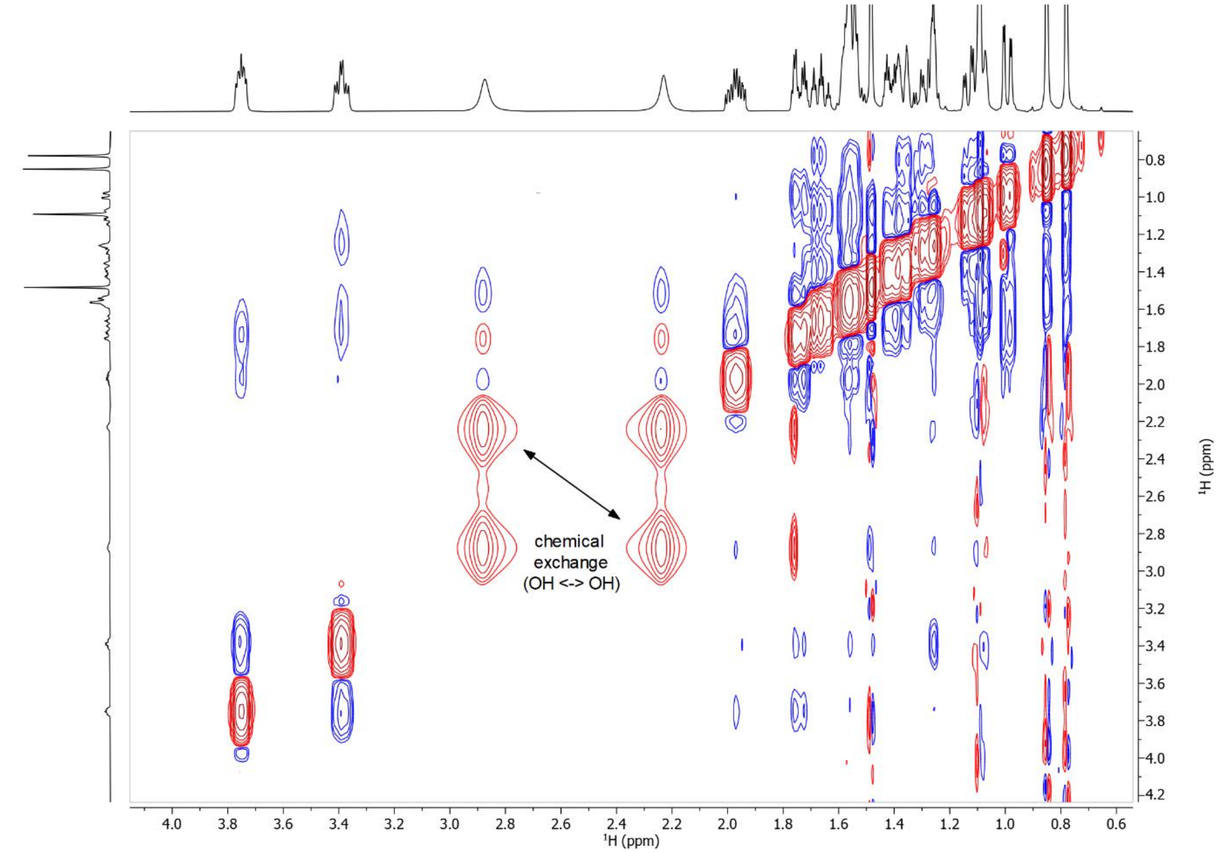

^{1}H,^{1}H-NOESY (501 MHz, $CDCl_3$) spectrum of (−)-9-*epi*-ambradiol **S21**

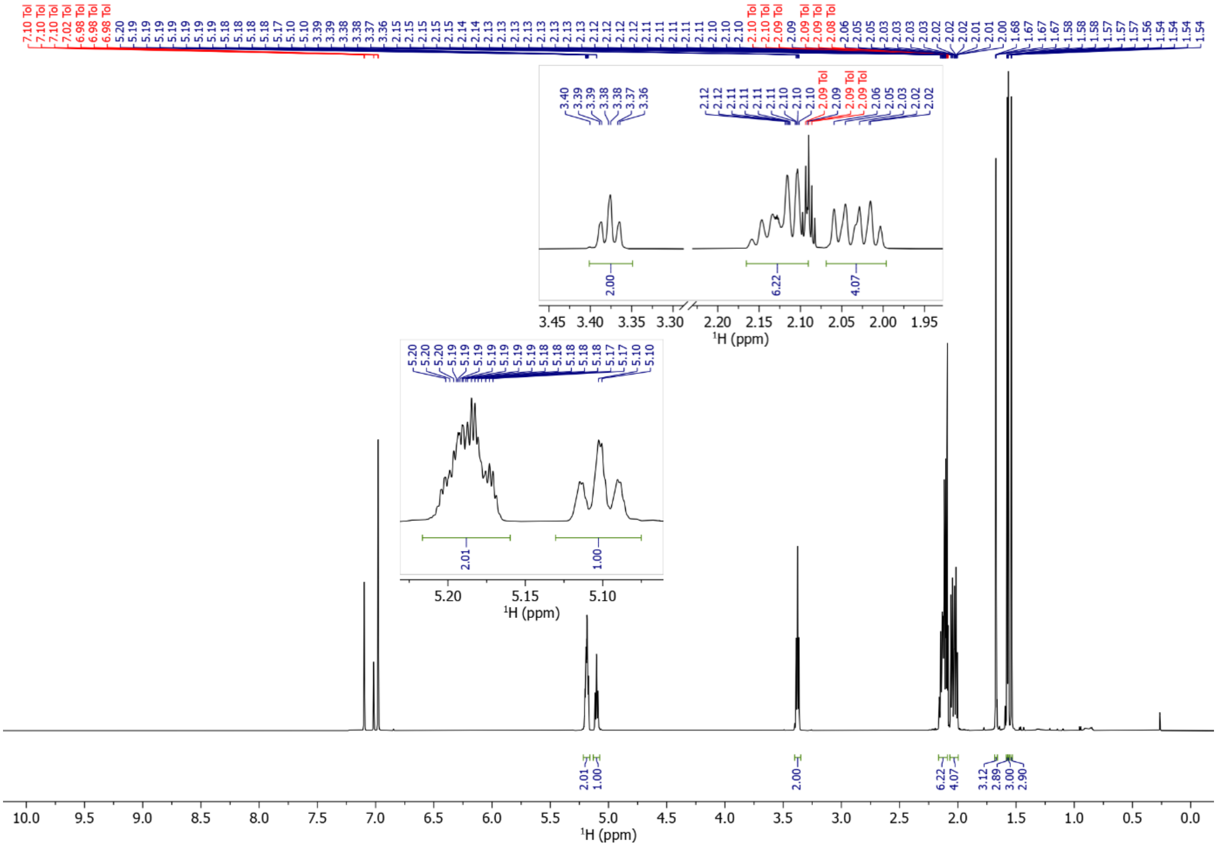

^{1}H NMR (600 MHz, toluene-d_8) spectrum of (3*E*,7*E*)-homofarnesol-*d*-1 **1a**-d_1 (ca. 90% D)

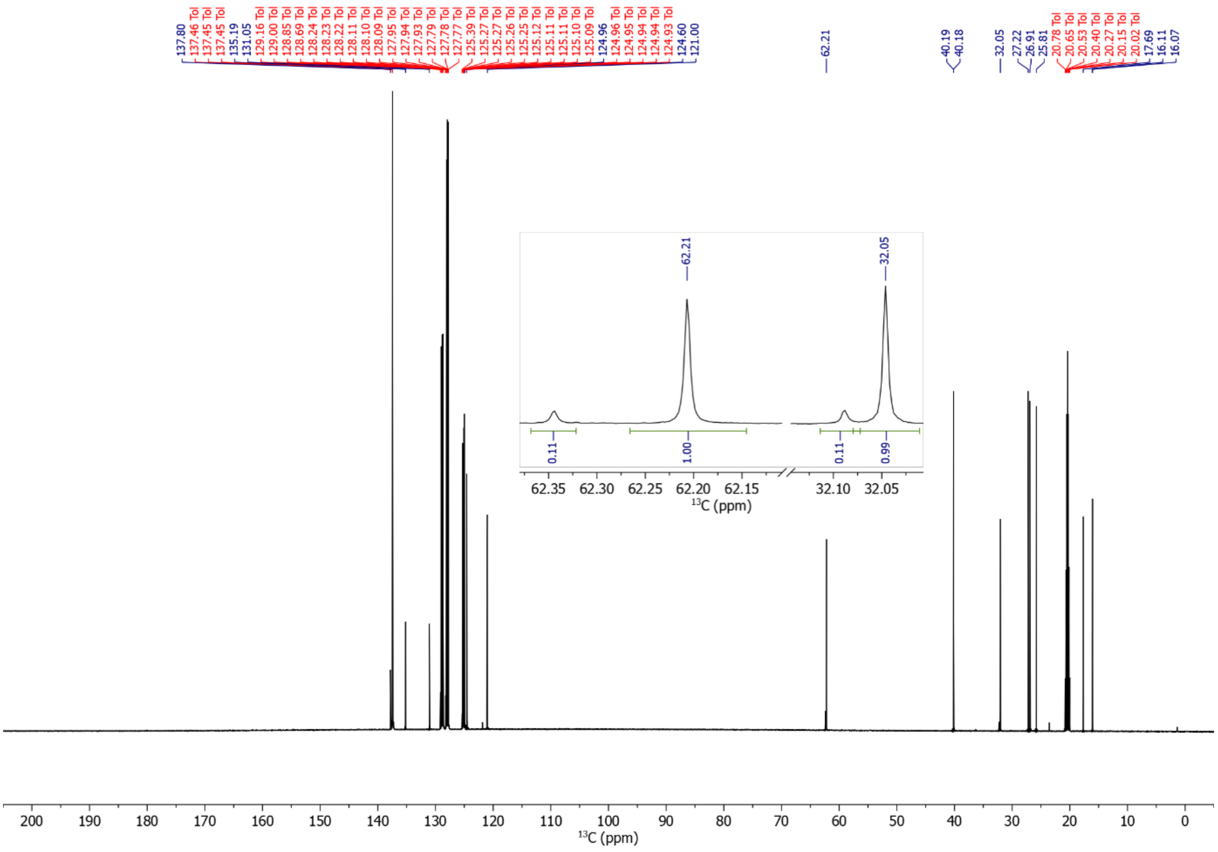

^{13}C NMR (151 MHz, toluene-d_8) spectrum of (3*E*,7*E*)-homofarnesol-*d*-1 **1a**-d_1 (ca. 90% D)

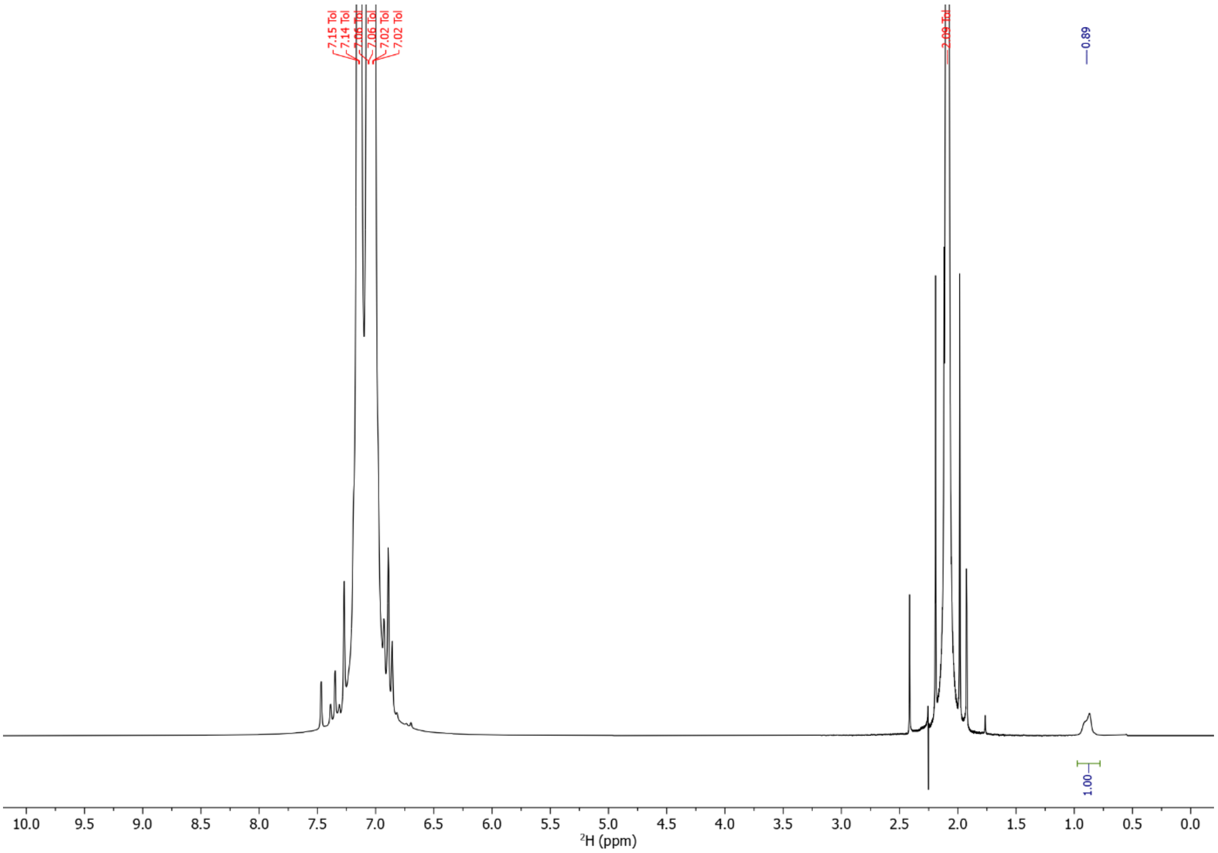

$^{2}H\{^{1}H\}$ NMR (92 MHz, toluene-d_8) spectrum of (3*E*,7*E*)-homofarnesol-*d*-1 **1a**-d_1 (ca. 90% D)

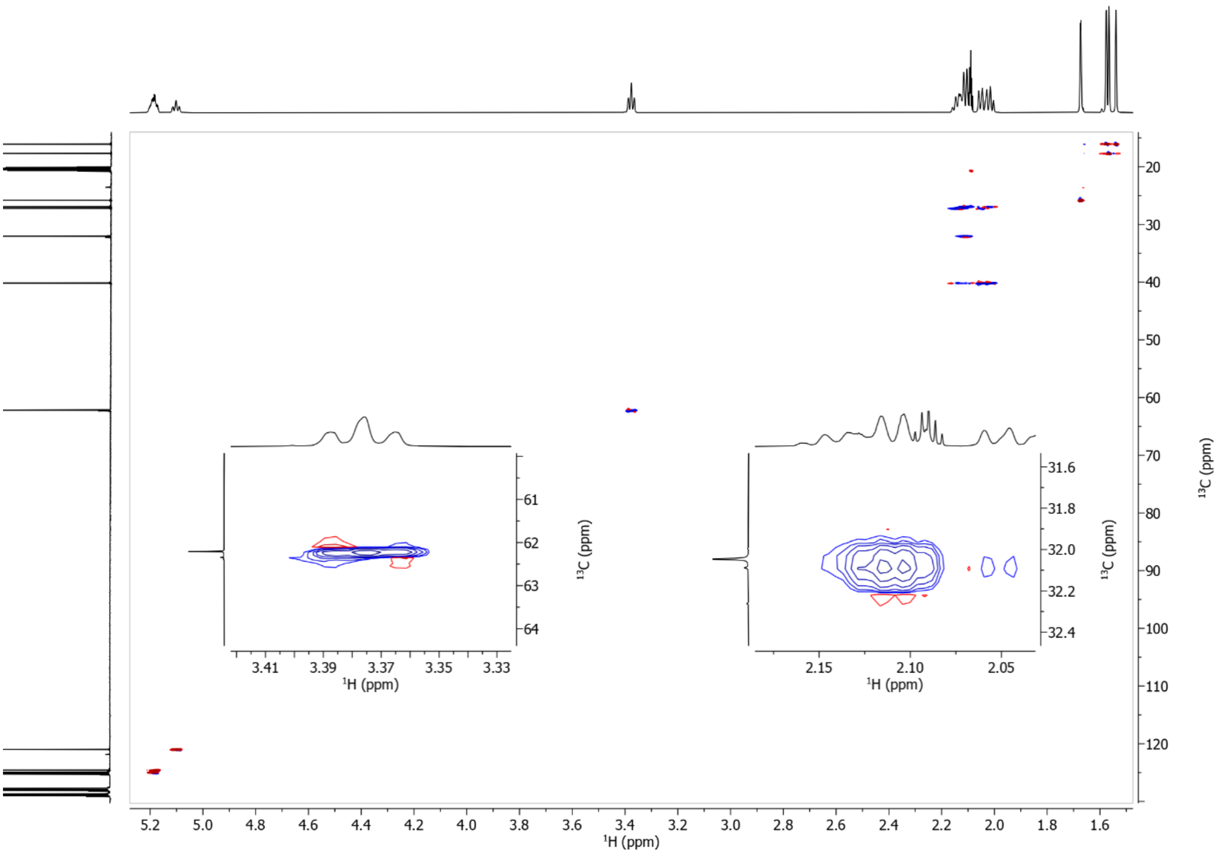

Multiplicity-edited **$^{1}H,^{13}C$-HSQC** (600 MHz, 151 MHz, toluene-d_8) spectrum of (3*E*,7*E*)-homofarnesol-*d*-1 **1a**-d_1 (ca. 90% D)

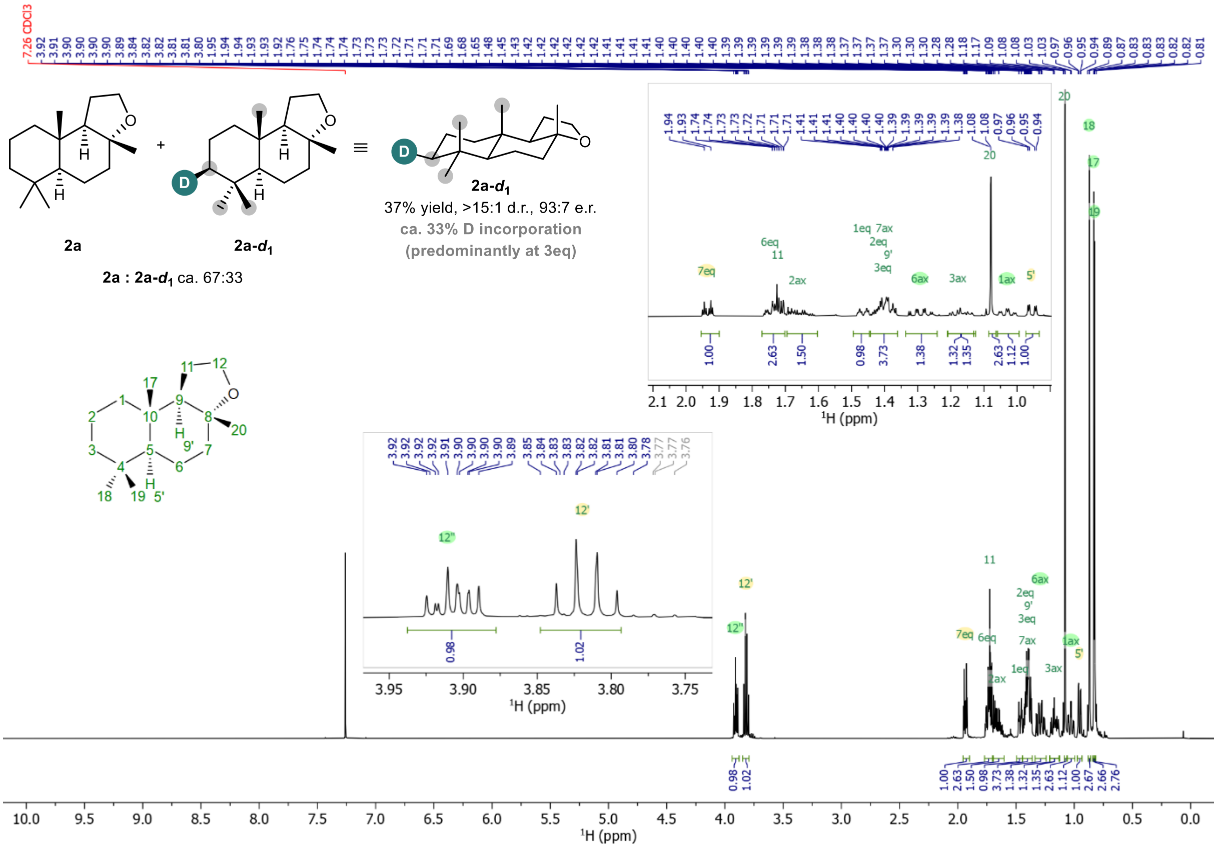

^{1}**H NMR** (600 MHz, $CDCl_3$) spectrum of a mixture of (−)-ambrox **2a** and (−)-ambrox-d_1 **2a**-d_1 (ca. 67:33) obtained in the deuterium-labeling study with IDPi **8g**

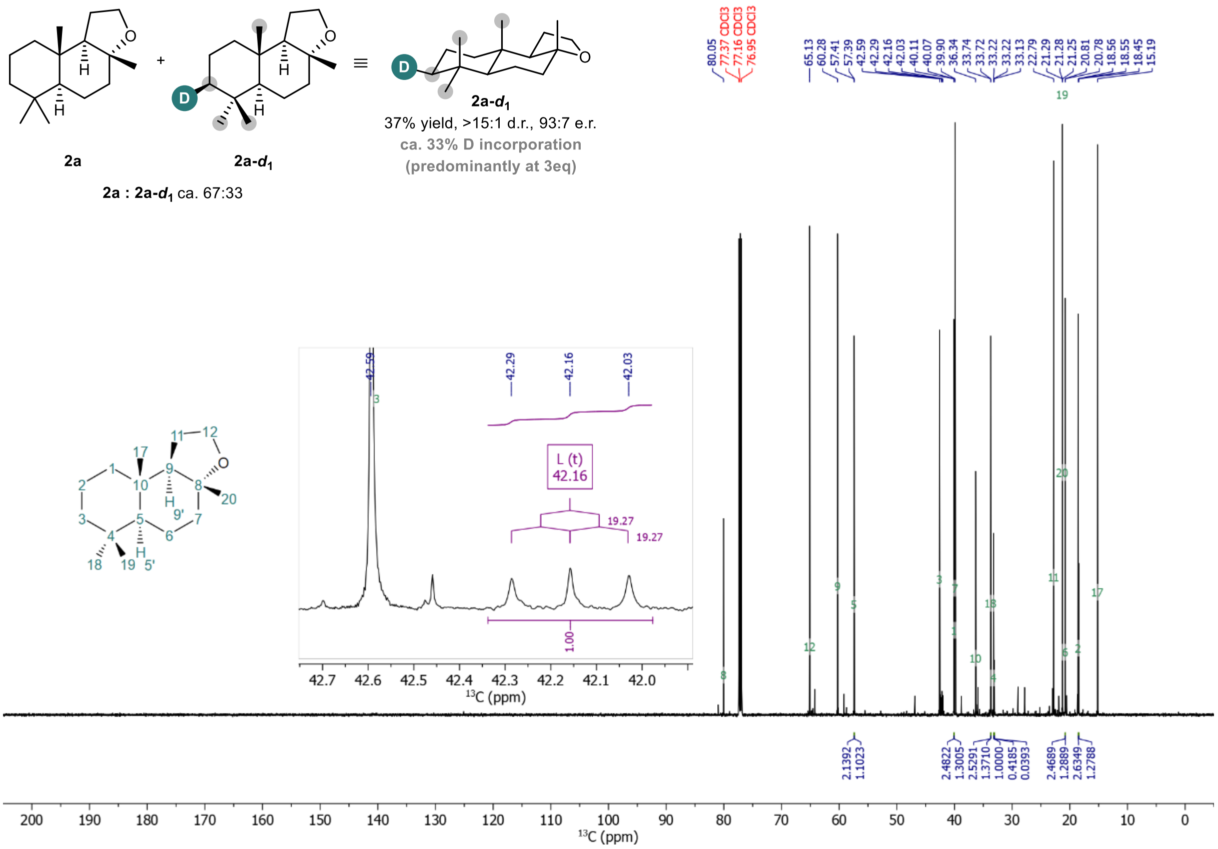

^{13}**C NMR** (151 MHz, $CDCl_3$) spectrum of a mixture of (−)-ambrox **2a** and (−)-ambrox-d_1 **2a**-d_1 (ca. 67:33) obtained in the deuterium-labeling study with IDPi **8g**

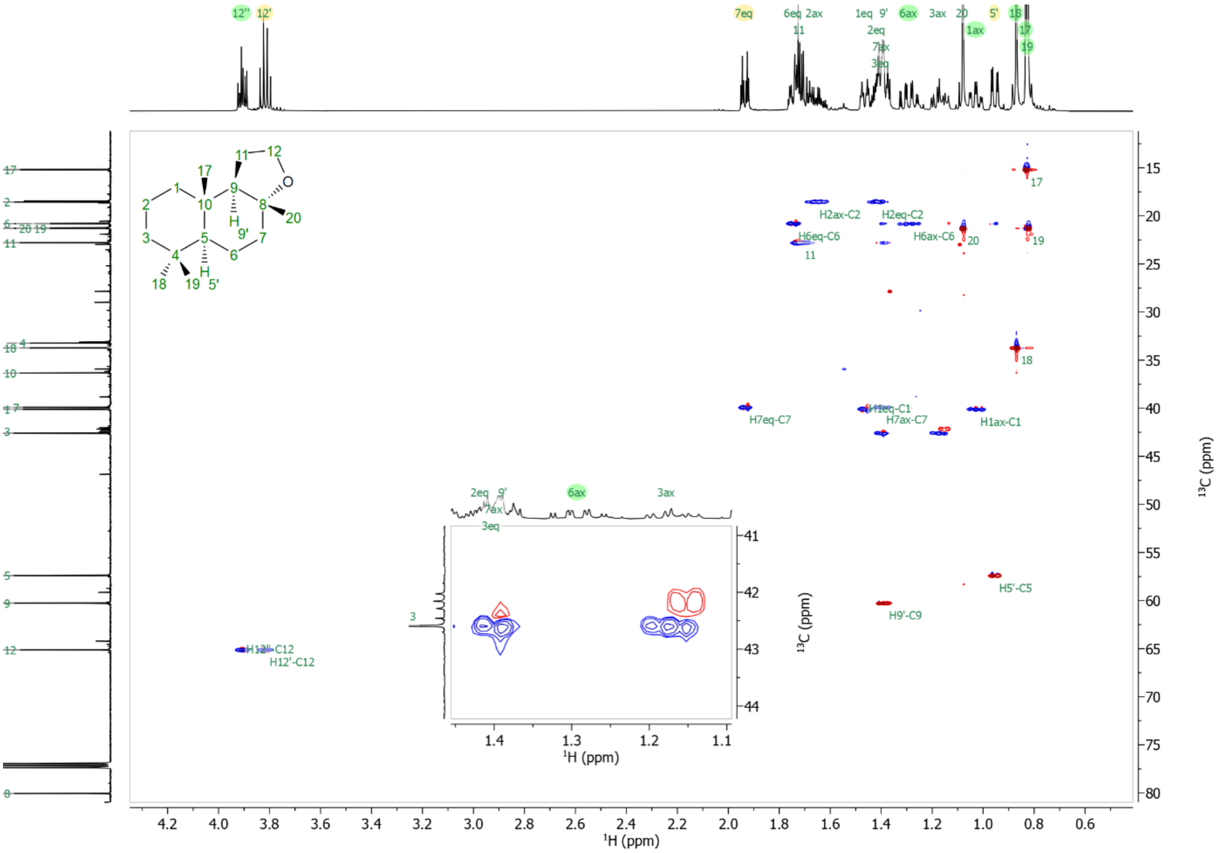

Multiplicity-edited **^{1}H,^{13}C-HSQC** (600 MHz, 151 MHz, $CDCl_3$) spectrum of a mixture of (−)-ambrox **2a** and (−)-ambrox-*d*$_1$ **2a-*d*$_1$** (ca. 67:33) obtained in the deuterium-labeling study with IDPi **8g**

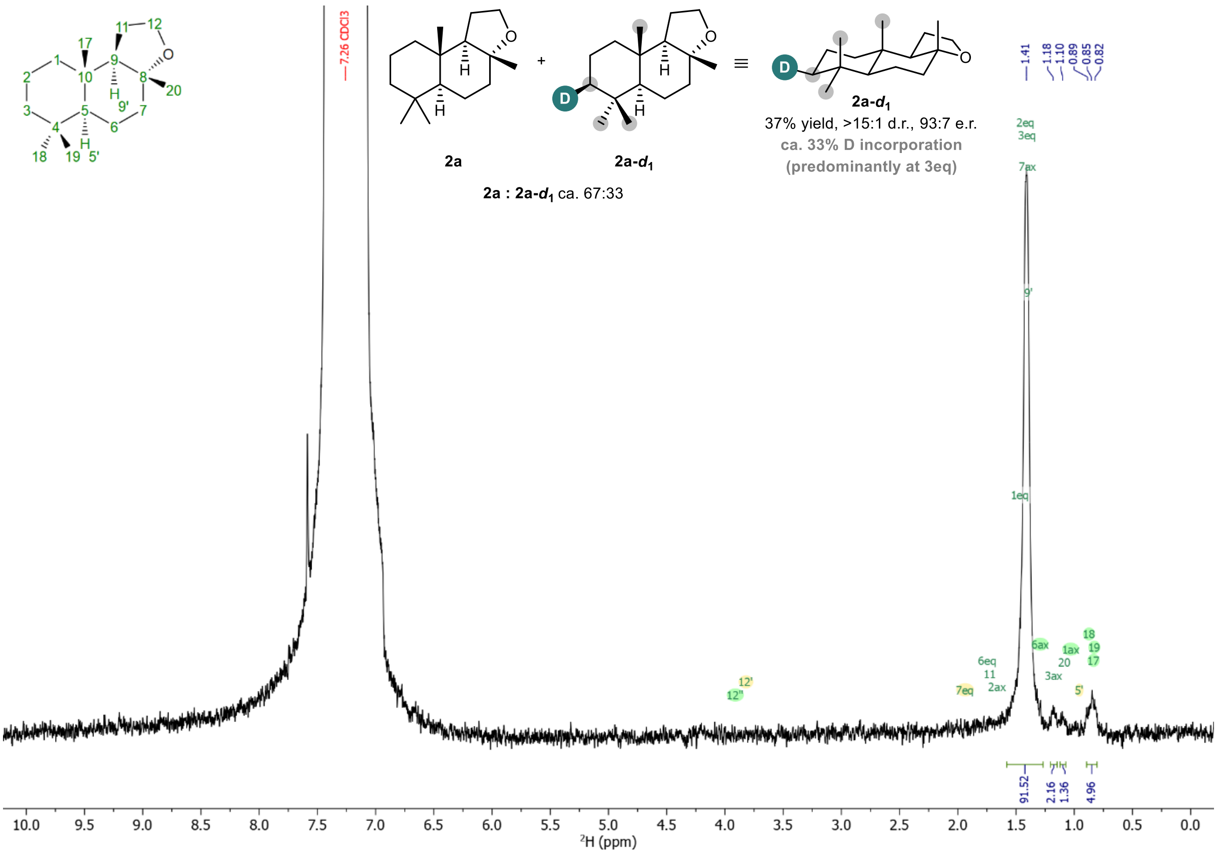

^{2}H{^{1}H}-NMR (92 MHz, $CDCl_3$) spectrum of a mixture of (−)-ambrox **2a** and (−)-ambrox-*d*$_1$ **2a-*d*$_1$** (ca. 67:33) obtained in the deuterium-labeling study with IDPi **8g**

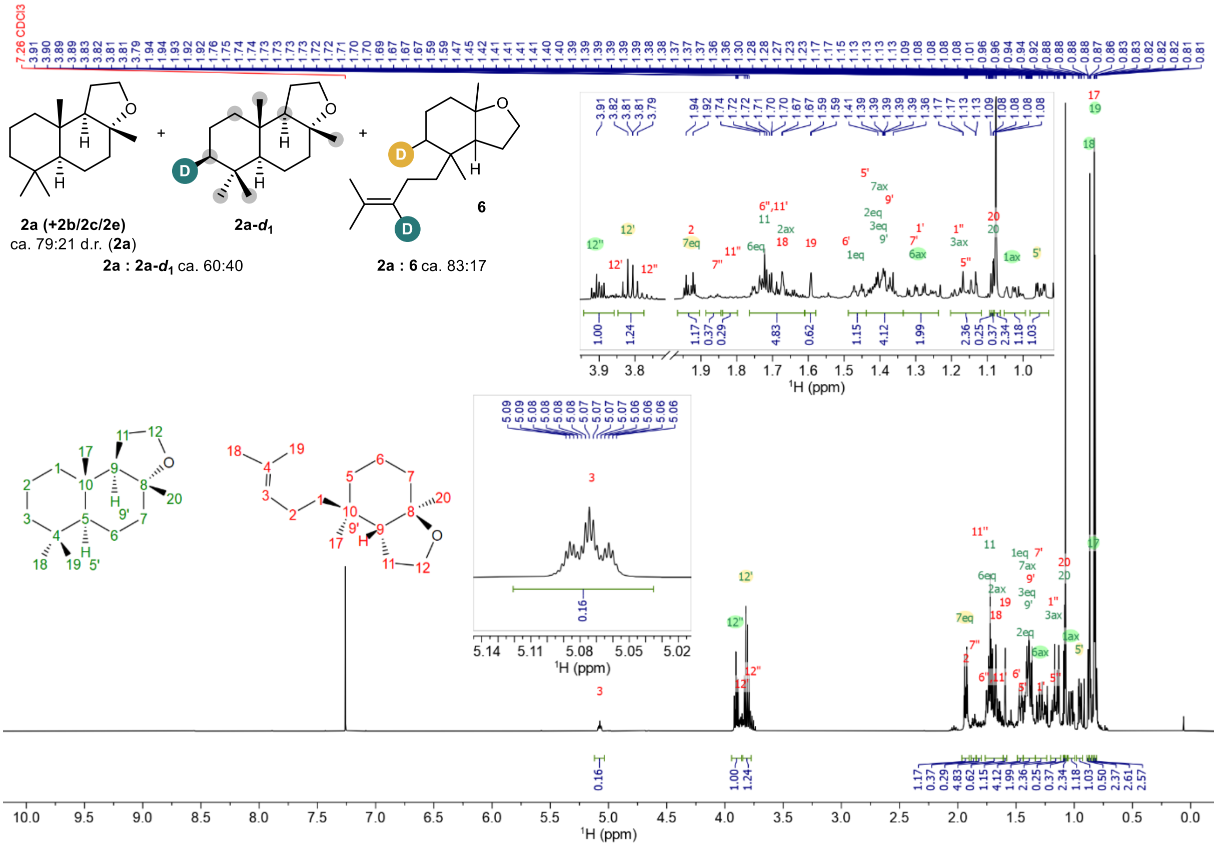

^{1}H NMR (600 MHz, $CDCl_3$) spectrum of a mixture of (−)-ambrox **2a** and (−)-ambrox-d_1 **2a**-d_1 (ca. 60:40) and side product **6** (**2a**:**6** ca. 83:17) obtained in the deuterium-labeling study with PADI **9**

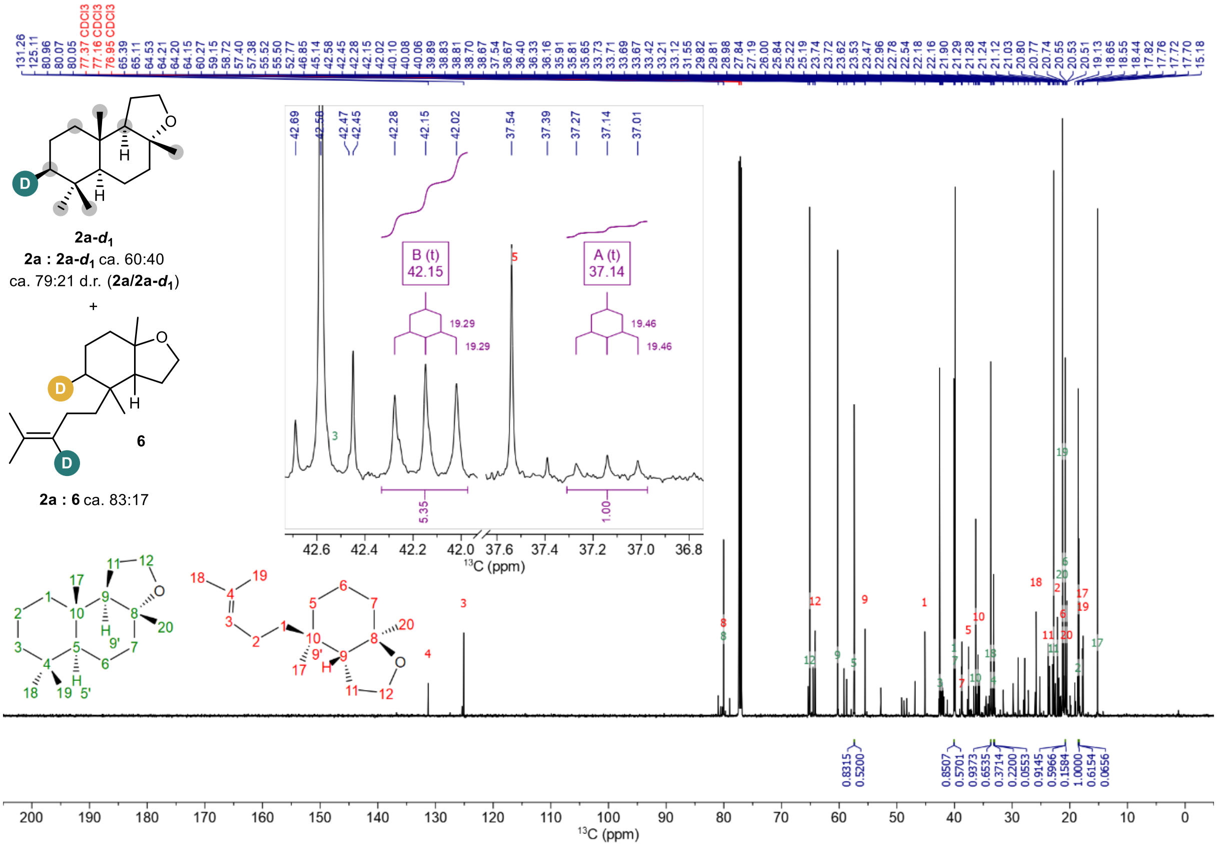

^{13}C NMR (151 MHz, $CDCl_3$) spectrum of a mixture of (−)-ambrox **2a** and (−)-ambrox-d_1 **2a**-d_1 (ca. 60:40) and side product **6** (**2a**:**6** ca. 83:17) obtained in the deuterium-labeling study with PADI **9**

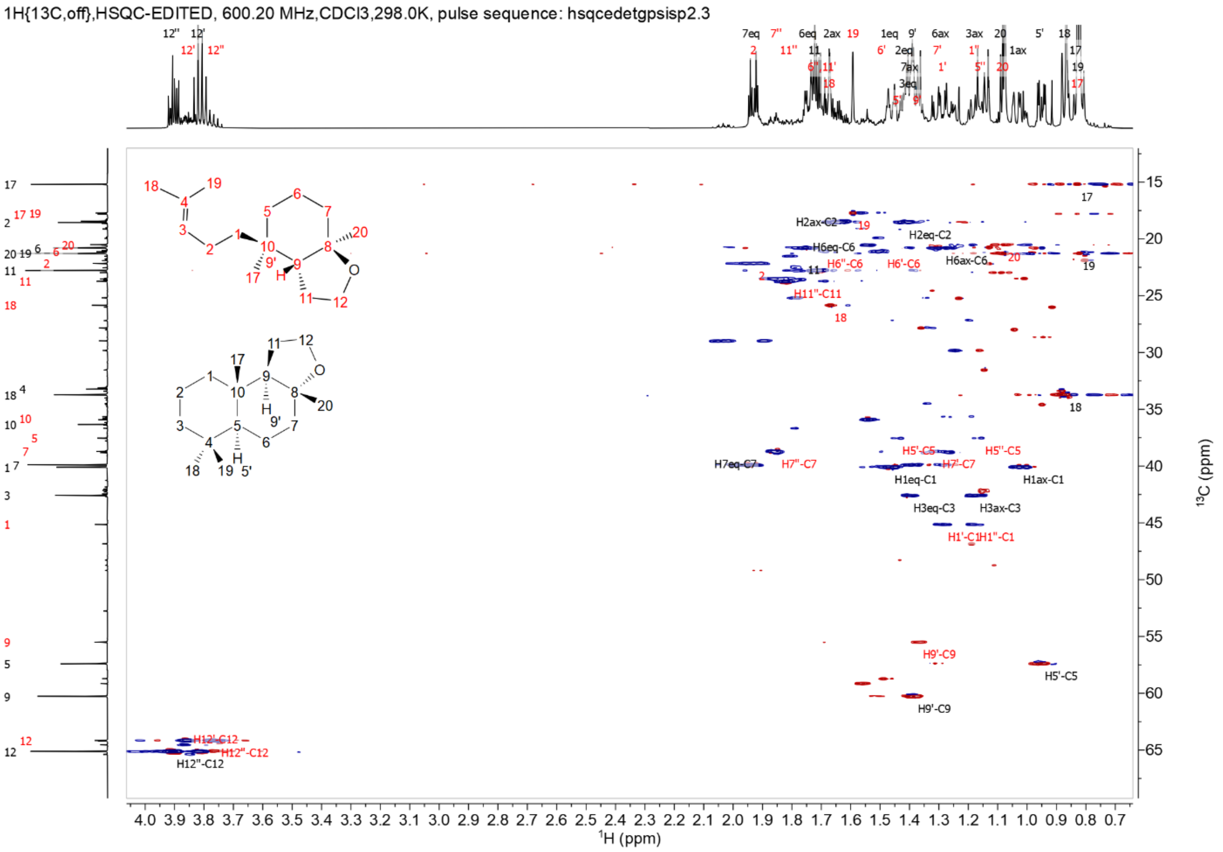

Multiplicity-edited **^{1}H,^{13}C-HSQC** (600 MHz, 151 MHz, CDCl$_3$) spectrum of a mixture of (−)-ambrox **2a** and (−)-ambrox-d_1 **2a**-d_1 (ca. 60:40) and side product **6** (**2a**:**6** ca. 83:17) obtained in the deuterium-labeling study with PADI **9**

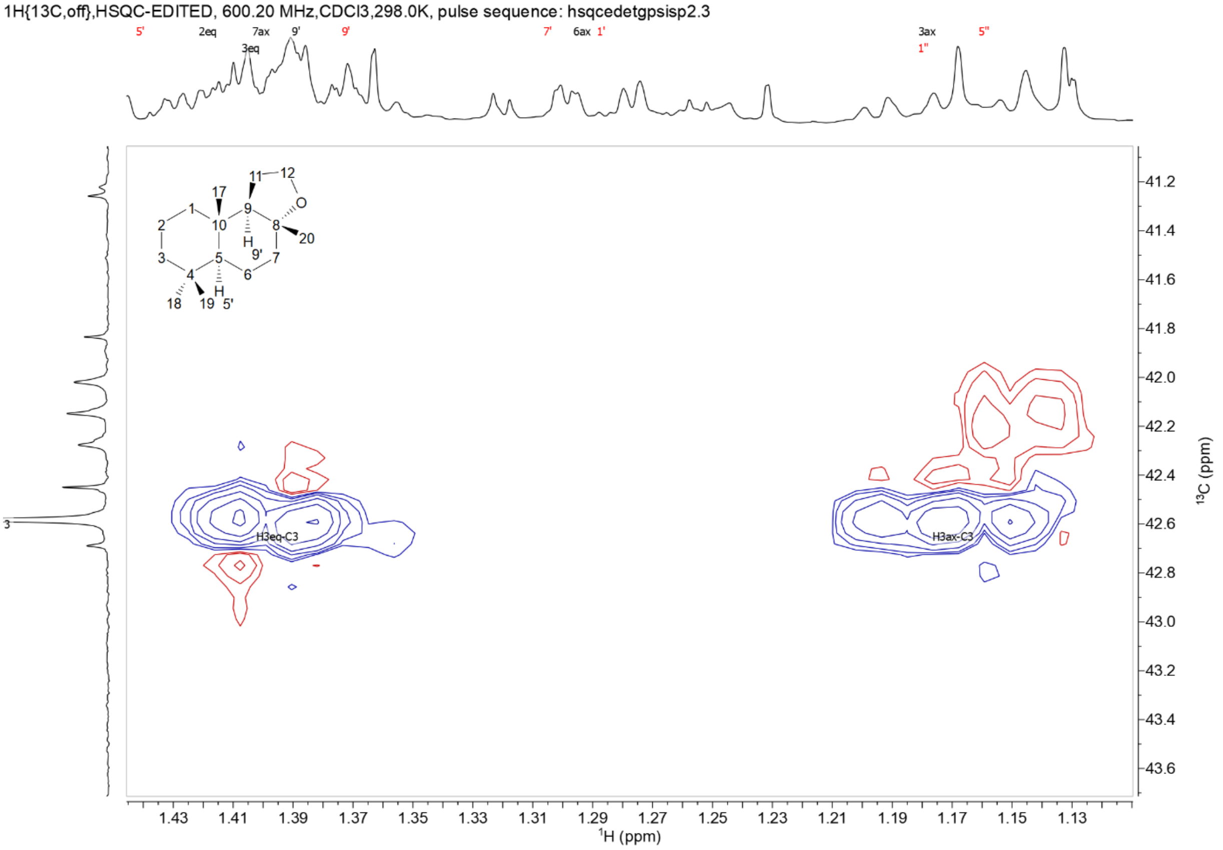

Excerpt of the multiplicity-edited **^{1}H,^{13}C-HSQC** (600 MHz, 151 MHz, CDCl$_3$) showing the chemical shift region of (−)-ambrox **2a** and (−)-ambrox-d_1 **2a**-d_1 (ca. 60:40)

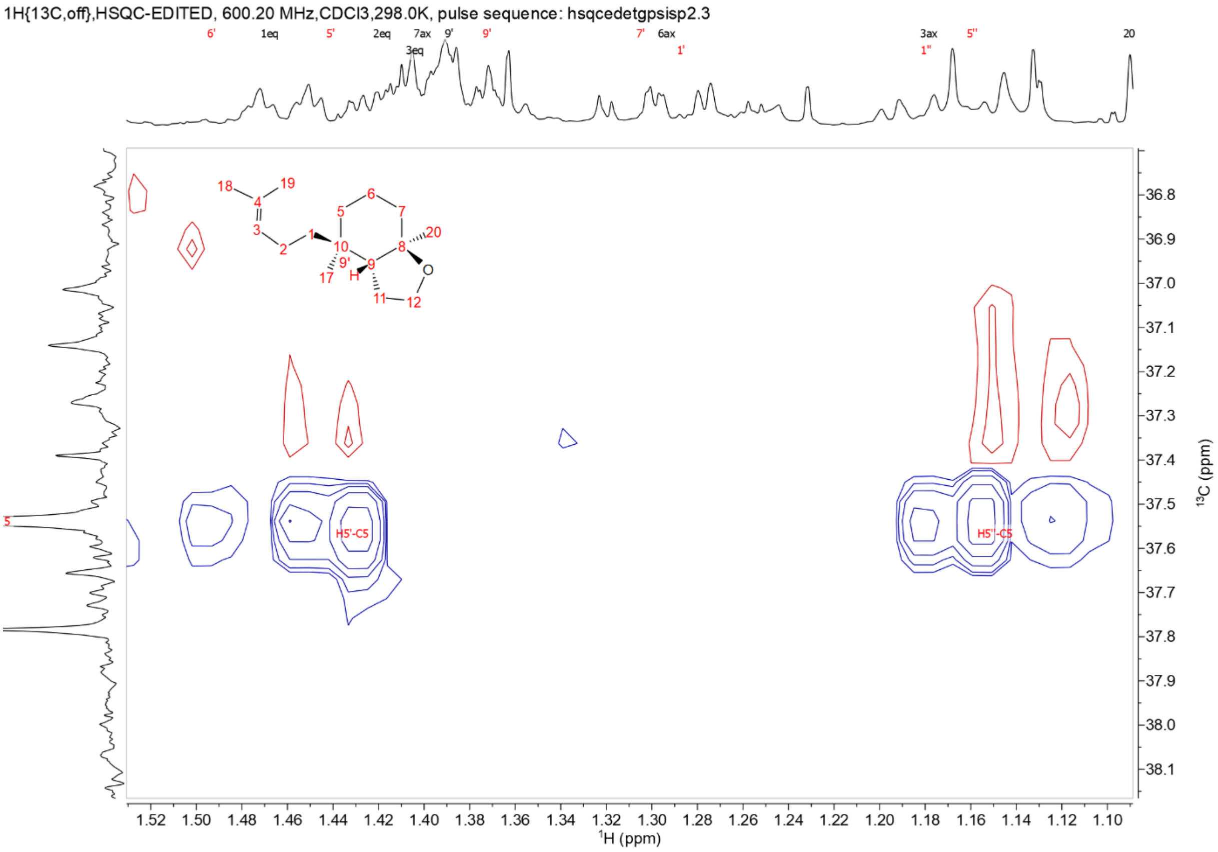

Excerpt of the multiplicity-edited **¹H,¹³C-HSQC** (600 MHz, 151 MHz, $CDCl_3$) showing the chemical shift region of side product **6**

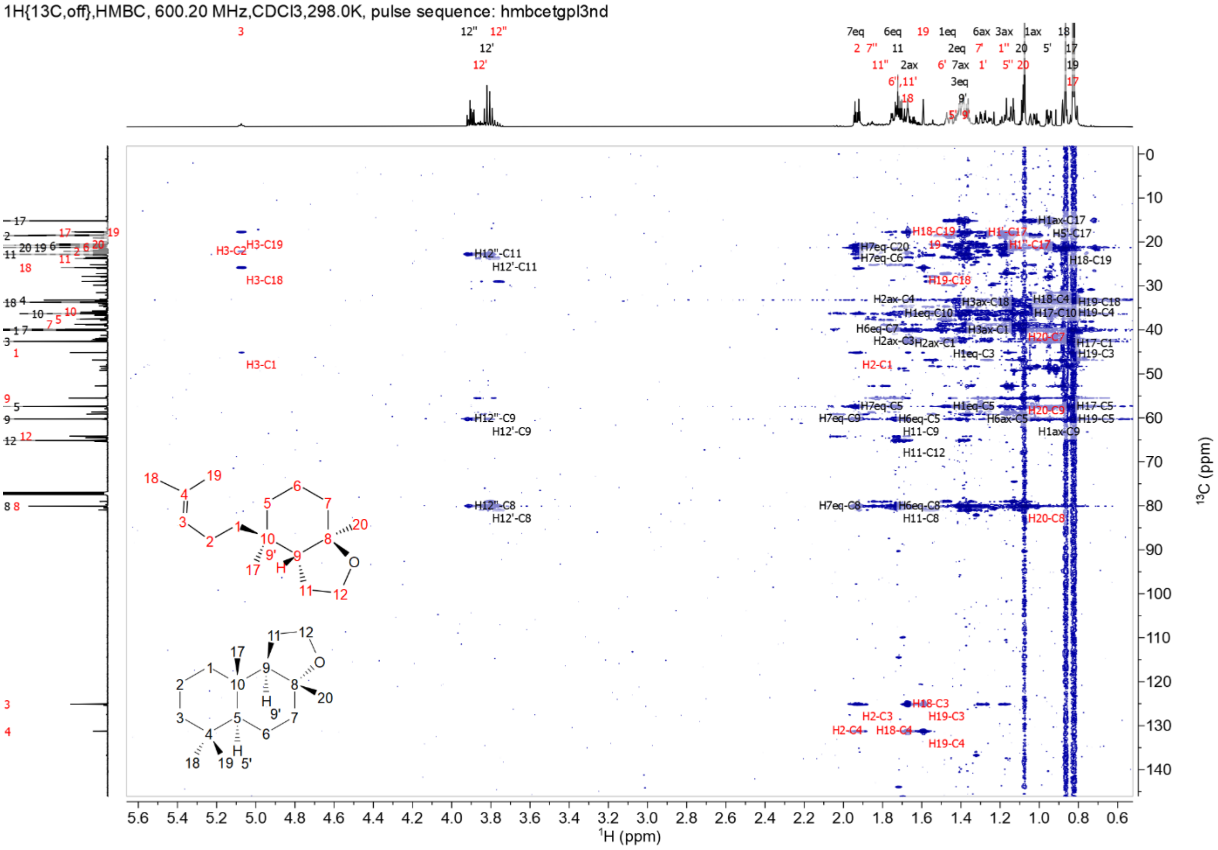

¹H,¹³C-HMBC (600 MHz, 151 MHz, $CDCl_3$) spectrum of a mixture of (−)-ambrox **2a** and (−)-ambrox-d_1 **2a**-d_1 (ca. 60:40) and side product **6** (**2a**:**6** ca. 83:17) obtained in the deuterium-labeling study with PADI **9**

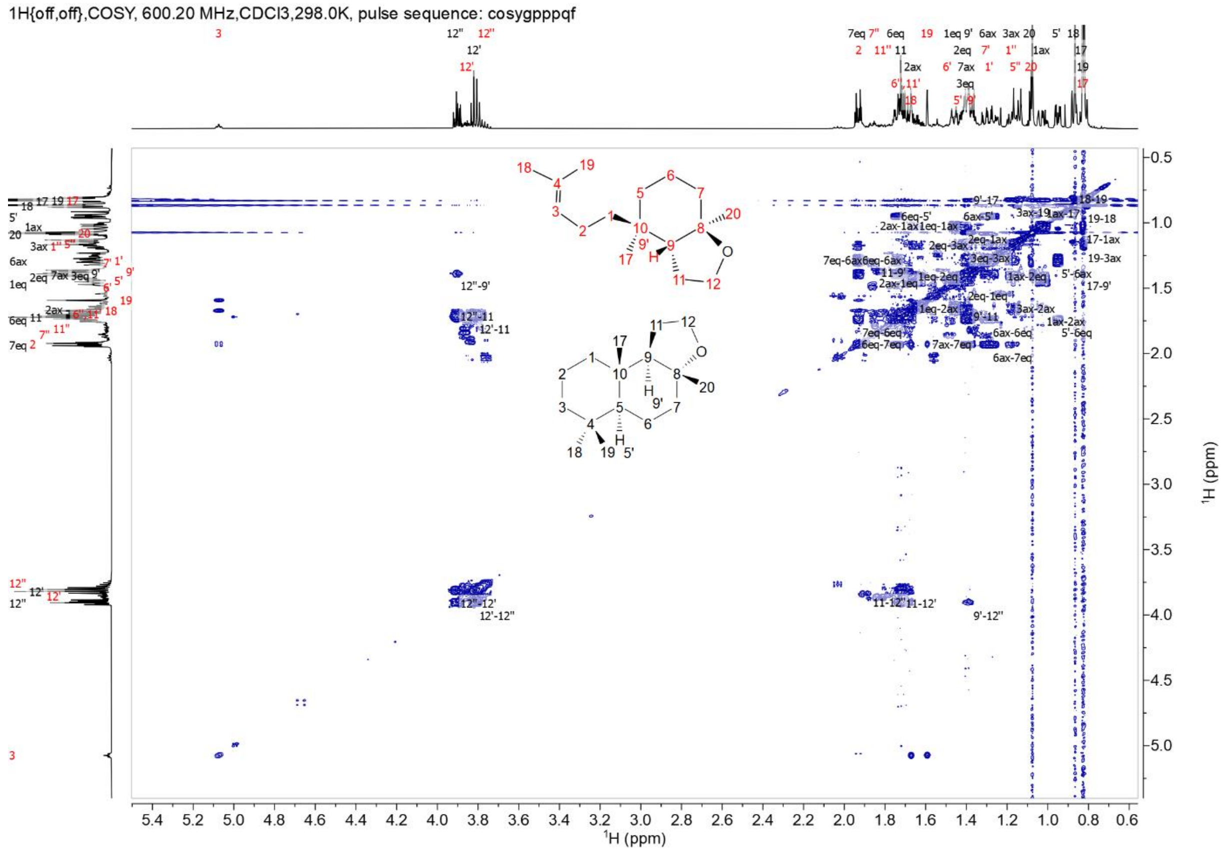

¹H,¹H-COSY (600 MHz, $CDCl_3$) spectrum of a mixture of (−)-ambrox **2a** and (−)-ambrox-d_1 **2a**-d_1 (ca. 60:40) and side product **6** (**2a**:**6** ca. 83:17) obtained in the deuterium-labeling study with PADI **9**

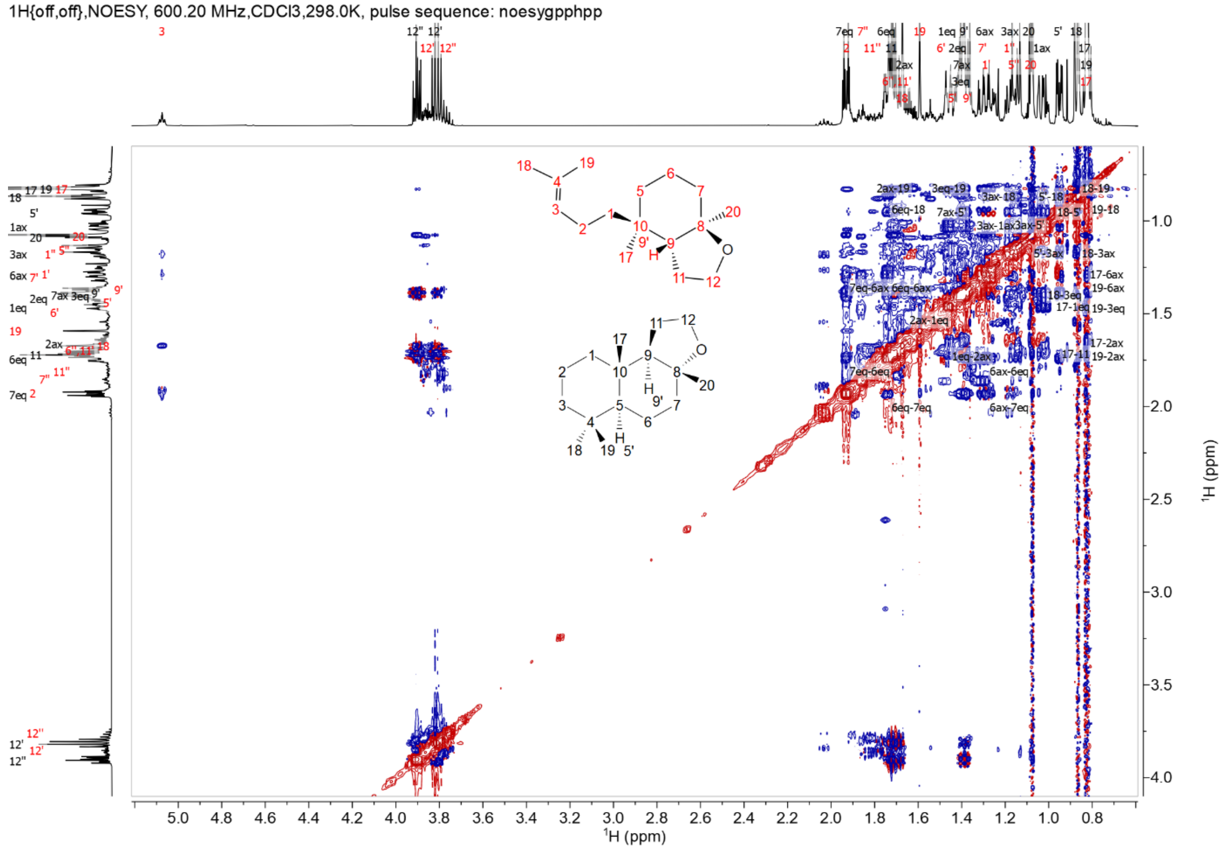

¹H,¹H-NOESY (600 MHz, $CDCl_3$) spectrum of a mixture of (−)-ambrox **2a** and (−)-ambrox-d_1 **2a**-d_1 (ca. 60:40) and side product **6** (**2a**:**6** ca. 83:17) obtained in the deuterium-labeling study with PADI **9**

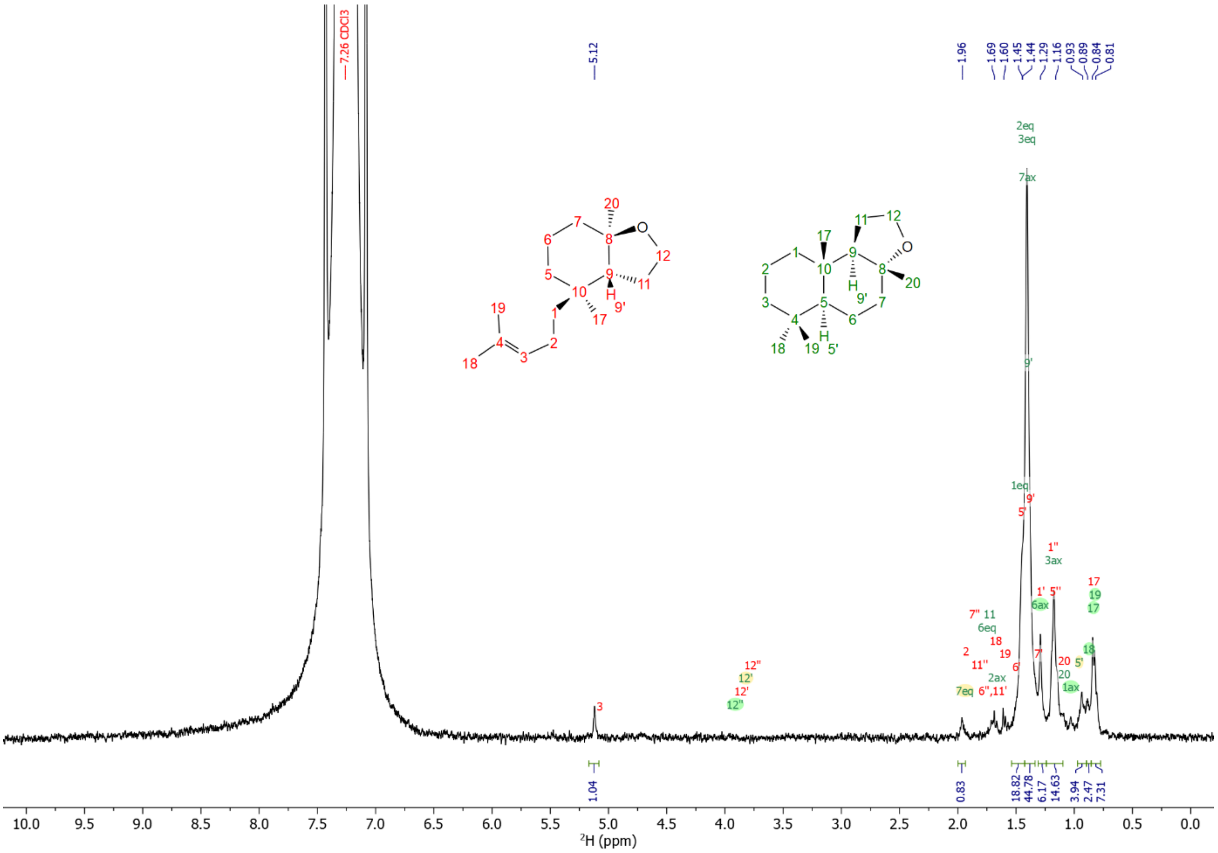

$^{2}H\{^{1}H\}$**-NMR** (92 MHz, $CDCl_3$) spectrum of a mixture of (−)-ambrox **2a** and (−)-ambrox-d_1 **2a**-d_1 (ca. 60:40) and side product **6** (**2a**:**6** ca. 83:17) obtained in the deuterium-labeling study with PADI **9**

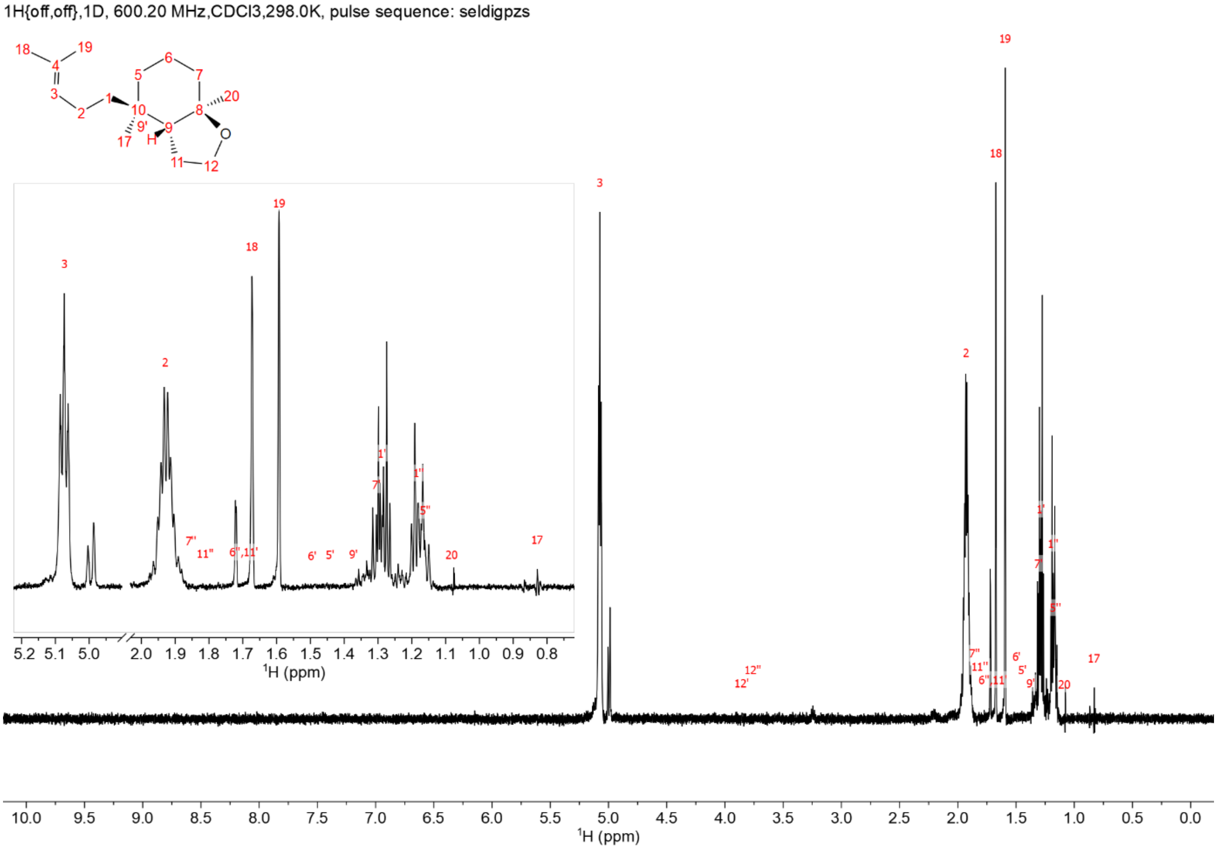

1D TOCSY (600 MHz, $CDCl_3$) spectrum (after irradiation at H3) of a mixture of (−)-ambrox **2a** and (−)-ambrox-d_1 **2a**-d_1 (ca. 60:40) and side product **6** (**2a**:**6** ca. 83:17) obtained in the deuterium-labeling study with PADI **9**

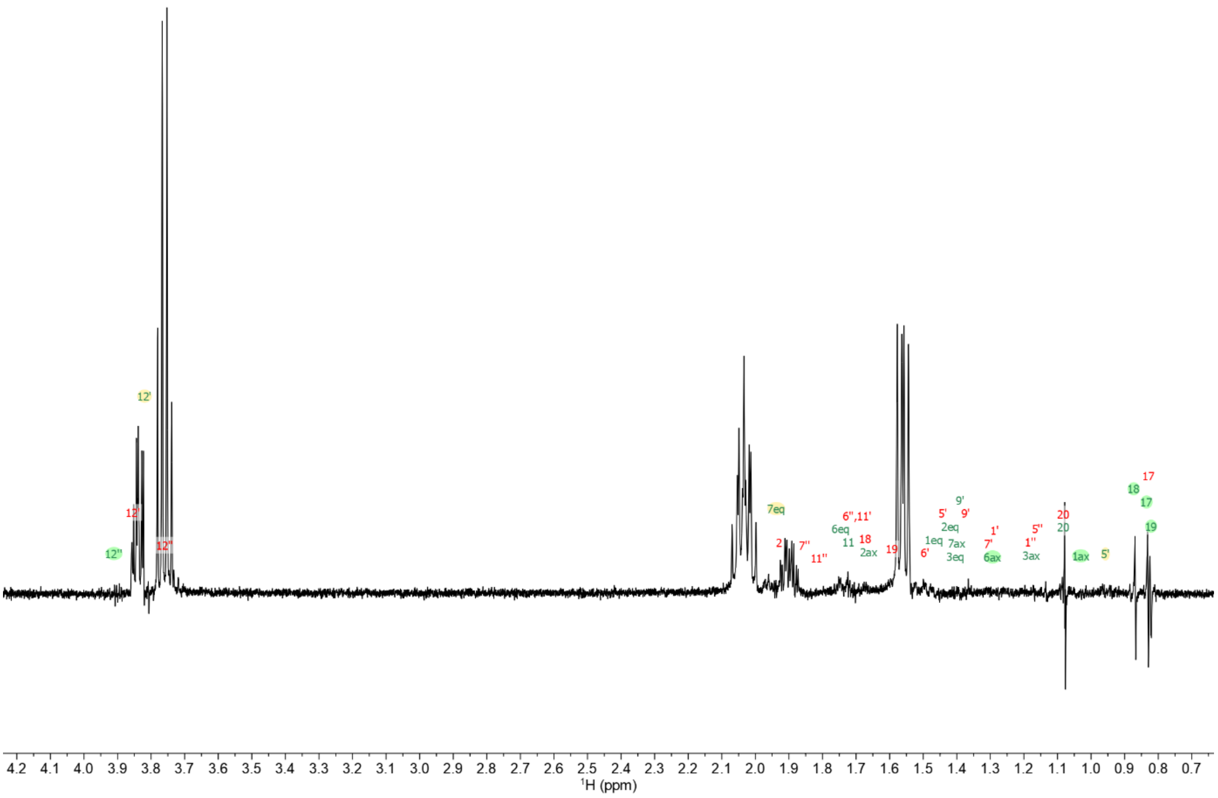

1D TOCSY (600 MHz, $CDCl_3$) spectrum (after irradiation at H12’) of a mixture of (−)-ambrox **2a** and (−)-ambrox-d_1 **2a**-d_1 (ca. 60:40) and side product **6** (**2a**:**6** ca. 83:17) obtained in the deuterium-labeling study with PADI **9** showing the presence of 9-*epi*-ambrox as minor diastereomer

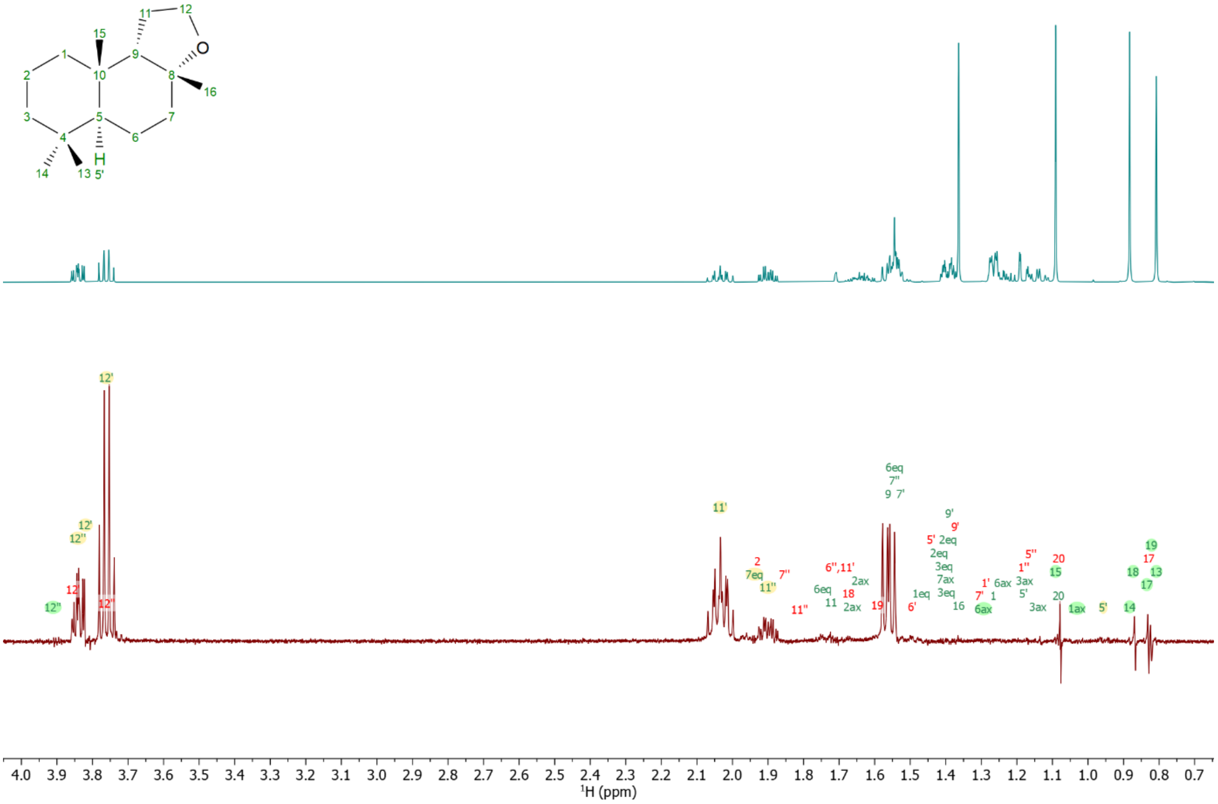

Comparison of the **^{1}H NMR** (600 MHz, $CDCl_3$, top, green) spectrum of (−)-9-*epi*-ambrox with the **1D TOCSY** (600 MHz, $CDCl_3$, bottom, red) spectrum

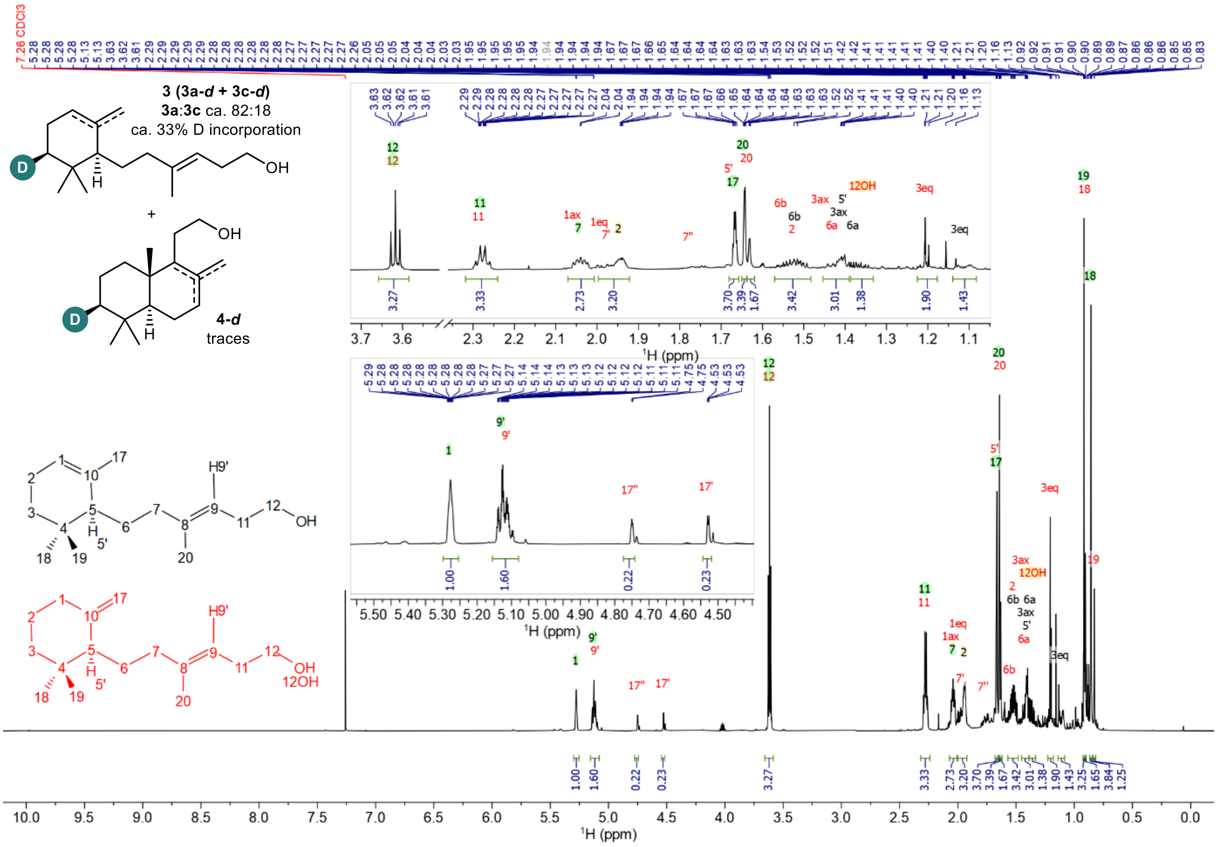

^{1}H NMR (600 MHz, $CDCl_3$) spectrum of a mixture of cyclohomofarnesols **3a** and **3c** (**3a**:**3c** ca. 82:18, ca. 33% D at C3ax) obtained in the deuterium-labeling study with IDPi **8g** after preparative HPLC

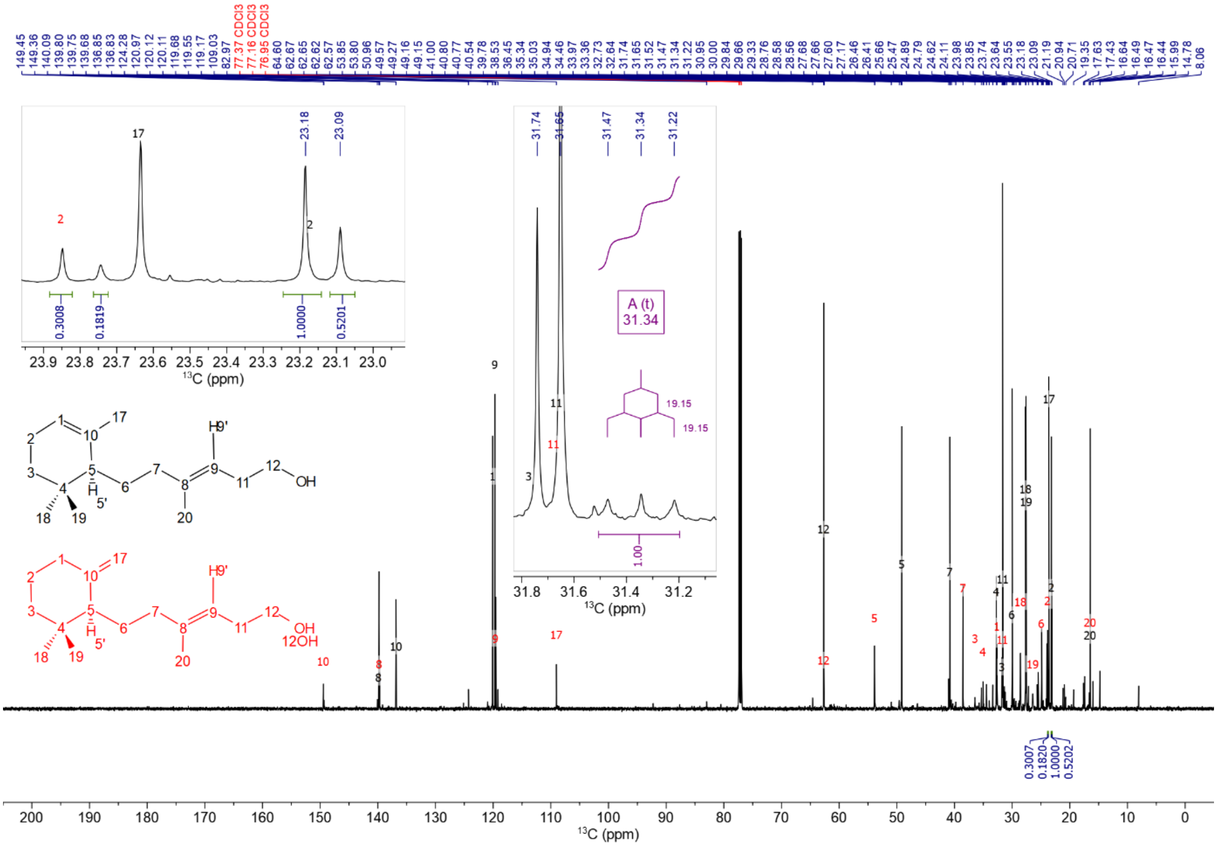

^{13}C NMR (151 MHz, $CDCl_3$) spectrum of a mixture of cyclohomofarnesols **3a** and **3c** (**3a**:**3c** ca. 82:18, ca. 33% D at C3ax) obtained in the deuterium-labeling study with IDPi **8g** after preparative HPLC

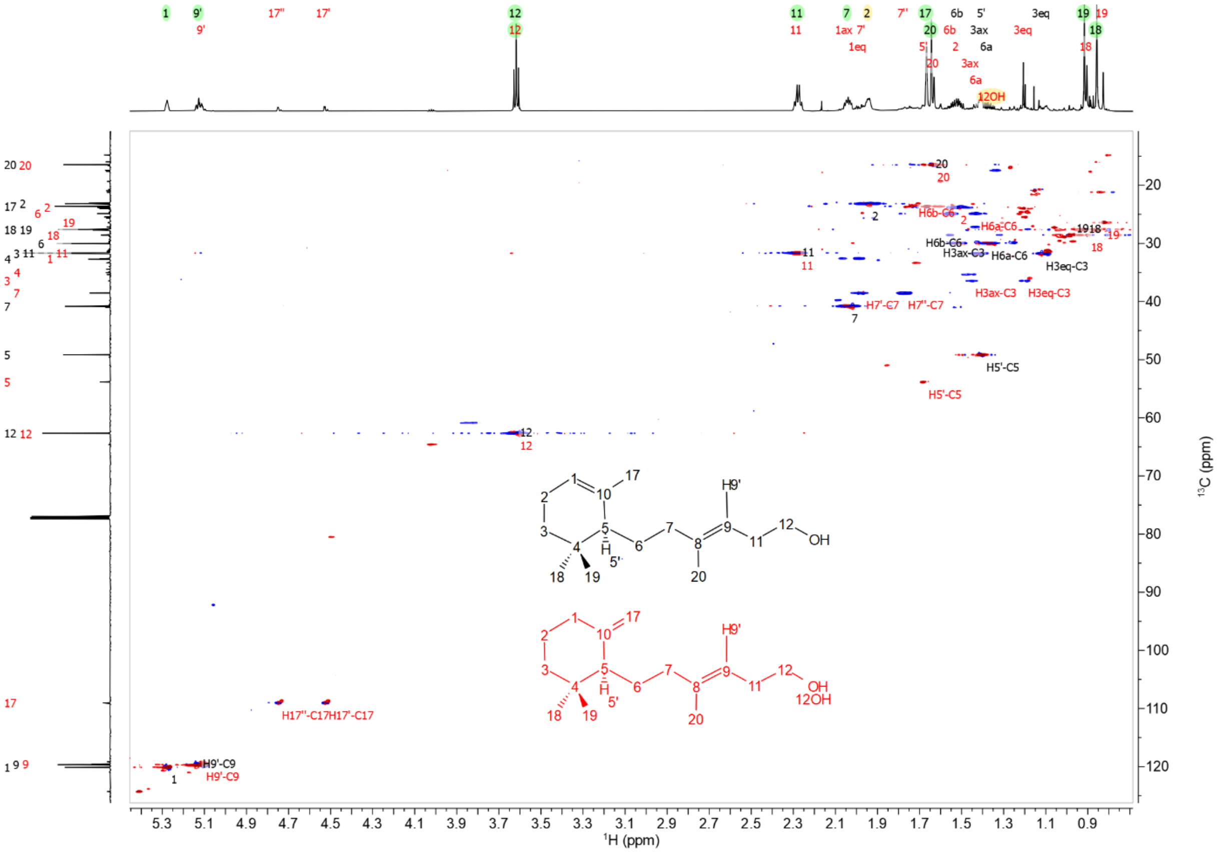

Multiplicity-edited **¹H,¹³C-HSQC** (600 MHz, 151 MHz, $CDCl_3$) spectrum of a mixture of cyclohomofarnesols **3a** and **3c** (**3a**:**3c** ca. 82:18, ca. 33% D at C3ax) obtained in the deuterium-labeling study with IDPi **8g** after preparative HPLC

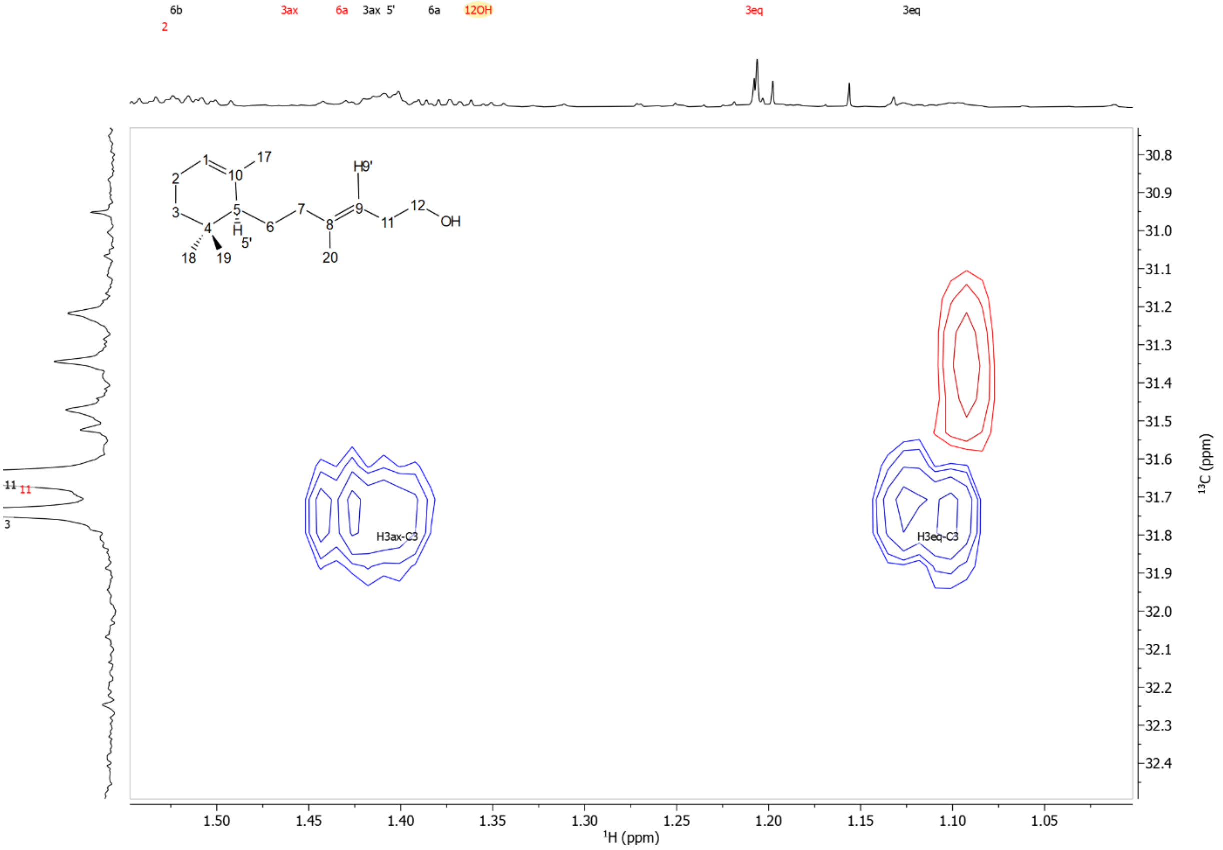

Excerpt of the multiplicity-edited **¹H,¹³C-HSQC** (600 MHz, 151 MHz, $CDCl_3$) spectrum showing **3a** and **3c**-d_1

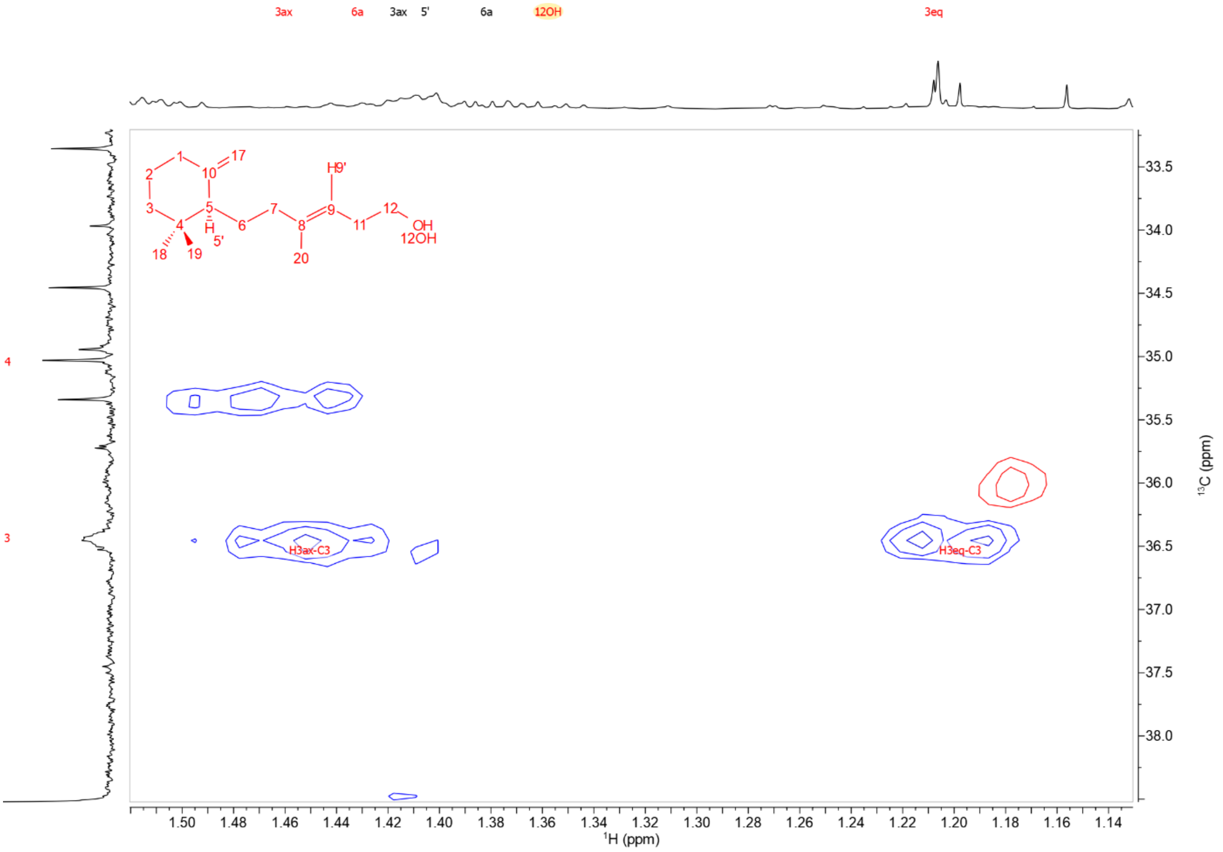

Excerpt of the multiplicity-edited $^{1}H,^{13}C$-**HSQC** (600 MHz, 151 MHz, $CDCl_3$) spectrum showing **3c** and **3c**-d_1

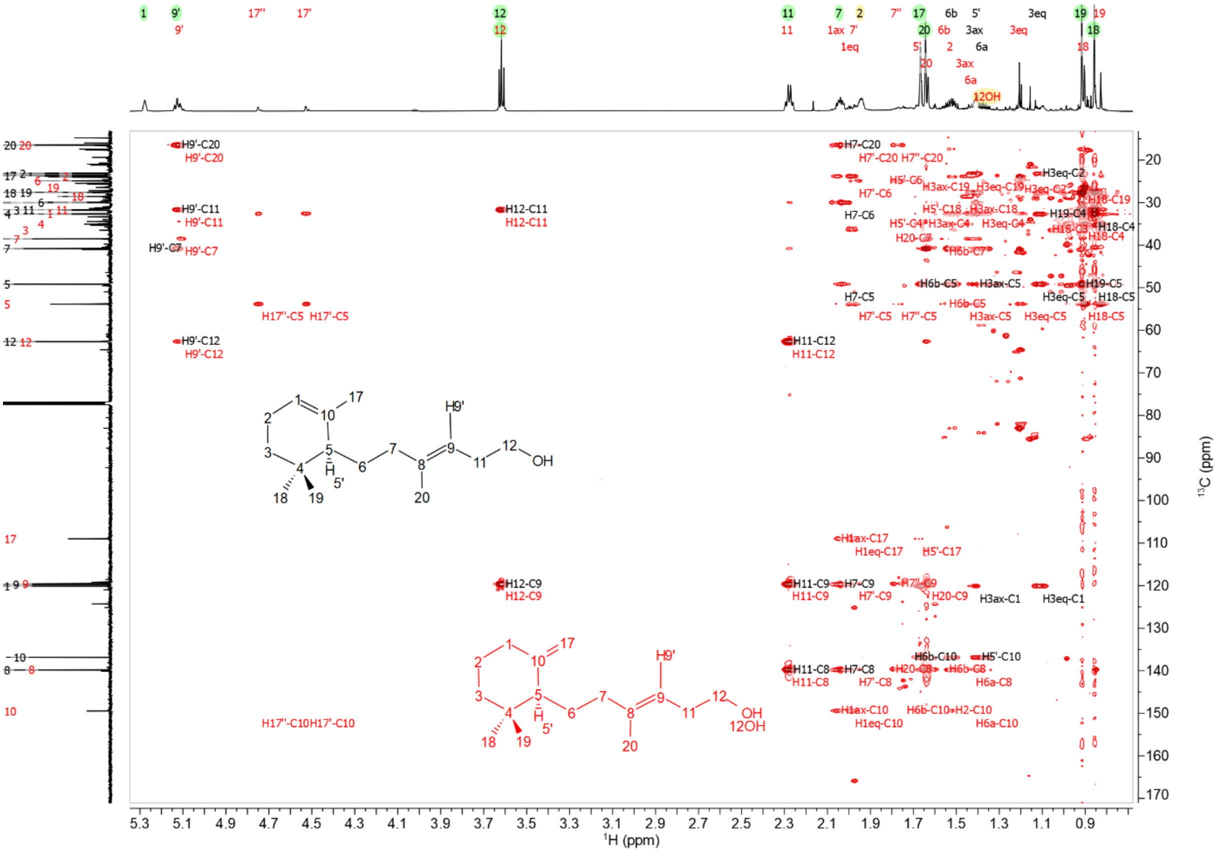

$^{1}H,^{13}C$-HMBC (600 MHz, 151 MHz, $CDCl_3$) spectrum of a mixture of cyclohomofarnesols **3a** and **3c** (**3a**:**3c** ca. 82:18, ca. 33% D at C3ax) obtained in the deuterium-labeling study with IDPi **8g** after preparative HPLC

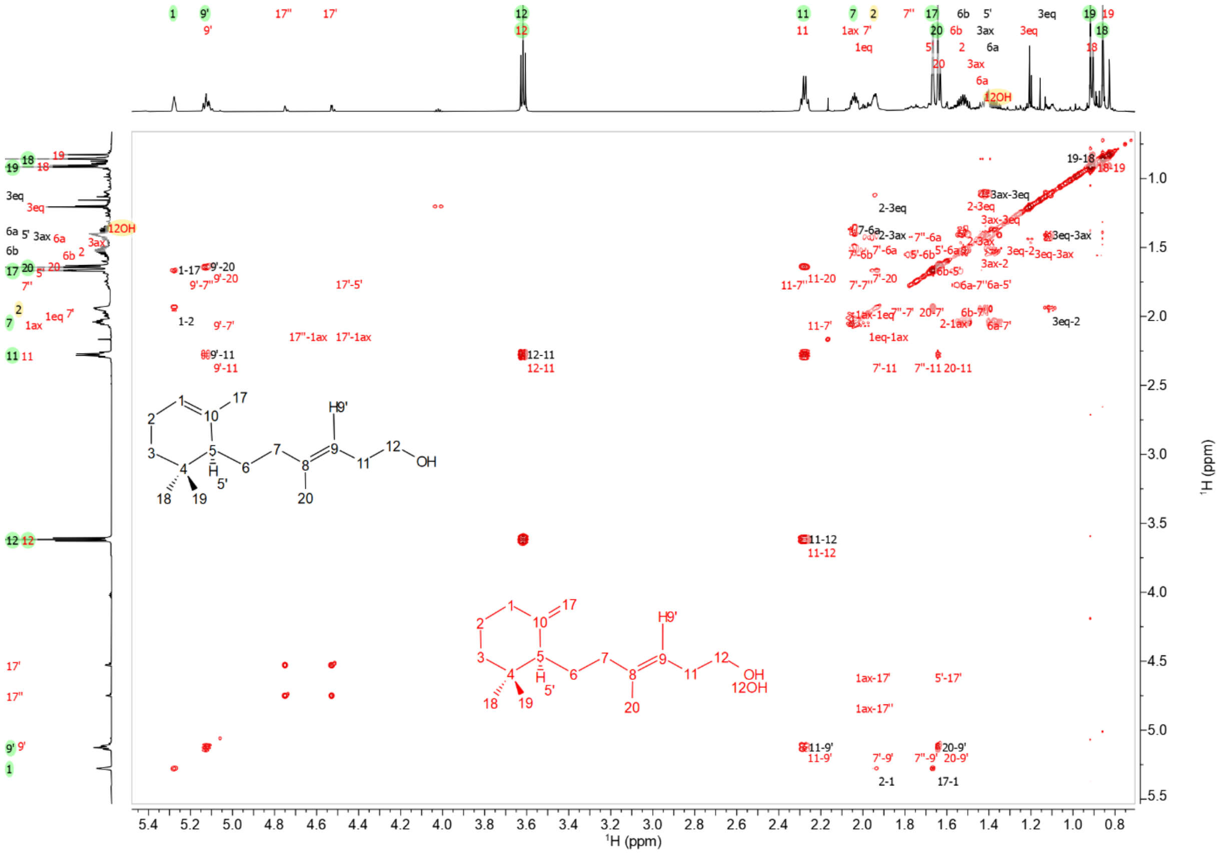

¹H,¹H-COSY (600 MHz, $CDCl_3$) spectrum of a mixture of cyclohomofarnesols **3a** and **3c** (**3a**:**3c** ca. 82:18, ca. 33% D at C3ax) obtained in the deuterium-labeling study with IDPi **8g** after preparative HPLC

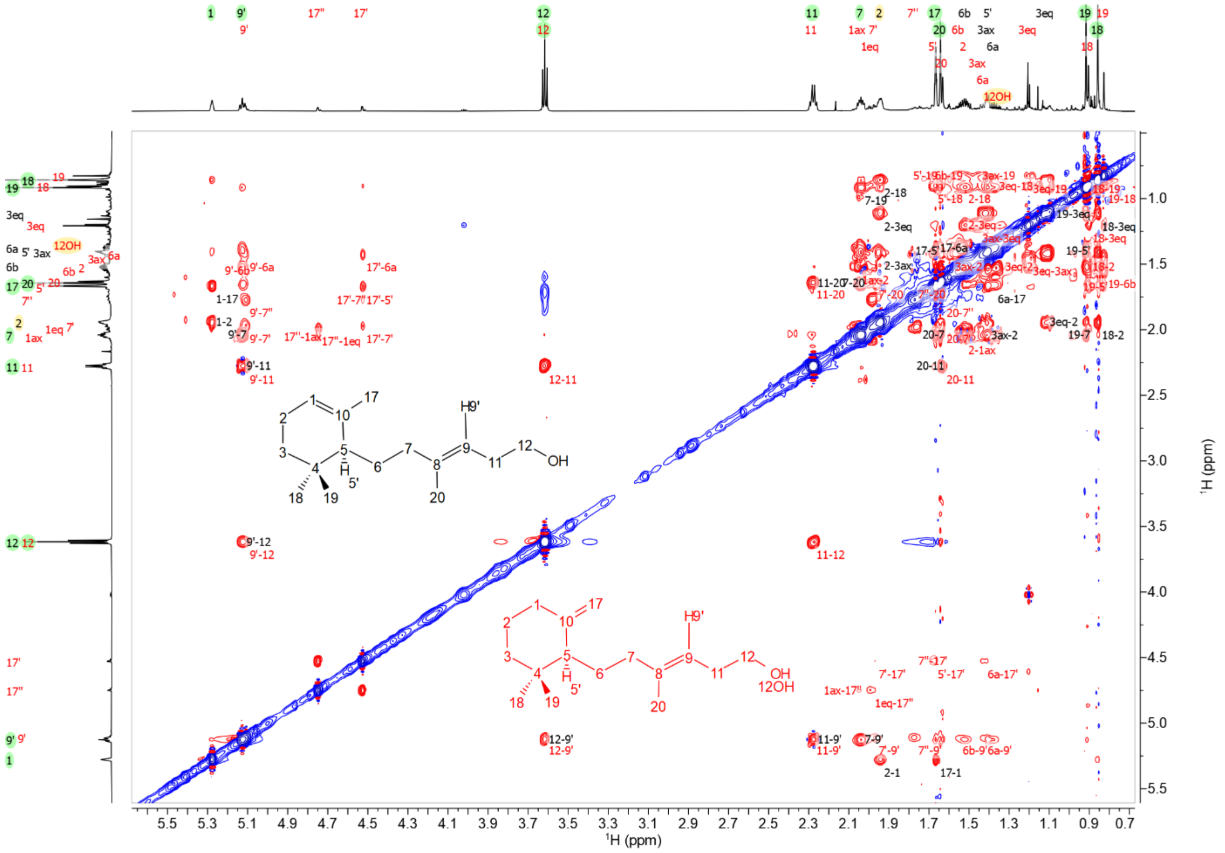

¹H,¹H-NOESY (600 MHz, $CDCl_3$) spectrum of a mixture of cyclohomofarnesols **3a** and **3c** (**3a**:**3c** ca. 82:18, ca. 33% D at C3ax) obtained in the deuterium-labeling study with IDPi **8g** after preparative HPLC

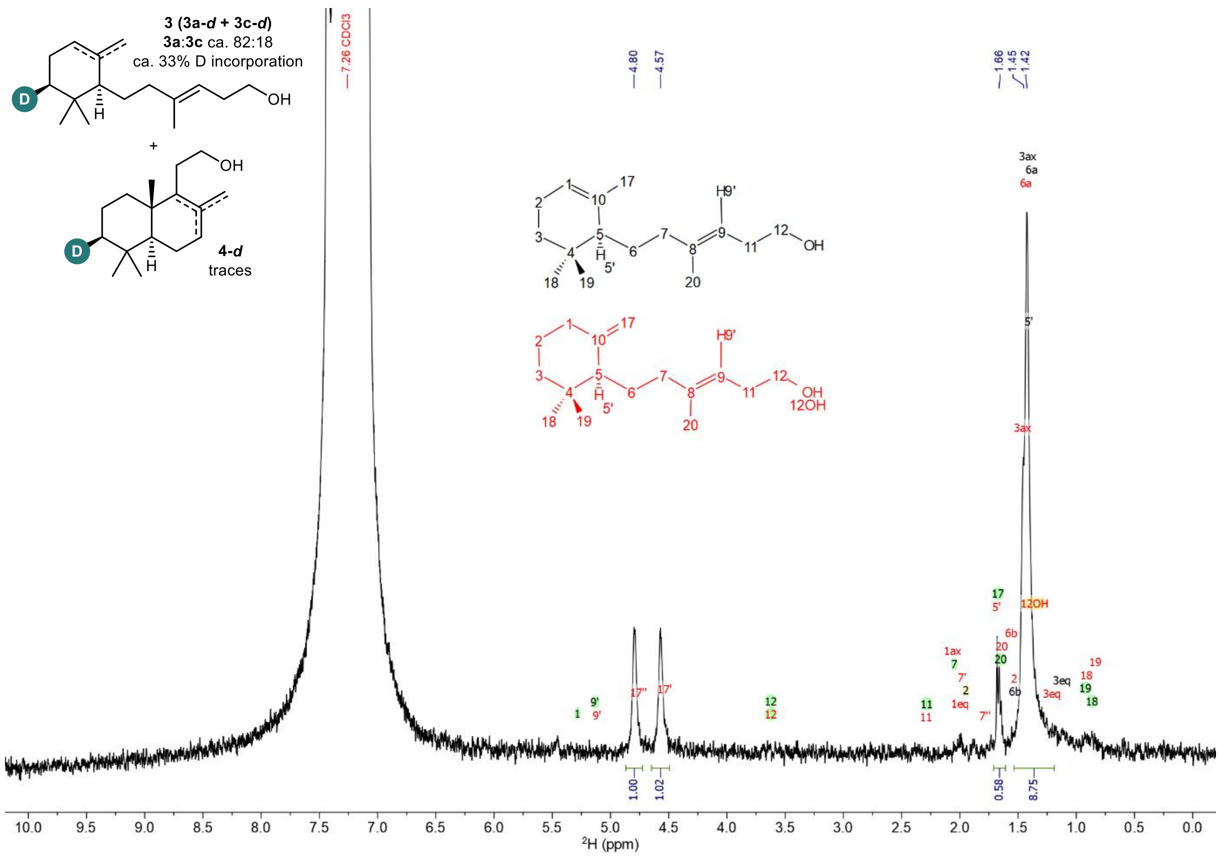

$^{2}H\{^{1}H\}$-NMR (92 MHz, $CDCl_3$) spectrum of a mixture of cyclohomofarnesols **3a** and **3c** (**3a**:**3c** ca. 82:18, ca. 33% D at C3ax) obtained in the deuterium-labeling study with IDPi **8g** after preparative HPLC

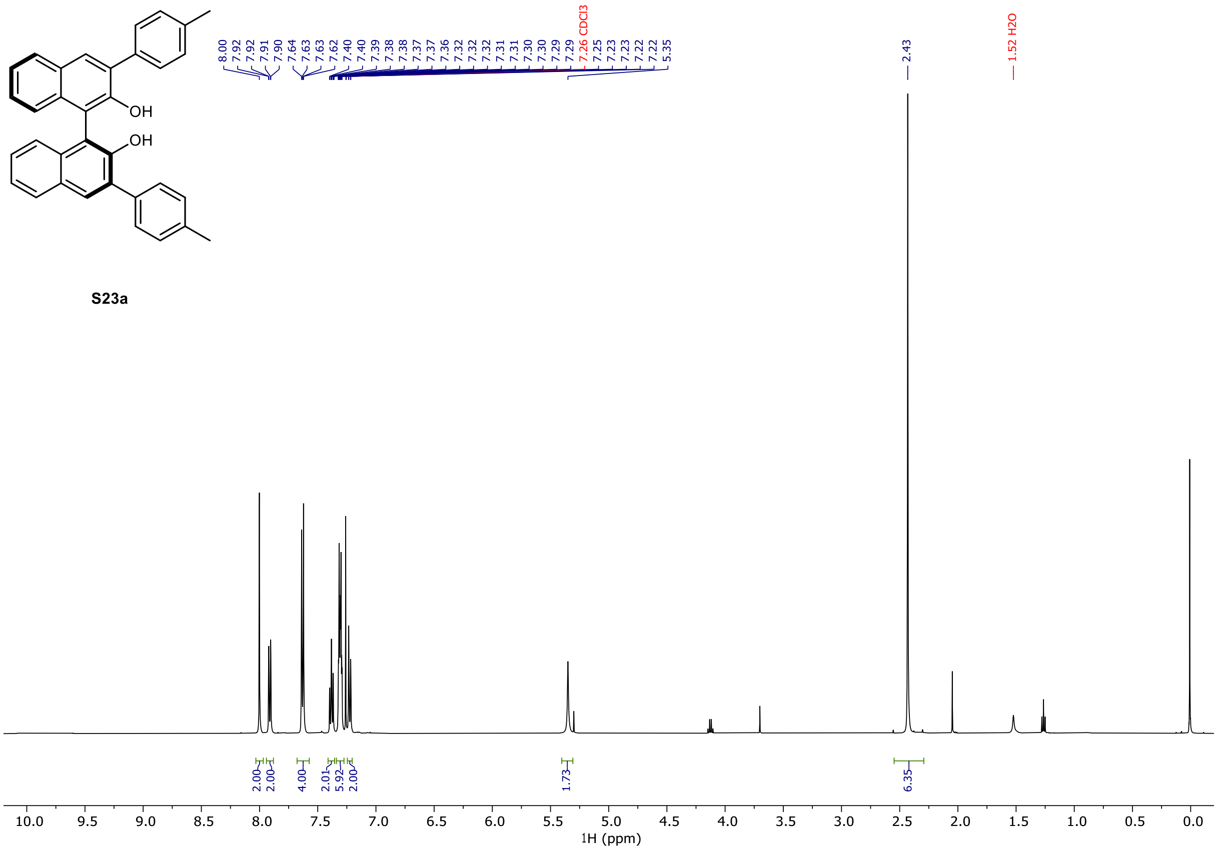

^{1}H NMR (501 MHz, $CDCl_3$) spectrum of diol **S23a**

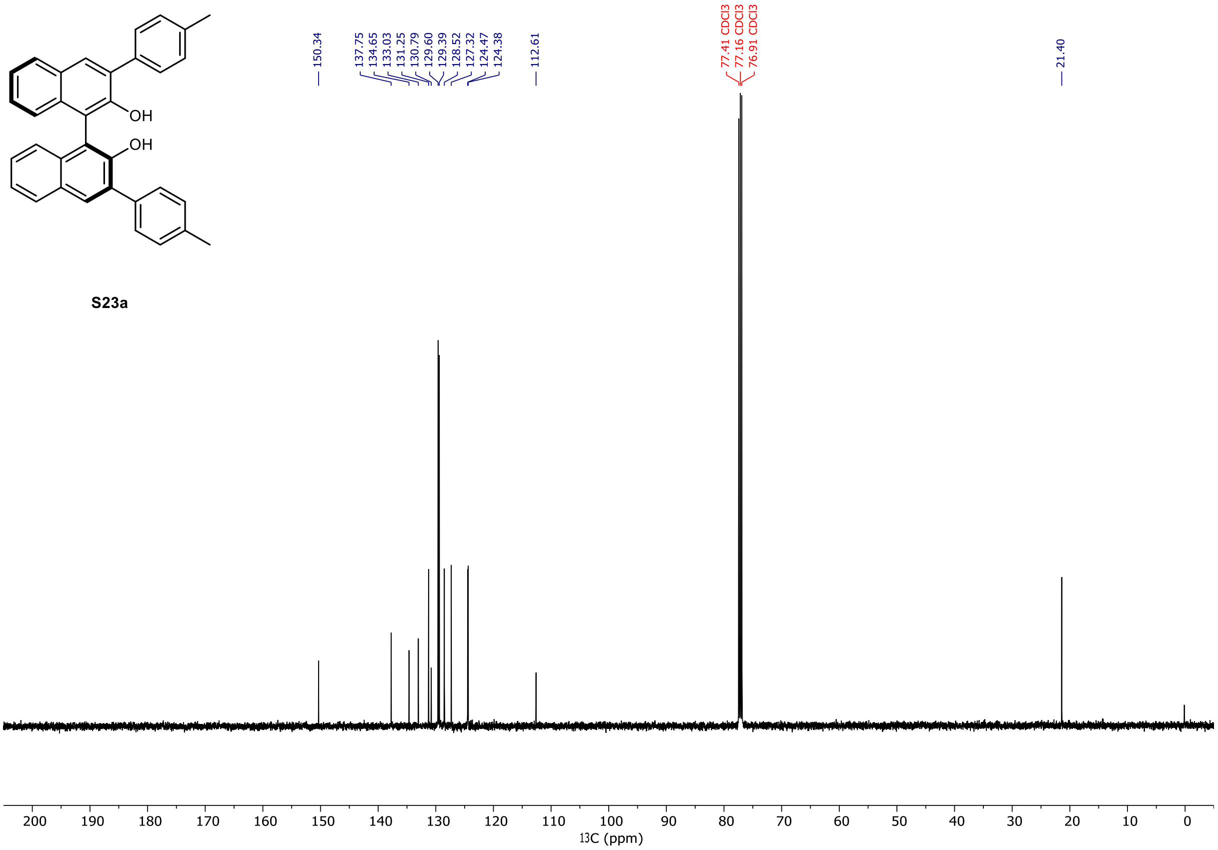

^{13}C NMR (126 MHz, $CDCl_3$) spectrum of diol **S23a**

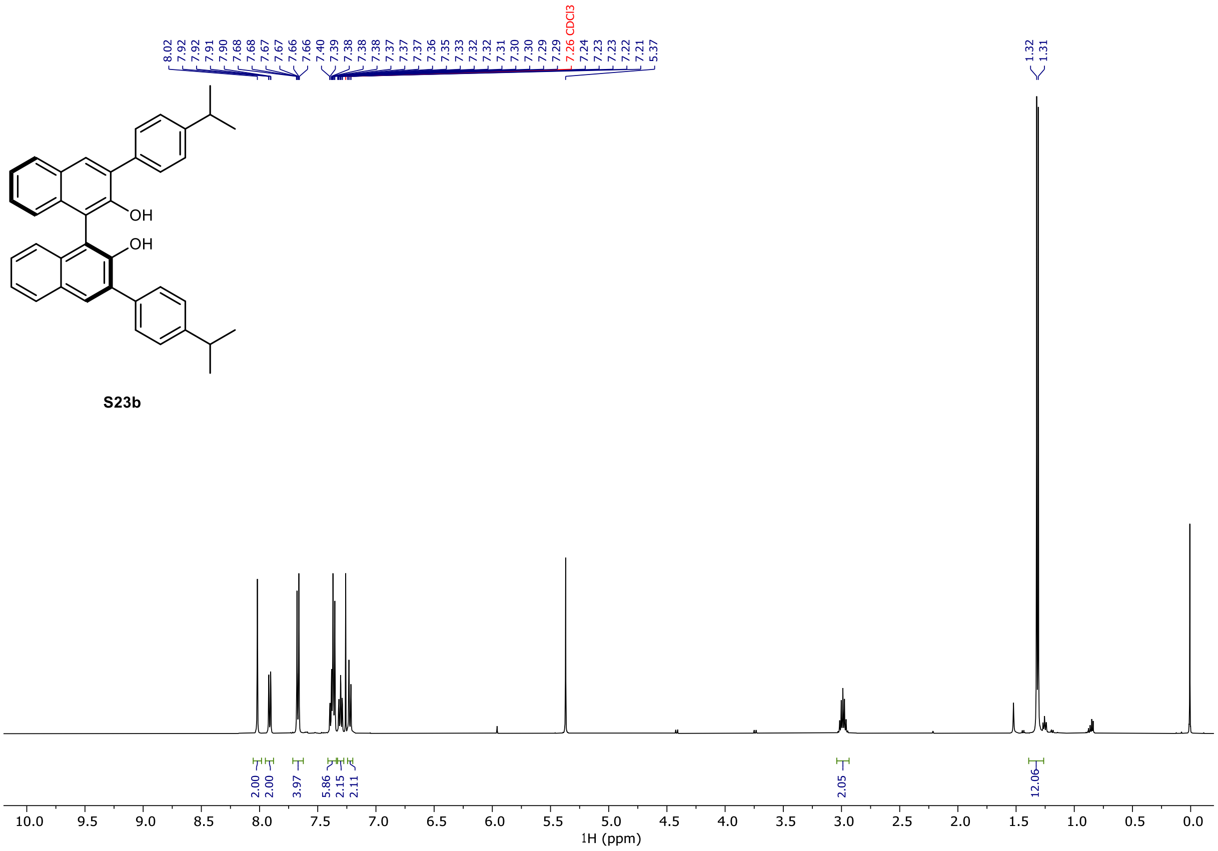

^{1}H NMR (501 MHz, $CDCl_3$) spectrum of diol **S23b**

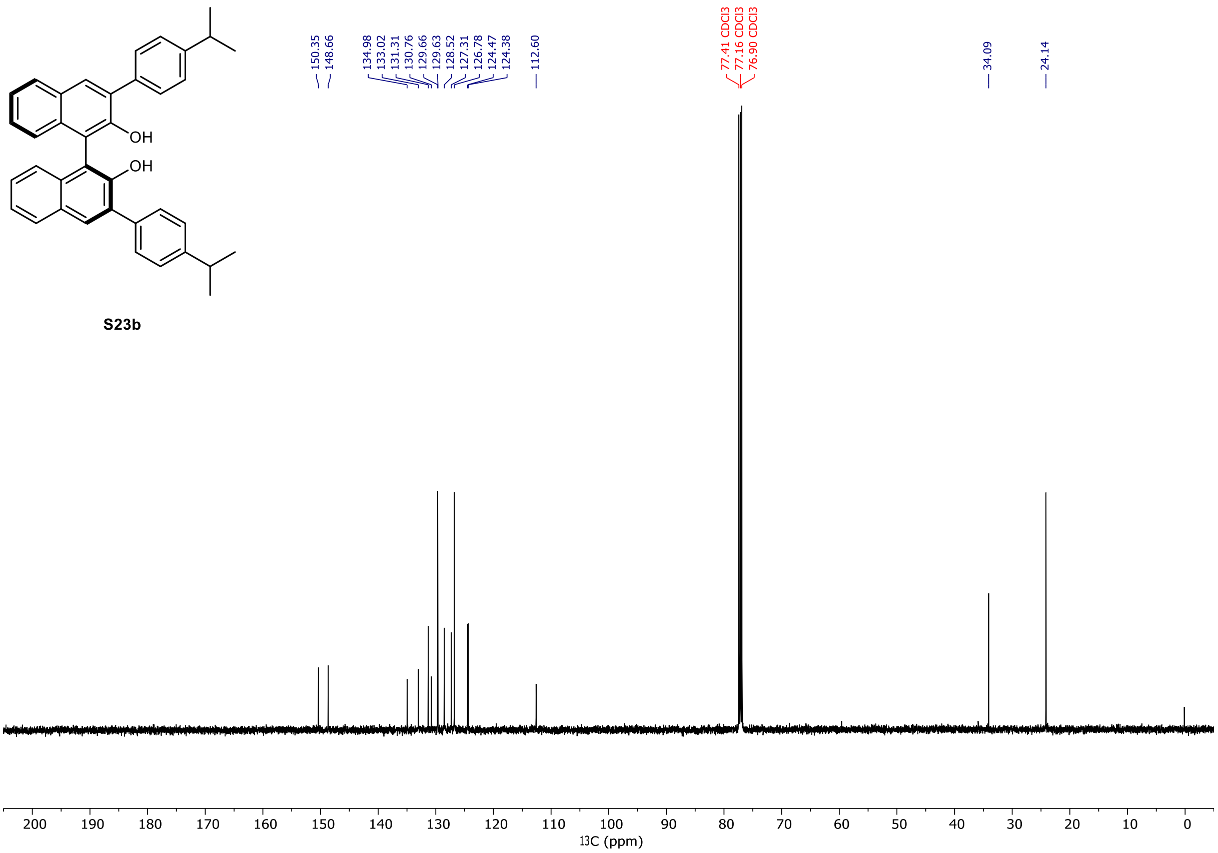

^{13}C NMR (126 MHz, $CDCl_3$) spectrum of diol **S23b**

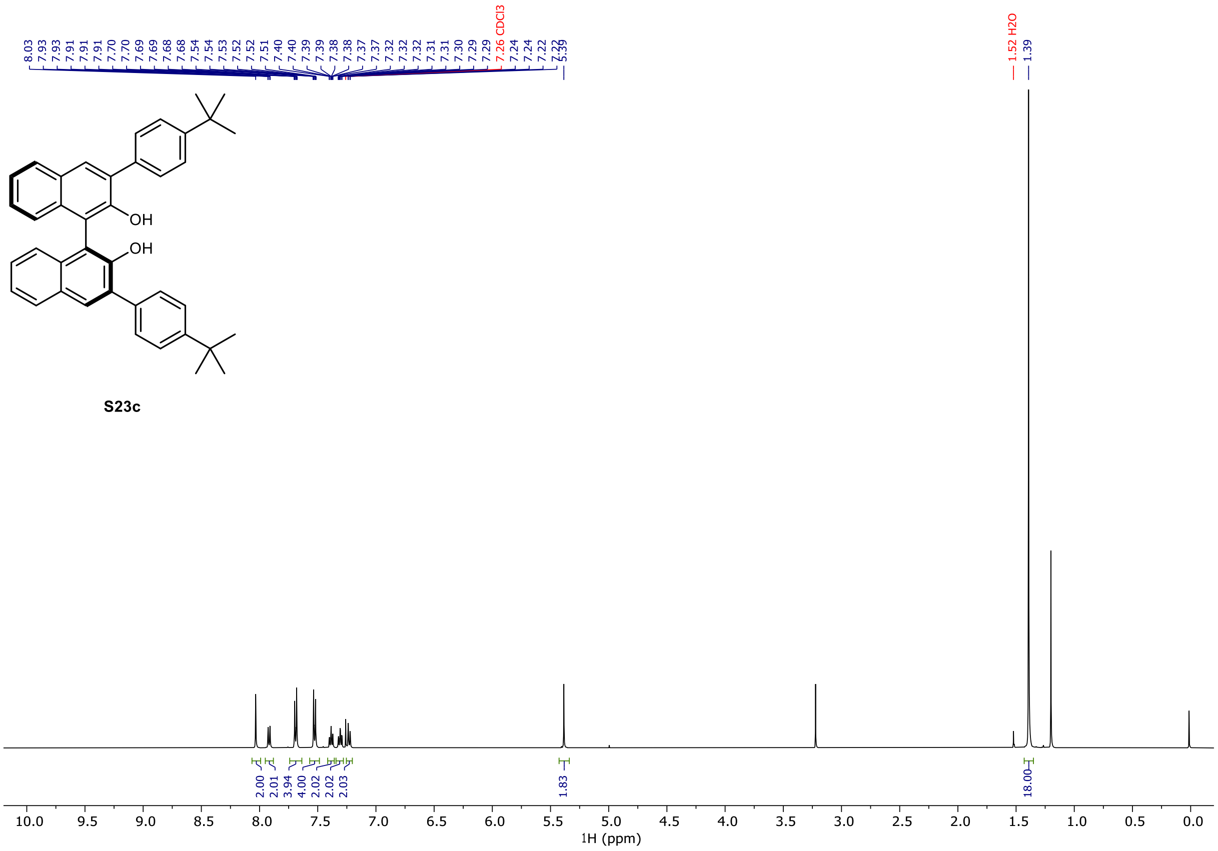

^{1}H NMR (501 MHz, $CDCl_3$) spectrum of diol **S23c**

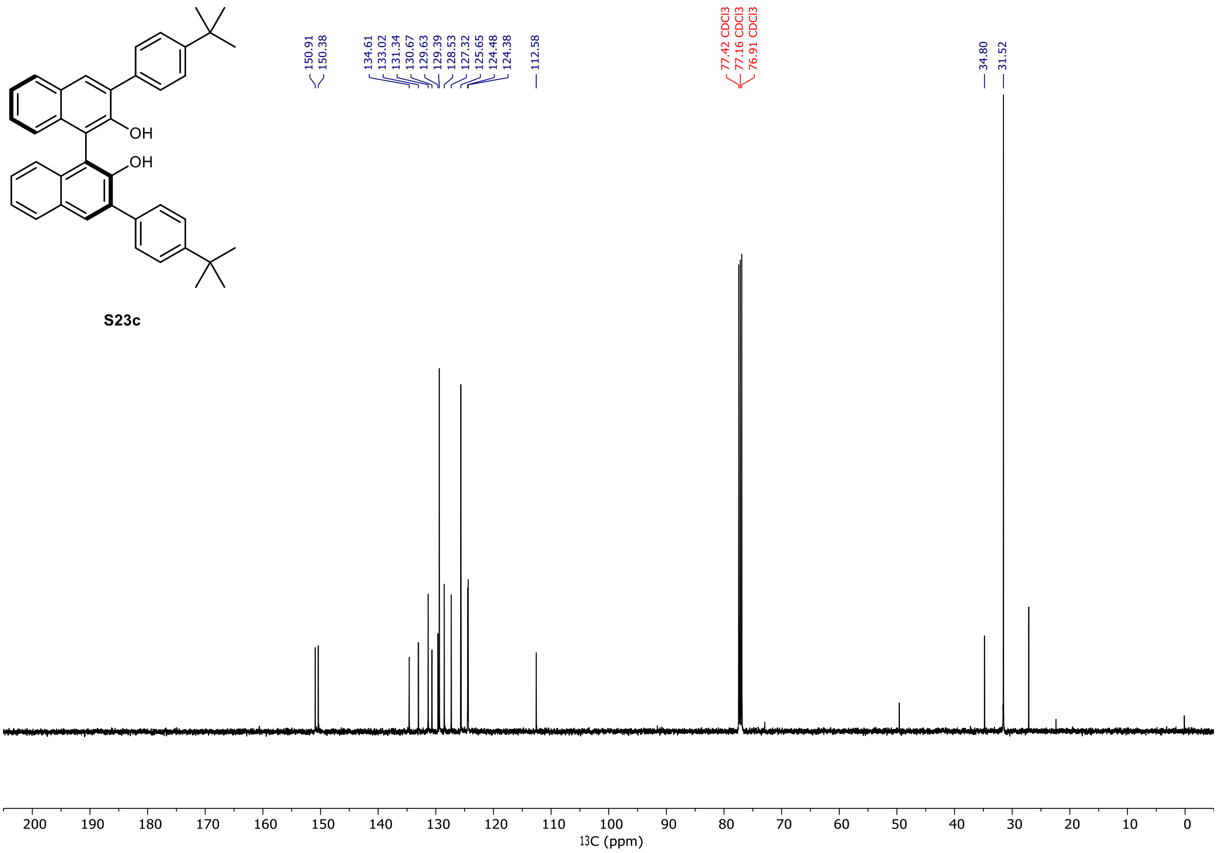

^{13}C NMR (126 MHz, $CDCl_3$) spectrum of diol **S23c**

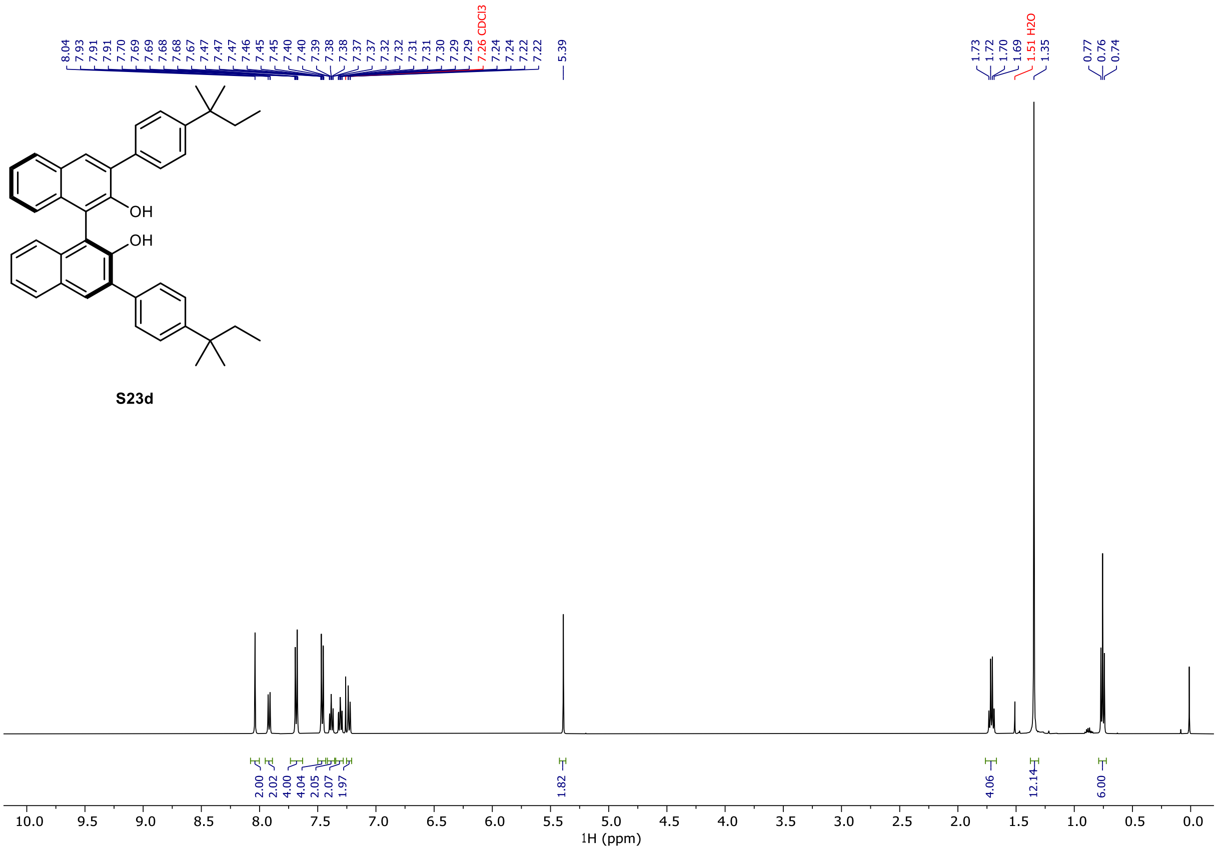

^{1}H NMR (501 MHz, $CDCl_3$) spectrum of diol **S23d**

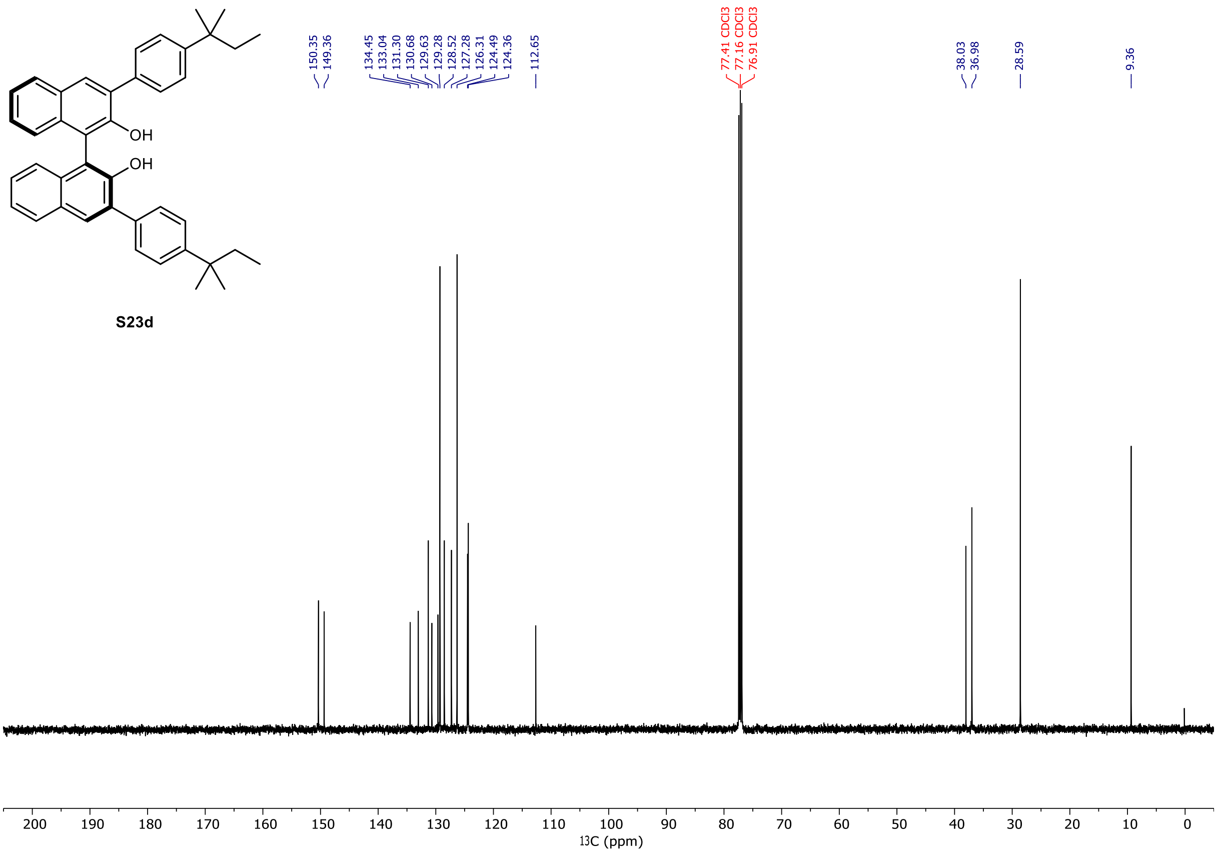

^{13}C NMR (126 MHz, $CDCl_3$) spectrum of diol **S23d**

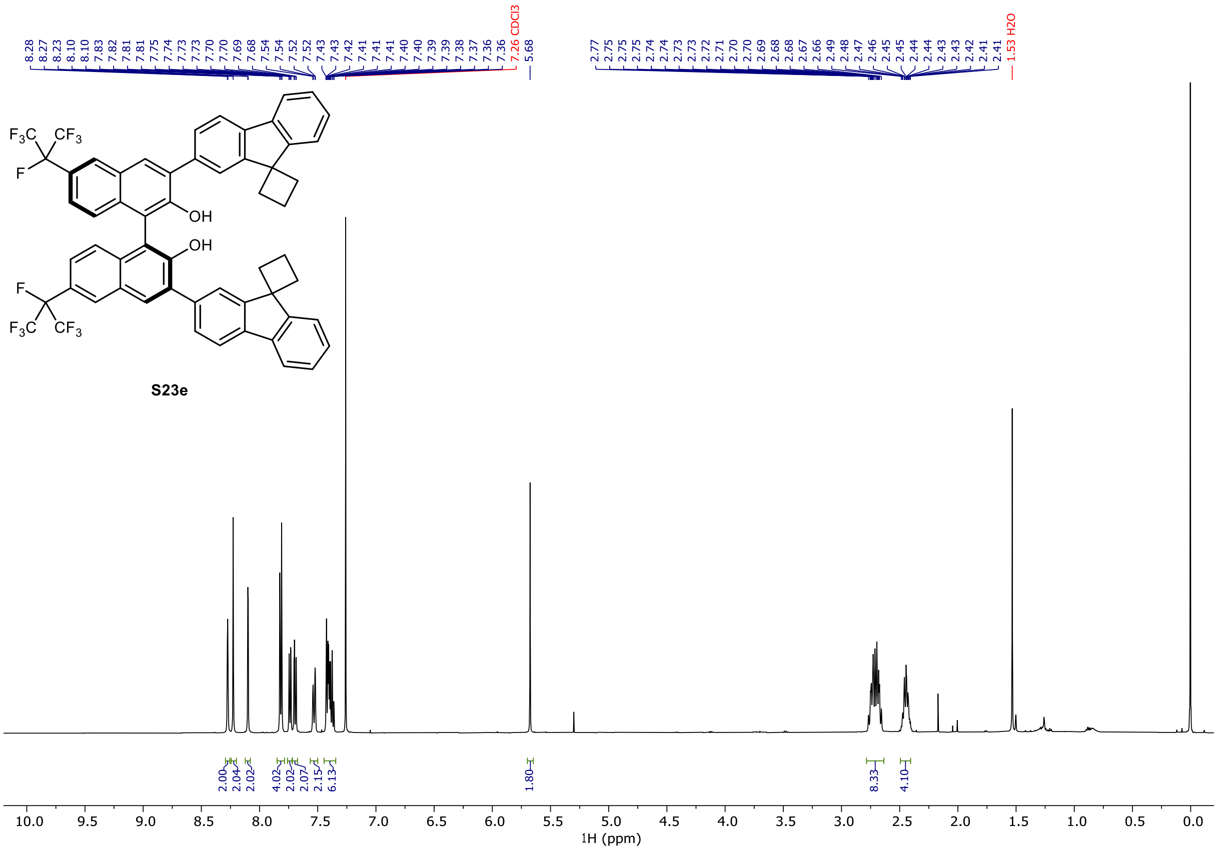

^{1}H NMR (501 MHz, $CDCl_3$) spectrum of diol **S23e**

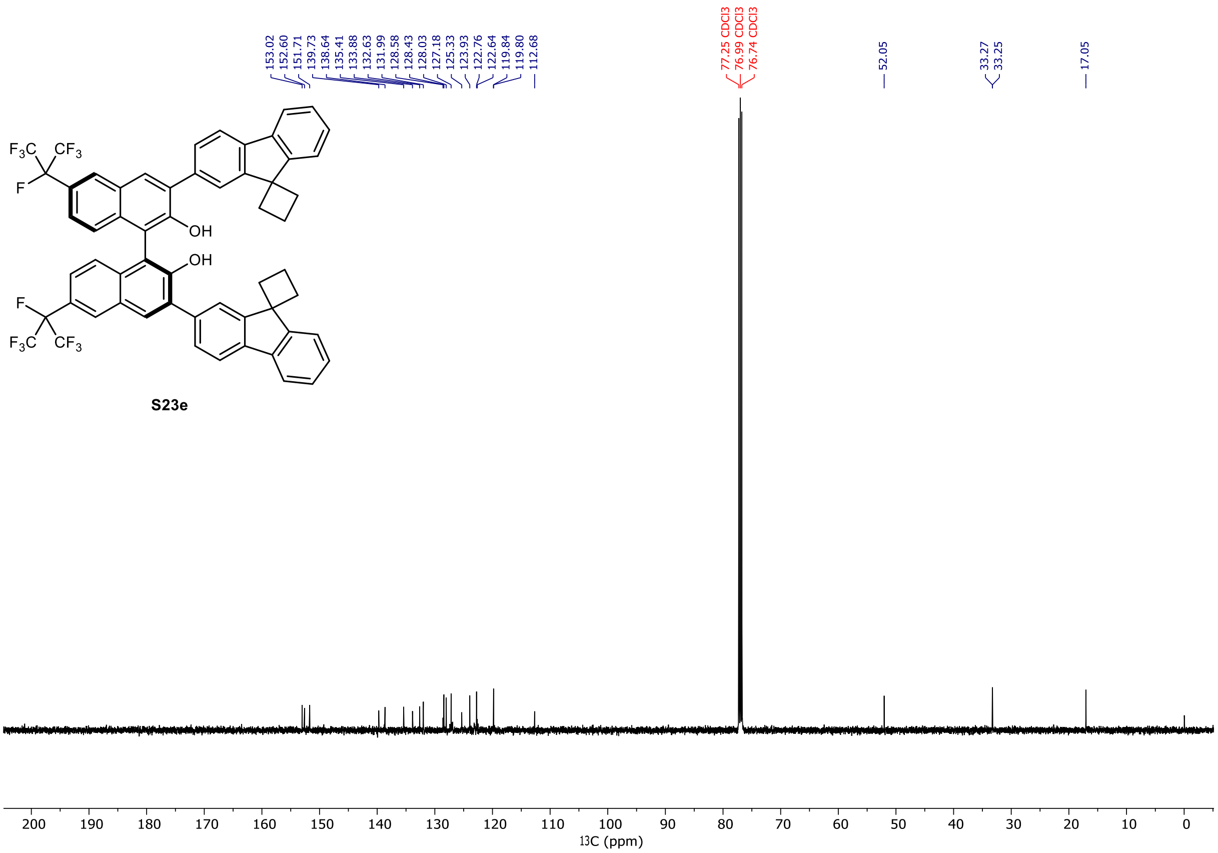

^{13}C NMR (126 MHz, $CDCl_3$) spectrum of diol **S23e**

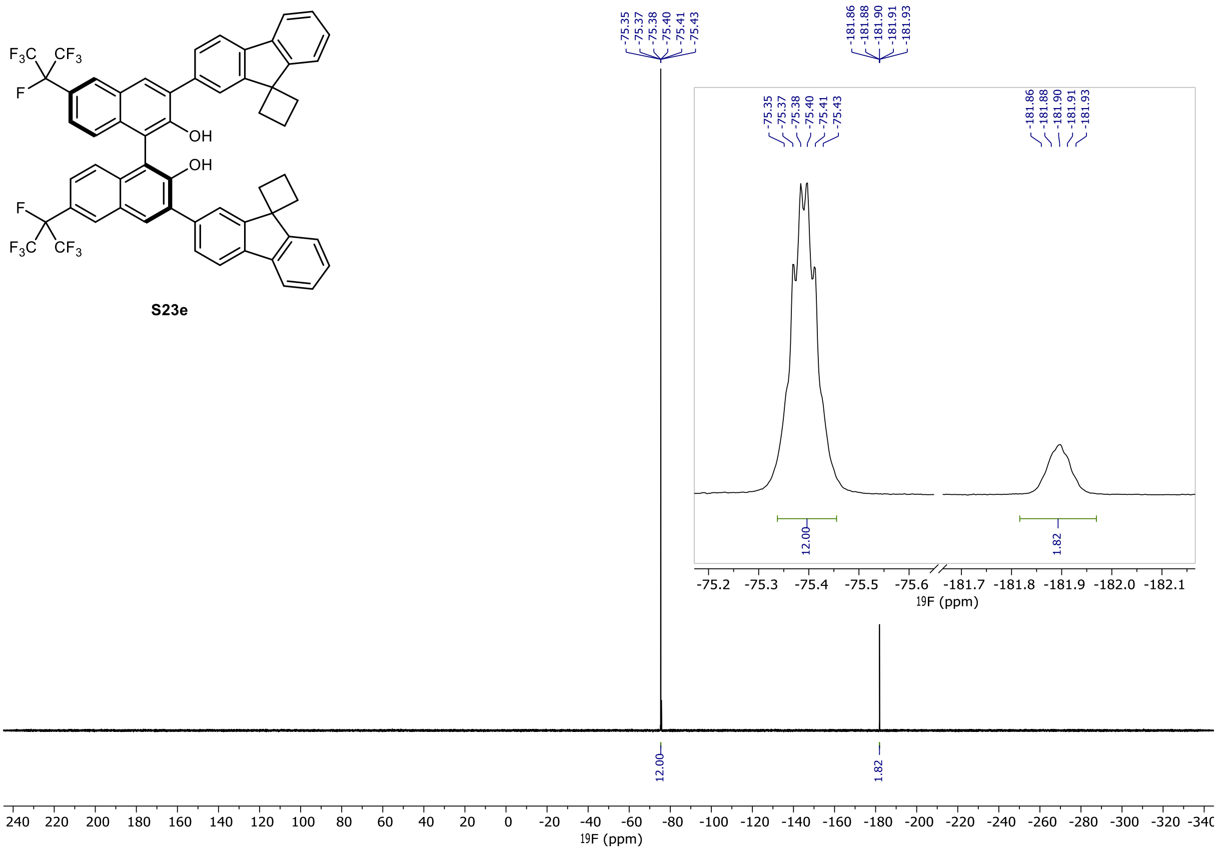

S23e

^{19}F NMR (471 MHz, $CDCl_3$) spectrum of diol **S23e**

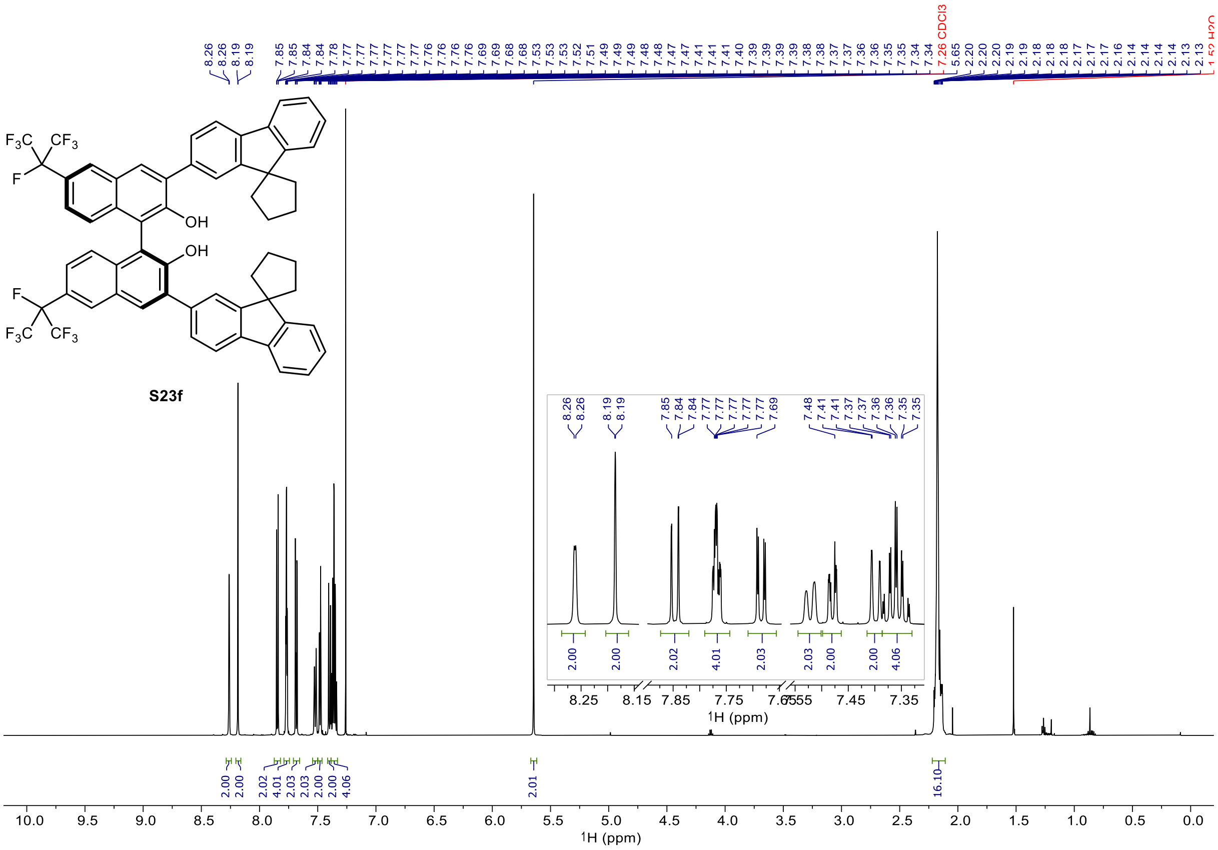

S23f

^{1}H NMR (600 MHz, $CDCl_3$) spectrum of diol **S23f**

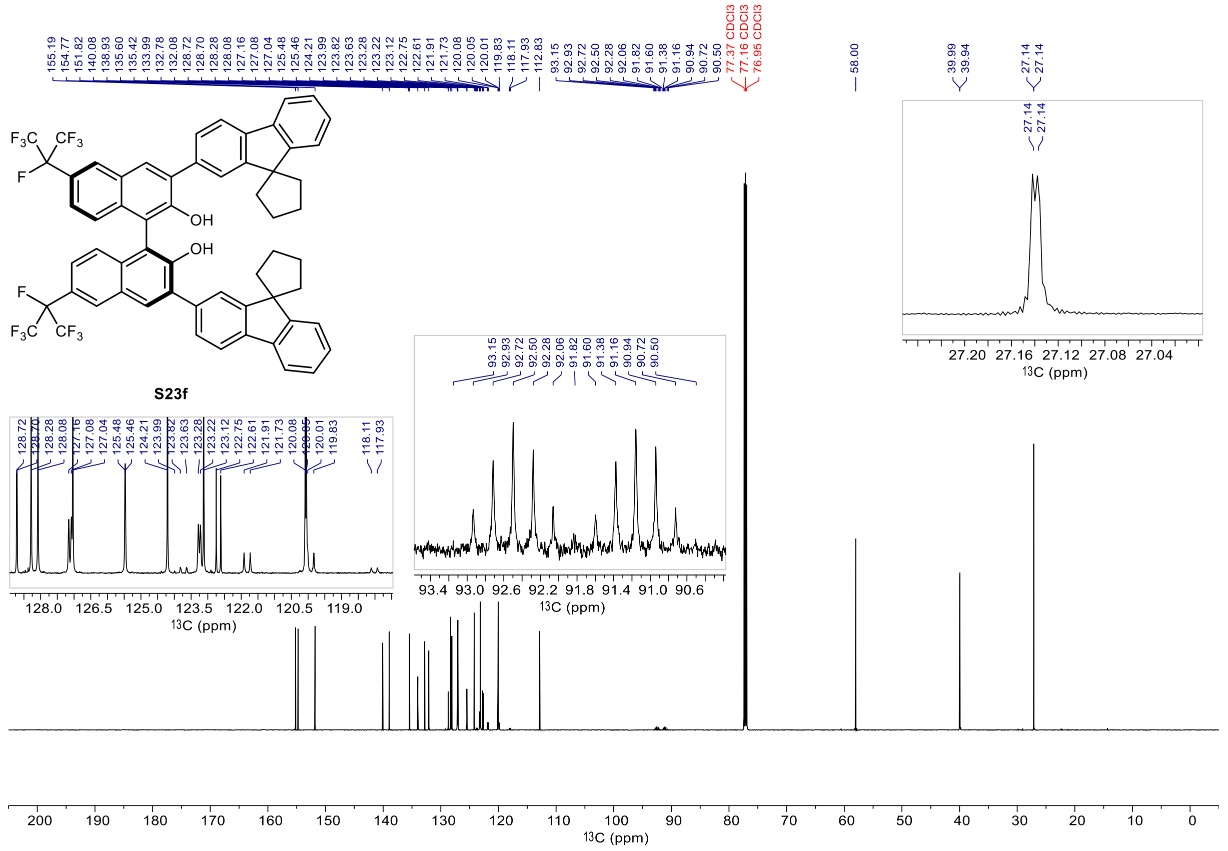

^{13}C NMR (151 MHz, $CDCl_3$) spectrum of diol **S23f**

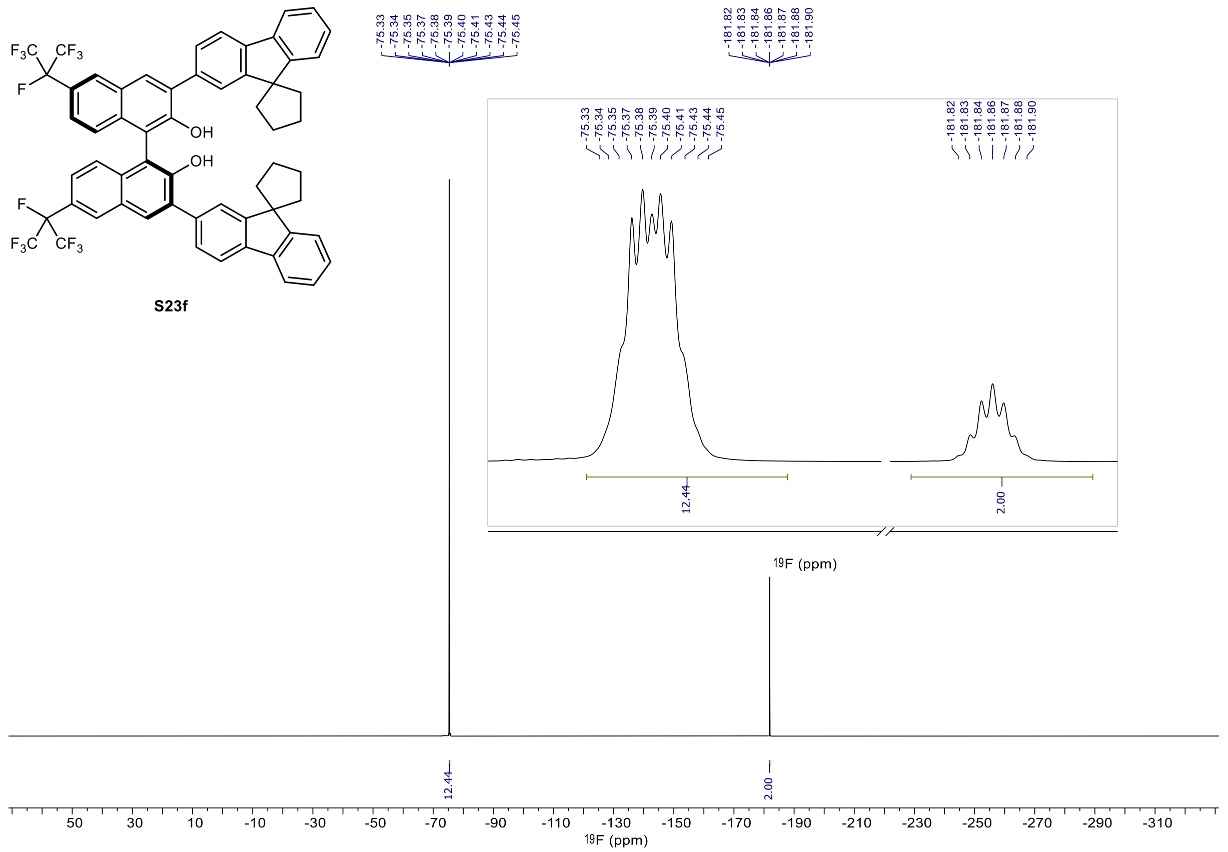

^{19}F NMR (565 MHz, $CDCl_3$) spectrum of diol **S23f**

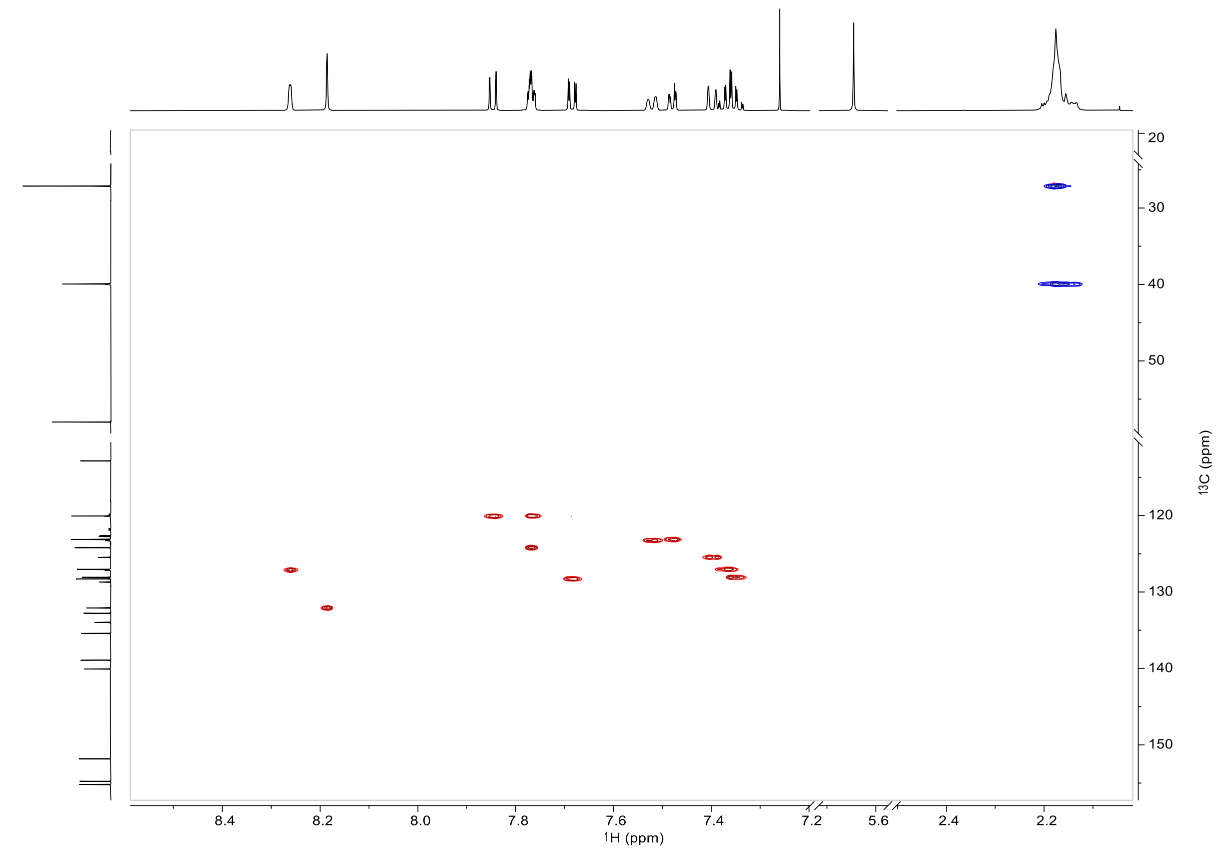

Multiplicity-edited **^{1}H,^{13}C-HSQC** (600 MHz, 151 MHz, $CDCl_3$) spectrum of diol **S23f**

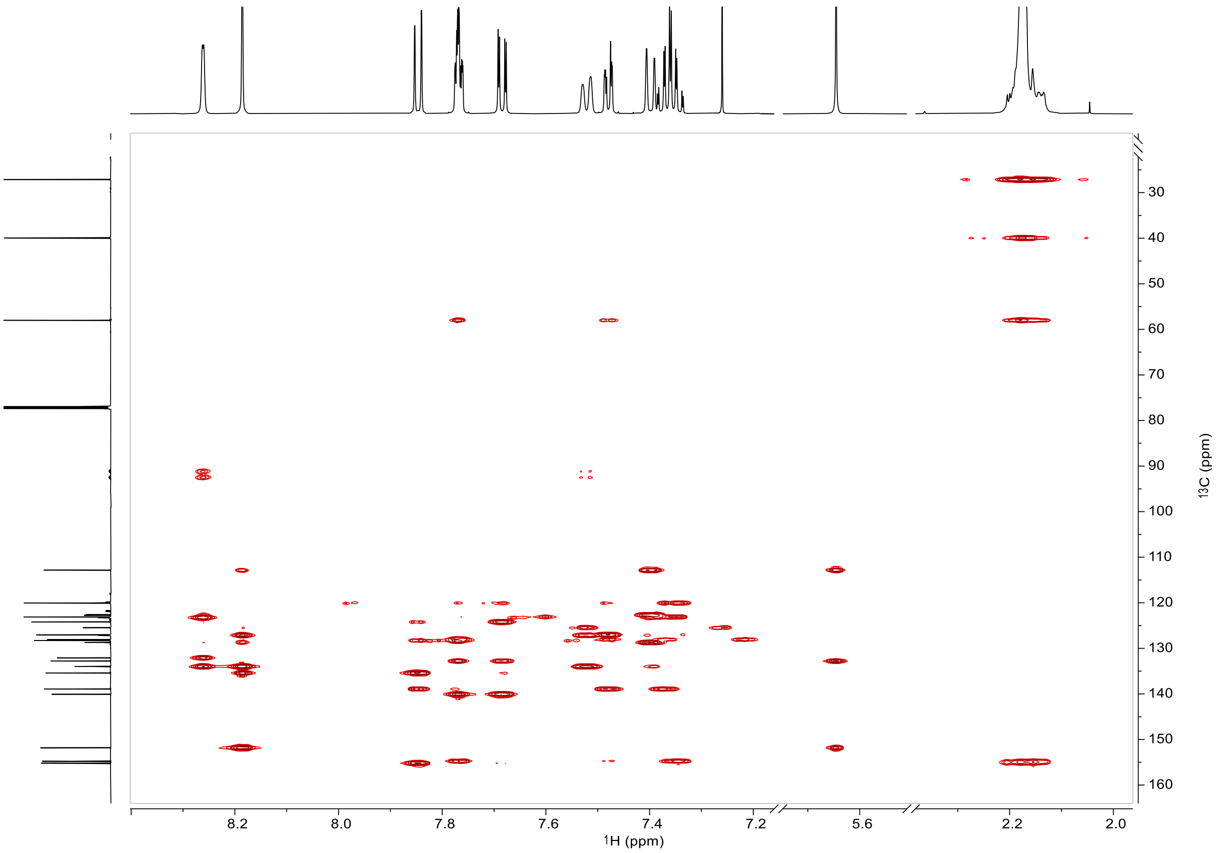

^{1}H,^{13}C-HMBC (600 MHz, 151 MHz, $CDCl_3$) spectrum of diol **S23f**

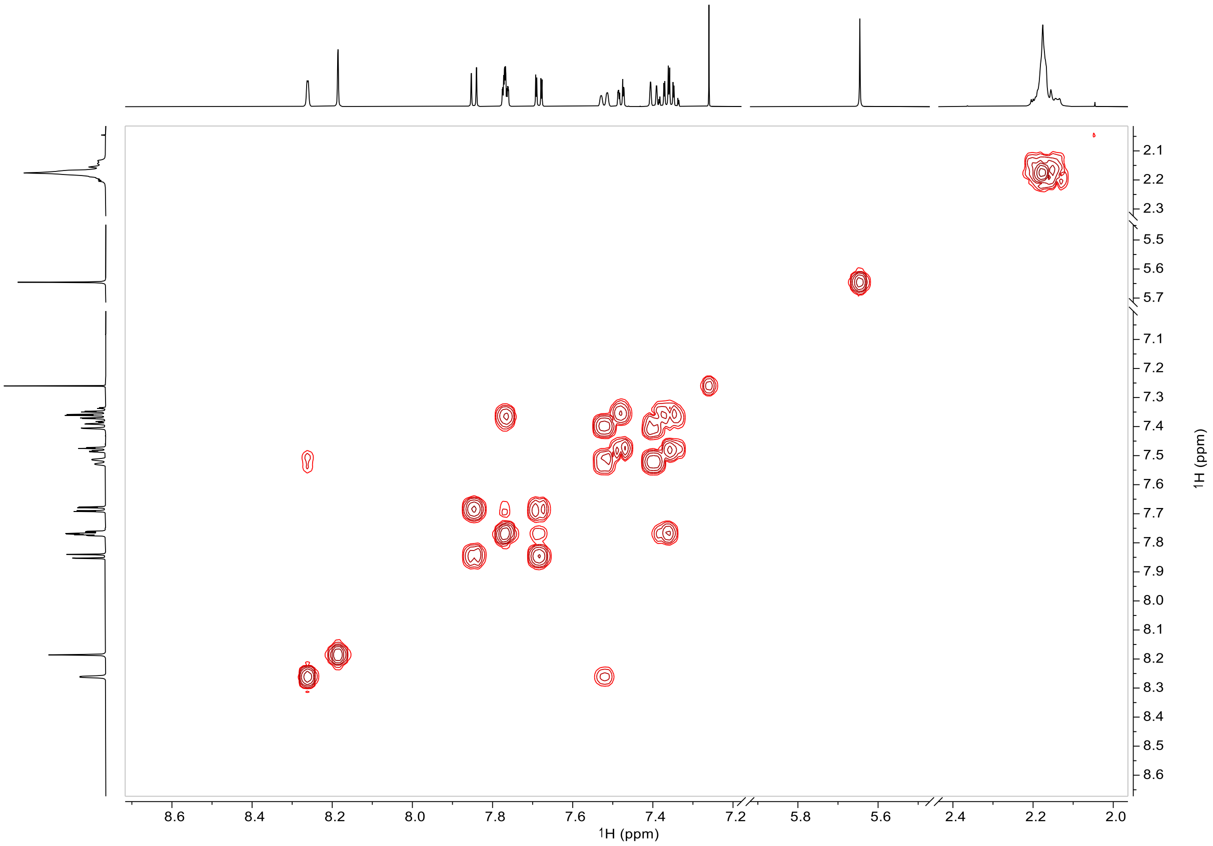

^{1}H,^{1}H-COSY (600 MHz, $CDCl_3$) spectrum of diol **S23f**

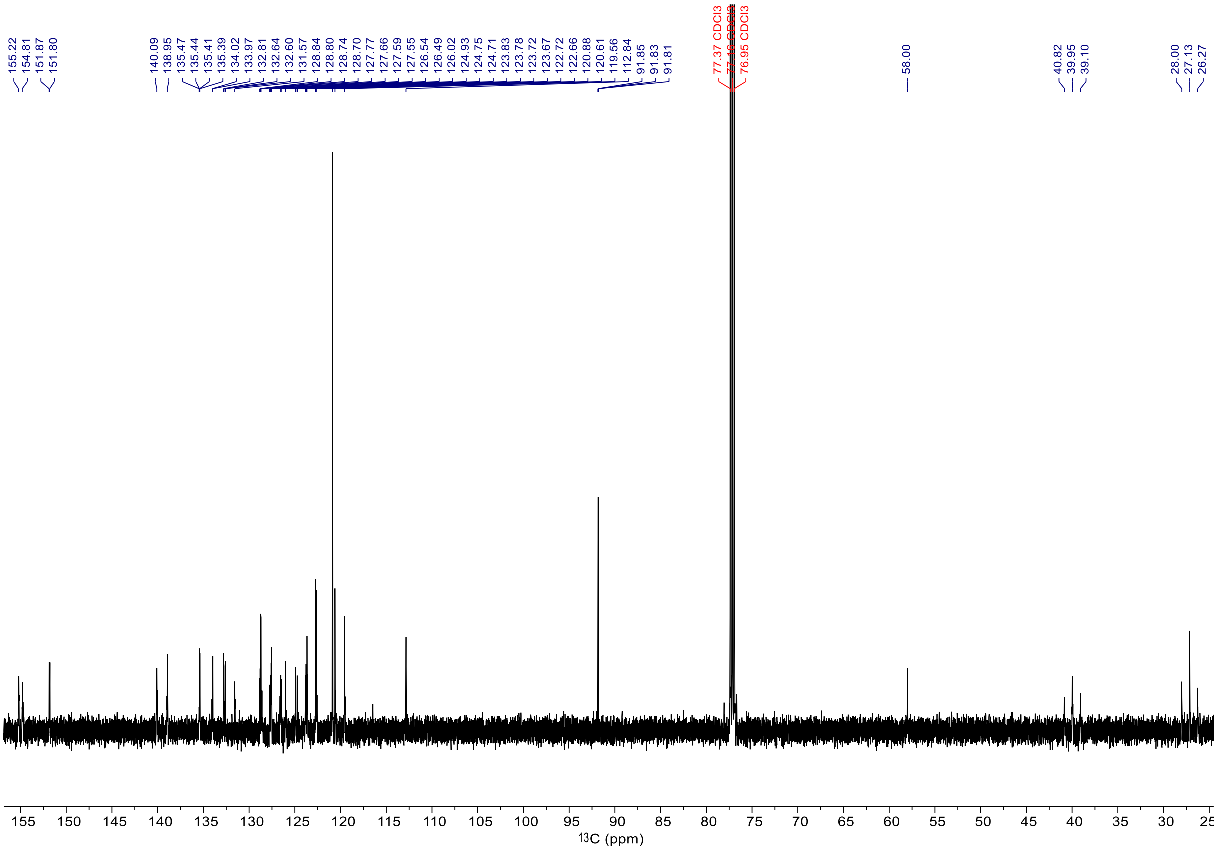

^{13}C{^{19}F} NMR (151 MHz, $CDCl_3$) spectrum of diol **S23f**

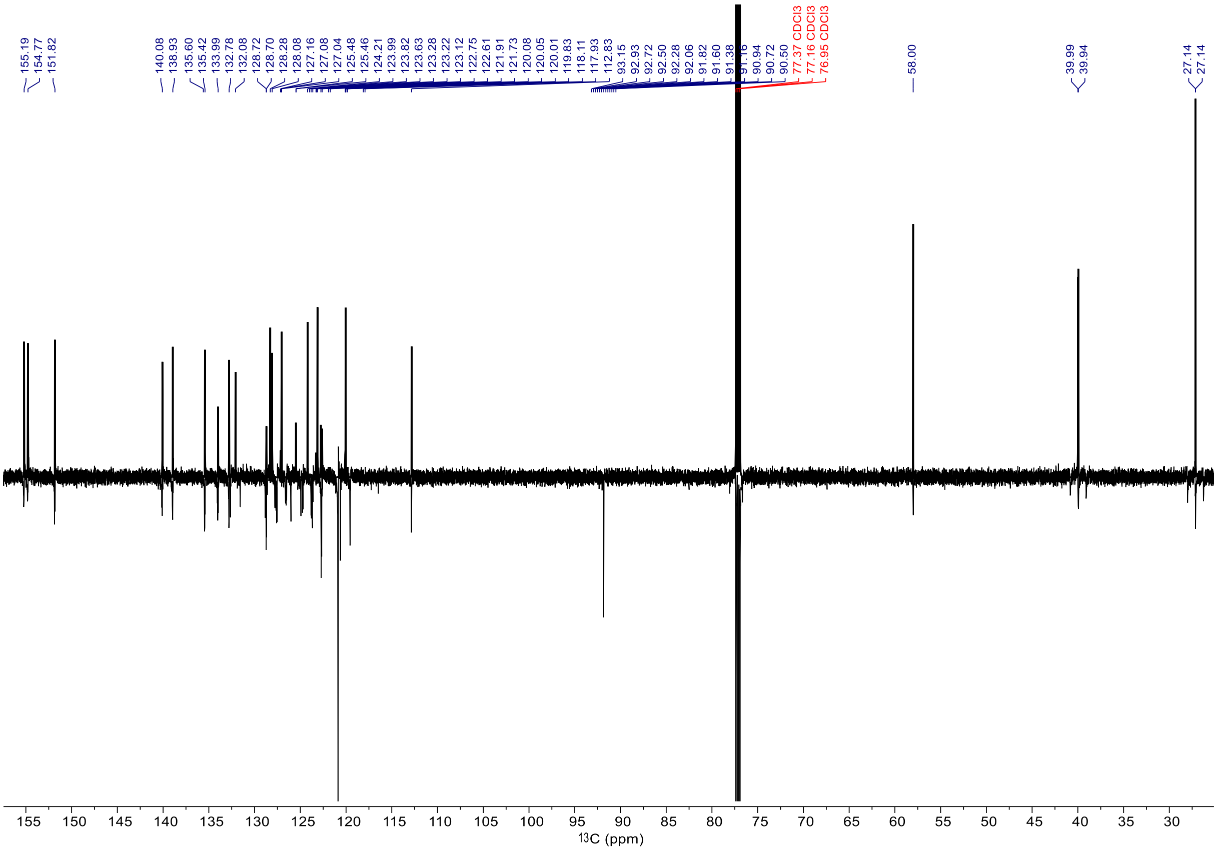

Superimposed **$^{13}C\{^{1}H\}$ NMR** (151 MHz, top) and **$^{13}C\{^{19}F\}$NMR** (151 MHz, bottom, mirrored) spectra

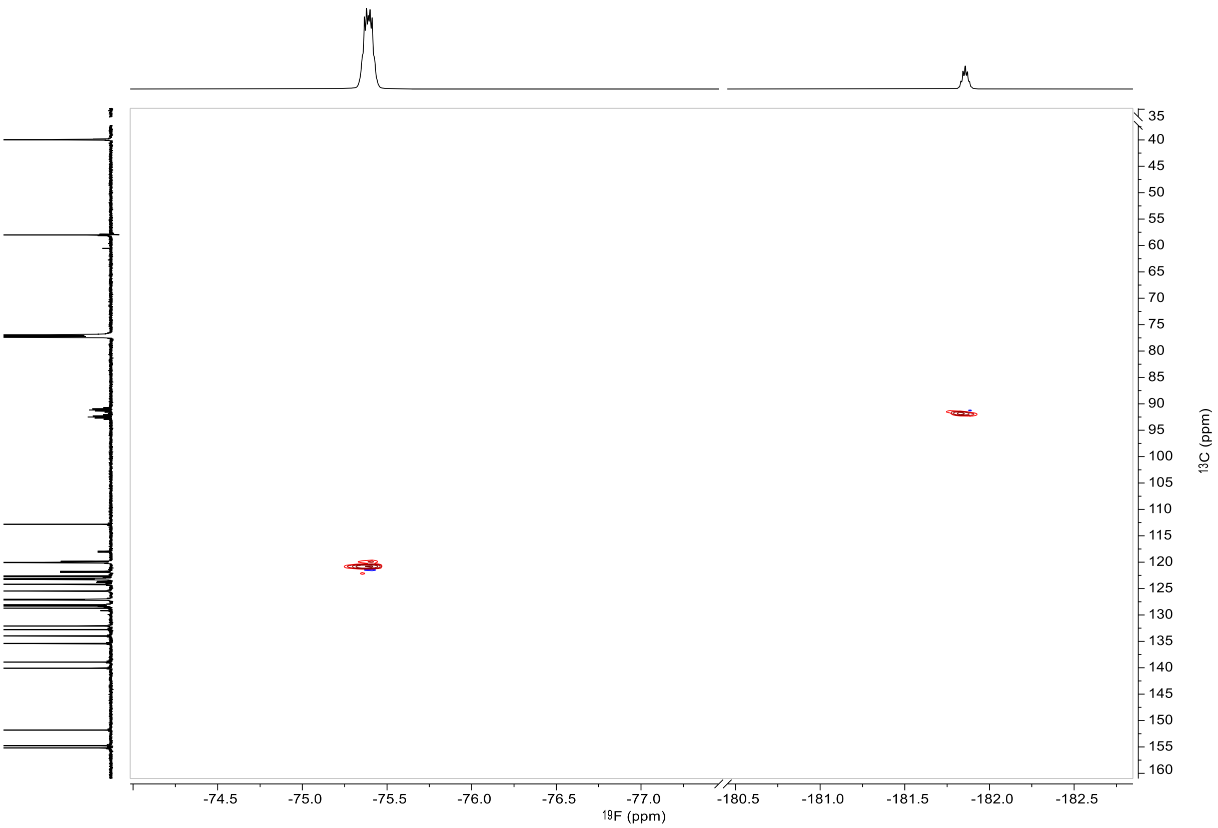

^{19}F,^{13}C-HSQC (565 MHz, 151 MHz, $CDCl_3$) spectrum of diol **S23f**

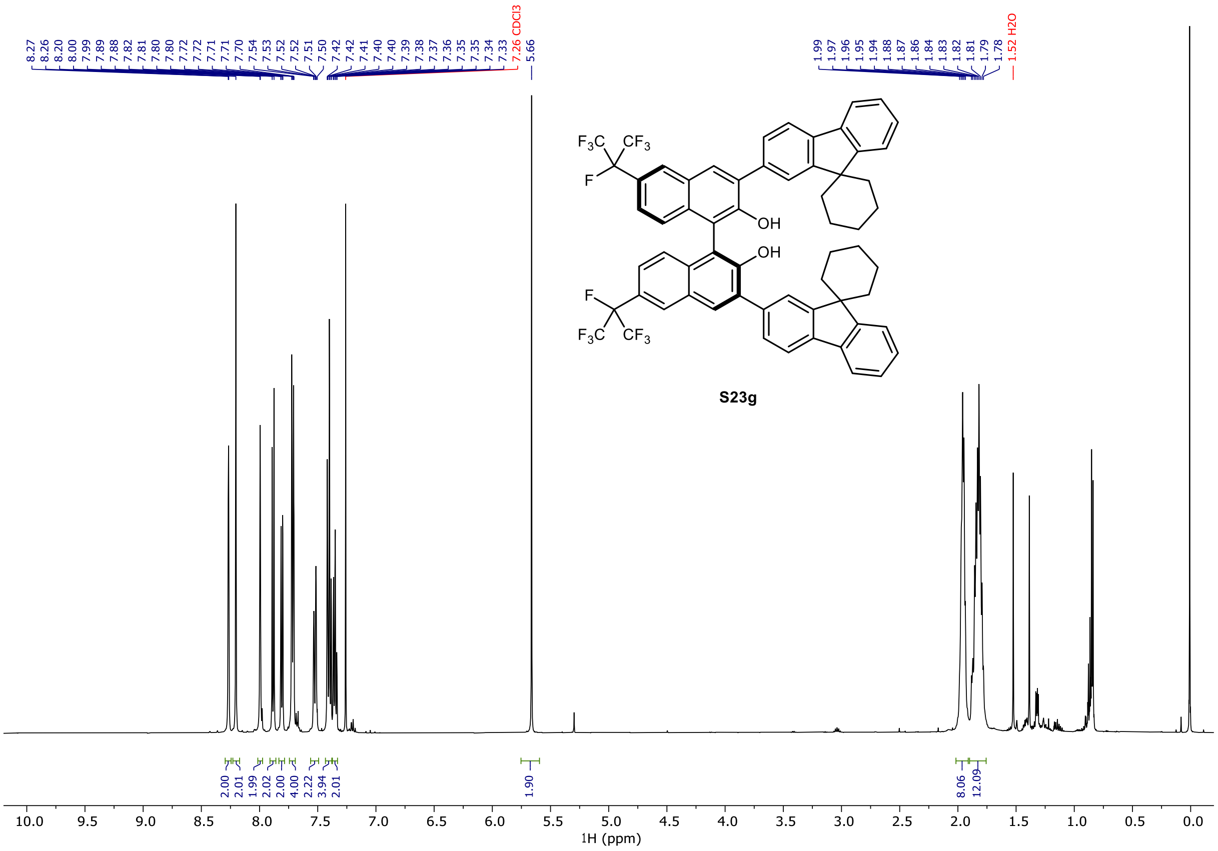

^{1}**H NMR** (501 MHz, $CDCl_3$) spectrum of diol **S23g**

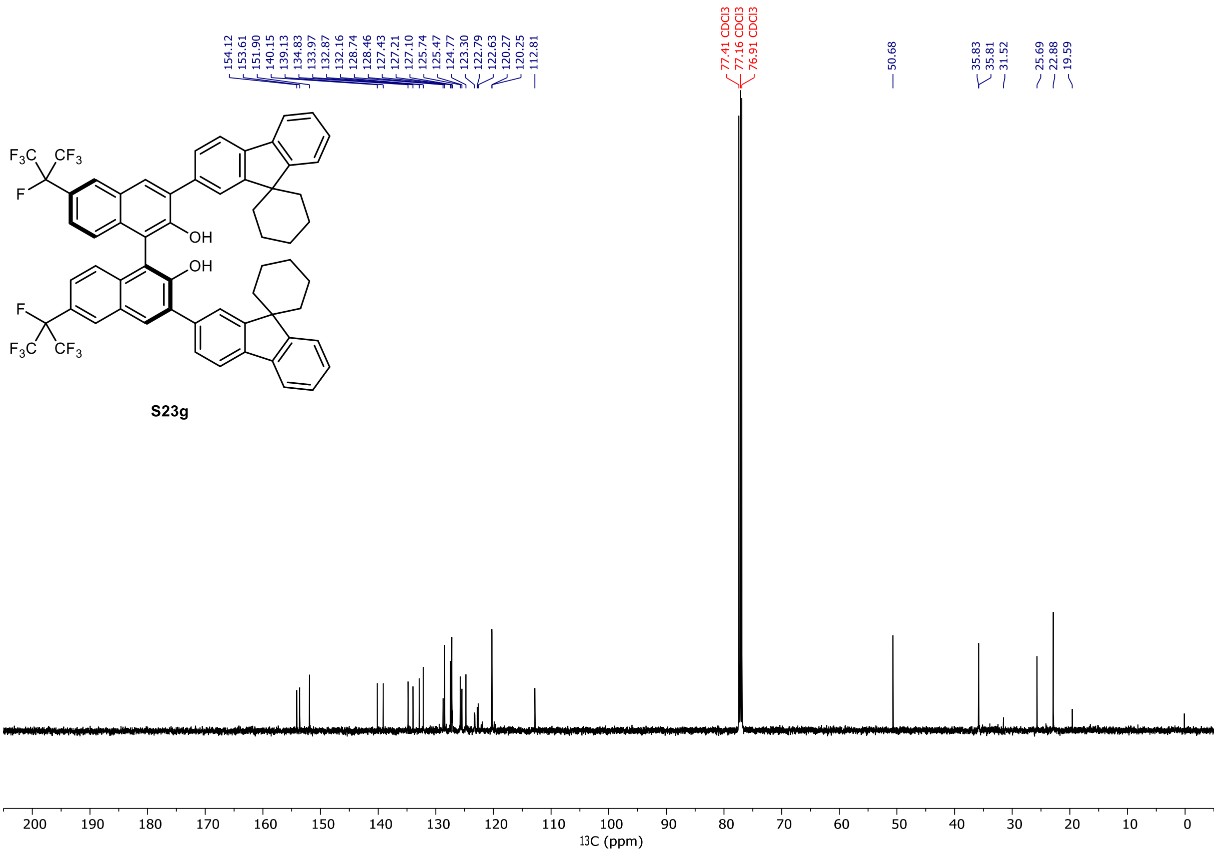

^{13}**C NMR** (126 MHz, $CDCl_3$) spectrum of diol **S23g**

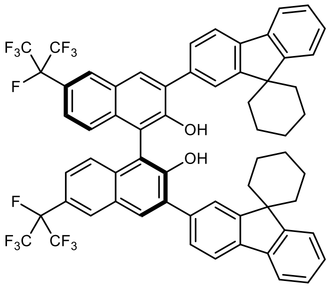

S23g

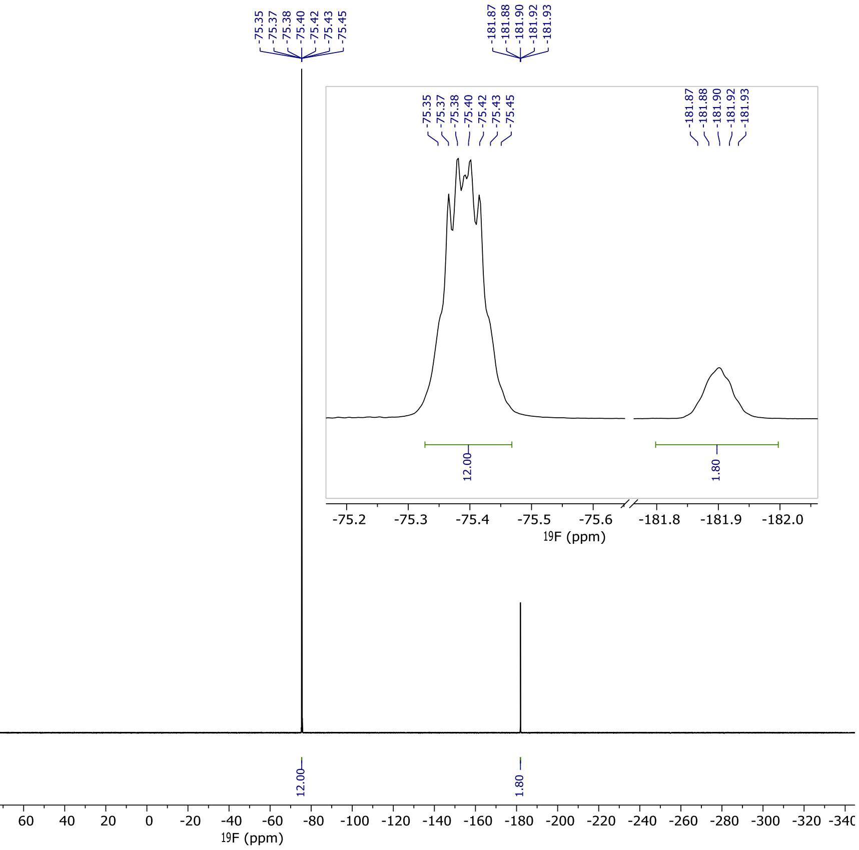

^{19}F NMR (471 MHz, $CDCl_3$) spectrum of diol **S23g**

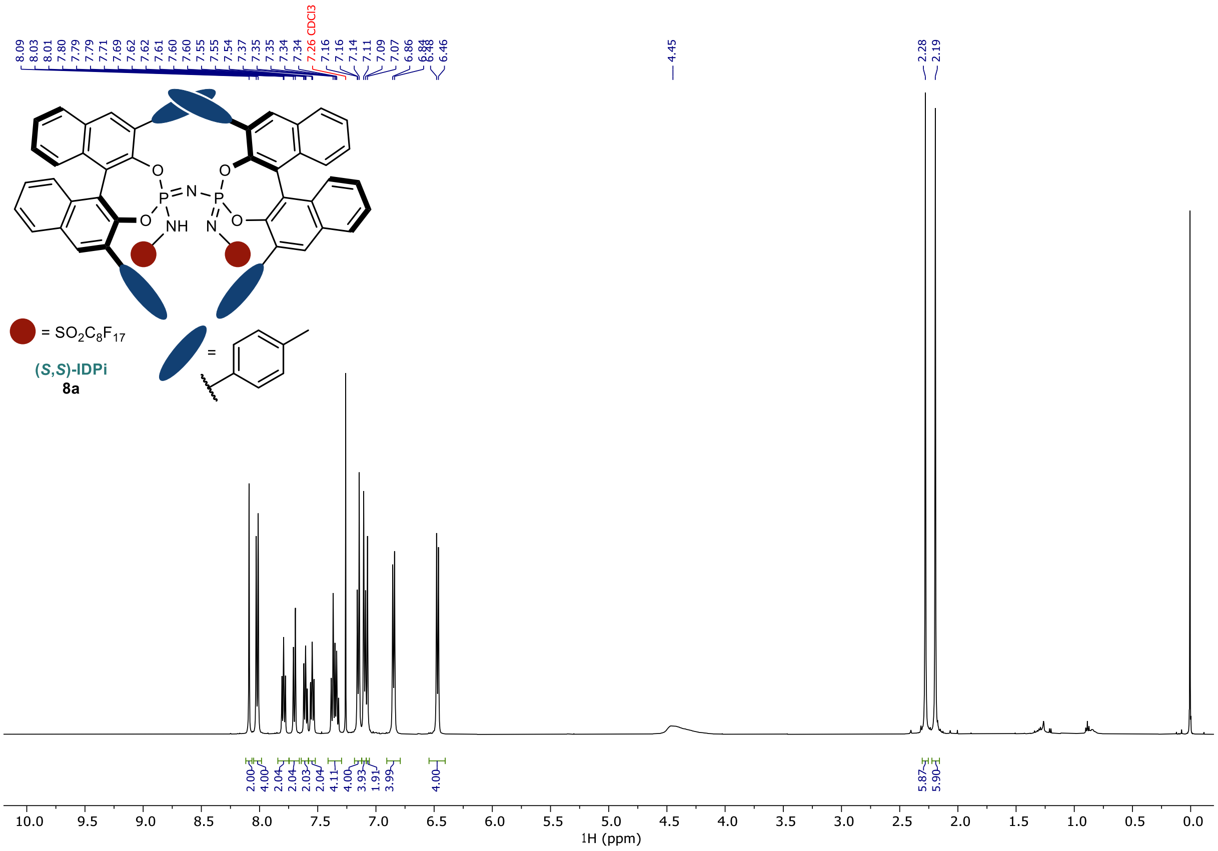

^{1}H NMR (501 MHz, $CDCl_3$) spectrum of (*S*,*S*)-IDPi catalyst **8a**

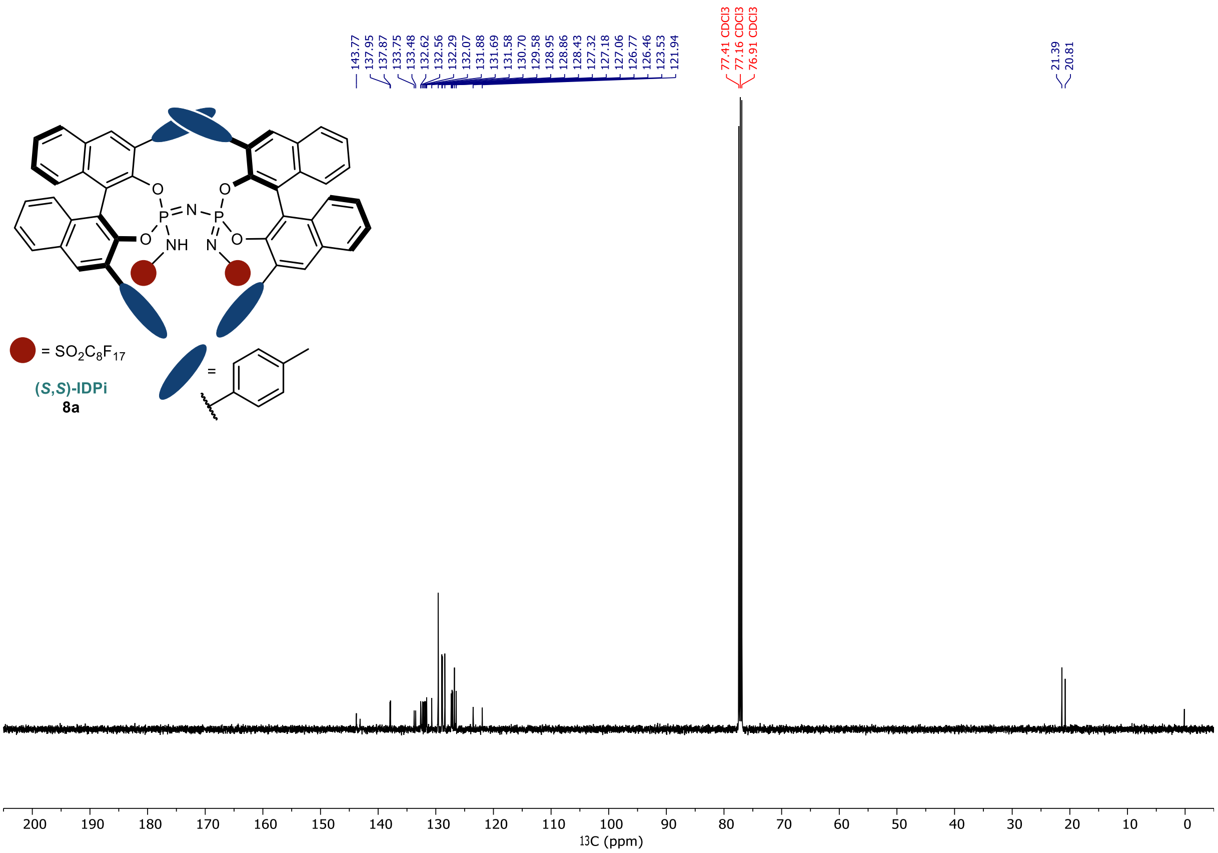

^{13}C NMR (126 MHz, $CDCl_3$) spectrum of (*S*,*S*)-IDPi catalyst **8a**

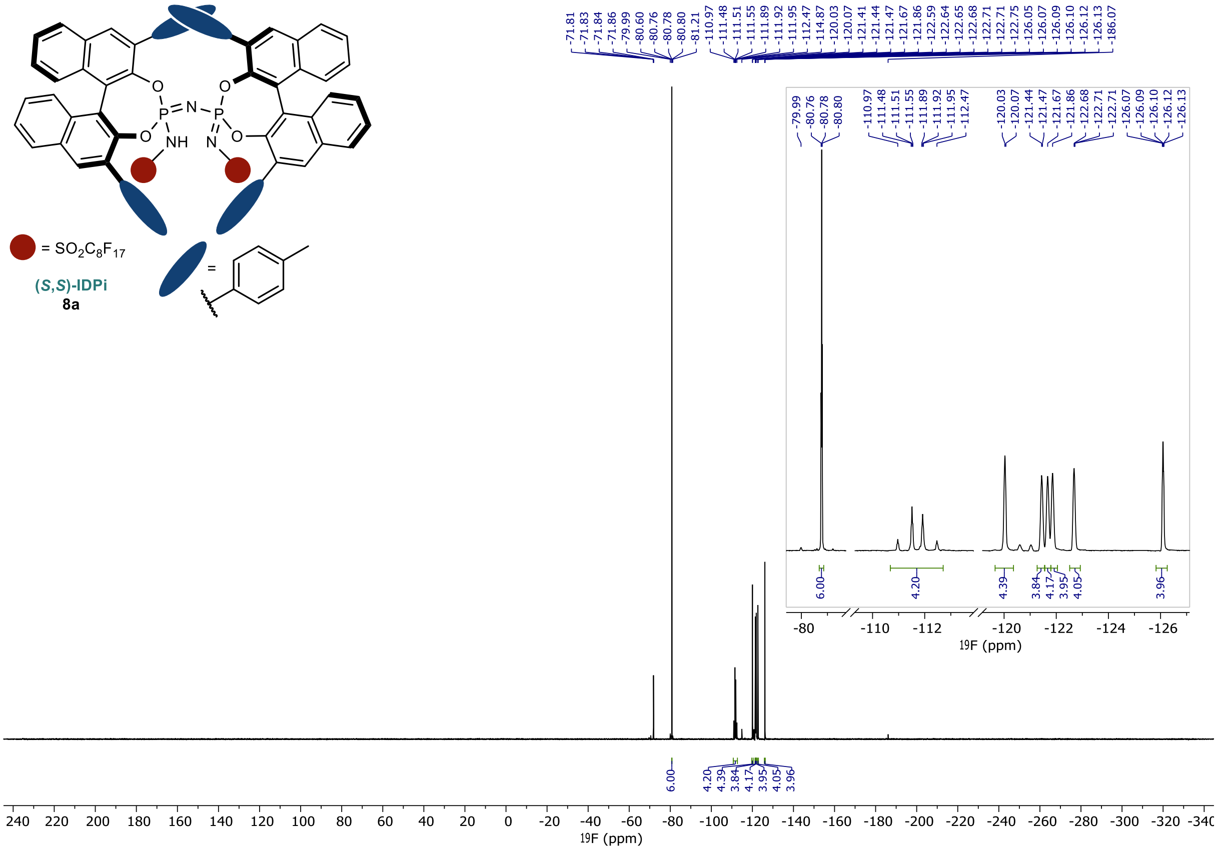

^{19}F NMR (471 MHz, $CDCl_3$) spectrum of (*S*,*S*)-IDPi catalyst **8a**

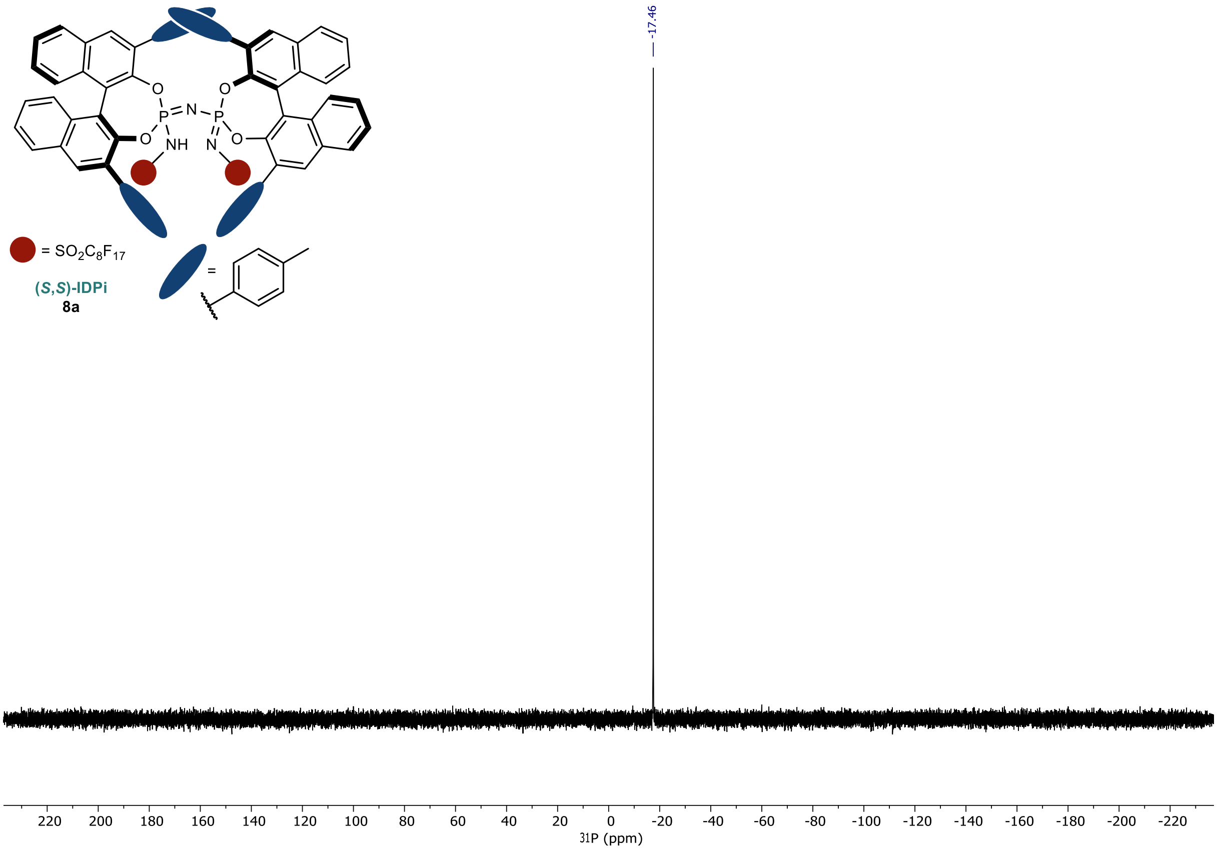

^{31}P NMR (203 MHz, $CDCl_3$) spectrum of (*S*,*S*)-IDPi catalyst **8a**

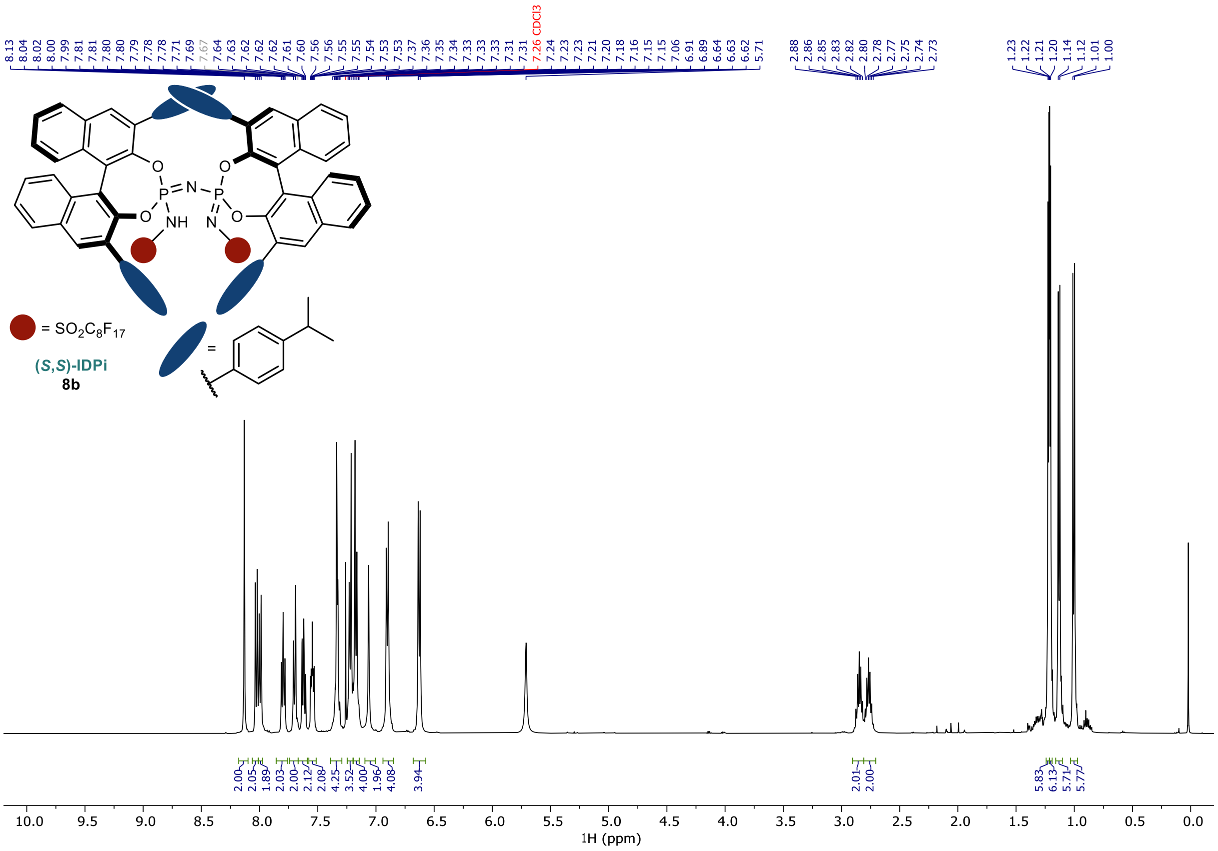

^{1}H NMR (501 MHz, $CDCl_3$) spectrum of (*S*,*S*)-IDPi catalyst **8b**

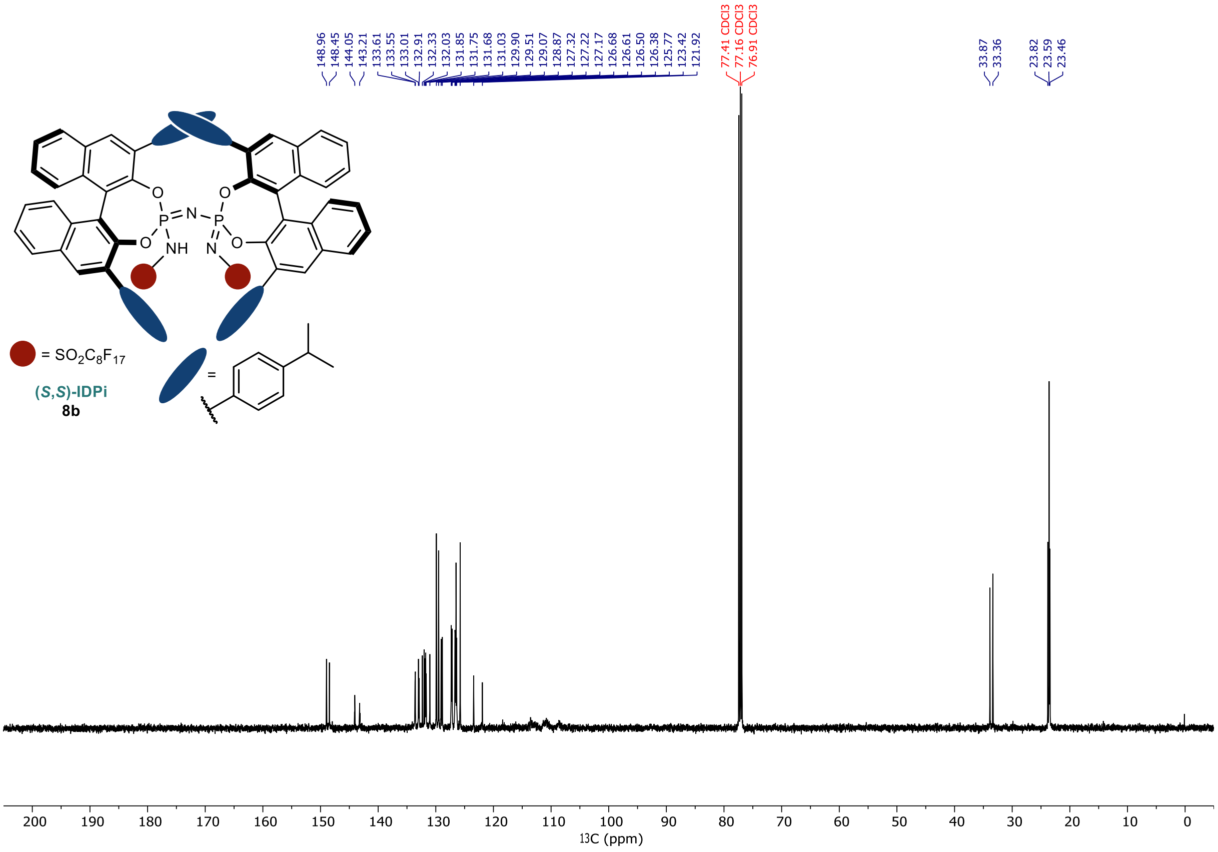

^{13}C NMR (126 MHz, $CDCl_3$) spectrum of (*S*,*S*)-IDPi catalyst **8b**

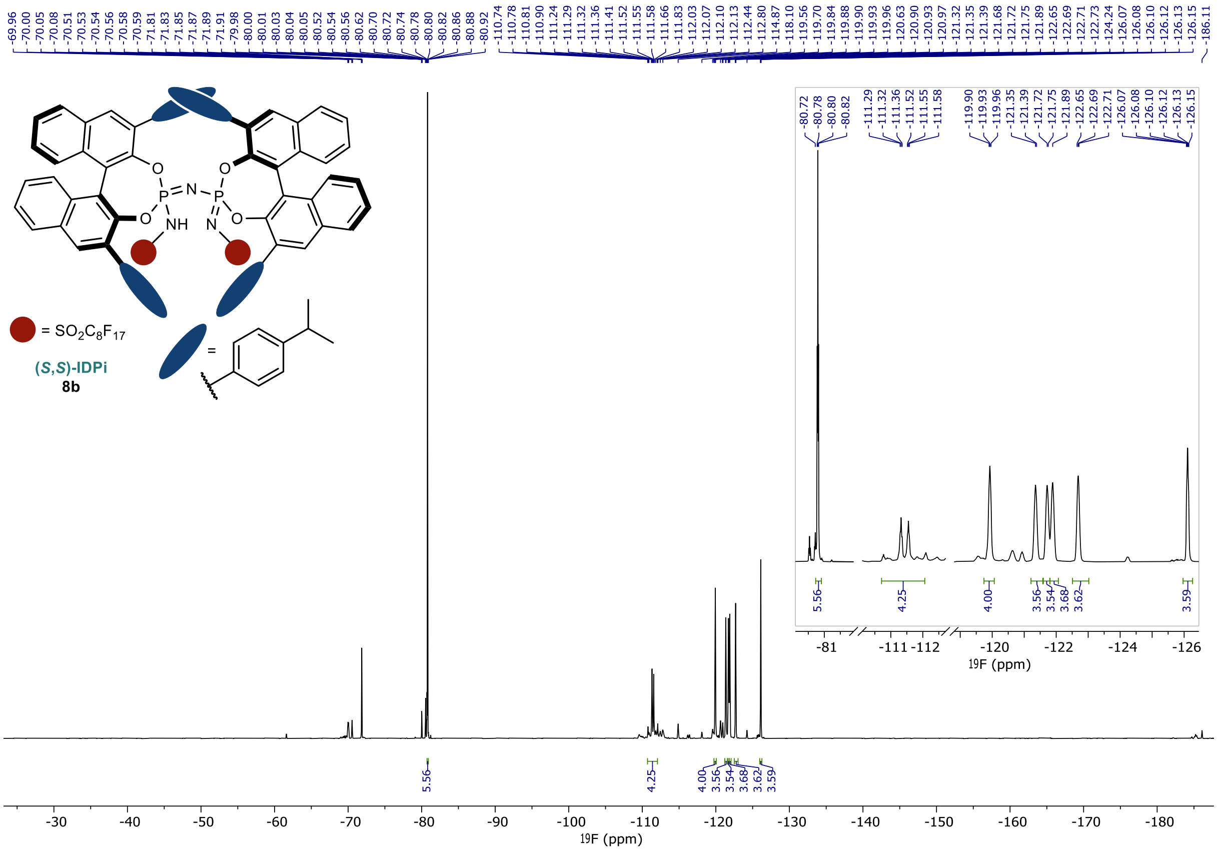

^{19}F NMR (471 MHz, $CDCl_3$) spectrum of (*S*,*S*)-IDPi catalyst **8b**

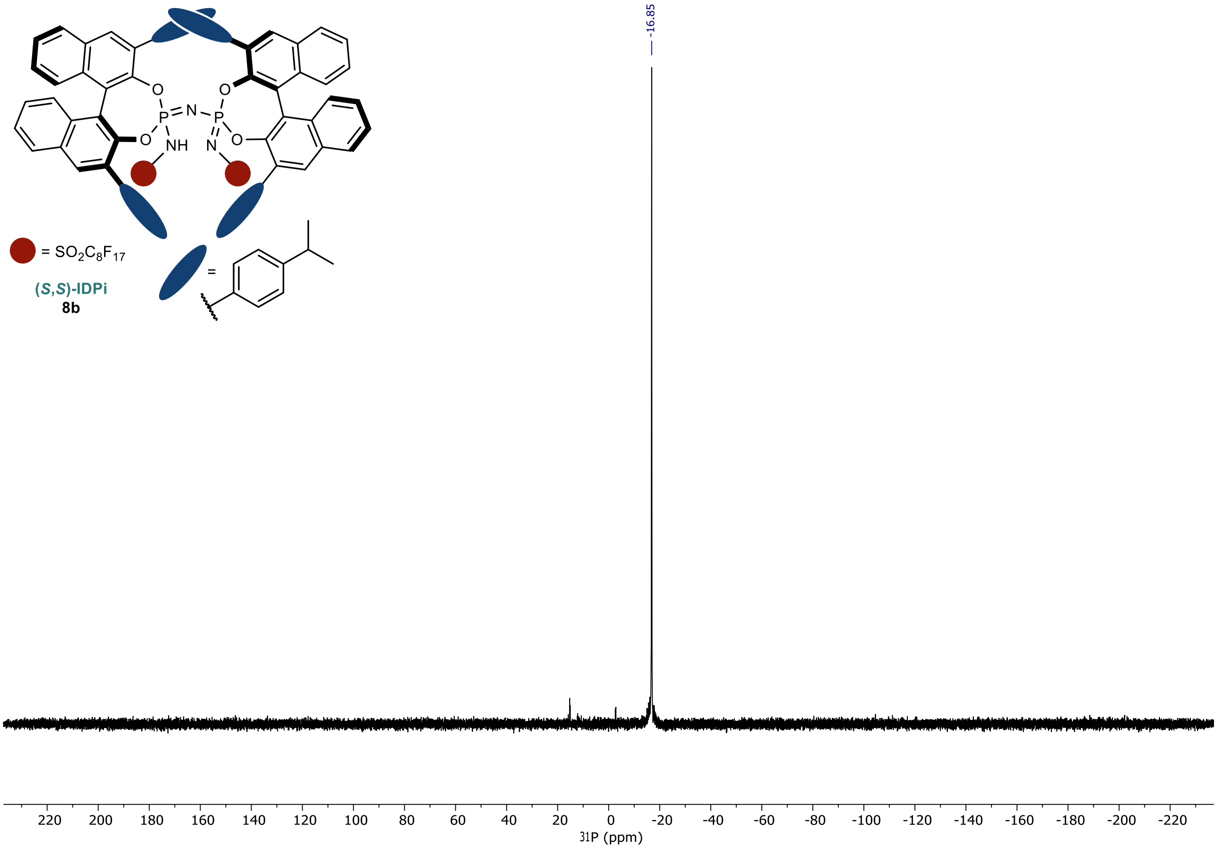

^{31}P NMR (203 MHz, $CDCl_3$) spectrum of (*S*,*S*)-IDPi catalyst **8b**

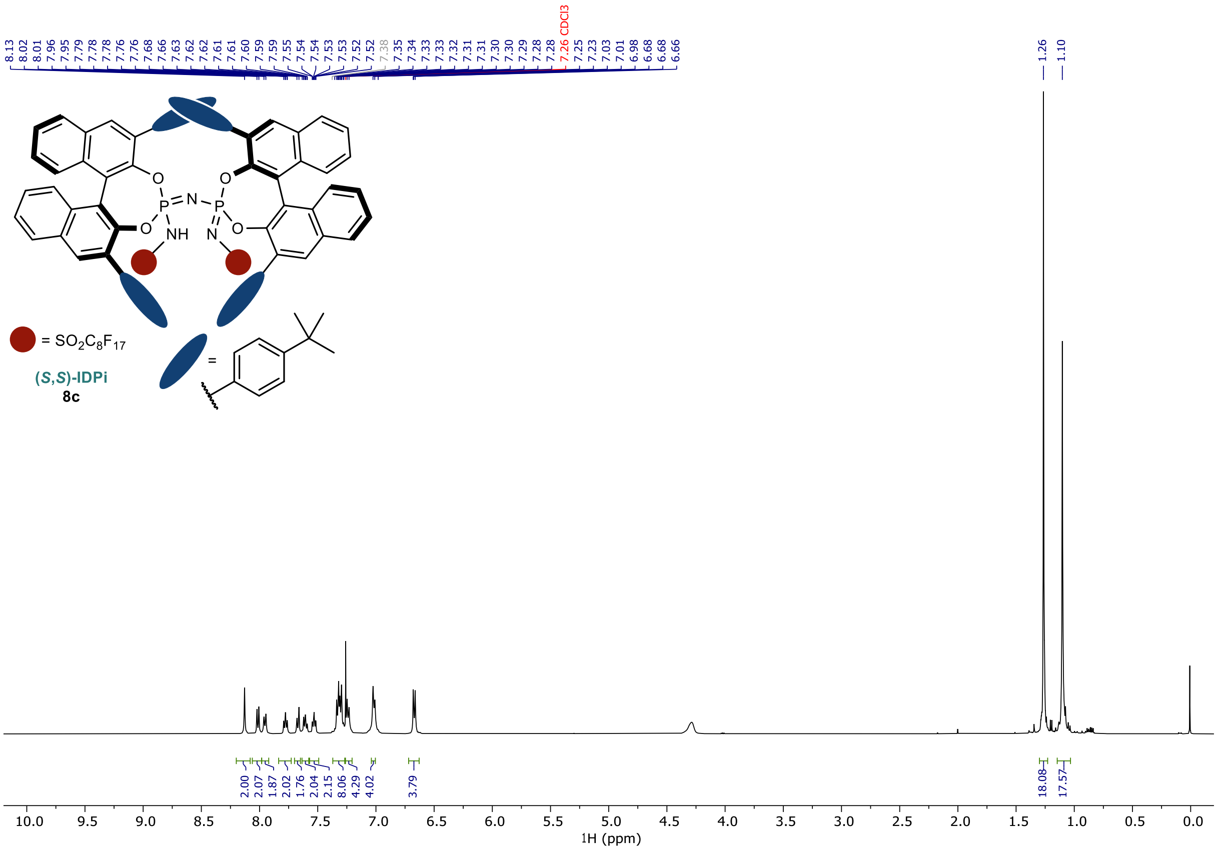

^{1}**H NMR** (501 MHz, $CDCl_3$) spectrum of (*S*,*S*)-IDPi catalyst **8c**

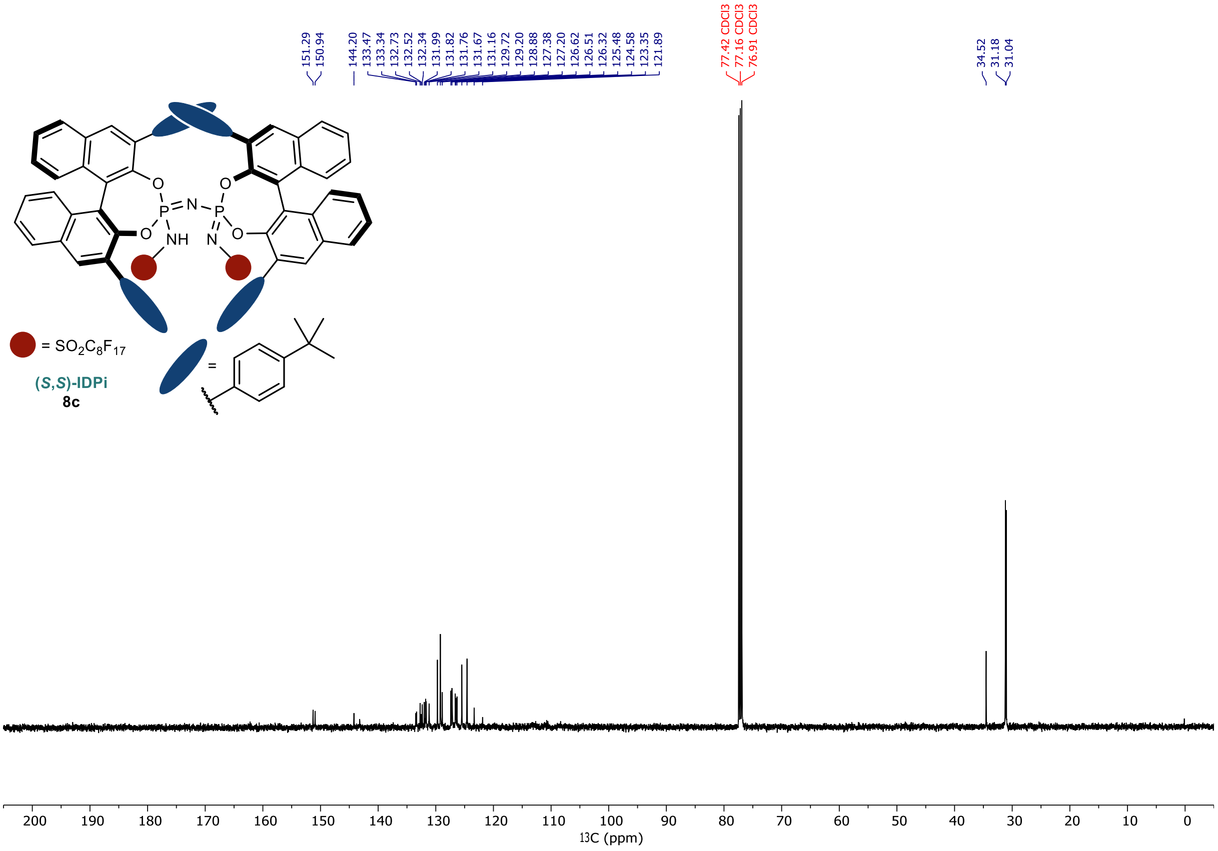

^{13}**C NMR** (126 MHz, $CDCl_3$) spectrum of (*S*,*S*)-IDPi catalyst **8c**

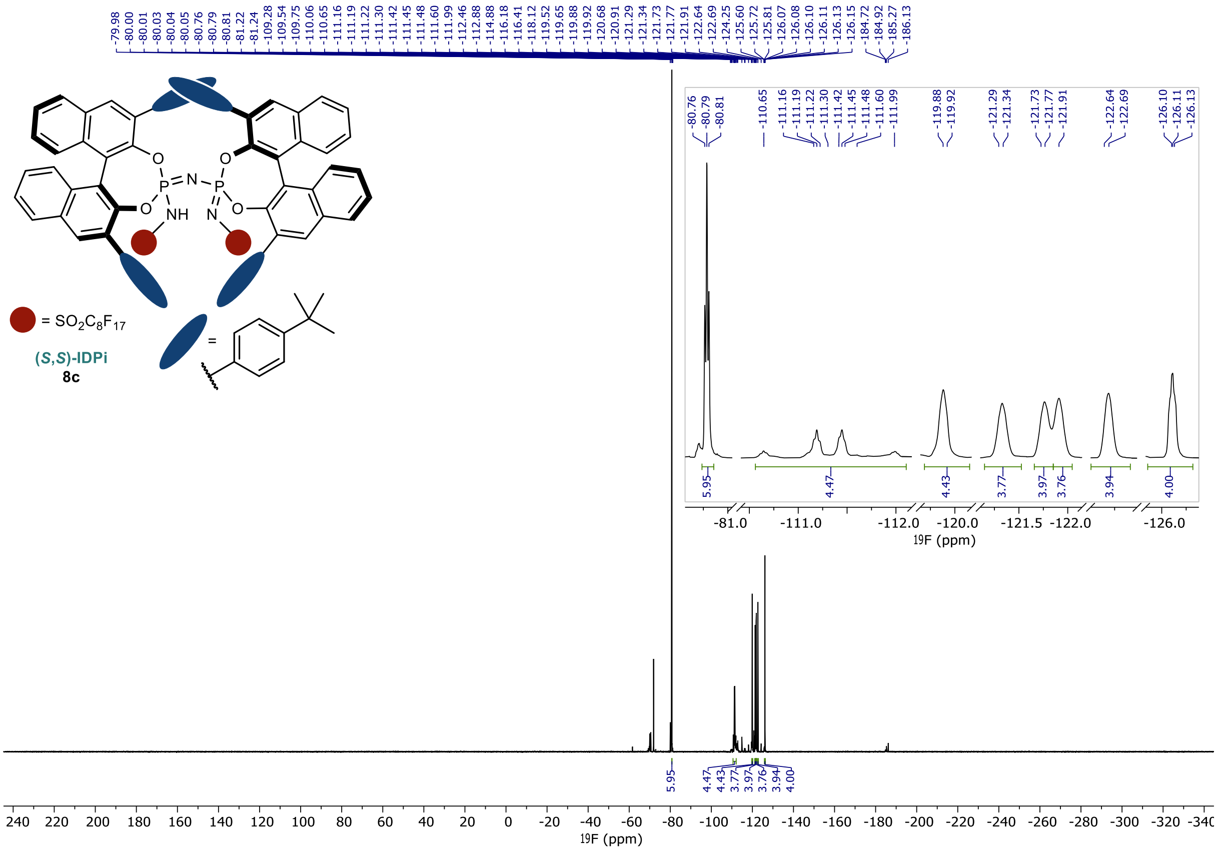

^{19}**F NMR** (471 MHz, $CDCl_3$) spectrum of (*S*,*S*)-IDPi catalyst **8c**

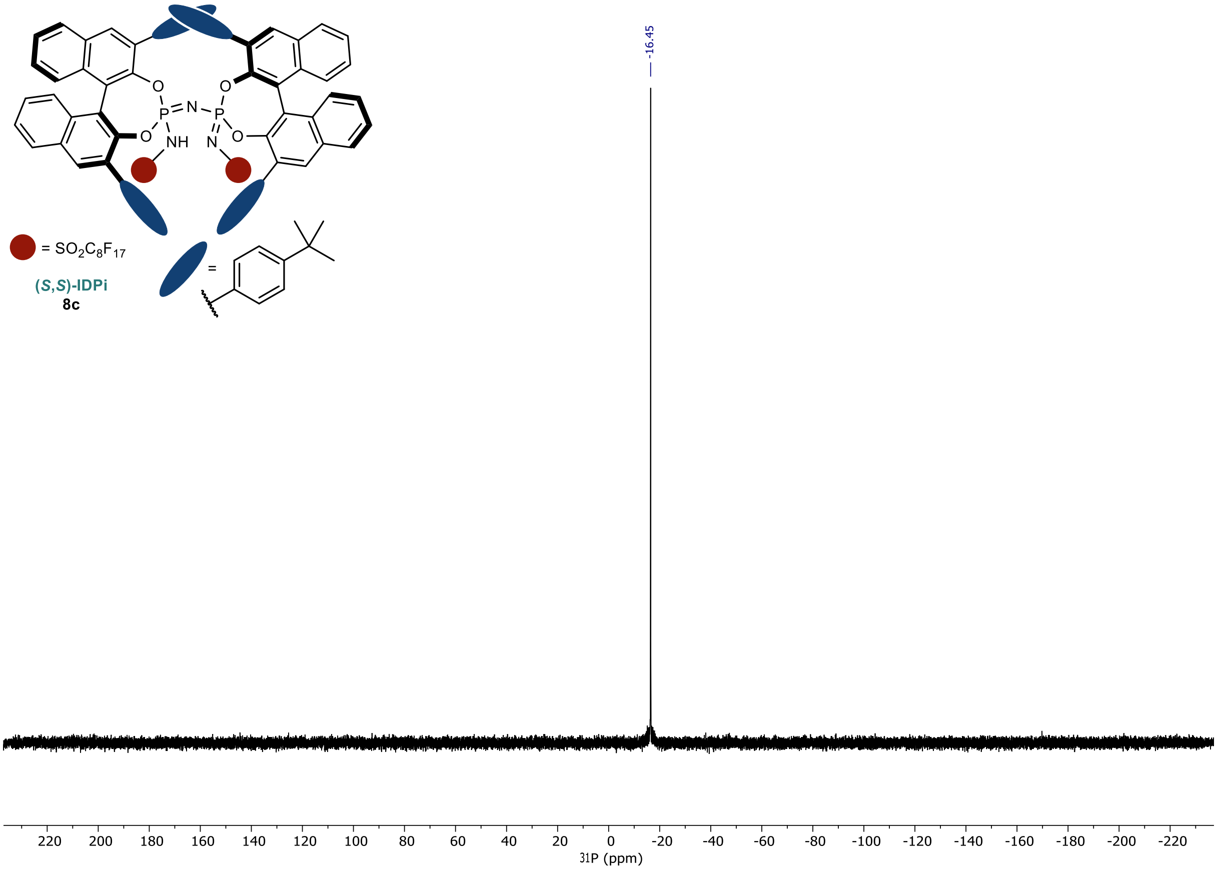

^{31}**P NMR** (203 MHz, $CDCl_3$) spectrum of (*S*,*S*)-IDPi catalyst **8c**

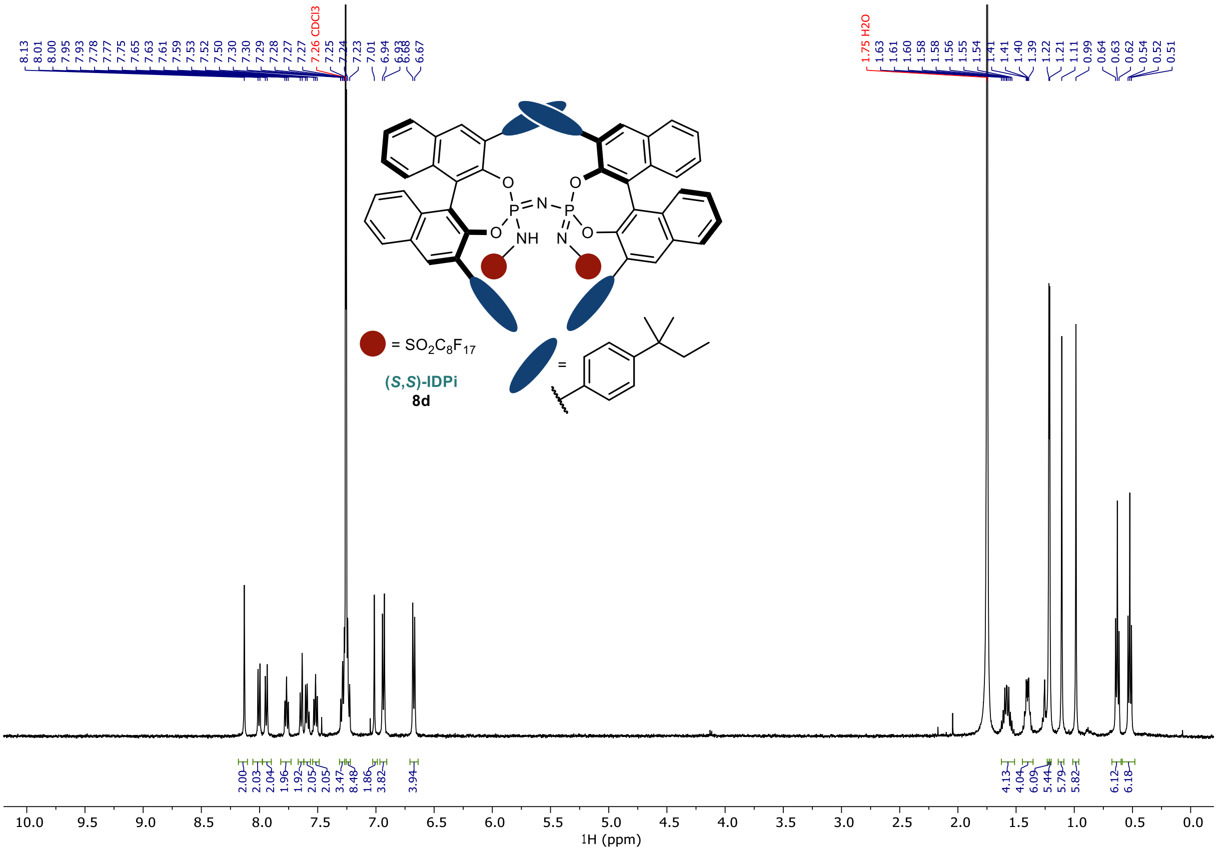

^{1}H NMR (501 MHz, $CDCl_3$) spectrum of (*S*,*S*)-IDPi catalyst **8d**

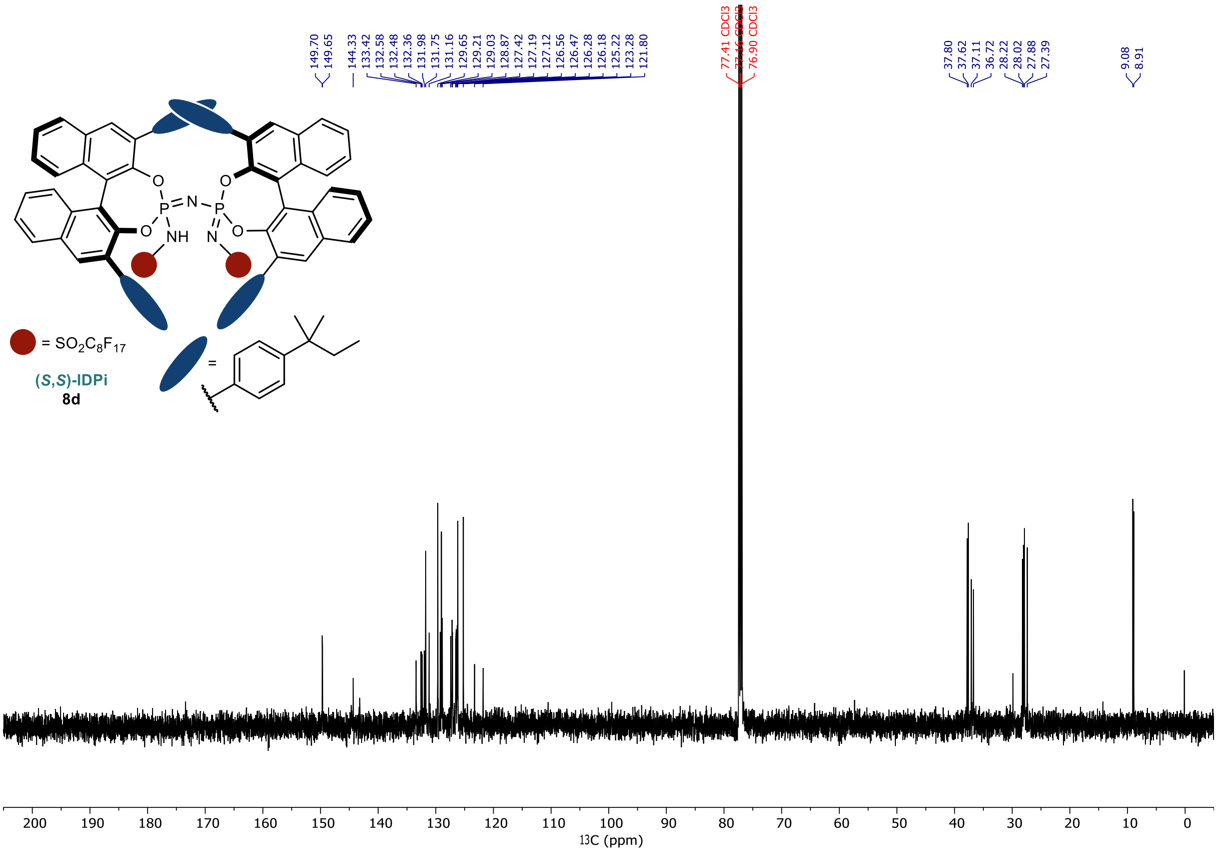

^{13}C NMR (126 MHz, $CDCl_3$) spectrum of (*S*,*S*)-IDPi catalyst **8d**

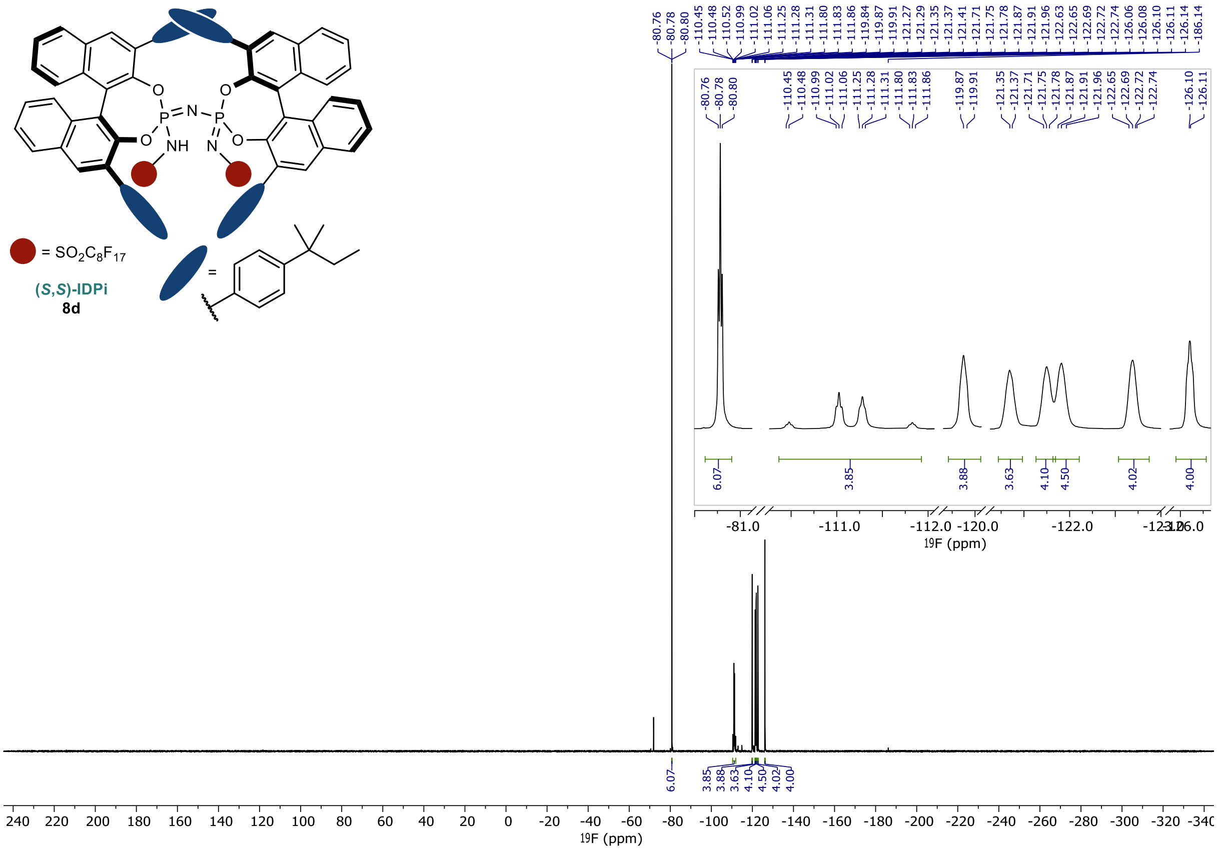

^{19}F NMR (471 MHz, $CDCl_3$) spectrum of (*S*,*S*)-IDPi catalyst **8d**

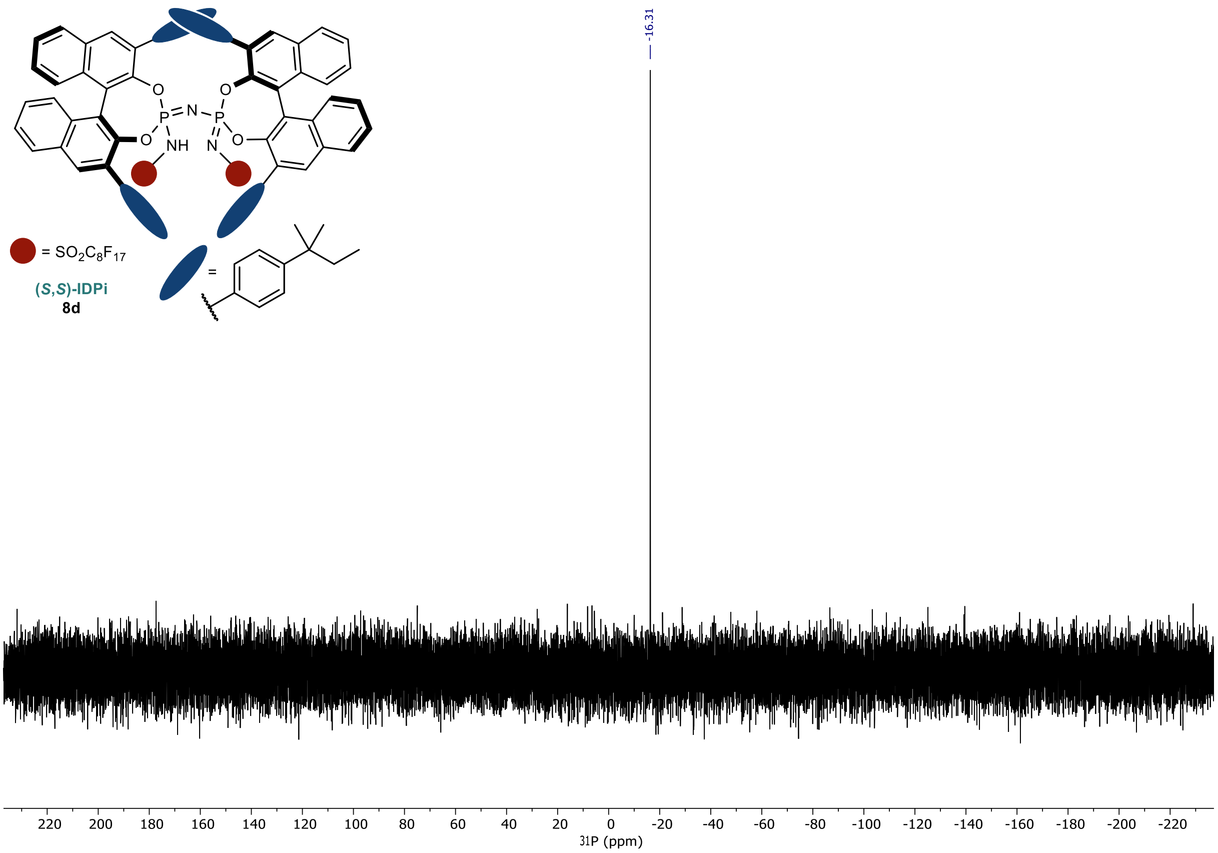

^{31}P NMR (203 MHz, $CDCl_3$) spectrum of (*S*,*S*)-IDPi catalyst **8d**

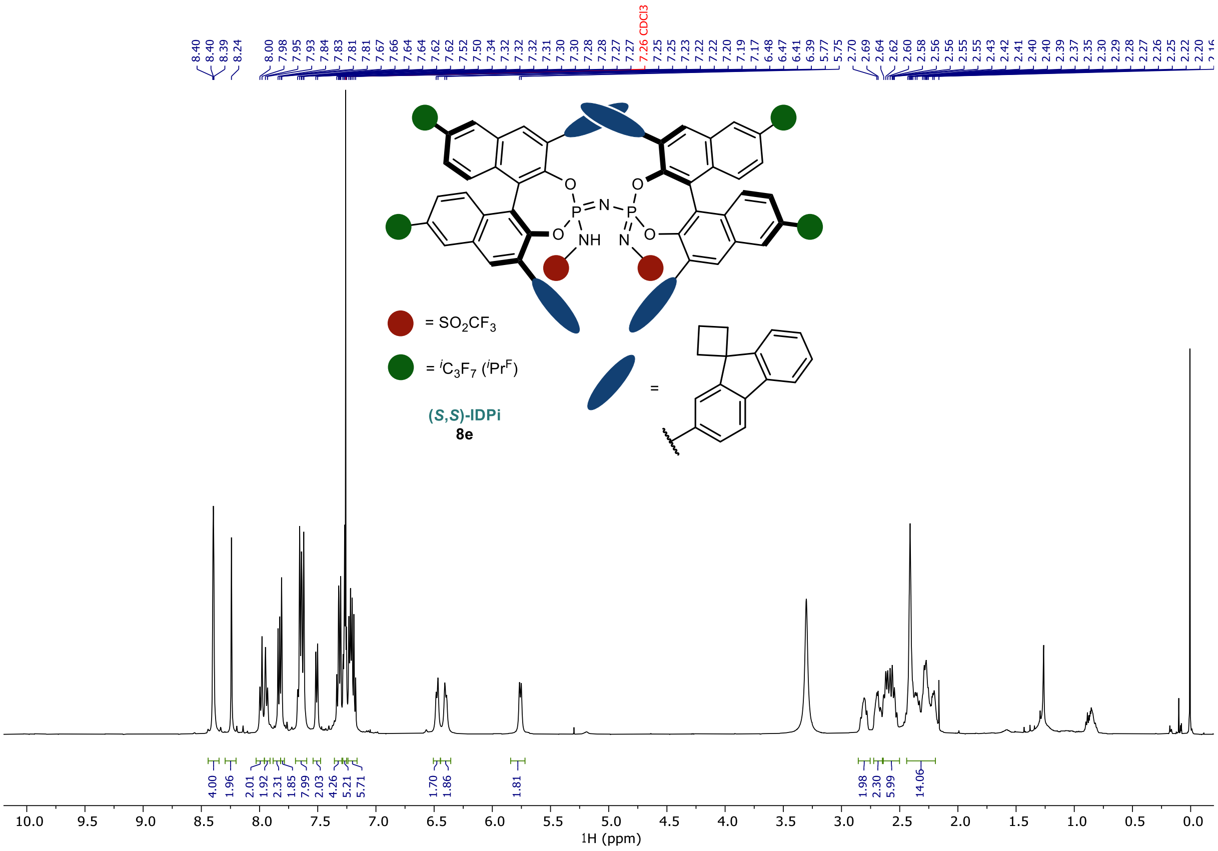

^{1}H NMR (501 MHz, $CDCl_3$) spectrum of (*S*,*S*)-IDPi catalyst **8e**

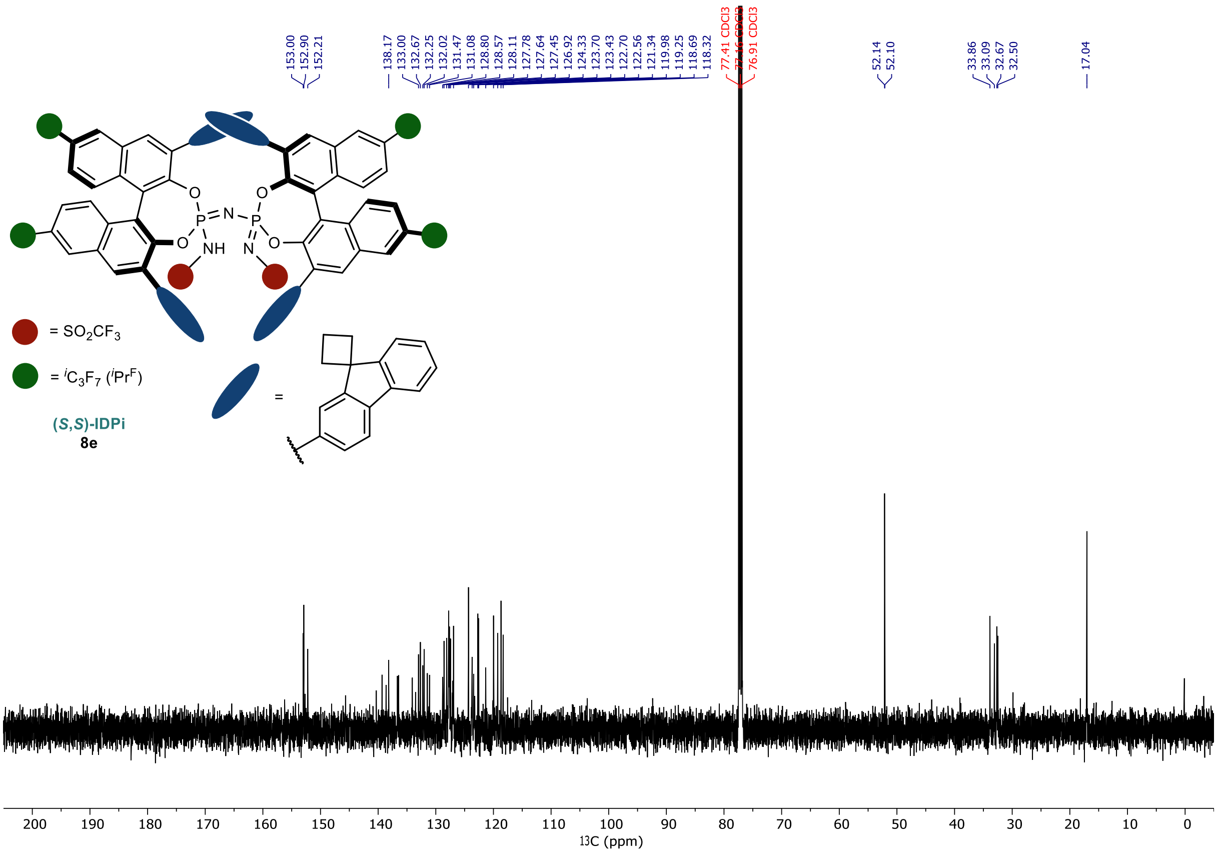

^{13}C NMR (126 MHz, $CDCl_3$) spectrum of (*S*,*S*)-IDPi catalyst **8e**

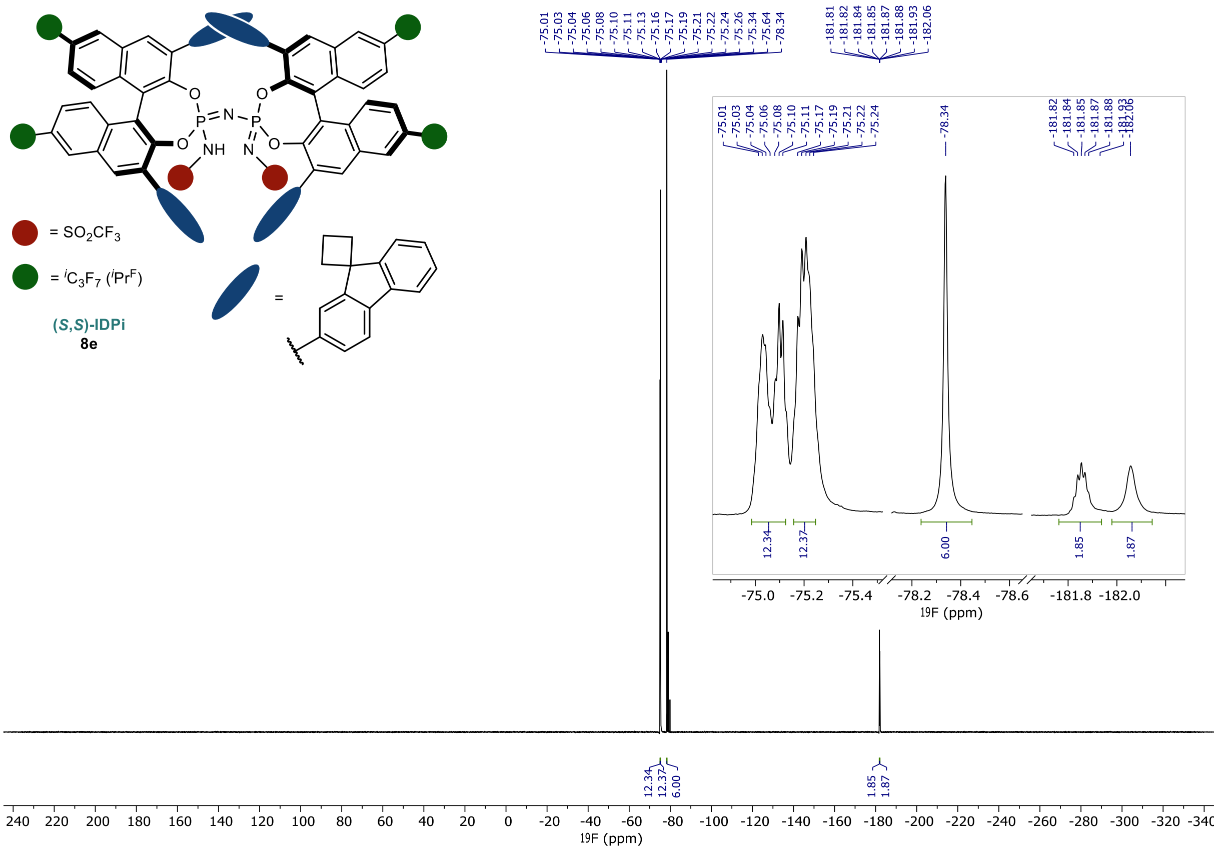

^{19}F NMR (471 MHz, $CDCl_3$) spectrum of (*S*,*S*)-IDPi catalyst **8e**

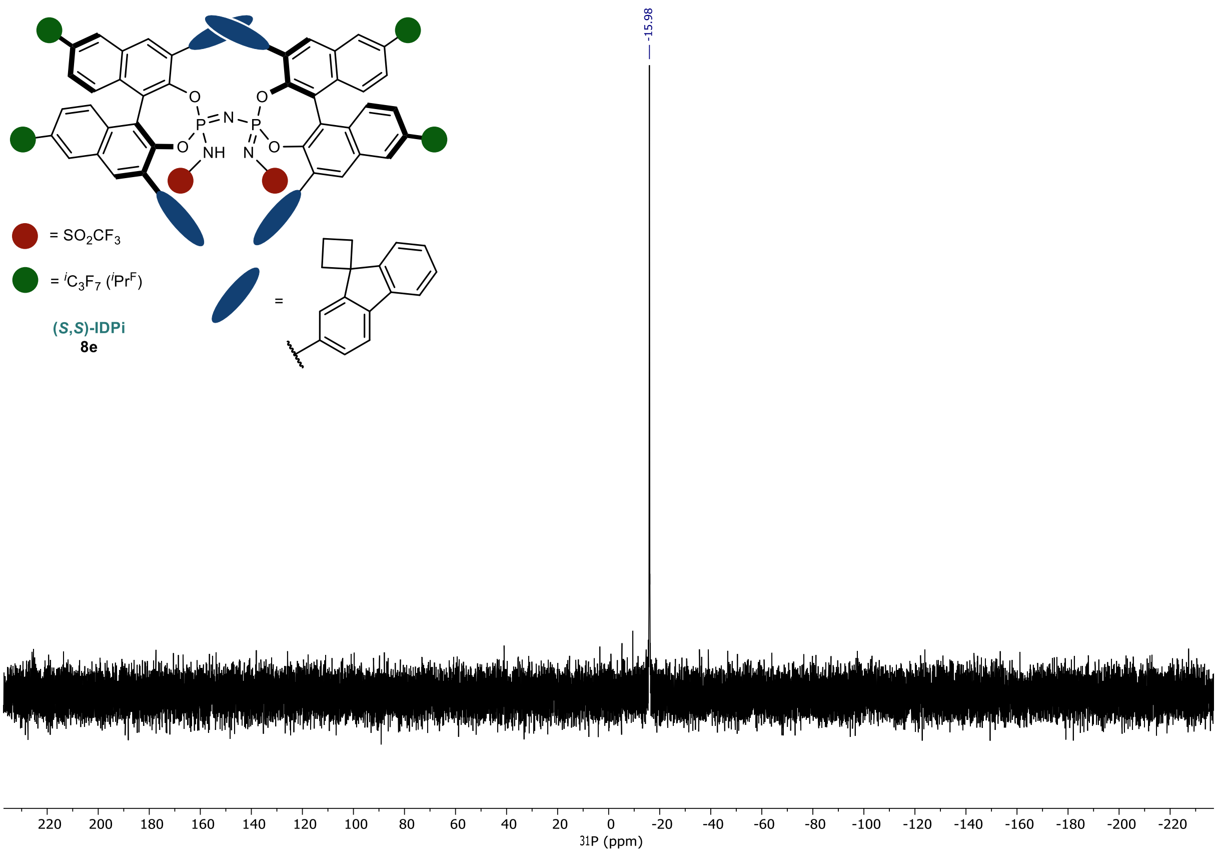

^{31}P NMR (203 MHz, $CDCl_3$) spectrum of (*S*,*S*)-IDPi catalyst **8e**

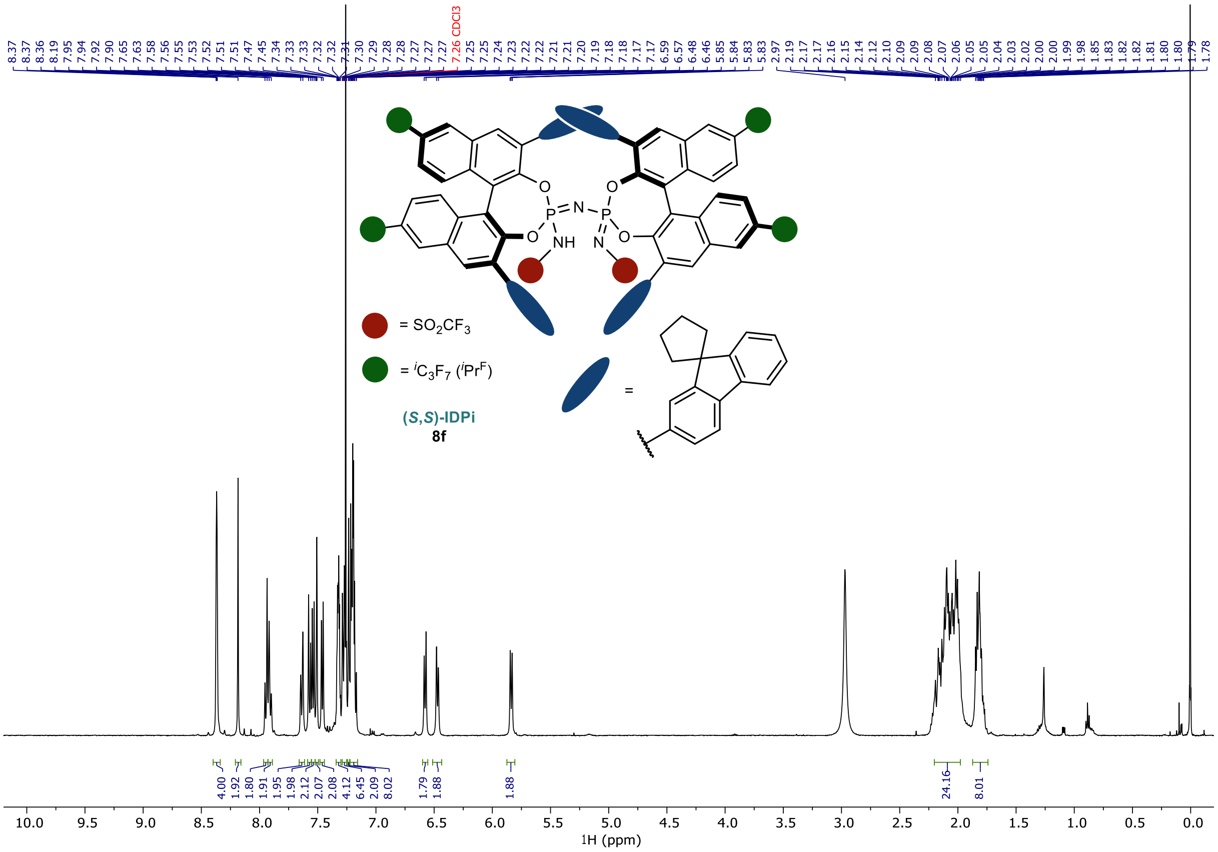

^{1}H NMR (501 MHz, $CDCl_3$) spectrum of (*S*,*S*)-IDPi catalyst **8f**

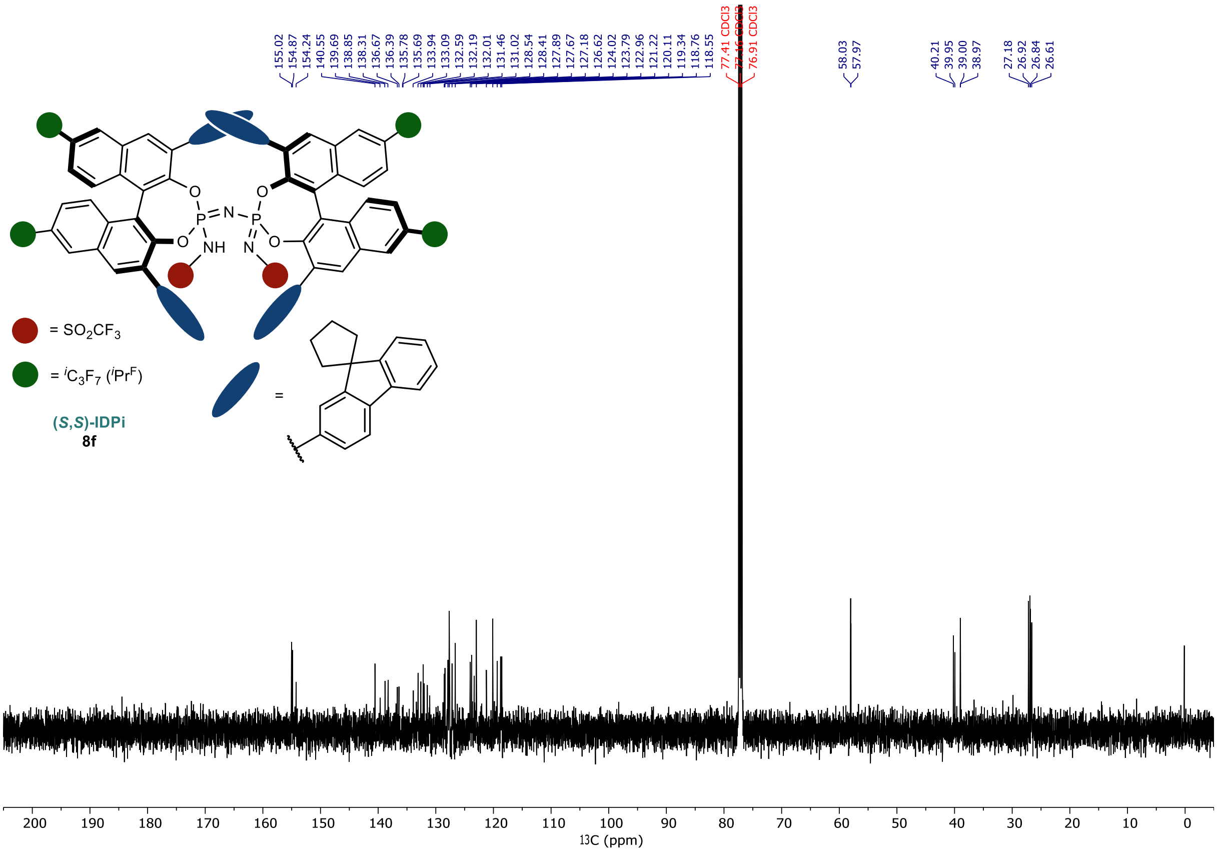

^{13}C NMR (126 MHz, $CDCl_3$) spectrum of (*S*,*S*)-IDPi catalyst **8f**

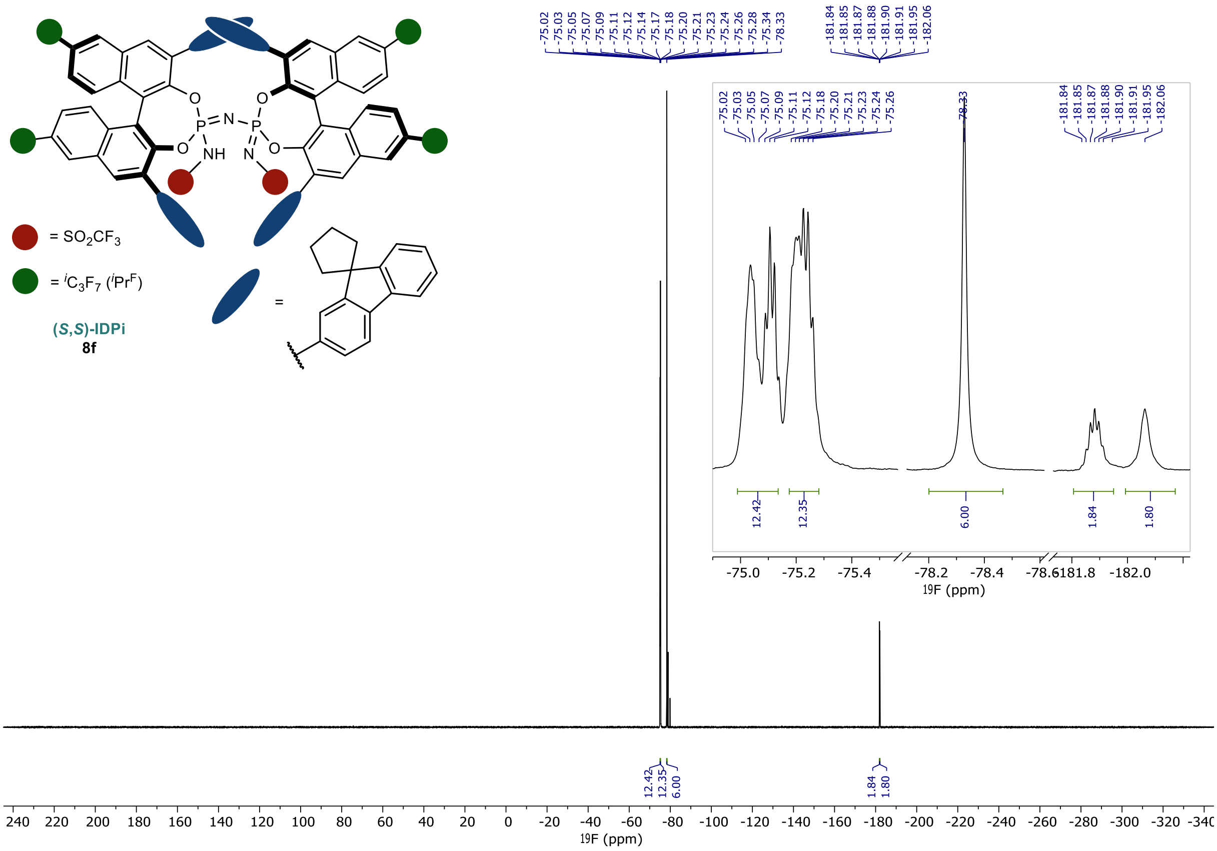

^{19}**F NMR** (471 MHz, $CDCl_3$) spectrum of (*S*,*S*)-IDPi catalyst **8f**

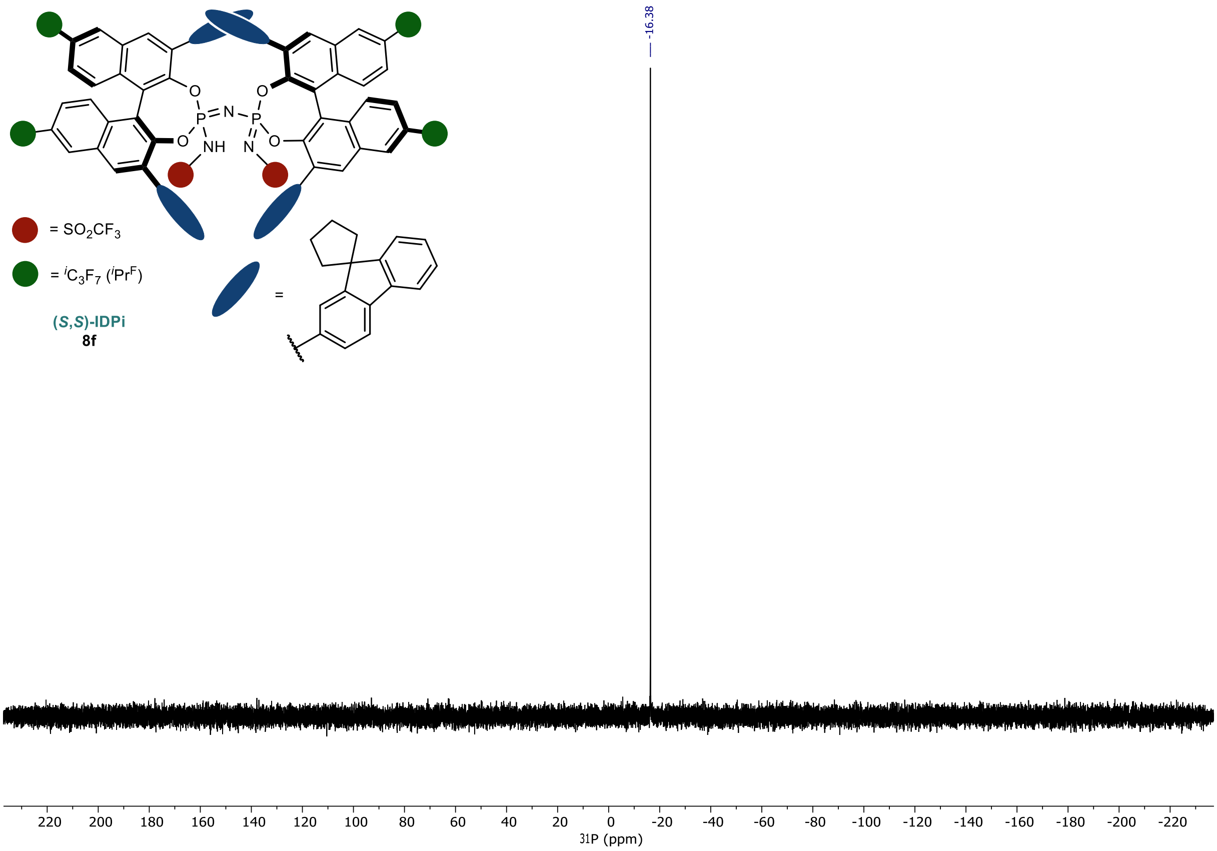

^{31}**P NMR** (203 MHz, $CDCl_3$) spectrum of (*S*,*S*)-IDPi catalyst **8f**

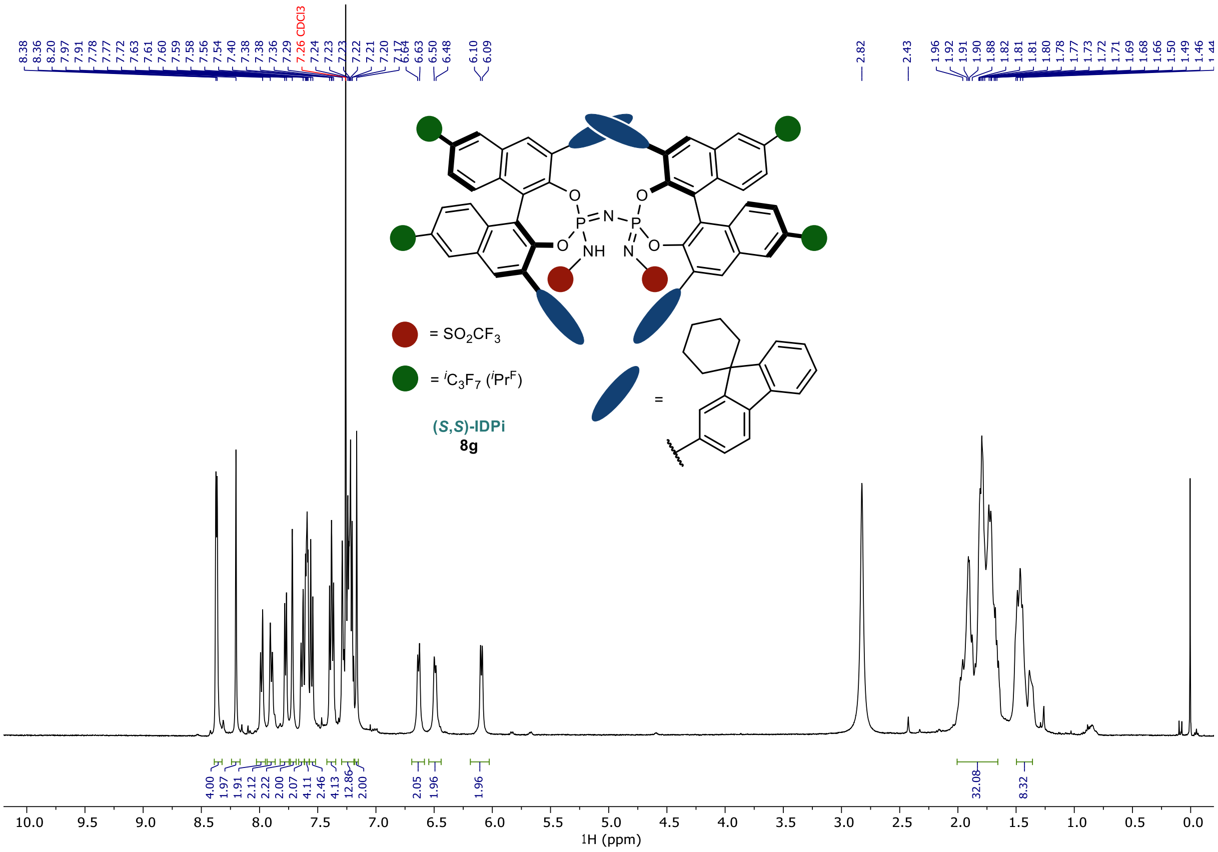

^{1}H NMR (501 MHz, $CDCl_3$) spectrum of (*S*,*S*)-IDPi catalyst **8g**

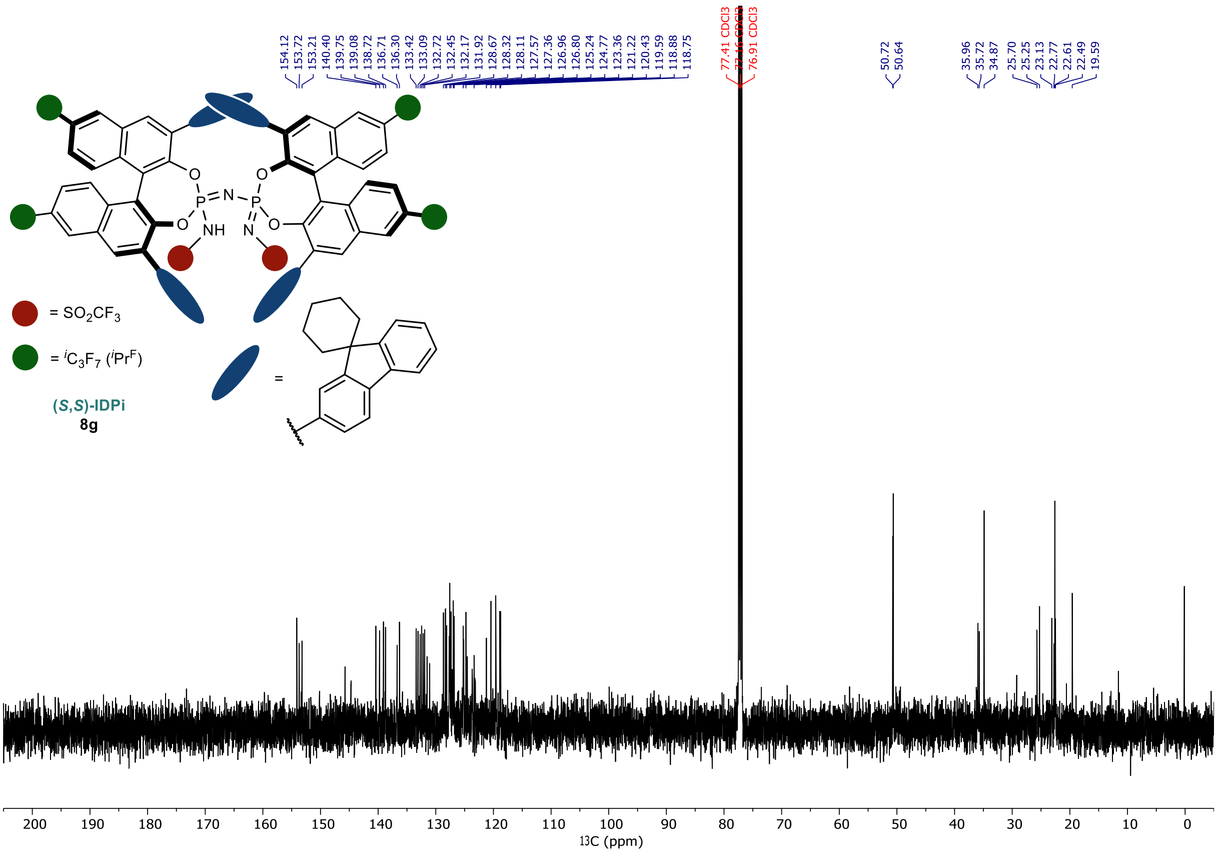

^{13}C NMR (126 MHz, $CDCl_3$) spectrum of (*S*,*S*)-IDPi catalyst **8g**

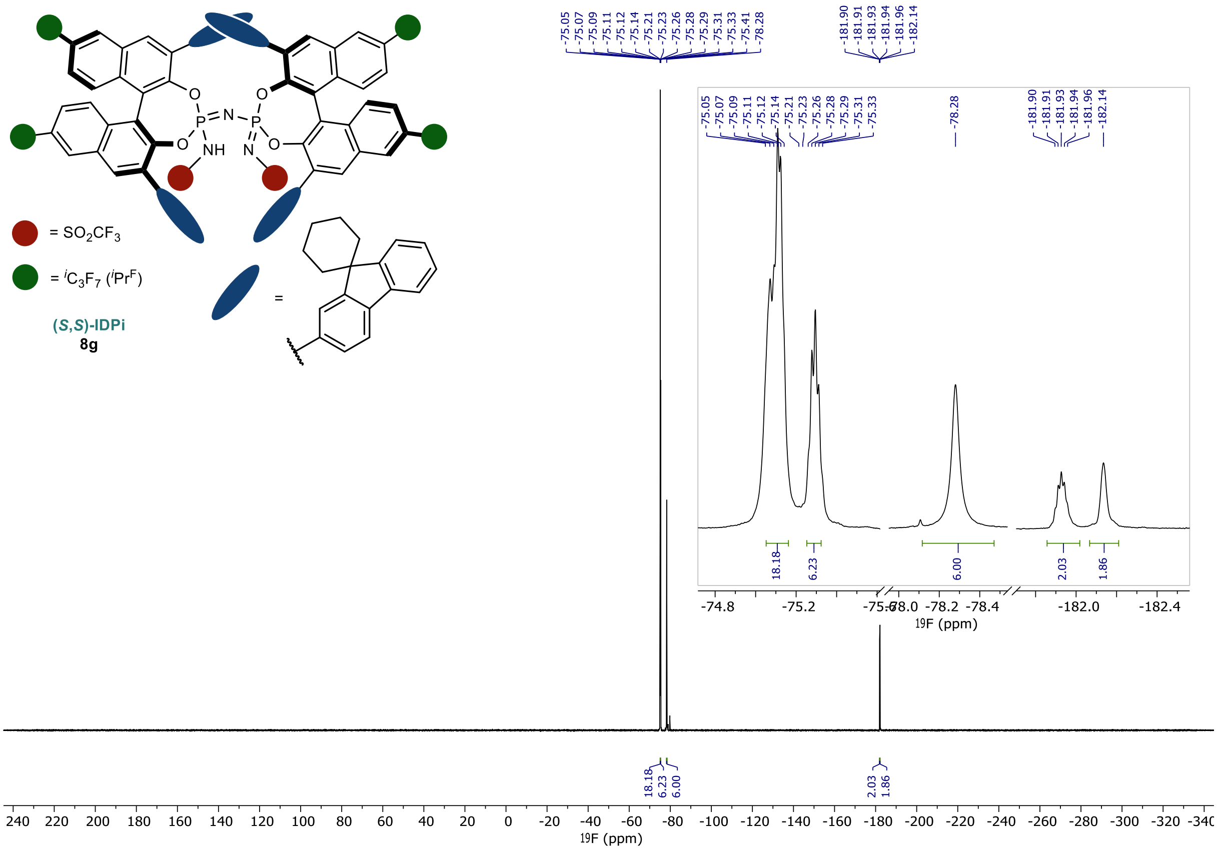

^{19}**F NMR** (471 MHz, CDCl3) spectrum of (*S*,*S*)-IDPi catalyst **8g**

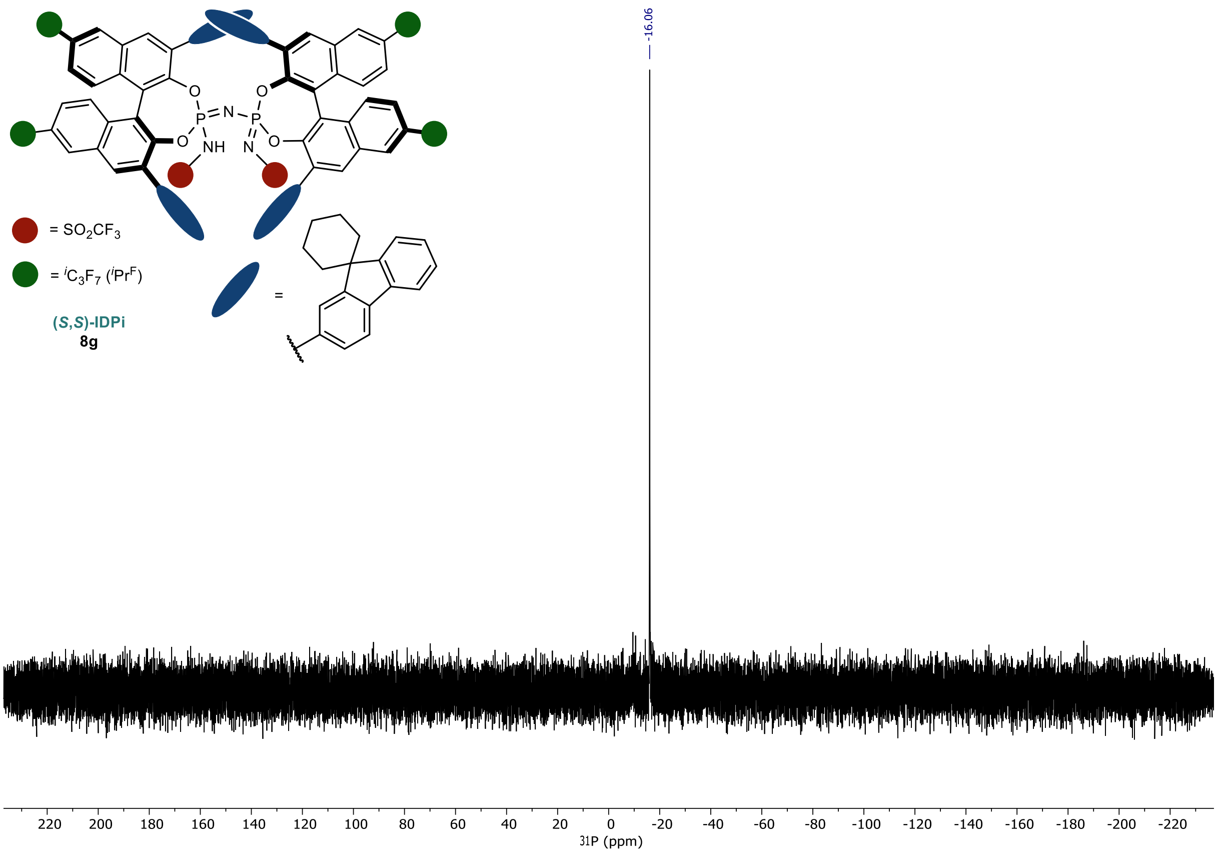

^{31}**P NMR** (203 MHz, CDCl3) spectrum of (*S*,*S*)-IDPi catalyst **8g**

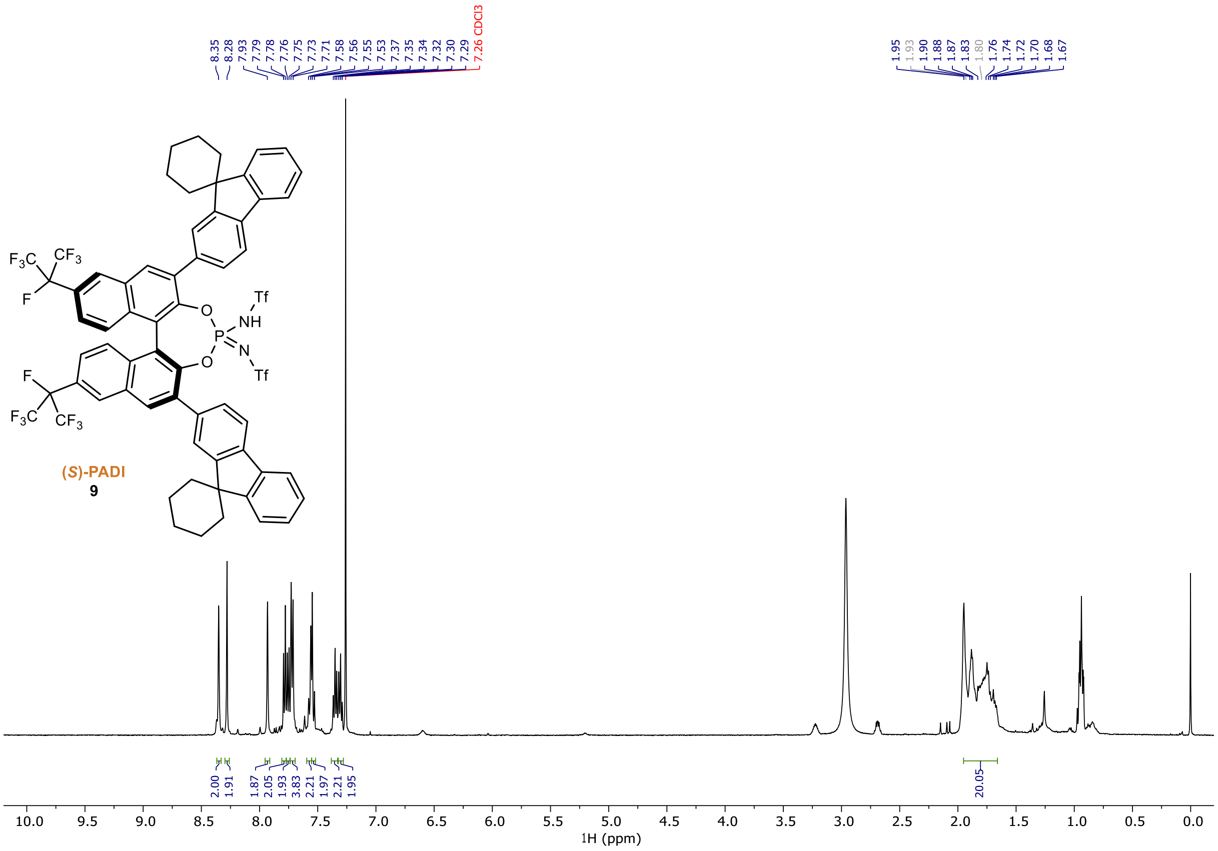

^{1}H NMR (501 MHz, $CDCl_3$) spectrum of (*S*)-PADI catalyst **9**

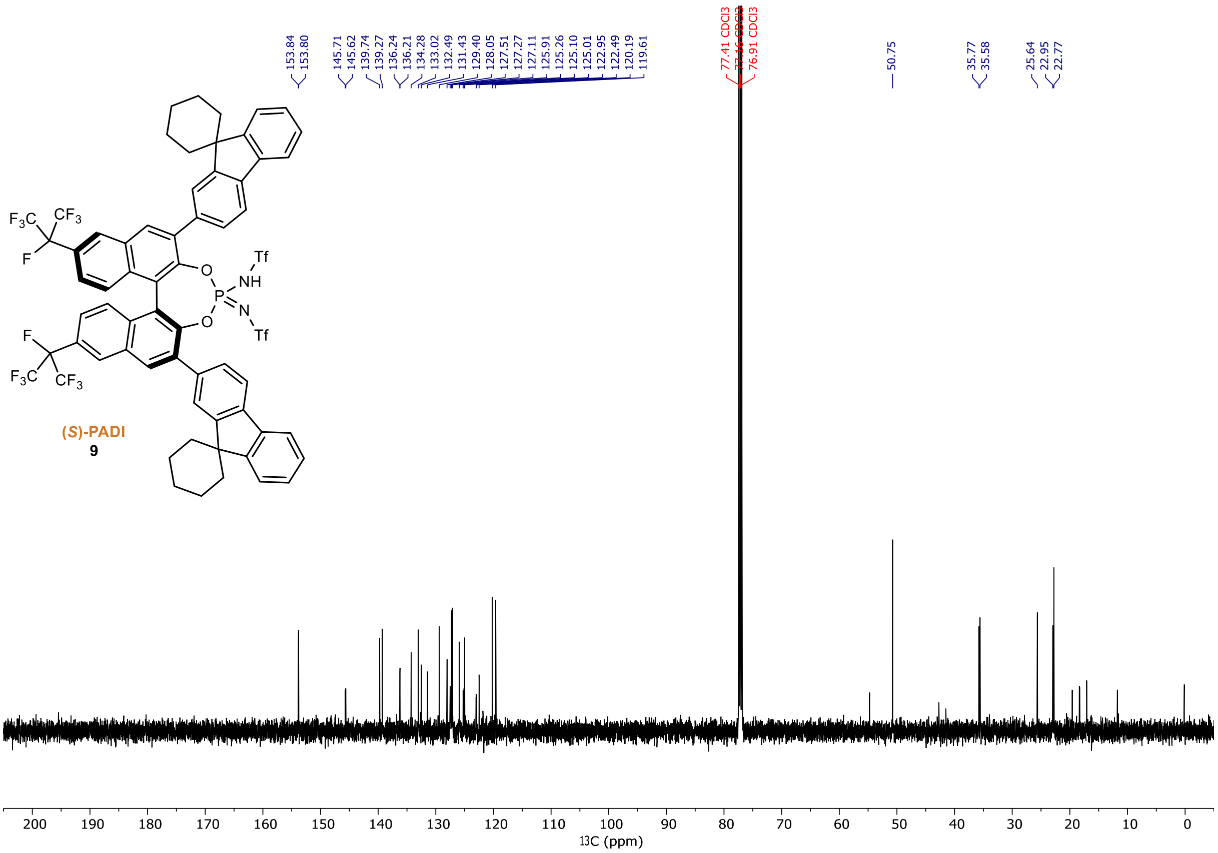

^{13}C NMR (126 MHz, $CDCl_3$) spectrum of (*S*)-PADI catalyst **9**

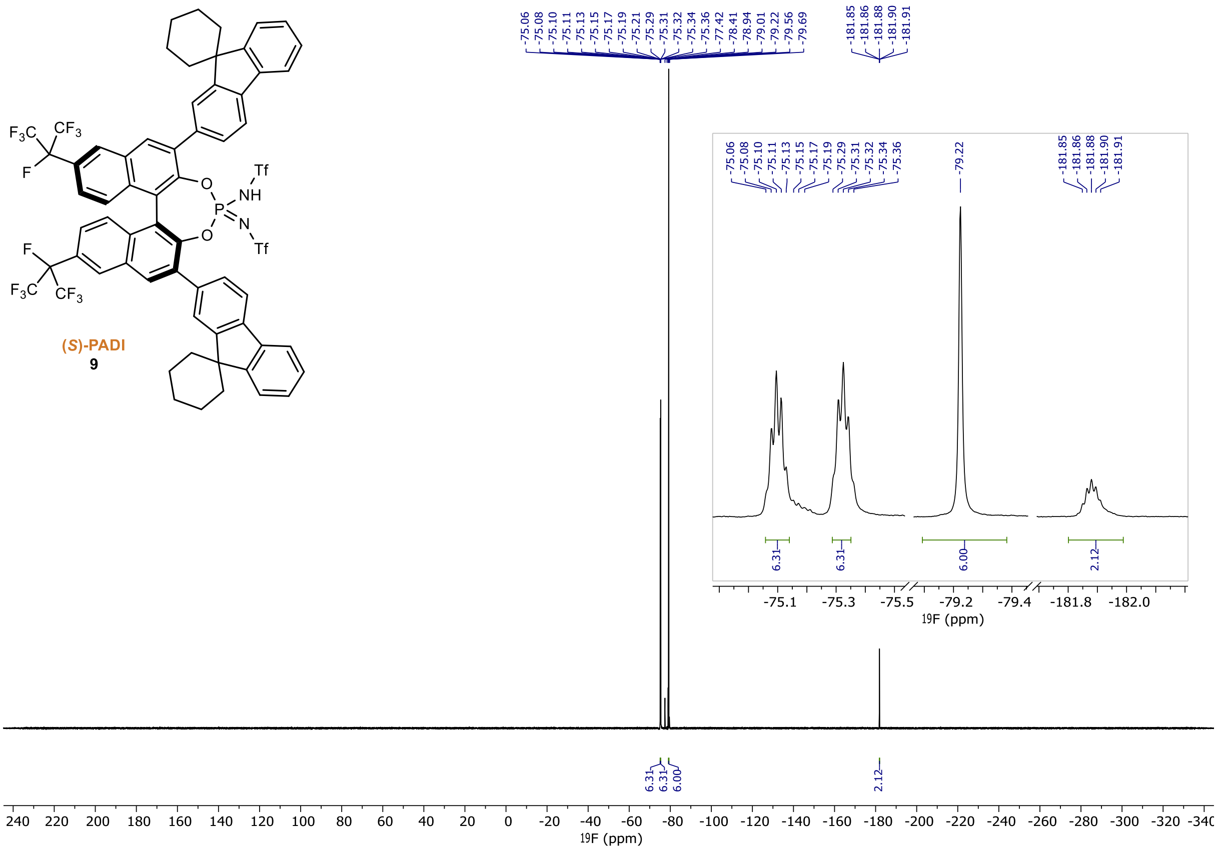

^{19}F NMR (471 MHz, $CDCl_3$) spectrum of (*S*)-PADI catalyst **9**

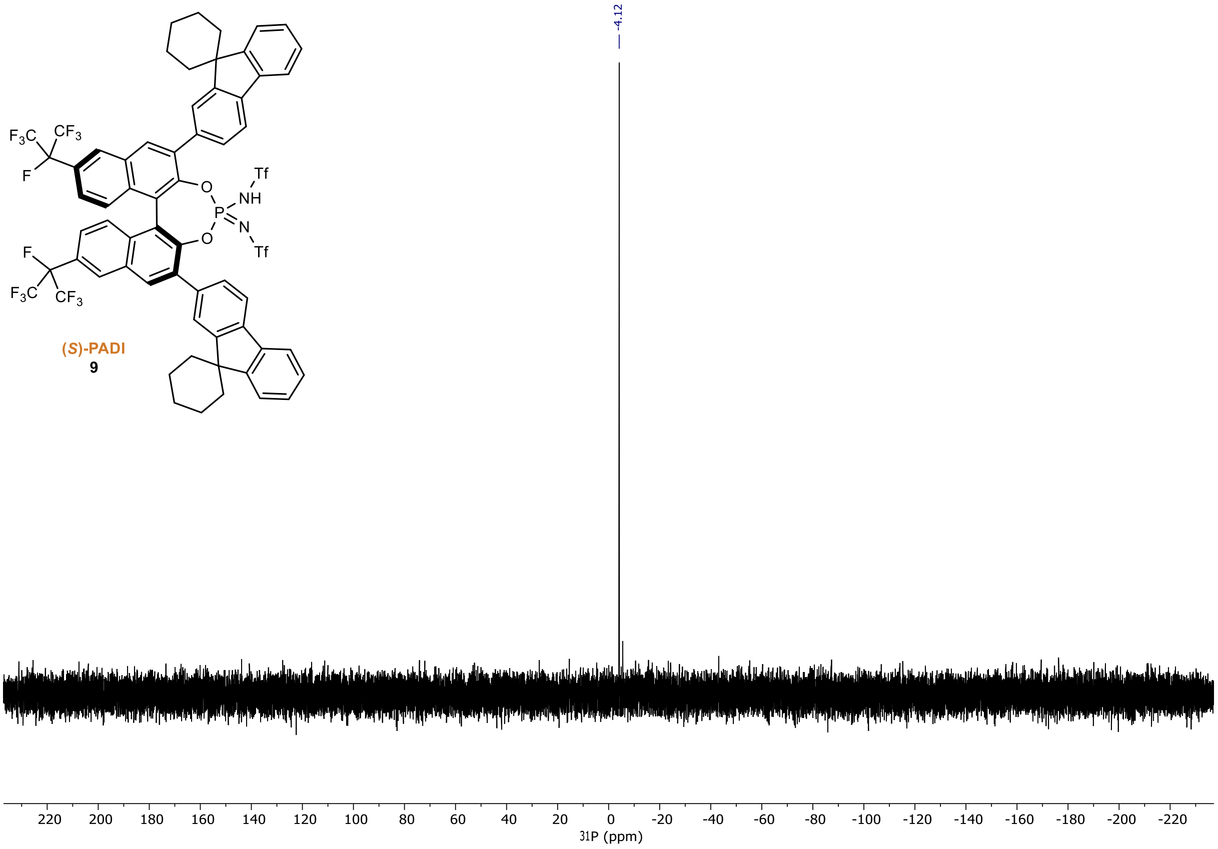

^{31}P NMR (203 MHz, $CDCl_3$) spectrum of (*S*)-PADI catalyst **9**
